# Genome-wide comprehensive analysis of transcriptomes and small RNAs offers insights into the molecular mechanism of alkaline stress tolerance in a citrus rootstock

**DOI:** 10.1038/s41438-018-0116-0

**Published:** 2019-03-01

**Authors:** Juxun Wu, Junying Cao, Mei Su, Guizhi Feng, Yanhui Xu, Hualin Yi

**Affiliations:** Key Laboratory of Horticultural Plant Biology, Ministry of Education, Huazhong Agricultural University, Wuhan, 430070 PR China

**Keywords:** Abiotic, Plant hormones

## Abstract

Alkaline stress has serious-negative effects on citrus production. Ziyang xiangcheng (*Citrus junos* Sieb. ex Tanaka) (Cj) is a rootstock that is tolerant to alkaline stress and iron deficiency. Trifoliate orange (*Poncirus trifoliata* (L.) Raf.) (Pt), the most widely used rootstock in China, is sensitive to alkaline stress. To investigate the molecular mechanism underlying the tolerance of Cj to alkaline stress, next-generation sequencing was employed to profile the root transcriptomes and small RNAs of Cj and Pt seedlings that were cultured in nutrient solutions along a three pH gradient. This two-level regulation data set provides a system-level view of molecular events with a precise resolution. The data suggest that the auxin pathway may play a central role in the inhibitory effect of alkaline stress on root growth and that the regulation of auxin homeostasis under alkaline stress is important for the adaptation of citrus to alkaline stress. Moreover, the jasmonate (JA) pathway exhibits the opposite response to alkaline stress in Cj and Pt and may contribute to the differences in the alkaline stress tolerance and iron acquisition between Cj and Pt. The dataset provides a wealth of genomic resources and new clues to further study the mechanisms underlying alkaline stress resistance in Cj.

## Molecular biology: Rooting out alkaline resistance mechanisms in citrus trees

The ability of citrus trees to tolerate alkaline soils may hinge upon plant hormones called auxins which regulate root growth. The discovery could aid the development of more resilient rootstocks. Alkaline stress, caused by e.g. industrial run-off, is a growing problem worldwide because it reduces the growth and survival of crops – including the most widely used citrus rootstock in China, Poncirus trifoliata. To better understand the mechanisms underpinning tolerance to soil alkalinity, Hualin Yi at Huazhong Agricultural University in Wuhan, China, and colleagues used next-generation sequencing to profile the transcription products of P. trifolata seedlings and those of an alkaline-tolerant rootstock, Ziyang xiangcheng, when they were grown in three different nutrient solutions. Auxin homeostasis appears to be a key element of citrus adaption to alkaline stress, they found - probably by encouraging lateral root branching.

## Introduction

Saline–alkaline soils are widespread throughout the world, limiting agricultural productivity globally^1–3^. Of the ~831 million ha of saline–alkaline soils, more than half are alkalinized^4,5^. Under alkaline stress, most plants cannot survive because of the high pH. Alkaline stress has a much stronger inhibitory effect on plant growth than salt stress^2,3,6^. However, research examining the effect of alkaline stress on plants and the adaptation mechanism of plants to alkaline stress are much scarcer than research on salt stress^2,7^. Thus, elucidation of the molecular mechanisms of plant responses to alkaline stress is urgently needed.

The root is an organ for the uptake and transport of water and nutrients, and it is also the first organ that perceives soil stresses. Root branching through lateral root (LR) formation is important for the root system to cope with various abiotic stresses^8,9^. Many studies have revealed that auxin acts as an integrator of many endogenous and exogenous signals for lateral root development regulation^9^. For instance, cytokinin (CK) inhibits LR formation by reducing auxin transport mediated by PIN1^10^ and by upregulating *MIZ1*^11^, a gene involved in the hydrotropism that reduces auxin accumulation in the roots. ABA can inhibit LR formation by upregulating miR393, which can suppress the expression of the TIR1/AFB auxin receptor^12^. Shkolnik-Inbar and Bar-Zvi^13^ have shown that ABA and CK inhibit LR formation by upregulating the *ABI4* transcription factor via reducing polar auxin transport, leading to a reduction in LR development. In addition, many nutrients also influence LR development by interfering with the auxin pathway, such as nitrates^14^, potassium^15^, and phosphate^16^.

Jasmonate (JA) is a significant signaling substance that is involved in plant responses to several abiotic and biotic stresses, such as drought stress, salt stress, wounding (mechanical stress), and pathogen infection^17^. JA is also involved in the regulation of developmental processes, including root growth, pollen production and senescence^18^. Many studies have shown that JA plays a positive role in salt stress responses in plants^19–25^. However, few studies have examined the role of JAs in response to alkaline stress compared with those on salt stress. Recently, Zhu et al.^26^ reported that TIFY10 proteins, which are JAZ proteins, positively regulate plant alkaline stress responses. A similar result was obtained by Zhu et al.^27^, who reported that the overexpression of *GsJAZ2* in Arabidopsis results in enhanced plant tolerance to salt and alkaline stress. In addition, JAs play important roles in root growth regulation. In Arabidopsis, treatment with JA can inhibit primary root growth^28^, whereas JA promotes lateral root formation at submicromolar concentrations by directly inducing the auxin biosynthesis gene *ASA1* and/or by modulating the PIN2 protein^29,30^. Raya-Gonzalez et al.^31^ also showed that low concentrations of JA inhibit primary root growth and promote lateral root formation in Arabidopsis seedlings. In rice, RSS3 interacts with the JA pathway to play a significant role in the regulation of root cell elongation under stressful conditions^32^.

Generally, alkaline stress accompanies iron deficiency. Iron is required for essential everyday processes in plants and is abundant in most soils. However, most iron exists as Fe^3+^ hydroxides, which are only somewhat soluble at neutral pH. The release of protons from roots increases the solubility of the Fe^3+^ hydroxides around the rhizosphere. The Fe^3+^ is reduced to Fe^2+^ by FRO2^33^, which was the rate-limiting step for iron acquisition from the soil^34^. Iron is then transported into the root epidermal cells by IRT1^35,36^. The FER-LIKE IRON DEFICIENCY-INDUCED TRANSCRIPTION FACTOR (FIT) plays a central role in upregulating the root-expressed genes involved in iron acquisition, which regulates *FRO2* at the transcription level and IRT1 at the protein level^37^. Indeed, a *fit* Arabidopsis mutant fails to take up iron and develops a lethal Fe deficiency, leading to leaf chlorosis^38^.

Citrus is one of the most important economic fruit crops worldwide. Grafting is the most important reproduction mode for citrus in the production industry. Moreover, rootstocks are significant for scions in citrus and can influence the fruit quality, canopy size, and resistance, among other aspects^39^. Ziyang xiangcheng (*Citrus junos* Sieb. ex Tanaka) (abbreviated Cj) is a special citrus germplasm native to China that is widely used as an iron-deficiency tolerant and alkaline-tolerant citrus rootstock in areas of China with calcareous soil. Trifoliate orange (*Poncirus trifoliata* (L.) Raf.) (abbreviated Pt) is the most widely used rootstock in China. However, Pt is sensitive to alkaline stress. Scions grafted on Pt show nutritional deficiency and growth retardation phenotypes in calcareous soil. However, little is known about the molecular mechanism underlying the differences in phenotypes between Cj and Pt regarding alkaline stress. Thus, in this study, the seedlings of Cj and Pt cultured in different pH nutrient solutions were used to perform comparative analyses, including assessments of the transcriptomes and small RNAs.

## Results

### Difference between Cj and Pt grown hydroponically with different pH gradients

**Fig. 1:**
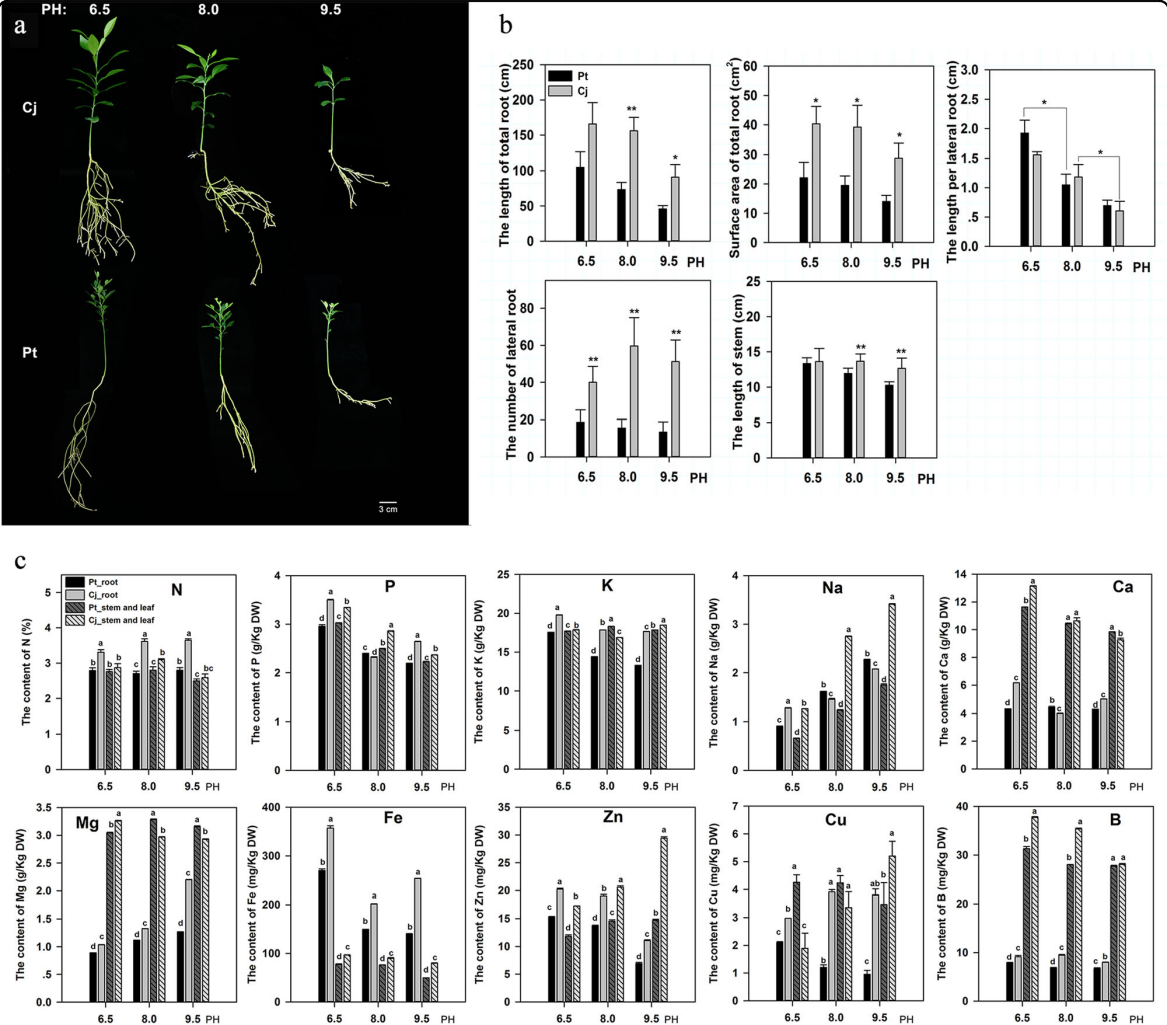
Phenotypic characterization of seedlings of Cj and Pt cultured in solutions along a pH gradient. **a** The morphological differences between Cj and Pt along a pH gradient **b** and the trends of several mineral element levels in the root, stem and leaf of Cj and Pt along a pH gradient **c**. In this figure, the samples are named “Sample_tissue.”. Pt_root indicates the root tissue of Pt. Bars represent the standard error (*n* = 3). A single asterisk (*) represents statistically significant differences (*P* < 0.05), and double asterisks (**) represent highly statistically significant differences (*P* < 0.01), analyzed using Student’s *t*-test. Lowercase represents statistically significant differences (*P* < 0.05), analyzed using one-way ANOVA

After 8 weeks of culture in nutrient solutions of different pH (6.5, 8.0, and 9.5), Cj and Pt showed different performances. As shown in Fig. 1a, b, the lengths of the stems and roots were markedly decreased in both Cj and Pt under extremely alkaline conditions (pH 9.5). At pH 6.5, the lengths of the total roots and stems did not differ between the Cj and Pt, but the number of lateral roots and total root surface area of the Cj were significantly increased compared with those of the Pt (Fig. 1b). As the pH value increased, the root architecture was altered in both Cj and Pt, and the differences gradually increased between Cj and Pt, including the total root and stem lengths, total root surface area and number of lateral roots (Fig. 1b). In particular, the number of lateral roots of Cj was much higher than that of Pt (approximately fourfold higher at pH 8.0 and pH 9.5) (Fig. 1a, b).

The mineral elements in Cj and Pt were also measured. As shown in Fig. 1c, the contents of all of the mineral elements exhibited significant differences between Cj and Pt in the root tissue and mixed tissue (stem and leaf) under all three conditions. In the root tissue, the levels of most mineral elements exhibited a decreasing trend as the pH increased, with the exception of Cu, Na, and Mg, and the trend curves of the mineral elements were similar for the Cj and Pt. The Na and Mg levels increased as the pH value increased. The Cu levels increased with increasing pH in Cj, whereas they decreased in Pt (Fig. 1c). Notably, the Fe levels in both tissues of the Cj were much higher than those in the Pt under all three conditions, and the Fe levels in both the Cj and Pt markedly decreased under alkaline stress (Fig. 1c).

### Global analysis of root transcriptomes, small RNAs, and degradomes of Cj and Pt

**Fig. 2:**
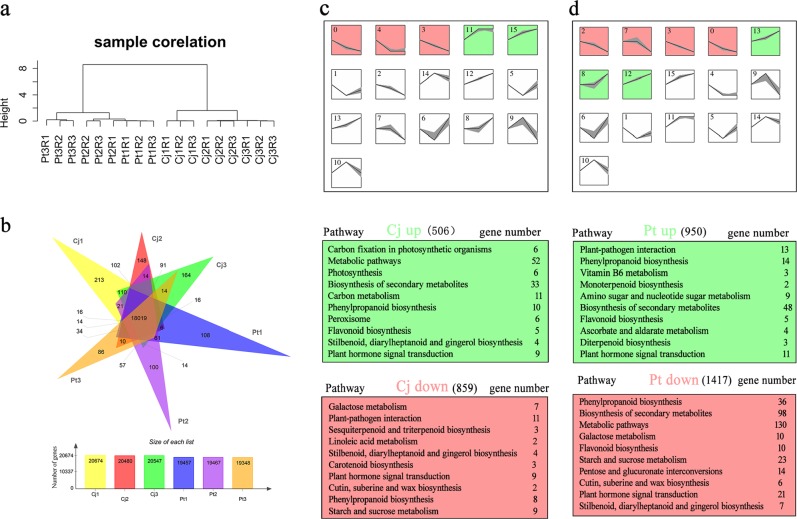
Analysis of global gene expression in Cj and Pt. **a** Cluster dendrogram depicting the global relationships between biological replicates and among different samples. In this figure, samples are named as “Cultivar_pH condition_replicate.” Cj1R1 indicates Cj_pH 6.5_Replicate 1. **b** Venn diagram depicting the number of commonly and uniquely expressed genes among the six samples. **c** Model profiles of genes in Cj along a pH gradient generated by the STEM clustering algorithm and top 10 enriched KEGG pathways of the Cj up and Cj down cluster profiles. **d** Model profiles of genes in Pt along a pH gradient generated by the STEM clustering algorithm and top 10 enriched KEGG pathways of the Pt up and Pt down cluster profiles. Each box corresponds to a model expression profile. The *X*-axis of each box from left to right is pH 6.5, pH 8.0, and pH 9.5. Colored model profiles contain a statistically significant number of assigned genes (*p*-value <0.05). Model profiles with the same color belong to the same cluster of profiles. The number in the top left corner indicates the model profile ID

To investigate the underlying molecular changes that accompanied the morphological and physiological changes described above, we used RNA-seq to generate transcriptome, small RNA and degradome profiles of the root tissues of Cj and Pt. All sequencing data are summarized in Supplementary Table S1. Regarding the transcriptomes, after the low-quality reads had been removed, 40–55 million clean reads per sample were mapped against the *Citrus sinensis* reference genome^40^ (Supplementary Table S1). The correlation dendrogram illustrates the global relative relationships among the 18 transcriptomes (Fig. 2a). All three biological replicates clustered together, indicating good repeatability of the RNA-seq data. The Cj and Pt samples were clustered together, respectively (Fig. 2a). A total of 28,470 genes were identified in these six samples (Supplementary Table S2). Between 19,348 (Pt3) and 20,674 (Cj1) genes were detected in each sample (Fig. 2b). In total, 18,019 genes were common among all six samples (Fig. 2b, Supplementary Table S2).

For small RNAs, a total of 18 small RNA libraries were constructed for six samples. Each sample was assessed in the three biological replicates. In total, 11–26 million clean reads per replicate were obtained by RNA-seq (Supplementary Table S1). A search of the Rfam, Silva, GtRNAdb, and Repbase databases was used to remove structural RNAs (rRNAs, tRNAs, scRNAs, snRNAs, and snoRNAs). Unannotated sRNAs (4.3–16.4 million reads) were mapped to the *Citrus sinensis* genome and were used to identify known miRNAs and predict novel miRNAs by miRDEEP2^41^ based on the structure and expression criteria^42^ (Supplementary Table S1). In total, 49 known miRNAs and 52 novel miRNAs were identified in the Cj and Pt samples (Supplementary Table S8 and Figure S5).

To identify the target genes of the miRNAs, degradome sequencing^43,44^ was performed to experimentally validate the target genes of the miRNAs by capturing mRNA cleavage segments. To maximize the identification of the miRNA targets, we pooled RNA from Cj1, Cj2, and Cj3 into one Cj library and pooled RNA from Pt1, Pt2, and Pt3 into one Pt library. In total, 27.04 and 26.15 million clean reads were obtained from the Cj and Pt libraries, respectively (Supplementary Table S1). After removing miRNAs, structural RNAs and others, 16.06 and 15.88 million reads of the Cj and Pt, respectively, were mapped to the *Citrus sinensis* genome (Supplementary Table S1). The reads that mapped to the cDNA (the reads in the cDNA_sense category in Supplementary Table S1) were analyzed to detect candidate targets of miRNAs.

### Identification of temporal expression trends accompanying gradient alkaline stress

STEM^45^ was used to perform clustering to identify the different gene expression profiles across all three pH conditions in the gradient. After the STEM clustering algorithm was applied, 16 model profiles with 5 statistically significant model profiles (colored profiles) were identified in Cj, and 16 model profiles with 7 statistically significant model profiles were identified in Pt (Fig. 2c, d, Supplementary Table S3). Model profiles with the same color belonged to the same cluster of profiles. Therefore, two cluster profiles (an upregulated profile and downregulated profile) were identified in Cj and Pt, respectively (Fig. 2c, d). These four cluster profiles were named Cj up, Cj down, Pt up, and Pt down. A total of 506, 859, 950, and 1417 genes were clustered into the Cj up, Cj down, Pt up, and Pt down profiles, respectively (Fig. 2c, d). A Venn diagram revealed that only 157 genes exhibited common expression trends in Cj and Pt (Supplementary Figure S1a). This result revealed that large differences existed between Cj and Pt at the transcription level in response to alkaline stress.

These four gene sets (Cj up, Cj down, Pt up, and Pt down) were subject to KEGG pathway enrichment analysis (Supplementary Table S4). As shown in Fig. 2c, d, the top 10 over-represented KEGG pathways are listed. Notably, the “Phenylpropanoid biosynthesis” and “Plant hormone signal transduction” pathways were enriched in all four gene sets. Then, we used a heatmap to display the gene expression trends of the genes that were enriched in these two pathways (Supplementary Figure S2). As shown in Figure S2a, b, in the phenylpropanoid biosynthesis pathway, 22 peroxidase genes (22/50) were downregulated in Pt with increasing pH values, whereas only 4 peroxidase genes (4/18) were downregulated in Cj. In the plant hormone signal transduction pathway, many auxin signal transduction genes (14 genes in Pt and 10 genes in Cj) were enriched (Supplementary Figure S2c, d). In Pt, most auxin signal transduction genes (12/14) were downregulated. In contrast, one-half of the auxin signal transduction genes (5/10) were downregulated in Cj. These results indicate that peroxidase and auxin signal transduction genes may play significant roles in response to alkaline stress.

In addition, we identified transcription factors (TFs) in these four profiles. In total, 66 and 97 TFs were identified in Cj and Pt, respectively (Supplementary Figure S1b, c, Table S3). Among these TFs, the number of ERF family TFs was considerably greater compared with the number of TFs in the other families (Supplementary Figure S1b, c). Moreover, 24 TFs were identified both in Cj and Pt (Supplementary Table S3). As shown in Supplementary Figure S1d, the expression trends of most of these 24 TFs differed between Cj and Pt. Dynamic expression changes associated with these TFs may reveal their key functions. For example, *MYC2* (orange1.1t01021), *GBF4* (orange1.1t01148), *WRKY46* (orange1.1t00472), and *ZAT12* (Cs8g17960) were specifically downregulated in Cj and upregulated in Pt as the pH value increased (Supplementary Figure S1d, Table S3). Notably, *MYC2* is a key gene in JA signal transduction. This finding indicates that JA signaling may also be involved in response to alkaline stress.

### Analysis of transcriptomic changes in response to alkaline stress

**Fig. 3:**
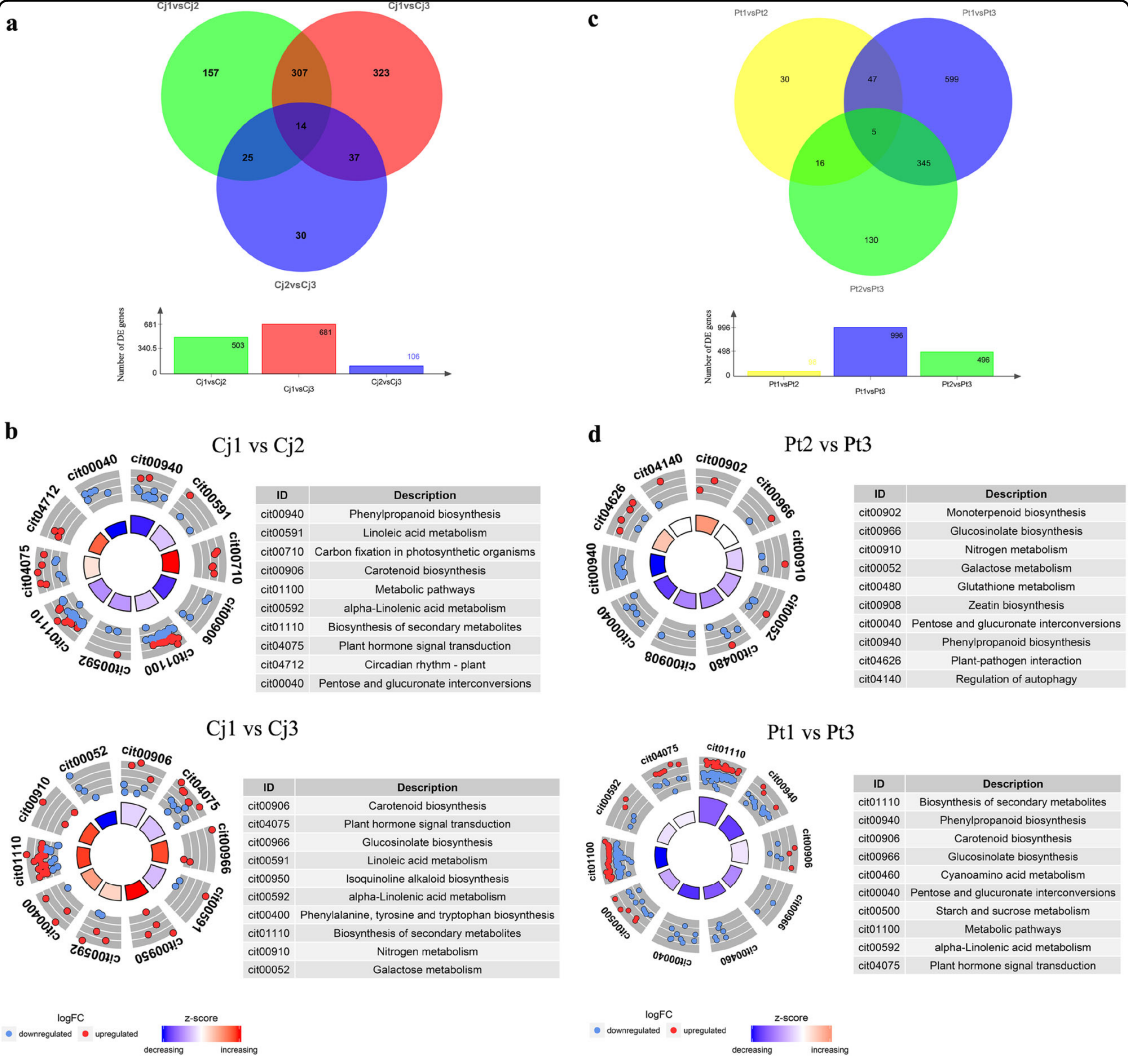
Differential expression genes of Cj and Pt among the different pH conditions. Venn diagrams depicting the number of common and unique DEGs among the different comparison groups in Cj **a** and Pt **c**, and the circular visualization of the KEGG pathway enrichment results of DEGs from different comparison groups in Cj **b** and Pt **d**. In Sample1 vs. Sample2, Sample1 serves as the control group. The term logFC means log_2_(FPKM_Sample2/FPKM_Sample1). The *Z*-score is an easily calculated value that provides a hint regarding the pathway that is more likely to be decreased (negative value, blue color) or increased (positive value, red color). DEG differentially expressed gene

As alkaline stress increased, the phenotypes were largely altered in both Cj and Pt. Simultaneously, the transcriptome profiles of Cj and Pt were also significantly altered. In Cj, 503, 681, and 106 DEGs (differentially expressed genes) were identified in the Cj1 vs. Cj2, Cj1 vs. Cj3, and Cj2 vs. Cj3 comparison groups, respectively (Fig. 3a). In Pt, 98, 996, and 496 DEGs were identified in the Pt1 vs. Pt2, Pt1 vs. Pt3, and Pt2 vs. Pt3 comparison groups, respectively (Fig. 3c). The smallest difference was noted between the Cj2 and Cj3 transcriptomes as well as between the P1 and Pt2 transcriptomes. This result suggests that Cj responds to alkaline stress more quickly than Pt.

**Fig. 4:**
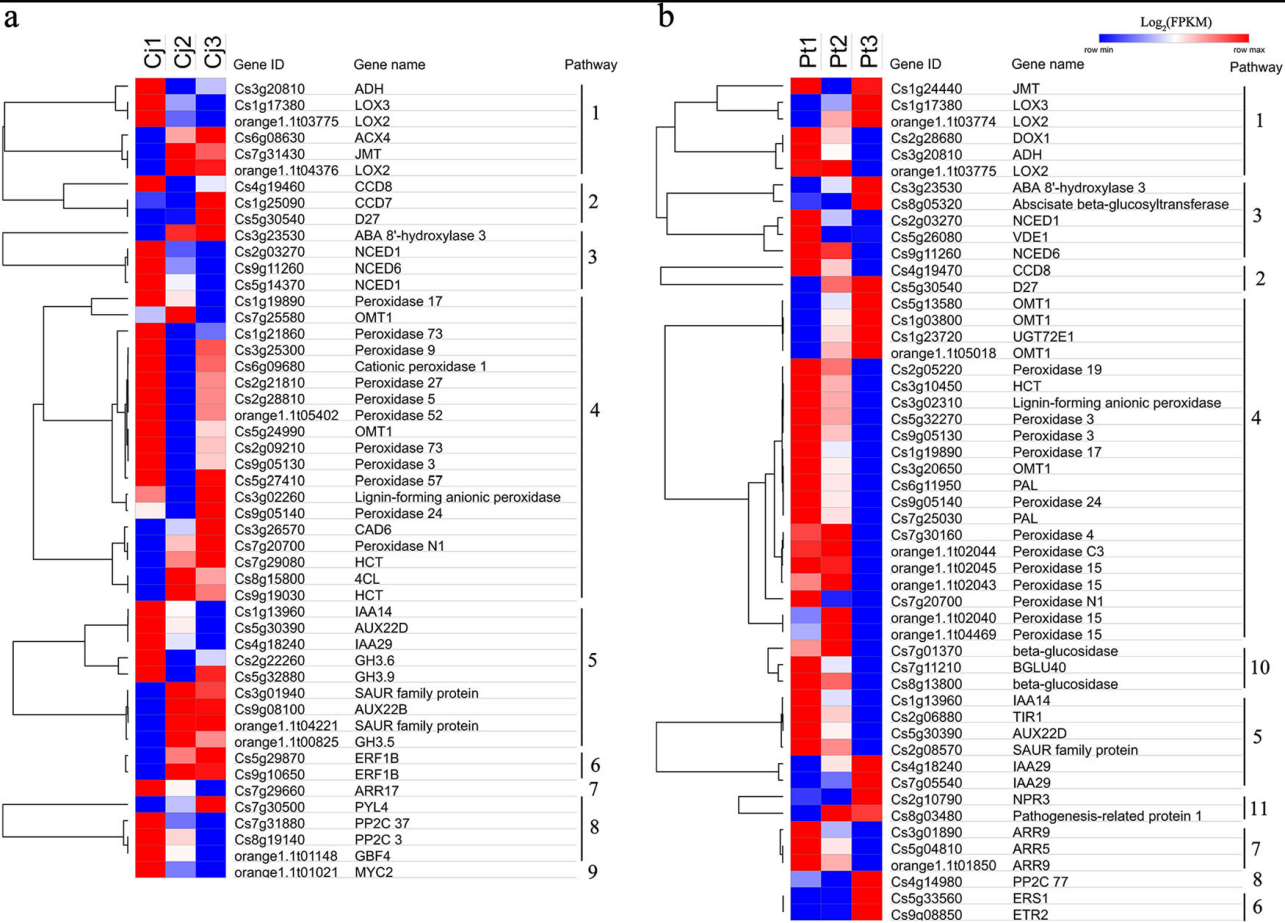
Hierarchical clustering of DEGs in several key pathways across the different pH conditions in Cj. **a** and Pt **b** Each row of the heatmap represents an individual gene, and the coloring represents the expression level. Pathways: 1, alpha-Linolenic acid metabolism; 2, Strigolactone biosynthesis; 3, ABA biosynthesis; 4, Lignin biosynthesis; 5, Auxin signal transduction; 6, Ethylene signal transduction; 7, Cytokinin signal transduction; 8, ABA signal transduction; 9, JA signal transduction; 10, Coumarin biosynthesis; 11, Salicylic acid signal transduction. DEG differentially expressed gene

To further characterize the significant roles of Cj and Pt in response to alkaline stress, the DEGs of these six comparison groups of Cj and Pt were used to perform GO-based term and KEGG-based pathway enrichment analyses (Supplementary Tables S6 and S7). Because the number of enriched pathways in Cj2 vs. Cj3 and P1 vs. Pt2 was minimal, the pathways enriched in the other four comparison groups were used to identify important pathways. The top 10 most enriched pathways and the DEGs distributed in these pathways were visualized using the GOplot package^46^ (Fig. 3b, d). The number of upregulated DEGs was more than that of downregulated DEGs in the top 10 pathways in the Cj1 vs. Cj3 group, whereas the opposite pattern was observed in the Pt1 vs. Pt3 group (Fig. 3b, d). Several key pathways were screened from these four comparison groups, including “Glucosinolate biosynthesis”, “Phenylpropanoid biosynthesis”, “Carotenoid biosynthesis”, “alpha-Linolenic acid metabolism”, and “Plant hormone signal transduction” (Fig. 3b, d). In the glucosinolate biosynthesis pathway, three genes (Cs7g29760, Cs7g29770, and orange1.1t01456) encoding phenylalanine N-monooxygenase (CYP79A2) were enriched. These genes were all upregulated in Cj and downregulated in Pt as the pH increased. CYP79A2 is a key gene in the biosynthesis of glucotropaeolin^47^. The expression patterns of the DEGs enriched in “Phenylpropanoid biosynthesis”, “Carotenoid biosynthesis”, “alpha-Linolenic acid metabolism”, and “Plant hormone signal transduction” were visualized by heatmaps (Fig. 4). As shown in Fig. 4, in the phenylpropanoid biosynthesis pathway, many peroxidase genes were enriched, displaying V-shaped expression patterns in Cj. However, these genes displayed downregulation trends in Pt. Moreover, several key genes involved in JA, ABA, and strigolactone (SL) biosynthesis were significantly altered by alkaline stress, such as *LOX3* (Cs1g17380) and *JMT* (Cs7g31430, Cs1g24440) for JA biosynthesis, *NCED1* (Cs5g14370, Cs2g03270) and *ABA 8’-hydroxylase 3* (Cs3g23530) for ABA biosynthesis, and *D27* (Cs5g30540) for SL biosynthesis (Fig. 4, Table 1). According to the expression trends of these key genes, the levels of ABA, JA, and SL may be altered by alkaline stress. This result indicates that ABA, JA, and SL may play important roles in tolerance to alkaline stress. In addition, many DEGs were enriched in plant hormone signal transduction, including auxin, CK, ABA, JA, ethylene (ETH) and salicylic acid signals (Fig. 4). More DEGs were enriched in auxin signal transduction in both Cj and Pt, and these DEGs displayed mixed expression patterns (some were upregulated, and some were downregulated). In Pt, the number of downregulated auxin signal genes was greater than that of the upregulated genes, and the opposite finding was observed for Cj. This result indicates that the auxin signal in Pt is more downregulated by alkaline stress compared with that in Cj. Moreover, the JA, ABA, CK, and ETH signals were also altered by alkaline stress in both Cj and Pt (Fig. 4).

**Table 1:** A list of important differentially expressed genes in Cj or Pt under different pH conditions

Gene ID	Gene name	FPKM					
		Cj1	Cj2	Cj3	Pt1	Pt2	Pt3
*Plant hormone biosynthesis*							
Cs1g17380	*LOX3*	126.20	60.53	43.40	51.93	76.67	177.22
orange1.1t03774	*LOX2*	231.01	289.28	324.75	234.67	380.47	483.81
Cs1g24440	*JMT*	4.87	8.52	5.89	79.69	37.49	77.35
Cs2g03270	*NCED1*	1.42	0.55	0.45	3.31	1.45	0.87
Cs5g14370	*NCED1*	59.44	19.47	7.08	19.29	12.78	22.50
Cs3g23530	*ABA 8’-hydroxylase 3*	0.29	0.87	0.96	1.75	3.48	8.42
*Auxin signal transduction*							
Cs2g06880	*TIR1*	14.56	11.52	12.04	12.48	9.11	5.72
Cs1g13960	*IAA14*	83.72	57.11	38.22	88.59	54.95	37.91
Cs3g01940	*SAUR family protein*	192.92	331.85	309.91	75.05	54.95	49.84
Cs4g18240	*IAA29*	35.45	11.17	4.40	0.45	1.04	2.04
Cs5g30390	*AUX22D*	33.49	13.10	4.49	5.12	3.04	1.70
*JA signal transduction*							
Cs1g17220	*TIFY 10* *A*	72.55	78.69	62.94	187.85	179.28	190.65
Cs7g02820	*TIFY 9*	104.68	109.34	71.07	267.47	279.69	337.96
orange1.1t01021	*MYC2*	159.59	83.55	67.92	123.03	170.53	227.57
*Cytokinin signal transduction*							
Cs5g04810	*ARR5*	68.03	114.63	111.99	107.62	57.38	26.45
Cs3g01890	*ARR9*	12.95	20.49	19.15	86.02	38.91	25.45
orange1.1t01850	*ARR9*	24.43	46.81	44.90	131.95	84.08	35.45
*ABA signal transduction*							
Cs7g30500	*PYL4*	10.59	15.90	31.14	15.66	15.16	10.68
Cs7g31880	*PP2C 37*	137.34	79.47	67.85	70.22	62.68	65.05
Cs4g14980	*PP2C 77*	17.38	13.60	9.11	2.93	2.20	6.36
orange1.1t01148	*GBF4*	25.27	17.10	11.24	7.30	11.06	14.95
*ETH signal transduction*							
Cs5g33560	*ERS1*	53.88	53.74	62.50	41.84	42.04	84.30
Cs9g08850	*ETR2*	25.49	22.29	25.90	13.98	14.13	28.92
Cs9g10650	*ERF1B*	0.59	2.40	2.28	11.72	6.31	35.82
Cs5g29870	*ERF1B*	3.91	8.79	11.45	17.88	19.25	28.21
*Ion transport*							
Cs6g09150	*Ferritin-1*	308.96	263.21	132.31	69.33	74.37	8.59
Cs8g15600	*FIT1*	10.02	14.90	25.94	5.30	5.71	1.88
orange1.1t00399	*FRO2*	1.32	1.44	14.88	2.38	5.40	1.61
Cs9g19350	*FRO4*	12.08	32.08	20.97	4.75	12.10	3.35
Cs3g01120	*FRO6*	45.44	64.64	77.65	54.26	36.29	35.95
Cs2g17670	*Vacuolar iron transporter 1*	7.96	8.24	6.91	3.24	6.01	1.08
Cs2g17680	*Vacuolar iron transporter 1*	1.03	0.87	0.88	2.59	4.52	0.39
Cs4g04460	*HKT1*	8.73	4.83	3.48	13.89	12.01	6.33
Cs1g17440	*Potassium transporter*	1.71	3.46	6.56	5.03	5.39	1.97
Cs8g19470	*COPT1*	105.38	280.36	146.19	239.85	307.49	89.99
Cs6g11670	*PIP2-2*	1.68	1.79	2.14	247.81	211.03	61.79
Cs6g11700	*PIP2-2*	742.85	515.42	512.74	327.20	237.80	98.21
Cs7g28650	*TIP1-3*	318.73	206.19	209.54	865.85	888.13	374.02
Cs8g17900	*TIP1-3*	525.44	309.47	308.31	445.38	478.62	193.00
Cs5g08710	*TIP2-2*	249.87	159.79	208.14	290.55	318.31	129.69
Cs6g20570	*PMA1*	93.09	84.97	103.70	71.72	80.69	156.91
Cs2g10720	*ZIP10*	1.29	9.05	68.08	3.88	23.69	8.59
Cs2g11620	*ZIP5*	17.59	34.08	62.11	58.95	59.75	92.42
Cs4g08930	*ZIP5*	52.70	110.94	76.30	77.90	83.16	66.52
Cs6g11470	*ZIP5*	71.45	130.31	184.01	77.77	123.44	232.80
*Response to stimulus*							
orange1.1t03944	*ORG2*	1.63	1.93	12.84	7.15	8.57	35.16
Cs1g11190	*ZFP1*	4.63	9.85	12.34	16.25	15.37	5.34
Cs6g05660	*ZAT11*	22.12	5.86	10.95	66.02	17.73	9.48
Cs1g18580	*DOF1.7*	44.31	20.81	11.51	10.18	11.45	24.69
Cs3g19420	*ERF012*	31.00	26.08	15.58	8.55	20.41	29.27
Cs3g21660	*ERF110*	0.61	1.99	2.25	11.41	3.06	2.17
Cs6g15360	*Dehydration-responsive element-binding protein 1D*	4.75	0.79	1.05	10.52	5.59	27.18
Cs6g15410	*ERF024*	1.06	7.37	5.34	4.86	8.53	1.97
Cs8g05910	*ERF109*	17.31	4.67	2.56	7.31	11.04	25.29
Cs9g13620	*ERF5*	361.93	154.96	168.66	45.62	46.22	117.12
Cs4g12130	*Chitin-inducible gibberellin-responsive protein 1*	172.13	61.51	40.99	54.90	46.03	92.31
Cs6g15680	*HRA1*	9.24	2.59	3.18	1.54	1.76	4.28
orange1.1t00472	*WRKY46*	224.68	138.04	83.44	113.21	101.83	265.74

As shown in Fig. 1c, the uptake of mineral elements, especially metal elements such as Fe, Zn, and Cu, is largely influenced by alkaline stress. The GO enrichment analysis results indicated that 49 and 68 DEGs were significantly enriched in the “ion transport” term in Cj and Pt, respectively (Supplementary Figure S3, Table S7). As shown in Supplementary Figure S4a, iron-transport-related genes (*FIT1*, *FRO2/4/6*, *Ferritin-1*), zinc-transport-related genes (*ZIP1/5/10*), and copper-transport-related genes (*COPT1*) were upregulated in Cj under alkaline stress (Table 1). However, iron-transport-related genes (*FIT1*, *FRO2/4*, Ferritin1, and vacuolar iron transport 1) were largely downregulated at pH 9.5 in Pt (Supplementary Figure S4b, Table 1). Zinc-transport-related genes (*ZIP5/10*) and copper-transport-related genes (*COPT1/6*) were also upregulated in Pt under alkaline stress. In addition, six aquaporin genes (two *TIP1-3s*, two *PIP2-2s*, *TIP2-2*, and *NIP2-1*) were largely downregulated in Pt at pH 9.5 (Supplementary Figure S4b, Table 1). These results suggest that Pt may be more susceptible to iron and water deficiencies under alkaline stress.

### Comparing Cj and Pt transcriptomes

**Fig. 5:**
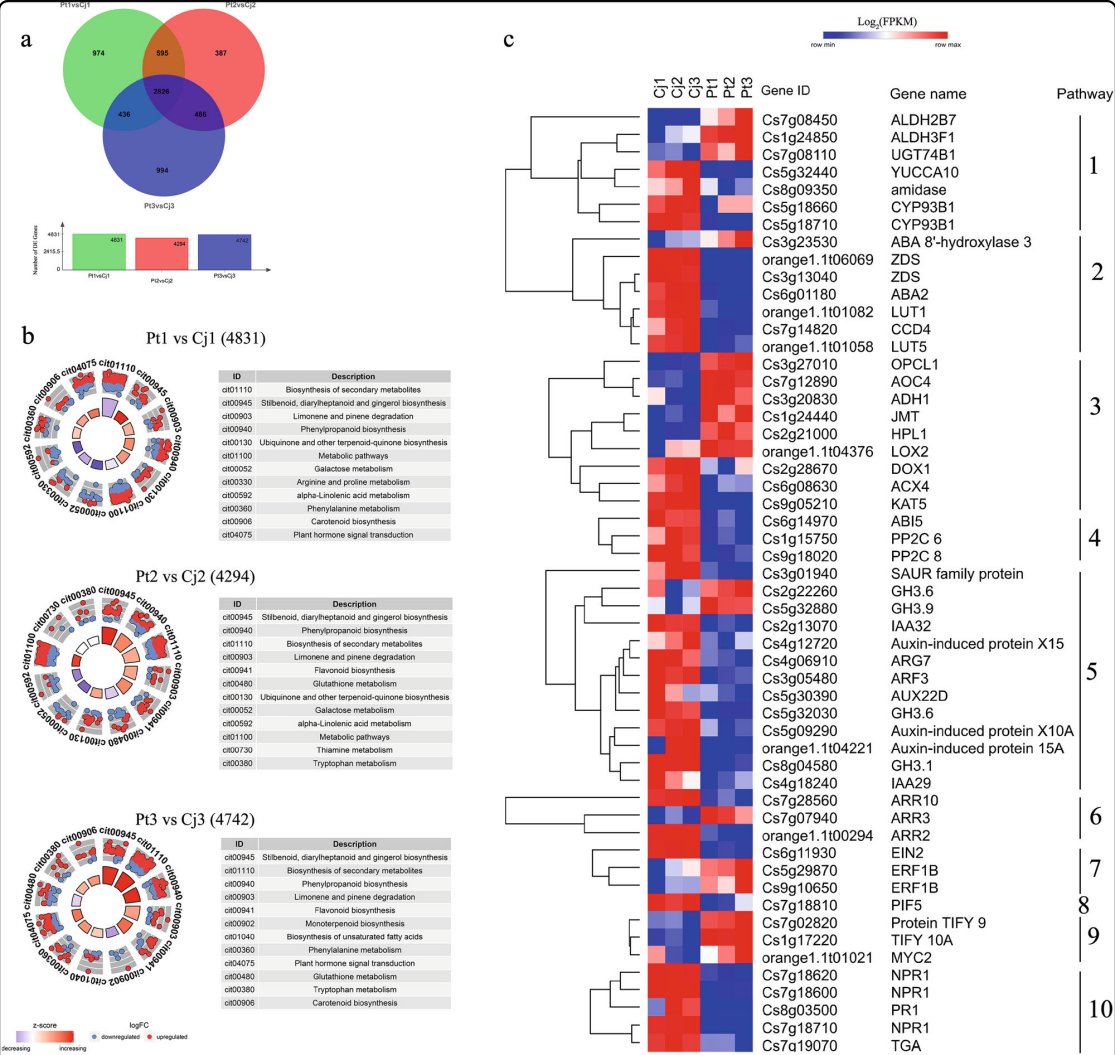
Gene expression comparisons between Cj and Pt. **a** A Venn diagram depicting the number of common and unique DEGs among the three comparison groups. **b** Circular visualization of the results of the KEGG pathway enrichment of DEGs from the different comparison groups between Cj and Pt. **c** A heatmap depicting the expression patterns of DEGs in several key pathways across all six samples. Pathways: 1, Auxin biosynthesis; 2, Carotenoid biosynthesis; 3, alpha-Linolenic acid metabolism; 4, ABA signal transduction; 5, Auxin signal transduction; 6, Cytokinin signal transduction; 7, Ethylene signal transduction; 8, Gibberellin signal transduction; 9, JA signal transduction; 10, Salicylic acid signal transduction. DEG differentially expressed gene

Cj is more tolerant to alkaline stress compared with Pt in citrus production. To compare the differences of Cj and Pt at the transcriptome level, we identified some key pathways and genes involved in the response to alkaline stress. As shown in Fig. 5a, 4831, 4294 and 4742 DEGs were identified in the Pt1 vs. Cj1, Pt2 vs. Cj2 and Pt3 vs. Cj3 comparison groups, respectively. The DEGs of these three groups were subject to GO-based term and KEGG-based pathway enrichment analyses (Supplementary Tables S6 and S7). The top 12 most enriched pathways and DEGs enriched in these pathways were visualized using the GOplot package^46^ (Fig. 5b). The top 4 most enriched pathways were the same in the three comparison groups. As the pH increased, the number of upregulated DEGs in the top 4 pathways increased (Fig. 5b). This result indicates that there are intrinsic differences in these pathways between Cj and Pt, which are magnified under alkaline stress. In particular, the “Biosynthesis of secondary metabolites” pathway was largely altered by alkaline stress in both Cj and Pt (Figs. 3b, d and 5b). In addition, the “Phenylpropanoid biosynthesis”, “alpha-Linolenic acid metabolism”, “Carotenoid biosynthesis”, and “Plant hormone signal transduction” remained enriched in these three or two comparison groups, which was consistent with the above result (Figs. 3b, d and 5b). In addition, the auxin biosynthesis pathway (“Tryptophan metabolism” pathway) was enriched in the Pt2 vs. Cj2 and Pt3 vs. Cj3 comparison groups. Therefore, we further analyzed the expression trends of the DEGs that were distributed in these five pathways and were differentially expressed in all three comparison groups. As shown in Fig. 5c and Supplementary Figure S4c, the expression of these genes exhibited significant differences between Cj and Pt. In the plant hormone signal transduction pathway, auxin signal genes comprised the largest group, and most of these genes (11/13) were downregulated in Pt. The ABA, ETH, JA, CK, and SA signaling genes were also differentially expressed between Cj and Pt. Moreover, several DEGs were enriched in the auxin, ABA, and JA biosynthesis pathways (Fig. 5c). In the phenylpropanoid biosynthesis pathway, the expression of most of the genes (28/40) in the Pt was reduced compared with that of the genes in Cj, and most of these genes were peroxidases (Supplementary Figure S4c). These results further demonstrate that auxin, ABA, JA signal pathways, and peroxidases may play important roles in tolerance to alkaline stress.

According to the results of the GO enrichment analysis, “response to stimulus” was the most enriched term (Supplementary Figure S3, Table S7). A total of 118 DEGs were enriched in the “response to stimulus” term in both the Pt2 vs. Cj2 and Pt3 vs. Cj3 comparison groups. As shown in Supplementary Figure S4d, the genes responding to the stimulus included disease resistance proteins; plant hormone biosynthesis and signal transduction genes; ion transport genes; and cell wall metabolism genes, such as *NCED1*, *LOX3*, *FRO2/4*, and peroxidases. Among these DEGs, TFs with increased expression in Cj3 or Pt3 included six ERF genes (*ERF012*, *ERF110*, Dehydration-responsive element-binding protein 1D, *ERF024*, *ERF109*, and *ERF5*), two bHLH genes (*FIT1* and *ORG2*), two C2H2 genes (*ZFP1* and *ZAT11*), *WRKY46*, *HRA1* and chitin-inducible gibberellin-responsive protein 1 (Supplementary Figure S4d, Table 1). *FIT1* is an essential gene that regulates iron uptake in *Arabidopsis thaliana*^37^, and *WRKY46* modulates the growth of *Arabidopsis* lateral roots under osmotic/salt stress conditions via regulation of ABA signaling and auxin homeostasis^48^. Hence, these TFs may also play important roles in citrus in response to alkaline stress.

### Identification and expression analysis of miRNAs in the roots of Cj and Pt

**Fig. 6:**
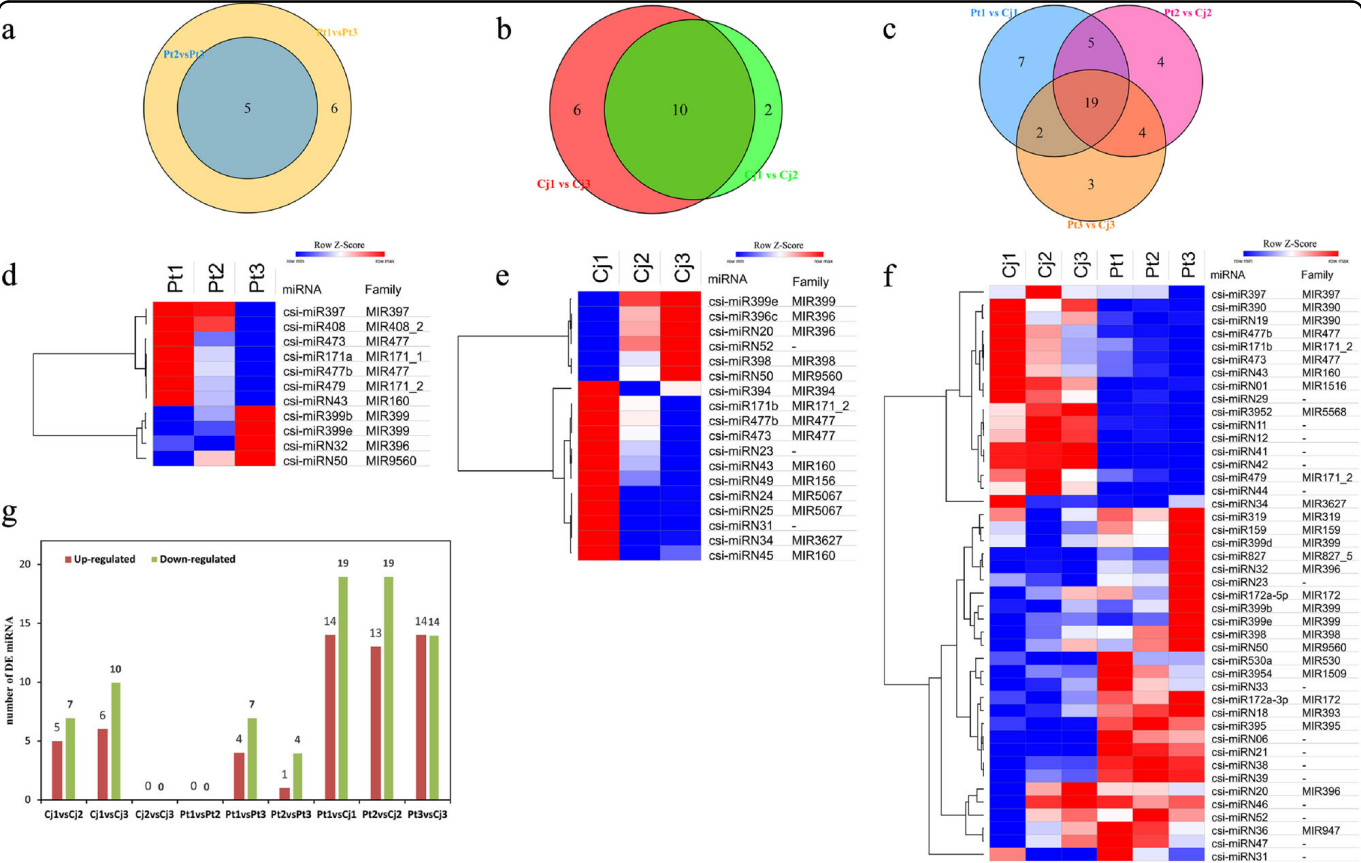
Global analysis of differentially expressed miRNAs. **a**, **b**, and **c** Venn diagrams depicting the number of differentially expressed miRNAs in different comparison groups. **d**, **e**, and **f** Heatmaps depicting the expression patterns of differentially expressed miRNAs across Cj samples, Pt samples and all six samples, respectively. **g** The number of differentially expressed miRNAs in each comparison group

A total of 101 miRNAs (49 known miRNAs and 52 novel miRNAs) belonging to 37 families were identified from the six samples of Cj and Pt (Supplementary Table S8). Only the miRNAs identified in all three biological replicates were retained. All precursors of novel miRNAs exhibited regular stem-loop secondary structures. The miRNA sequences are shown in blue, and the sequences of the miRNAs* are shown in red (Supplementary Figure S5). Based on the criteria of significant difference (FDR < 0.05), 12, 16, 5, 11, 33, 32, and 28 DE miRNAs (differentially expressed miRNAs) were identified in the Cj1 vs. Cj2, Cj1 vs. Cj3, Pt2 vs. Pt3, Pt1 vs. Pt3, Pt1 vs. Cj1, Pt2 vs. Cj2, and Pt3 vs. Cj3 comparison groups, respectively (Fig. 6g, Supplementary Table S9). However, no DE miRNA was identified in the Cj2 vs. Cj3 and Pt1 vs. Pt2 comparison groups, indicating that the difference between Cj2 and Cj3 or Pt1 and Pt2 was minimal (Fig. 6g). This result was consistent with the transcriptome results (Figs. 3a, c and 6g). Venn diagrams and heatmaps were used to demonstrate the inclusion relations and expression patterns of DE miRNAs in these seven comparison groups (Fig. 6a–f), respectively. In total, 11 and 18 DE miRNAs were identified among the different pH conditions in Pt and Cj, respectively, and the number of downregulated miRNAs was much more than that of upregulated miRNAs in both Cj and Pt (Fig. 6a, b, d, e). A total of 44 DE miRNAs was identified between Cj and Pt, and the expression of most of the DE miRNAs in Pt was higher than that of the DE miRNAs in Cj (Fig. 6c, f).

### Identification of *PHAS* genes

From the eighteen deep sequencing sRNA libraries of Cj and Pt, 28 *PHAS* genes (the gene targeted by miRNAs to generate phasiRNAs) were identified that were capable of secondary siRNA production, with a *P*-value ≤0.0001 (Supplementary Table S13). In total, 24 *PHAS* genes that encoded resistance proteins were targeted by the MIR482 family (csi-482-3p, csi-482b/c, and csi-miR48). In addition, csi-miR393a triggered two TIR/AFB auxin receptor proteins, and csi-miR1515 triggered two dicer-like proteins (Supplementary Table S13). Moreover, the expression of csi-miR393a in Pt was higher than that in Cj under all three conditions (Supplementary Table S8). In this study, many genes in the auxin signal pathway were significantly altered by alkaline stress in both Cj and Pt, including *TIR1* (Table 1). Hence, this result suggests that csi-miR393a may be involved in the regulation of auxin signals in response to alkaline stress.

### Identification and functional analysis of the targets of miRNAs

From the degradome data, a total of 260 transcripts from 150 genes were predicted to be targeted by 62 miRNAs (39 known miRNAs and 23 novel miRNAs) in Cj, with 416 miRNA-target pairs (Supplementary Figure S6, Table S10). A total of 215 transcripts from 121 genes were predicted to be targeted by 56 miRNAs (38 known miRNAs and 18 novel miRNAs) in Pt, with 353 miRNA-target pairs (Supplementary Figure S7, Table S10). These miRNA-transcript pairs were classified into five categories (Category 0, 1, 2, 3, and 4) based on the confidence evaluation of the degradome data. In Categories 3 and 4, the miRNA-target pairs are not reliable; therefore, only those miRNA-target pairs that were distributed in Categories 0, 1, and 2 were retained. Finally, we identified 157 target genes (128 targets from Cj and 103 from Pt) for 58 miRNAs (52 miRNAs of Cj and 49 miRNAs of Pt) (Supplementary Figure S8a, b).

**Fig. 7:**
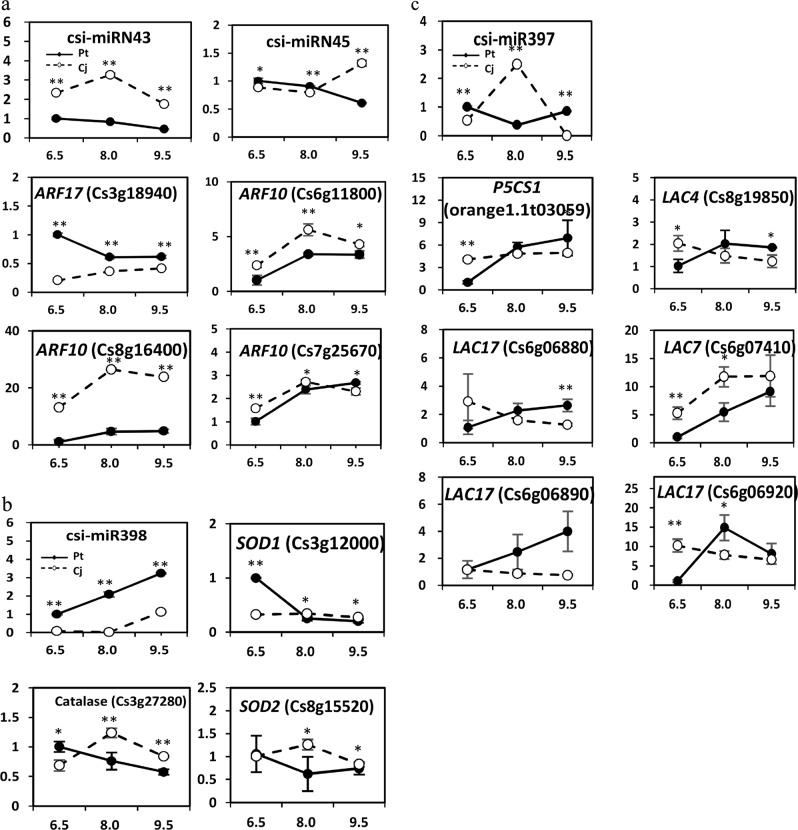
Expression patterns of miRNAs and their target genes in Cj and Pt under different pH conditions. **a** csi-miRN43/45; **b** csi-miR398; **c** csi-miR397. A single asterisk (*) represents statistically significant differences (*P* < 0.05), and double asterisks (**) represent highly statistically significant differences (*P* < 0.01), analyzed using Student’s *t*-test

To further elucidate the roles of miRNAs in response to alkaline stress, the targets of the DE miRNAs were used to perform GO-based term and KEGG-based pathway enrichment analyses. These targets of the DE miRNAs of each comparison group are listed in Supplementary Table S11 and are shown in Supplementary Figure S8c, d. Given that the number of targets was small in each set, no enrichment pathways were identified. According to the GO enrichment analysis results, several GO terms were highly enriched, including the response to stimulus, root cap development, lignin catabolic process, and reactive oxygen species metabolic process (Supplementary Figure S9, Table S12). The expression patterns of several important miRNAs and their targets were verified in Cj and Pt (Fig. 7). The targets of csi-miRN43/45 included four ARFs (*ARF10/17*) that are important TFs for root cap development in *Arabidopsis*^49^ (Fig. 7a). *LAC4/7/17/22* and *P5CS1* are the targets of csi-miR397 (Fig. 7c). *LACs* are involved in lignin catabolism, and *P5CS1* is a key gene for proline biosynthesis that plays important roles in scavenging reactive oxygen species (ROS)^50^. The csi-miR398 targeted several ROS scavenging genes, such as *SOD1/2* and catalase (Fig. 7b). These results further suggest that the auxin pathway and ROS scavenging system may be important in the resistance to alkaline stress.

### Changes of phytohormone and antioxidants in Cj and Pt under alkaline stress

**Fig. 8:**
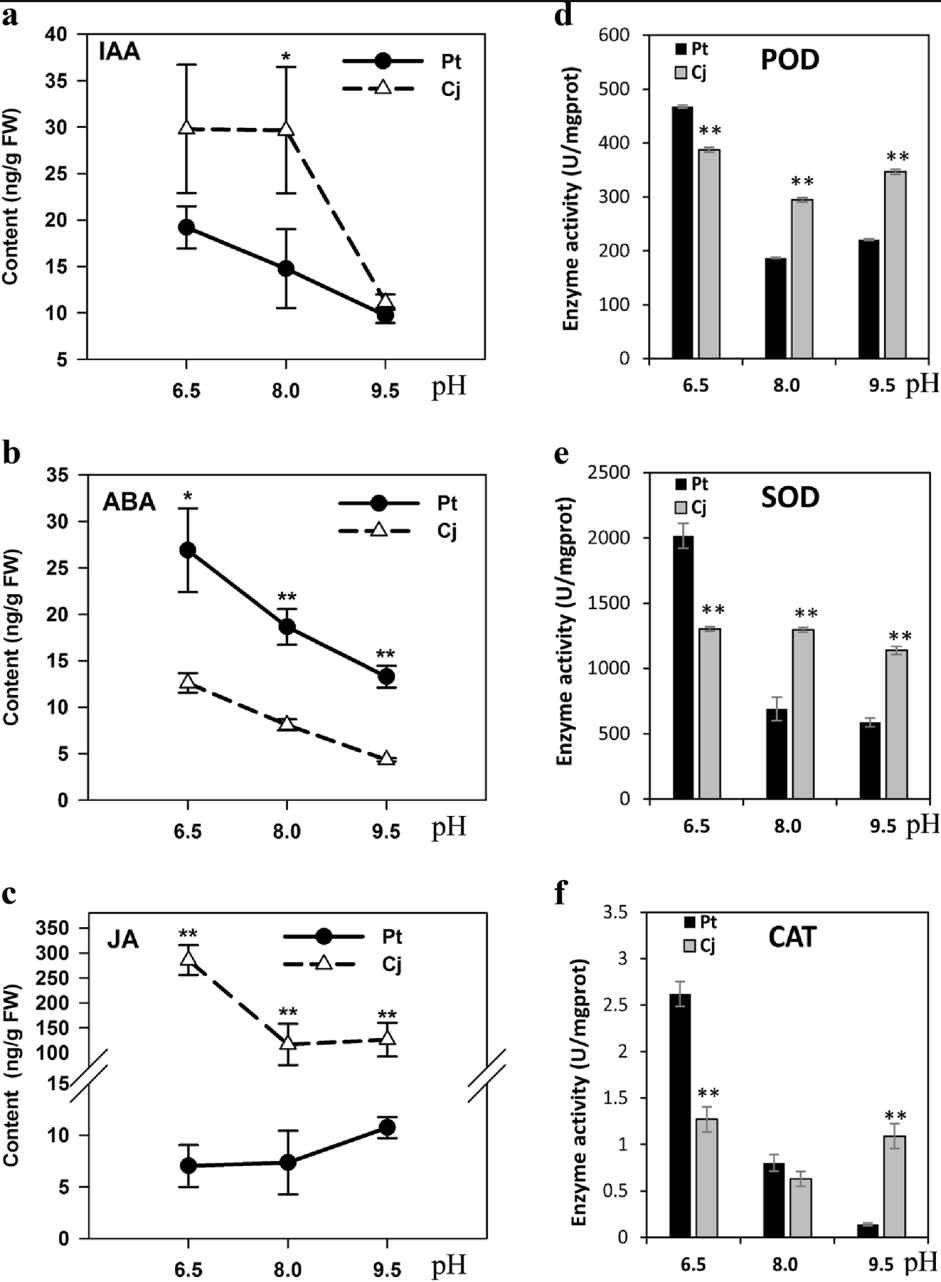
Content of several types of plant hormones. **a**–**c** and antioxidants **d**–**f** in the roots of Cj and Pt under different pH conditions. A single asterisk (*) represents statistically significant differences (*P* < 0.05), and double asterisks (**) represent highly statistically significant differences (*P* < 0.01), analyzed using Student’s *t*-test

According to the comprehensive analysis of transcriptomes and small RNAs of Cj and Pt, the auxin, ABA, and JA signal pathways and antioxidants may play more important roles in tolerance to alkaline stress. To further verify the difference of phytohormone and antioxidants between Cj and Pt in response to alkaline stress, we measured the JA, ABA, IAA, POD, SOD, and CAT levels in the roots of Cj and Pt under different pH conditions. As shown in Fig. 8a, the IAA levels in Cj were significantly higher than those in Pt at pH 8.0, and no significant difference was noted between the IAA levels in Cj and Pt under the other two conditions. The ABA levels in Cj were significantly lower than those in Pt under all three pH conditions (Fig. 8b). The IAA and ABA levels decreased in both Cj and Pt as the pH increased. The JA levels in Cj were much higher than those in Pt (from approximately 12-fold to approximately 40-fold) under all three pH conditions, and the JA levels in Pt increased as the pH increased. By contrast, the JA levels in Cj were downregulated under high pH conditions (Fig. 8c). Regarding antioxidants, the levels of POD, SOD, and CAT decreased markedly in Pt as the pH increased; however, the levels of these antioxidants were minimally altered in Cj (Fig. 8d–f). These results are consistent with the results of the bioinformatics analysis.

### Exogenous JA and auxin analogs enhance the tolerance of citrus to alkaline stress

**Fig. 9:**
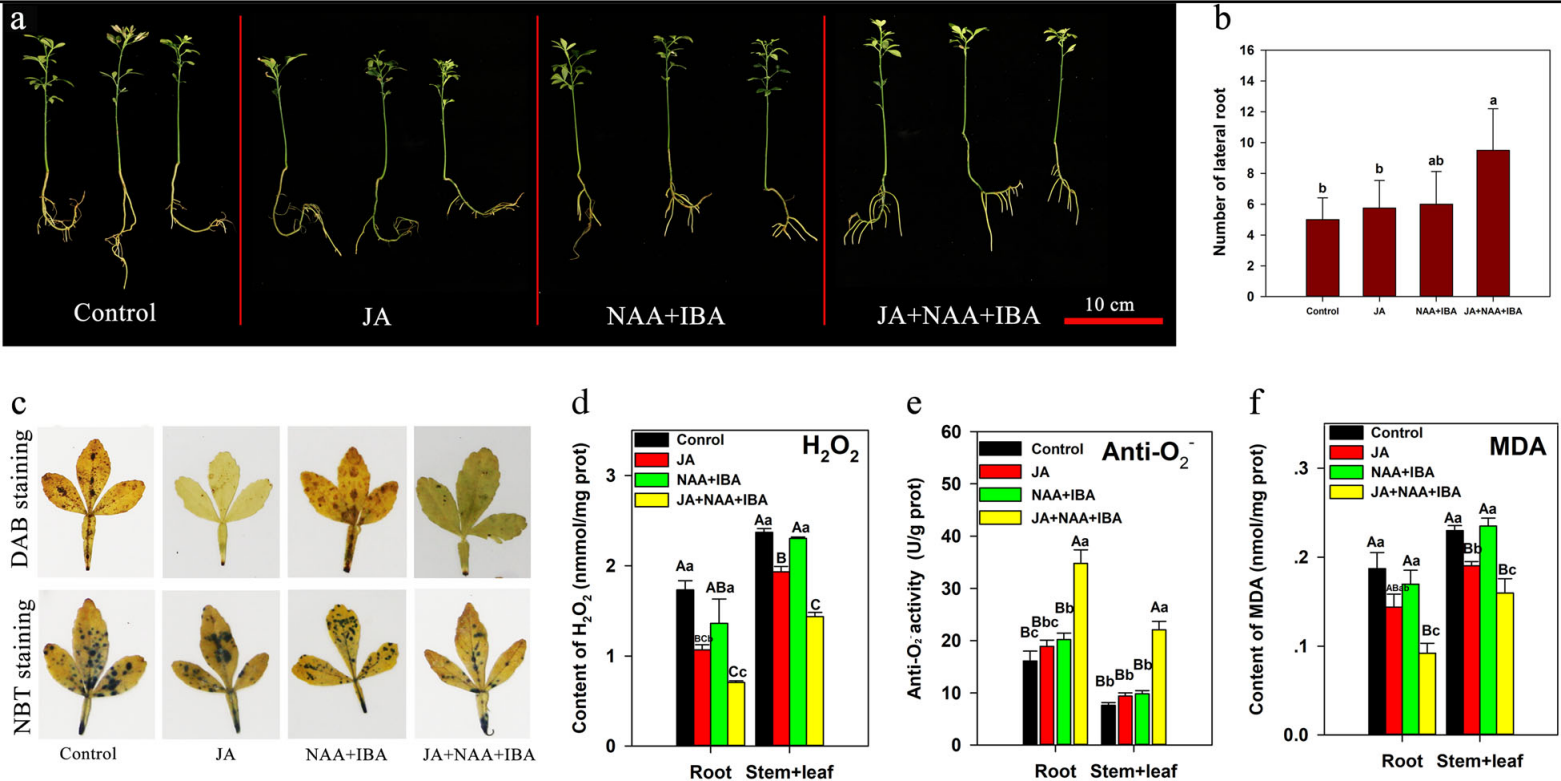
Exogenous JA, NAA, and IBA treatment of Pt seedlings under alkaline stress. **a** The phenotypes. **b** Number of LRs of Pt seedlings cultured at pH 8.5 for 12 weeks, and three treatment groups (JA (5 μM), NAA + IBA (0.5 mg/L + 0.1 mg/L) and JA + NAA + IBA (5 μM + 0.5 mg/L + 0.1 mg/L)) were defined. **c** In situ accumulation of H_2_O_2_ and O_2_·^–^; examined by histochemical staining with DAB and NBT. **d**–**f** The content of H_2_O_2,_ anti-O_2_·^–^ and MDA. Lowercase and capital letters represent statistically significant differences (*P* < 0.05) and highly statistically significant differences (*P* < 0.01), respectively. Data were analyzed using one-way ANOVA

To further verify the roles of JA and auxin in alkaline stress tolerance in citrus, we designed a treatment experiment using JA and auxin analogs (NAA and IBA) to treat Pt seedlings at pH 8.5 (Fig. 9a). After 12 weeks of culture, the JA + NAA + IBA-treatment group exhibited the best growth, generating the largest number of LRs with healthy root caps and the lowest ROS level (Fig. 9). In addition, the status of the JA-treatment group was not good, which generated tiny short LRs with necrotic root caps; whereas, the ROS levels were lower than those of the control and NAA + IBA-treatment groups (Fig. 9). This result demonstrated that JA played a positive role in scavenging ROS and a negative role in LR growth and formation under alkaline stress. Moreover, the simultaneous application of auxin and JA to Pt can promote LR growth and scavenge ROS under alkaline stress. These results indicated that auxin and JA synergistically played important roles in the tolerance of citrus to alkaline stress.

## Discussion

Rootstock is important for the citrus industry and can significantly affect the characteristics of scions^39^. Cj has increasingly been used as rootstock for many scion cultivars in recent years in China given its excellent tolerance to alkaline stress and iron deficiency. However, the molecular mechanism has rarely been elucidated. Our work reported here provides crucial molecular insights into the tolerance of Cj to alkaline stress. The comprehensive transcriptomes and small RNA data sets of Cj and Pt along a pH gradient enabled genome-scale analyses at a high resolution. The genome-scale approach allowed us to examine the expression profiles of all of the members of gene/miRNA families in an unbiased manner and to simultaneously analyze multiple pathways. In addition, the integrated analysis of the transcriptomes and small RNAs provided more information from different regulation levels in response to alkaline stress.

### The auxin pathway may play a central role in hormonal regulation of LR development in citrus in response to alkaline stress

In this study, alkaline stress largely inhibited the formation and growth of LR in both Cj and Pt. In *Arabidopsis*, auxin serves as an integrator of numerous signals that are involved in LR development, such as ABA, CK, JA, and nutrients. These signals affect the LR development by interacting with auxin homeostasis (synthesis, conjugation, and degradation), transport, or response^9,16,29,51^. In this study, the LR development of Cj and Pt was closely related to the content of IAA (Figs. 1a, b and 8a). Moreover, a number of genes/miRNAs related to the auxin signal transduction pathway were differentially expressed in the roots of Cj and Pt under gradient alkaline stress, such as *IAA14* (Cs1g13960), csi-miR43 and others (Figs. 4 and 6, Supplementary Figure S2). A previous study reported that *IAA14* is a key regulator in auxin-regulated growth and development, particularly in lateral root formation and that the *slr-1/iaa14* mutant of *Arabidopsis* completely lacked lateral roots^52^. In addition, under alkaline stress, treatment with exogenous JA, IBA, and NAA significantly increased the number of LRs (Fig. 9). These results revealed that auxin played an important role in citrus lateral root development under alkaline stress. Moreover, in this study, the expression of genes involved in IAA biosynthesis was slightly altered in Cj and Pt under gradient alkaline stress. However, some GH3 genes that function in conjugating amino acids to IAA^53–55^ were upregulated at pH 9.5 in Cj and differentially expressed between Cj and Pt, such as *GH3.6* (Cs2g22260) and *GH3.9* (Cs5g32880) (Figs. 4 and 5c). This result suggests that the regulation of IAA homeostasis may contribute to alterations of the free IAA content in Cj and Pt. Overexpression *GH3.6* in *Arabidopsis* severely reduces the number of prebranch sites and LRs^56^. Moreover, several IAA-amino acid conjugates function in inhibiting root elongation, such as IAA-Ala and IAA-Leu^57^.

Lateral root formation and development appear to be the result of the balancing effects of auxin-promoting and ABA-repressive signaling pathways. For instance, ABA inhibits LR formation by reducing the expression of the TIR1/AFB auxin receptor through miR393 upregulation^58^ and upregulating the expression of *ABI4*, which functions to repress *PIN1*^13^. In this study, several key ABA biosynthesis genes were downregulated under alkaline conditions, and the ABA levels were also reduced in Cj and Pt (Figs. 2 and 8b). However, the ABA levels in Pt were higher than those in Cj for all three conditions (Fig. 8b). This result indicates that ABA may also play an important role in LR development under alkaline stress by interacting with the auxin pathway.

### Jasmonate may play a significant role in tolerance to alkaline stress

Previous studies have demonstrated that JA is closely related to salt stress^19,20^; however, few studies have assessed the relationship between JA and alkaline stress. In this study, under alkaline stress, exogenous JA treatment could significantly reduce the level of H_2_O_2_ and MDA in Pt, and JA with auxin analogs treatment could promote LR generation and growth (Fig. 9). These results showed a positive role of JA in alkaline stress responses in citrus. However, under alkaline stress, exogenous JA treatment played a negative role in LR formation and growth in Pt (Fig. 9a, b). In addition, the expression of key genes involved in JA biosynthesis and signal transduction and the JA levels also showed opposite patterns in Cj and Pt in response to alkaline stress; these genes included *LOX3* and *MYC2*, which were downregulated in Cj, but upregulated in Pt (Table 1). This result elucidated that JA signaling was enhanced in Pt, but weakened in Cj under alkaline stress. Jasmonate-induced primary root growth inhibition has been well studied in Arabidopsis. JA reduces both the cell number and cell length of the root through *MYC2* repressing the expression of *PLT1* and *PLT2*^59^. On the other hand, JA inhibits primary root growth and regulates LR formation in a dose-dependent manner, with low micromolar concentrations of JA inhibiting the primary root growth and promoting LR formation in Arabidopsis^30,31^. Moreover, JA is involved in LR growth inhibition under Al stress and salt stress^60,61^. Hence, in this study, weakened JA signaling in Cj may play a positive role in the tolerance to alkaline stress. The differential response to exogenous JA may be due to the dose-dependent activity of JA.

In addition, many TFs, such as *ZAT12*, *WRKY46*, *FIT1*, *ERF105*, and others, also exhibited opposite patterns in Cj and Pt (Supplementary Figure S1d, Table 1, Supplementary Table S3). The expression patterns of these TFs were closely related with the JA levels in Cj and Pt. This result indicated that interactions may exist between these TFs and the JA pathway. Ding et al.^48^ reported that *WRKY46* contributes to the feed-forward inhibition of osmotic/salt stress-dependent LR inhibition in *Arabidopsis* via regulation of ABA signaling and auxin homeostasis. *ZAT12* is induced by H_2_O_2_ and functions as a negative regulator of iron acquisition by repressing *FIT* expression^62^.

### ROS metabolic process is important for citrus to resist alkaline stress

When plants are exposed to stress conditions, reactive oxygen species (ROS) are generated^63^. A lower level of ROS following stress exposure is generally regarded as better tolerance. ROS scavenging enzymes, such as POD, SOD, and CAT, are indispensable for ROS detoxification so that plants can combat the ROS-associated cellular damage and maintain better survival under stressful conditions^64^. In this study, the expression of genes/miRNAs of ROS metabolic process were affected by alkaline stress in Cj and Pt, such as peroxidase genes and csi-miR398 (Figs. 4 and 7b, Supplementary Figure S2). Moreover, most ROS metabolic process genes/miRNAs were more downregulated/upregulated in Pt than those in Cj in response to alkaline stress (Figs. 4 and 7b, Supplementary Figure S2). In addition, the SOD, POD, and CAT levels were also much higher in Cj than those in Pt under alkaline stress, and these antioxidant levels changed slightly in Cj in response to alkaline stress (Fig. 8d–f). Therefore, these results showed the ROS scavenging system of Cj was stronger than that of Pt under alkaline stress. Several studies have shown that exogenous JA treatment of soybean significantly increases the activities of SOD, POD, APX, and CAT under salt or drought stress^65,66^. In this study, under alkaline stress, exogenous JA treatment could significantly reduce the levels of MDA and H_2_O_2_ in Pt seedlings (Fig. 9d, f). Hence, the interaction between JA and ROS metabolic process may play roles in the tolerance of citrus to alkaline stress.

### Differential response of Cj and Pt to iron deficiency under alkaline stress

Cj is more tolerant to iron deficiency compared with Pt under alkaline stress. In this study, a number of genes related to iron acquisition were differentially expressed among the different pH conditions in Cj and Pt, including *FIT1* and *FRO2/4/6* (Table 1). Moreover, these genes were upregulated in Cj but downregulated in Pt as the pH increased (Table 1, Supplementary Figure S4a, b). This result indicates improved iron acquisition in Cj compared with Pt under alkaline stress. Previous studies demonstrated that FIT plays a central role in iron acquisition^38^. FRO2 and IRT1 are targets of FIT that regulate *FRO2* at the level of mRNA accumulation and IRT1 at the level of protein accumulation^37^. FIT expression is upregulated by iron deficiency^37^ and is downregulated by jasmonate^67,68^. In this study, the JA levels were decreased in Cj but increased in Pt as the pH increased (Fig. 8c). This result suggests that *JA* may repress *FIT1* expression in citrus under alkaline stress. The interaction between *FIT1* and JA should be studied in the future.

Taken together, our results demonstrate that the inhibitory effect of alkaline stress on root growth involves the auxin, ABA and JA signaling pathways in citrus. Regulation of auxin homeostasis under alkaline stress is important for adapting to alkaline stress in citrus. Moreover, the JA pathway, which exhibits the opposite response to alkaline stress in Cj and Pt, may contribute to the differences in the alkaline stress and iron deficiency tolerance between Cj and Pt. Further studies are needed to elucidate the regulation network of JA in resistance to alkaline stress and iron deficiency in citrus.

## Materials and methods

### Plant materials, growth conditions, and sample collections

According to the method described by Zhou et al.^69^, the seeds of Cj and Pt were surface sterilized, and their germination was accelerated. The seedlings were grown in a growth chamber until they had four leaves. Then, seedlings were selected based on a uniform size and transferred into a hydroponics system with a 4 L solution in a greenhouse. Three different pH gradients (6.5, 8.0, and 9.5) were established. Given that most natural soils have weak acidity, we used pH 6.5 as the normal condition. After 8 weeks of culture, three biological replicates (three seedling plants per replicate) were collected randomly for each treatment. Cj1, Cj2, and Cj3 represented Cj seedlings cultured at pH 6.5, 8.0, and 9.5, respectively. A similar naming scheme was employed for the Pt seedlings. The roots, stems, and leaves of the seedlings were sampled separately. A portion of root samples was scanned with an Epson digital scanner (Expression 10000XL 1.0, Epson, Inc., Japan), and WinRhizo Pro (S) v. 2009c (Regent Instruments, Inc., Canada) software was used to analyze the root morphology. Another portion of samples were frozen by liquid nitrogen and immediately stored under −80 ℃ for other experiments.

### Measurement of mineral element concentrations

The plant mineral element concentrations were measured as described in previous studies^69,70^, with some modification. Briefly, fresh samples were placed into a forced air oven at 105 °C for 30 min, and then at 75 °C until a constant weight was reached to determine the sample dry weight. All the dried samples were ground into fine powder. Then, 0.50 g of each sample was dry-ashed in a muffle furnace at 200 °C for 1 h, 300 °C for 1 h, and 500 °C for 8 h, followed by dissolution in 10 mL 0.1 N HCl. The mineral elements (except nitrogen) were determined using inductively coupled plasma atomic spectroscopy (ICP-AES; Thermo, Inc., IRIS Advan, USA). The total nitrogen content of the plant samples, the pH of the soil samples and the soil mineral element concentrations were measured using the methods described by Bao^71^. Each sample was assayed using three replicates.

### RNA-seq, data processing, and gene annotation

Total RNA isolation was performed as described previously^72^. Six samples of the root tissues of Cj and Pt were collected, and three biological replicates were collected for each sample. A total of 18 transcriptomes, 18 small RNAs and two degradomes profiles were obtained by RNA-seq using Illumina HiSeq X-ten at Beijing Biomarker Technologies Limited Company (Beijing) in 2017. The sequencing raw data have been submitted to NCBI Gene Expression Omnibus (GEO), and the accession number is GSE115050. The method for data processing and gene annotation was listed in Supplementary Methods 1.

### Expression data analysis

The dendrogram in Fig. 2a was made by the function *cor*() in R with default settings. The *y*-axis is computed as 1 minus cor (correlation) to reflect the degree of variance. Log_2_ (FPKM) was used in the computation. The expression modules in Fig. 2c, d were generated by the Short Time-series Expression Miner (v1.3.7) (STEM)^45^ based on FPKM, with the STEM Clustering Method, with default settings used for the Filtering, Model Profiles and Clustering Profiles. Figure 3b, d was generated by the function *GOCircle* () of R package GOplot (1.0.2)^46^. Hierarchical clustering of the gene sets was performed using Morpheus (https://software.broadinstitute.org/morpheus/) with one minus the Pearson correlation as the distance metric and average as the linkage method. Log_2_ (FPKM) of the differentially expressed genes from the comparison groups was used for clustering. The *Z*-score in here was calculated for each miRNA per sample using the formula (*X*–*X*_av_)/SD, where *X* is the TPM value in a particular sample, and *X*_av_ and SD are the mean and standard deviation of the TPM values across all samples used for clustering, respectively.

### Verification of miRNA and target gene expression by qRT-PCR

Stem-loop qRT-PCR was performed to validate the expressions of miRNAs with three biological replicates based on our previous method^73^. U6 was used as the endogenous reference gene^74^. Next, qRT-PCR was performed with three biological replicates according to our previous study^75^, and *CsActin* was used as the endogenous reference gene^76^. The primers are listed in Supplementary Table S14. Data are presented as the means ± standard error (SE) (*n* = 3).

### Quantification of ABA, JA, and IAA

The samples for ABA, JA, and IAA quantification were prepared according to the method described by Pan et al.^77^, with slight modifications, which were described in detail in our previous study^75^. For this purpose, d_6_-ABA (Icon Isotopes, cat. no. ID1001) was used as an internal standard for ABA, d_5_-IAA (Aldrich, cat. no. 492817) was used as an internal standard for IAA, and H_2_JA (dihydrojasmonic acid, OlChemim, cat. no. 0145324) was used as an internal standard for JA. The reaction monitoring conditions (Q1/Q3) for ABA and d_6_-ABA, IAA and d_5_-IAA, and JA and H_2_JA were 262.8/152.6 and 269.1/158.8, 174.0/129.6 and 179.0/134.8, and 209.0/59.0 and 211.0/58.8, respectively. Each sample was characterized using four replicates.

### Physiological analyses and histochemical staining of ROS

Malondialdehyde (MDA) levels were determined according to the method described by Liu et al.^78^. Peroxidase (POD), superoxide dismutase (SOD), catalase (CAT) and H_2_O_2_ were measured using relevant detection kits (A064-1 for H_2_O_2_, A084-3 for POD, A001-1 for SOD, and A007-1 for CAT, Nanjing Jiancheng Bioengineering Institute, Jiangsu, China) following the manufacturer’s instructions. Anti-O_2_·^–^ measurement and total protein concentration measurement were performed according to Geng and Liu^64^. Histochemical staining with 3,3’-diaminobenzidine (DAB) and nitroblue tetrazolium (NBT) was used to examine the in situ accumulation of H_2_O_2_ and O_2_·^–^, respectively^79^.

### JA, naphthylacetic acid (NAA), and indolebutyric acid (IBA) treatment of the seedlings of Cj and Pt

A stock solution of 1 mM JA was made in ethanol and diluted with water before application. For the Pt seedlings cultured at pH 8.5, we set up the JA (5 μM), NAA + IBA (0.5 mg/L + 0.1 mg/L) and JA + NAA + IBA (5 μM + 0.5 mg/L + 0.1 mg/L) treatment groups, and the seedlings were cultured for 12 weeks. The solution was replaced every week.

## Supplementary information

Instruction of Supplementary information

Supplementary methods 1

Figure S1

Figure S2

Figure S3

Figure S4

Figure S5

Figure S6

Figure S7

Figure S8

Figure S9

Table S1

Table S2

Table S3

Table S4

Table S5

Table S6

Table S7

Table S8

Table S9

Table S10

Table S11

Table S12

Table S13

Table S14
