# Supplementary material for: Genome-wide comprehensive analysis of transcriptomes and small RNAs offers insights into the molecular mechanism of alkaline stress tolerance in a citrus rootstock
Source: Hortic Res. 2019 Mar 1;6:33. doi: 10.1038/s41438-018-0116-0 (PMC6395741; doi:10.1038/s41438-018-0116-0)
Supplement: Supplementary file 1 — Instruction of Supplementary information [file 41438_2018_116_MOESM1_ESM.docx]

**Supplementary information**

**Figure S1.** Genes and TFs of the Cj up, Cj down, Pt up and Pt down clustering profiles generated by STEM.

(a) Venn diagram revealing the overlapping sets of genes among the Cj up, Cj down, Pt up and Pt down clustering profiles. (b) Families of TFs identified in the Pt up and Pt down clustering profiles. (c) Families of TFs identified in the Cj up and Cj down clustering profiles. (d) Heatmap demonstrating the expression patterns of the TFs identified in all four clustering profiles.

**Figure S2.** Heatmaps demonstrating the expression patterns of plant hormone signal transduction and phenylpropanoid biosynthesis pathway genes identified in the Cj up, Cj down, Pt up and Pt down clustering profiles**.**

**Figure S3.** GO enrichment analysis of the differentially expressed genes in each comparison group of Cj and Pt. The bubble colour indicates the P-value; the plot size indicates the frequency of the GO term in the underlying GOA database (bubbles of more general terms are larger).

**Figure S4.** Heatmaps depicting the expression patterns of differentially expressed genes enriched in ion transport term (a and b), phenylpropanoid biosynthesis pathway (c) and response to stimuli term (d).

**Figure S5.** Predicted secondary structures of the novel miRNAs. Mature miRNA sequences are highlighted in blue, and miRNA* sequences are highlighted in red.

**Figure S6.** T-plots of the miRNA targets identified in Cj.

**Figure S7.** T-plots of the miRNA targets identified in Pt.

**Figure S8.** Venn diagrams revealing the number of target genes and miRNAs with identified target genes in Cj and Pt. (a) The number of miRNAs with identified target genes in Cj and Pt; (b) the number of all identified target genes in Cj and Pt; (c) the number of target genes of differentially expressed miRNAs of Cj and Pt; (d) the number of target genes of differentially expressed miRNAs between Cj and Pt.

**Figure S9.** Biological process enrichment analysis of the target genes of differentially expressed miRNAs in Cj (a) or Pt (b) or between Cj and Pt (c, d and e). The bubble colour indicates the P-value; the plot size indicates the frequency of the GO term in the underlying GOA database (bubbles of more general terms are larger).

**Table S1.** Summary of the RNA-seq results of the transcriptomes, small RNAs and degradomes.

**Table S2.** Expression of all genes identified in 18 transcriptomes.

**Table S3.** Genes and transcription factors identified in cluster profiles by STEM.

**Table S4.** Enriched KEGG pathways of genes in cluster profiles.

**Table S5.** Integration of DEGs of different comparison groups.

**Table S6.** Enriched KEGG pathways of DEGs of different comparison groups.

**Table S7.** Enriched Gene Ontology (GO) terms of the DEGs of different comparison groups.

**Table S8.** Known miRNAs and novel miRNAs identified in Cj and Pt.

**Table S9.** Differentially expressed miRNAs identified in different comparison groups.

**Table S10.** Targets of the miRNAs identified in Cj and Pt.

**Table S11.** Targets of differentially expressed miRNAs.

**Table S12.** Enriched Gene Ontology terms of the targets of differentially expressed miRNAs.

**Table S13.** Identified *PHAS* genes in the root of Cj and Pt.

**Table S14.** Primer sequences for stem-loop qRT-PCR and qRT-PCR.

**Supplementary methods 1.** Deep sequencing data analysis.
