# Supplementary material for: Genome-wide comprehensive analysis of transcriptomes and small RNAs offers insights into the molecular mechanism of alkaline stress tolerance in a citrus rootstock
Source: Hortic Res. 2019 Mar 1;6:33. doi: 10.1038/s41438-018-0116-0 (PMC6395741; doi:10.1038/s41438-018-0116-0)
Supplement: Supplementary file 2 — Supplementary methods 1 [file 41438_2018_116_MOESM2_ESM.docx]

**Supplementary Methods 1**

**Deep sequencing data analysis**

Clean reads (high quality reads) of 18 transcriptome libraries were filtered from raw reads by removing low-quality reads with ambiguous nucleotides and adaptor sequences, and Tophat2 ([Kim *et al.*, 2013](#_ENREF_4)) was used to map clean reads to the reference genome *C. sinensis* ([Xu *et al.*, 2013](#_ENREF_11)) (Table S1). Gene expression levels were calculated by the FPKM (Fragments Per Kilobase of transcript per Million mapped reads) method using the RSEM tool ([Li and Dewey, 2011](#_ENREF_6)). Differential expression analysis was performed using the DESeq R package (1.10.1) ([Anders and Huber, 2010](#_ENREF_1)). The resulting P values were adjusted using the Benjamini and Hochberg’s approach for controlling the false discovery rate. Genes with an adjusted P-value (FDR) < 0.05 found by DESeq were assigned as differentially expressed genes.

The raw reads of small RNA libraries were pre-processed to remove low-quality reads, adaptors and contaminants to obtain clean reads. The clean reads were used to search Silva database, GtRNAdb database, Rfam database (Rfam 13.0) and Repbase database by Bowtie ([Langmead *et al.*, 2009](#_ENREF_5)) to filter ribosomal RNA (rRNA), transfer RNA (tRNA), small nuclear RNA (snRNA), small nucleolar RNA (snoRNA) and other ncRNA and repeats. Then, reads matching the reference genome *C. sinensis* ([Xu *et al.*, 2013](#_ENREF_11)) were annotated (Table S1). These reads were used to detect known miRNA (comparing with miRBase 22 database) and novel miRNA predicted by miRDEEP2 ([Zhang *et al.*, 2015](#_ENREF_12)). The novel miRNAs were confirmed based on the criteria of a previous publication ([Meyers *et al.*, 2008](#_ENREF_7)) and secondary structure of the hairpins visualized by VisSR (version 1.0). Raw miRNA counts were normalized to TPM (reads per million) using the equation TPM = (Read count/Total mapped reads) × 1 000 000. The differential expression analysis was performed using the R-package DESeq (1.10.1) ([Anders and Huber, 2010](#_ENREF_1)). miRNAs with an adjusted P-value < 0.05 found by DESeq were assigned as differentially expressed miRNAs.

The raw data of the degradomes were trimmed by pre-analysis similar to the sRNAome raw data to obtain clean reads. The clean reads were used to search Silva database, GtRNAdb database, Rfam database (Rfam 13.0) and Repbase database by Bowtie ([Langmead *et al.*, 2009](#_ENREF_5)) to filter ribosomal RNA (rRNA), transfer RNA (tRNA), small nuclear RNA (snRNA), small nucleolar RNA (snoRNA) and other ncRNA and repeats. The remaining unannotated sequences were mapped to the reference genes (cDNA) of the *C. sinensis* genome ([Xu *et al.*, 2013](#_ENREF_11)) by Botwie (Table S1). The reads mapped to cDNA_sense were used to predict cleavage sites by PAREsnip (version 2.3) ([Folkes *et al.*, 2012](#_ENREF_3)). The cleaved target transcripts were categorized into five classes based on the abundance of degradome reads indicative of miRNA-mediated cleavage. Category 0 comprised the sequences whose abundance at the cleavage site was the only maximum on the transcript; in category 1, the reads abundance at the cleavage site was the maximum but not unique; category 2 consisted of sequences whose abundance at the cleavage site was higher than the median but not the maximum; category 3 included sequences whose abundance at the cleavage site was equal to or below the median; the remaining sequences, which were the only raw reads at the cleavage site, were classified as category 4. When the alignment score was no more than 4.5, the transcripts were considered as miRNA targets. The alignment score was calculated by the Rule-Based Complementarity Search algorithm ([Folkes *et al.*, 2012](#_ENREF_3)). The T-Plot figures were generated by VisSR (version 1.0).

Ta-si prediction tool (version 2.0), one tool of the UEA sRNA workbench ([Stocks *et al.*, 2012](#_ENREF_8)), was used to identify phasiRNAs and *PHAS* genes. The clean reads of MT and WT were supplied as sRNA dataset and the *C. sinensis* genome (Xu et al., 2013) was supplied as reference genome. The cutoff P-value calculated by the algorithm described by Chen et al. ([2007](#_ENREF_2)) was set as 1.0E-4. The sRNAs that did not match the genome were discarded, and only 21 nt sRNAs were used in the phasing analysis.

Gene annotation was conducted using the Blastp search against the nr database in NCBI, Swiss-Prot database and Pfam database. BlastKOALA was used to annotate the KOs of the KEGG ORTHOLOGY database. The protein sequences of genes were aligned against the GO database and KEGG pathway database using KOBAS 3.0 (http://kobas.cbi.pku.edu.cn/) ([Xie *et al.*, 2011](#_ENREF_10)) to perform the enrichment analysis. A corrected P-value < 0.01 was set as a cut-off for GO enrichment. REVIGO ([Supek *et al.*, 2011](#_ENREF_9)) was used to visualize and summarize the biological process, cell component and molecular function terms identified by KOBAS 3.0.

**Anders S, Huber W**. 2010. Differential expression analysis for sequence count data. *Genome Biol* **11**, R106.

**Chen HM, Li YH, Wu SH**. 2007. Bioinformatic prediction and experimental validation of a microRNA-directed tandem trans-acting siRNA cascade in Arabidopsis. *Proceedings of the National Academy of Sciences of the United States of America* **104**, 3318-3323.

**Folkes L, Moxon S, Woolfenden HC, Stocks MB, Szittya G, Dalmay T, Moulton V**. 2012. PAREsnip: a tool for rapid genome-wide discovery of small RNA/target interactions evidenced through degradome sequencing. *Nucleic Acids Research* **40**, e103.

**Kim D, Pertea G, Trapnell C, Pimentel H, Kelley R, Salzberg SL**. 2013. TopHat2: accurate alignment of transcriptomes in the presence of insertions, deletions and gene fusions. *Genome Biol* **14**.

**Langmead B, Trapnell C, Pop M, Salzberg SL**. 2009. Ultrafast and memory-efficient alignment of short DNA sequences to the human genome. *Genome Biol* **10**.

**Li B, Dewey CN**. 2011. RSEM: accurate transcript quantification from RNA-Seq data with or without a reference genome. *BMC Bioinformatics* **12**, 323.

**Meyers BC, Axtell MJ, Bartel B, Bartel DP, Baulcombe D, Bowman JL, Cao X, Carrington JC, Chen X, Green PJ, Griffiths-Jones S, Jacobsen SE, Mallory AC, Martienssen RA, Poethig RS, Qi Y, Vaucheret H, Voinnet O, Watanabe Y, Weigel D, Zhu JK**. 2008. Criteria for annotation of plant MicroRNAs. *Plant Cell* **20**, 3186-3190.

**Stocks MB, Moxon S, Mapleson D, Woolfenden HC, Mohorianu I, Folkes L, Schwach F, Dalmay T, Moulton V**. 2012. The UEA sRNA workbench: a suite of tools for analysing and visualizing next generation sequencing microRNA and small RNA datasets. *Bioinformatics* **28**, 2059-2061.

**Supek F, Bosnjak M, Skunca N, Smuc T**. 2011. REVIGO Summarizes and Visualizes Long Lists of Gene Ontology Terms. *Plos One* **6**.

**Xie C, Mao XZ, Huang JJ, Ding Y, Wu JM, Dong S, Kong L, Gao G, Li CY, Wei LP**. 2011. KOBAS 2.0: a web server for annotation and identification of enriched pathways and diseases. *Nucleic Acids Research* **39**, W316-W322.

**Xu Q, Chen LL, Ruan XA, Chen DJ, Zhu AD, Chen CL, Bertrand D, Jiao WB, Hao BH, Lyon MP, Chen JJ, Gao S, Xing F, Lan H, Chang JW, Ge XH, Lei Y, Hu Q, Miao Y, Wang L, Xiao SX, Biswas MK, Zeng WF, Guo F, Cao HB, Yang XM, Xu XW, Cheng YJ, Xu J, Liu JH, Luo OJ, Tang ZH, Guo WW, Kuang HH, Zhang HY, Roose ML, Nagarajan N, Deng XX, Ruan YJ**. 2013. The draft genome of sweet orange (Citrus sinensis). *Nature Genetics* **45**, 59-U92.

**Zhang Z, Jiang L, Wang J, Gu P, Chen M**. 2015. MTide: an integrated tool for the identification of miRNA-target interaction in plants. *Bioinformatics* **31**, 290-291.
