# Supplementary figures and images for: Genome-wide comprehensive analysis of transcriptomes and small RNAs offers insights into the molecular mechanism of alkaline stress tolerance in a citrus rootstock
Source: Hortic Res. 2019 Mar 1;6:33. doi: 10.1038/s41438-018-0116-0 (PMC6395741; doi:10.1038/s41438-018-0116-0)

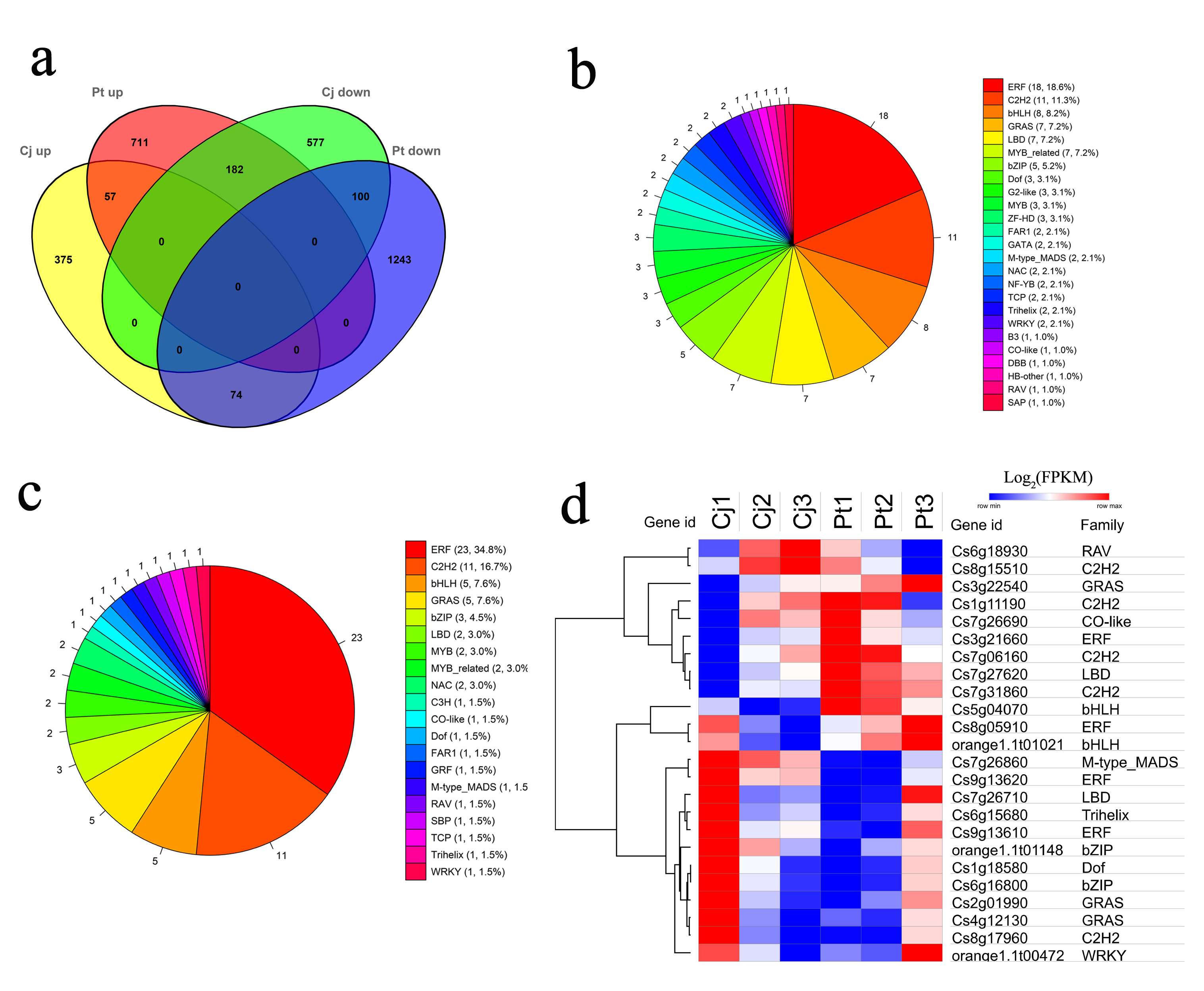

Supplement: Supplementary file 3 — Figure S1 [file 41438_2018_116_MOESM3_ESM.tif]

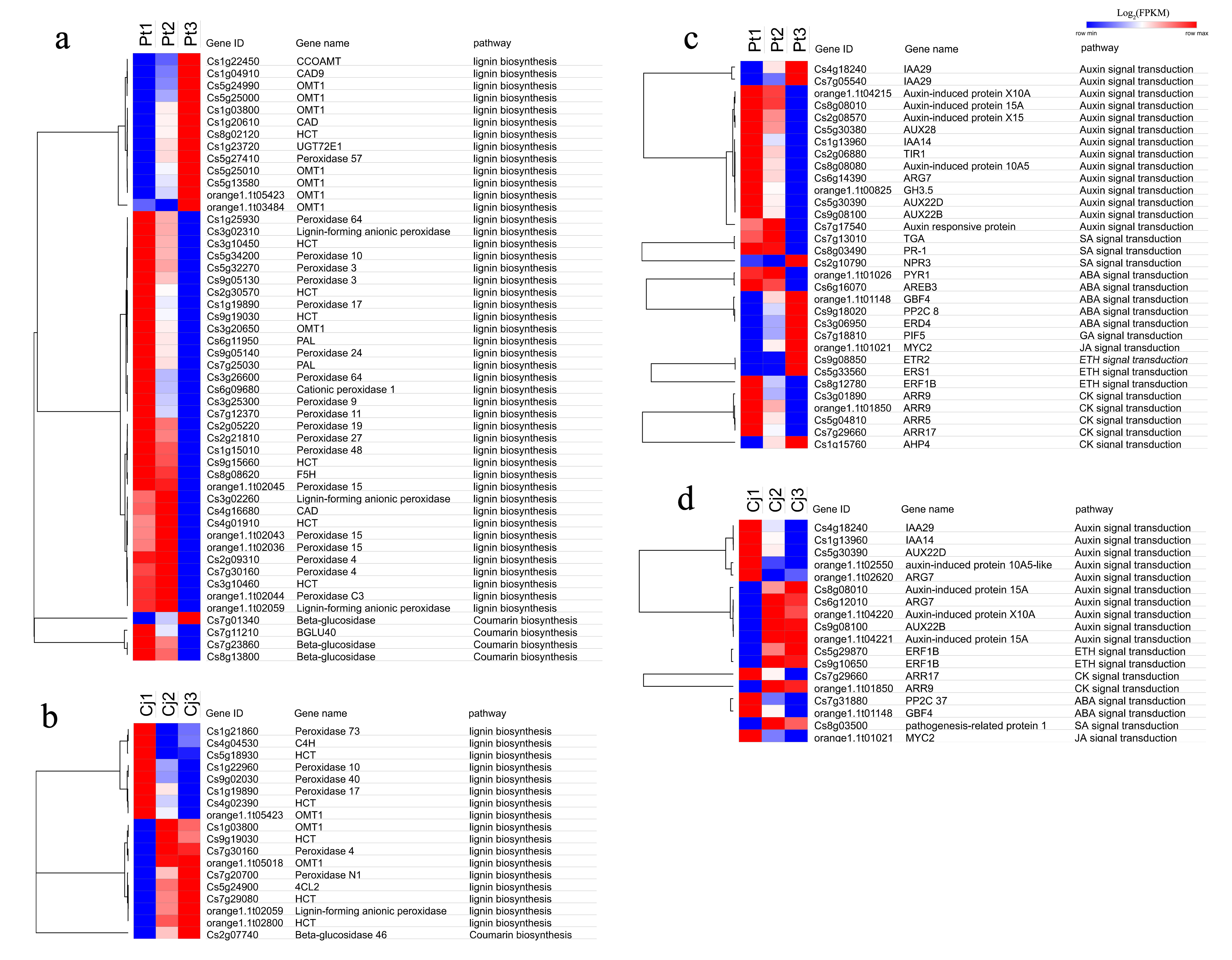

Supplement: Supplementary file 4 — Figure S2 [file 41438_2018_116_MOESM4_ESM.tif]

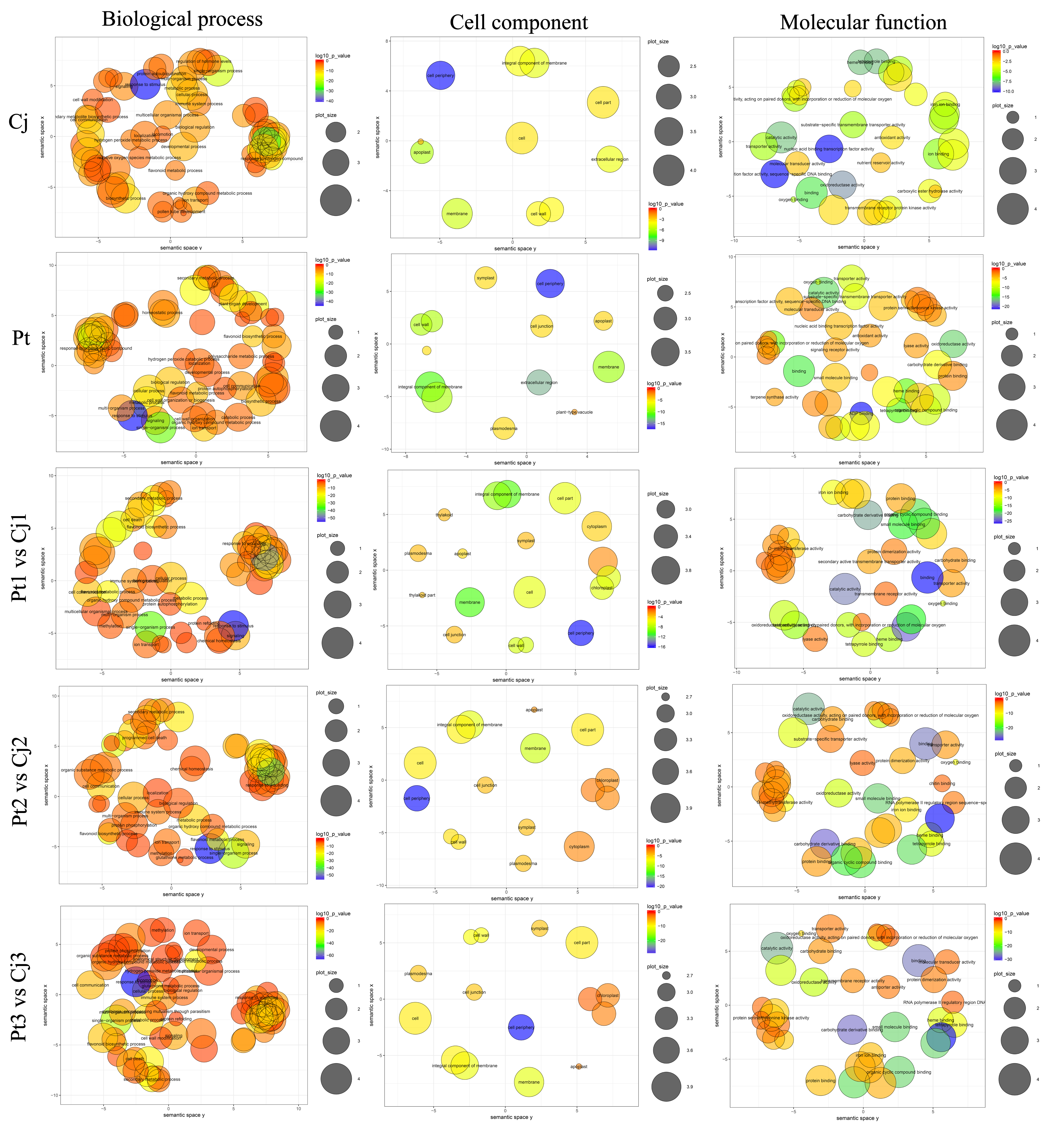

Supplement: Supplementary file 5 — Figure S3 [file 41438_2018_116_MOESM5_ESM.tif]

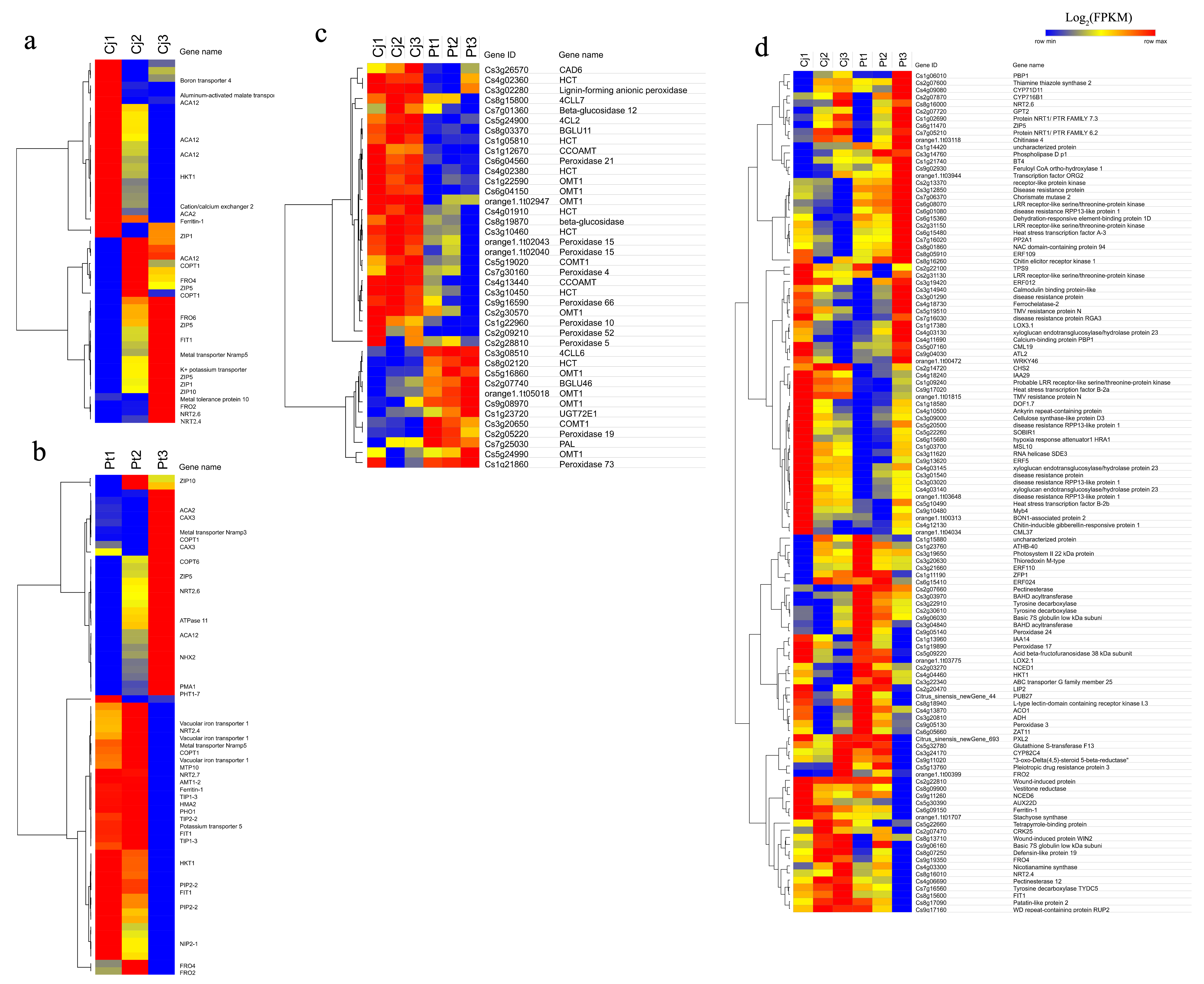

Supplement: Supplementary file 6 — Figure S4 [file 41438_2018_116_MOESM6_ESM.tif]

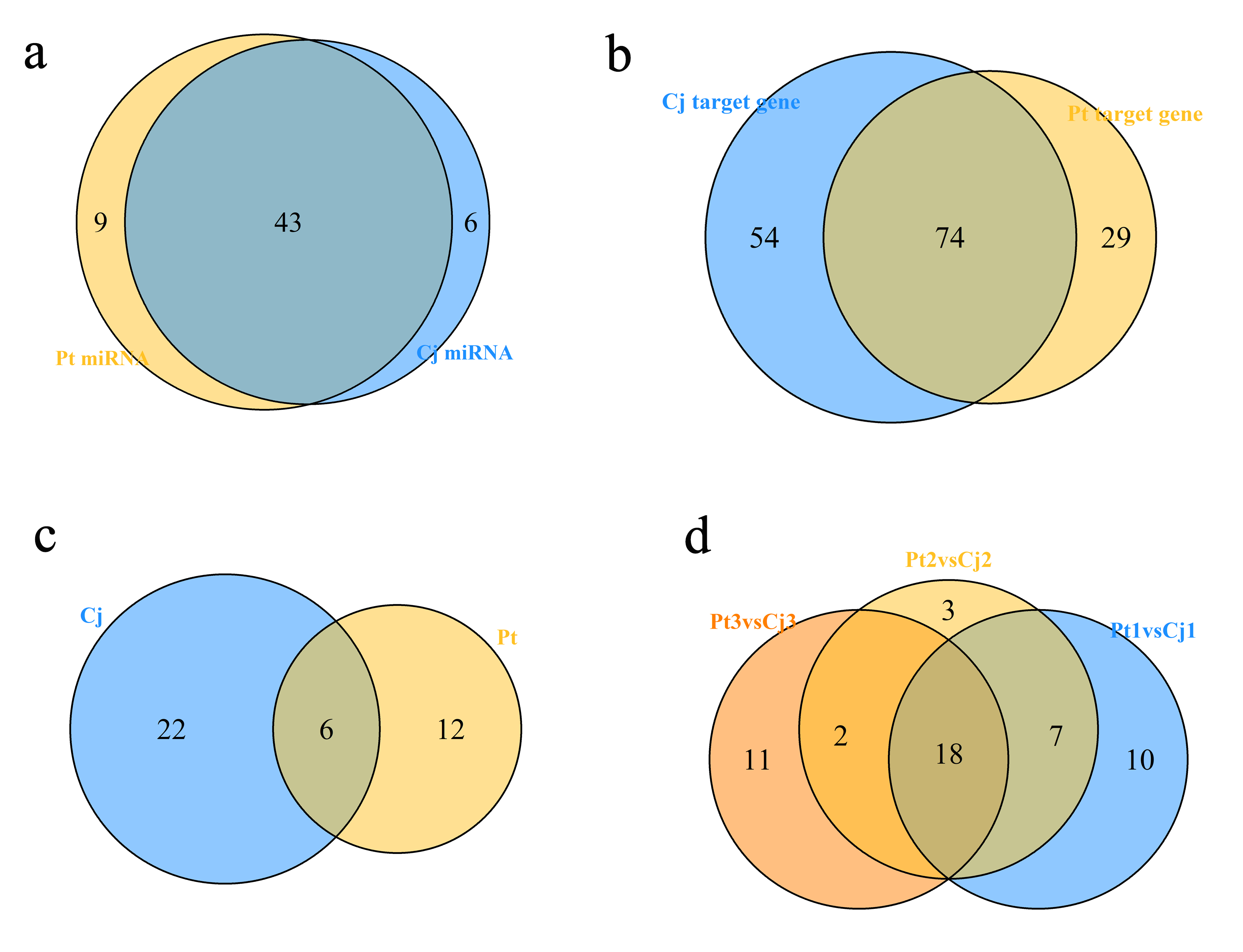

Supplement: Supplementary file 10 — Figure S8 [file 41438_2018_116_MOESM10_ESM.tif]

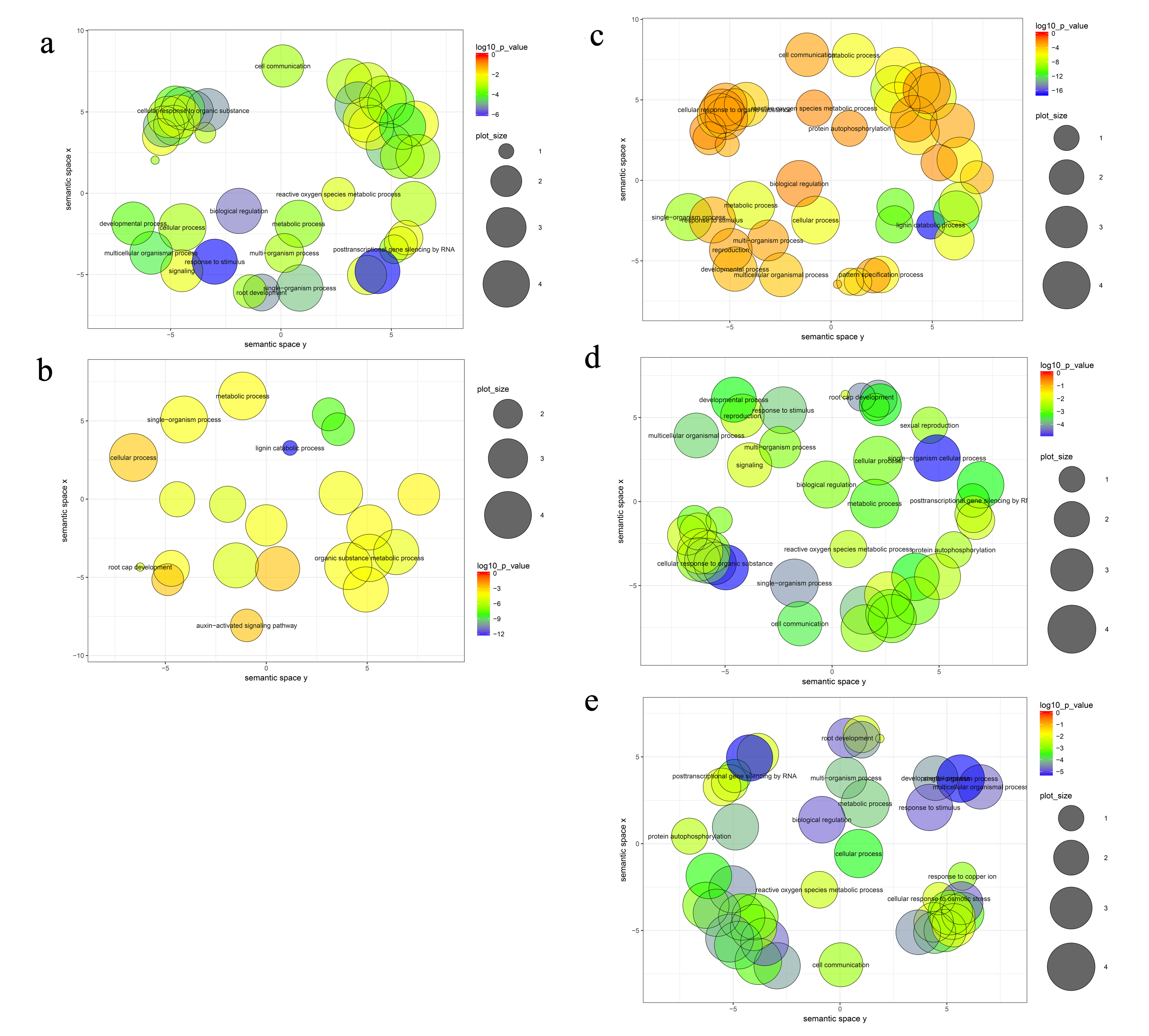

Supplement: Supplementary file 11 — Figure S9 [file 41438_2018_116_MOESM11_ESM.tif]
