# Supplementary material for: Genome-wide comprehensive analysis of transcriptomes and small RNAs offers insights into the molecular mechanism of alkaline stress tolerance in a citrus rootstock
Source: Hortic Res. 2019 Mar 1;6:33. doi: 10.1038/s41438-018-0116-0 (PMC6395741; doi:10.1038/s41438-018-0116-0)

csi-MIRN01

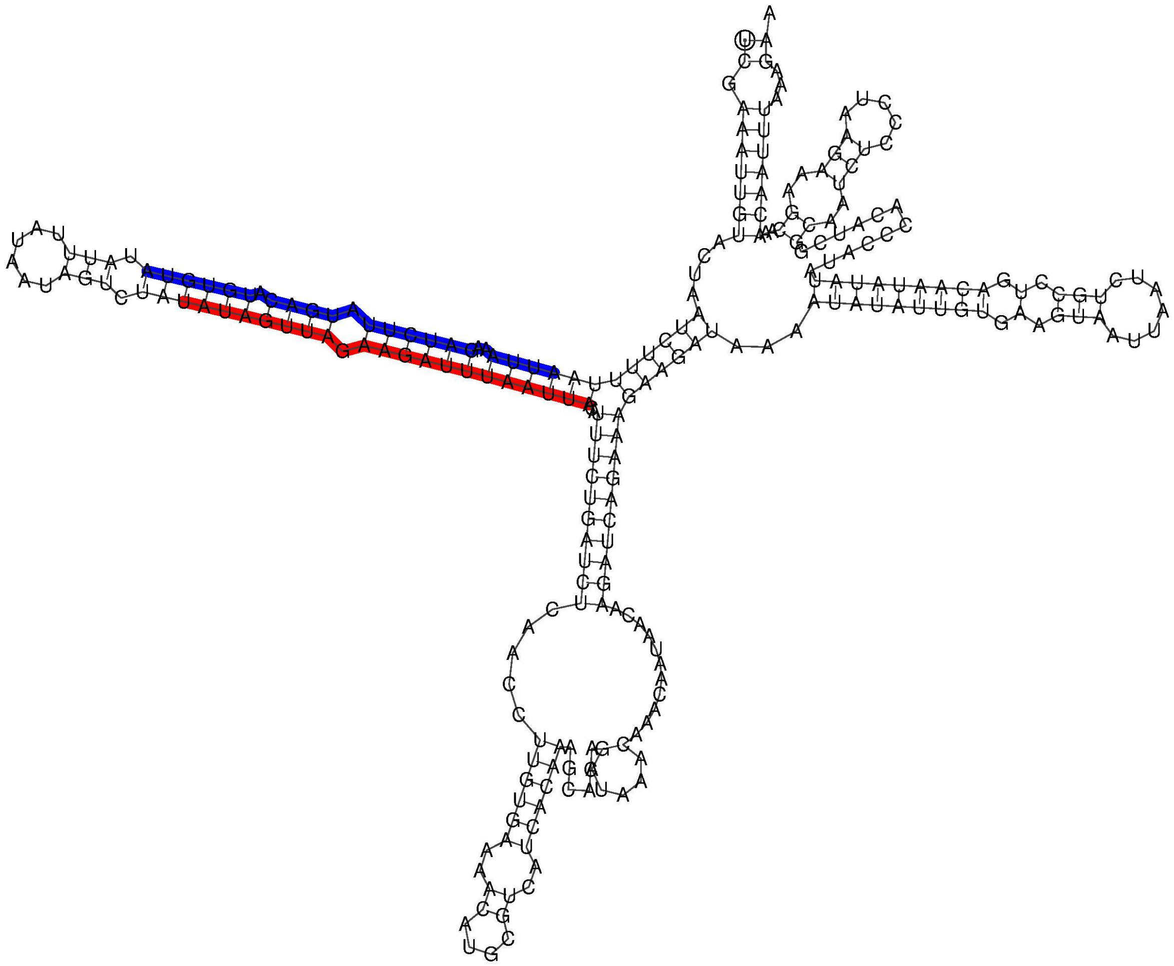

csi-MIRN02

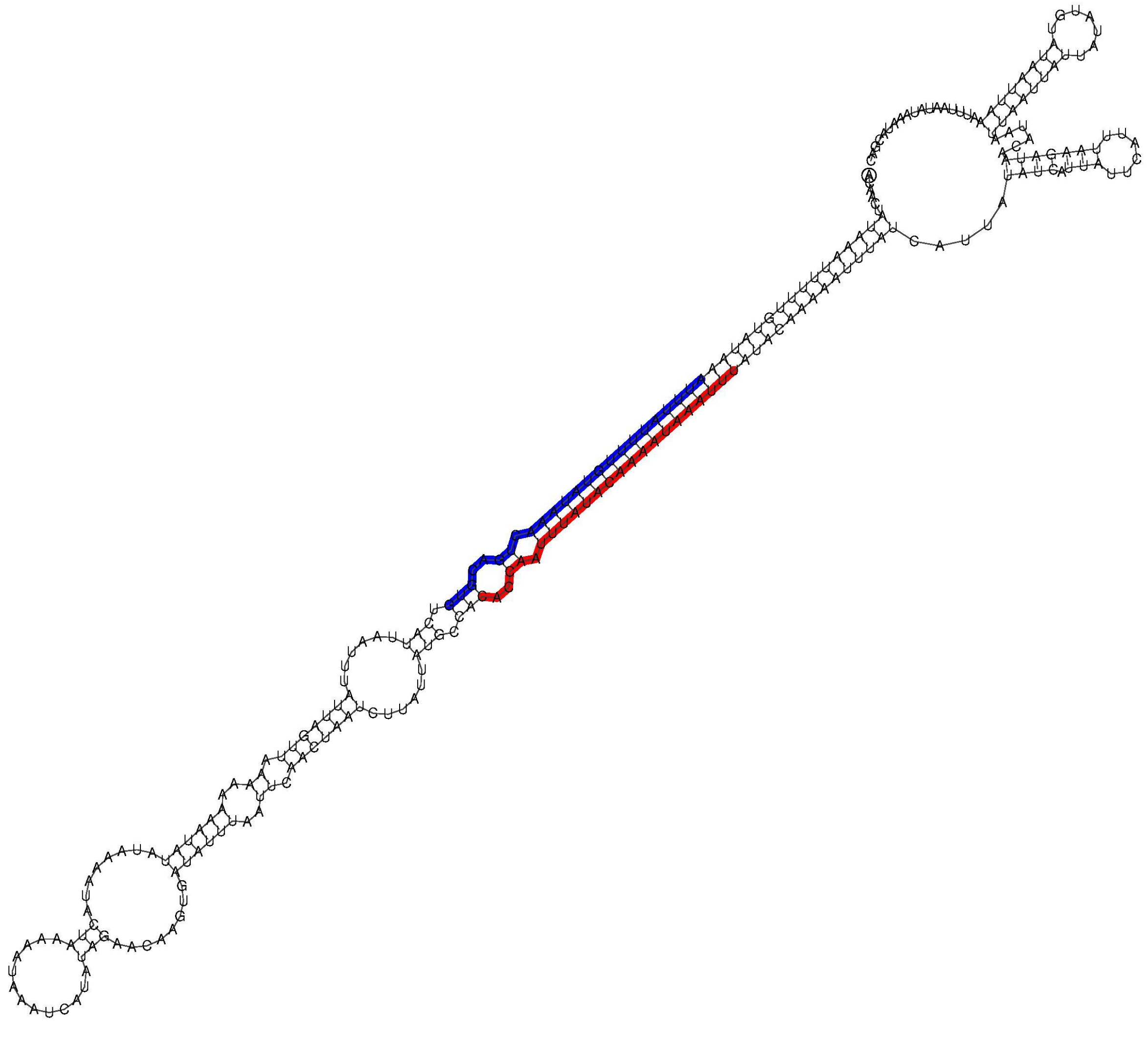

csi-MIRN03

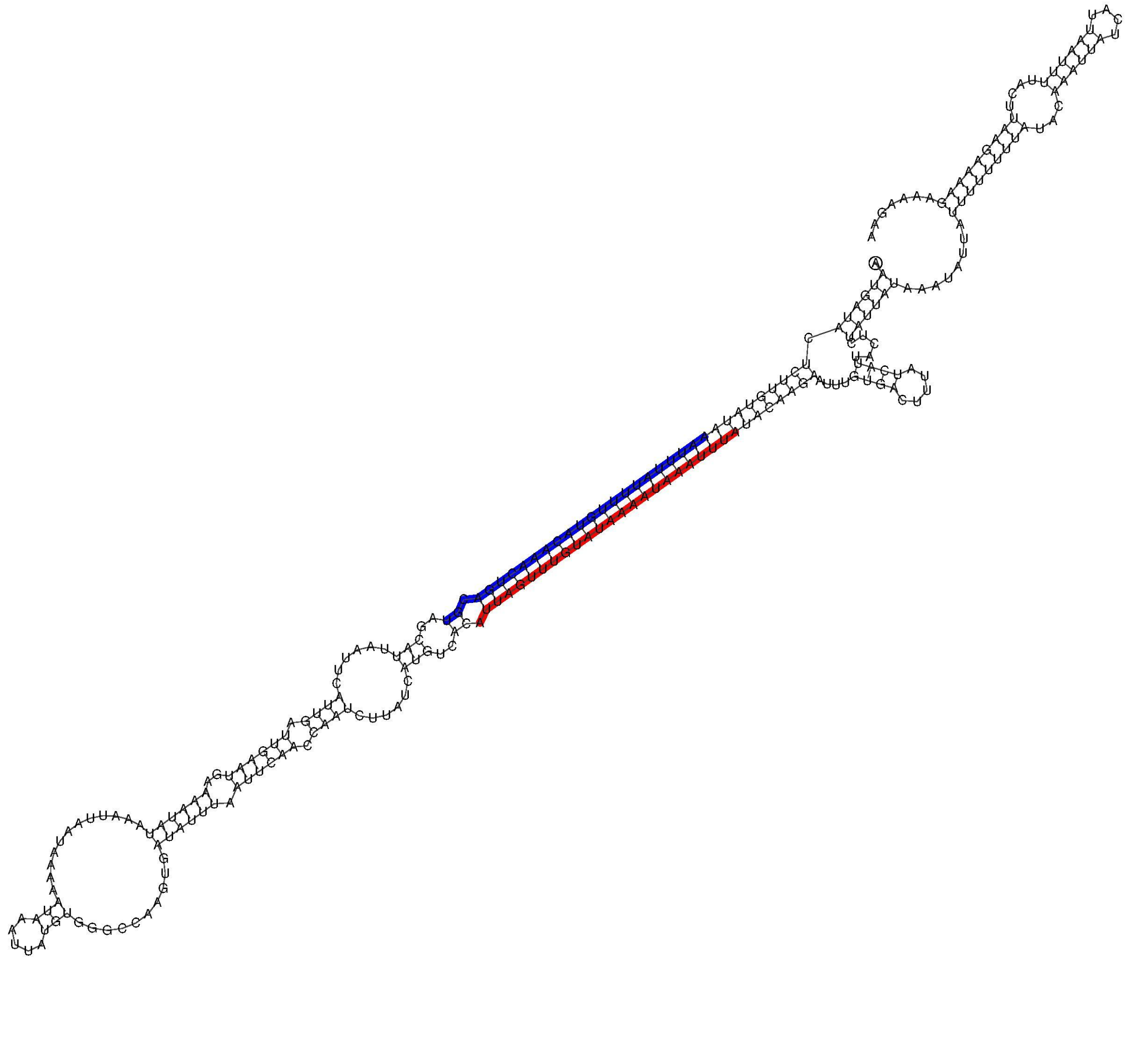

csi-MIRN04

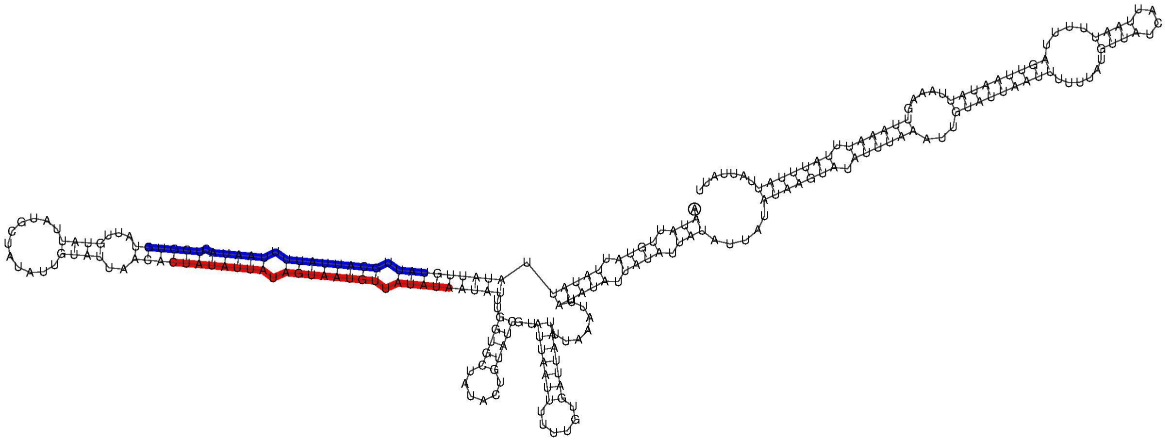

csi-MIRN05

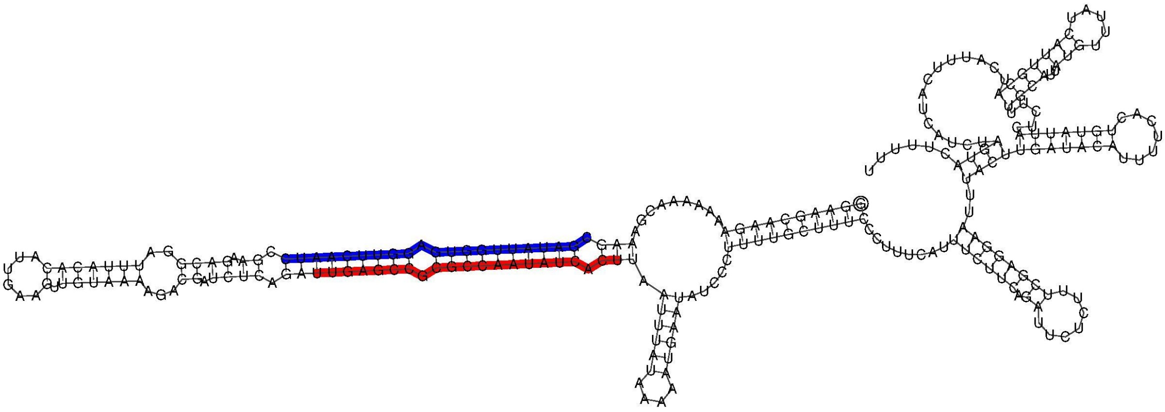

csi-MIRN06

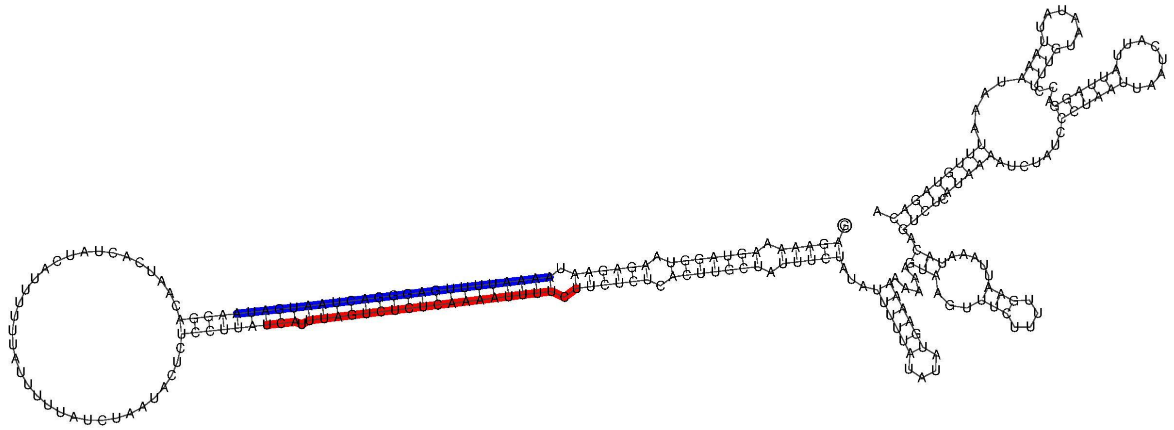

csi-MIRN07

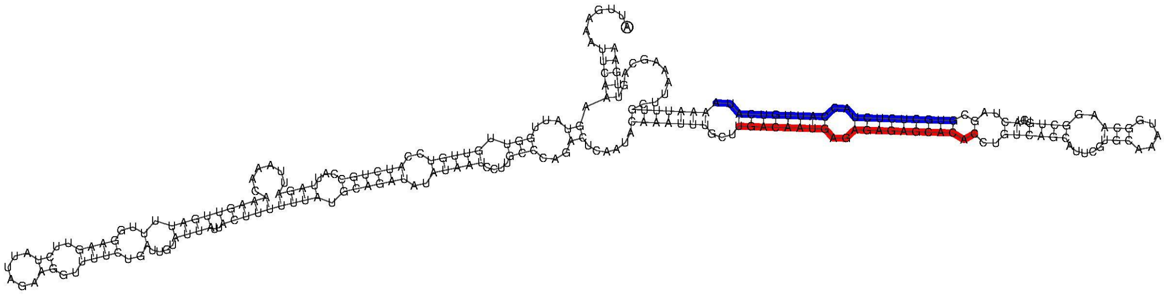

csi-MIRN08

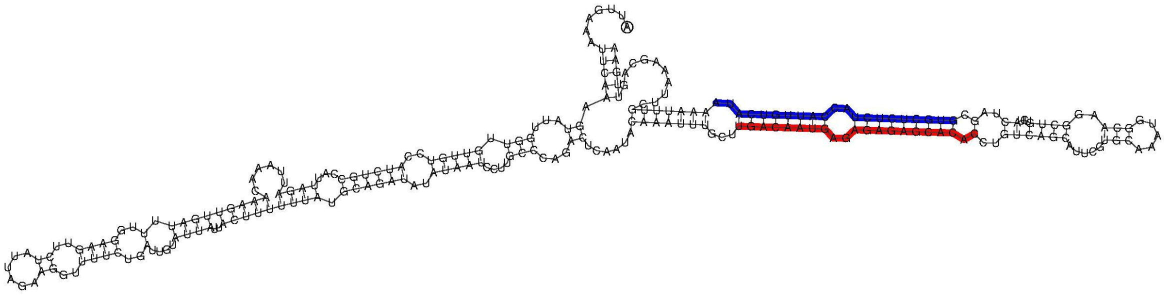

csi-MIRN09

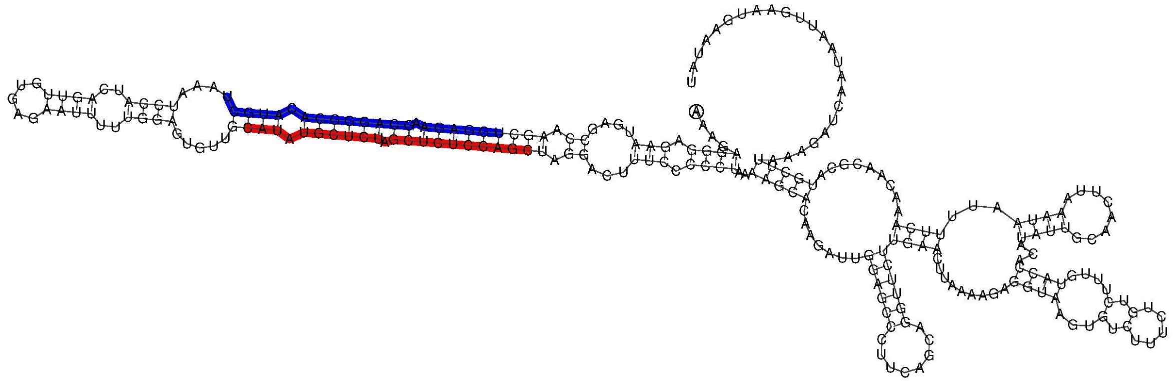

csi-MIRN10

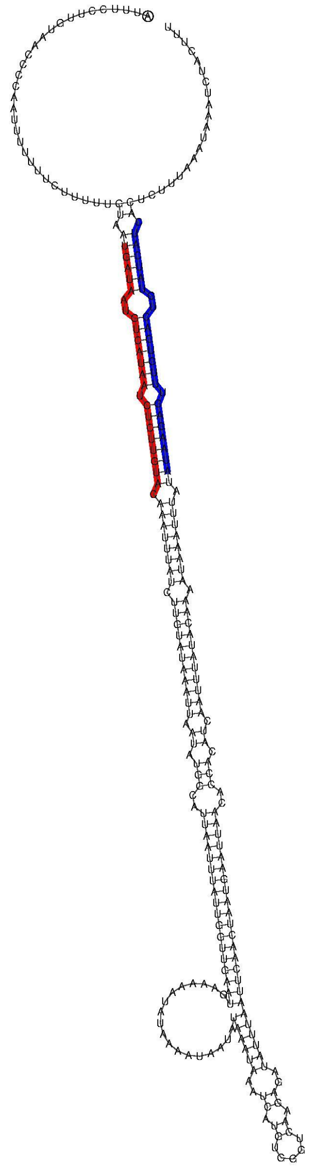

csi-MIRN11

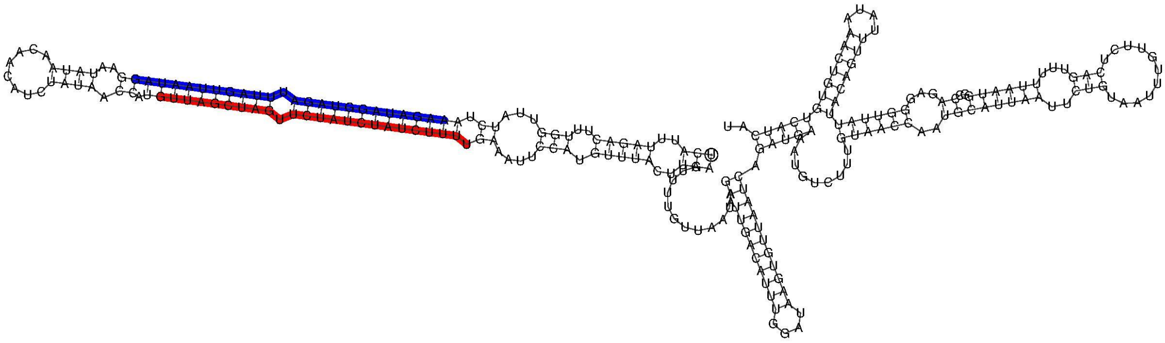

csi-MIRN12

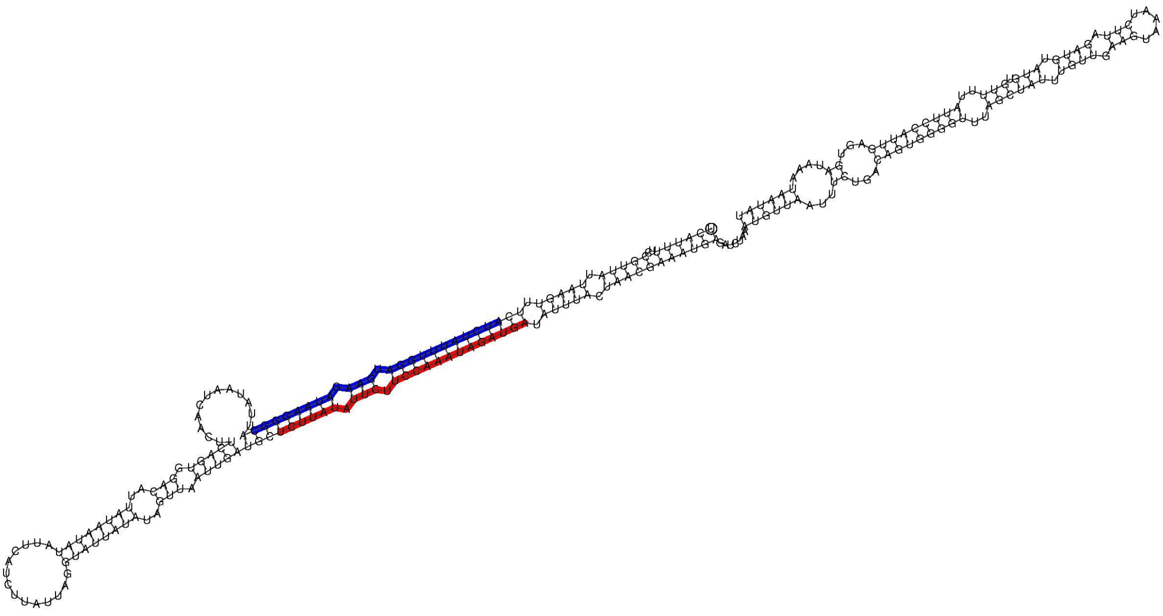

csi-MIRN13

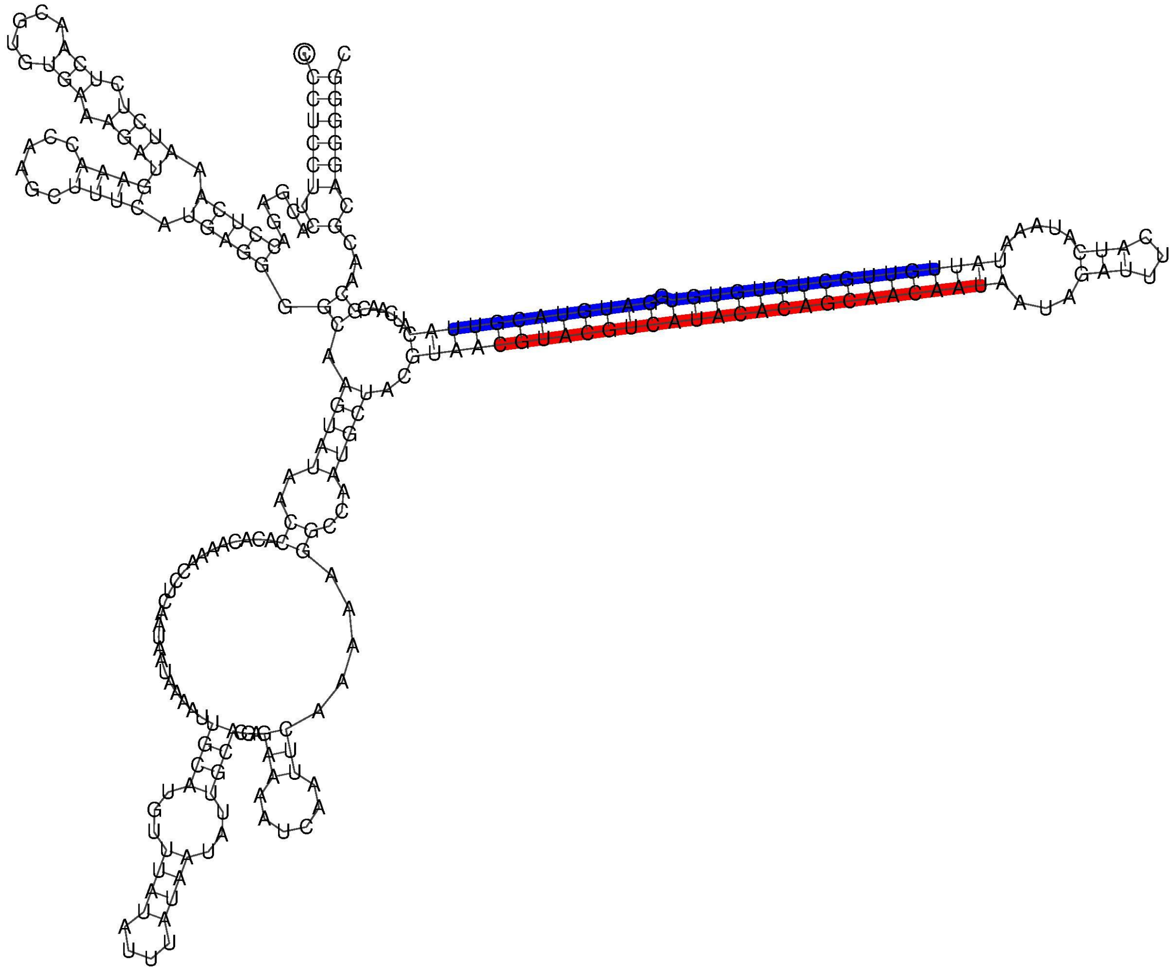

csi-MIRN14

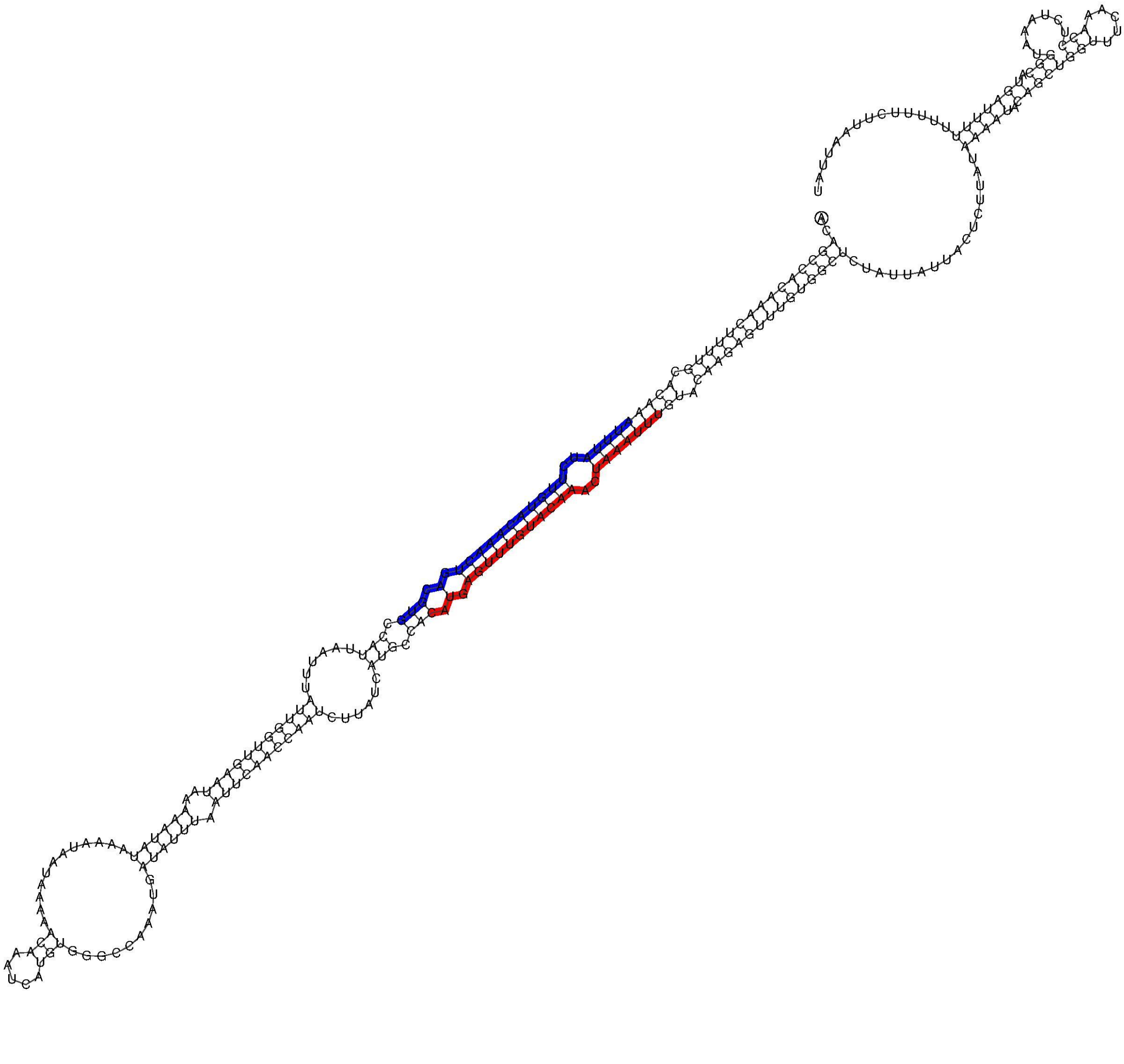

csi-MIRN15

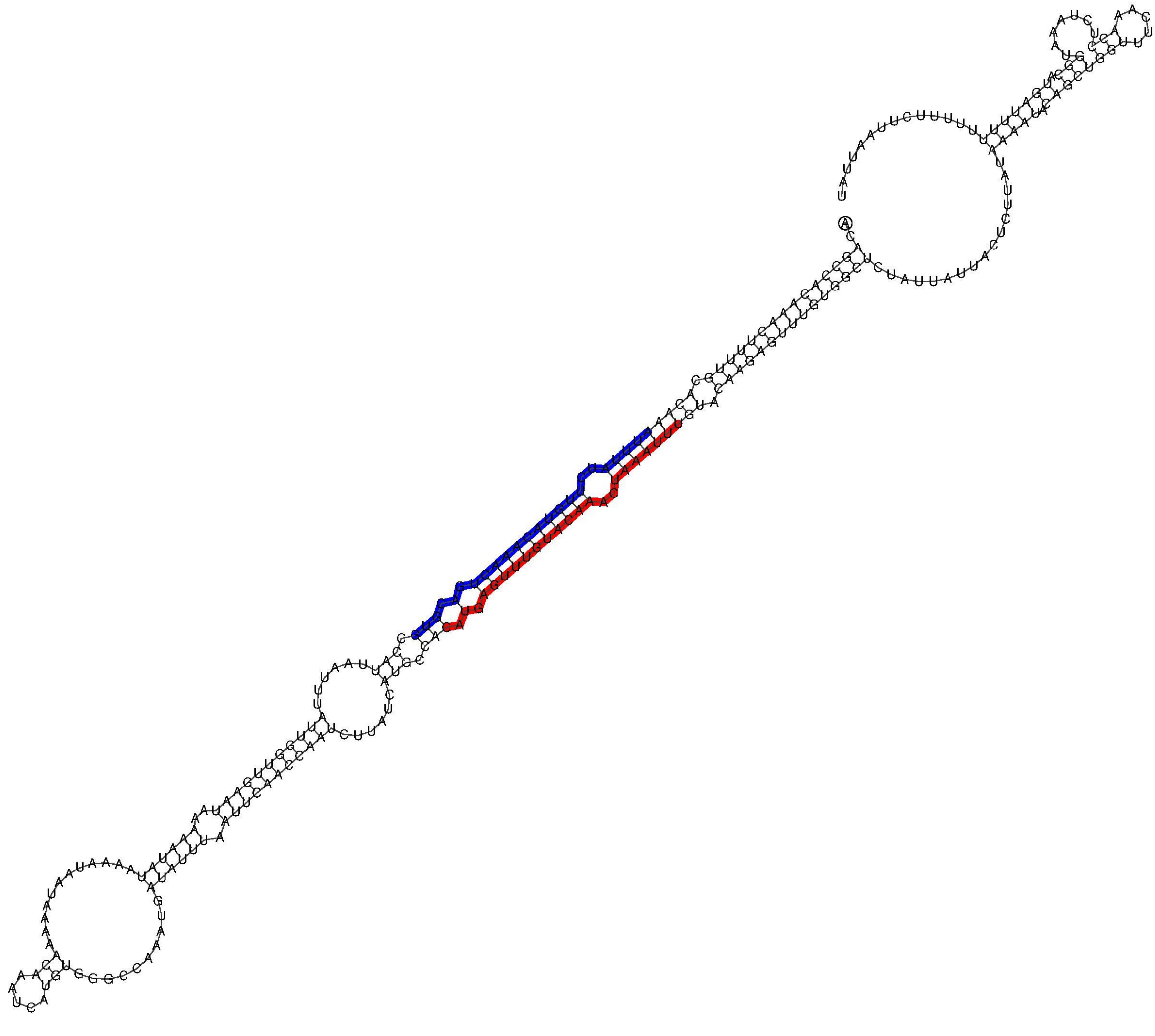

csi-MIRN16

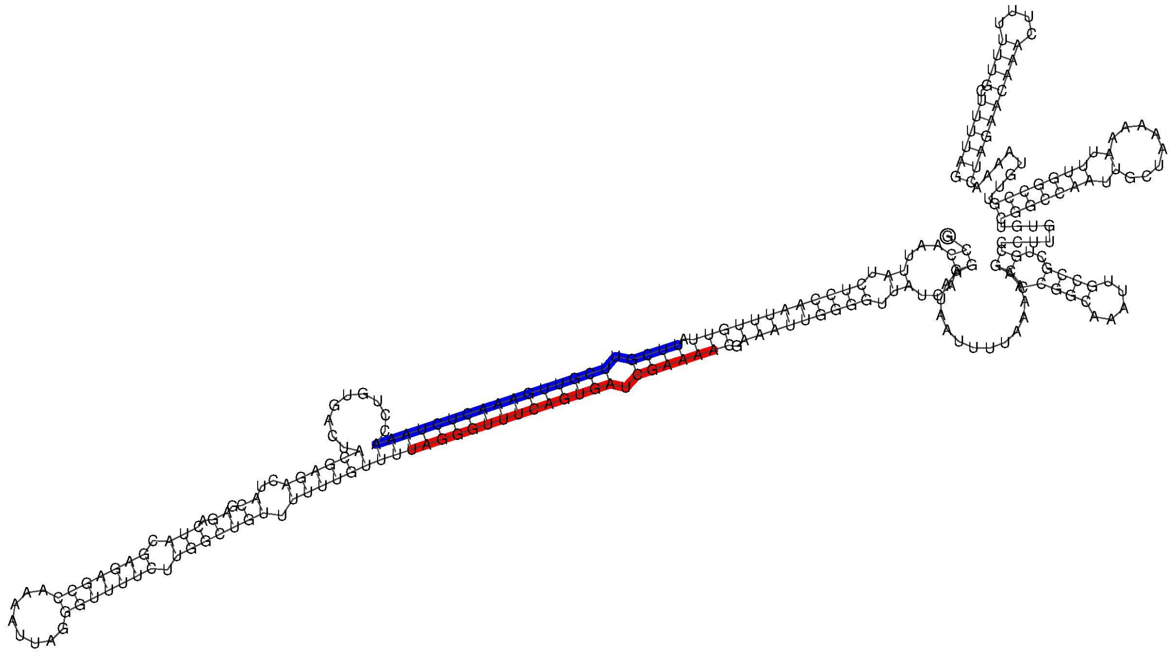

csi-MIRN17

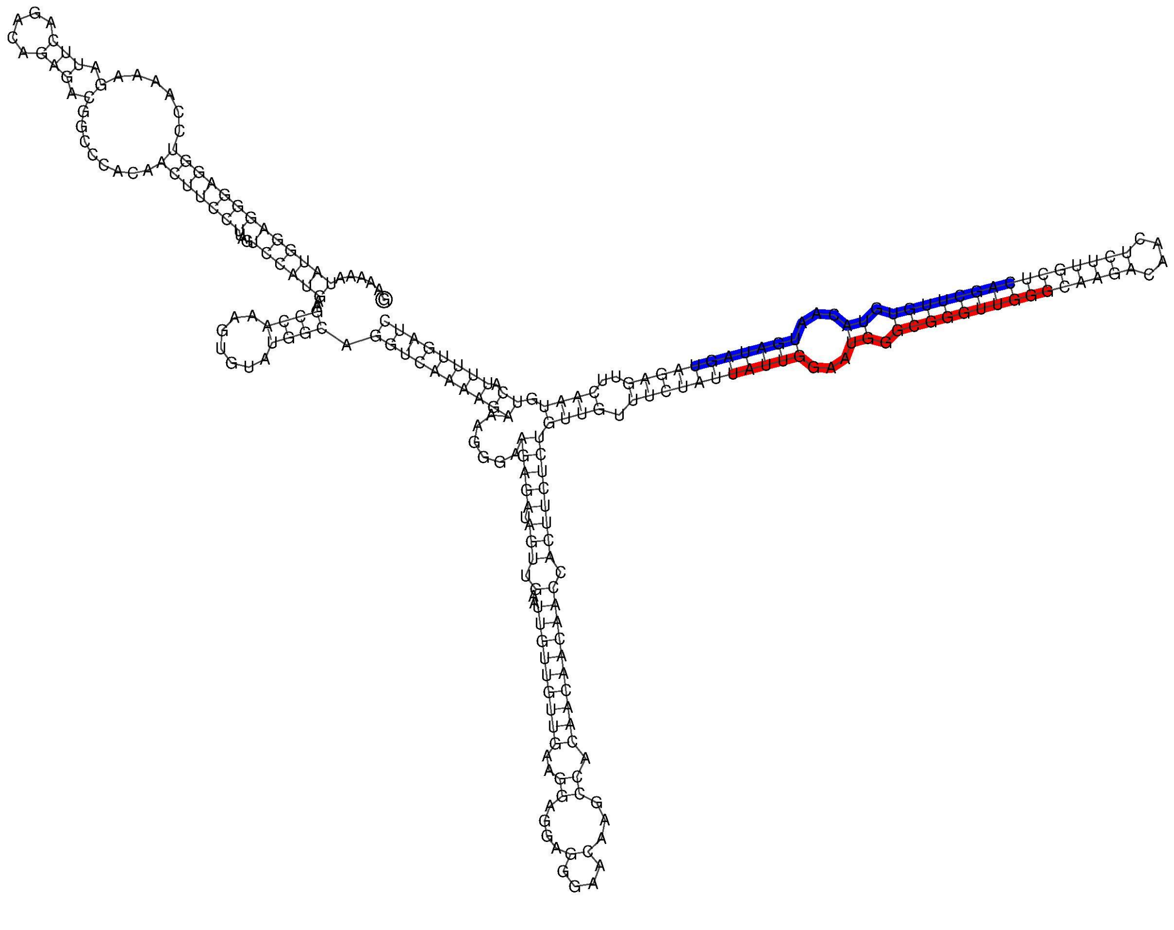

csi-MIRN18

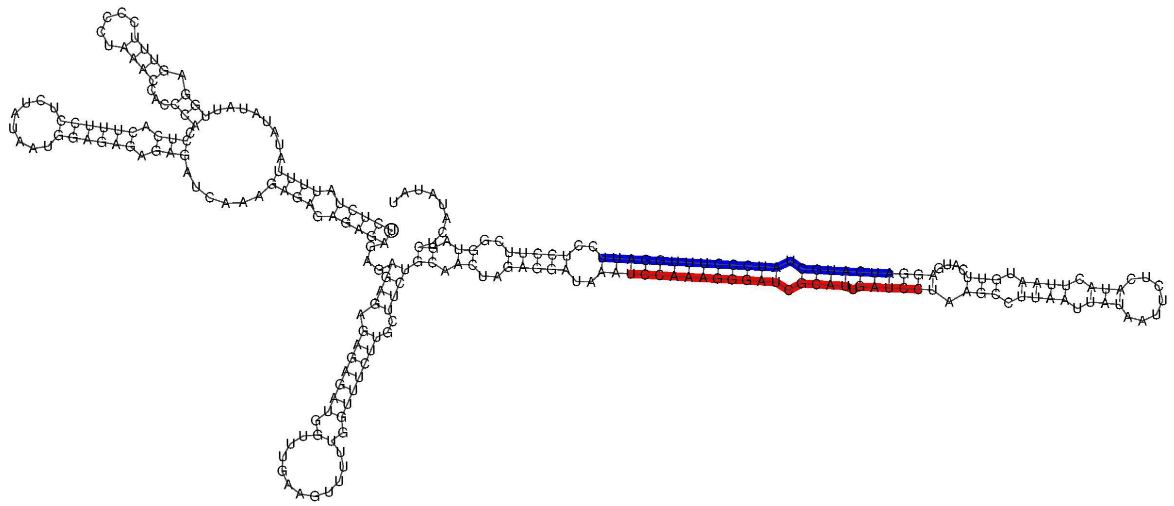

csi-MIRN19

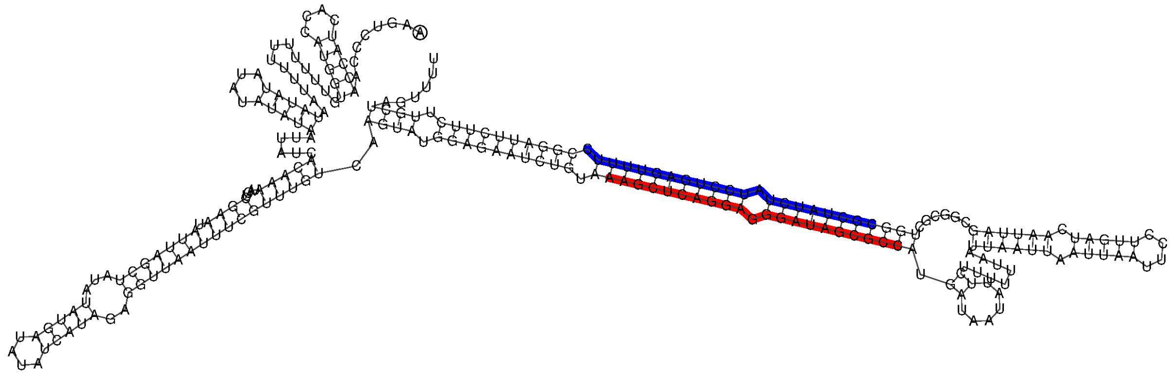

csi-MIRN20

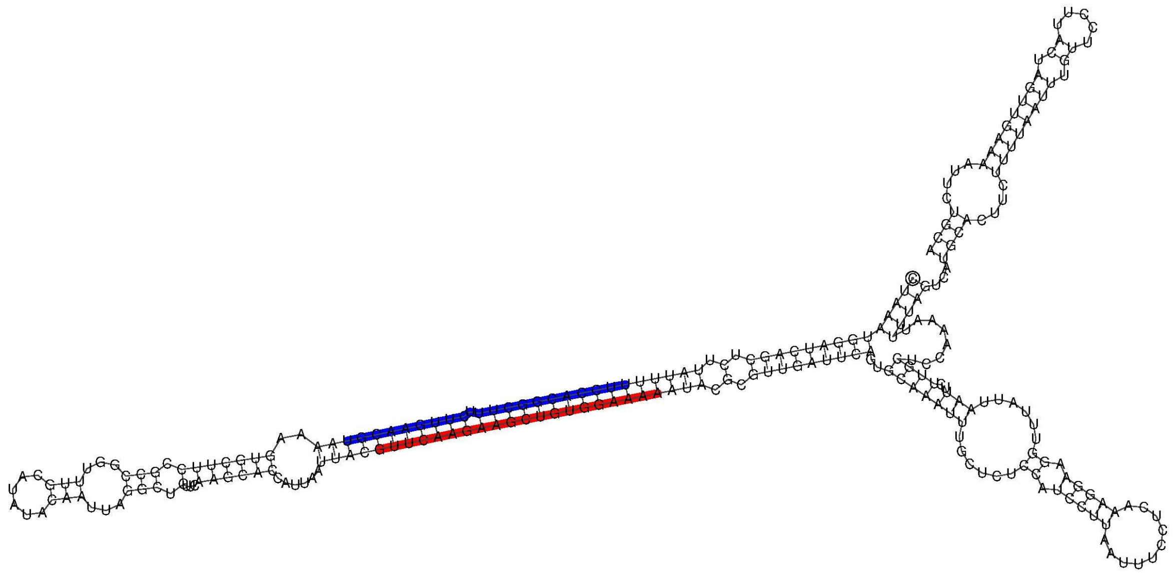

csi-MIRN21

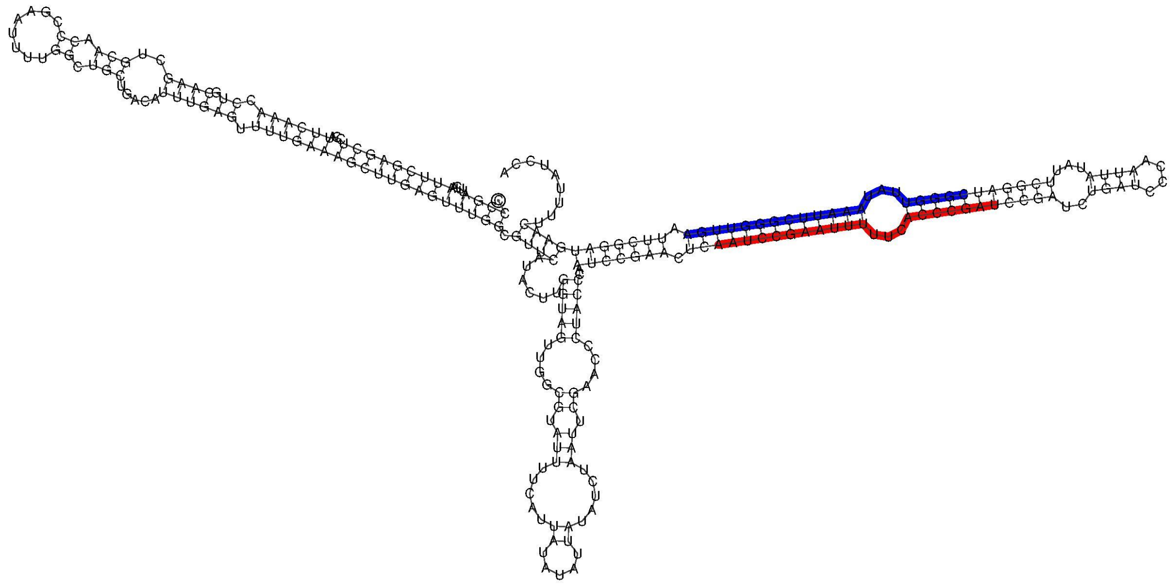

csi-MIRN22

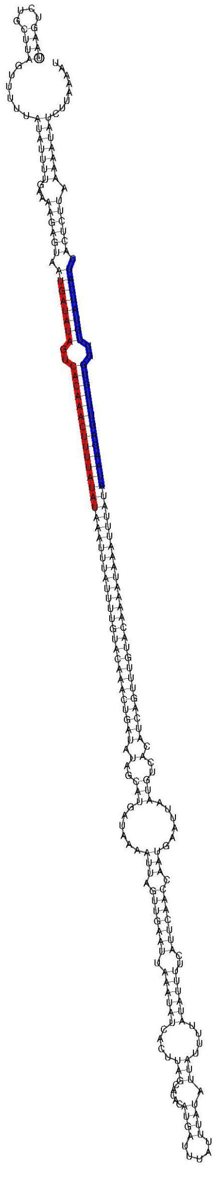

csi-MIRN23

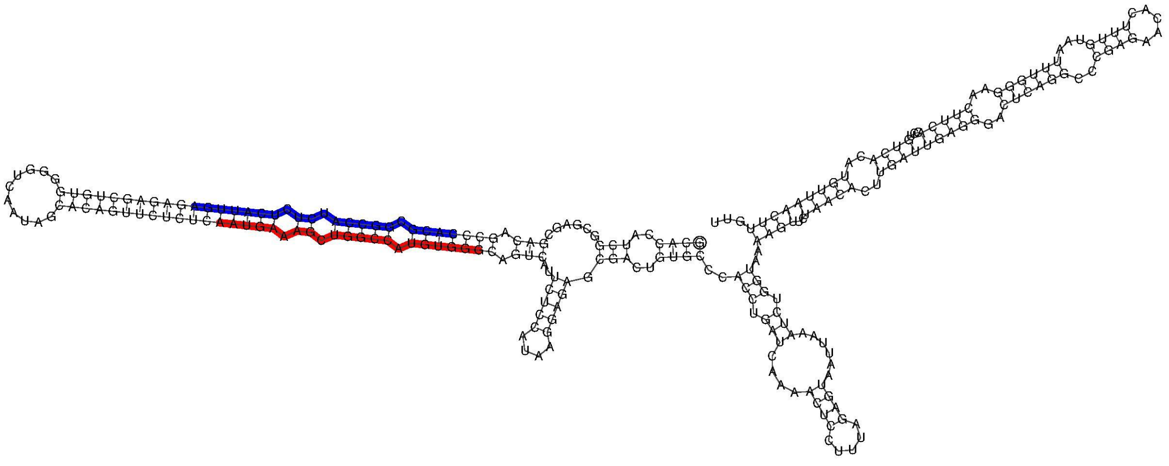

csi-MIRN24

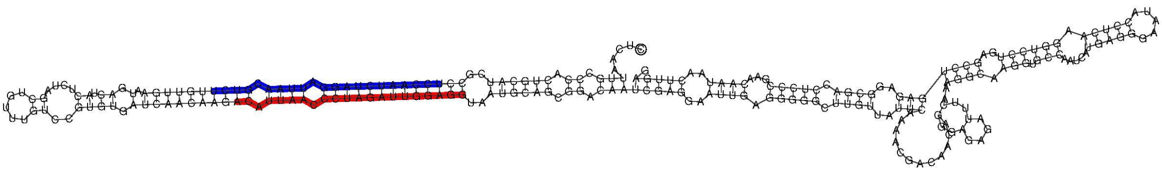

csi-MIRN25

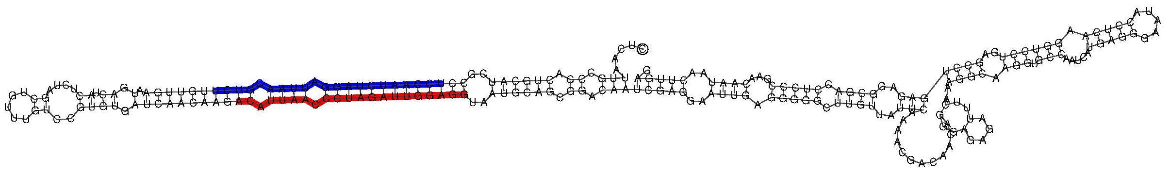

csi-MIRN26

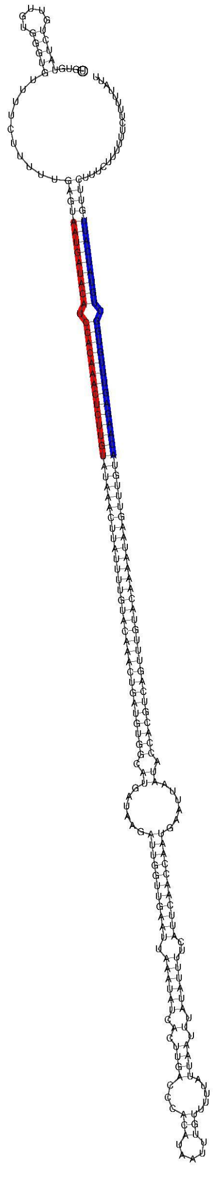

csi-MIRN27

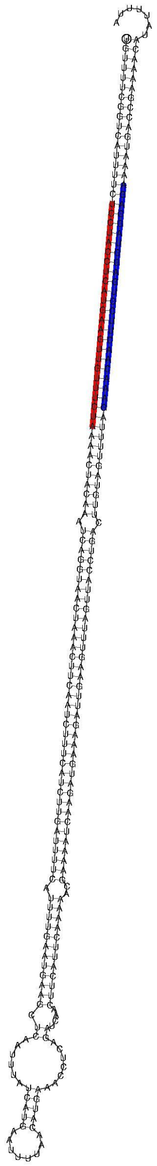

csi-MIRN28

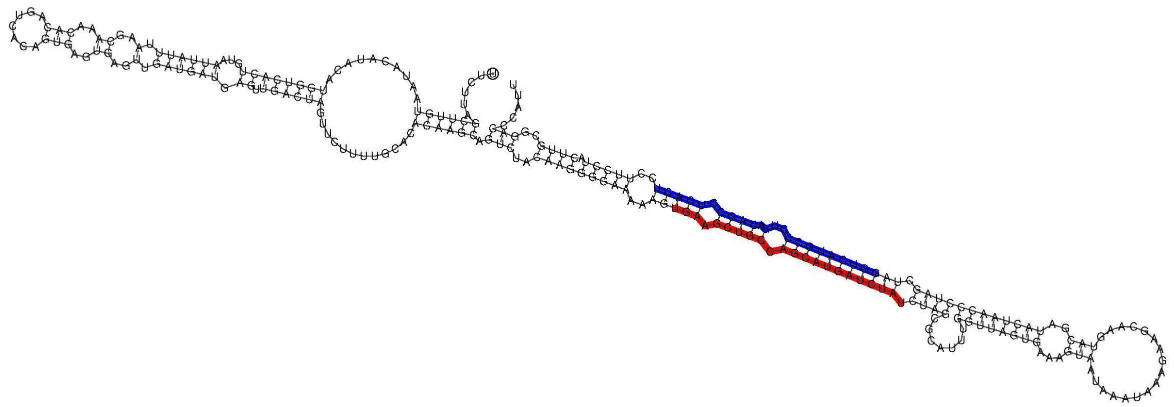

csi-MIRN29

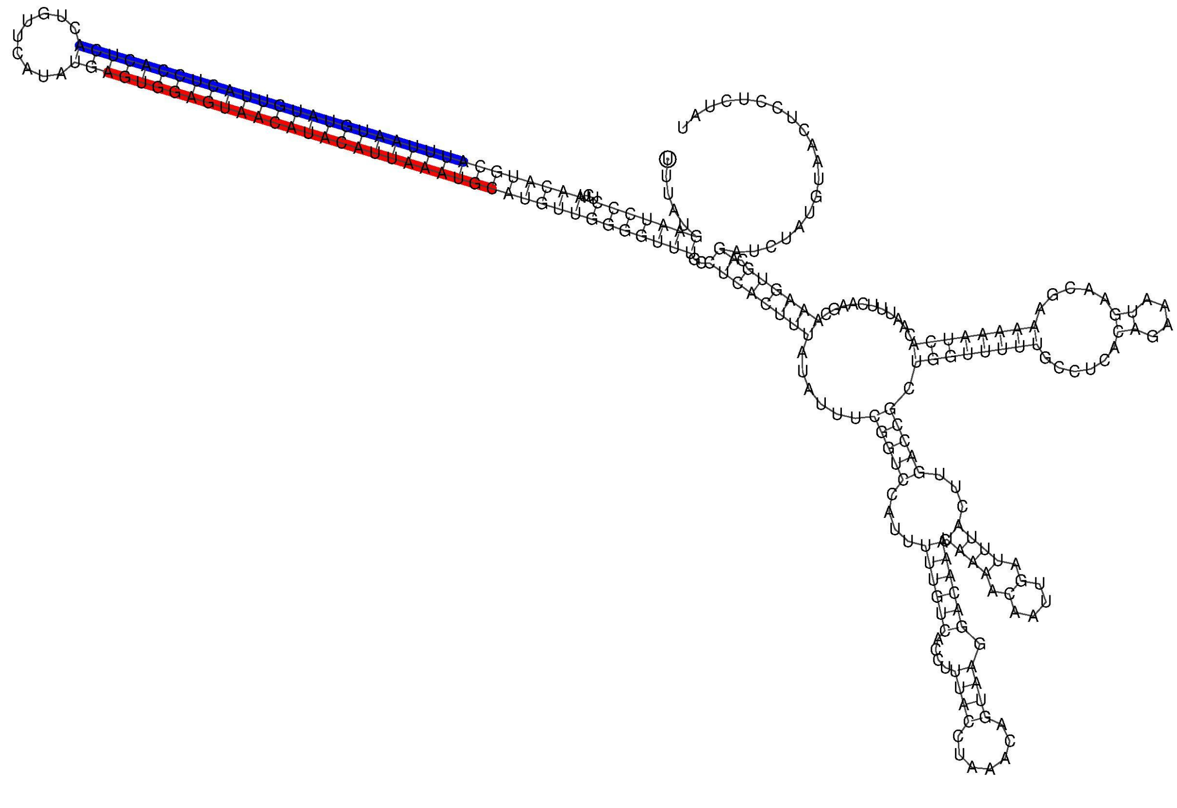

csi-MIRN30

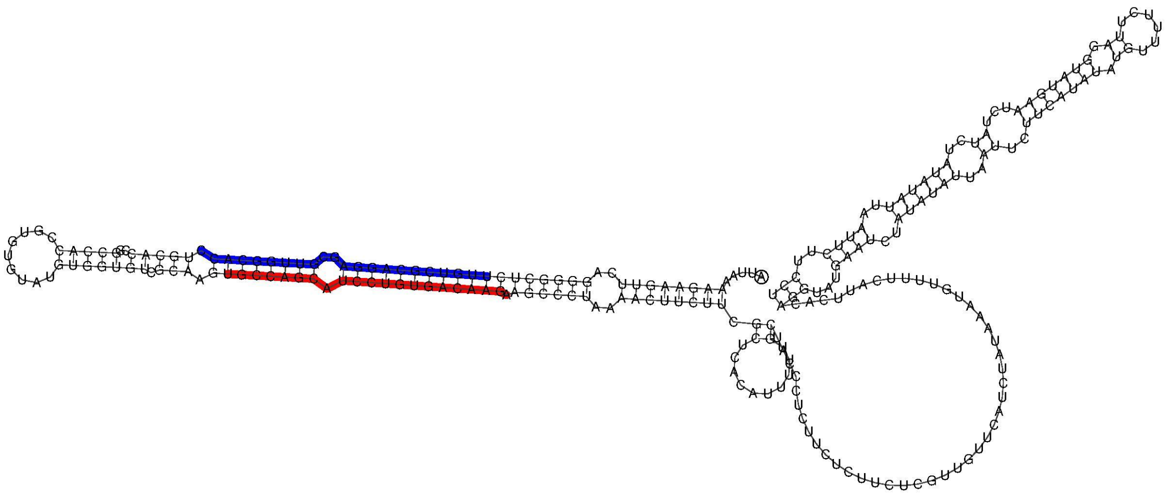

csi-MIRN31

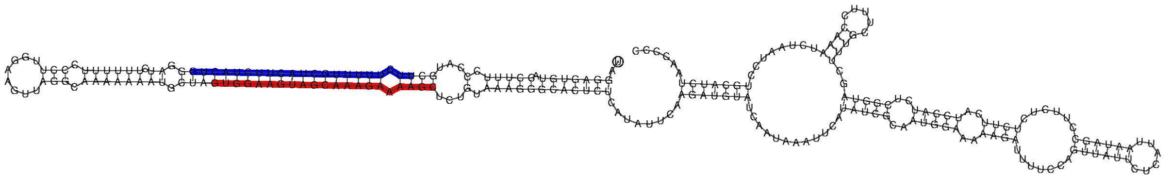

csi-MIRN32

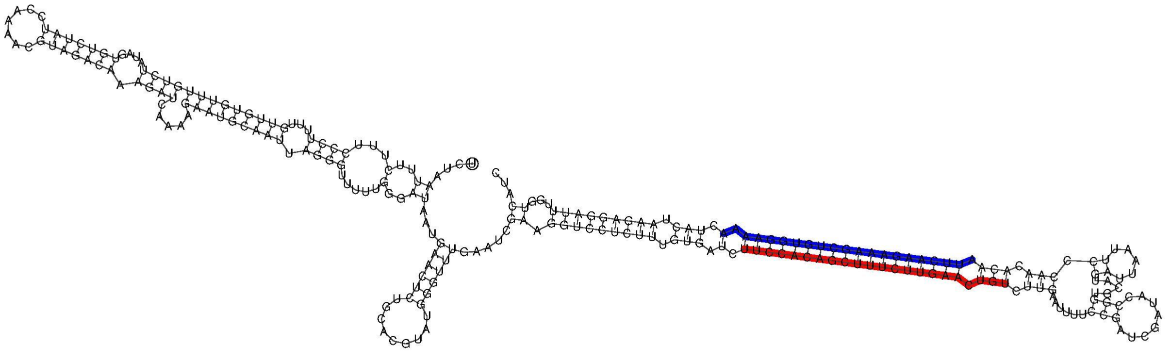

csi-MIRN33

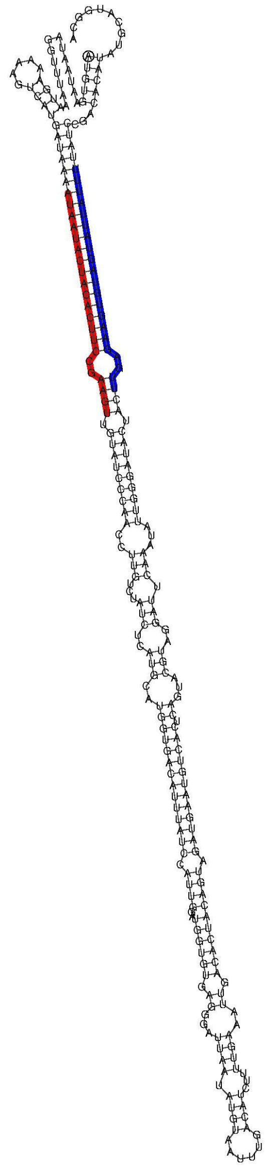

csi-MIRN34

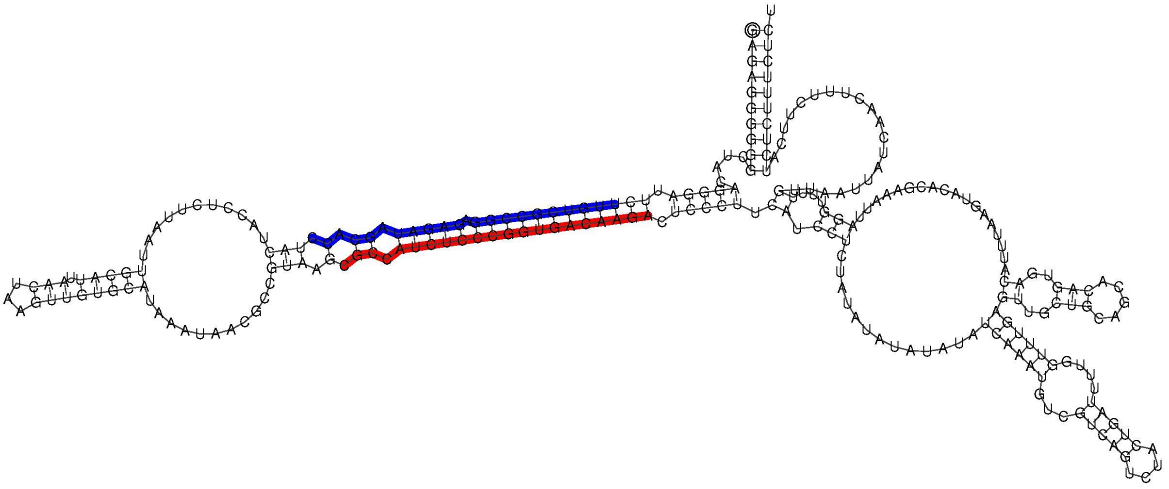

csi-MIRN35

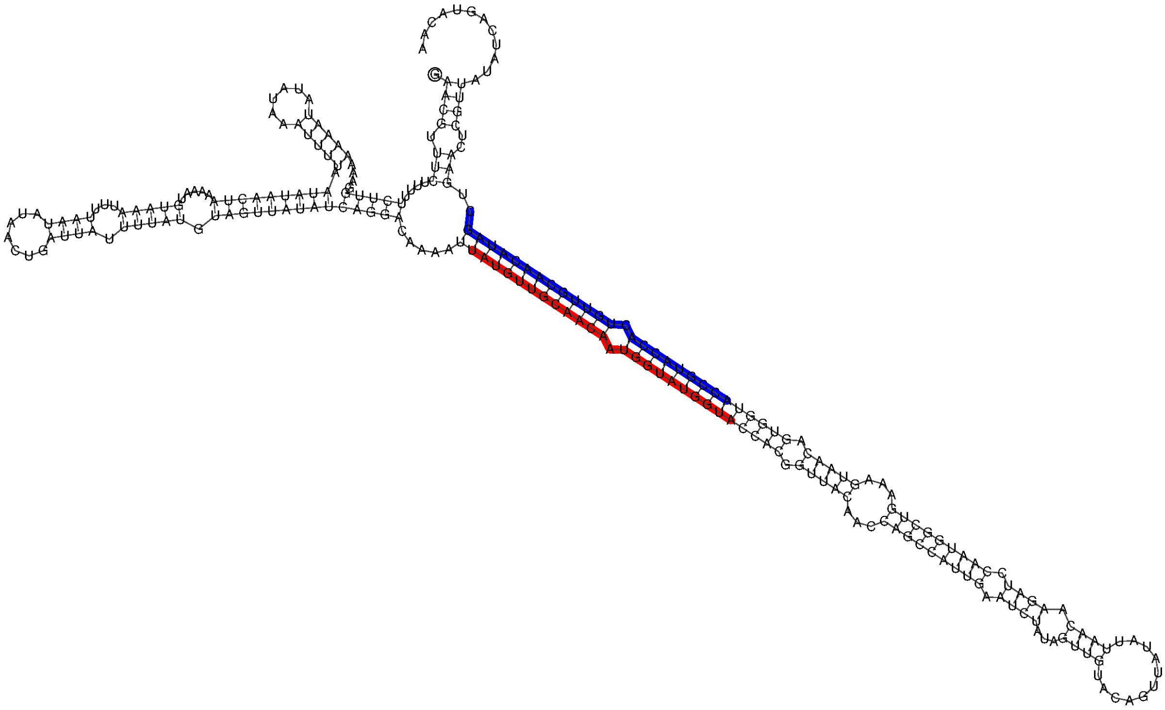

csi-MIRN36

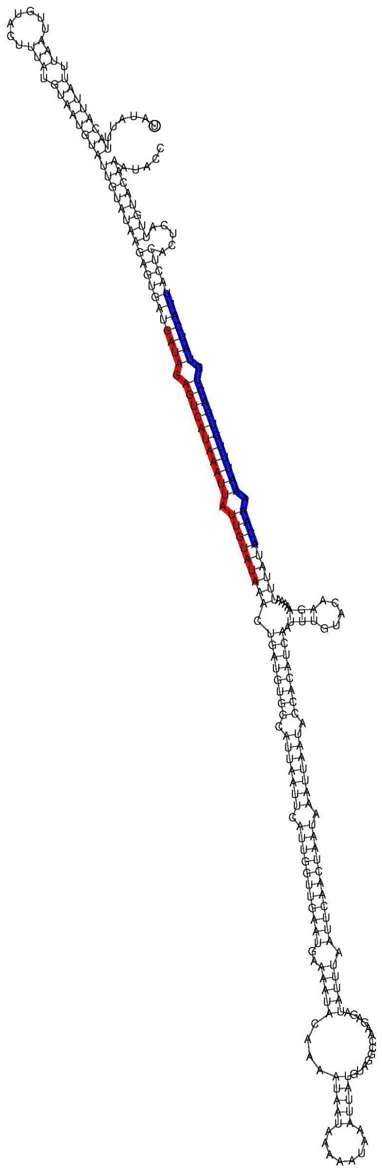

csi-MIRN37

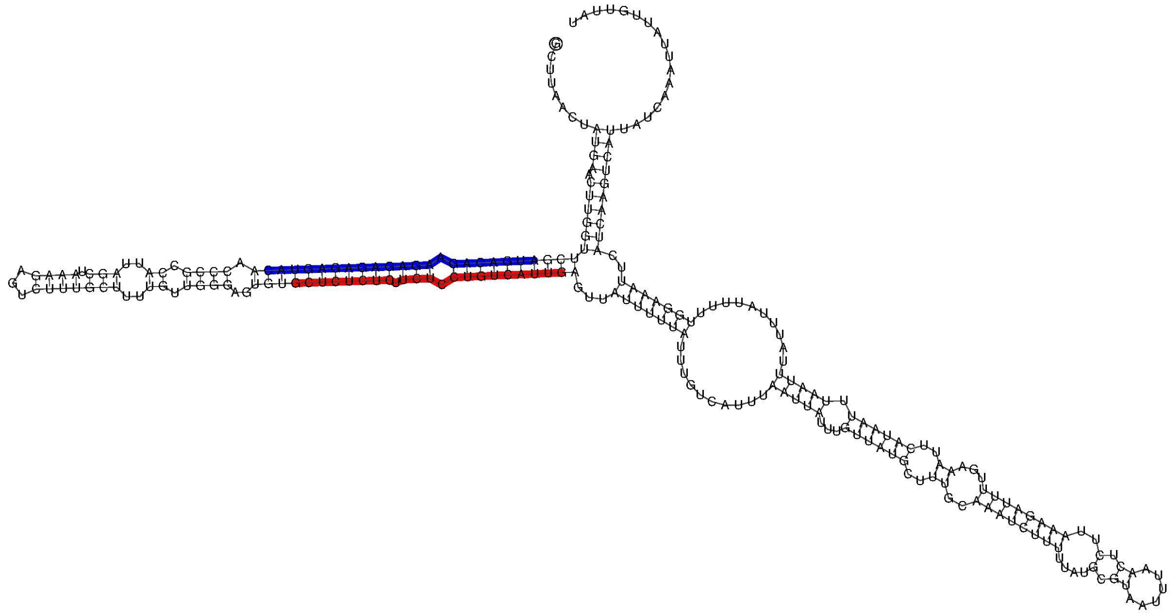

csi-MIRN38

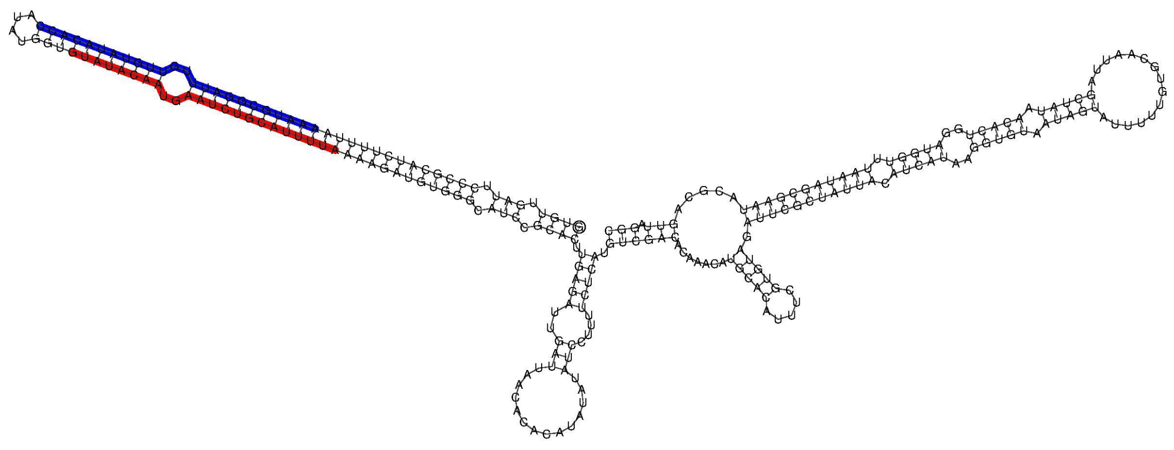

csi-MIRN39

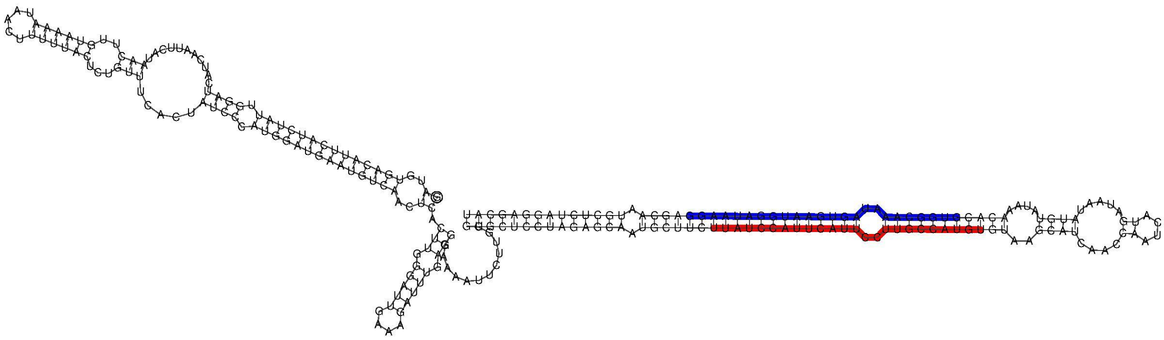

csi-MIRN40

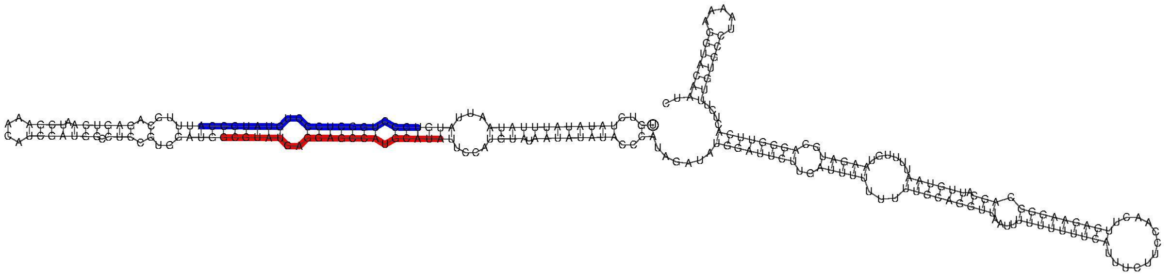

csi-MIRN41

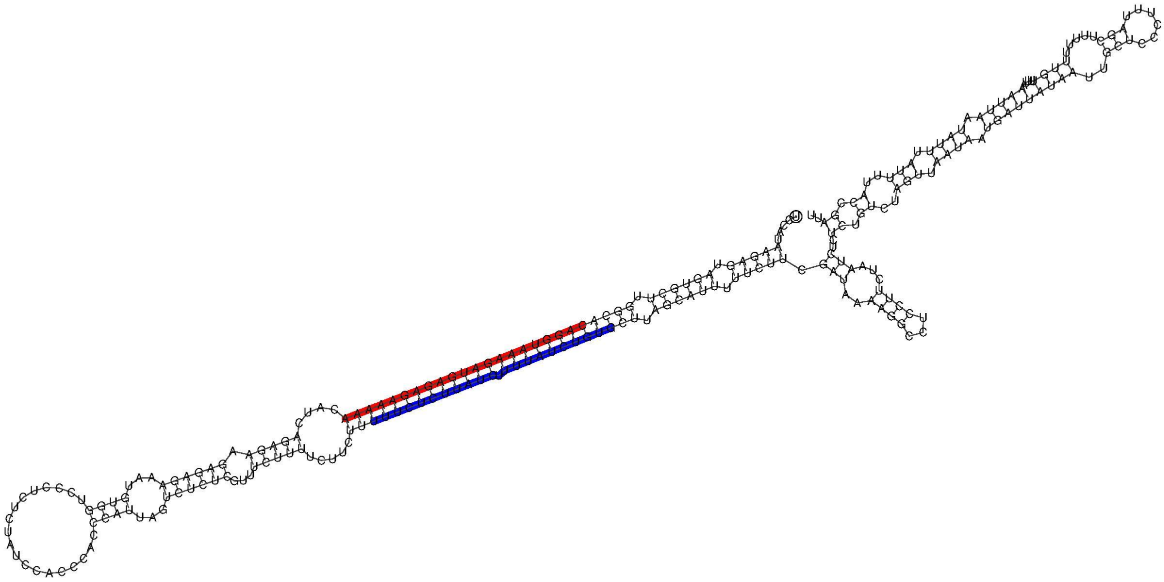

csi-MIRN42

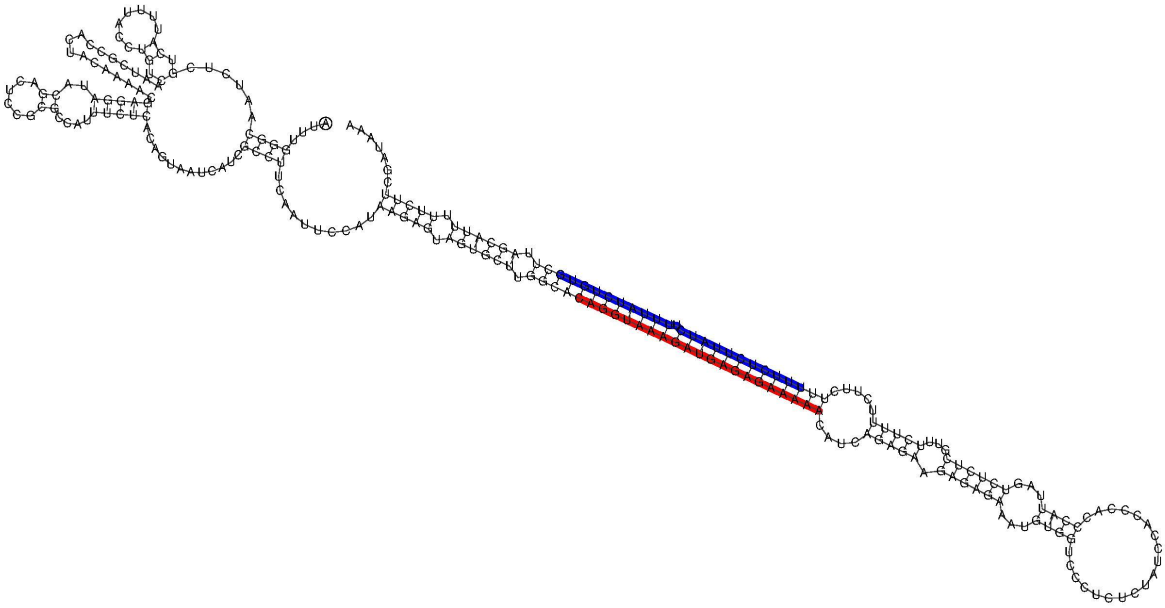

csi-MIRN43

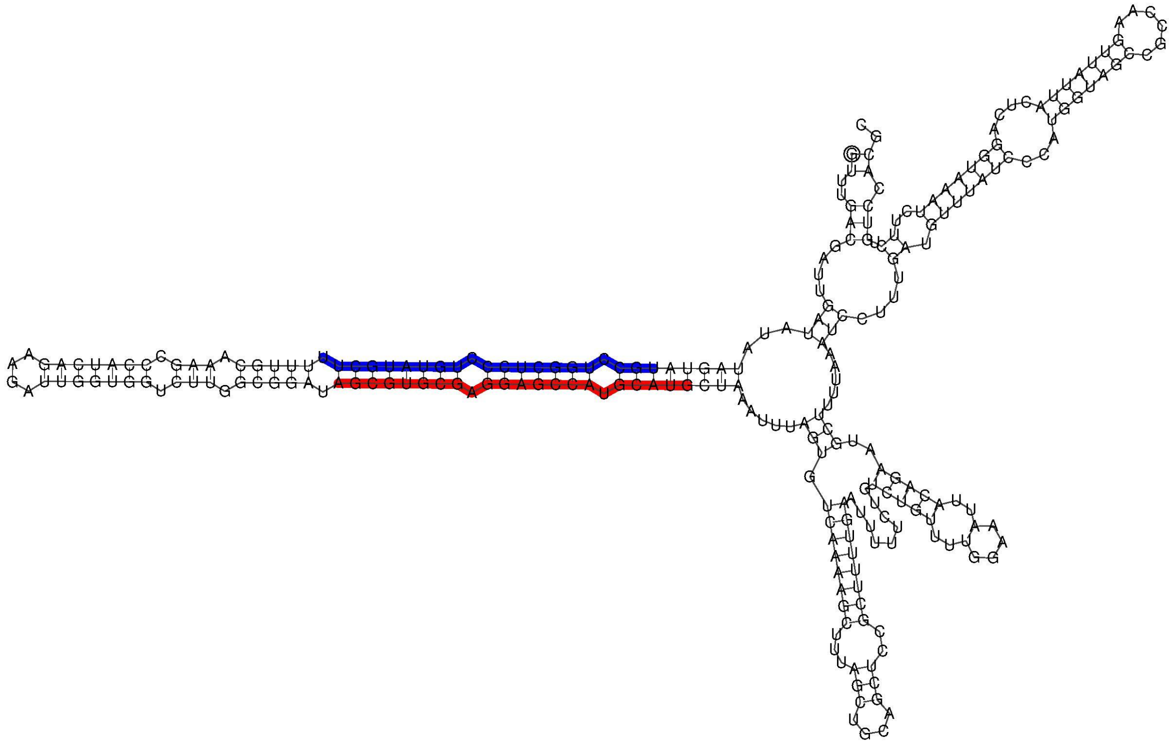

csi-MIRN44

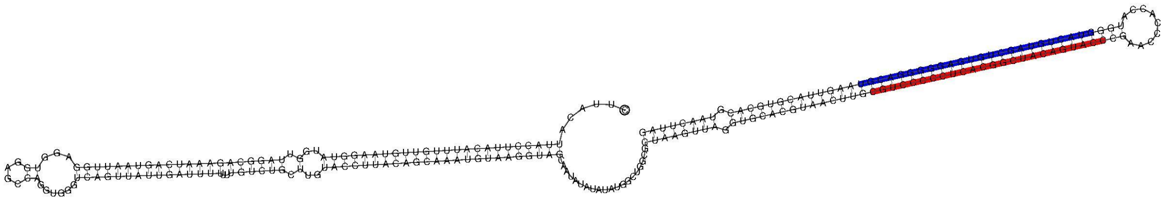

csi-MIRN45

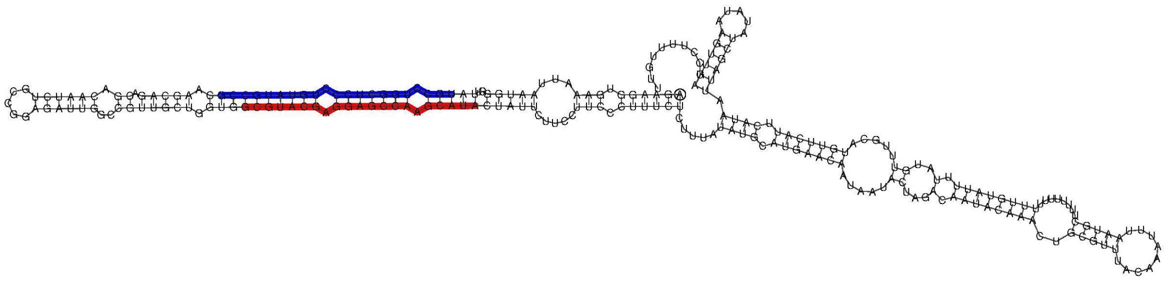

csi-MIRN46

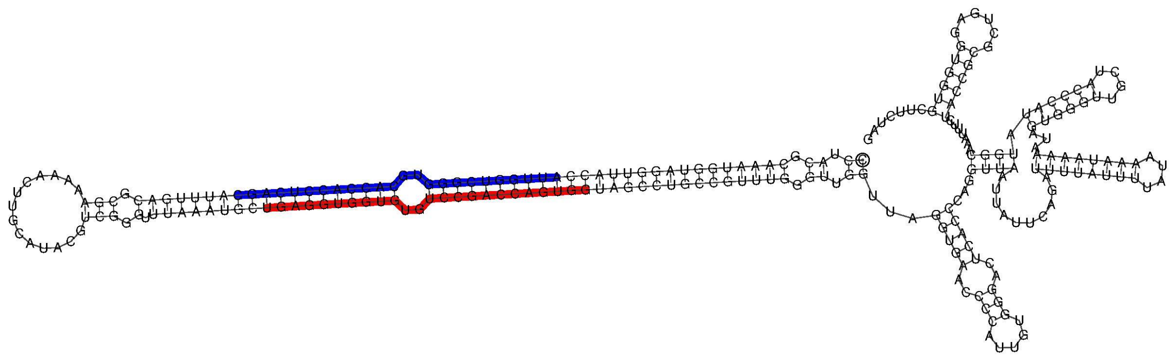

csi-MIRN47

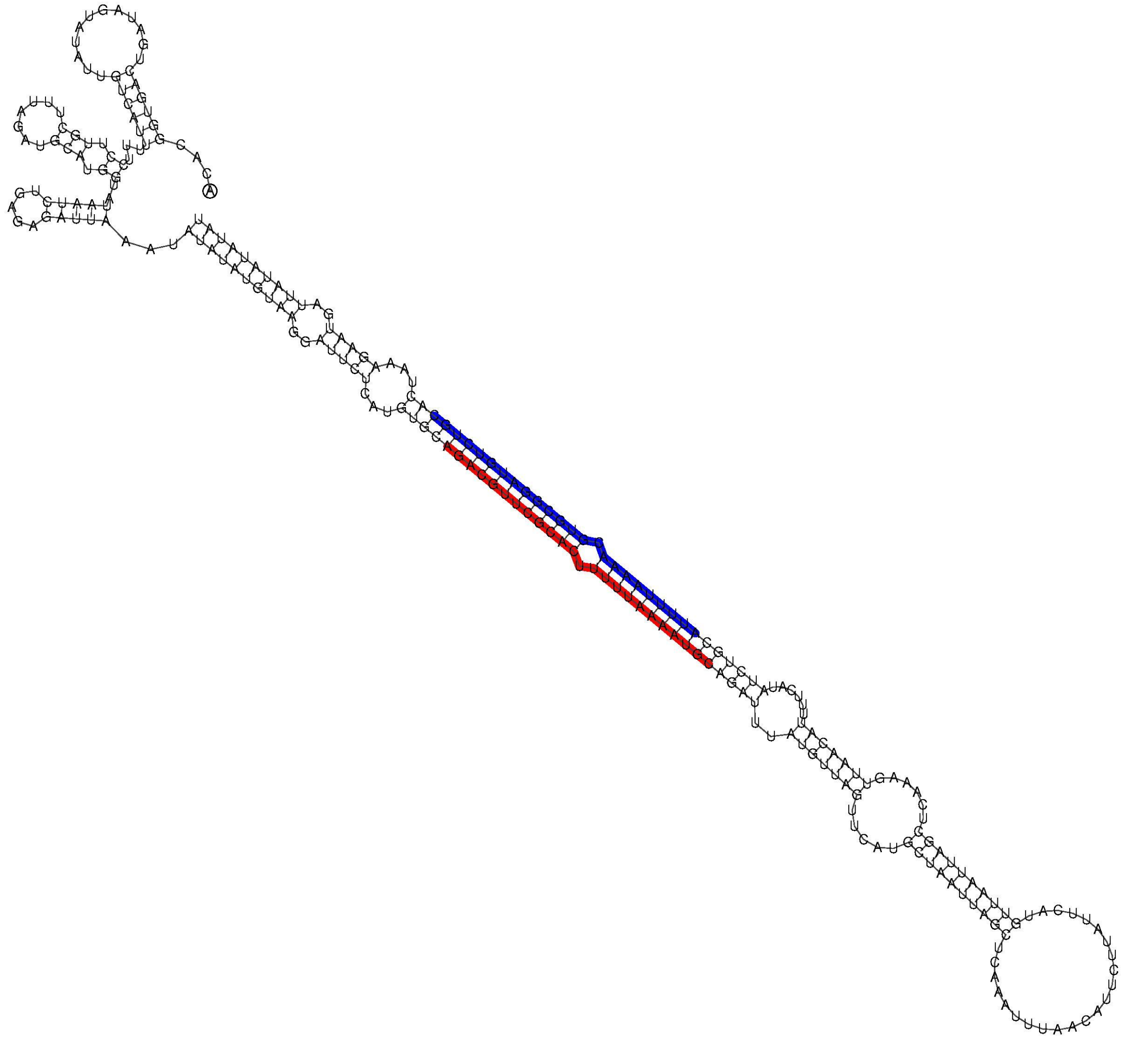

csi-MIRN48

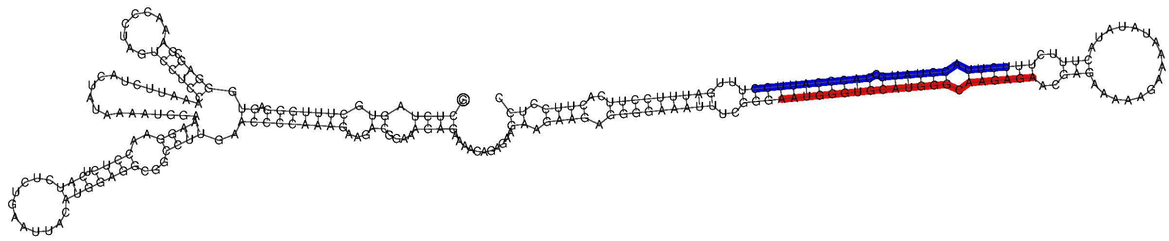

csi-MIRN49

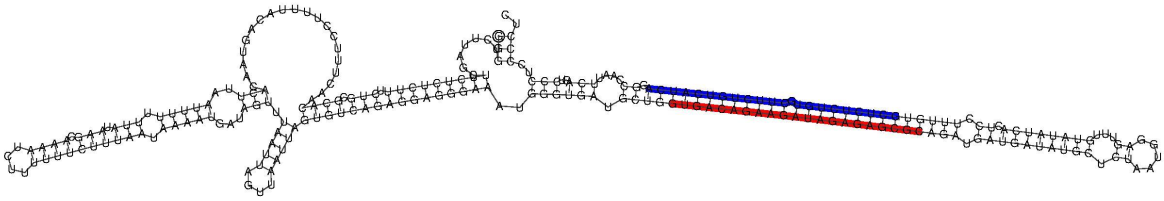

csi-MIRN50

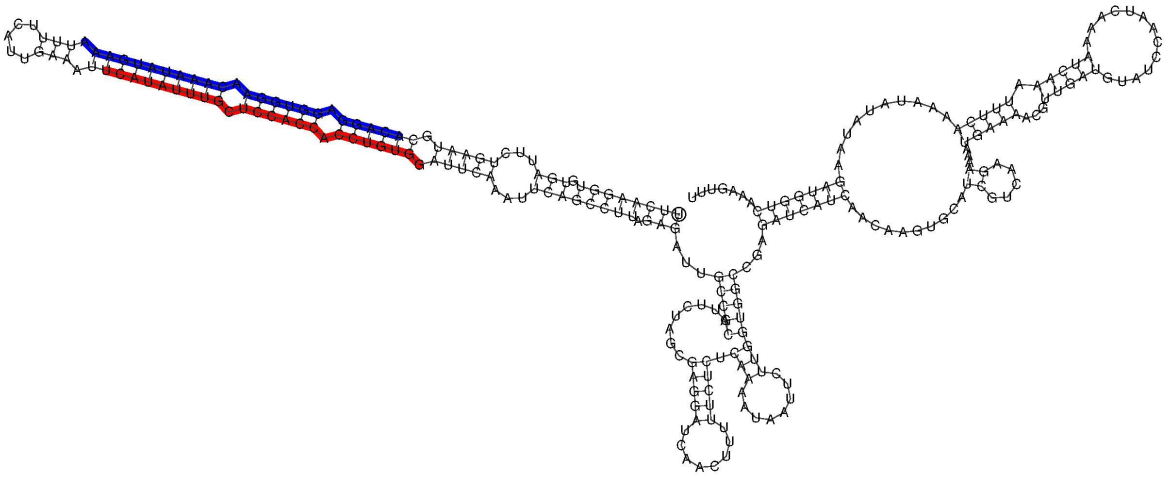

csi-MIRN51

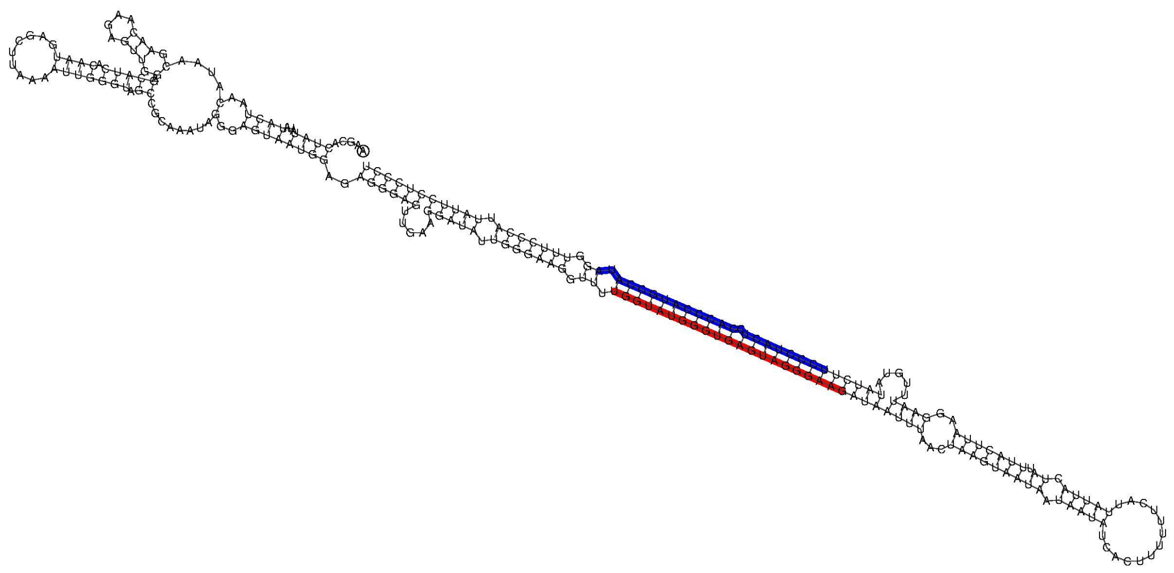

csi-MIRN52

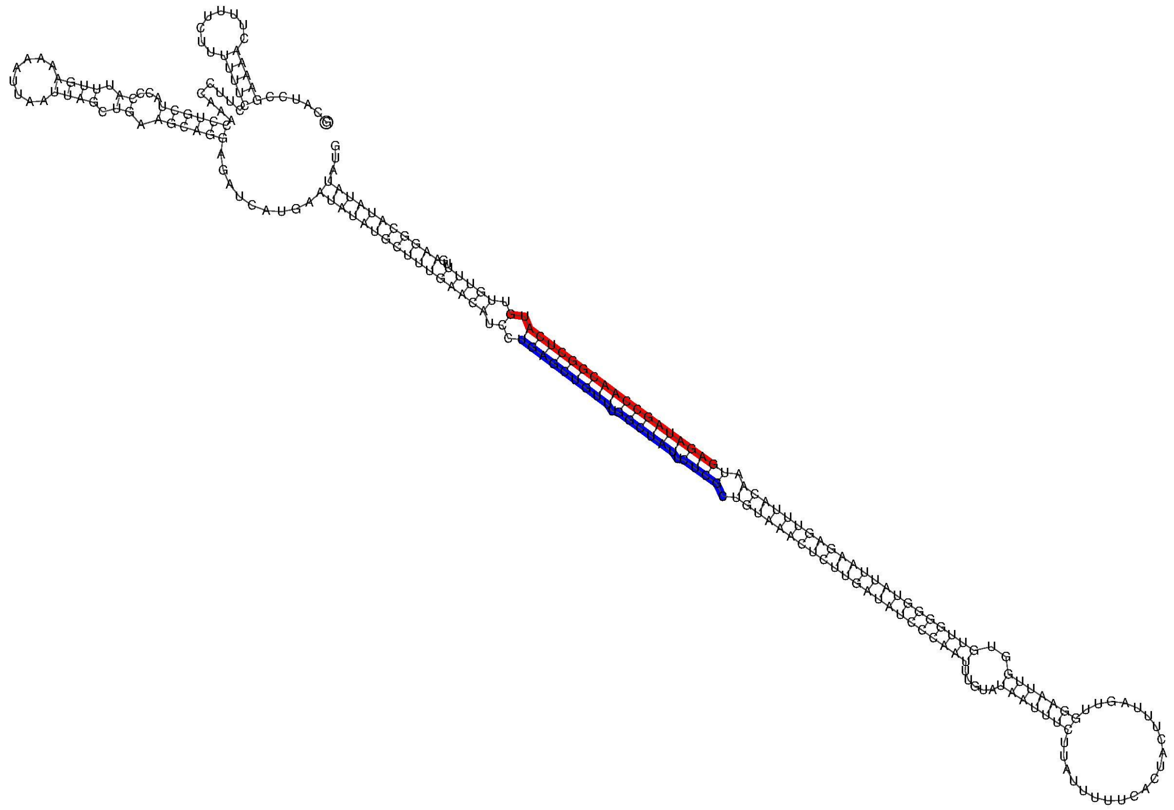

csi-MIRN52

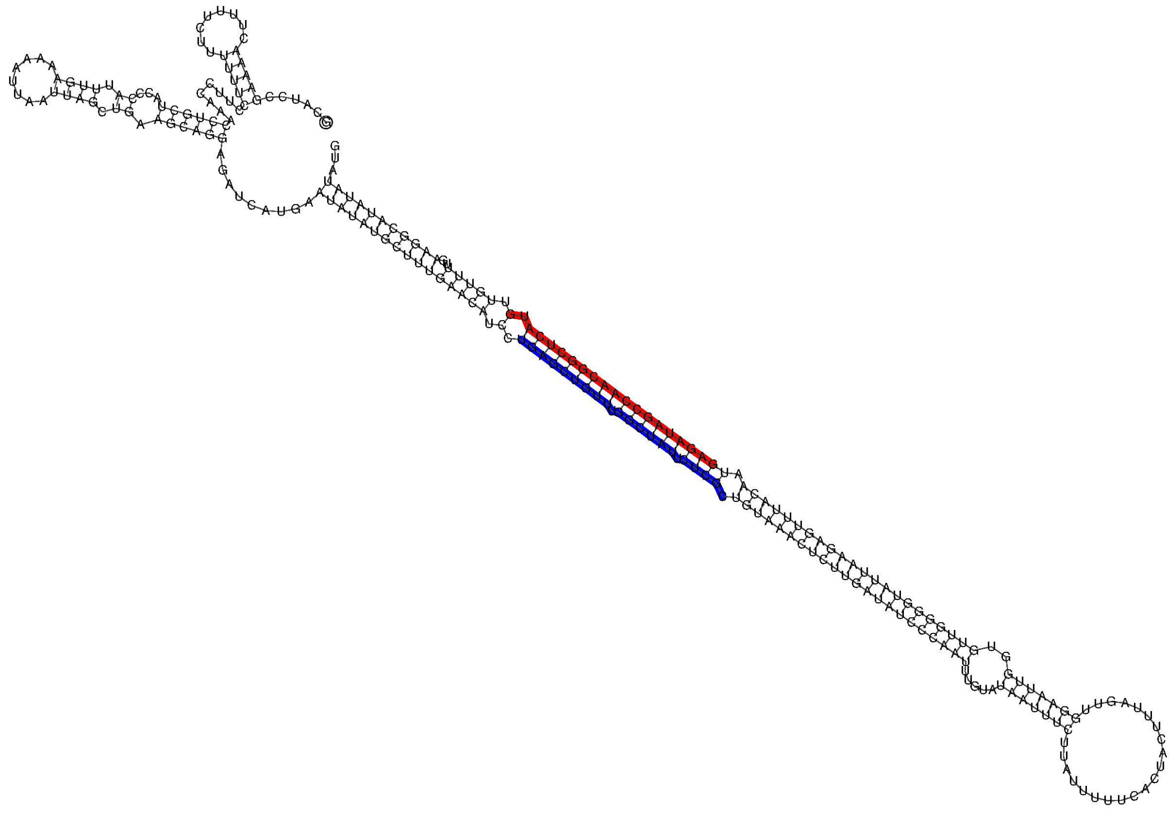

csi-MIRN52

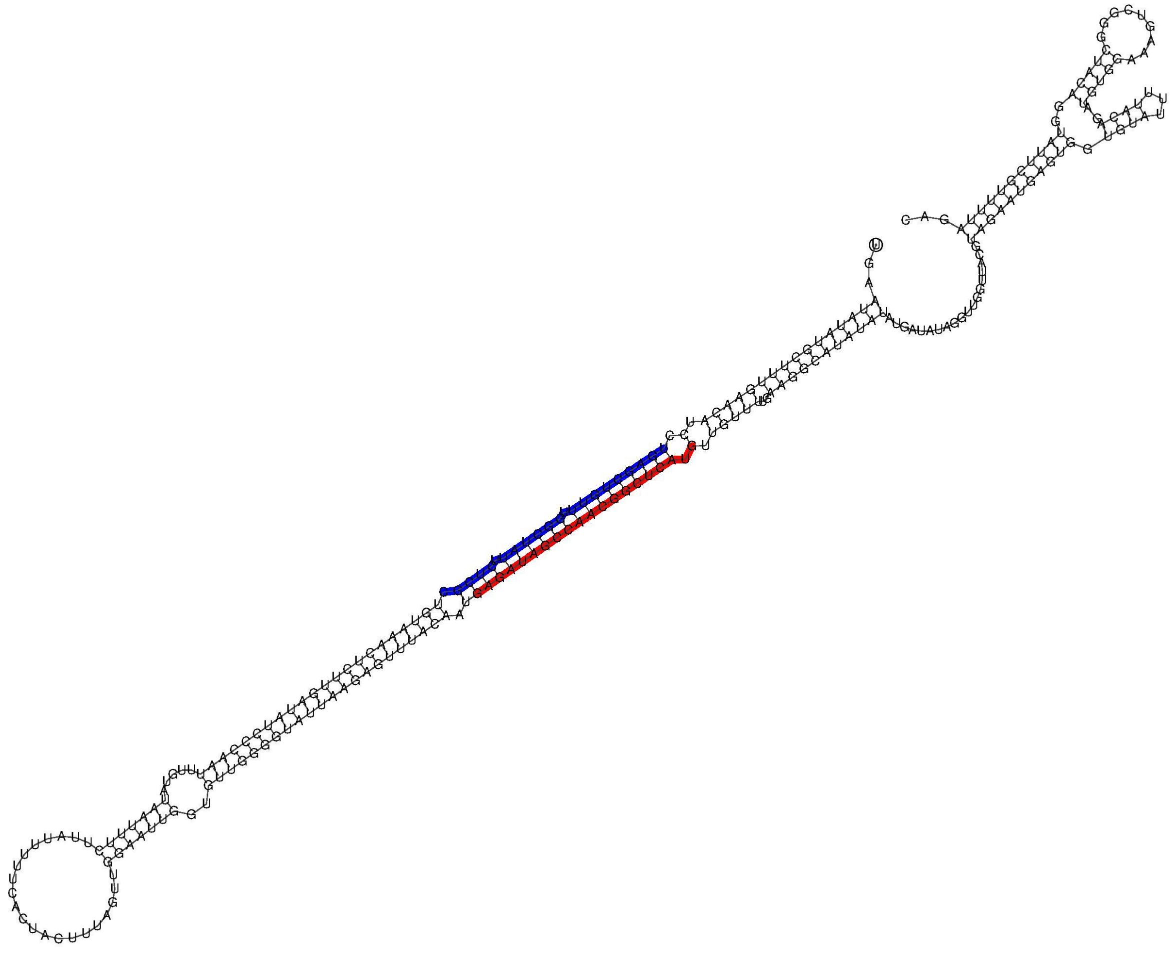

csi-MIRN52

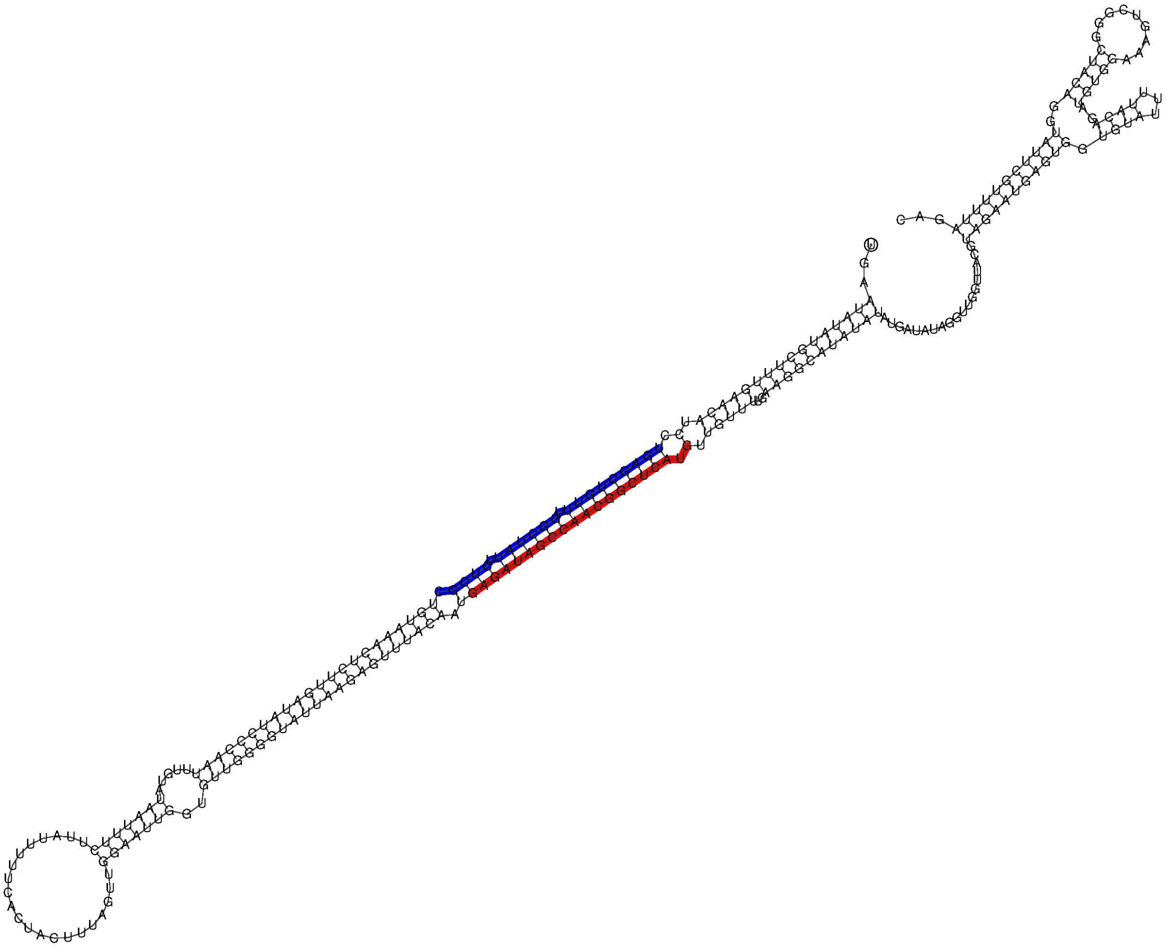

Supplement: Supplementary file 7 — Figure S5 [file 41438_2018_116_MOESM7_ESM.pdf]
