# Supplementary material for: Genome-wide comprehensive analysis of transcriptomes and small RNAs offers insights into the molecular mechanism of alkaline stress tolerance in a citrus rootstock
Source: Hortic Res. 2019 Mar 1;6:33. doi: 10.1038/s41438-018-0116-0 (PMC6395741; doi:10.1038/s41438-018-0116-0)
Supplement: Supplementary file 8 — Figure S6 [file 41438_2018_116_MOESM8_ESM.pdf]

orange1.1t05622.1 gene=orange1.1t05622 CDS=3-347

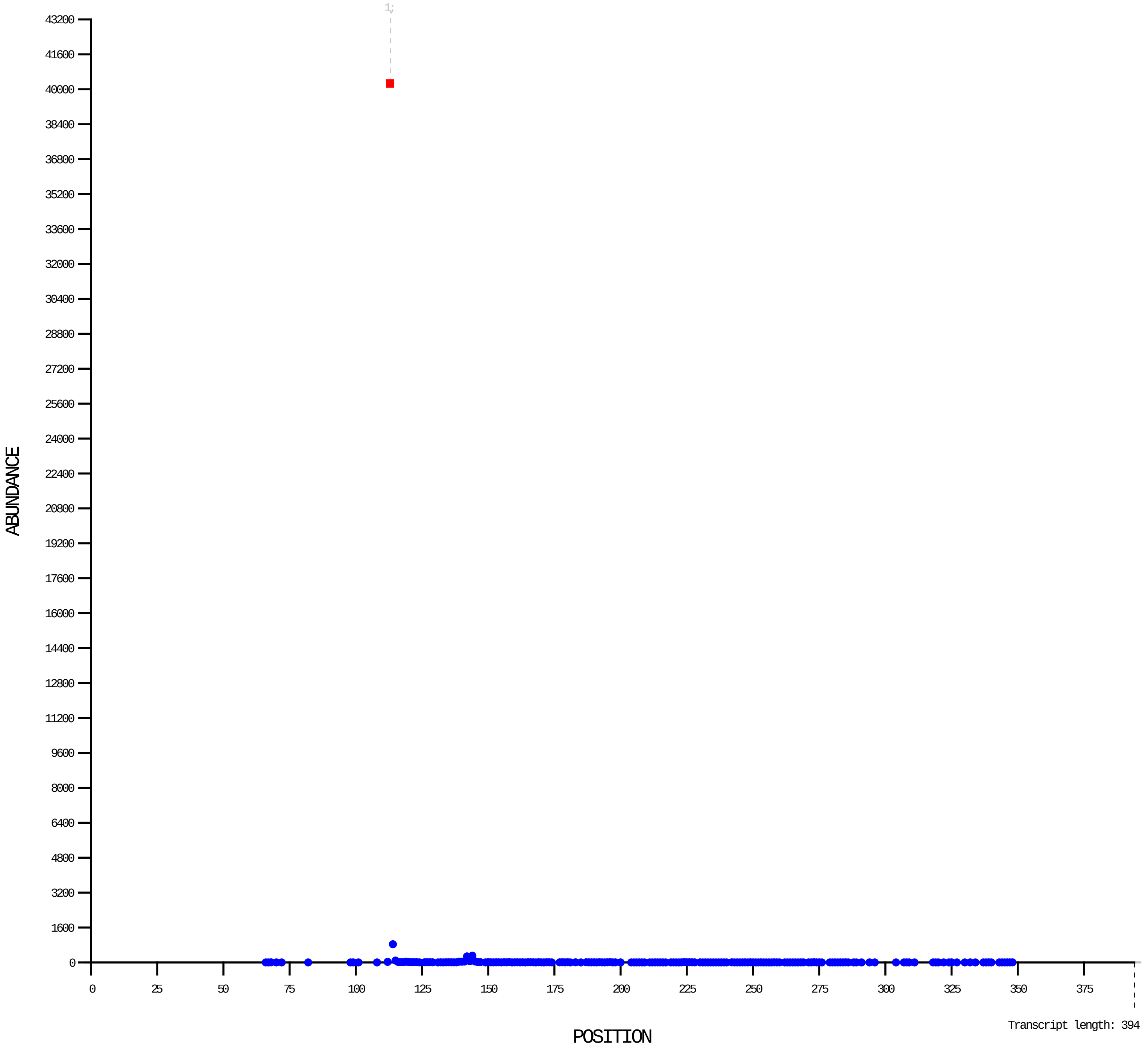

Category: 0 1 2 3 4

Degradome alignment: ● Median:

```
0  #1  Position:113  Abundance: 40271.00(deg) 1(sRNA)
   5'     TAGATAAAGATGAGAGAAAAA        3'  ID:
          o|||||||||| ||||||||||           Score: 1.5
   3' GGGTGTCTATTTCT-CTCTCTTTTTTTTTCTC 5'  p-value: 0.0
```

Cs1g06060.1 gene=Cs1g06060 CDS=1-387

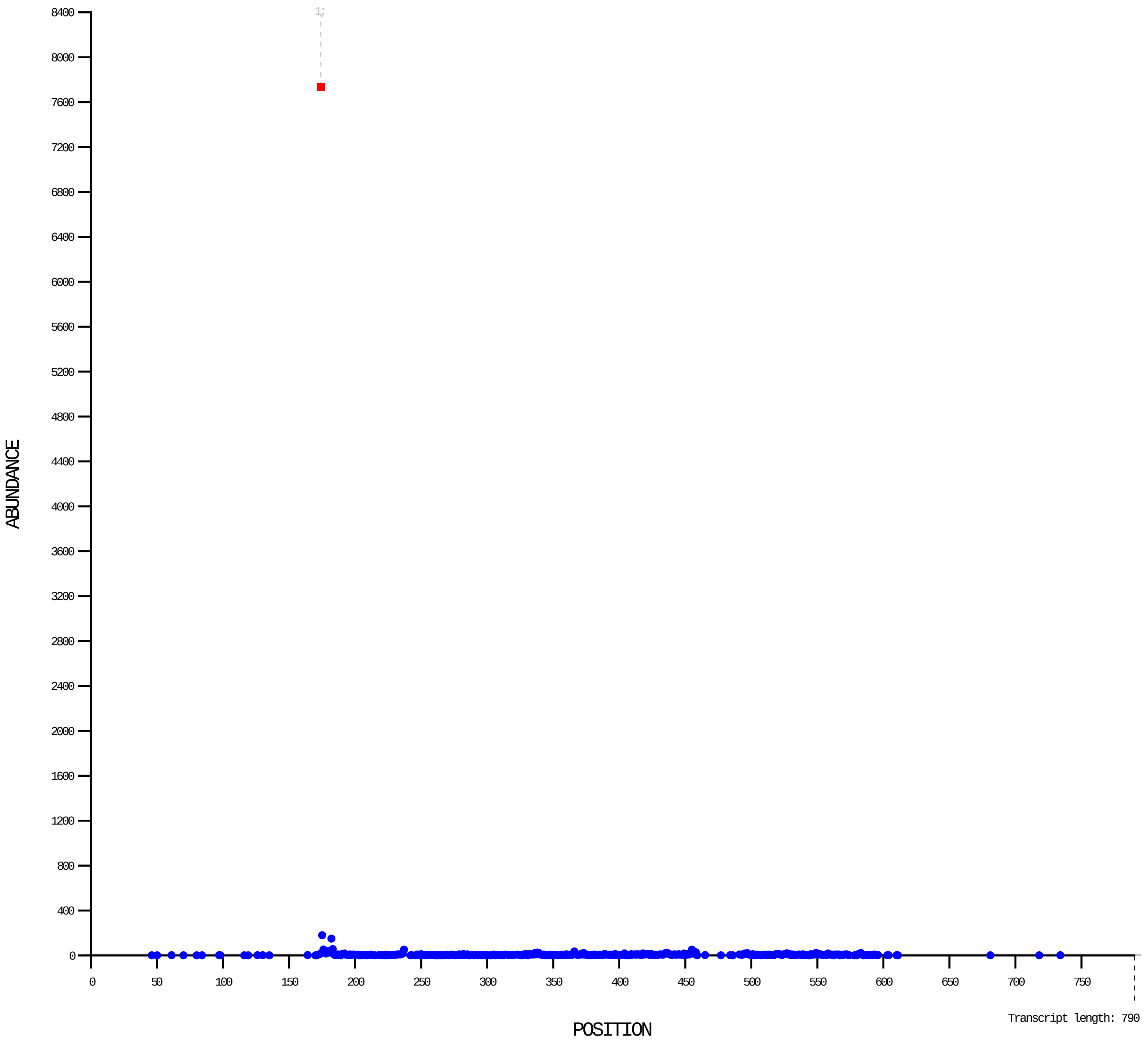

Category: 0 1 2 3 4

Degradome alignment: ● Median: —

```
0  #1  Position:174  Abundance: 7736.00(deg) 1(sRNA)
   5'     TAGATAAAGATGAGAGAAAAA        3'  ID:
          o||||||||| ||||||||||            Score: 1.5
   3' GGGTGTCTATTTCT-CTCTCTTTTTTTTTTCCC 5'  p-value: 0.0
```

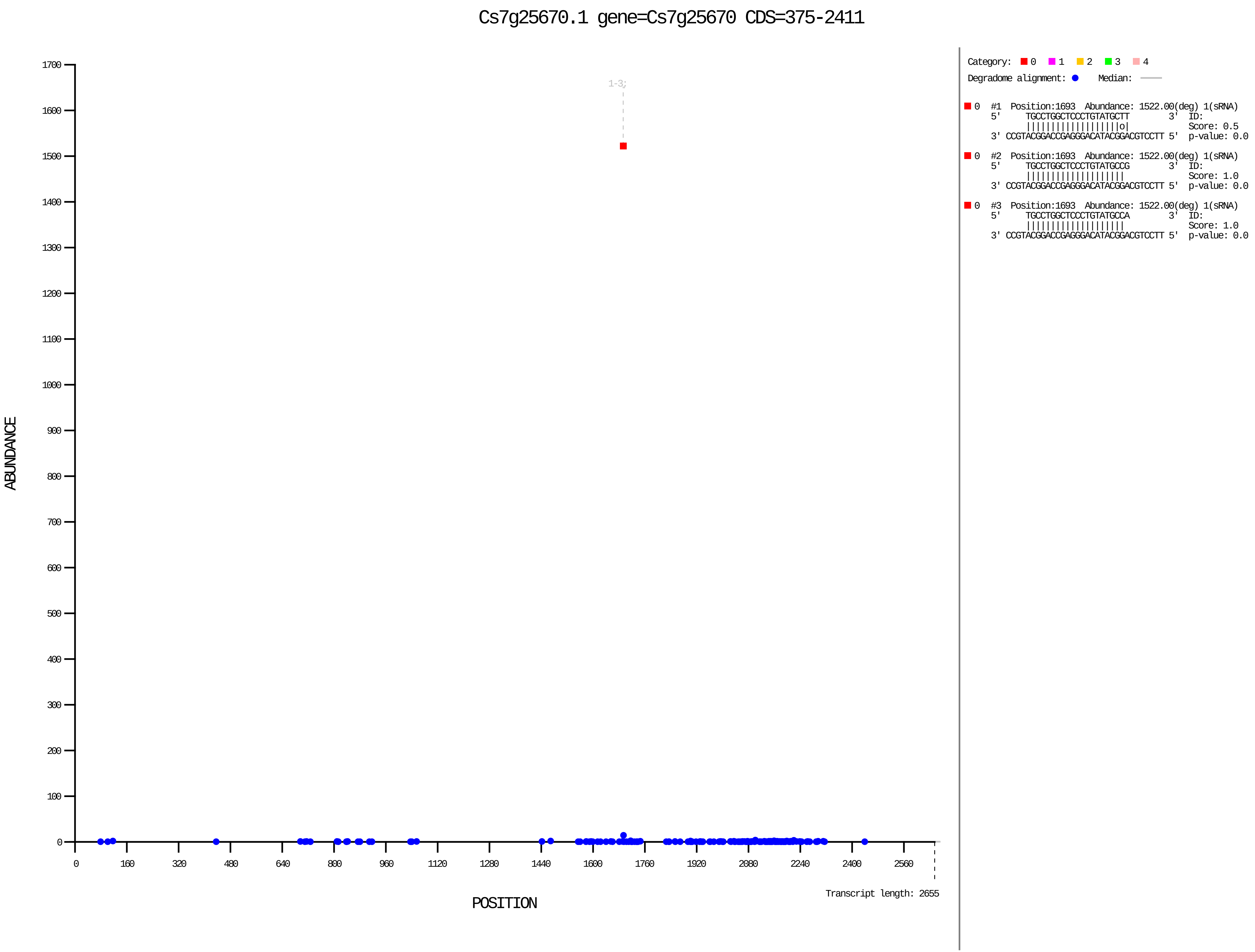
Cs7g25670.1 gene=Cs7g25670 CDS=375-2411
ABUNDANCE
POSITION
Transcript length: 2655
1-3;
Category: 0 1 2 3 4
Degradome alignment: Median:
0 #1 Position:1693 Abundance: 1522.00(deg) 1(sRNA)
5' TGCCTGGCTCCCTGTATGCTT 3' ID:
||||||||||||||||||o|
Score: 0.5
3' CCGTACGGACCGAGGGACATACGGACGTCCTT 5' p-value: 0.0
0 #2 Position:1693 Abundance: 1522.00(deg) 1(sRNA)
5' TGCCTGGCTCCCTGTATGCCG 3' ID:
||||||||||||||||||||
Score: 1.0
3' CCGTACGGACCGAGGGACATACGGACGTCCTT 5' p-value: 0.0
0 #3 Position:1693 Abundance: 1522.00(deg) 1(sRNA)
5' TGCCTGGCTCCCTGTATGCCA 3' ID:
||||||||||||||||||||
Score: 1.0
3' CCGTACGGACCGAGGGACATACGGACGTCCTT 5' p-value: 0.0

Cs7g25670.2 gene=Cs7g25670 CDS=375-2459

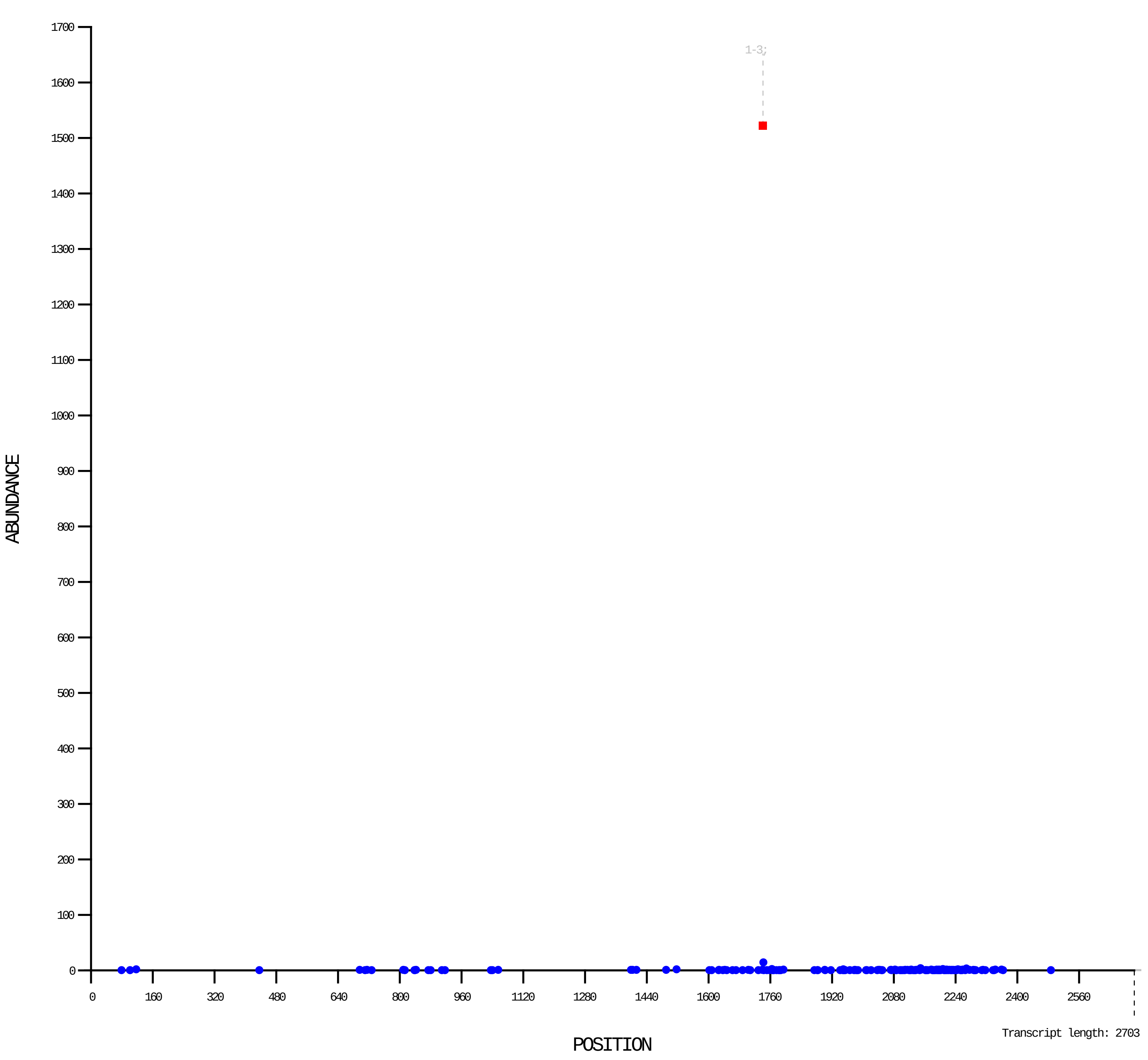

Category: 0 1 2 3 4

Degradome alignment: ● Median:

```
0  #1  Position:1741  Abundance: 1522.00(deg) 1(sRNA)
   5'     TGCCTGGCTCCCTGTATGCTT        3'  ID:
          ||||||||||||||||||o|             Score: 0.5
   3' CCGTACGGACCGAGGGACATACGGACGTCCTT 5'  p-value: 0.0

0  #2  Position:1741  Abundance: 1522.00(deg) 1(sRNA)
   5'     TGCCTGGCTCCCTGTATGCCG        3'  ID:
          ||||||||||||||||||||             Score: 1.0
   3' CCGTACGGACCGAGGGACATACGGACGTCCTT 5'  p-value: 0.0

0  #3  Position:1741  Abundance: 1522.00(deg) 1(sRNA)
   5'     TGCCTGGCTCCCTGTATGCCA        3'  ID:
          ||||||||||||||||||||             Score: 1.0
   3' CCGTACGGACCGAGGGACATACGGACGTCCTT 5'  p-value: 0.0
```

Cs5g06580.1 gene=Cs5g06580 CDS=84-671

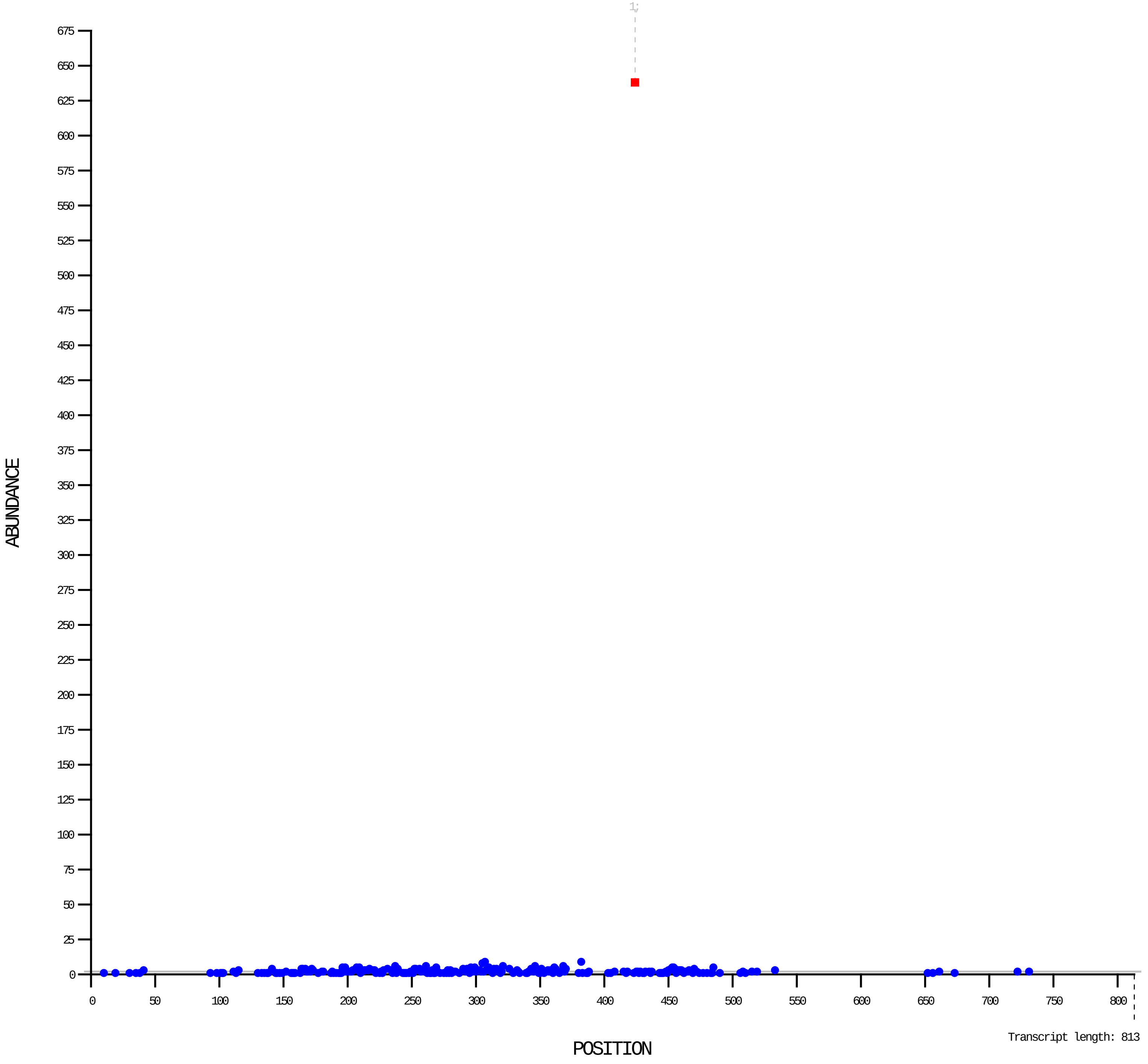


```
Category: 0  1  2  3  4
Degradome alignment:      Median:

0  #1  Position:424  Abundance: 638.00(deg) 1(sRNA)
   5'     TGTGTTCTCAGGTCACC-CCTT       3'  ID:
          |||||o|o|| |||||| ||o|           Score: 3.5
   3' CATGACACAGGGGTGCAGTGGAGGGACCGCTA 5'  p-value: 0.0
```

Cs3g12000.2 gene=Cs3g12000 CDS=235-693

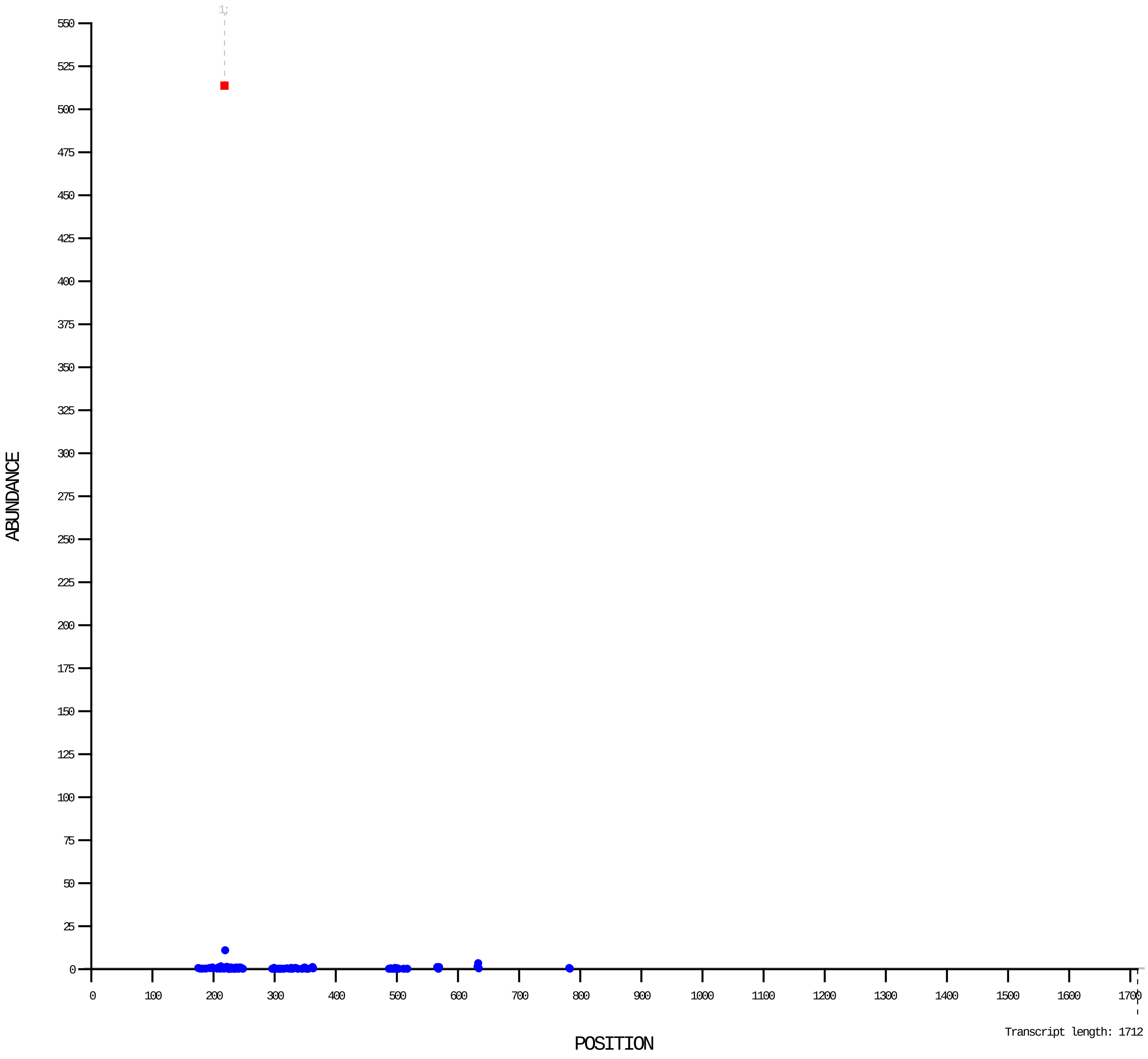

Category: 0 1 2 3 4
Degradome alignment: Median:

0 #1 Position:218 Abundance: 513.67(deg) 1(sRNA)

```
5'     TGTGTTCTCAGGTCACCCCTT        3'  ID:
       |||| ||||||o ||||||||            Score: 2.5
3' CGACACACTAGAGTCTTGTGGGGAACCTCCCT 5'  p-value: 0.0
```

Cs3g12000.3 gene=Cs3g12000 CDS=235-717

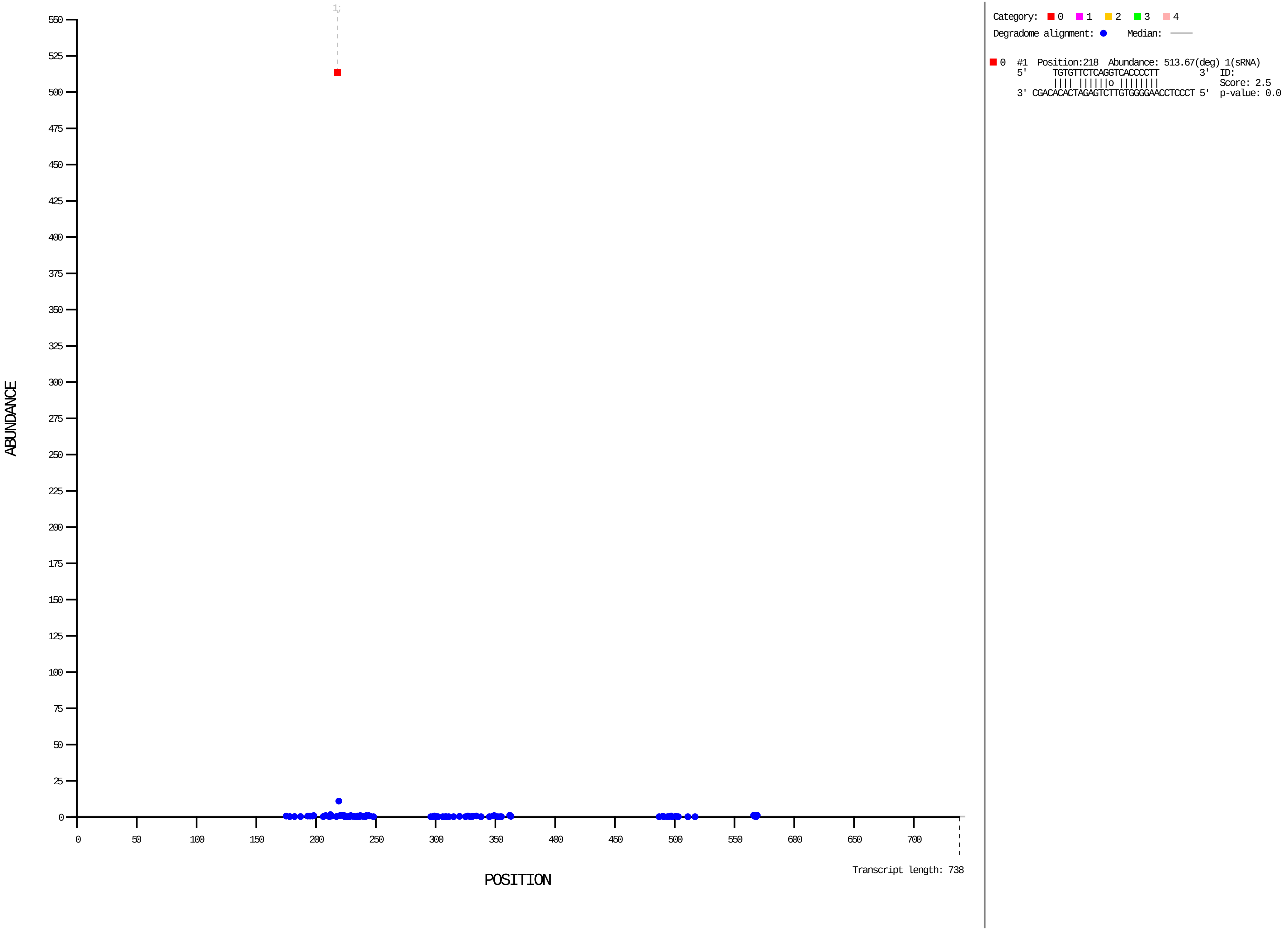

Cs3g12000.4 gene=Cs3g12000 CDS=235-705

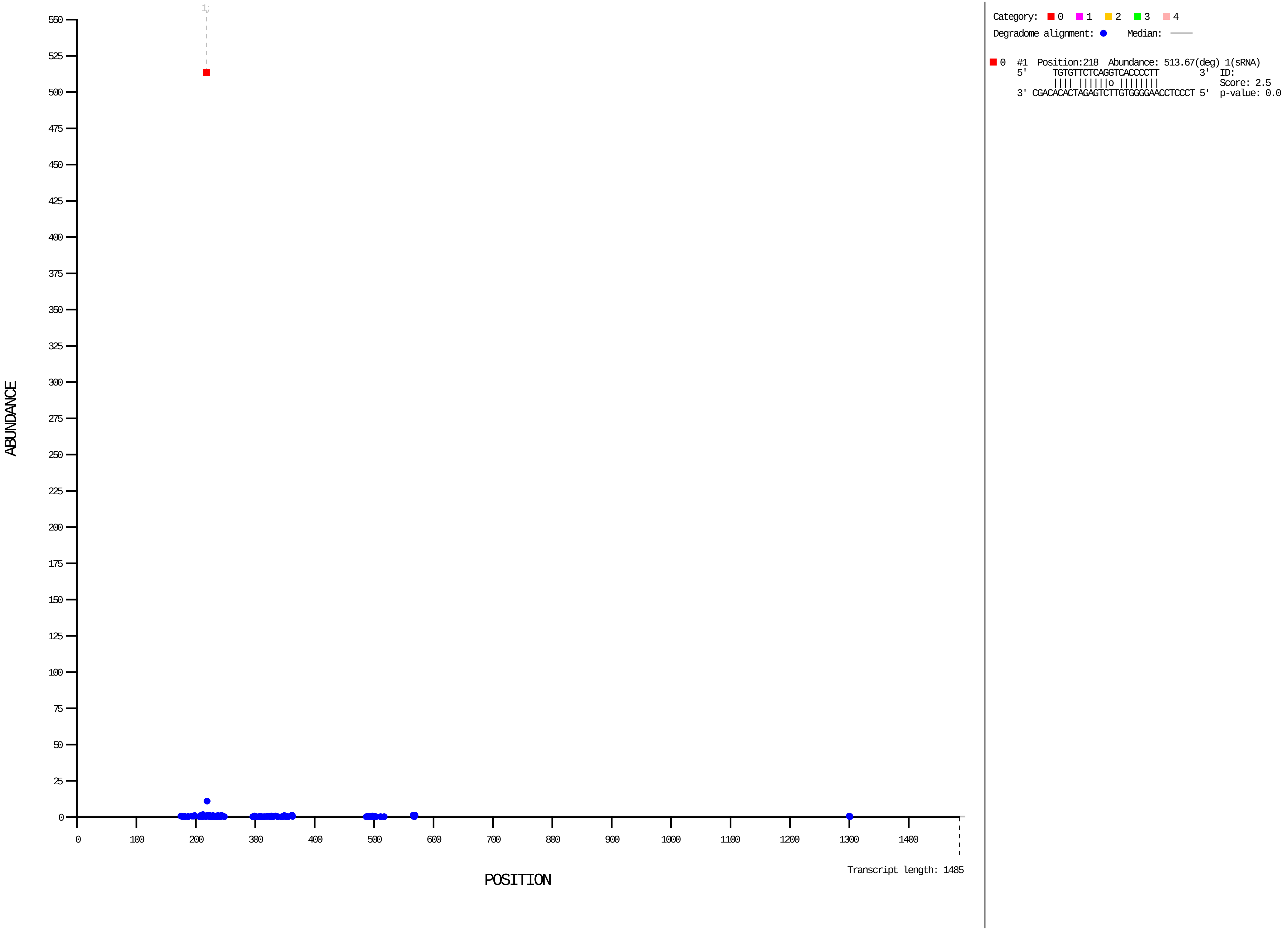

Cs3g18940.1 gene=Cs3g18940 CDS=177-1901

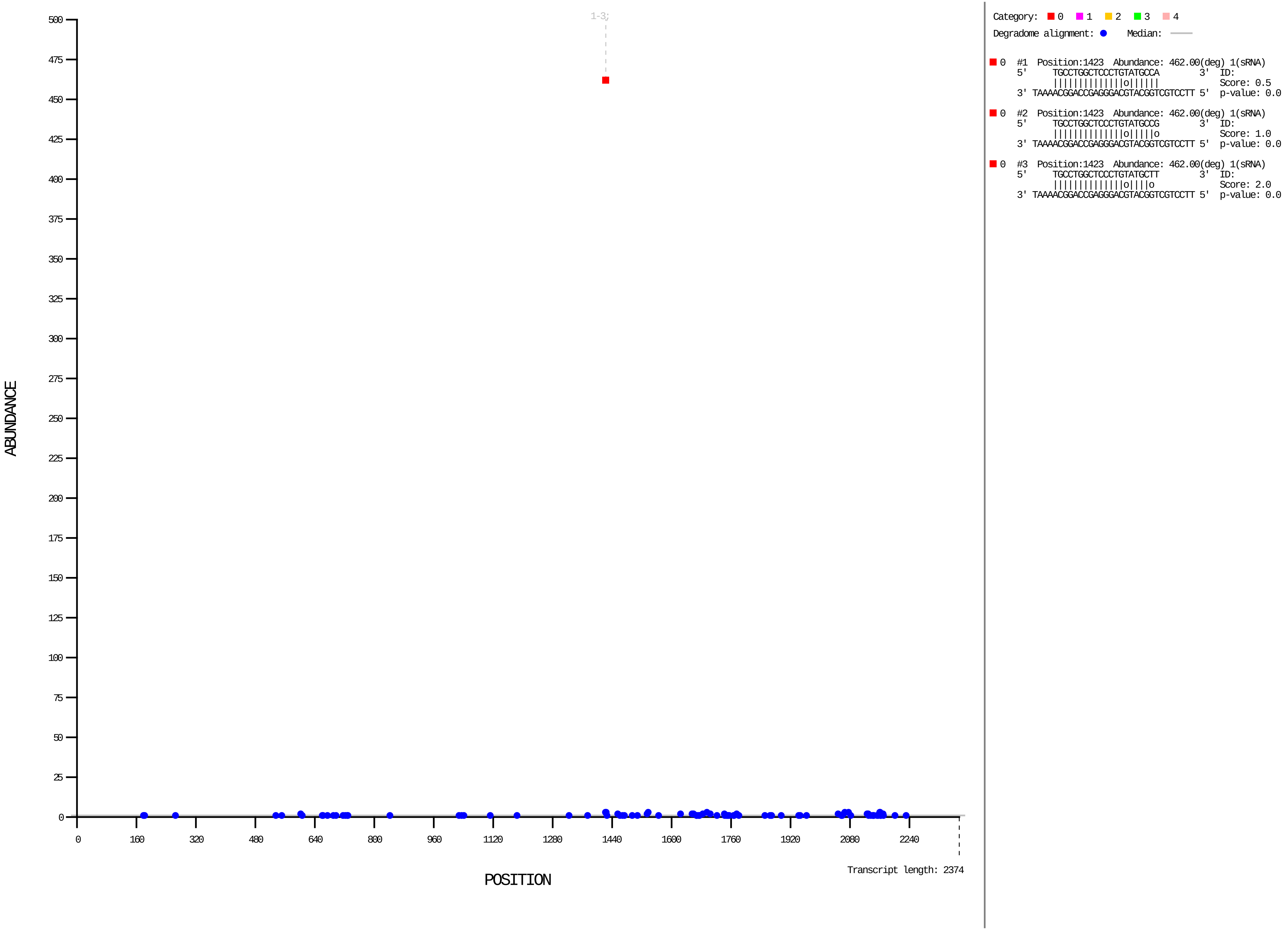

Cs3g06390.1 gene=Cs3g06390 CDS=101-1471

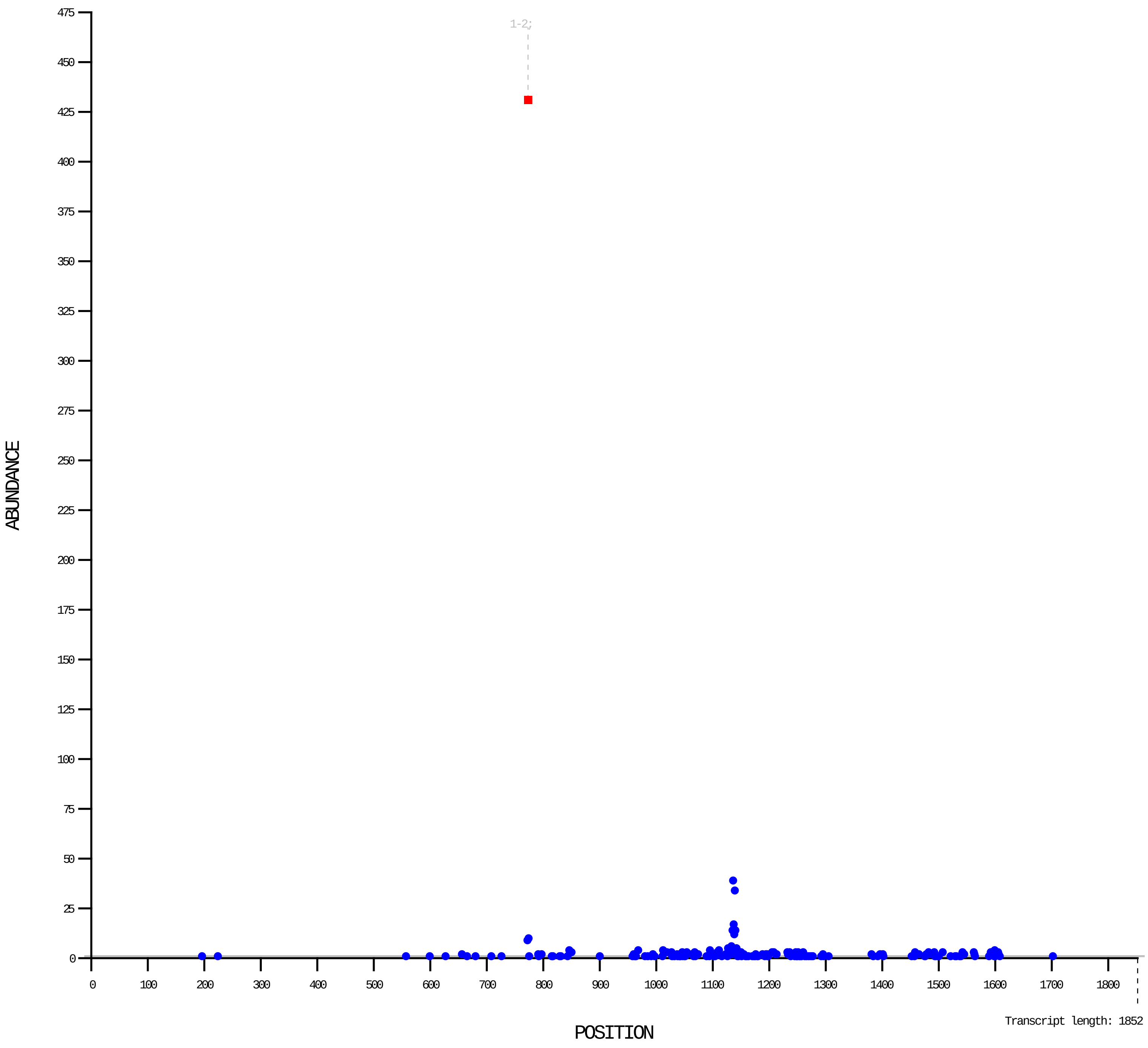

Category: 0 1 2 3 4

Degradome alignment: ● Median: —

0 #1 Position:773 Abundance: 431.00(deg) 1(sRNA)

```
5'     TTTGGATTGAAGGGAGCTCTA        3'  ID:
        ||||| ||||||||||||o|            Score: 2.5
3' CATATAACCTCACTTCCCTCGAGGTTGAAGTG 5'  p-value: 0.0
```

0 #2 Position:773 Abundance: 431.00(deg) 1(sRNA)

```
5'     TTTGGACTGAAGGGAGCTCCT        3'  ID:
        ||||| |||||||||||||             Score: 3.0
3' CATATAACCTCACTTCCCTCGAGGTTGAAGTG 5'  p-value: 0.0
```

Cs8g16440.1 gene=Cs8g16440 CDS=606-2780

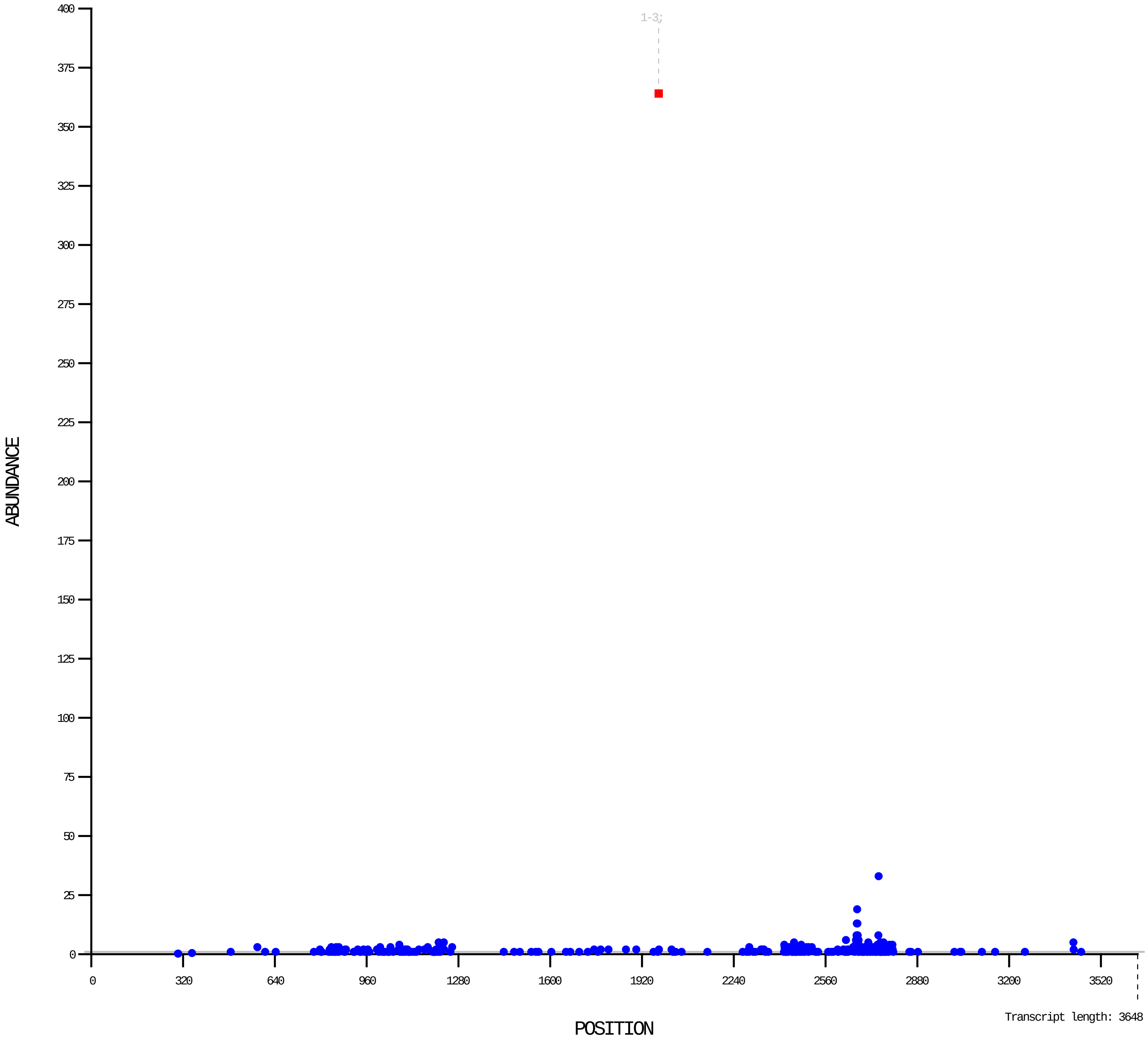

Category: 0 1 2 3 4
Degradome alignment: Median:

0 #1 Position:1978 Abundance: 364.00(deg) 1(sRNA)
```
5'     TGCCTGGCTCCCTGTATGCTT        3'  ID:
       ||||||||||||||||||o|             Score: 0.5
3' TCGTACGGACCGAGGGACATACGGACGTCTTC 5'  p-value: 0.0
```

0 #2 Position:1978 Abundance: 364.00(deg) 1(sRNA)
```
5'     TGCCTGGCTCCCTGTATGCCG        3'  ID:
       ||||||||||||||||||||             Score: 1.0
3' TCGTACGGACCGAGGGACATACGGACGTCTTC 5'  p-value: 0.0
```

0 #3 Position:1978 Abundance: 364.00(deg) 1(sRNA)
```
5'     TGCCTGGCTCCCTGTATGCCA        3'  ID:
       ||||||||||||||||||||             Score: 1.0
3' TCGTACGGACCGAGGGACATACGGACGTCTTC 5'  p-value: 0.0
```

Cs5g10870.2 gene=Cs5g10870 CDS=88-987

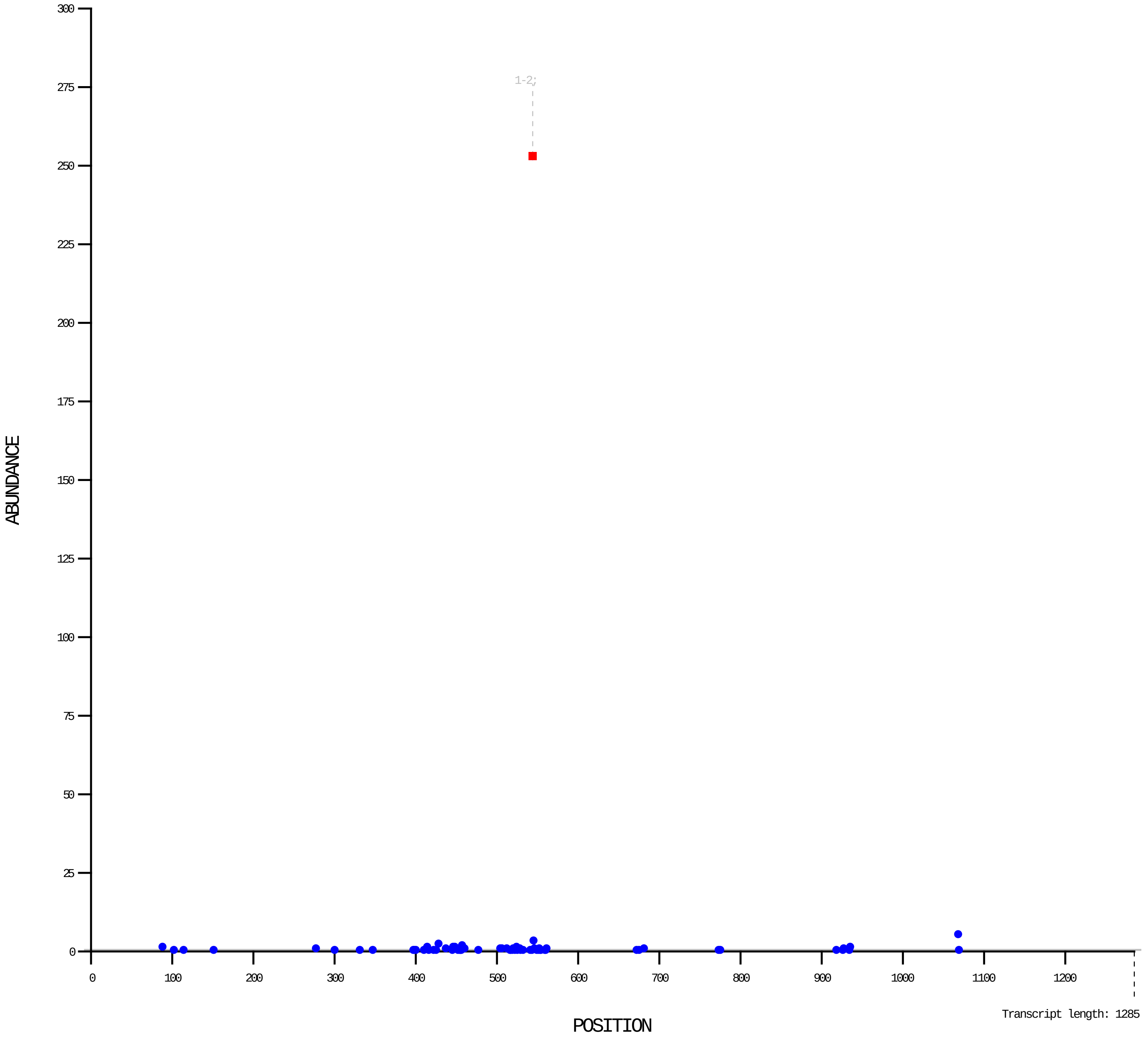

Category: 0 1 2 3 4
Degradome alignment: ● Median:

0 #1 Position:544 Abundance: 253.00(deg) 1(sRNA)
```
5'     TGGAGAAGCAGGGCACGTGCA          3'  ID:
       ||||||||||||o|||||o                Score: 3.0
3' CTTAACCTCTTCGTCCTGTGCATTCGGCTTAG 5'  p-value: 0.0
```

0 #2 Position:544 Abundance: 253.00(deg) 1(sRNA)
```
5'     TGGAGAAGCAGGGCACATGCT          3'  ID:
       ||||||||||||o||| |o                Score: 4.0
3' CTTAACCTCTTCGTCCTGTGCATTCGGCTTAG 5'  p-value: 0.0
```

Cs5g10870.1 gene=Cs5g10870 CDS=408-1505

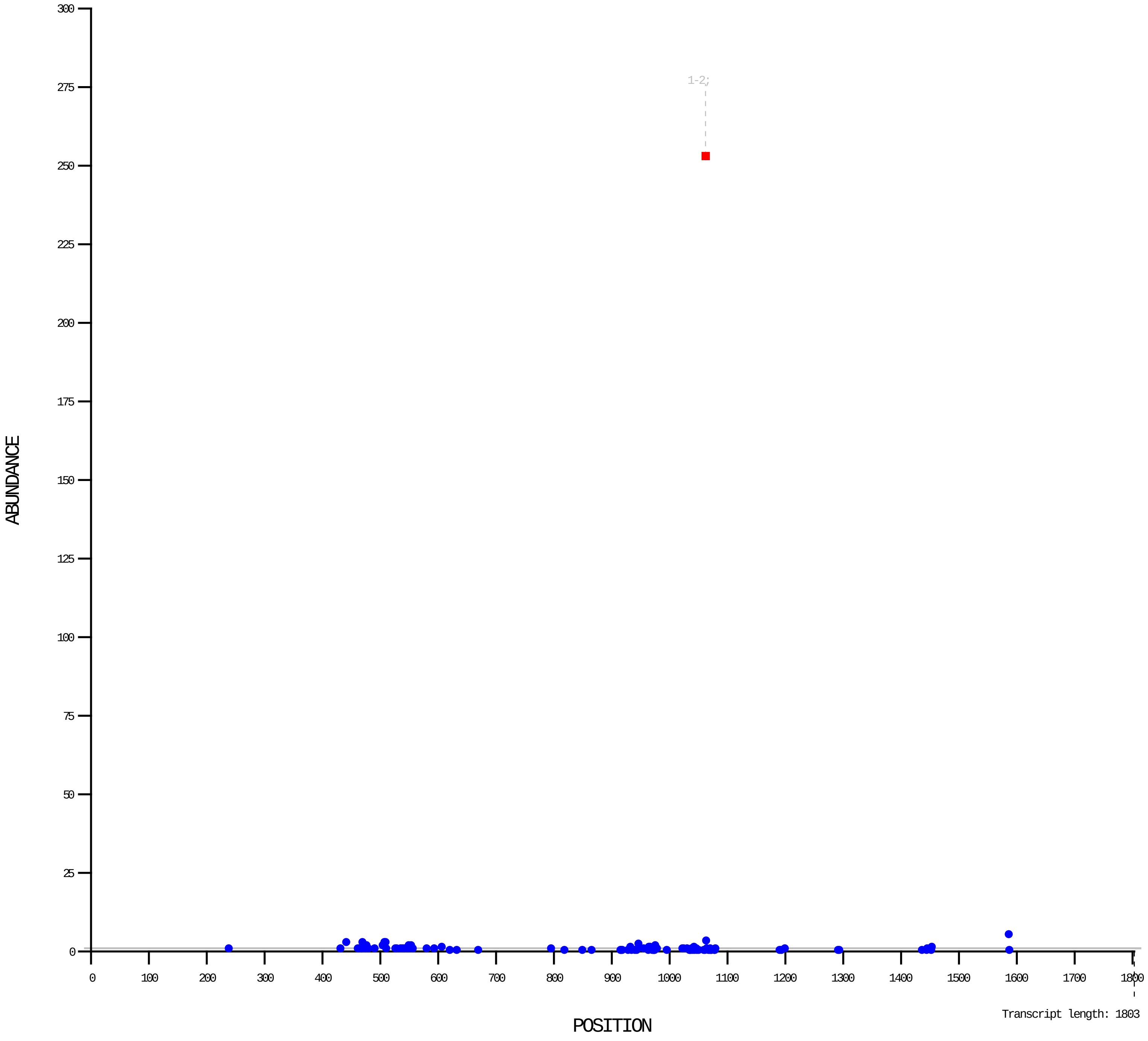

Category: 0 1 2 3 4
Degradome alignment: ● Median:

0 #1 Position:1062 Abundance: 253.00(deg) 1(sRNA)
```
5'     TGGAGAAGCAGGGCACGTGCA        3'  ID:
       ||||||||||||o|||||o              Score: 3.0
3' CTTAACCTCTTCGTCCTGTGCATTCGGCTTAG 5'  p-value: 0.0
```

0 #2 Position:1062 Abundance: 253.00(deg) 1(sRNA)
```
5'     TGGAGAAGCAGGGCACATGCT        3'  ID:
       ||||||||||||o||| |o              Score: 4.0
3' CTTAACCTCTTCGTCCTGTGCATTCGGCTTAG 5'  p-value: 0.03
```

orange1.1t02254.1 gene=orange1.1t02254 CDS=544-1587

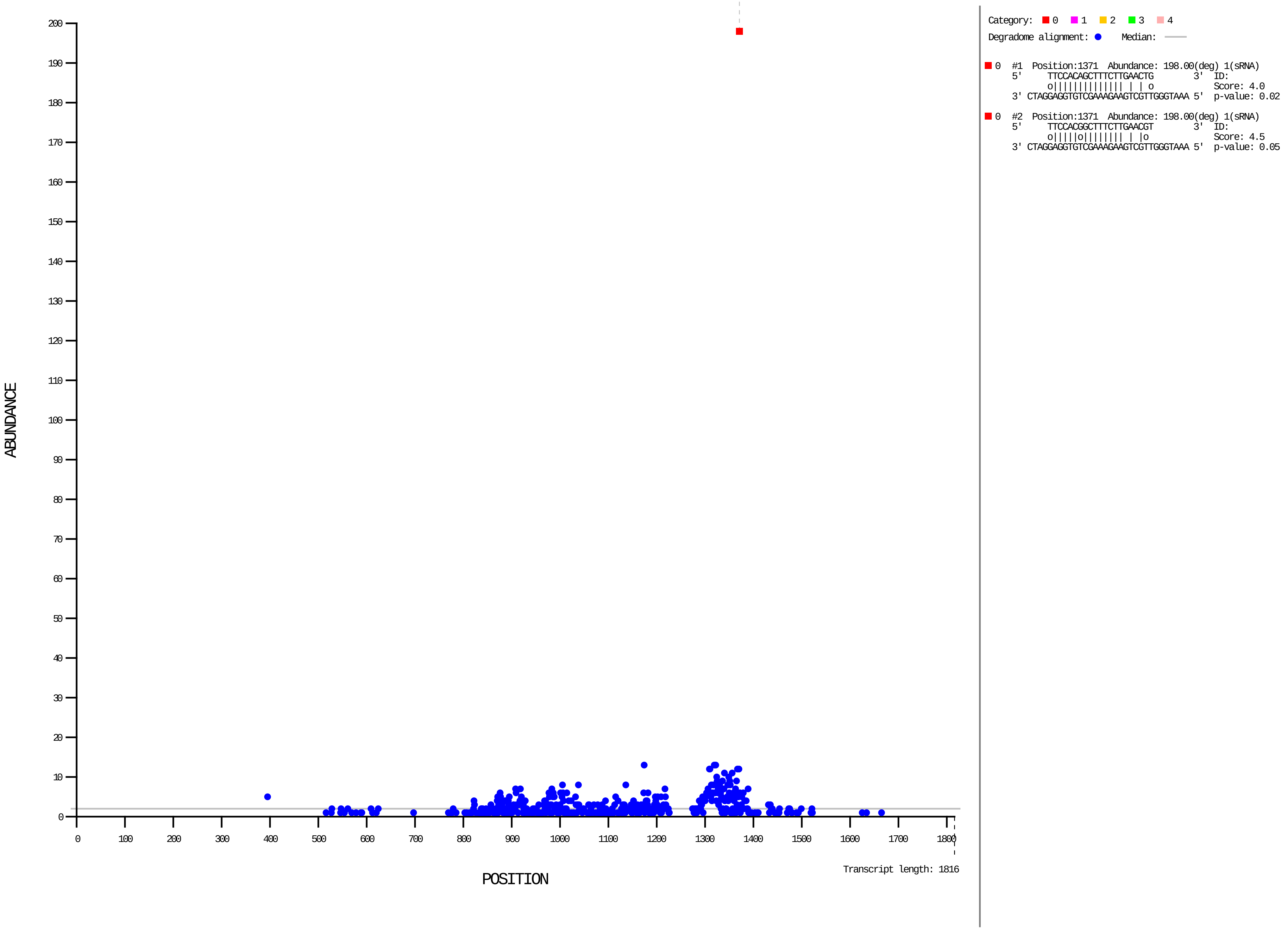

Cs9g01780.1 gene=Cs9g01780 CDS=99-254

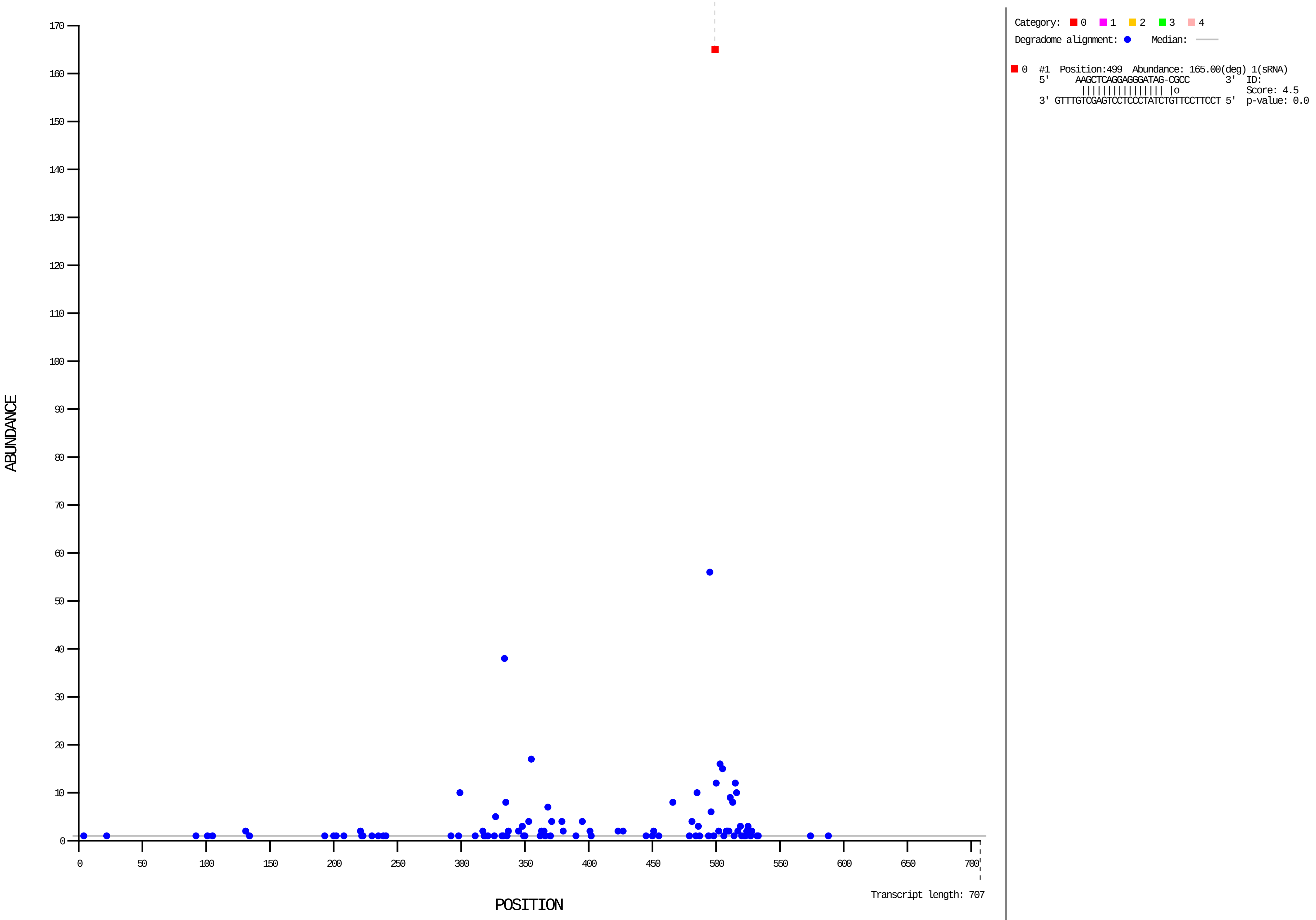

orange1.1t00850.1 gene=orange1.1t00850 CDS=1-3210

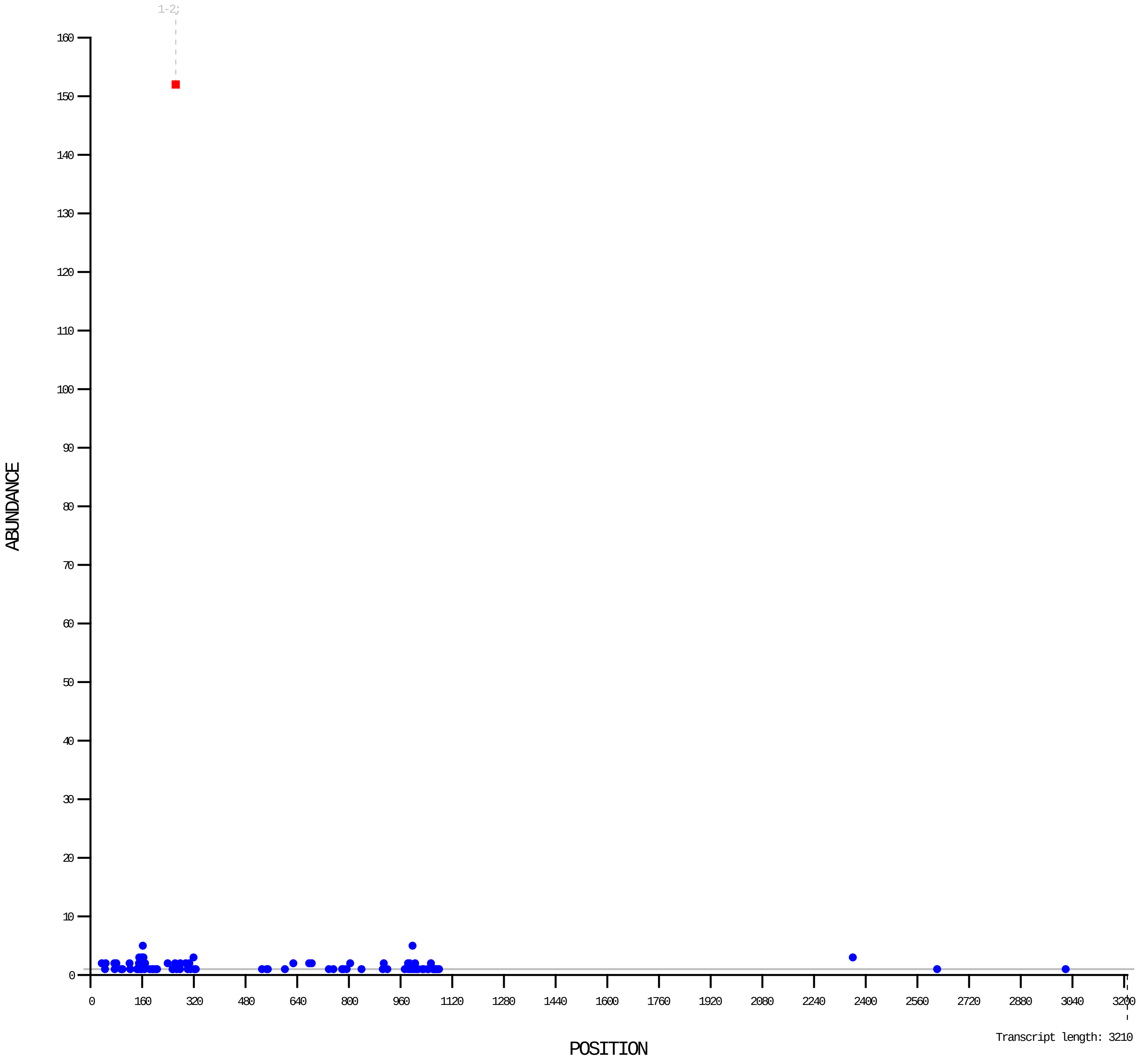

0 #1 Position:264 Abundance: 152.00(deg) 1(sRNA)

```
5'     TTCCACAGCTTTCTTGAACTG        3'  ID:
       | ||||||||||||| ||||             Score: 3.0
3' GAAAATGGTGTCGAAAGAAGTTGAAGACACTA 5'  p-value: 0.02
```

0 #2 Position:264 Abundance: 152.00(deg) 1(sRNA)

```
5'     TTCCACGGCTTTCTTGAACGT        3'  ID:
       | ||||o|||||||| ||| |            Score: 3.5
3' GAAAATGGTGTCGAAAGAAGTTGAAGACACTA 5'  p-value: 0.01
```

orange1.1t00199.1 gene=orange1.1t00199 CDS=501-2621

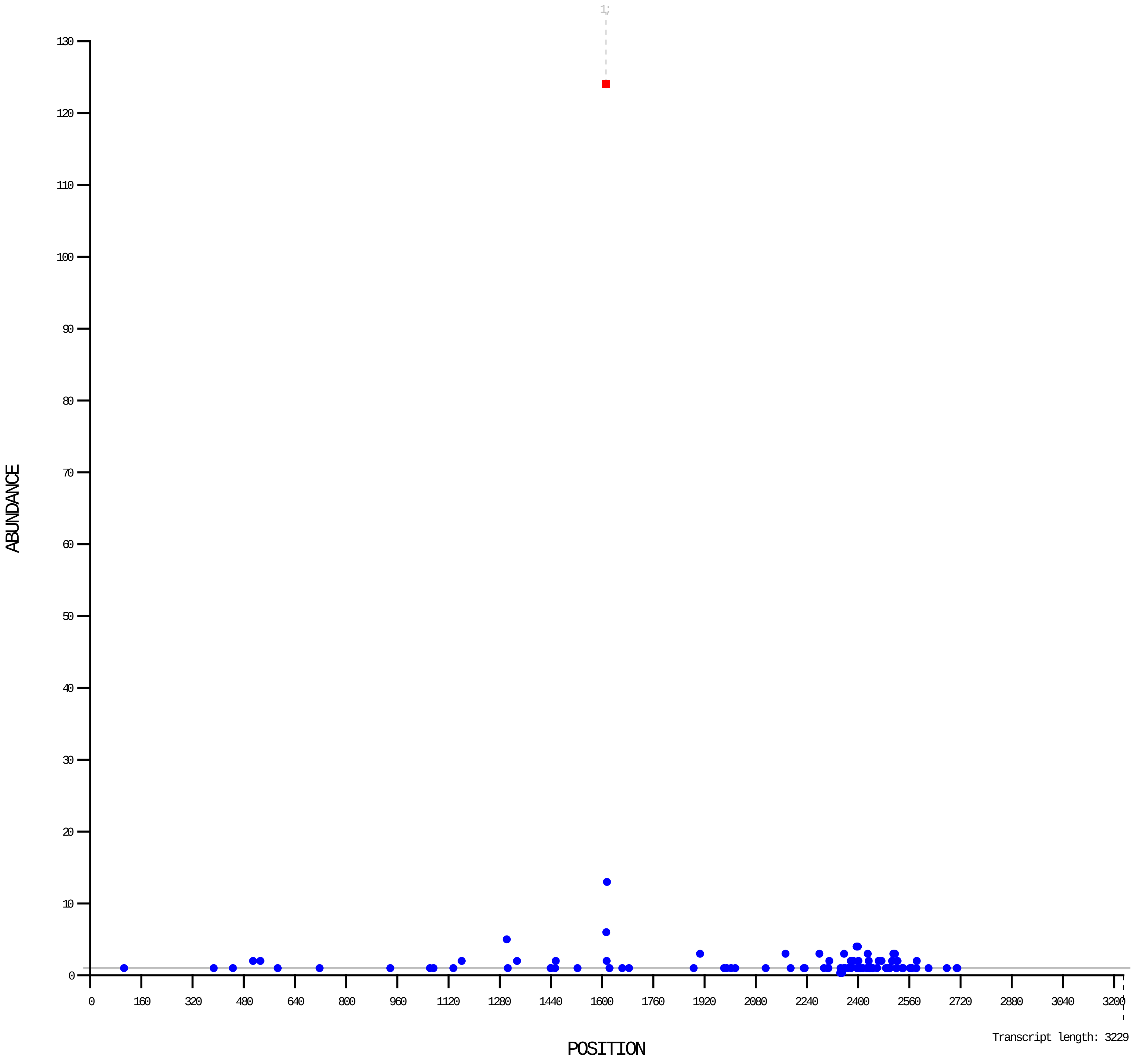

Category: 0 1 2 3 4
Degradome alignment: Median:

0 #1 Position:1612 Abundance: 124.00(deg) 1(sRNA)

```
5'     TTGAGCCGCGCCAATATCAC       3'  ID:
       ||||||||||||||||| |            Score: 1.0
3' CACTAACTCGGCGCGGTTATAGGGAACGCGCT 5'  p-value: 0.0
```

Cs2g10760.1 gene=Cs2g10760 CDS=227-3172

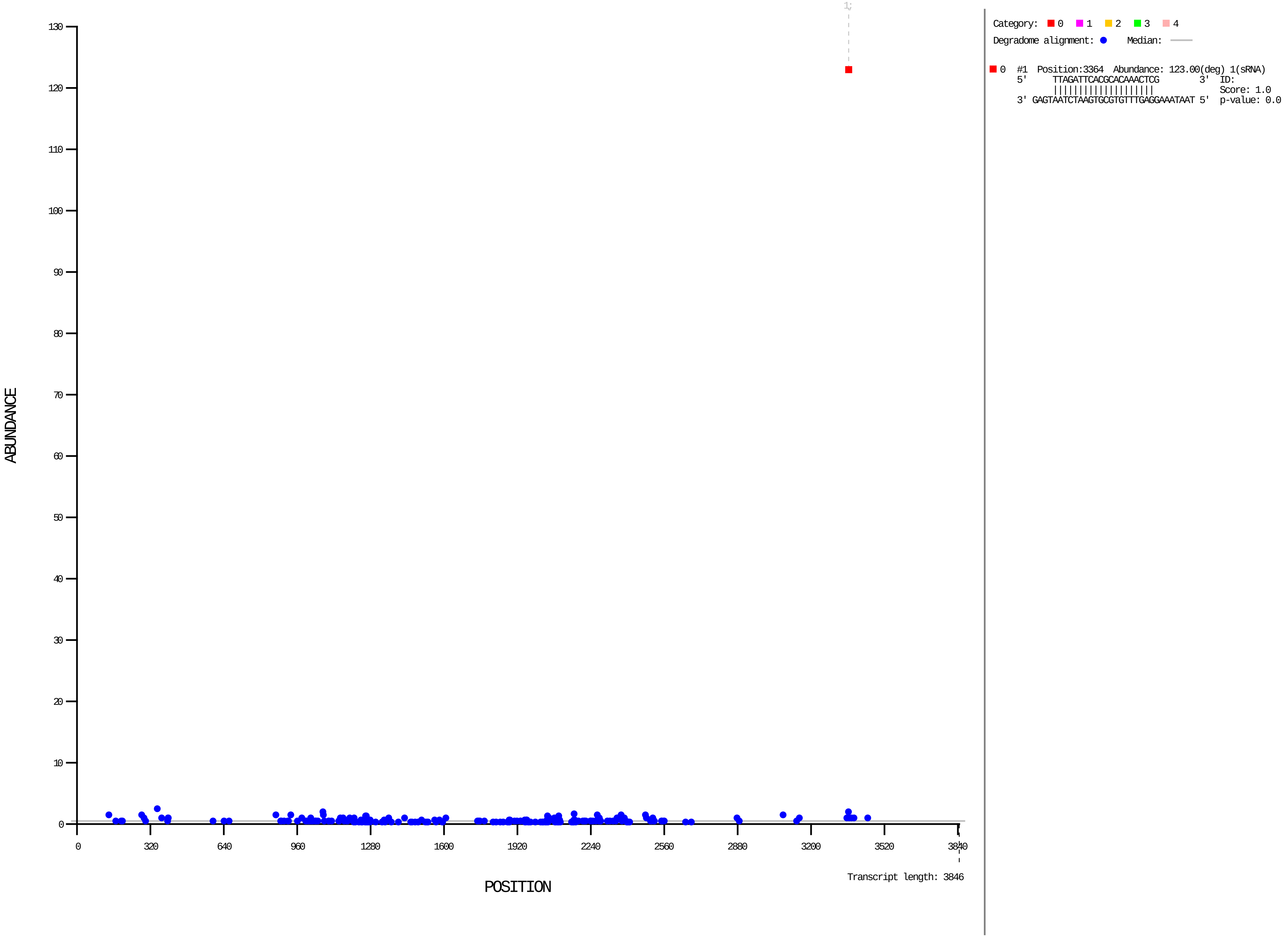

Cs7g10850.1 gene=Cs7g10850 CDS=1-1407

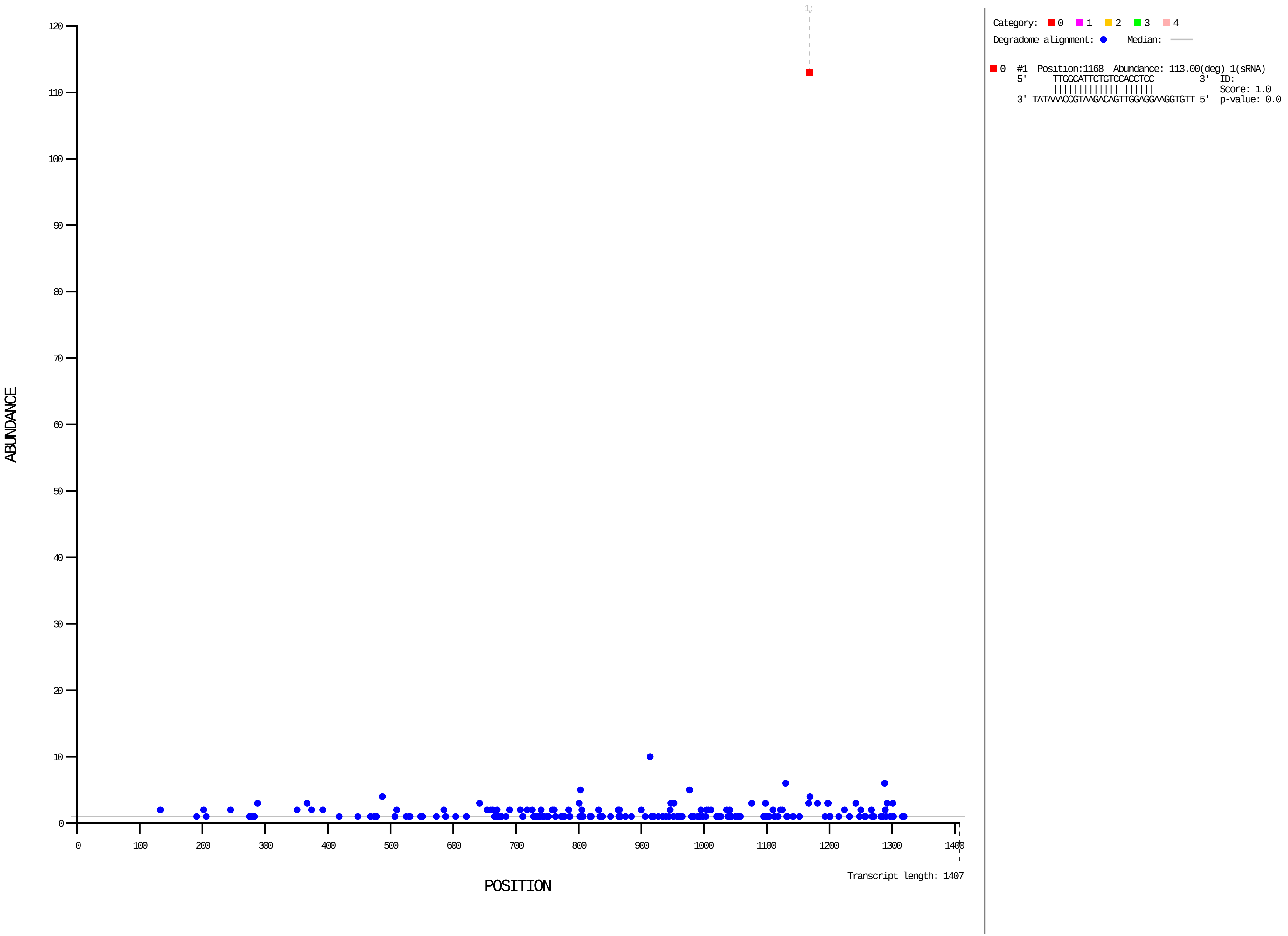

Cs8g17390.2 gene=Cs8g17390 CDS=494-1981

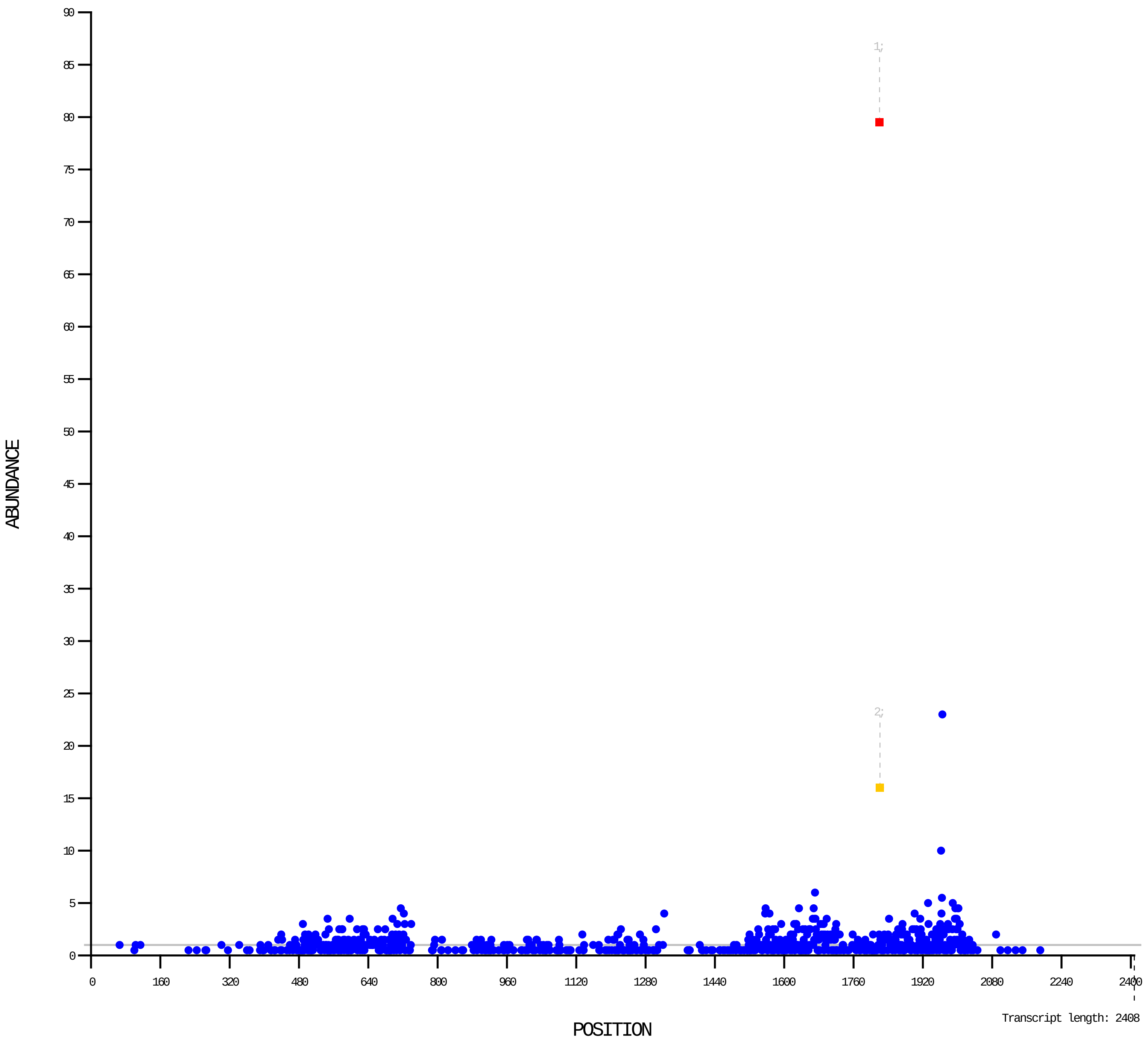

Category: 0 1 2 3 4
Degradome alignment: Median:

0 #1 Position:1820 Abundance: 79.50(deg) 1(sRNA)
5' AGAATCTTGATGATGCTGCA 3' ID:
|||||o||||||||||||| Score: 1.5
3' TTACCCTTAGGACTACTACGACGTGATCTATT 5' p-value: 0.0

2 #2 Position:1821 Abundance: 16.00(deg) 1(sRNA)
5' TGGAATCTTGATGATGCTGCAG 3' ID:
||||||o|||||||||||||| Score: 2.5
3' TTTACCCTTAGGACTACTACGACGTGATCTAT 5' p-value: 0.02

Cs8g17390.1 gene=Cs8g17390 CDS=494-2026

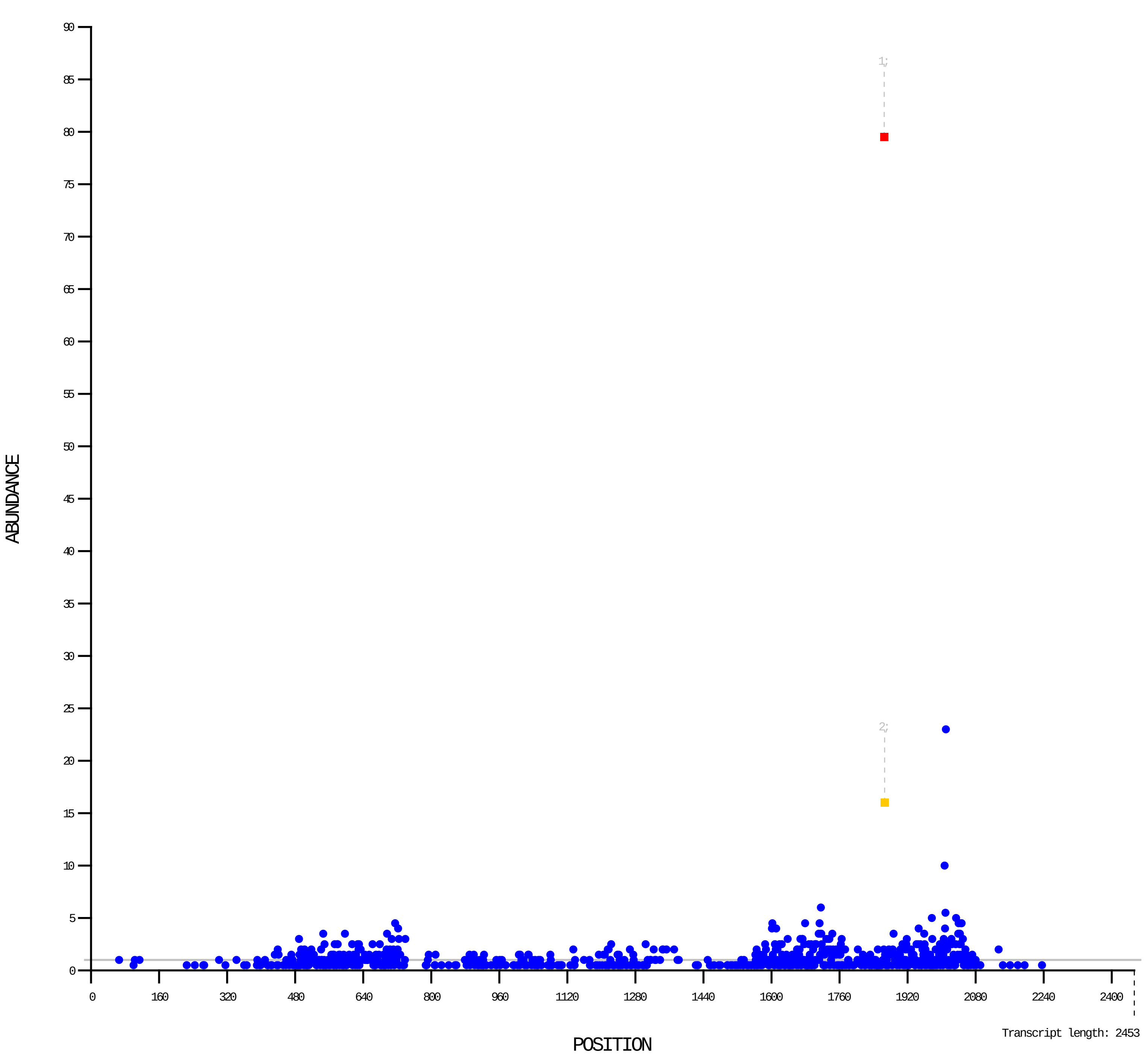


```
Category: ■0 ■1 ■2 ■3 ■4
Degradome alignment: ●    Median: —

■ 0  #1  Position:1865  Abundance: 79.50(deg) 1(sRNA)
     5'     AGAATCTTGATGATGCTGCA          3'  ID:
             |||||o||||||||||||||             Score: 1.5
     3' TTACCCTTAGGACTACTACGACGTGATCTATT 5'  p-value: 0.0

■ 2  #2  Position:1866  Abundance: 16.00(deg) 1(sRNA)
     5'     TGGAATCTTGATGATGCTGCAG        3'  ID:
             ||||||o|||||||||||||||           Score: 2.5
     3' TTTACCCTTAGGACTACTACGACGTGATCTAT 5'  p-value: 0.01
```

Cs9g06846.1 gene=Cs9g06846 CDS=857-1000

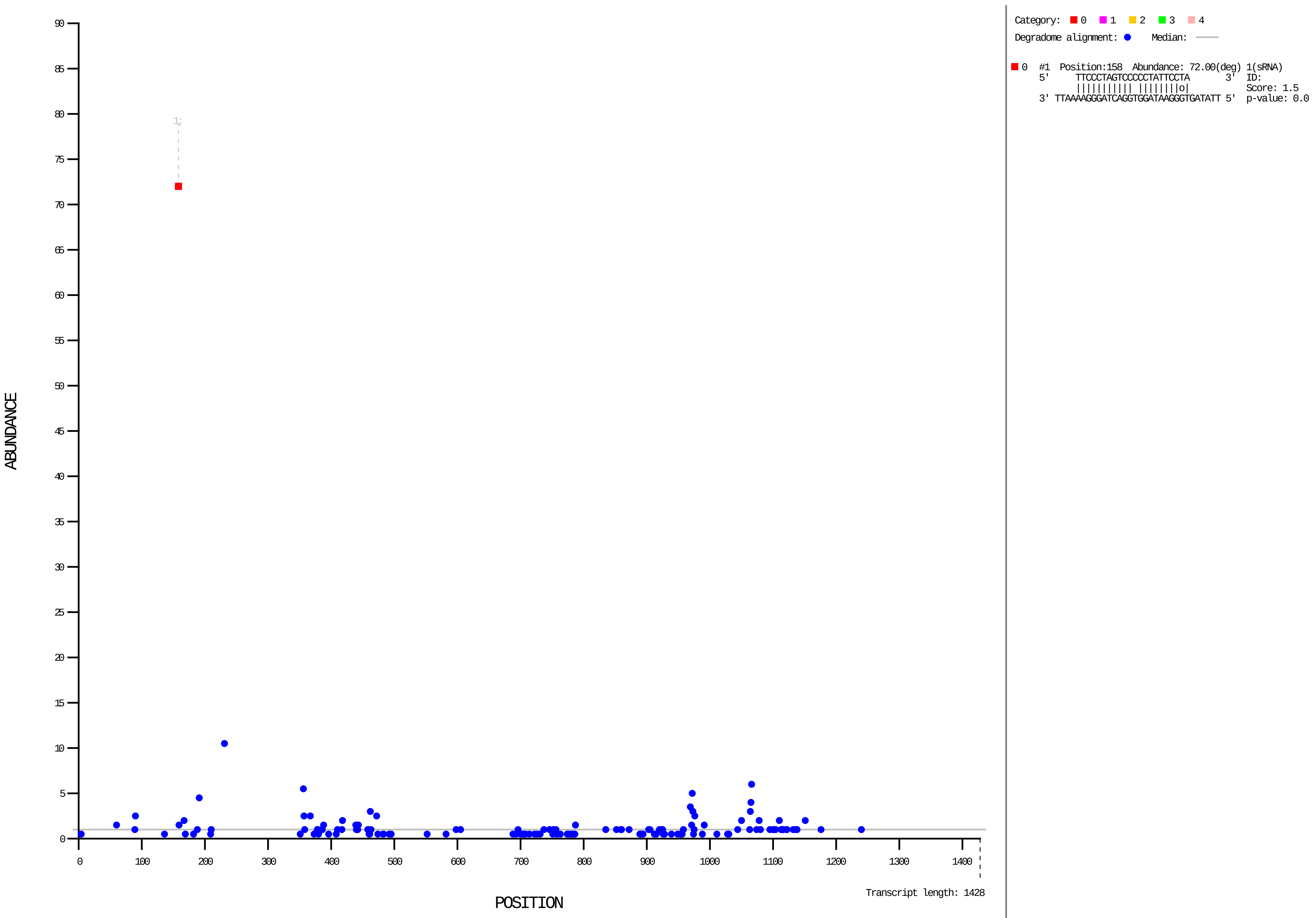

Cs9g06846.2 gene=Cs9g06846 CDS=857-1000

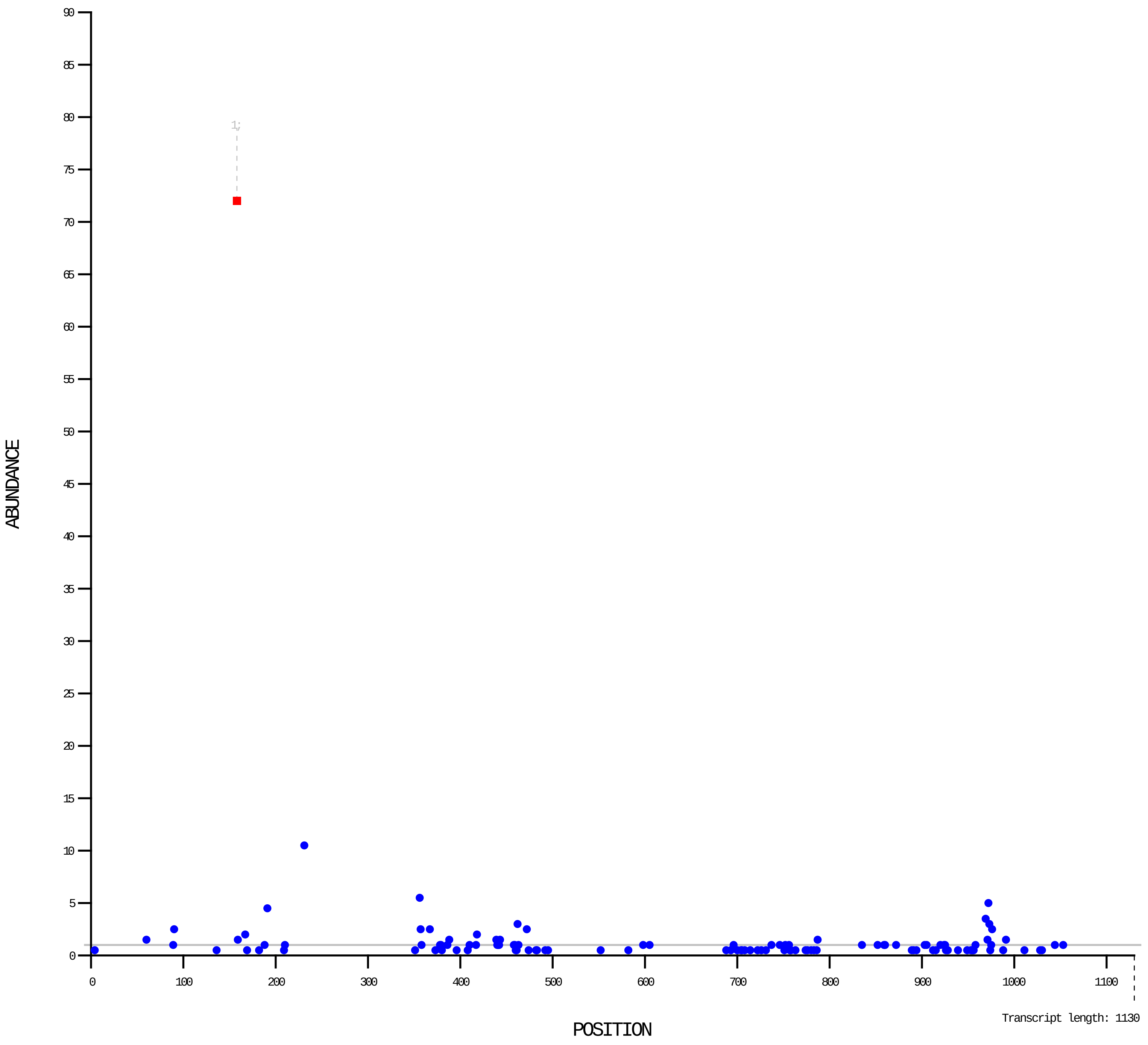

0 #1 Position:158 Abundance: 72.00(deg) 1(sRNA)

```
5'     TTCCCTAGTCCCCCTATTCCTA      3'  ID:
       |||||||||| ||||||||o|           Score: 1.5
3' TTAAAAGGGATCAGGTGGATAAGGGTGATATT 5'  p-value: 0.0
```

orange1.1t00200.2 gene=orange1.1t00200 CDS=412-2520

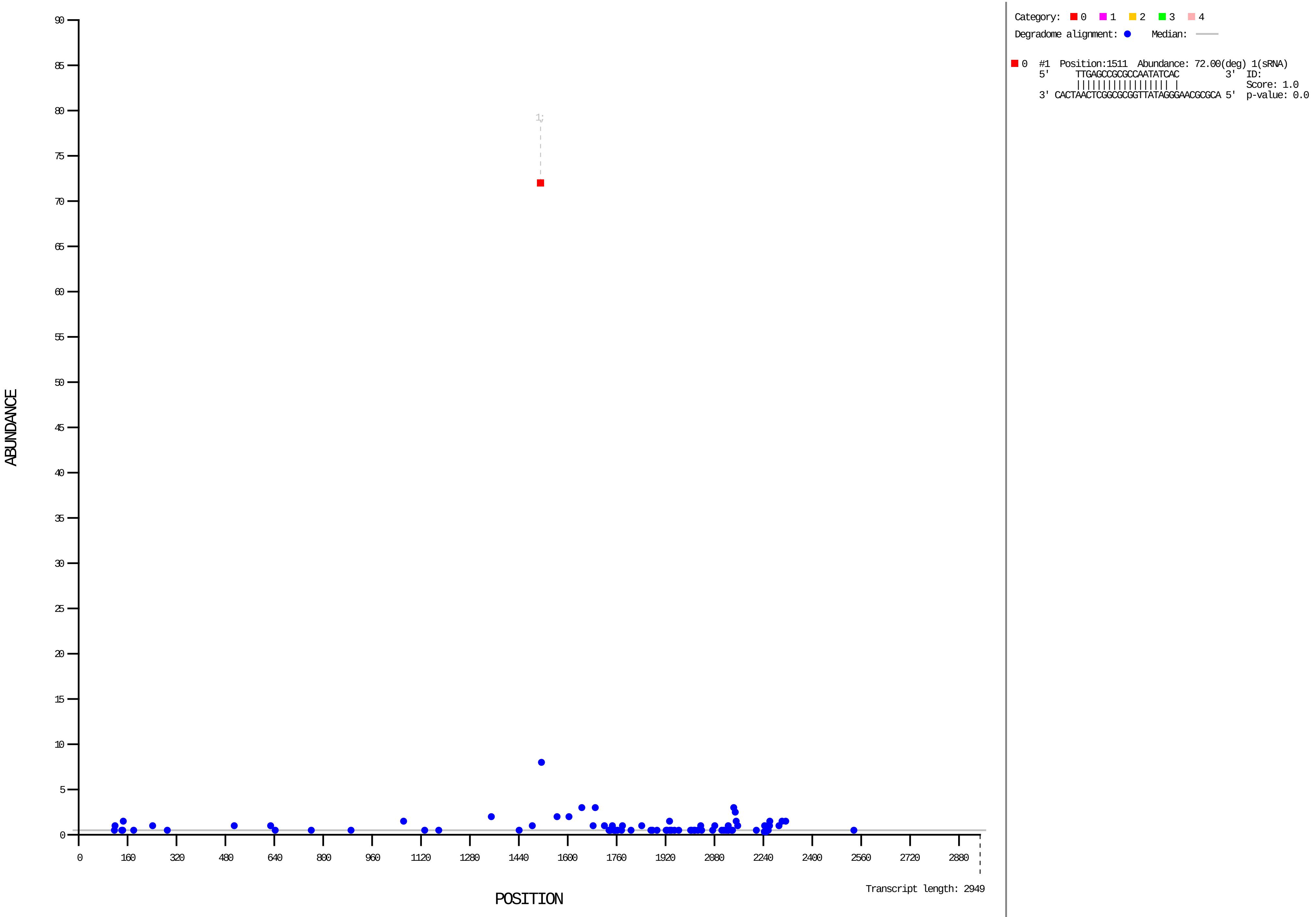

Cs3g12810.1 gene=Cs3g12810 CDS=1-3225

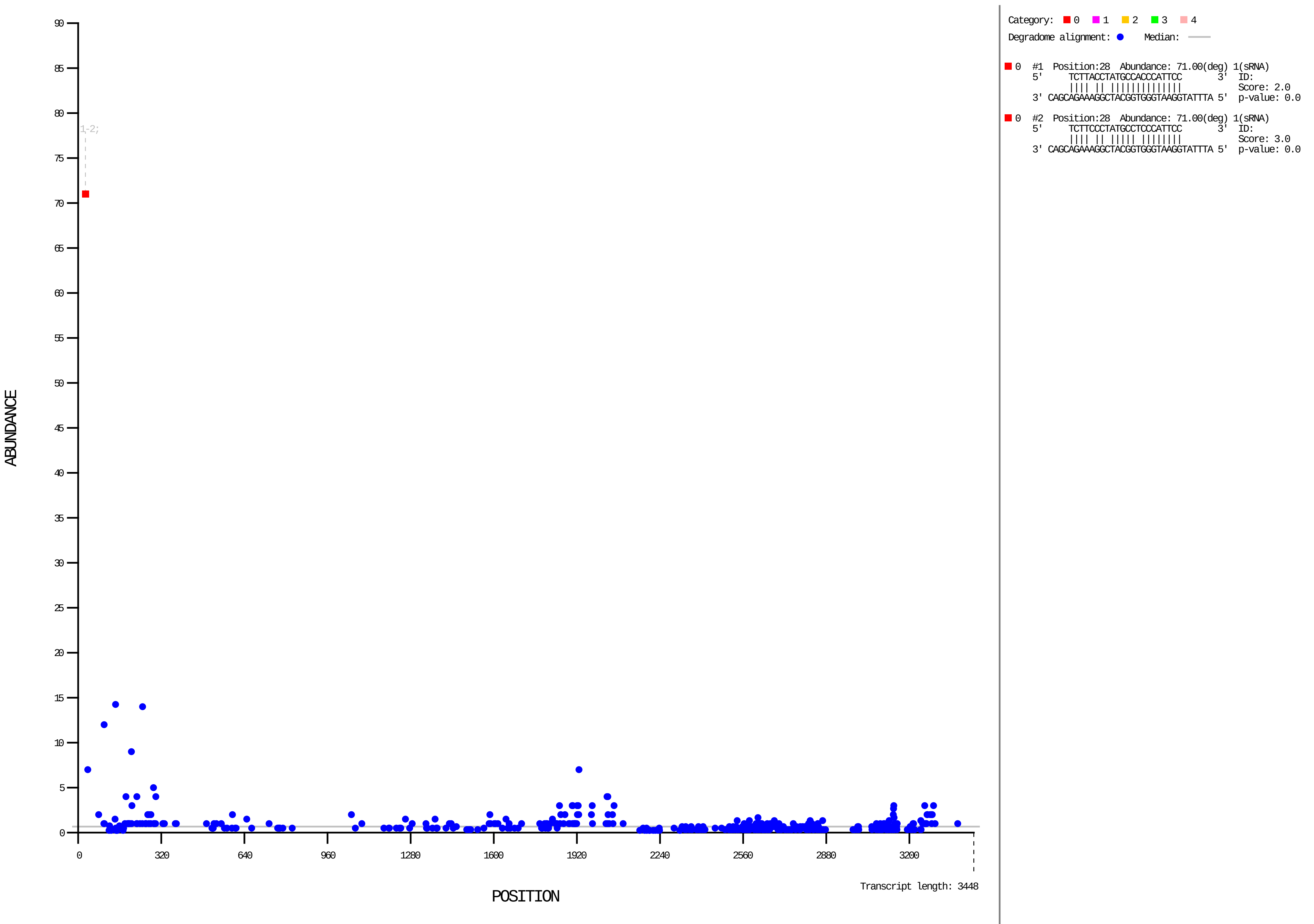

Cs6g07410.1 gene=Cs6g07410 CDS=96-1796

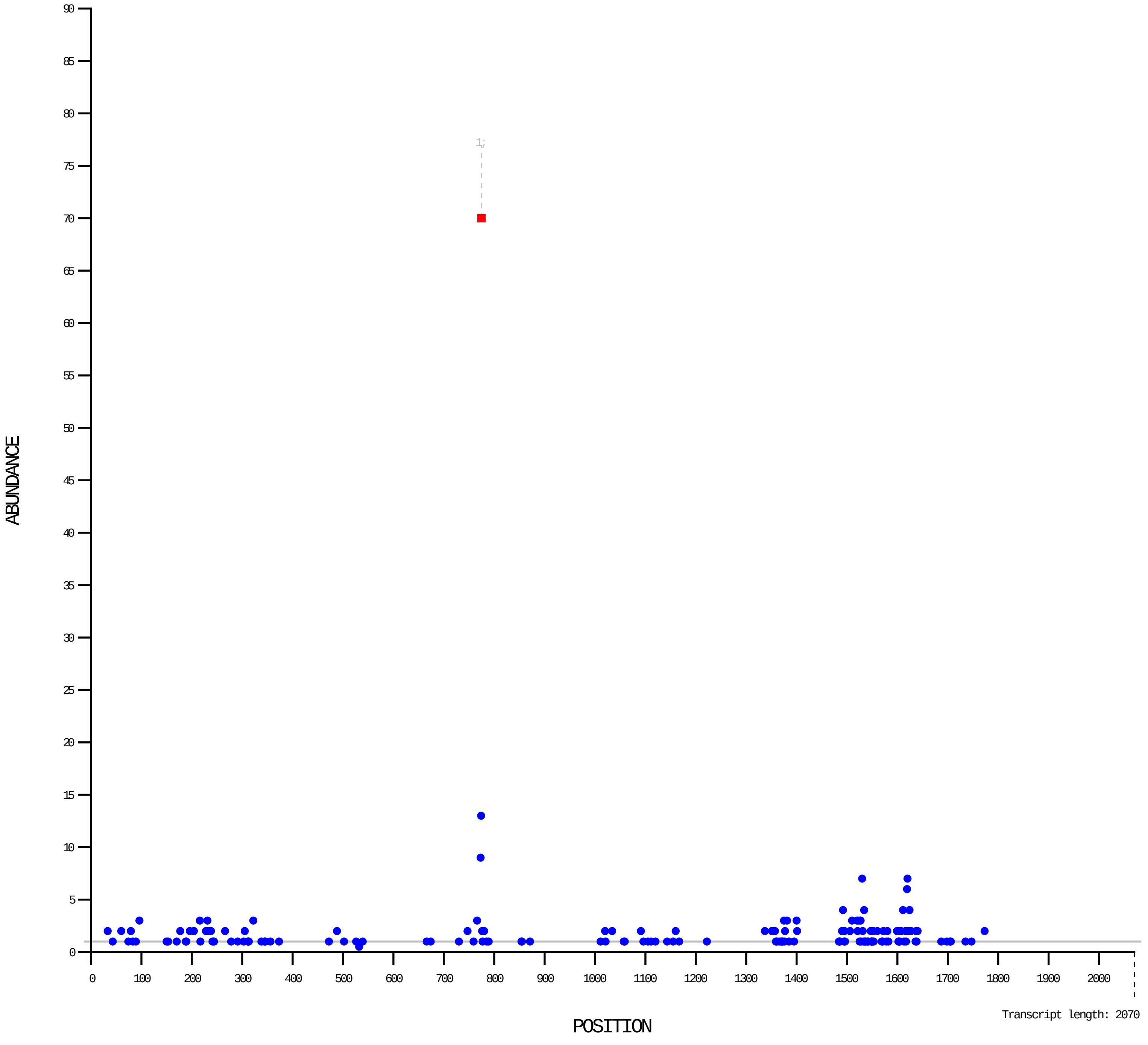

Category: 0 1 2 3 4
Degradome alignment: Median:

0 #1 Position:775 Abundance: 70.00(deg) 1(sRNA)
5' TCATTGAGTGCAGCGTTGATG 3' ID:
Score: 1.0
3' GACCAATAACTCACGTCGCAACTACTATGCAT 5' p-value: 0.0

Cs8g15520.1 gene=Cs8g15520 CDS=158-916

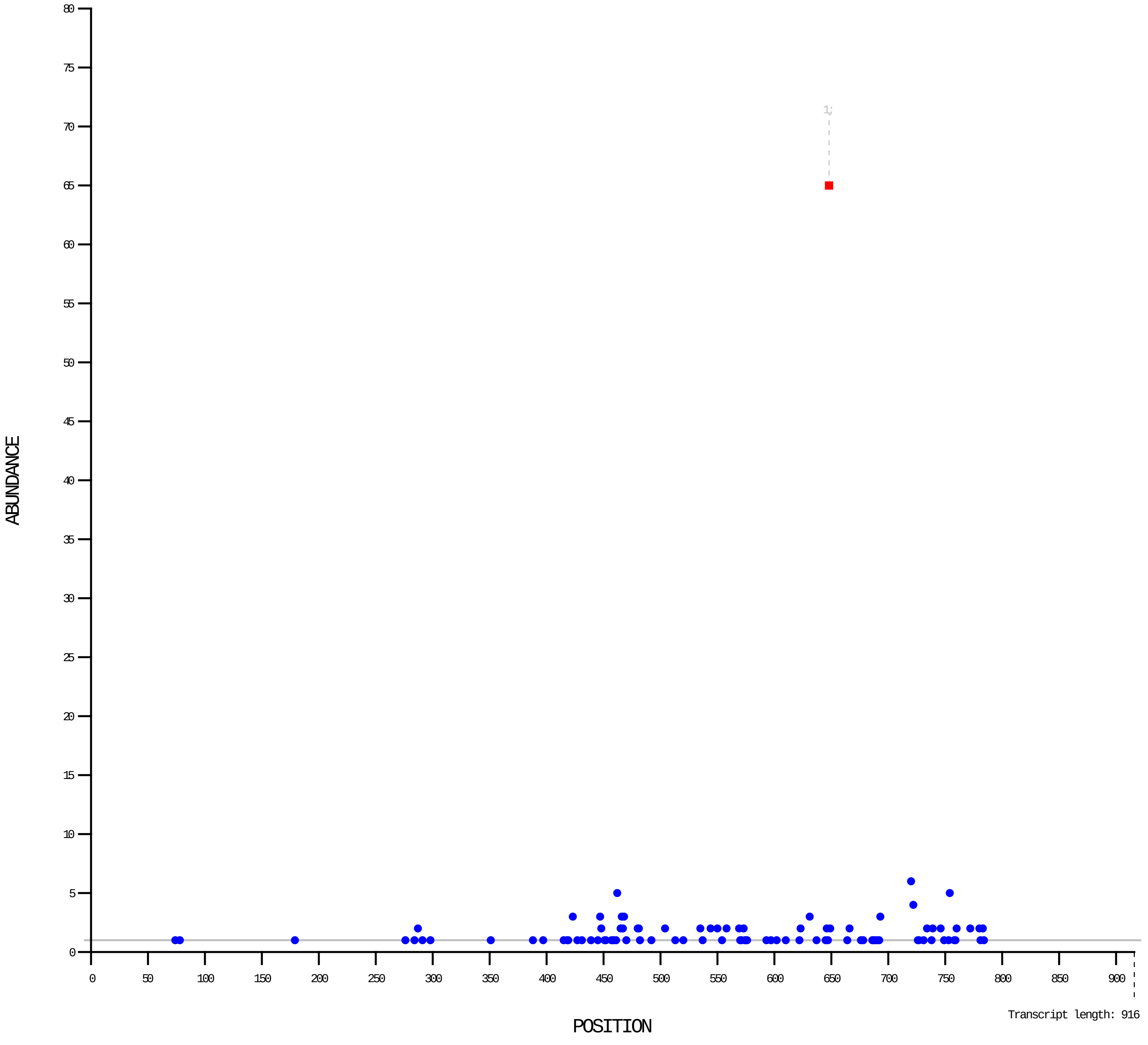


```
Category: ■ 0  ■ 1  ■ 2  ■ 3  ■ 4
Degradome alignment: ●     Median: ——

■ 0  #1  Position:648  Abundance: 65.00(deg) 1(sRNA)
     5'     TGTGTTCTCAGGTCACCCCTT        3'  ID:
            ||| |||o||||||||||| o            Score: 4.0
     3' TGATACA-AAGGGTCCAGTGGGCGCACAGCGT 5'  p-value: 0.01
```

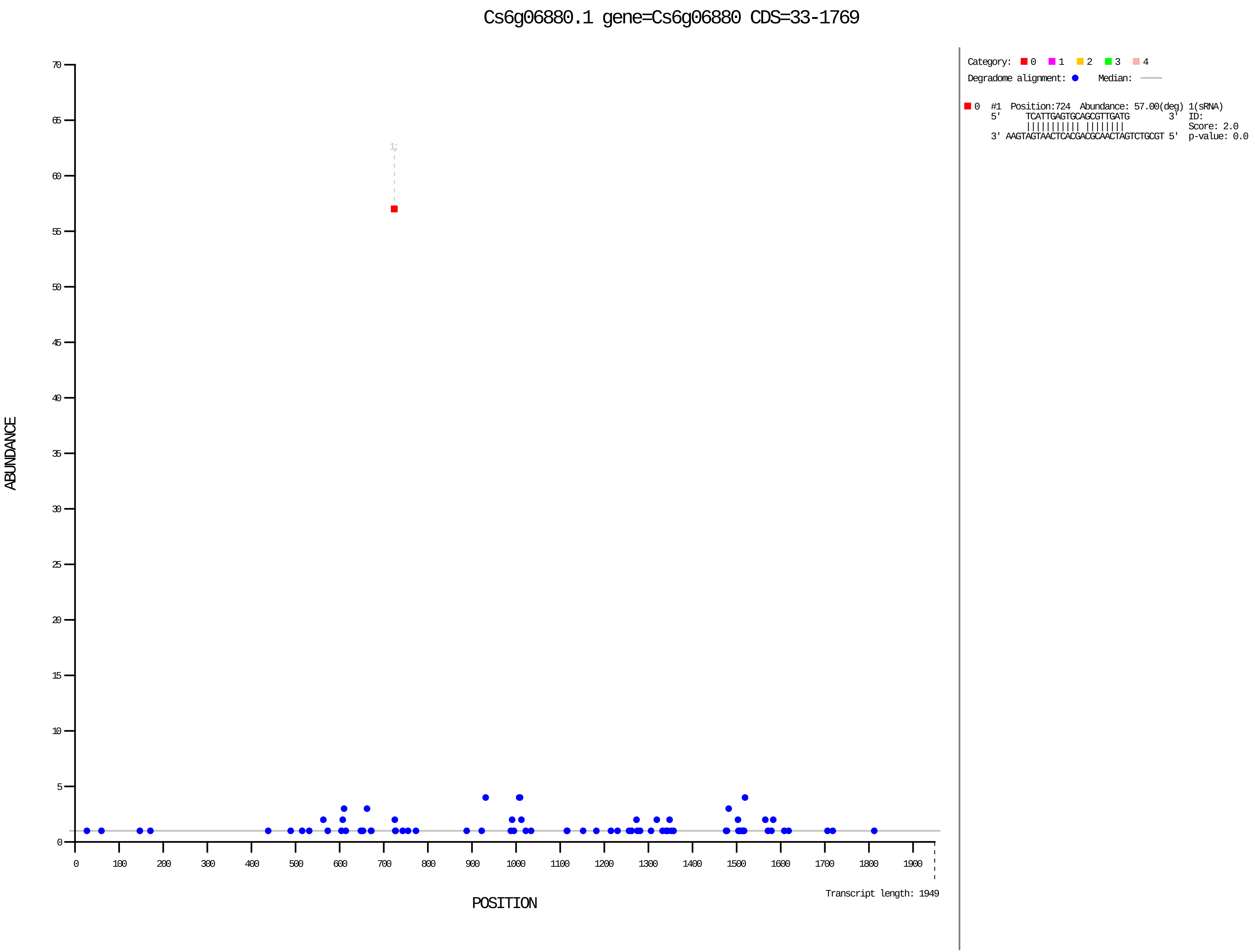
Cs6g06880.1 gene=Cs6g06880 CDS=33-1769
ABUNDANCE
POSITION
Transcript length: 1949
Category: 0 1 2 3 4
Degradome alignment:
Median:
0 #1 Position:724 Abundance: 57.00(deg) 1(sRNA)
5' TCATTGAGTGCAGCGTTGATG 3' ID:
Score: 2.0
3' AAGTAGTAACTCACGACGCAACTAGTCTGCGT 5' p-value: 0.0

Cs8g16510.3 gene=Cs8g16510 CDS=1500-3476

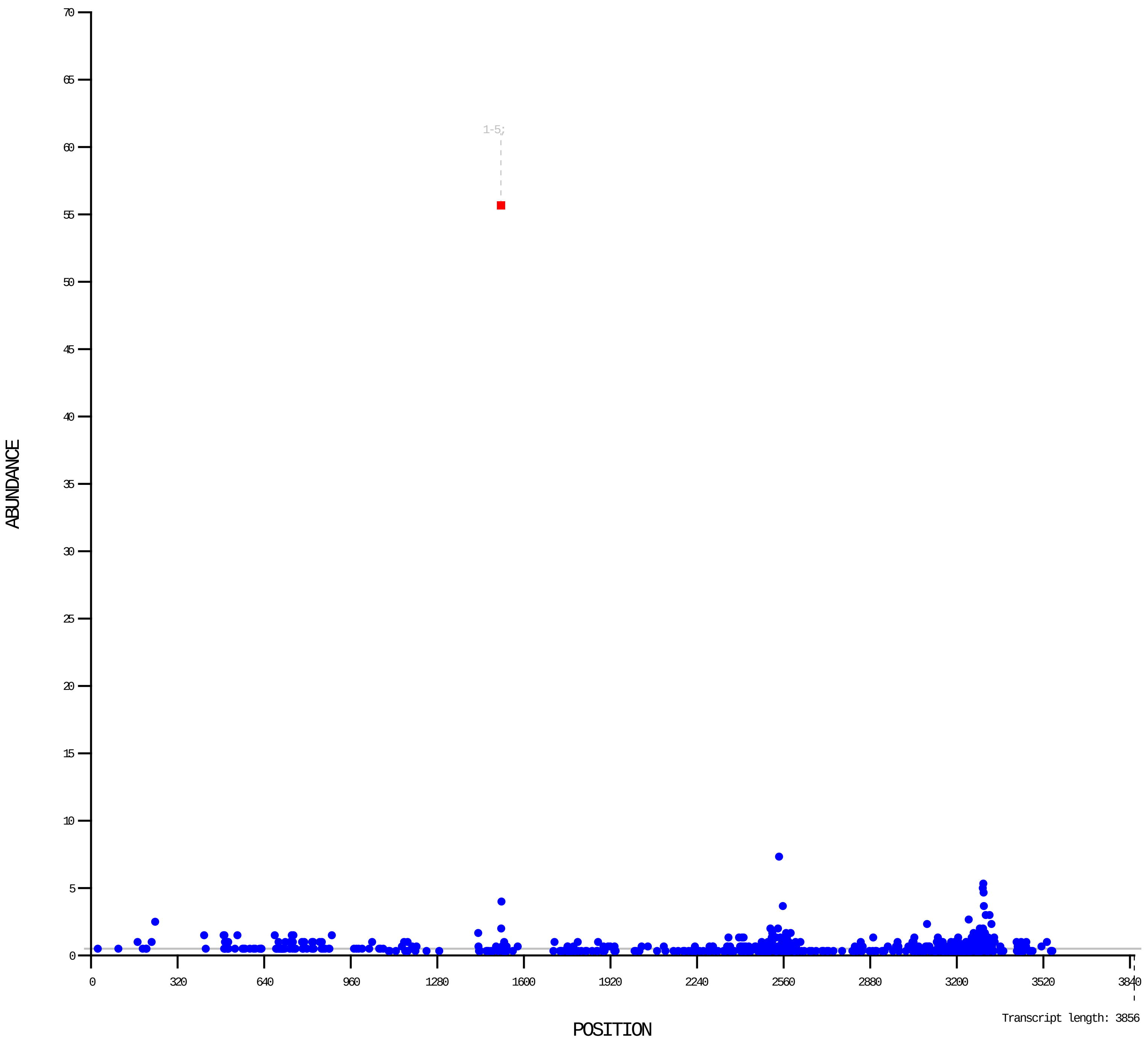

Category: 0 1 2 3 4
Degradome alignment: Median:

```
0  #1  Position:1515  Abundance: 55.67(deg) 1(sRNA)
   5'     TCGGACCAGGCTTCATTCCC        3'  ID:
          o|||||||||||||| |||             Score: 1.5
   3' CTTAGGCCTGGTCCGAAGTA-GGGTCCGTAGA 5'  p-value: 0.0

0  #2  Position:1515  Abundance: 55.67(deg) 1(sRNA)
   5'     TCGGACCAGGCTTCATTCCCT       3'  ID:
          o|||||||||||||| |||             Score: 2.5
   3' CTTAGGCCTGGTCCGAAGTA-GGGTCCGTAGA 5'  p-value: 0.0

0  #3  Position:1515  Abundance: 55.67(deg) 1(sRNA)
   5'     TCGGACCAGGCTTCATTCCCC       3'  ID:
          o|||||||||||||| |||             Score: 2.5
   3' CTTAGGCCTGGTCCGAAGTA-GGGTCCGTAGA 5'  p-value: 0.0

0  #4  Position:1515  Abundance: 55.67(deg) 1(sRNA)
   5'     TCGGACCAGGCTTCATTCCCGT      3'  ID:
          o|||||||||||||||o|| |           Score: 3.0
   3' CTTAGGCCTGGTCCGAAGTAGGGTCCGTAGAC 5'  p-value: 0.0

0  #5  Position:1515  Abundance: 55.67(deg) 1(sRNA)
   5'     TCGGACCAGGCTTCATTCCCCC      3'  ID:
          o|||||||||||||| |||             Score: 3.5
   3' CTTAGGCCTGGTCCGAAGTA-GGGTCCGTAGA 5'  p-value: 0.0
```

Cs8g16510.2 gene=Cs8g16510 CDS=184-2415

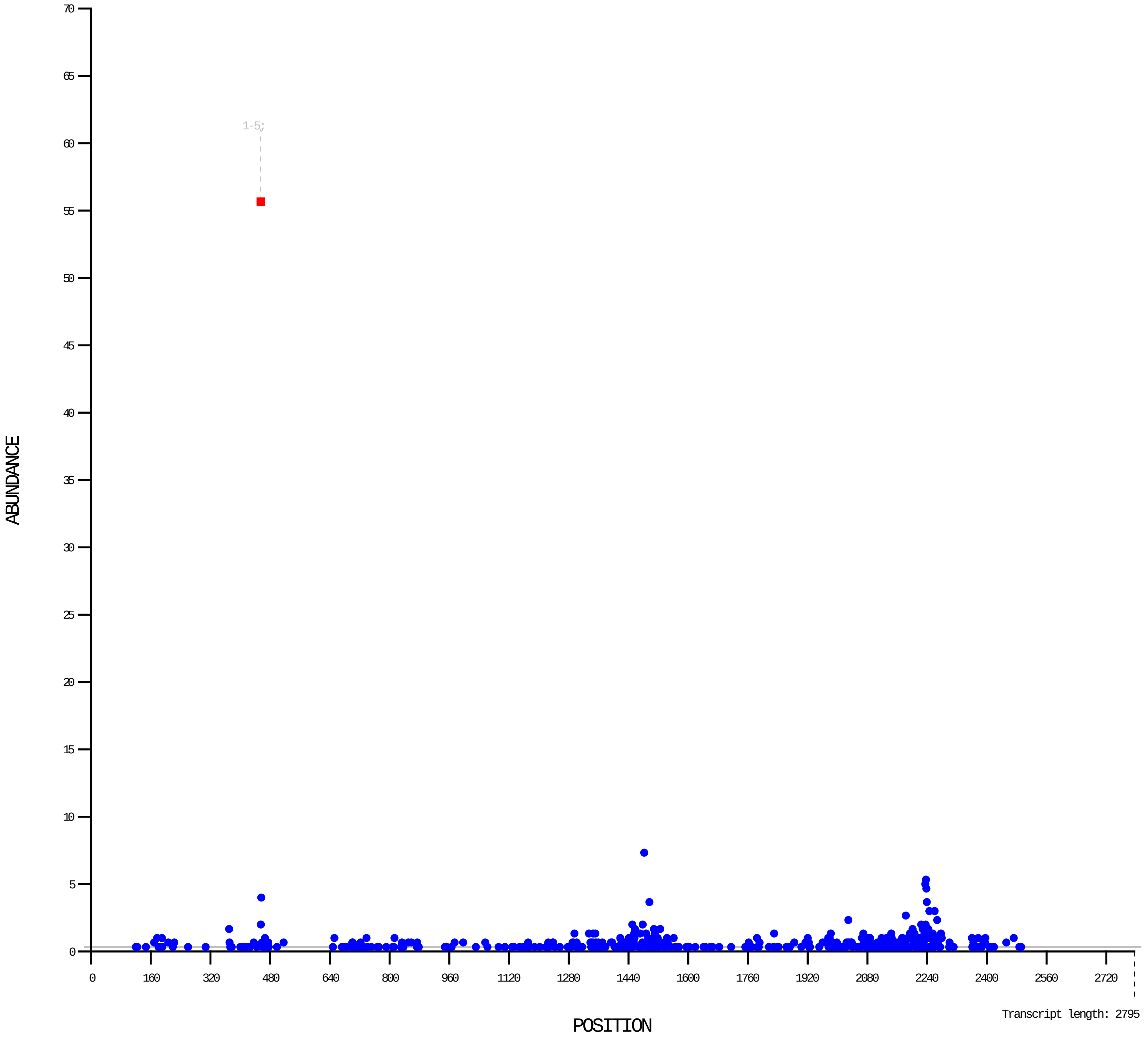

Category: 0 1 2 3 4
Degradome alignment: Median:

```
0  #1  Position:454  Abundance: 55.67(deg) 1(sRNA)
   5'      TCGGACCAGGCTTCATTCCC        3'  ID:
           o|||||||||||||||| |||            Score: 1.5
   3' CTTAGGCCTGGTCCGAAGTA-GGGTCCGTAGA 5'  p-value: 0.0

0  #2  Position:454  Abundance: 55.67(deg) 1(sRNA)
   5'      TCGGACCAGGCTTCATTCCCT       3'  ID:
           o|||||||||||||||| |||            Score: 2.5
   3' CTTAGGCCTGGTCCGAAGTA-GGGTCCGTAGA 5'  p-value: 0.0

0  #3  Position:454  Abundance: 55.67(deg) 1(sRNA)
   5'      TCGGACCAGGCTTCATTCCCC       3'  ID:
           o|||||||||||||||| |||            Score: 2.5
   3' CTTAGGCCTGGTCCGAAGTA-GGGTCCGTAGA 5'  p-value: 0.0

0  #4  Position:454  Abundance: 55.67(deg) 1(sRNA)
   5'      TCGGACCAGGCTTCATTCCCGT      3'  ID:
           o||||||||||||||||o|| |           Score: 3.0
   3' CTTAGGCCTGGTCCGAAGTAGGGTCCGTAGAC 5'  p-value: 0.0

0  #5  Position:454  Abundance: 55.67(deg) 1(sRNA)
   5'      TCGGACCAGGCTTCATTCCCCC      3'  ID:
           o|||||||||||||||| |||            Score: 3.5
   3' CTTAGGCCTGGTCCGAAGTA-GGGTCCGTAGA 5'  p-value: 0.0
```

Cs8g16510.1 gene=Cs8g16510 CDS=855-3395

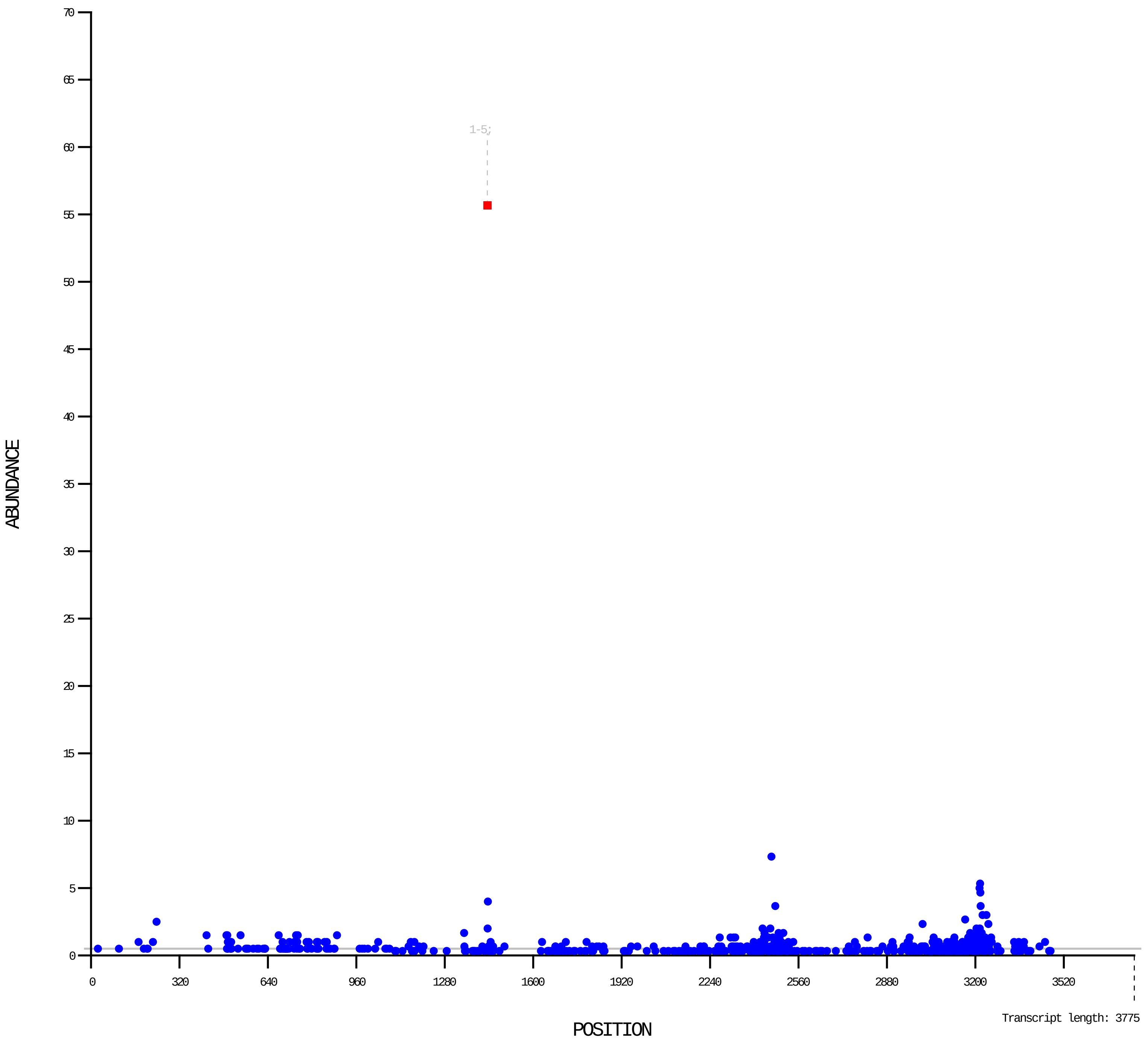

Category: 0 1 2 3 4
Degradome alignment: ● Median:

```
0  #1  Position:1434  Abundance: 55.67(deg) 1(sRNA)
   5'     TCGGACCAGGCTTCATTCCC        3'  ID:
          o||||||||||||||| |||            Score: 1.5
   3' CTTAGGCCTGGTCCGAAGTA-GGGTCCGTAGA 5'  p-value: 0.0

0  #2  Position:1434  Abundance: 55.67(deg) 1(sRNA)
   5'     TCGGACCAGGCTTCATTCCCT       3'  ID:
          o||||||||||||||| |||            Score: 2.5
   3' CTTAGGCCTGGTCCGAAGTA-GGGTCCGTAGA 5'  p-value: 0.0

0  #3  Position:1434  Abundance: 55.67(deg) 1(sRNA)
   5'     TCGGACCAGGCTTCATTCCCC       3'  ID:
          o||||||||||||||| |||            Score: 2.5
   3' CTTAGGCCTGGTCCGAAGTA-GGGTCCGTAGA 5'  p-value: 0.0

0  #4  Position:1434  Abundance: 55.67(deg) 1(sRNA)
   5'     TCGGACCAGGCTTCATTCCCGT      3'  ID:
          o|||||||||||||||o|| |           Score: 3.0
   3' CTTAGGCCTGGTCCGAAGTAGGGTCCGTAGAC 5'  p-value: 0.0

0  #5  Position:1434  Abundance: 55.67(deg) 1(sRNA)
   5'     TCGGACCAGGCTTCATTCCCCC      3'  ID:
          o||||||||||||||| |||            Score: 3.5
   3' CTTAGGCCTGGTCCGAAGTA-GGGTCCGTAGA 5'  p-value: 0.0
```

Cs1g15640.3 gene=Cs1g15640 CDS=1130-3646

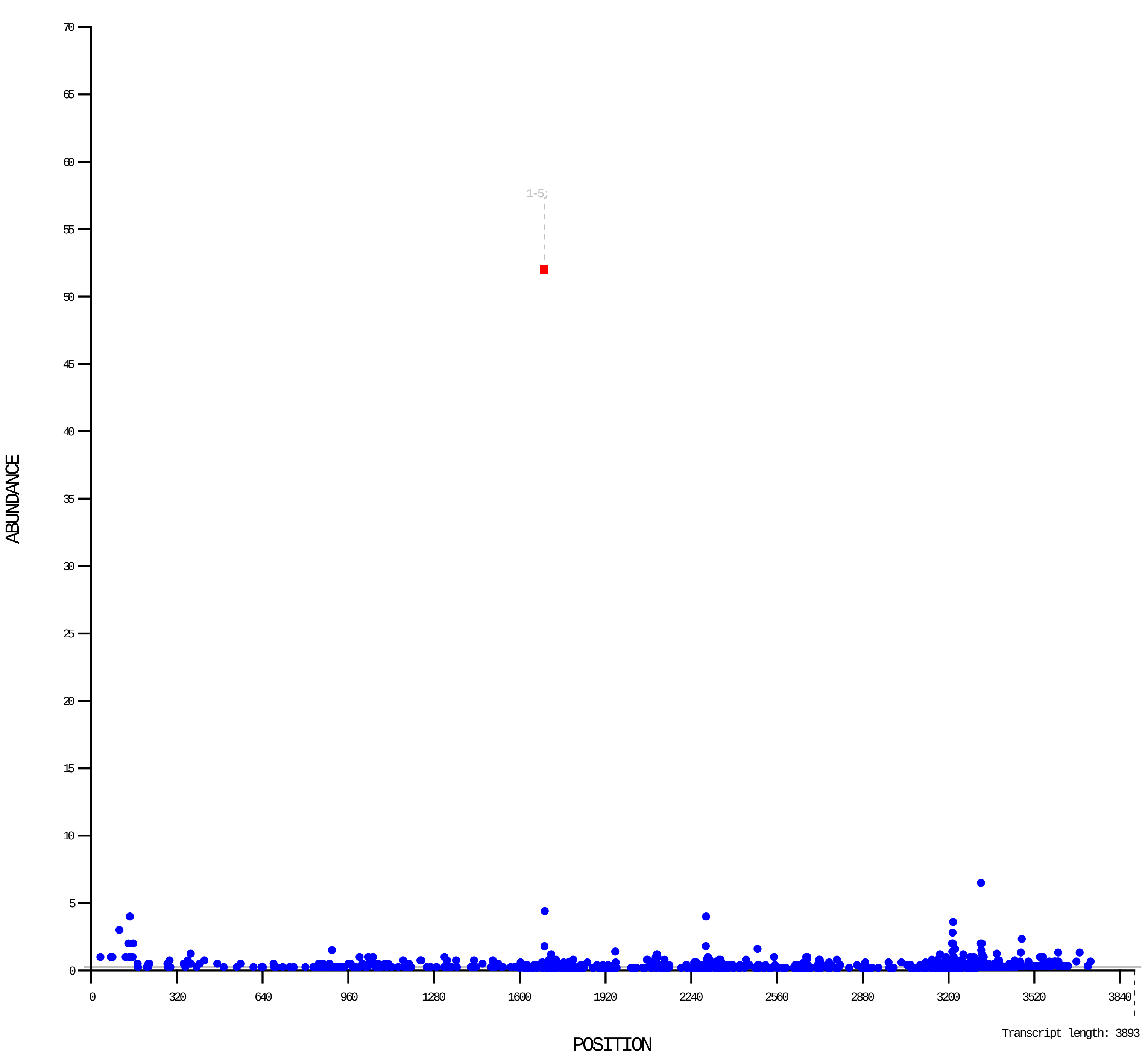

0 #1 Position:1691 Abundance: 52.00(deg) 1(sRNA)
```
5'     TCGGACCAGGCTTCATTCCC         3'  ID:
       o|||||||||||||||||||             Score: 1.5
3' CTTAGGCCTGGTCCGAAGTAAGGTCCGTAAAC 5'  p-value: 0.0
```

0 #2 Position:1691 Abundance: 52.00(deg) 1(sRNA)
```
5'     TCGGACCAGGCTTCATTCCCT        3'  ID:
       o|||||||||||||||||||             Score: 2.5
3' CTTAGGCCTGGTCCGAAGTAAGGTCCGTAAAC 5'  p-value: 0.01
```

0 #3 Position:1691 Abundance: 52.00(deg) 1(sRNA)
```
5'     TCGGACCAGGCTTCATTCCCGT       3'  ID:
       o||||||||||||||||||| |           Score: 2.5
3' CTTAGGCCTGGTCCGAAGTAAGGTCCGTAAAC 5'  p-value: 0.0
```

0 #4 Position:1691 Abundance: 52.00(deg) 1(sRNA)
```
5'     TCGGACCAGGCTTCATTCCCC        3'  ID:
       o|||||||||||||||||||             Score: 2.5
3' CTTAGGCCTGGTCCGAAGTAAGGTCCGTAAAC 5'  p-value: 0.0
```

0 #5 Position:1691 Abundance: 52.00(deg) 1(sRNA)
```
5'     TCGGACCAGGCTTCATTCCCCC       3'  ID:
       o|||||||||||||||||||             Score: 3.5
3' CTTAGGCCTGGTCCGAAGTAAGGTCCGTAAAC 5'  p-value: 0.0
```

Cs1g15640.1 gene=Cs1g15640 CDS=1220-3736

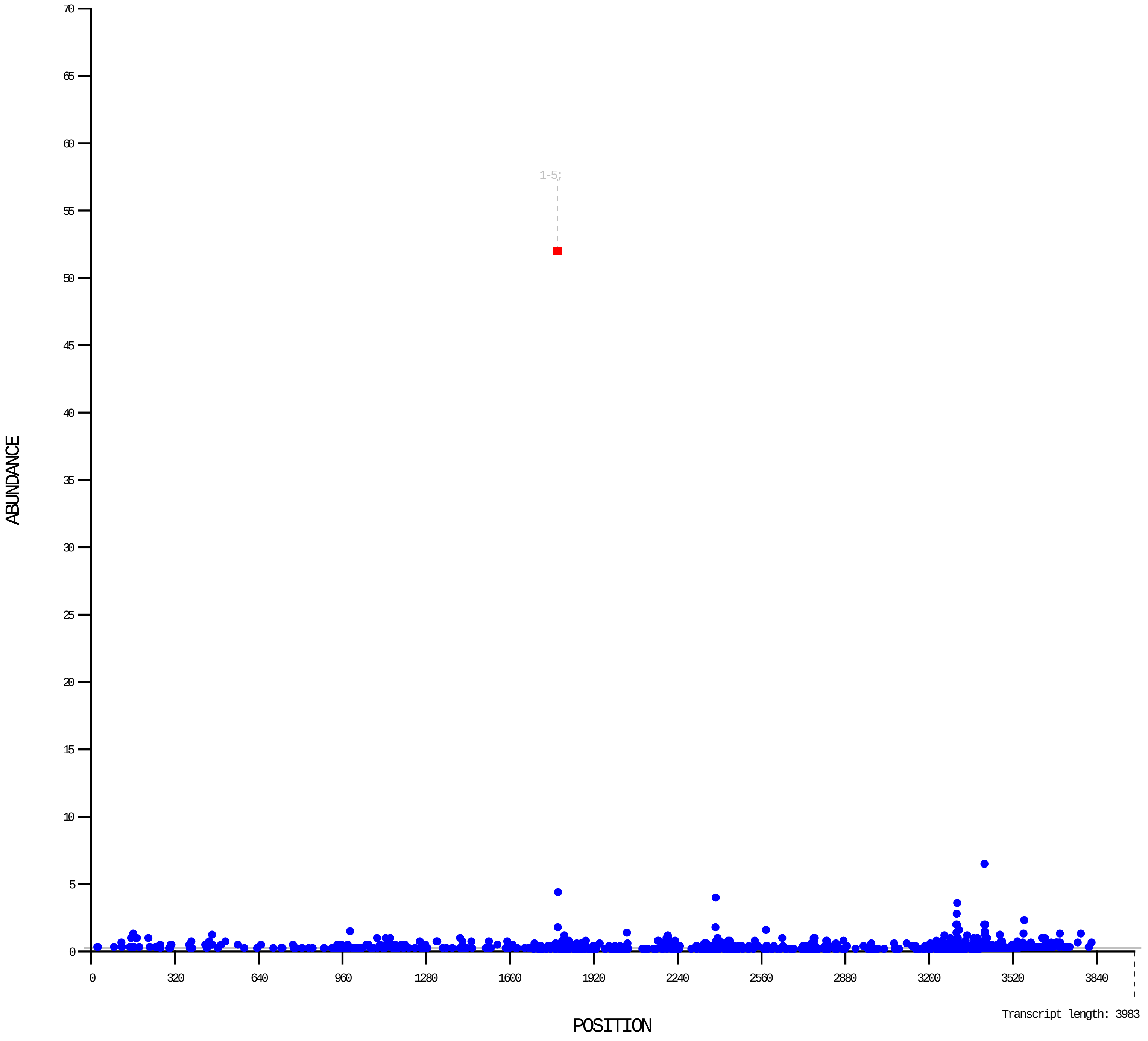

Category: 0 1 2 3 4
Degradome alignment: Median:

0 #1 Position:1781 Abundance: 52.00(deg) 1(sRNA)
```
5'     TCGGACCAGGCTTCATTCCC         3'  ID:
       o|||||||||||||||||||             Score: 1.5
3' CTTAGGCCTGGTCCGAAGTAAGGTCCGTAAAC 5'  p-value: 0.0
```

0 #2 Position:1781 Abundance: 52.00(deg) 1(sRNA)
```
5'     TCGGACCAGGCTTCATTCCCT        3'  ID:
       o|||||||||||||||||||             Score: 2.5
3' CTTAGGCCTGGTCCGAAGTAAGGTCCGTAAAC 5'  p-value: 0.0
```

0 #3 Position:1781 Abundance: 52.00(deg) 1(sRNA)
```
5'     TCGGACCAGGCTTCATTCCCGT       3'  ID:
       o||||||||||||||||||| |           Score: 2.5
3' CTTAGGCCTGGTCCGAAGTAAGGTCCGTAAAC 5'  p-value: 0.0
```

0 #4 Position:1781 Abundance: 52.00(deg) 1(sRNA)
```
5'     TCGGACCAGGCTTCATTCCCC        3'  ID:
       o|||||||||||||||||||             Score: 2.5
3' CTTAGGCCTGGTCCGAAGTAAGGTCCGTAAAC 5'  p-value: 0.0
```

0 #5 Position:1781 Abundance: 52.00(deg) 1(sRNA)
```
5'     TCGGACCAGGCTTCATTCCCCC       3'  ID:
       o|||||||||||||||||||             Score: 3.5
3' CTTAGGCCTGGTCCGAAGTAAGGTCCGTAAAC 5'  p-value: 0.0
```

Cs1g15640.4 gene=Cs1g15640 CDS=1220-3505

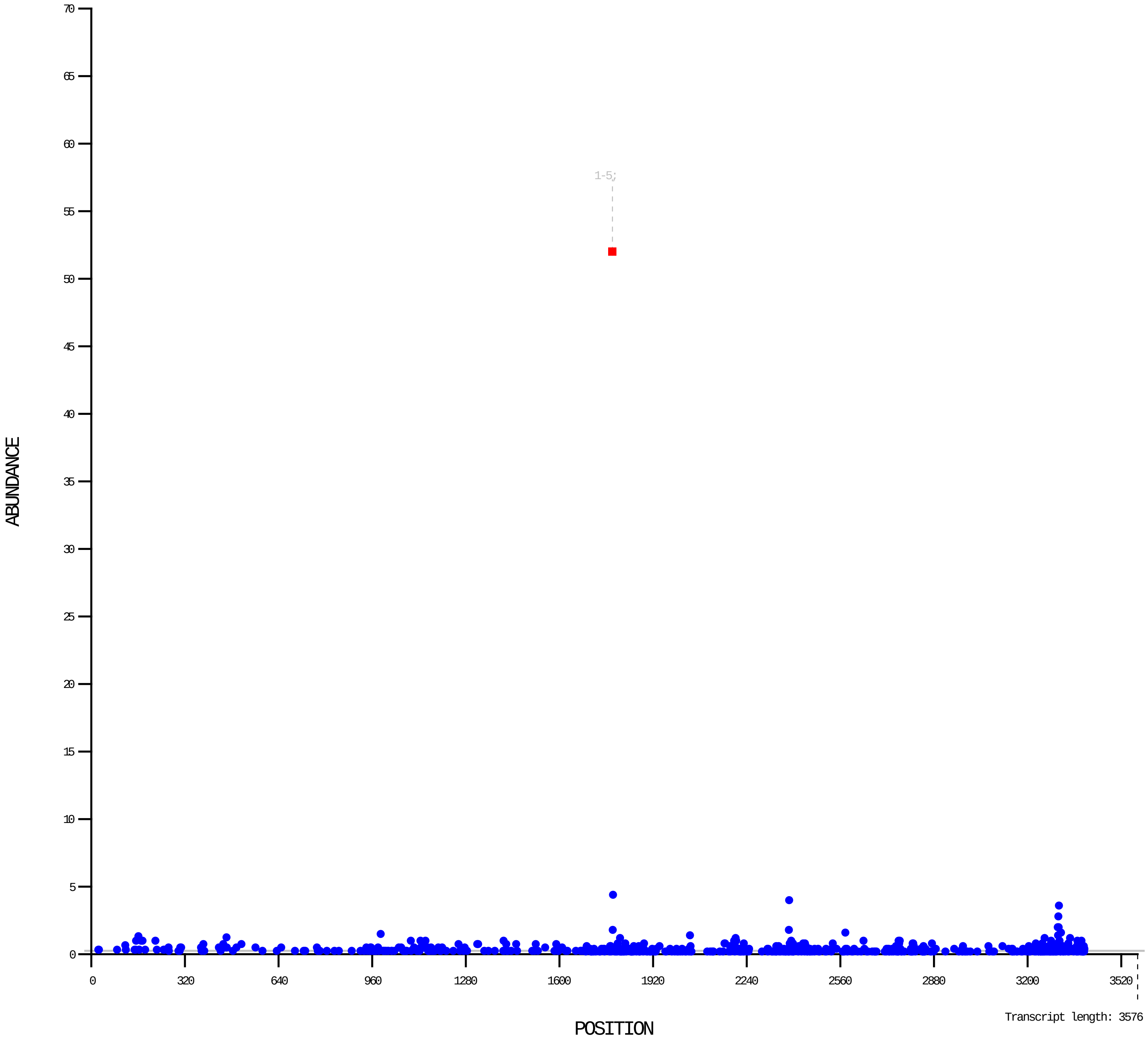

Category: 0 1 2 3 4
Degradome alignment: Median:

```
0  #1  Position:1781  Abundance: 52.00(deg) 1(sRNA)
   5'      TCGGACCAGGCTTCATTCCC          3'  ID:
           o|||||||||||||||||                Score: 1.5
   3' CTTAGGCCTGGTCCGAAGTAAGGTCCGTAAAC 5'  p-value: 0.0

0  #2  Position:1781  Abundance: 52.00(deg) 1(sRNA)
   5'      TCGGACCAGGCTTCATTCCCT         3'  ID:
           o|||||||||||||||||                Score: 2.5
   3' CTTAGGCCTGGTCCGAAGTAAGGTCCGTAAAC 5'  p-value: 0.0

0  #3  Position:1781  Abundance: 52.00(deg) 1(sRNA)
   5'      TCGGACCAGGCTTCATTCCCGT        3'  ID:
           o||||||||||||||||| |              Score: 2.5
   3' CTTAGGCCTGGTCCGAAGTAAGGTCCGTAAAC 5'  p-value: 0.0

0  #4  Position:1781  Abundance: 52.00(deg) 1(sRNA)
   5'      TCGGACCAGGCTTCATTCCCC         3'  ID:
           o|||||||||||||||||                Score: 2.5
   3' CTTAGGCCTGGTCCGAAGTAAGGTCCGTAAAC 5'  p-value: 0.0

0  #5  Position:1781  Abundance: 52.00(deg) 1(sRNA)
   5'      TCGGACCAGGCTTCATTCCCCC        3'  ID:
           o|||||||||||||||||                Score: 3.5
   3' CTTAGGCCTGGTCCGAAGTAAGGTCCGTAAAC 5'  p-value: 0.0
```

Cs1g15640.5 gene=Cs1g15640 CDS=121-2091

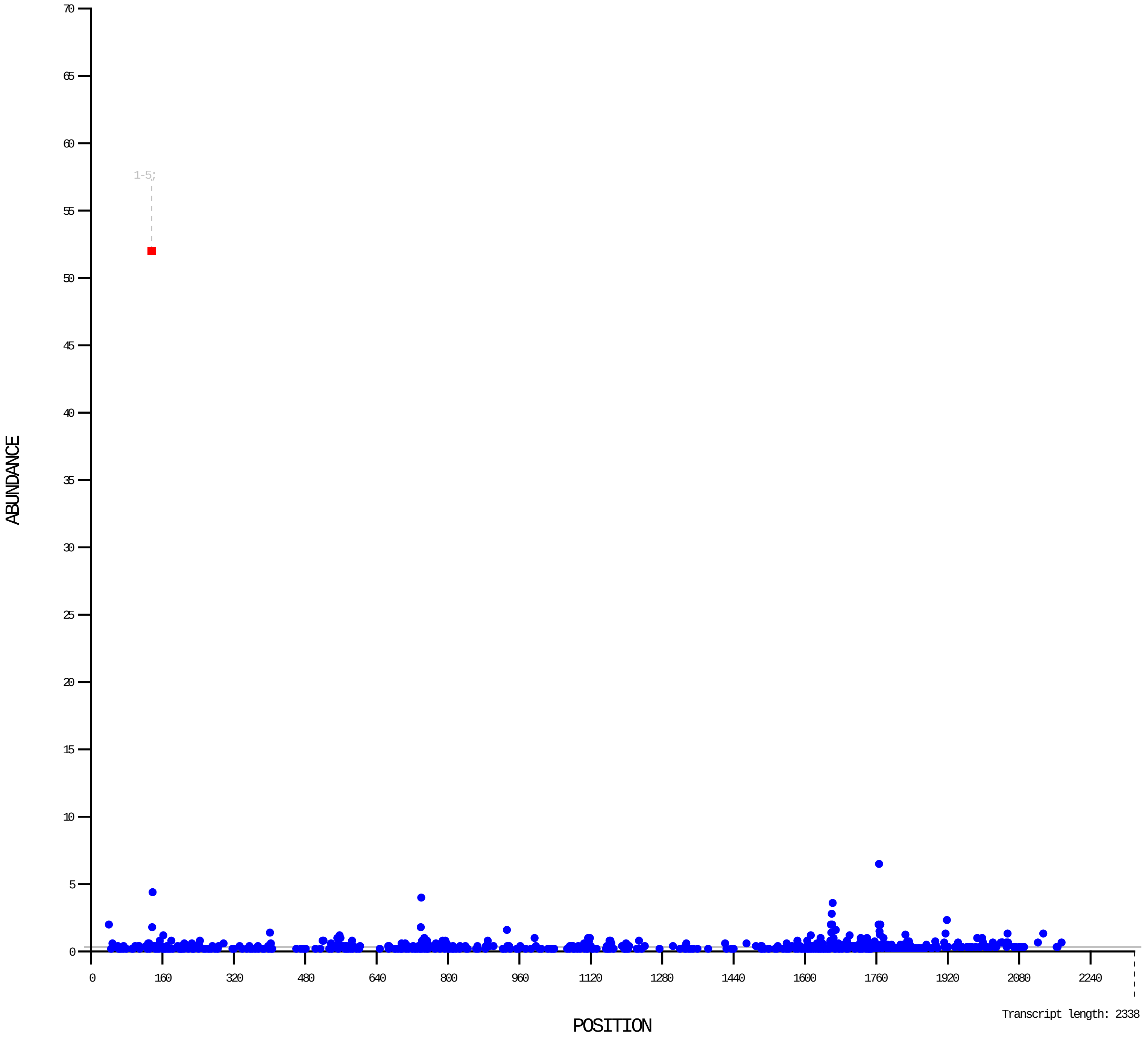

Category: 0 1 2 3 4
Degradome alignment: Median:

0 #1 Position:136 Abundance: 52.00(deg) 1(sRNA)
```
5'     TCGGACCAGGCTTCATTCCC         3'  ID:
       o|||||||||||||||||||             Score: 1.5
3' CTTAGGCCTGGTCCGAAGTAAGGTCCGTAAAC 5'  p-value: 0.0
```

0 #2 Position:136 Abundance: 52.00(deg) 1(sRNA)
```
5'     TCGGACCAGGCTTCATTCCCT        3'  ID:
       o|||||||||||||||||||             Score: 2.5
3' CTTAGGCCTGGTCCGAAGTAAGGTCCGTAAAC 5'  p-value: 0.0
```

0 #3 Position:136 Abundance: 52.00(deg) 1(sRNA)
```
5'     TCGGACCAGGCTTCATTCCCGT       3'  ID:
       o||||||||||||||||||| |           Score: 2.5
3' CTTAGGCCTGGTCCGAAGTAAGGTCCGTAAAC 5'  p-value: 0.0
```

0 #4 Position:136 Abundance: 52.00(deg) 1(sRNA)
```
5'     TCGGACCAGGCTTCATTCCCC        3'  ID:
       o|||||||||||||||||||             Score: 2.5
3' CTTAGGCCTGGTCCGAAGTAAGGTCCGTAAAC 5'  p-value: 0.0
```

0 #5 Position:136 Abundance: 52.00(deg) 1(sRNA)
```
5'     TCGGACCAGGCTTCATTCCCCC       3'  ID:
       o|||||||||||||||||||             Score: 3.5
3' CTTAGGCCTGGTCCGAAGTAAGGTCCGTAAAC 5'  p-value: 0.01
```

Cs1g15640.2 gene=Cs1g15640 CDS=1220-3649

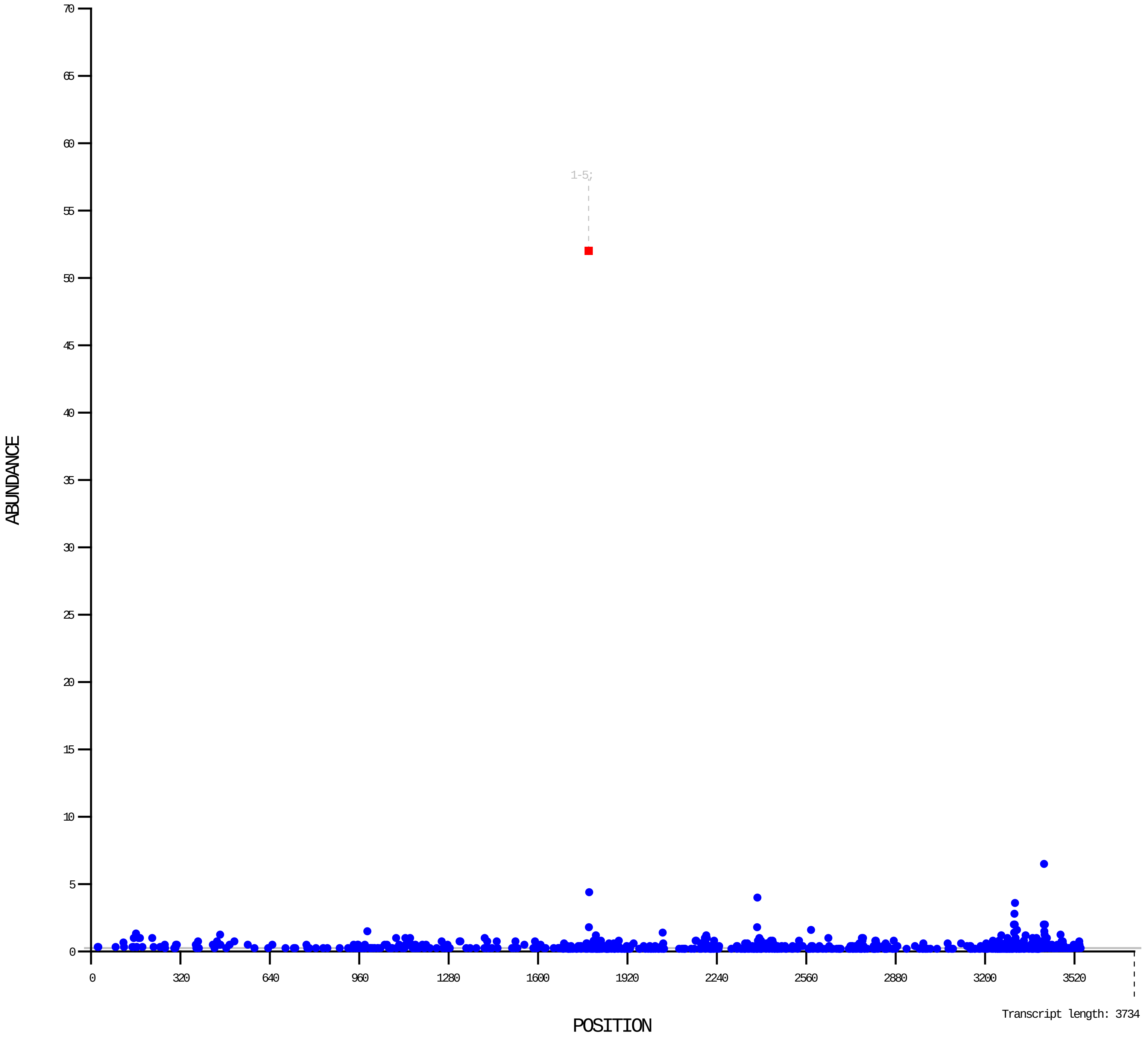

Category: 0 1 2 3 4

Degradome alignment: ● Median: —

```
0  #1  Position:1781  Abundance: 52.00(deg) 1(sRNA)
   5'     TCGGACCAGGCTTCATTCCC          3'  ID:
          o||||||||||||||||||               Score: 1.5
   3' CTTAGGCCTGGTCCGAAGTAAGGTCCGTAAAC 5'  p-value: 0.0

0  #2  Position:1781  Abundance: 52.00(deg) 1(sRNA)
   5'     TCGGACCAGGCTTCATTCCCT         3'  ID:
          o||||||||||||||||||               Score: 2.5
   3' CTTAGGCCTGGTCCGAAGTAAGGTCCGTAAAC 5'  p-value: 0.0

0  #3  Position:1781  Abundance: 52.00(deg) 1(sRNA)
   5'     TCGGACCAGGCTTCATTCCCGT        3'  ID:
          o|||||||||||||||||| |             Score: 2.5
   3' CTTAGGCCTGGTCCGAAGTAAGGTCCGTAAAC 5'  p-value: 0.0

0  #4  Position:1781  Abundance: 52.00(deg) 1(sRNA)
   5'     TCGGACCAGGCTTCATTCCCC         3'  ID:
          o||||||||||||||||||               Score: 2.5
   3' CTTAGGCCTGGTCCGAAGTAAGGTCCGTAAAC 5'  p-value: 0.0

0  #5  Position:1781  Abundance: 52.00(deg) 1(sRNA)
   5'     TCGGACCAGGCTTCATTCCCCC        3'  ID:
          o||||||||||||||||||               Score: 3.5
   3' CTTAGGCCTGGTCCGAAGTAAGGTCCGTAAAC 5'  p-value: 0.0
```

Cs5g19920.1 gene=Cs5g19920 CDS=1-3840

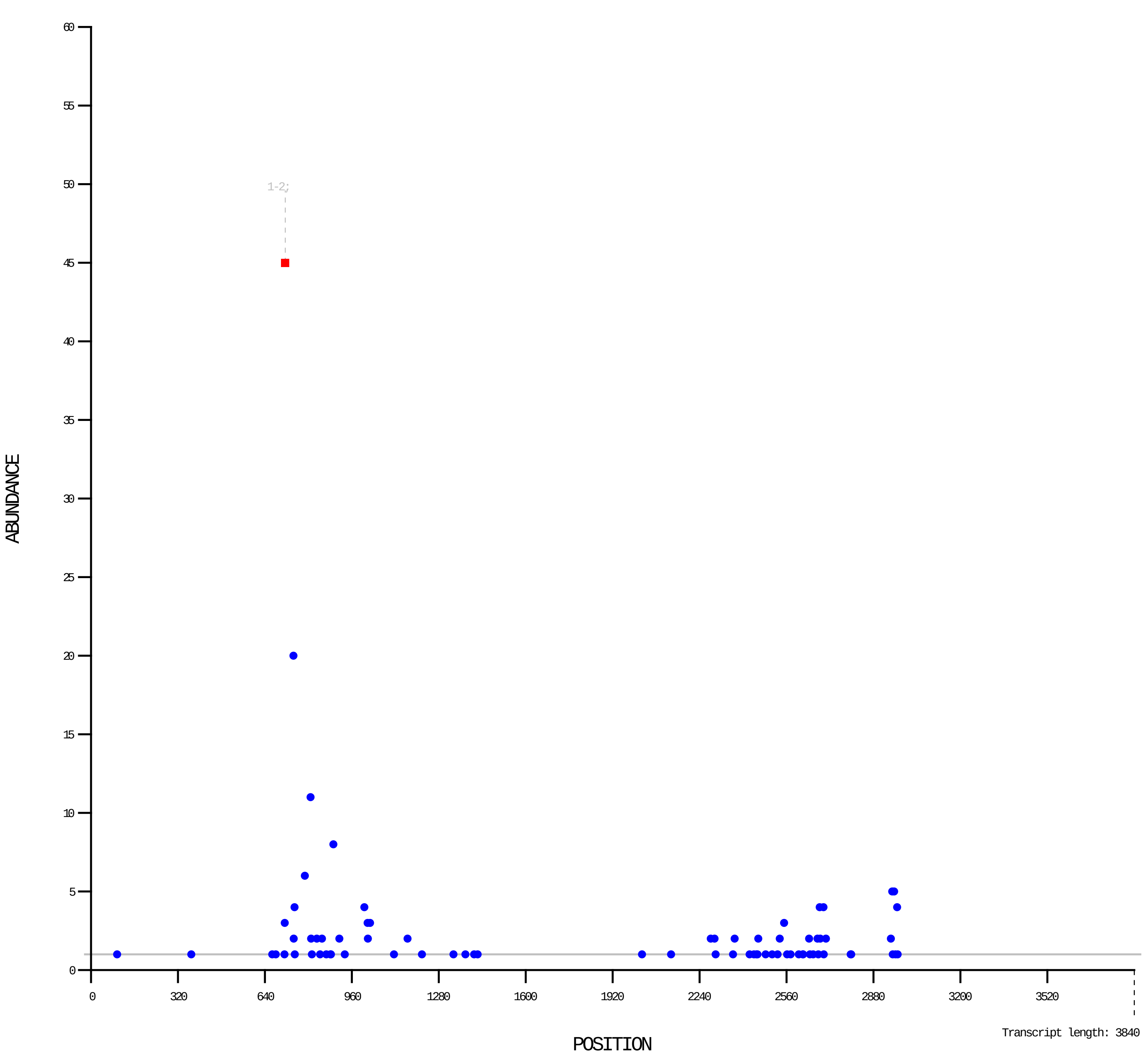

Category: 0 1 2 3 4

Degradome alignment: ● Median: —

```
0  #1  Position:715  Abundance: 45.00(deg) 1(sRNA)
   5'     TCTTACCTATGCCACCCATTCC       3'  ID:
          |||| |||||o|||||||| ||           Score: 2.5
   3' CAACAGAACGGATATGGTGGGTACGGGGTTTA 5'  p-value: 0.0

0  #2  Position:715  Abundance: 45.00(deg) 1(sRNA)
   5'     TCTTCCCTATGCCTCCCATTCC       3'  ID:
          |||| |||||o|| ||||| ||           Score: 3.5
   3' CAACAGAACGGATATGGTGGGTACGGGGTTTA 5'  p-value: 0.0
```

Cs8g10560.1 gene=Cs8g10560 CDS=1-1557

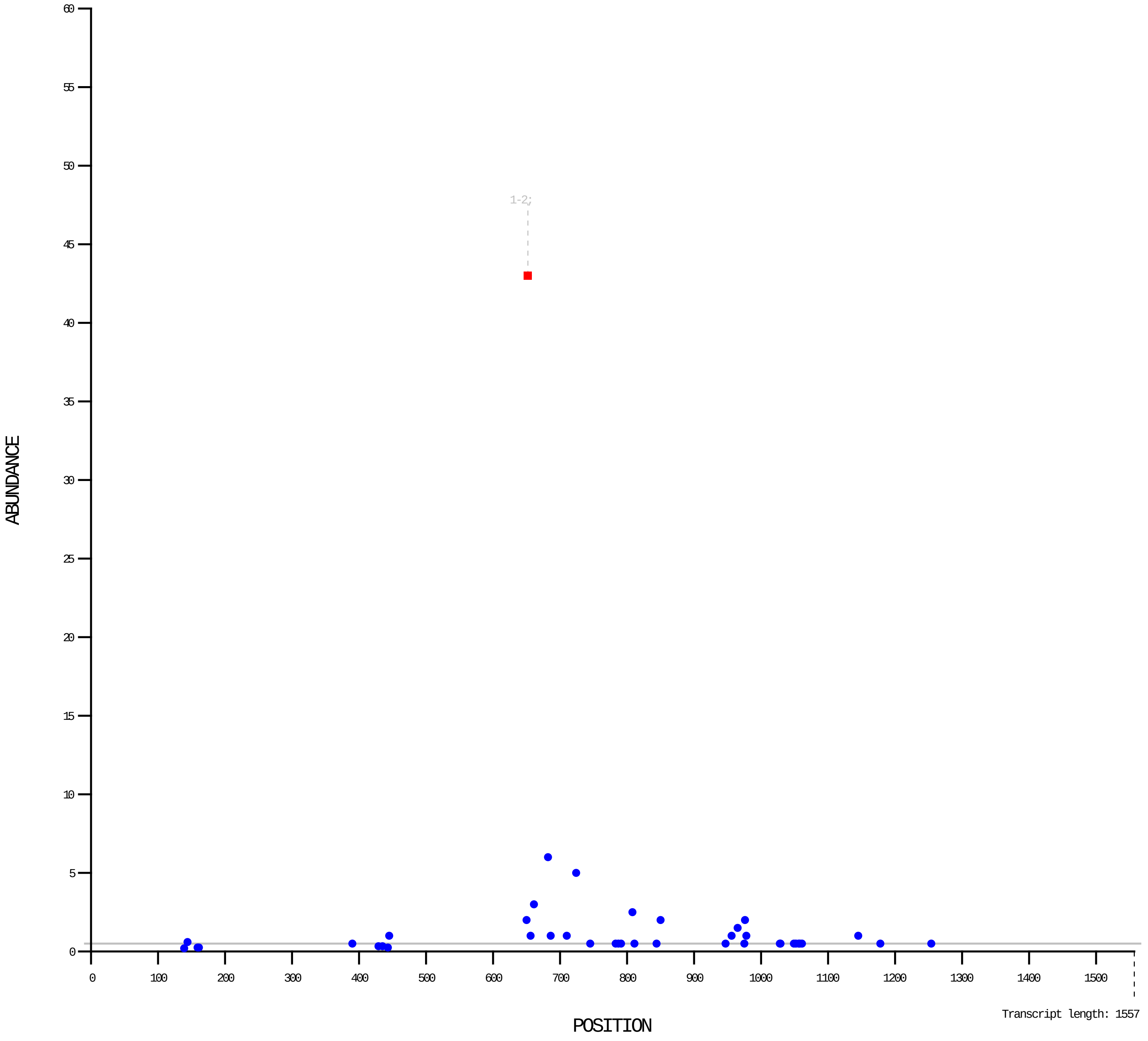

Category: 0 1 2 3 4

Degradome alignment: ● Median:

```
0  #1  Position:652  Abundance: 43.00(deg) 1(sRNA)
   5'     TCTTACCTATGCCACCCATTCC       3'  ID:
          |||| |||||o||||| || ||           Score: 3.5
   3' CAACAGAACGGATATGGTGGCTACGGGGTTTA 5'  p-value: 0.01

0  #2  Position:652  Abundance: 43.00(deg) 1(sRNA)
   5'     TCTTCCCTATGCCTCCCATTCC       3'  ID:
          |||| |||||o|| || || ||           Score: 4.5
   3' CAACAGAACGGATATGGTGGCTACGGGGTTTA 5'  p-value: 0.04
```

orange1.1t03122.1 gene=orange1.1t03122 CDS=243-1316

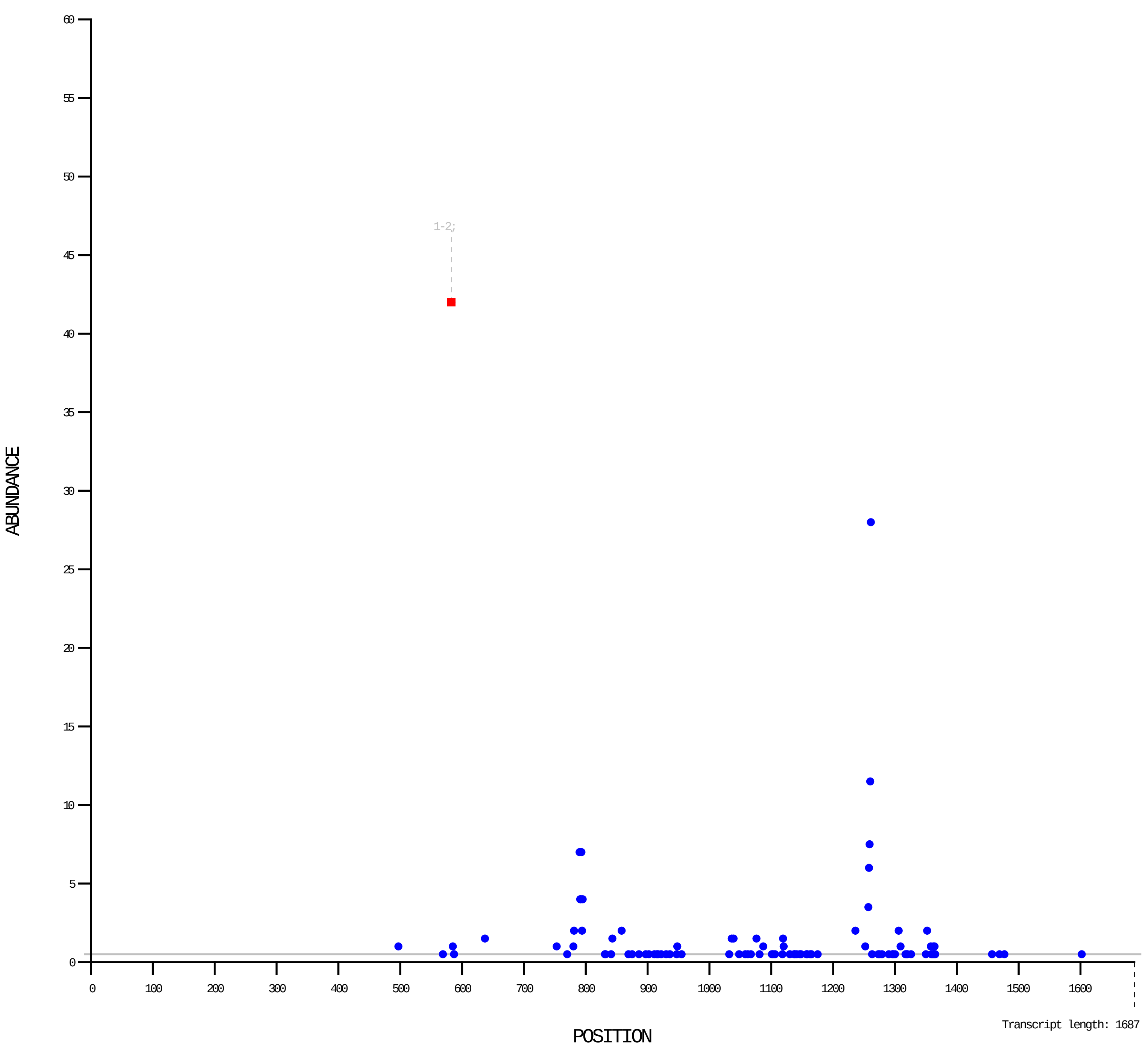


```
Category: ■ 0  ■ 1  ■ 2  ■ 3  ■ 4
Degradome alignment: ●    Median: ——

■ 0  #1  Position:583  Abundance: 42.00(deg) 1(sRNA)
     5'    TTCCAC-GGCTTTCTTGAACGT        3'  ID:
           |||||| ||||||||||||||             Score: 2.0
     3' ACTAAGGTGTCCGAAAGAACTTGCCATAGCTG 5'  p-value: 0.0

■ 0  #2  Position:583  Abundance: 42.00(deg) 1(sRNA)
     5'    TTCCACA-GCTTTCTTGAACTG        3'  ID:
           ||||||| |||||||||||| |            Score: 2.0
     3' ACTAAGGTGTCCGAAAGAACTTGCCATAGCTG 5'  p-value: 0.0
```

orange1.1t03122.2 gene=orange1.1t03122 CDS=243-1328

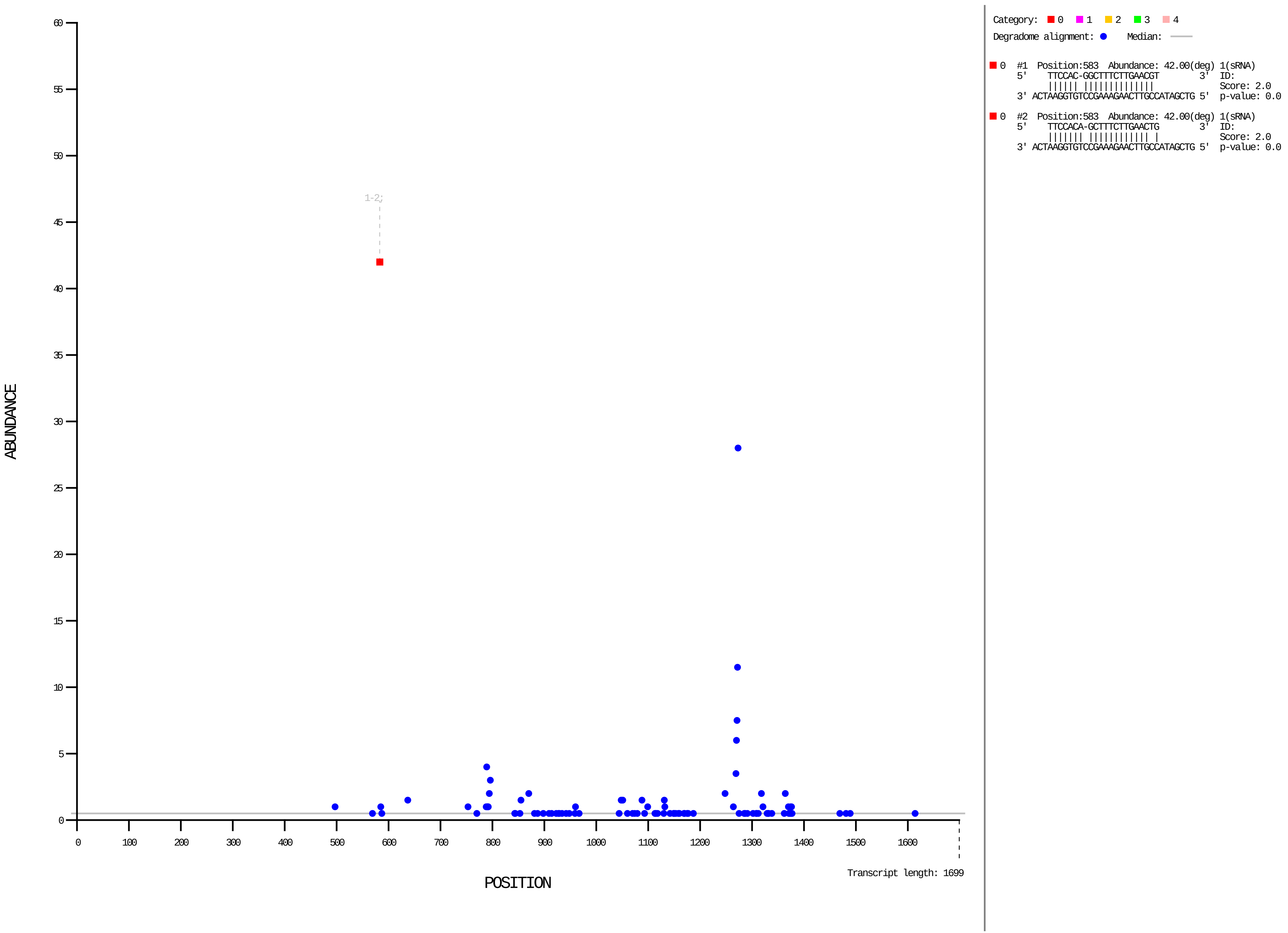

Cs7g27480.1 gene=Cs7g27480 CDS=1-783

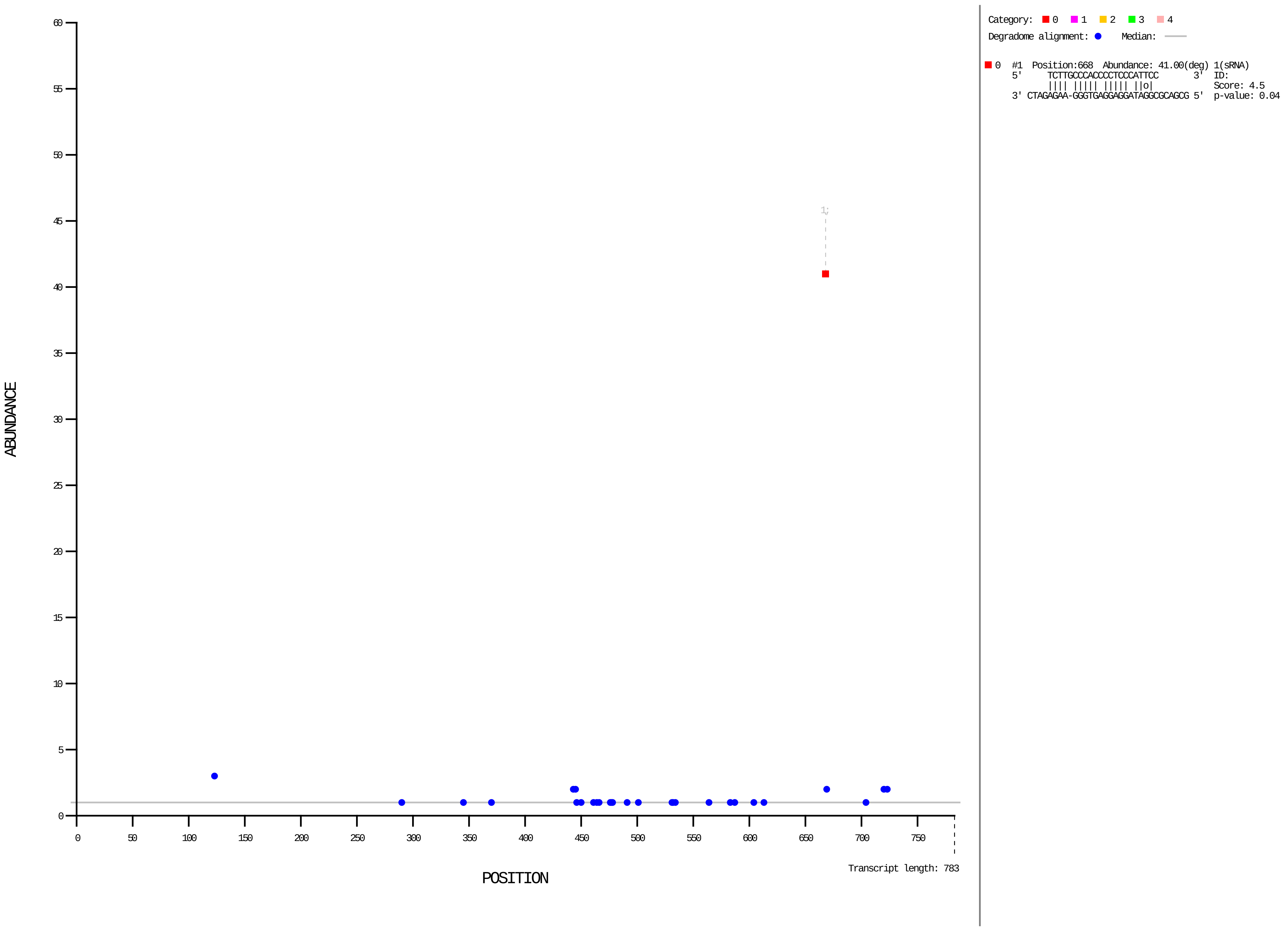

Cs4g11200.1 gene=Cs4g11200 CDS=299-613

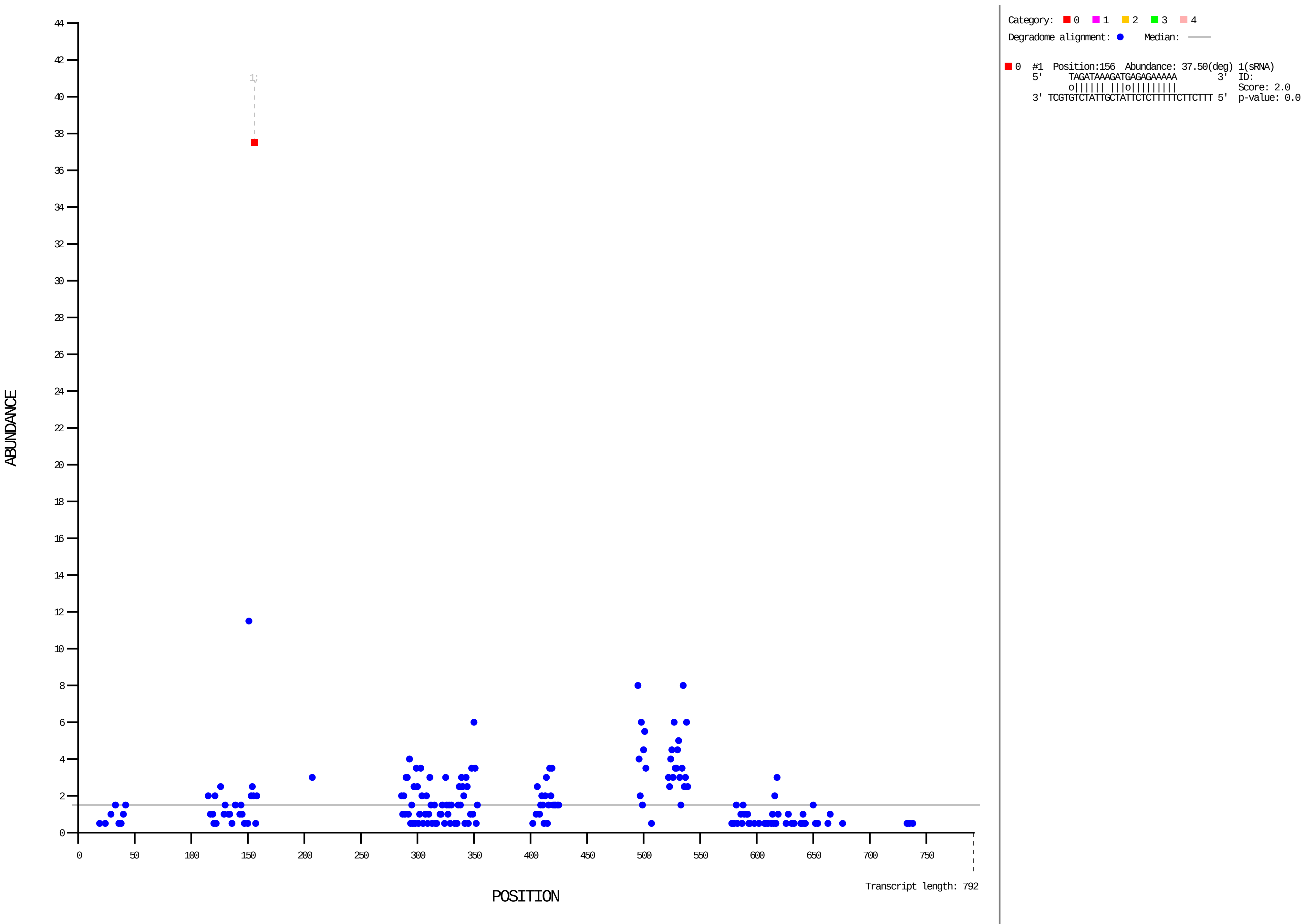

Cs4g11200.2 gene=Cs4g11200 CDS=833-1147

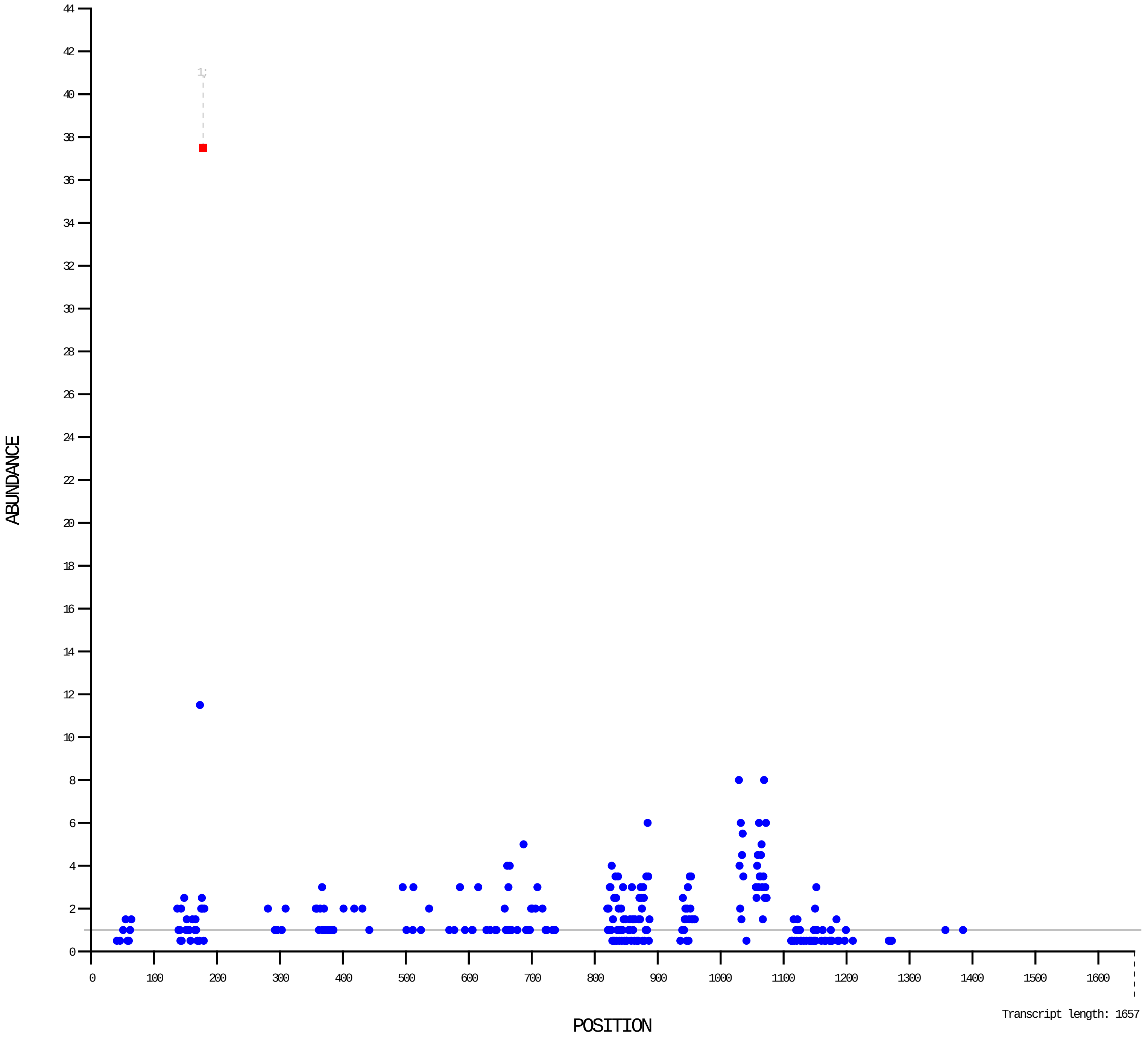

Category: 0 1 2 3 4

Degradome alignment: ● Median:

```
0  #1  Position:178  Abundance: 37.50(deg) 1(sRNA)
   5'     TAGATAAAGATGAGAGAAAAA        3'  ID:
          o|||||| |||o||||||||||           Score: 2.0
   3' TCGTGTCTATTGCTATTCTCTTTTTCTTCTTT 5'  p-value: 0.0
```

Cs3g27280.3 gene=Cs3g27280 CDS=92-1369

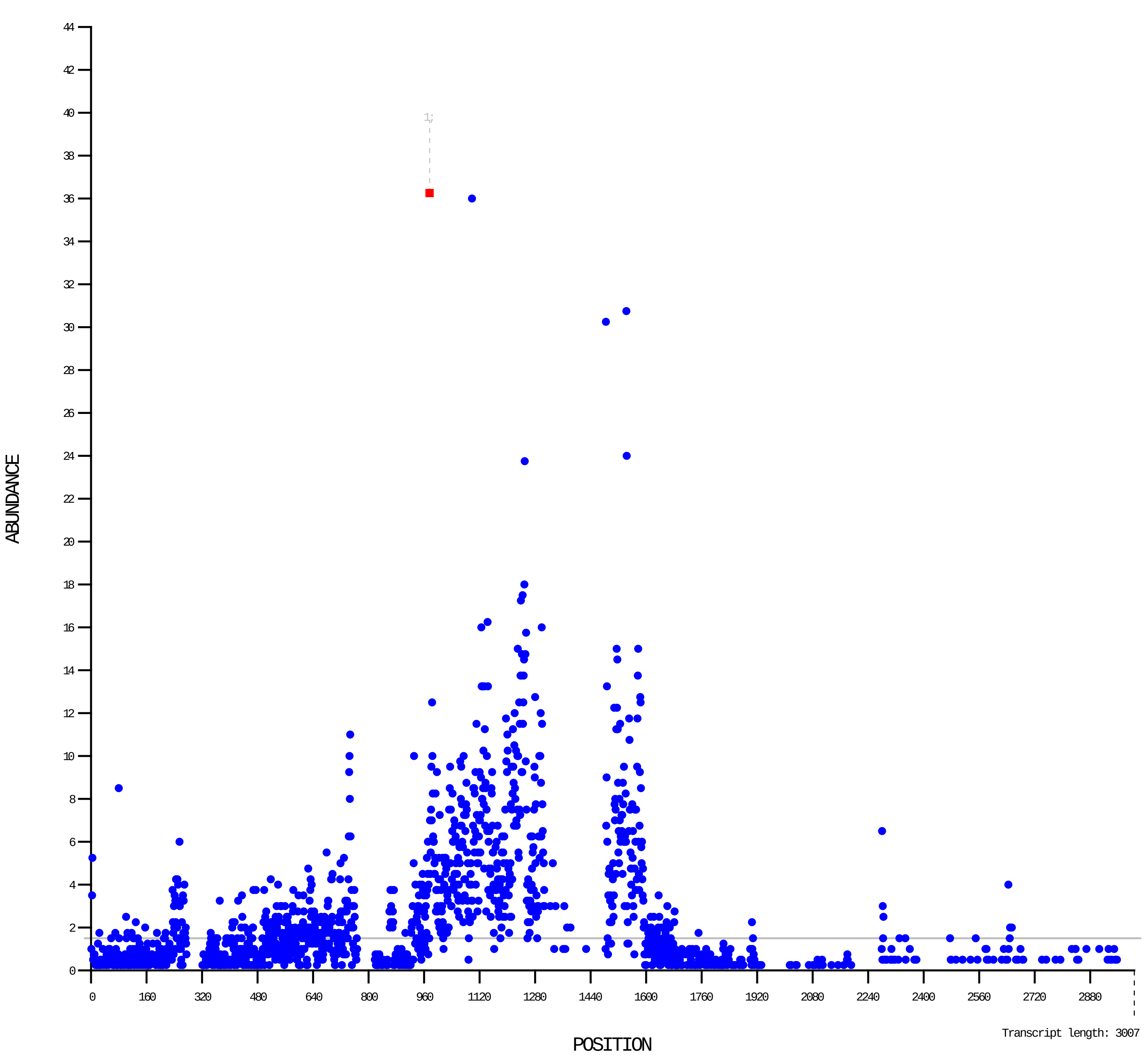

Category: 0 1 2 3 4
Degradome alignment: Median:

0 #1 Position:976 Abundance: 36.25(deg) 1(sRNA)
5' TG-TGTTCTCAGGTCACCCCTT 3' ID:
|| |||o||||||o|||| |||
3' CCTACTACAGGAGTCCGGTGGTGAACCAATGC 5'
Score: 3.0
p-value: 0.0

Cs7g31620.1 gene=Cs7g31620 CDS=26-1687

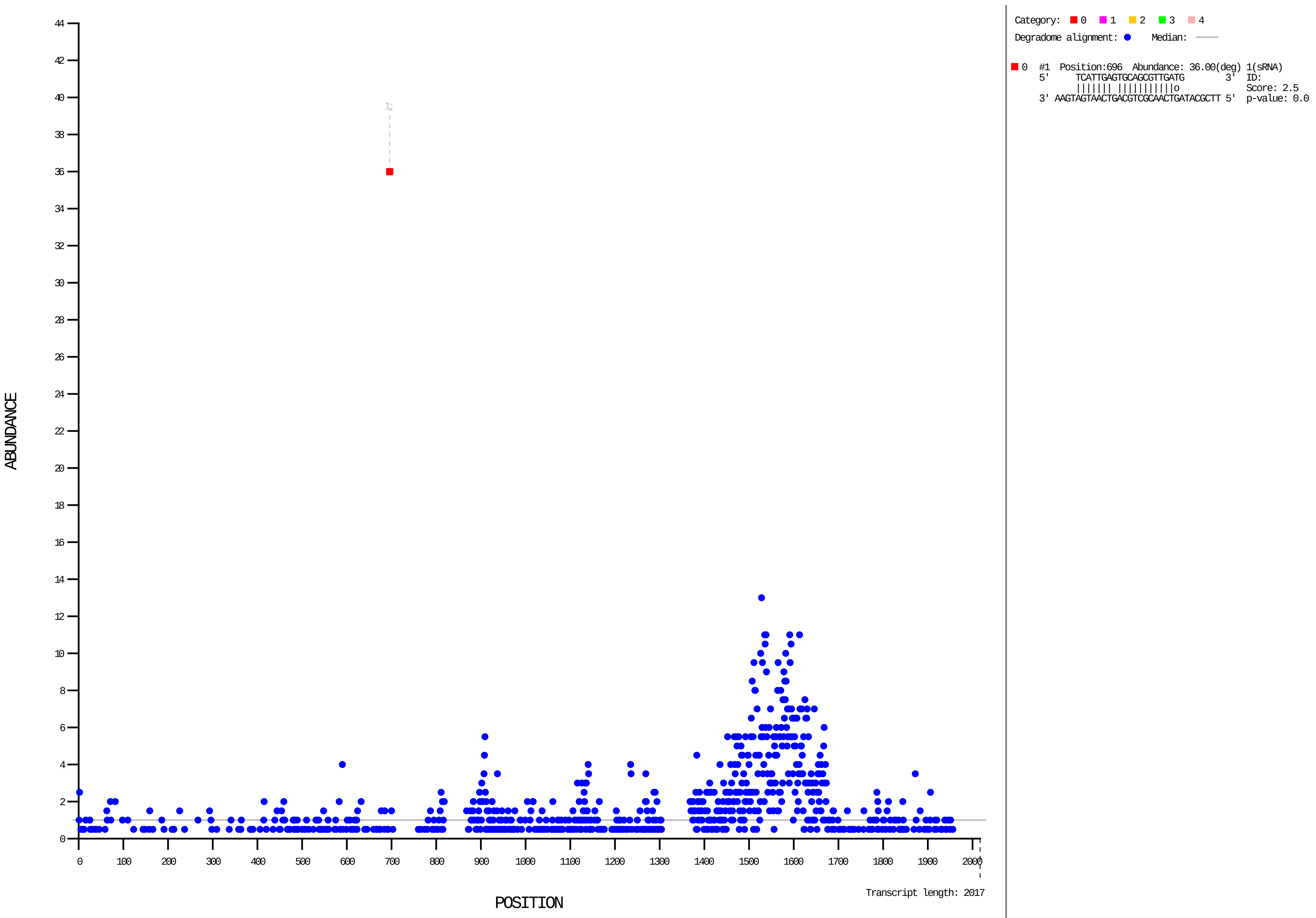

Cs7g31620.2 gene=Cs7g31620 CDS=197-1777

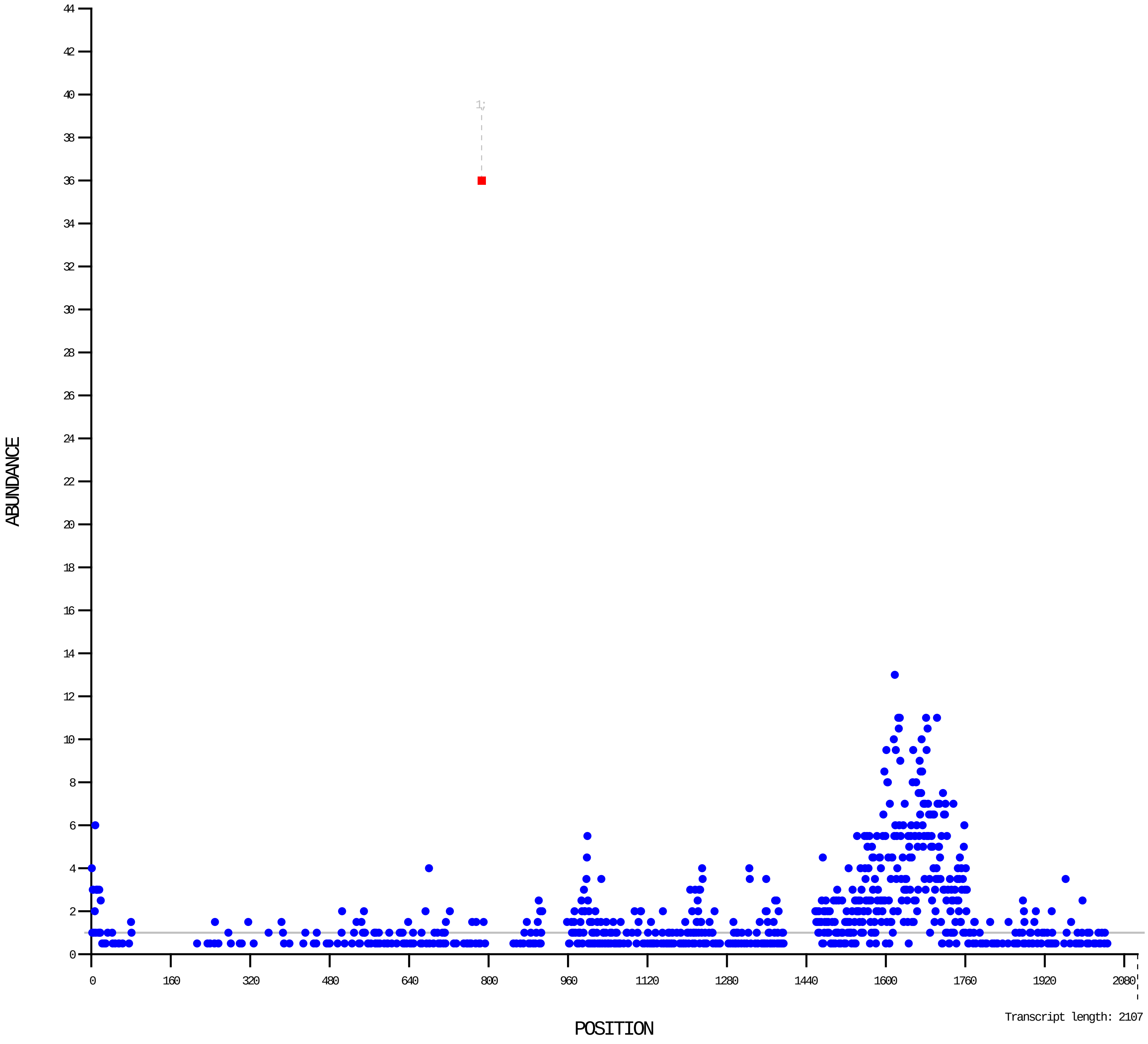

Category: 0 1 2 3 4
Degradome alignment: Median:

0 #1 Position:786 Abundance: 36.00(deg) 1(sRNA)
5' TCATTGAGTGCAGCGTTGATG 3' ID:
||||||| |||||||||||o Score: 2.5
3' AAGTAGTAACTGACGTCGCAACTGATACGCTT 5' p-value: 0.0

Cs6g11800.1 gene=Cs6g11800 CDS=650-2761

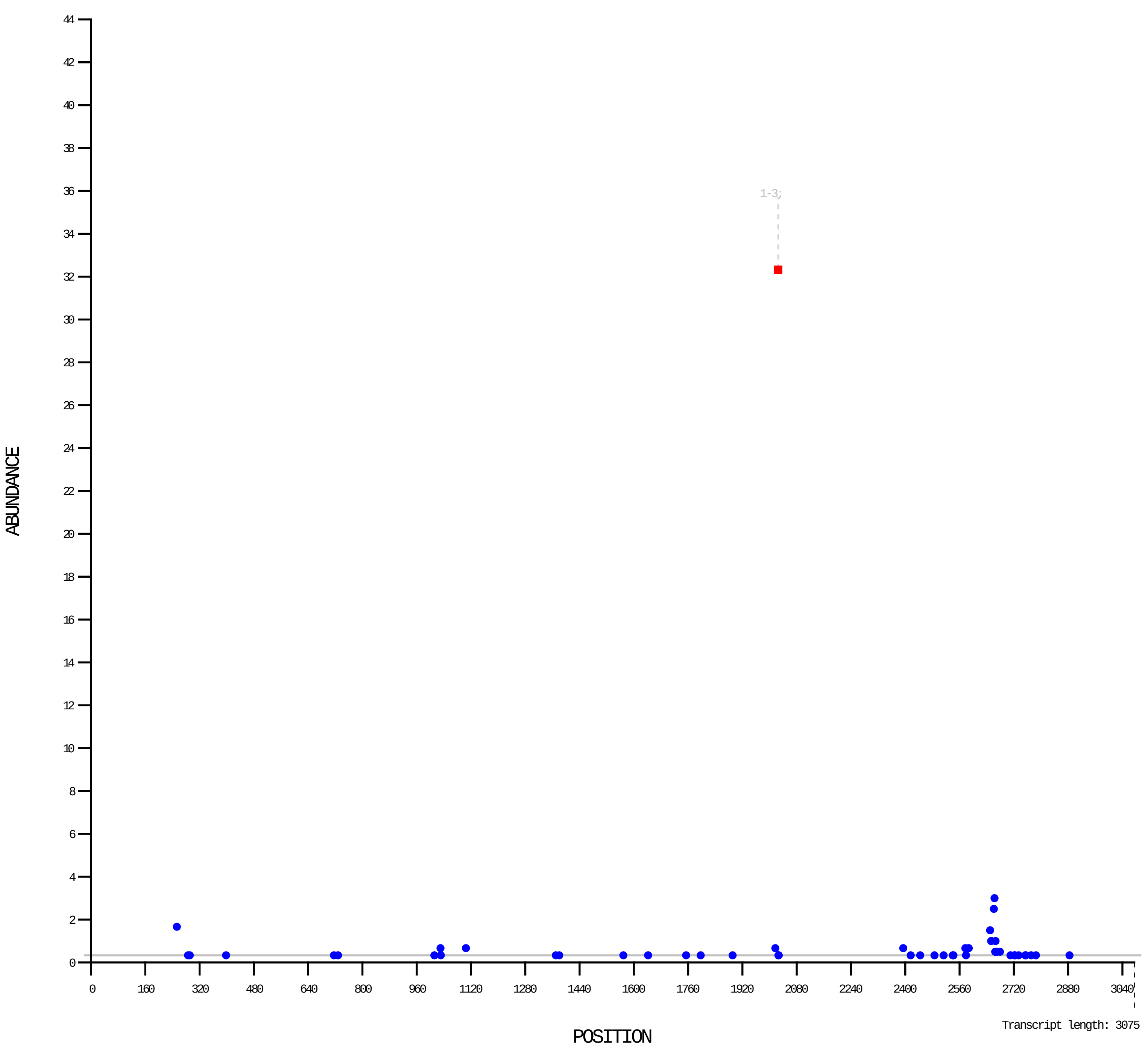

Category: 0 1 2 3 4

Degradome alignment: ● Median:

```
0  #1  Position:2025  Abundance: 32.33(deg) 1(sRNA)
   5'     TGCCTGGCTCCCTGTATGCCA        3'  ID:
          ||||||||||||||o||||||            Score: 0.5
   3' TCGTACGGACCGAGGGACGTACGGTCGTCCTC 5'  p-value: 0.0

0  #2  Position:2025  Abundance: 32.33(deg) 1(sRNA)
   5'     TGCCTGGCTCCCTGTATGCCG        3'  ID:
          ||||||||||||||o|||||o            Score: 1.0
   3' TCGTACGGACCGAGGGACGTACGGTCGTCCTC 5'  p-value: 0.0

0  #3  Position:2025  Abundance: 32.33(deg) 1(sRNA)
   5'     TGCCTGGCTCCCTGTATGCTT        3'  ID:
          ||||||||||||||o||||o             Score: 2.0
   3' TCGTACGGACCGAGGGACGTACGGTCGTCCTC 5'  p-value: 0.0
```

Cs6g11800.3 gene=Cs6g11800 CDS=650-2797

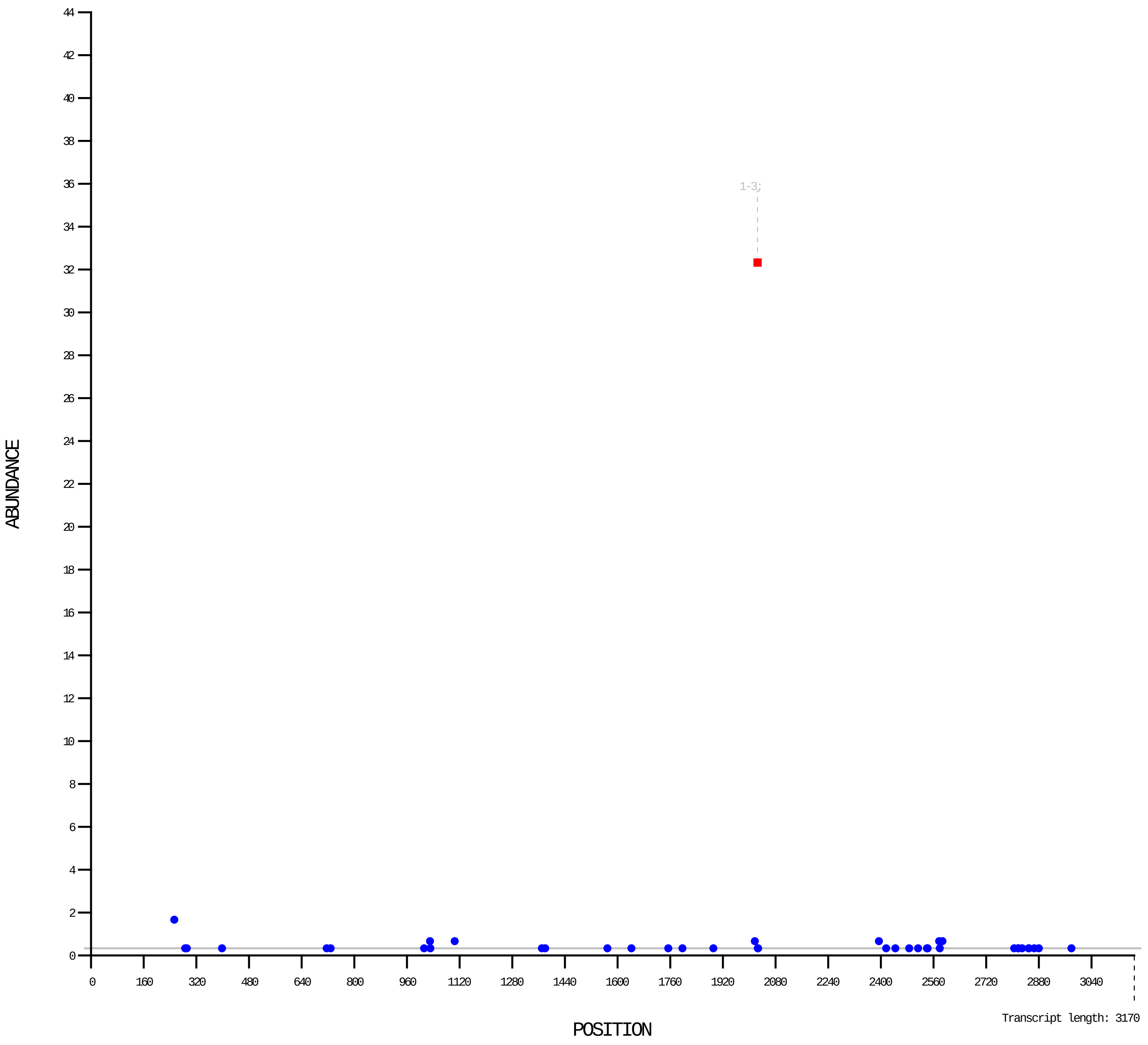

Category: 0 1 2 3 4
Degradome alignment: ● Median:

■ 0 #1 Position:2025 Abundance: 32.33(deg) 1(sRNA)
```
5'     TGCCTGGCTCCCTGTATGCCA        3'  ID:
       ||||||||||||||o||||||            Score: 0.5
3' TCGTACGGACCGAGGGACGTACGGTCGTCCTC 5'  p-value: 0.0
```

■ 0 #2 Position:2025 Abundance: 32.33(deg) 1(sRNA)
```
5'     TGCCTGGCTCCCTGTATGCCG        3'  ID:
       ||||||||||||||o|||||o            Score: 1.0
3' TCGTACGGACCGAGGGACGTACGGTCGTCCTC 5'  p-value: 0.0
```

■ 0 #3 Position:2025 Abundance: 32.33(deg) 1(sRNA)
```
5'     TGCCTGGCTCCCTGTATGCTT        3'  ID:
       ||||||||||||||o||||o             Score: 2.0
3' TCGTACGGACCGAGGGACGTACGGTCGTCCTC 5'  p-value: 0.0
```

Cs6g11800.2 gene=Cs6g11800 CDS=778-2889

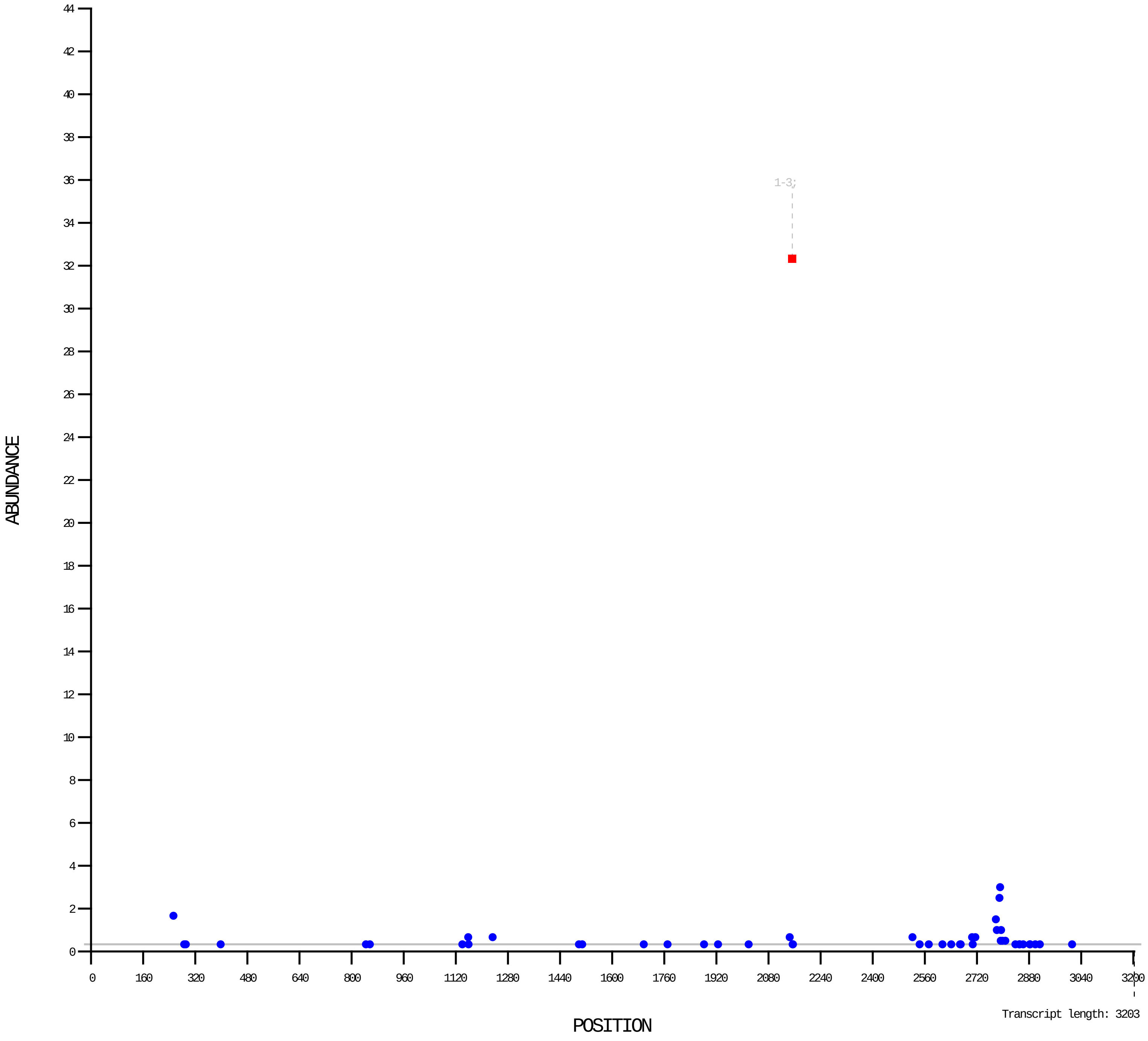

Category: 0 1 2 3 4
Degradome alignment: Median:

```
0  #1  Position:2153  Abundance: 32.33(deg) 1(sRNA)
   5'     TGCCTGGCTCCCTGTATGCCA        3'  ID:
          ||||||||||||||o||||||            Score: 0.5
   3' TCGTACGGACCGAGGGACGTACGGTCGTCCTC 5'  p-value: 0.0

0  #2  Position:2153  Abundance: 32.33(deg) 1(sRNA)
   5'     TGCCTGGCTCCCTGTATGCCG        3'  ID:
          ||||||||||||||o|||||o            Score: 1.0
   3' TCGTACGGACCGAGGGACGTACGGTCGTCCTC 5'  p-value: 0.0

0  #3  Position:2153  Abundance: 32.33(deg) 1(sRNA)
   5'     TGCCTGGCTCCCTGTATGCTT        3'  ID:
          ||||||||||||||o||||o             Score: 2.0
   3' TCGTACGGACCGAGGGACGTACGGTCGTCCTC 5'  p-value: 0.0
```

Cs6g21520.1 gene=Cs6g21520 CDS=283-1329

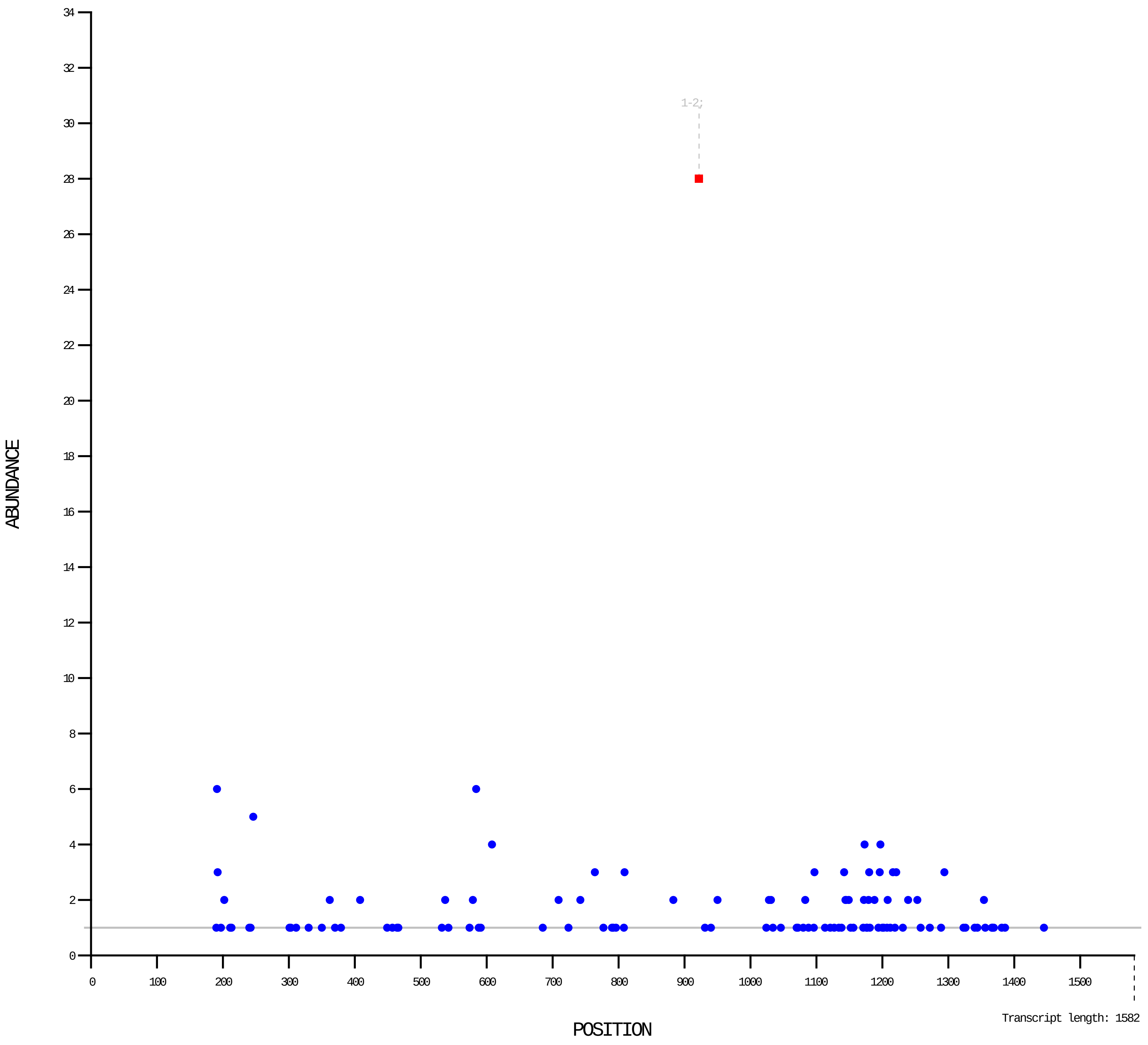

Category: 0 1 2 3 4
Degradome alignment: Median:

0 #1 Position:922 Abundance: 28.00(deg) 1(sRNA)
```
5'     TGGAGAAGCAGGGCACGTGCA         3'  ID:
       o|||||||||||| |||||| |            Score: 2.5
3' CGTAGCCTCTTCGTCCAGTGCAC-TCTCCAAA 5'  p-value: 0.0
```

0 #2 Position:922 Abundance: 28.00(deg) 1(sRNA)
```
5'     TGGAGAAGCAGGGCACATGCT         3'  ID:
       o|||||||||||| ||| ||              Score: 4.5
3' CGTAGCCTCTTCGTCCAGTGCACTCTCCAAAG 5'  p-value: 0.01
```

Cs1g14030.1 gene=Cs1g14030 CDS=1128-3785

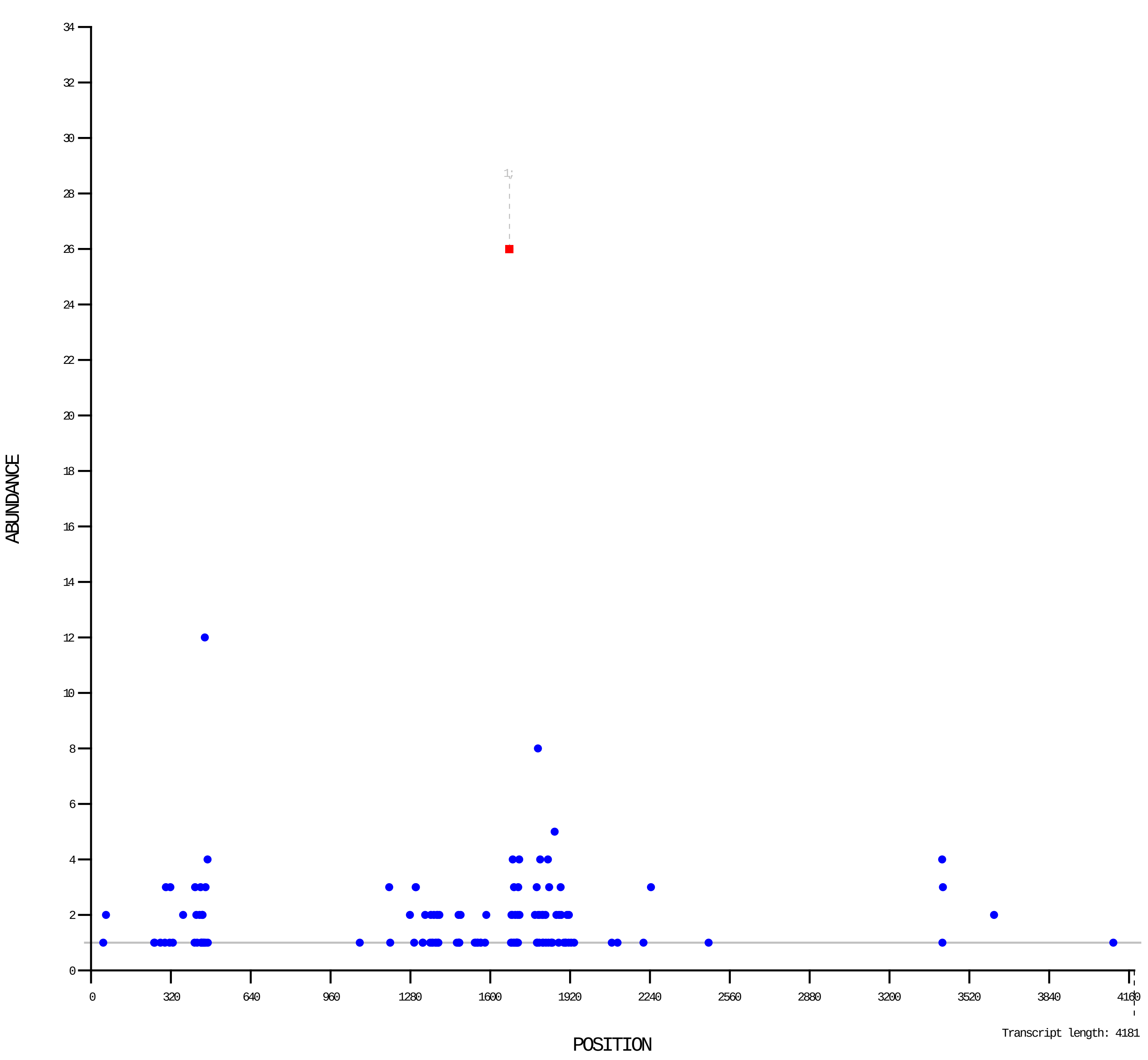

Category: 0 1 2 3 4
Degradome alignment: Median:

0 #1 Position:1677 Abundance: 26.00(deg) 1(sRNA)

```
5'     TCTTGCCCACCCCTCCCATTCC      3'  ID:
       |||| || || || ||||||||          Score: 4.0
3' CACCAGAAGGGCTGAGGTGGGTAAGGCATGTT 5'  p-value: 0.0
```

Cs7g15220.1 gene=Cs7g15220 CDS=1-999

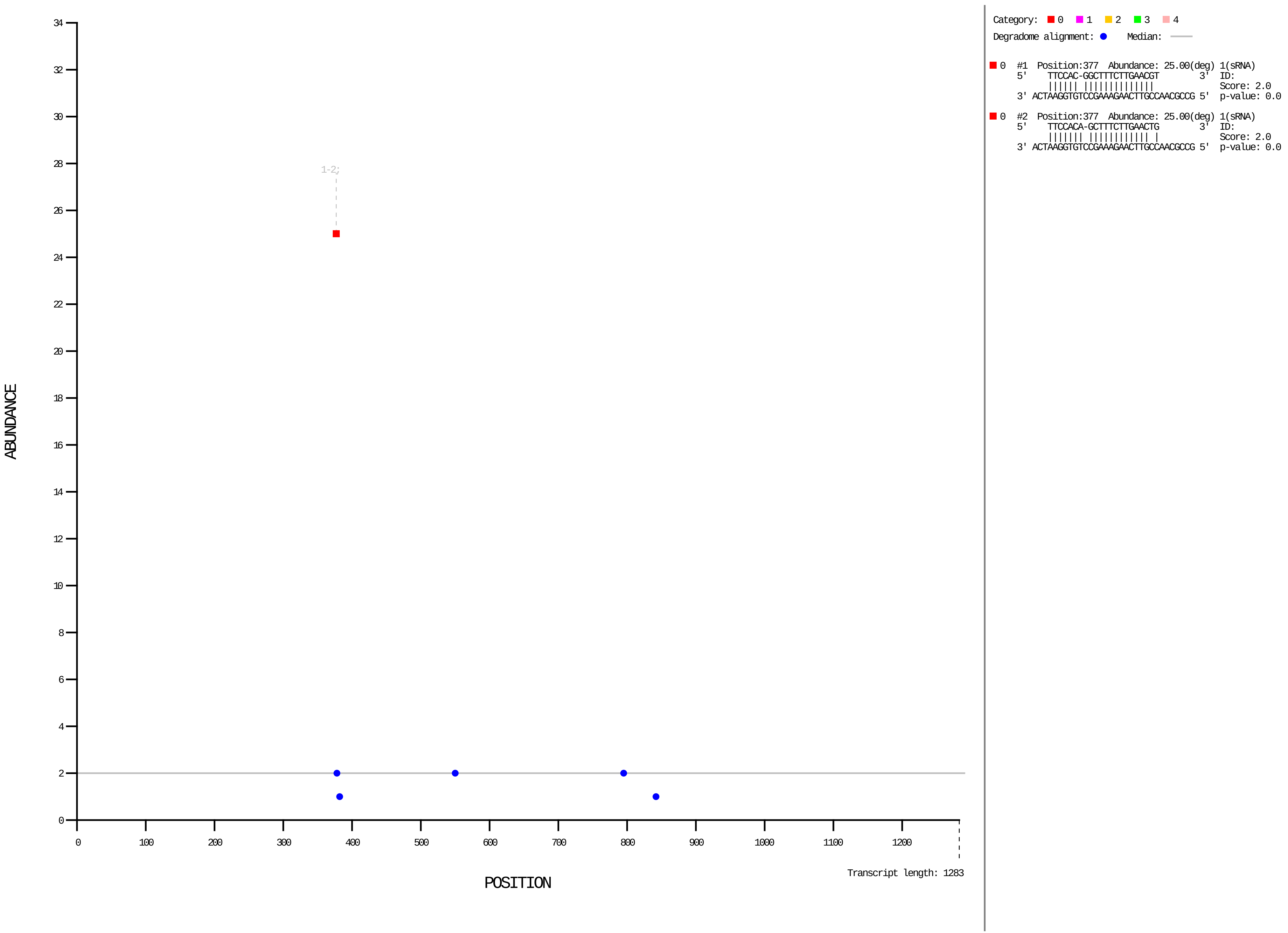

Cs1g03640.1 gene=Cs1g03640 CDS=825-1994

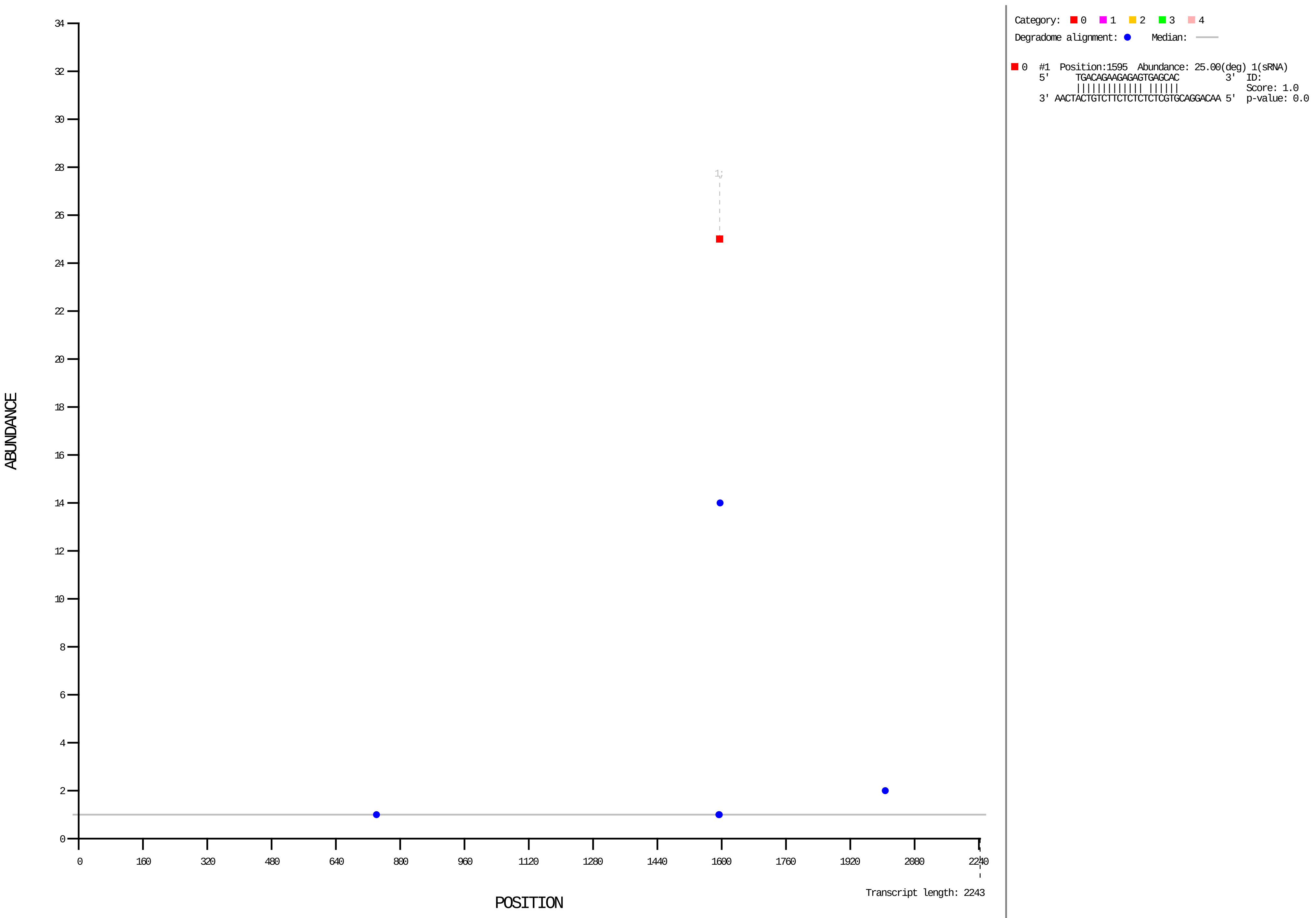

Cs6g06920.1 gene=Cs6g06920 CDS=1-1737

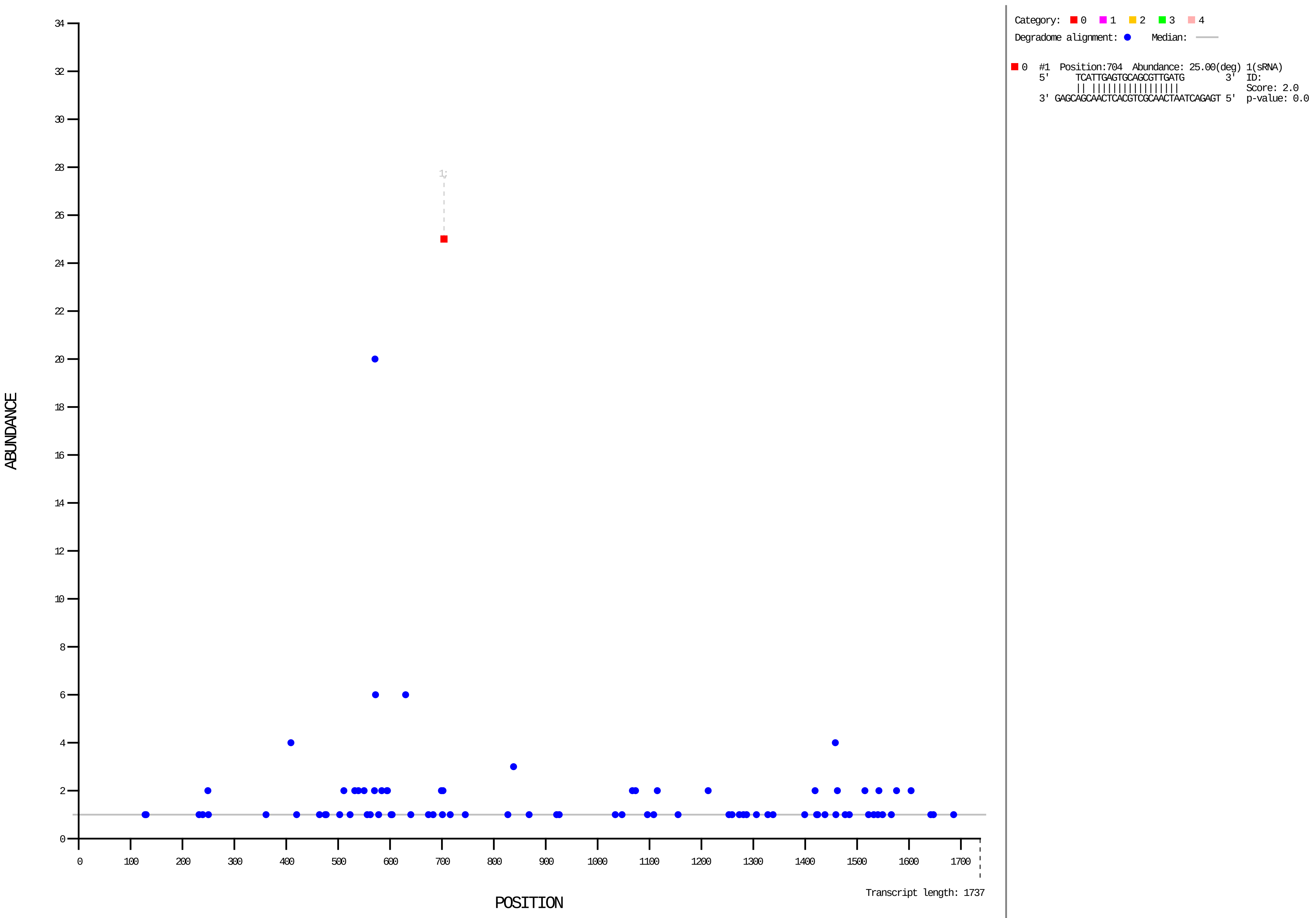

Cs5g21975.1 gene=Cs5g21975 CDS=82-3237

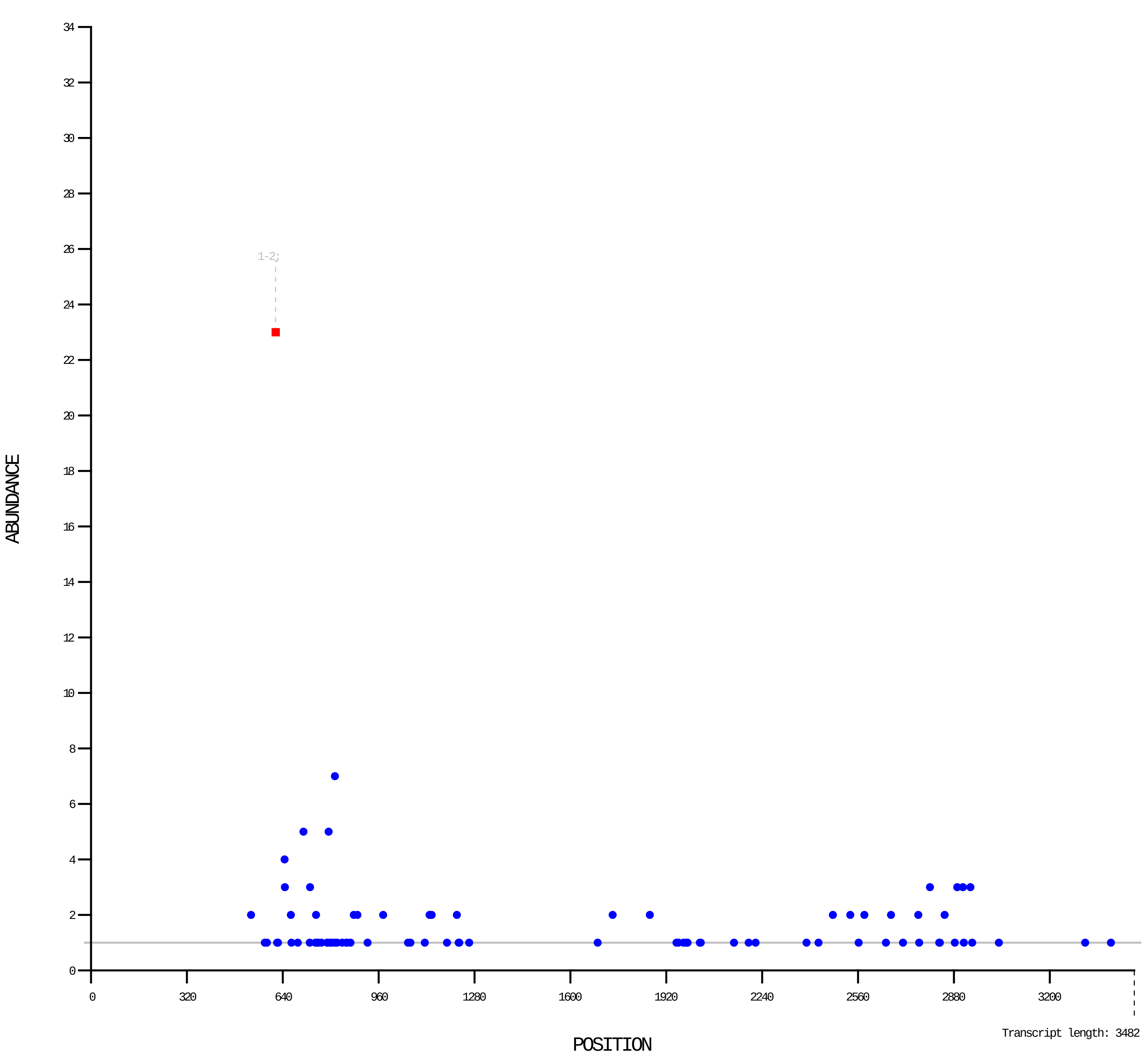

0 #1 Position:616 Abundance: 23.00(deg) 1(sRNA)

```
5'     TCTTACCTATGCCACCCATTCC       3'  ID:
       |||| ||||| ||||||||o||           Score: 2.5
3' TATCAGAAAGGATAAGGTGGGTAGGGGTGTTA 5'  p-value: 0.0
```

0 #2 Position:616 Abundance: 23.00(deg) 1(sRNA)

```
5'     TCTTCCCTATGCCTCCCATTCC       3'  ID:
       |||| ||||| || |||||o||           Score: 3.5
3' TATCAGAAAGGATAAGGTGGGTAGGGGTGTTA 5'  p-value: 0.0
```

orange1.1t03289.1 gene=orange1.1t03289 CDS=136-1209

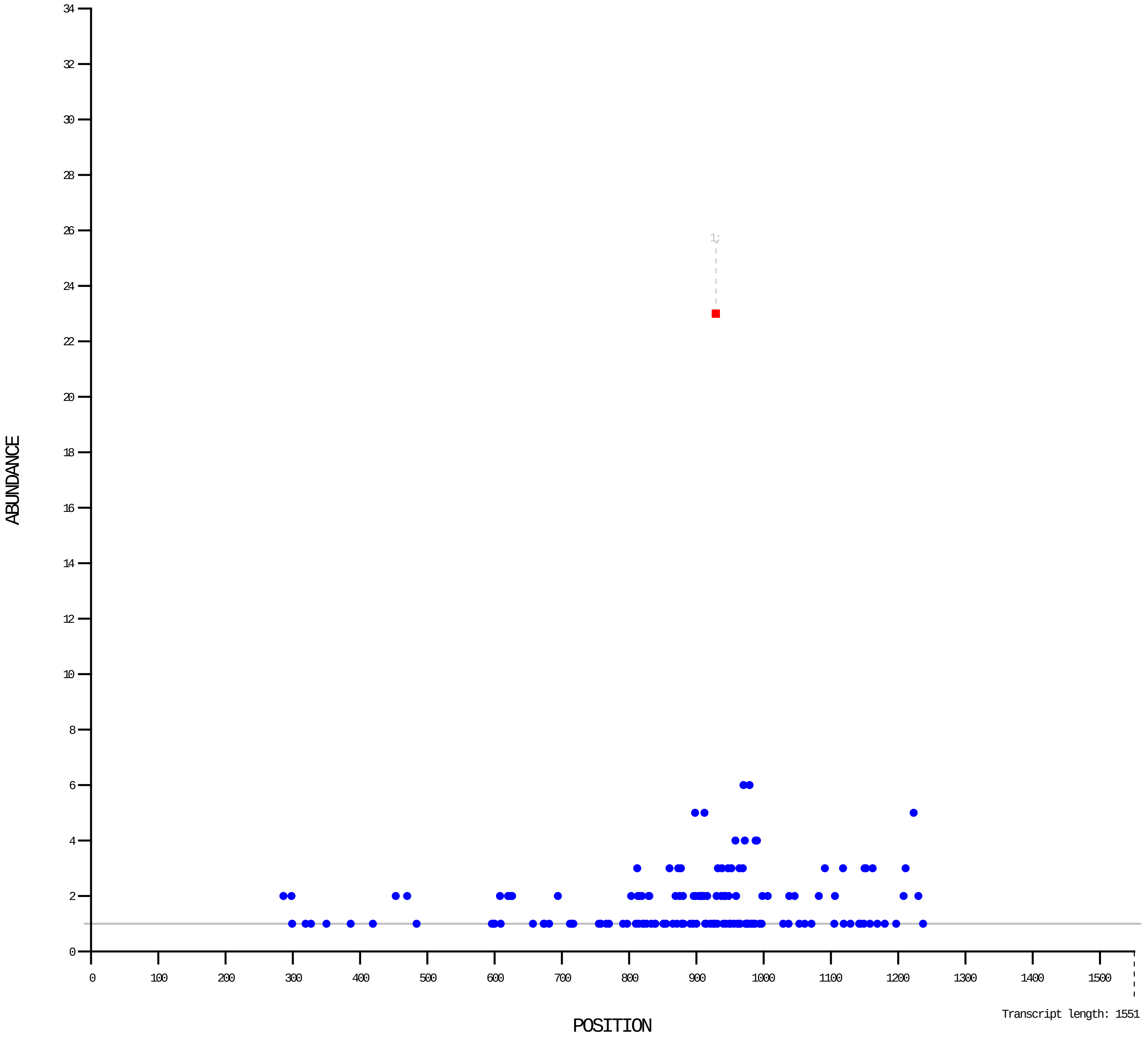

Category: 0 1 2 3 4
Degradome alignment: Median:

0 #1 Position:929 Abundance: 23.00(deg) 1(sRNA)

```
5'     TTTGGATTGAAGGGAGCTCTA       3'  ID:
       |o|||||||| o|||o|||||           Score: 2.5
3' TCGAAGACCTAACTCTCCTTGAGATATGGTAA 5'  p-value: 0.0
```

Cs1g24520.1 gene=Cs1g24520 CDS=191-2740

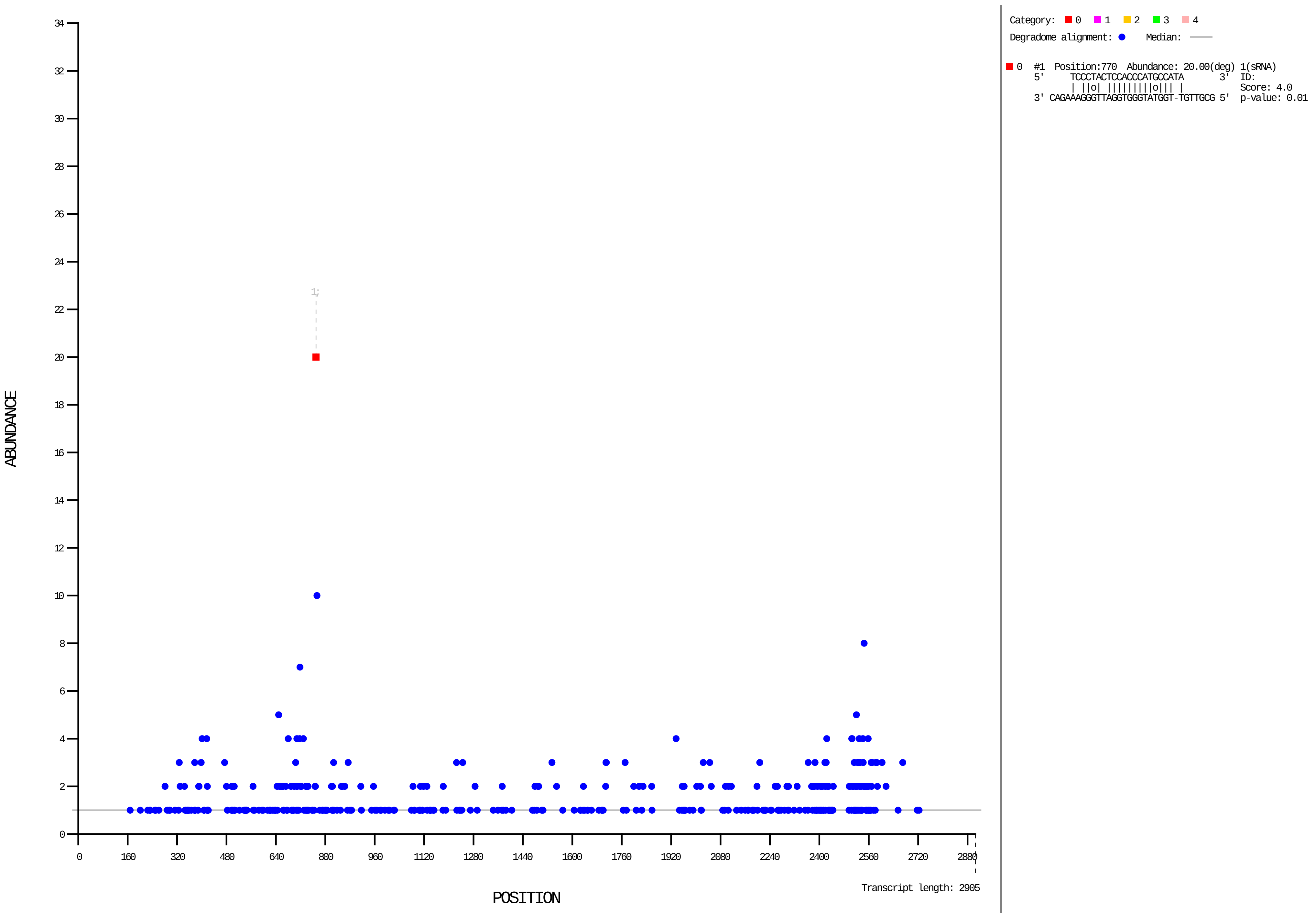

Cs6g07450.1 gene=Cs6g07450 CDS=204-1913

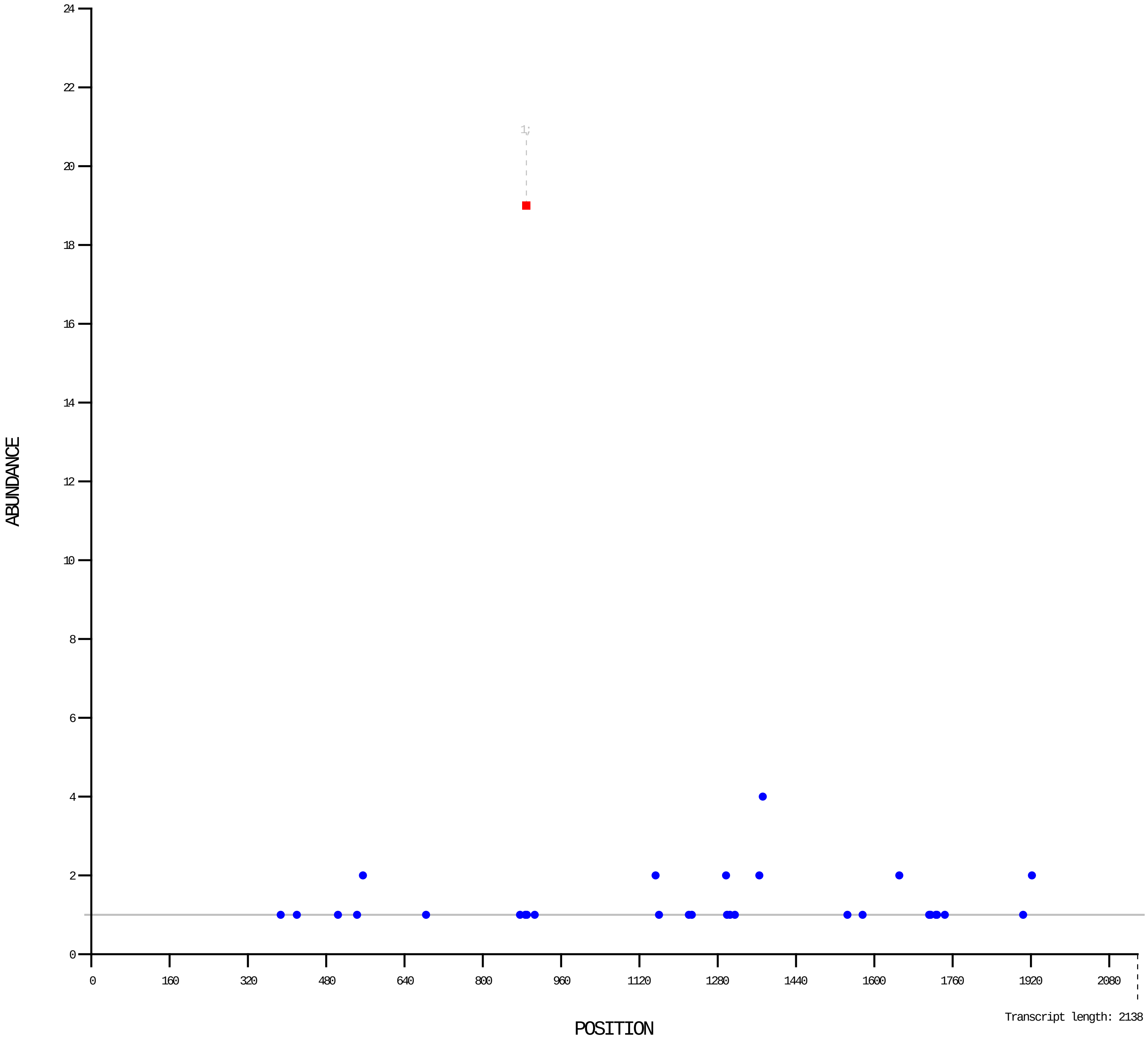

Category: 0 1 2 3 4
Degradome alignment: Median:

0 #1 Position:889 Abundance: 19.00(deg) 1(sRNA)

```
5'     TCATTGAGTGCAGCGTTGATG        3'  ID:
       | |||||||||||o|||||              Score: 1.5
3' GACCAATAACTCACGTCGTAACTACTATGCAT 5'  p-value: 0.0
```

Cs3g12340.1 gene=Cs3g12340 CDS=1-4731

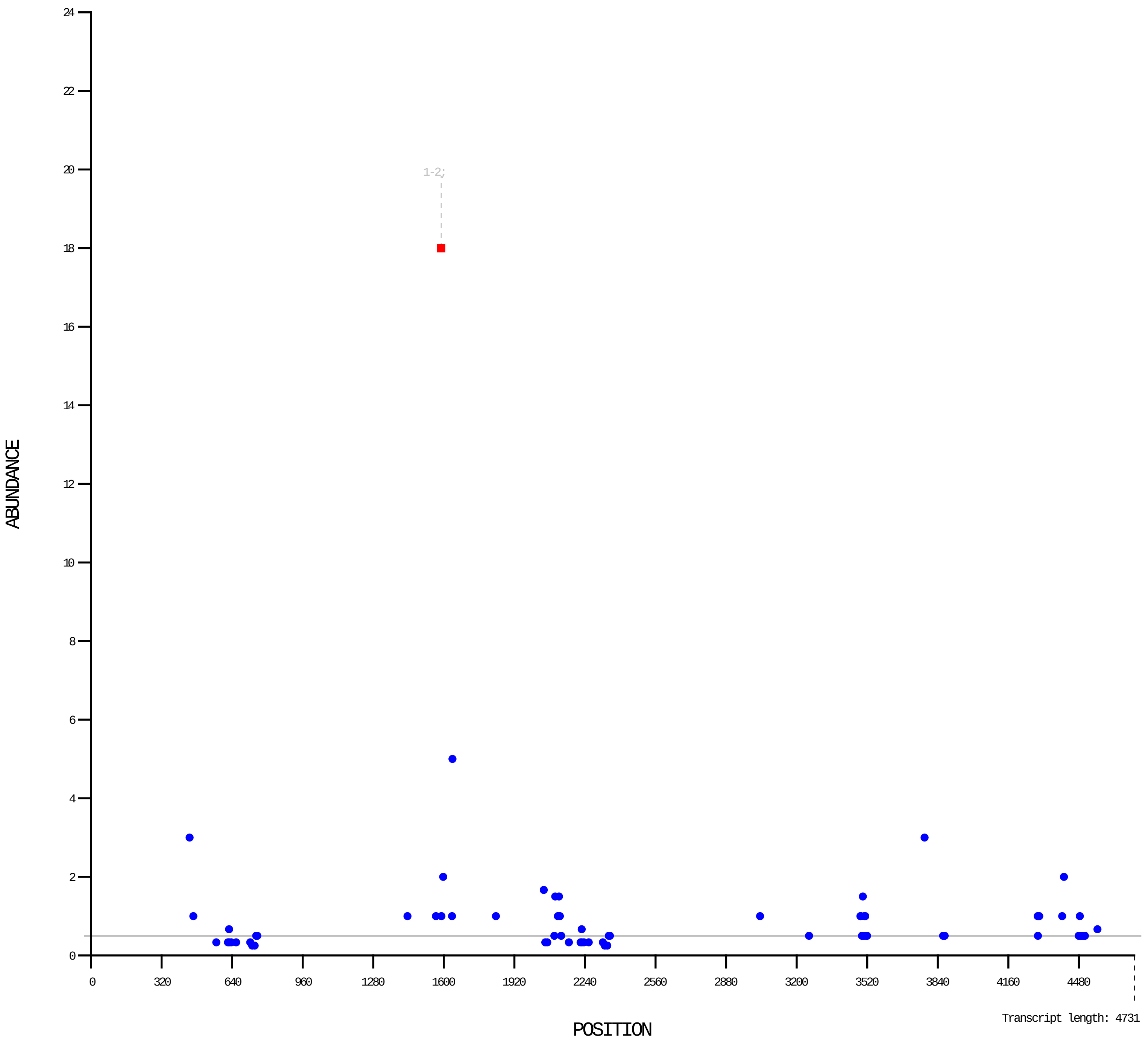

Category: ■ 0 ■ 1 ■ 2 ■ 3 ■ 4
Degradome alignment: ● Median: —

■ 0 #1 Position:1588 Abundance: 18.00(deg) 1(sRNA)
```
5'     TCTTACCTATGCCACCCATTCC       3'  ID:
       |||| || |||||||||||o||           Score: 2.5
3' CACCAGAAAGGTTACGGTGGGTAGGGCATGTG 5'  p-value: 0.0
```

■ 0 #2 Position:1588 Abundance: 18.00(deg) 1(sRNA)
```
5'     TCTTCCCTATGCCTCCCATTCC       3'  ID:
       |||| || ||||| |||||o||           Score: 3.5
3' CACCAGAAAGGTTACGGTGGGTAGGGCATGTG 5'  p-value: 0.0
```

orange1.1t01918.1 gene=orange1.1t01918 CDS=1-2835

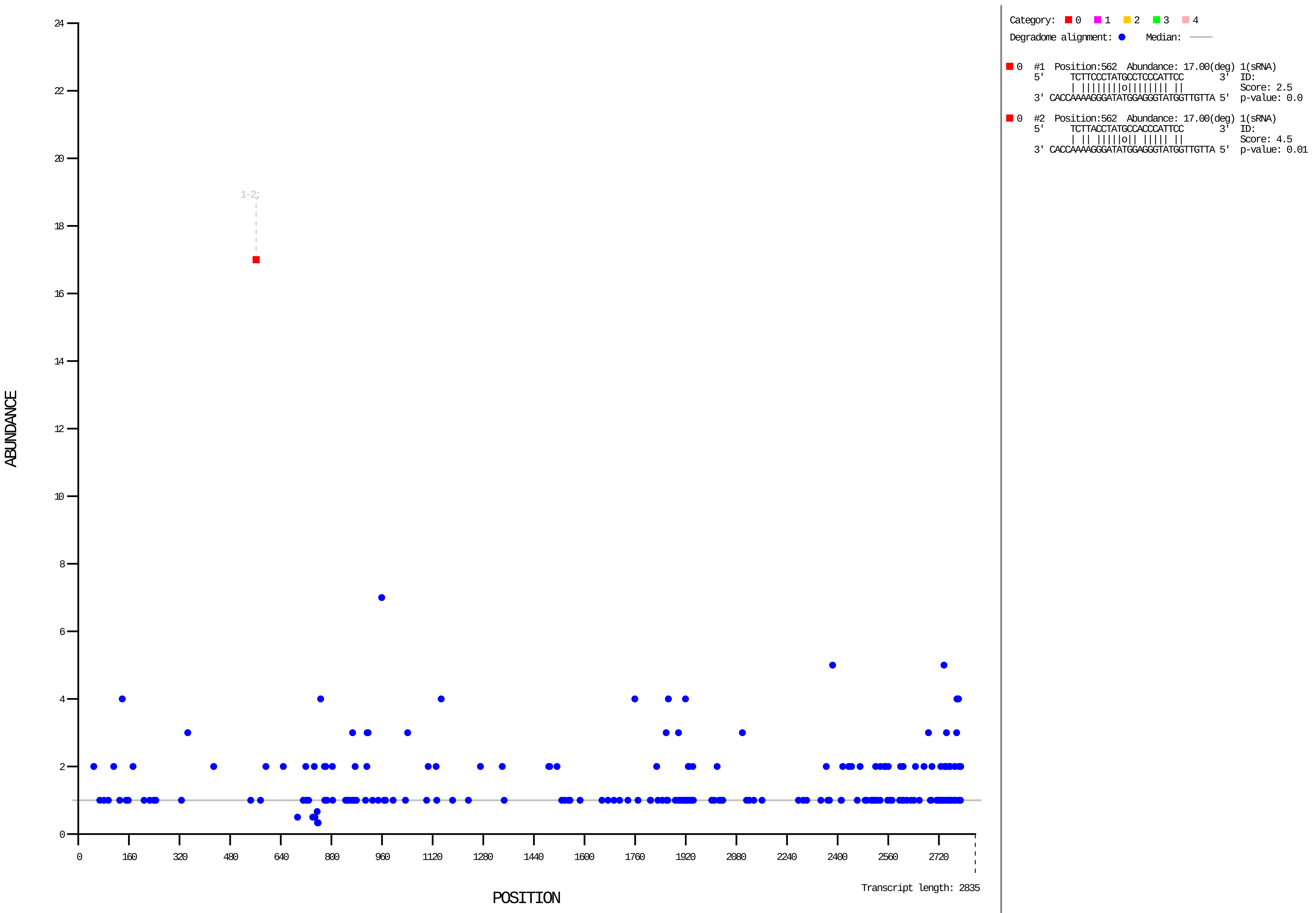

orange1.1t03707.1 gene=orange1.1t03707 CDS=1-2796

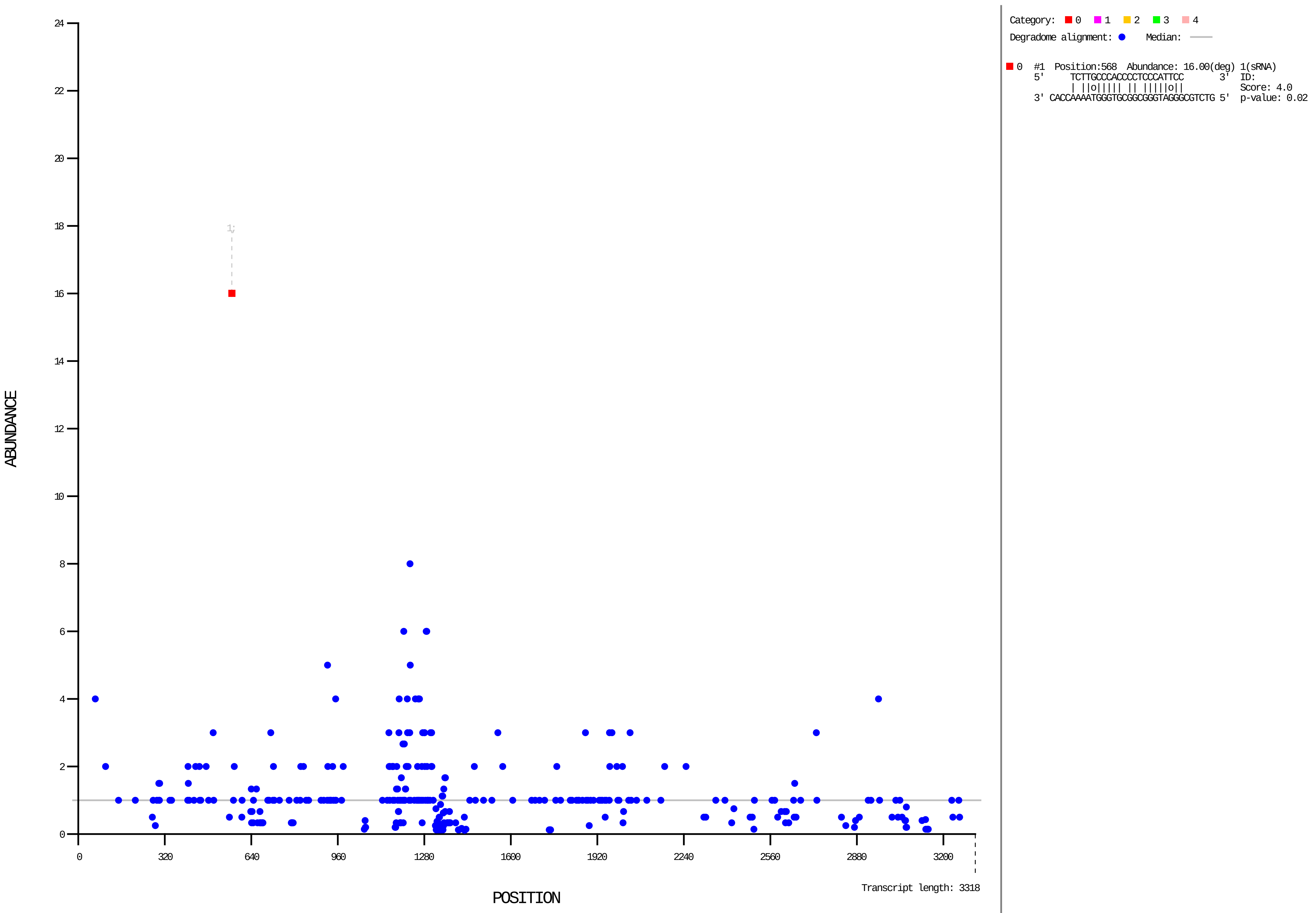

Cs2g09440.2 gene=Cs2g09440 CDS=826-3525

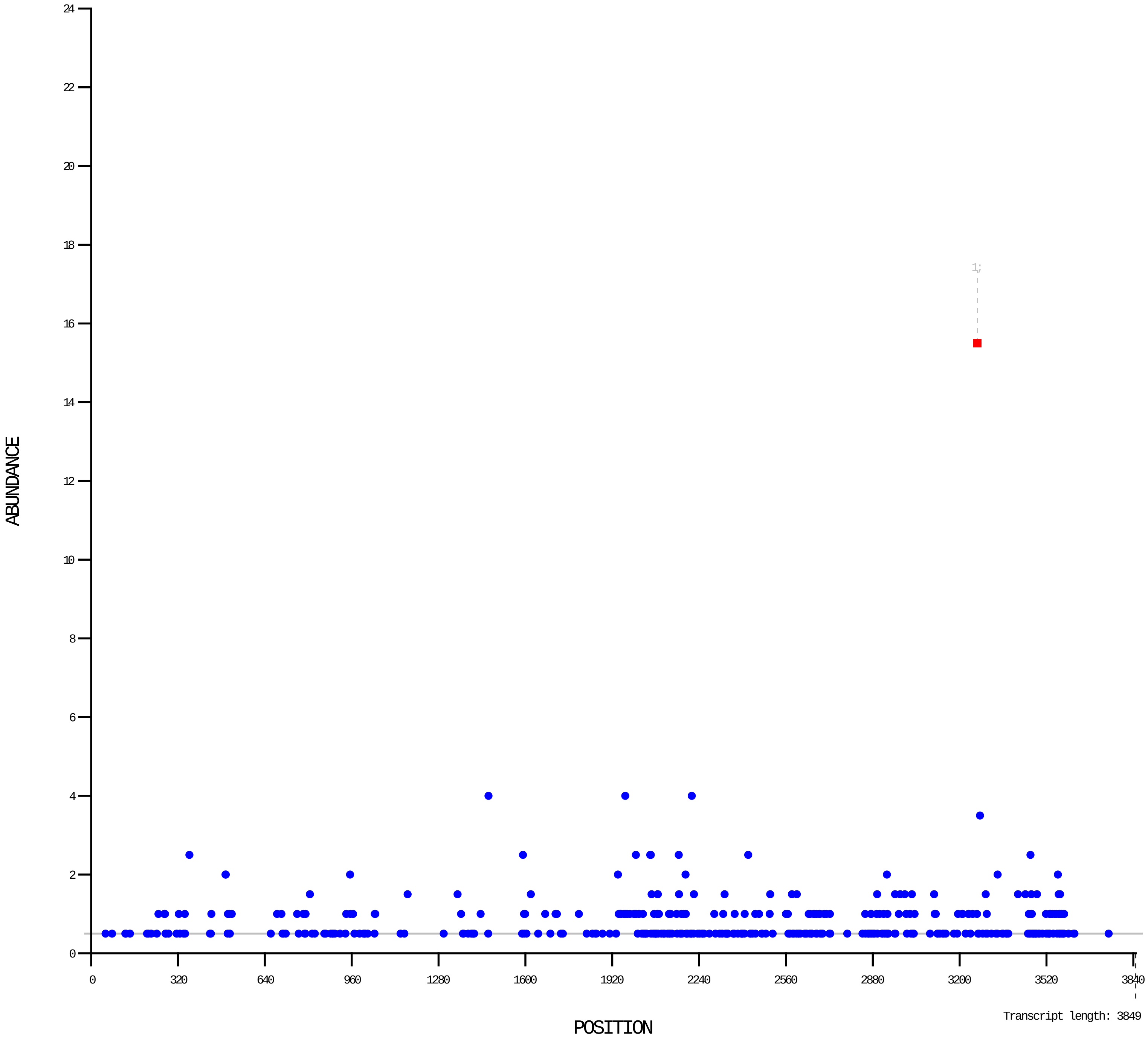

Category: 0 1 2 3 4
Degradome alignment: Median:

0 #1 Position:3266 Abundance: 15.50(deg) 1(sRNA)

```
5'     TGAAGCTGCCAGCATGATCTT        3'  ID:
          |||||||||| ||||||o             Score: 3.5
3' CTTATGTTCGACGGTCGGACTAGAGTCCCCCA 5'  p-value: 0.02
```

Cs2g09440.1 gene=Cs2g09440 CDS=826-3516

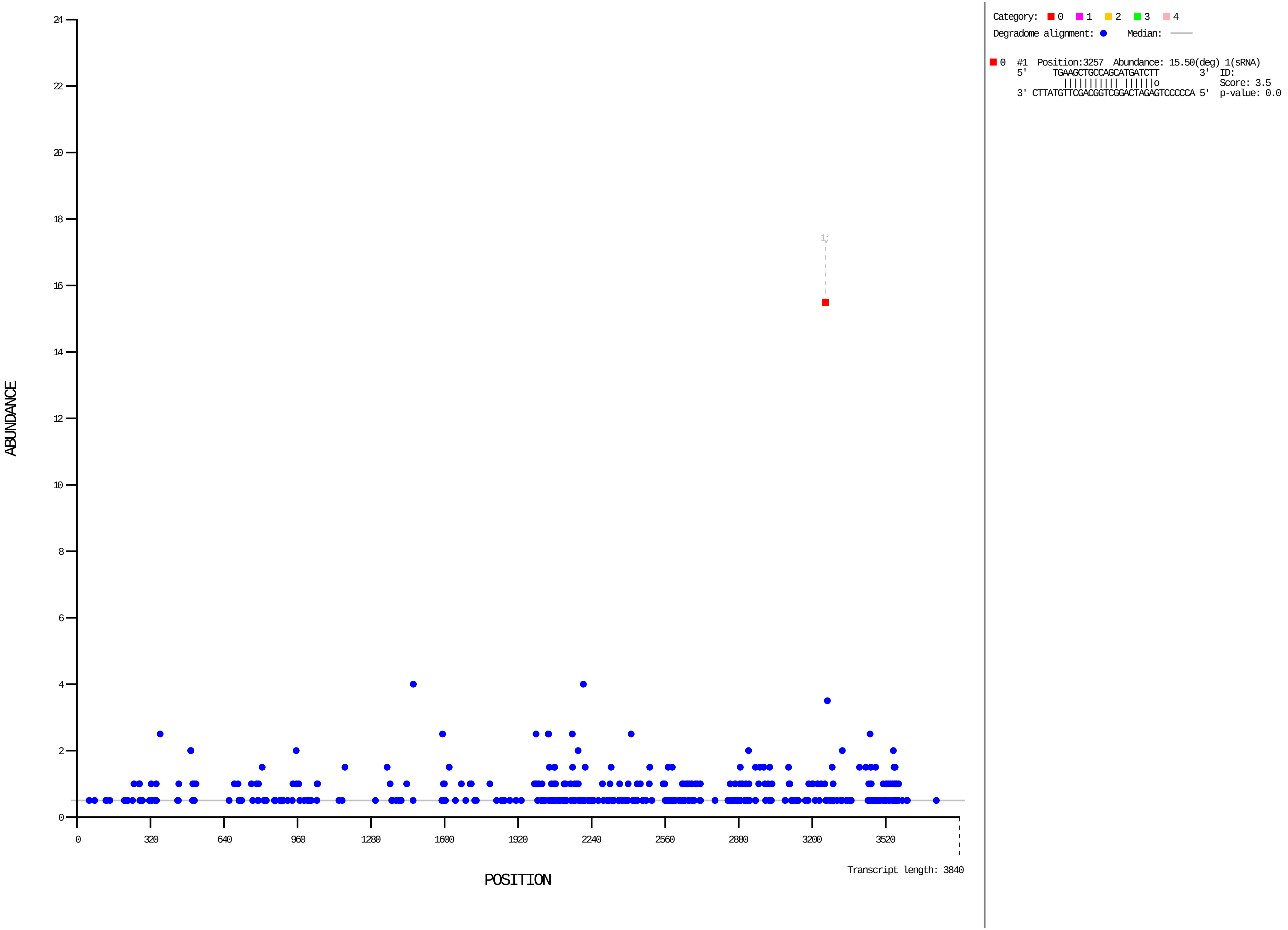

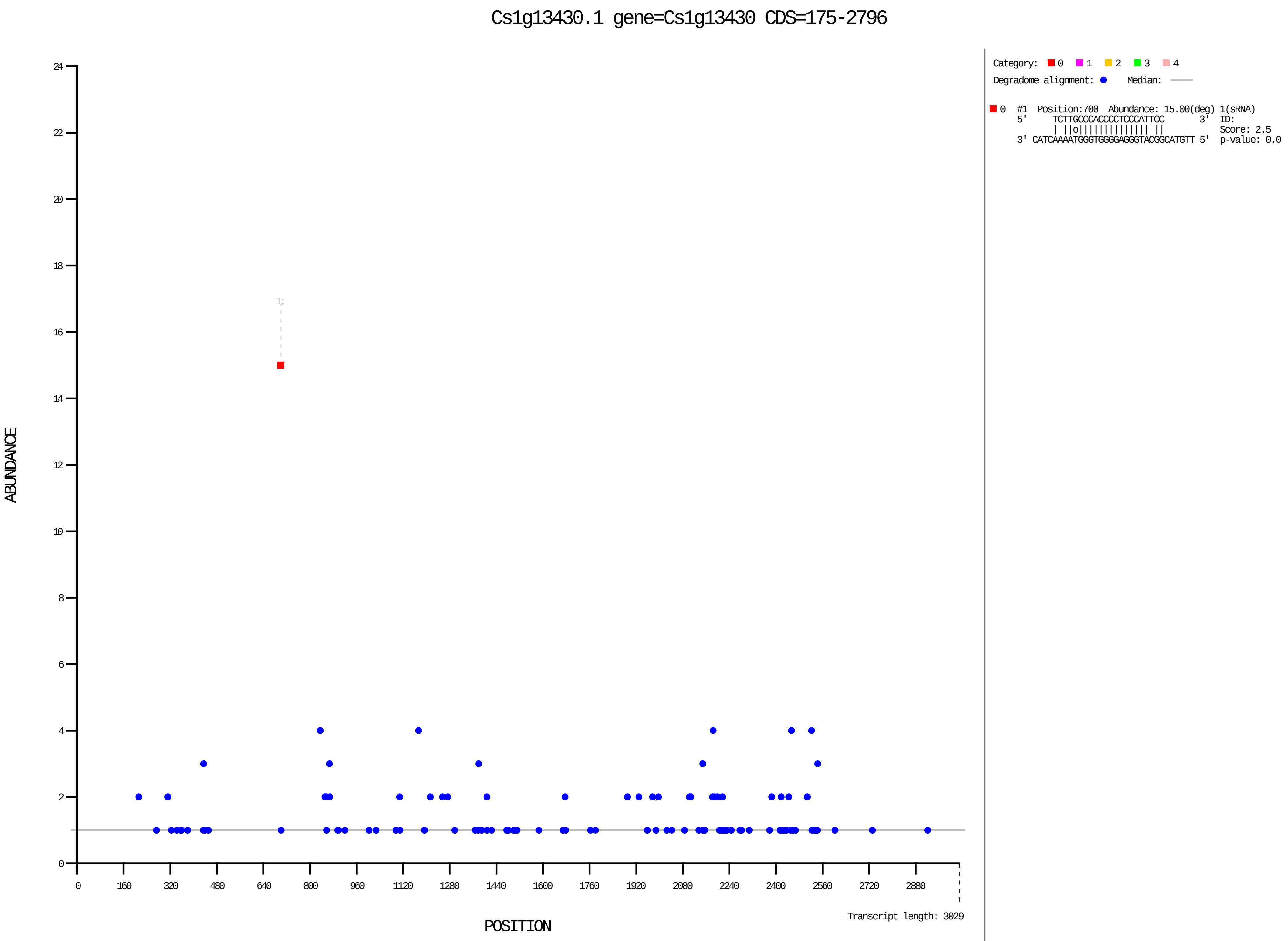

Cs1g13430.1 gene=Cs1g13430 CDS=175-2796
ABUNDANCE
POSITION
Transcript length: 3029
Category: 0 1 2 3 4
Degradome alignment:
Median:
0 #1 Position:700 Abundance: 15.00(deg) 1(sRNA)
5' TCTTGCCCACCCCTCCCATTCC 3' ID:
| ||o||||||||||||| ||
Score: 2.5
3' CATCAAAATGGGTGGGGAGGGTACGGCATGTT 5' p-value: 0.0

orange1.1t03734.1 gene=orange1.1t03734 CDS=197-2974

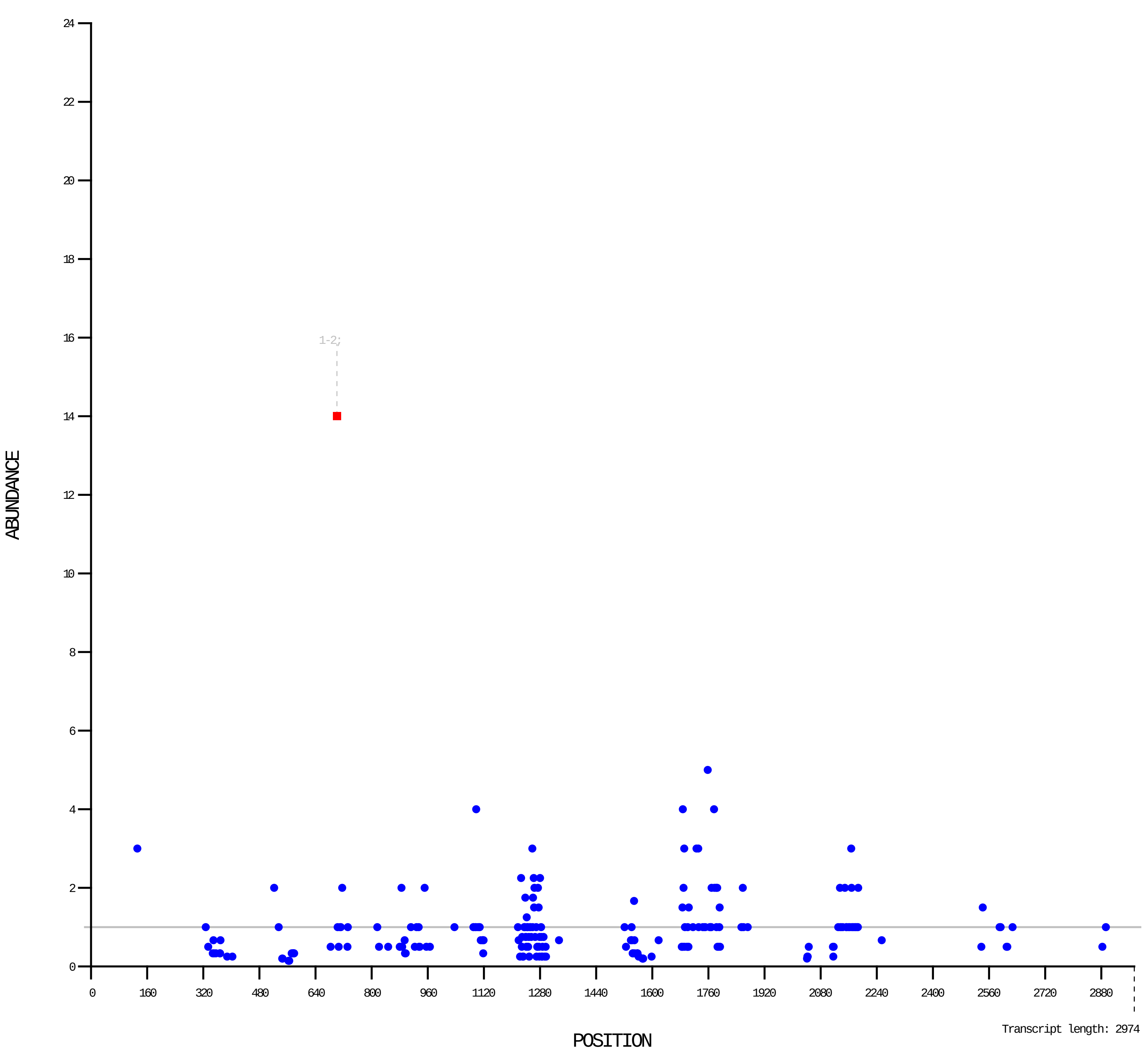

0 #1 Position:701 Abundance: 14.00(deg) 1(sRNA)
```
5'     TCTTCCCTATGCCTCCCATTCC       3'  ID:
       | |||||||||||||||||  ||          Score: 2.0
3' CATCAAAAGGGATACGGAGGGTATGGTTGTTA 5'  p-value: 0.0
```

0 #2 Position:701 Abundance: 14.00(deg) 1(sRNA)
```
5'     TCTTACCTATGCCACCCATTCC       3'  ID:
       | || |||||||| |||||  ||          Score: 4.0
3' CATCAAAAGGGATACGGAGGGTATGGTTGTTA 5'  p-value: 0.0
```

Cs3g16860.1 gene=Cs3g16860 CDS=44-1603

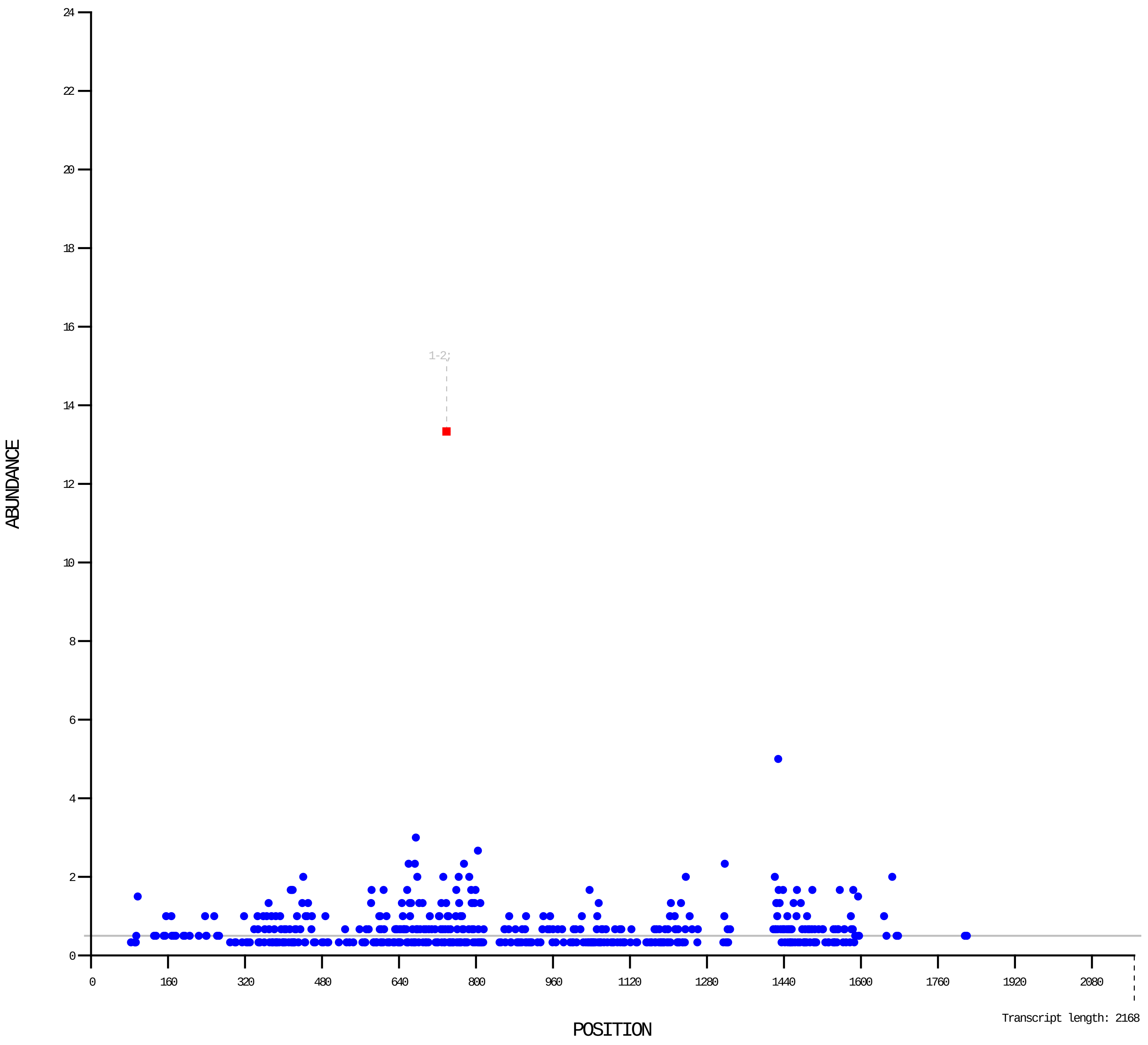

Category: 0 1 2 3 4
Degradome alignment: Median:

```
0  #1  Position:739  Abundance: 13.33(deg) 1(sRNA)
   5'     TGCATTTGCACCTGCATCT-TG       3'  ID:
           |||o||||||||||| || ||           Score: 3.5
   3' GTATTCGTGAACGTGGACGTTGATACTCAAAA 5'  p-value: 0.01

0  #2  Position:739  Abundance: 13.33(deg) 1(sRNA)
   5'     TGCATTTGCACCTGCACCT-TG       3'  ID:
           |||o||||||||||| || ||           Score: 3.5
   3' GTATTCGTGAACGTGGACGTTGATACTCAAAA 5'  p-value: 0.0
```

Cs3g16860.2 gene=Cs3g16860 CDS=44-1603

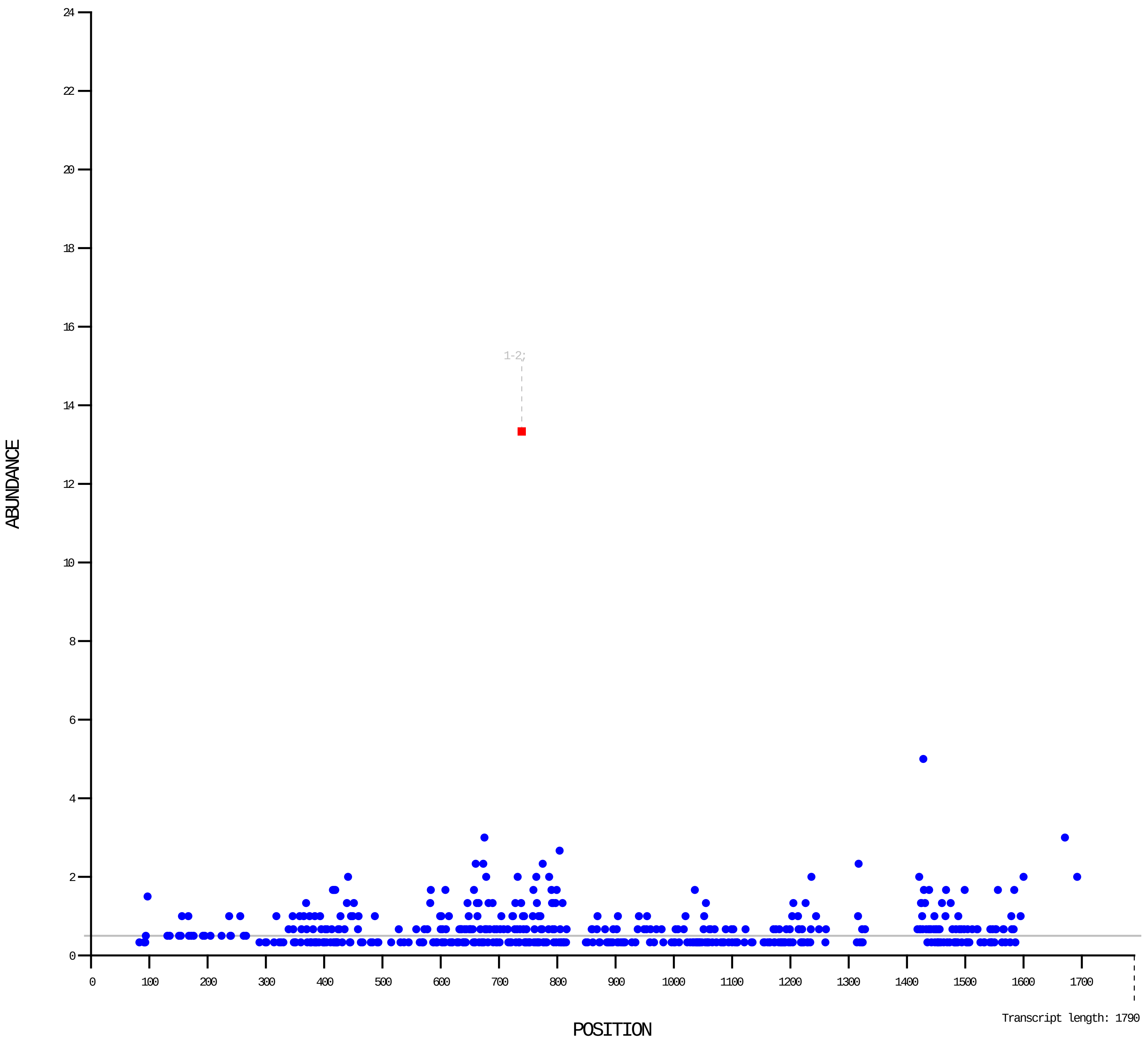

Category: 0 1 2 3 4
Degradome alignment: Median:

0 #1 Position:739 Abundance: 13.33(deg) 1(sRNA)
5' TGCATTTGCACCTGCATCT-TG 3' ID:
|||o||||||||||| || || Score: 3.5
3' GTATTCGTGAACGTGGACGTTGATACTCAAAA 5' p-value: 0.0

0 #2 Position:739 Abundance: 13.33(deg) 1(sRNA)
5' TGCATTTGCACCTGCACCT-TG 3' ID:
|||o||||||||||| || || Score: 3.5
3' GTATTCGTGAACGTGGACGTTGATACTCAAAA 5' p-value: 0.0

Cs3g16860.3 gene=Cs3g16860 CDS=44-1468

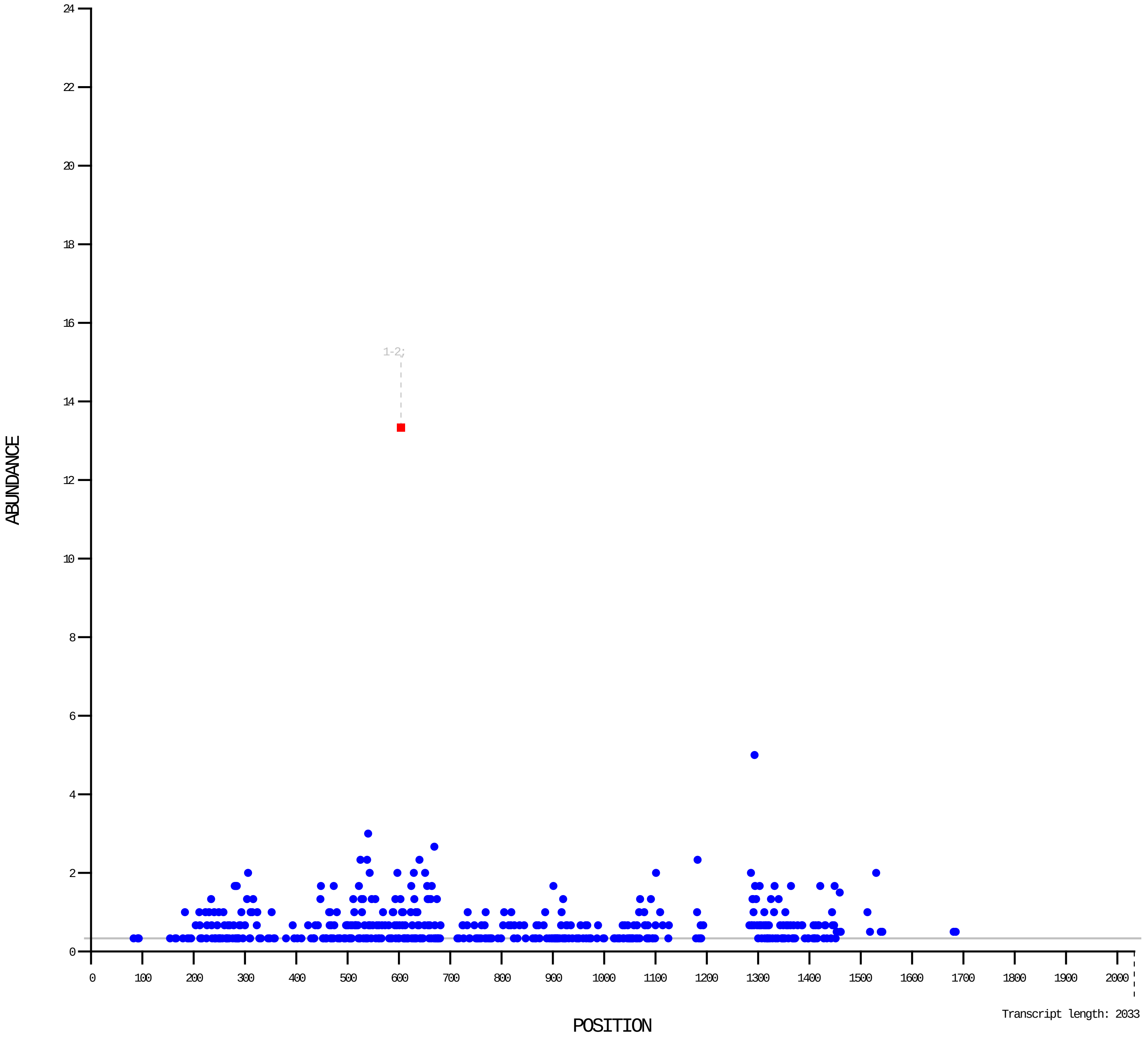

Category: ■ 0 ■ 1 ■ 2 ■ 3 ■ 4
Degradome alignment: ● Median: —

■ 0 #1 Position:604 Abundance: 13.33(deg) 1(sRNA)

```
5'     TGCATTTGCACCTGCATCT-TG      3'  ID:
        |||o||||||||||| || ||          Score: 3.5
3' GTATTCGTGAACGTGGACGTTGATACTCAAAA 5'  p-value: 0.02
```

■ 0 #2 Position:604 Abundance: 13.33(deg) 1(sRNA)

```
5'     TGCATTTGCACCTGCACCT-TG      3'  ID:
        |||o||||||||||| || ||          Score: 3.5
3' GTATTCGTGAACGTGGACGTTGATACTCAAAA 5'  p-value: 0.01
```

orange1.1t00584.2 gene=orange1.1t00584 CDS=1-5202

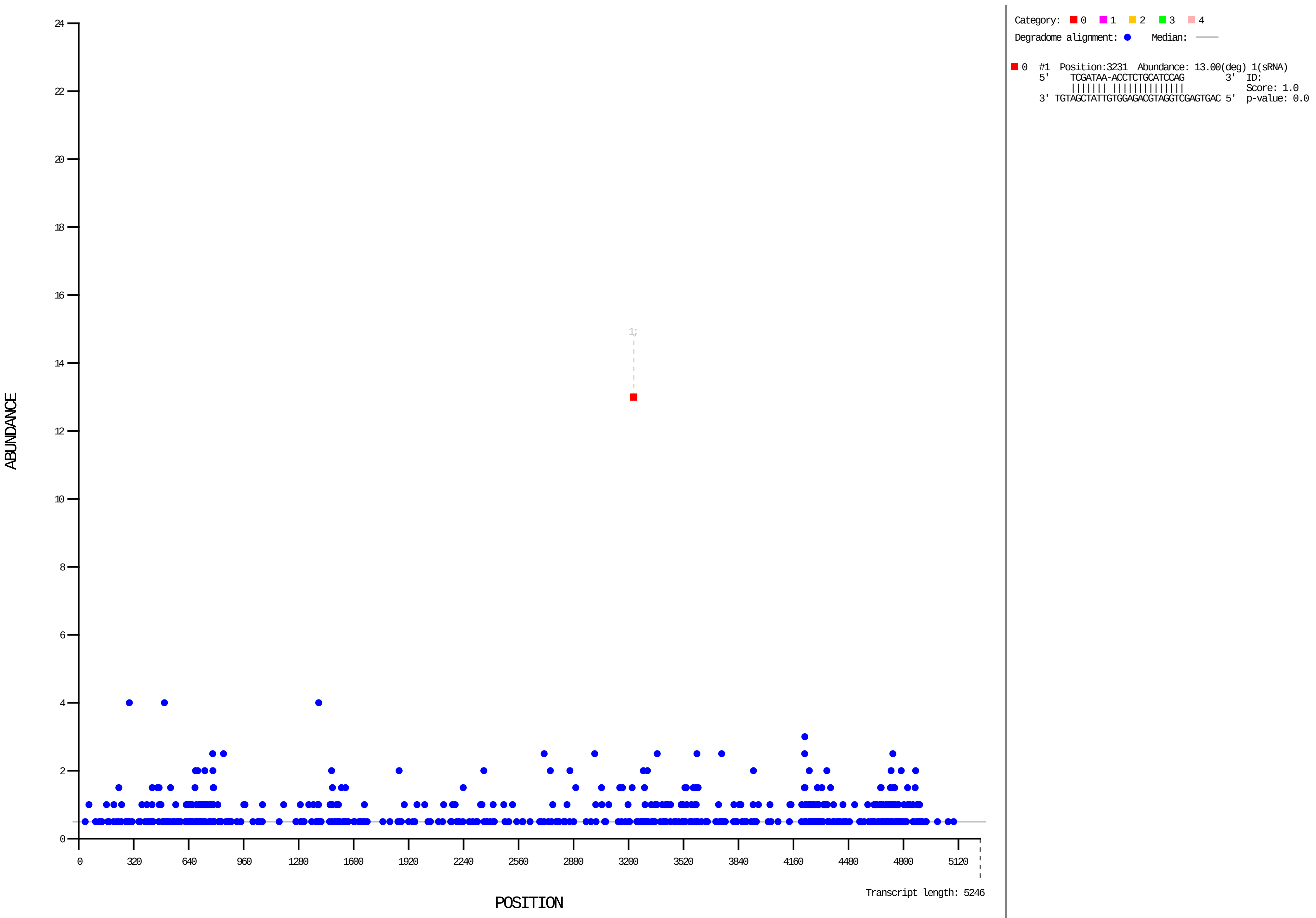

Cs6g16030.4 gene=Cs6g16030 CDS=495-2915

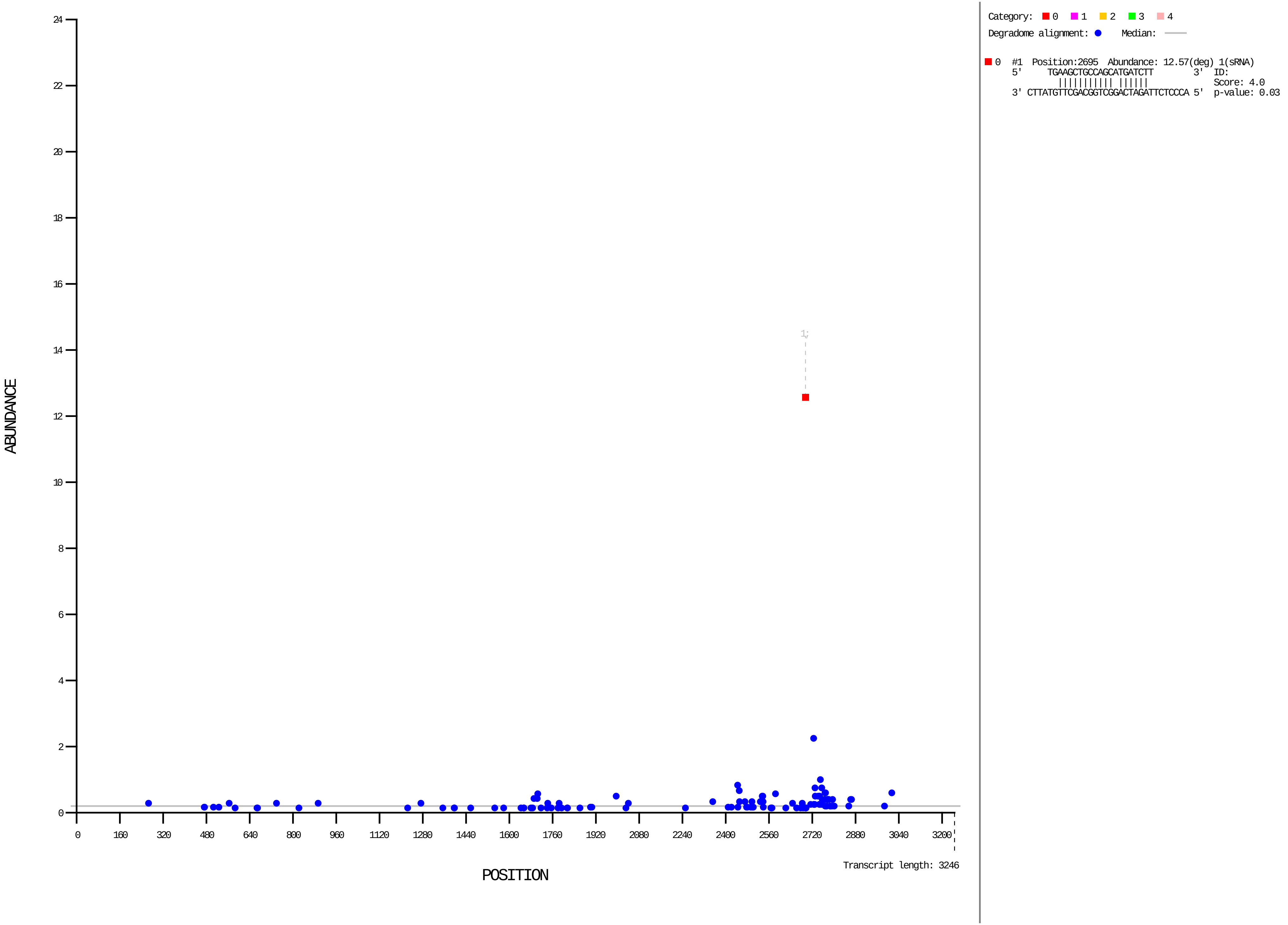

Cs6g16030.5 gene=Cs6g16030 CDS=1041-3518

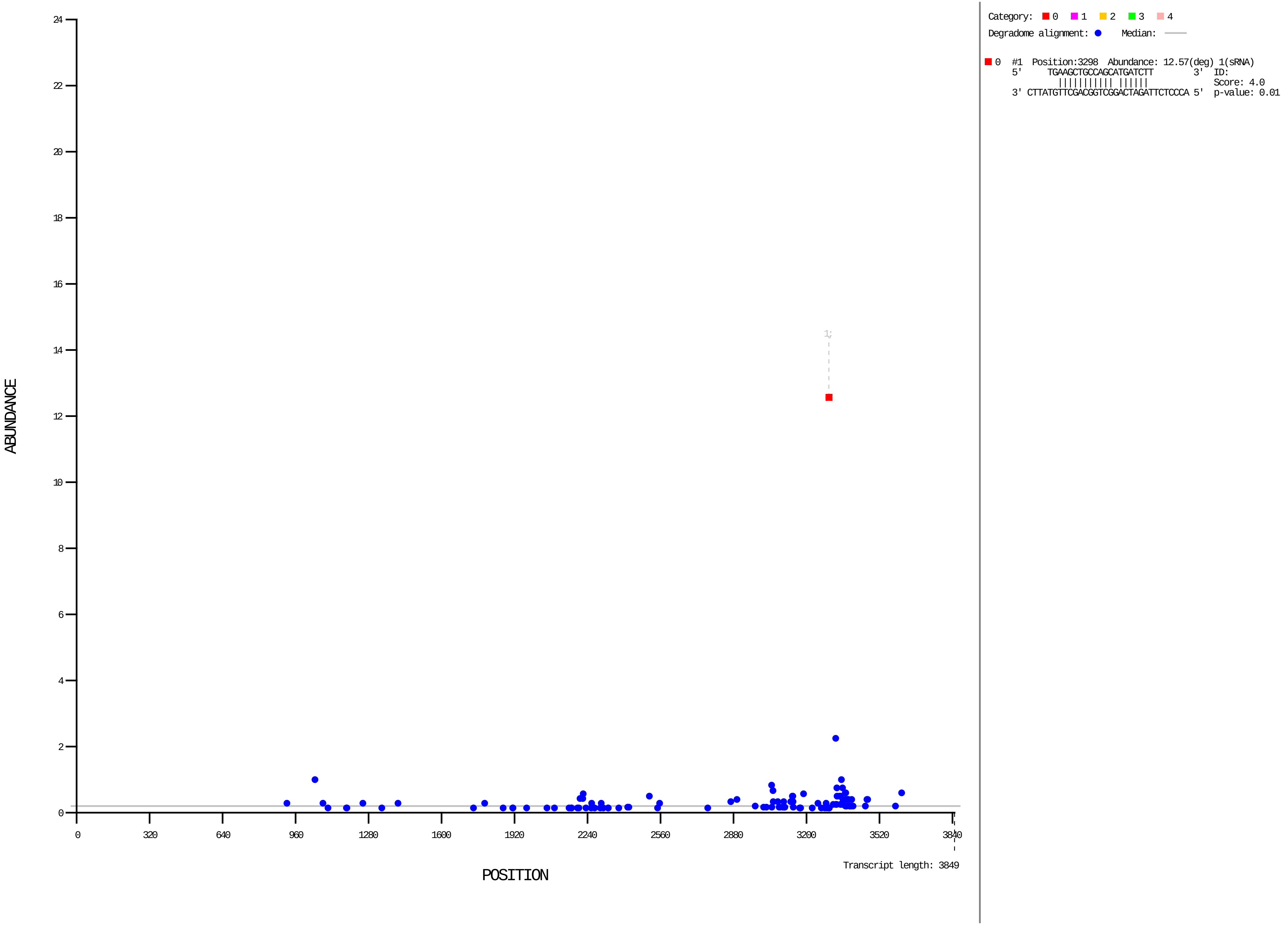

Cs6g16030.1 gene=Cs6g16030 CDS=1151-3463

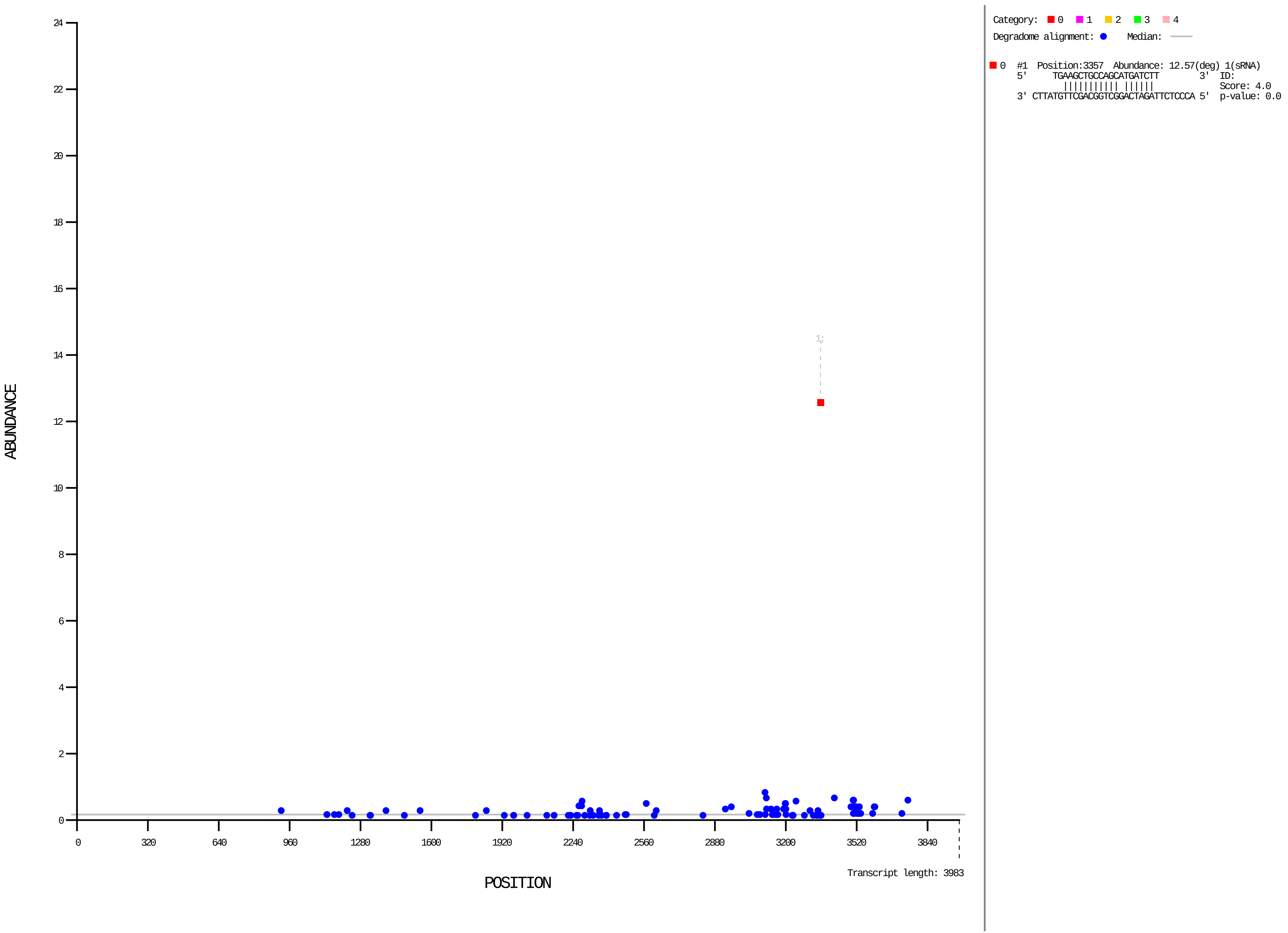

Cs6g16030.7 gene=Cs6g16030 CDS=495-2534

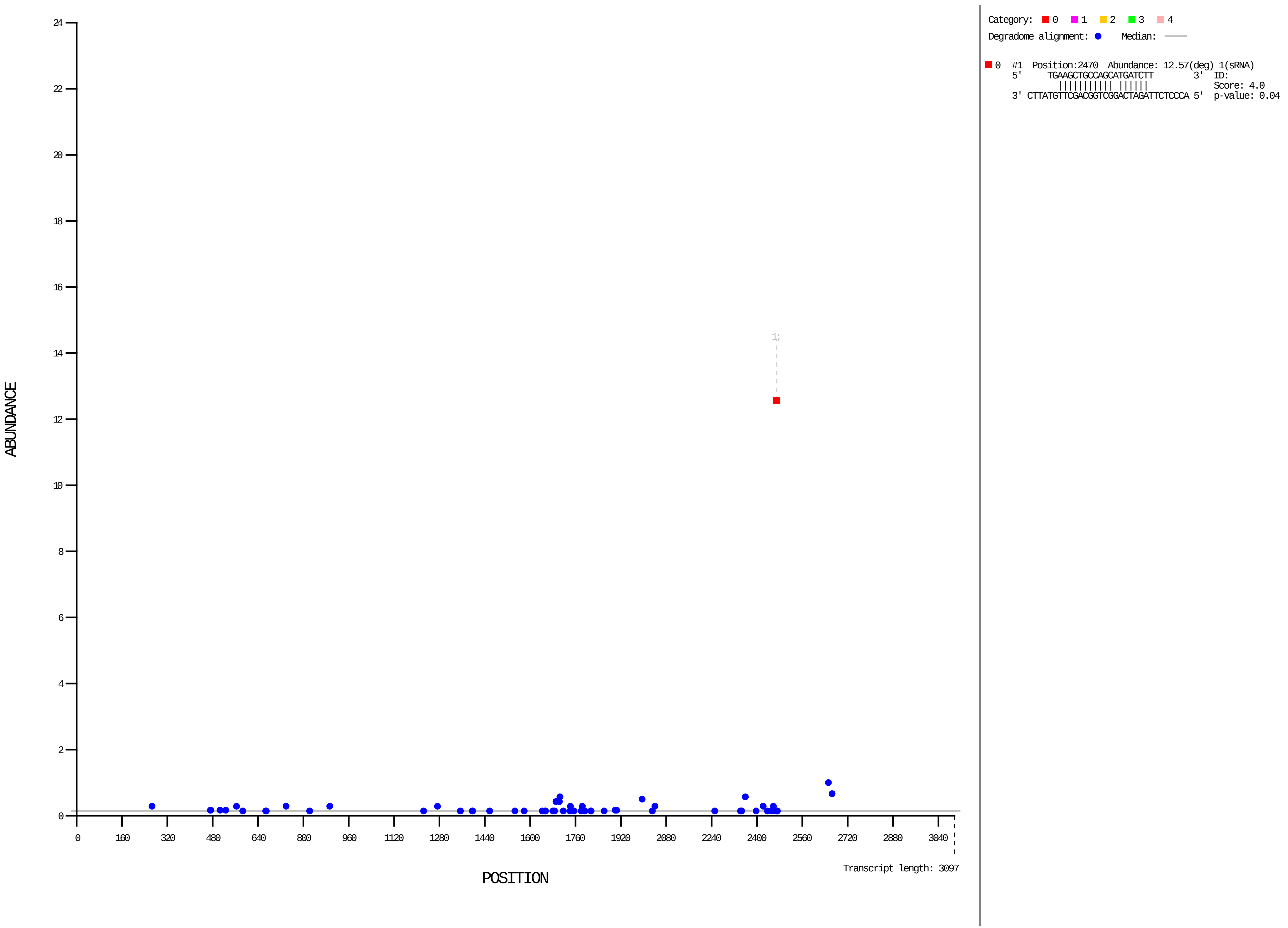

Cs6g16030.6 gene=Cs6g16030 CDS=495-3002

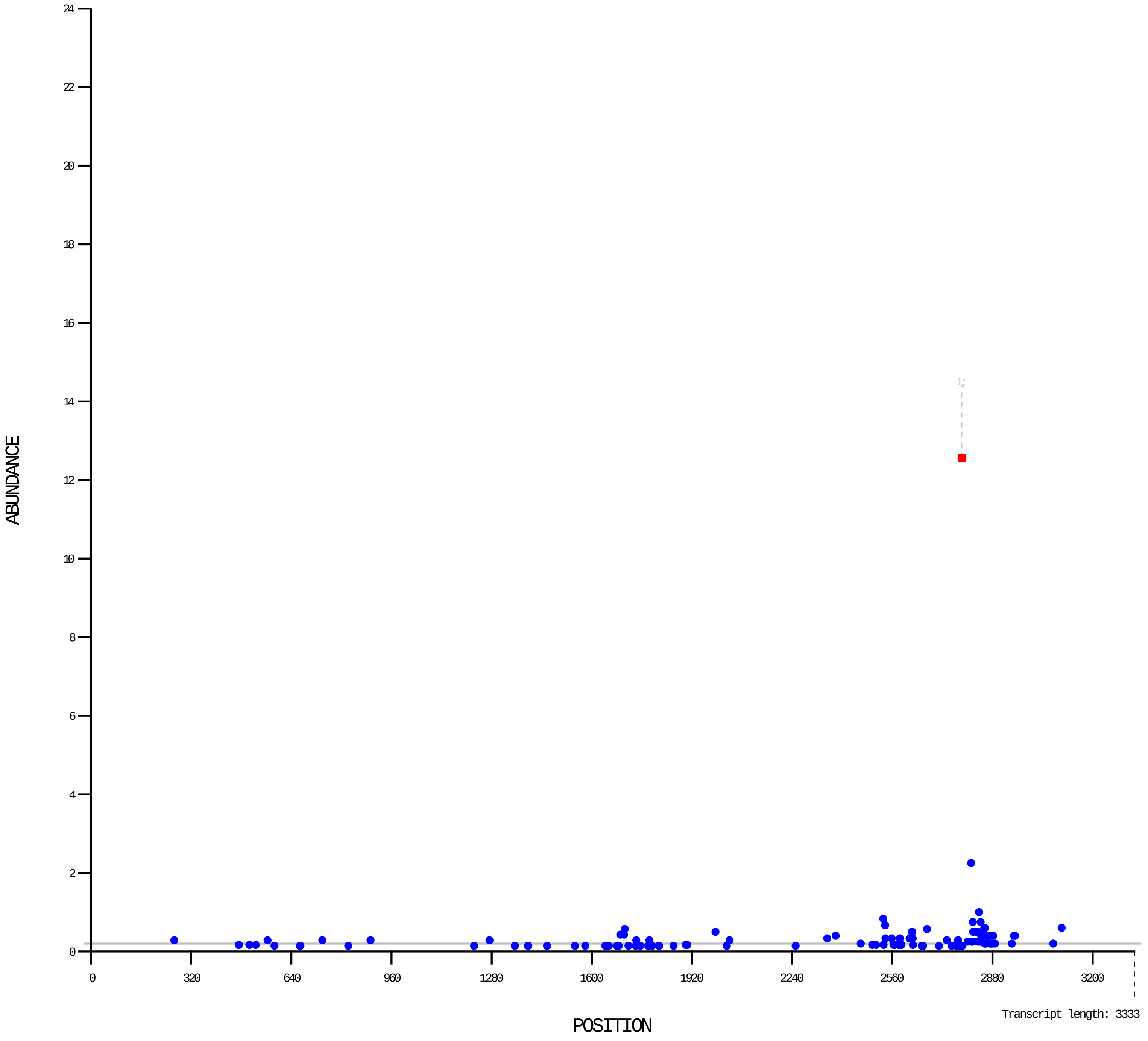

Category: 0 1 2 3 4
Degradome alignment: Median:

0 #1 Position:2782 Abundance: 12.57(deg) 1(sRNA)
5' TGAAGCTGCCAGCATGATCTT 3' ID:
|||||||||| ||||||| Score: 4.0
3' CTTATGTTCGACGGTCGGACTAGATTCTCCCA 5' p-value: 0.0

Cs6g16030.2 gene=Cs6g16030 CDS=495-2888

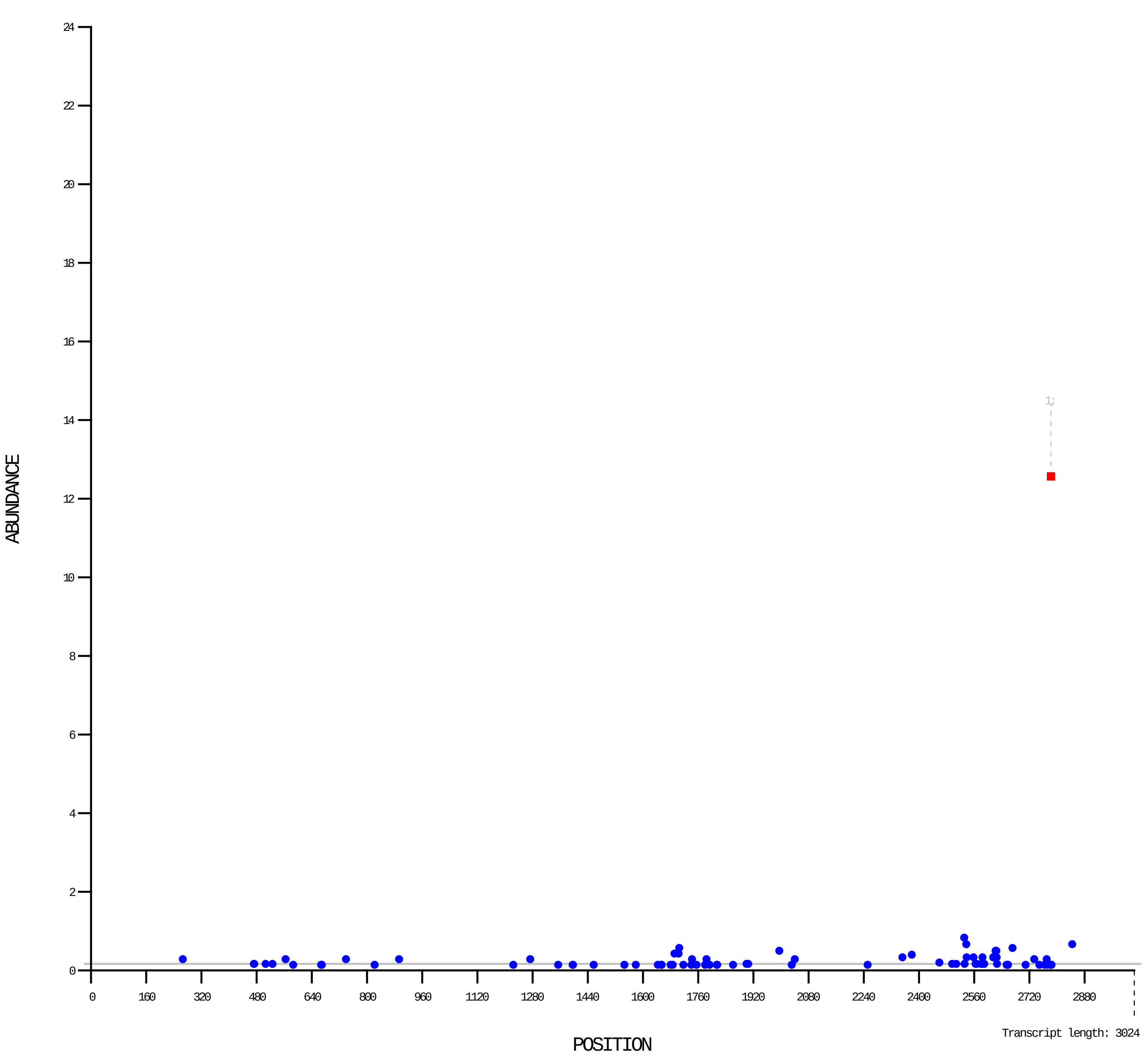

Cs6g16030.3 gene=Cs6g16030 CDS=495-2930

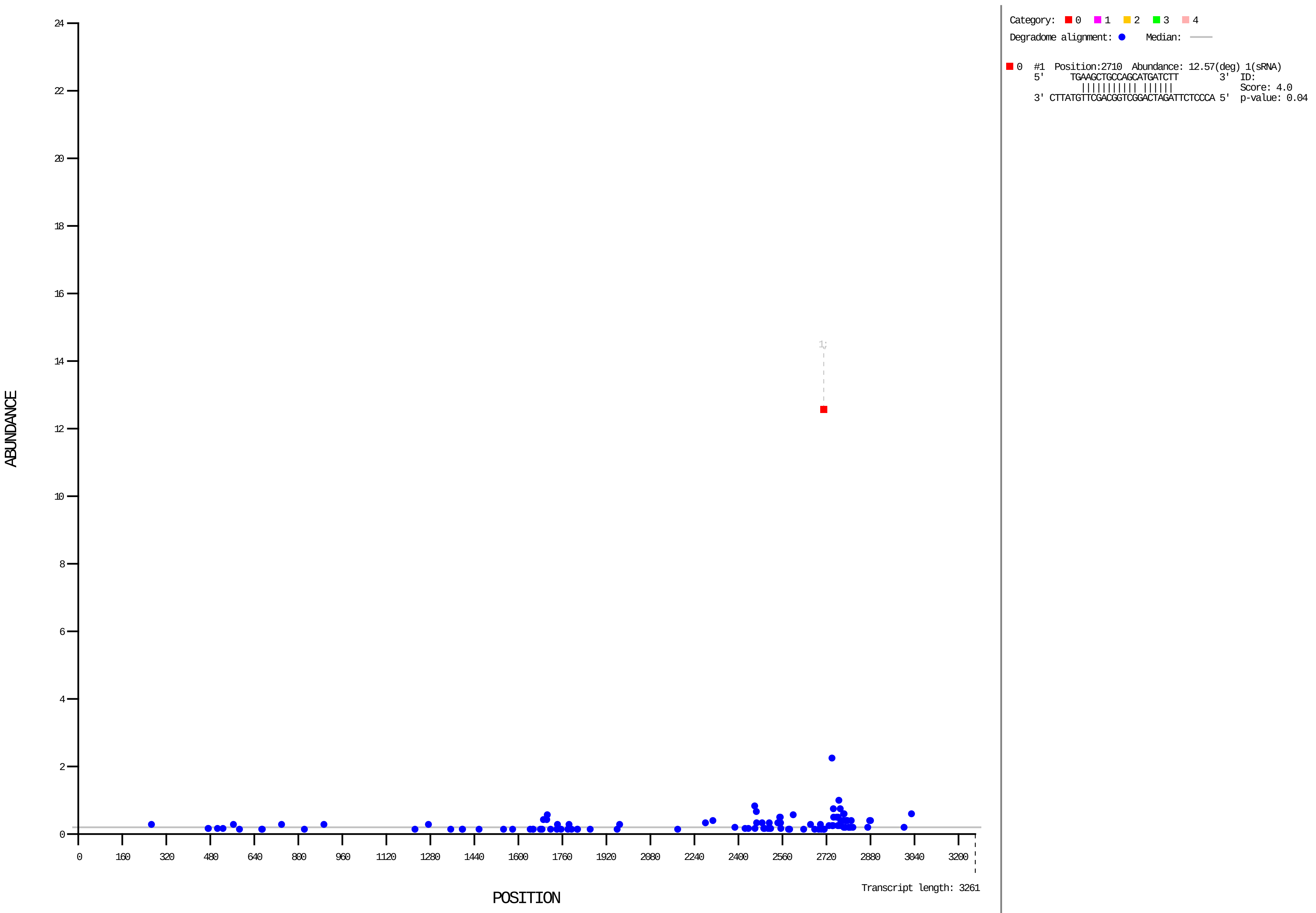

Cs7g10990.1 gene=Cs7g10990 CDS=851-1789

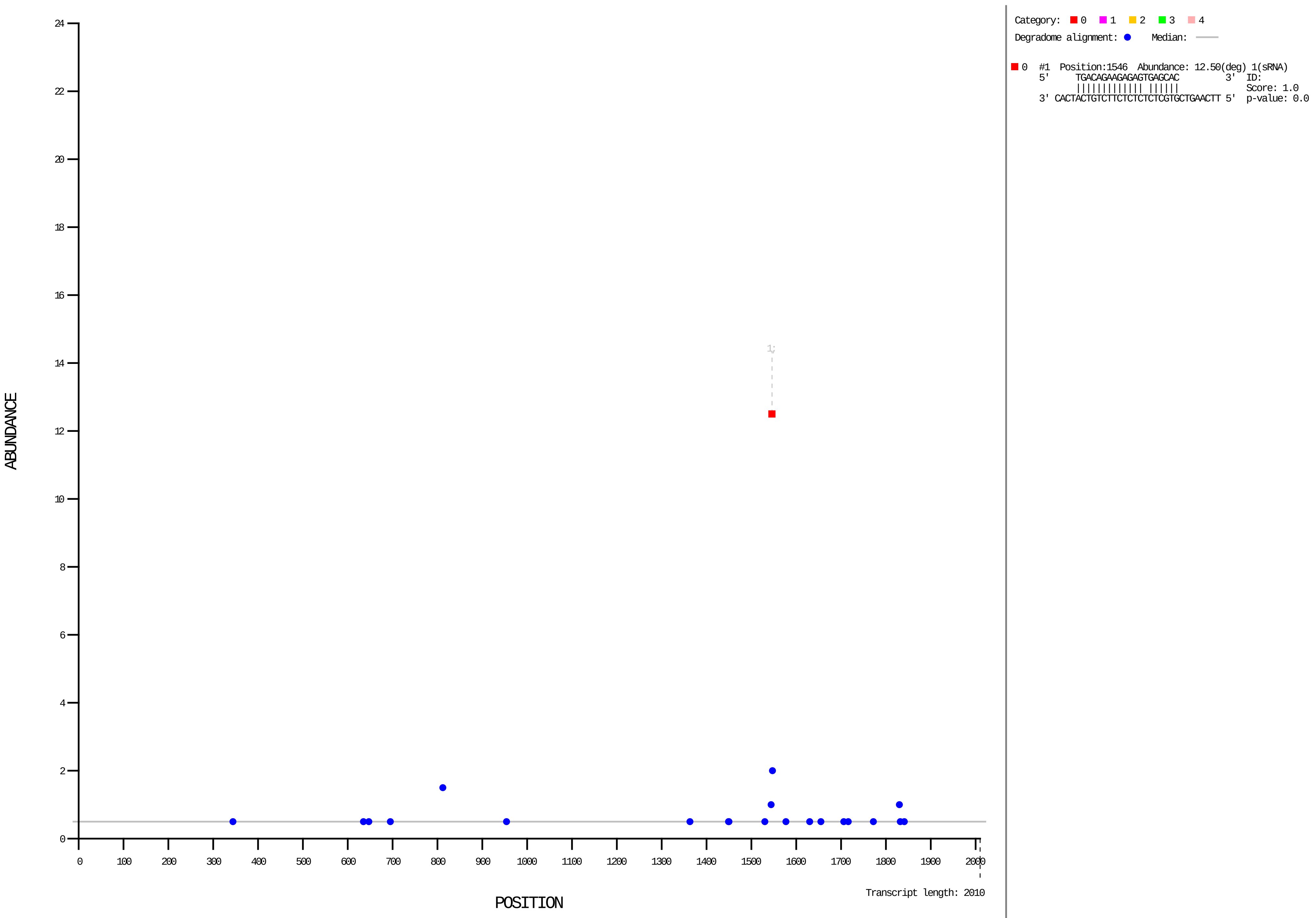

Cs7g10990.2 gene=Cs7g10990 CDS=1040-1978

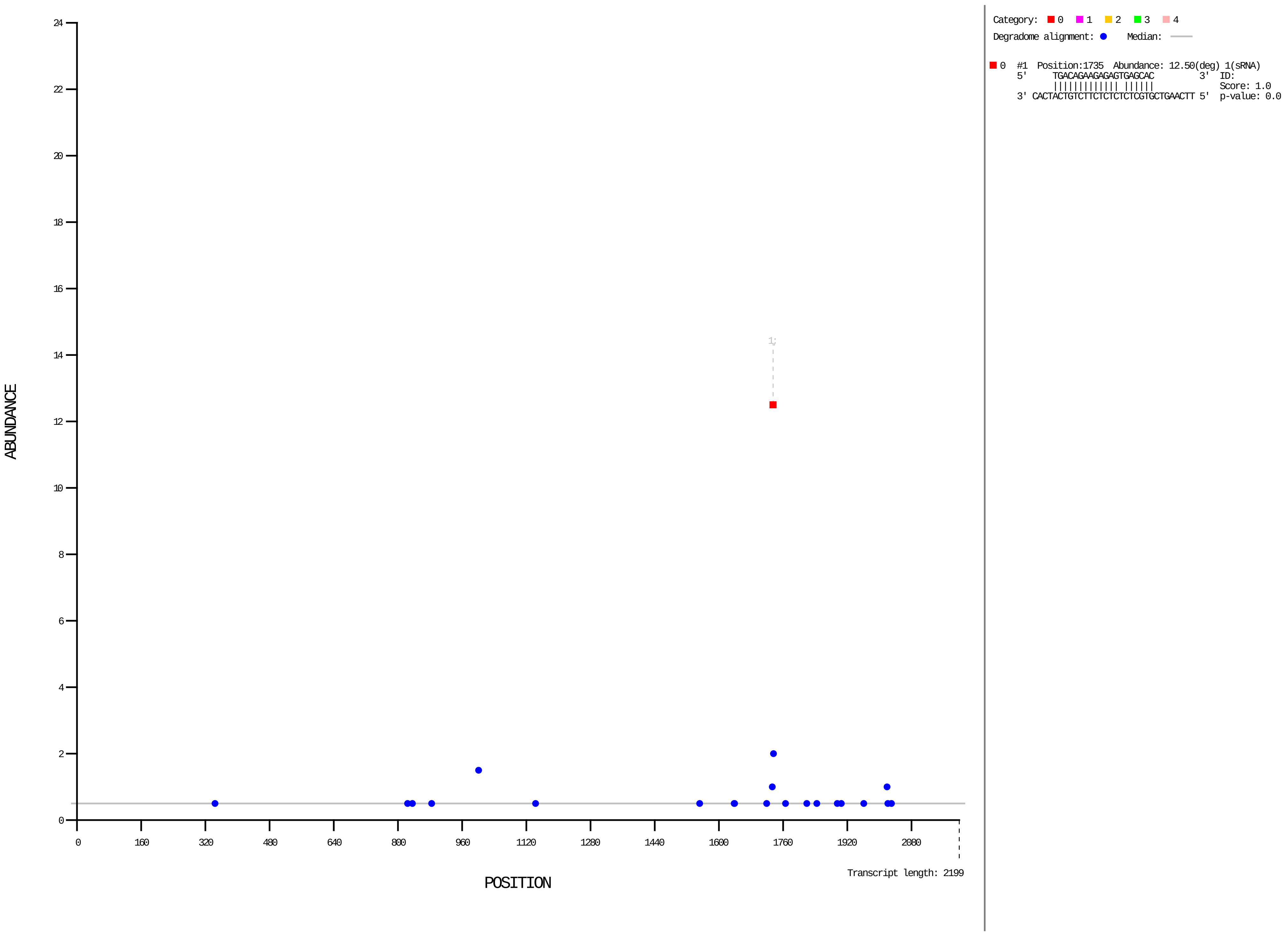

Cs6g18380.1 gene=Cs6g18380 CDS=1-2145

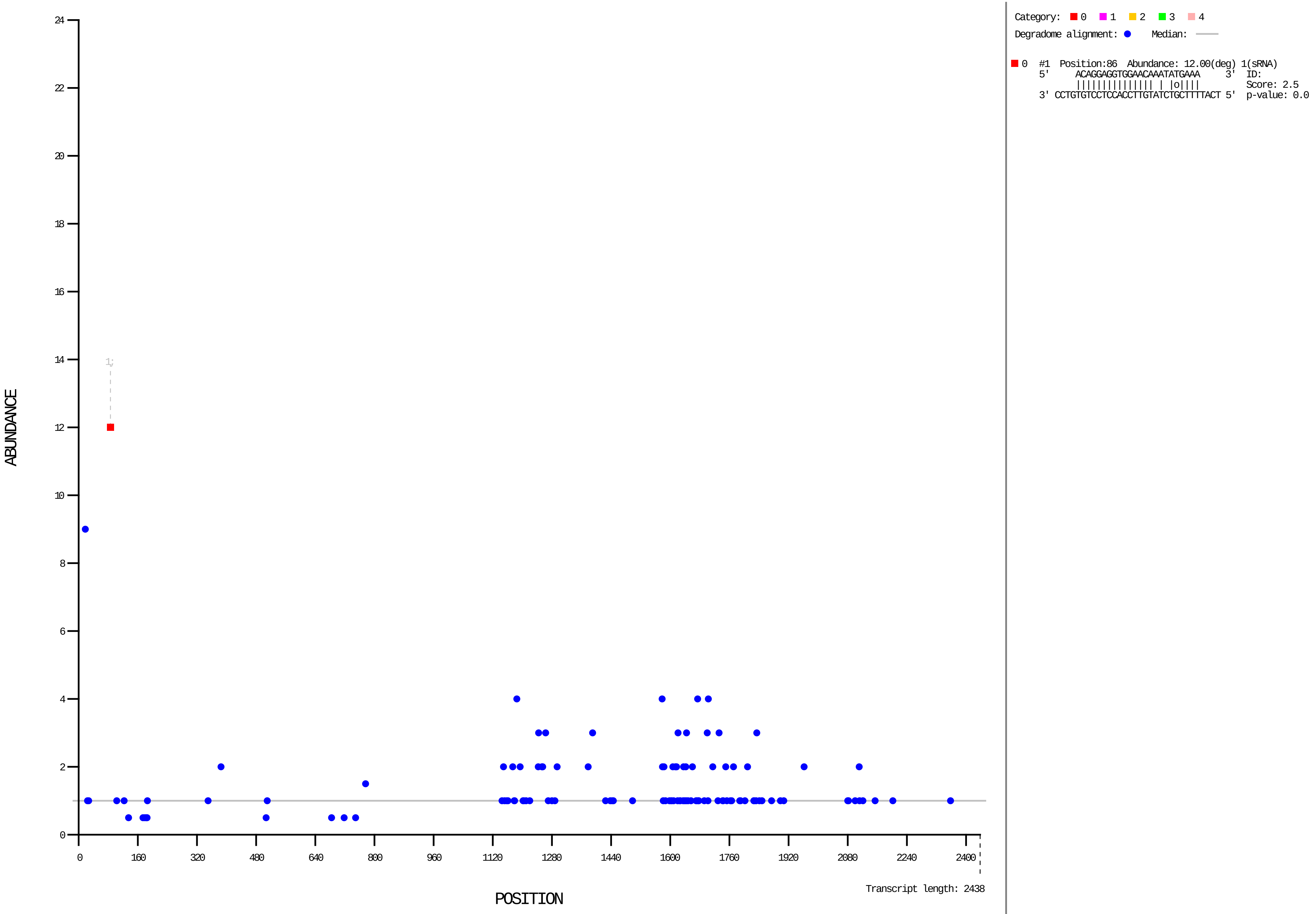

Cs1g21350.1 gene=Cs1g21350 CDS=350-2152

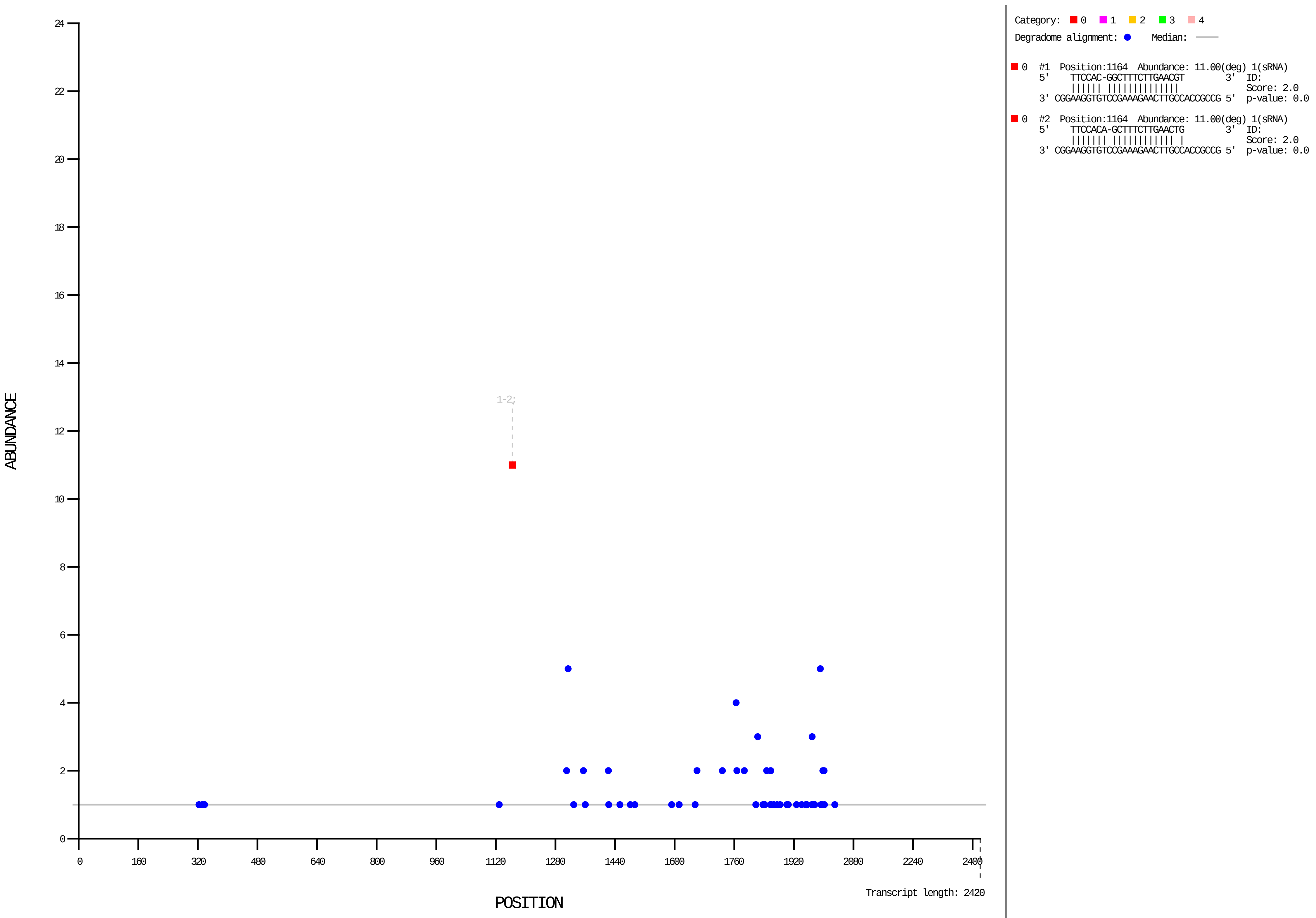

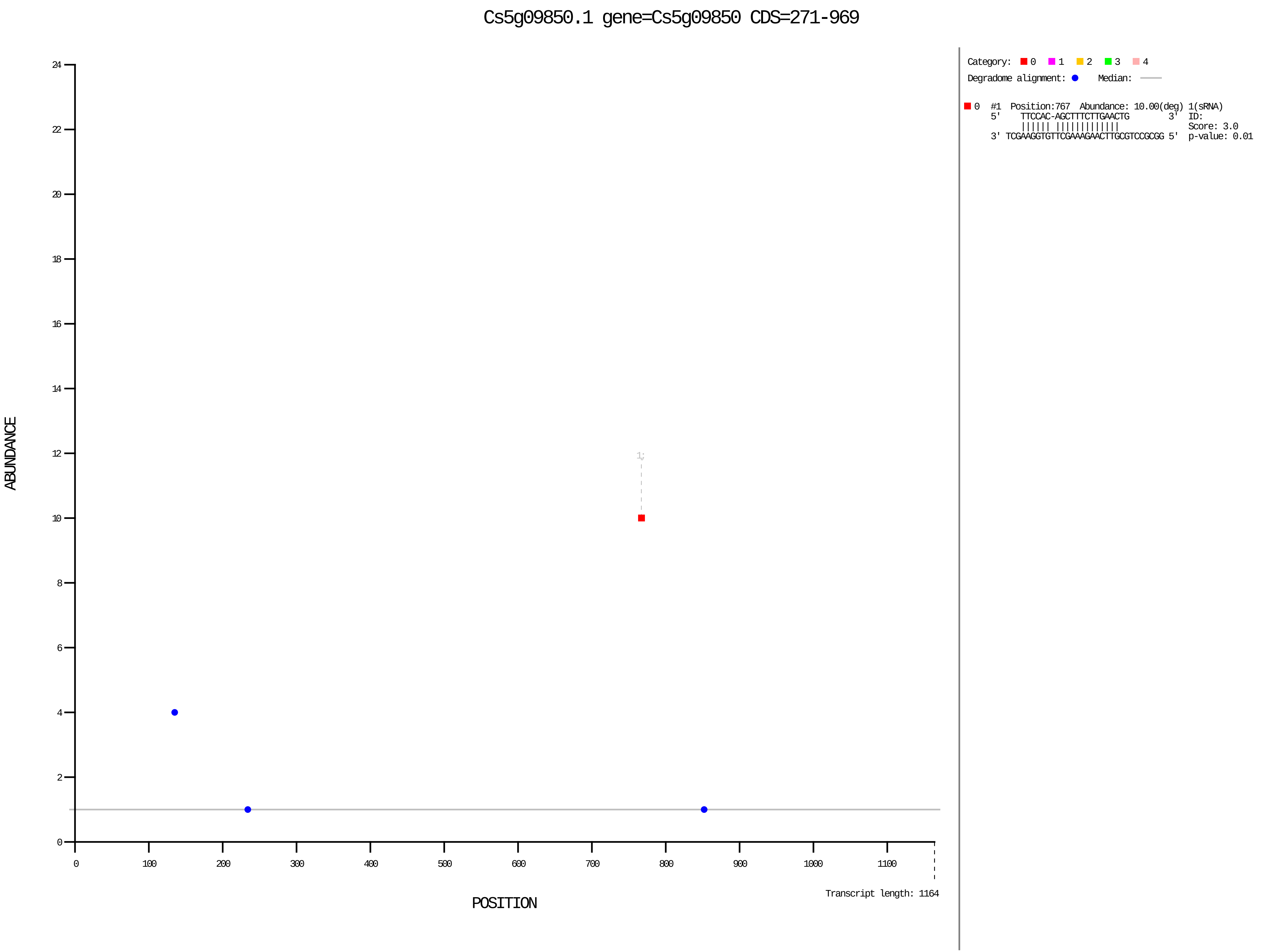
Cs5g09850.1 gene=Cs5g09850 CDS=271-969
ABUNDANCE
POSITION
Transcript length: 1164
Category: 0 1 2 3 4
Degradome alignment:
Median:
0 #1 Position:767 Abundance: 10.00(deg) 1(sRNA)
5' TTCCAC-AGCTTTCTTGAACTG 3' ID:
|||||| |||||||||||||
Score: 3.0
3' TCGAAGGTGTTCGAAAGAACTTGCGTCCGCGG 5' p-value: 0.01

Cs5g19570.1 gene=Cs5g19570 CDS=1483-3201

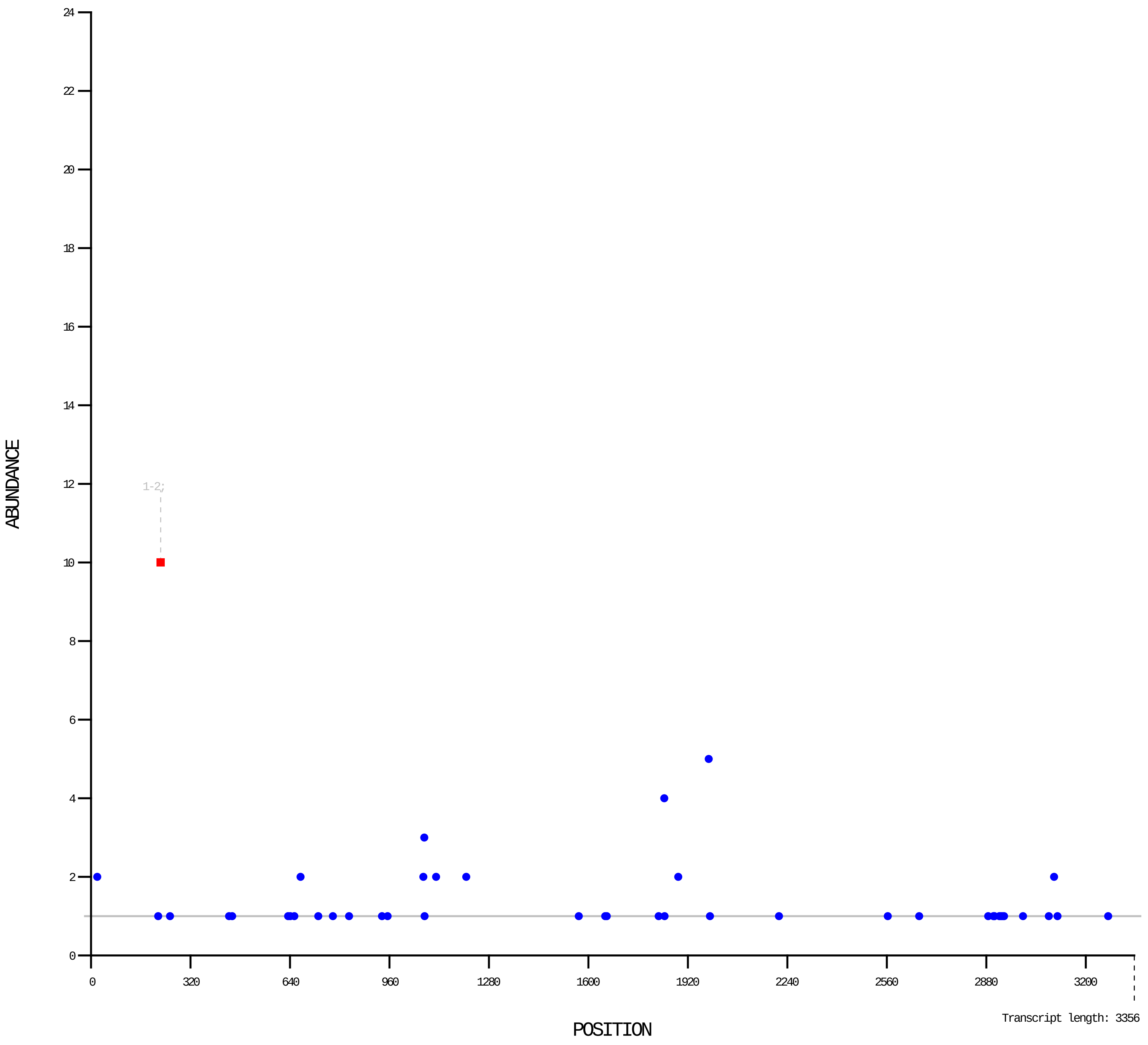

Category: ■ 0 ■ 1 ■ 2 ■ 3 ■ 4
Degradome alignment: ● Median: —

```
■ 0  #1  Position:224  Abundance: 10.00(deg) 1(sRNA)
     5'     TCTTACCTATGCCACCCATTCC       3'  ID:
            |||| |||||o||||| |||||           Score: 2.5
     3' CAACAGAAAGGATATGGTGGCTAAGGGGTTTA 5'  p-value: 0.0

■ 0  #2  Position:224  Abundance: 10.00(deg) 1(sRNA)
     5'     TCTTCCCTATGCCTCCCATTCC       3'  ID:
            |||| |||||o|| || |||||           Score: 3.5
     3' CAACAGAAAGGATATGGTGGCTAAGGGGTTTA 5'  p-value: 0.0
```

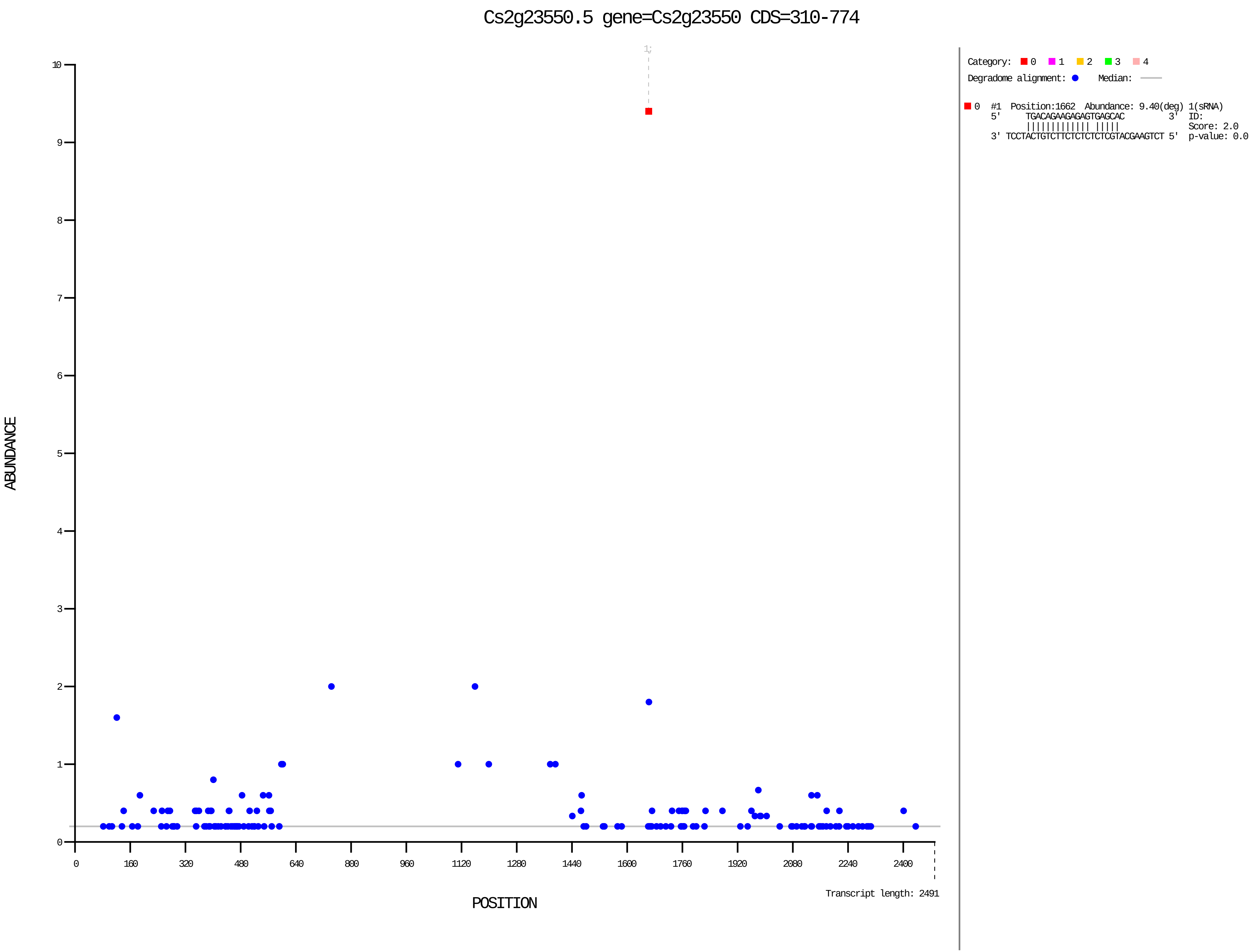

Cs2g23550.5 gene=Cs2g23550 CDS=310-774
ABUNDANCE
POSITION
Transcript length: 2491
Category: 0 1 2 3 4
Degradome alignment:
Median:
0 #1 Position:1662 Abundance: 9.40(deg) 1(sRNA)
5' TGACAGAAGAGAGTGAGCAC 3' ID:
||||||||||||| |||||
Score: 2.0
3' TCCTACTGTCTTCTCTCTCTCGTACGAAGTCT 5' p-value: 0.0

Cs2g23550.1 gene=Cs2g23550 CDS=389-910

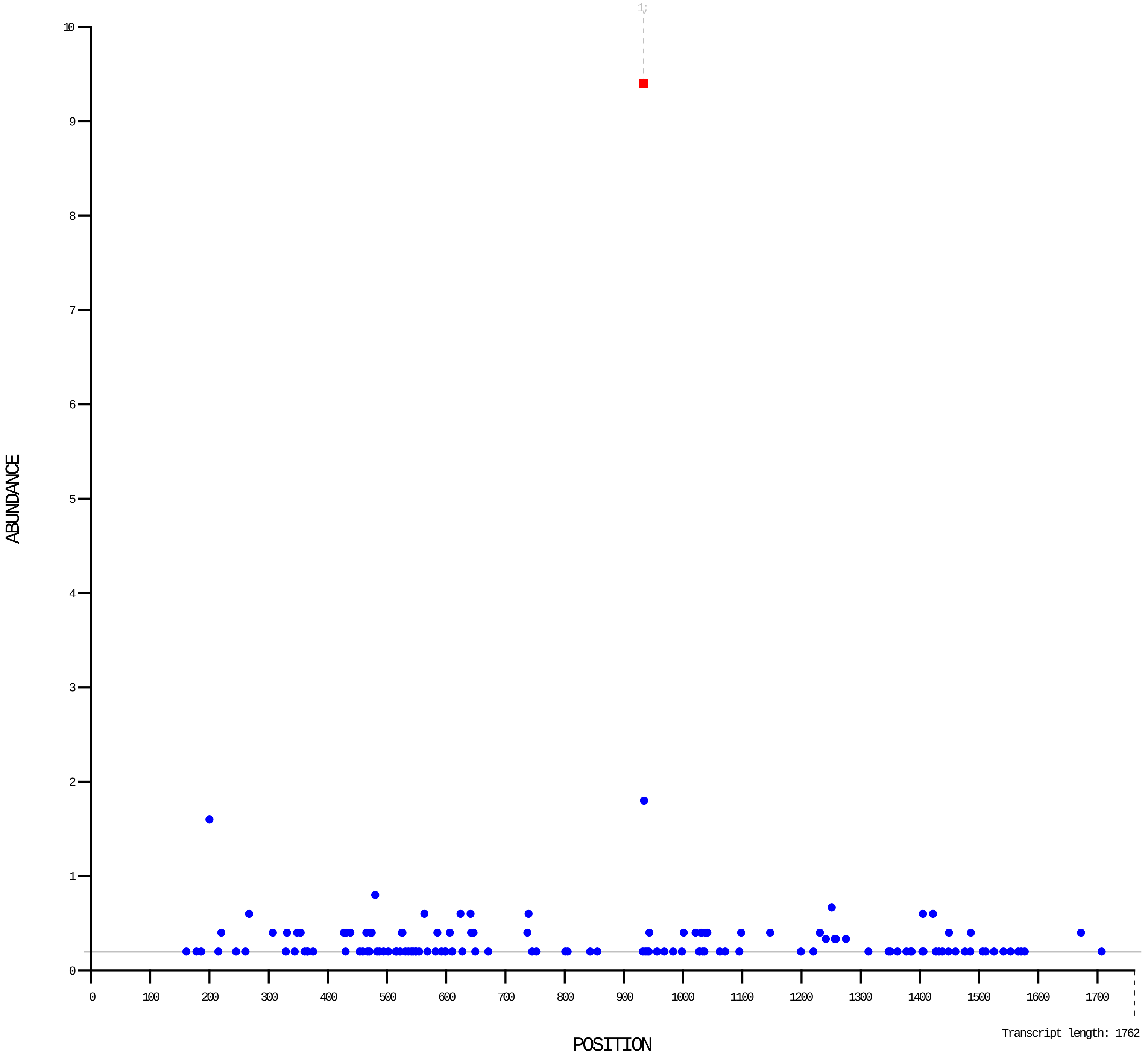

Category: 0 1 2 3 4
Degradome alignment: Median:

0 #1 Position:933 Abundance: 9.40(deg) 1(sRNA)

```
5'    TGACAGAAGAGAGTGAGCAC        3'  ID:
      ||||||||||||| |||||             Score: 2.0
3' TCCTACTGTCTTCTCTCTCGTACGAAGTCT 5'  p-value: 0.0
```

Cs2g23550.4 gene=Cs2g23550 CDS=389-958

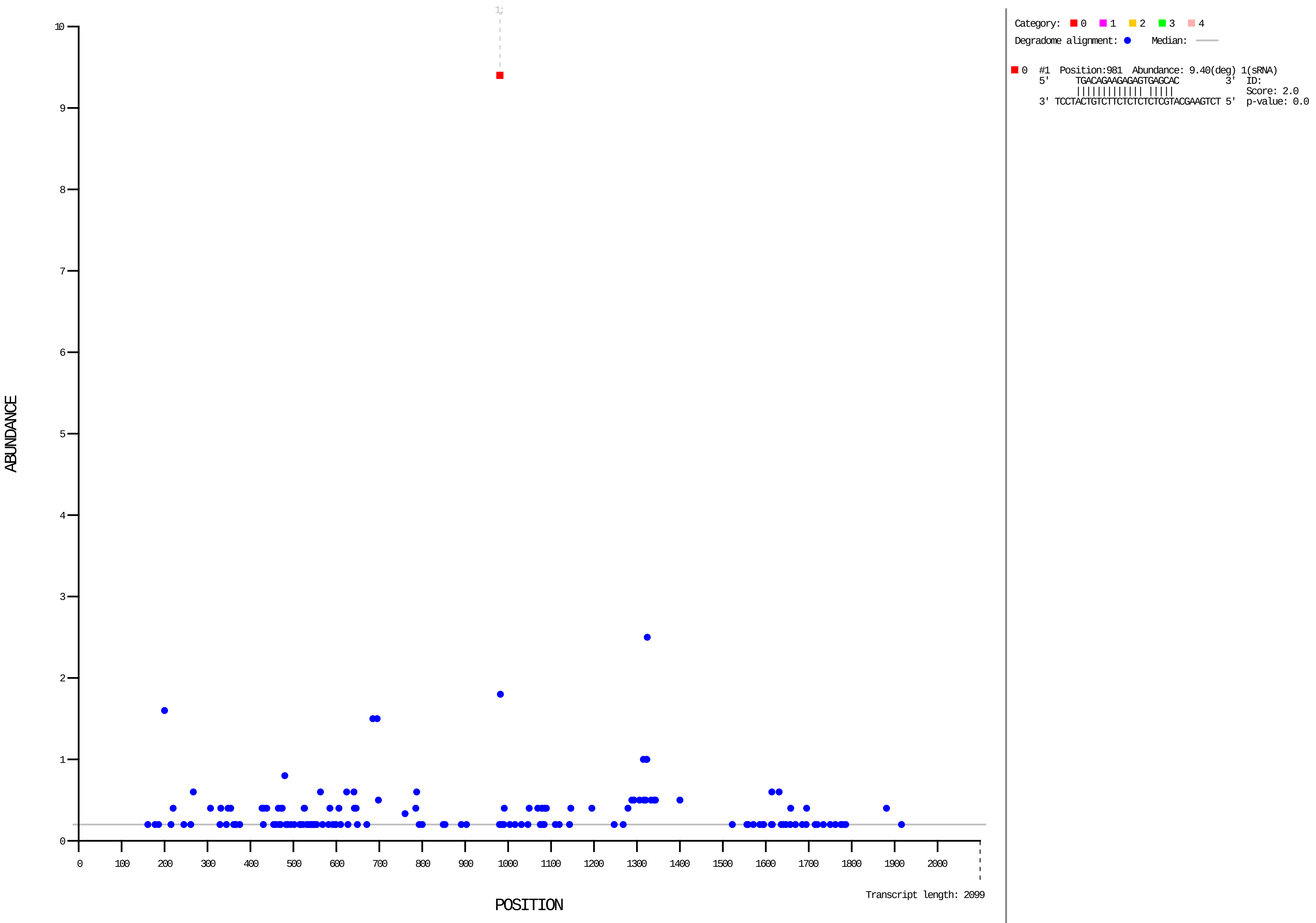

Cs2g23550.2 gene=Cs2g23550 CDS=310-879

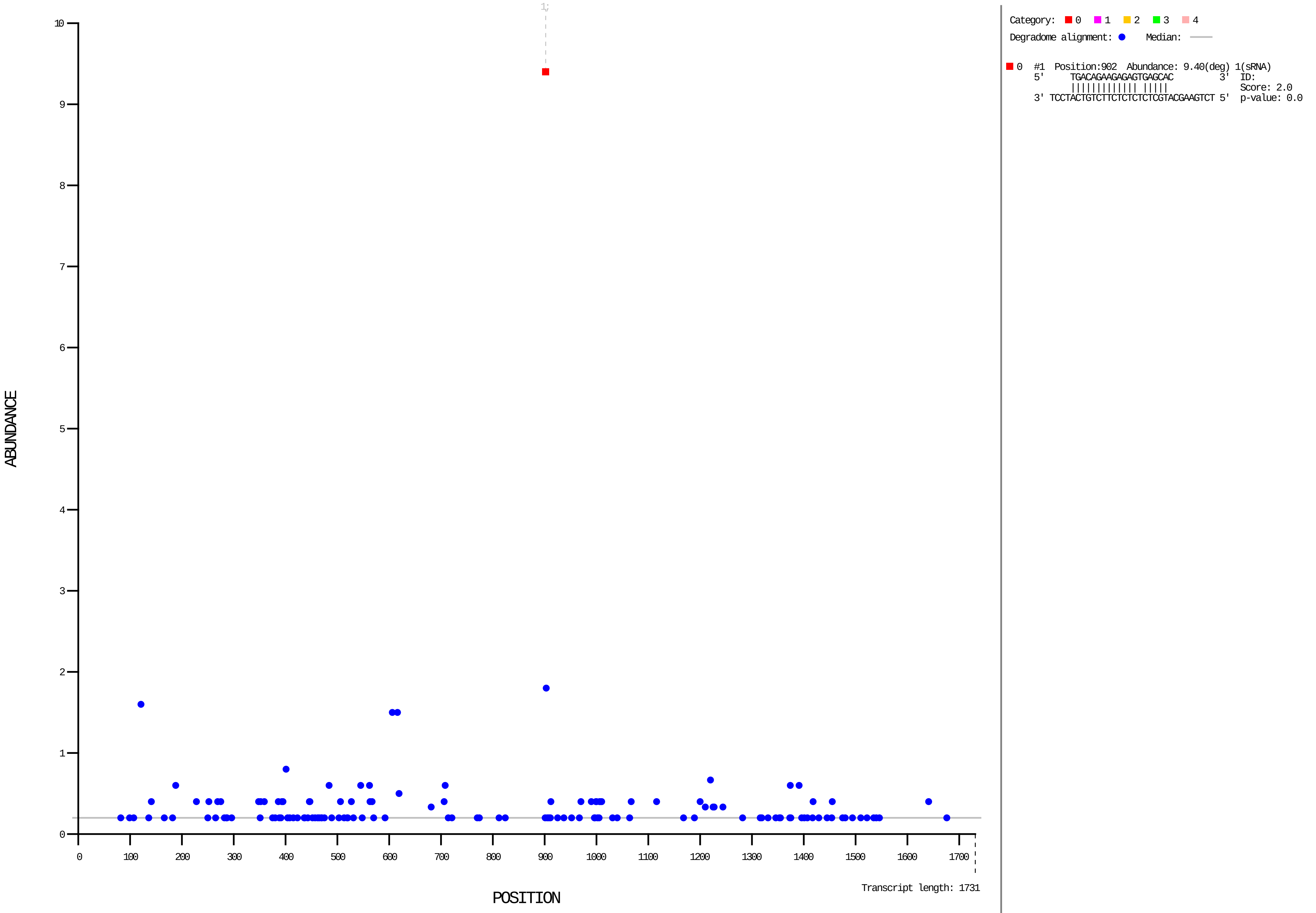

Cs2g23550.3 gene=Cs2g23550 CDS=310-831

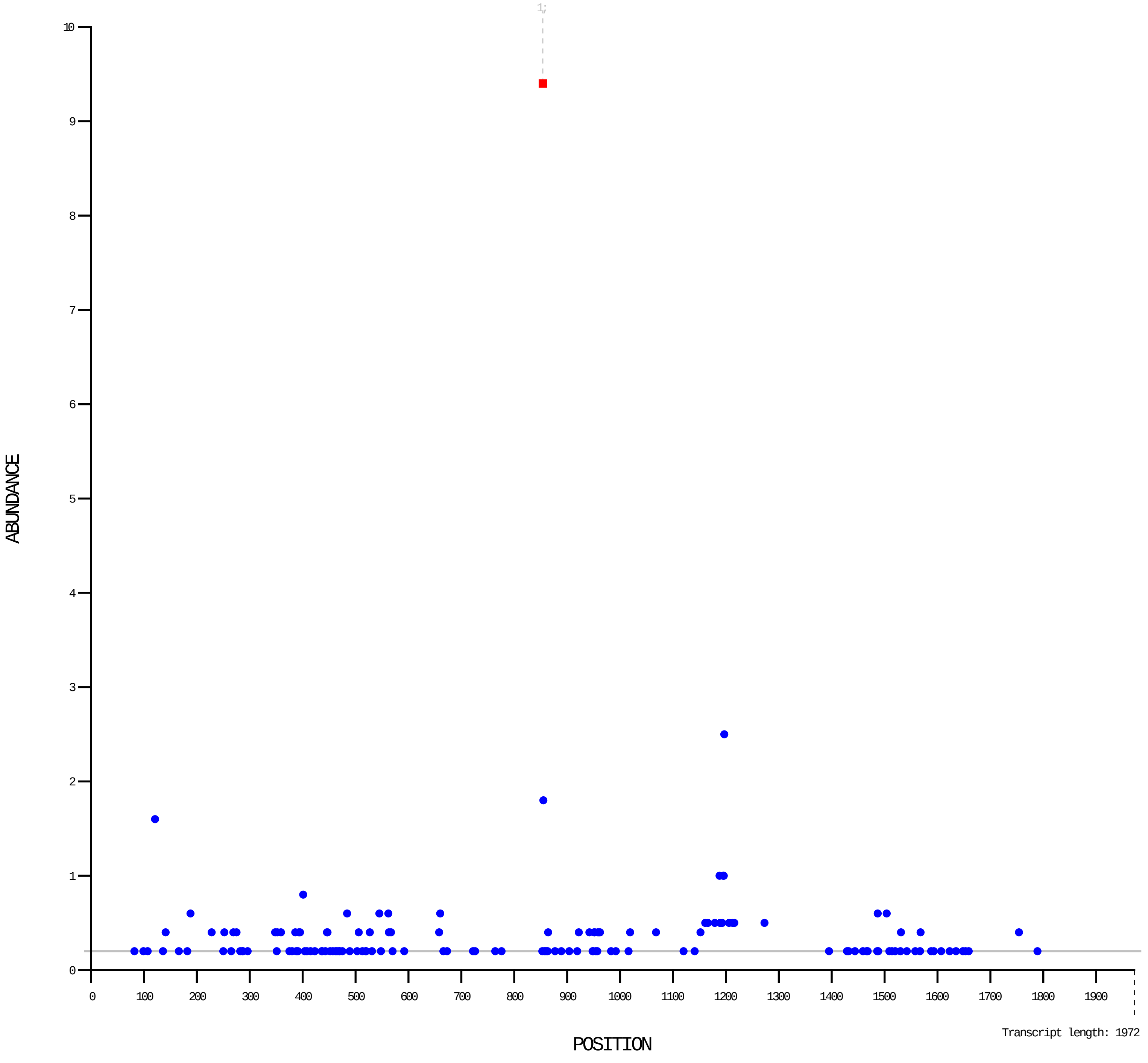


```
Category: 0 1 2 3 4
Degradome alignment:    Median:

0  #1  Position:854  Abundance: 9.40(deg) 1(sRNA)
   5'     TGACAGAAGAGAGTGAGCAC          3'  ID:
          ||||||||||||| |||||               Score: 2.0
   3' TCCTACTGTCTTCTCTCTCTCGTACGAAGTCT 5'  p-value: 0.01
```

Cs1g19720.1 gene=Cs1g19720 CDS=236-1567

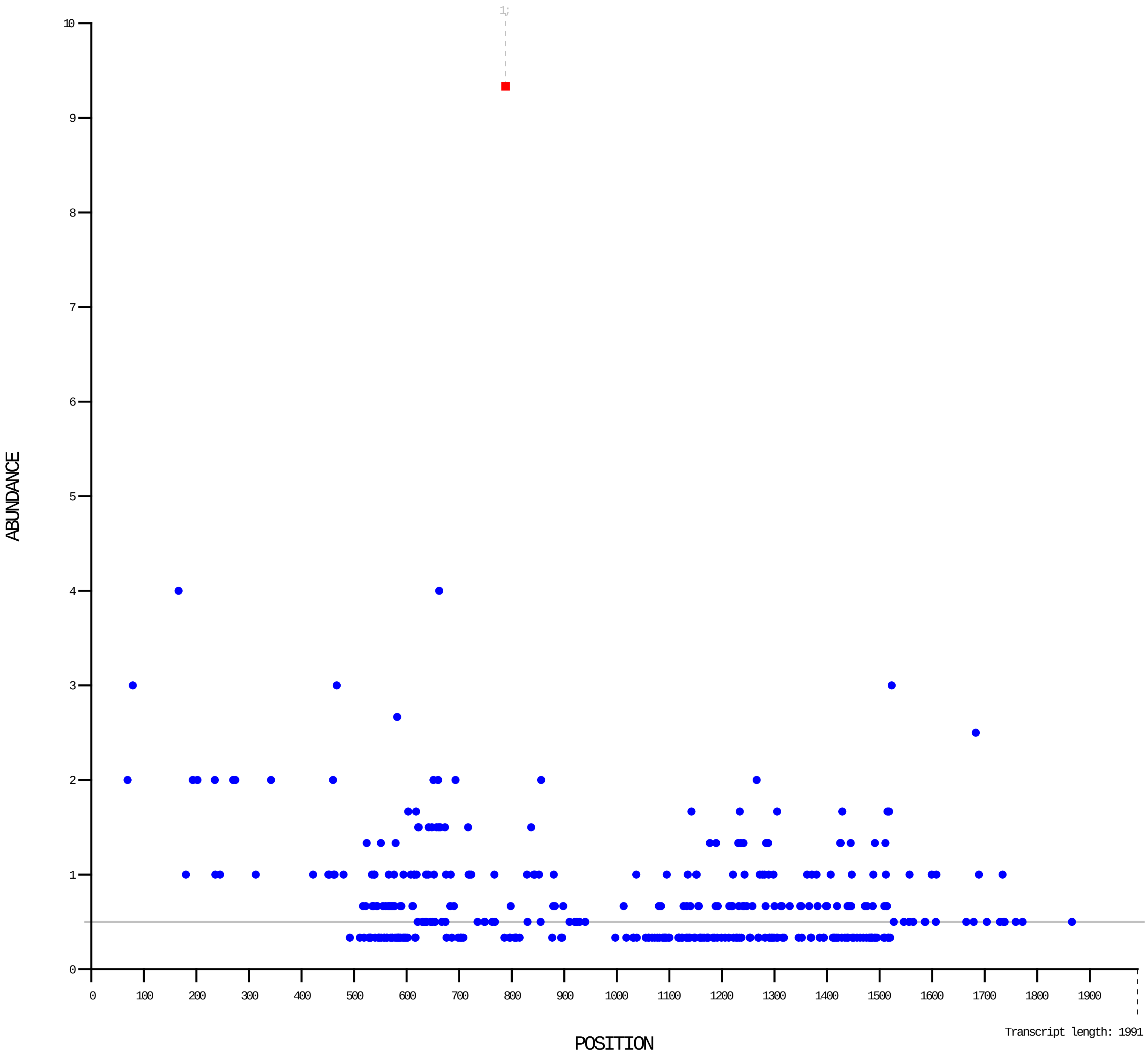

Category: 0 1 2 3 4

Degradome alignment: Median:

```
0   #1  Position:788  Abundance: 9.33(deg) 1(sRNA)
    5'     TCTTCCCTATGCCTCCCATTCC        3'  ID:
            | ||||o|||||o||||||||            Score: 3.0
    3' GAGACG-AGGGGTACGGGGGGTAAGGCGGGTA 5'  p-value: 0.0
```

Cs1g19720.2 gene=Cs1g19720 CDS=12-1136

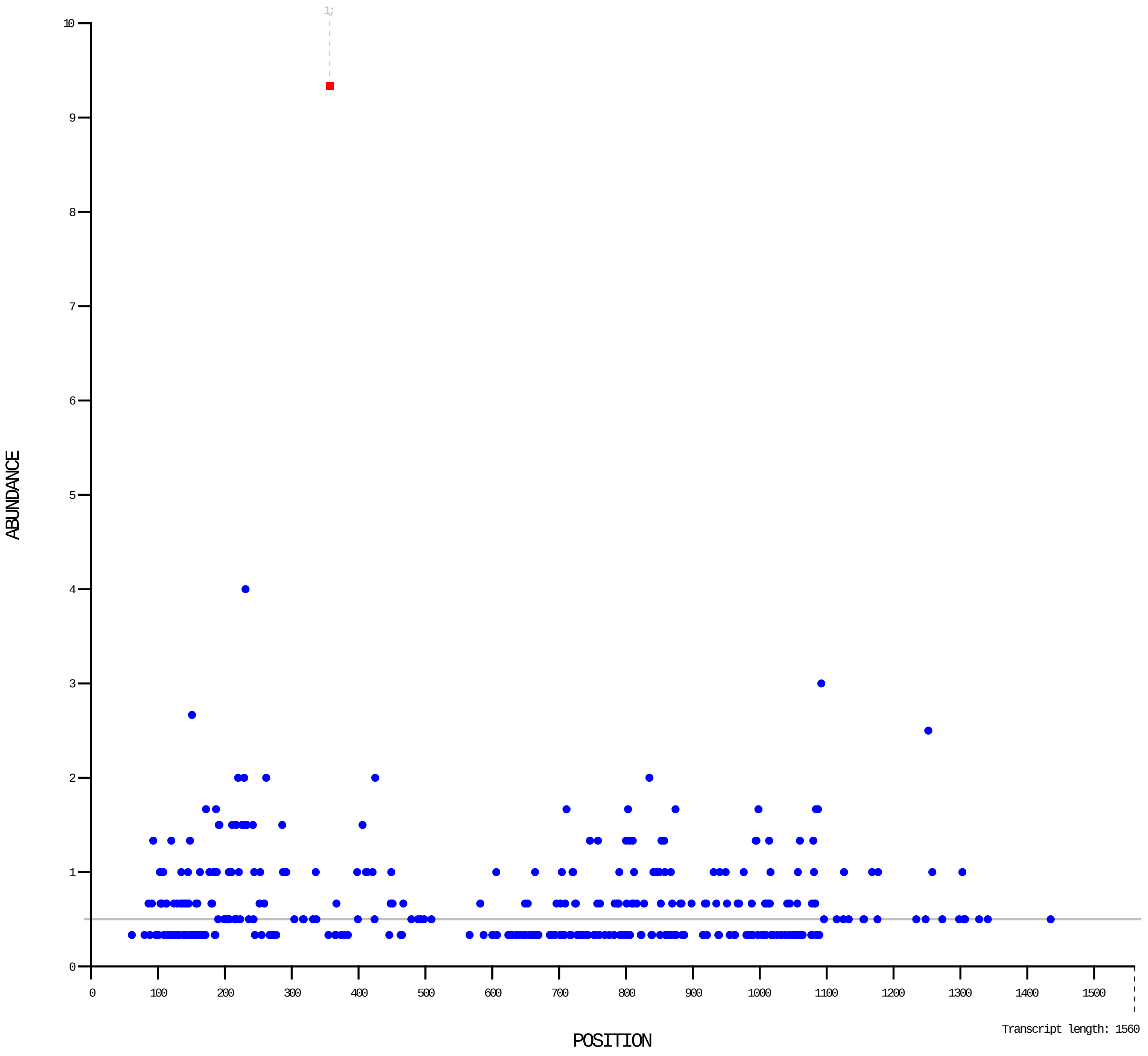

Category: 0 1 2 3 4

Degradome alignment: ● Median: —

0 #1 Position:357 Abundance: 9.33(deg) 1(sRNA)

```
5'     TCTTCCCTATGCCTCCCATTCC       3'  ID:
         | ||||o|||||o||||||||          Score: 3.0
3' GAGACG-AGGGGTACGGGGGGTAAGGCGGGTA 5'  p-value: 0.0
```

Cs1g19760.1 gene=Cs1g19760 CDS=1-1332

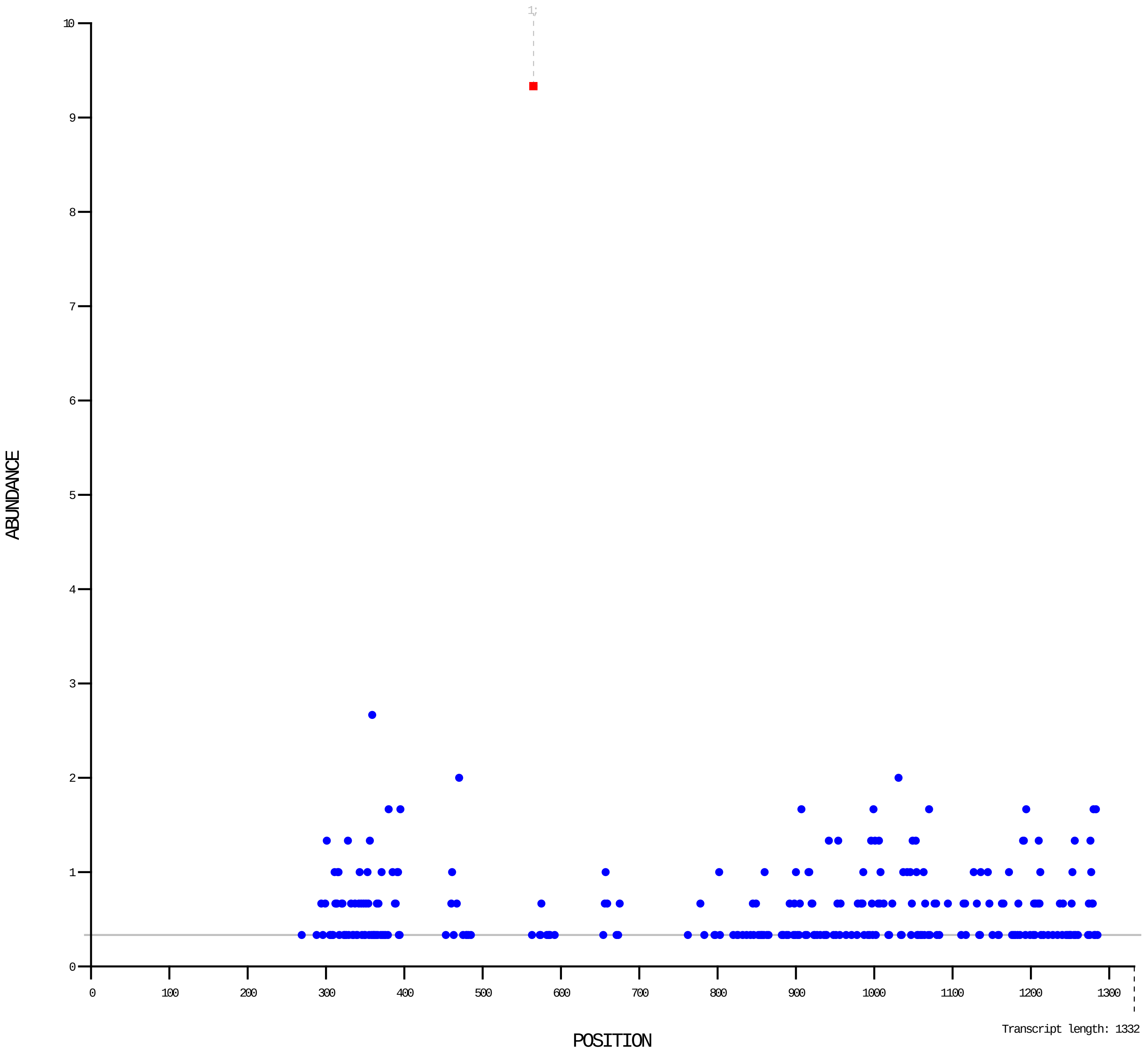


```
Category: 0 1 2 3 4
Degradome alignment:      Median:

0  #1  Position:565  Abundance: 9.33(deg) 1(sRNA)
   5'     TCTTCCCTATGCCTCCCATTCC        3'  ID:
           | ||||o|||| o||||||||            Score: 4.0
   3' GAGACG-AGGGGTACGAGGGGTAAGGCGGGTA 5'  p-value: 0.03
```

Cs5g12260.1 gene=Cs5g12260 CDS=458-2131

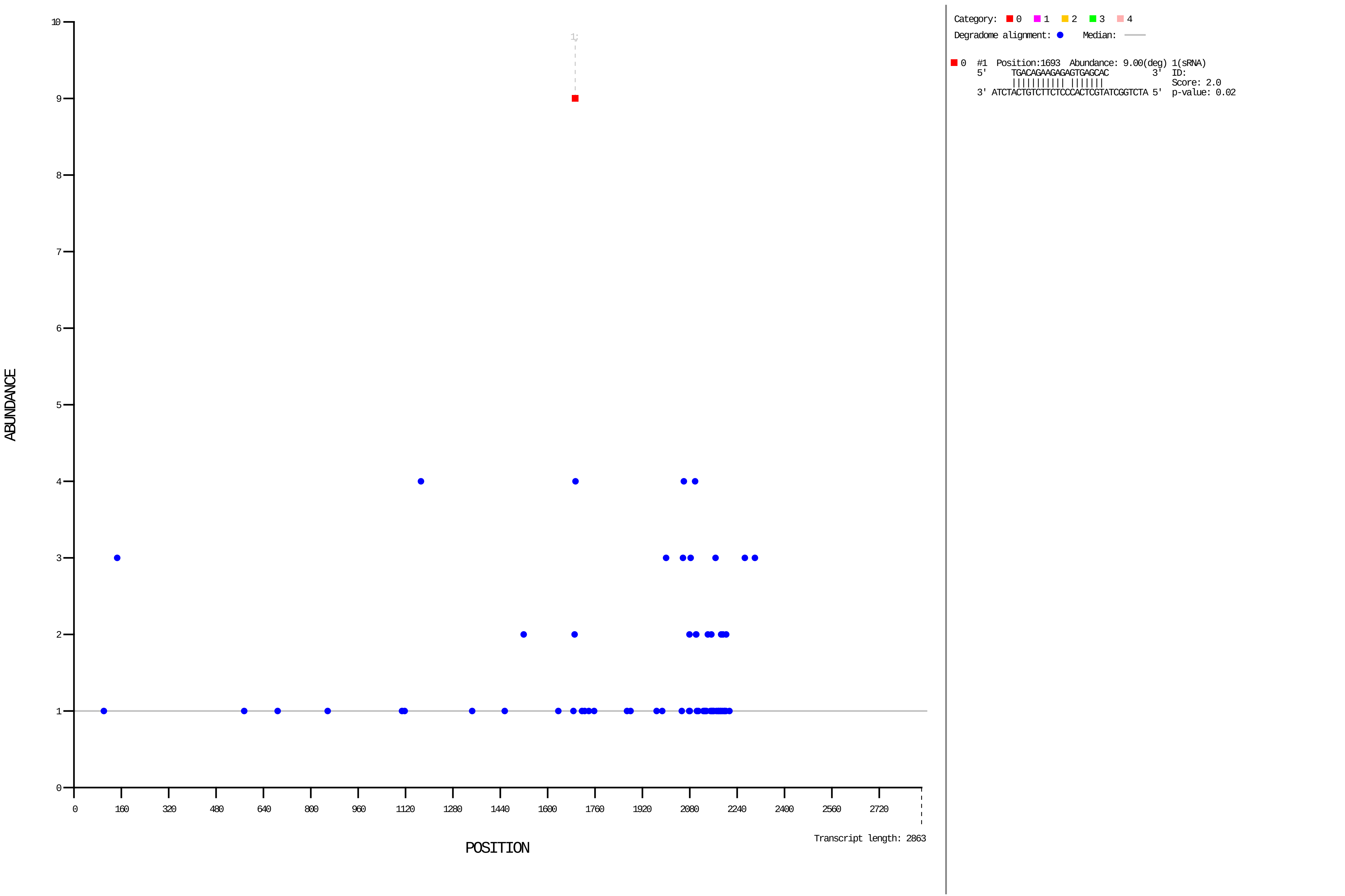

orange1.1t04537.1 gene=orange1.1t04537 CDS=344-3817

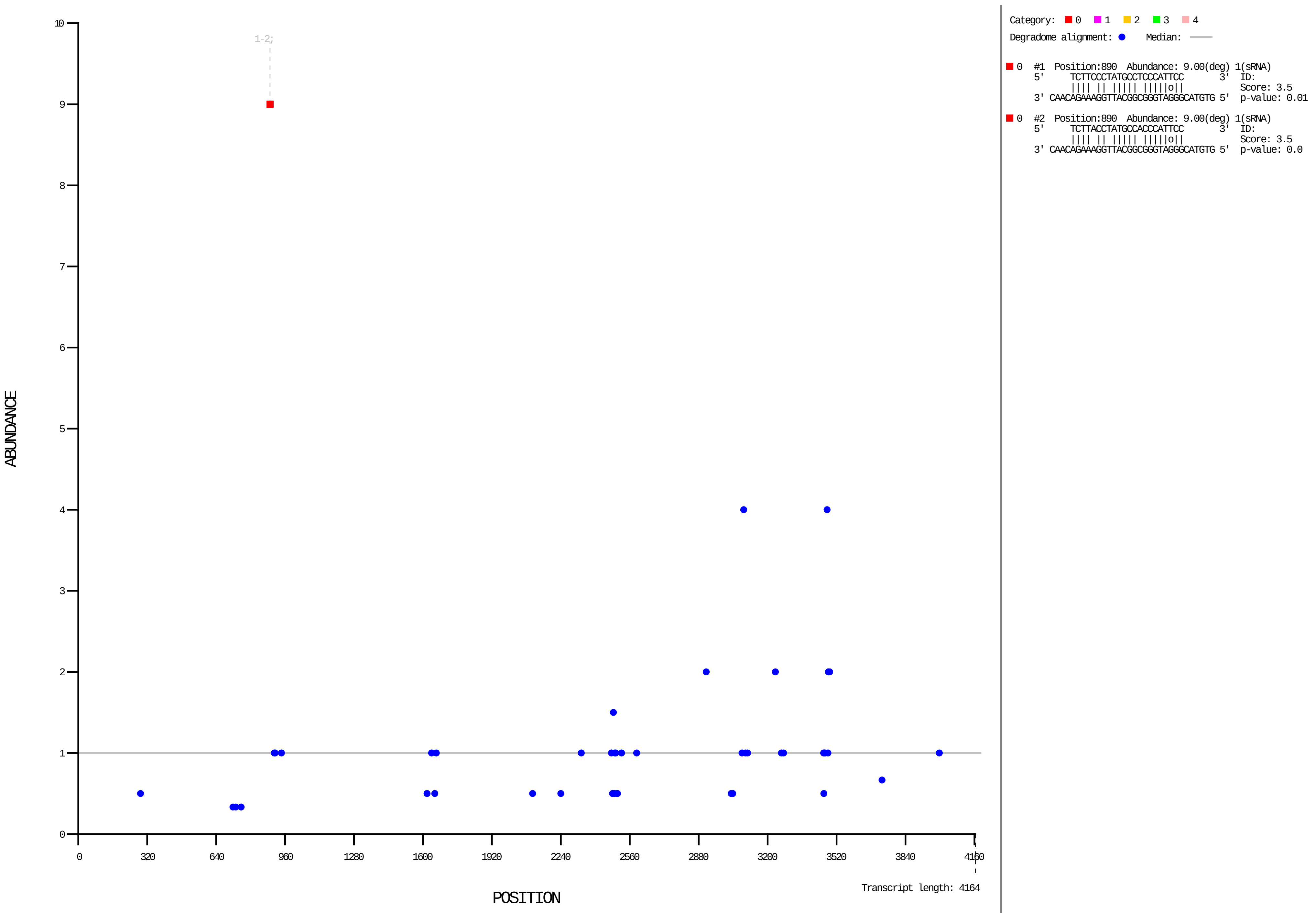

Cs6g04120.1 gene=Cs6g04120 CDS=213-1679

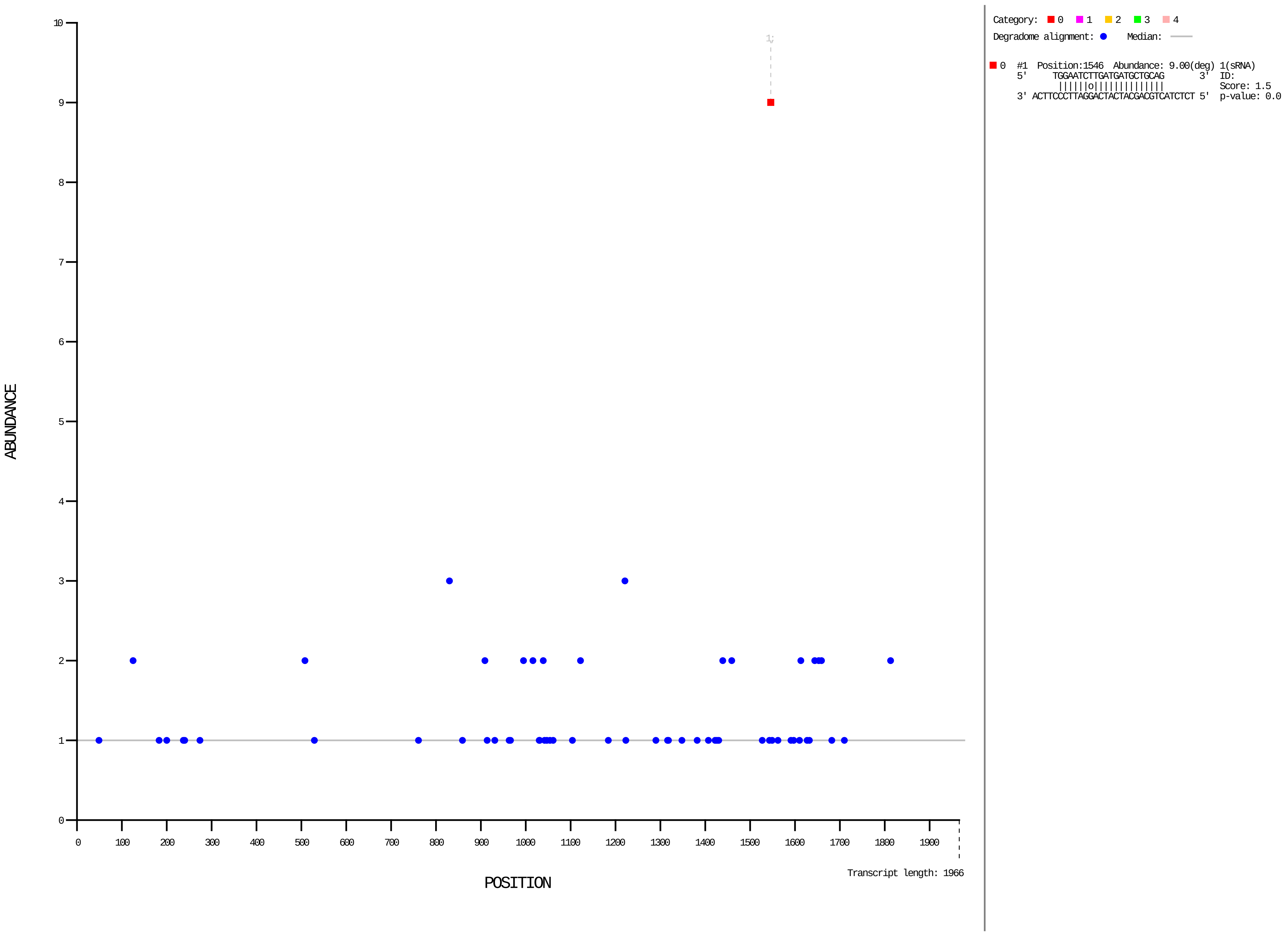

Cs6g07400.1 gene=Cs6g07400 CDS=121-1827

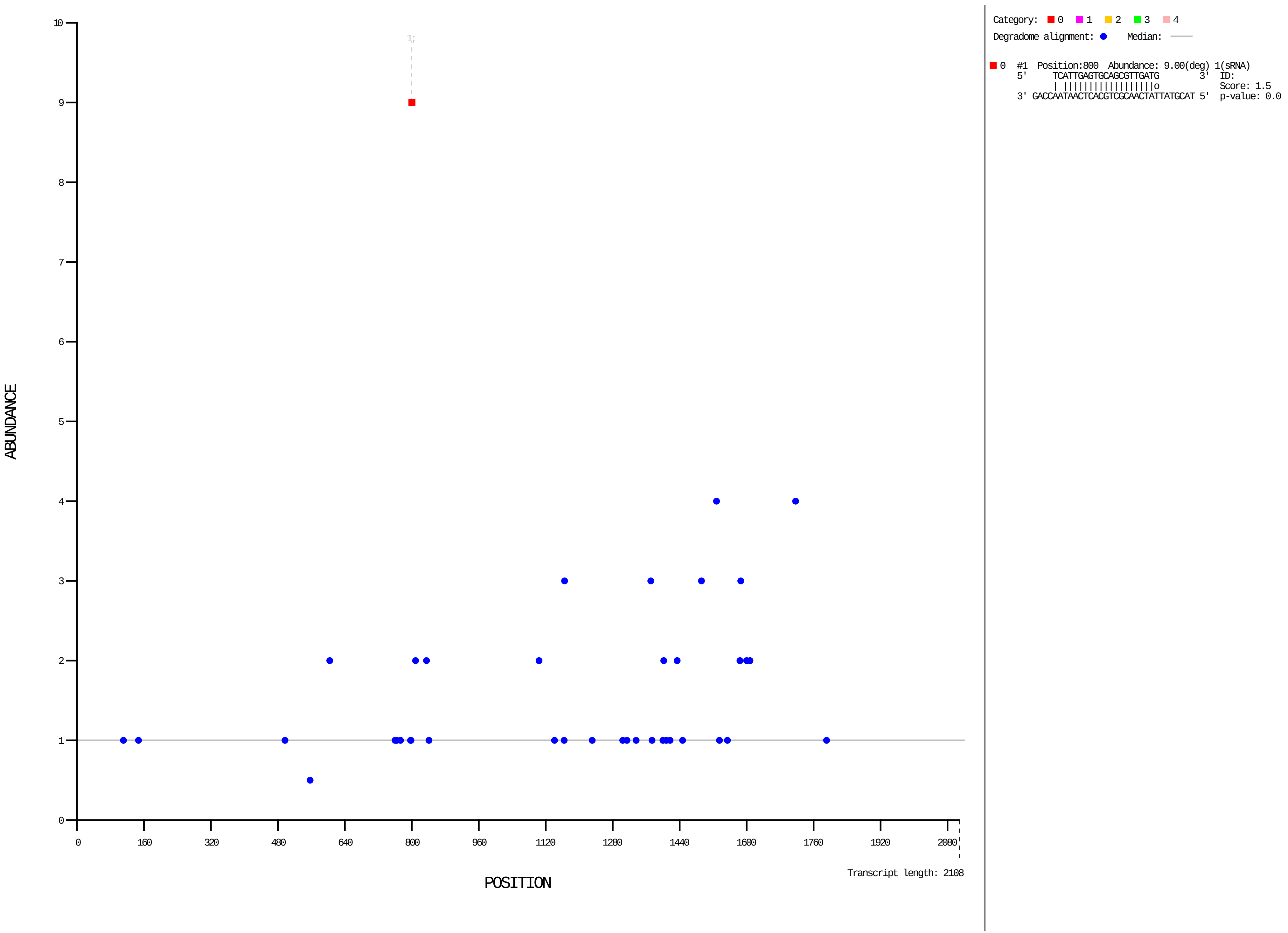

Cs3g13180.1 gene=Cs3g13180 CDS=1-1536

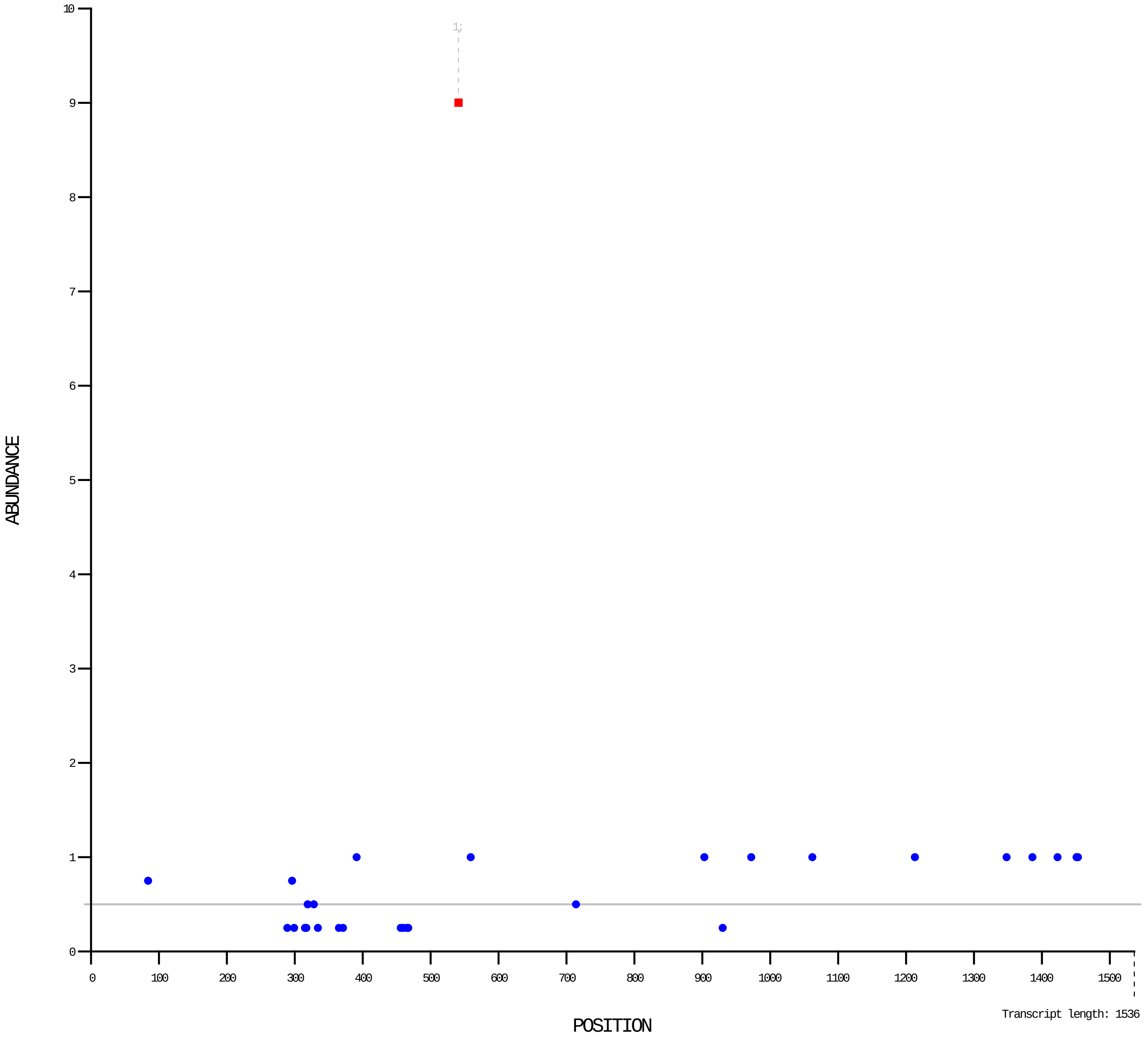

Category: 0 1 2 3 4
Degradome alignment: Median:

```
0  #1  Position:541  Abundance: 9.00(deg) 1(sRNA)
   5'     TCTTGCCCACCCCTCCCATTCC       3'  ID:
          |||| || |||||  |||||||           Score: 4.0
   3' TAACAGAAGGGTTGGGGCCGGTAAGGTATGCG 5'  p-value: 0.01
```

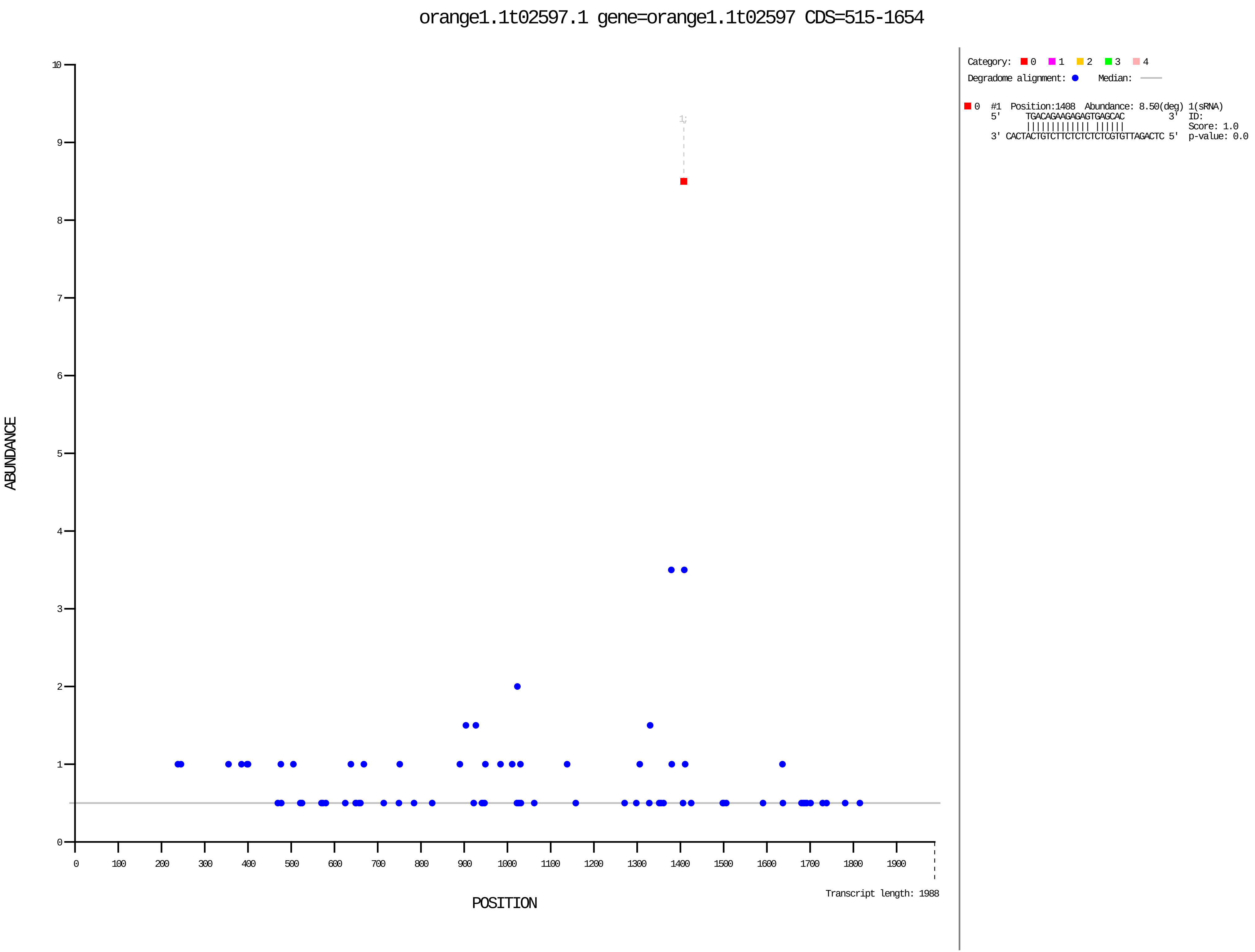

orange1.1t02597.1 gene=orange1.1t02597 CDS=515-1654
ABUNDANCE
POSITION
Transcript length: 1988
Category: 0 1 2 3 4
Degradome alignment:
Median:
0 #1 Position:1408 Abundance: 8.50(deg) 1(sRNA)
5' TGACAGAAGAGAGTGAGCAC 3' ID:
Score: 1.0
3' CACTACTGTCTTCTCTCTCTCGTGTTAGACTC 5' p-value: 0.0

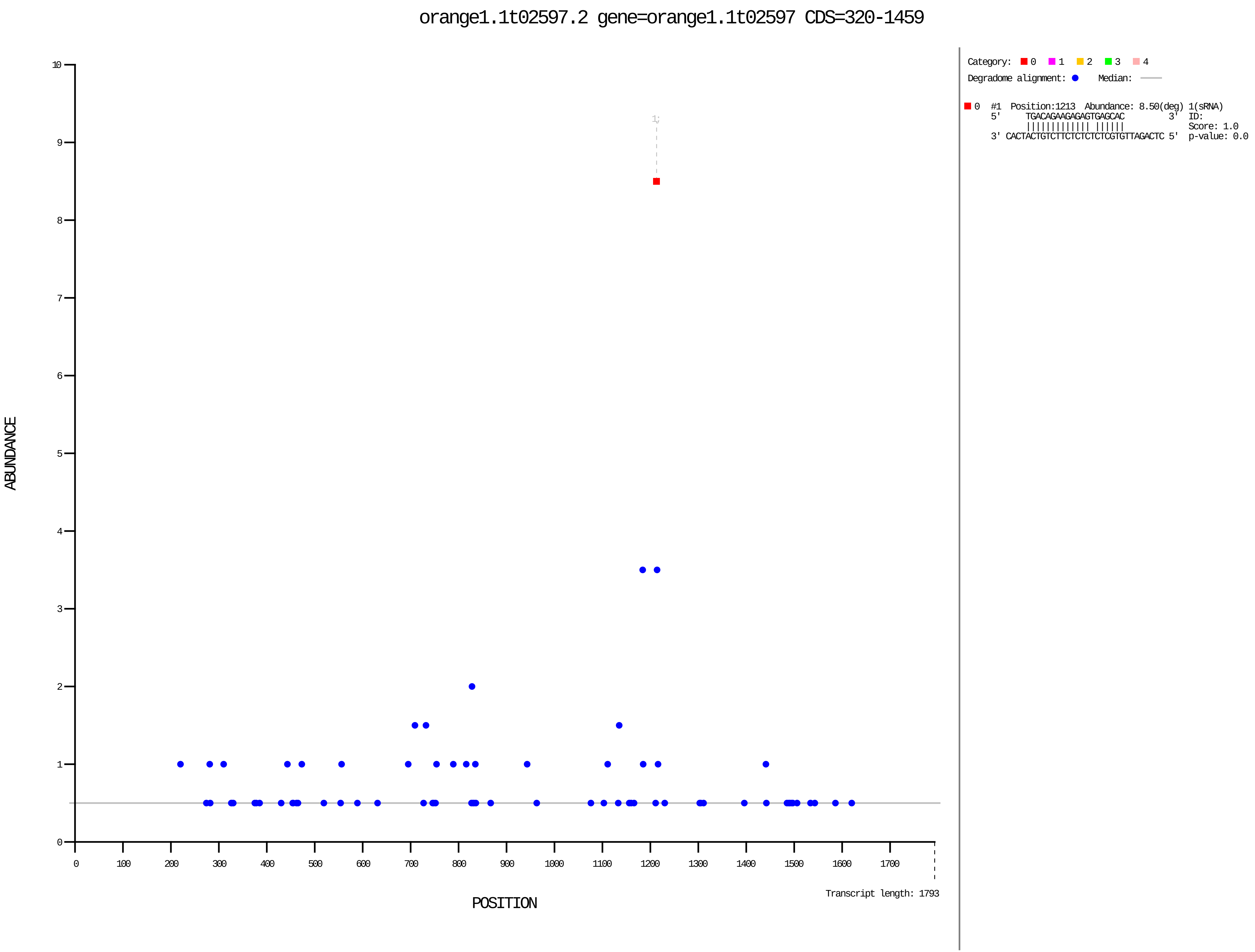
orange1.1t02597.2 gene=orange1.1t02597 CDS=320-1459
Category: 0 1 2 3 4
Degradome alignment:
Median:
0 #1 Position:1213 Abundance: 8.50(deg) 1(sRNA)
5' TGACAGAAGAGAGTGAGCAC 3' ID:
Score: 1.0
3' CACTACTGTCTTCTCTCTCTCGTGTTAGACTC 5' p-value: 0.0
ABUNDANCE
POSITION
Transcript length: 1793

Cs4g05000.1 gene=Cs4g05000 CDS=185-1870

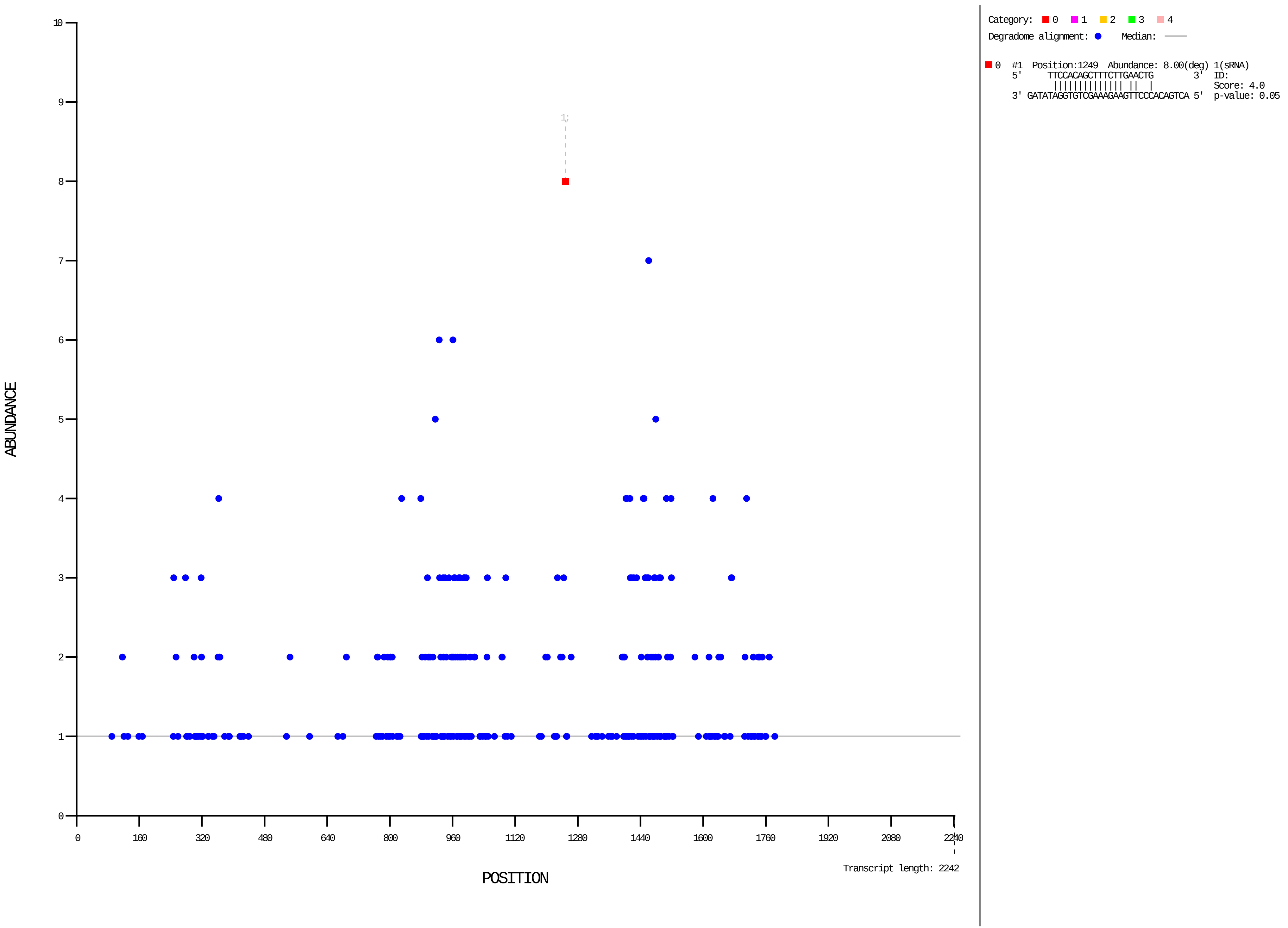

Cs3g04930.1 gene=Cs3g04930 CDS=150-3866

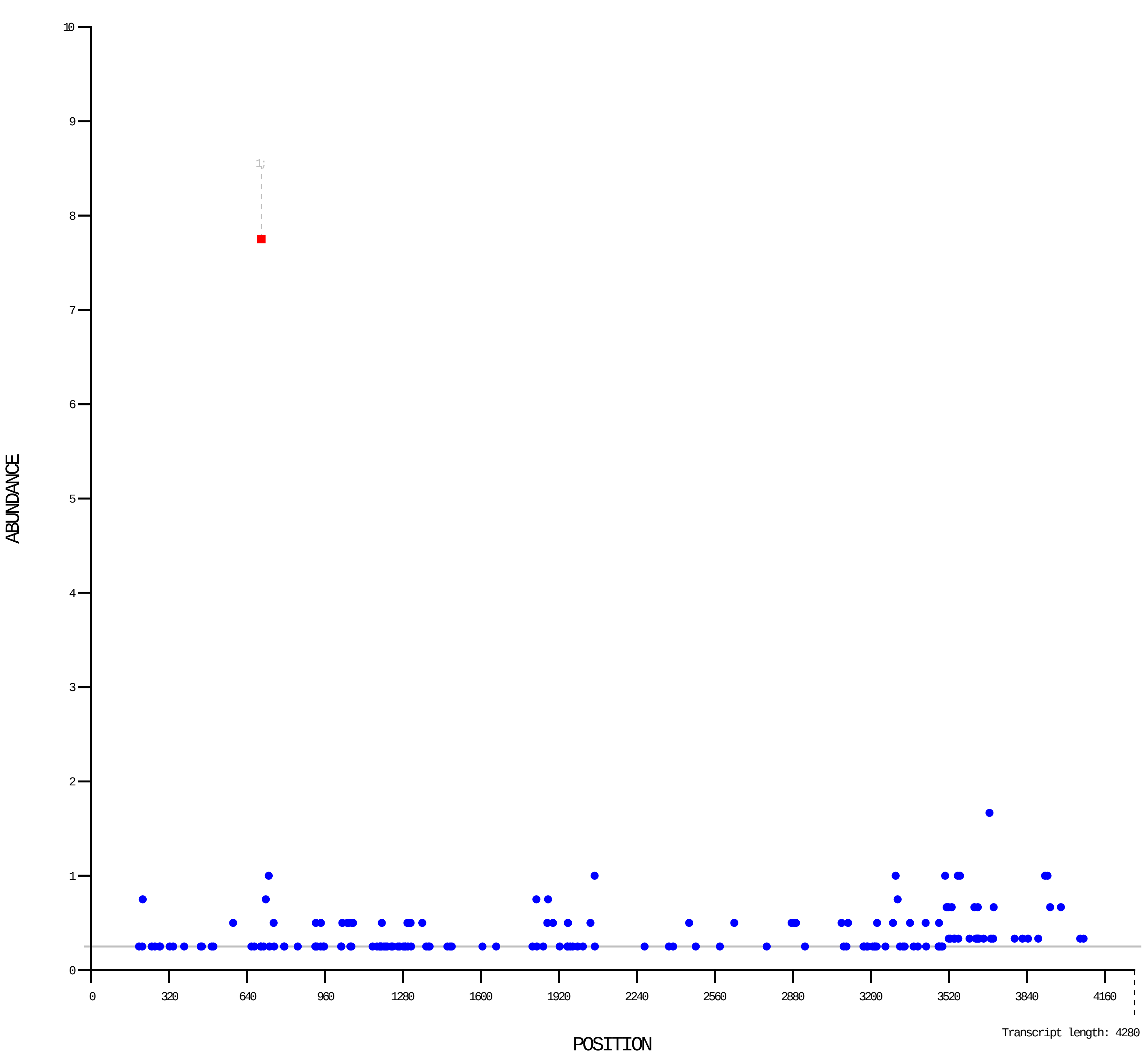

Category: 0 1 2 3 4

Degradome alignment: ● Median: —

```
0  #1  Position:699  Abundance: 7.75(deg) 1(sRNA)
   5'     TCTTGCCCACCCCTCCCATTCC       3'  ID:
          | ||||| || || |||||o||           Score: 4.5
   3' CACCAAAACGGTTGTGGTGGGTAGGGTATGTT 5'  p-value: 0.02
```

Cs3g04930.4 gene=Cs3g04930 CDS=456-4172

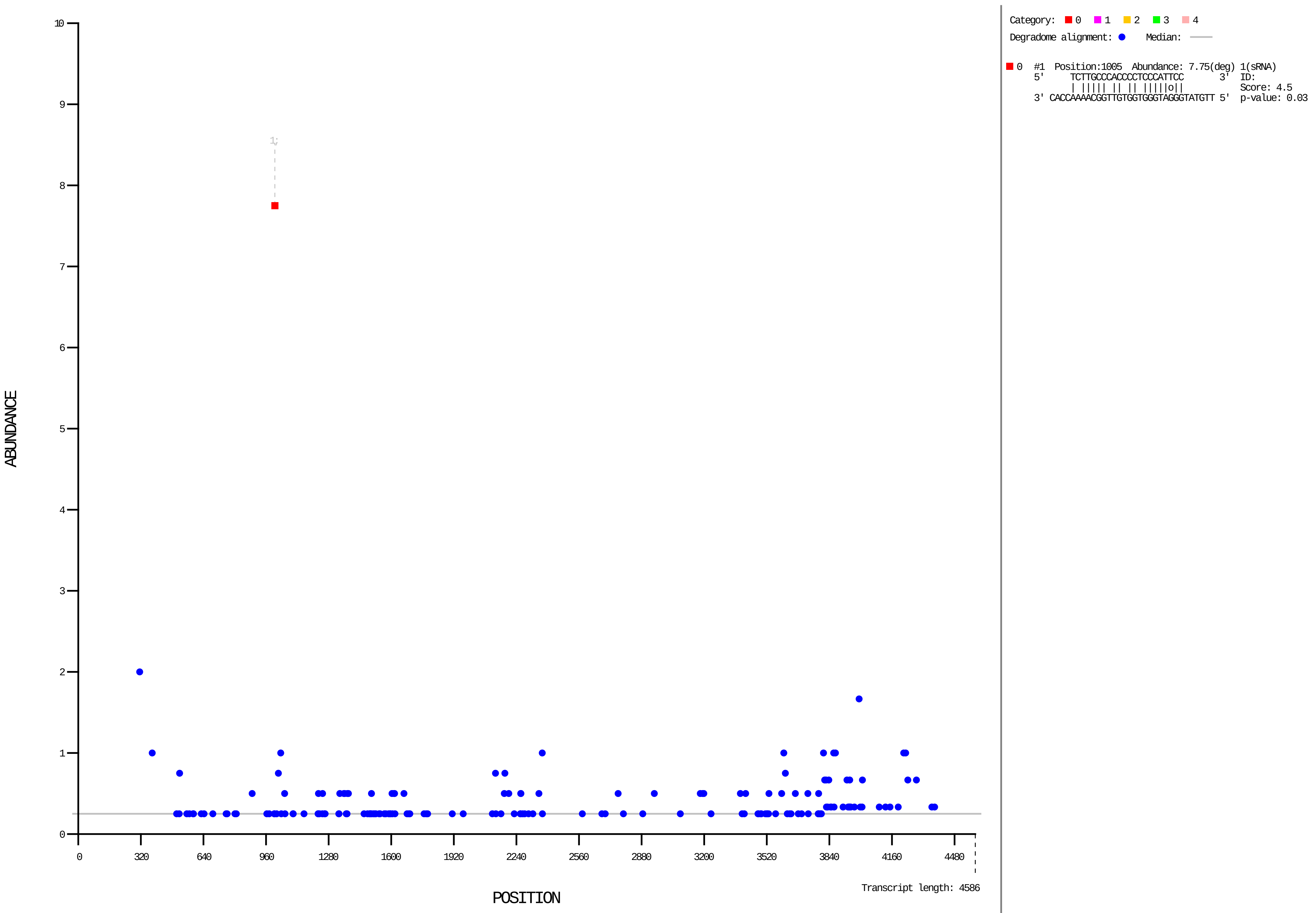

Cs3g04930.2 gene=Cs3g04930 CDS=150-3770

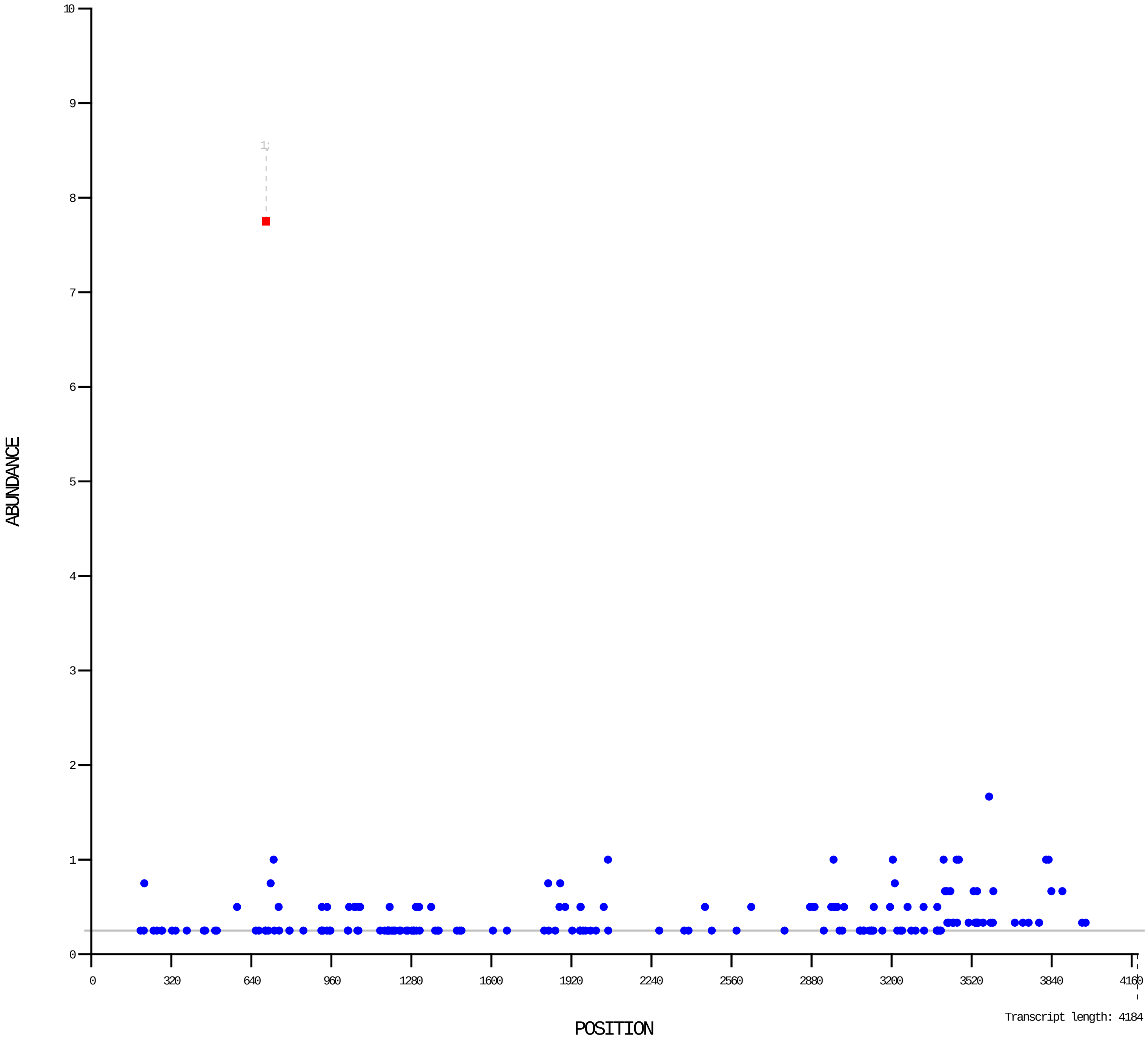

Category: 0 1 2 3 4

Degradome alignment: Median:

```
0  #1  Position:699  Abundance: 7.75(deg) 1(sRNA)
   5'     TCTTGCCCACCCCTCCCATTCC       3'  ID:
          | ||||| || || |||||o||           Score: 4.5
   3' CACCAAAACGGTTGTGGTGGGTAGGGTATGTT 5'  p-value: 0.04
```

Cs3g04930.3 gene=Cs3g04930 CDS=150-3509

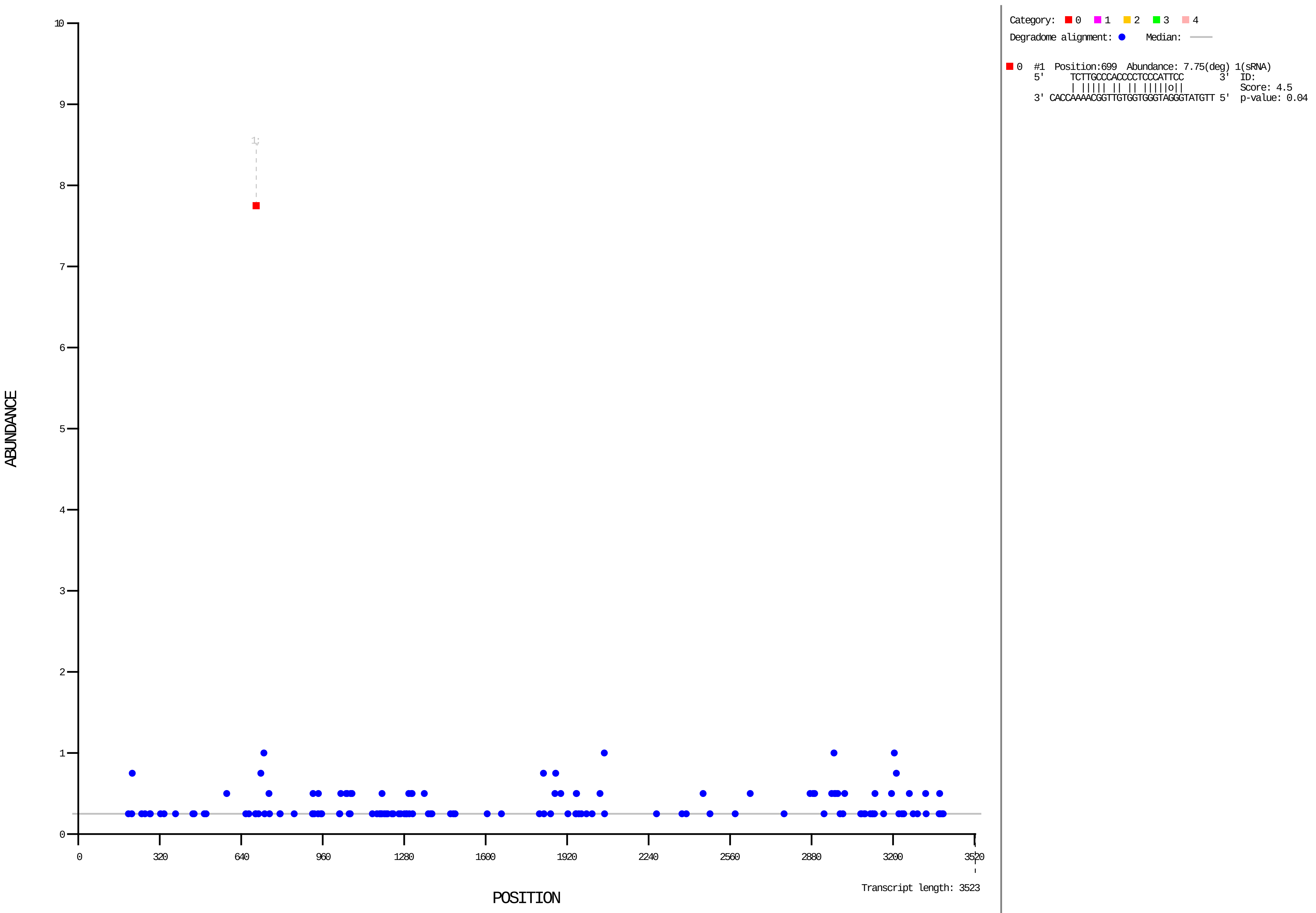

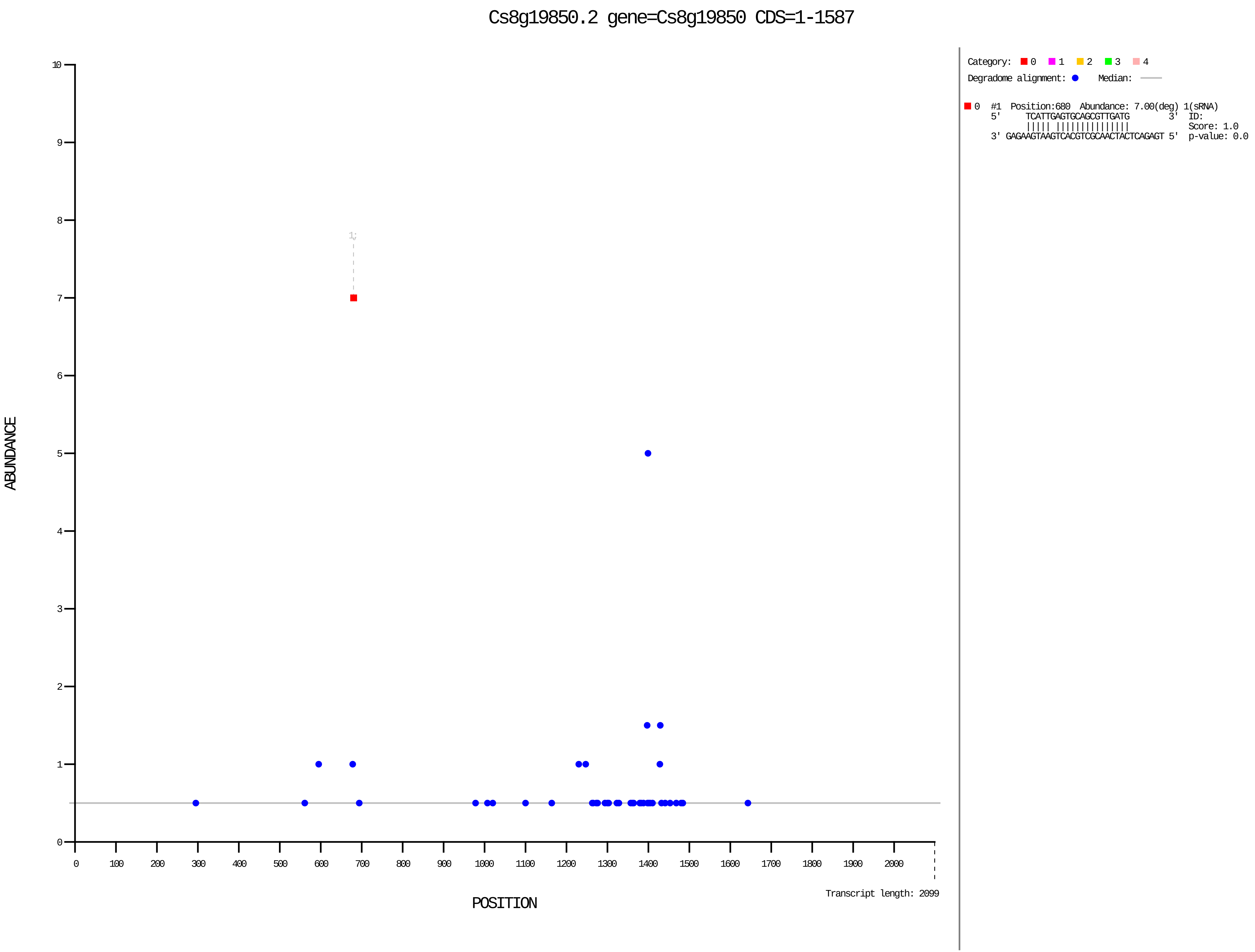

Cs8g19850.2 gene=Cs8g19850 CDS=1-1587
ABUNDANCE
POSITION
Transcript length: 2099
Category: 0 1 2 3 4
Degradome alignment:
Median:
0 #1 Position:680 Abundance: 7.00(deg) 1(sRNA)
5' TCATTGAGTGCAGCGTTGATG 3' ID:
||||| |||||||||||||||
3' GAGAAGTAAGTCACGTCGCAACTACTCAGAGT 5'
Score: 1.0
p-value: 0.0

orange1.1t02234.2 gene=orange1.1t02234 CDS=268-990

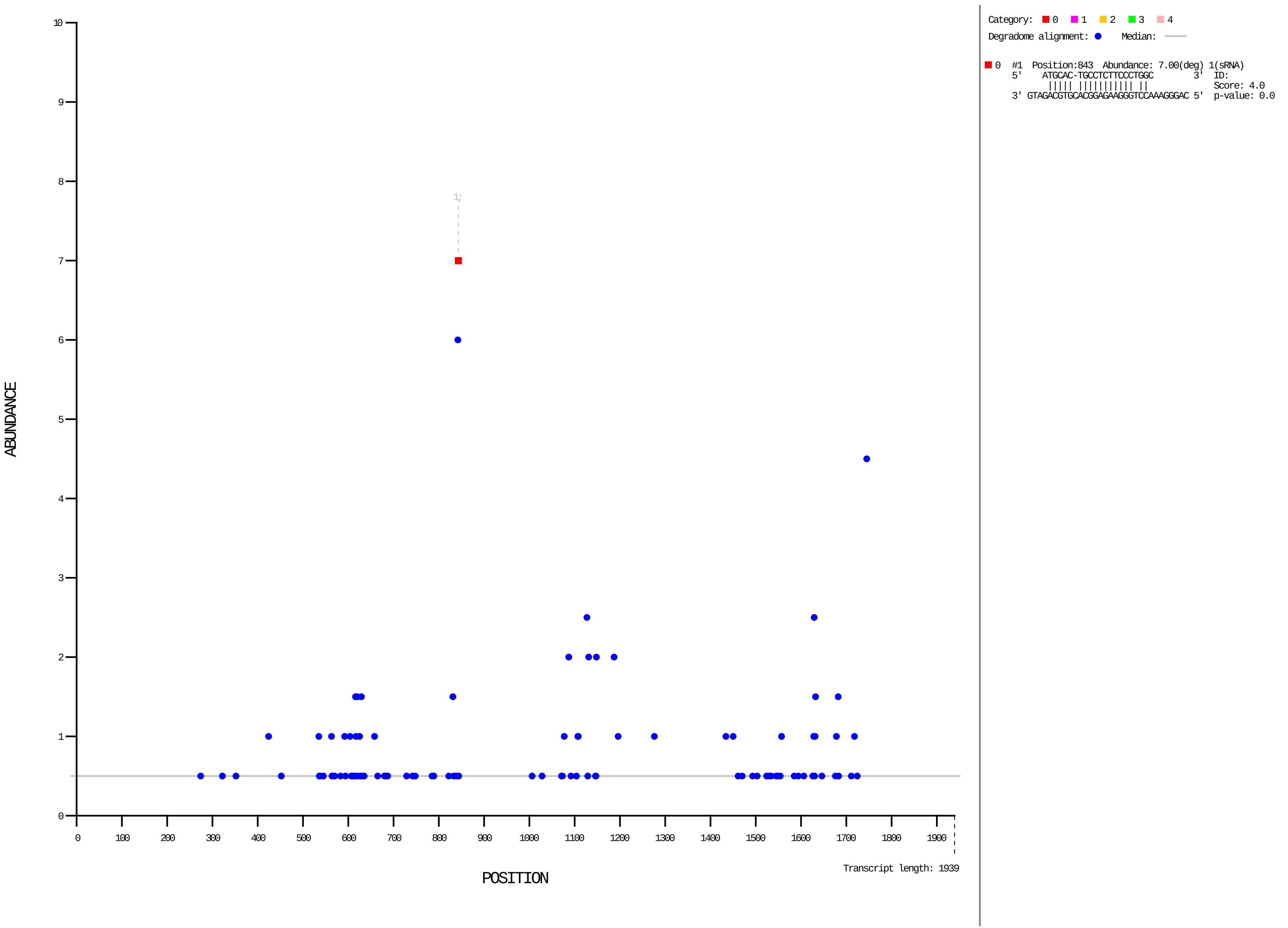

Cs8g19850.1 gene=Cs8g19850 CDS=1-1674

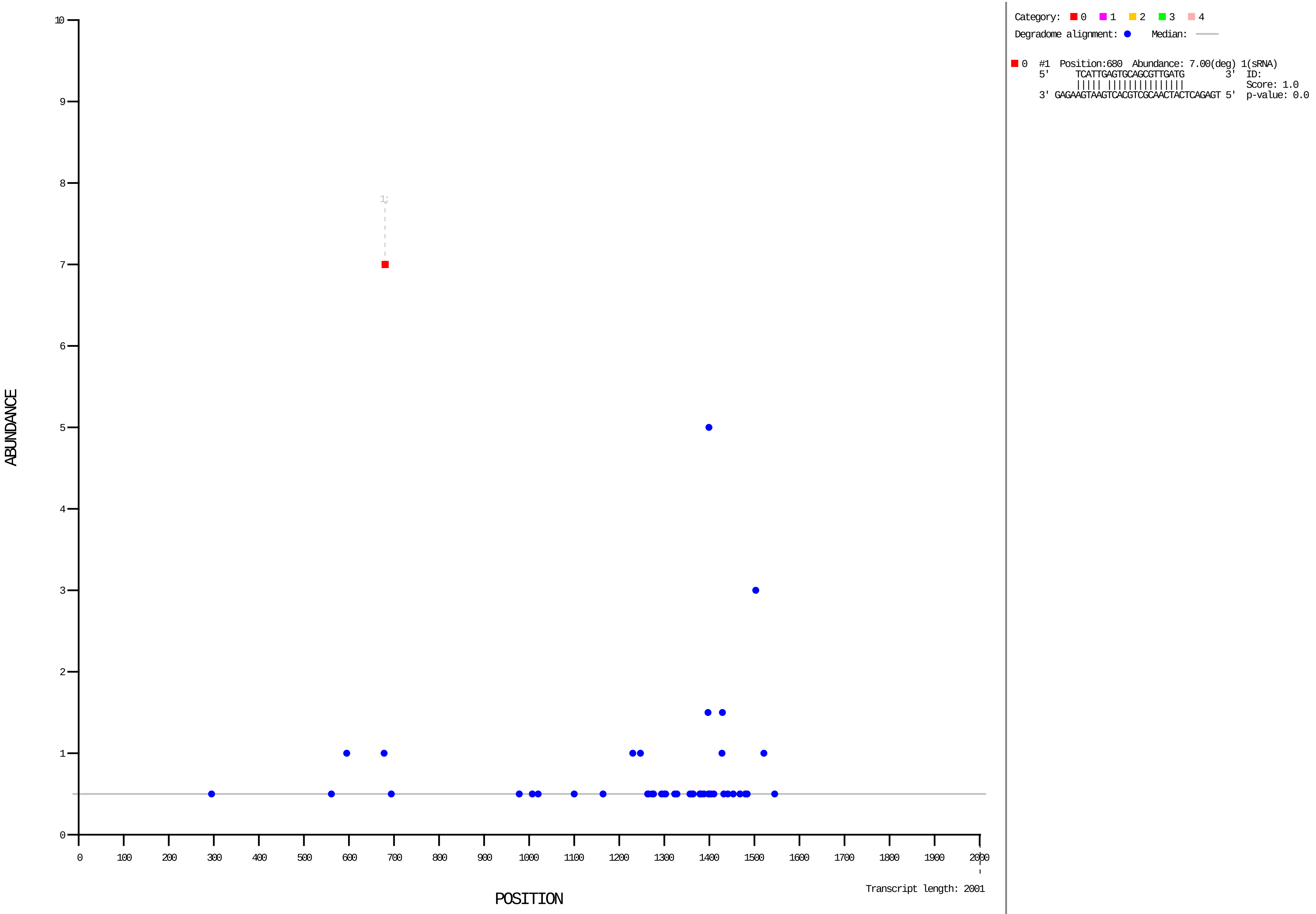

Cs1g09370.1 gene=Cs1g09370 CDS=1-921

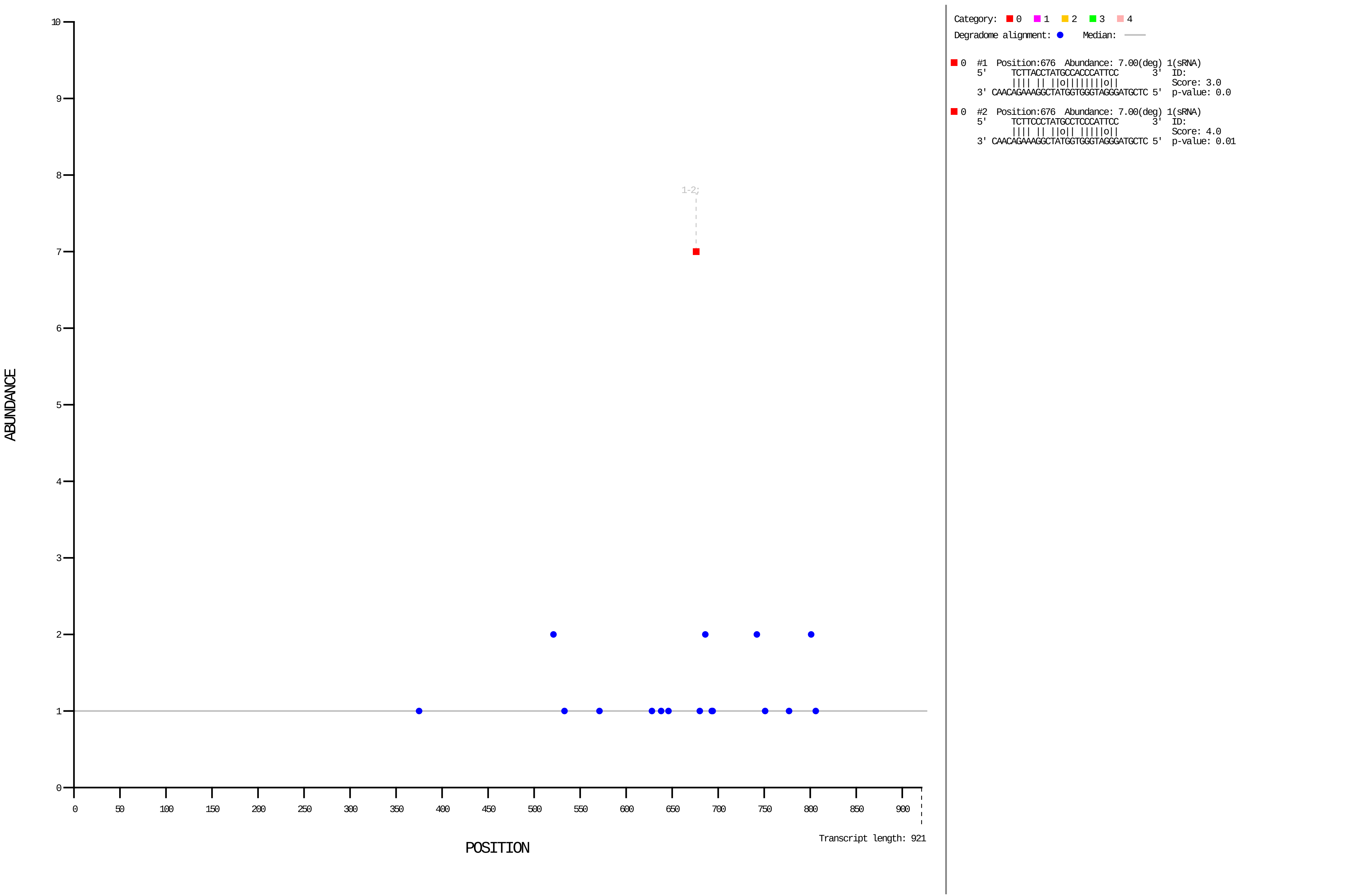

Cs1g03790.1 gene=Cs1g03790 CDS=911-1666

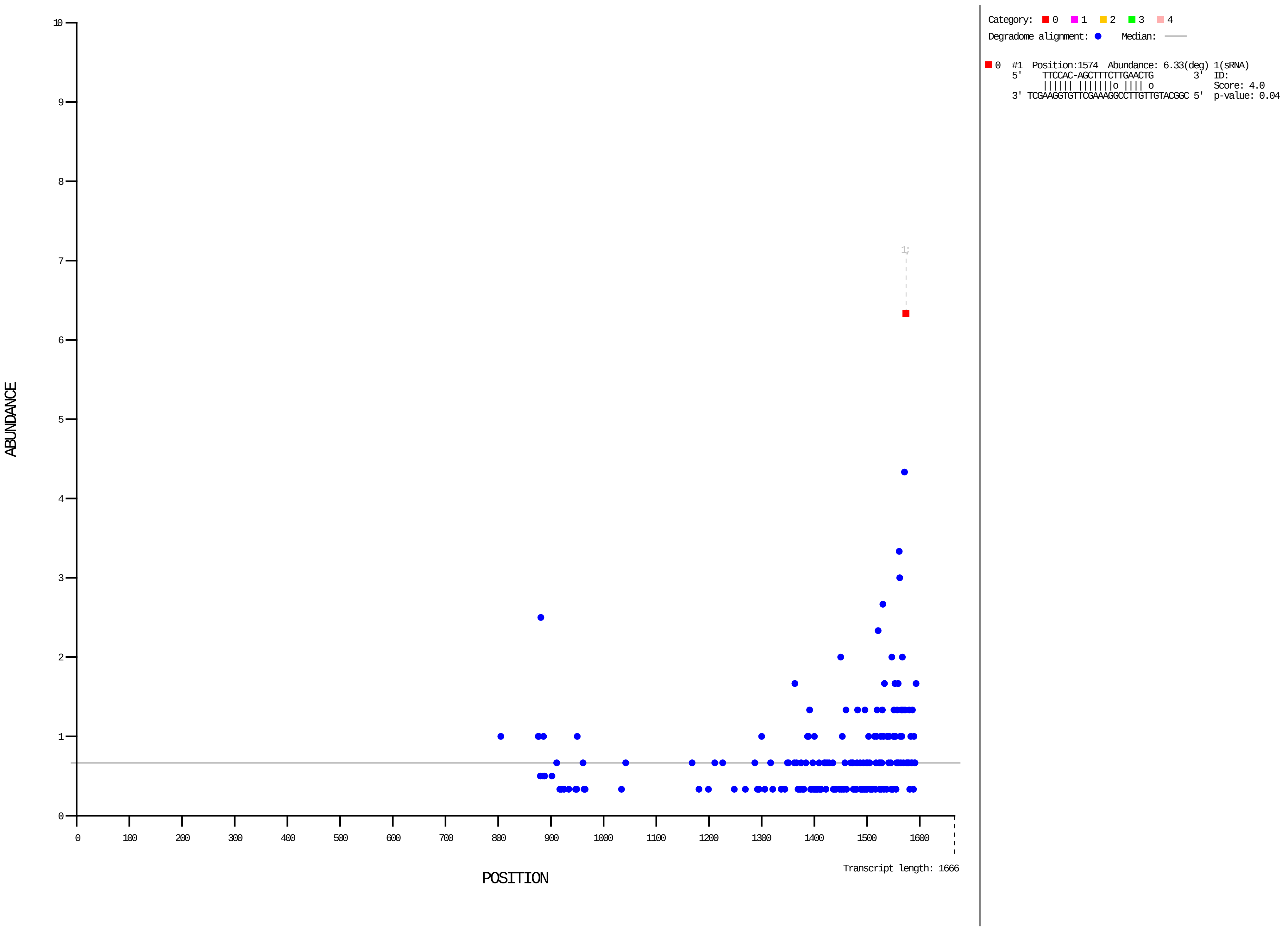

Cs2g15130.1 gene=Cs2g15130 CDS=1007-3703

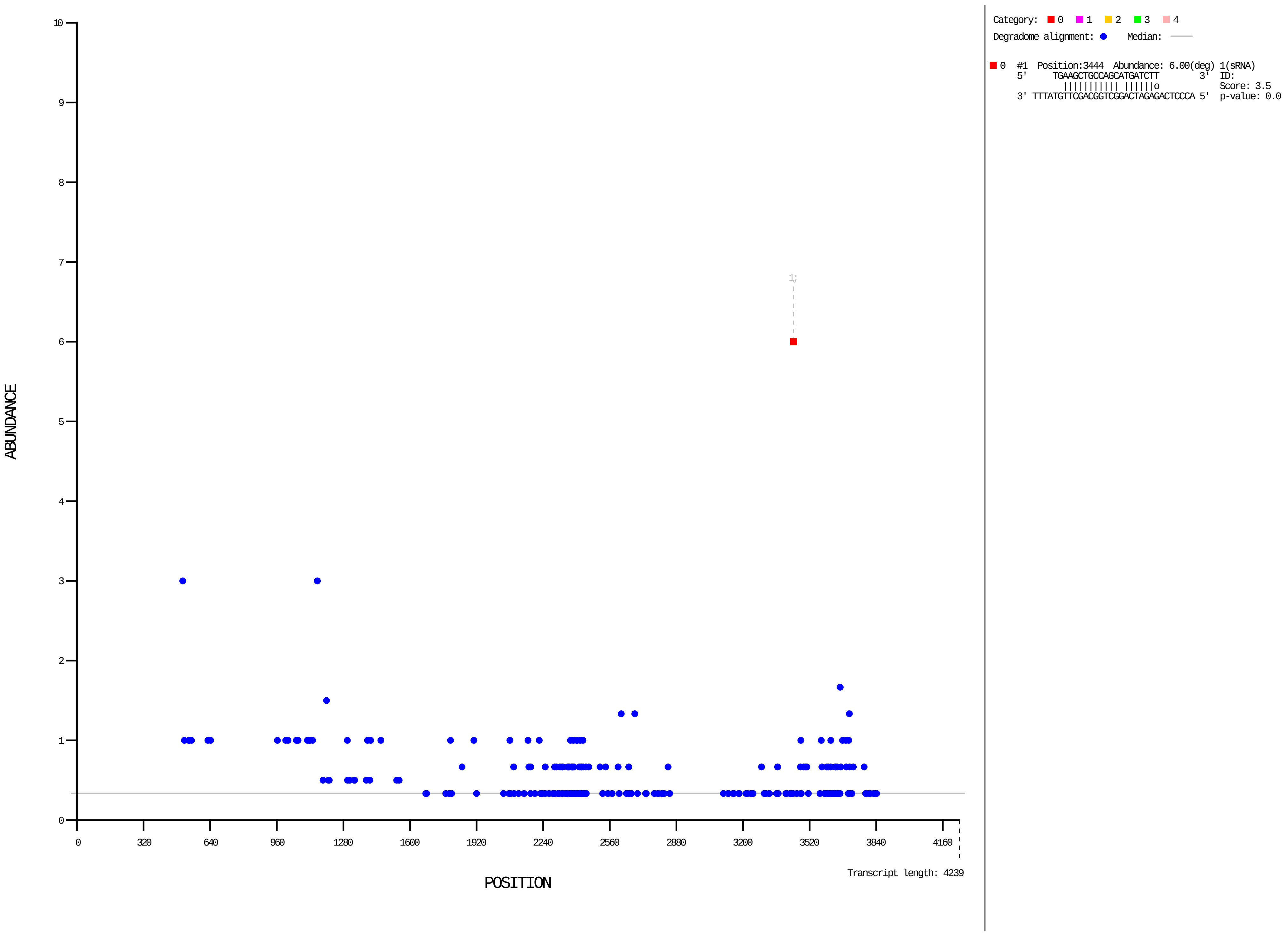

orange1.1t01362.1 gene=orange1.1t01362 CDS=205-2940

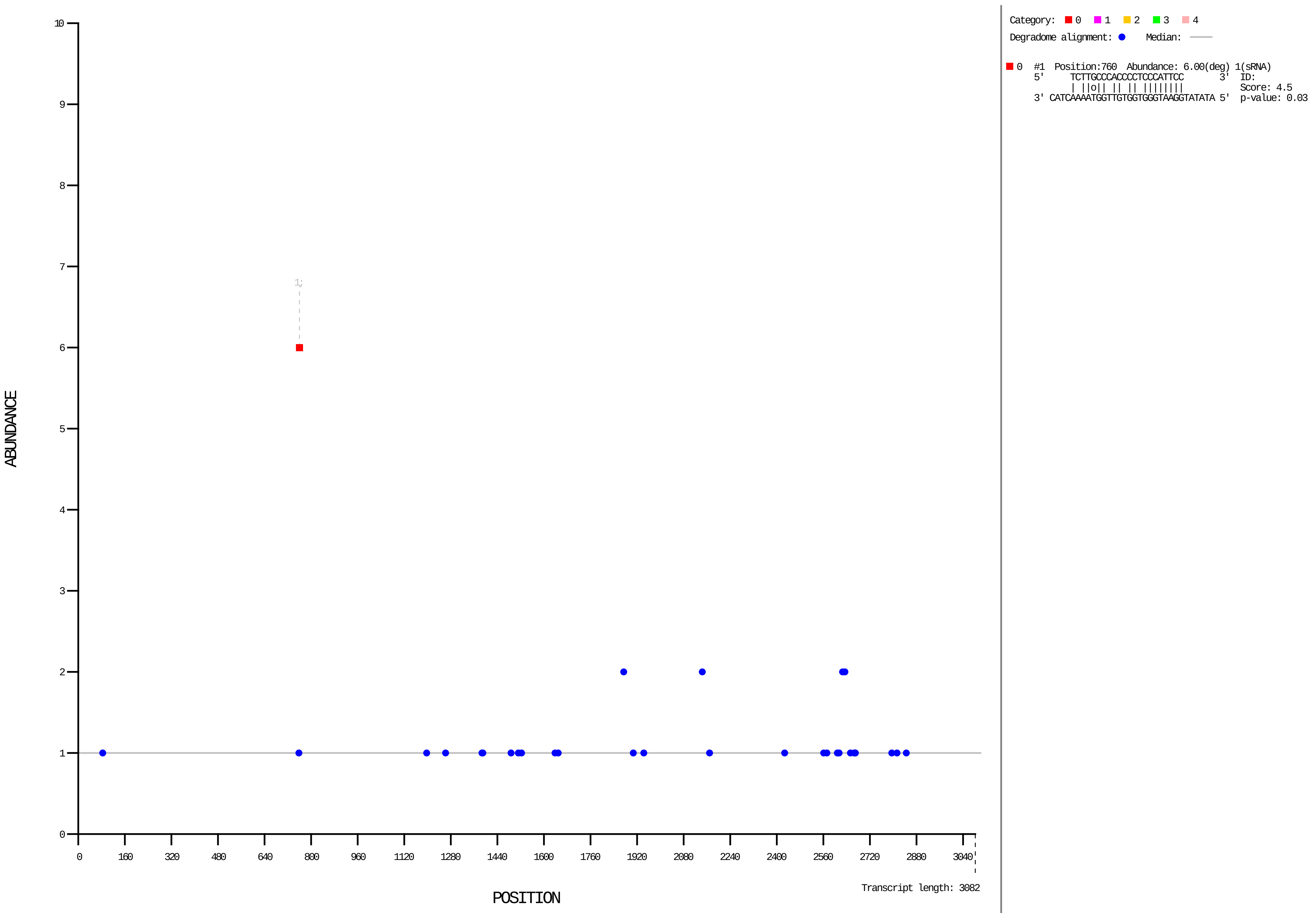

Cs6g03500.1 gene=Cs6g03500 CDS=289-4494

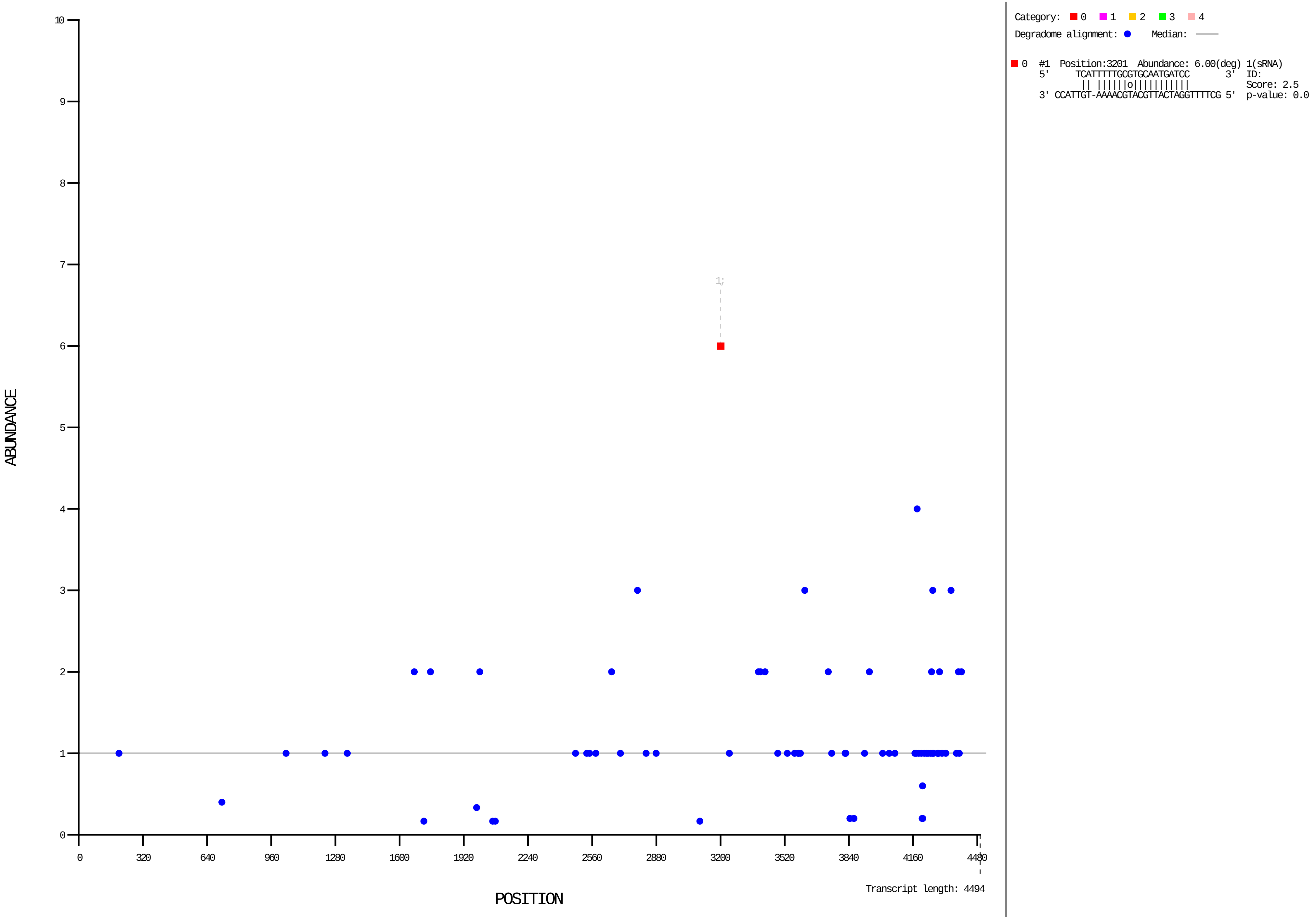

Cs2g15130.2 gene=Cs2g15130 CDS=135-2582

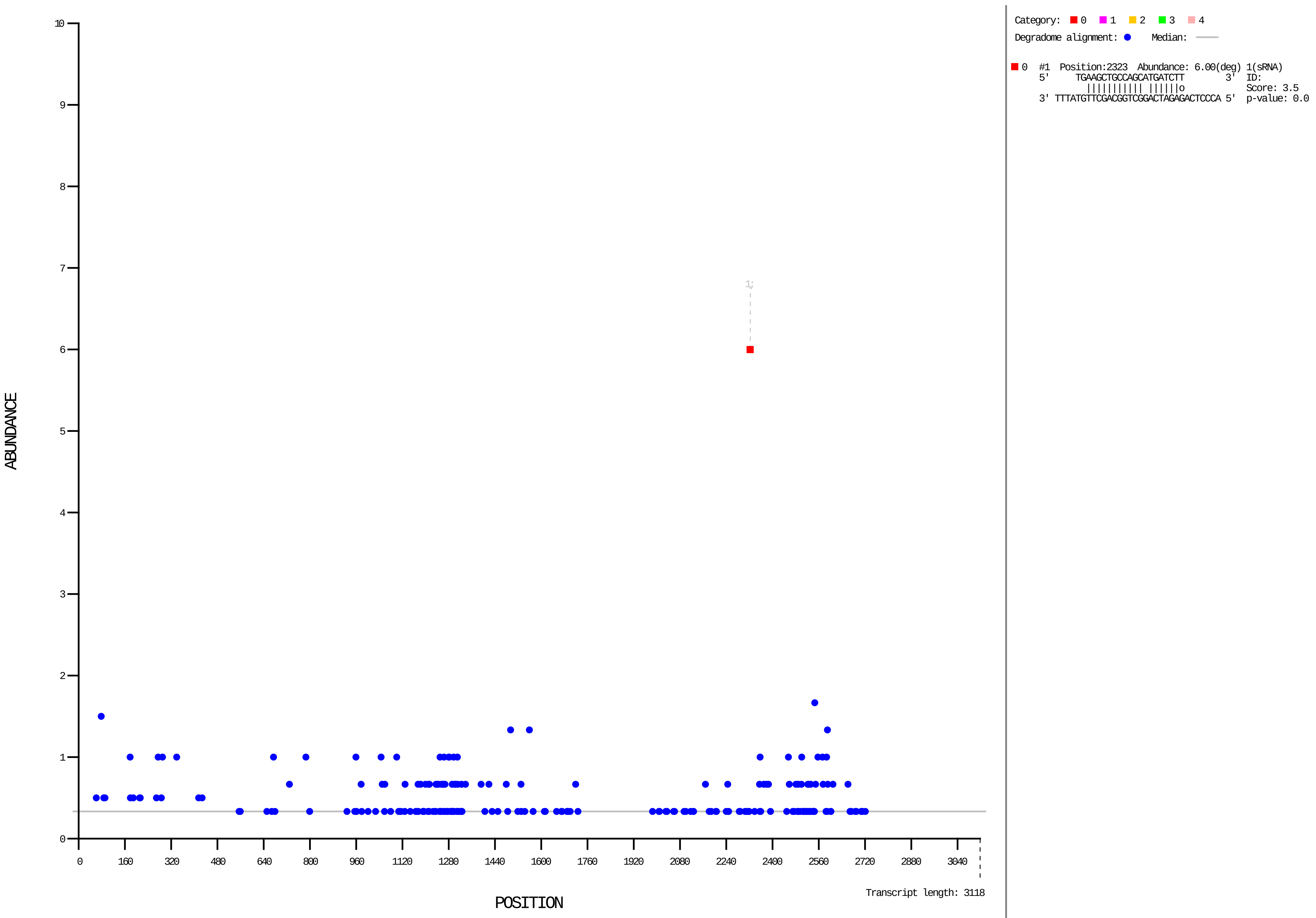

Cs2g15130.3 gene=Cs2g15130 CDS=243-2234

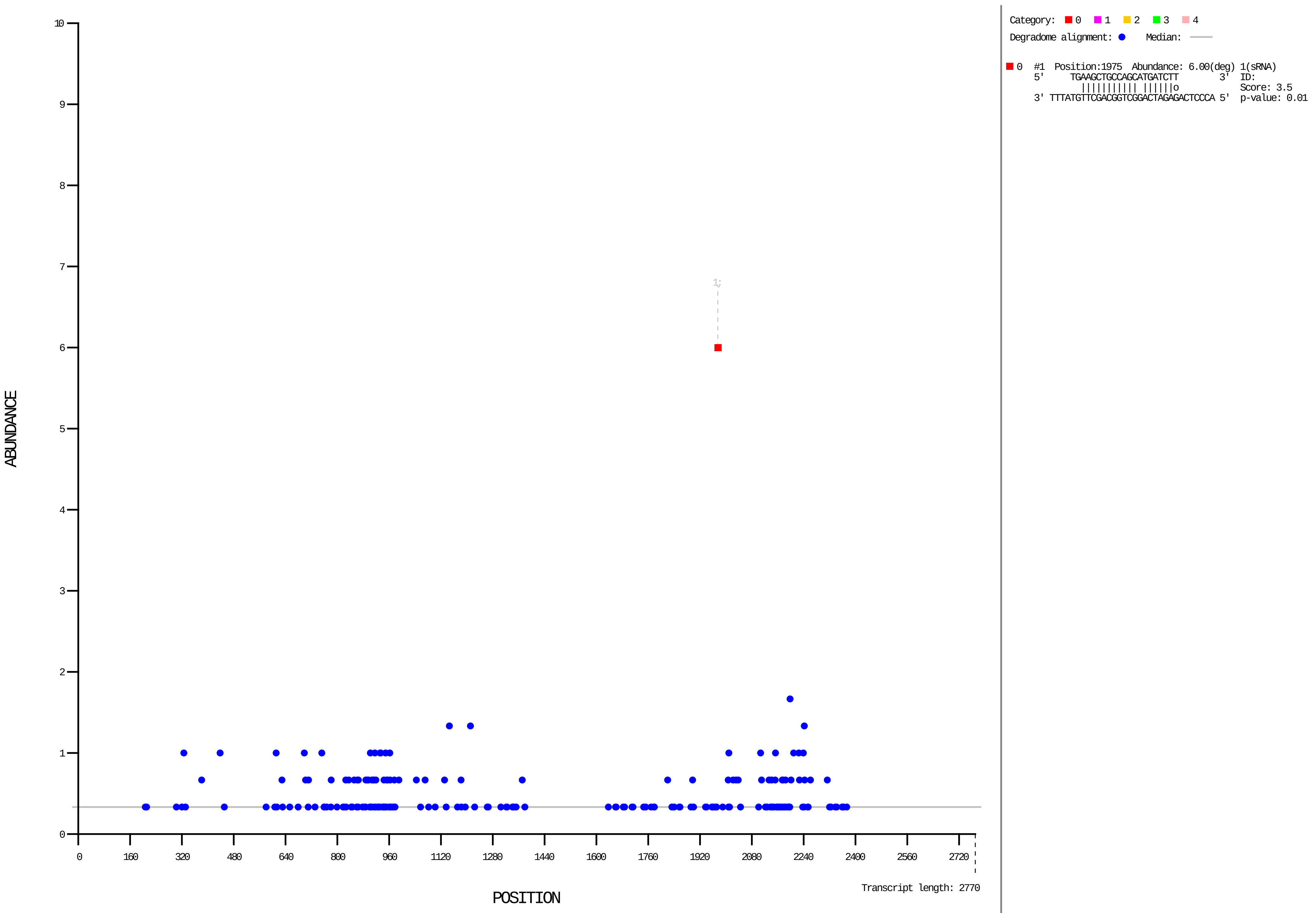

orange1.1t01829.2 gene=orange1.1t01829 CDS=135-3863

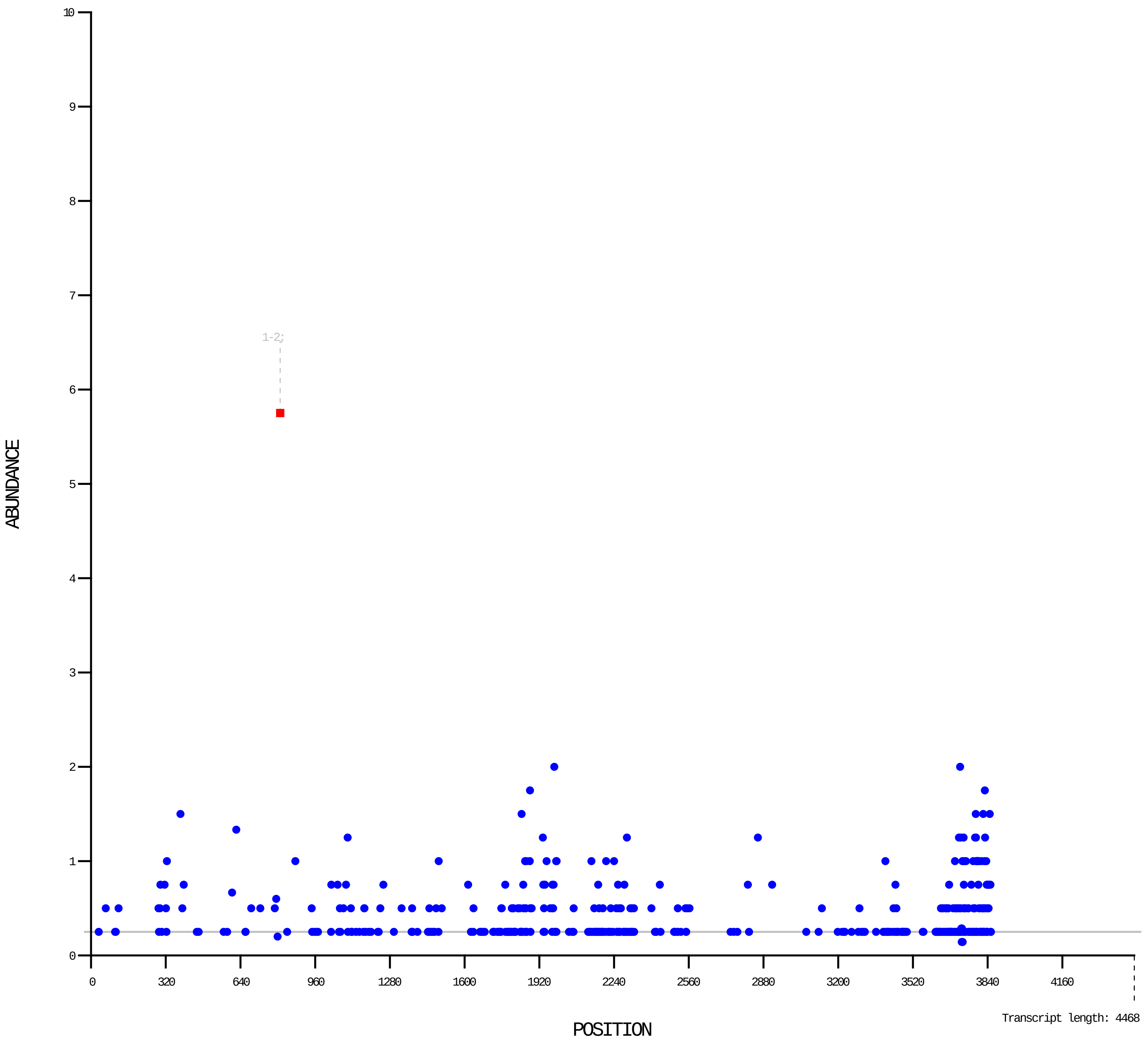

Category: 0 1 2 3 4
Degradome alignment: Median:

0 #1 Position:810 Abundance: 5.75(deg) 1(sRNA)
5' TCTTCCCTATGCCTCCCATTCC 3' ID:
|||| |||||o|||||||| || Score: 2.5
3' CAACAGAATGGATATGGAGGGTATGGTGTCTA 5' p-value: 0.0

0 #2 Position:810 Abundance: 5.75(deg) 1(sRNA)
5' TCTTACCTATGCCACCCATTCC 3' ID:
||||||||||o|| ||||| || Score: 2.5
3' CAACAGAATGGATATGGAGGGTATGGTGTCTA 5' p-value: 0.0

orange1.1t01829.3 gene=orange1.1t01829 CDS=135-3863

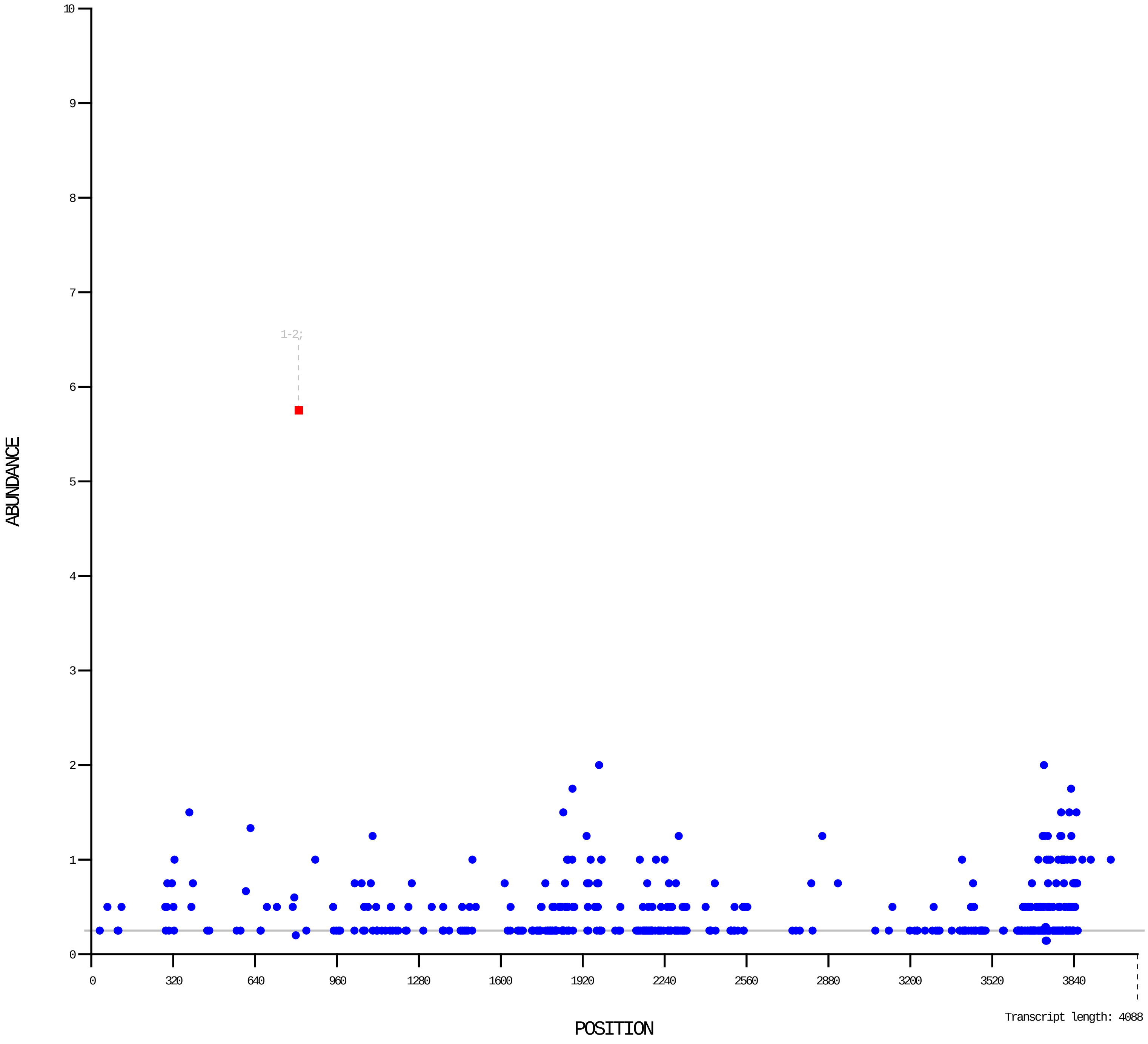

Category: 0 1 2 3 4

Degradome alignment: Median:

0 #1 Position:810 Abundance: 5.75(deg) 1(sRNA)

```
5'     TCTTCCCTATGCCTCCCATTCC       3'  ID:
       |||| |||||o|||||||| ||           Score: 2.5
3' CAACAGAATGGATATGGAGGGTATGGTGTCTA 5'  p-value: 0.0
```

0 #2 Position:810 Abundance: 5.75(deg) 1(sRNA)

```
5'     TCTTACCTATGCCACCCATTCC       3'  ID:
       ||||||||||o|| ||||| ||           Score: 2.5
3' CAACAGAATGGATATGGAGGGTATGGTGTCTA 5'  p-value: 0.0
```

orange1.1t01829.4 gene=orange1.1t01829 CDS=135-3905

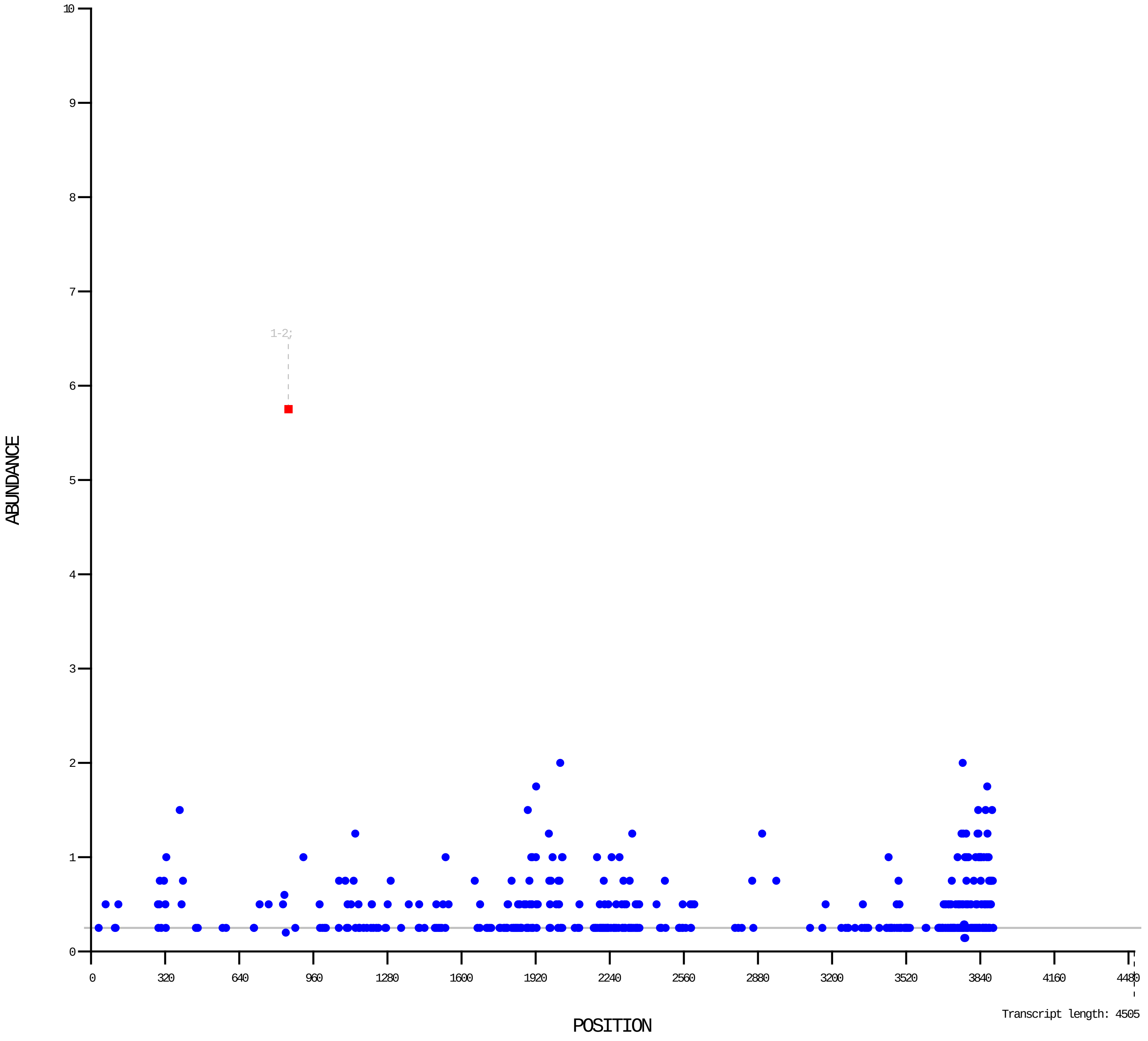

Category: 0 1 2 3 4
Degradome alignment: Median:

0 #1 Position:852 Abundance: 5.75(deg) 1(sRNA)
5' TCTTCCCTATGCCTCCCATTCC 3' ID:
|||| |||||o|||||||| || Score: 2.5
3' CAACAGAATGGATATGGAGGGTATGGTGTCTA 5' p-value: 0.0

0 #2 Position:852 Abundance: 5.75(deg) 1(sRNA)
5' TCTTACCTATGCCACCCATTCC 3' ID:
||||||||||o|| ||||| || Score: 2.5
3' CAACAGAATGGATATGGAGGGTATGGTGTCTA 5' p-value: 0.0

orange1.1t01829.1 gene=orange1.1t01829 CDS=135-3863

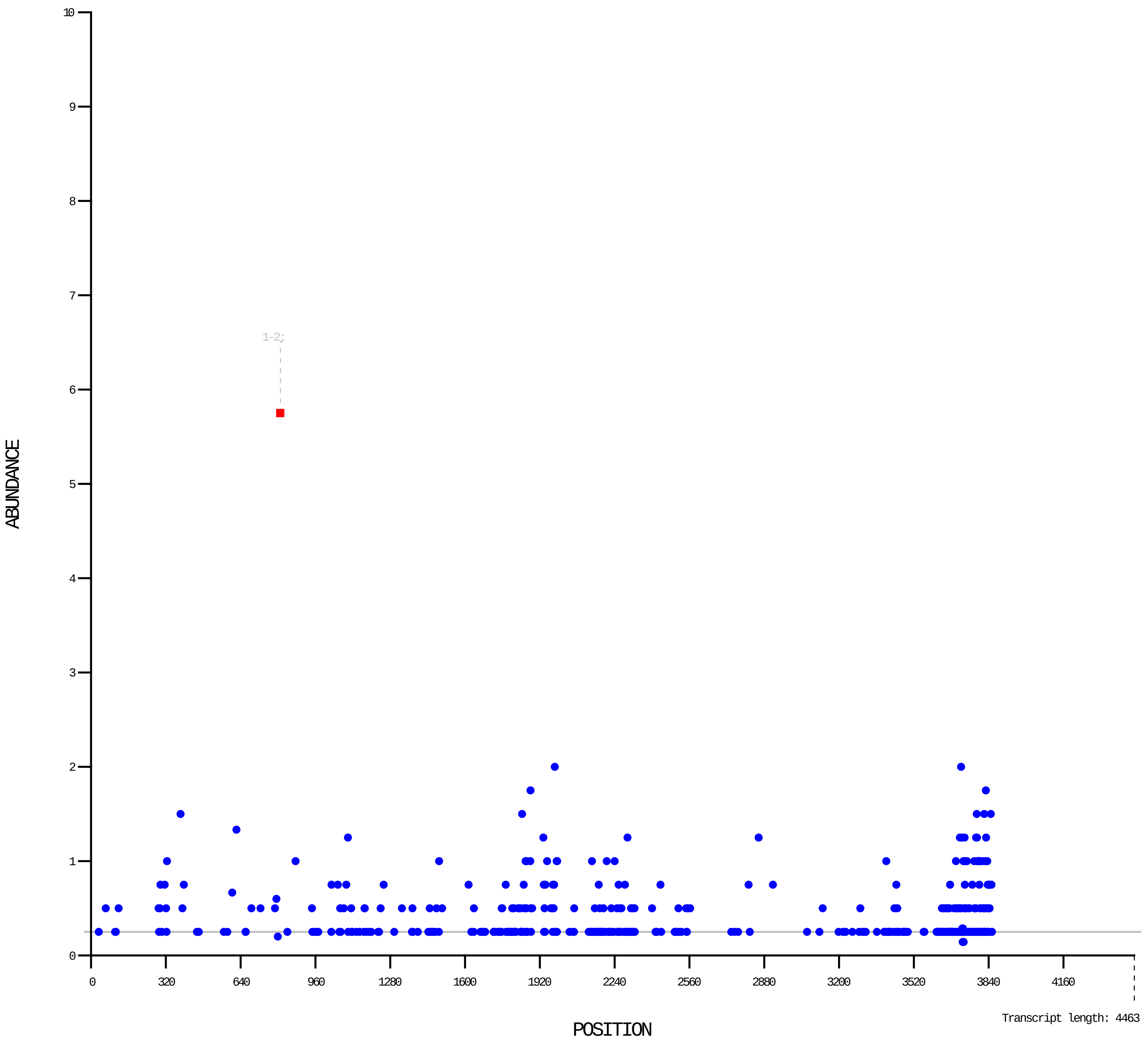

Category: 0 1 2 3 4
Degradome alignment: Median:

0 #1 Position:810 Abundance: 5.75(deg) 1(sRNA)
5' TCTTCCCTATGCCTCCCATTCC 3' ID:
|||| |||||o|||||||| ||
Score: 2.5
3' CAACAGAATGGATATGGAGGGTATGGTGTCTA 5' p-value: 0.0

0 #2 Position:810 Abundance: 5.75(deg) 1(sRNA)
5' TCTTACCTATGCCACCCATTCC 3' ID:
||||||||||o|| ||||| ||
Score: 2.5
3' CAACAGAATGGATATGGAGGGTATGGTGTCTA 5' p-value: 0.0

Cs5g22030.5 gene=Cs5g22030 CDS=36-4529

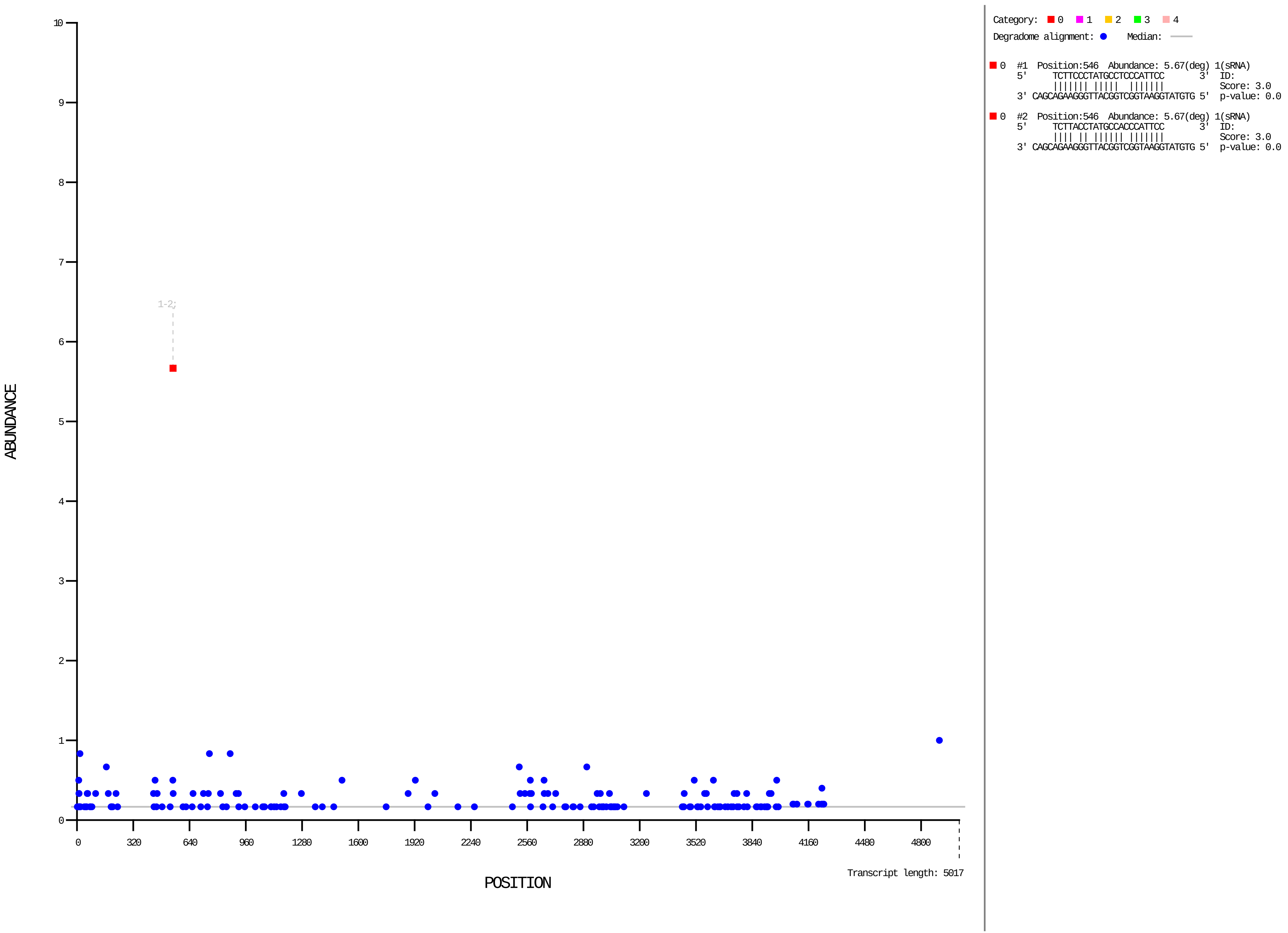

Cs5g22030.6 gene=Cs5g22030 CDS=36-4064

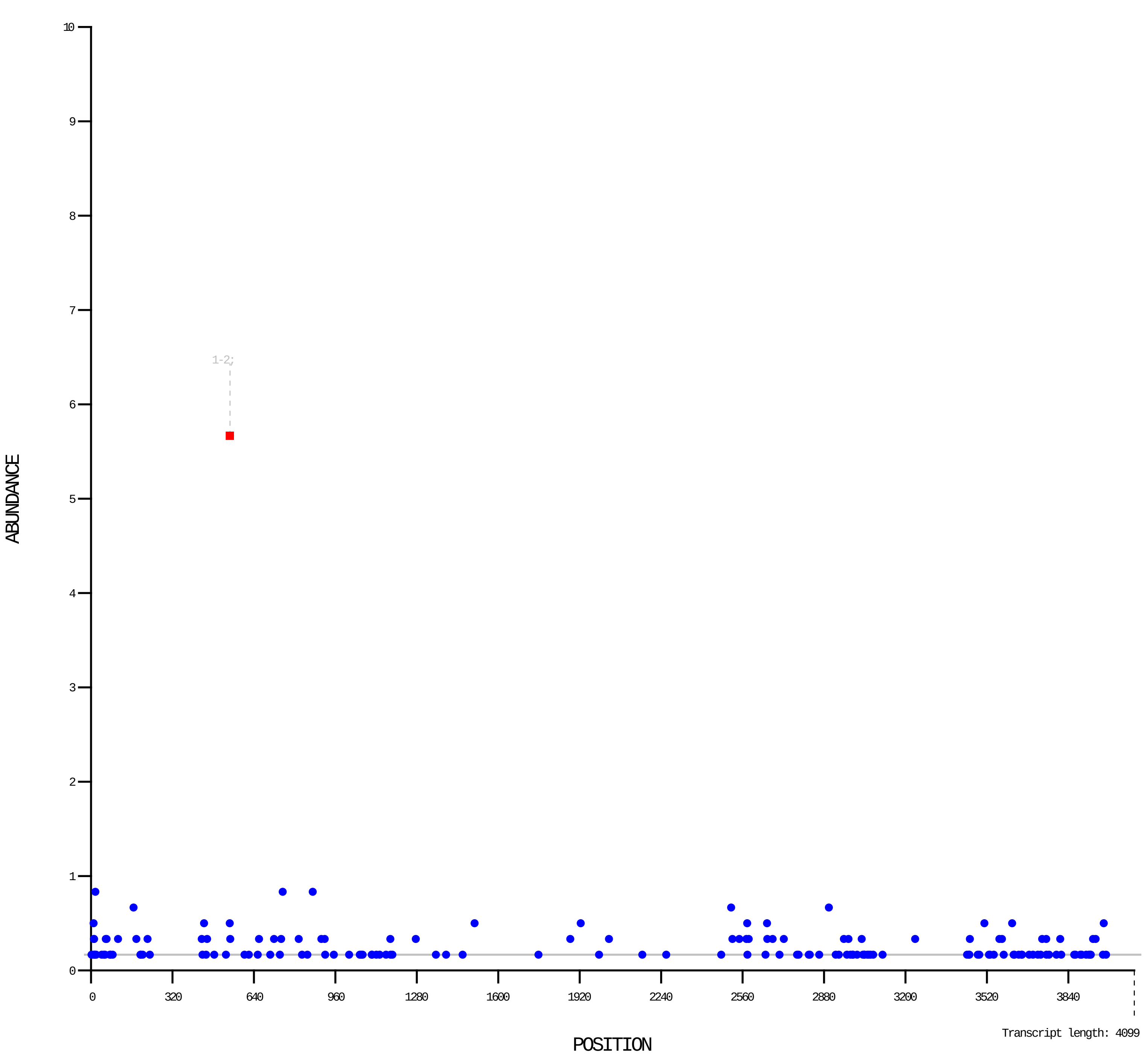

Category: 0 1 2 3 4

Degradome alignment: Median:

```
0  #1  Position:546  Abundance: 5.67(deg) 1(sRNA)
   5'     TCTTCCCTATGCCTCCCATTCC       3'  ID:
          ||||||| |||||  |||||||           Score: 3.0
   3' CAGCAGAAGGGTTACGGTCGGTAAGGTATGTG 5'  p-value: 0.0

0  #2  Position:546  Abundance: 5.67(deg) 1(sRNA)
   5'     TCTTACCTATGCCACCCATTCC       3'  ID:
          |||| || |||||| |||||||           Score: 3.0
   3' CAGCAGAAGGGTTACGGTCGGTAAGGTATGTG 5'  p-value: 0.0
```

Cs5g22030.2 gene=Cs5g22030 CDS=36-4454

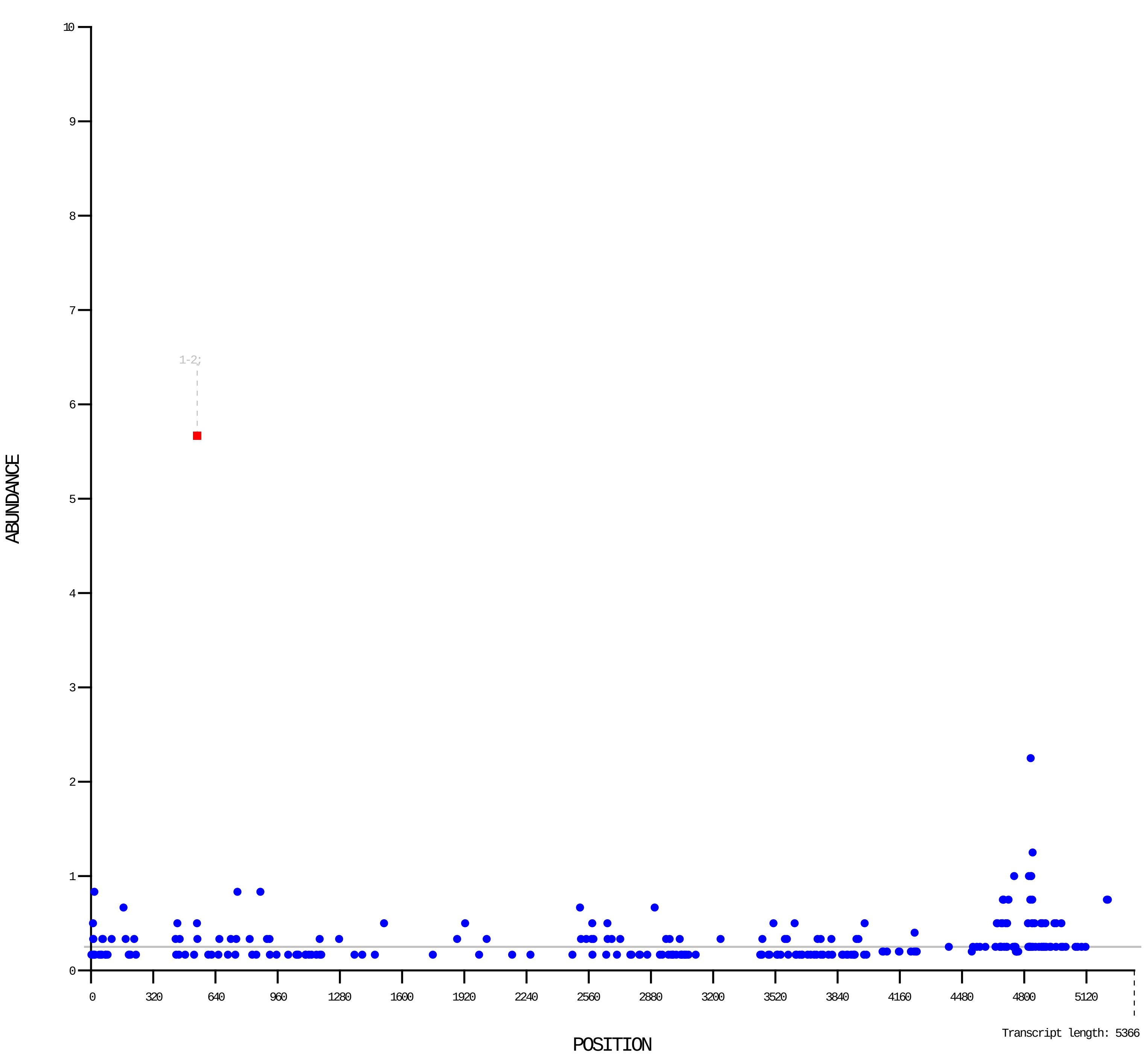

Category: 0 1 2 3 4
Degradome alignment: Median:

0 #1 Position:546 Abundance: 5.67(deg) 1(sRNA)
5' TCTTCCCTATGCCTCCCATTCC 3' ID:
||||||| ||||| ||||||| Score: 3.0
3' CAGCAGAAGGGTTACGGTCGGTAAGGTATGTG 5' p-value: 0.0

0 #2 Position:546 Abundance: 5.67(deg) 1(sRNA)
5' TCTTACCTATGCCACCCATTCC 3' ID:
|||| || |||||| ||||||| Score: 3.0
3' CAGCAGAAGGGTTACGGTCGGTAAGGTATGTG 5' p-value: 0.0

Cs5g22030.1 gene=Cs5g22030 CDS=36-4808

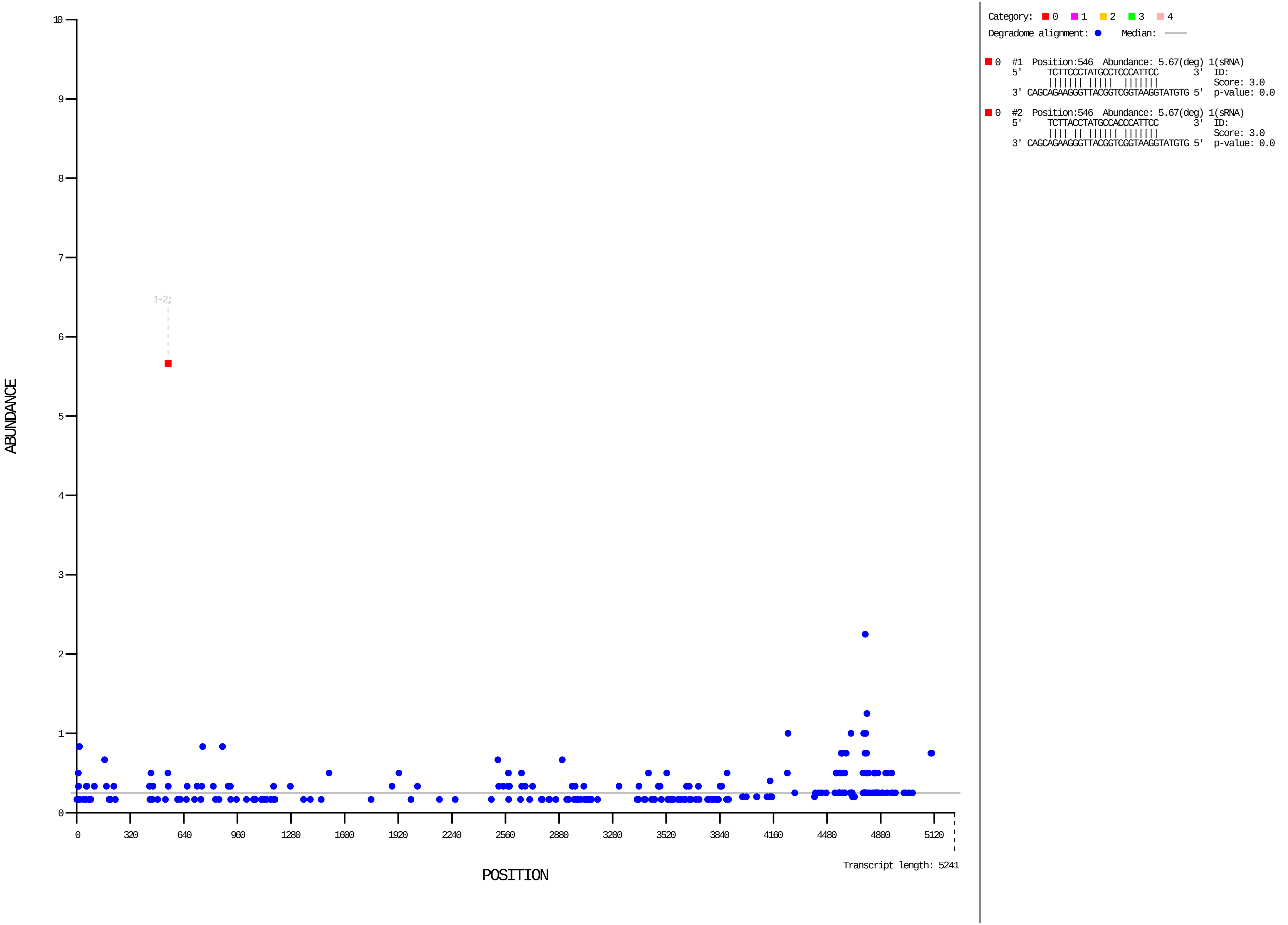

Cs5g22030.3 gene=Cs5g22030 CDS=36-4382

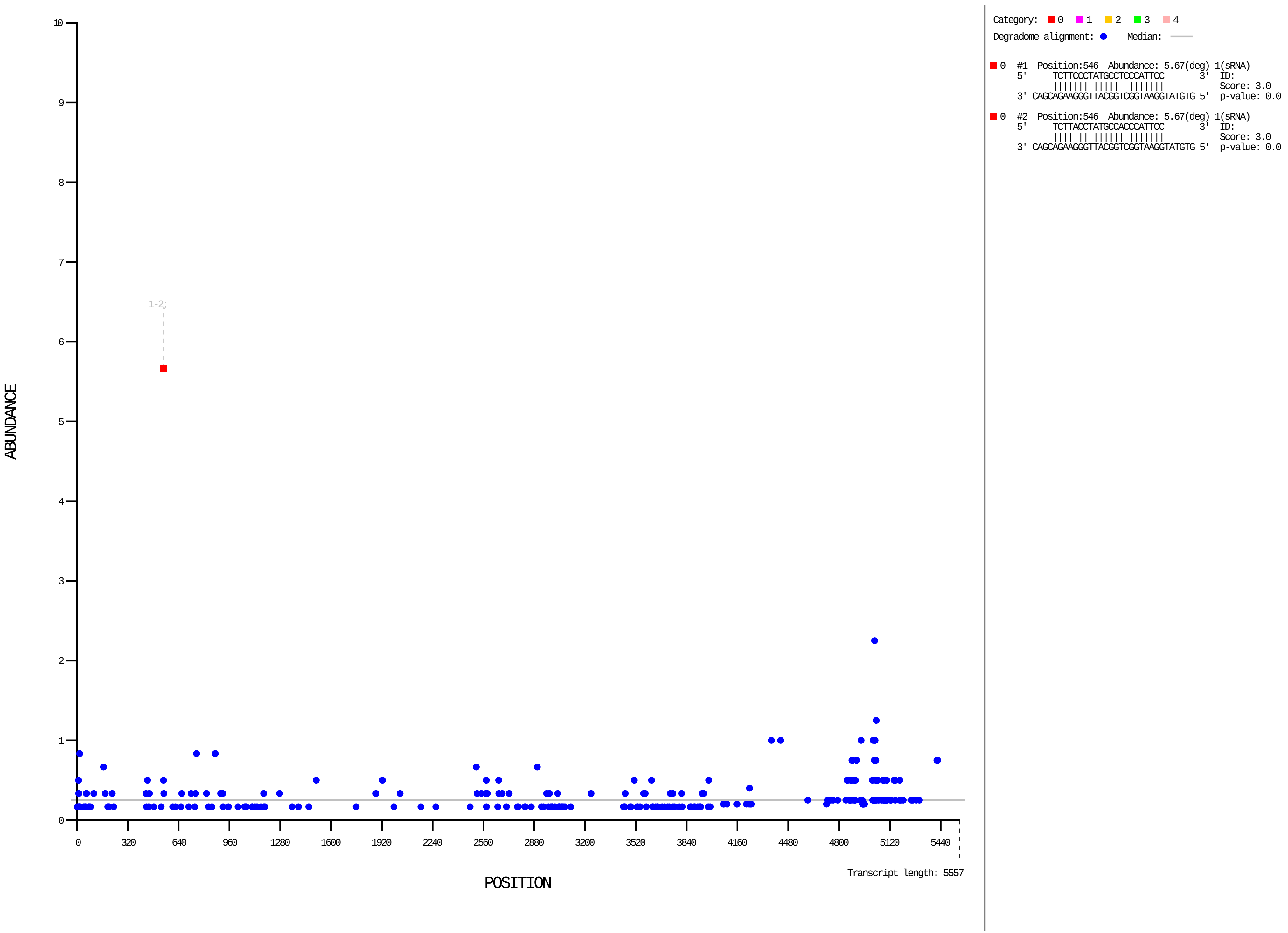

Cs5g22030.4 gene=Cs5g22030 CDS=36-4904

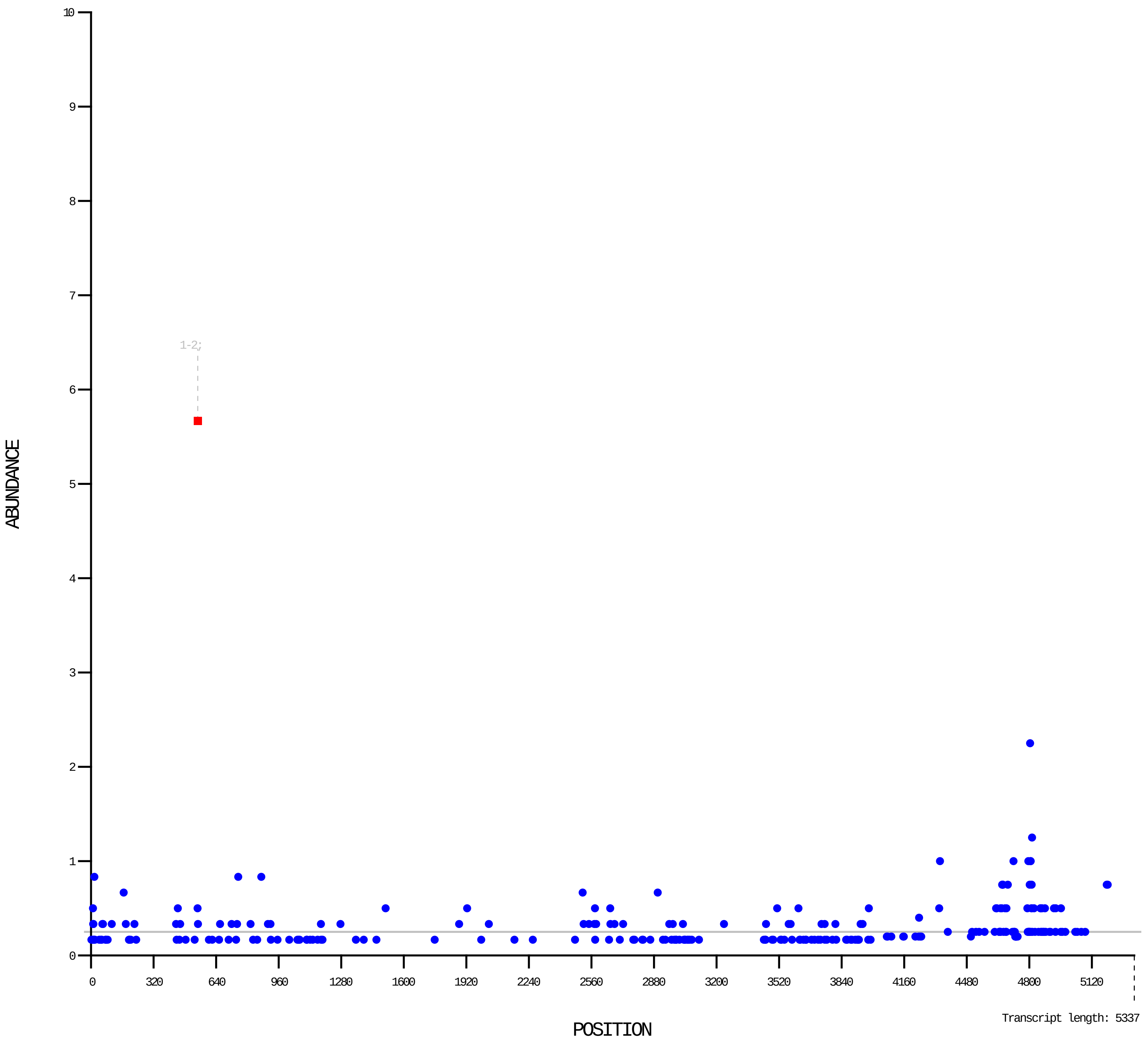

Category: 0 1 2 3 4
Degradome alignment: Median:

0 #1 Position:546 Abundance: 5.67(deg) 1(sRNA)
5' TCTTCCCTATGCCTCCCATTCC 3' ID:
||||||| ||||| ||||||| Score: 3.0
3' CAGCAGAAGGGTTACGGTCGGTAAGGTATGTG 5' p-value: 0.0

0 #2 Position:546 Abundance: 5.67(deg) 1(sRNA)
5' TCTTACCTATGCCACCCATTCC 3' ID:
|||| || |||||| ||||||| Score: 3.0
3' CAGCAGAAGGGTTACGGTCGGTAAGGTATGTG 5' p-value: 0.0

orange1.1t02555.1 gene=orange1.1t02555 CDS=289-1848

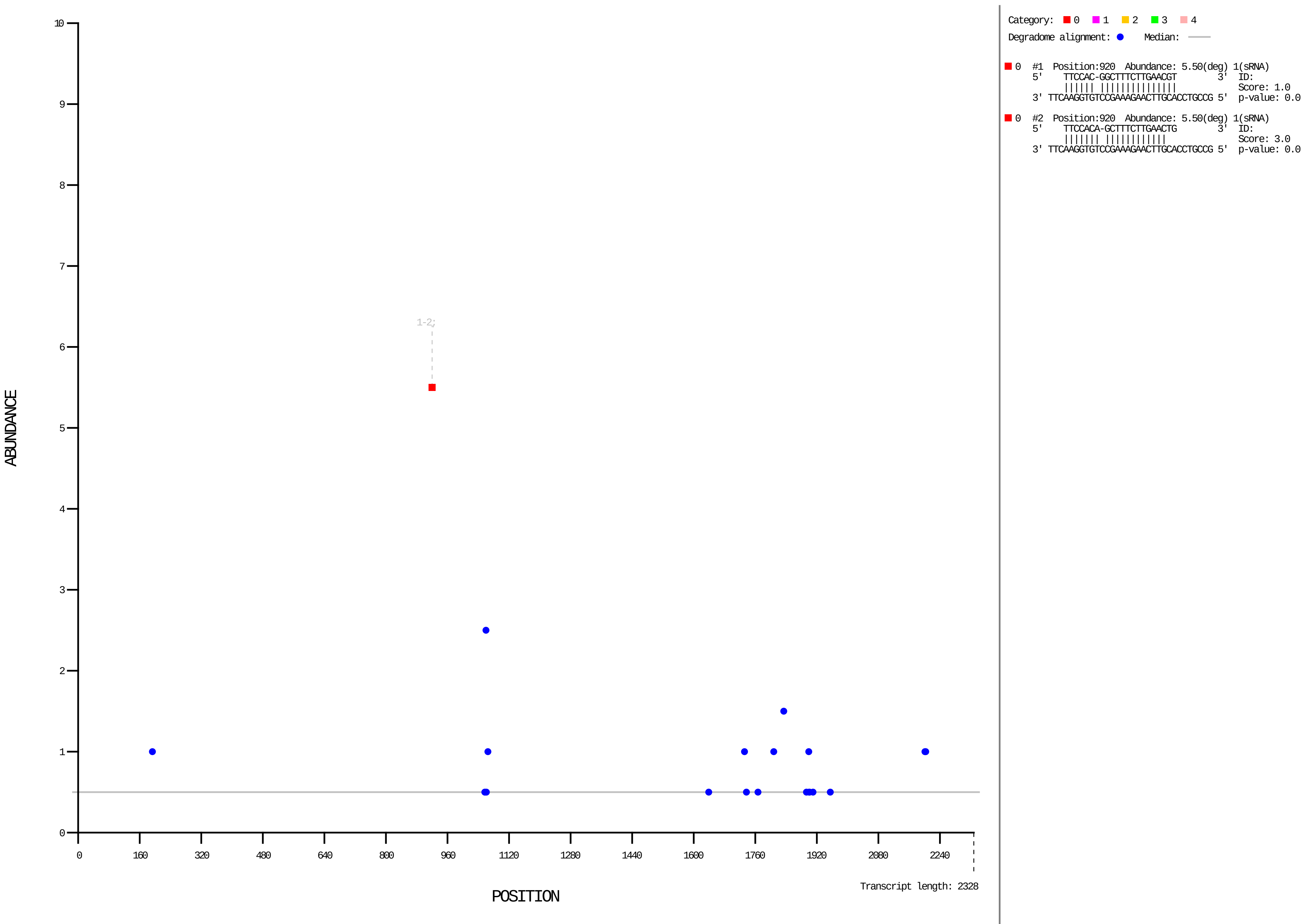

orange1.1t05193.1 gene=orange1.1t05193 CDS=1-1208

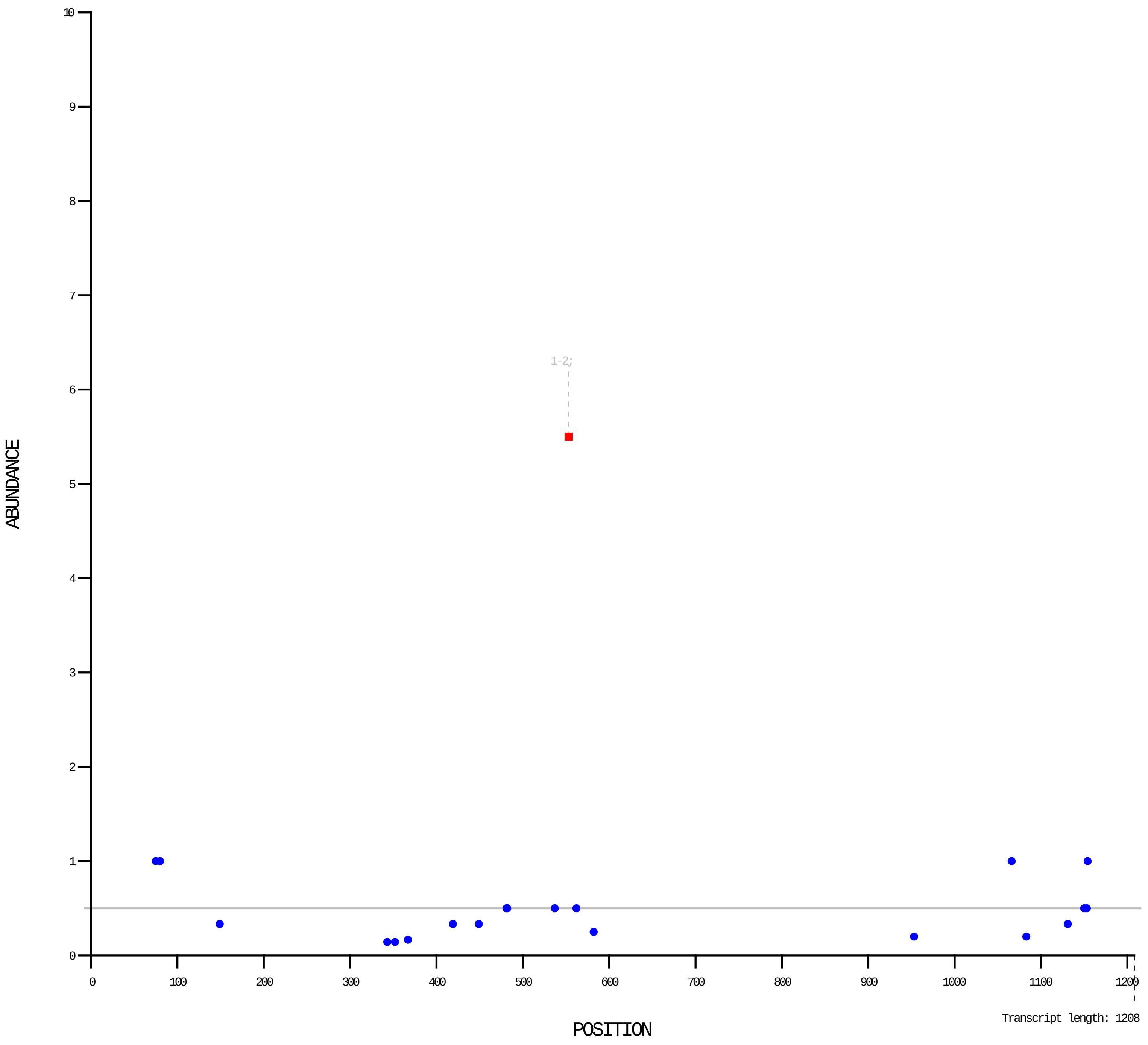

Category: 0 1 2 3 4
Degradome alignment: Median:

```
0   #1  Position:553  Abundance: 5.50(deg) 1(sRNA)
    5'      TCTTCCCTATGCCTCCCATTCC       3'  ID:
            |||| || ||||| ||||| ||           Score: 4.0
    3' CAGCAGAAAGGTTACGGCGGGTACGGCATGTG 5'  p-value: 0.01

0   #2  Position:553  Abundance: 5.50(deg) 1(sRNA)
    5'      TCTTACCTATGCCACCCATTCC       3'  ID:
            |||| || ||||| ||||| ||           Score: 4.0
    3' CAGCAGAAAGGTTACGGCGGGTACGGCATGTG 5'  p-value: 0.0
```

orange1.1t02555.2 gene=orange1.1t02555 CDS=289-1953

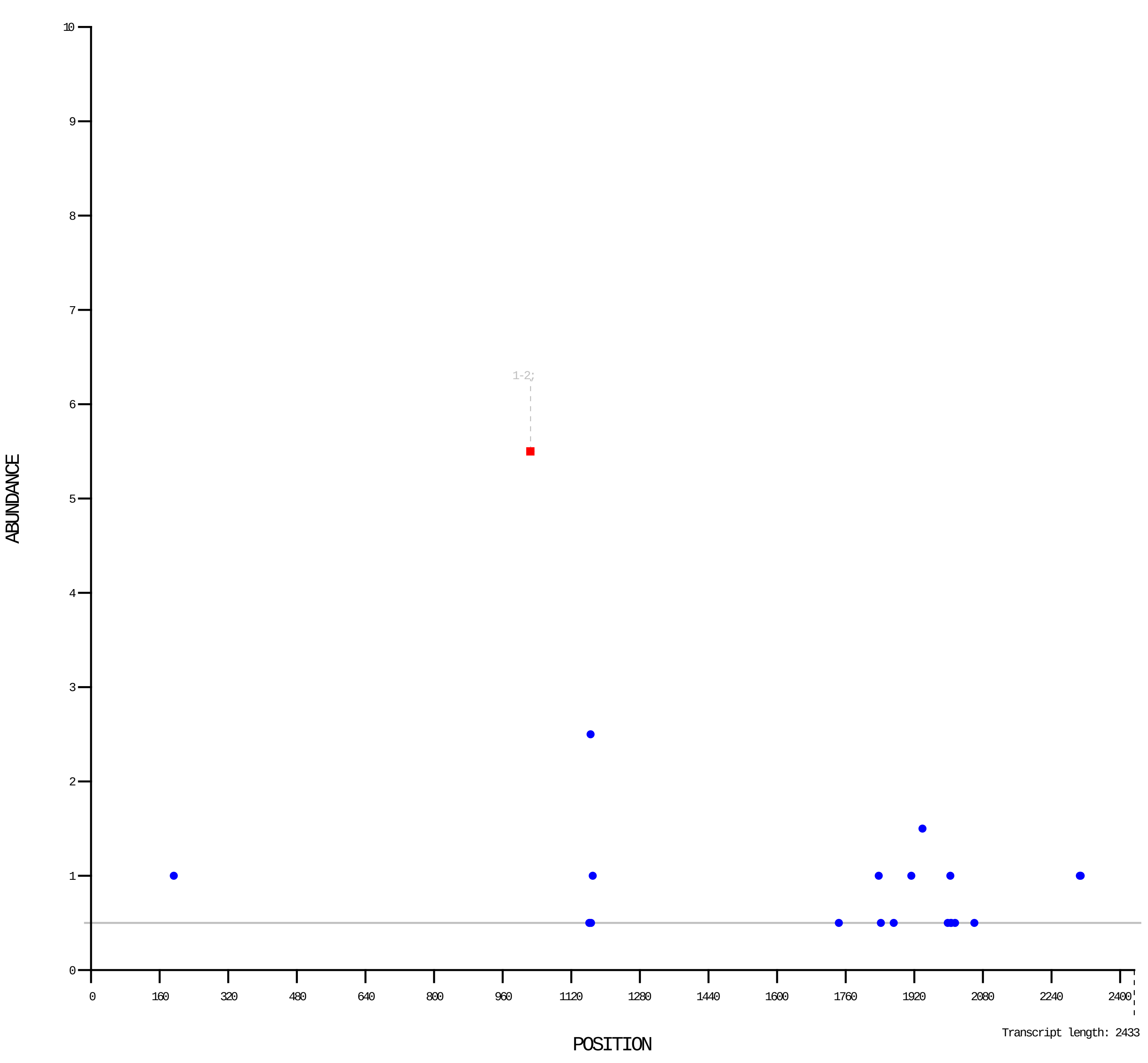


```
Category: 0 1 2 3 4
Degradome alignment:    Median:

0  #1  Position:1025  Abundance: 5.50(deg) 1(sRNA)
   5'    TTCCAC-GGCTTTCTTGAACGT         3'  ID:
         |||||| |||||||||||||||             Score: 1.0
   3' TTCAAGGTGTCCGAAAGAACTTGCACCTGCCG 5'  p-value: 0.0

0  #2  Position:1025  Abundance: 5.50(deg) 1(sRNA)
   5'    TTCCACA-GCTTTCTTGAACTG         3'  ID:
         ||||||| ||||||||||||               Score: 3.0
   3' TTCAAGGTGTCCGAAAGAACTTGCACCTGCCG 5'  p-value: 0.0
```

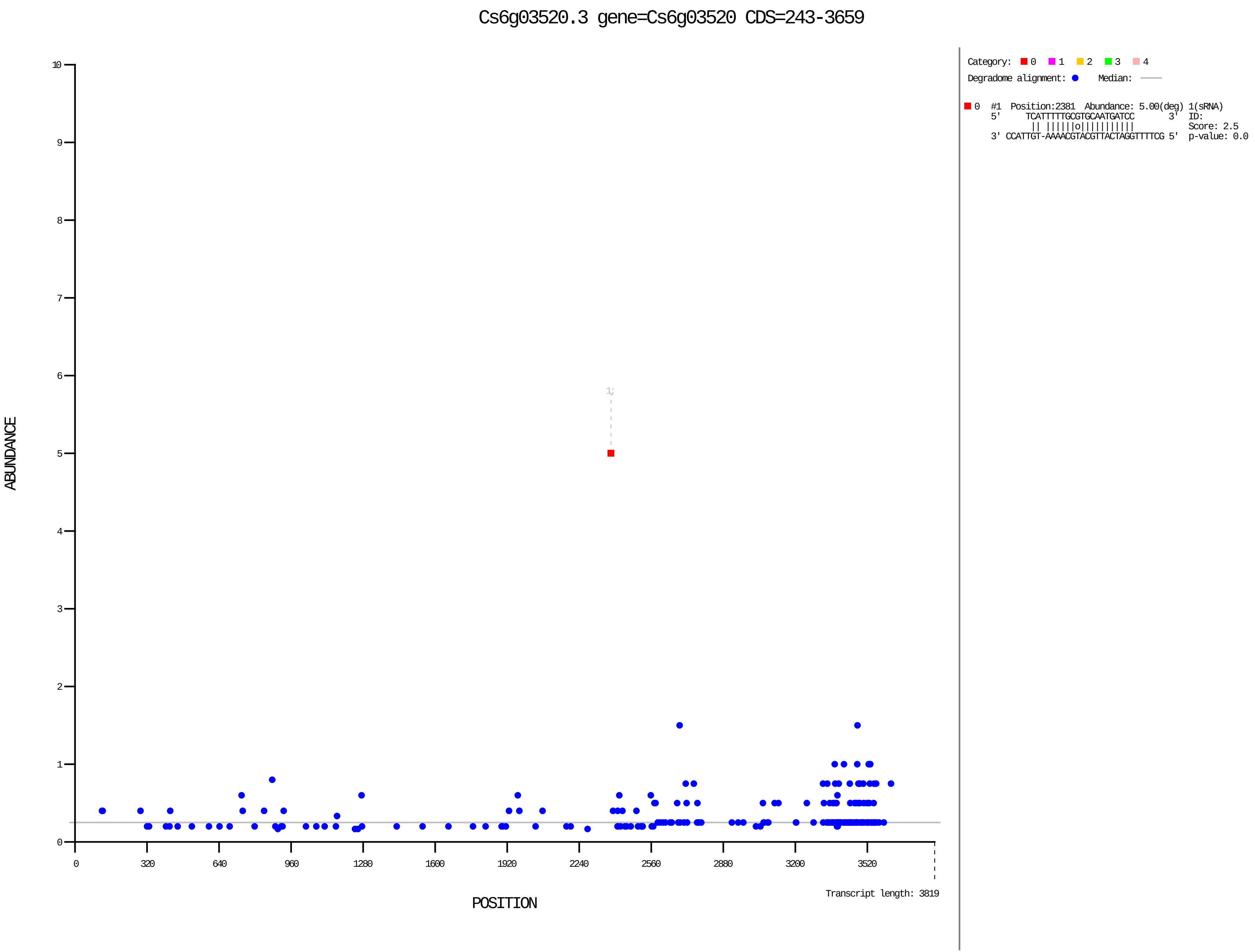
Cs6g03520.3 gene=Cs6g03520 CDS=243-3659
ABUNDANCE
POSITION
0 320 640 960 1280 1600 1920 2240 2560 2880 3200 3520
0 1 2 3 4 5 6 7 8 9 10
Transcript length: 3819
Category: 0 1 2 3 4
Degradome alignment:
Median:
0 #1 Position:2381 Abundance: 5.00(deg) 1(sRNA)
5' TCATTTTTGCGTGCAATGATCC 3' ID:
|| ||||||o||||||||||| Score: 2.5
3' CCATTGT-AAAACGTACGTTACTAGGTTTTCG 5' p-value: 0.0

Cs6g03520.4 gene=Cs6g03520 CDS=457-4275

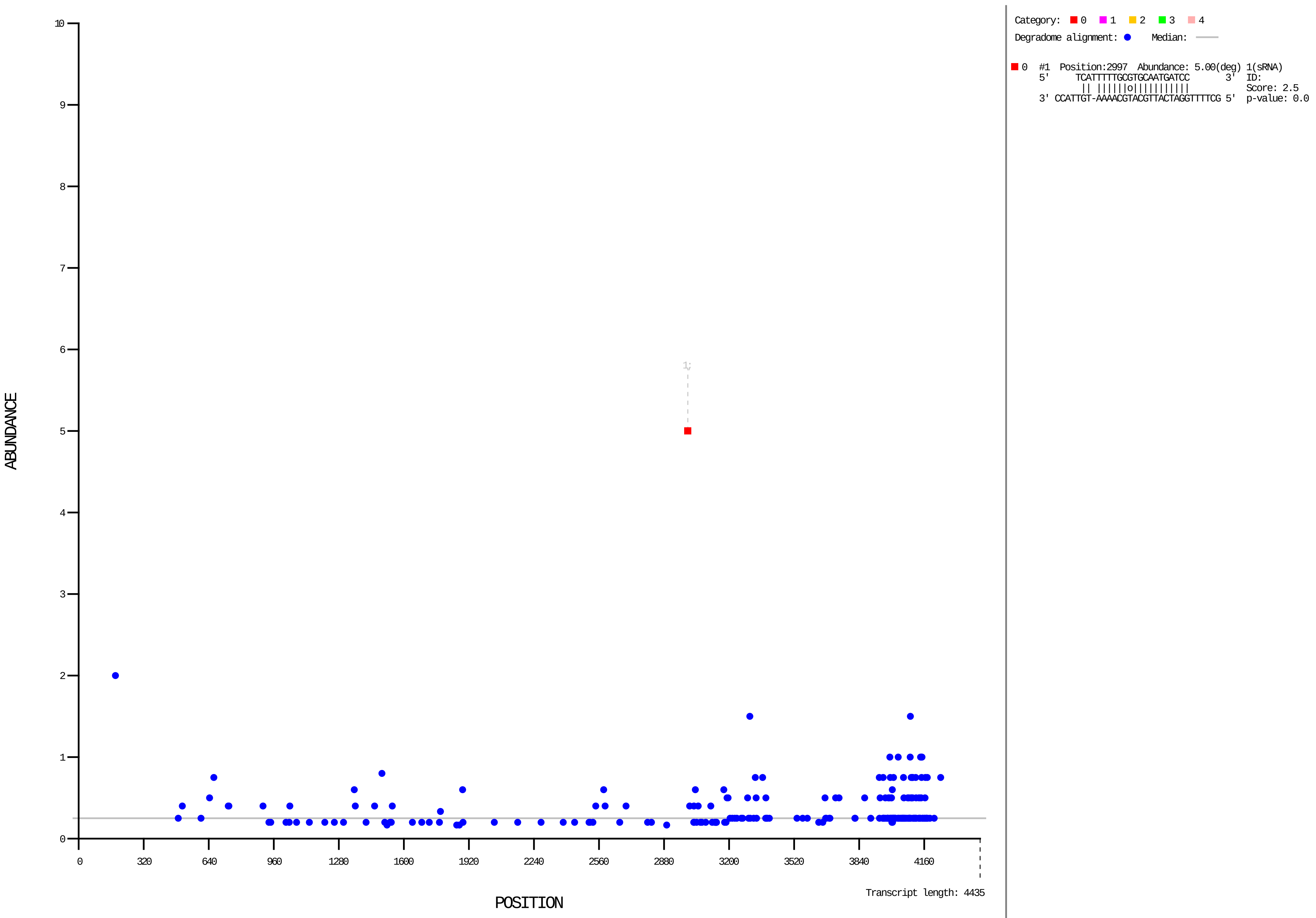

Cs6g03520.1 gene=Cs6g03520 CDS=223-4413

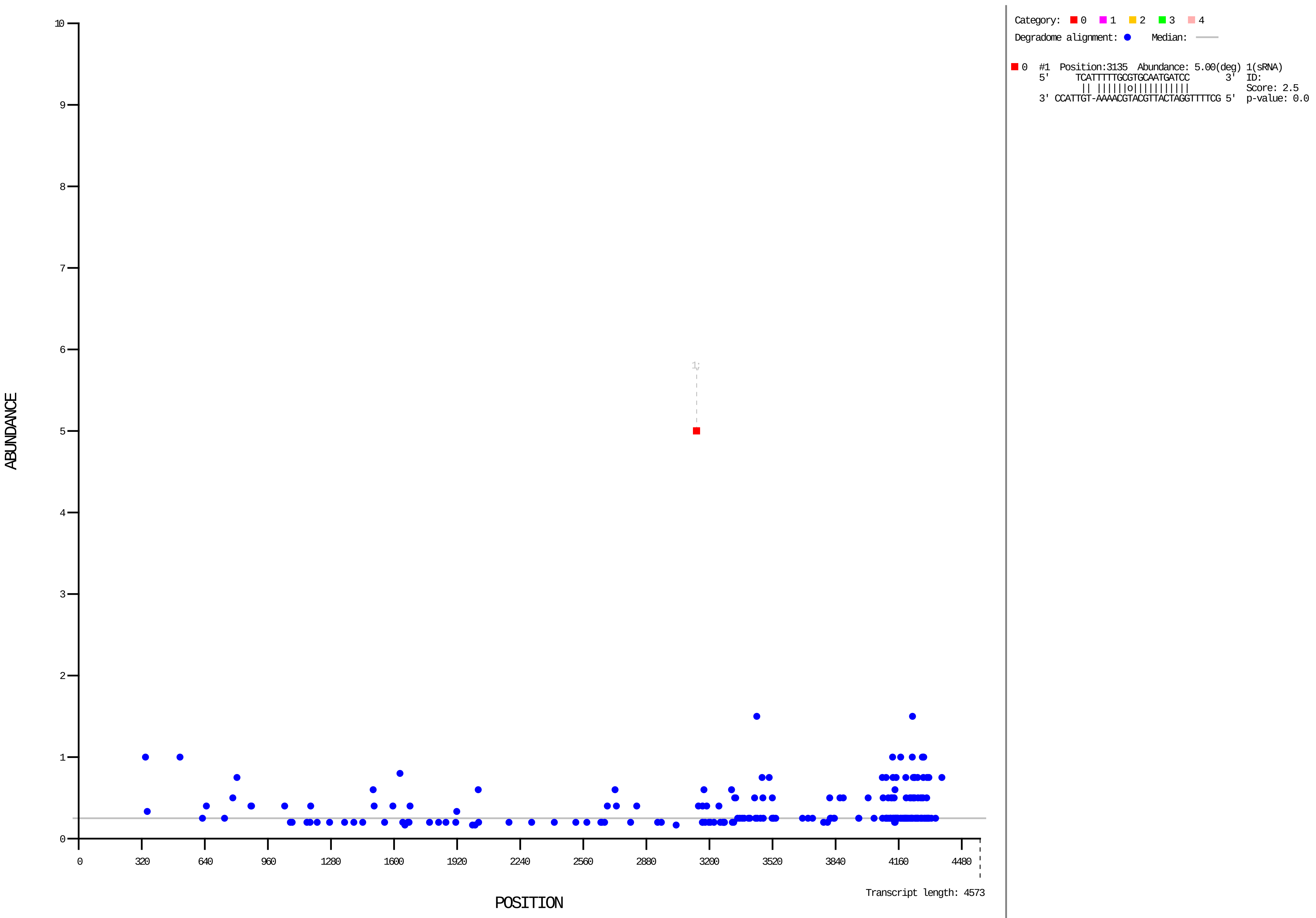

Cs2g10770.1 gene=Cs2g10770 CDS=192-3137

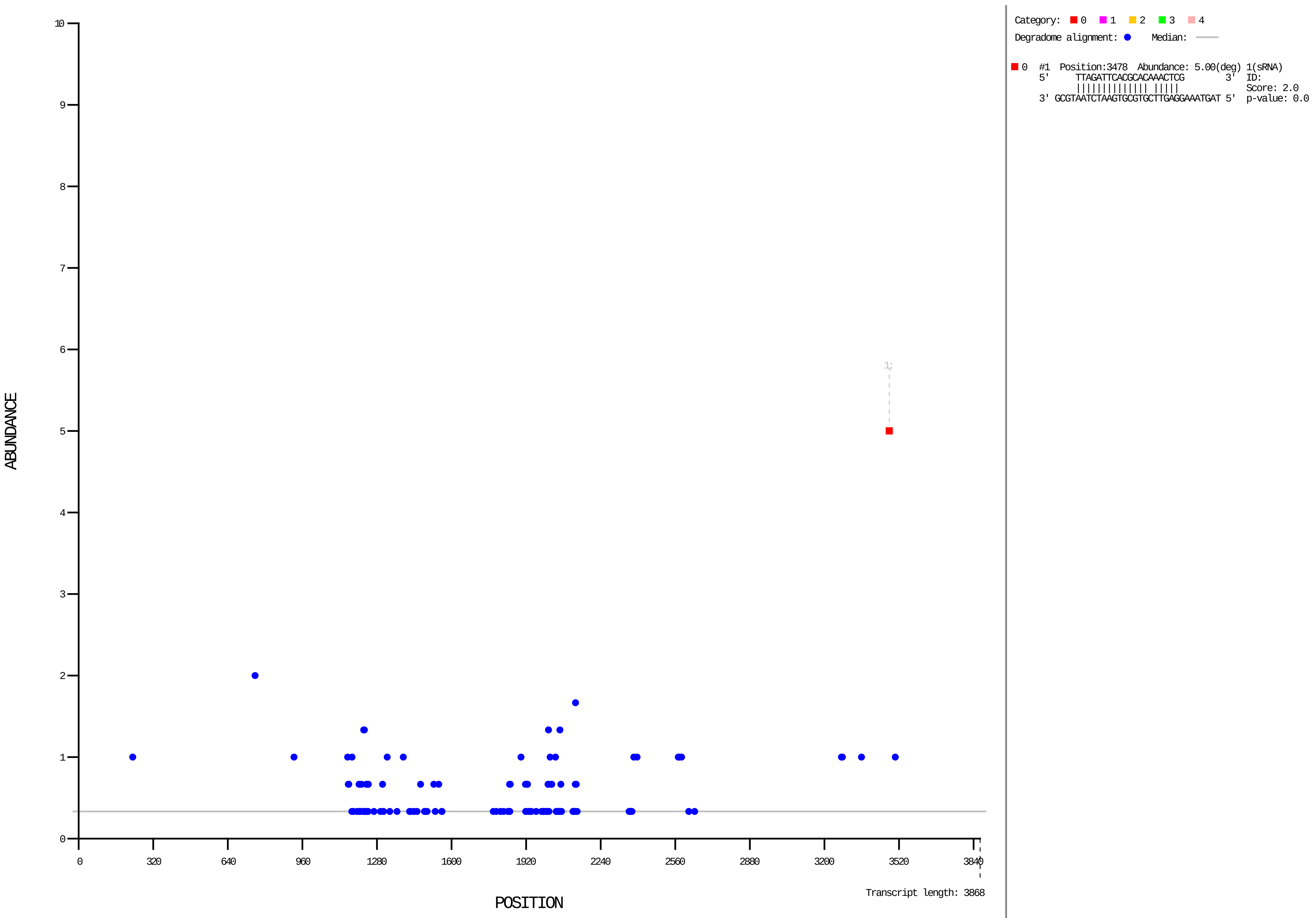

Cs6g03520.2 gene=Cs6g03520 CDS=223-3402

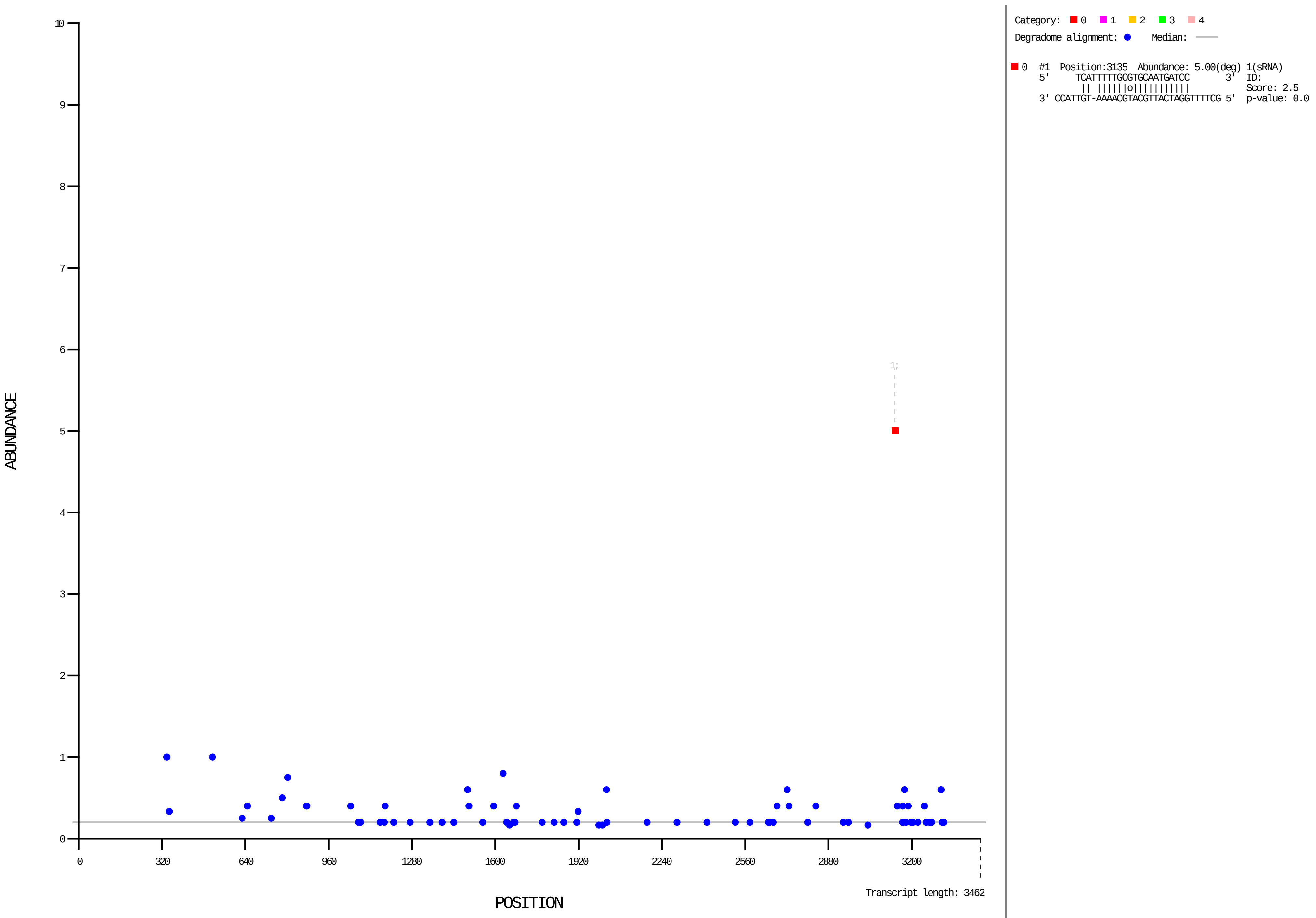

Cs6g03520.5 gene=Cs6g03520 CDS=166-4197

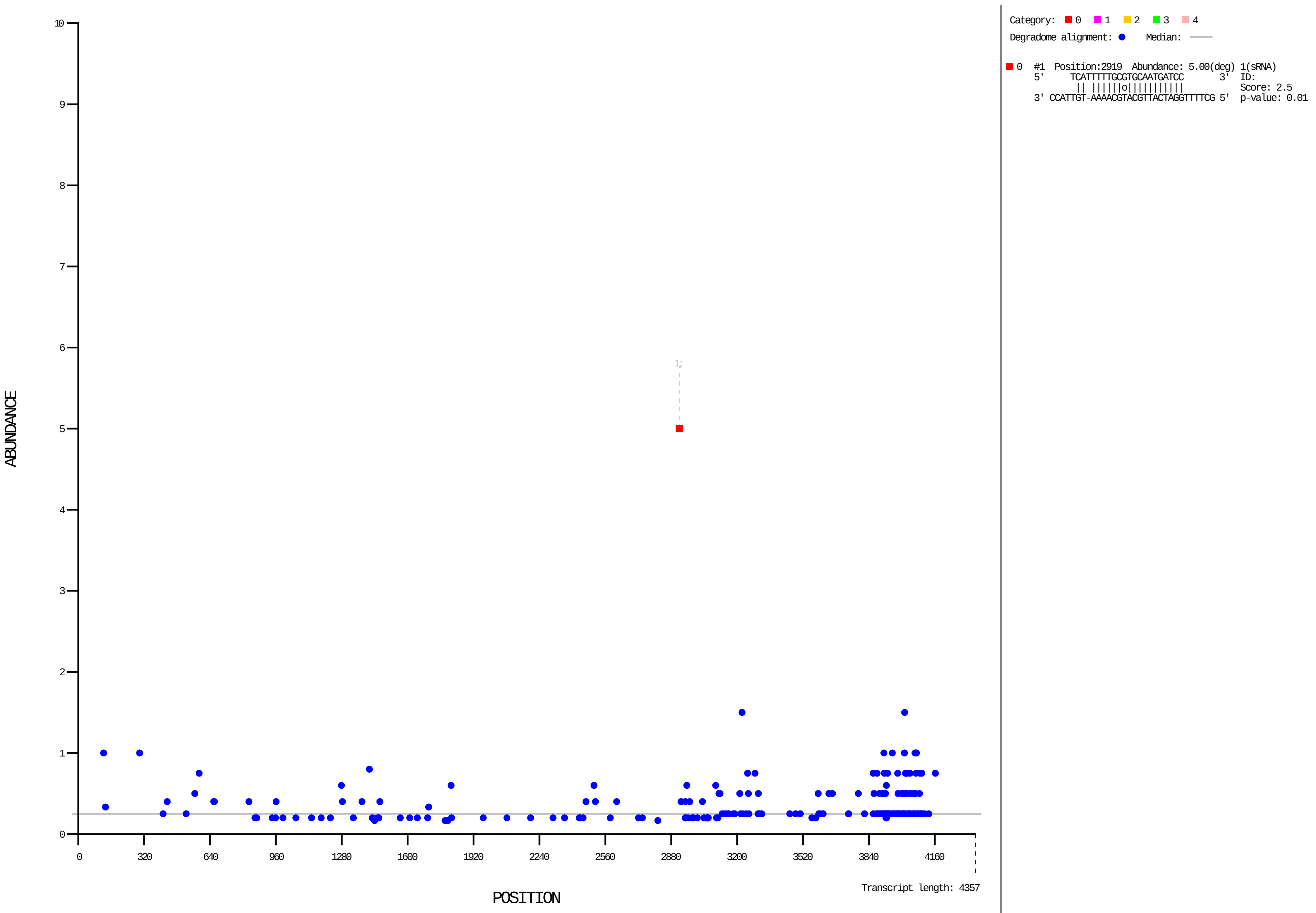

Cs3g24010.3 gene=Cs3g24010 CDS=152-964

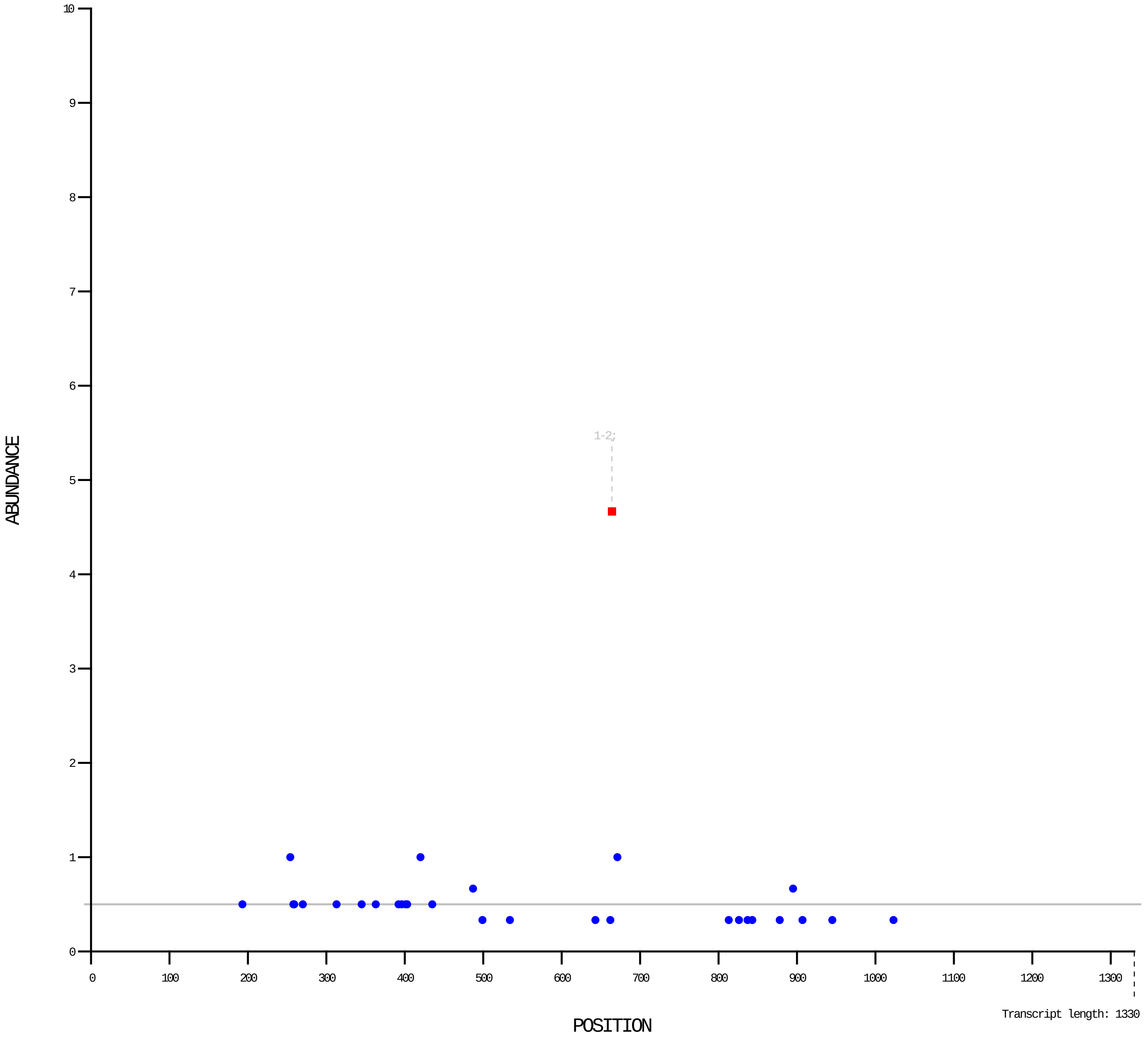

Category: 0 1 2 3 4
Degradome alignment: ● Median:

0 #1 Position:664 Abundance: 4.67(deg) 1(sRNA)

```
5'      TTCCACGGCTTTCTTGAACGT         3'  ID:
        ||||||o| ||||||||| |o             Score: 3.0
3' AACTAAGGTGTCTAAAGAACTTCCGCTCAGGA 5'  p-value: 0.0
```

0 #2 Position:664 Abundance: 4.67(deg) 1(sRNA)

```
5'      TTCCACAGCTTTCTTGAACTG         3'  ID:
        |||||||| |||||||||                Score: 4.0
3' AACTAAGGTGTCTAAAGAACTTCCGCTCAGGA 5'  p-value: 0.02
```

Cs3g24010.1 gene=Cs3g24010 CDS=152-964

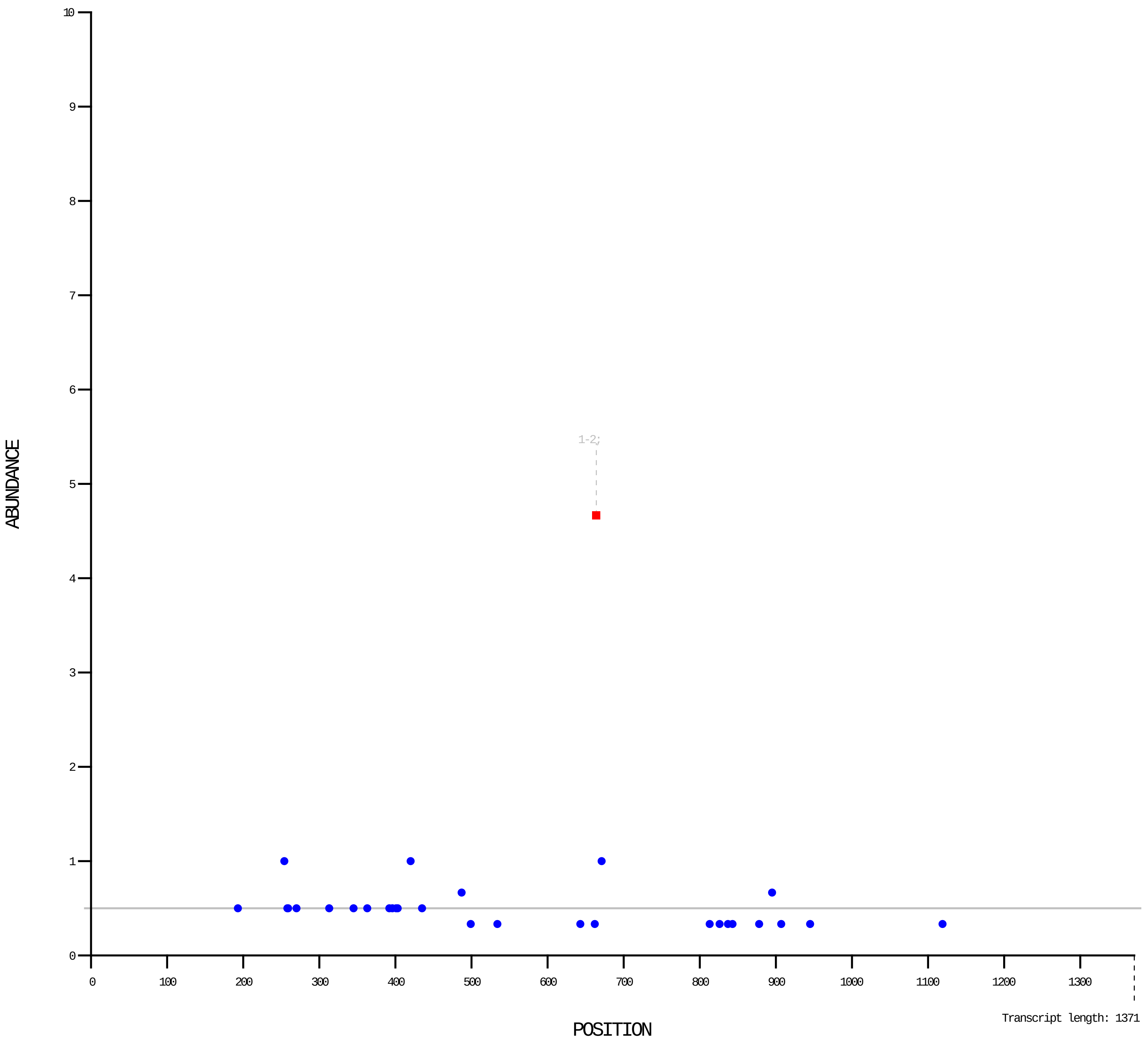

Category: 0 1 2 3 4

Degradome alignment: ● Median: —

```
0  #1  Position:664  Abundance: 4.67(deg) 1(sRNA)
   5'     TTCCACGGCTTTCTTGAACGT         3'  ID:
          ||||||o| ||||||||| |o             Score: 3.0
   3' AACTAAGGTGTCTAAAGAACTTCCGCTCAGGA 5'  p-value: 0.0

0  #2  Position:664  Abundance: 4.67(deg) 1(sRNA)
   5'     TTCCACAGCTTTCTTGAACTG         3'  ID:
          |||||||| |||||||||                Score: 4.0
   3' AACTAAGGTGTCTAAAGAACTTCCGCTCAGGA 5'  p-value: 0.02
```

Cs3g24010.2 gene=Cs3g24010 CDS=43-678

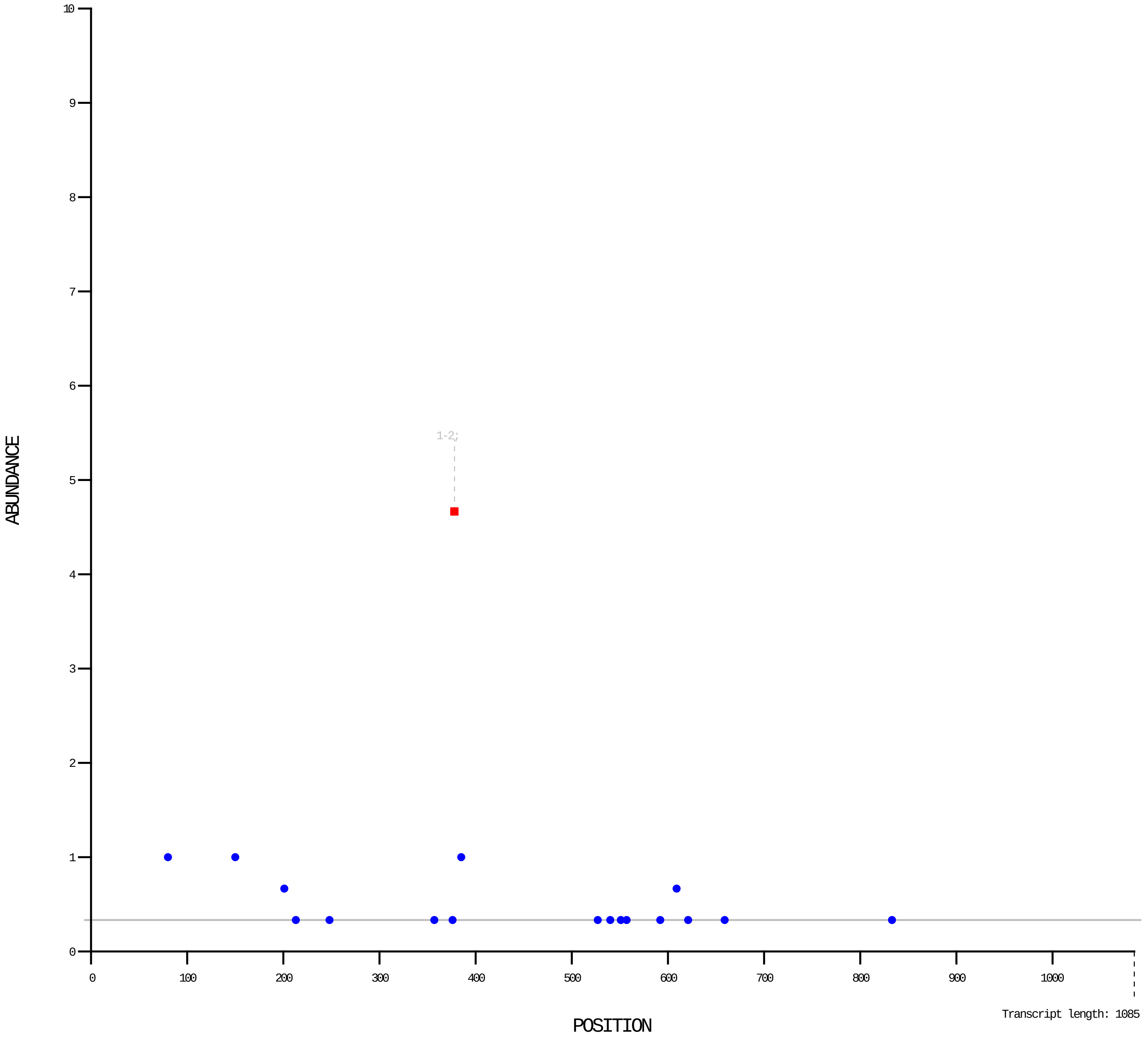


```
Category: 0 1 2 3 4
Degradome alignment:     Median:

0  #1  Position:378  Abundance: 4.67(deg) 1(sRNA)
   5'     TTCCACGGCTTTCTTGAACGT          3'  ID:
          ||||||o| ||||||||| |o              Score: 3.0
   3' AACTAAGGTGTCTAAAGAACTTCCGCTCAGGA 5'  p-value: 0.0

0  #2  Position:378  Abundance: 4.67(deg) 1(sRNA)
   5'     TTCCACAGCTTTCTTGAACTG          3'  ID:
          |||||||| |||||||||                 Score: 4.0
   3' AACTAAGGTGTCTAAAGAACTTCCGCTCAGGA 5'  p-value: 0.0
```

Cs5g07330.1 gene=Cs5g07330 CDS=1-630

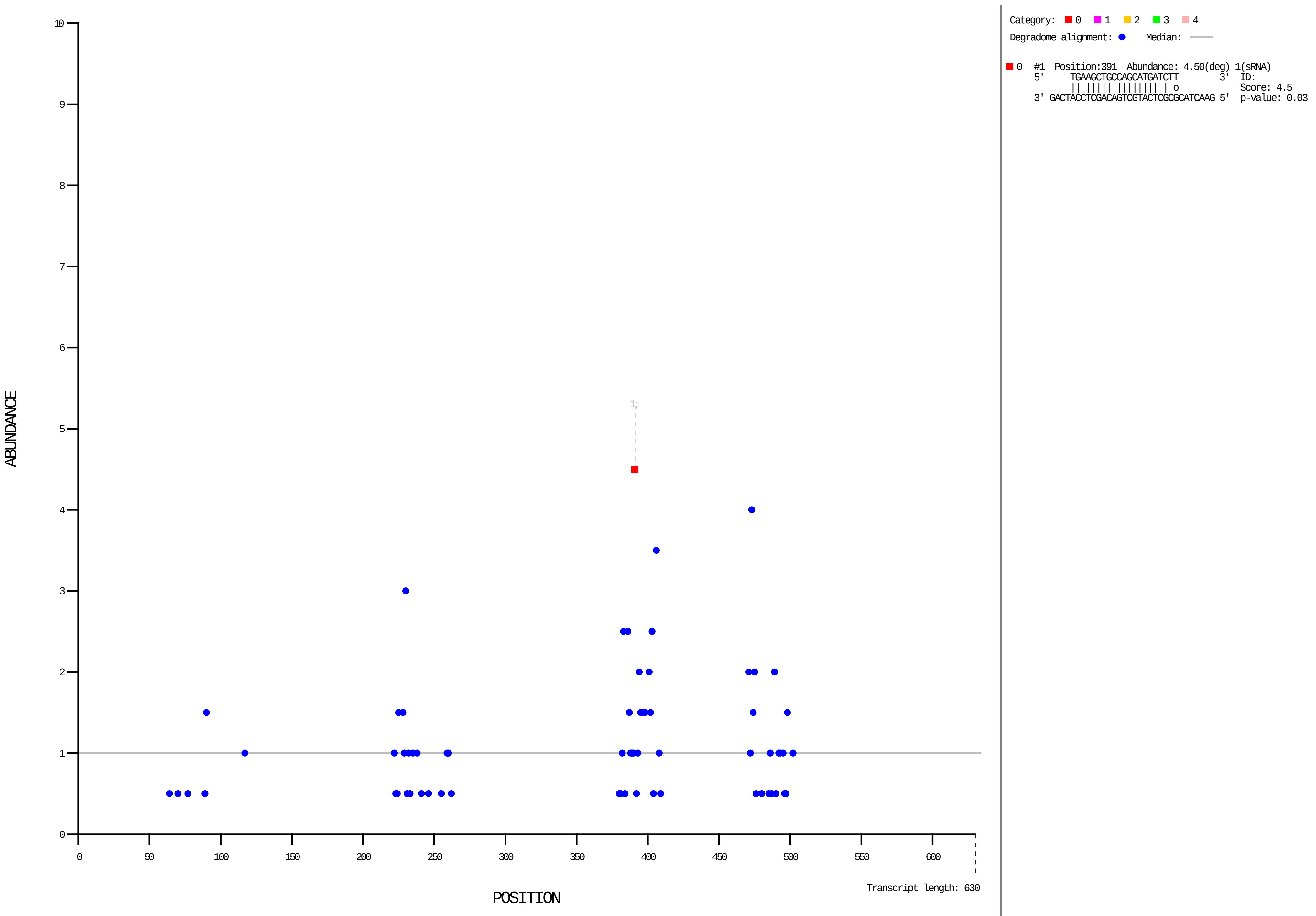

Cs2g18480.1 gene=Cs2g18480 CDS=67-4203

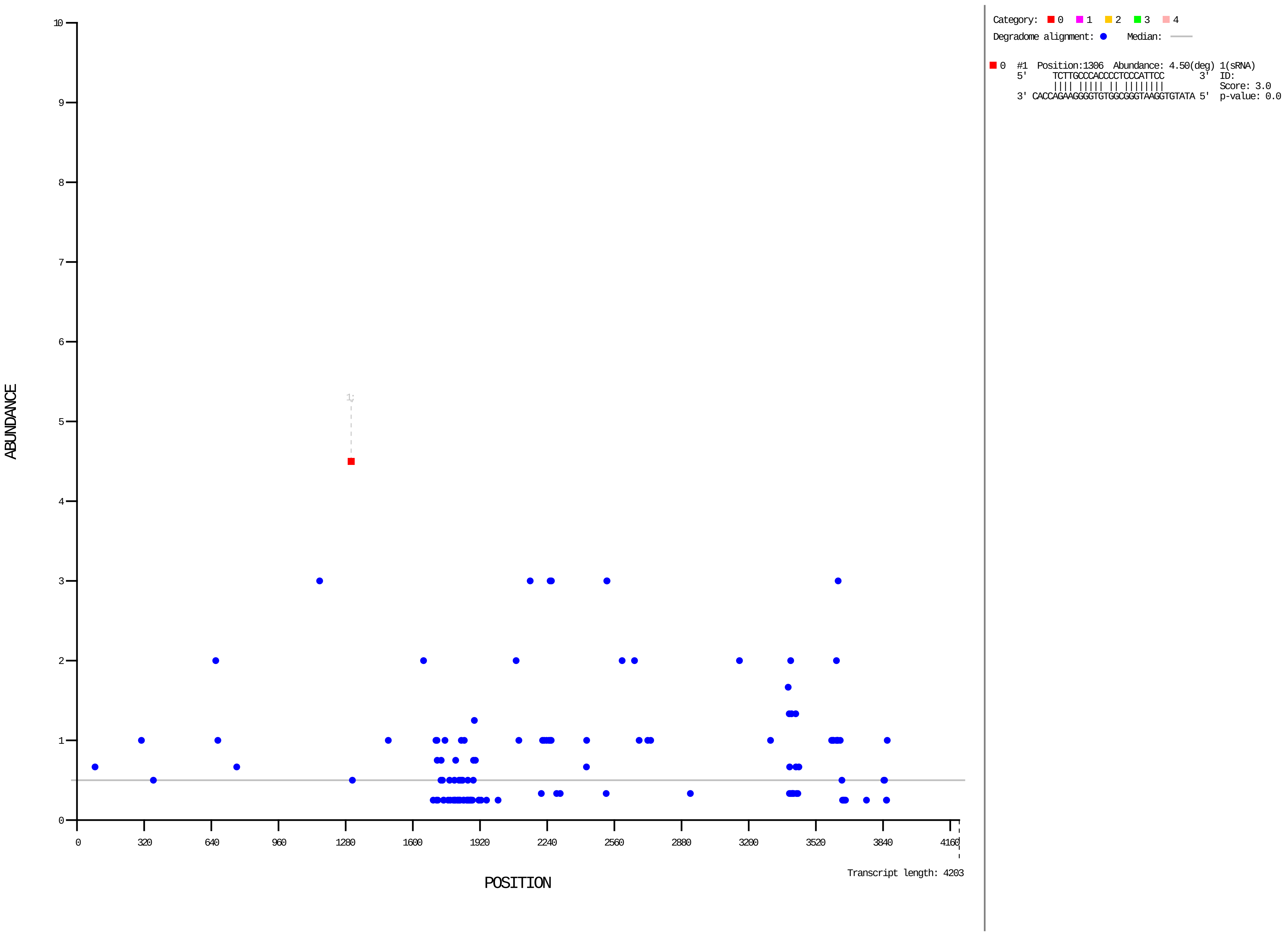

orange1.1t04538.1 gene=orange1.1t04538 CDS=1-2040

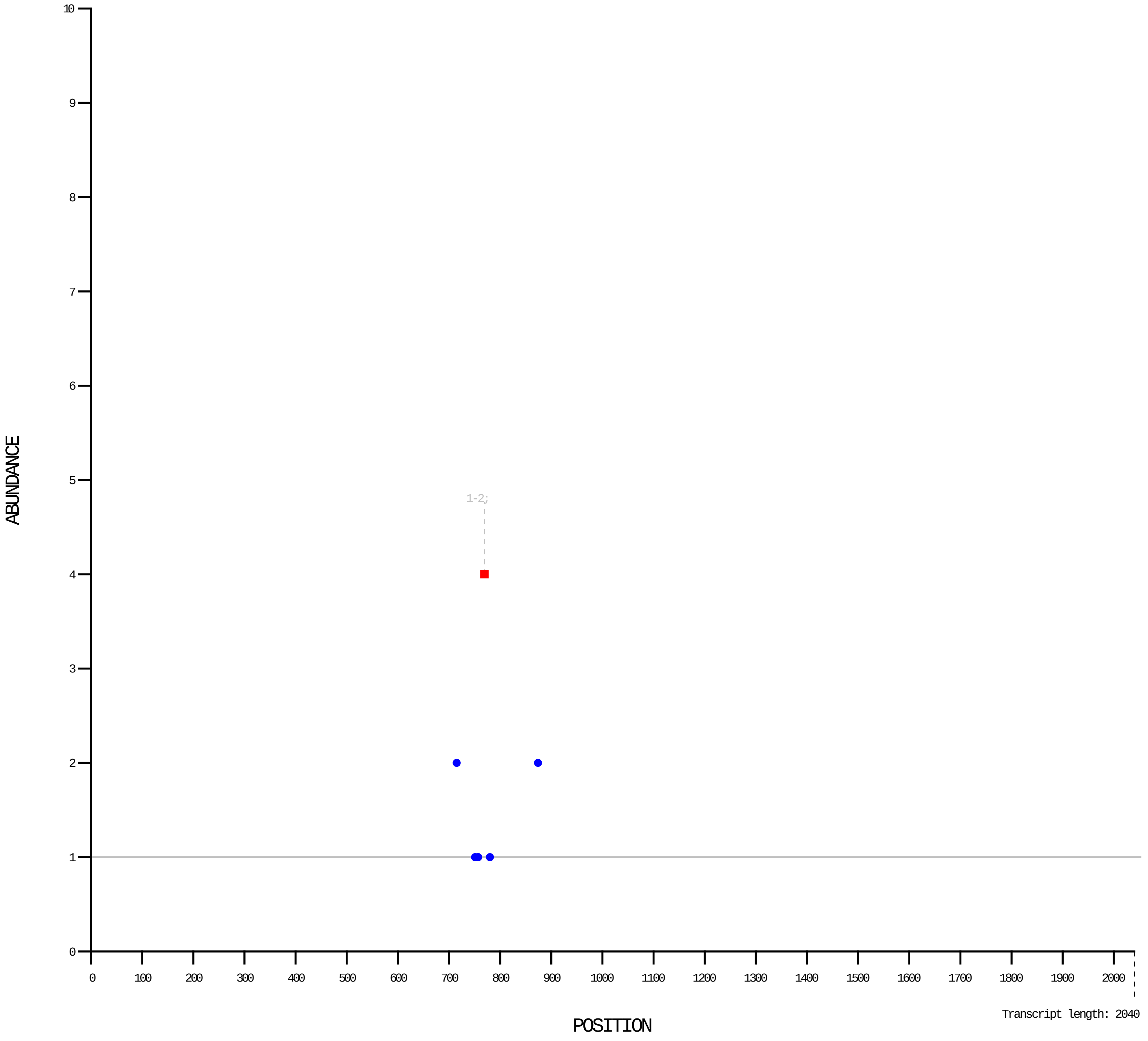

Category: 0 1 2 3 4
Degradome alignment: Median:

```
0  #1  Position:769  Abundance: 4.00(deg) 1(sRNA)
   5'     TCTTACCTATGCCACCCATTCC       3'  ID:
          |||| || |||||||||||o||           Score: 2.5
   3' TAACAGAAAGGTTACGGTGGGTAGGGCATATG 5'  p-value: 0.0

0  #2  Position:769  Abundance: 4.00(deg) 1(sRNA)
   5'     TCTTCCCTATGCCTCCCATTCC       3'  ID:
          |||| || ||||| |||||o||           Score: 3.5
   3' TAACAGAAAGGTTACGGTGGGTAGGGCATATG 5'  p-value: 0.0
```

Cs7g29270.1 gene=Cs7g29270 CDS=41-1783

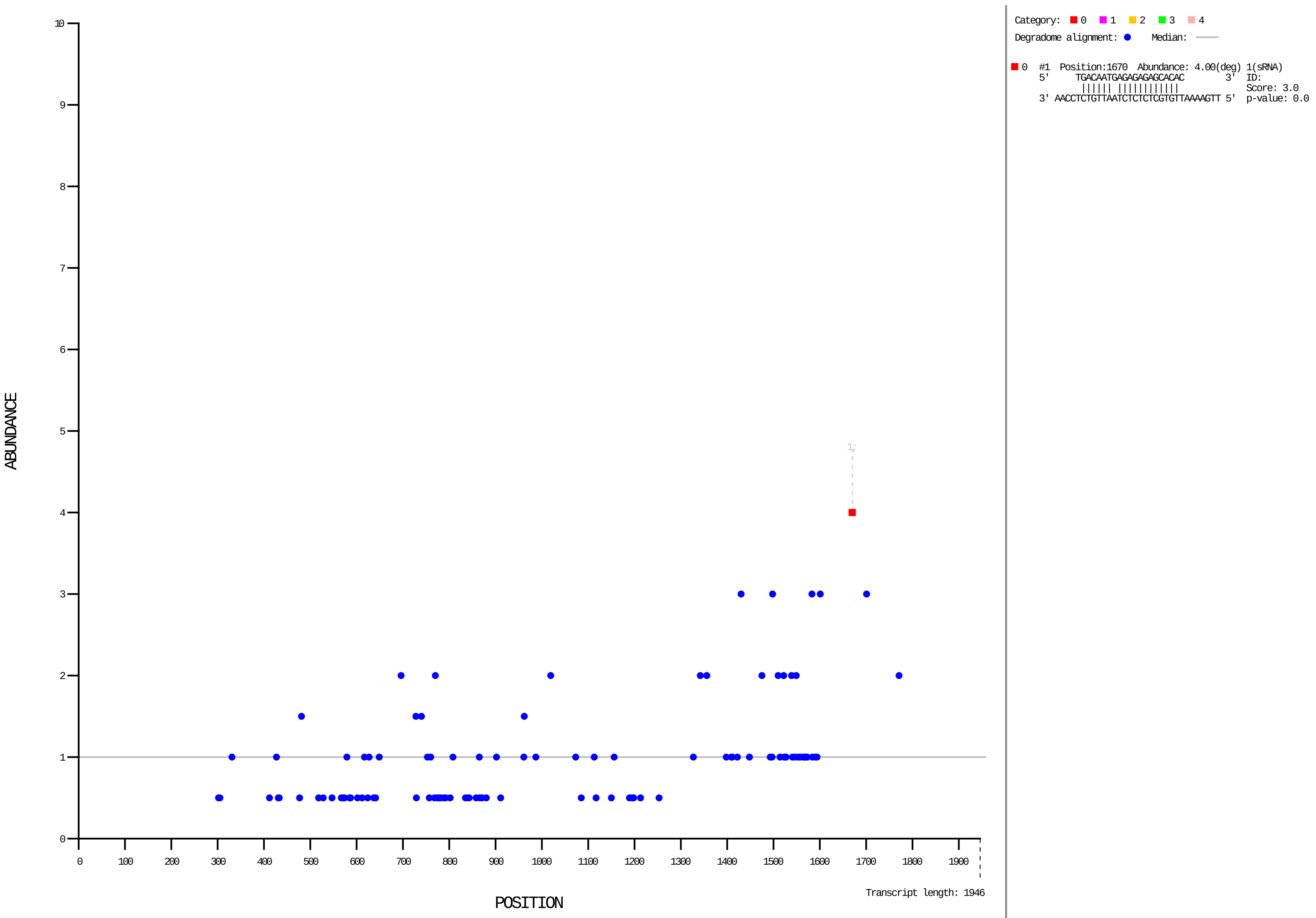

Cs8g18400.1 gene=Cs8g18400 CDS=84-1217

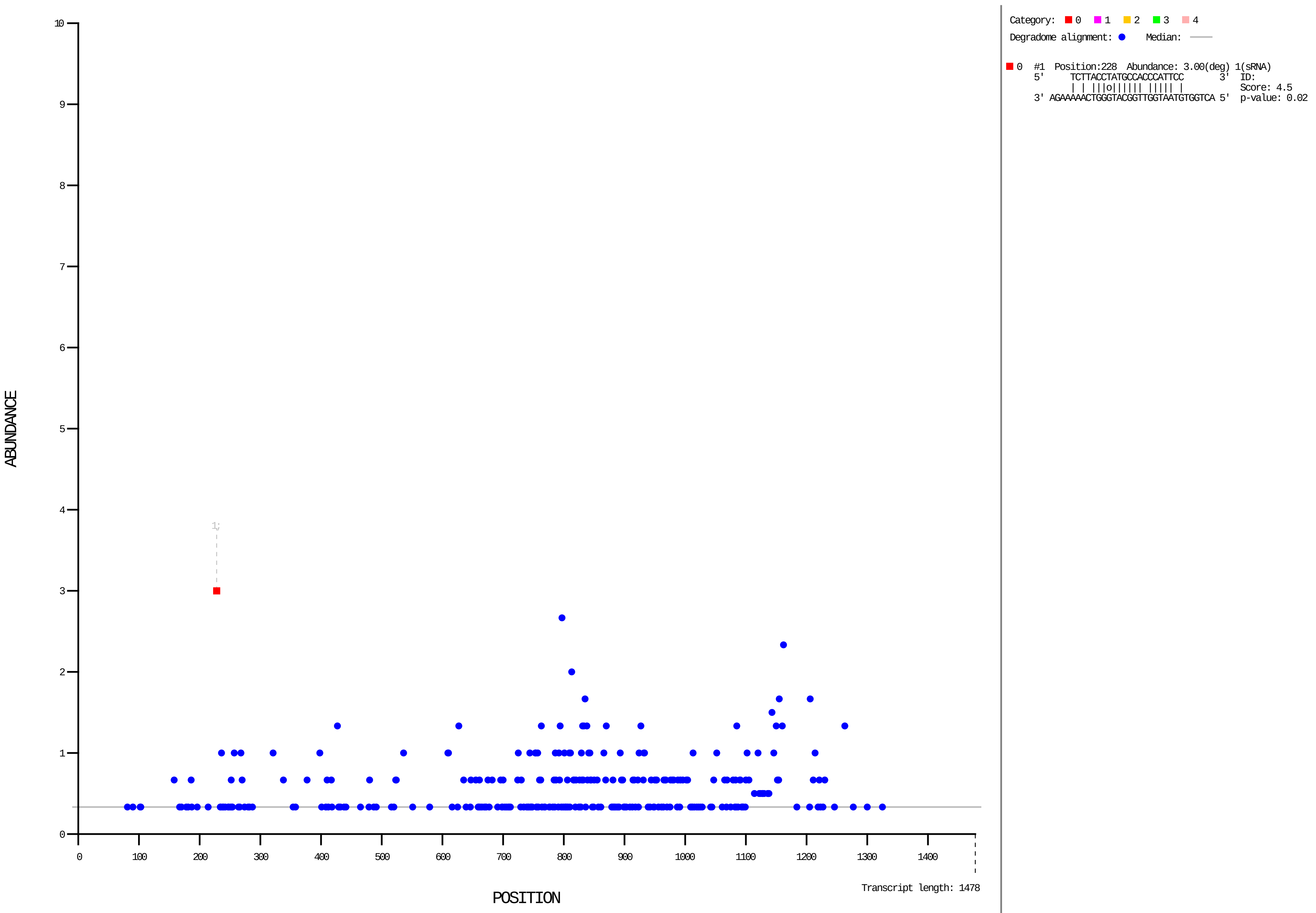

Cs4g19310.1 gene=Cs4g19310 CDS=74-2626

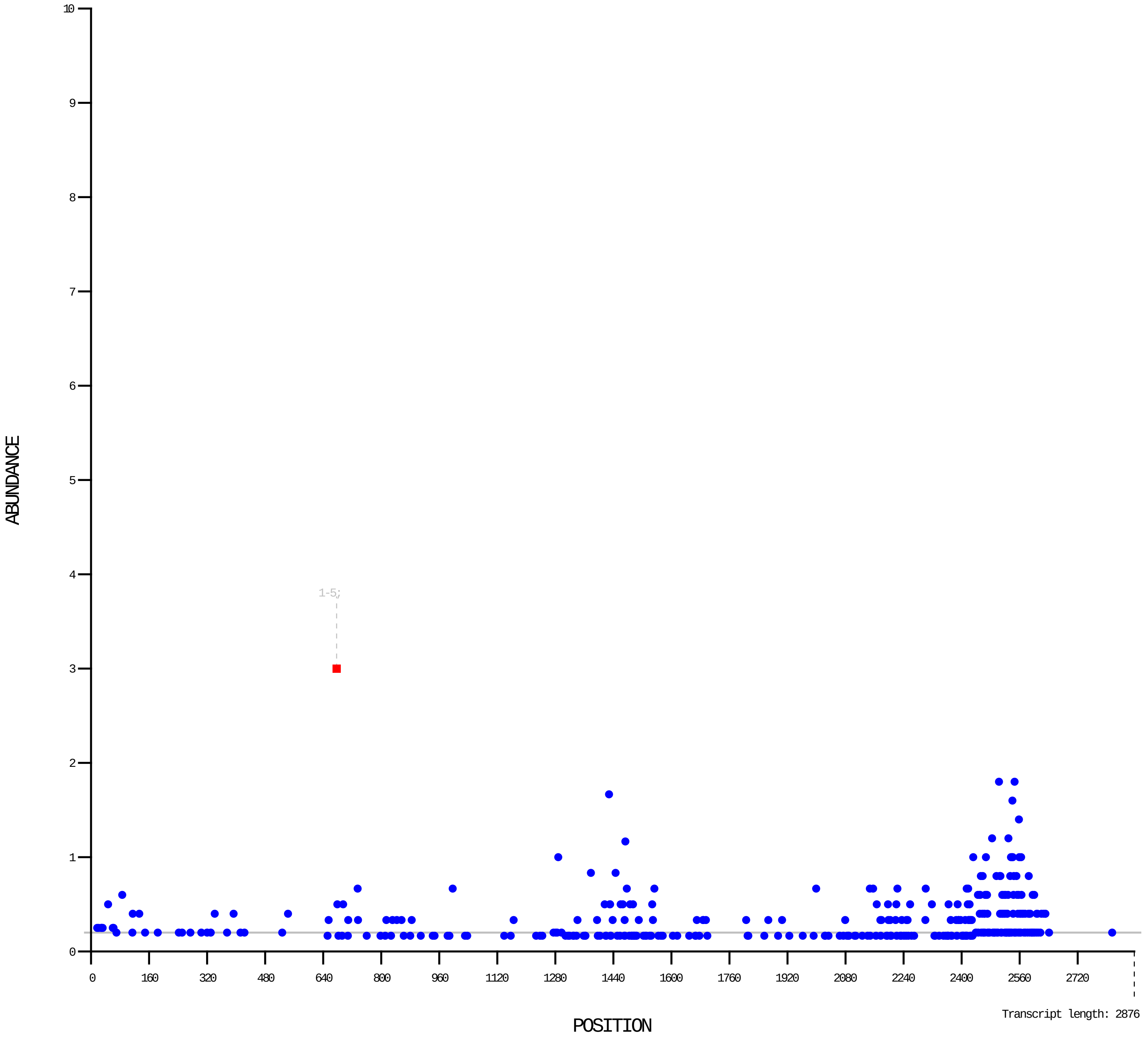


```
Category: ■ 0 ■ 1 ■ 2 ■ 3 ■ 4
Degradome alignment: ●    Median: ——

■ 0  #1  Position:677  Abundance: 3.00(deg) 1(sRNA)
     5'     TCGGACCAGGCTTCATTCCC        3'  ID:
            o||||||||||||||| |||            Score: 1.5
     3' CTTAGGCCTGGTCCGAAGTA-GGGCCCGTAAA 5'  p-value: 0.0

■ 0  #2  Position:677  Abundance: 3.00(deg) 1(sRNA)
     5'     TCGGACCAGGCTTCATTCCCT       3'  ID:
            o||||||||||||||| |||            Score: 2.5
     3' CTTAGGCCTGGTCCGAAGTA-GGGCCCGTAAA 5'  p-value: 0.0

■ 0  #3  Position:677  Abundance: 3.00(deg) 1(sRNA)
     5'     TCGGACCAGGCTTCATTCCCGT      3'  ID:
            o||||||||||||||| ||||           Score: 2.5
     3' CTTAGGCCTGGTCCGAAGTA-GGGCCCGTAAA 5'  p-value: 0.0

■ 0  #4  Position:677  Abundance: 3.00(deg) 1(sRNA)
     5'     TCGGACCAGGCTTCATTCCCC       3'  ID:
            o||||||||||||||| |||            Score: 2.5
     3' CTTAGGCCTGGTCCGAAGTA-GGGCCCGTAAA 5'  p-value: 0.0

■ 0  #5  Position:677  Abundance: 3.00(deg) 1(sRNA)
     5'     TCGGACCAGGCTTCATTCCCCC      3'  ID:
            o||||||||||||||| |||            Score: 3.5
     3' CTTAGGCCTGGTCCGAAGTA-GGGCCCGTAAA 5'  p-value: 0.0
```

Cs8g18400.3 gene=Cs8g18400 CDS=204-1391

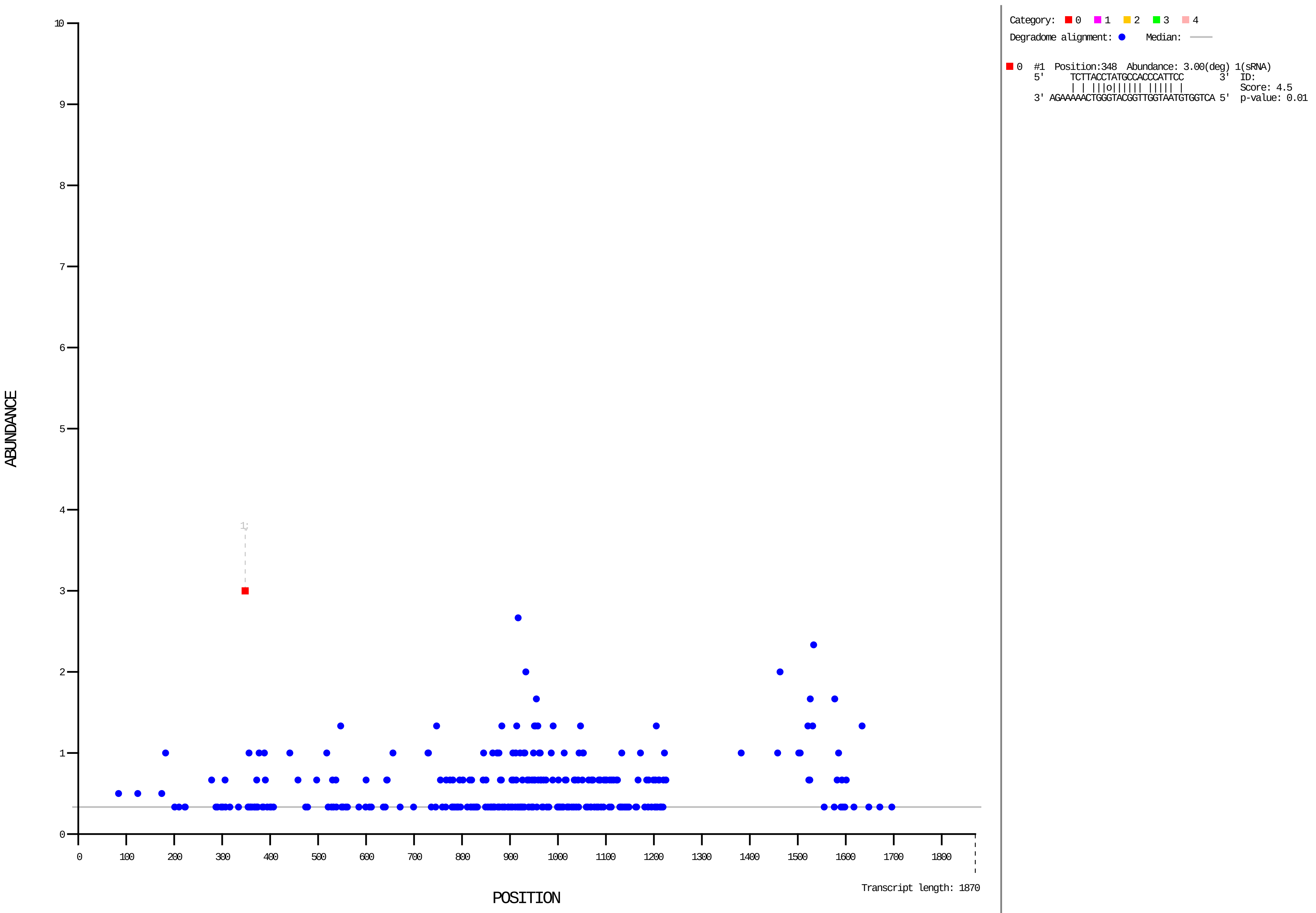

Cs8g18400.2 gene=Cs8g18400 CDS=204-1337

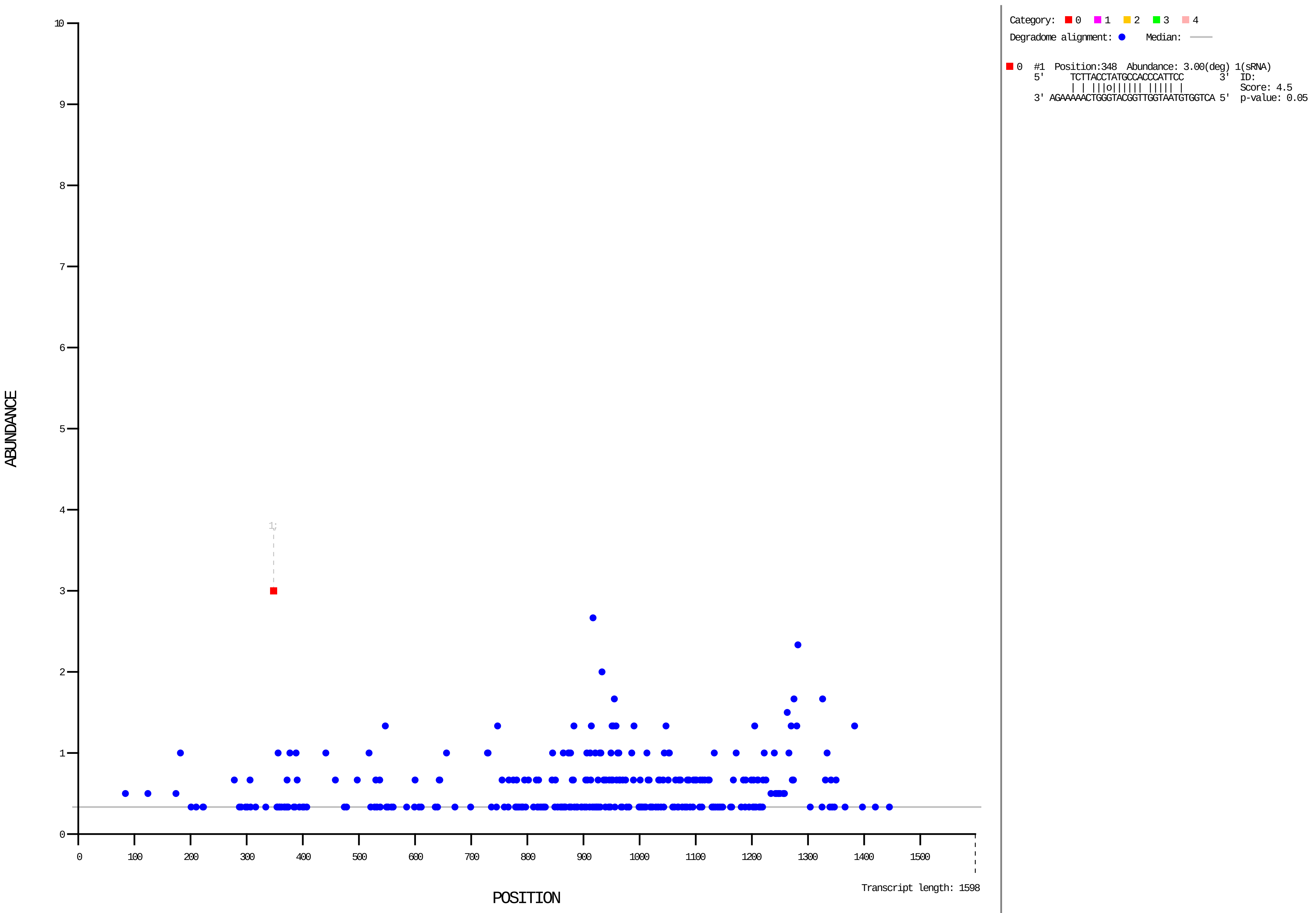

Cs6g11860.1 gene=Cs6g11860 CDS=53-1741

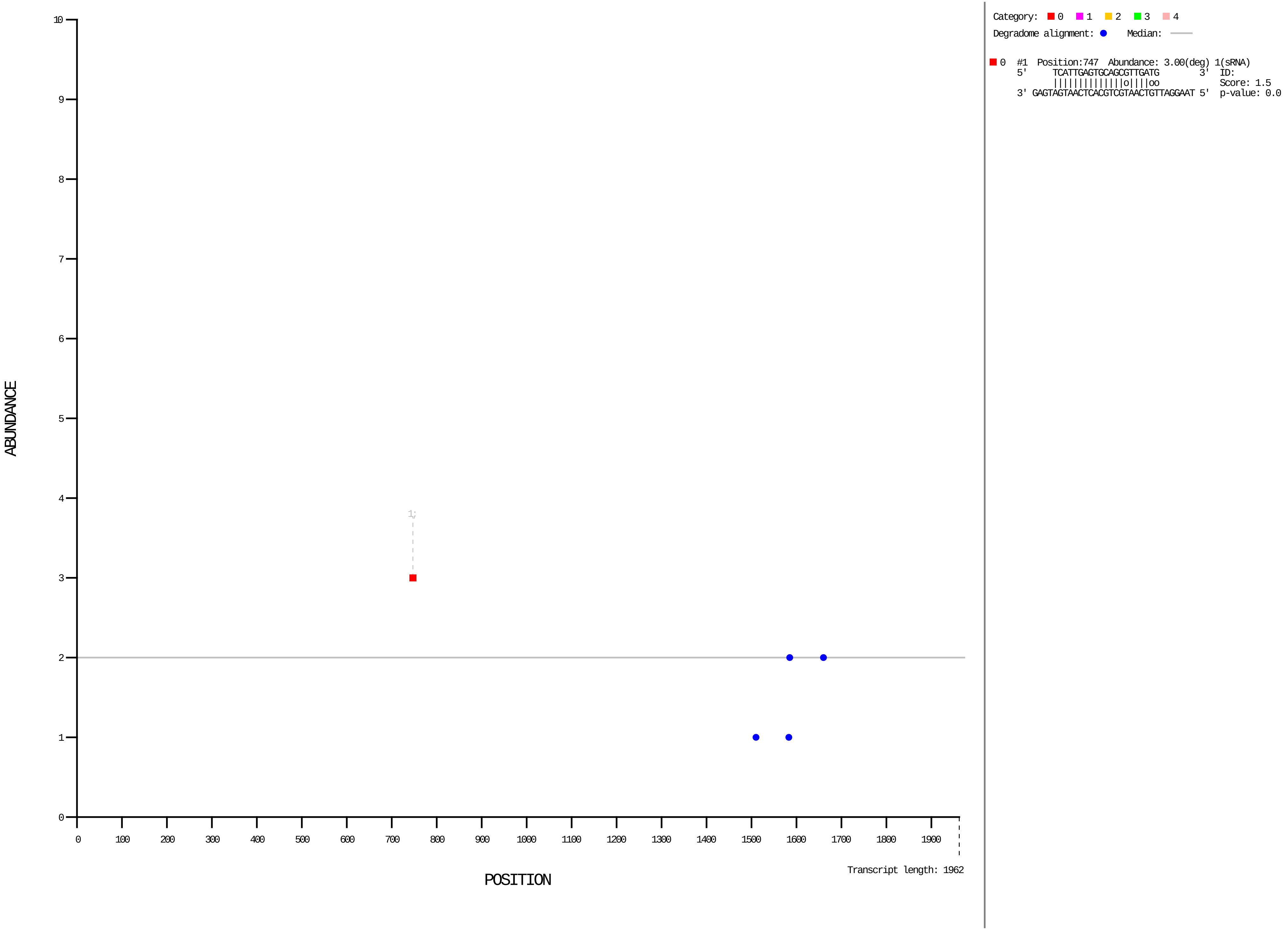

Cs4g19310.6 gene=Cs4g19310 CDS=142-2106

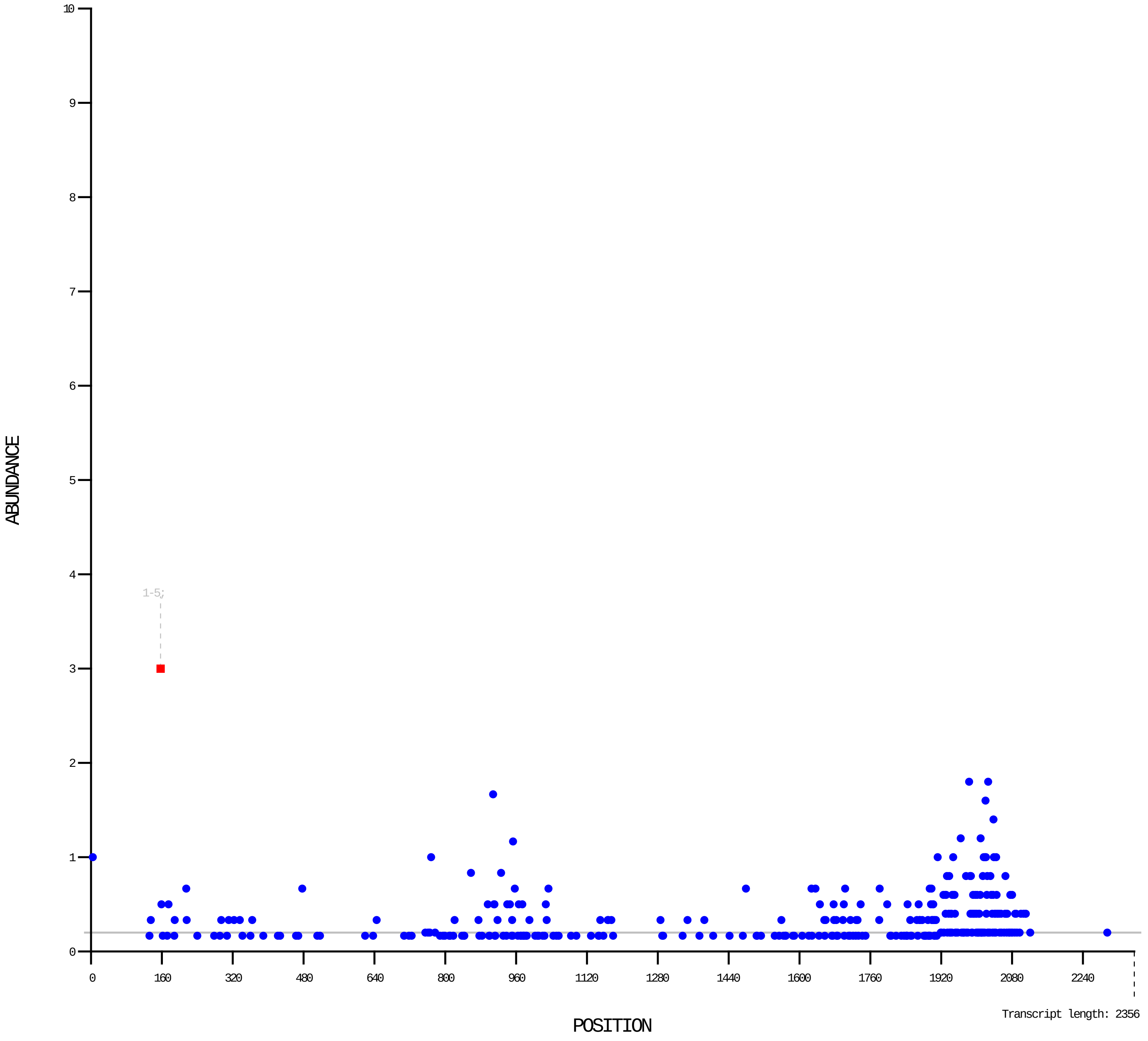

Category: 0 1 2 3 4
Degradome alignment: Median:

```
0  #1  Position:157  Abundance: 3.00(deg) 1(sRNA)
   5'     TCGGACCAGGCTTCATTCCC        3'  ID:
          o|||||||||||||||| |||           Score: 1.5
   3' CTTAGGCCTGGTCCGAAGTA-GGGCCCGTAAA 5'  p-value: 0.0

0  #2  Position:157  Abundance: 3.00(deg) 1(sRNA)
   5'     TCGGACCAGGCTTCATTCCCT       3'  ID:
          o|||||||||||||||| |||           Score: 2.5
   3' CTTAGGCCTGGTCCGAAGTA-GGGCCCGTAAA 5'  p-value: 0.0

0  #3  Position:157  Abundance: 3.00(deg) 1(sRNA)
   5'     TCGGACCAGGCTTCATTCCCGT      3'  ID:
          o|||||||||||||||| ||||          Score: 2.5
   3' CTTAGGCCTGGTCCGAAGTA-GGGCCCGTAAA 5'  p-value: 0.0

0  #4  Position:157  Abundance: 3.00(deg) 1(sRNA)
   5'     TCGGACCAGGCTTCATTCCCC       3'  ID:
          o|||||||||||||||| |||           Score: 2.5
   3' CTTAGGCCTGGTCCGAAGTA-GGGCCCGTAAA 5'  p-value: 0.0

0  #5  Position:157  Abundance: 3.00(deg) 1(sRNA)
   5'     TCGGACCAGGCTTCATTCCCCC      3'  ID:
          o|||||||||||||||| |||           Score: 3.5
   3' CTTAGGCCTGGTCCGAAGTA-GGGCCCGTAAA 5'  p-value: 0.01
```

Cs4g19310.2 gene=Cs4g19310 CDS=78-2630

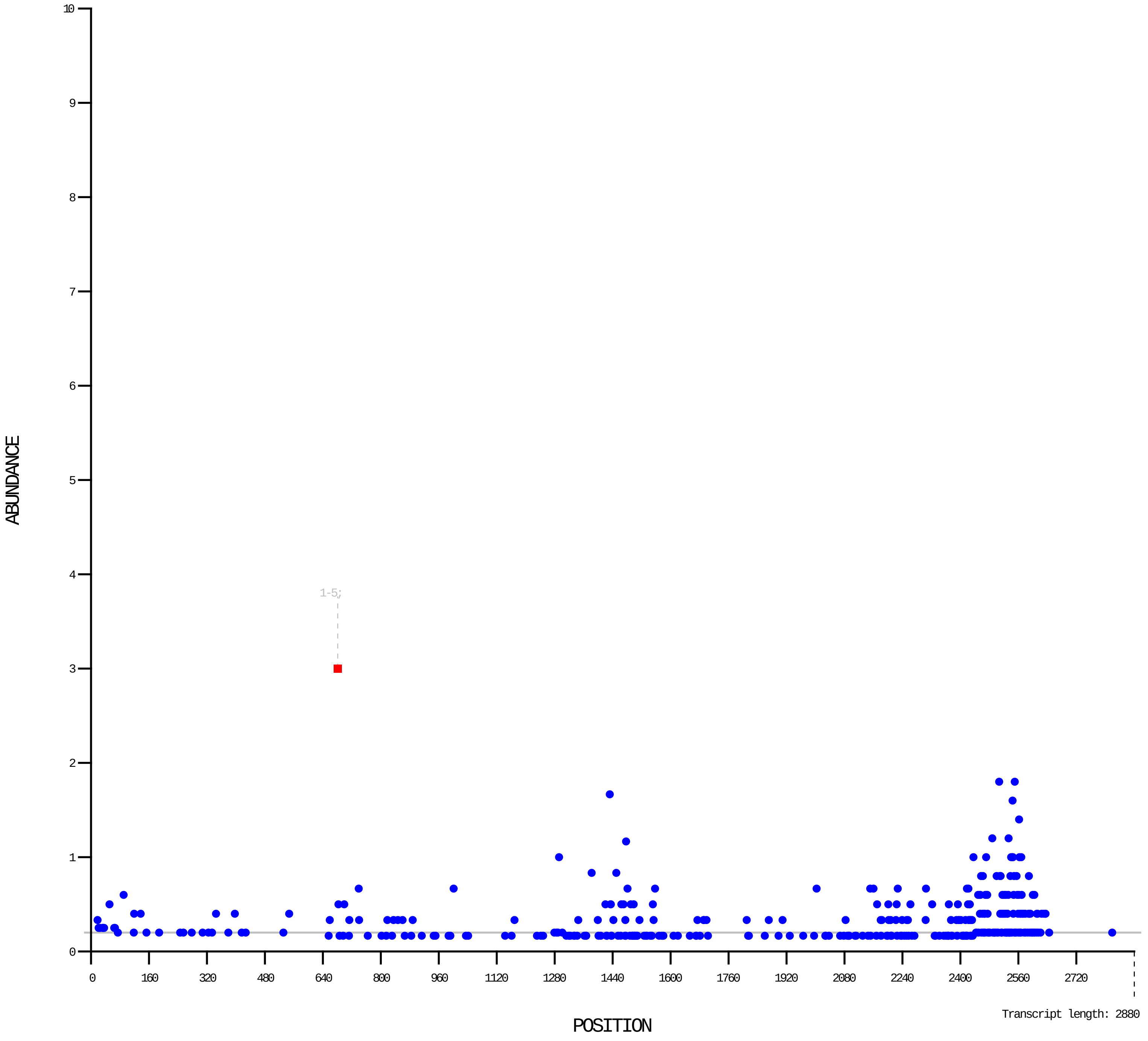

Category: 0 1 2 3 4
Degradome alignment: Median:

```
0  #1  Position:681  Abundance: 3.00(deg) 1(sRNA)
   5'     TCGGACCAGGCTTCATTCCC        3'  ID:
          o|||||||||||||| |||             Score: 1.5
   3' CTTAGGCCTGGTCCGAAGTA-GGGCCCGTAAA 5'  p-value: 0.0

0  #2  Position:681  Abundance: 3.00(deg) 1(sRNA)
   5'     TCGGACCAGGCTTCATTCCCT       3'  ID:
          o|||||||||||||| |||             Score: 2.5
   3' CTTAGGCCTGGTCCGAAGTA-GGGCCCGTAAA 5'  p-value: 0.0

0  #3  Position:681  Abundance: 3.00(deg) 1(sRNA)
   5'     TCGGACCAGGCTTCATTCCCGT      3'  ID:
          o|||||||||||||| ||||            Score: 2.5
   3' CTTAGGCCTGGTCCGAAGTA-GGGCCCGTAAA 5'  p-value: 0.0

0  #4  Position:681  Abundance: 3.00(deg) 1(sRNA)
   5'     TCGGACCAGGCTTCATTCCC        3'  ID:
          o|||||||||||||| |||             Score: 2.5
   3' CTTAGGCCTGGTCCGAAGTA-GGGCCCGTAAA 5'  p-value: 0.0

0  #5  Position:681  Abundance: 3.00(deg) 1(sRNA)
   5'     TCGGACCAGGCTTCATTCCCCC      3'  ID:
          o|||||||||||||| |||             Score: 3.5
   3' CTTAGGCCTGGTCCGAAGTA-GGGCCCGTAAA 5'  p-value: 0.0
```

Cs4g19310.4 gene=Cs4g19310 CDS=39-2591

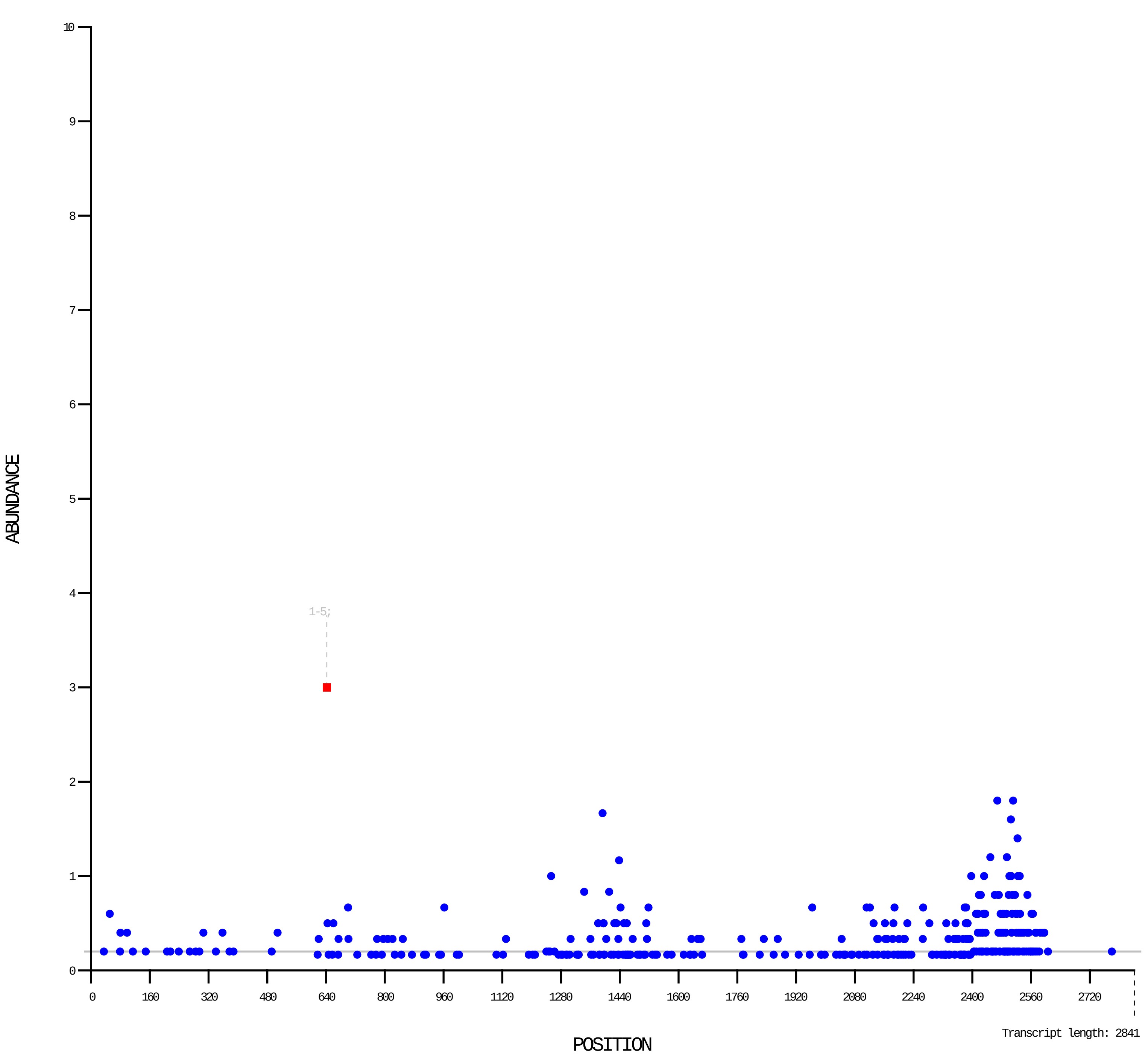

0 #1 Position:642 Abundance: 3.00(deg) 1(sRNA)
5' TCGGACCAGGCTTCATTCCC 3' ID:
o|||||||||||||||| ||| Score: 1.5
3' CTTAGGCCTGGTCCGAAGTA-GGGCCCGTAAA 5' p-value: 0.0

0 #2 Position:642 Abundance: 3.00(deg) 1(sRNA)
5' TCGGACCAGGCTTCATTCCCT 3' ID:
o|||||||||||||||| ||| Score: 2.5
3' CTTAGGCCTGGTCCGAAGTA-GGGCCCGTAAA 5' p-value: 0.0

0 #3 Position:642 Abundance: 3.00(deg) 1(sRNA)
5' TCGGACCAGGCTTCATTCCCGT 3' ID:
o|||||||||||||||| |||| Score: 2.5
3' CTTAGGCCTGGTCCGAAGTA-GGGCCCGTAAA 5' p-value: 0.0

0 #4 Position:642 Abundance: 3.00(deg) 1(sRNA)
5' TCGGACCAGGCTTCATTCCCC 3' ID:
o|||||||||||||||| ||| Score: 2.5
3' CTTAGGCCTGGTCCGAAGTA-GGGCCCGTAAA 5' p-value: 0.0

0 #5 Position:642 Abundance: 3.00(deg) 1(sRNA)
5' TCGGACCAGGCTTCATTCCCCC 3' ID:
o|||||||||||||||| ||| Score: 3.5
3' CTTAGGCCTGGTCCGAAGTA-GGGCCCGTAAA 5' p-value: 0.0

Cs3g24450.2 gene=Cs3g24450 CDS=81-1241

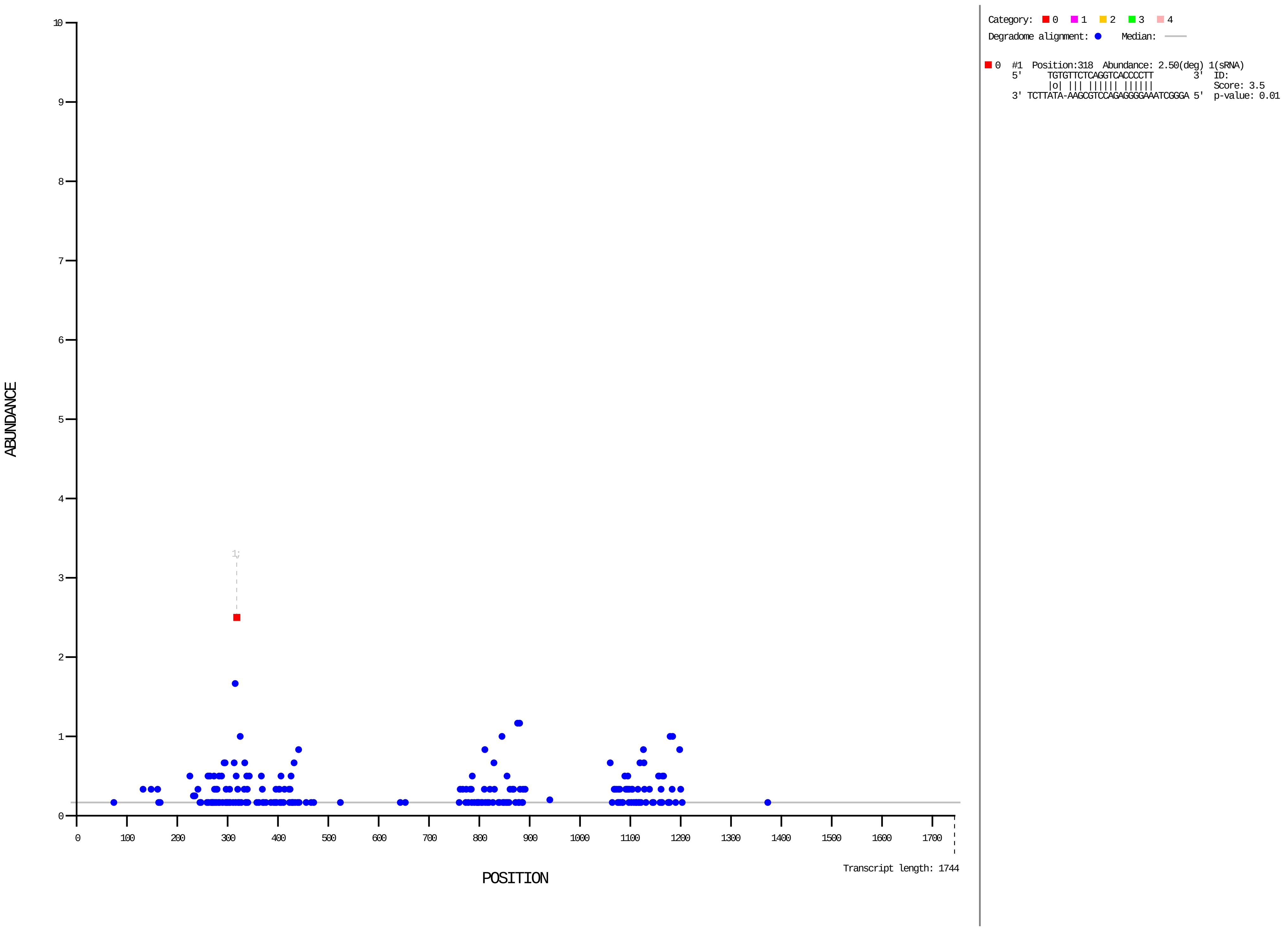

Cs3g24450.5 gene=Cs3g24450 CDS=506-1423

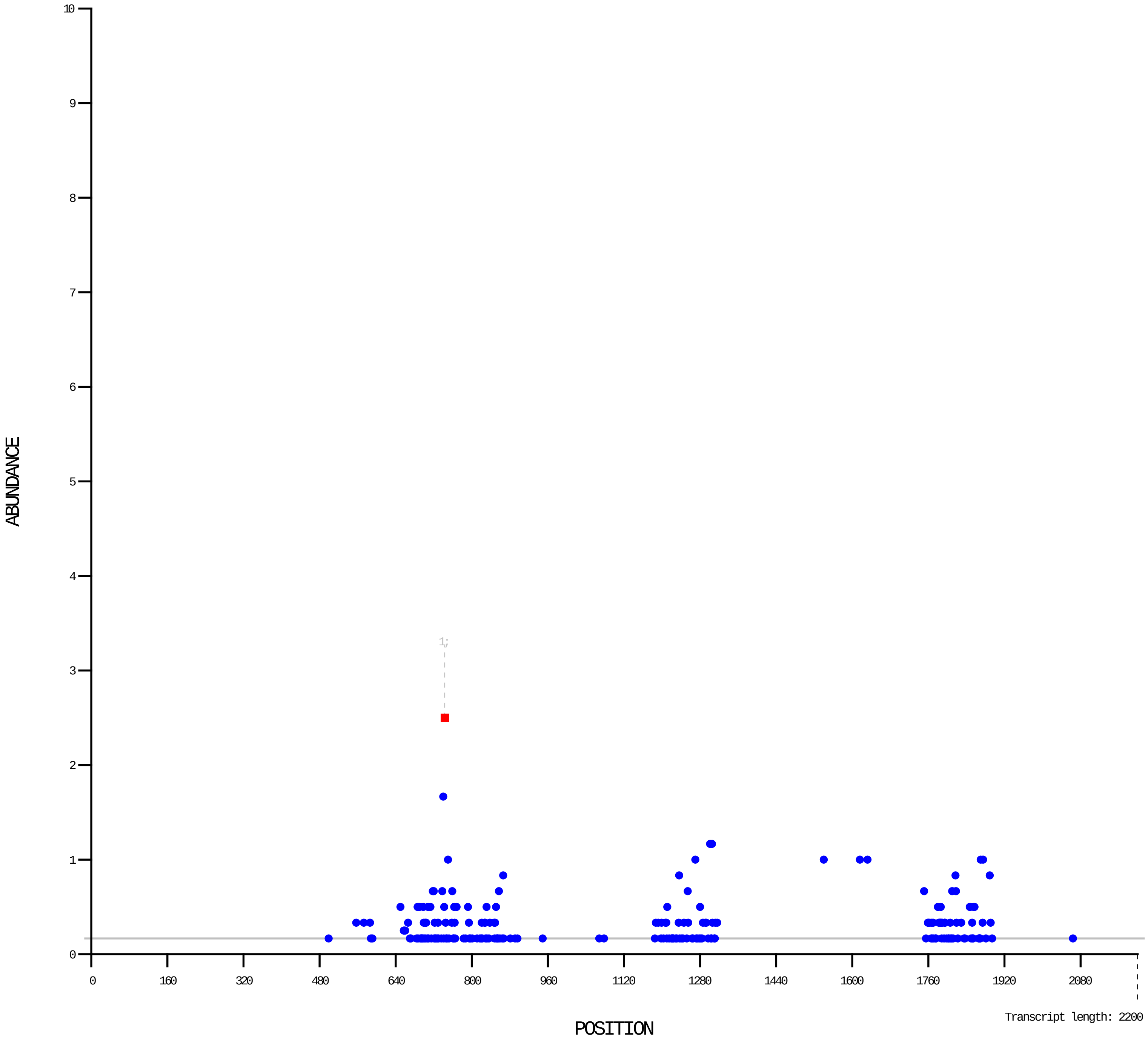

Category: 0 1 2 3 4
Degradome alignment: Median:

0 #1 Position:743 Abundance: 2.50(deg) 1(sRNA)

```
5'     TGTGTTCTCAGGTCACCCCTT        3'  ID:
       |o| ||| |||||| ||||||            Score: 3.5
3' TCTTATA-AAGCGTCCAGAGGGGAAATCGGGA 5'  p-value: 0.01
```

Cs3g24450.1 gene=Cs3g24450 CDS=310-1470

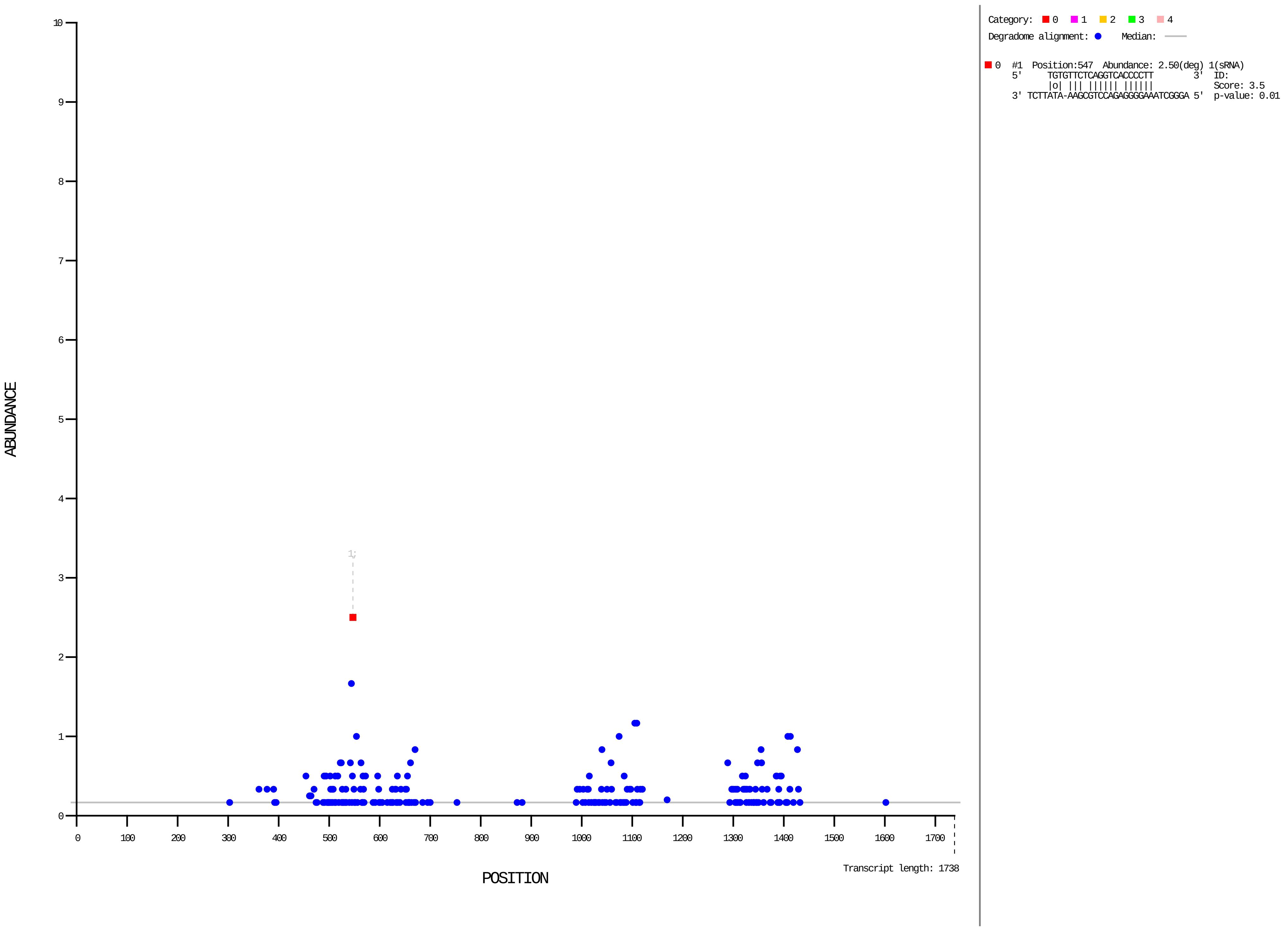

Cs3g24450.3 gene=Cs3g24450 CDS=506-1666

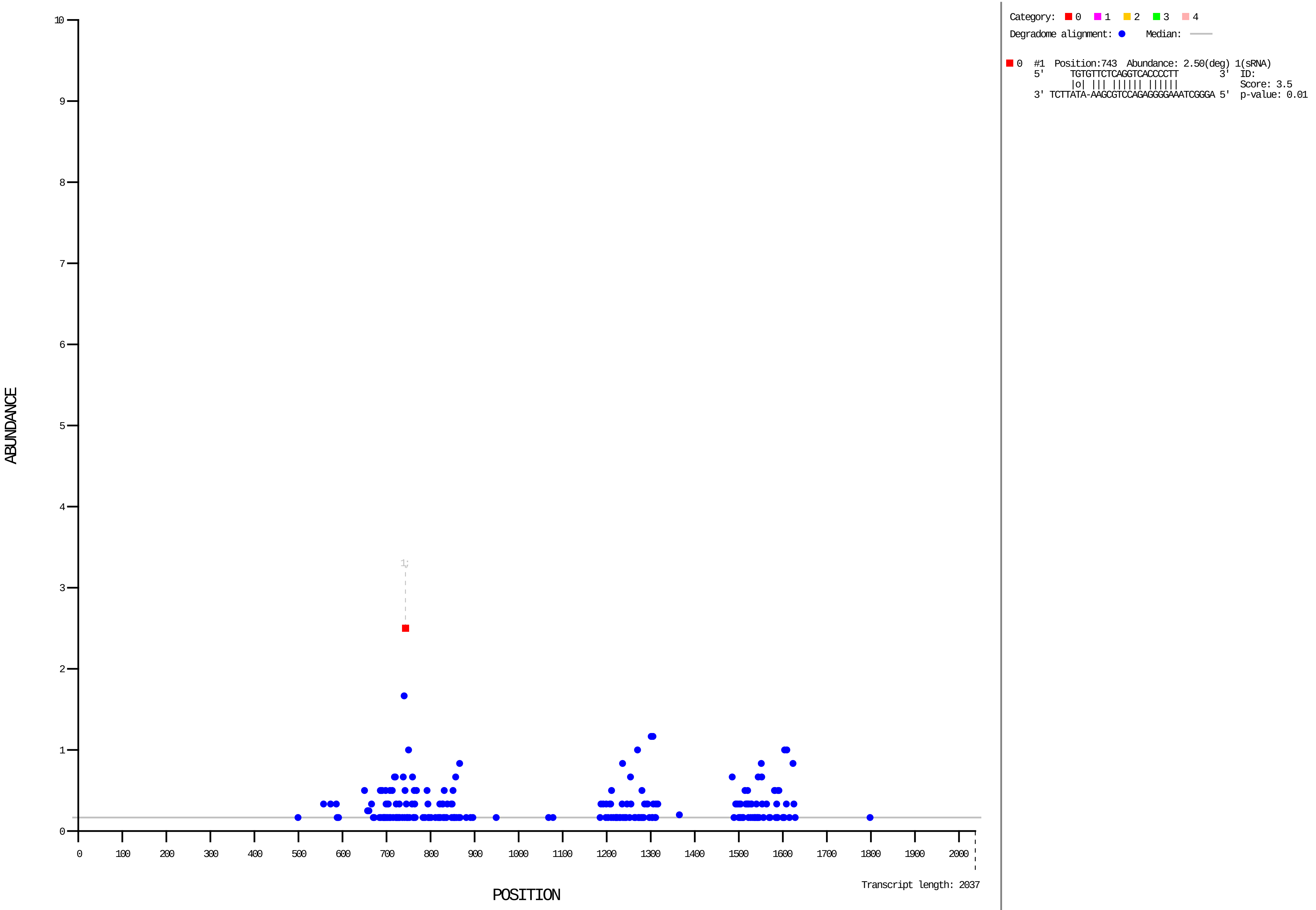

Cs8g17630.1 gene=Cs8g17630 CDS=44-1777

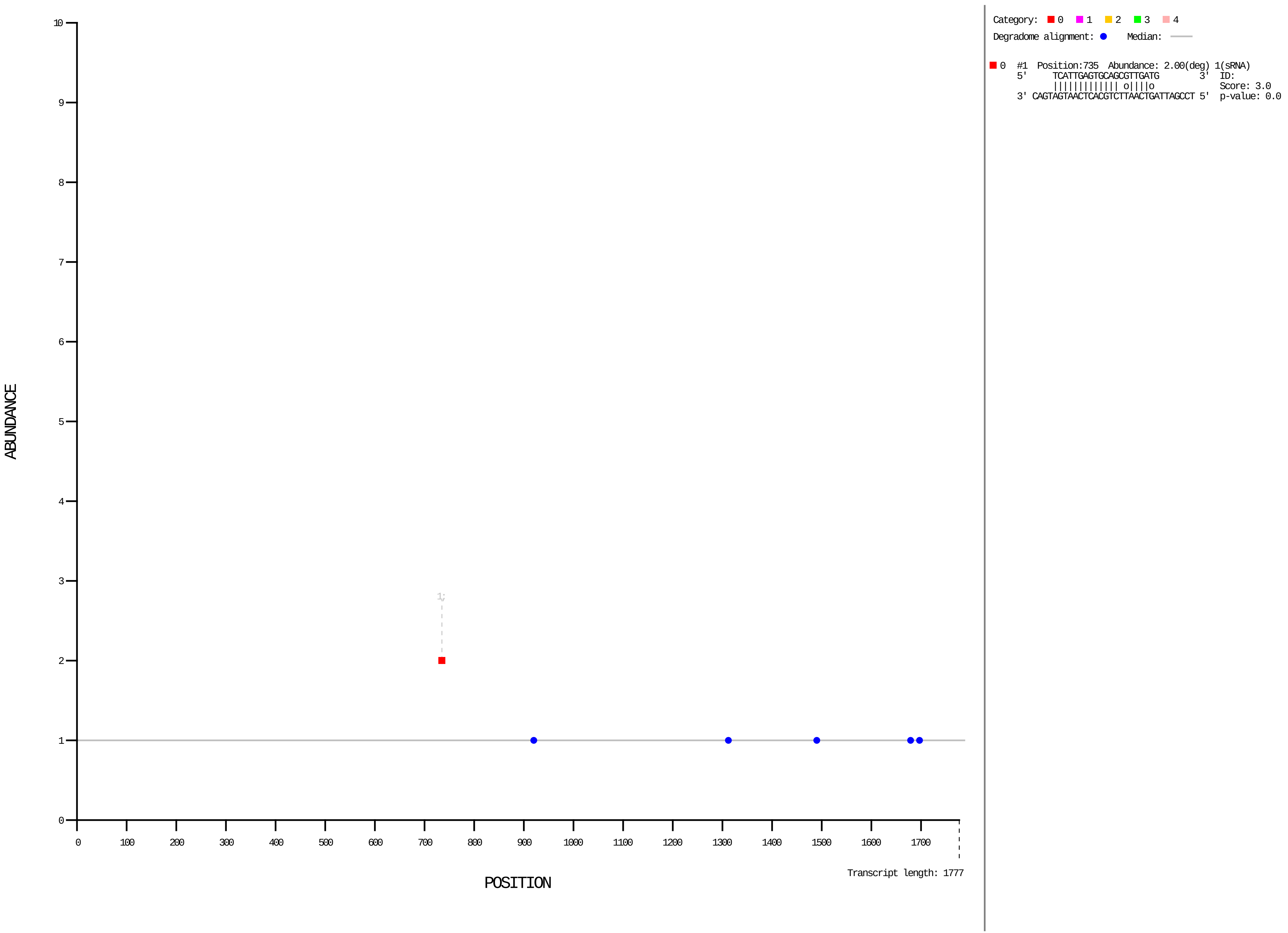

Cs5g22330.2 gene=Cs5g22330 CDS=682-1314

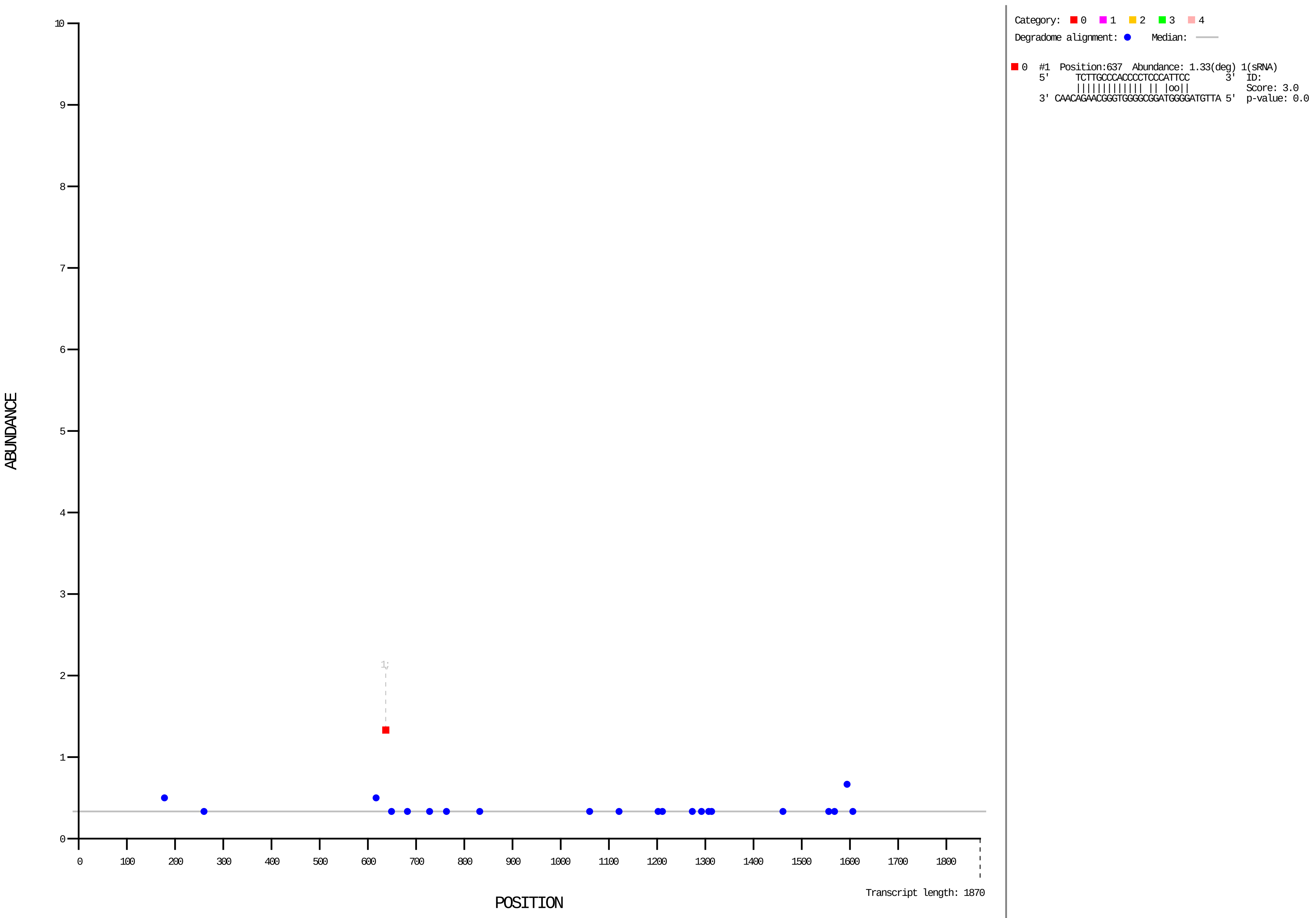

orange1.1t00584.1 gene=orange1.1t00584 CDS=1-5892

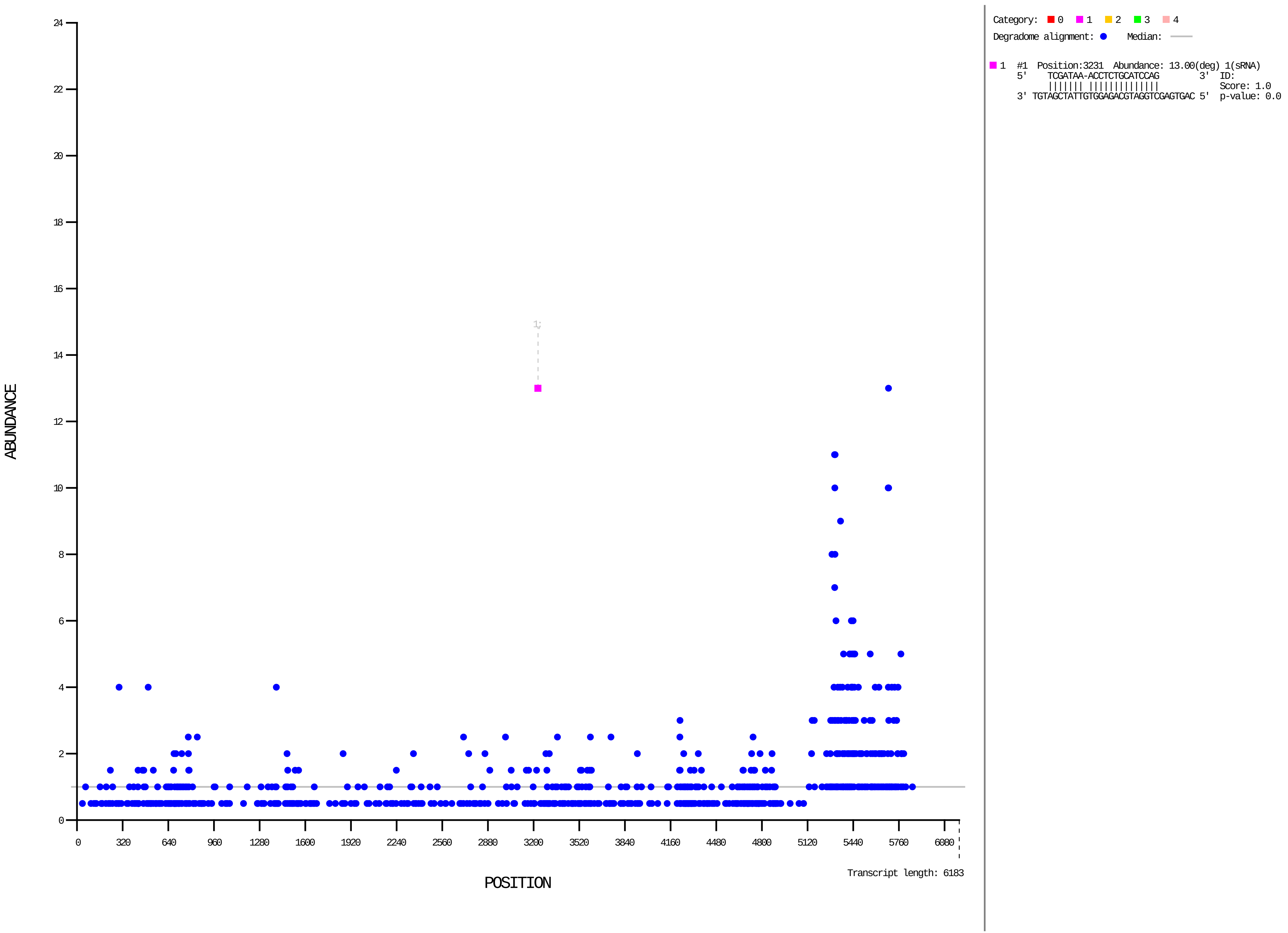

orange1.1t02234.1 gene=orange1.1t02234 CDS=268-1167

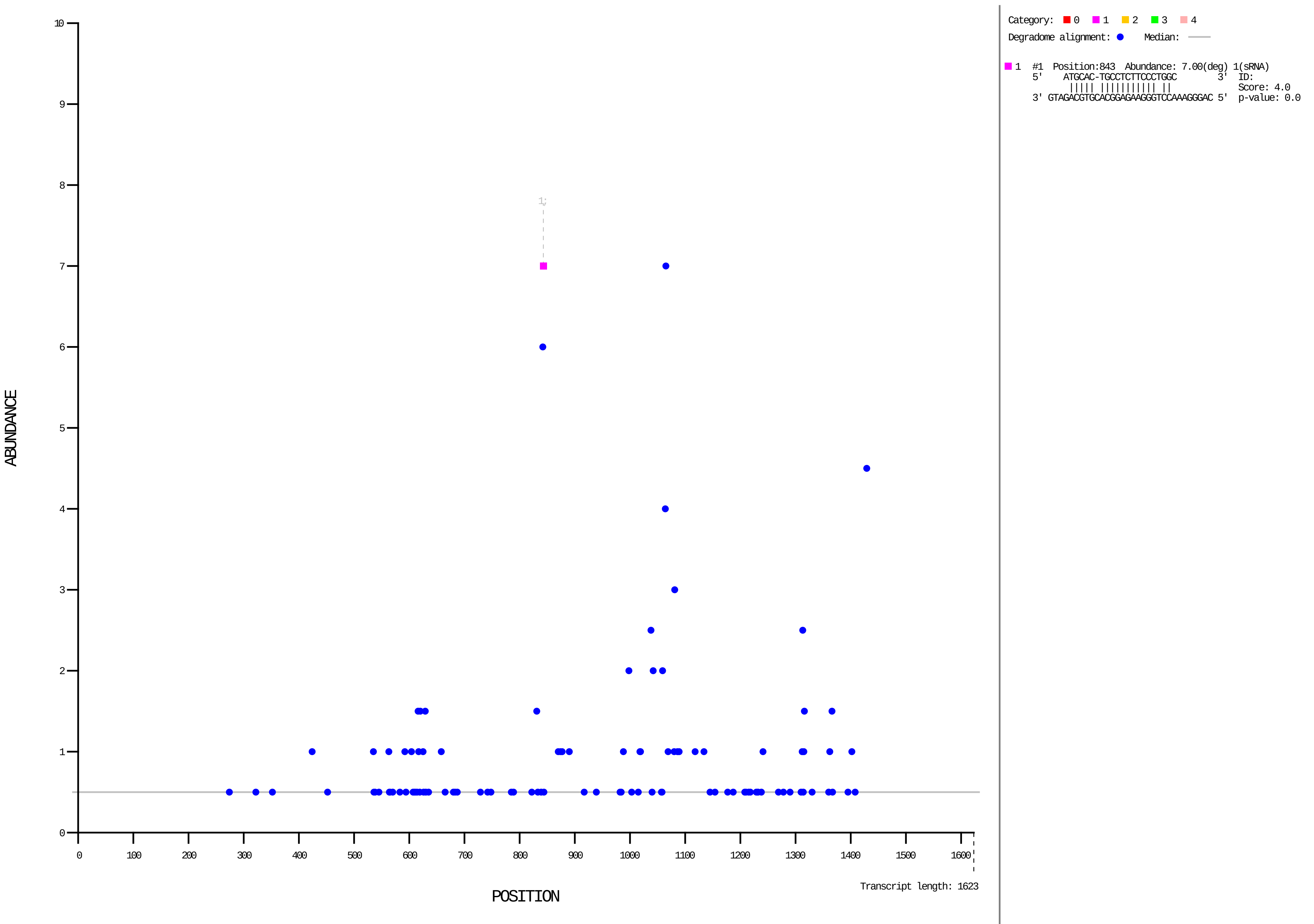

Cs6g16890.2 gene=Cs6g16890 CDS=584-2059

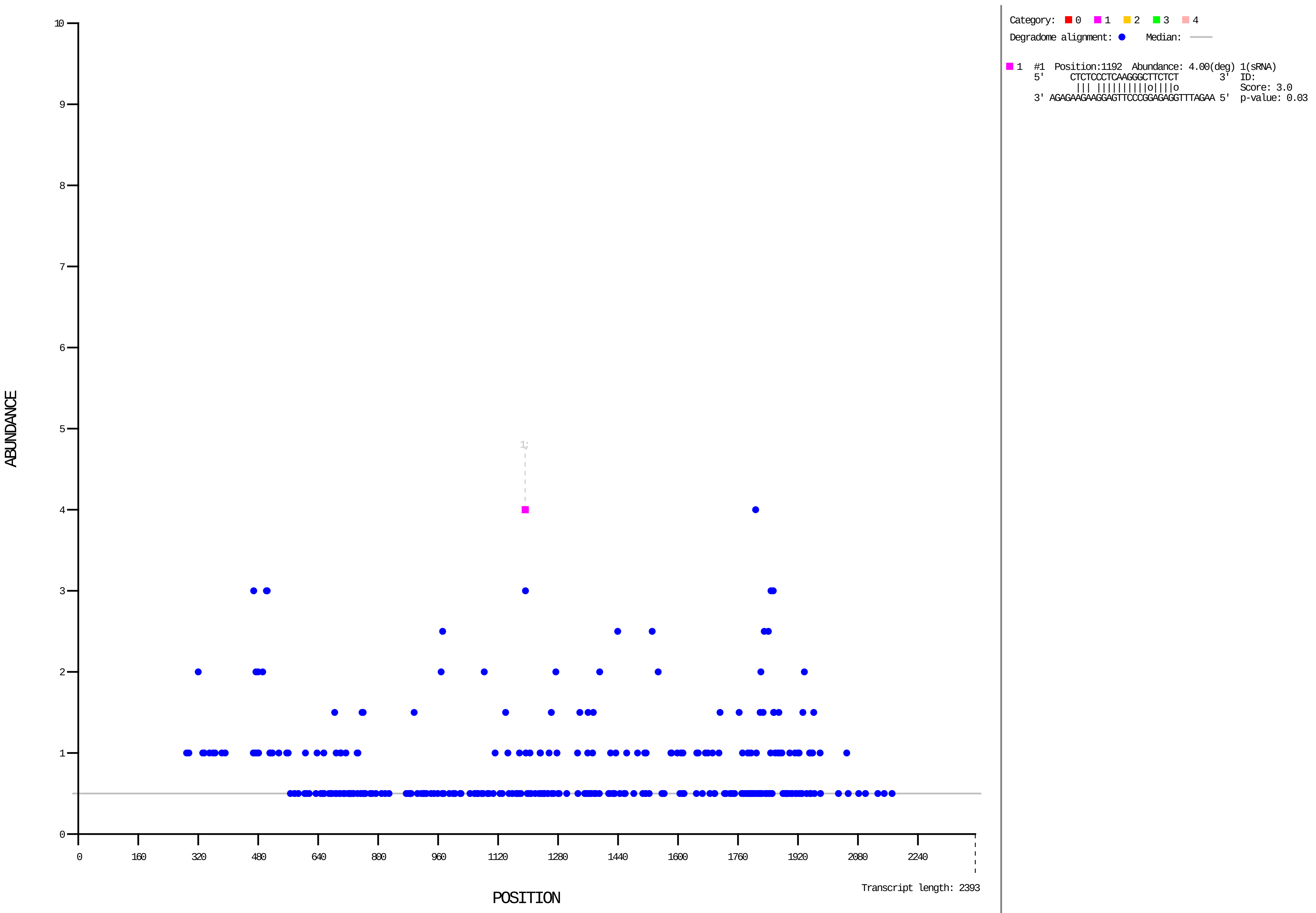

Cs6g16890.1 gene=Cs6g16890 CDS=51-1526

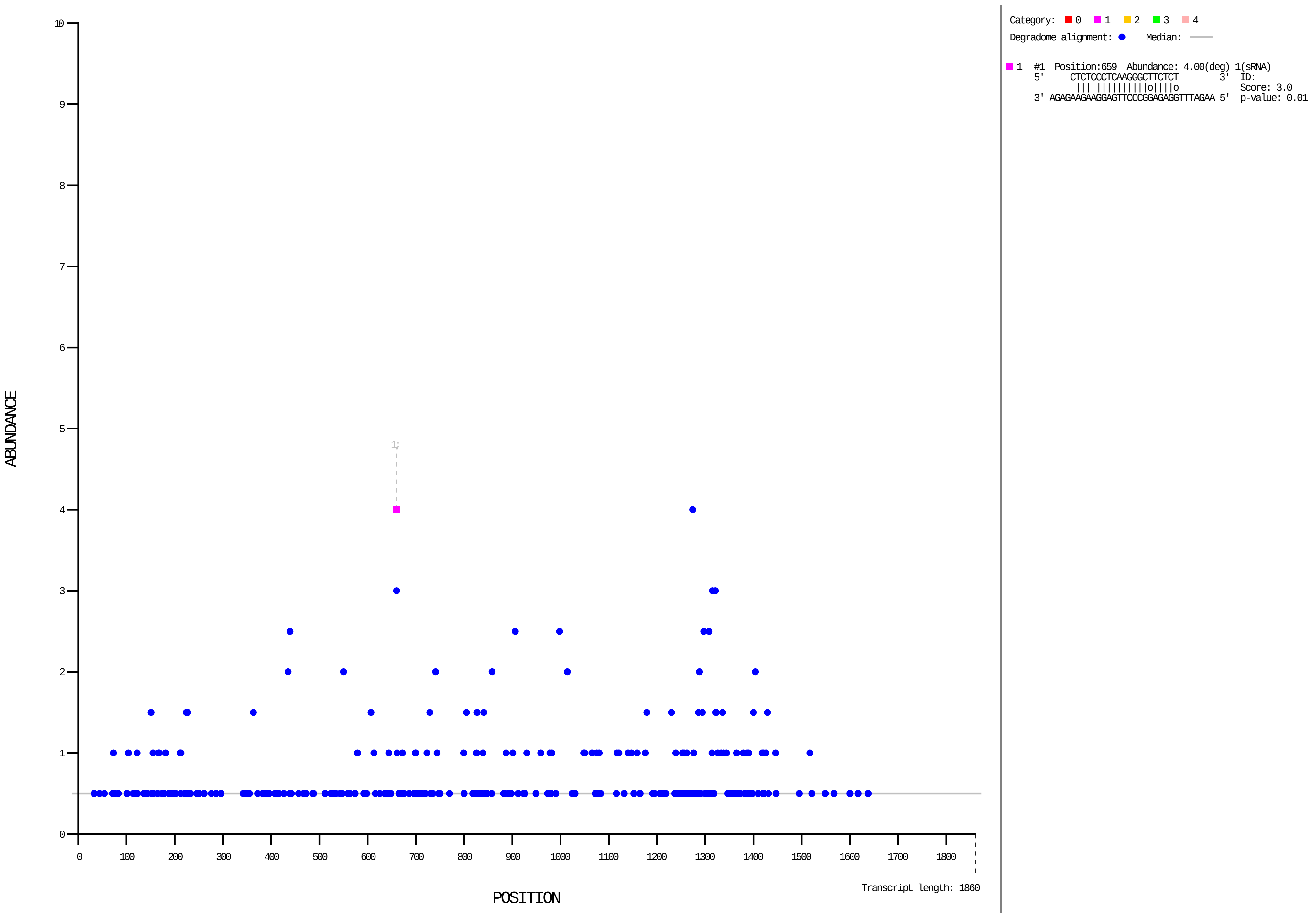

Cs3g26830.1 gene=Cs3g26830 CDS=3227-3550

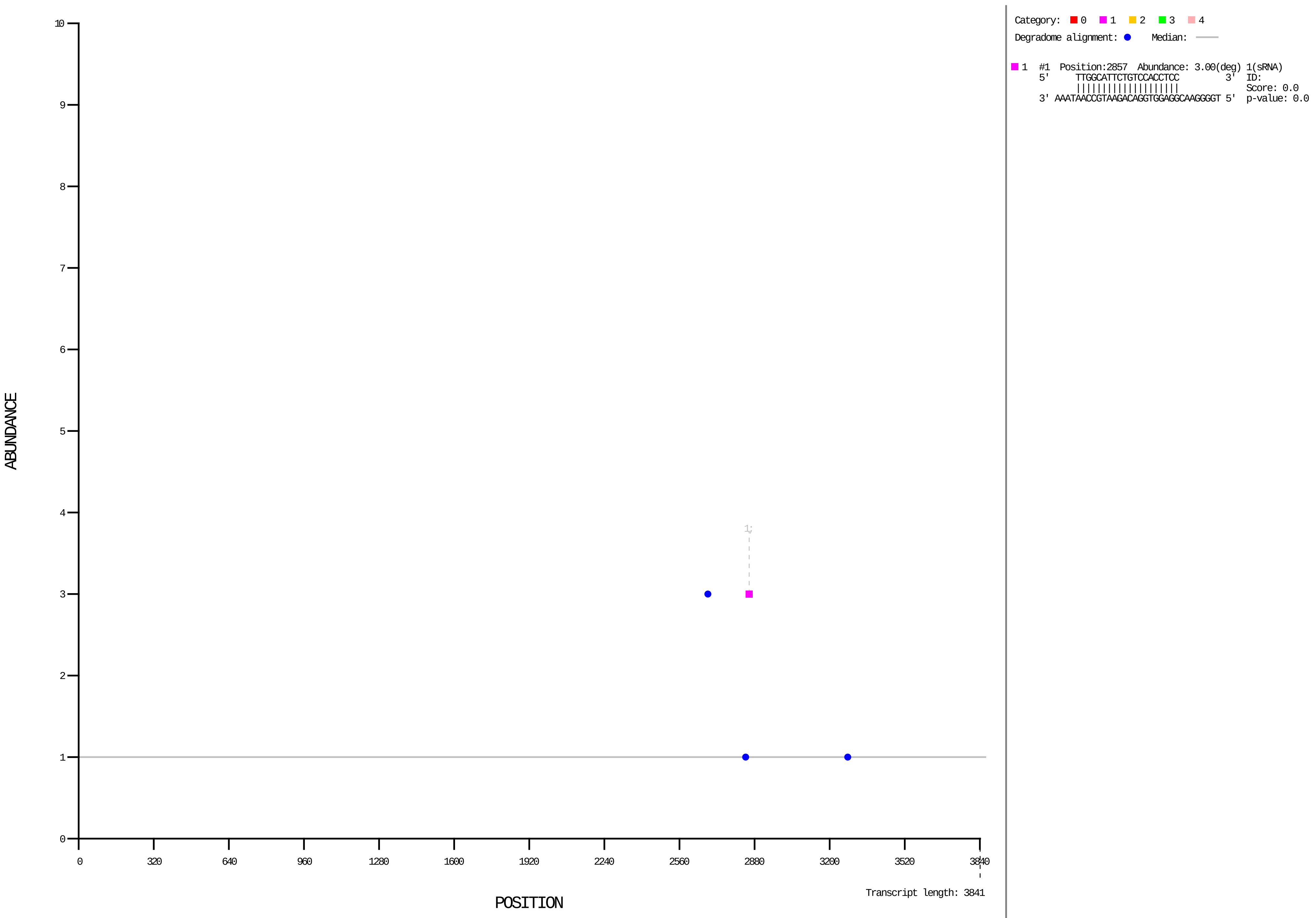

Cs1g24430.1 gene=Cs1g24430 CDS=114-962

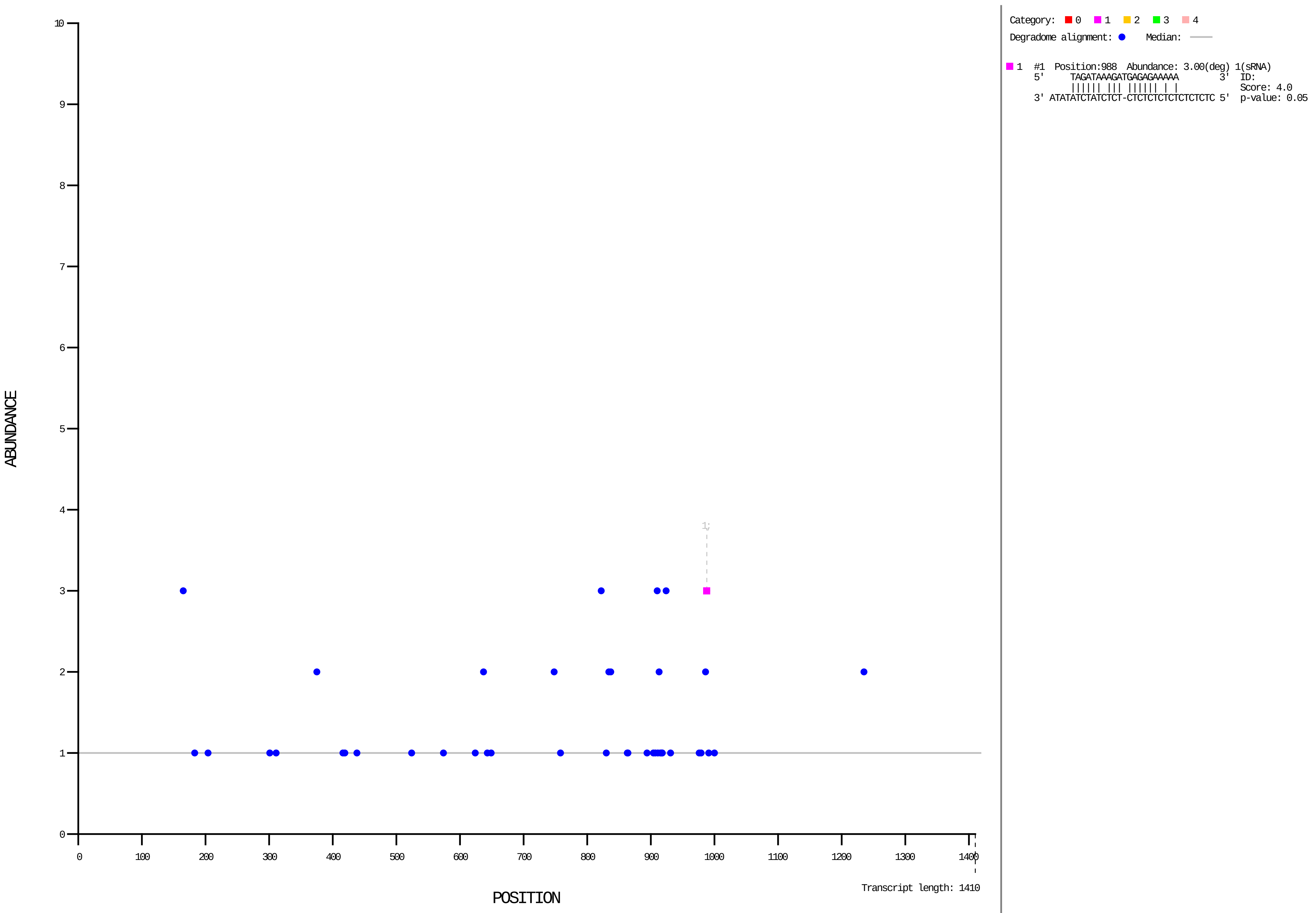

Cs2g30970.1 gene=Cs2g30970 CDS=1-3558

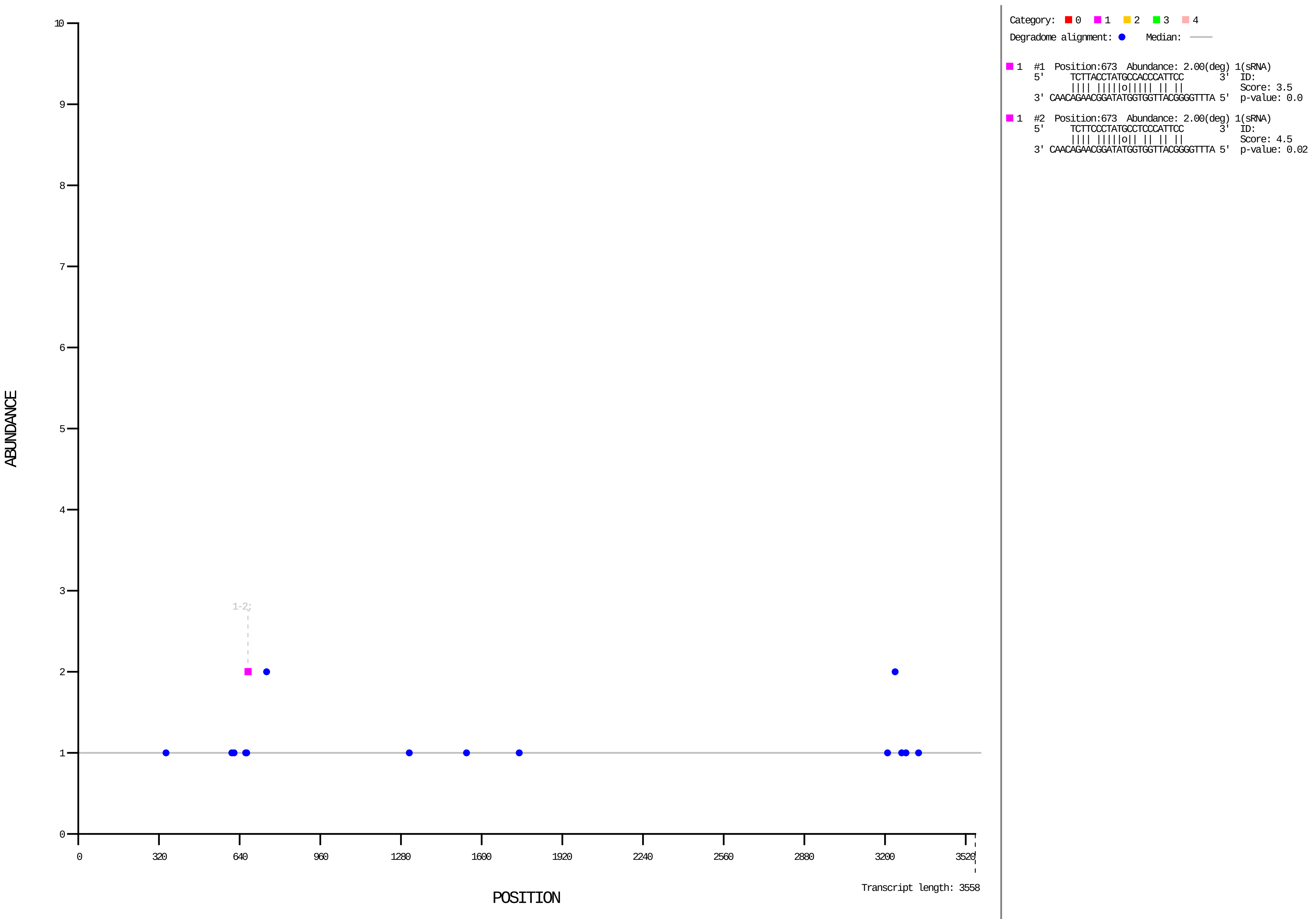

Cs5g08750.1 gene=Cs5g08750 CDS=1-528

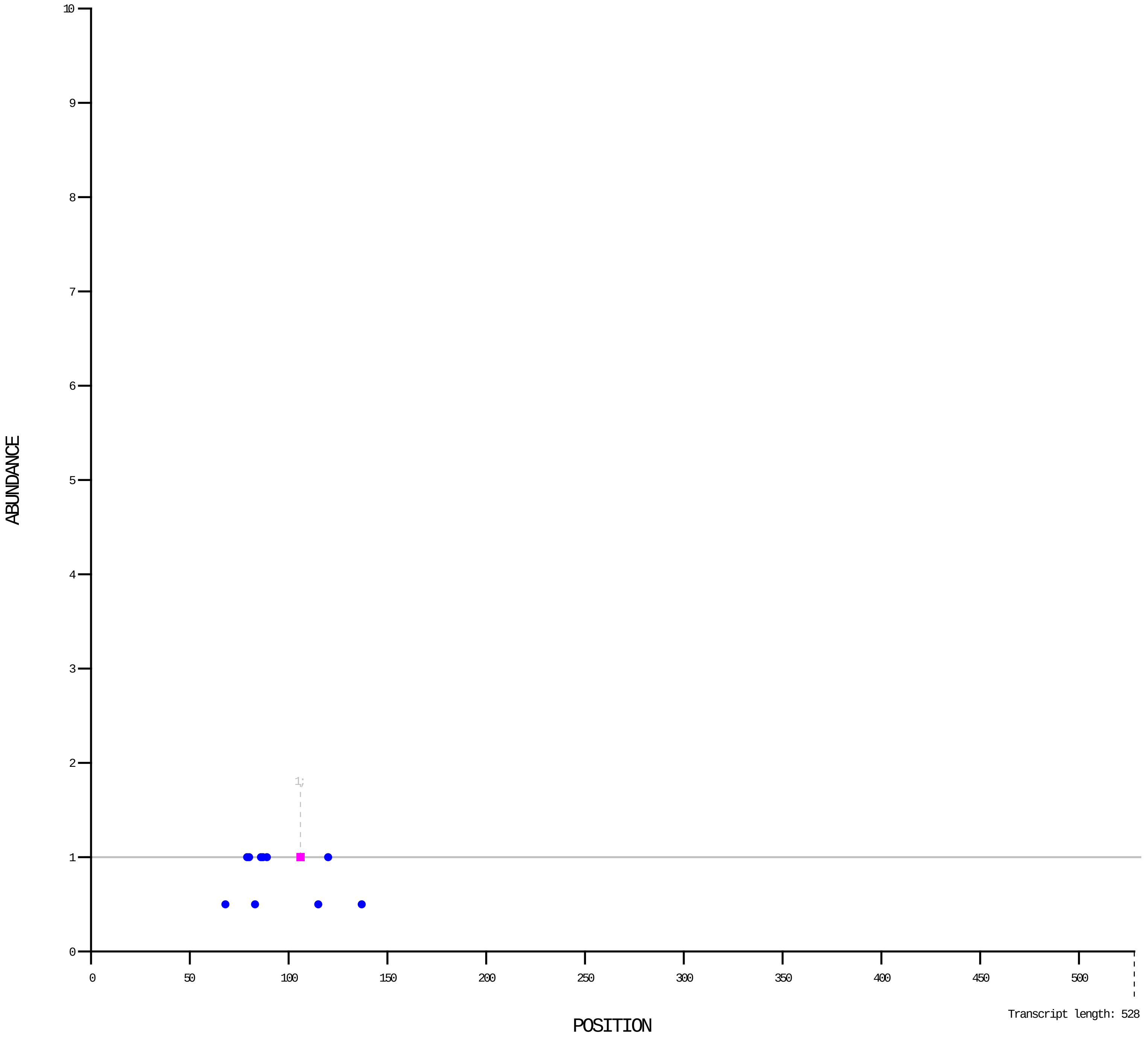

Category: 0 1 2 3 4

Degradome alignment: Median:

```
1  #1  Position:106  Abundance: 1.00(deg) 1(sRNA)
   5'     TCTTGCCCACCCCTCCCATTCC        3'  ID:
          ||||||| || ||o||| ||||            Score: 3.5
   3' GAACAGAACGGATGAGGGGGG-AAGGGTGGAT 5'  p-value: 0.01
```

Cs9g07750.1 gene=Cs9g07750 CDS=208-1602

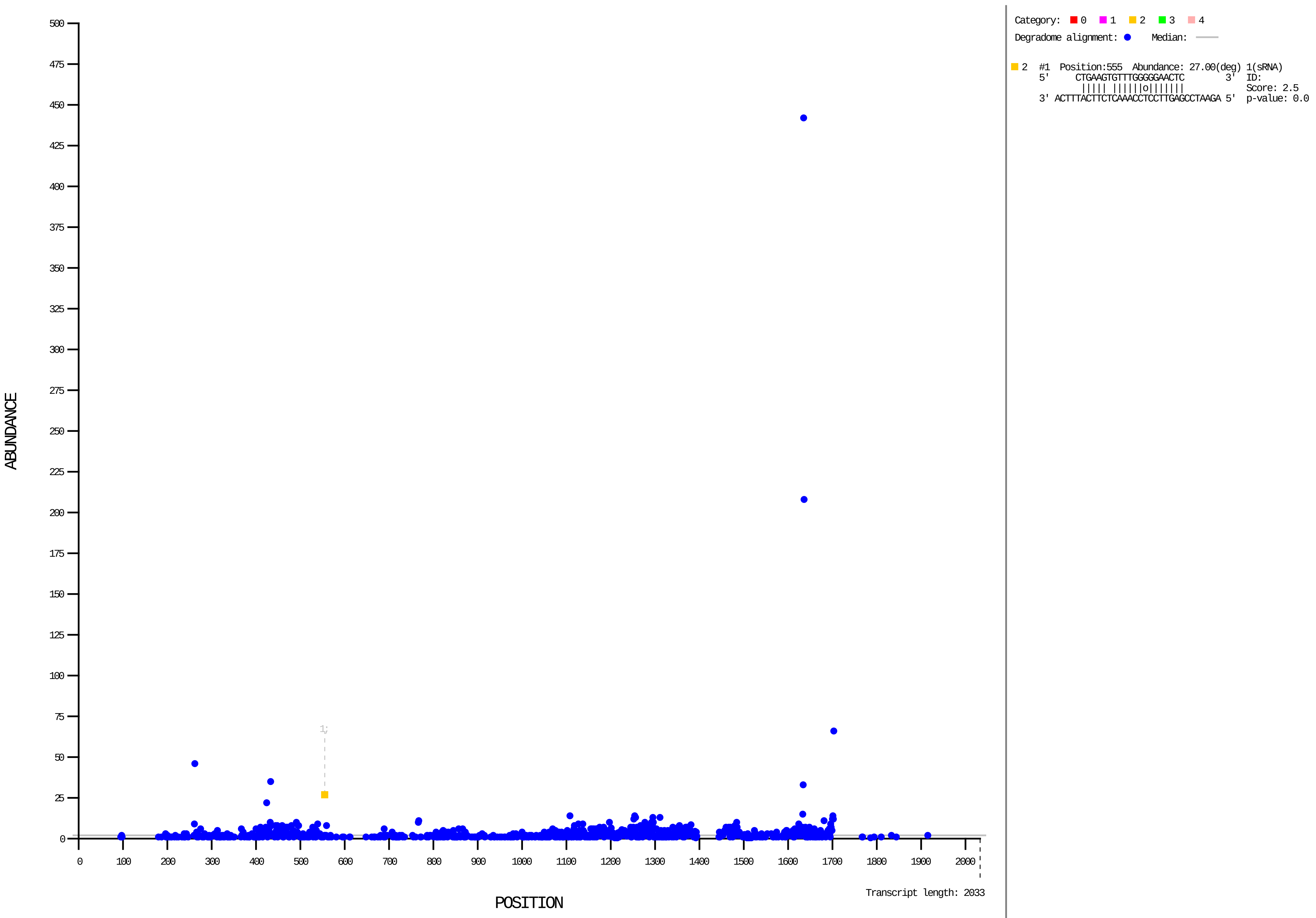

Cs2g14270.1 gene=Cs2g14270 CDS=985-2697

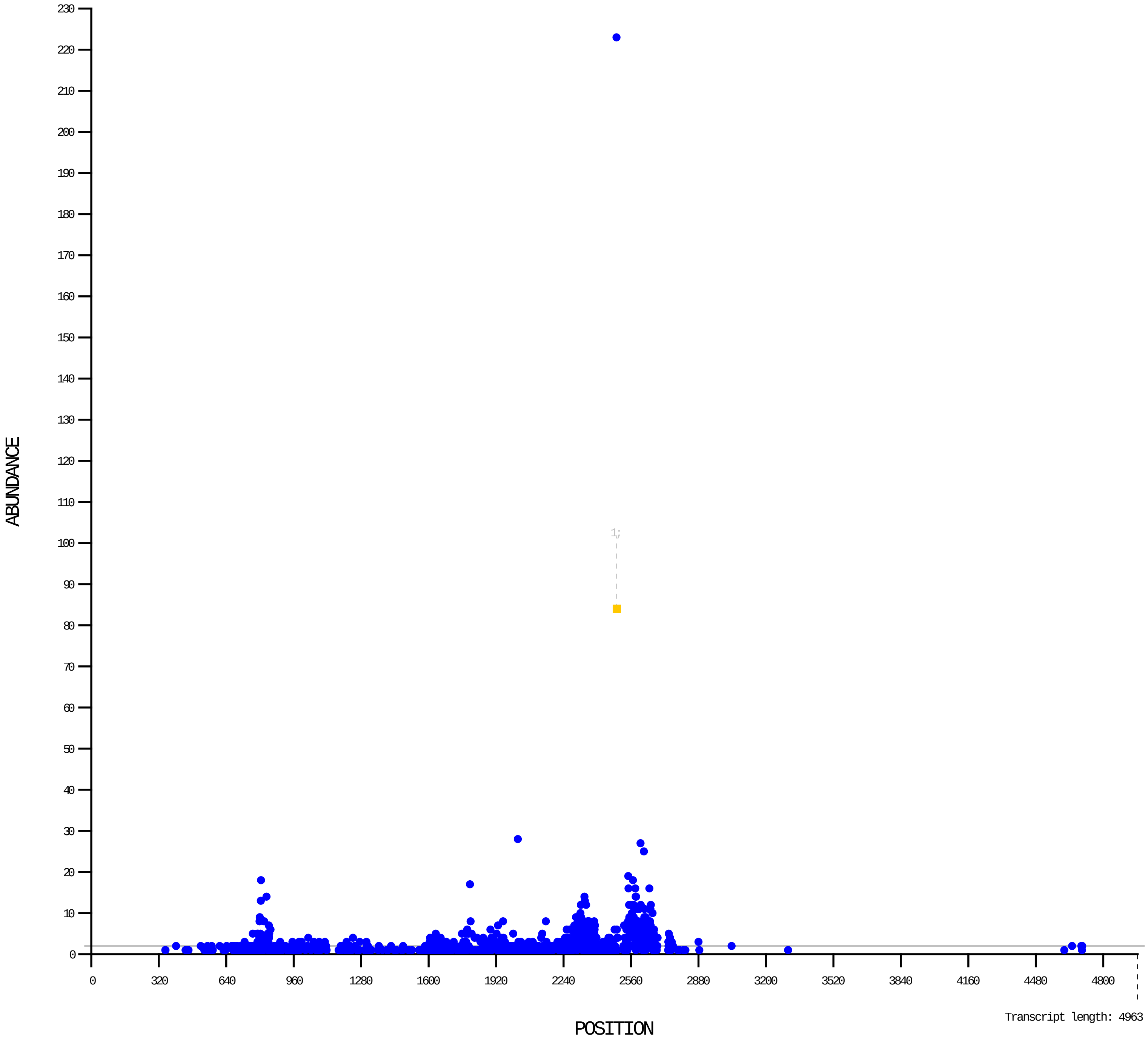

Category: 0 1 2 3 4
Degradome alignment: Median:

2 #1 Position:2492 Abundance: 84.00(deg) 1(sRNA)

```
5'     ATCCAAAGGGATCGCATTGATC      3'  ID:
       |||||||||||||||||| ||           Score: 1.0
3' GCTGTAGGTTTCCCTAGCGTAAC-AGAGTATG 5'  p-value: 0.01
```

Cs1g09660.1 gene=Cs1g09660 CDS=74-1249

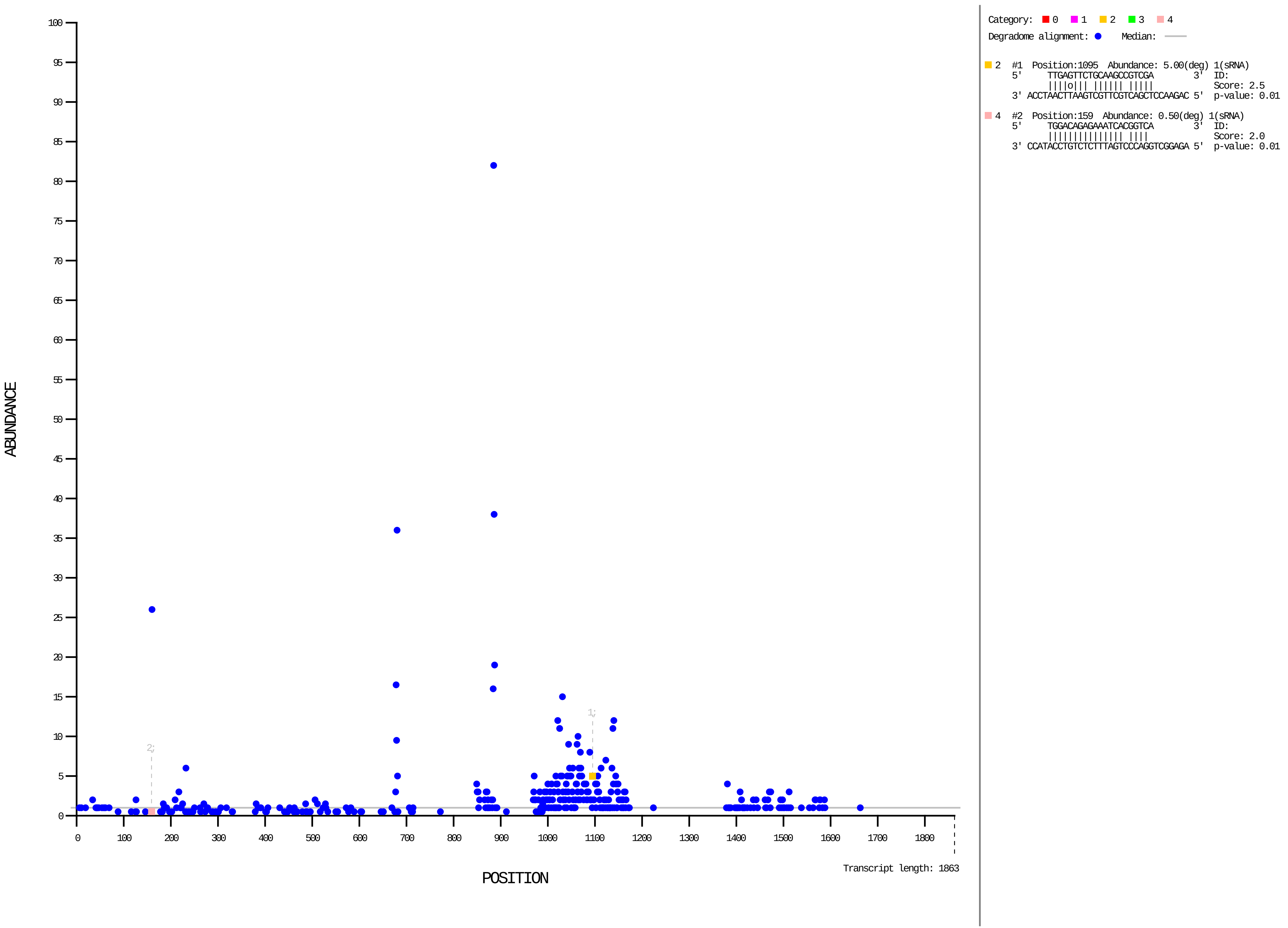

Cs5g32500.1 gene=Cs5g32500 CDS=368-2125

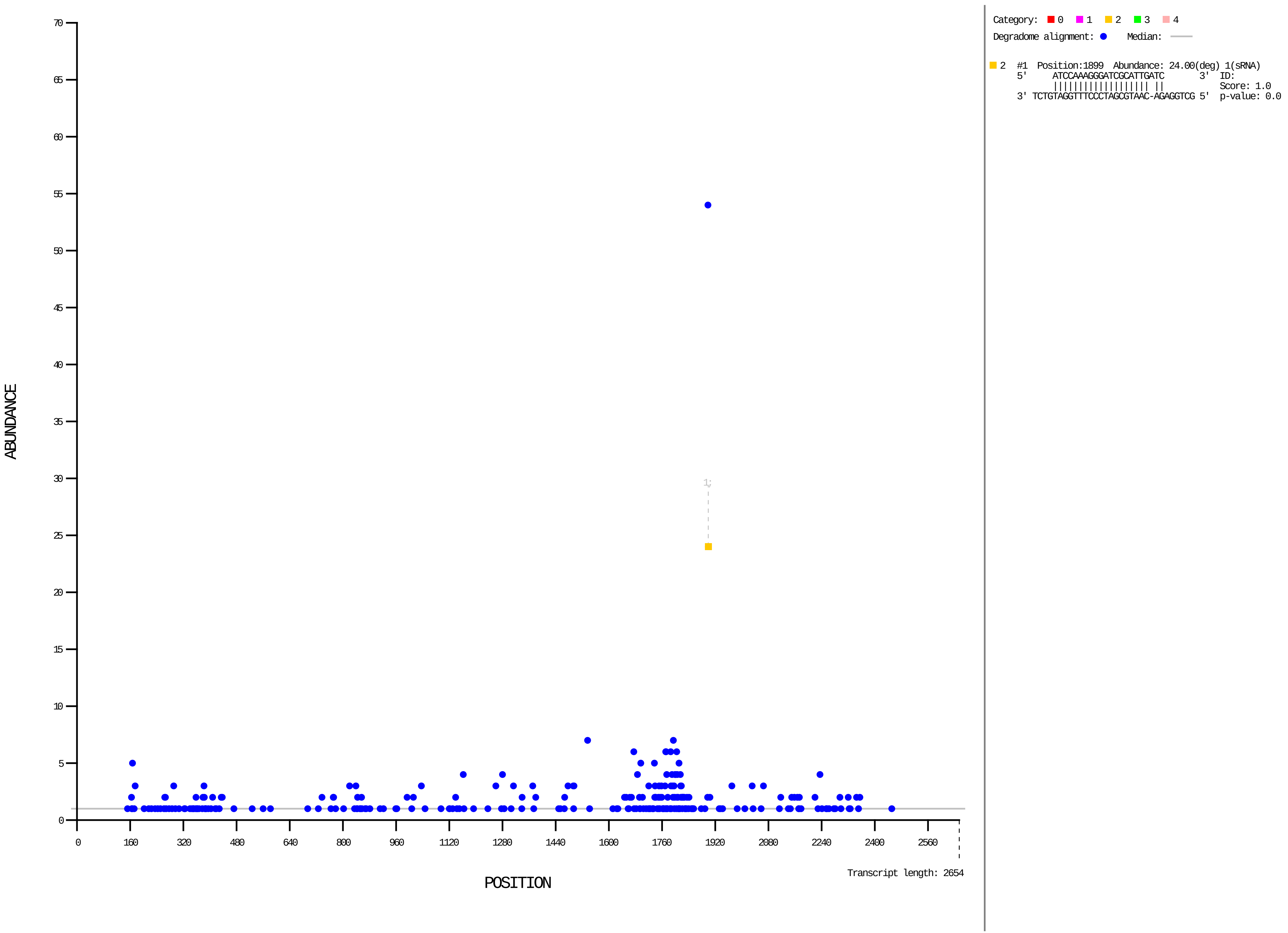

Cs7g27790.1 gene=Cs7g27790 CDS=309-1775

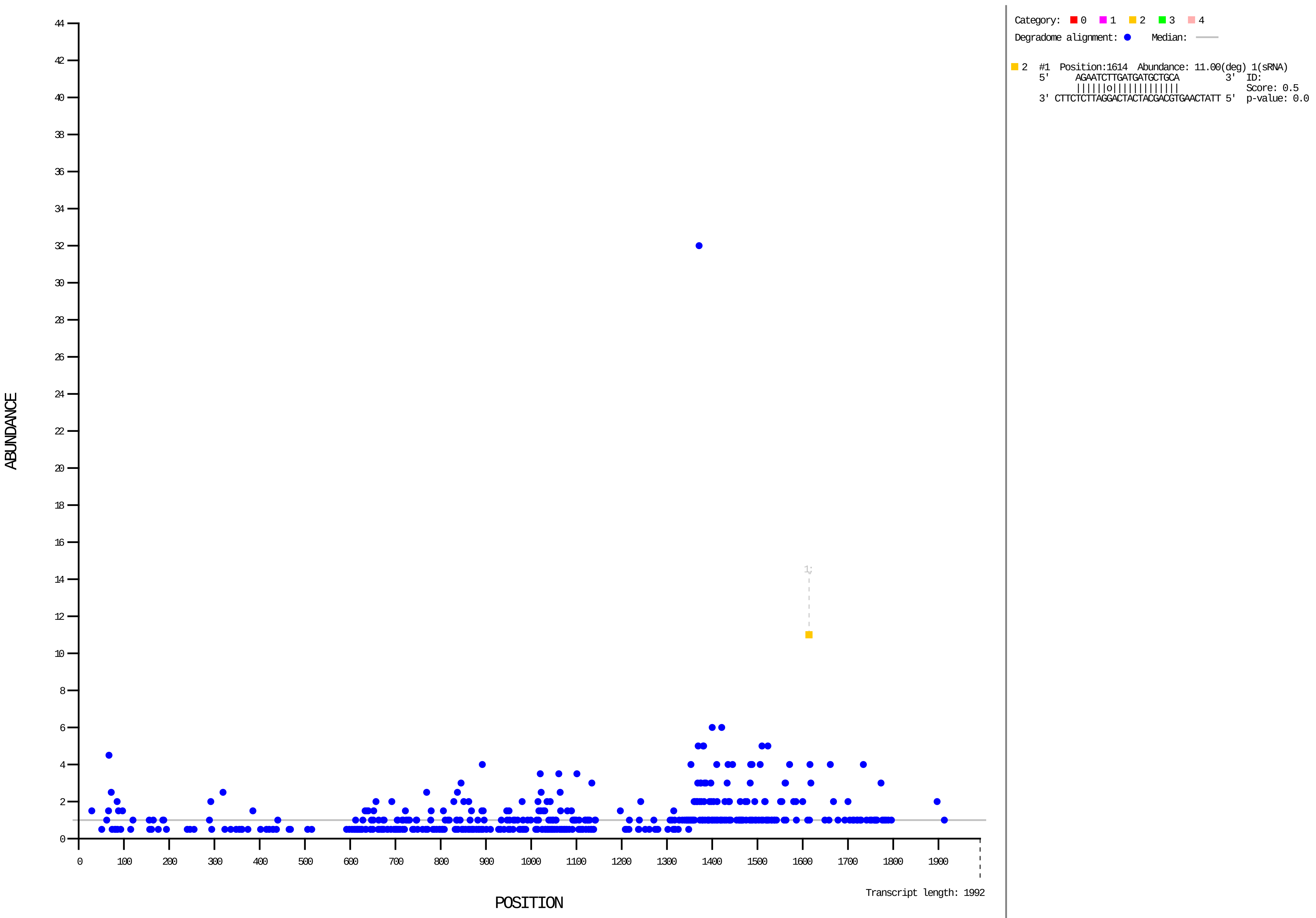

orange1.1t02265.2 gene=orange1.1t02265 CDS=185-1345

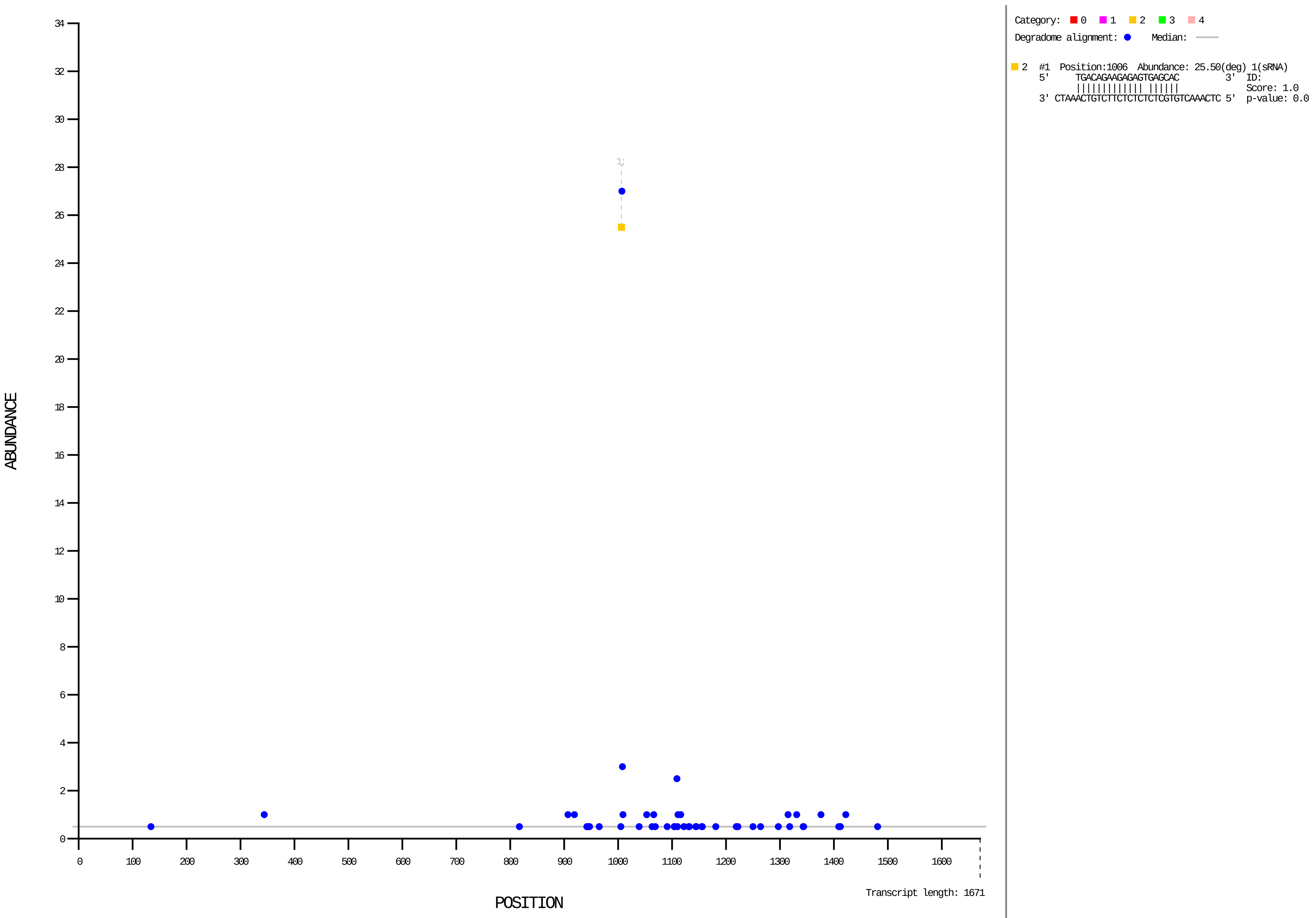

orange1.1t02265.1 gene=orange1.1t02265 CDS=185-1321

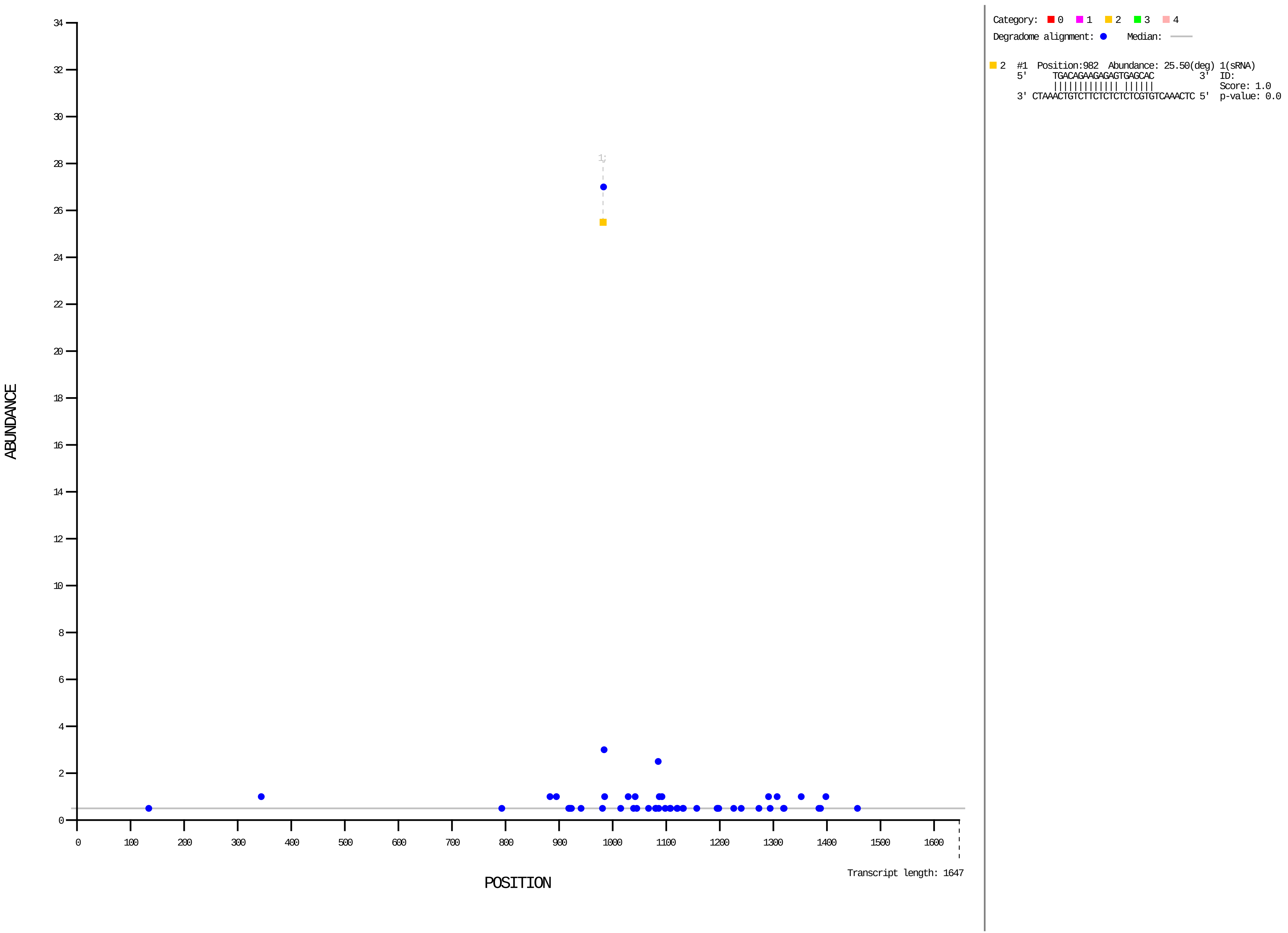

Cs4g18660.3 gene=Cs4g18660 CDS=320-1201

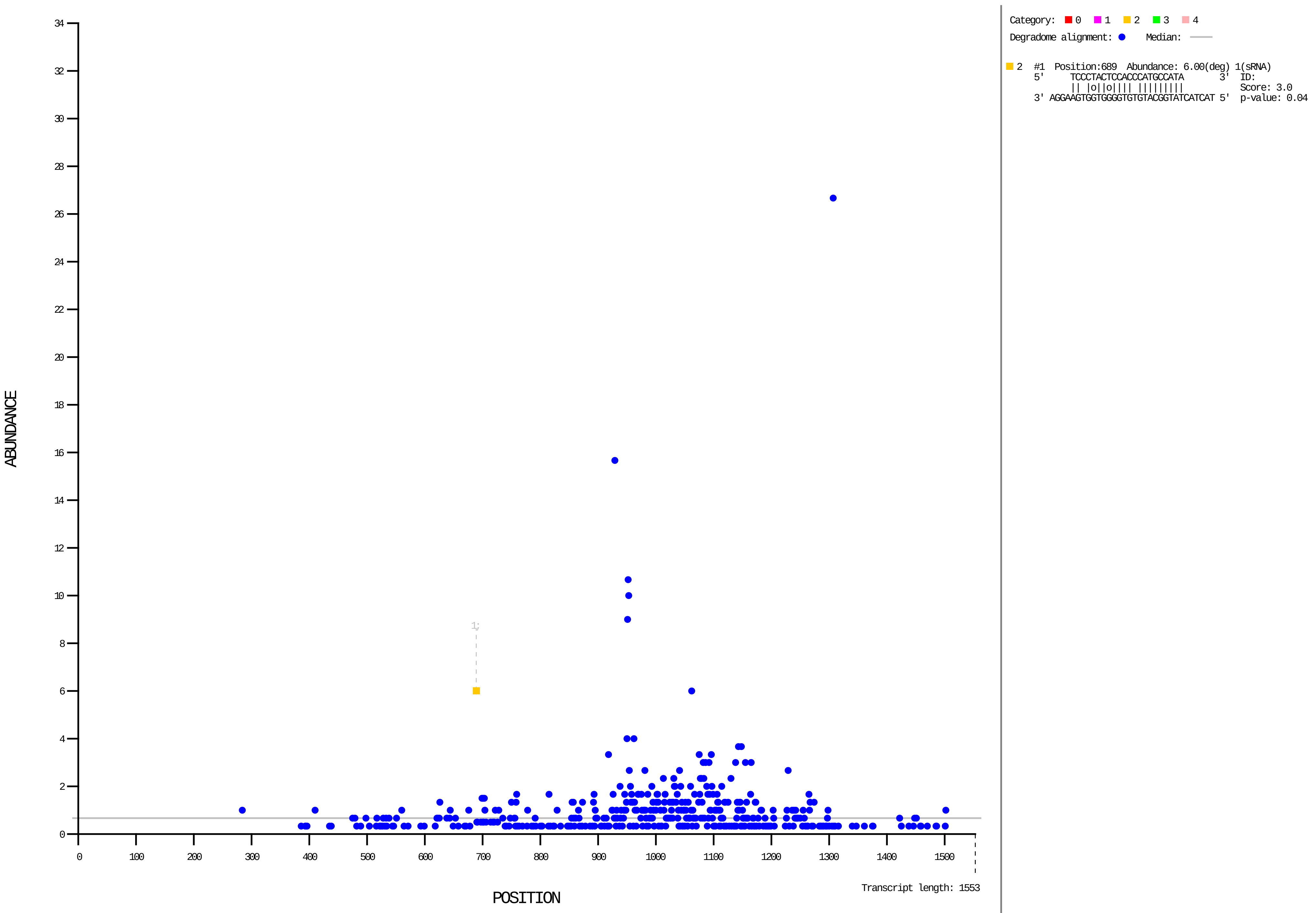

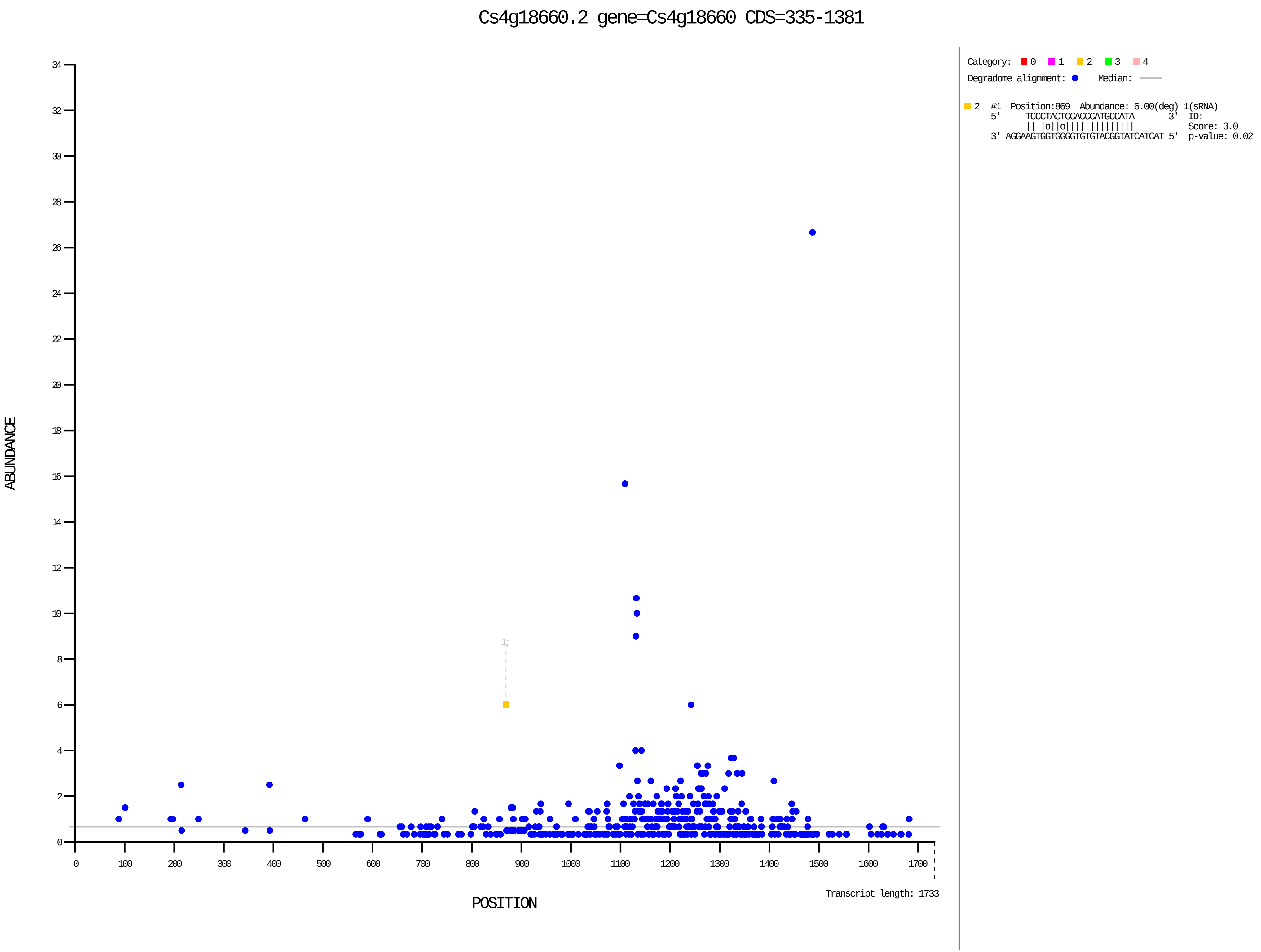

Cs4g18660.2 gene=Cs4g18660 CDS=335-1381
ABUNDANCE
POSITION
Transcript length: 1733
Category: 0 1 2 3 4
Degradome alignment:
Median:
2 #1 Position:869 Abundance: 6.00(deg) 1(sRNA)
5' TCCCTACTCCACCCATGCCATA 3' ID:
|| |o||o|||| |||||||||
Score: 3.0
3' AGGAAGTGGTGGGGTGTGTACGGTATCATCAT 5' p-value: 0.02

orange1.1t03059.3 gene=orange1.1t03059 CDS=574-2721

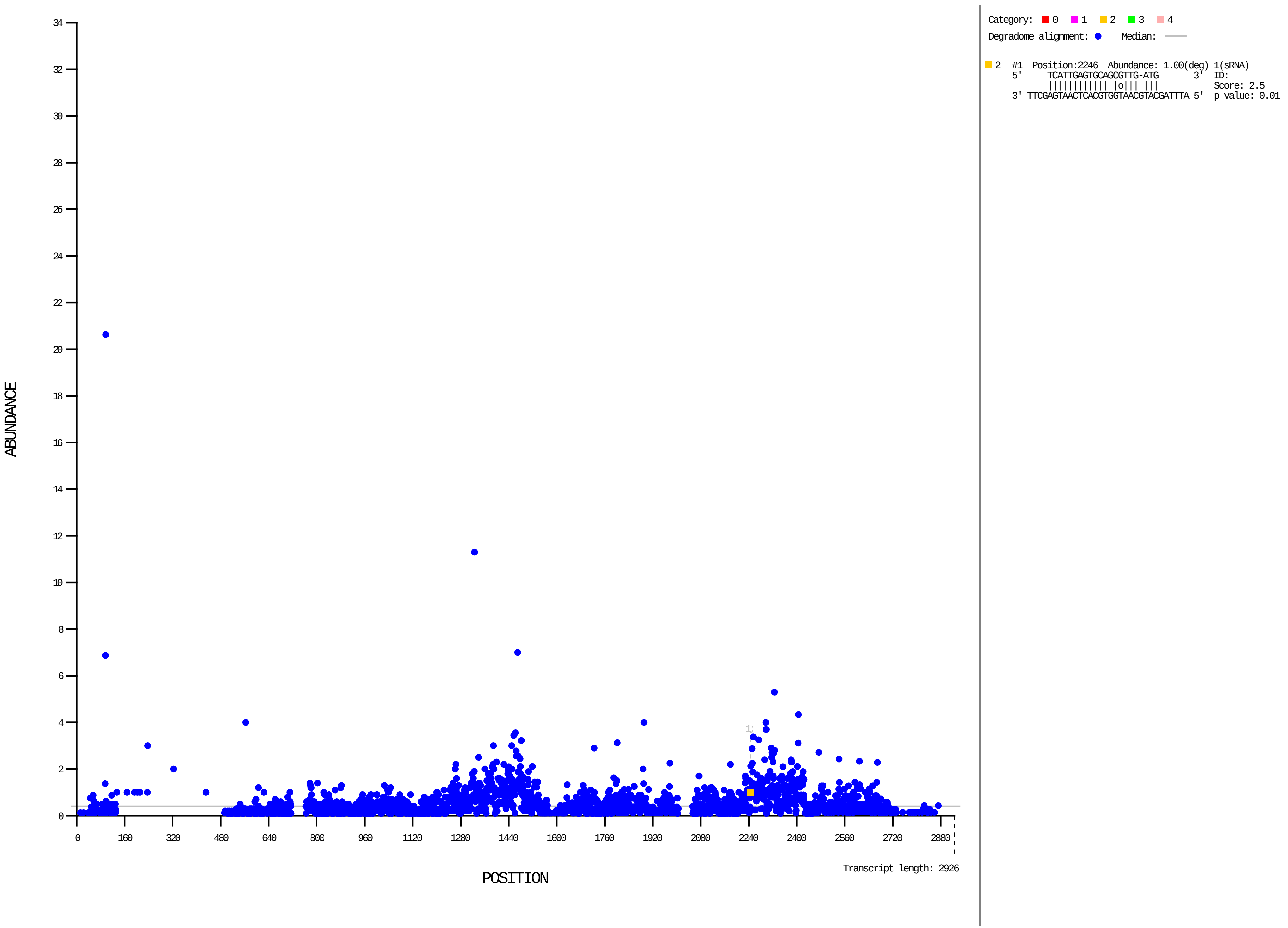

orange1.1t03059.4 gene=orange1.1t03059 CDS=276-2423

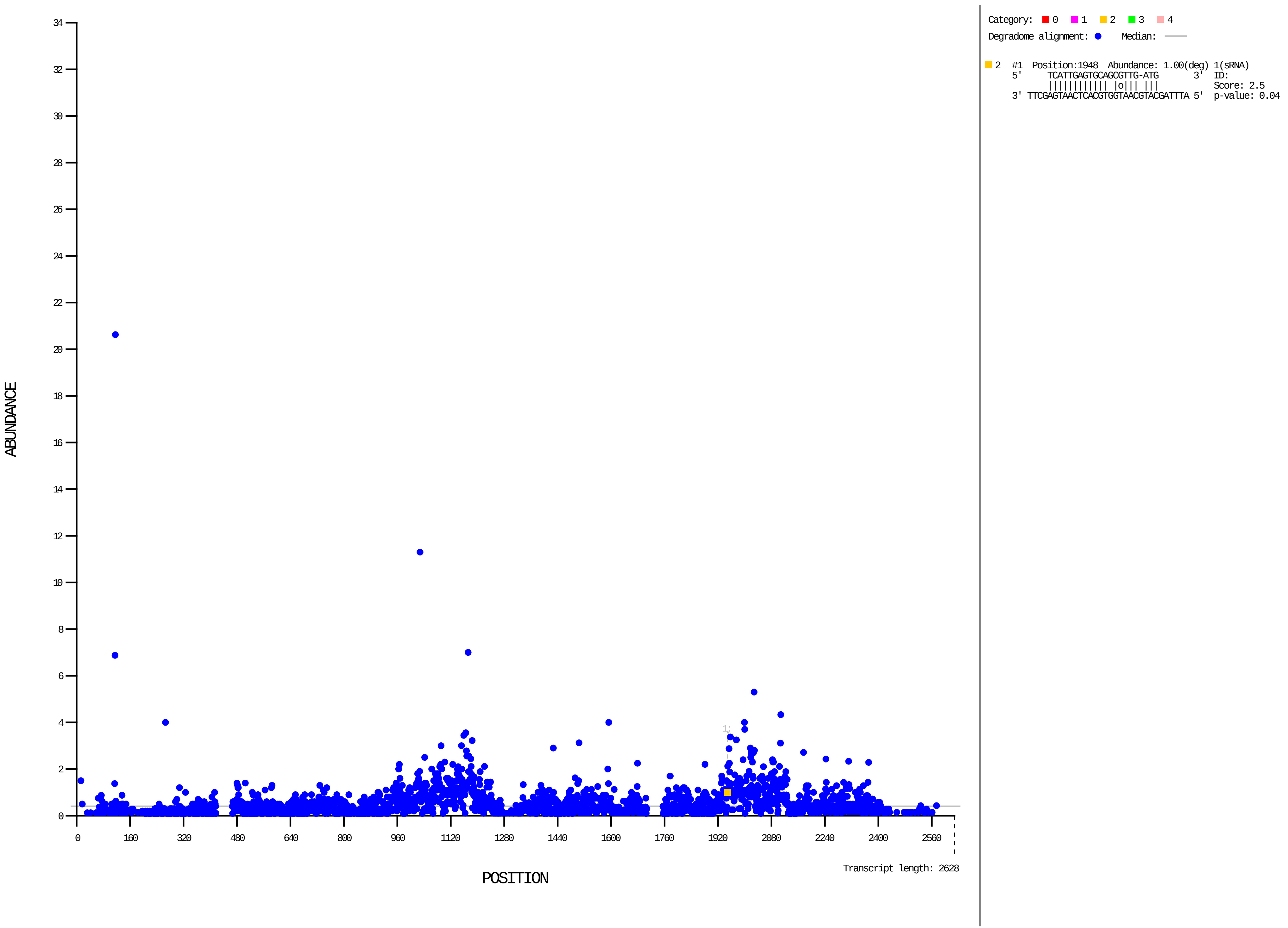

orange1.1t03059.7 gene=orange1.1t03059 CDS=257-2188

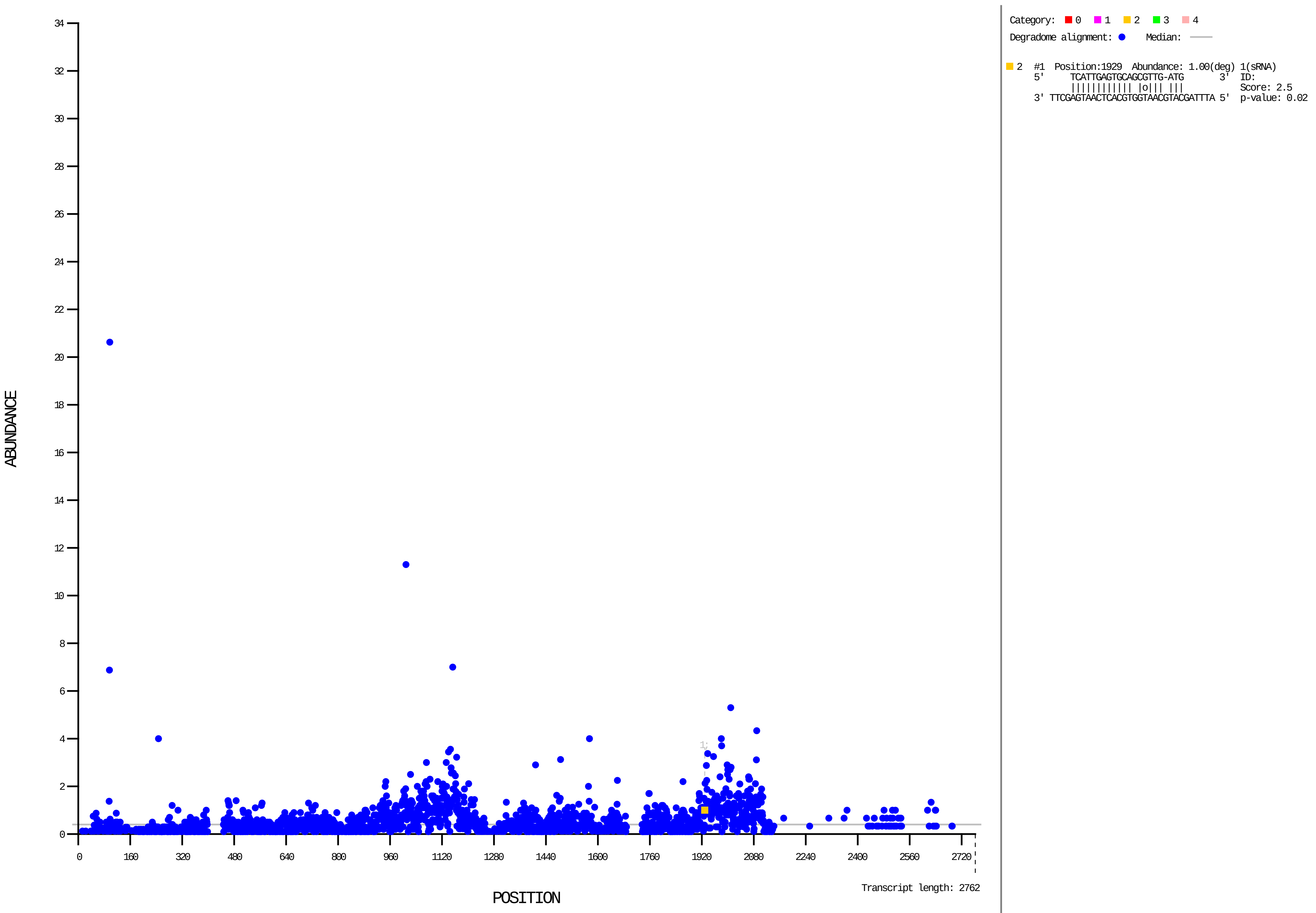

orange1.1t03059.1 gene=orange1.1t03059 CDS=257-2413

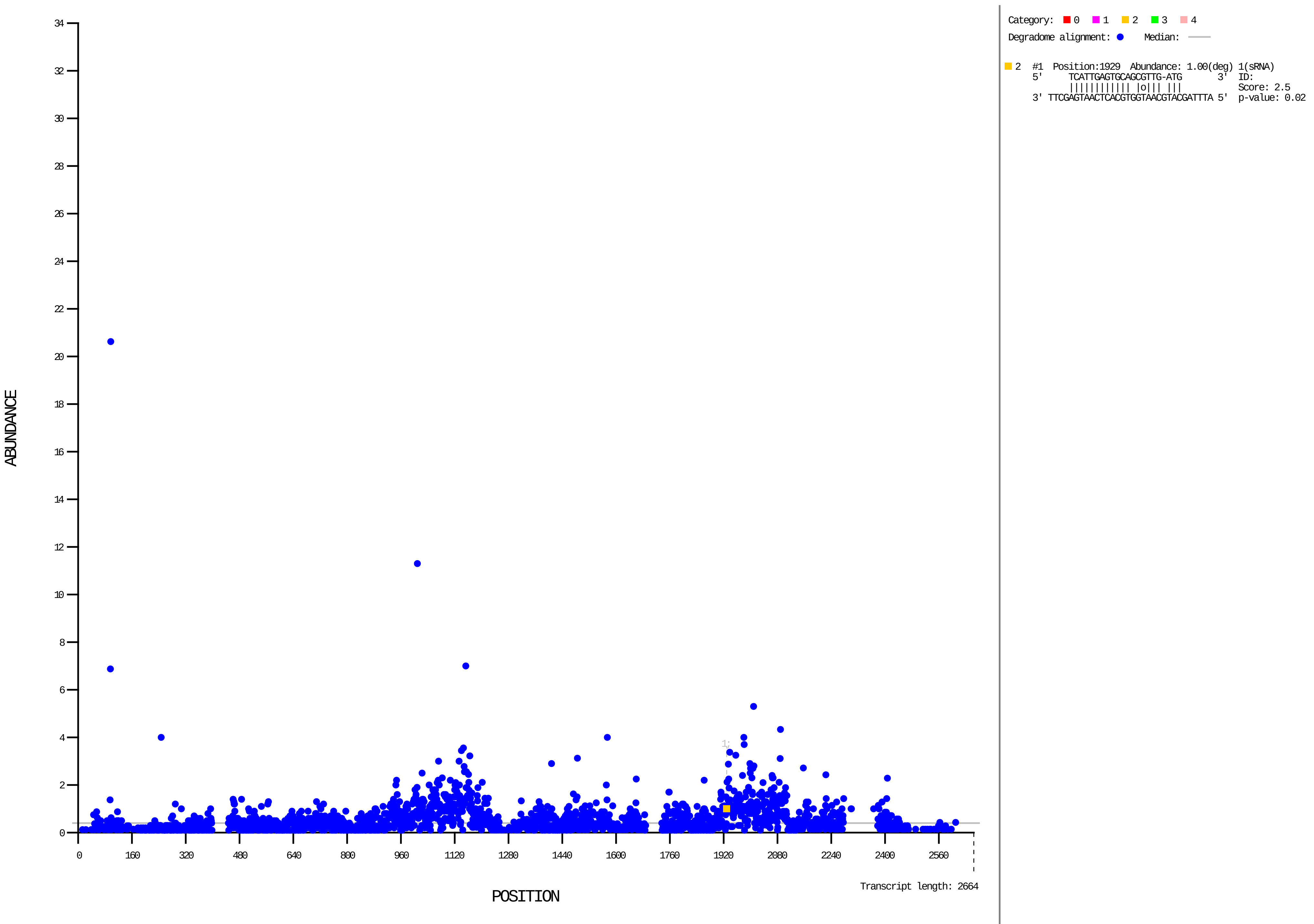

orange1.1t03059.6 gene=orange1.1t03059 CDS=285-2258

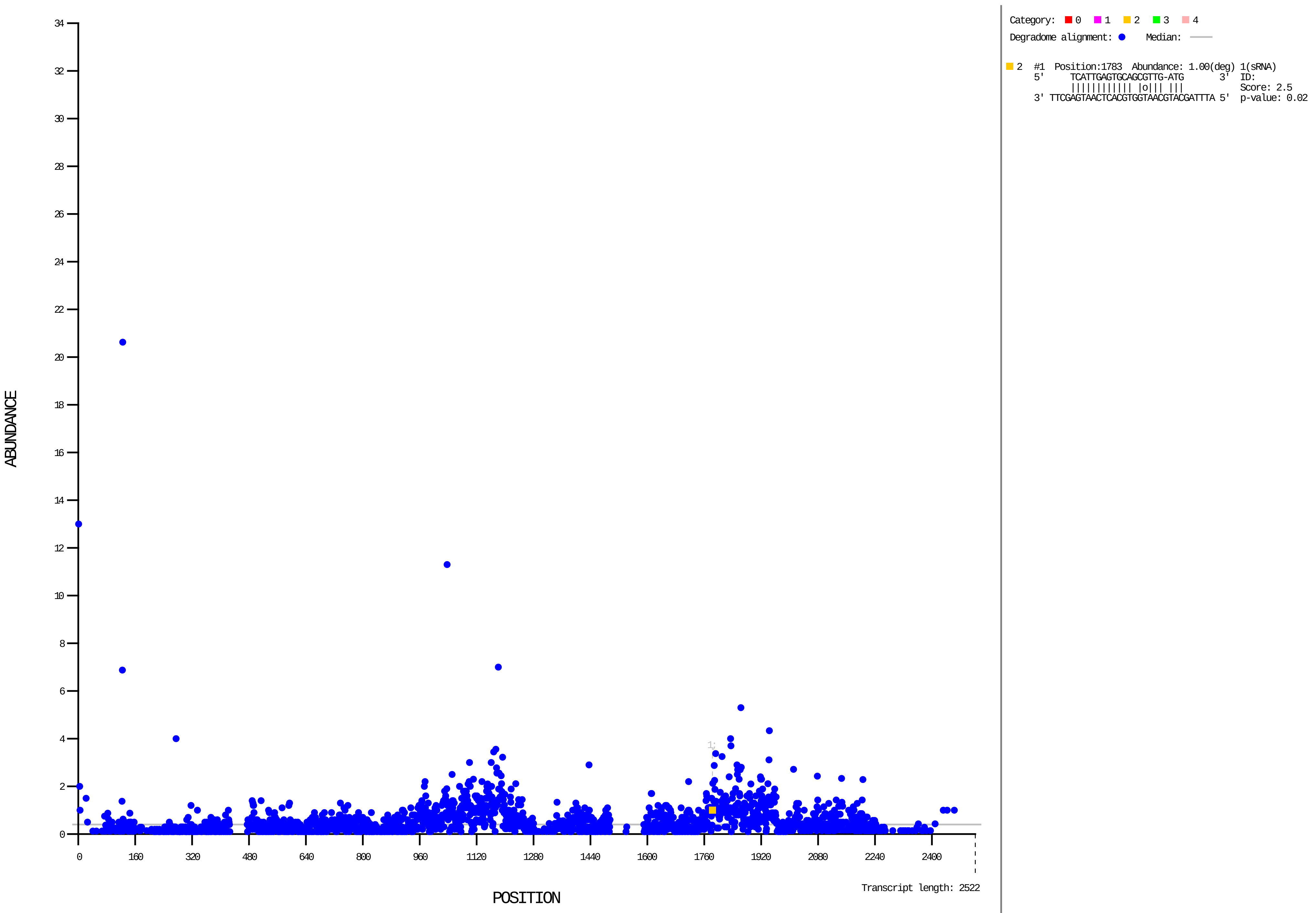

orange1.1t03059.9 gene=orange1.1t03059 CDS=257-2023

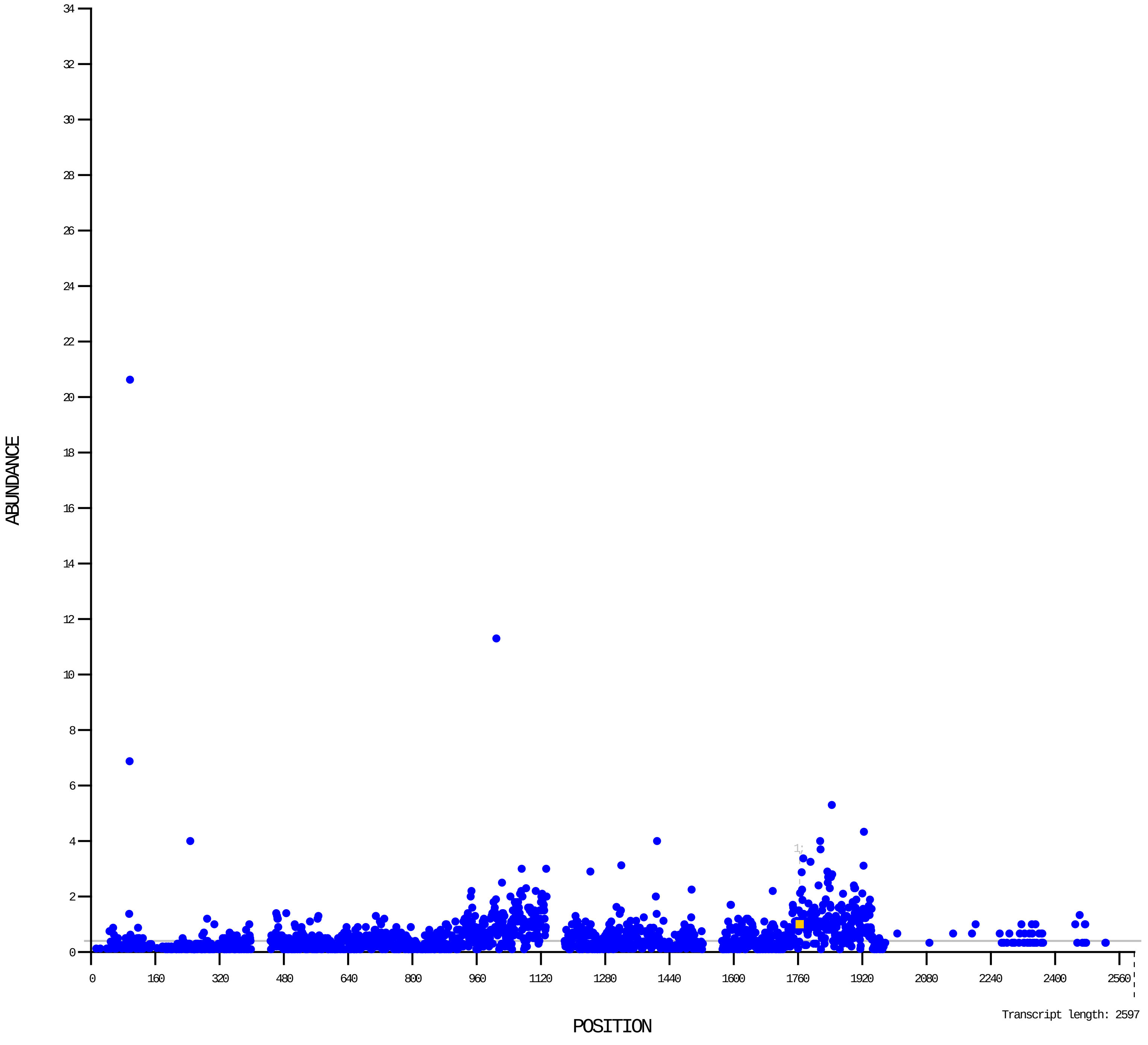

Category: 0 1 2 3 4

Degradome alignment: ● Median: —

```
2  #1  Position:1764  Abundance: 1.00(deg) 1(sRNA)
   5'     TCATTGAGTGCAGCGTTG-ATG       3'  ID:
          |||||||||||| |o||| |||           Score: 2.5
   3' TTCGAGTAACTCACGTGGTAACGTACGATTTA 5'  p-value: 0.03
```

Cs7g02980.1 gene=Cs7g02980 CDS=528-1901

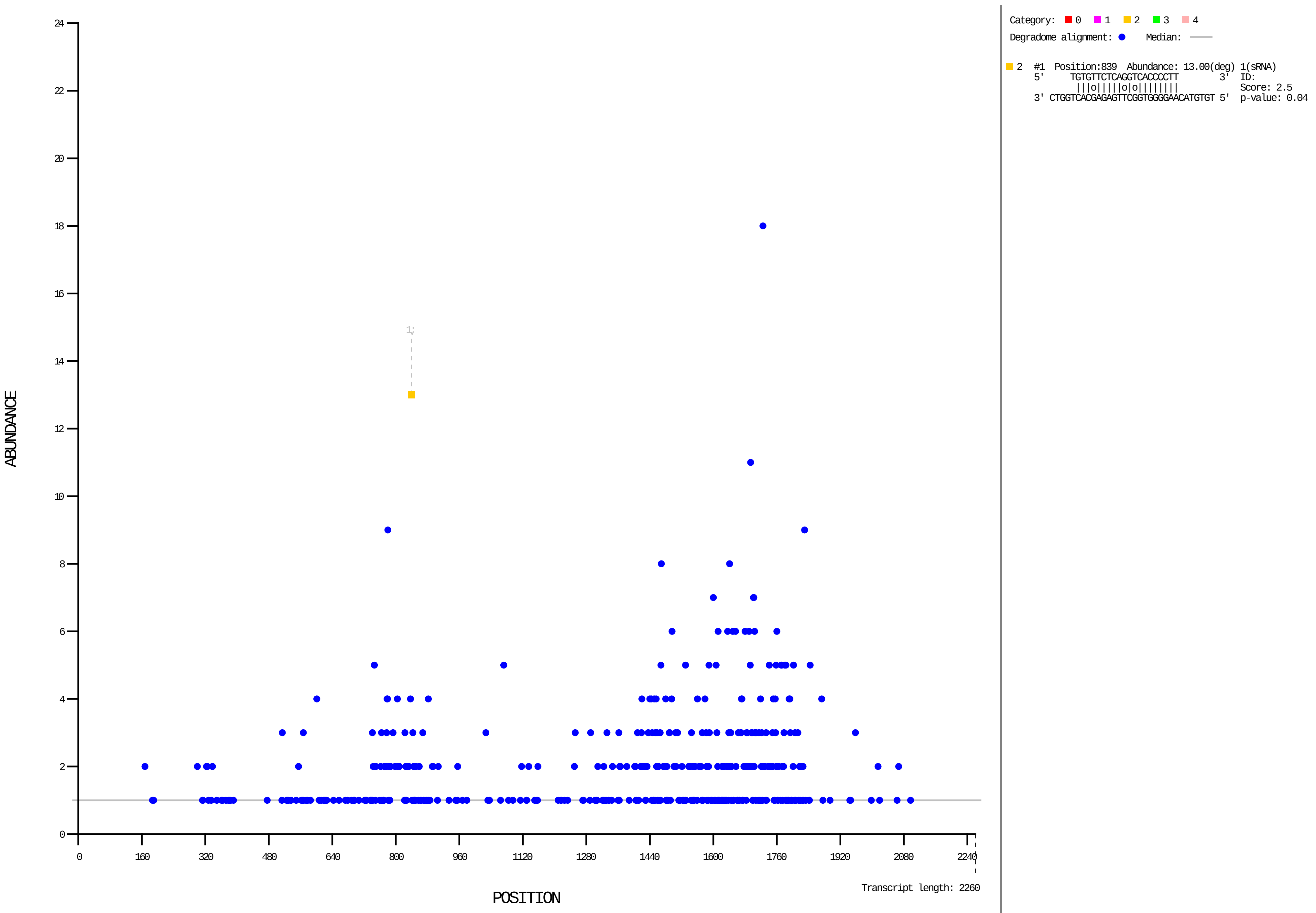

orange1.1t04055.1 gene=orange1.1t04055 CDS=576-2135

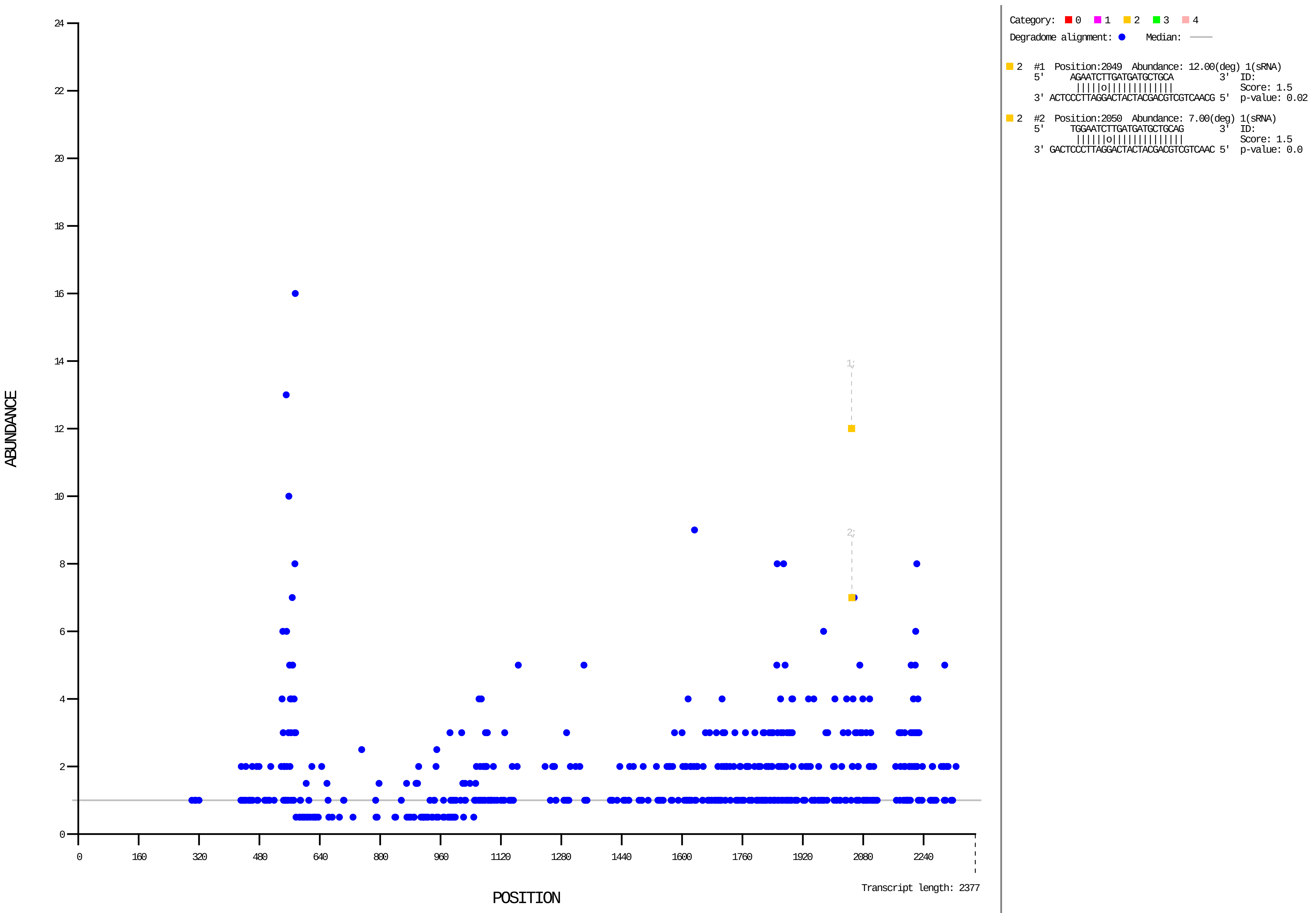

Cs4g05570.2 gene=Cs4g05570 CDS=263-1081

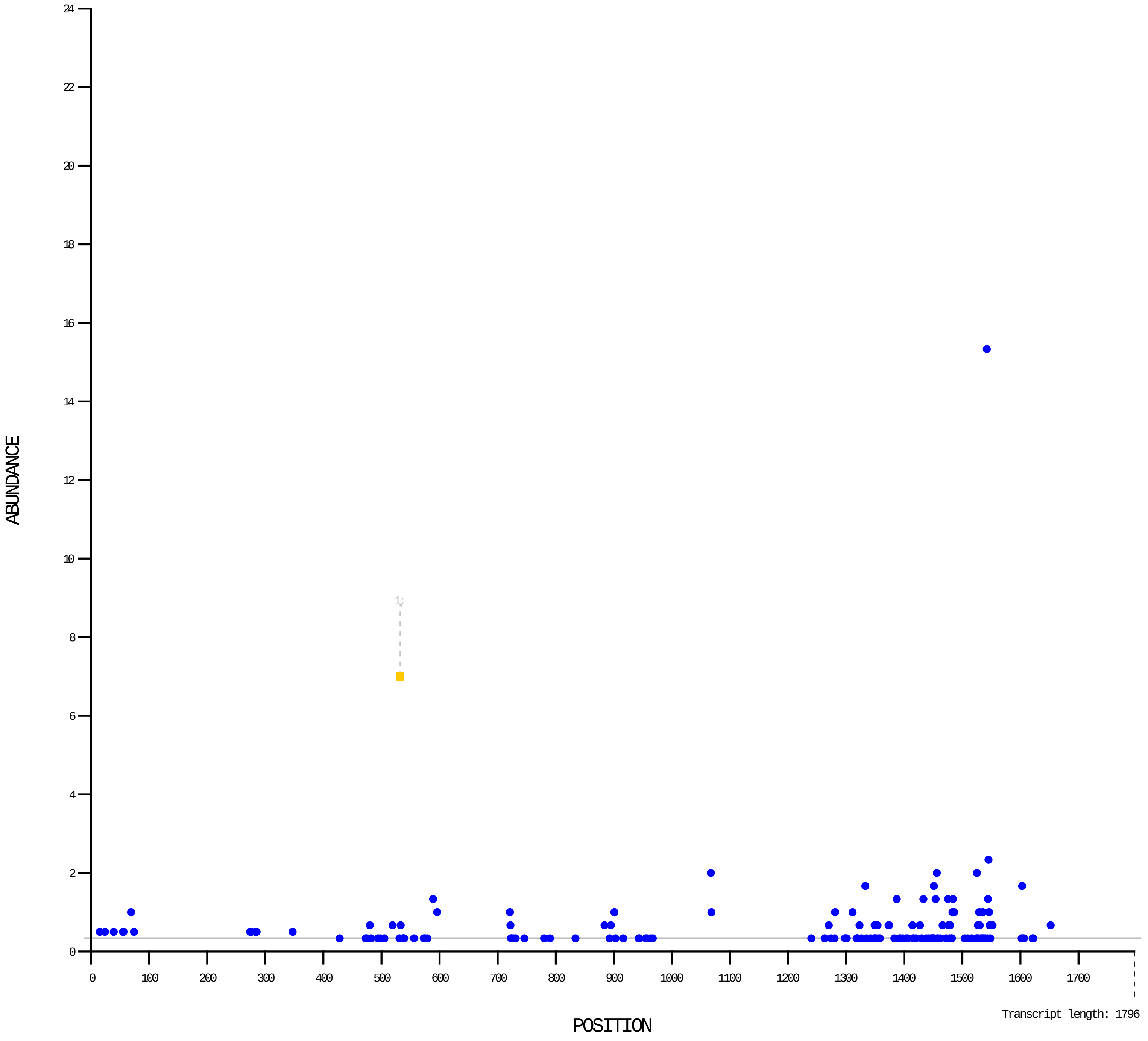


```
Category: 0  1  2  3  4
Degradome alignment:     Median:

2  #1  Position:532  Abundance: 7.00(deg) 1(sRNA)
   5'     TAGATAAAGATGAGAGAAAAA         3'  ID:
          o|||||||o|||||| |||||             Score: 2.0
   3' GGAAGTCTATTTTTACTCT-TTTTTATCTCTC 5'  p-value: 0.04
```

Cs4g05570.3 gene=Cs4g05570 CDS=271-1128

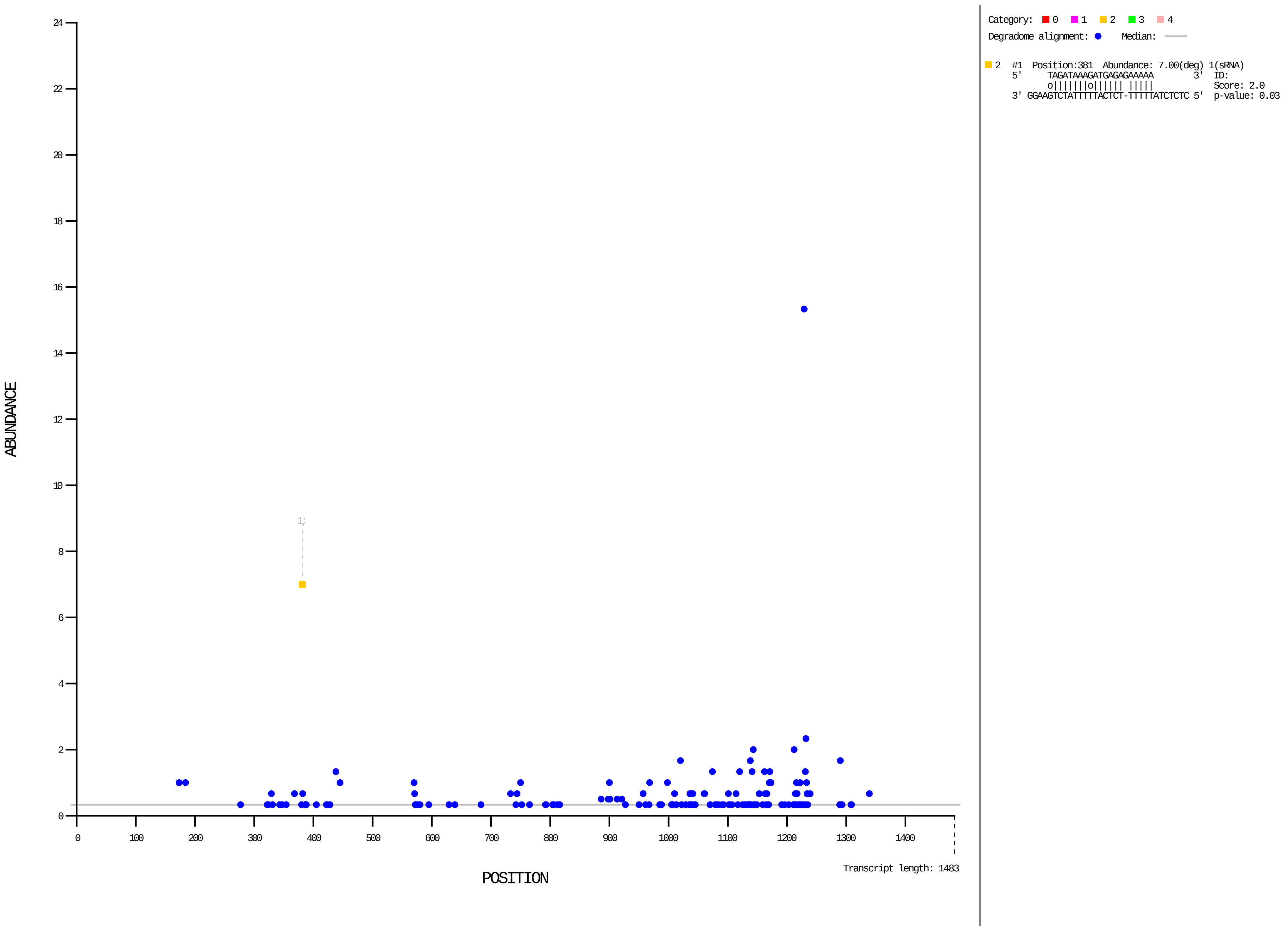

Cs4g05570.1 gene=Cs4g05570 CDS=263-1279

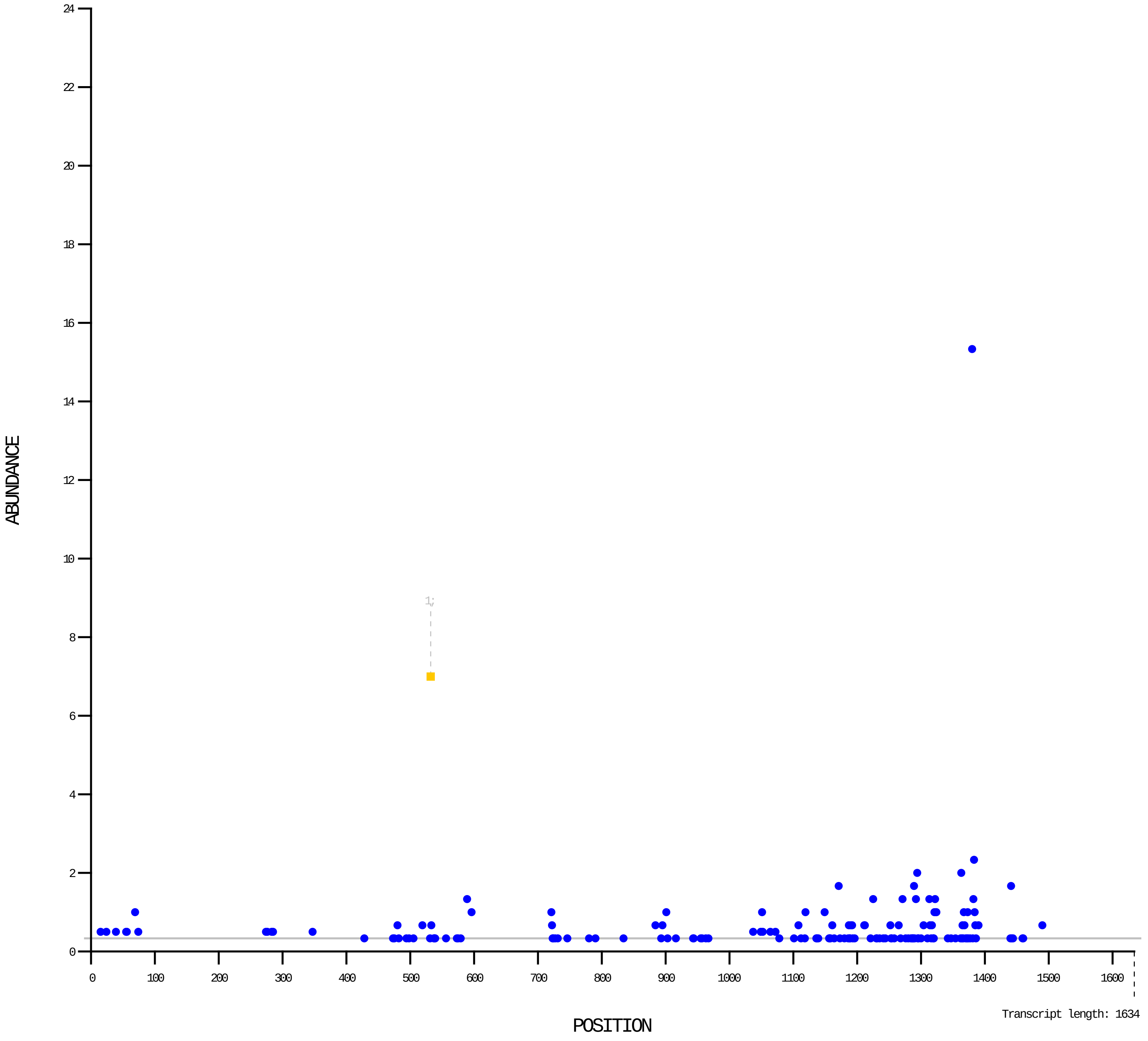

Category: 0 1 2 3 4
Degradome alignment: Median:

```
2  #1  Position:532  Abundance: 7.00(deg) 1(sRNA)
5'     TAGATAAAGATGAGAGAAAAA        3'  ID:
       o|||||||o|||||| |||||            Score: 2.0
3' GGAAGTCTATTTTTACTCT-TTTTTATCTCTC 5'  p-value: 0.02
```

Cs7g10830.2 gene=Cs7g10830 CDS=464-1906

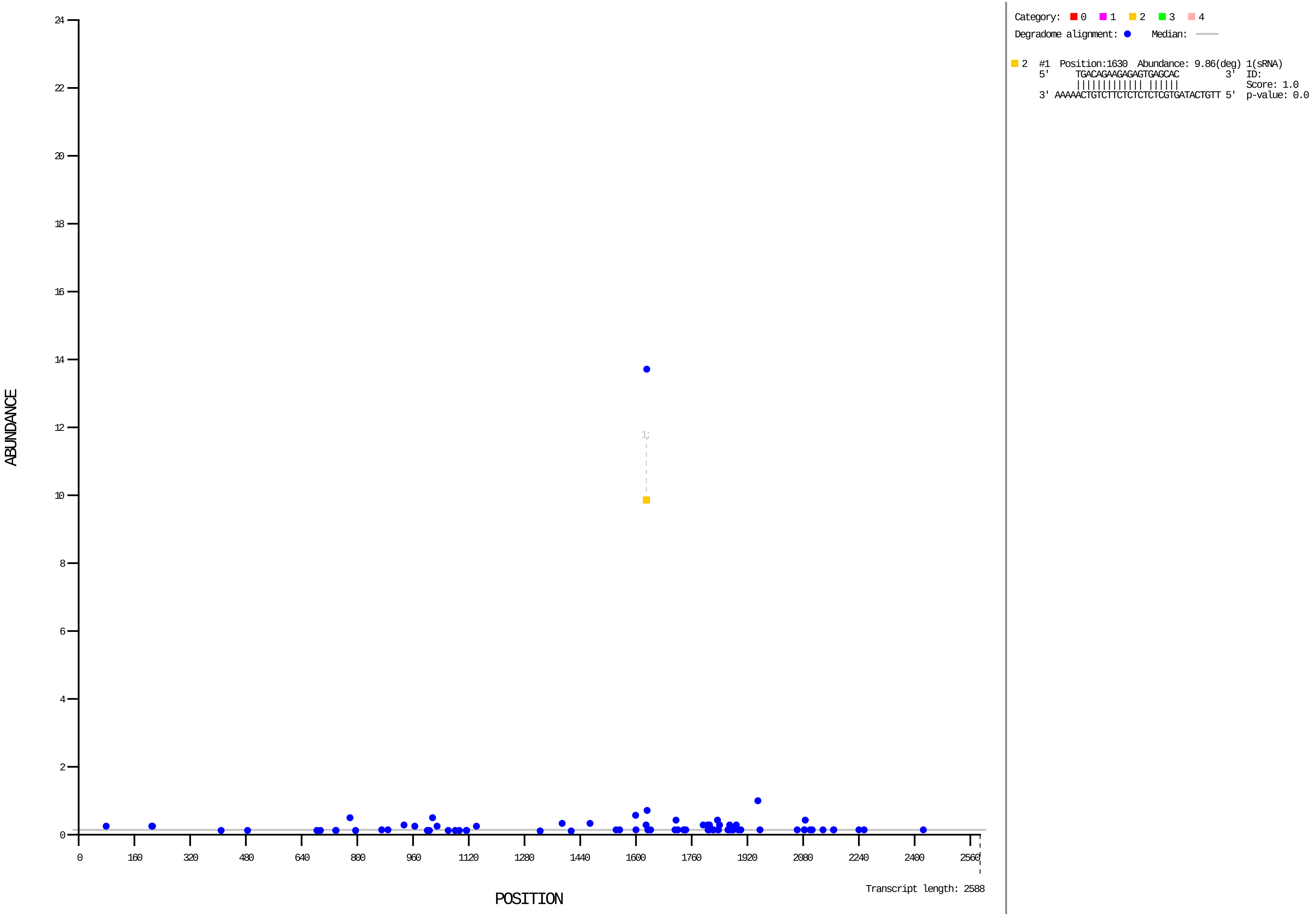

Cs7g10830.6 gene=Cs7g10830 CDS=195-1637

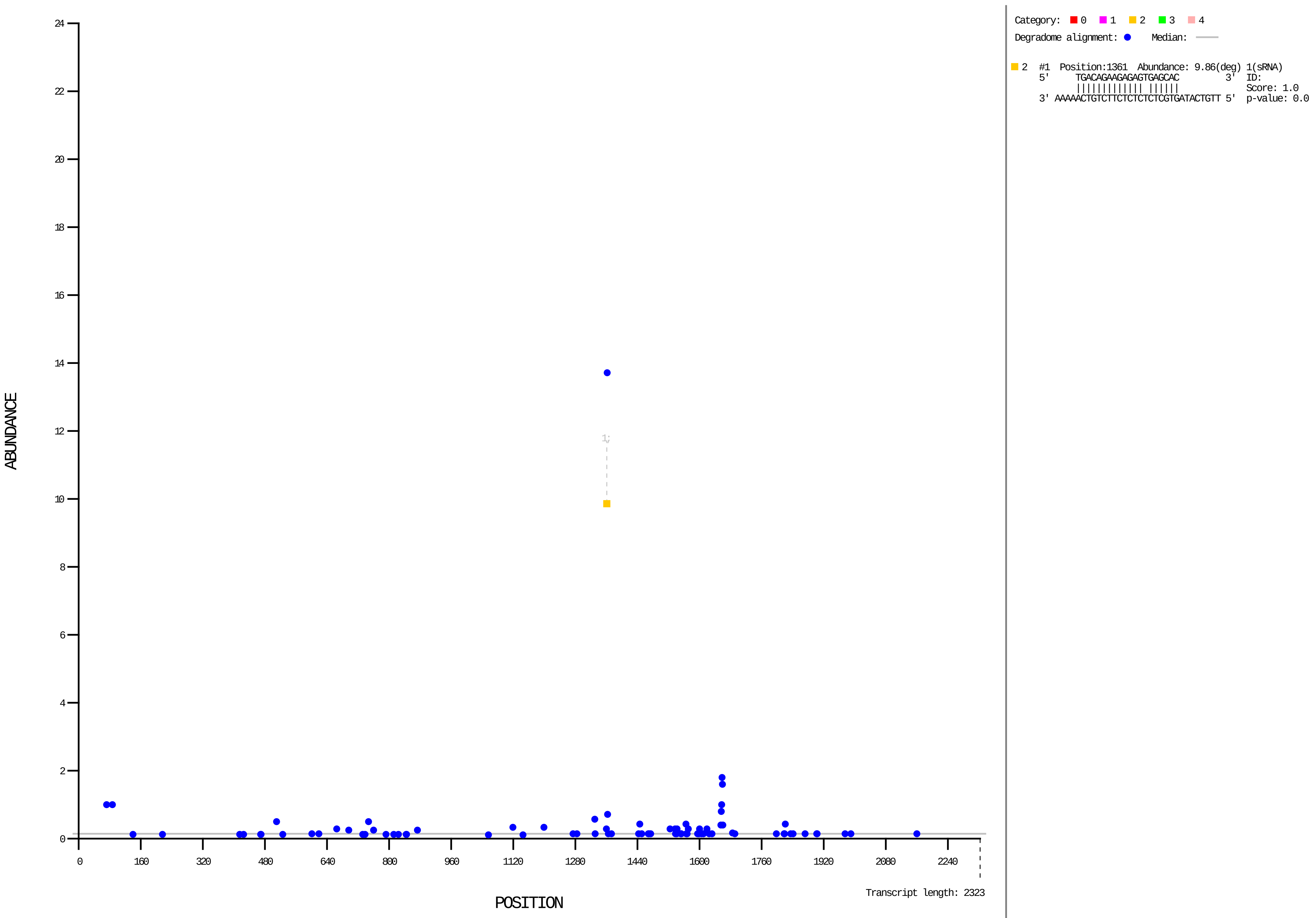

Cs7g10830.1 gene=Cs7g10830 CDS=109-1458

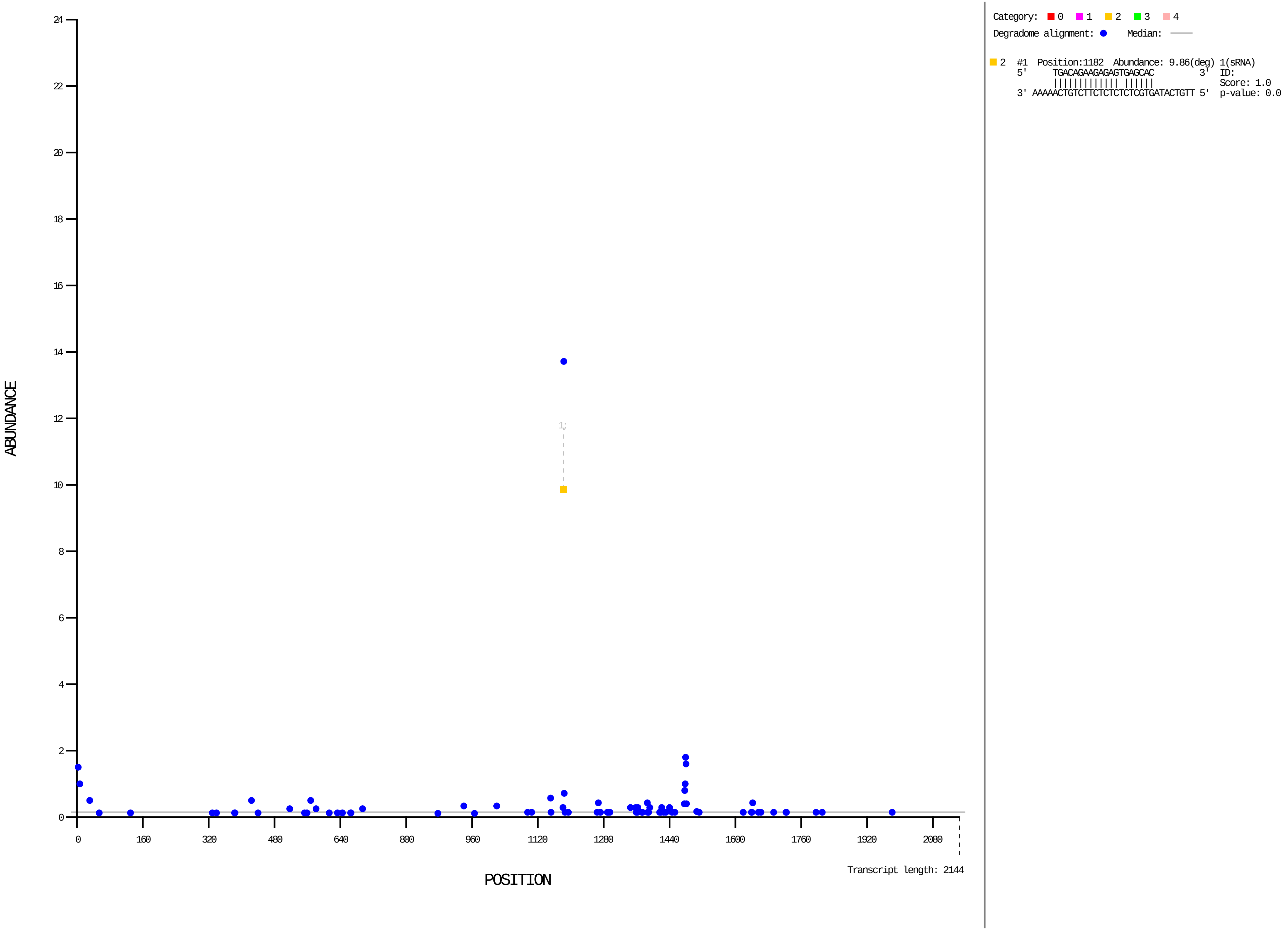

Cs7g10830.4 gene=Cs7g10830 CDS=187-1629

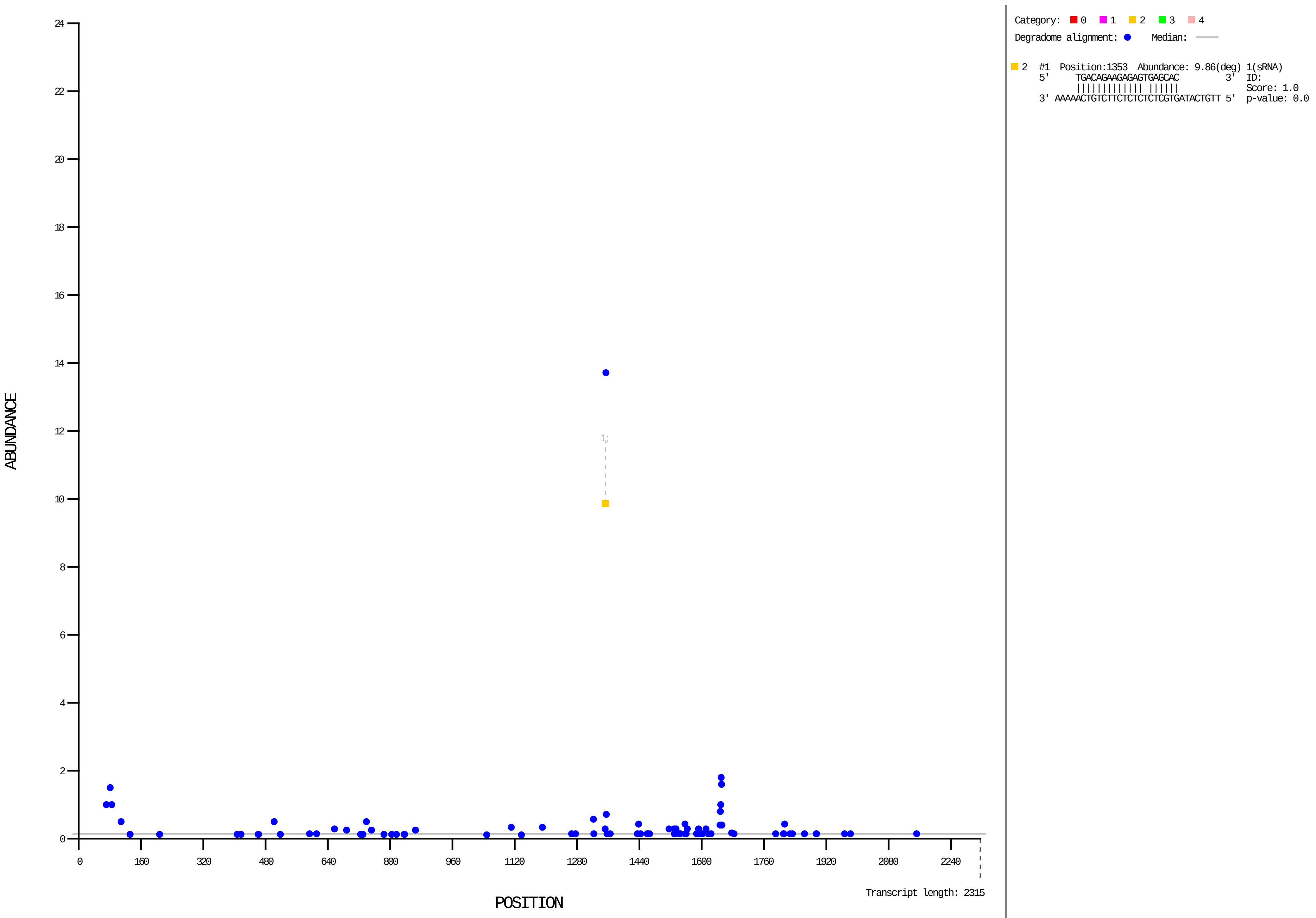

Cs7g10830.5 gene=Cs7g10830 CDS=464-1906

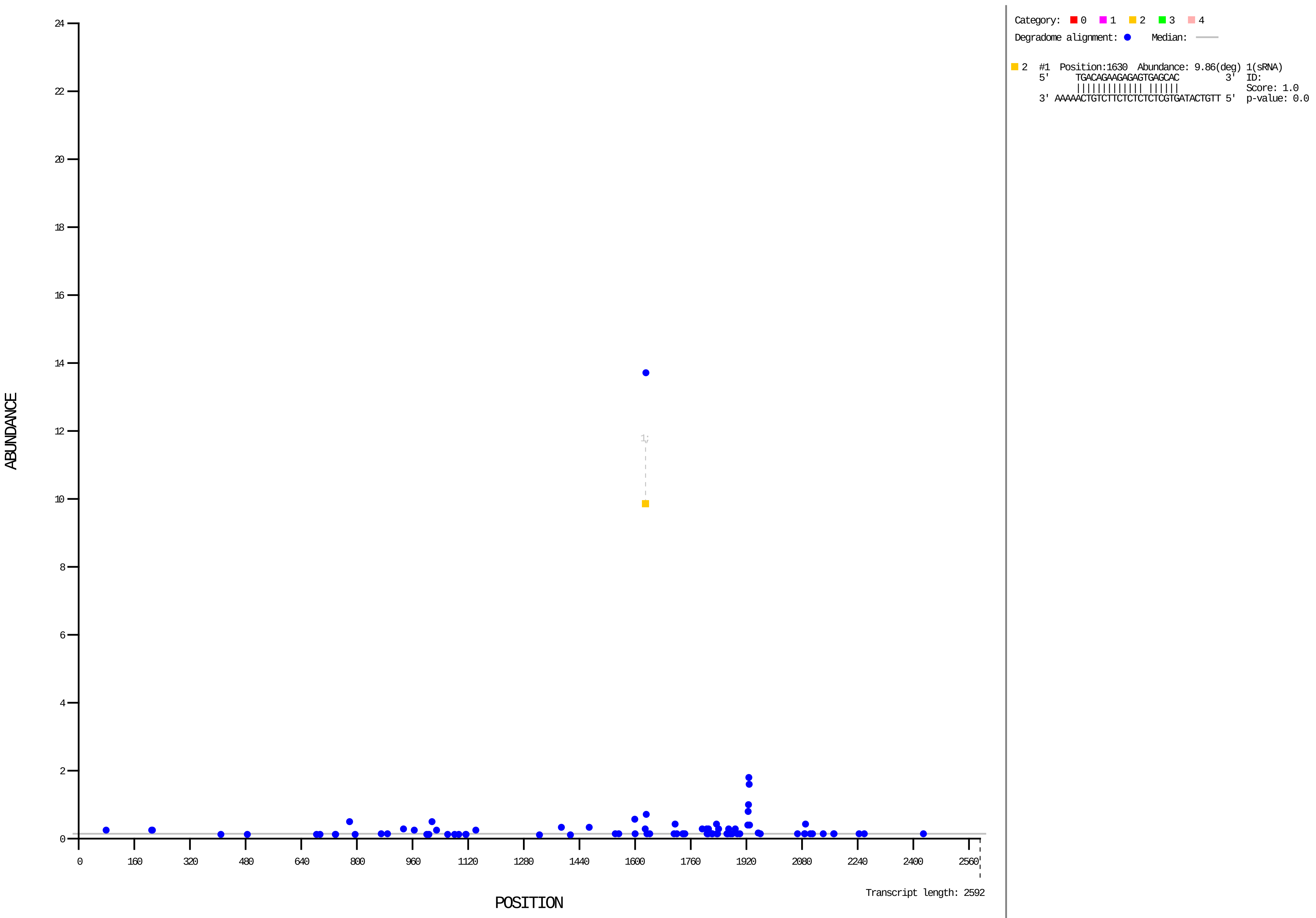

Cs7g10830.3 gene=Cs7g10830 CDS=100-1542

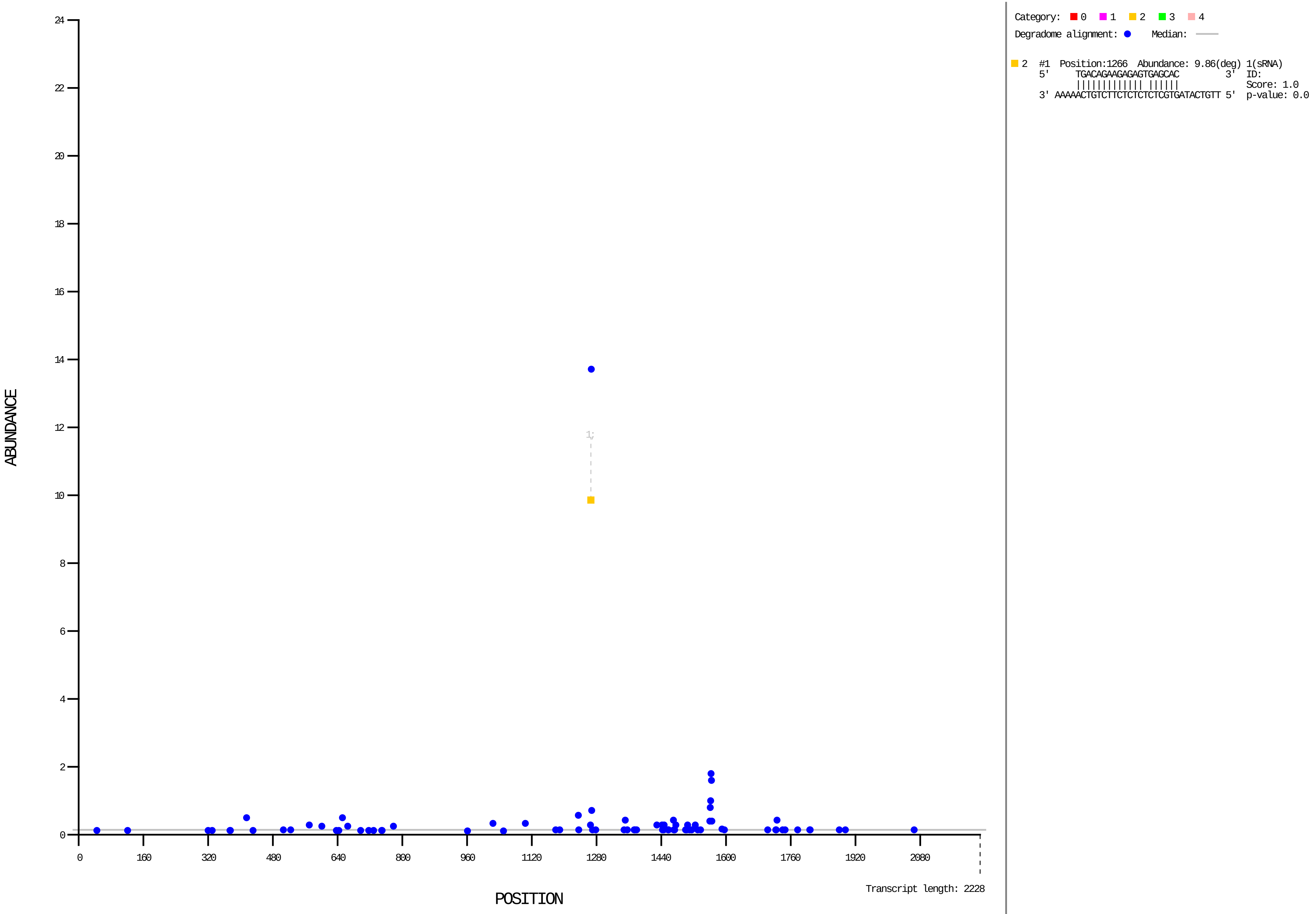

Cs7g10830.7 gene=Cs7g10830 CDS=464-1906

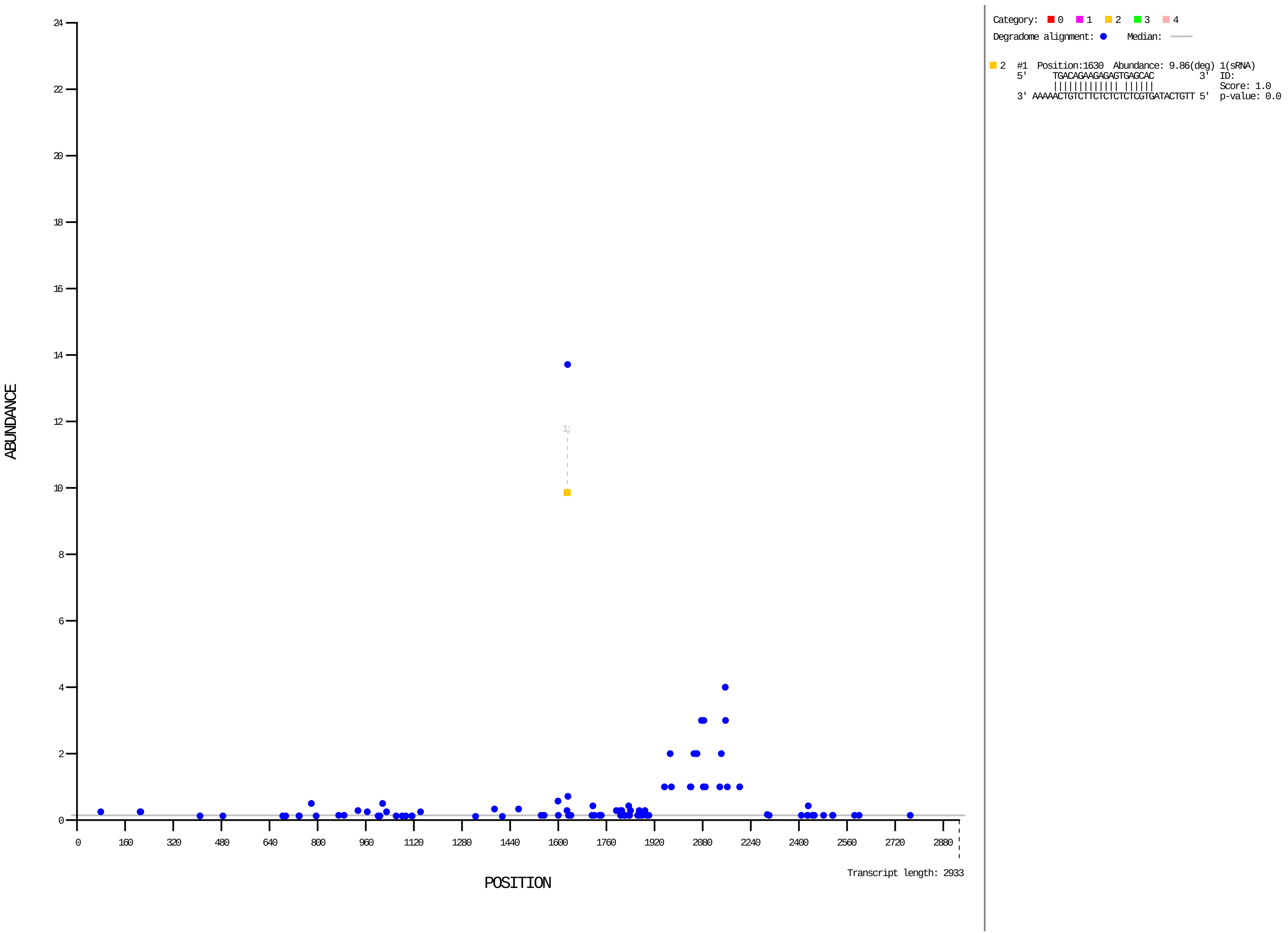

Cs2g18470.1 gene=Cs2g18470 CDS=284-4603

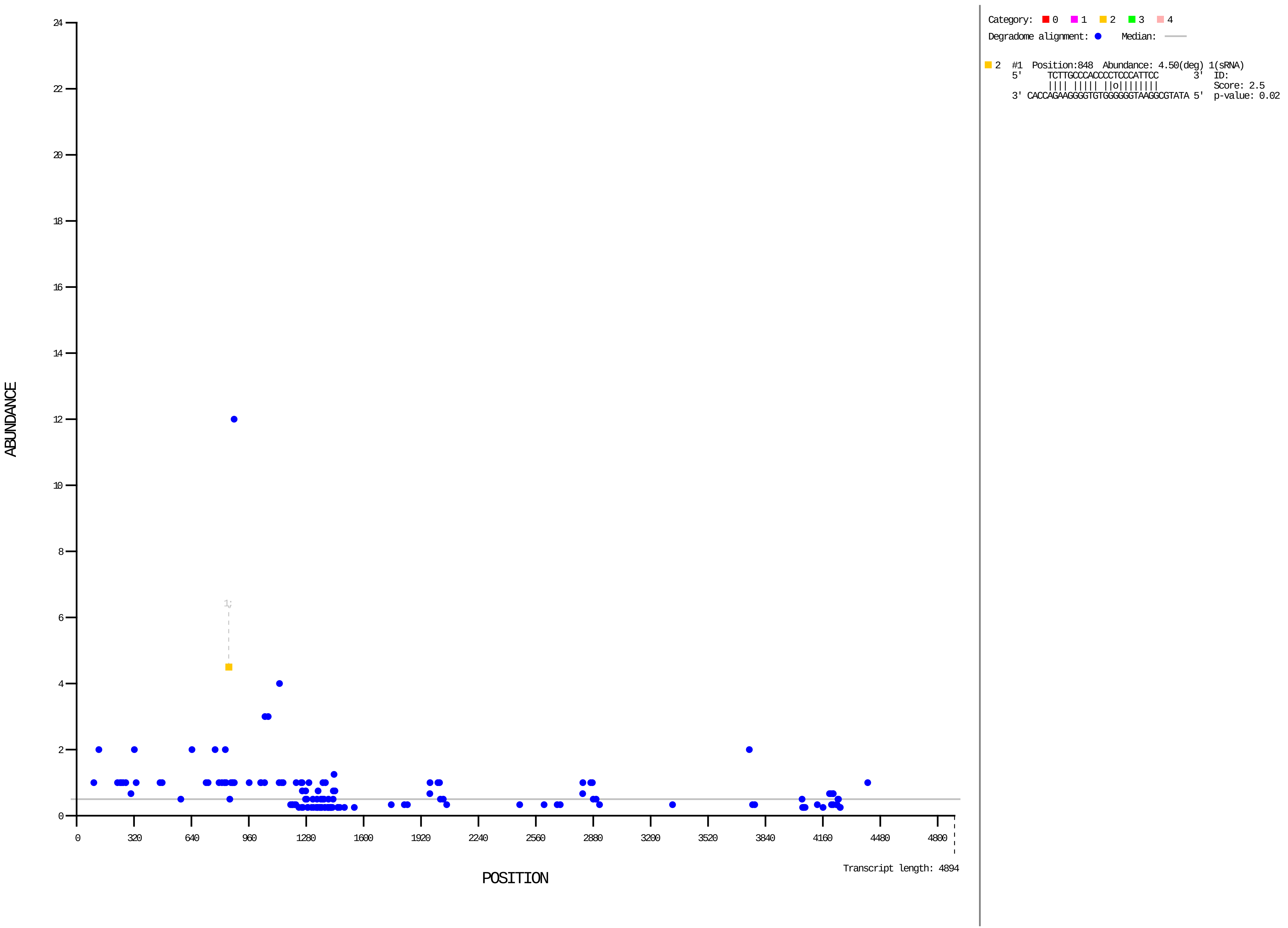

orange1.1t03059.8 gene=orange1.1t03059 CDS=131-2104

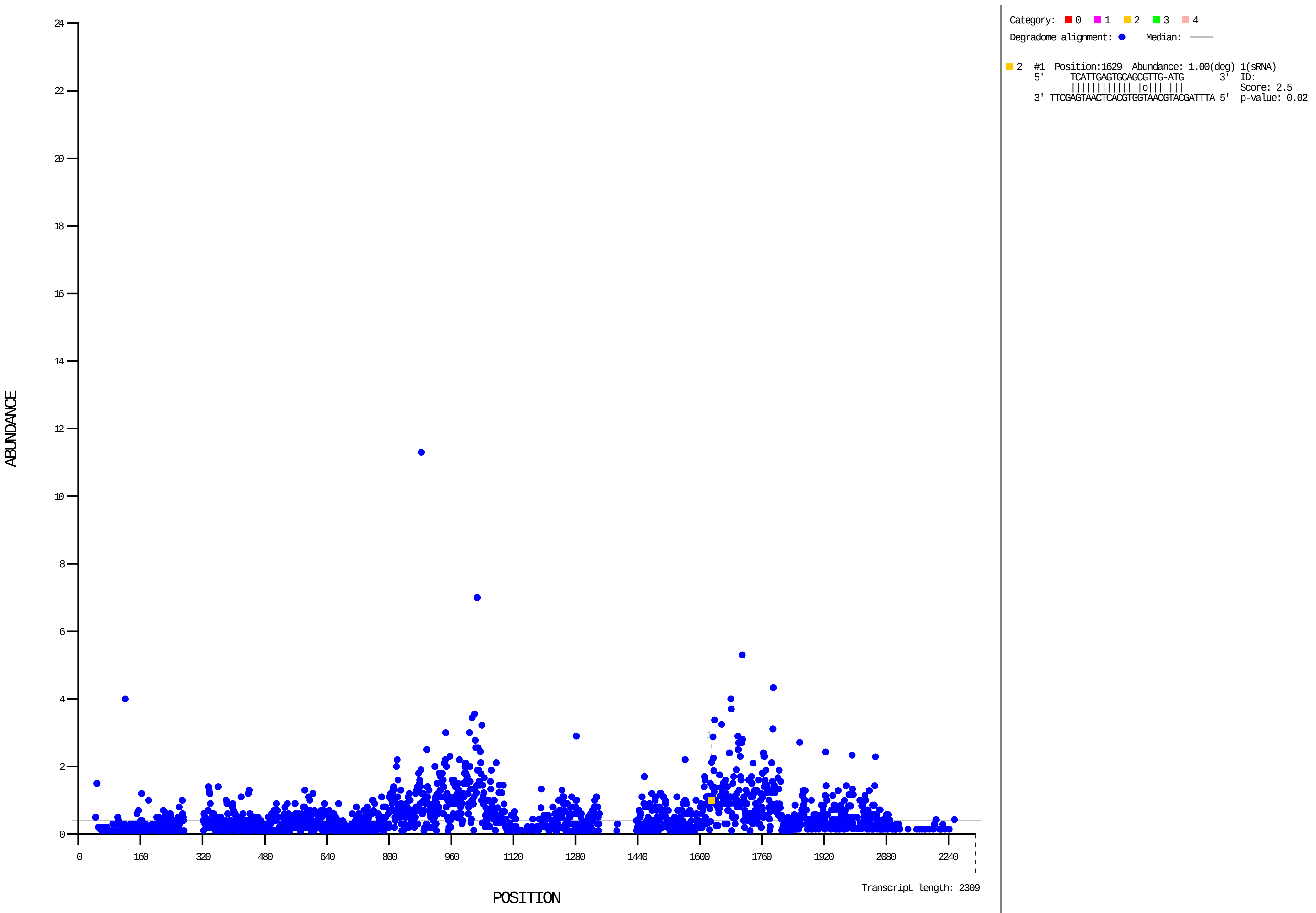

orange1.1t03059.5 gene=orange1.1t03059 CDS=515-2662

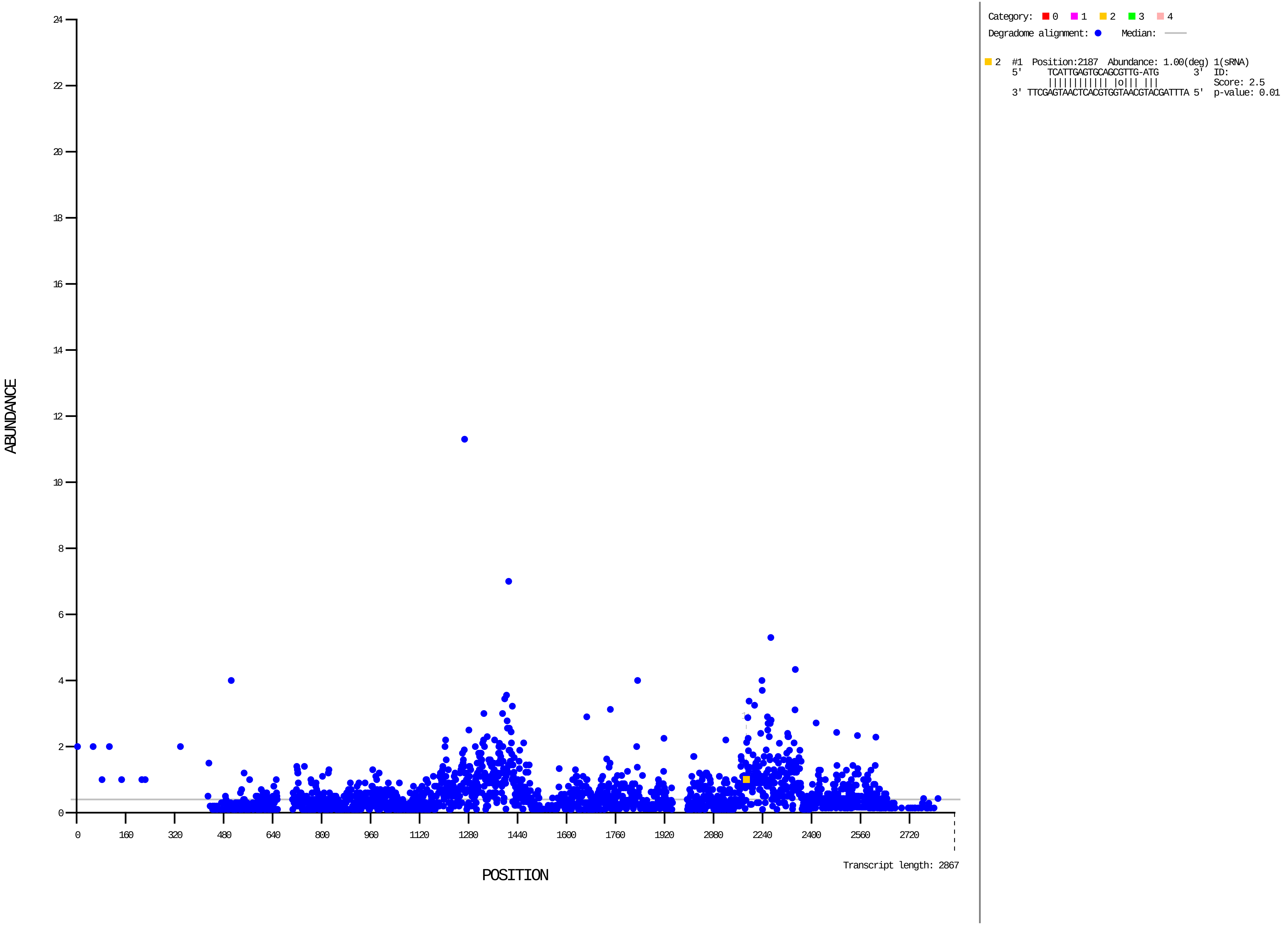

Cs5g01380.1 gene=Cs5g01380 CDS=451-1941

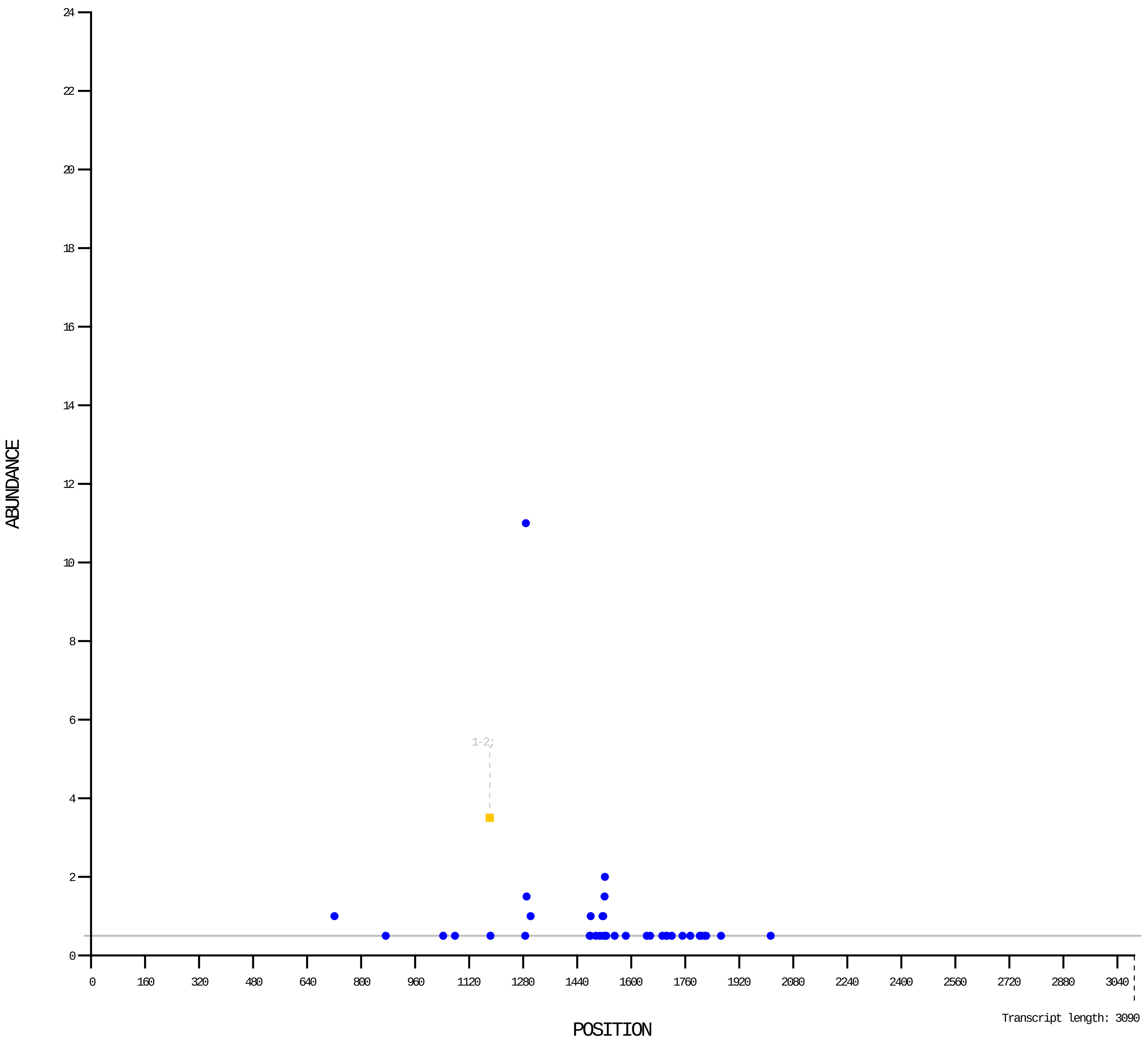

Category: 0 1 2 3 4
Degradome alignment: ● Median:

```
2  #1  Position:1181  Abundance: 3.50(deg) 1(sRNA)
   5'    TTCCAC-GGCTTTCTTGAACGT        3'  ID:
         |||||| ||||||||||||||             Score: 2.0
   3' ATTAAGGTGTCCGAAAGAACTTGCCCCTGCCG 5'  p-value: 0.02

2  #2  Position:1181  Abundance: 3.50(deg) 1(sRNA)
   5'    TTCCACA-GCTTTCTTGAACTG        3'  ID:
         ||||||| |||||||||||| |            Score: 2.0
   3' ATTAAGGTGTCCGAAAGAACTTGCCCCTGCCG 5'  p-value: 0.02
```

Cs5g01380.2 gene=Cs5g01380 CDS=95-1213

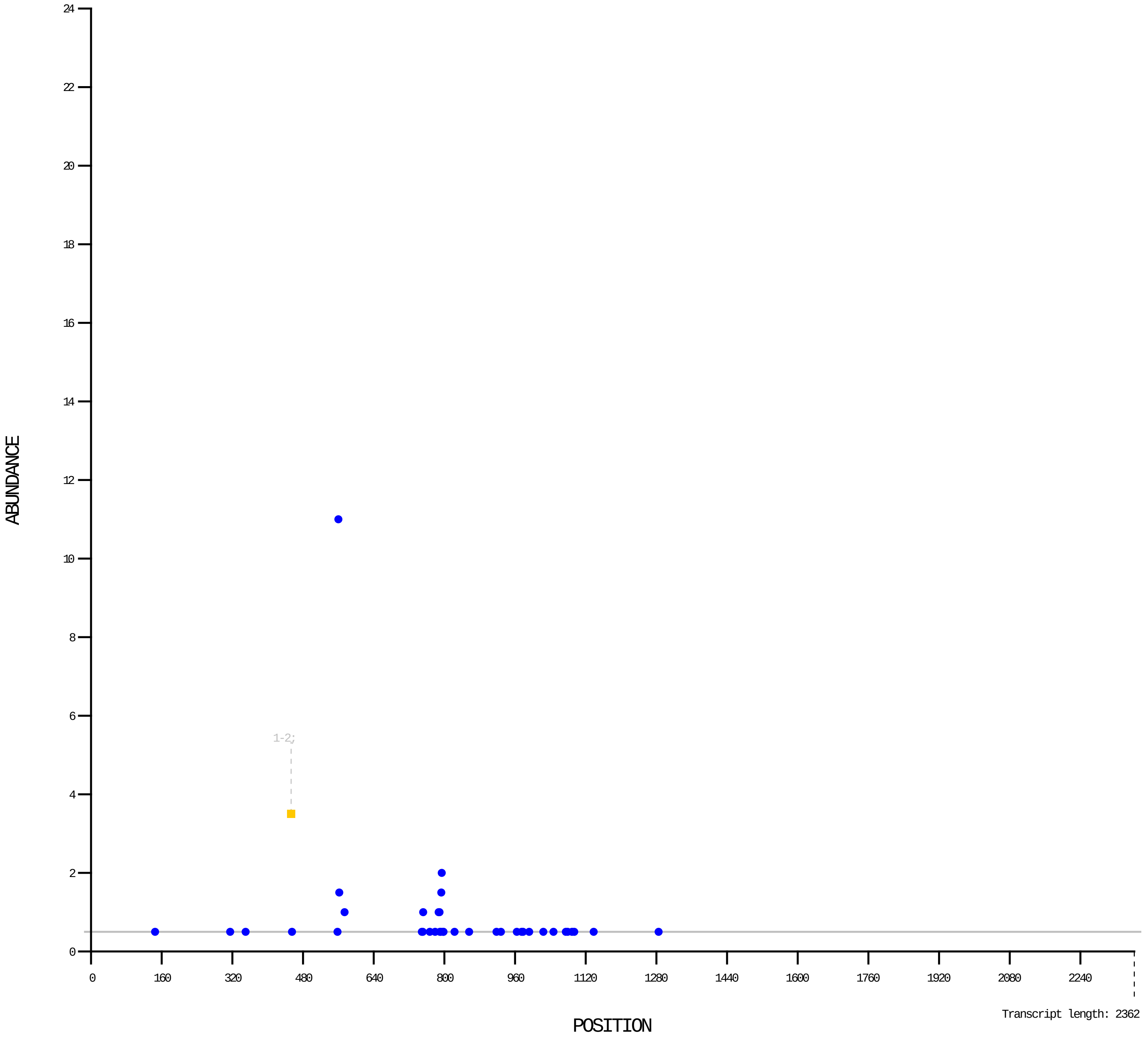

Category: 0 1 2 3 4
Degradome alignment: Median:

```
2  #1  Position:453  Abundance: 3.50(deg) 1(sRNA)
   5'    TTCCAC-GGCTTTCTTGAACGT        3'  ID:
         |||||| ||||||||||||||             Score: 2.0
   3' ATTAAGGTGTCCGAAAGAACTTGCCCCTGCCG 5'  p-value: 0.01

2  #2  Position:453  Abundance: 3.50(deg) 1(sRNA)
   5'    TTCCACA-GCTTTCTTGAACTG        3'  ID:
         ||||||| |||||||||||| |            Score: 2.0
   3' ATTAAGGTGTCCGAAAGAACTTGCCCCTGCCG 5'  p-value: 0.03
```

Cs5g19430.1 gene=Cs5g19430 CDS=1-3363

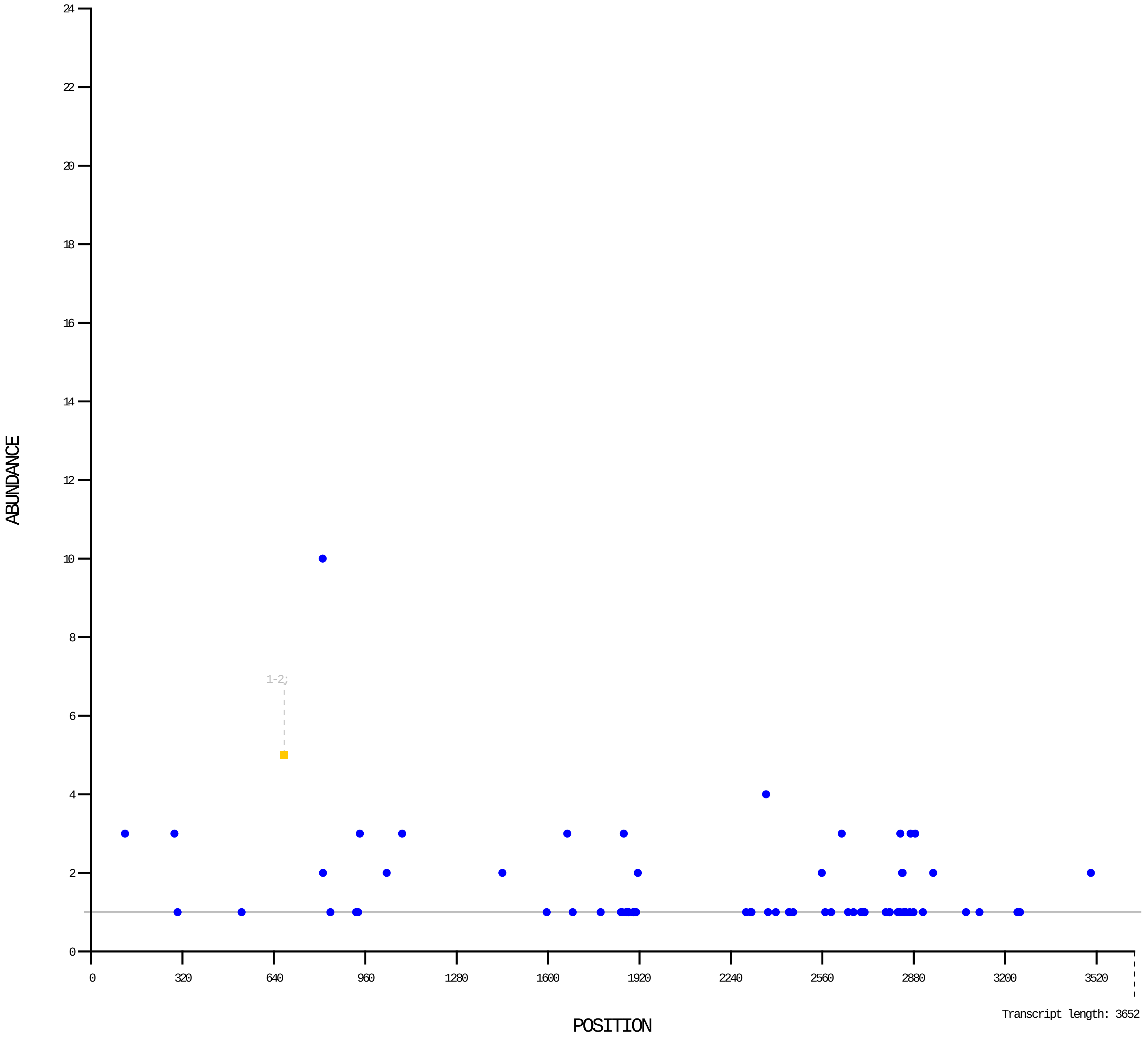

Category: 0 1 2 3 4
Degradome alignment: ● Median: —

```
2  #1  Position:676  Abundance: 5.00(deg) 1(sRNA)
   5'     TCTTCCCTATGCCTCCCATTCC       3'  ID:
          ||||||||||o||||||||  ||          Score: 1.5
   3' CAACAGAAGGGATATGGAGGGTATGGCGTATA 5'  p-value: 0.0

2  #2  Position:676  Abundance: 5.00(deg) 1(sRNA)
   5'     TCTTACCTATGCCACCCATTCC       3'  ID:
          |||| |||||o|| |||||  ||          Score: 3.5
   3' CAACAGAAGGGATATGGAGGGTATGGCGTATA 5'  p-value: 0.04
```

Cs7g11770.2 gene=Cs7g11770 CDS=381-1778

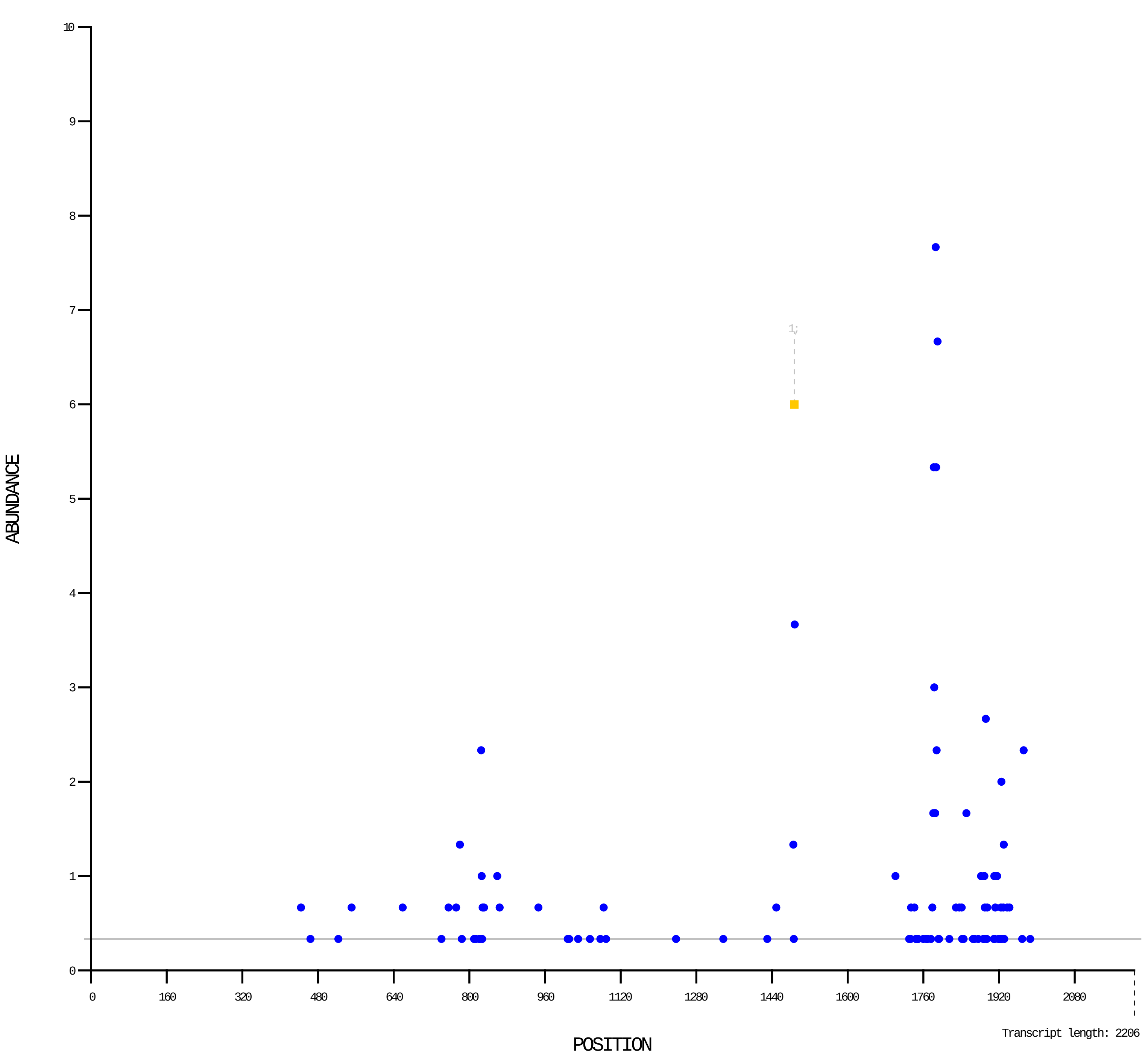

Cs7g11770.1 gene=Cs7g11770 CDS=381-1607

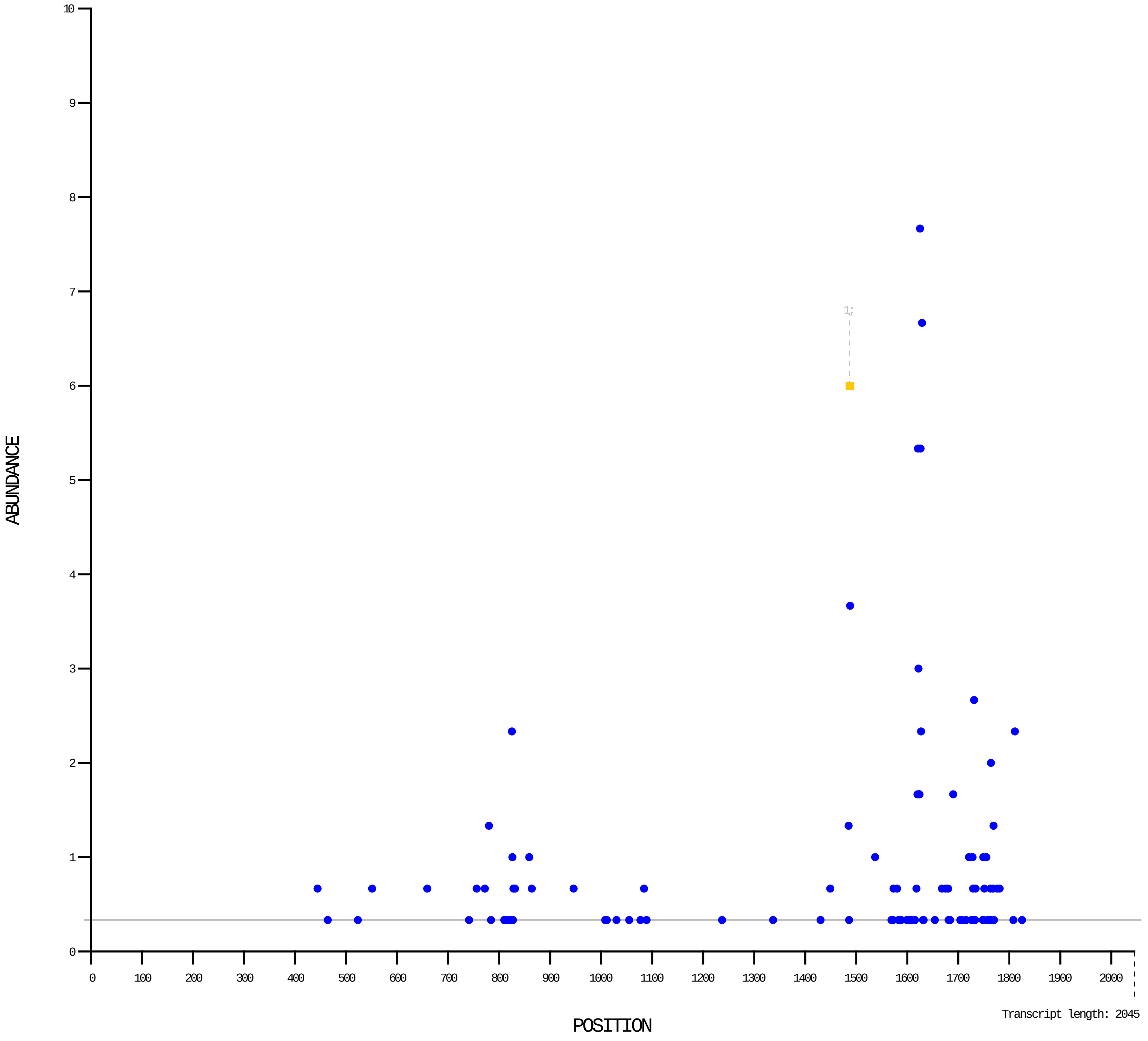

Category: 0 1 2 3 4
Degradome alignment: Median:

2 #1 Position:1487 Abundance: 6.00(deg) 1(sRNA)

```
5'     TGACAGAAGAGAGTGAGCAC        3'  ID:
       |||||||||||| ||||||             Score: 1.0
3' CTCGACTGTCTTCTCTCTCTCGTGGTGACCTC 5'  p-value: 0.0
```

Cs7g11770.3 gene=Cs7g11770 CDS=381-1901

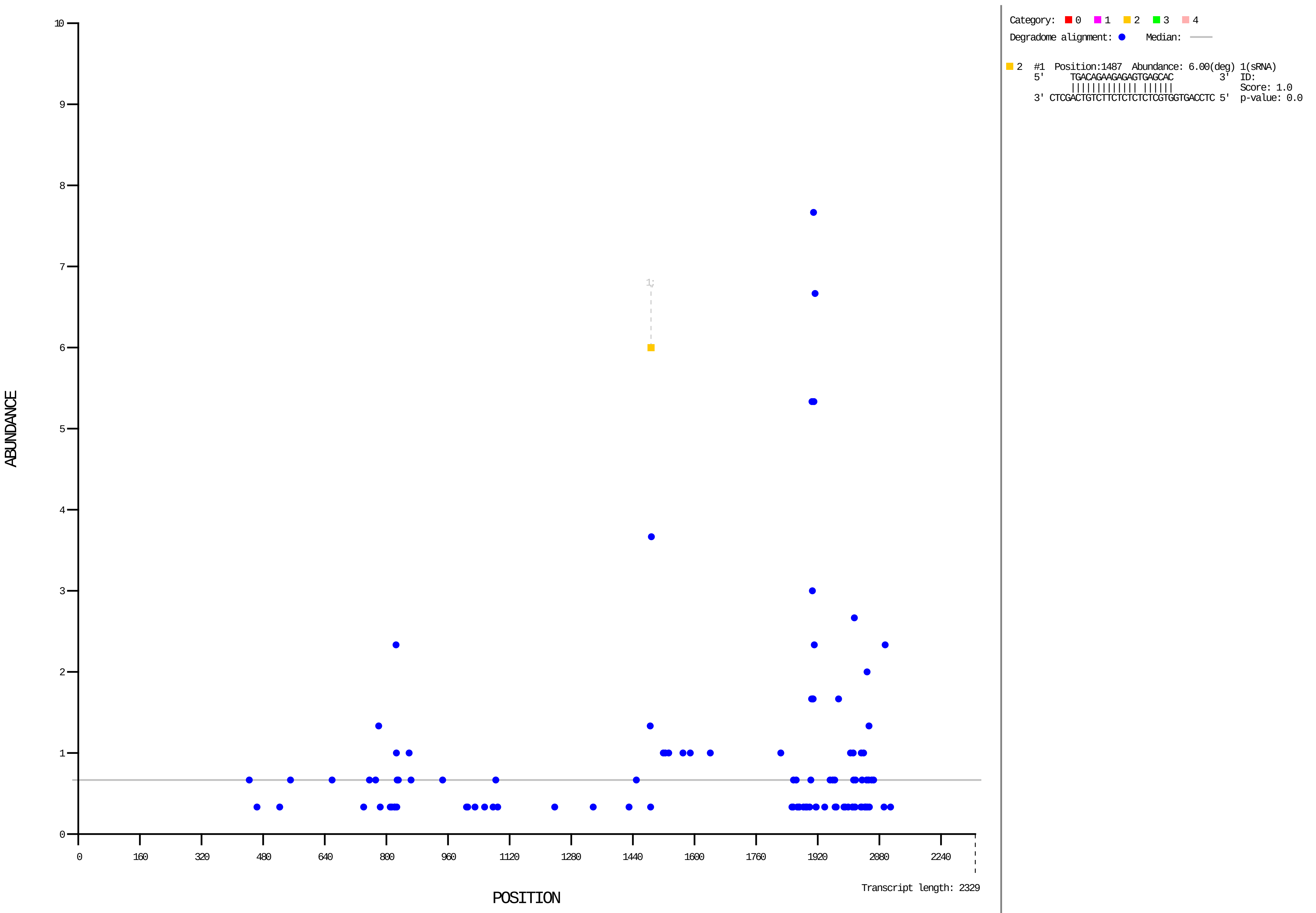

Cs1g08080.2 gene=Cs1g08080 CDS=116-3190

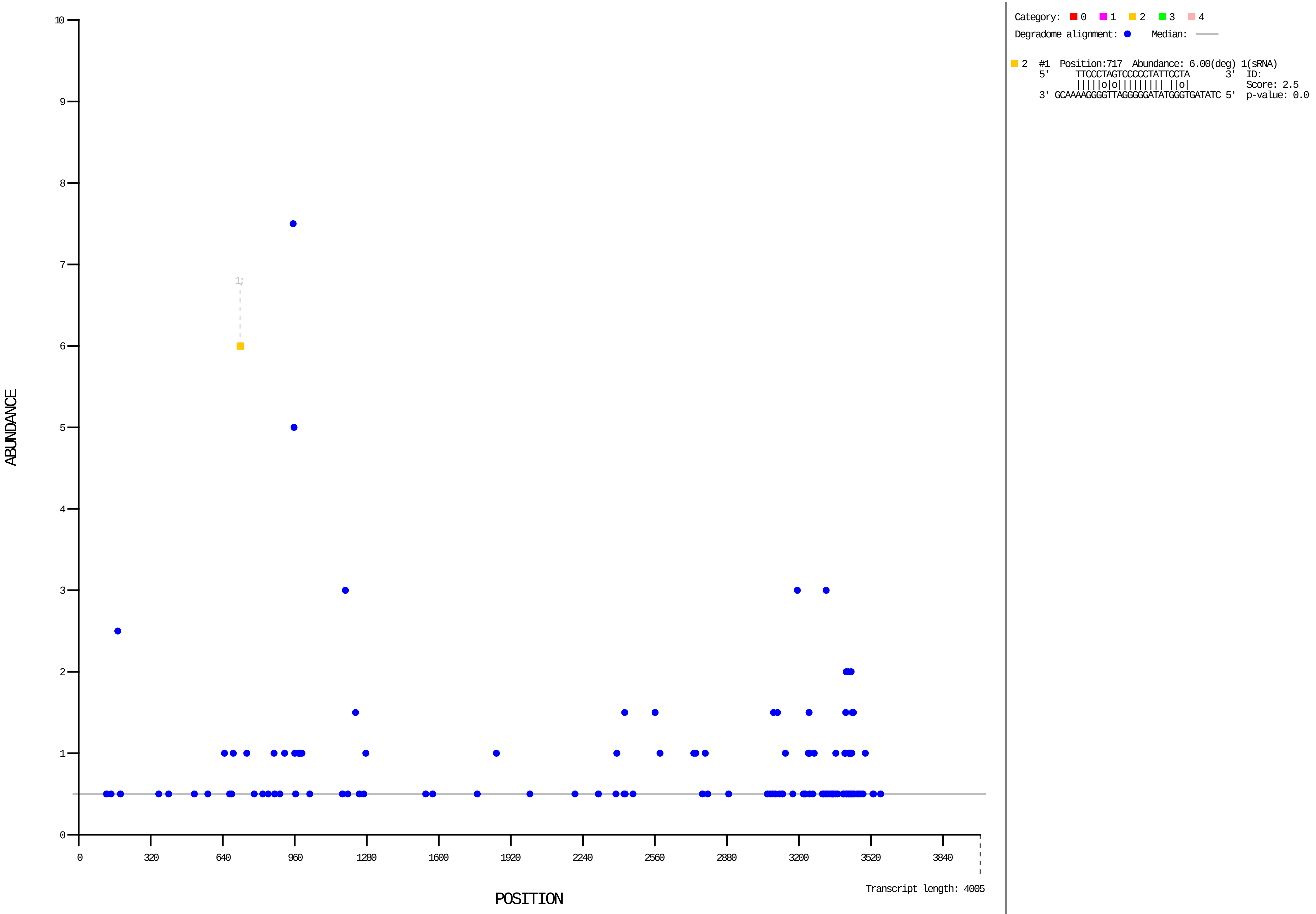

Cs1g08080.1 gene=Cs1g08080 CDS=116-3190

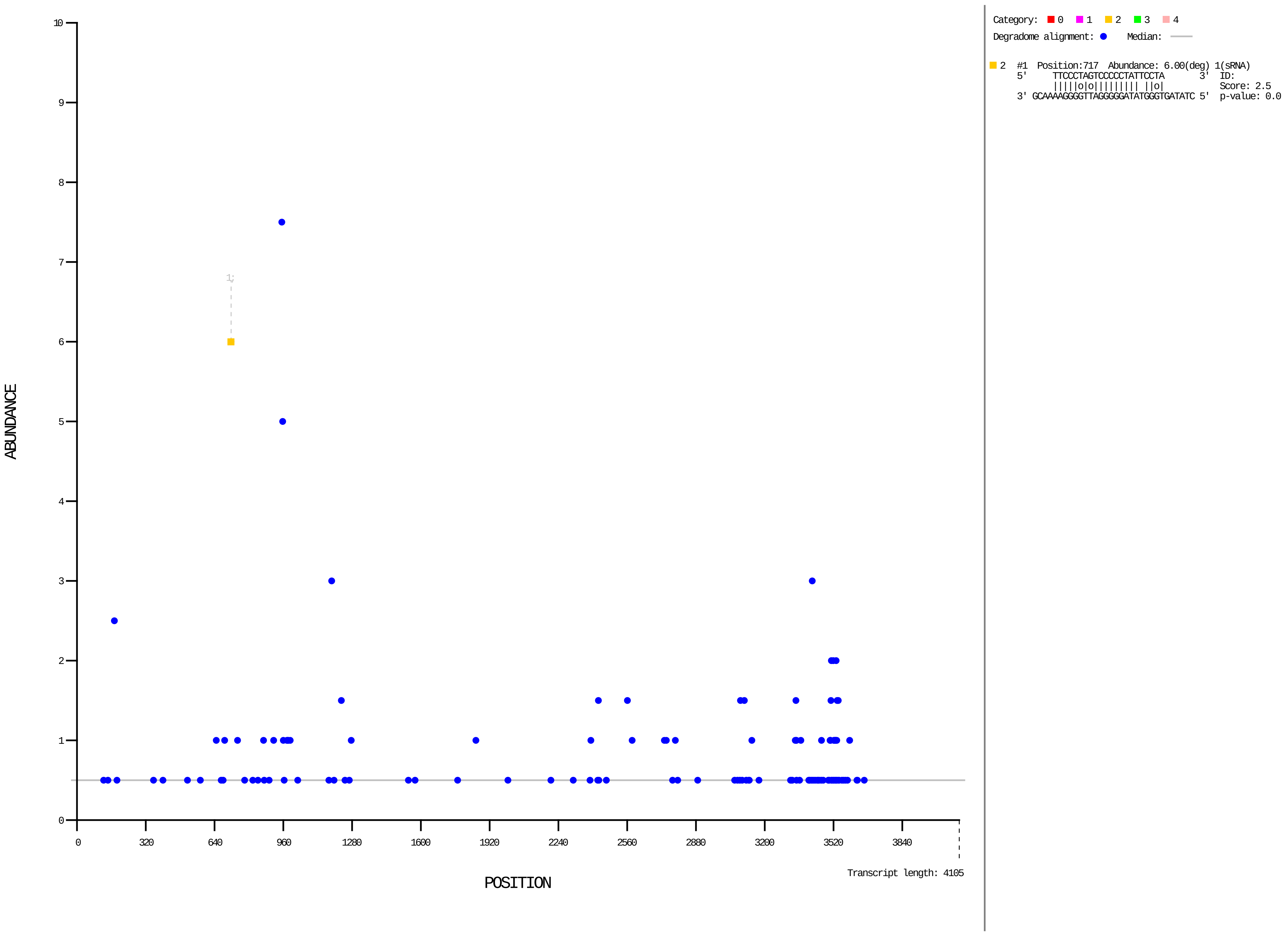

Cs1g19500.8 gene=Cs1g19500 CDS=173-1768

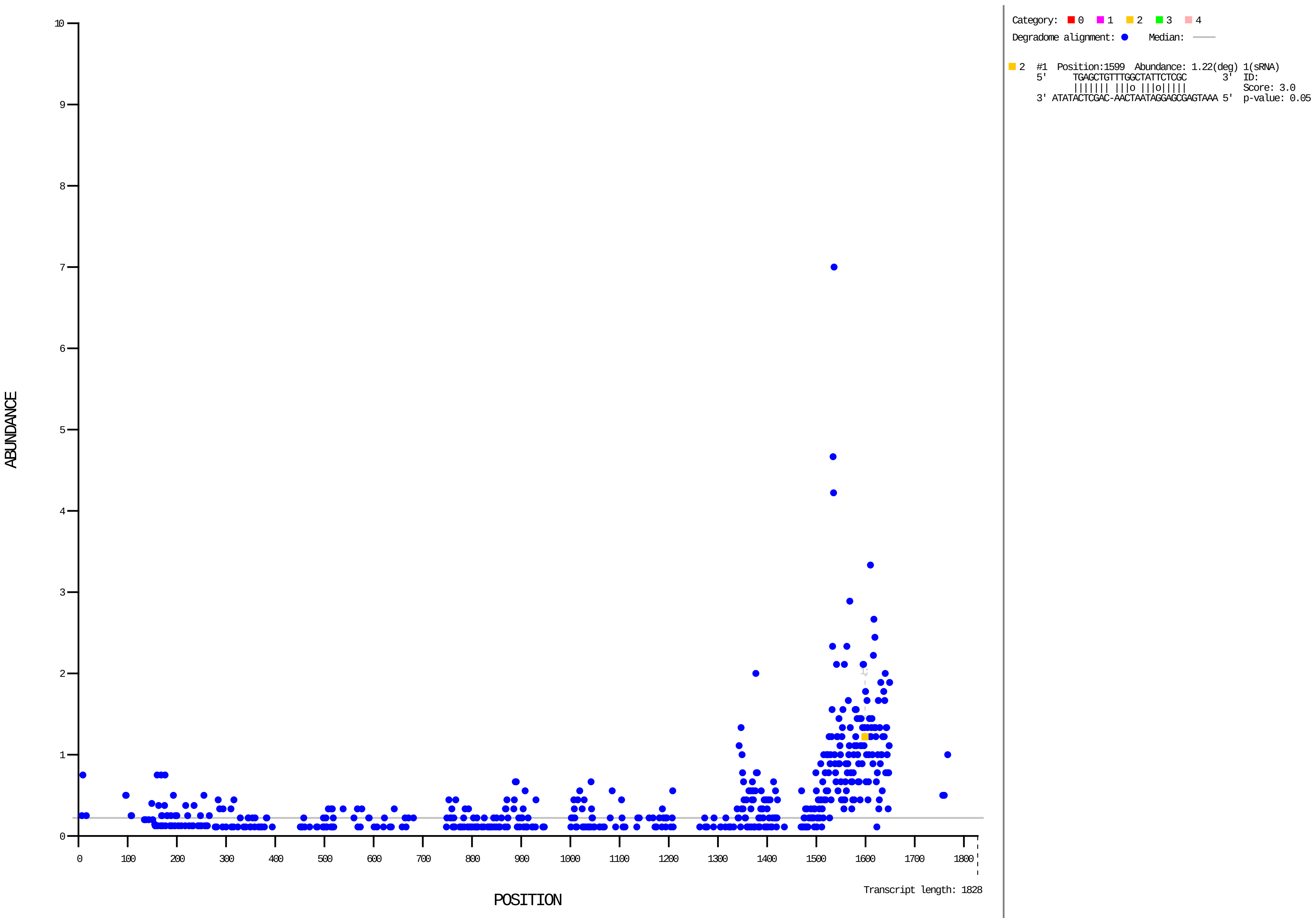

Cs1g09350.1 gene=Cs1g09350 CDS=1-3087

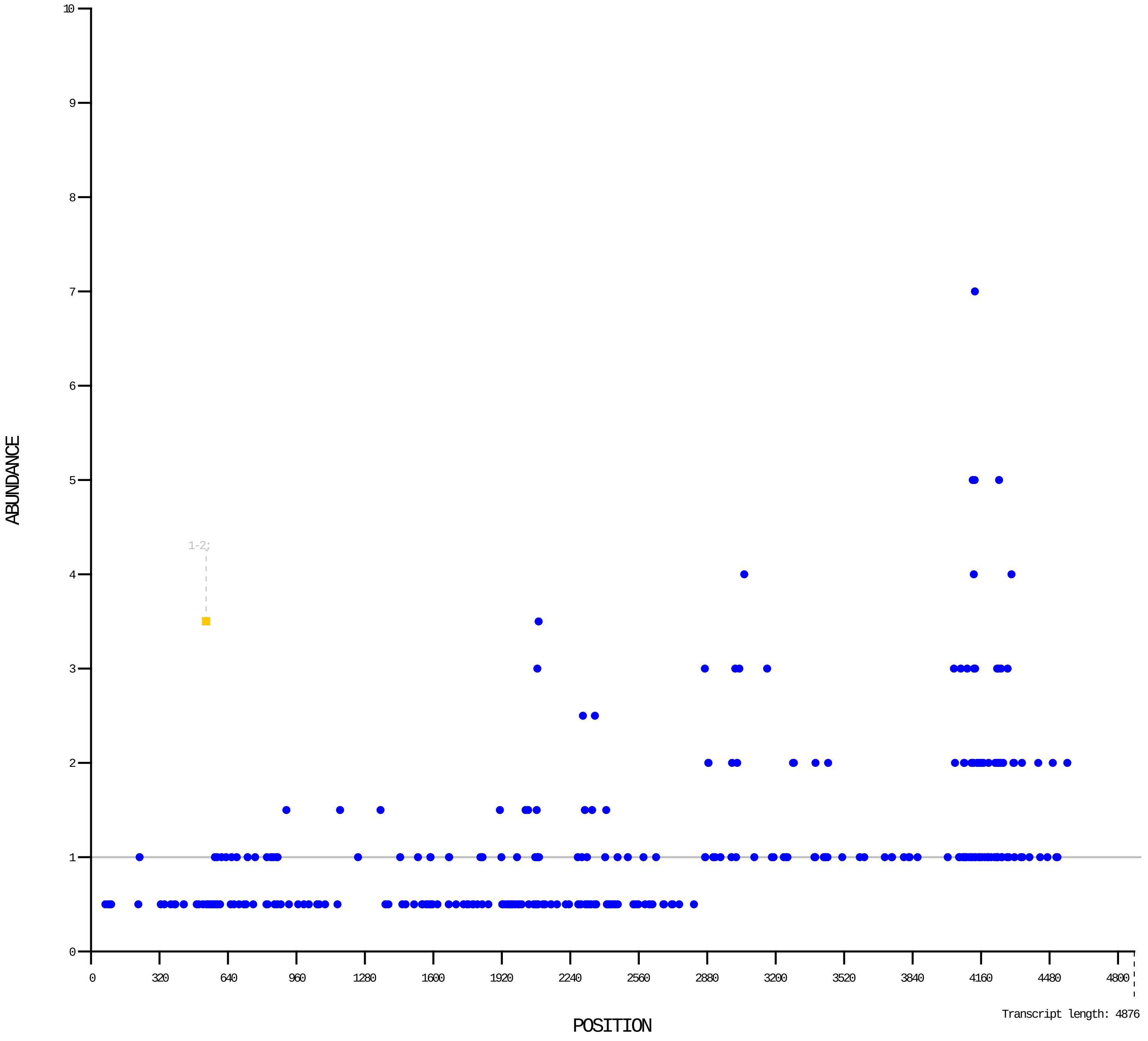

Category: 0 1 2 3 4
Degradome alignment: Median:

2 #1 Position:538 Abundance: 3.50(deg) 1(sRNA)
5' TCTTCCCTATGCCTCCCATTCC 3' ID:
|||| |||||o|| |||||o|| Score: 3.0
3' CAACAGAAAGGATATGGCGGGTAGGGATACTC 5' p-value: 0.05

2 #2 Position:538 Abundance: 3.50(deg) 1(sRNA)
5' TCTTACCTATGCCACCCATTCC 3' ID:
|||| |||||o|| |||||o|| Score: 3.0
3' CAACAGAAAGGATATGGCGGGTAGGGATACTC 5' p-value: 0.03

Cs1g19500.9 gene=Cs1g19500 CDS=86-1681

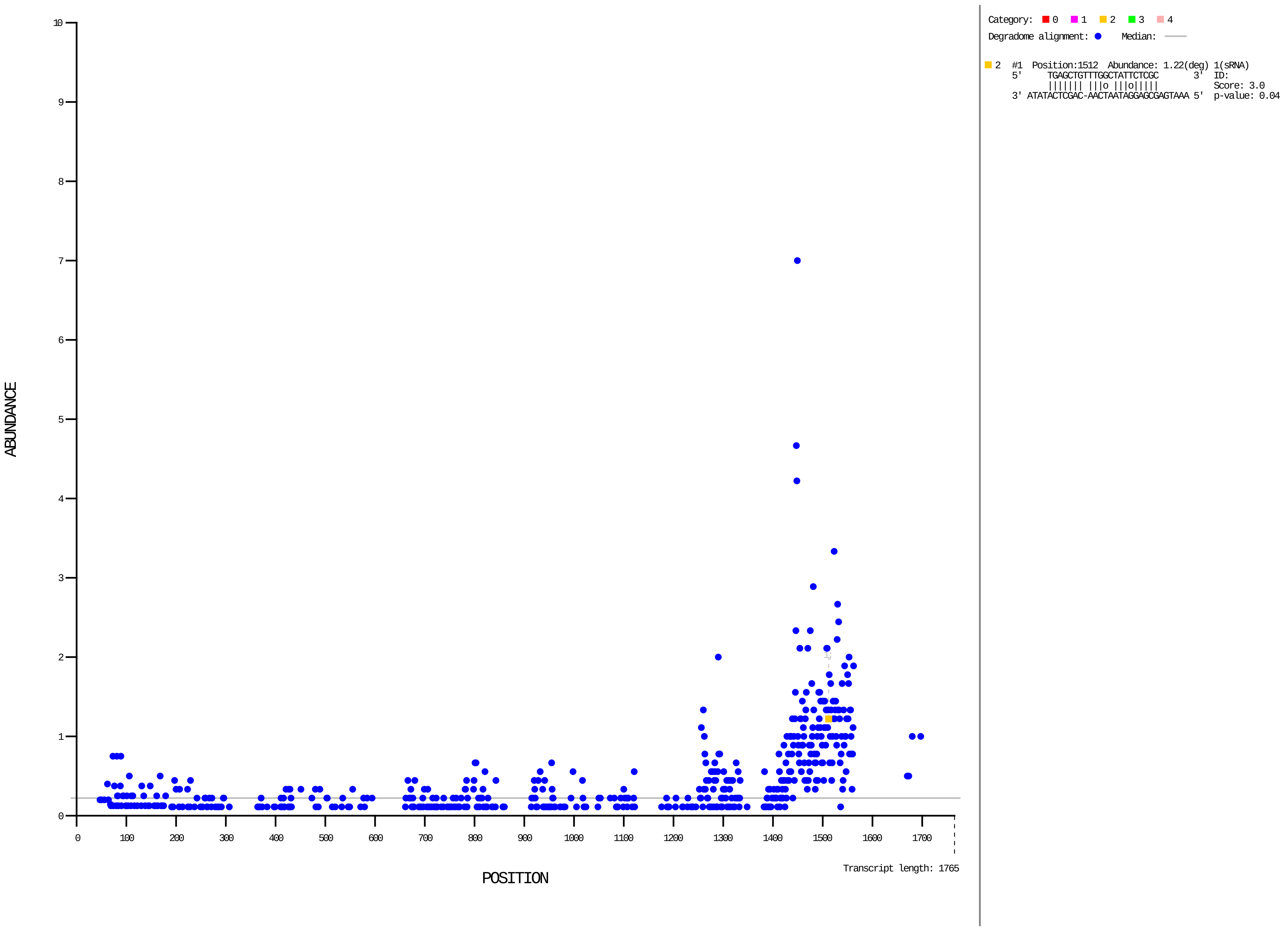

Cs1g19500.6 gene=Cs1g19500 CDS=86-2059

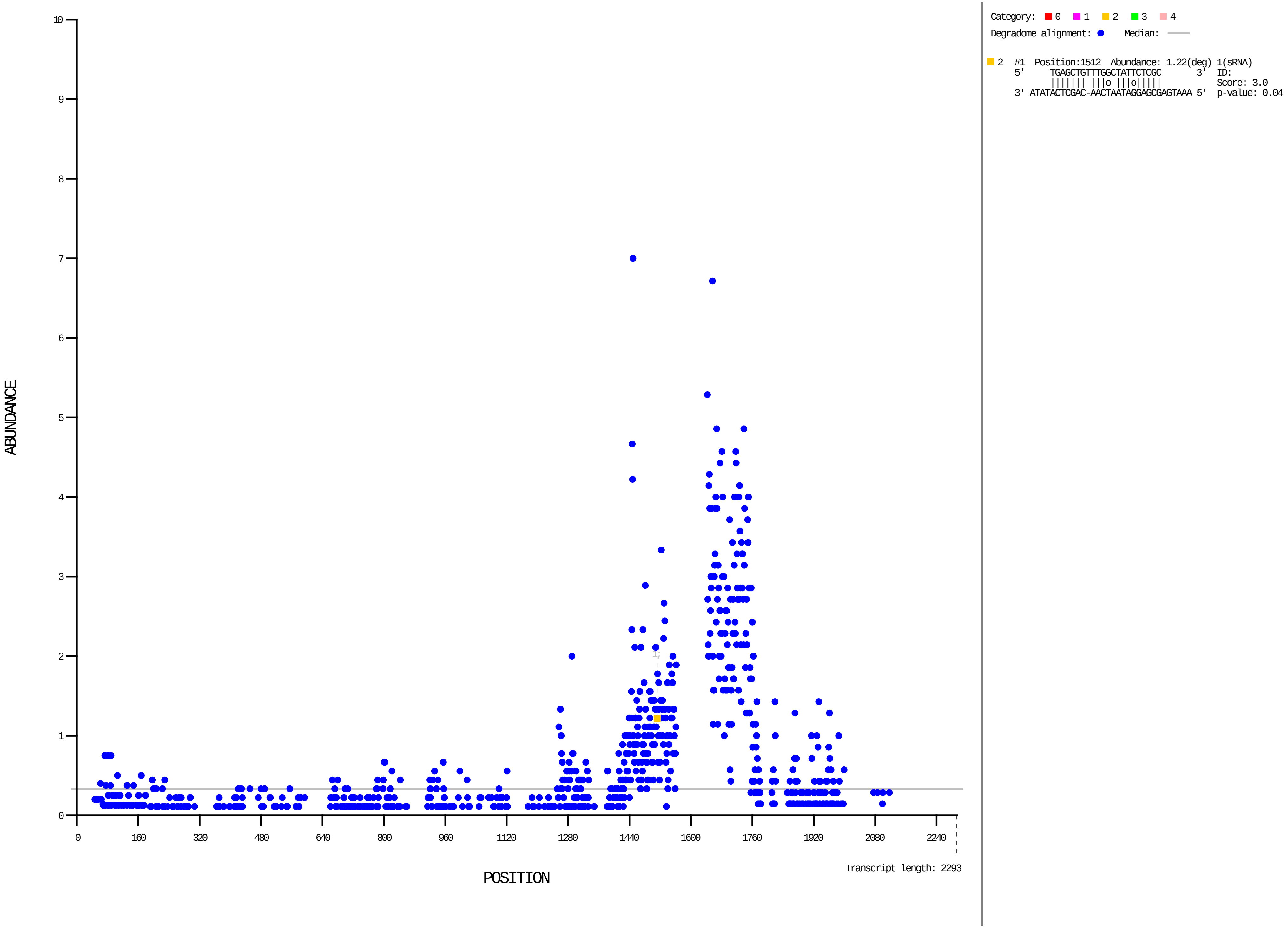

Cs1g15550.1 gene=Cs1g15550 CDS=101-2779

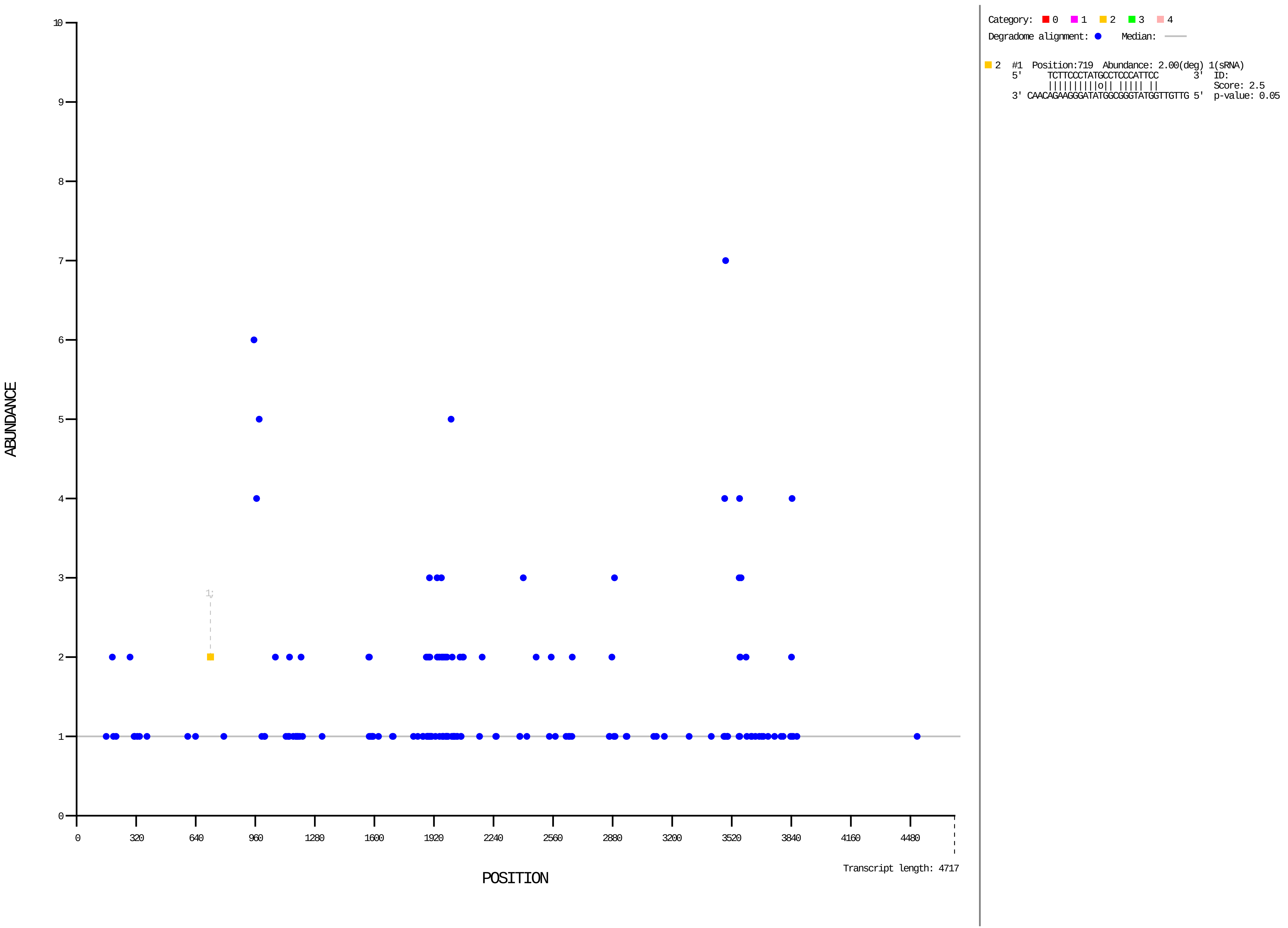

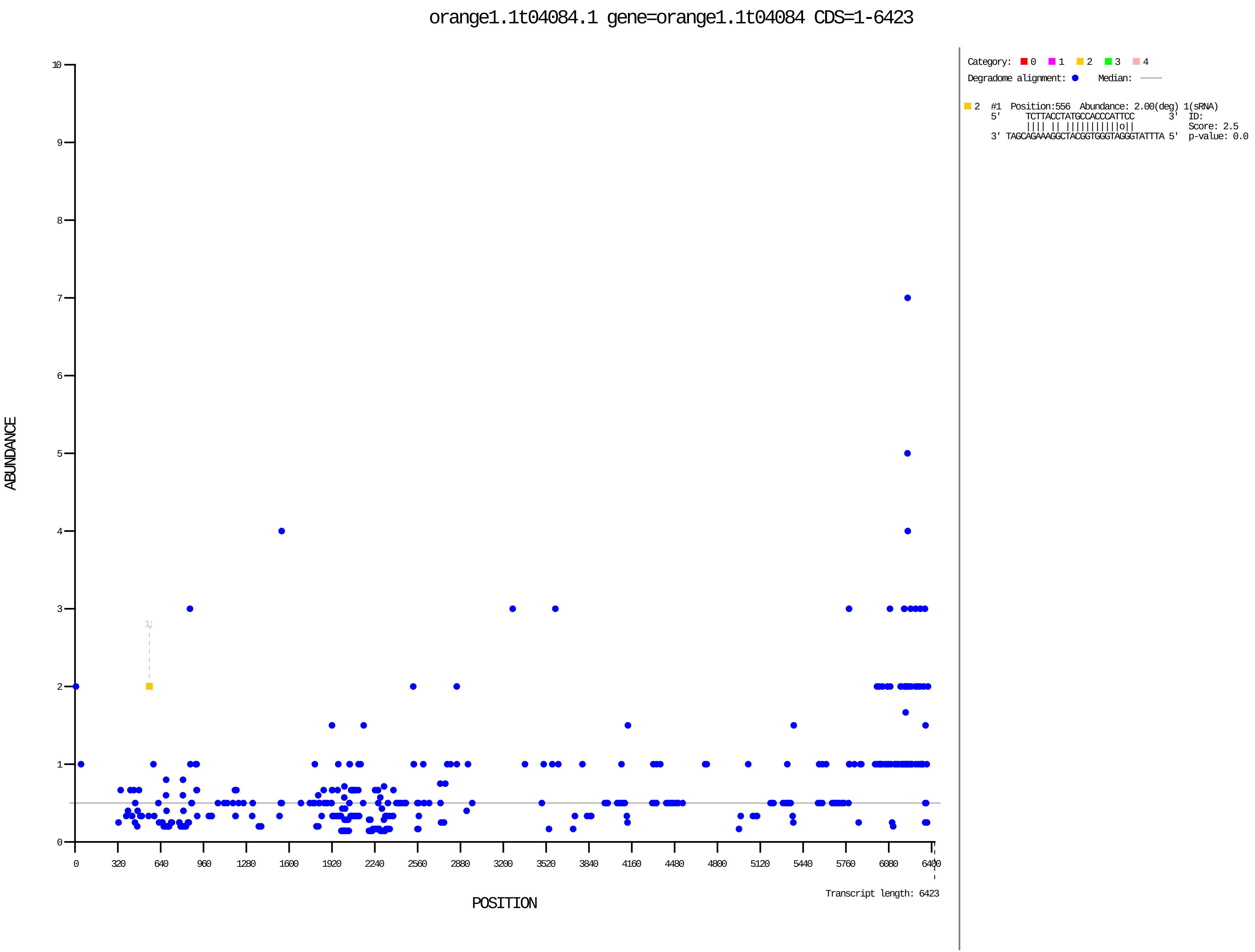
orange1.1t04084.1 gene=orange1.1t04084 CDS=1-6423
Category: 0 1 2 3 4
Degradome alignment:
Median:
2 #1 Position:556 Abundance: 2.00(deg) 1(sRNA)
5' TCTTACCTATGCCACCCATTCC 3' ID:
|||| || |||||||||||o||
Score: 2.5
3' TAGCAGAAAGGCTACGGTGGGTAGGGTATTTA 5' p-value: 0.0
ABUNDANCE
POSITION
Transcript length: 6423

Cs7g04970.2 gene=Cs7g04970 CDS=177-1004

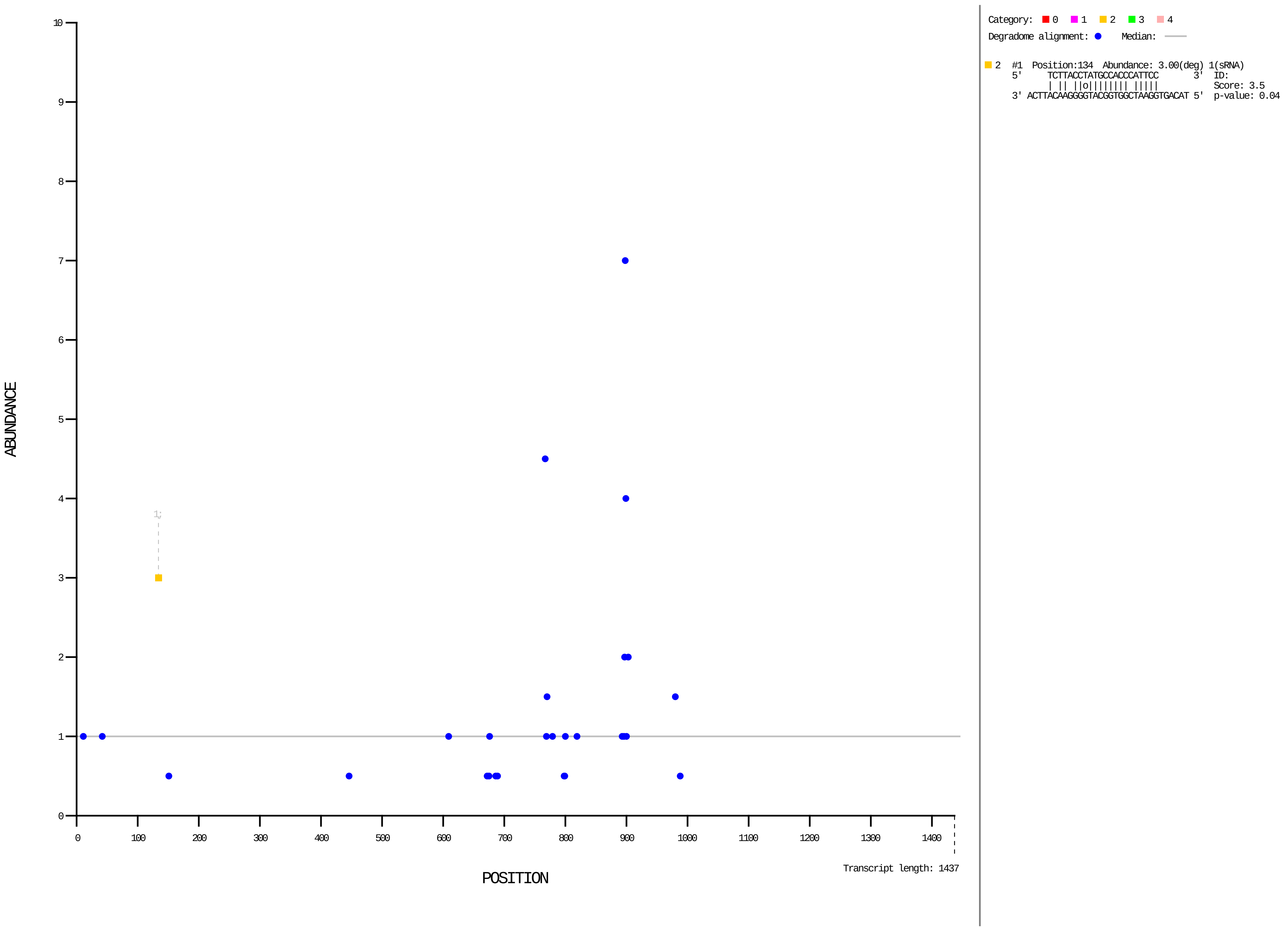

Cs1g19500.3 gene=Cs1g19500 CDS=405-2378

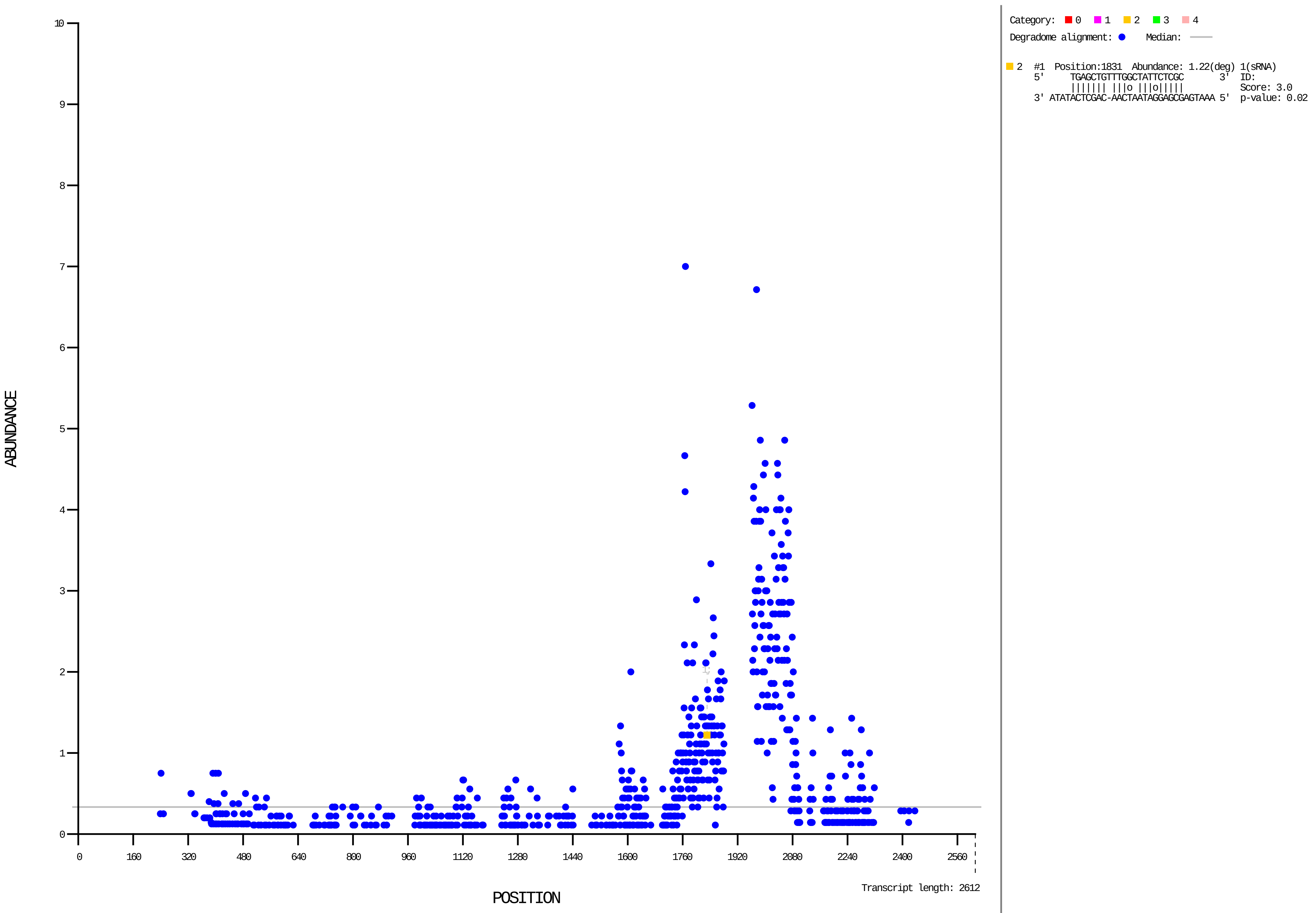

Cs1g19500.2 gene=Cs1g19500 CDS=78-2051

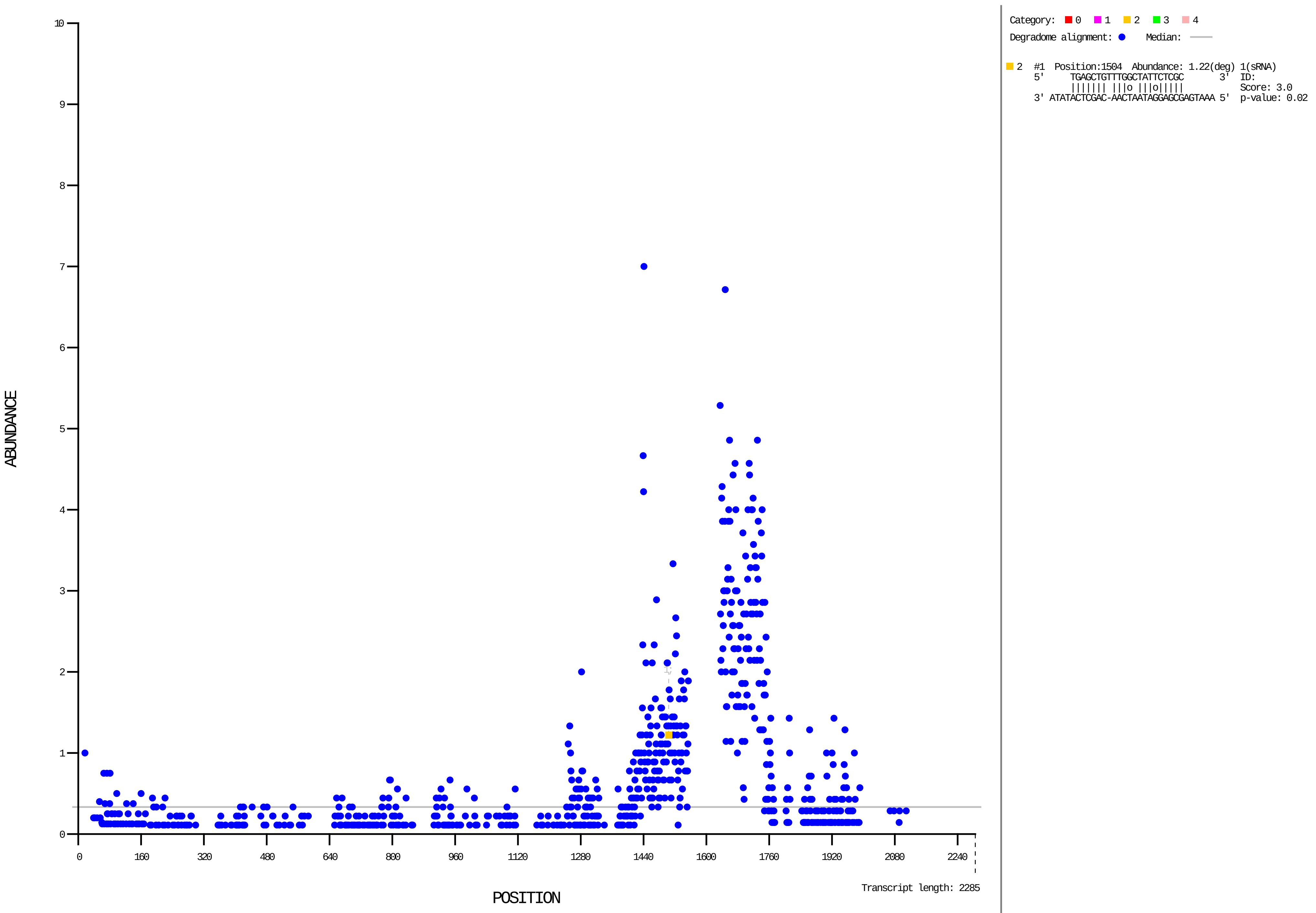

Cs1g19500.1 gene=Cs1g19500 CDS=414-2108

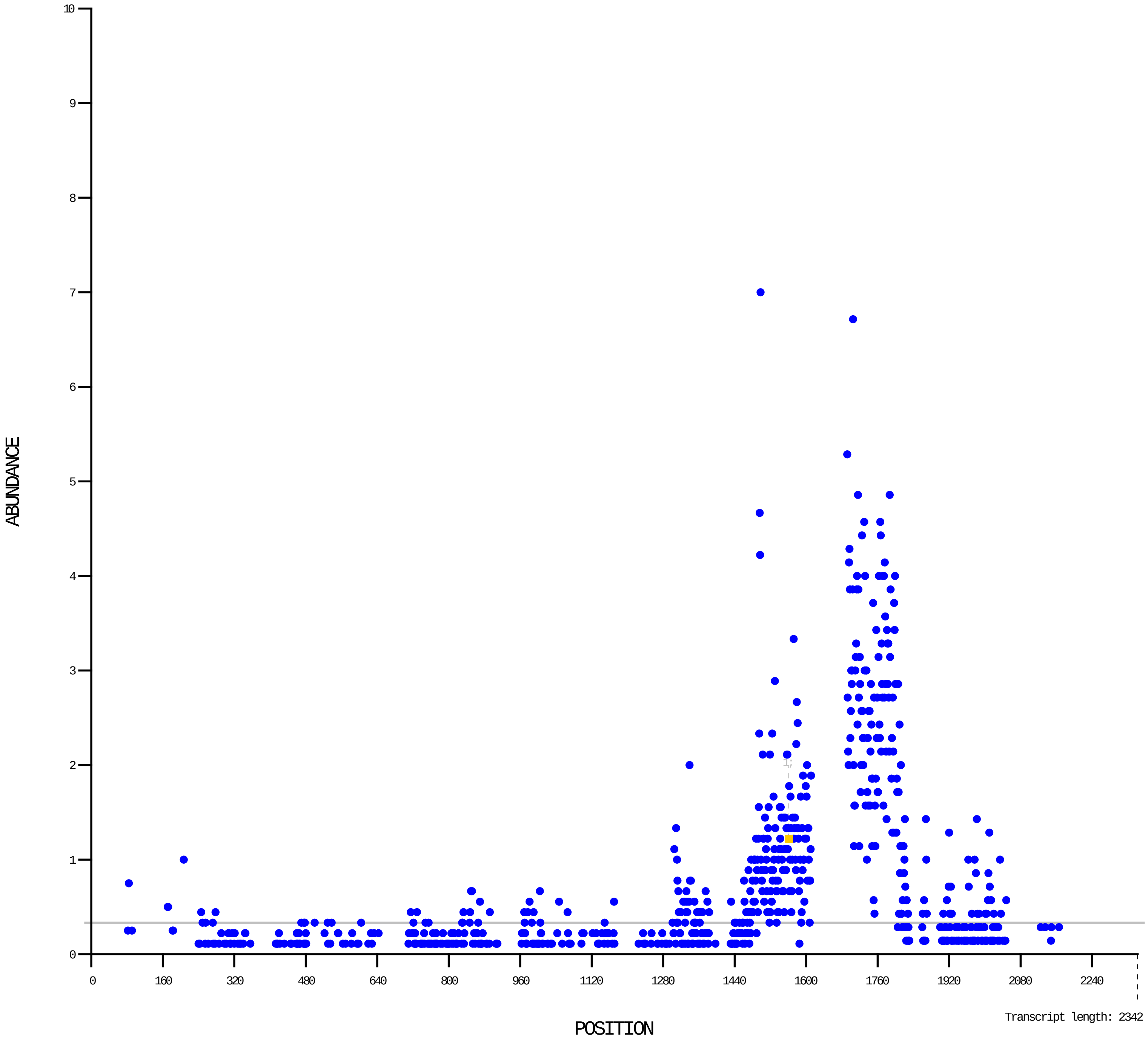

Category: 0 1 2 3 4

Degradome alignment: ● Median: —

```
2  #1  Position:1561  Abundance: 1.22(deg) 1(sRNA)
   5'     TGAGCTGTTTGGCTATTCTCGC        3'  ID:
          ||||||| |||o |||o|||||            Score: 3.0
   3' ATATACTCGAC-AACTAATAGGAGCGAGTAAA 5'  p-value: 0.04
```

Cs2g06030.2 gene=Cs2g06030 CDS=1325-4183

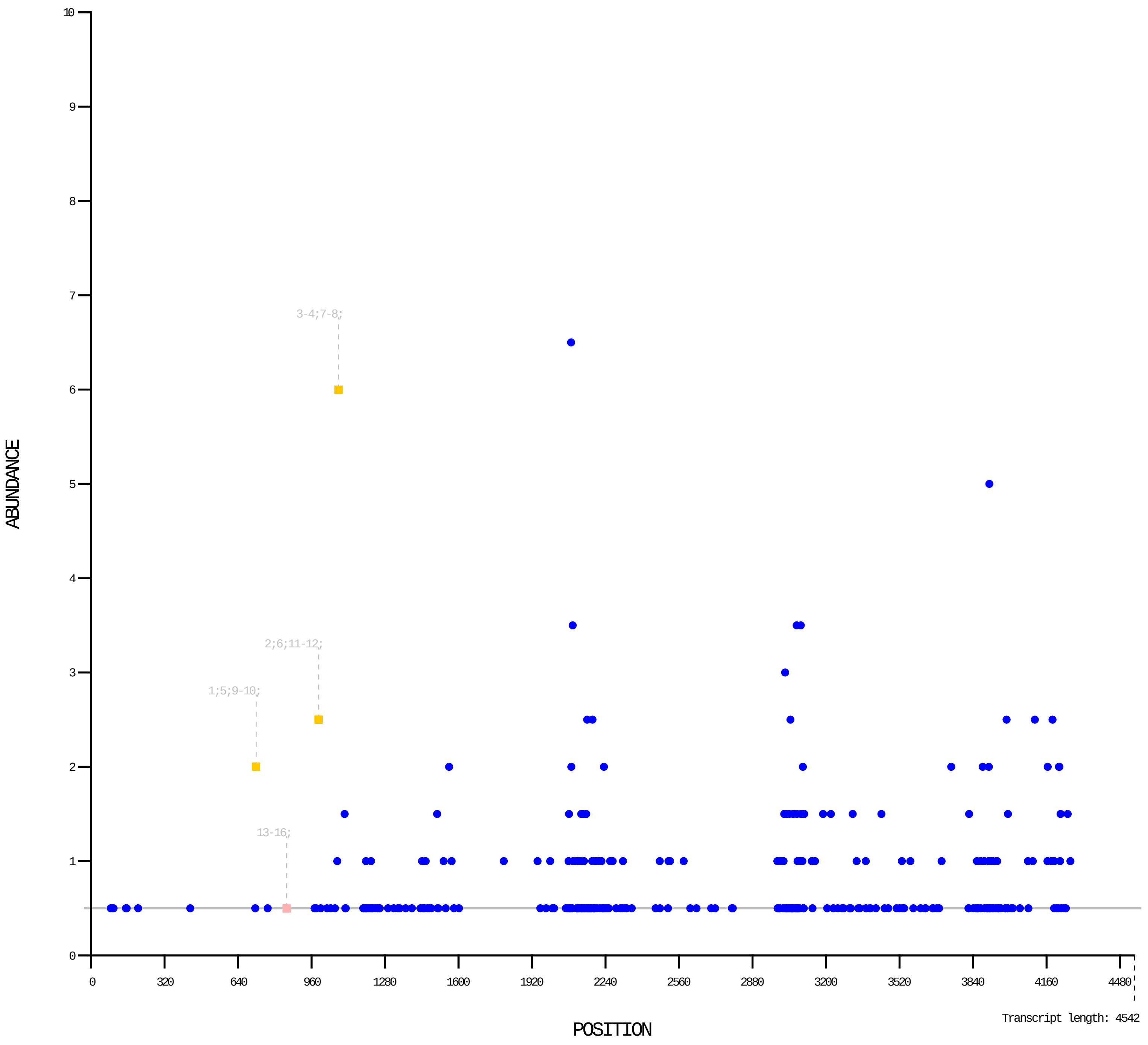

Category: 0 1 2 3 4
Degradome alignment: Median:

```
2  #1  Position:719  Abundance: 2.00(deg) 1(sRNA)
   5'     TGCCAAAGGAGATTTGCCCGG        3'  ID:
          ||||||||||||||||||oo             Score: 1.0
   3' AAAAACGGTTTCCTCTAAACGGGTTGTCACCT 5'  p-value: 0.0

2  #2  Position:991  Abundance: 2.50(deg) 1(sRNA)
   5'     TGCCAAAGGAGATTTGCCCGG        3'  ID:
          ||||||||||||||||||oo             Score: 1.0
   3' GTTTACGGTTTCCTCTAAACGGGTTACTTGTC 5'  p-value: 0.0

2  #3  Position:1077  Abundance: 6.00(deg) 1(sRNA)
   5'     TGCCAAAGGAGATTTGCCCGG        3'  ID:
          |||||||||||||||||| o             Score: 1.5
   3' GATAACGGTTTCCTCTAAACGGGGTTGTCCCT 5'  p-value: 0.0

2  #4  Position:1077  Abundance: 6.00(deg) 1(sRNA)
   5'     TGCCAAAGGAGAGTTGCCCTA        3'  ID:
          |||||||||||| ||||||o|            Score: 1.5
   3' GATAACGGTTTCCTCTAAACGGGGTTGTCCCT 5'  p-value: 0.0

2  #5  Position:719  Abundance: 2.00(deg) 1(sRNA)
   5'     TGCCAAAGGAGAGTTGCCCTA        3'  ID:
          |||||||||||| |||||| |            Score: 2.0
   3' AAAAACGGTTTCCTCTAAACGGGTTGTCACCT 5'  p-value: 0.0

2  #6  Position:991  Abundance: 2.50(deg) 1(sRNA)
   5'     TGCCAAAGGAGAGTTGCCCTA        3'  ID:
          |||||||||||| |||||| |            Score: 2.0
   3' GTTTACGGTTTCCTCTAAACGGGTTACTTGTC 5'  p-value: 0.0

2  #7  Position:1077  Abundance: 6.00(deg) 1(sRNA)
   5'     TGCCAAAGGAGAGTTGCCCTG        3'  ID:
          |||||||||||| ||||||oo            Score: 2.0
   3' GATAACGGTTTCCTCTAAACGGGGTTGTCCCT 5'  p-value: 0.02

2  #8  Position:1077  Abundance: 6.00(deg) 1(sRNA)
   5'     TGCCAAAGGAGAATTGCCCTG        3'  ID:
          |||||||||||| ||||||oo            Score: 2.0
   3' GATAACGGTTTCCTCTAAACGGGGTTGTCCCT 5'  p-value: 0.01

2  #9  Position:719  Abundance: 2.00(deg) 1(sRNA)
   5'     TGCCAAAGGAGAGTTGCCCTG        3'  ID:
          |||||||||||| |||||| o            Score: 2.5
   3' AAAAACGGTTTCCTCTAAACGGGTTGTCACCT 5'  p-value: 0.0

2  #10  Position:719  Abundance: 2.00(deg) 1(sRNA)
   5'     TGCCAAAGGAGAATTGCCCTG        3'  ID:
          |||||||||||| |||||| o            Score: 2.5
   3' AAAAACGGTTTCCTCTAAACGGGTTGTCACCT 5'  p-value: 0.01

2  #11  Position:991  Abundance: 2.50(deg) 1(sRNA)
   5'     TGCCAAAGGAGAGTTGCCCTG        3'  ID:
          |||||||||||| |||||| o            Score: 2.5
   3' GTTTACGGTTTCCTCTAAACGGGTTACTTGTC 5'  p-value: 0.01

2  #12  Position:991  Abundance: 2.50(deg) 1(sRNA)
   5'     TGCCAAAGGAGAATTGCCCTG        3'  ID:
          |||||||||||| |||||| o            Score: 2.5
   3' GTTTACGGTTTCCTCTAAACGGGTTACTTGTC 5'  p-value: 0.01

4  #13  Position:852  Abundance: 0.50(deg) 1(sRNA)
   5'     TGCCAAAGGAGATTTGCCCGG        3'  ID:
          ||||||||||||o||||| |             Score: 1.5
   3' ACTTACGGTTTCCTCTAGACGGGACGTCACCA 5'  p-value: 0.0

4  #14  Position:852  Abundance: 0.50(deg) 1(sRNA)
   5'     TGCCAAAGGAGAGTTGCCCTG        3'  ID:
          |||||||||||| o||||||             Score: 1.5
   3' ACTTACGGTTTCCTCTAGACGGGACGTCACCA 5'  p-value: 0.01

4  #15  Position:852  Abundance: 0.50(deg) 1(sRNA)
   5'     TGCCAAAGGAGAATTGCCCTG        3'  ID:
          |||||||||||| o||||||             Score: 1.5
   3' ACTTACGGTTTCCTCTAGACGGGACGTCACCA 5'  p-value: 0.0

4  #16  Position:852  Abundance: 0.50(deg) 1(sRNA)
   5'     TGCCAAAGGAGAGTTGCCCTA        3'  ID:
          |||||||||||| o|||||              Score: 2.5
   3' ACTTACGGTTTCCTCTAGACGGGACGTCACCA 5'  p-value: 0.02
```

Cs2g06030.1 gene=Cs2g06030 CDS=1325-4045

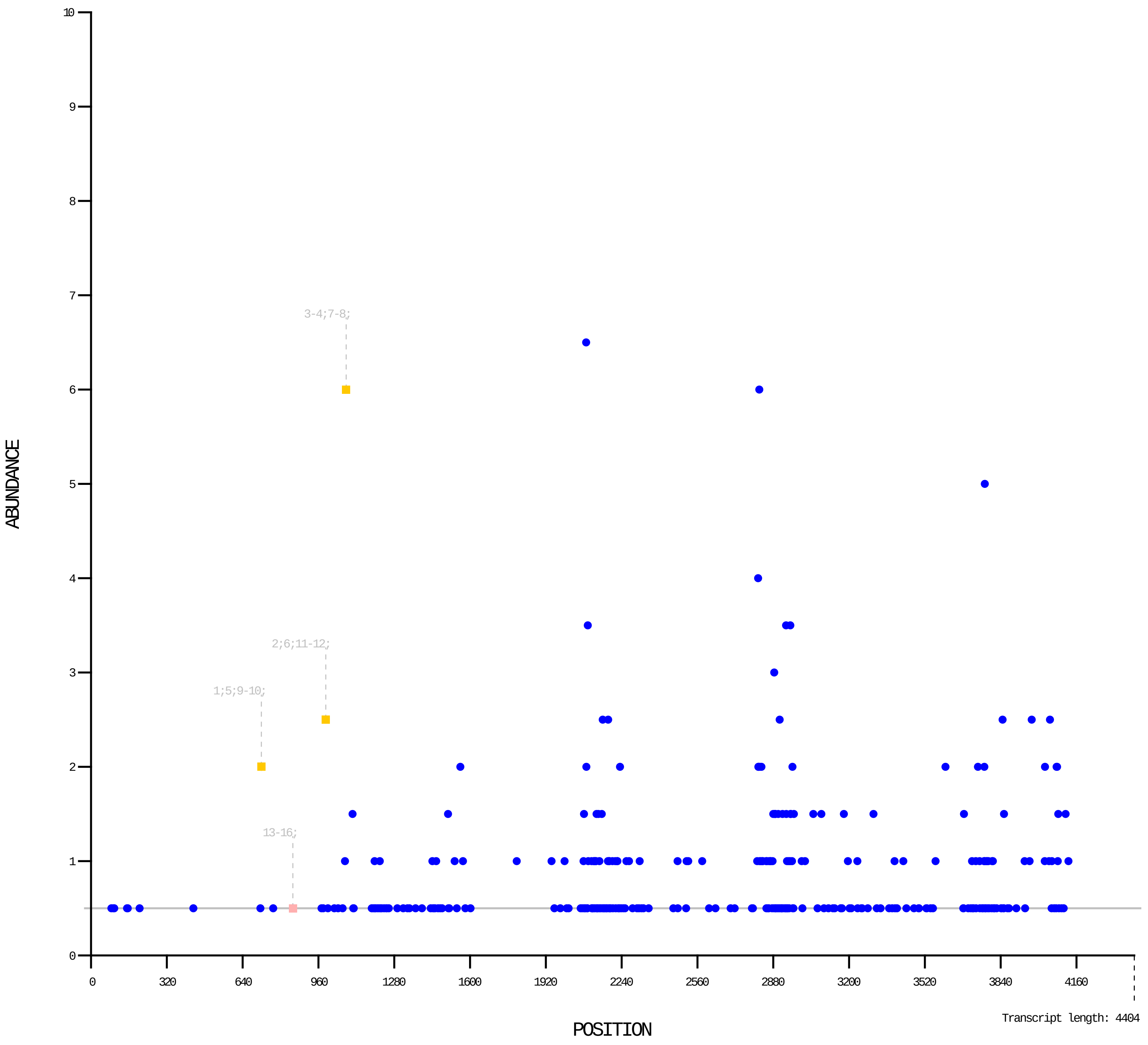

Category: 0 1 2 3 4

Degradome alignment: ● Median: —

```
2  #1  Position:719  Abundance: 2.00(deg) 1(sRNA)
   5'     TGCCAAAGGAGATTTGCCCGG        3'  ID:
          |||||||||||||||||||oo             Score: 1.0
   3' AAAAACGGTTTCCTCTAAACGGGTTGTCACCT 5'  p-value: 0.0

2  #2  Position:991  Abundance: 2.50(deg) 1(sRNA)
   5'     TGCCAAAGGAGATTTGCCCGG        3'  ID:
          |||||||||||||||||||oo             Score: 1.0
   3' GTTTACGGTTTCCTCTAAACGGGTTACTTGTC 5'  p-value: 0.0

2  #3  Position:1077  Abundance: 6.00(deg) 1(sRNA)
   5'     TGCCAAAGGAGATTTGCCCGG        3'  ID:
          ||||||||||||||||||| o             Score: 1.5
   3' GATAACGGTTTCCTCTAAACGGGGTTGTCCCT 5'  p-value: 0.01

2  #4  Position:1077  Abundance: 6.00(deg) 1(sRNA)
   5'     TGCCAAAGGAGAGTTGCCCTA        3'  ID:
          |||||||||||| ||||||o|             Score: 1.5
   3' GATAACGGTTTCCTCTAAACGGGGTTGTCCCT 5'  p-value: 0.0

2  #5  Position:719  Abundance: 2.00(deg) 1(sRNA)
   5'     TGCCAAAGGAGAGTTGCCCTA        3'  ID:
          |||||||||||| ||||||  |            Score: 2.0
   3' AAAAACGGTTTCCTCTAAACGGGTTGTCACCT 5'  p-value: 0.0

2  #6  Position:991  Abundance: 2.50(deg) 1(sRNA)
   5'     TGCCAAAGGAGAGTTGCCCTA        3'  ID:
          |||||||||||| ||||||  |            Score: 2.0
   3' GTTTACGGTTTCCTCTAAACGGGTTACTTGTC 5'  p-value: 0.0

2  #7  Position:1077  Abundance: 6.00(deg) 1(sRNA)
   5'     TGCCAAAGGAGAGTTGCCCTG        3'  ID:
          |||||||||||| ||||||oo             Score: 2.0
   3' GATAACGGTTTCCTCTAAACGGGGTTGTCCCT 5'  p-value: 0.0

2  #8  Position:1077  Abundance: 6.00(deg) 1(sRNA)
   5'     TGCCAAAGGAGAATTGCCCTG        3'  ID:
          |||||||||||| ||||||oo             Score: 2.0
   3' GATAACGGTTTCCTCTAAACGGGGTTGTCCCT 5'  p-value: 0.0

2  #9  Position:719  Abundance: 2.00(deg) 1(sRNA)
   5'     TGCCAAAGGAGAGTTGCCCTG        3'  ID:
          |||||||||||| |||||| o             Score: 2.5
   3' AAAAACGGTTTCCTCTAAACGGGTTGTCACCT 5'  p-value: 0.04

2  #10  Position:719  Abundance: 2.00(deg) 1(sRNA)
   5'     TGCCAAAGGAGAATTGCCCTG        3'  ID:
          |||||||||||| |||||| o             Score: 2.5
   3' AAAAACGGTTTCCTCTAAACGGGTTGTCACCT 5'  p-value: 0.01

2  #11  Position:991  Abundance: 2.50(deg) 1(sRNA)
   5'     TGCCAAAGGAGAGTTGCCCTG        3'  ID:
          |||||||||||| |||||| o             Score: 2.5
   3' GTTTACGGTTTCCTCTAAACGGGTTACTTGTC 5'  p-value: 0.03

2  #12  Position:991  Abundance: 2.50(deg) 1(sRNA)
   5'     TGCCAAAGGAGAATTGCCCTG        3'  ID:
          |||||||||||| |||||| o             Score: 2.5
   3' GTTTACGGTTTCCTCTAAACGGGTTACTTGTC 5'  p-value: 0.04

4  #13  Position:852  Abundance: 0.50(deg) 1(sRNA)
   5'     TGCCAAAGGAGATTTGCCCGG        3'  ID:
          |||||||||||||o||||| |             Score: 1.5
   3' ACTTACGGTTTCCTCTAGACGGGACGTCACCA 5'  p-value: 0.0

4  #14  Position:852  Abundance: 0.50(deg) 1(sRNA)
   5'     TGCCAAAGGAGAGTTGCCCTG        3'  ID:
          |||||||||||| o|||||||             Score: 1.5
   3' ACTTACGGTTTCCTCTAGACGGGACGTCACCA 5'  p-value: 0.0

4  #15  Position:852  Abundance: 0.50(deg) 1(sRNA)
   5'     TGCCAAAGGAGAATTGCCCTG        3'  ID:
          |||||||||||| o|||||||             Score: 1.5
   3' ACTTACGGTTTCCTCTAGACGGGACGTCACCA 5'  p-value: 0.0

4  #16  Position:852  Abundance: 0.50(deg) 1(sRNA)
   5'     TGCCAAAGGAGAGTTGCCCTA        3'  ID:
          |||||||||||| o||||||              Score: 2.5
   3' ACTTACGGTTTCCTCTAGACGGGACGTCACCA 5'  p-value: 0.0
```

orange1.1t04079.1 gene=orange1.1t04079 CDS=1-7716

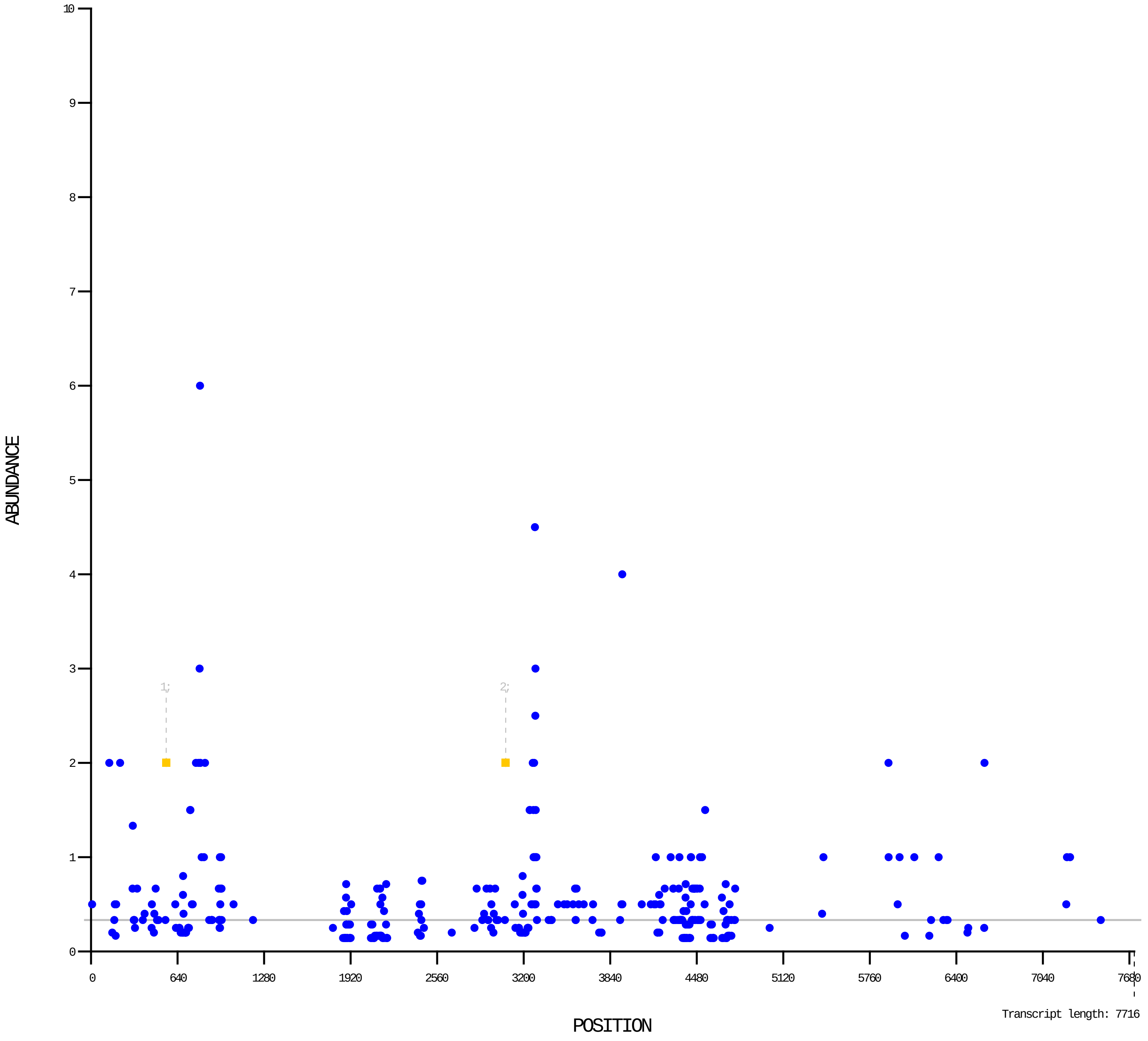

Category: 0 1 2 3 4
Degradome alignment: Median:

2 #1 Position:556 Abundance: 2.00(deg) 1(sRNA)
5' TCTTACCTATGCCACCCATTCC 3' ID:
|||| || |||||||||||o|| Score: 2.5
3' TAGCAGAAAGGCTACGGTGGGTAGGGTATTTA 5' p-value: 0.01

2 #2 Position:3067 Abundance: 2.00(deg) 1(sRNA)
5' TCTTACCTATGCCACCCATTCC 3' ID:
|||| || |||||||||||o|| Score: 2.5
3' TAGCAGAAAGGCTACGGTGGGTAGGGTATTTA 5' p-value: 0.0

Cs1g09350.2 gene=Cs1g09350 CDS=74-2956

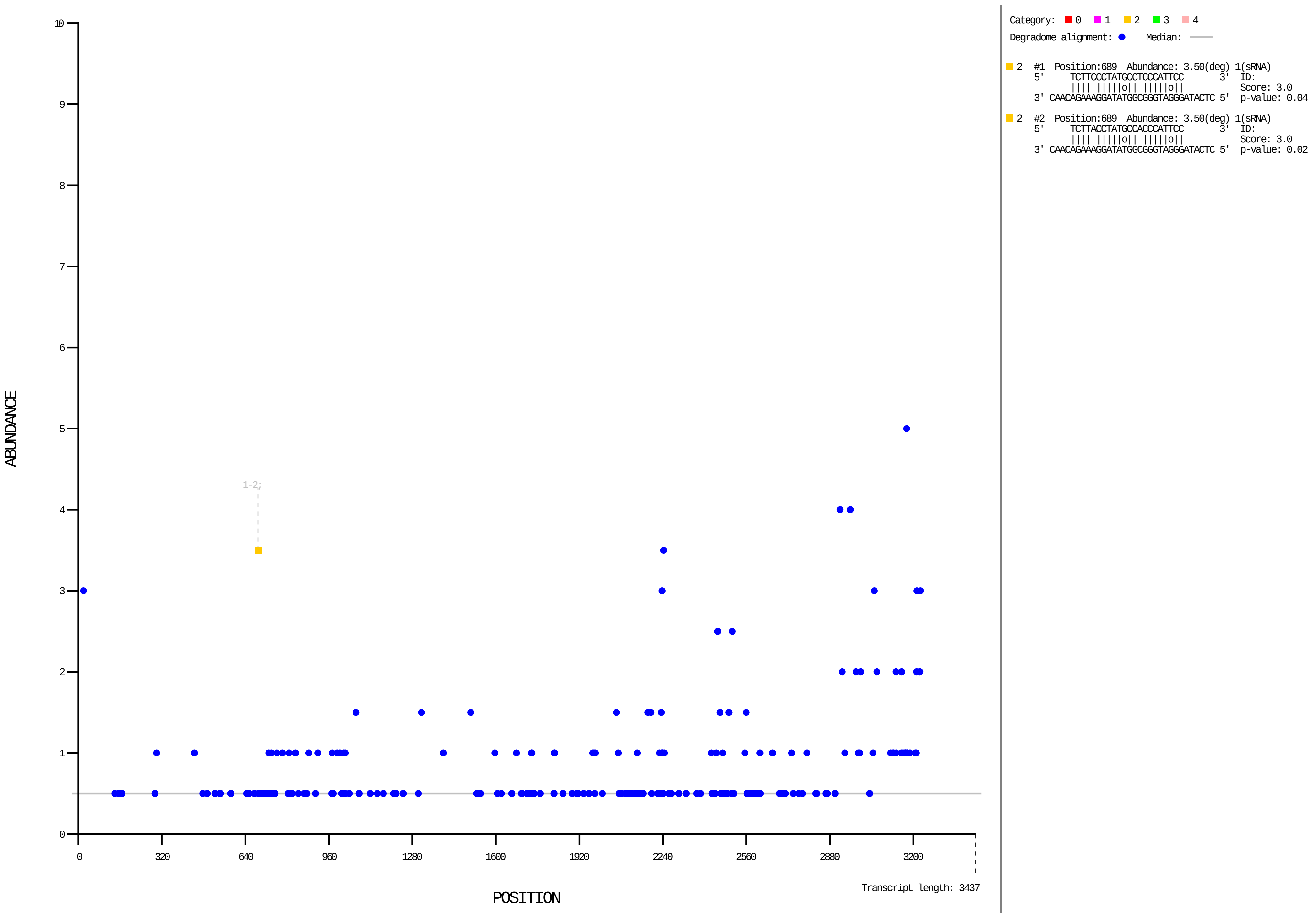

Cs4g19310.5 gene=Cs4g19310 CDS=275-2770

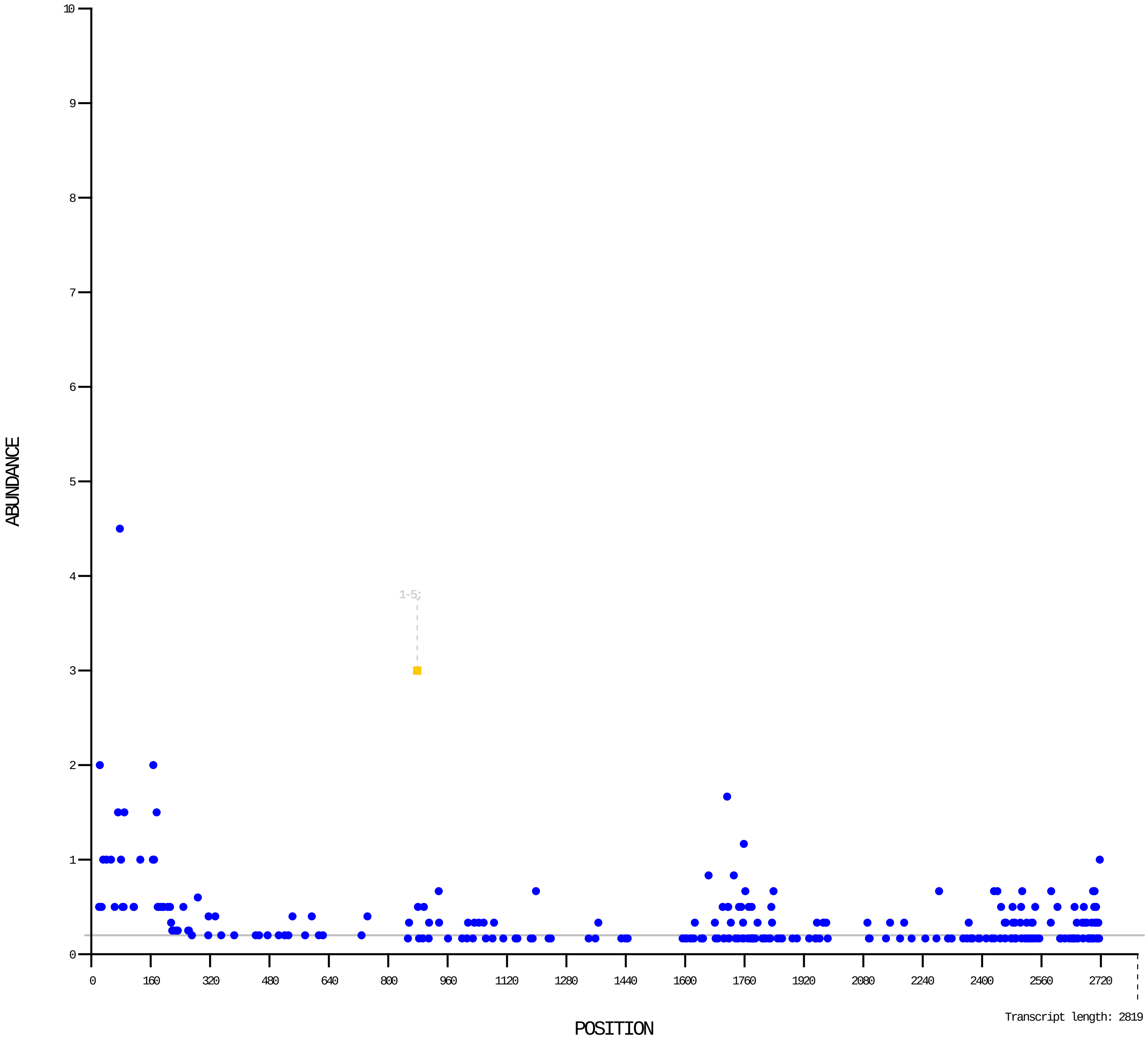

Category: 0 1 2 3 4
Degradome alignment: Median:

```
2  #1  Position:878  Abundance: 3.00(deg) 1(sRNA)
   5'     TCGGACCAGGCTTCATTCCC        3'  ID:
          o||||||||||||||| |||            Score: 1.5
   3' CTTAGGCCTGGTCCGAAGTA-GGGCCCGTAAA 5'  p-value: 0.0

2  #2  Position:878  Abundance: 3.00(deg) 1(sRNA)
   5'     TCGGACCAGGCTTCATTCCCT       3'  ID:
          o||||||||||||||| |||            Score: 2.5
   3' CTTAGGCCTGGTCCGAAGTA-GGGCCCGTAAA 5'  p-value: 0.01

2  #3  Position:878  Abundance: 3.00(deg) 1(sRNA)
   5'     TCGGACCAGGCTTCATTCCCGT      3'  ID:
          o||||||||||||||| ||||           Score: 2.5
   3' CTTAGGCCTGGTCCGAAGTA-GGGCCCGTAAA 5'  p-value: 0.0

2  #4  Position:878  Abundance: 3.00(deg) 1(sRNA)
   5'     TCGGACCAGGCTTCATTCCC        3'  ID:
          o||||||||||||||| |||            Score: 2.5
   3' CTTAGGCCTGGTCCGAAGTA-GGGCCCGTAAA 5'  p-value: 0.03

2  #5  Position:878  Abundance: 3.00(deg) 1(sRNA)
   5'     TCGGACCAGGCTTCATTCCCCC      3'  ID:
          o||||||||||||||| |||            Score: 3.5
   3' CTTAGGCCTGGTCCGAAGTA-GGGCCCGTAAA 5'  p-value: 0.05
```

Cs4g19310.3 gene=Cs4g19310 CDS=275-2827

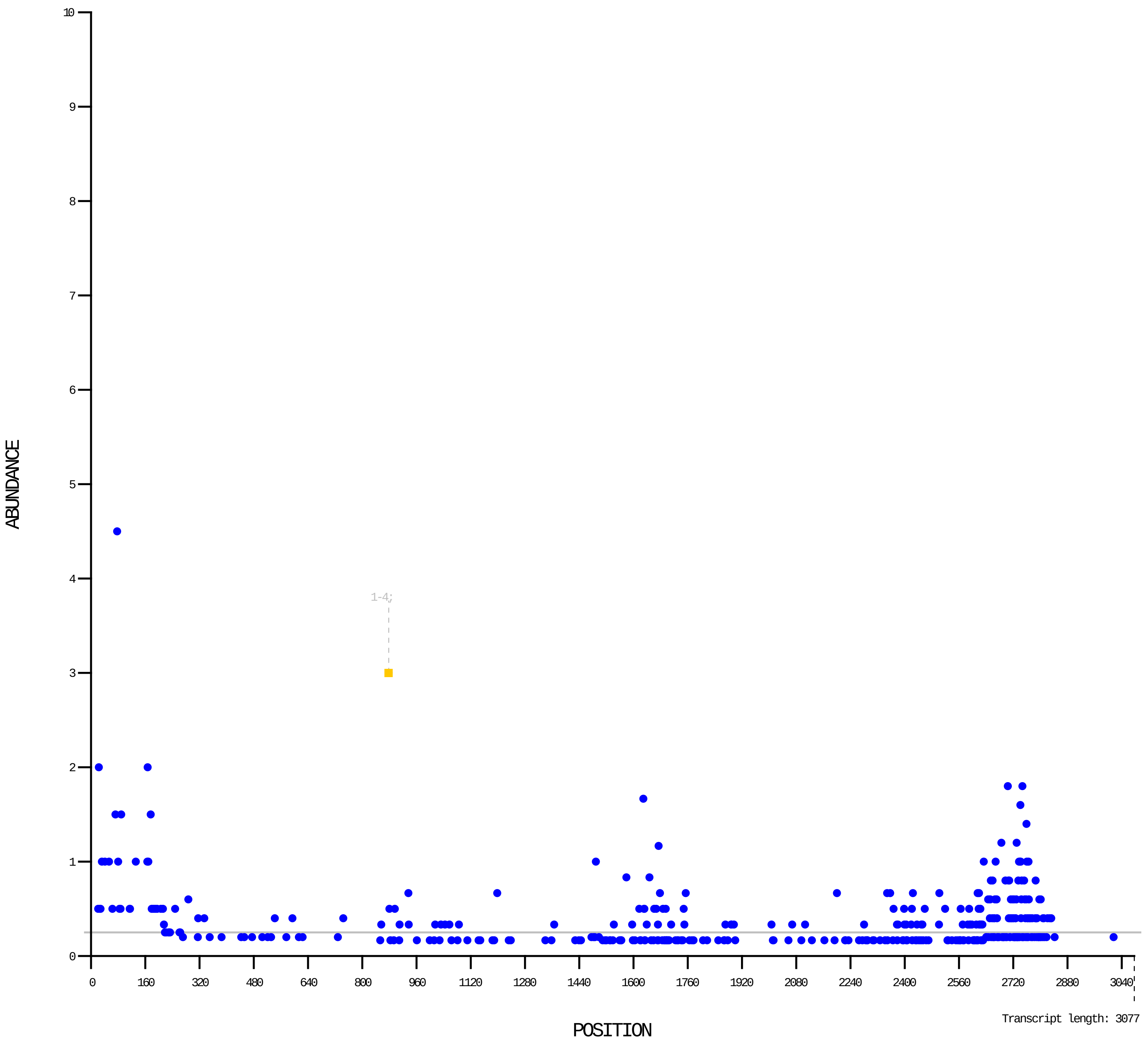

Category: 0 1 2 3 4

Degradome alignment: ● Median:

```
2  #1  Position:878  Abundance: 3.00(deg) 1(sRNA)
   5'     TCGGACCAGGCTTCATTCCC         3'  ID:
          o||||||||||||||| |||             Score: 1.5
   3' CTTAGGCCTGGTCCGAAGTA-GGGCCCGTAAA 5'  p-value: 0.0

2  #2  Position:878  Abundance: 3.00(deg) 1(sRNA)
   5'     TCGGACCAGGCTTCATTCCCT        3'  ID:
          o||||||||||||||| |||             Score: 2.5
   3' CTTAGGCCTGGTCCGAAGTA-GGGCCCGTAAA 5'  p-value: 0.01

2  #3  Position:878  Abundance: 3.00(deg) 1(sRNA)
   5'     TCGGACCAGGCTTCATTCCCGT       3'  ID:
          o||||||||||||||| ||||            Score: 2.5
   3' CTTAGGCCTGGTCCGAAGTA-GGGCCCGTAAA 5'  p-value: 0.01

2  #4  Position:878  Abundance: 3.00(deg) 1(sRNA)
   5'     TCGGACCAGGCTTCATTCCCC        3'  ID:
          o||||||||||||||| |||             Score: 2.5
   3' CTTAGGCCTGGTCCGAAGTA-GGGCCCGTAAA 5'  p-value: 0.0
```

Cs4g06220.2 gene=Cs4g06220 CDS=292-2955

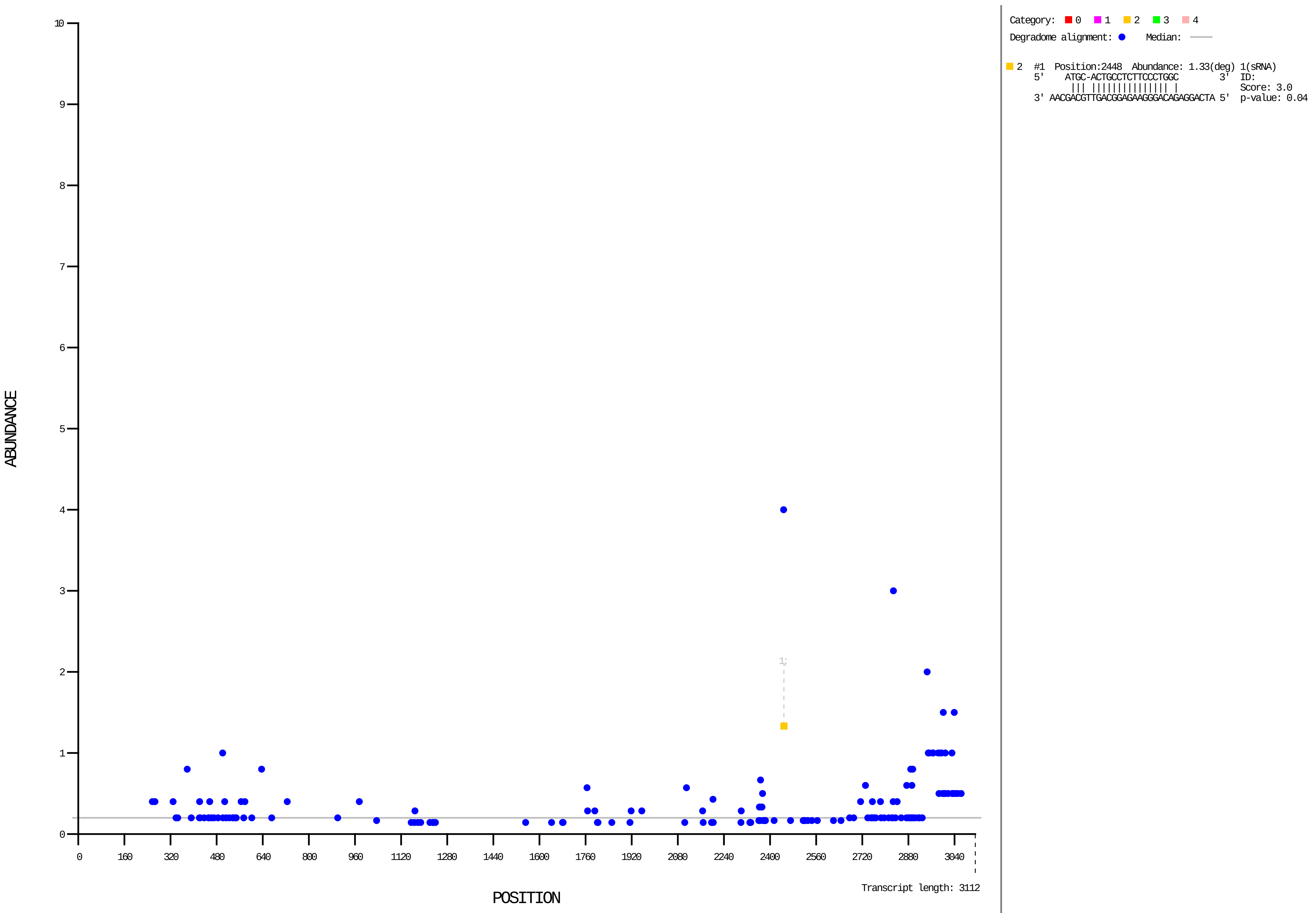

orange1.1t01536.2 gene=orange1.1t01536 CDS=299-2251

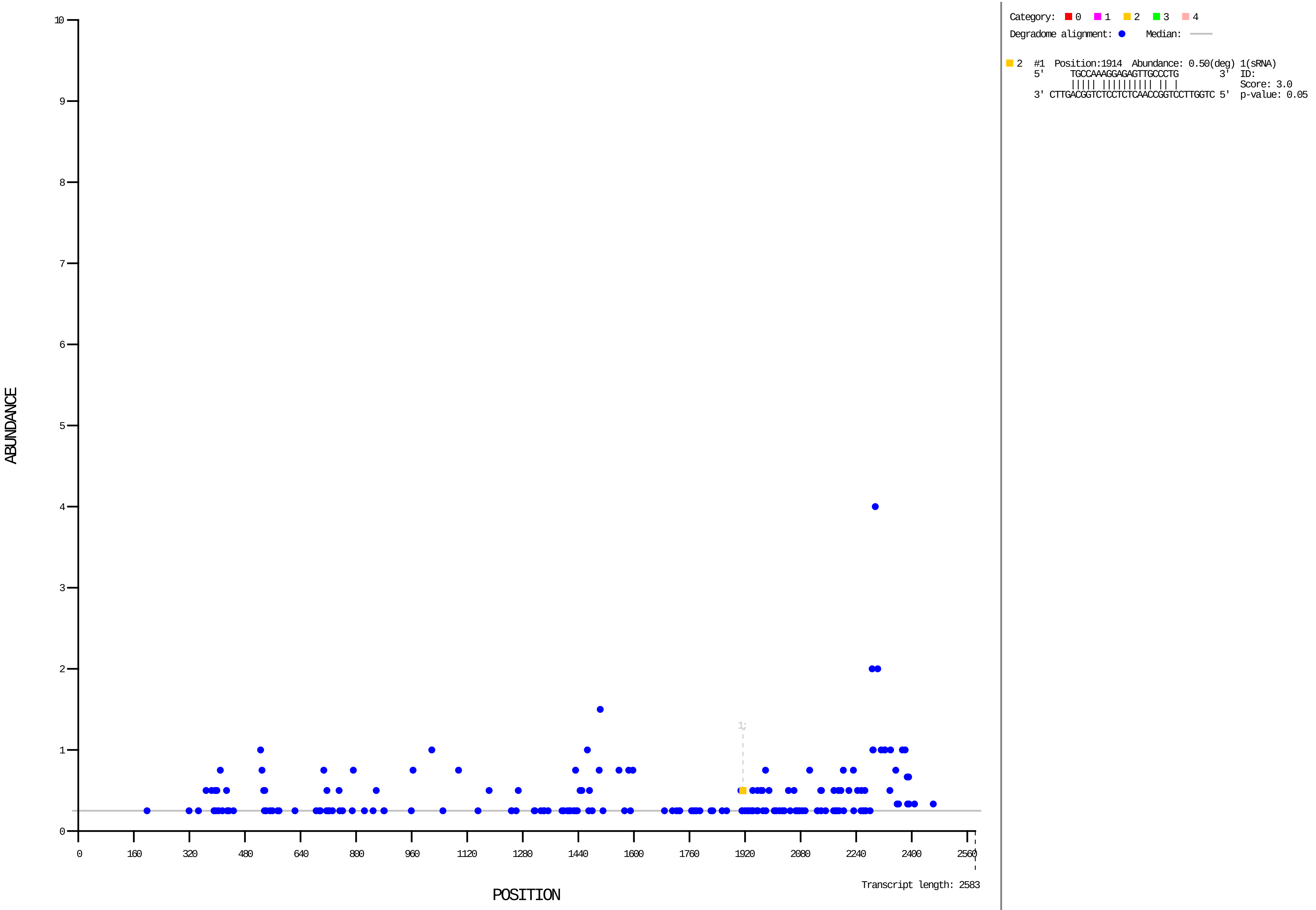

Cs1g13460.1 gene=Cs1g13460 CDS=1-2121

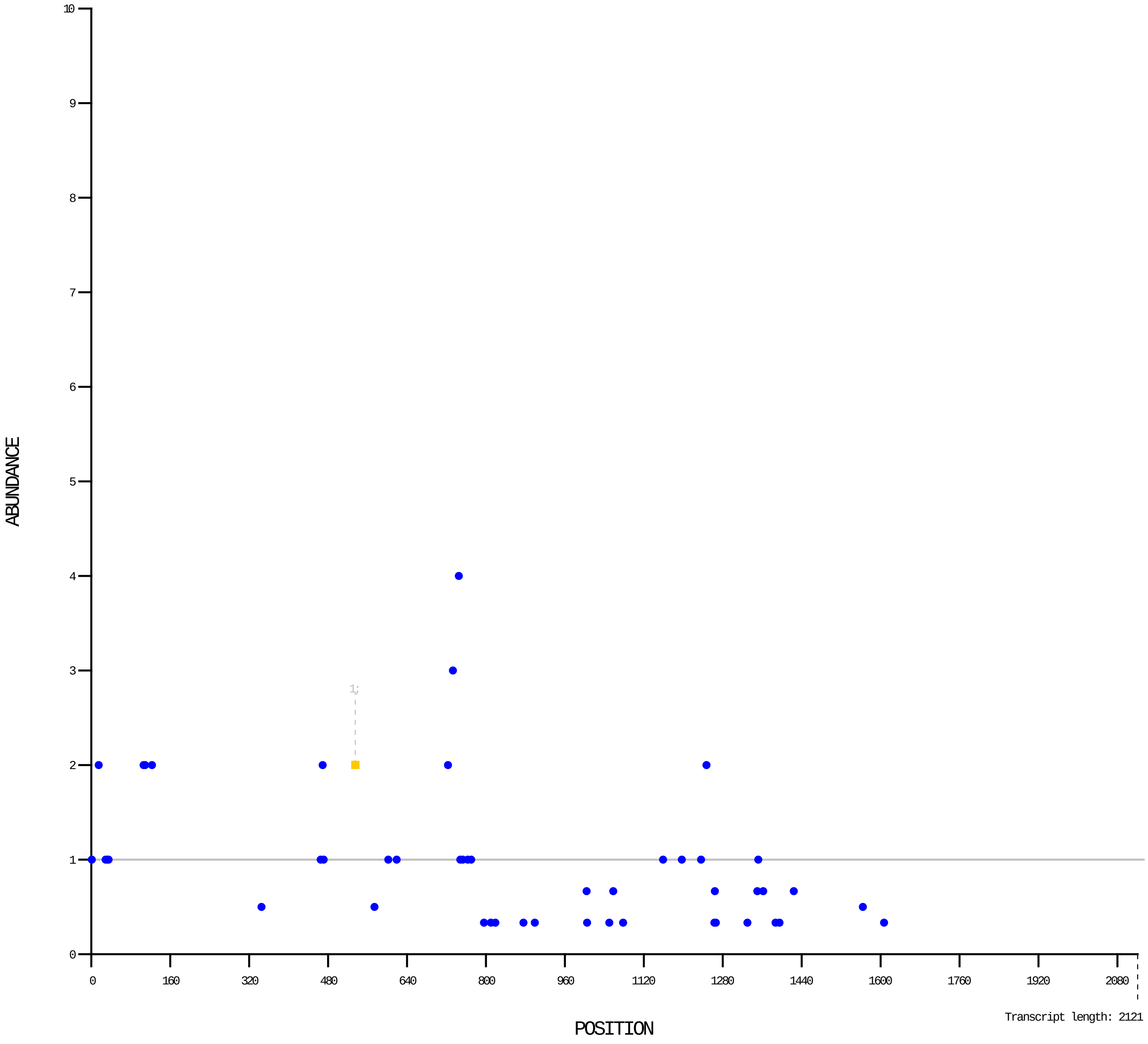

Category: 0 1 2 3 4
Degradome alignment: Median:

2 #1 Position:535 Abundance: 2.00(deg) 1(sRNA)

```
5'     TCTTGCCCACCCCTCCCATTCC       3'  ID:
       | ||o||||||||||||||| ||          Score: 2.5
3' CACCAAAATGGGTGGGGAGGGTATGGCATGTC 5'  p-value: 0.01
```

Cs3g12720.1 gene=Cs3g12720 CDS=1-1950

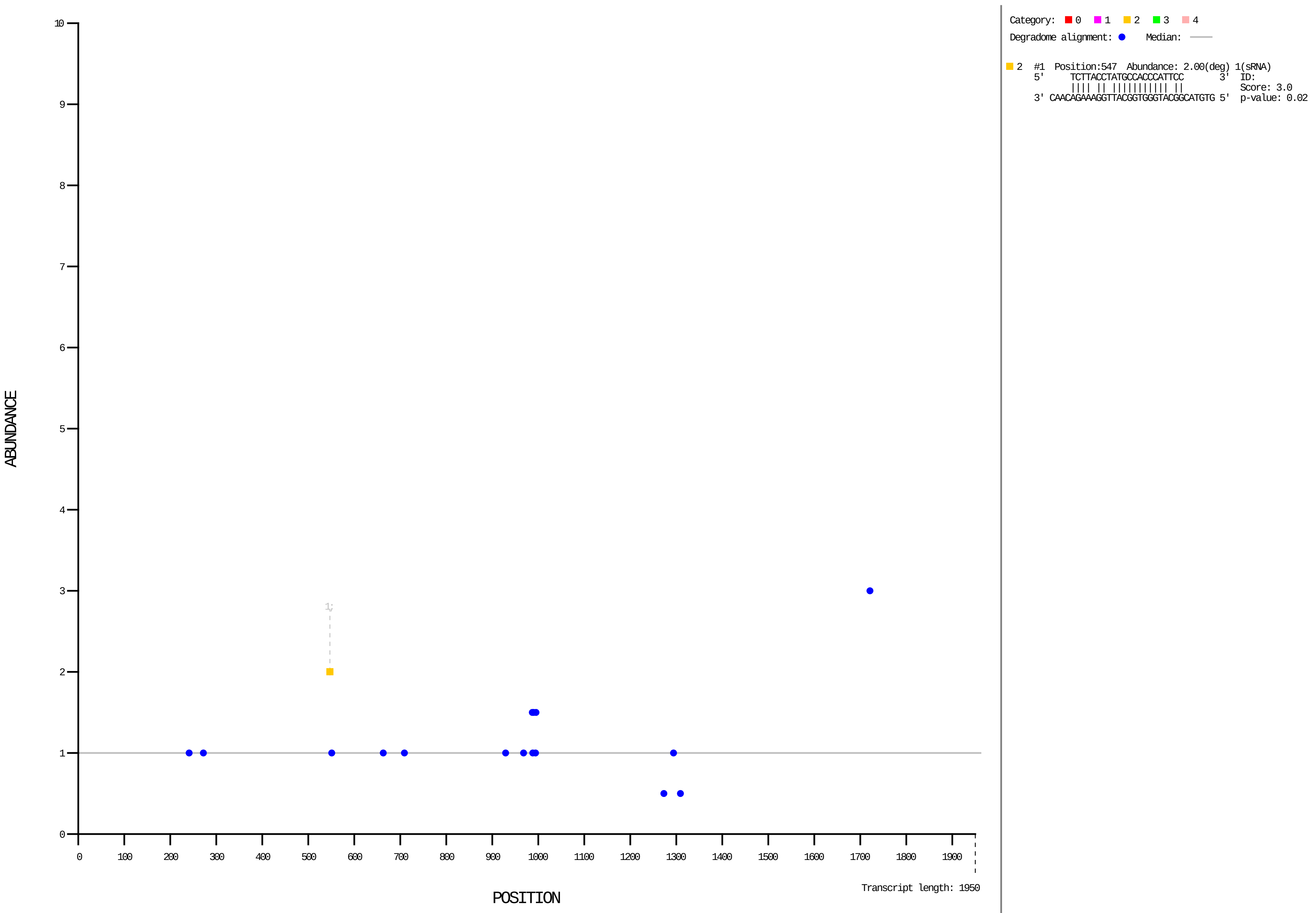

Cs9g09320.2 gene=Cs9g09320 CDS=442-2355

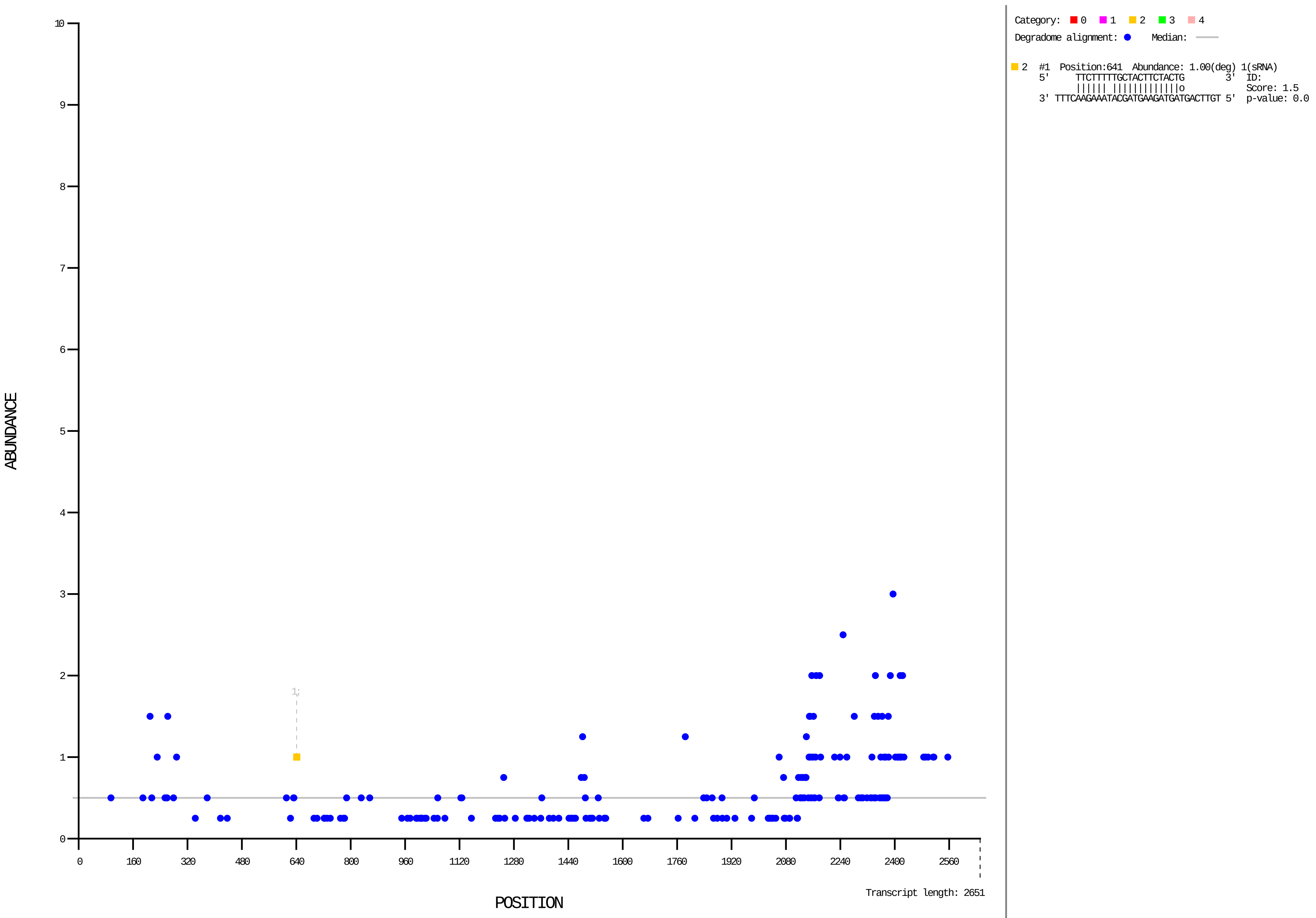

Cs2g26800.1 gene=Cs2g26800 CDS=378-1988

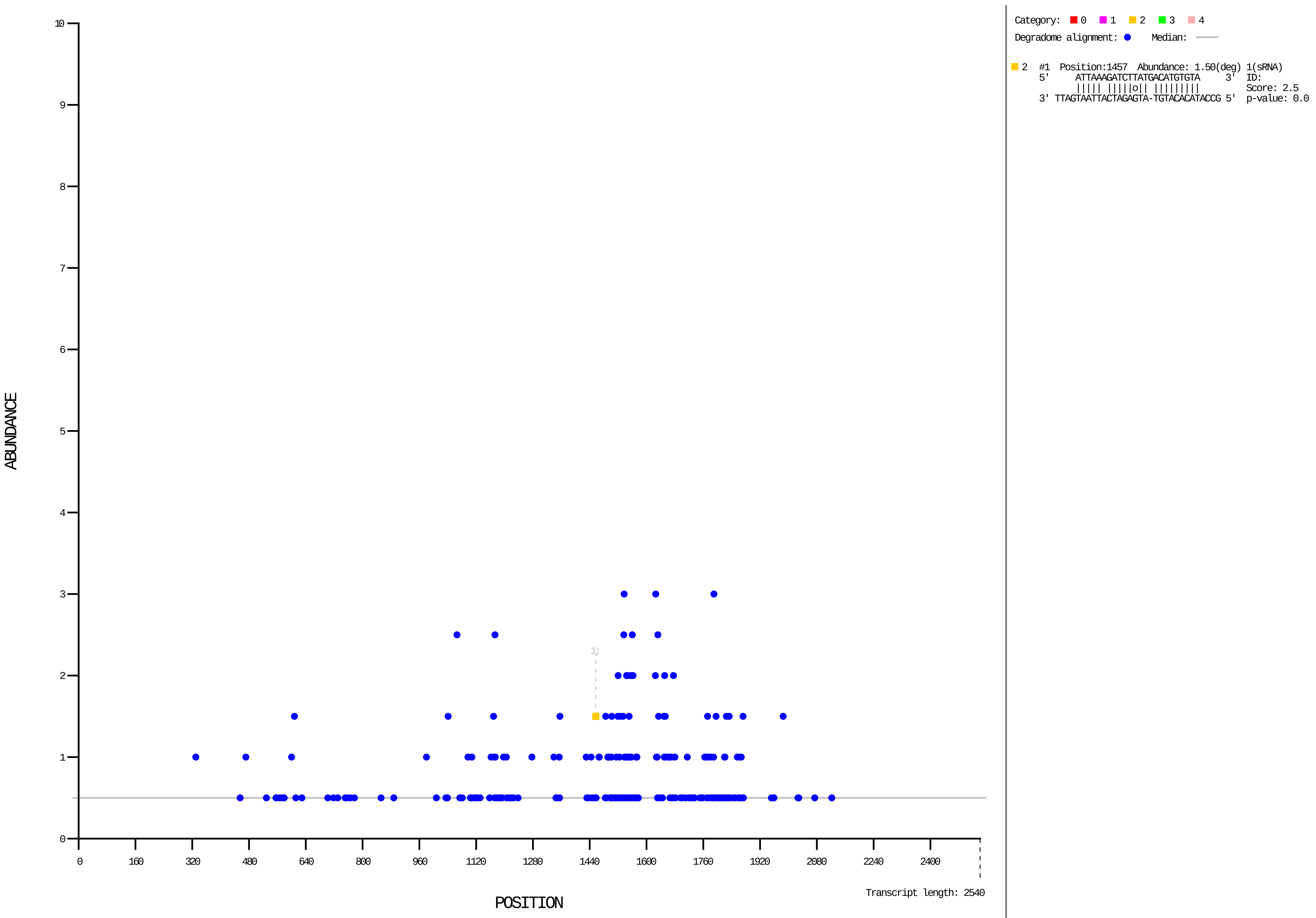

Cs2g26800.2 gene=Cs2g26800 CDS=176-1786

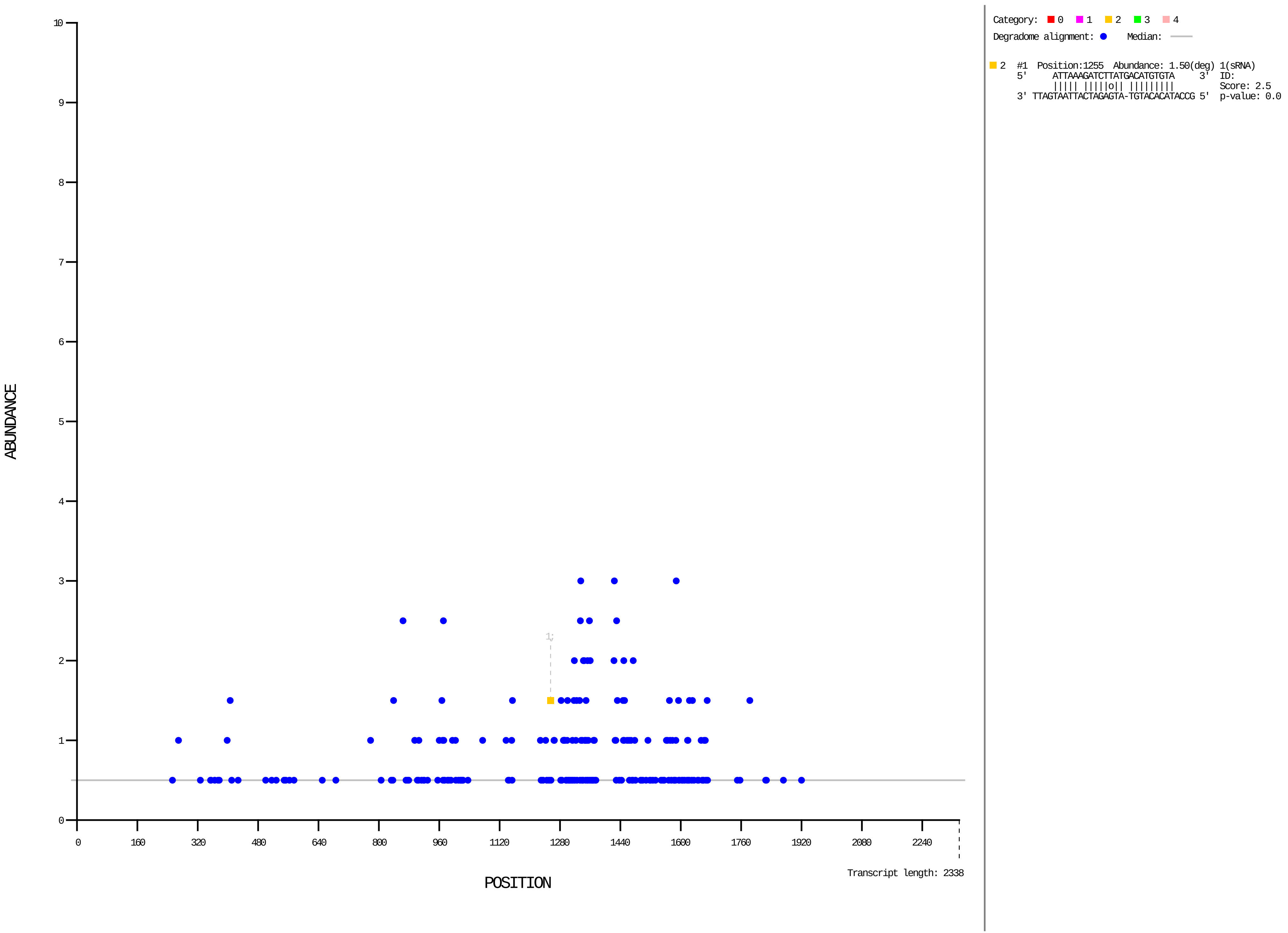

Cs3g05280.1 gene=Cs3g05280 CDS=121-3564

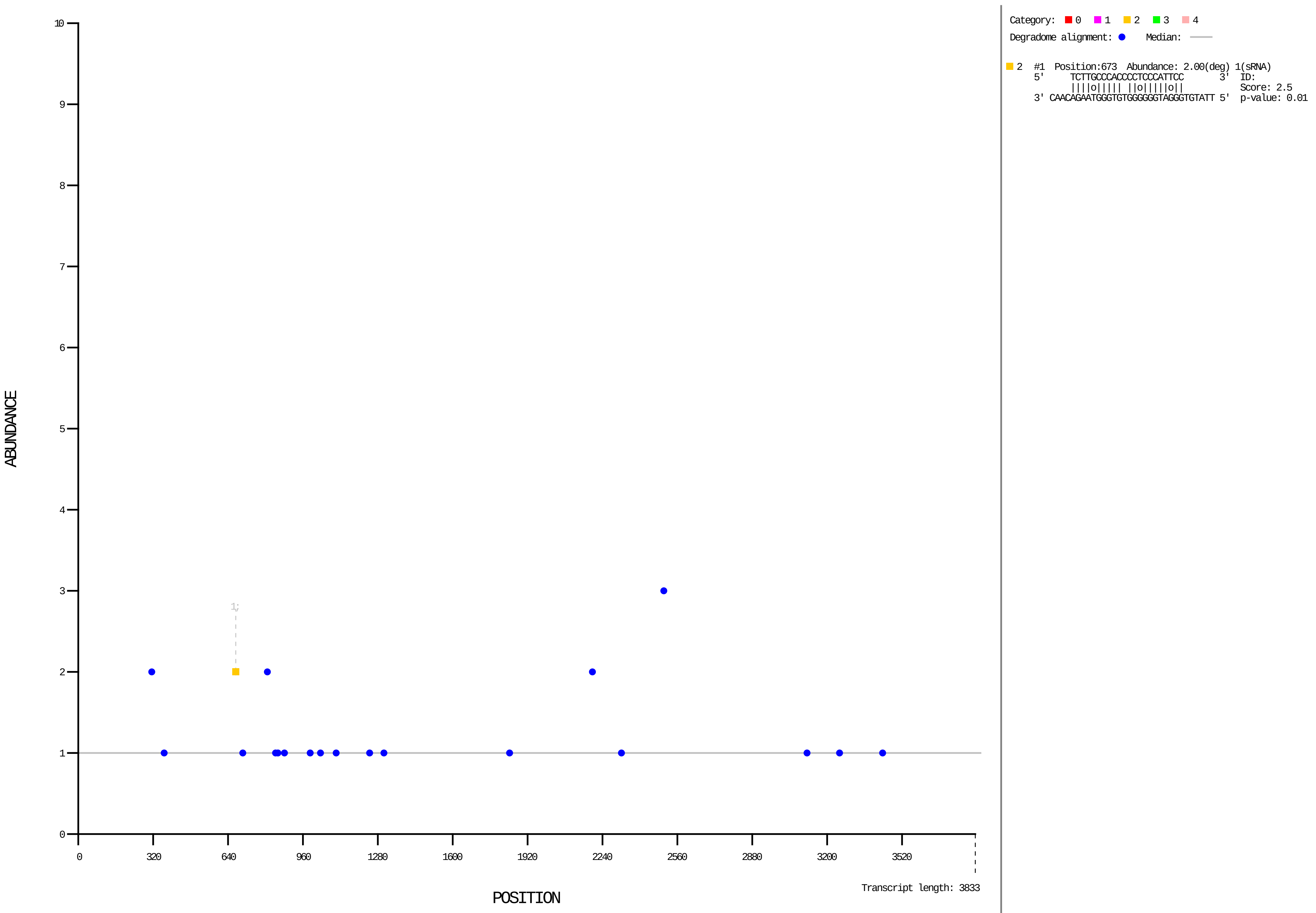

Cs9g09320.1 gene=Cs9g09320 CDS=312-2225

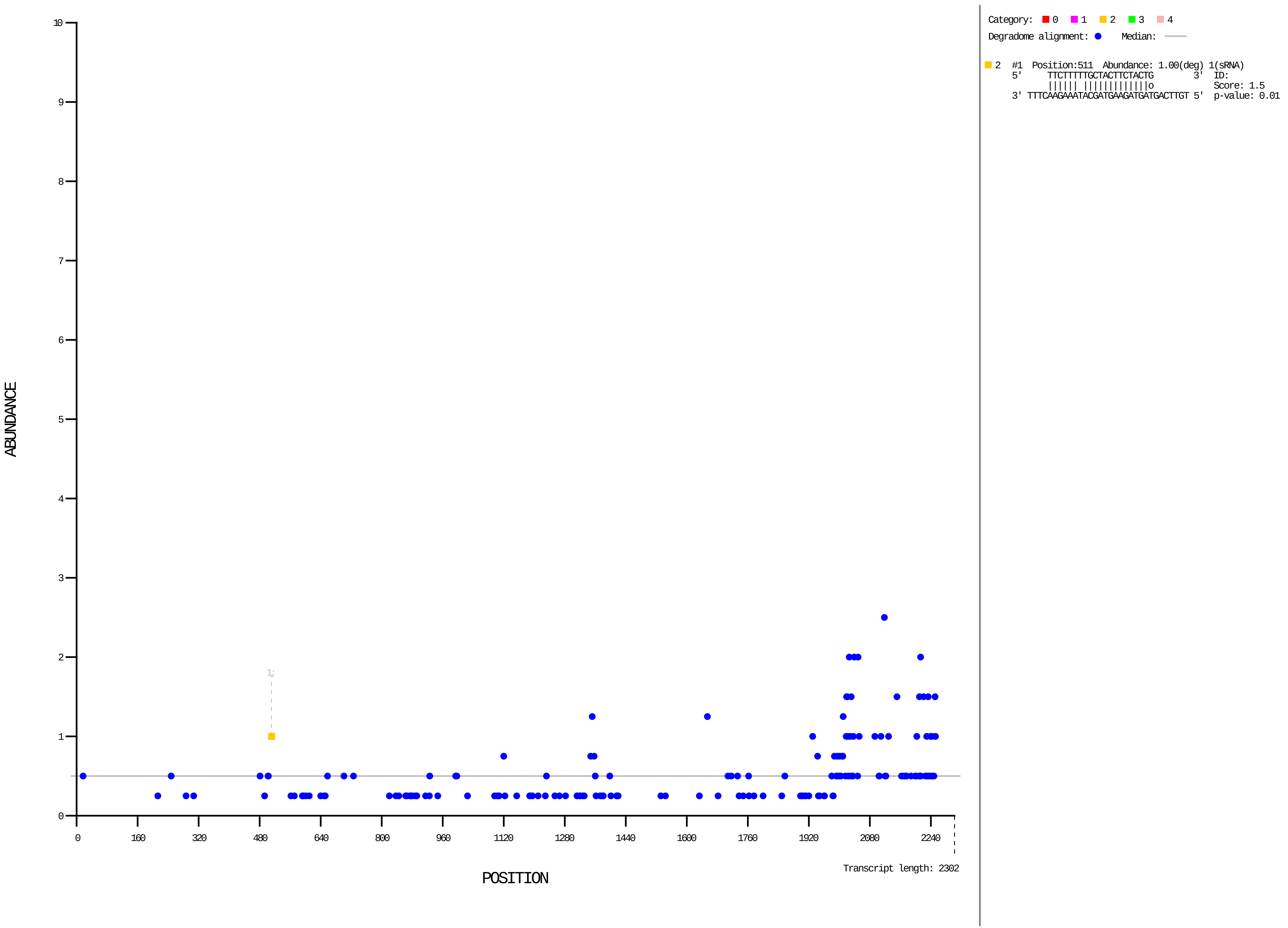

Cs7g19370.1 gene=Cs7g19370 CDS=138-3200

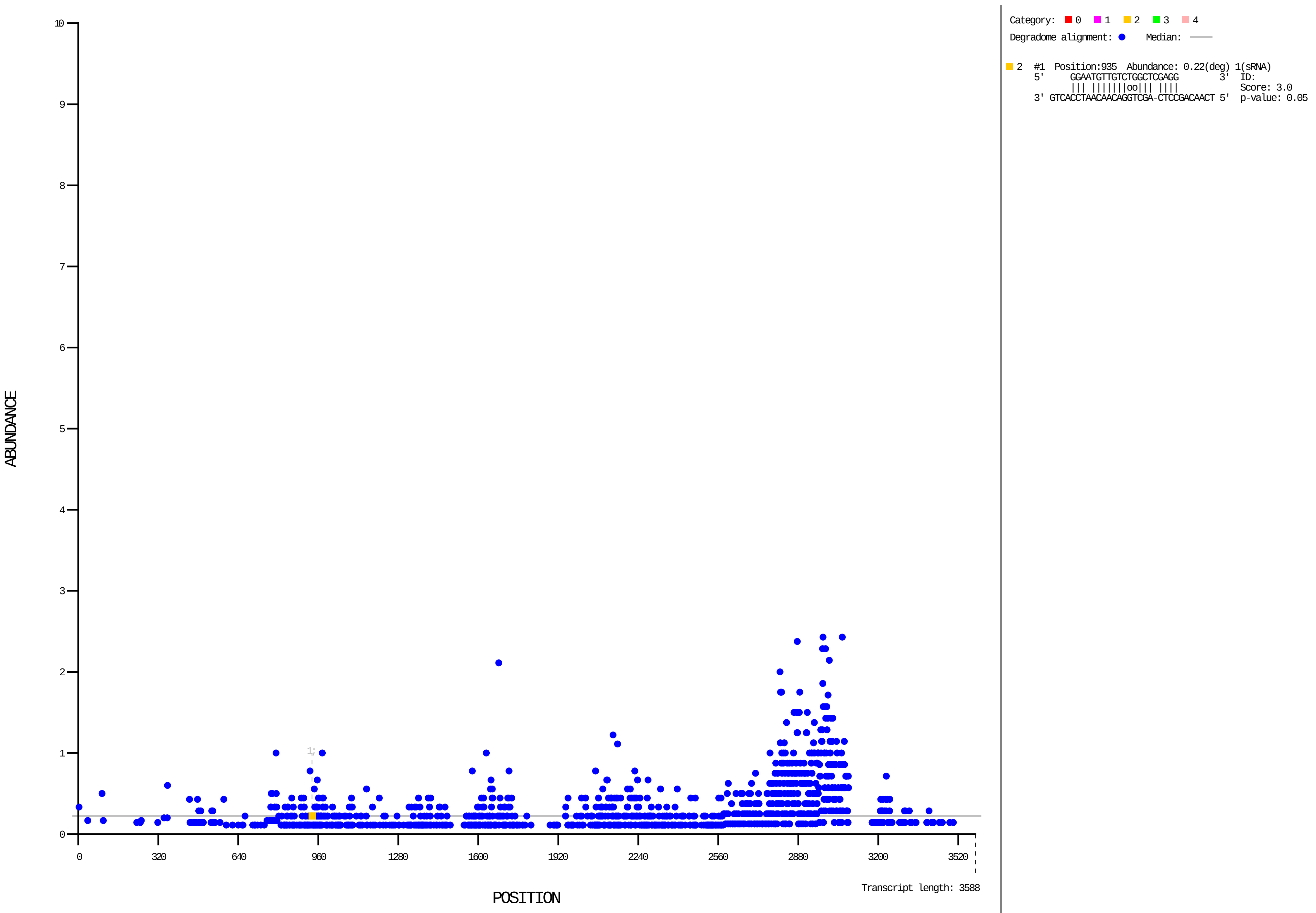

Cs7g19370.7 gene=Cs7g19370 CDS=451-3405

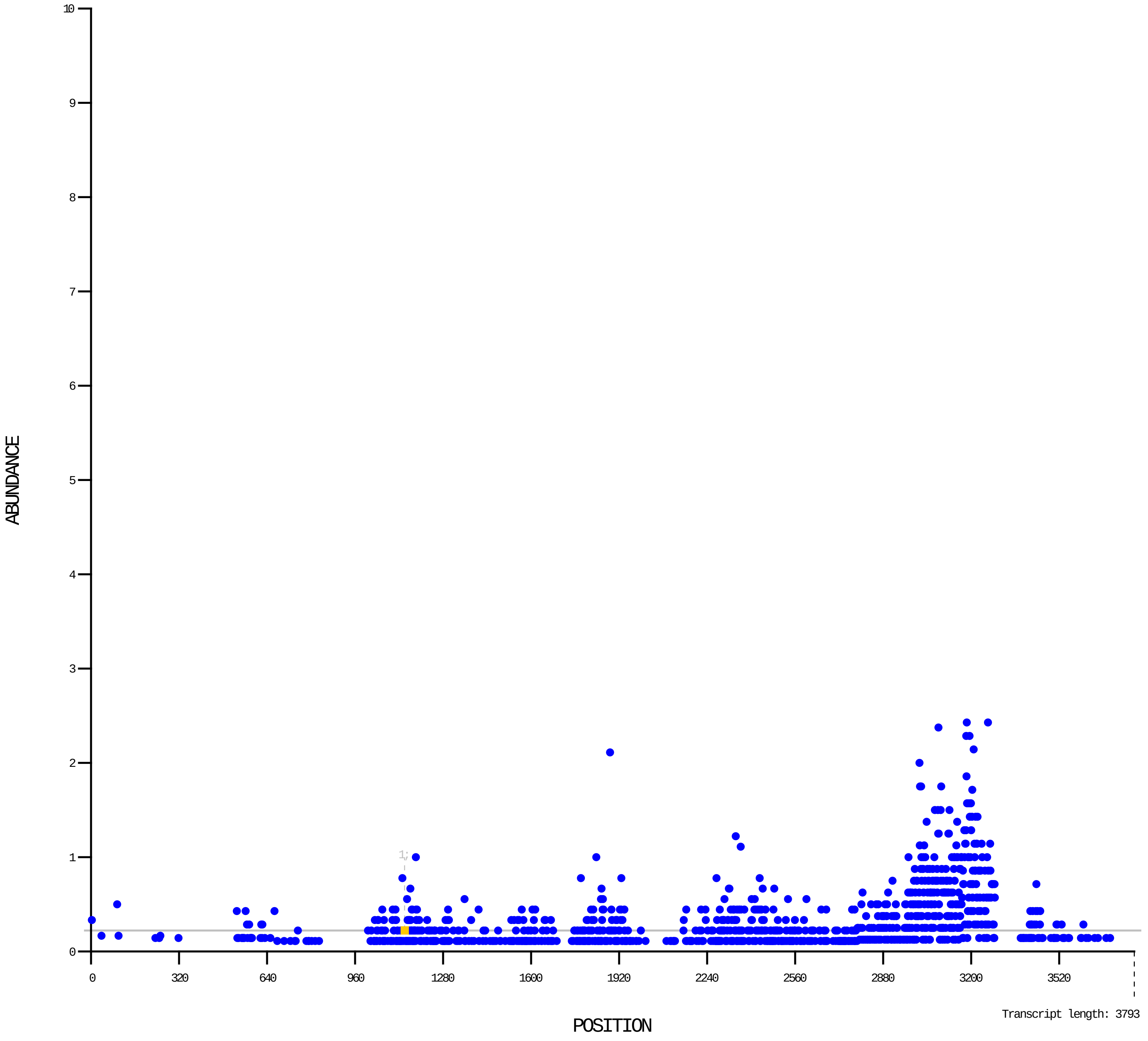

Category: 0 1 2 3 4
Degradome alignment: Median:

2 #1 Position:1140 Abundance: 0.22(deg) 1(sRNA)

```
5'     GGAATGTTGTCTGGCTCGAGG        3'  ID:
       ||| |||||||oo||| ||||            Score: 3.0
3' GTCACCTAACAACAGGTCGA-CTCCGACAACT 5'  p-value: 0.05
```

Cs7g19370.5 gene=Cs7g19370 CDS=138-2675

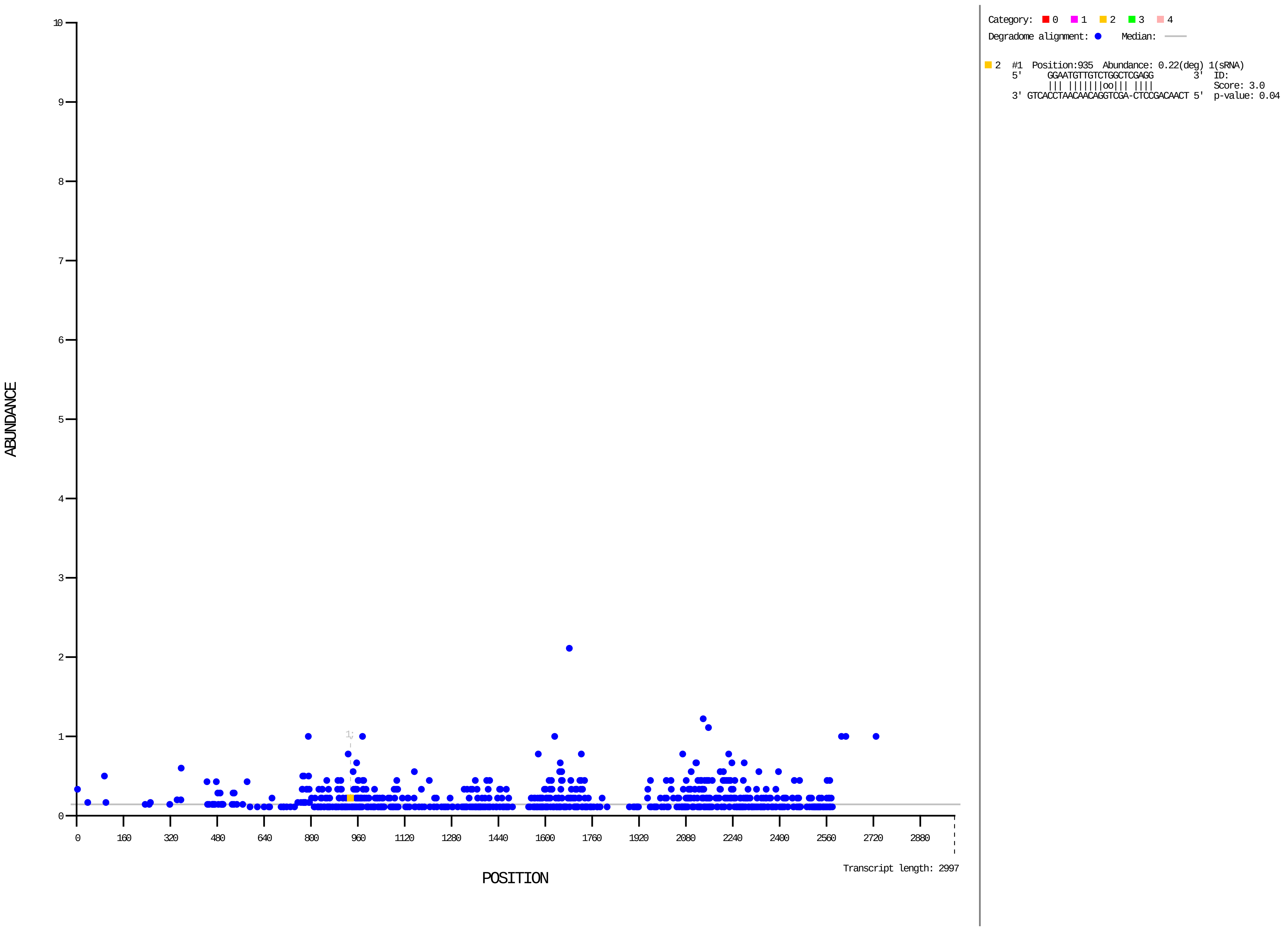

Cs5g22330.3 gene=Cs5g22330 CDS=864-1496

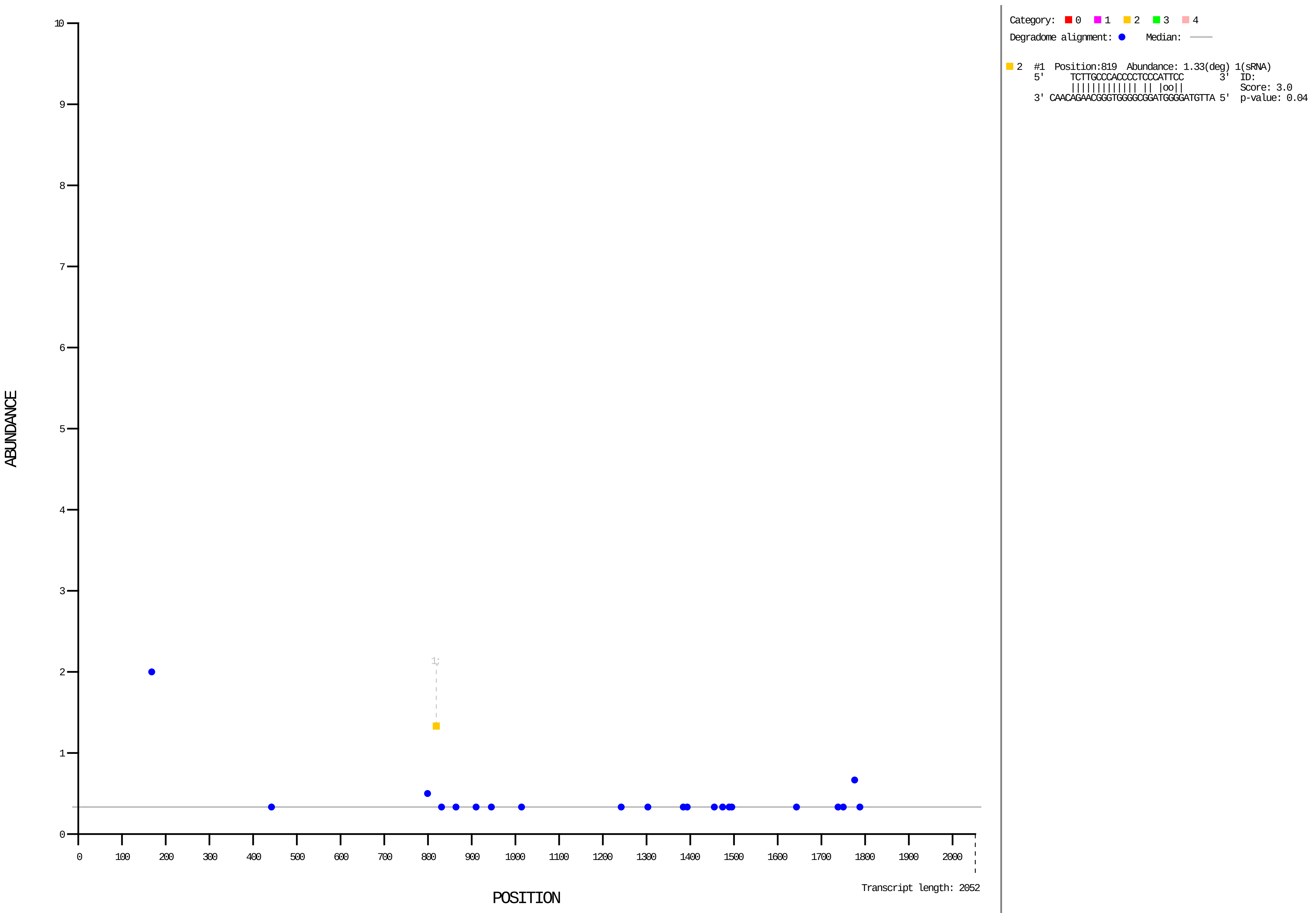

Cs5g22330.1 gene=Cs5g22330 CDS=411-1145

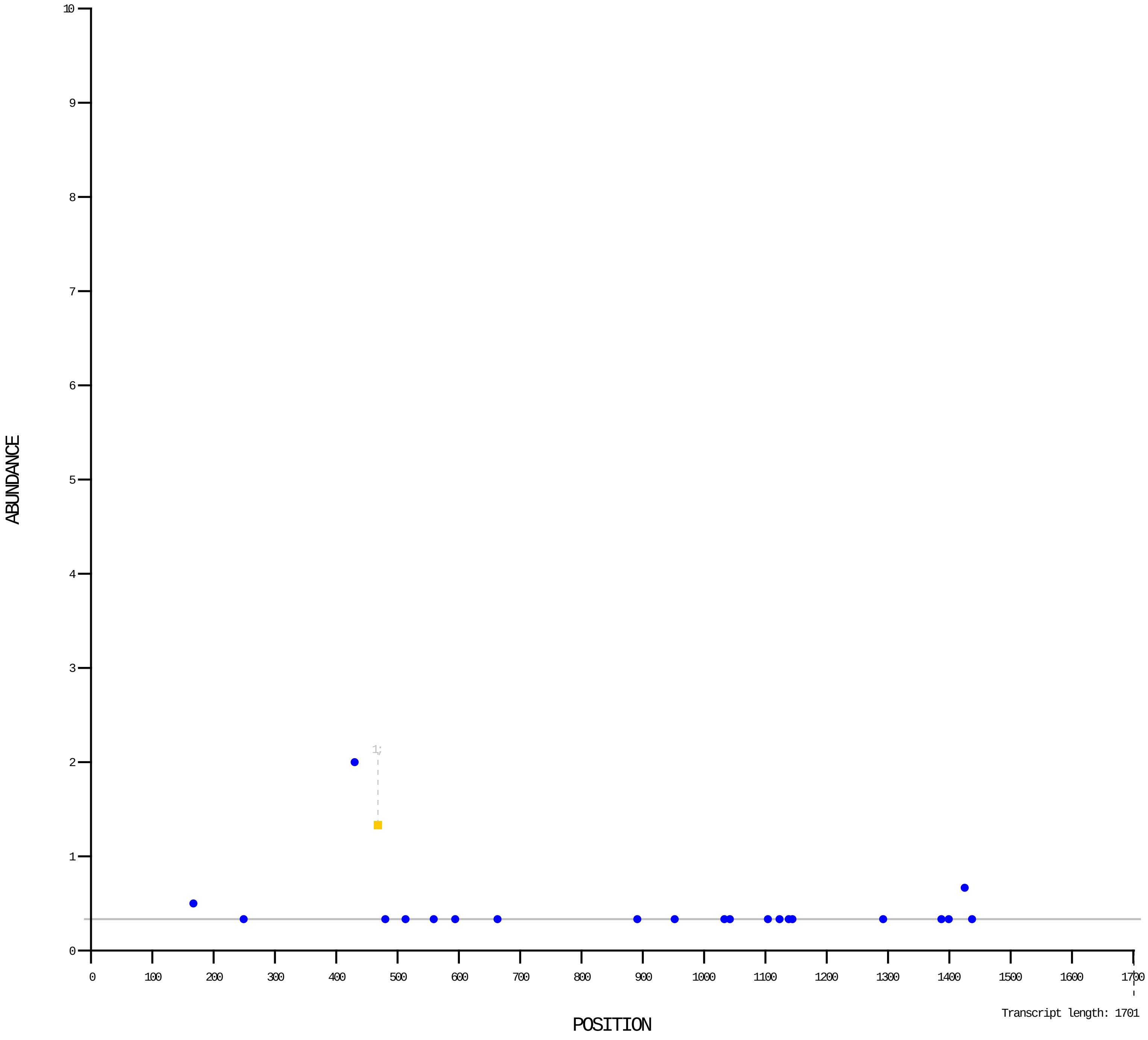

Category: 0 1 2 3 4
Degradome alignment: Median:

2 #1 Position:468 Abundance: 1.33(deg) 1(sRNA)

```
5'     TCTTGCCCACCCCTCCCATTCC      3'  ID:
       ||||||||||||| || |oo||          Score: 3.0
3' CAACAGAACGGGTGGGGCGGATGGGGACTTAA 5'  p-value: 0.04
```

orange1.1t01536.3 gene=orange1.1t01536 CDS=377-2329

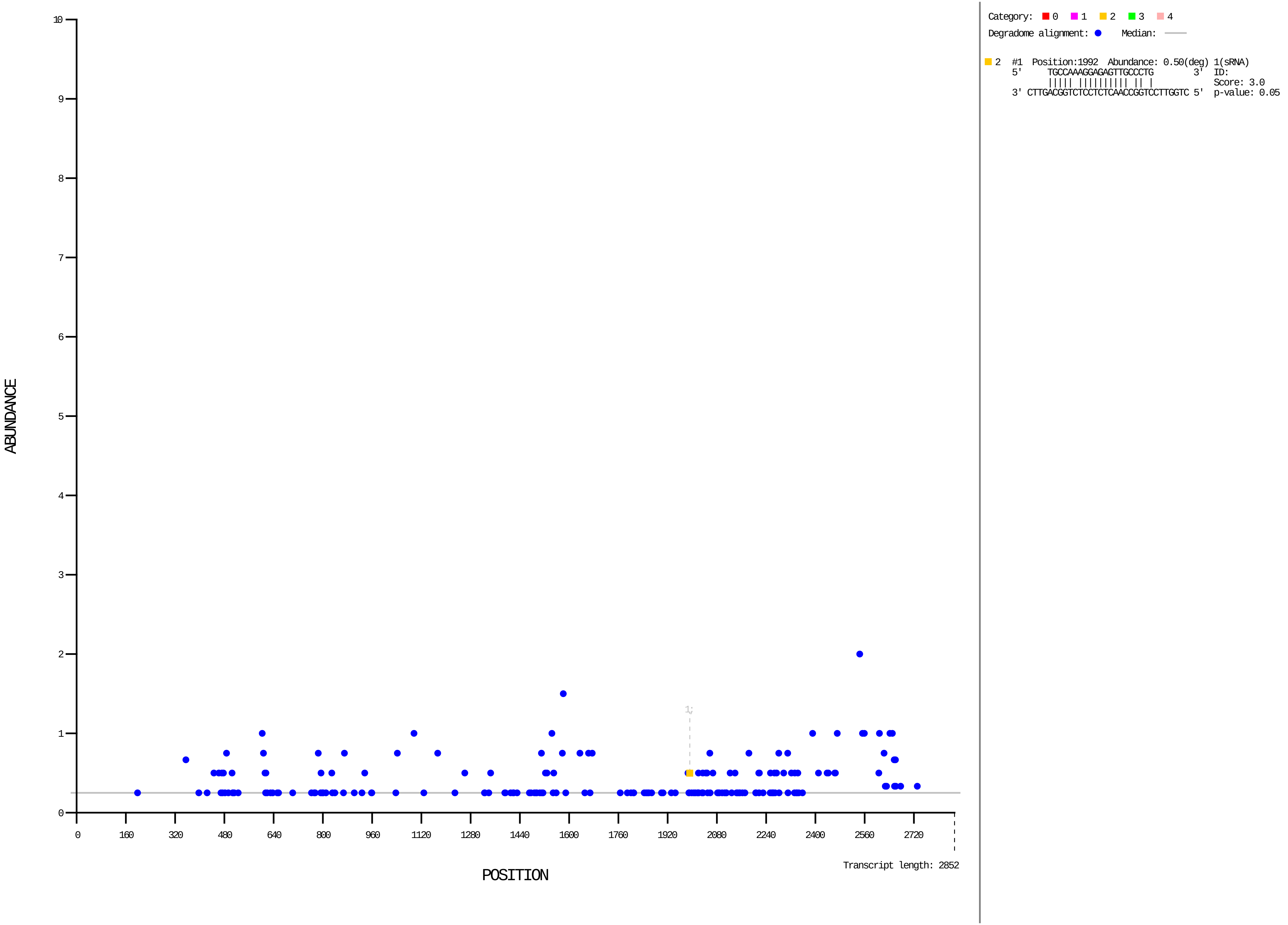

Cs9g09320.4 gene=Cs9g09320 CDS=367-2259

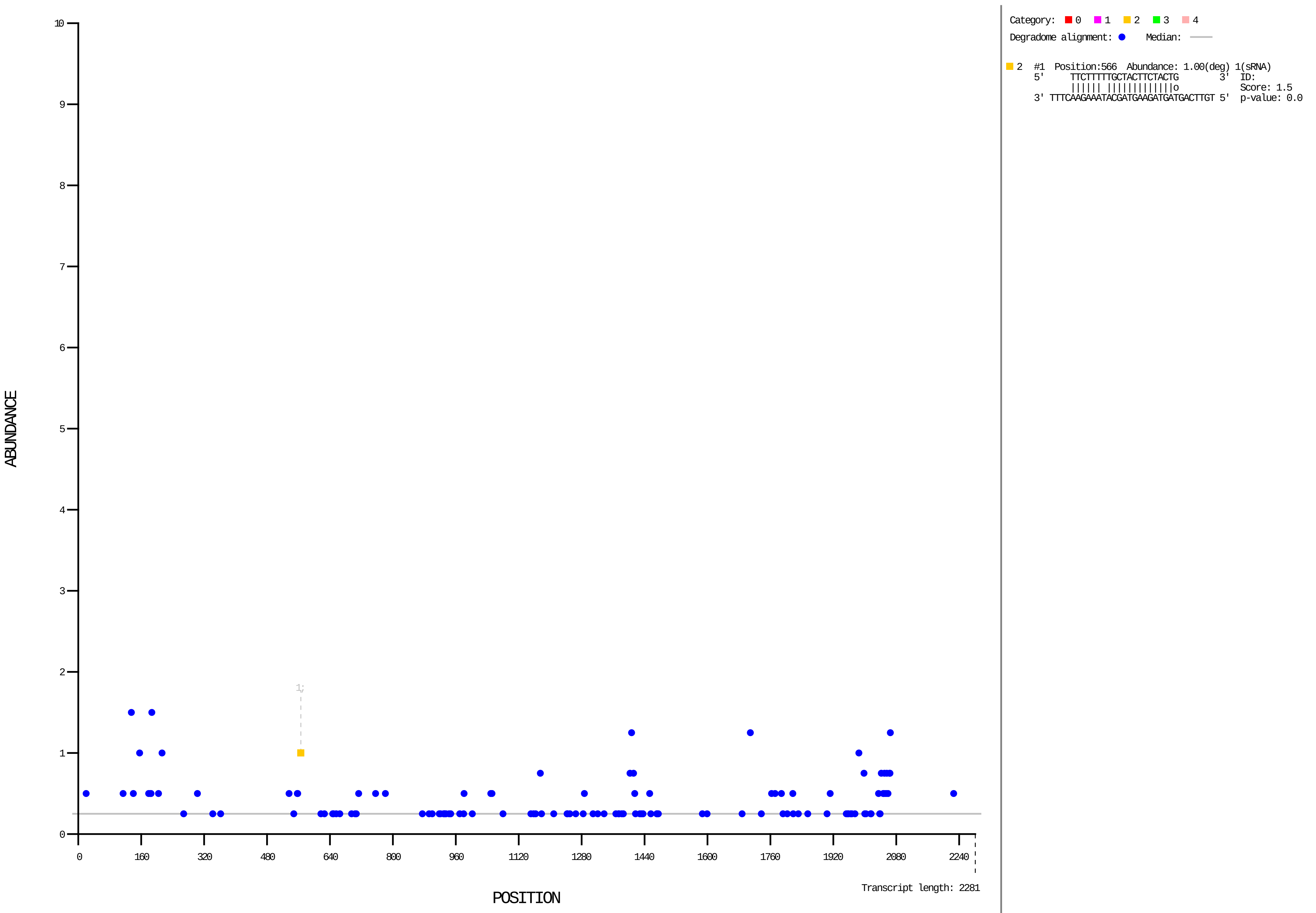

Cs9g09320.3 gene=Cs9g09320 CDS=328-2220

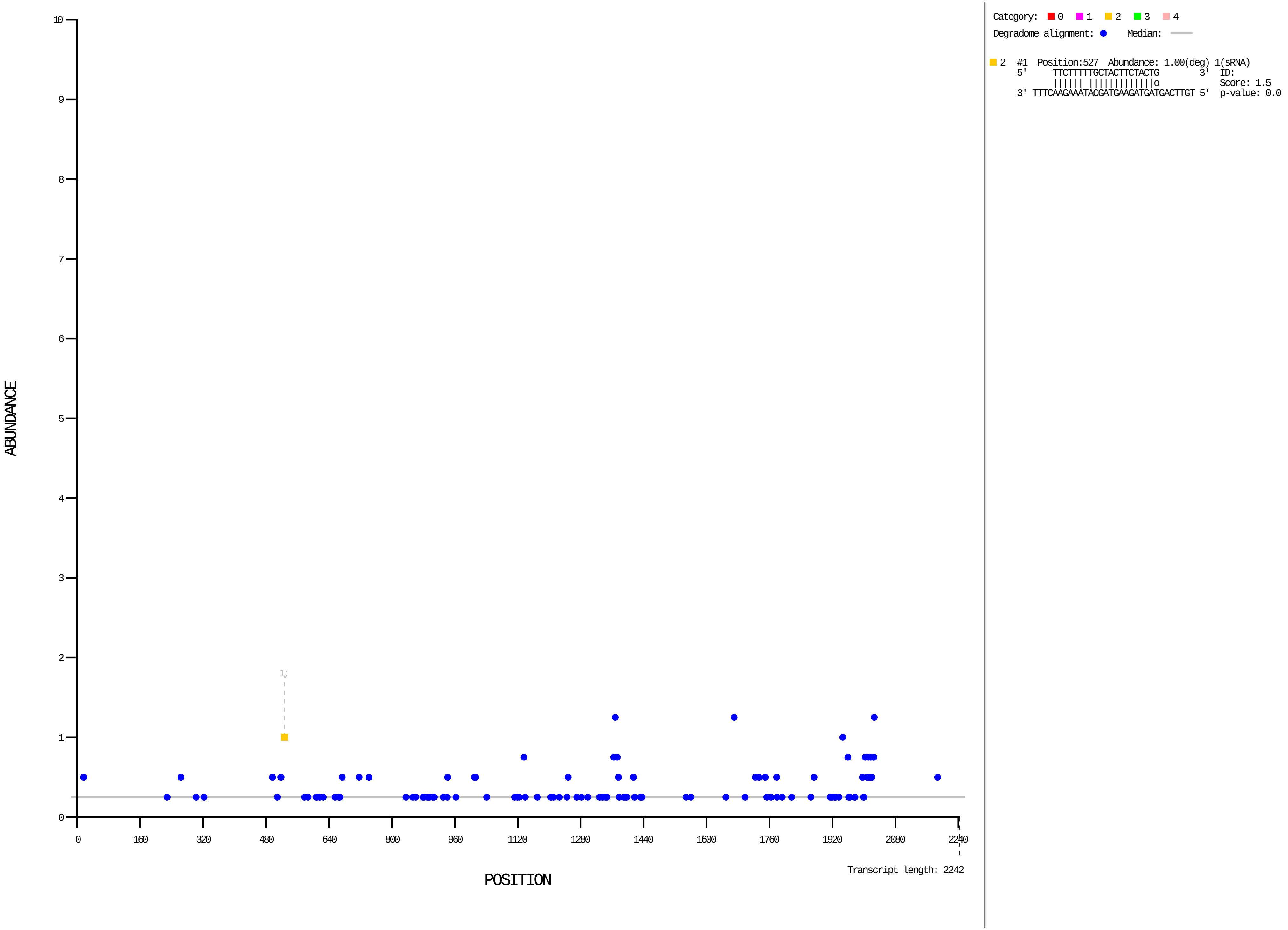

orange1.1t02132.3 gene=orange1.1t02132 CDS=877-4056

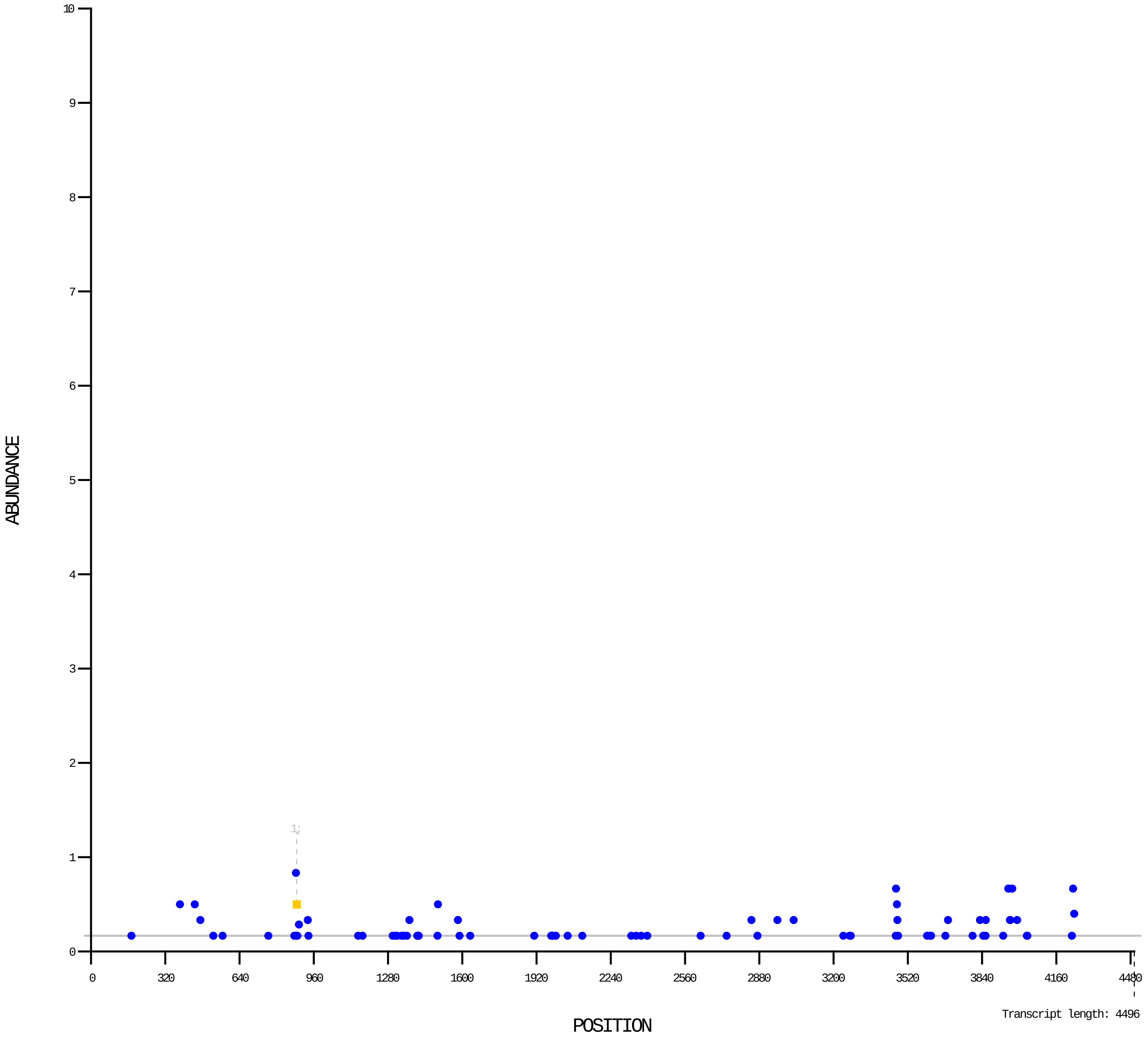

Category: 0 1 2 3 4

Degradome alignment: Median:

2 #1 Position:886 Abundance: 0.50(deg) 1(sRNA)

```
5'    TCTTACCTATGCCACCCATTCC       3'  ID:
      | || |||||o||||||||| ||          Score: 3.5
3' CAACAAAAGGGATATGGTGGGTACGGGGTTTA 5'  p-value: 0.05
```

Cs2g19590.1 gene=Cs2g19590 CDS=238-1779

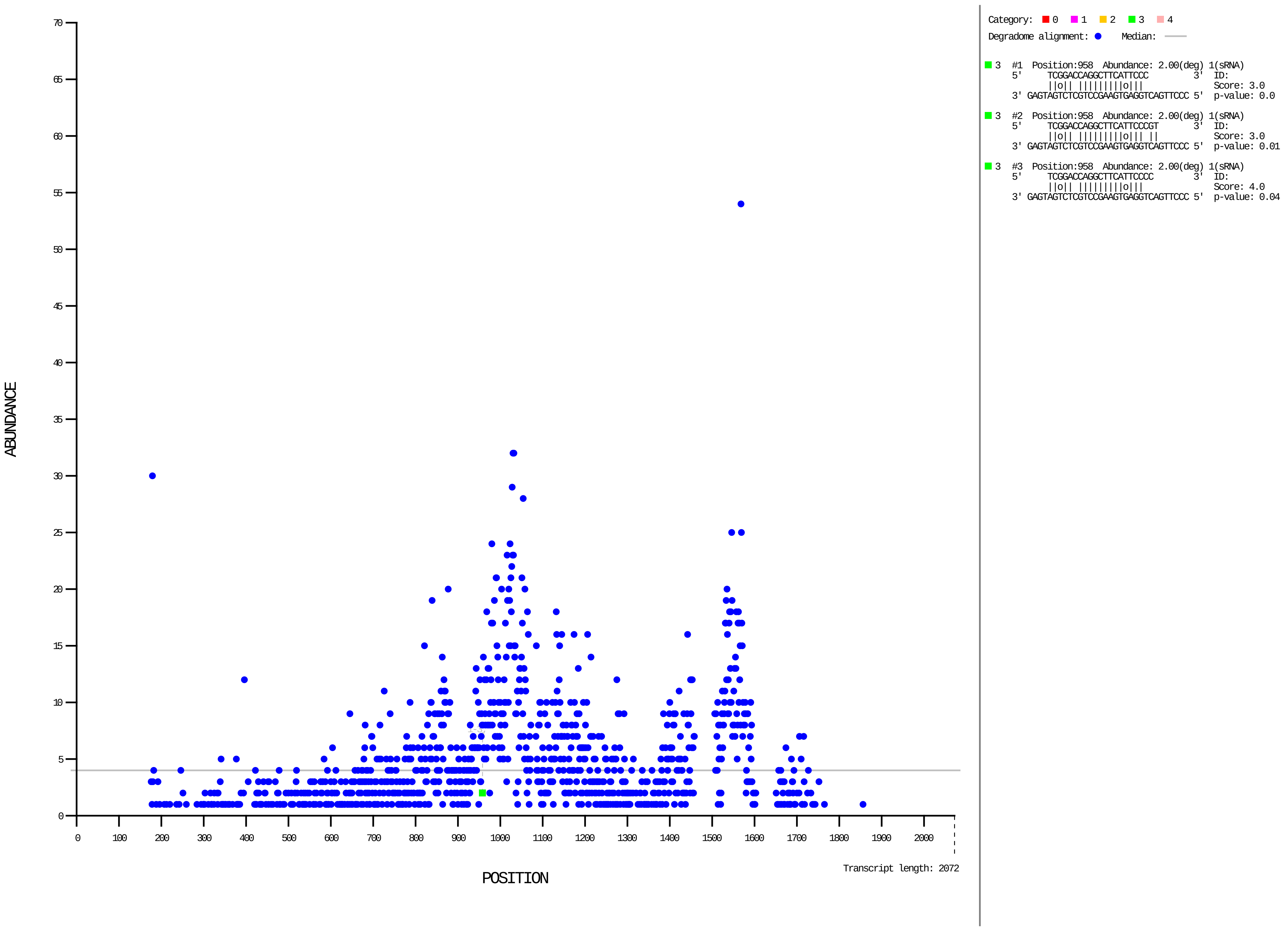

Cs4g11090.1 gene=Cs4g11090 CDS=103-1173

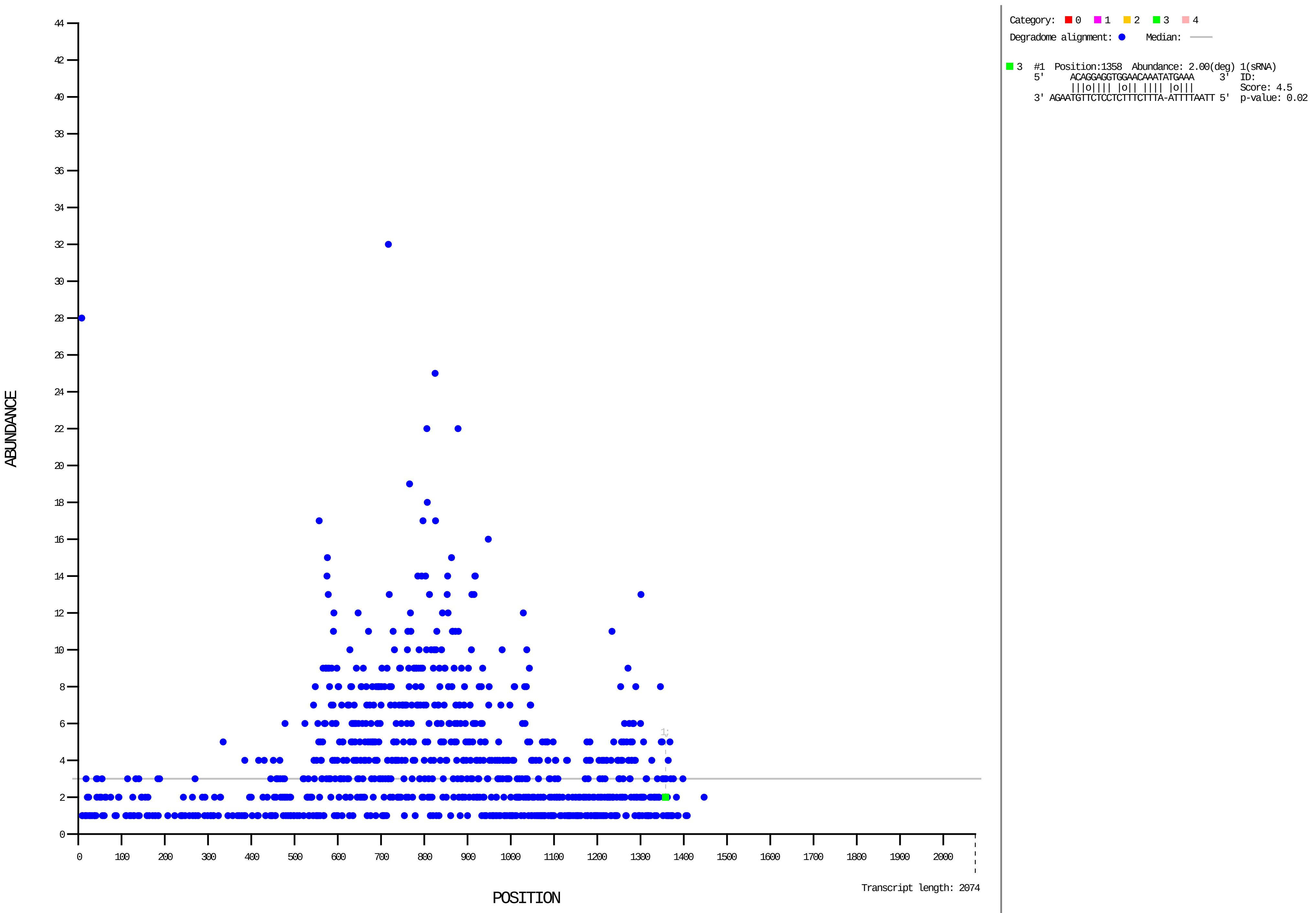

orange1.1t02219.2 gene=orange1.1t02219 CDS=328-1344

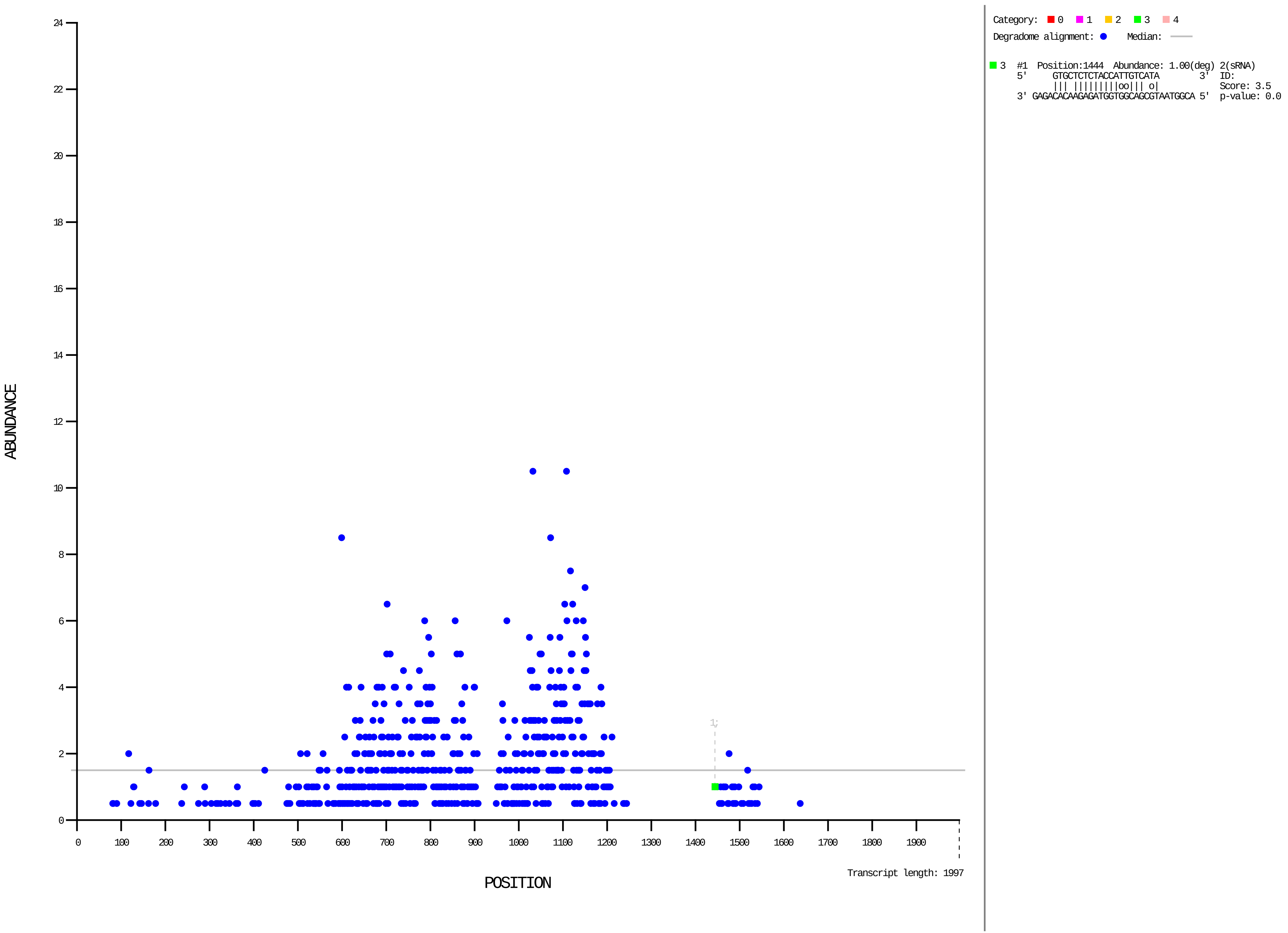

Cs3g06640.1 gene=Cs3g06640 CDS=1-4224

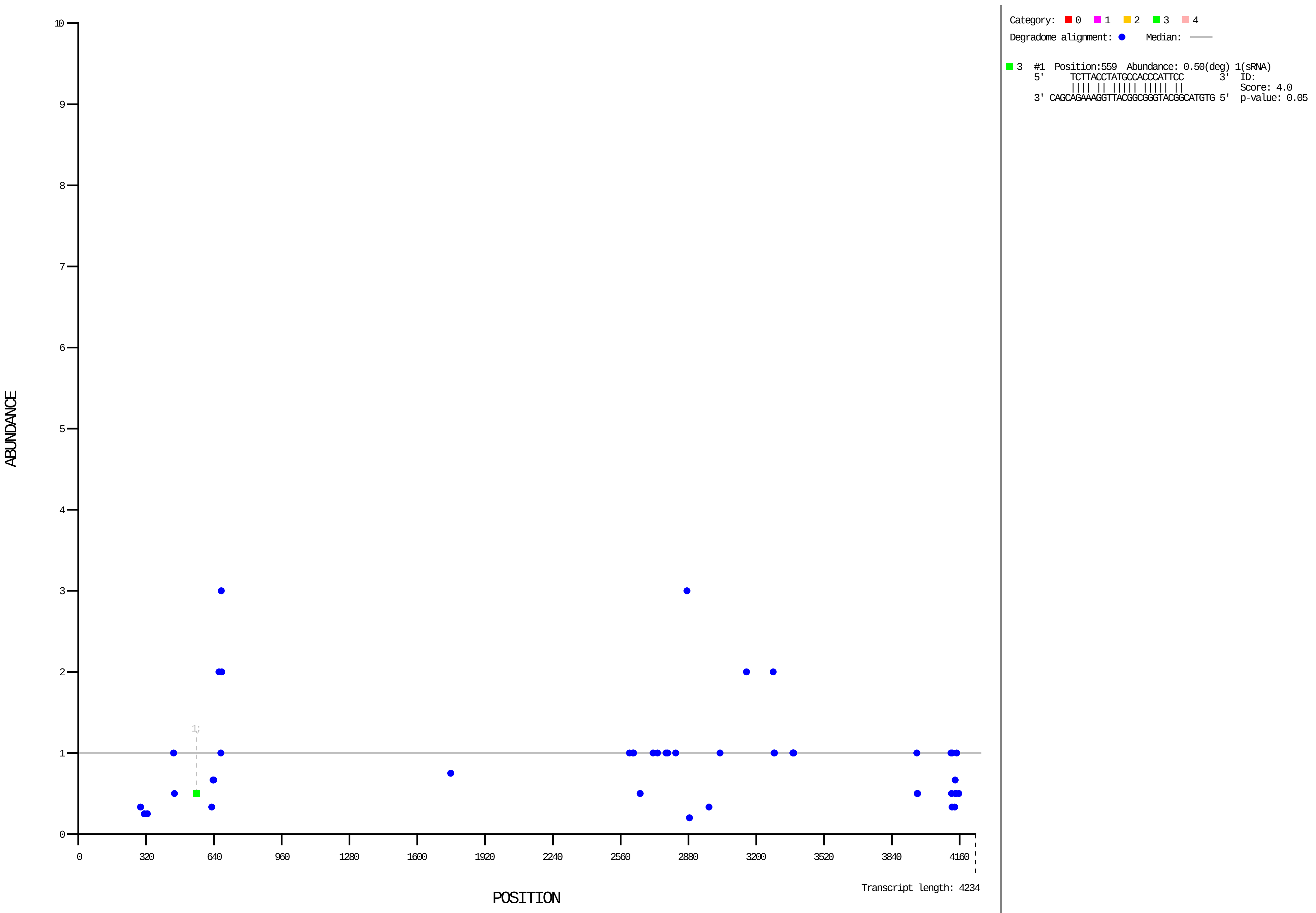

Cs7g22420.1 gene=Cs7g22420 CDS=222-1310

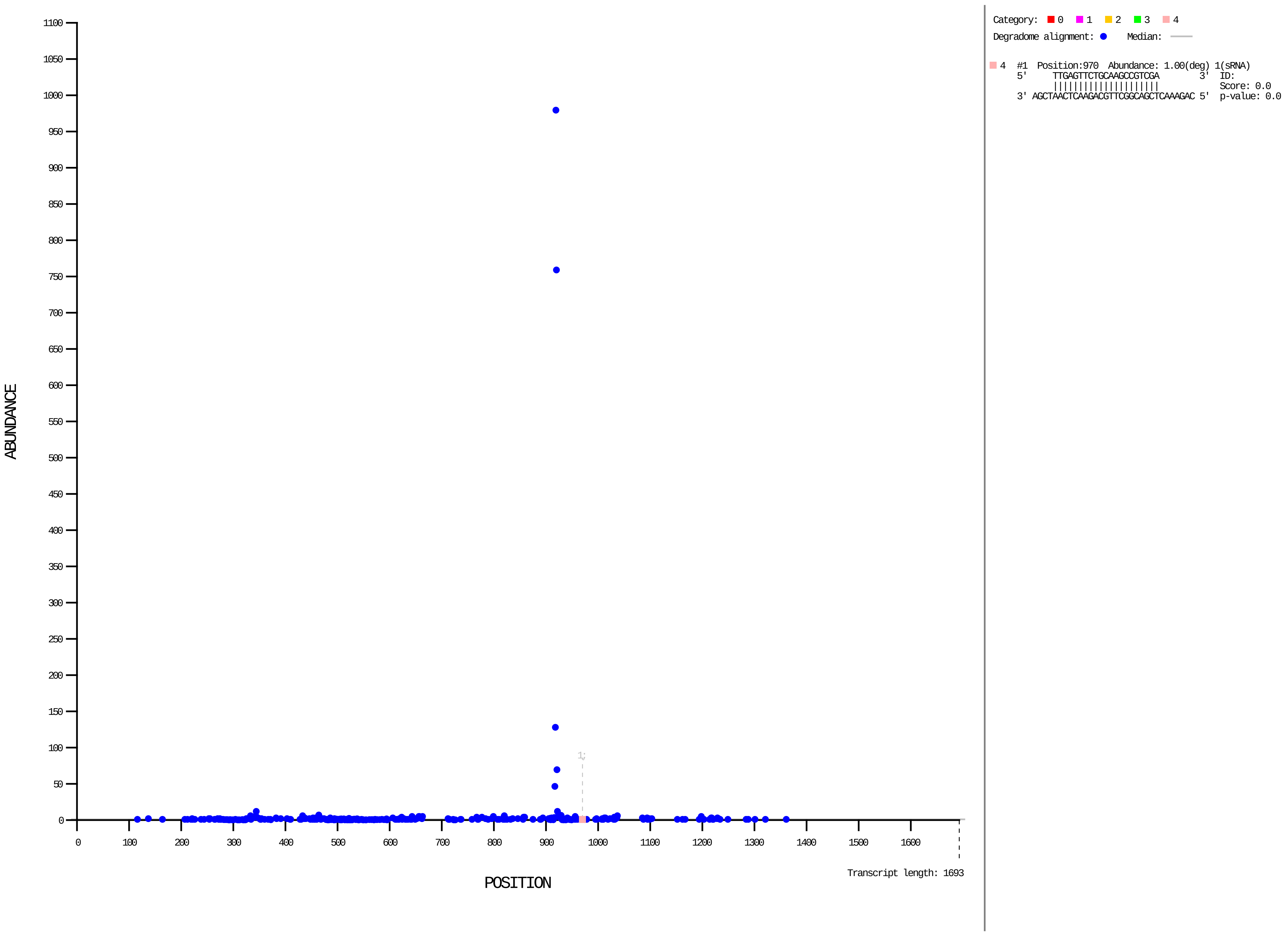

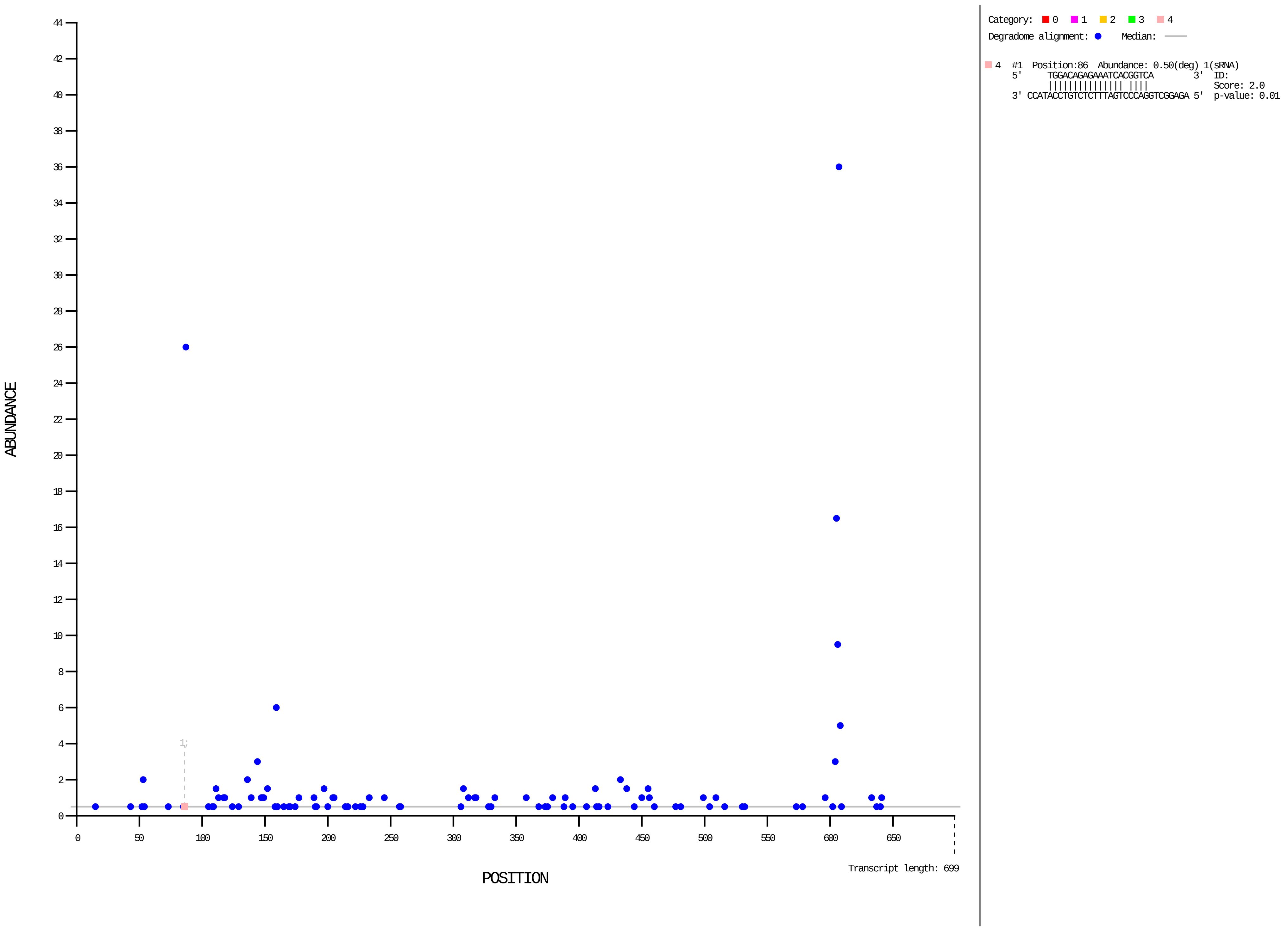
orange1.1t05093.1 gene=orange1.1t05093 CDS=1-699
ABUNDANCE
POSITION
Transcript length: 699
Category: 0 1 2 3 4
Degradome alignment:
Median:
4 #1 Position:86 Abundance: 0.50(deg) 1(sRNA)
5' TGGACAGAGAAATCACGGTCA 3' ID:
Score: 2.0
3' CCATACCTGTCTCTTTAGTCCCAGGTCGGAGA 5' p-value: 0.01

Cs4g18660.1 gene=Cs4g18660 CDS=335-1384

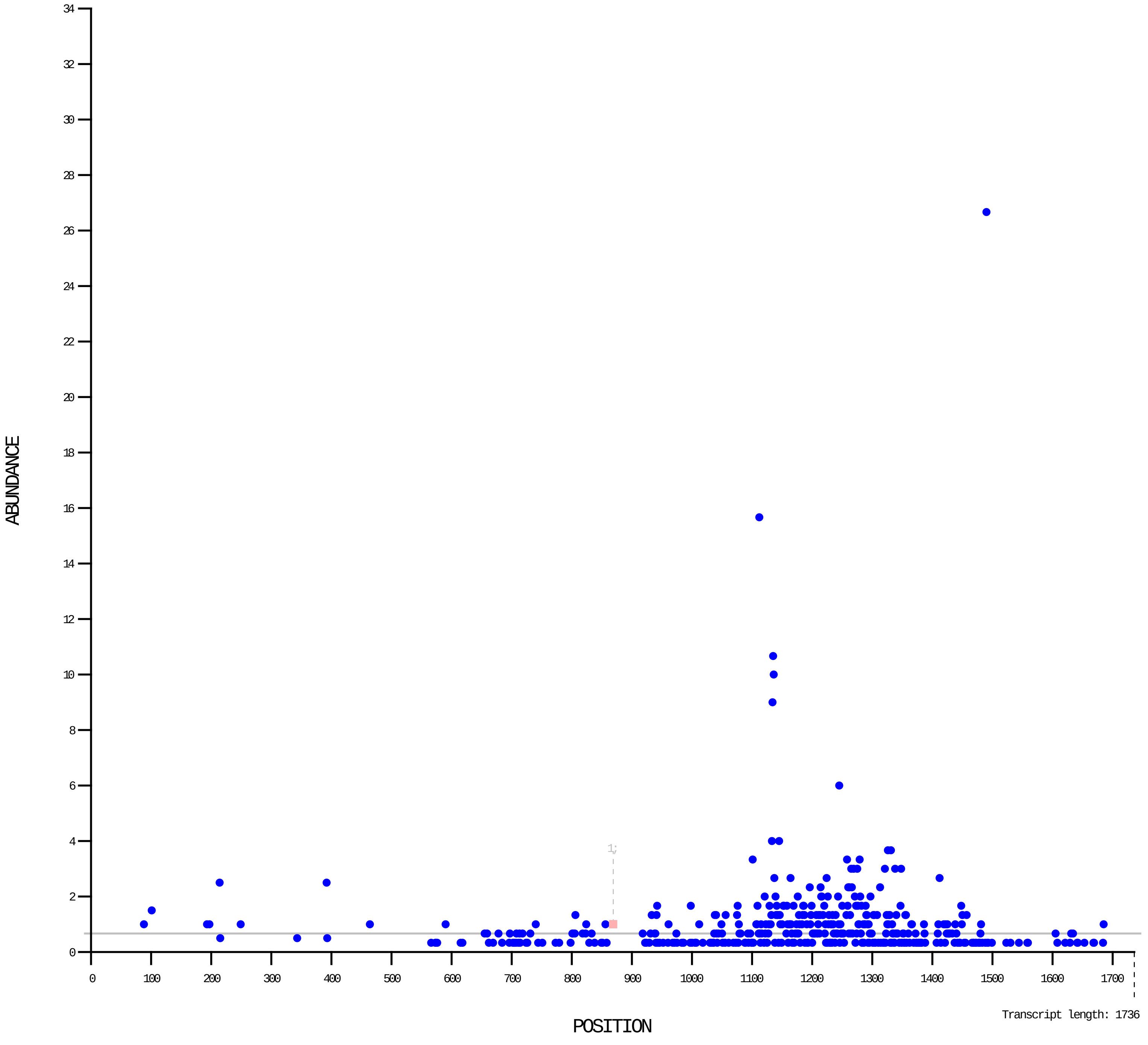

Category: 0 1 2 3 4

Degradome alignment: ● Median: —

```
4  #1  Position:869  Abundance: 1.00(deg) 1(sRNA)
   5'     TCCCTACTCCACCCATGCCATA       3'  ID:
          || |o||o|||| |||||||||           Score: 3.0
   3' AGGAAGTGGTGGGGTGTGTACGGTATCATCAT 5'  p-value: 0.02
```

Cs7g01530.1 gene=Cs7g01530 CDS=802-1908

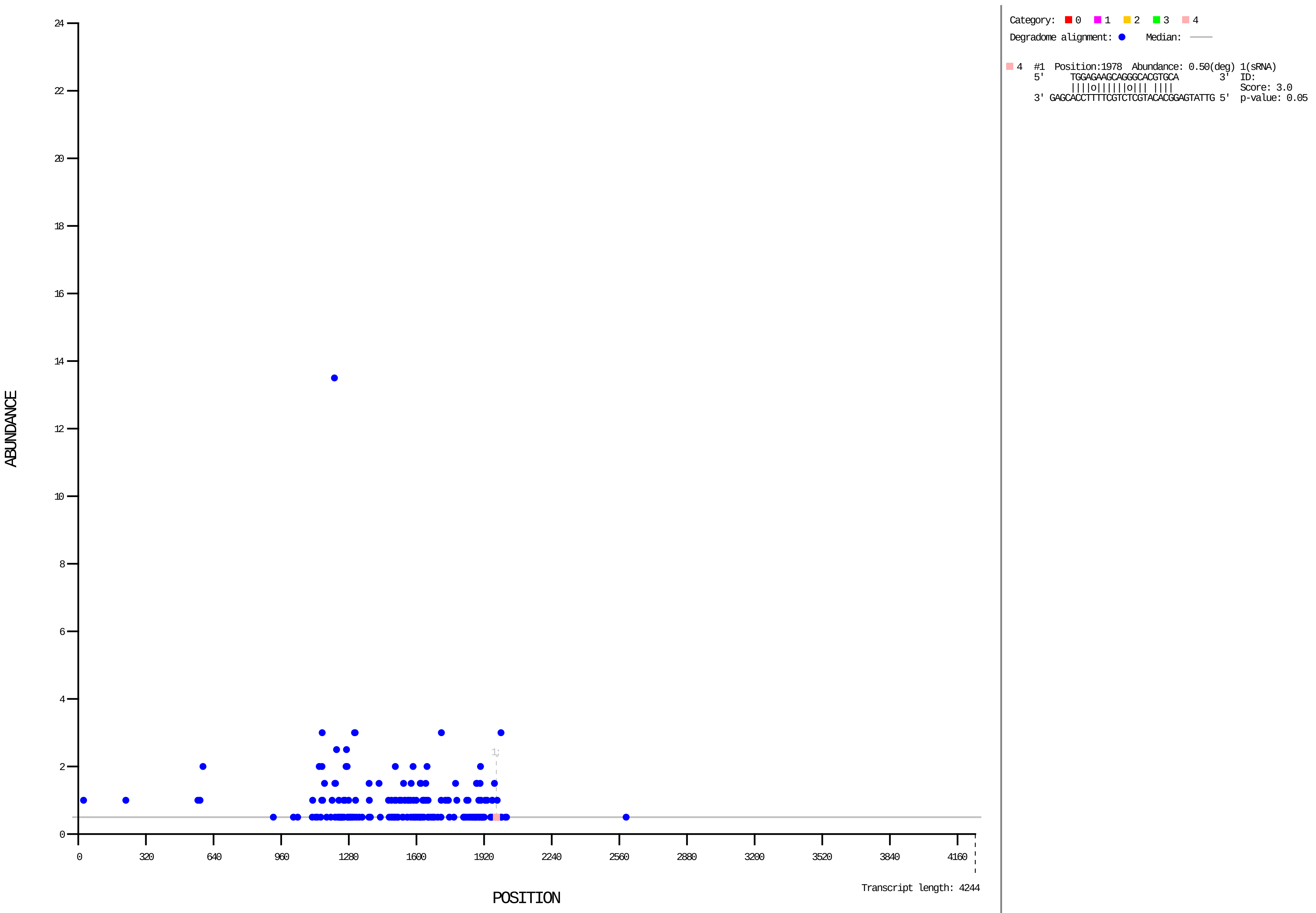

Cs4g04300.2 gene=Cs4g04300 CDS=220-3438

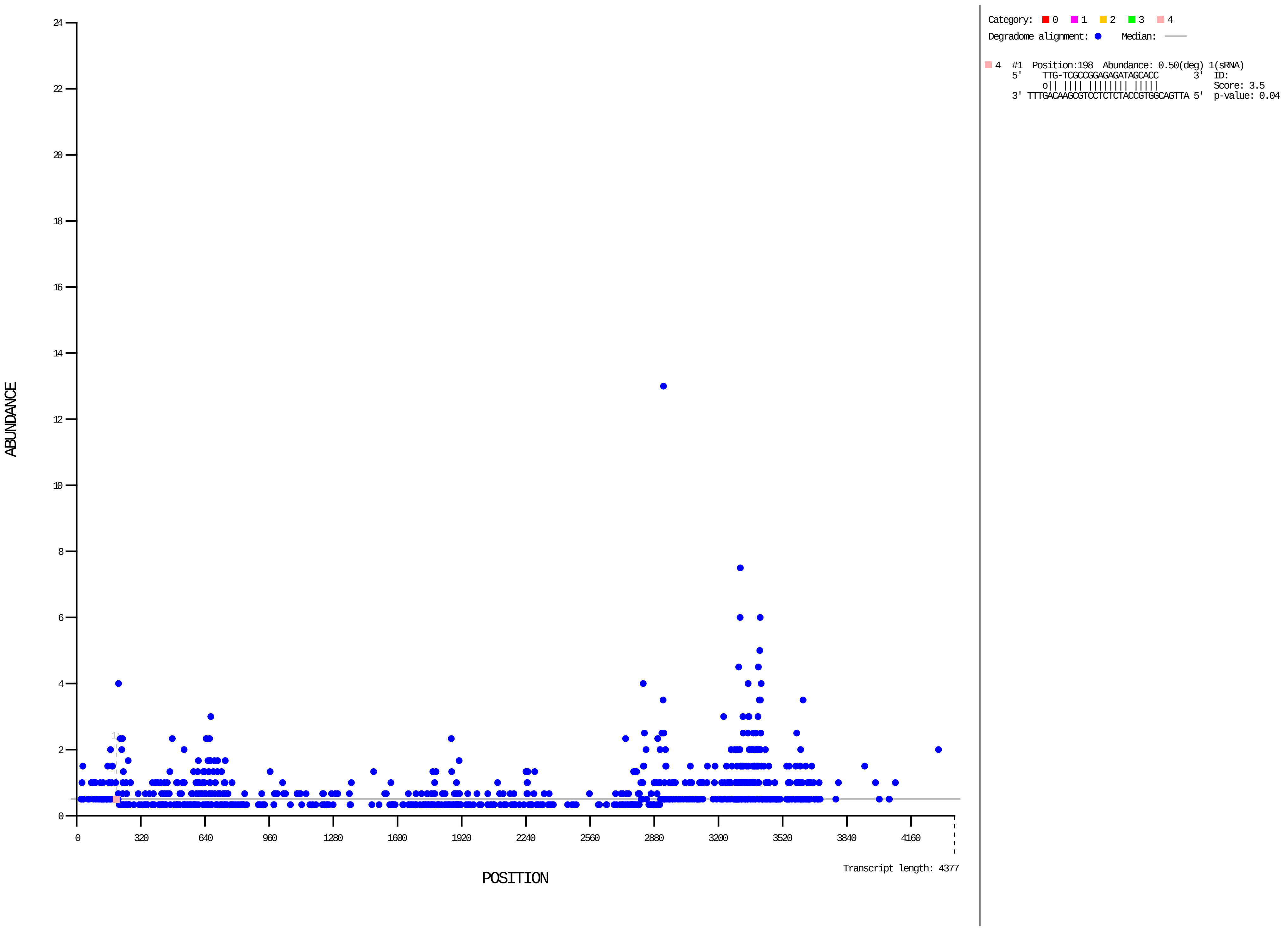

Cs9g05070.2 gene=Cs9g05070 CDS=367-1641

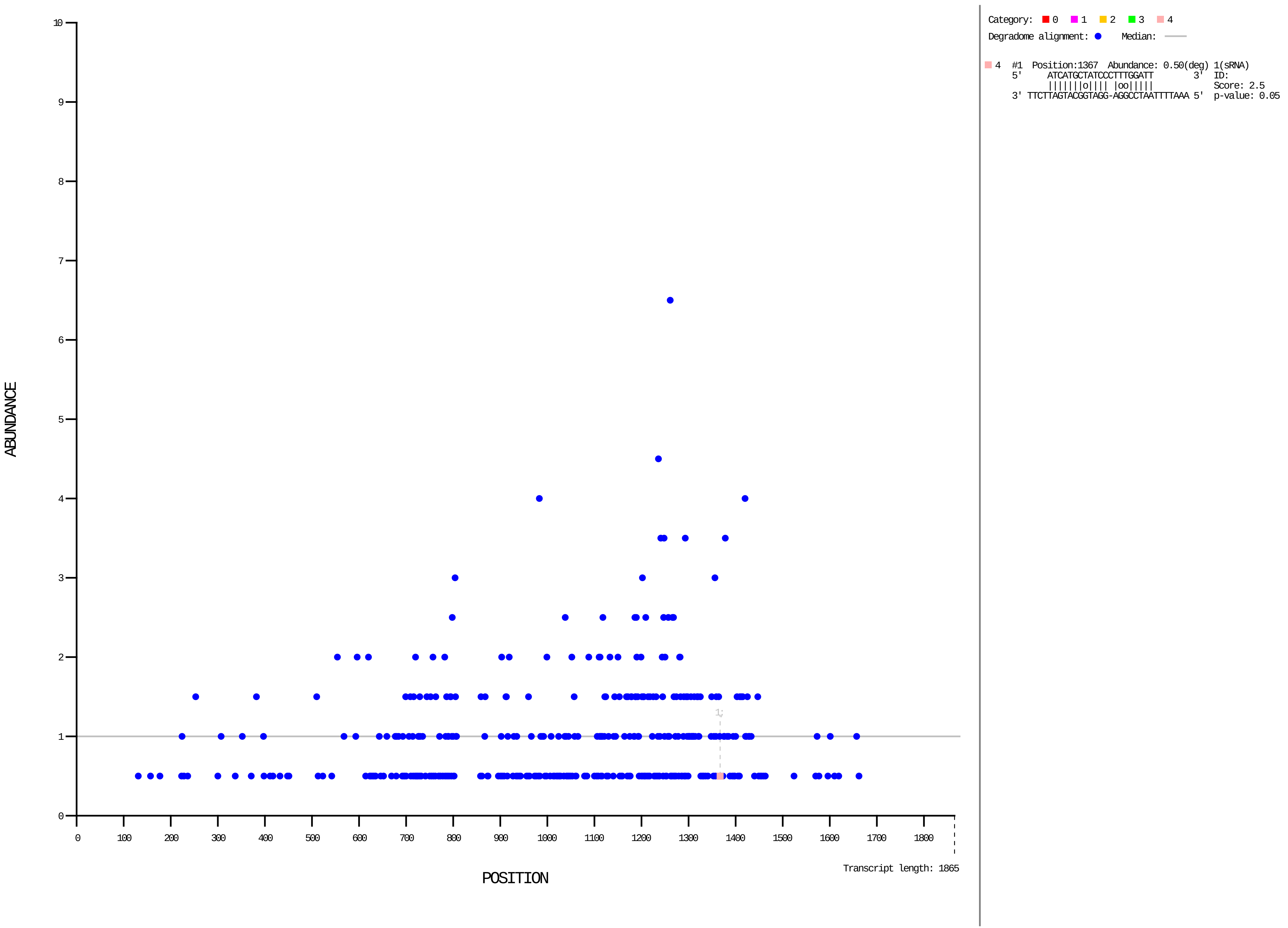

Cs8g12370.1 gene=Cs8g12370 CDS=14-2044

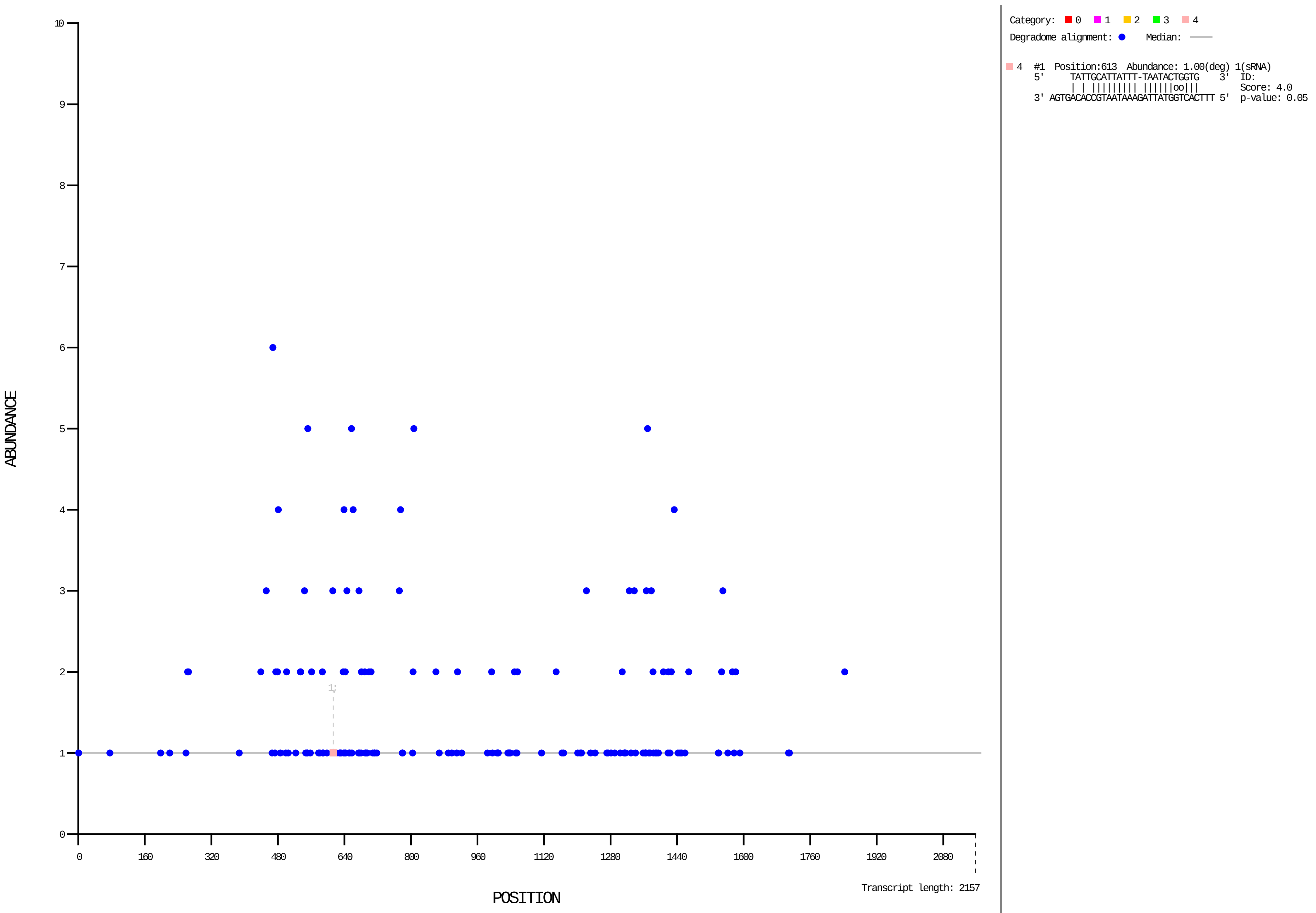

Cs6g14050.2 gene=Cs6g14050 CDS=123-1097

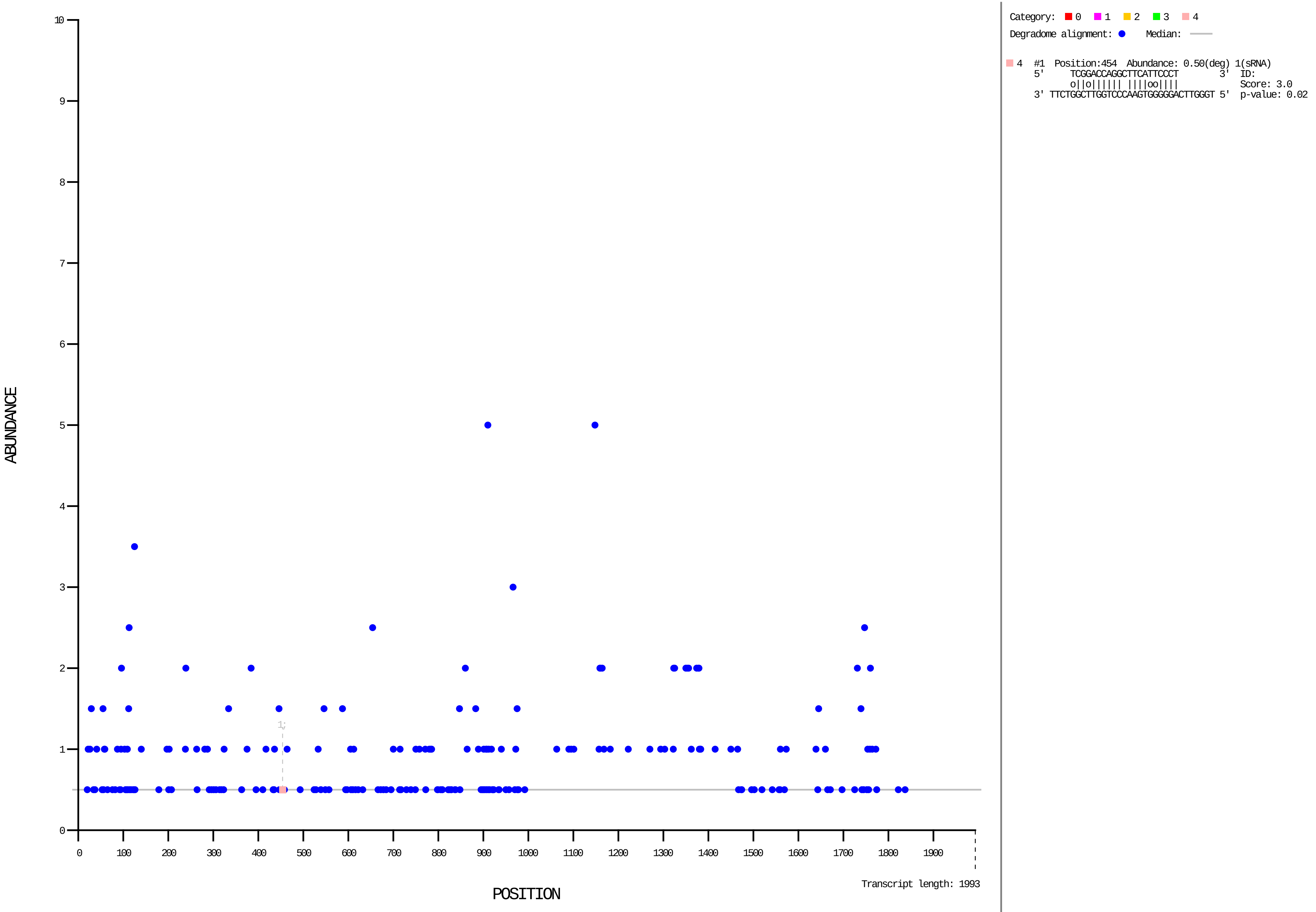

Cs5g19440.1 gene=Cs5g19440 CDS=1-2436

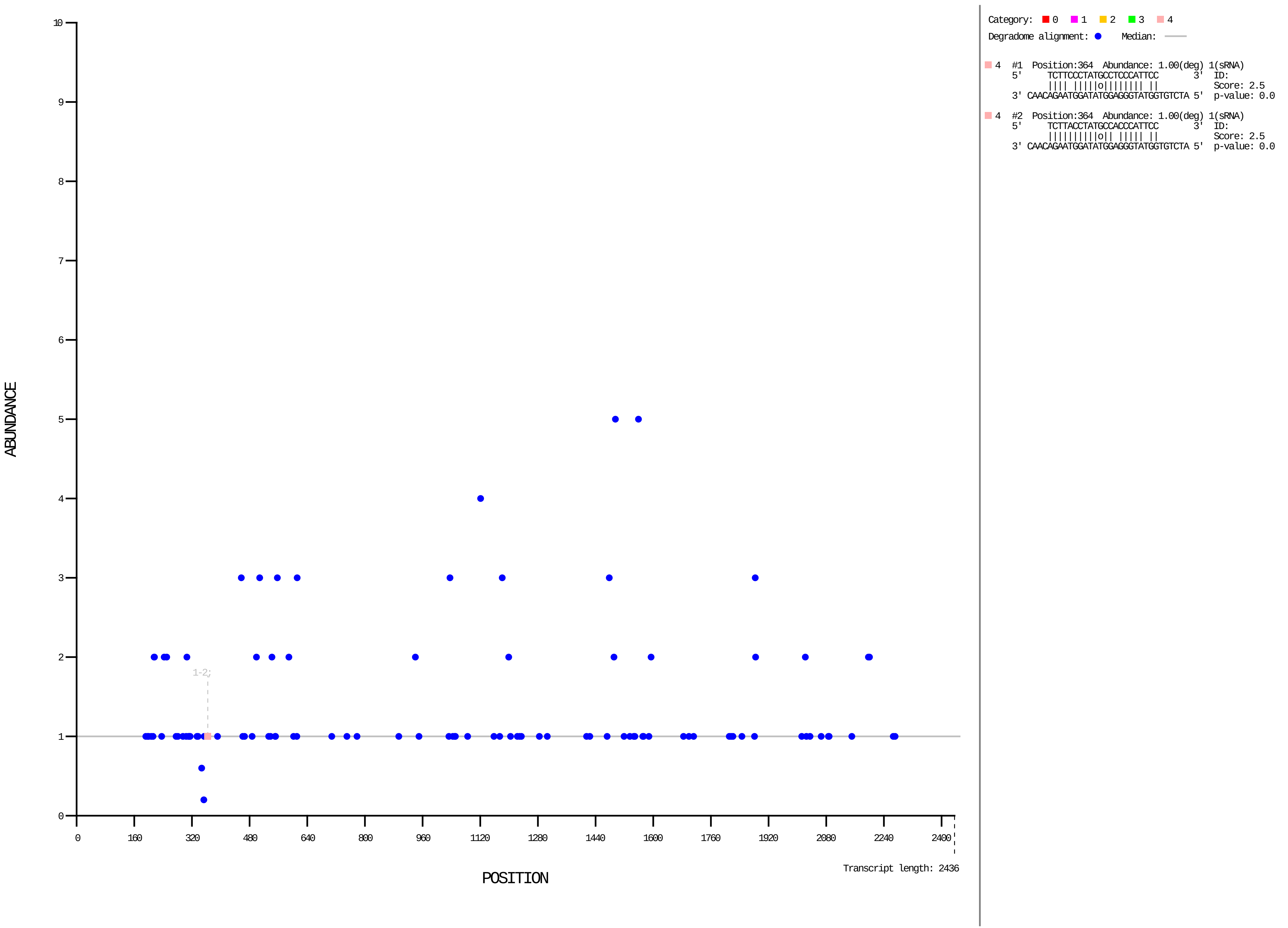

Cs8g18800.1 gene=Cs8g18800 CDS=1-1752

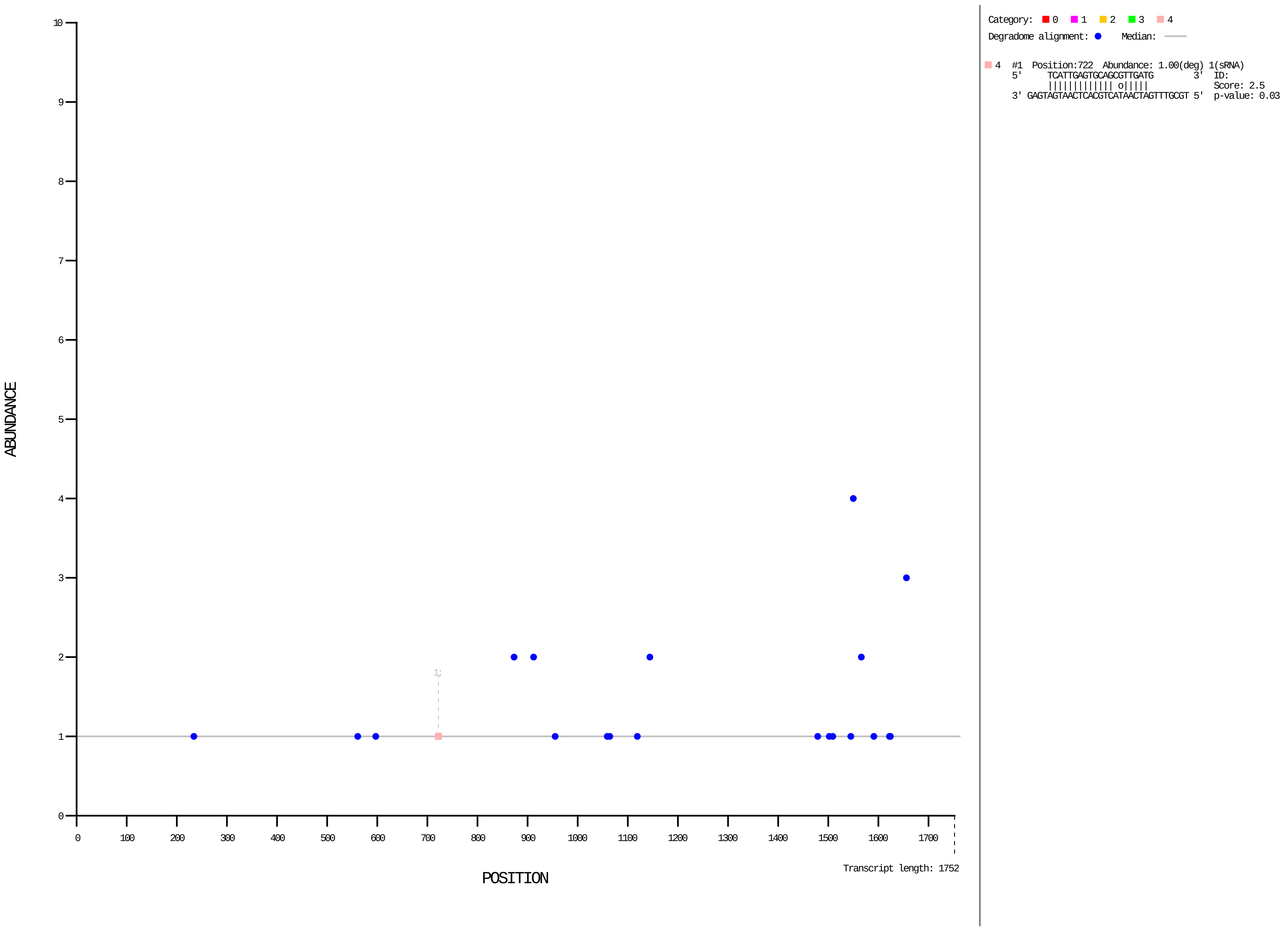

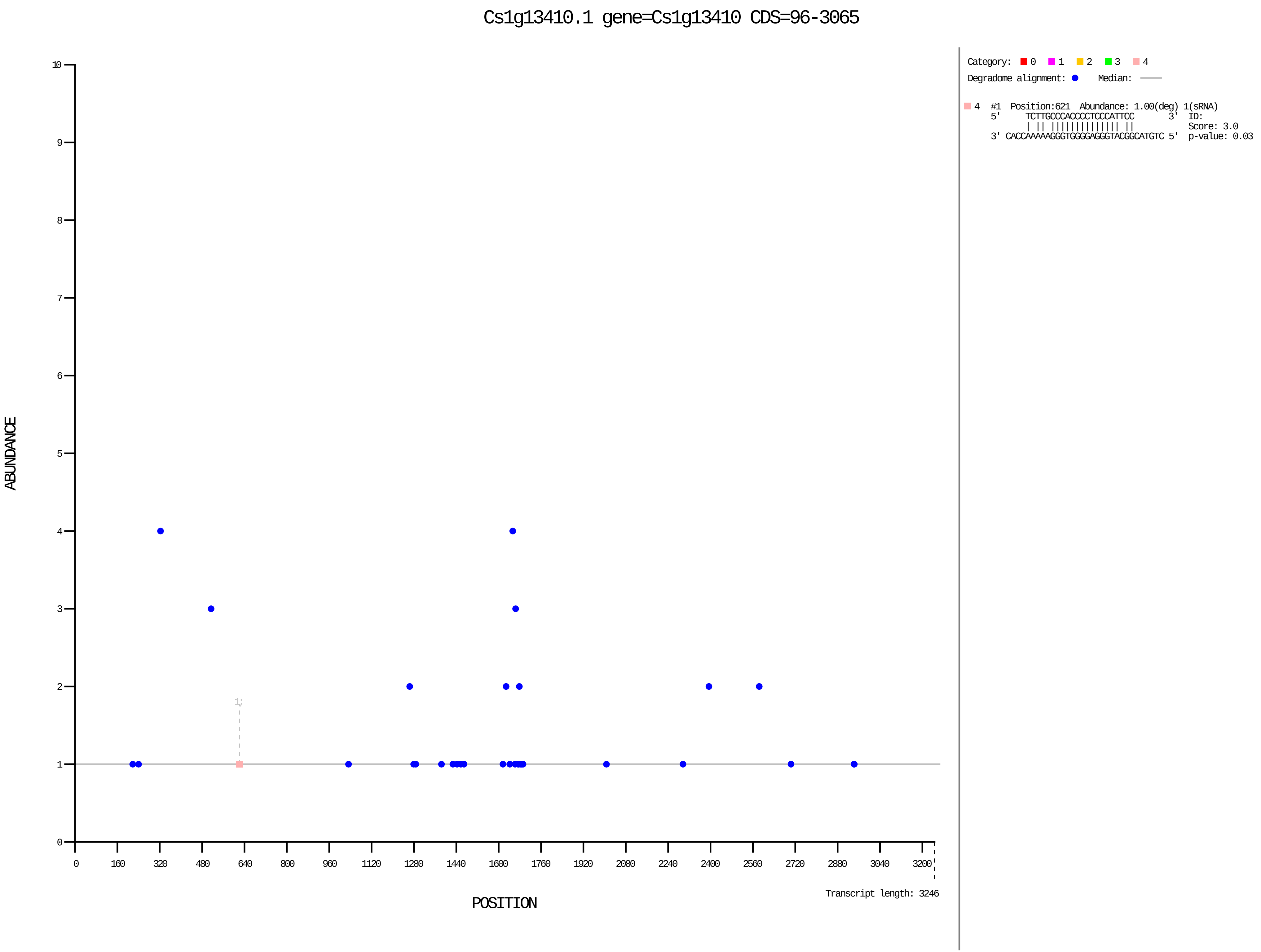
Cs1g13410.1 gene=Cs1g13410 CDS=96-3065
ABUNDANCE
POSITION
Transcript length: 3246
Category: 0 1 2 3 4
Degradome alignment:
Median:
4 #1 Position:621 Abundance: 1.00(deg) 1(sRNA)
5' TCTTGCCCACCCCTCCCATTCC 3' ID:
Score: 3.0
3' CACCAAAAAGGGTGGGGAGGGTACGGCATGTC 5' p-value: 0.03

Cs1g13480.1 gene=Cs1g13480 CDS=1-2559

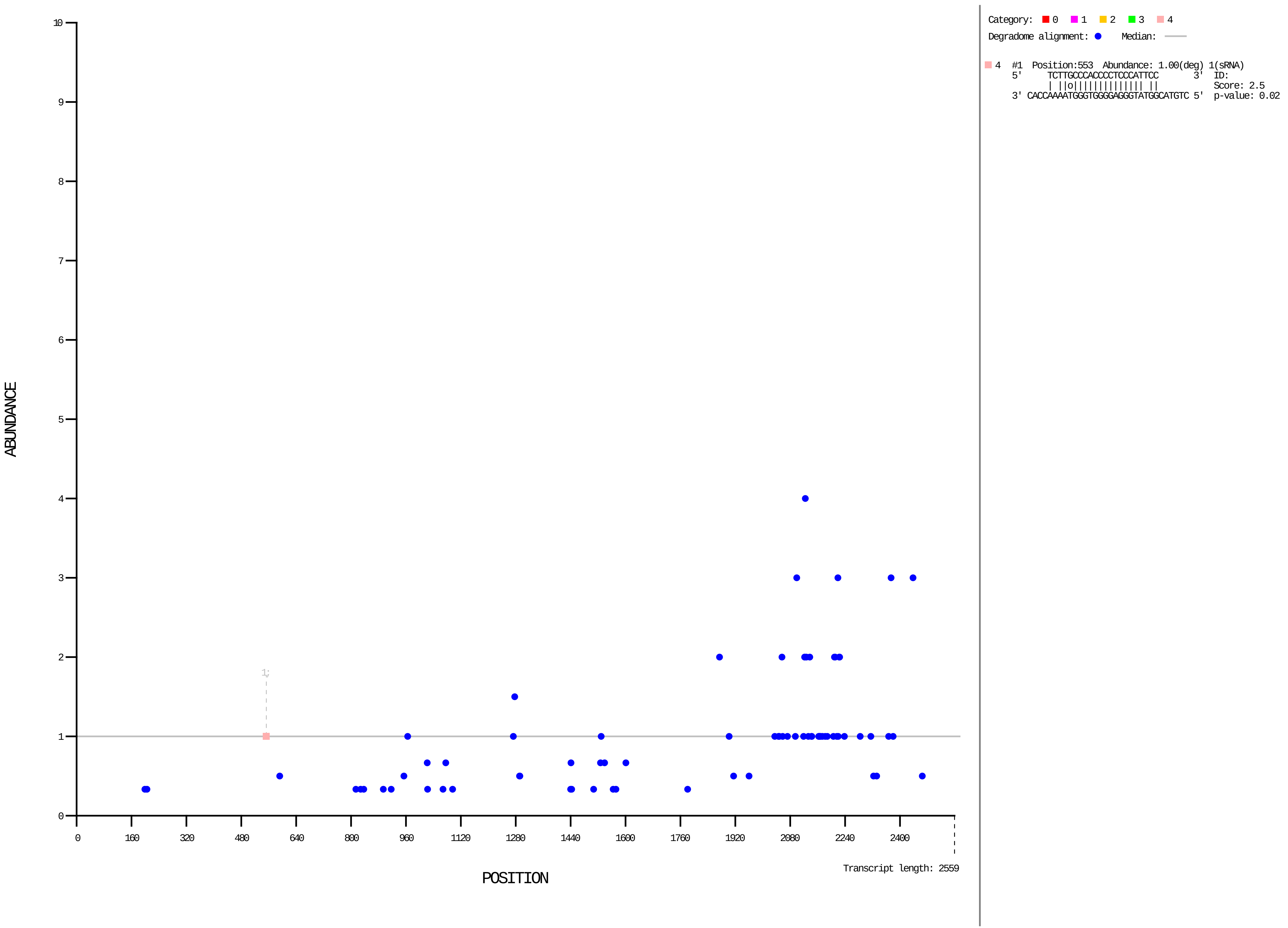

Cs4g04300.3 gene=Cs4g04300 CDS=201-2867

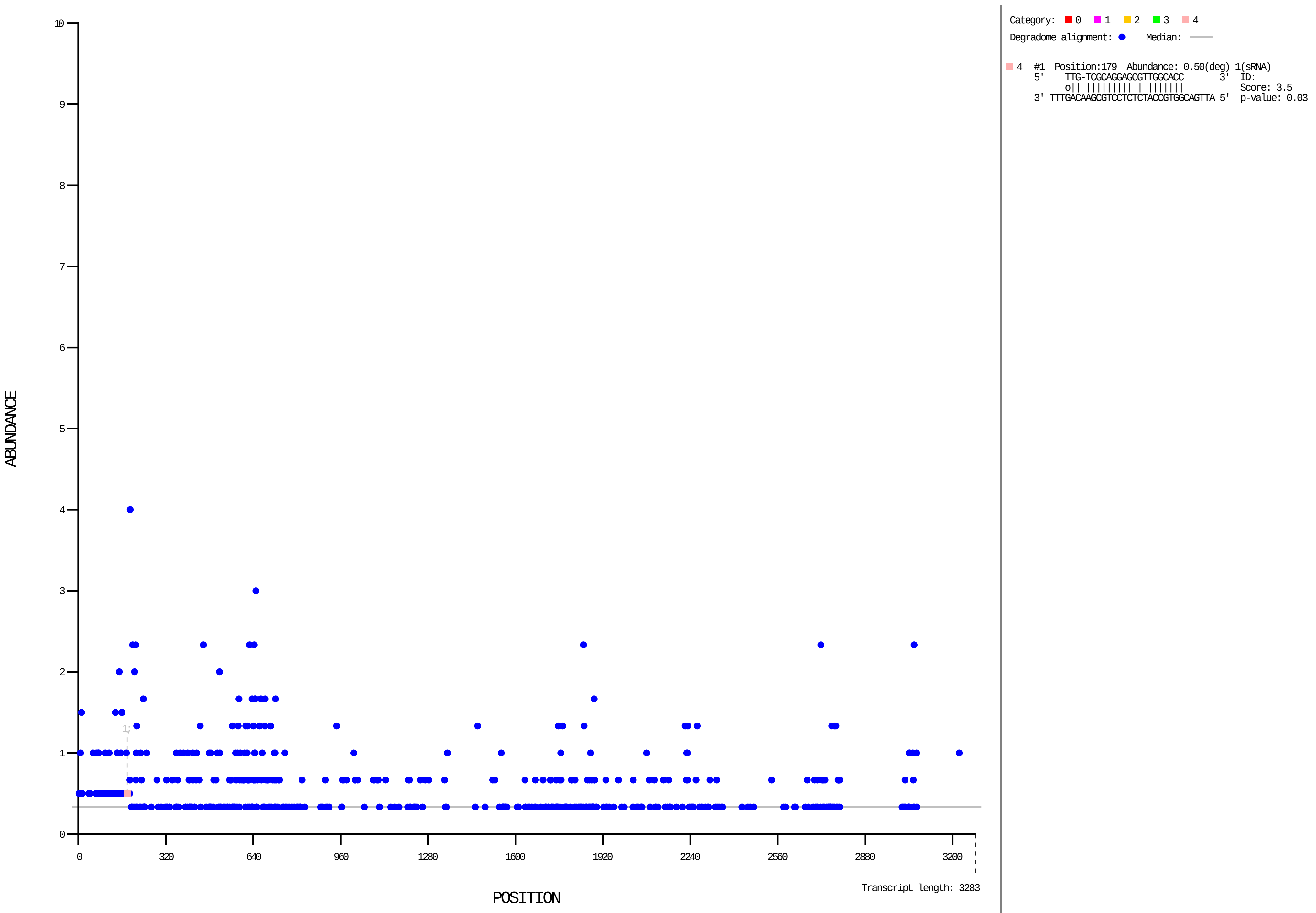

Cs1g01180.1 gene=Cs1g01180 CDS=1-3129

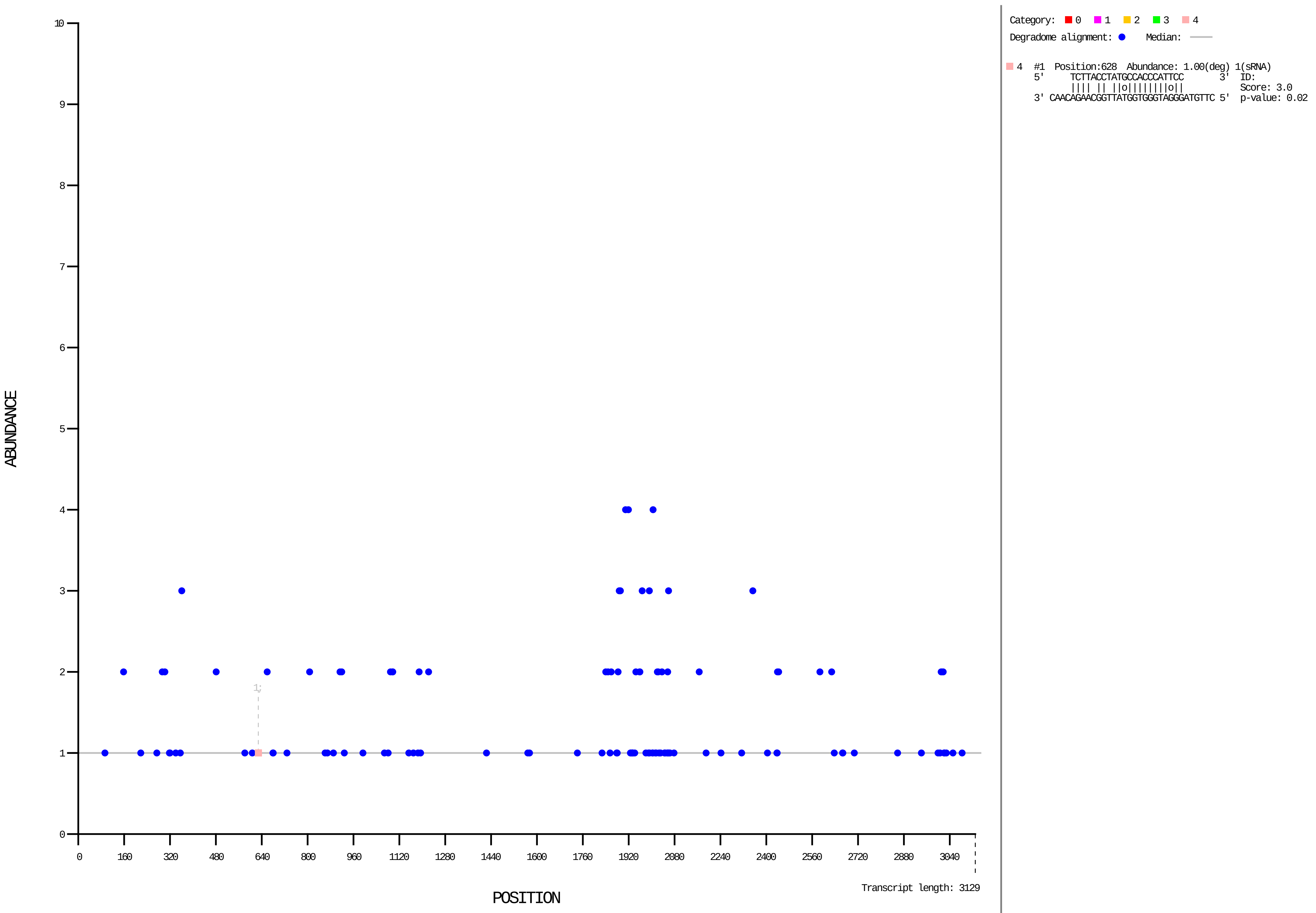

Cs1g03150.1 gene=Cs1g03150 CDS=1-528

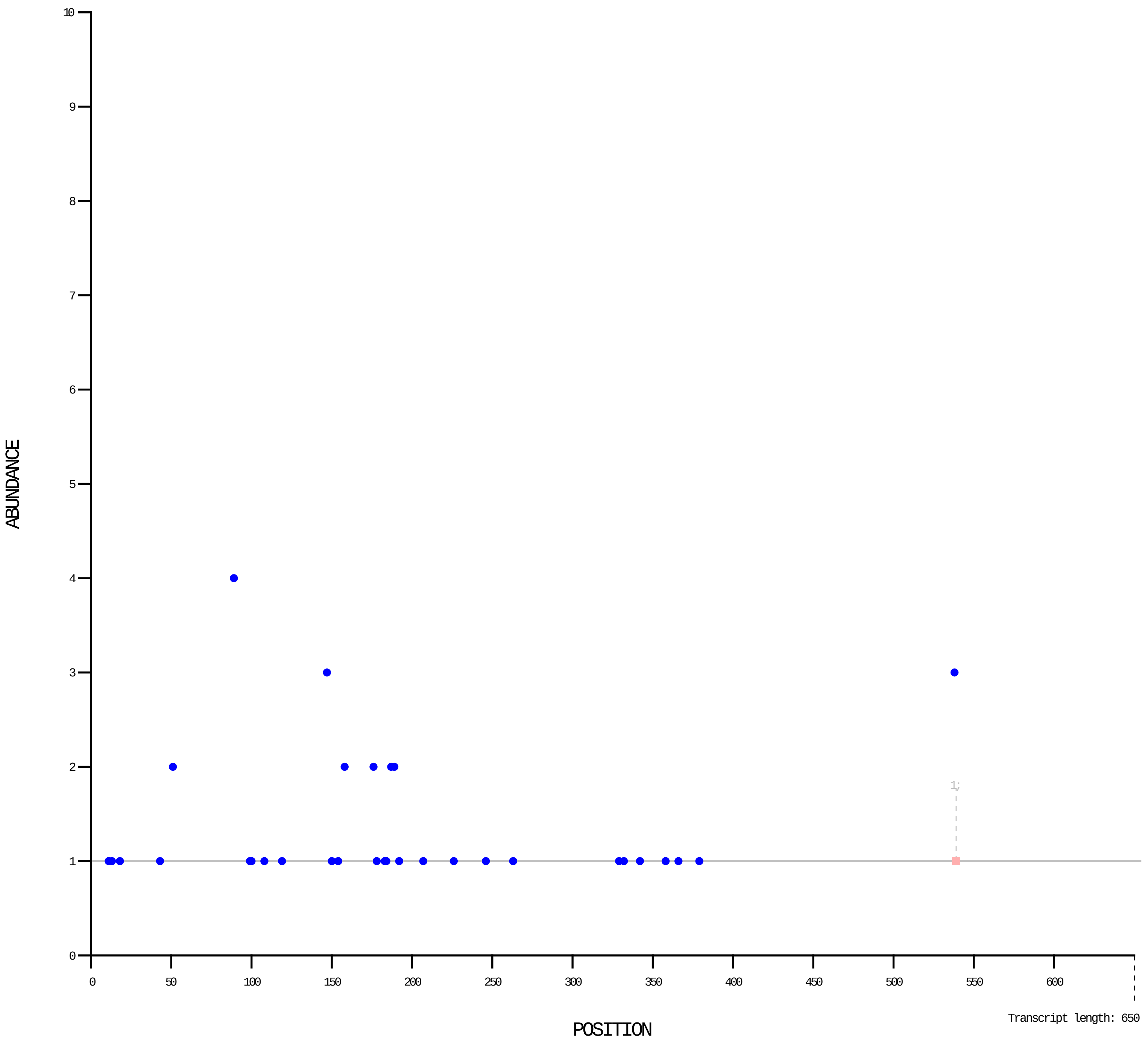

Category: 0 1 2 3 4
Degradome alignment: Median:

4 #1 Position:539 Abundance: 1.00(deg) 1(sRNA)

```
5'     ATGCACTGCCTCTTCCCTGGC       3'  ID:
       | |||||||||||o||||o||           Score: 2.0
3' GTTAT-CGTGACGGAGAGGGGATCGATCGGGT 5'  p-value: 0.0
```

Cs6g11220.1 gene=Cs6g11220 CDS=78-1568

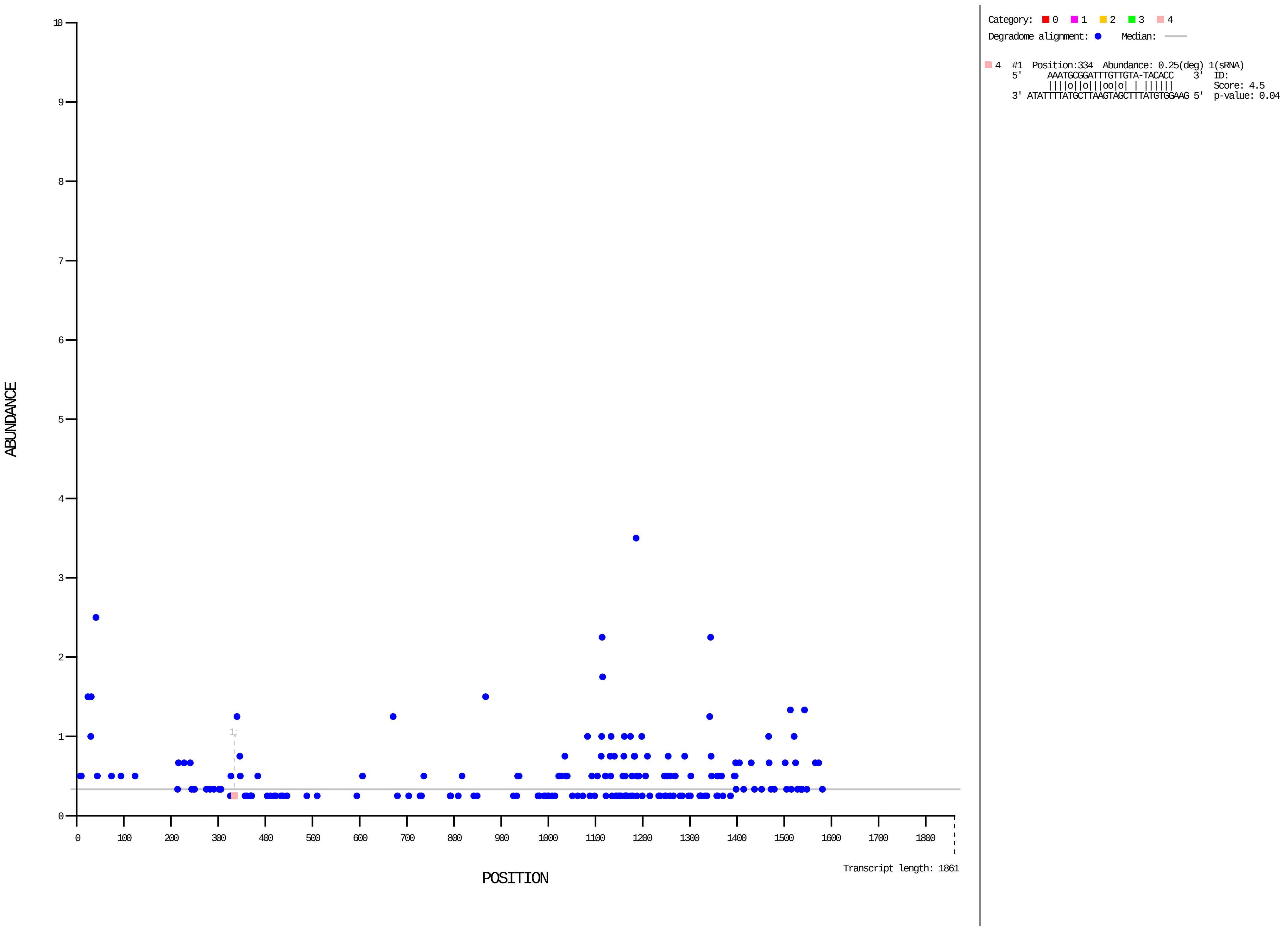

Cs9g05200.3 gene=Cs9g05200 CDS=661-2589

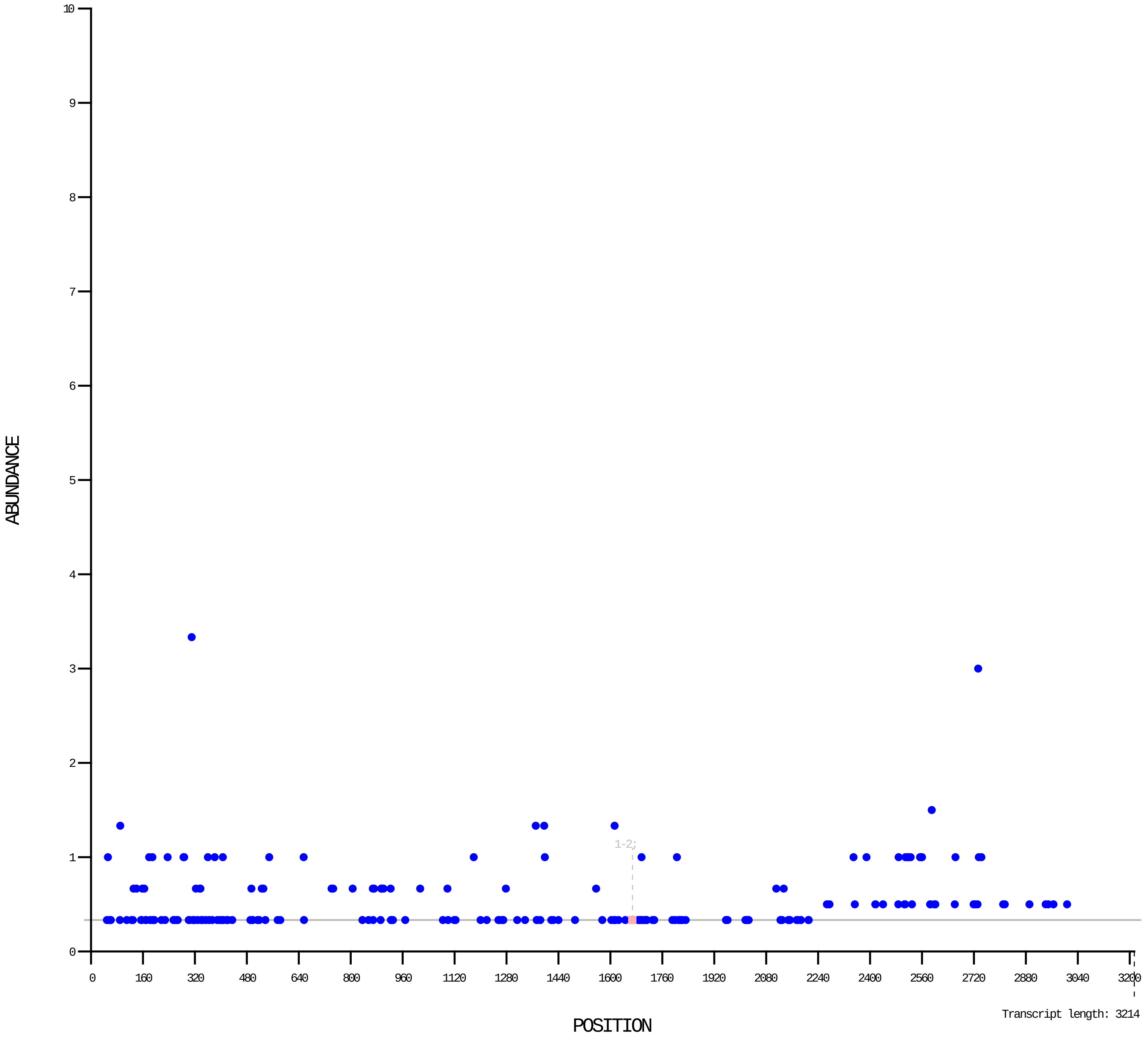

4 #1 Position:1668 Abundance: 0.33(deg) 2(sRNA)

```
5'     ATTTATCTTGTACAAACTGACGTG     3'  ID:
       |||||||||||| ||||||| ||          Score: 3.0
3' CTTGTAAATAGAACAT-TTTGACTACAAGTTC 5'  p-value: 0.02
```

4 #2 Position:1668 Abundance: 0.33(deg) 1(sRNA)

```
5'     ATTTATTTTGTATAAACTGACGTG     3'  ID:
       ||||||o||||| ||||||| ||          Score: 3.5
3' CTTGTAAATAGAACAT-TTTGACTACAAGTTC 5'  p-value: 0.0
```

Cs9g05200.2 gene=Cs9g05200 CDS=197-2311

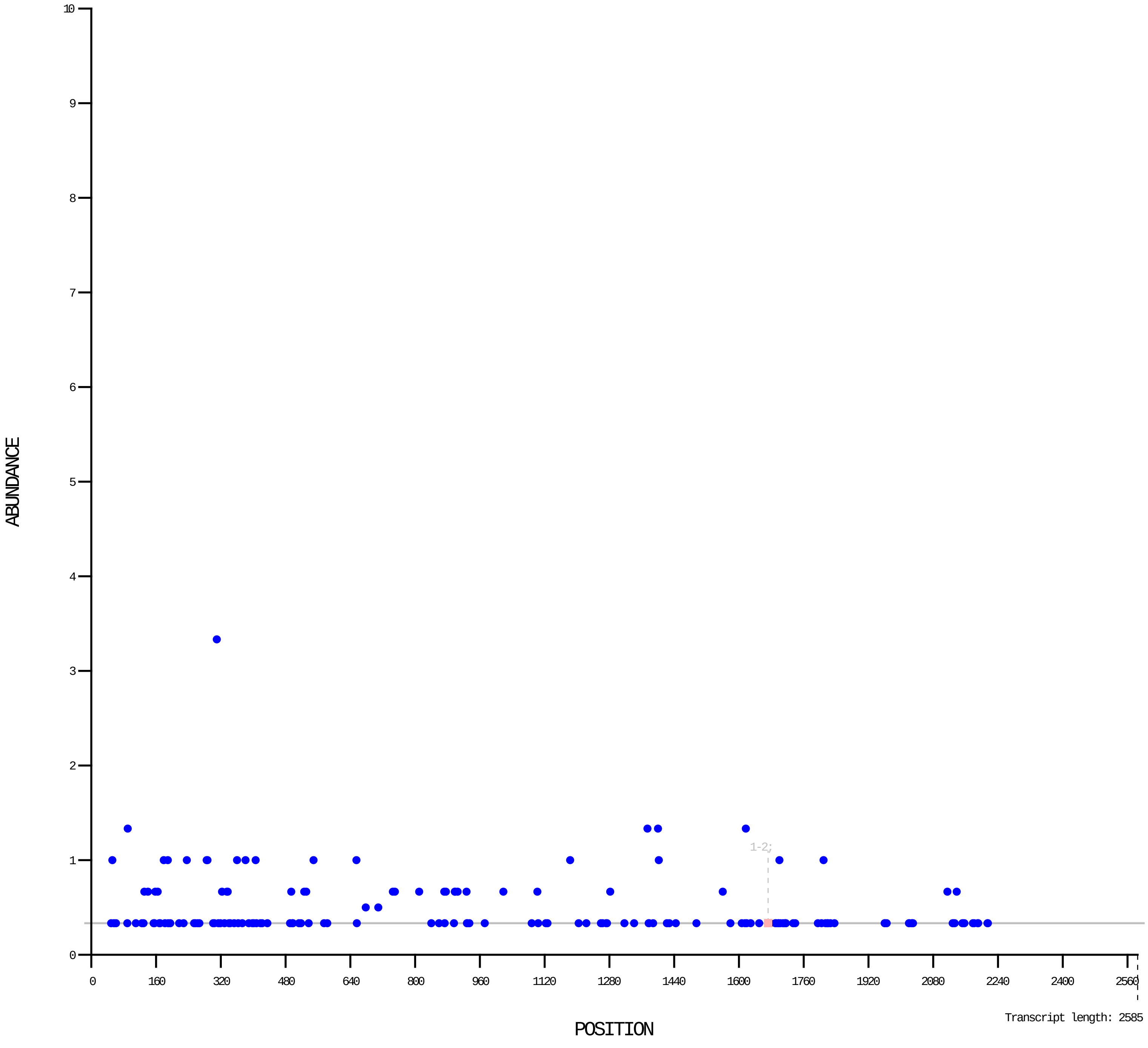

Category: 0 1 2 3 4
Degradome alignment: Median:

4 #1 Position:1672 Abundance: 0.33(deg) 2(sRNA)
5' ATTTATCTTGTACAAACTGACGTG 3' ID:
|||||||||||| ||||||| || Score: 3.0
3' CTTGTAAATAGAACAT-TTTGACTACAAGTTC 5' p-value: 0.0

4 #2 Position:1672 Abundance: 0.33(deg) 1(sRNA)
5' ATTTATTTTGTATAAACTGACGTG 3' ID:
||||||o||||| ||||||| || Score: 3.5
3' CTTGTAAATAGAACAT-TTTGACTACAAGTTC 5' p-value: 0.03

Cs9g05200.1 gene=Cs9g05200 CDS=197-2593

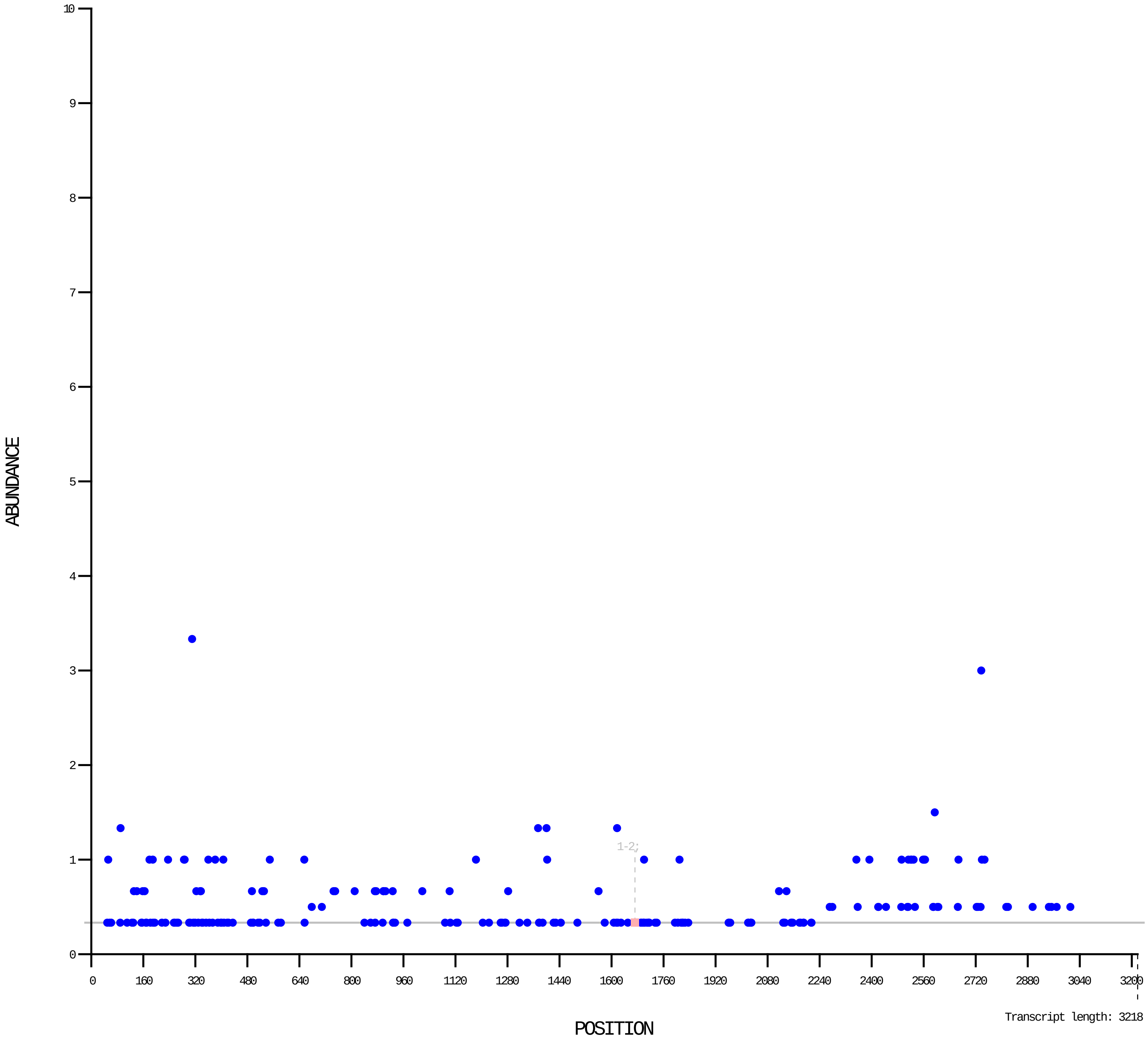

Category: 0 1 2 3 4
Degradome alignment: Median:

4 #1 Position:1672 Abundance: 0.33(deg) 2(sRNA)
```
5'     ATTTATCTTGTACAAACTGACGTG     3'  ID:
       ||||||||||| ||||||| ||           Score: 3.0
3' CTTGTAAATAGAACAT-TTTGACTACAAGTTC 5'  p-value: 0.0
```

4 #2 Position:1672 Abundance: 0.33(deg) 1(sRNA)
```
5'     ATTTATTTTGTATAAACTGACGTG     3'  ID:
       ||||||o||||| ||||||| ||          Score: 3.5
3' CTTGTAAATAGAACAT-TTTGACTACAAGTTC 5'  p-value: 0.0
```

Cs6g15330.2 gene=Cs6g15330 CDS=203-1426

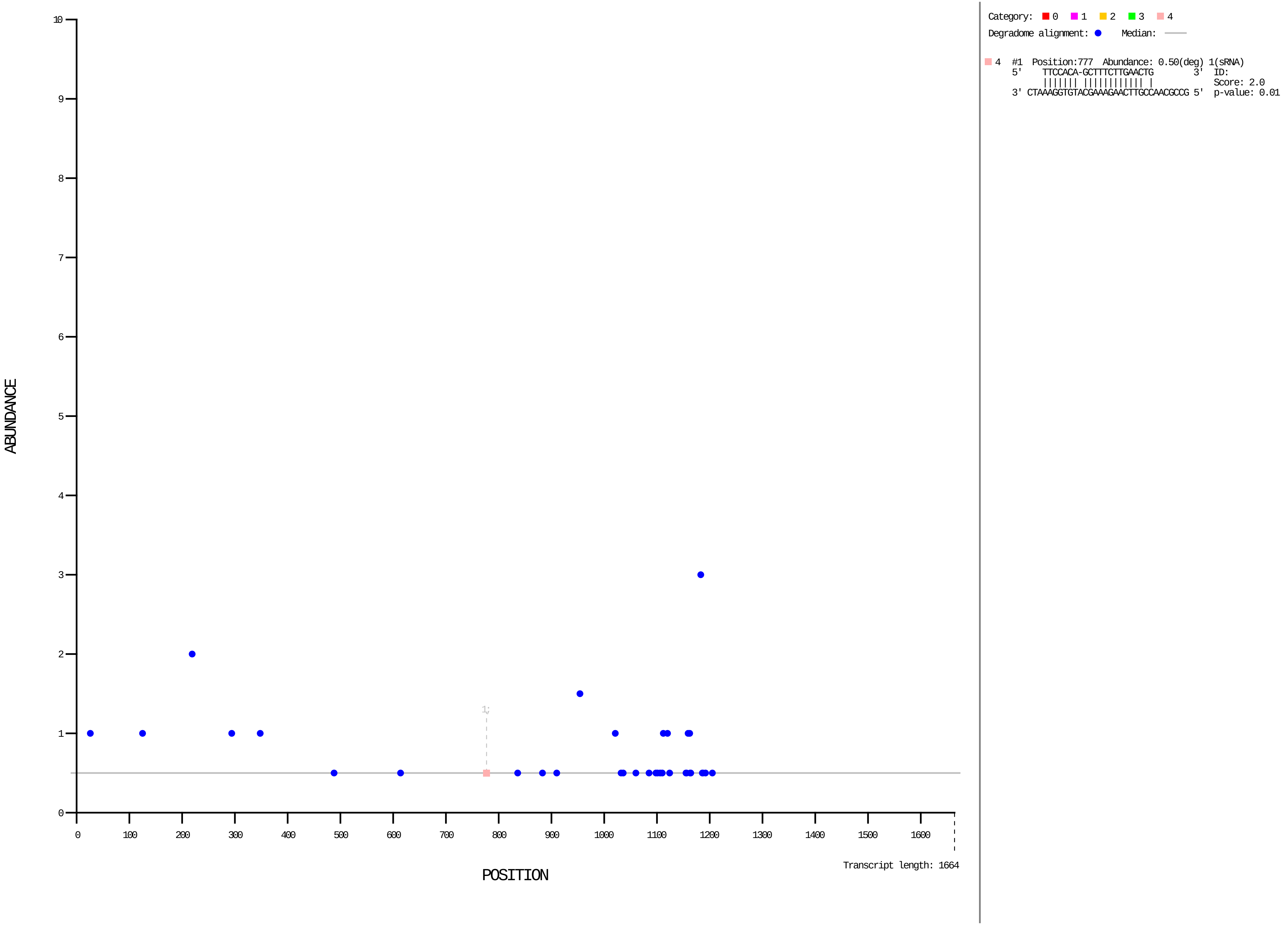

Cs6g15330.1 gene=Cs6g15330 CDS=37-1098

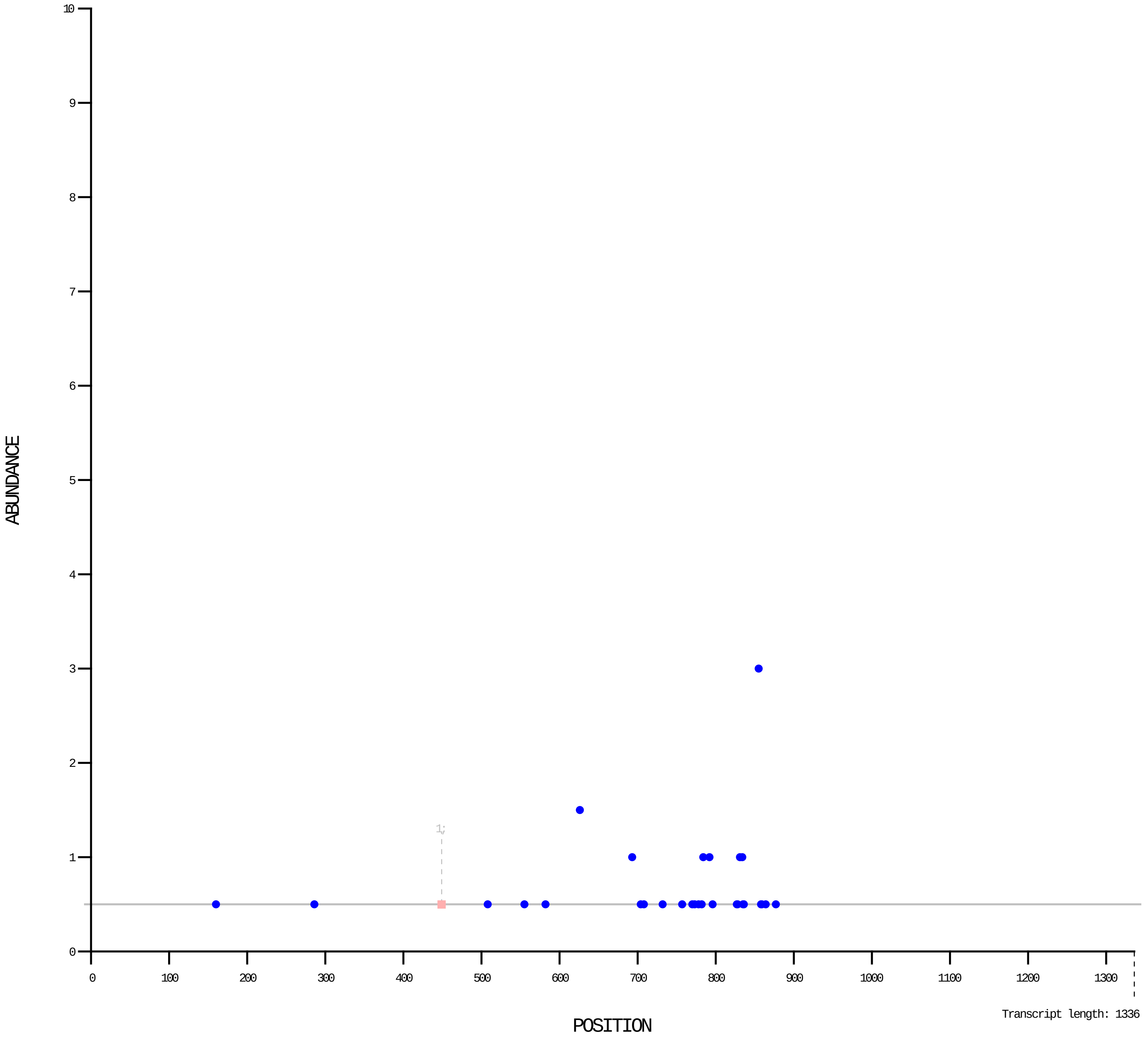

Category: 0 1 2 3 4

Degradome alignment: Median:

4 #1 Position:449 Abundance: 0.50(deg) 1(sRNA)

```
5'    TTCCACA-GCTTTCTTGAACTG        3'  ID:
      ||||||| |||||||||||| |            Score: 2.0
3' CTAAAGGTGTACGAAAGAACTTGCCAACGCCG 5'  p-value: 0.02
```

Cs3g06590.1 gene=Cs3g06590 CDS=1-2781

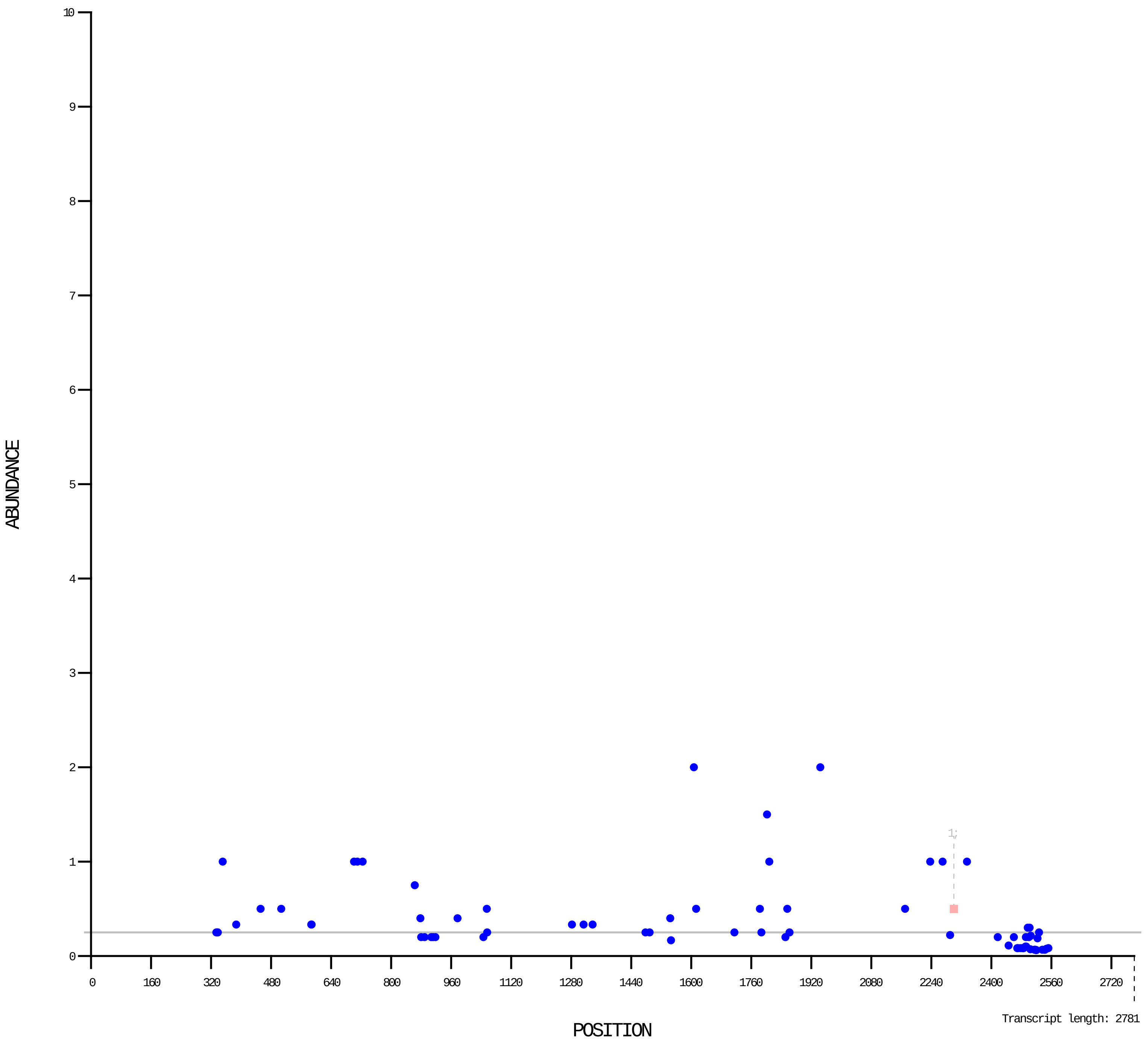

Category: 0 1 2 3 4

Degradome alignment: Median:

```
4  #1  Position:2300  Abundance: 0.50(deg) 1(sRNA)
   5'     CACGCGGCCATCTCTCATTGA        3'  ID:
          || |||o|||||||o| ||||            Score: 3.0
   3' TAGTGTTCGCTGGTAGAGGG-AACTTAGCTGC 5'  p-value: 0.05
```

Cs6g15640.1 gene=Cs6g15640 CDS=73-1503

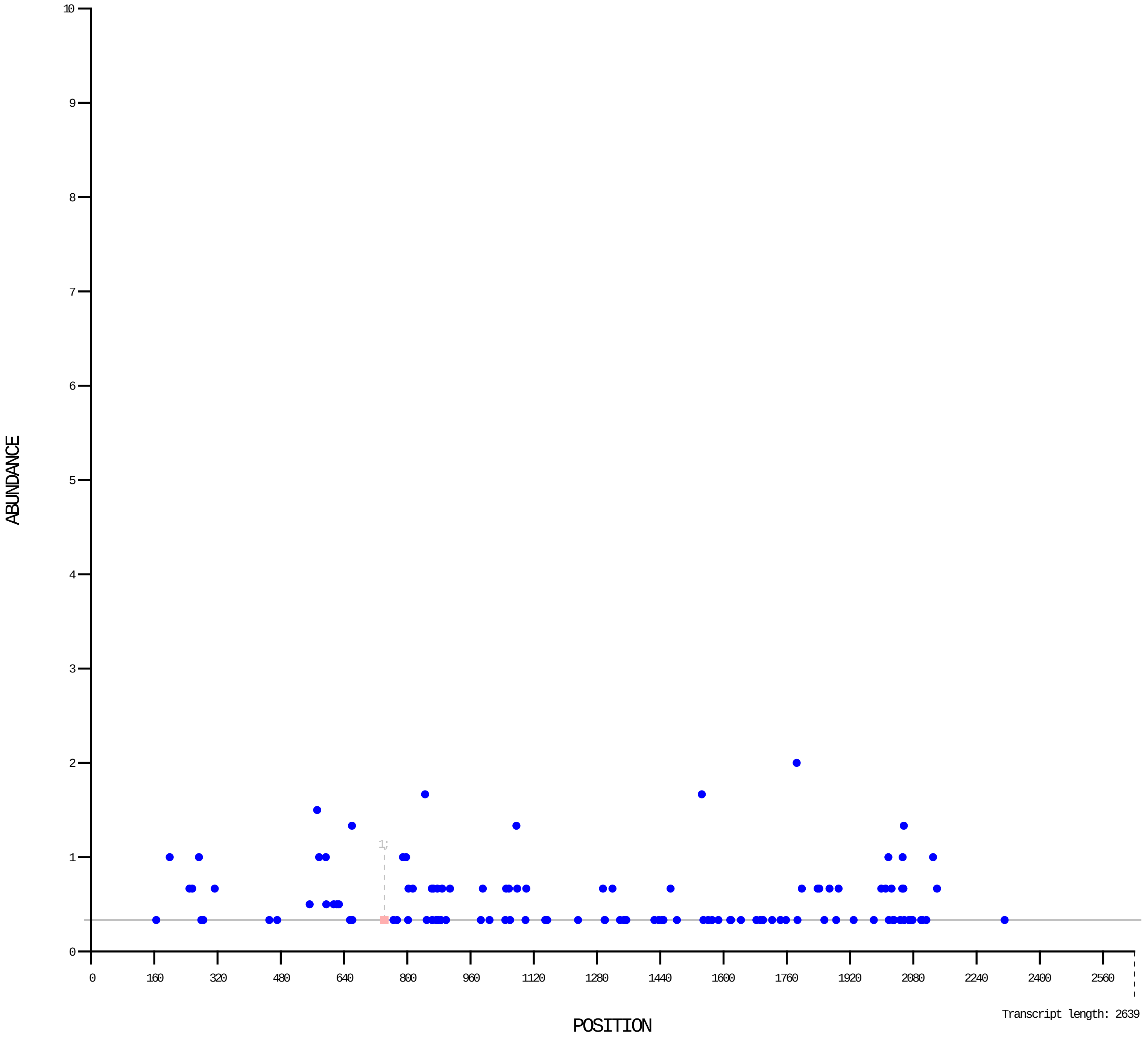

Category: 0 1 2 3 4
Degradome alignment: Median:

4 #1 Position:742 Abundance: 0.33(deg) 1(sRNA)

```
5'     AAAATTTTGAGGG-ACTAATGATA     3'  ID:
       ||||||||||| o| | ||||||||         Score: 3.5
3'  TTCCTTTTAAAACTGTCGTTATTACTATAGTC 5'  p-value: 0.03
```

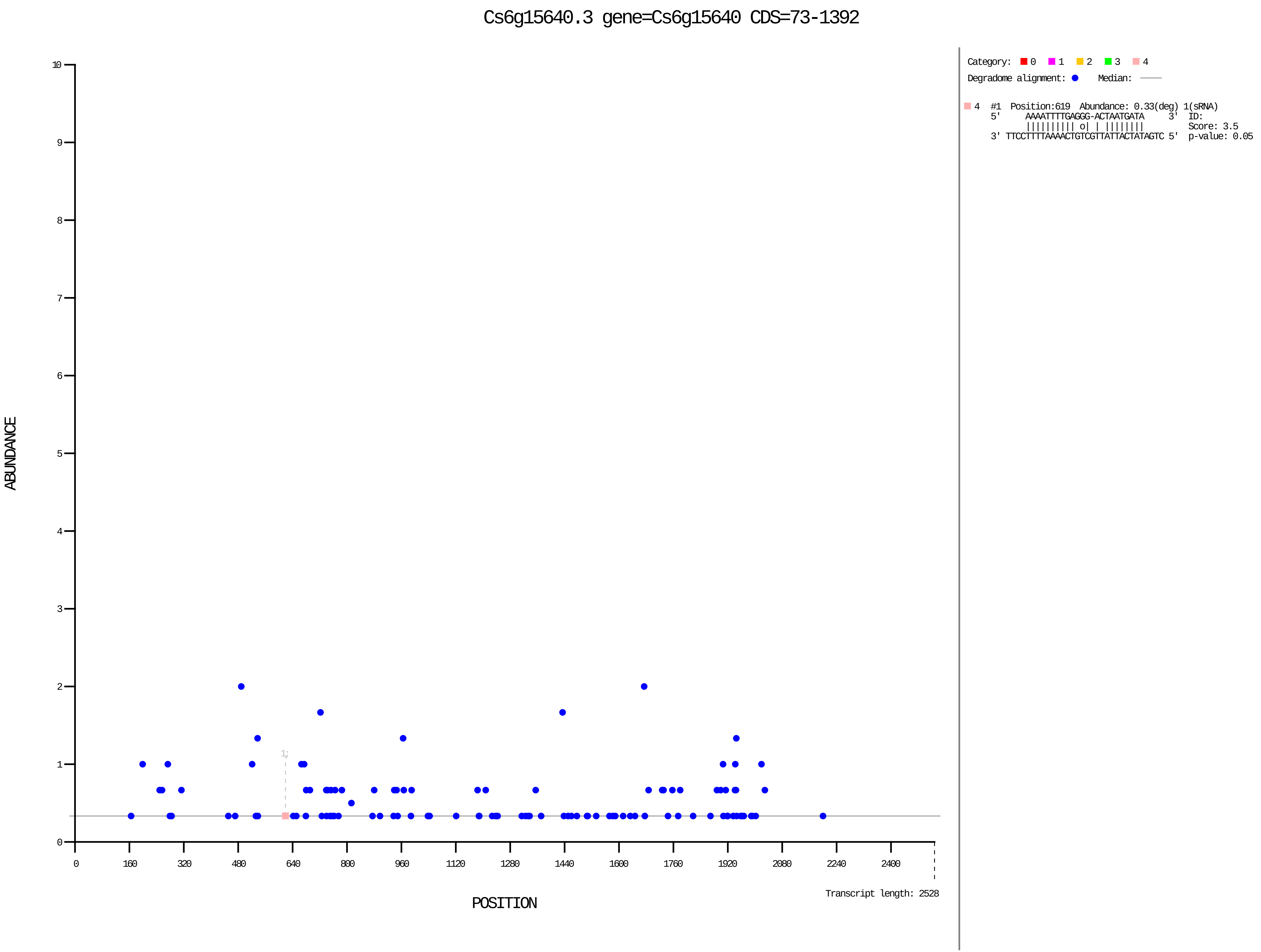

Cs6g15640.3 gene=Cs6g15640 CDS=73-1392
ABUNDANCE
POSITION
Transcript length: 2528
Category: 0 1 2 3 4
Degradome alignment:
Median:
4 #1 Position:619 Abundance: 0.33(deg) 1(sRNA)
5' AAAATTTTGAGGG-ACTAATGATA 3' ID:
Score: 3.5
3' TTCCTTTTAAAACTGTCGTTATTACTATAGTC 5' p-value: 0.05
