# Supplementary material for: Genome-wide comprehensive analysis of transcriptomes and small RNAs offers insights into the molecular mechanism of alkaline stress tolerance in a citrus rootstock
Source: Hortic Res. 2019 Mar 1;6:33. doi: 10.1038/s41438-018-0116-0 (PMC6395741; doi:10.1038/s41438-018-0116-0)

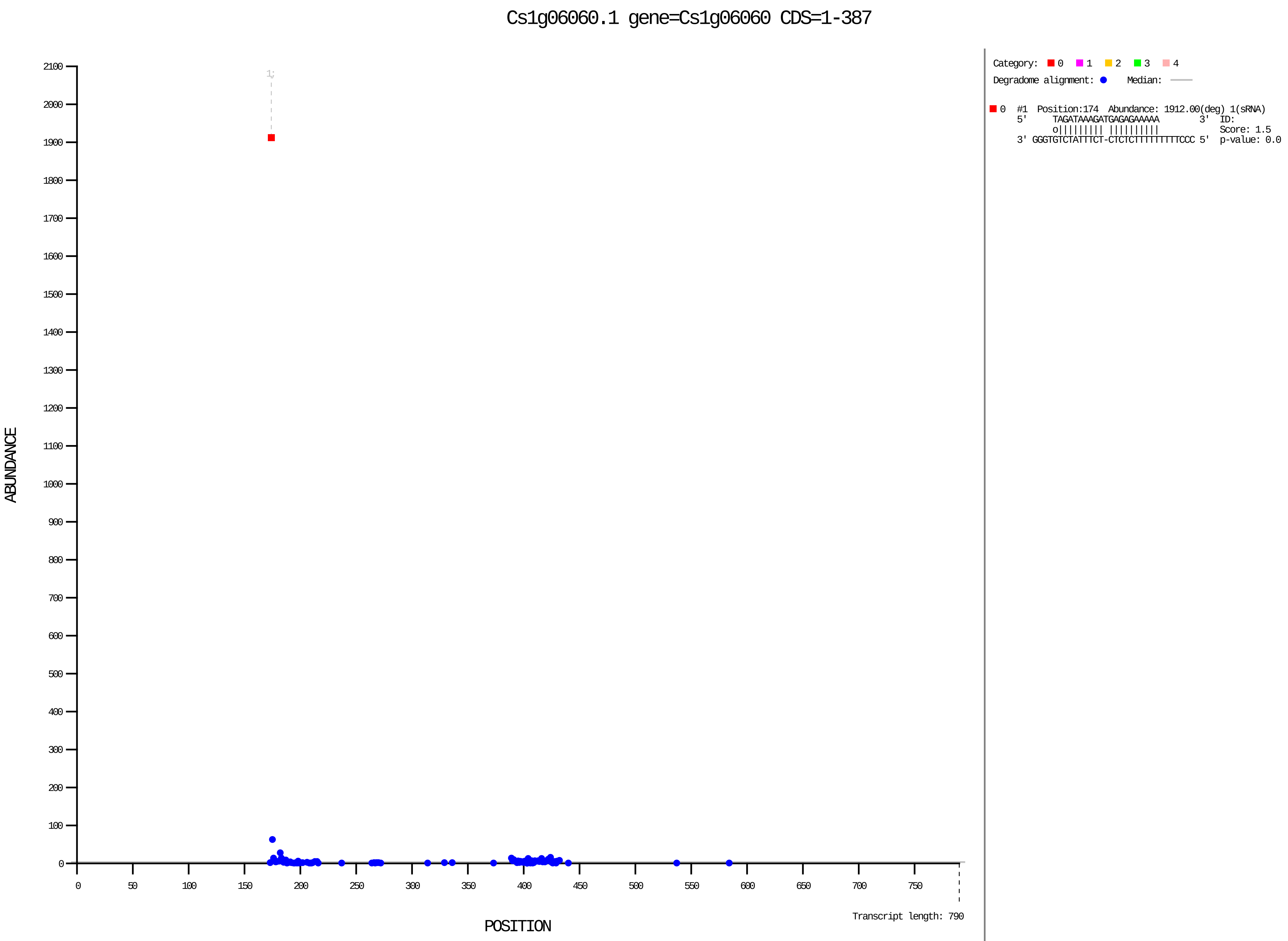

Cs1g06060.1 gene=Cs1g06060 CDS=1-387
ABUNDANCE
POSITION
Transcript length: 790
Category: 0 1 2 3 4
Degradome alignment:
Median:
0 #1 Position:174 Abundance: 1912.00(deg) 1(sRNA)
5' TAGATAAAGATGAGAGAAAAA 3' ID:
o||||||||| |||||||||||
Score: 1.5
3' GGGTGTCTATTTCT-CTCTCTTTTTTTTTTCCC 5' p-value: 0.0

Cs7g25670.1 gene=Cs7g25670 CDS=375-2411

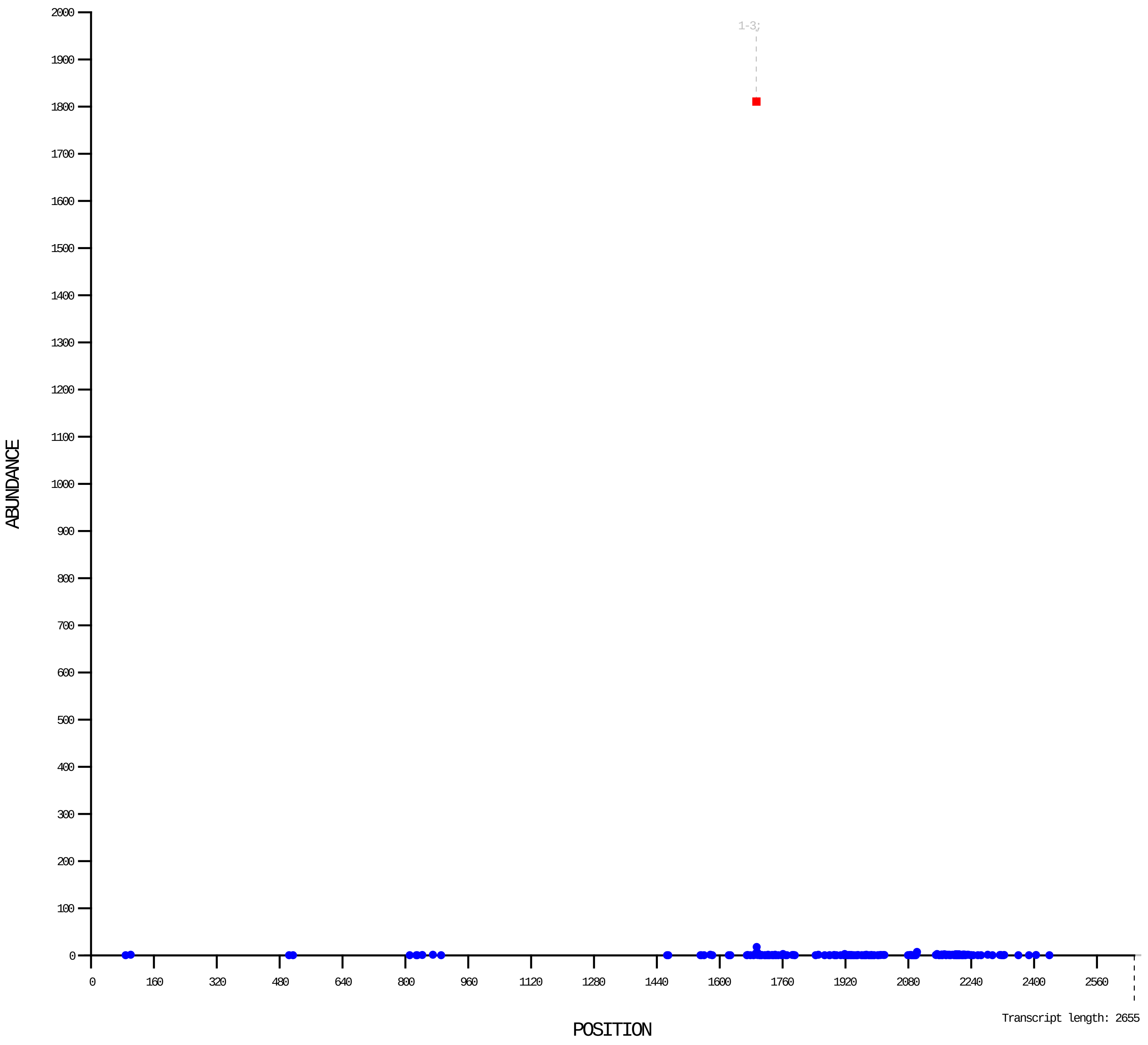

Category: 0 1 2 3 4

Degradome alignment: Median:

```
0  #1  Position:1693  Abundance: 1810.50(deg) 1(sRNA)
   5'      TGCCTGGCTCCCTGTATGCTT        3'  ID:
           ||||||||||||||||||o|             Score: 0.5
   3'  CCGTACGGACCGAGGGACATACGGACGTCCTT 5'  p-value: 0.0

0  #2  Position:1693  Abundance: 1810.50(deg) 1(sRNA)
   5'      TGCCTGGCTCCCTGTATGCCG        3'  ID:
           ||||||||||||||||||||             Score: 1.0
   3'  CCGTACGGACCGAGGGACATACGGACGTCCTT 5'  p-value: 0.0

0  #3  Position:1693  Abundance: 1810.50(deg) 1(sRNA)
   5'      TGCCTGGCTCCCTGTATGCCA        3'  ID:
           ||||||||||||||||||||             Score: 1.0
   3'  CCGTACGGACCGAGGGACATACGGACGTCCTT 5'  p-value: 0.0
```

Cs7g25670.2 gene=Cs7g25670 CDS=375-2459

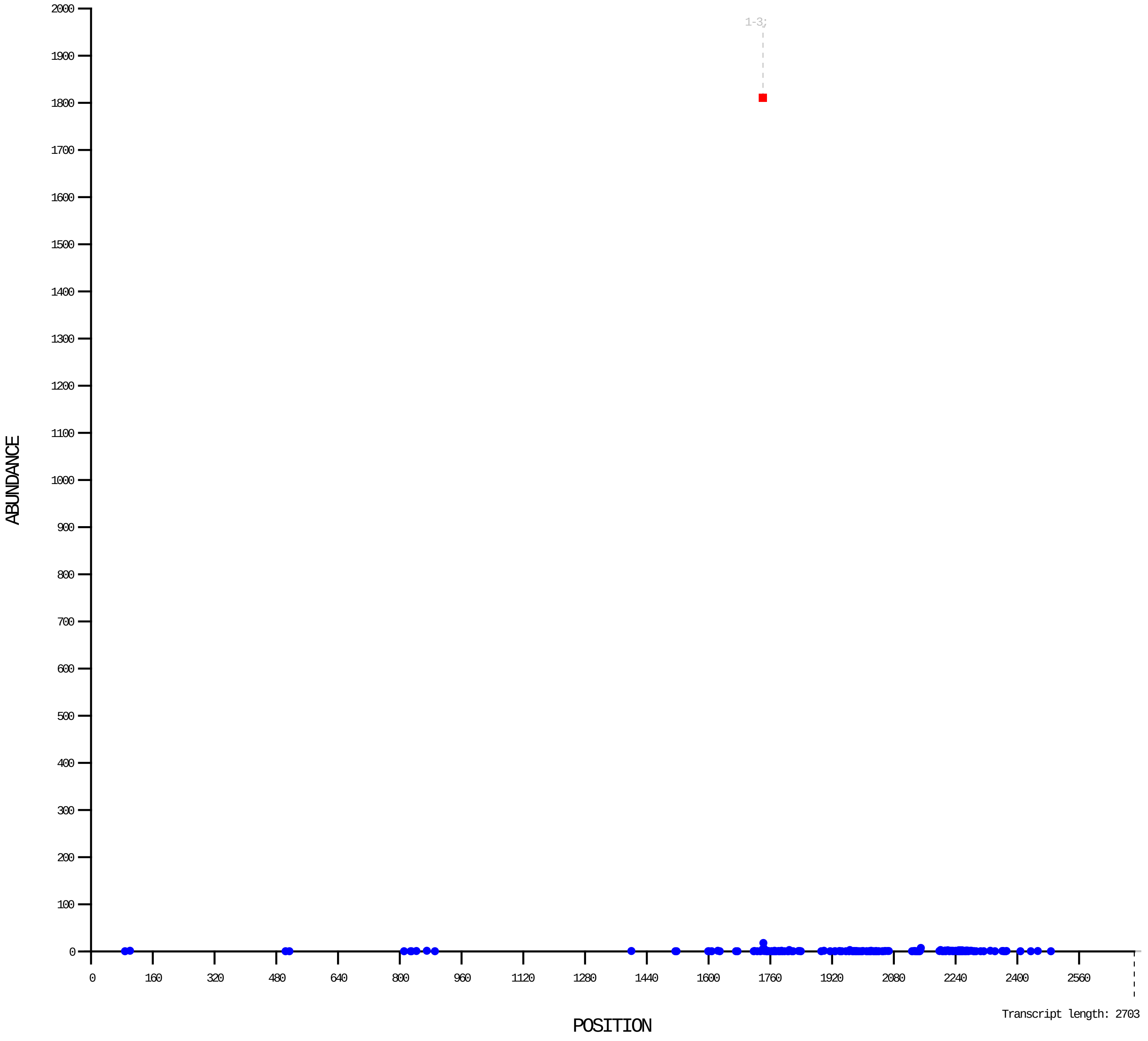

Category: 0 1 2 3 4
Degradome alignment: Median:

■ 0 #1 Position:1741 Abundance: 1810.50(deg) 1(sRNA)
```
5'     TGCCTGGCTCCCTGTATGCTT        3'  ID:
       ||||||||||||||||||o|             Score: 0.5
3' CCGTACGGACCGAGGGACATACGGACGTCCTT 5'  p-value: 0.0
```

■ 0 #2 Position:1741 Abundance: 1810.50(deg) 1(sRNA)
```
5'     TGCCTGGCTCCCTGTATGCCG        3'  ID:
       ||||||||||||||||||||             Score: 1.0
3' CCGTACGGACCGAGGGACATACGGACGTCCTT 5'  p-value: 0.0
```

■ 0 #3 Position:1741 Abundance: 1810.50(deg) 1(sRNA)
```
5'     TGCCTGGCTCCCTGTATGCCA        3'  ID:
       ||||||||||||||||||||             Score: 1.0
3' CCGTACGGACCGAGGGACATACGGACGTCCTT 5'  p-value: 0.0
```

Cs3g18940.1 gene=Cs3g18940 CDS=177-1901

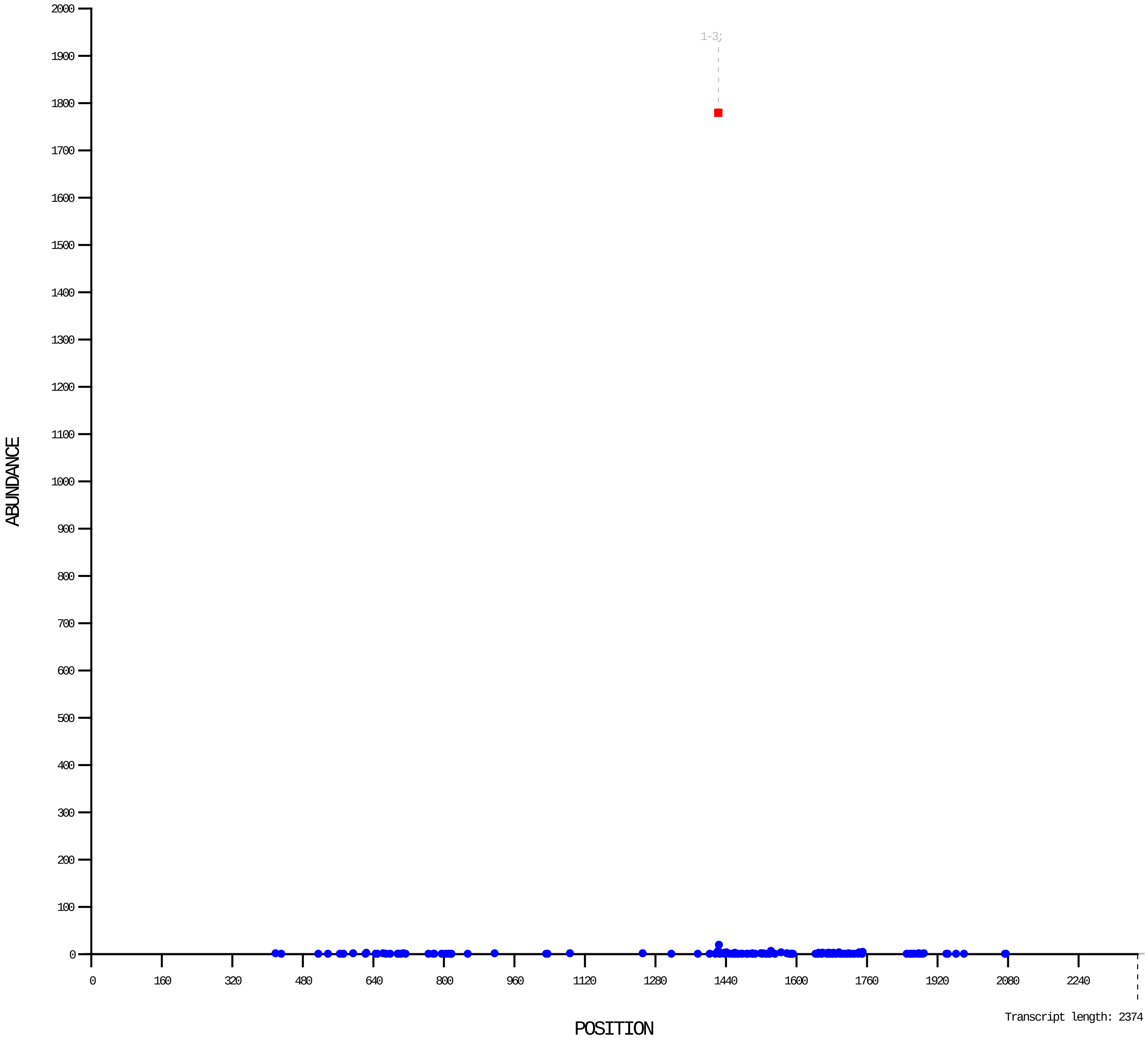

Category: 0 1 2 3 4
Degradome alignment: ● Median:

0 #1 Position:1423 Abundance: 1780.00(deg) 1(sRNA)
```
5'     TGCCTGGCTCCCTGTATGCCA        3'  ID:
       ||||||||||||||o||||||            Score: 0.5
3' TAAAACGGACCGAGGGACGTACGGTCGTCCTT 5'  p-value: 0.0
```

0 #2 Position:1423 Abundance: 1780.00(deg) 1(sRNA)
```
5'     TGCCTGGCTCCCTGTATGCCG        3'  ID:
       ||||||||||||||o|||||o            Score: 1.0
3' TAAAACGGACCGAGGGACGTACGGTCGTCCTT 5'  p-value: 0.0
```

0 #3 Position:1423 Abundance: 1780.00(deg) 1(sRNA)
```
5'     TGCCTGGCTCCCTGTATGCTT        3'  ID:
       ||||||||||||||o||||o             Score: 2.0
3' TAAAACGGACCGAGGGACGTACGGTCGTCCTT 5'  p-value: 0.0
```

Cs3g06390.1 gene=Cs3g06390 CDS=101-1471

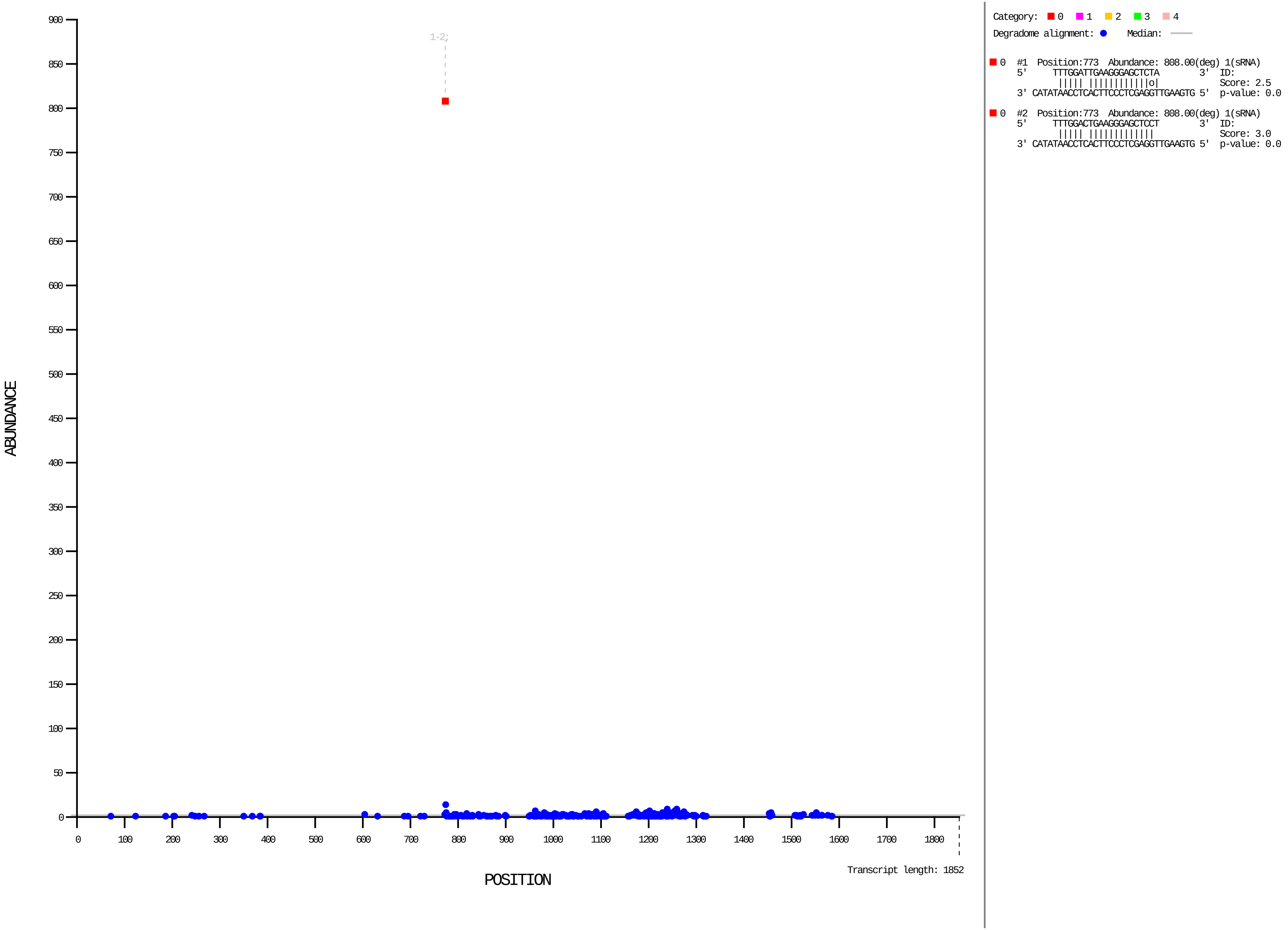

Cs3g12000.2 gene=Cs3g12000 CDS=235-693

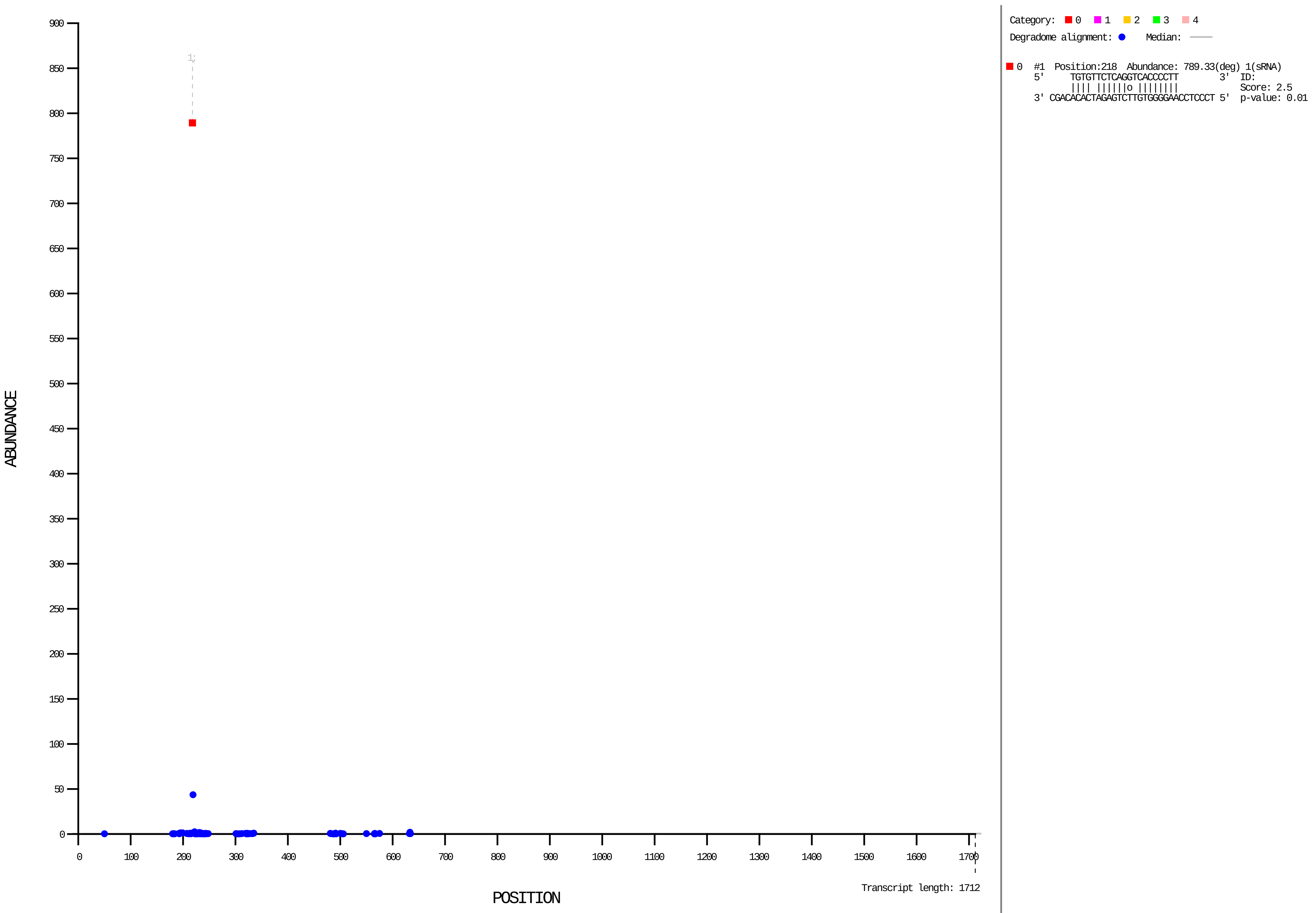

Cs3g12000.3 gene=Cs3g12000 CDS=235-717

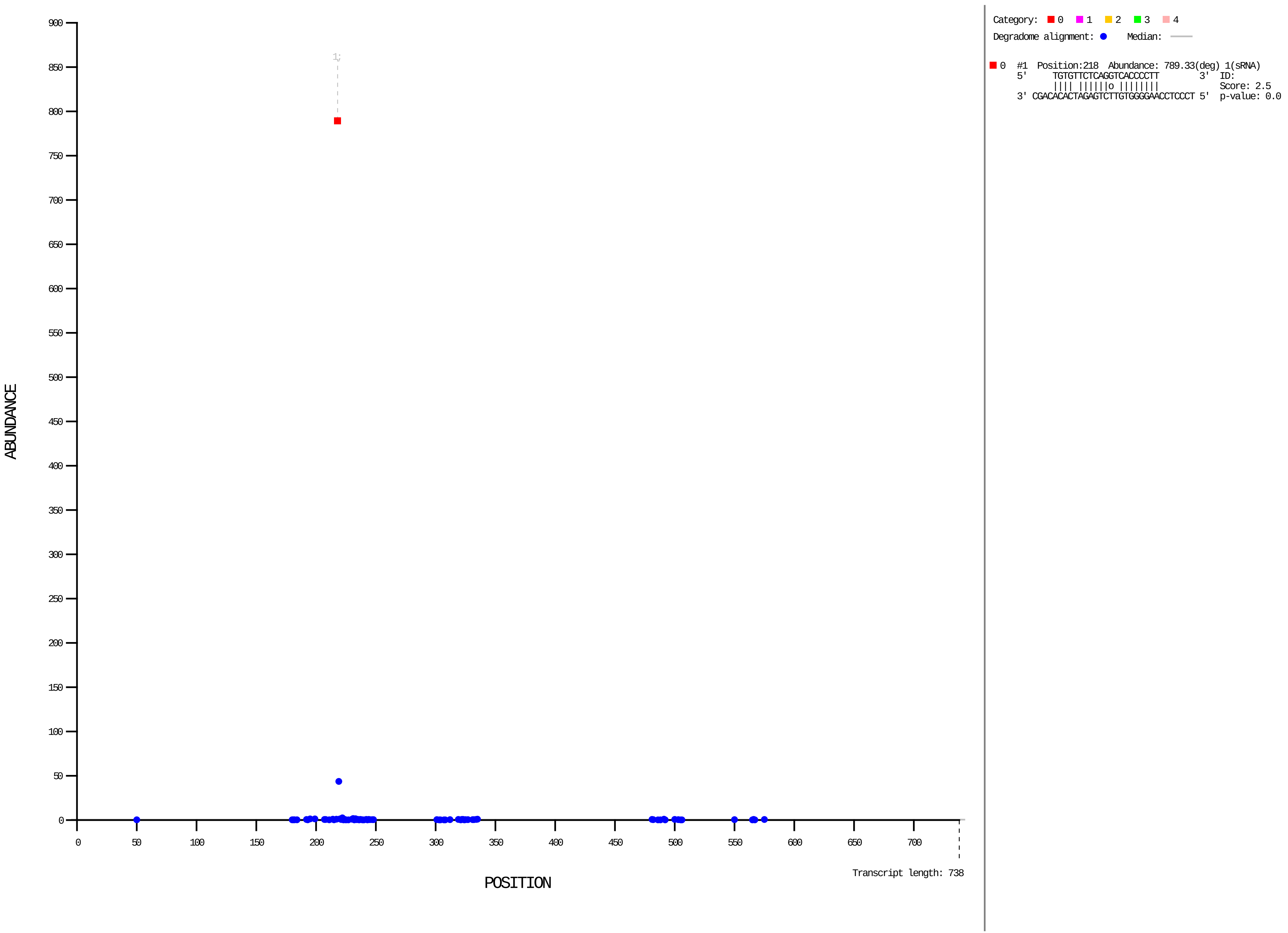

Cs3g12000.4 gene=Cs3g12000 CDS=235-705

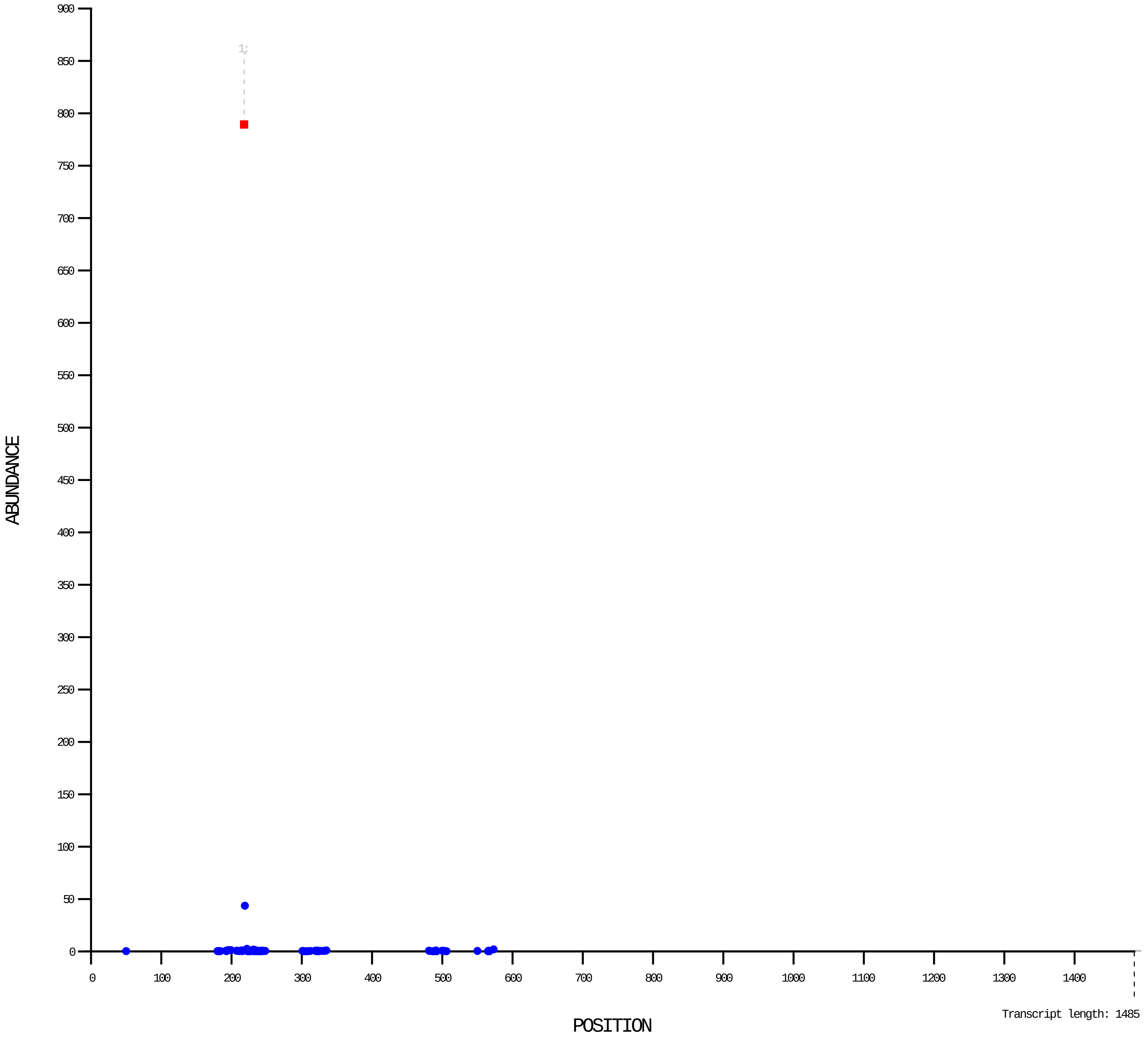

Category: 0 1 2 3 4
Degradome alignment: Median:

0 #1 Position:218 Abundance: 789.33(deg) 1(sRNA)

```
5'     TGTGTTCTCAGGTCACCCCTT       3'  ID:
       |||| ||||||o ||||||||           Score: 2.5
3' CGACACACTAGAGTCTTGTGGGGAACCTCCCT 5'  p-value: 0.0
```

Cs5g10870.2 gene=Cs5g10870 CDS=88-987

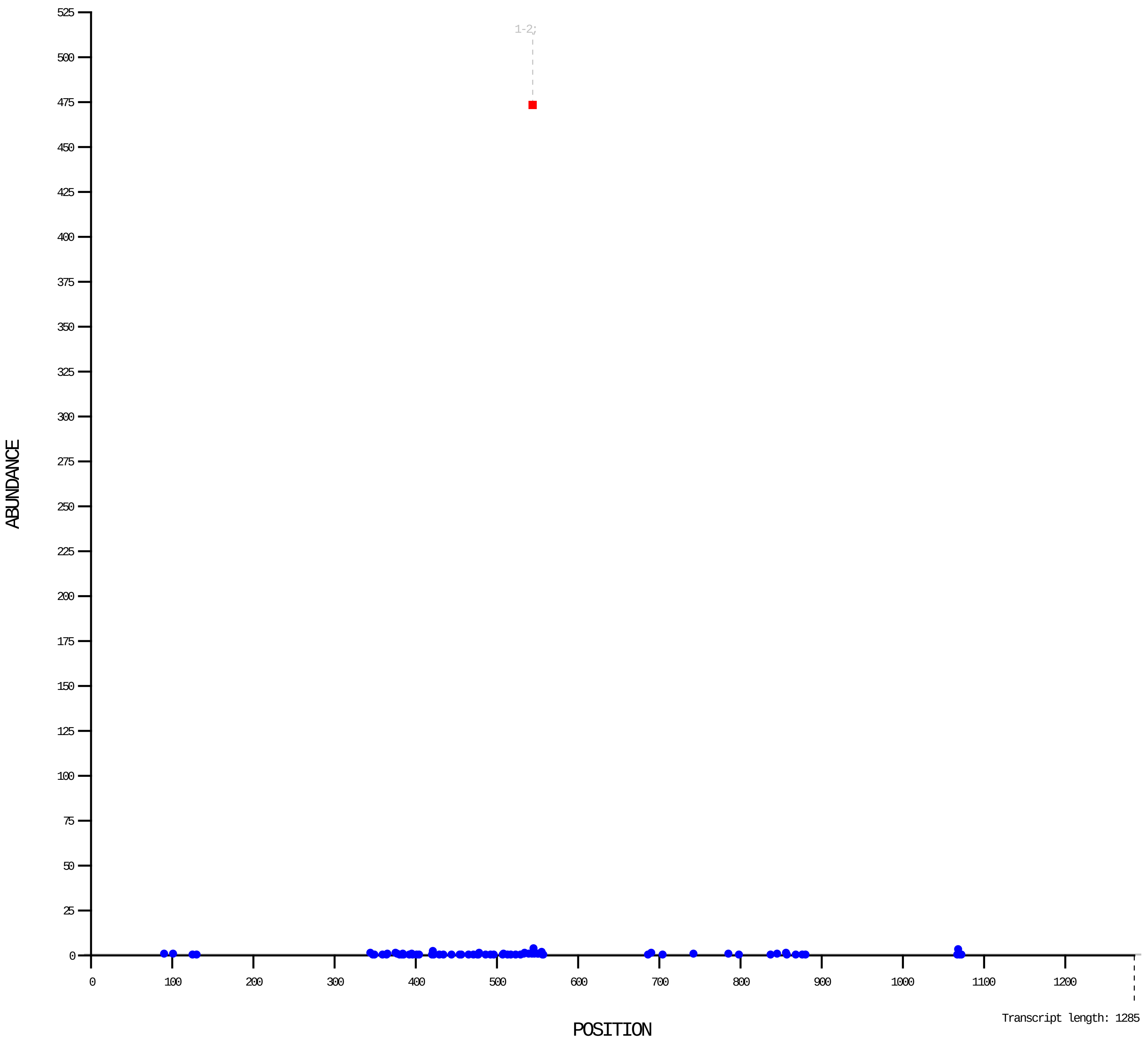

Category: 0 1 2 3 4
Degradome alignment: Median:

0 #1 Position:544 Abundance: 473.50(deg) 1(sRNA)
```
5'     TGGAGAAGCAGGGCACGTGCA        3'  ID:
       |||||||||||o|||||o               Score: 3.0
3' CTTAACCTCTTCGTCCTGTGCATTCGGCTTAG 5'  p-value: 0.0
```

0 #2 Position:544 Abundance: 473.50(deg) 1(sRNA)
```
5'     TGGAGAAGCAGGGCACATGCT        3'  ID:
       |||||||||||o||| |o               Score: 4.0
3' CTTAACCTCTTCGTCCTGTGCATTCGGCTTAG 5'  p-value: 0.0
```

Cs5g10870.1 gene=Cs5g10870 CDS=408-1505

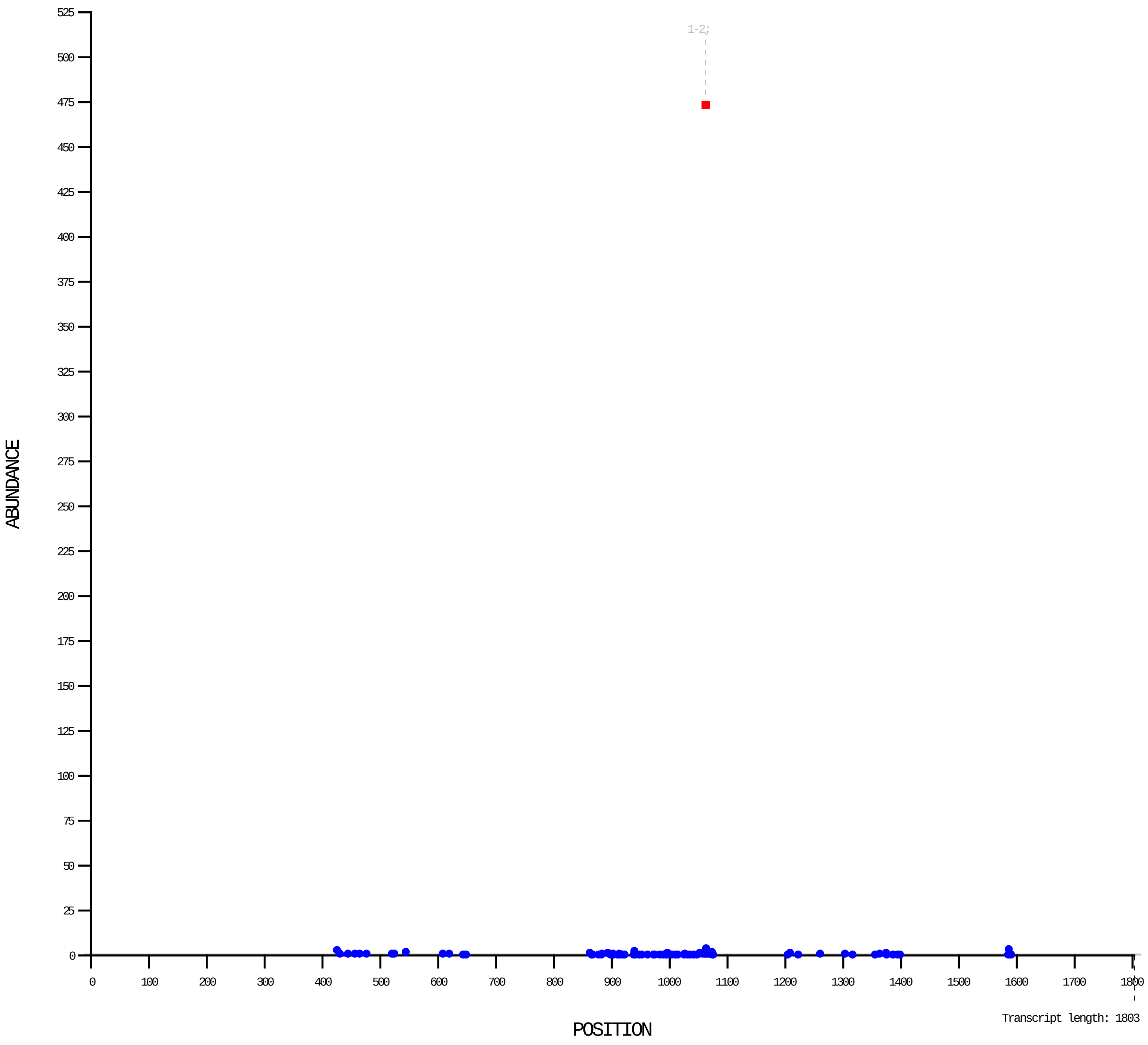

Category: 0 1 2 3 4
Degradome alignment: ● Median: —

0 #1 Position:1062 Abundance: 473.50(deg) 1(sRNA)
```
5'     TGGAGAAGCAGGGCACGTGCA        3'  ID:
       ||||||||||||o|||||o              Score: 3.0
3' CTTAACCTCTTCGTCCTGTGCATTCGGCTTAG 5'  p-value: 0.0
```

0 #2 Position:1062 Abundance: 473.50(deg) 1(sRNA)
```
5'     TGGAGAAGCAGGGCACATGCT        3'  ID:
       ||||||||||||o||| |o              Score: 4.0
3' CTTAACCTCTTCGTCCTGTGCATTCGGCTTAG 5'  p-value: 0.03
```

Cs8g16440.1 gene=Cs8g16440 CDS=606-2780

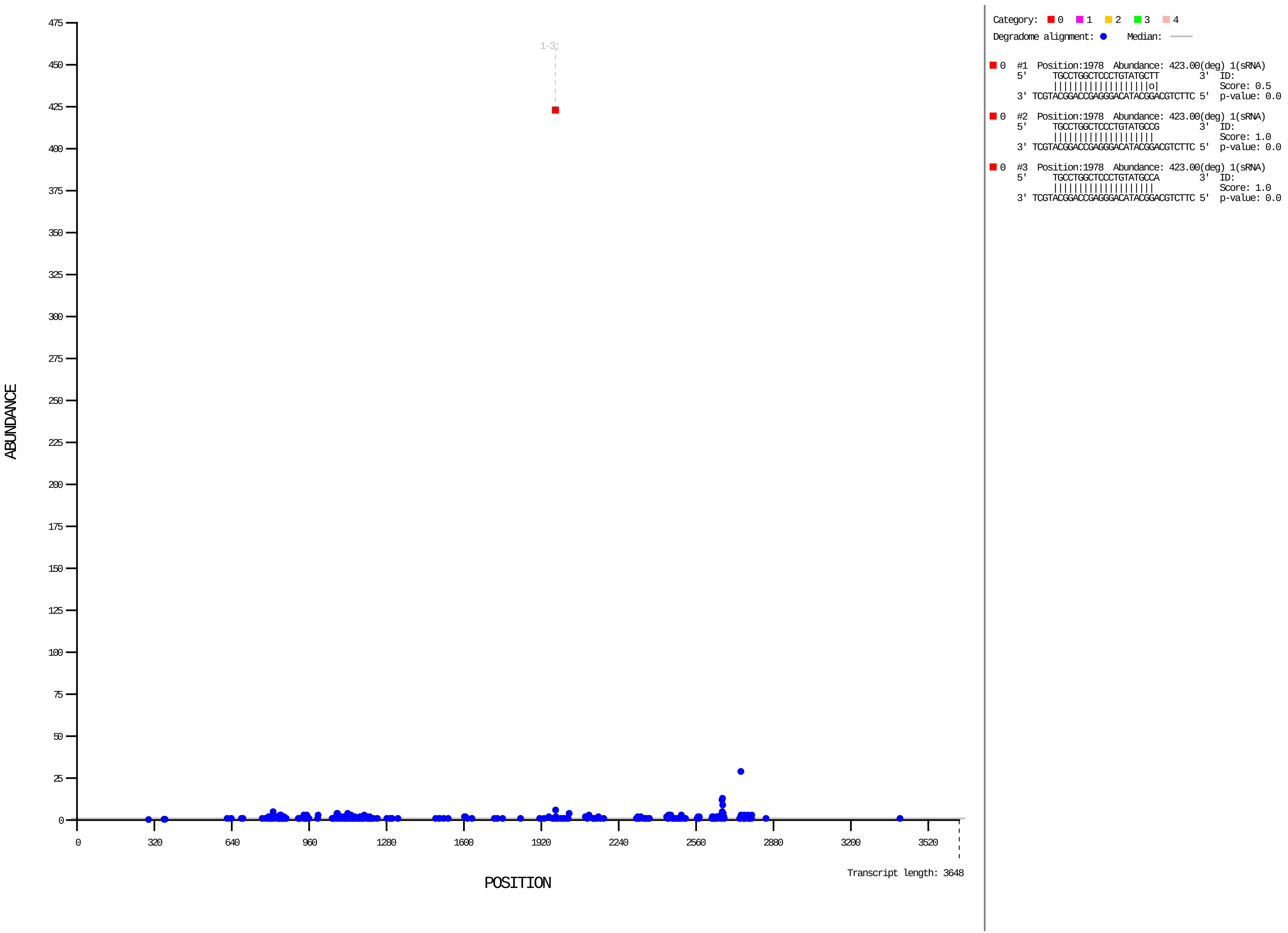

orange1.1t05622.1 gene=orange1.1t05622 CDS=3-347

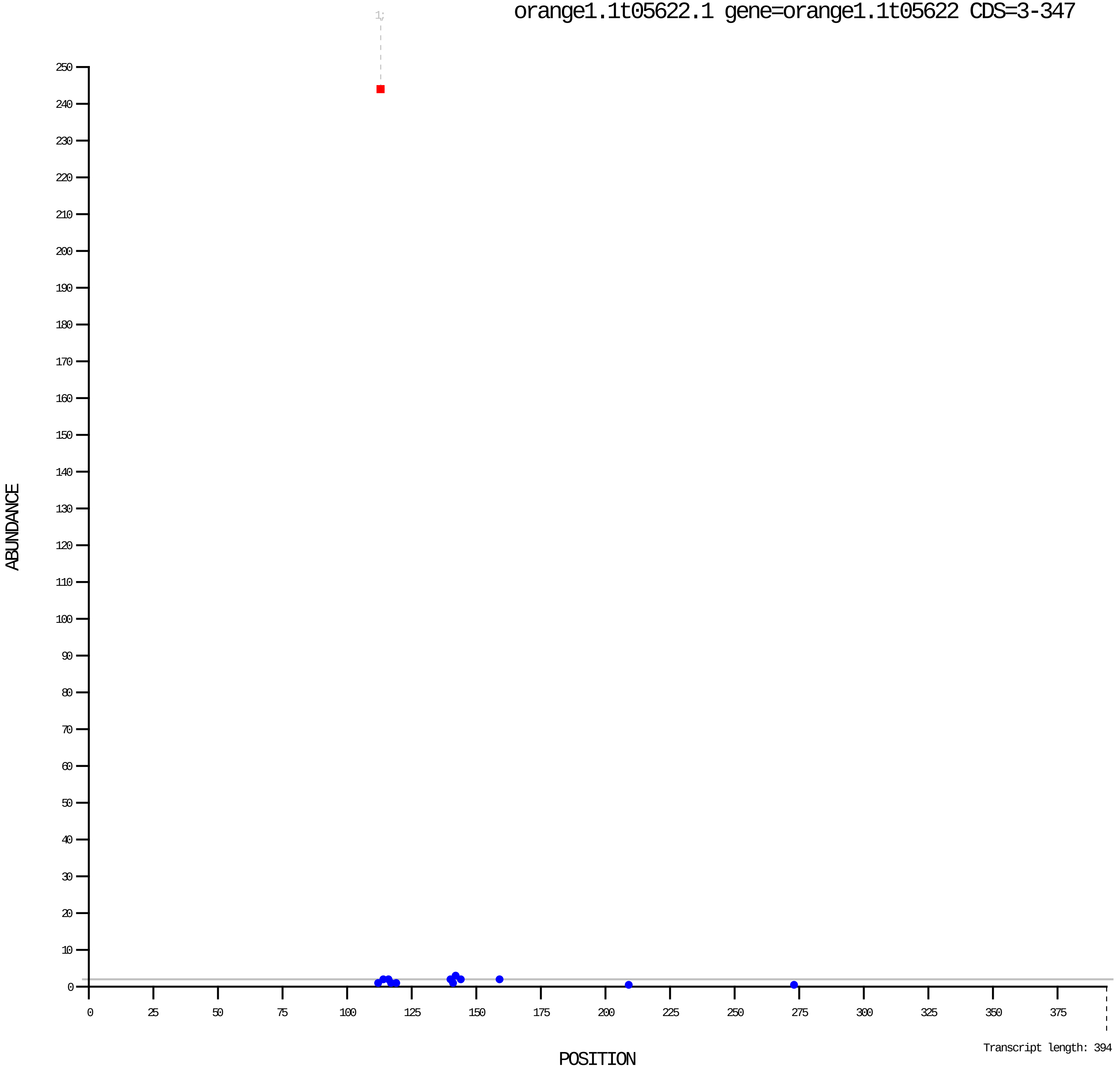

Category: 0 1 2 3 4

Degradome alignment: Median:

0 #1 Position:113 Abundance: 244.00(deg) 1(sRNA)

```
5'     TAGATAAAGATGAGAGAAAAA        3'  ID:
       o||||||||| ||||||||||            Score: 1.5
3' GGGTGTCTATTTCT-CTCTCTTTTTTTTTCTC 5'  p-value: 0.0
```

Cs9g01780.1 gene=Cs9g01780 CDS=99-254

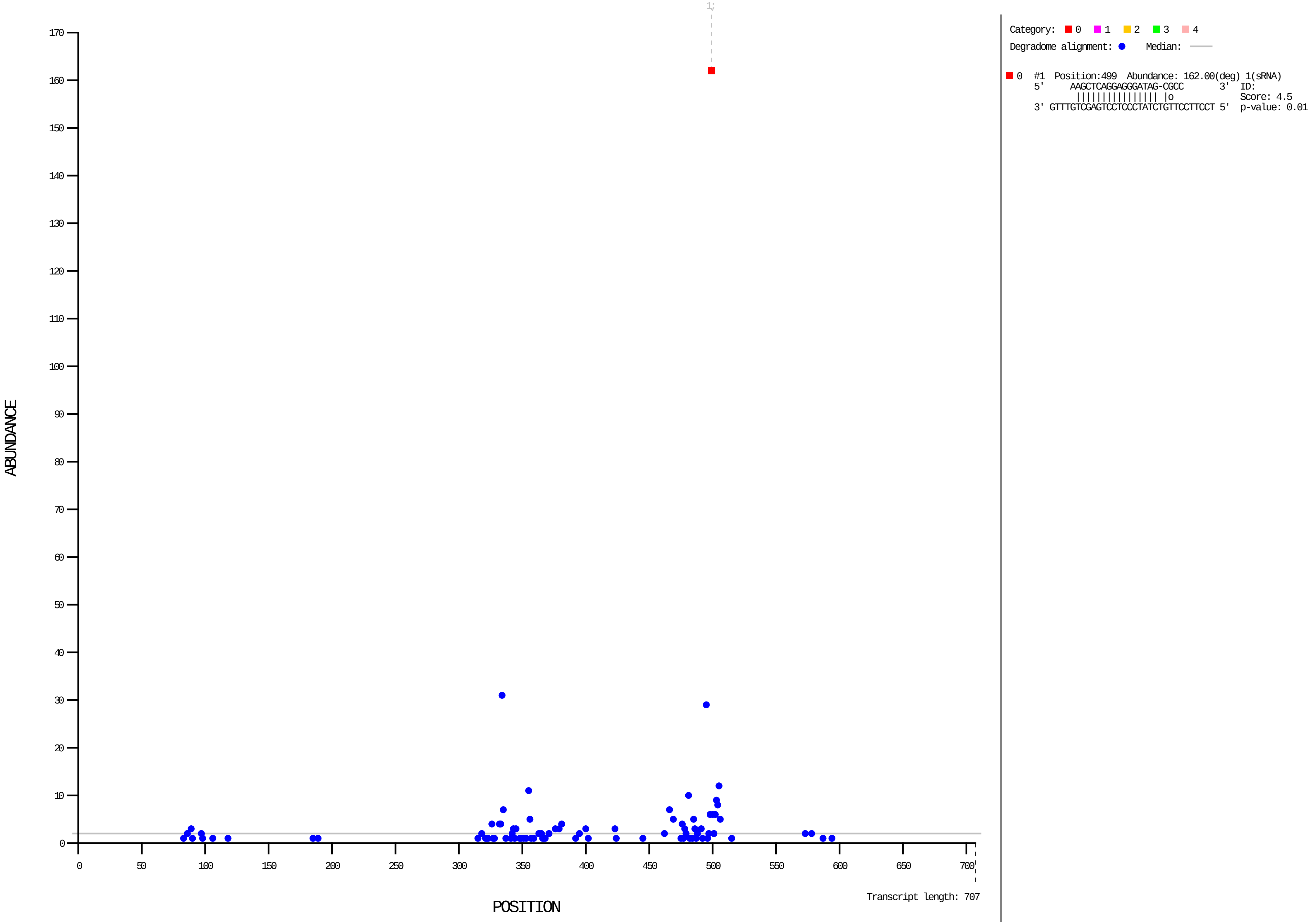

orange1.1t00199.1 gene=orange1.1t00199 CDS=501-2621

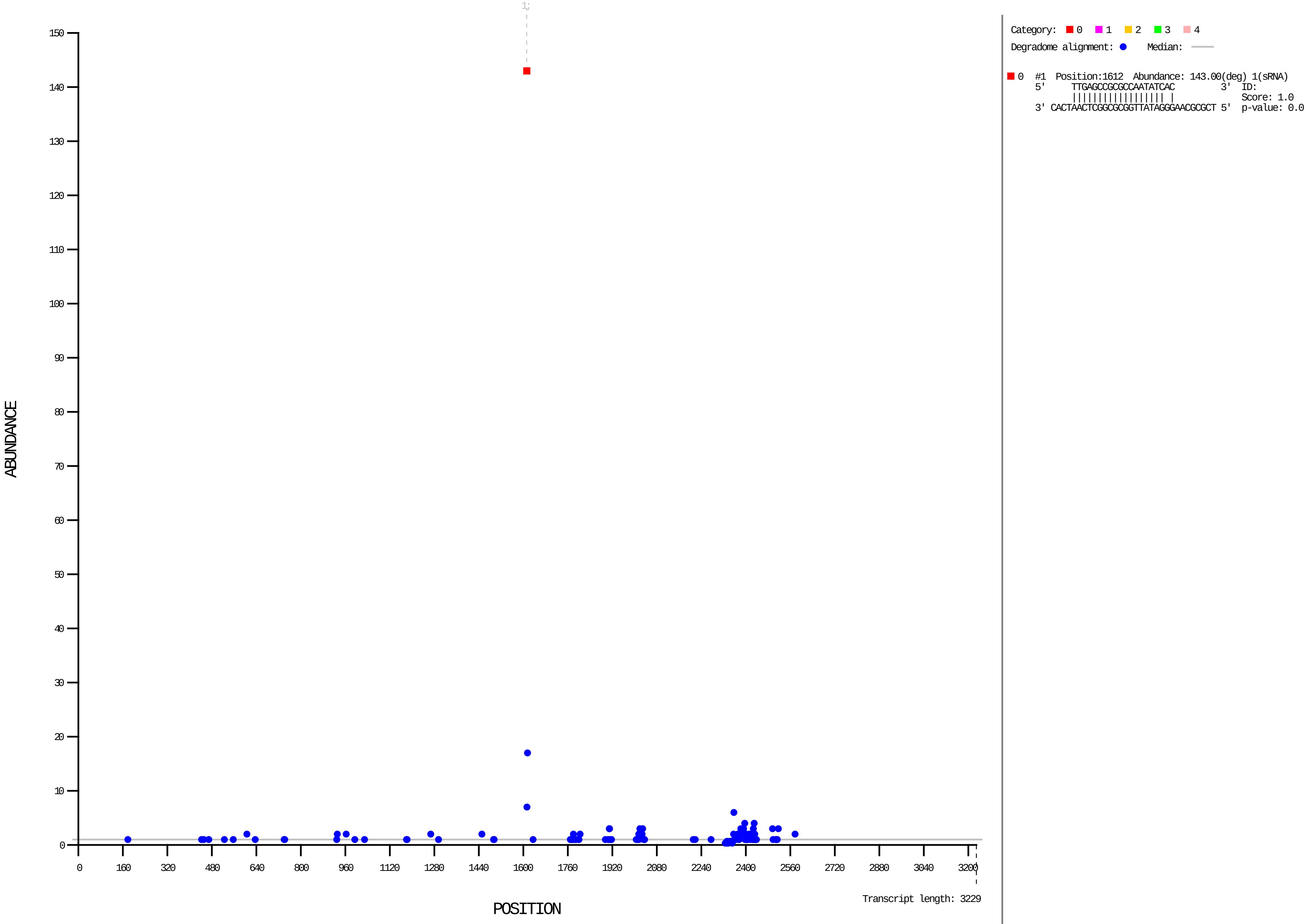

Cs8g15520.1 gene=Cs8g15520 CDS=158-916

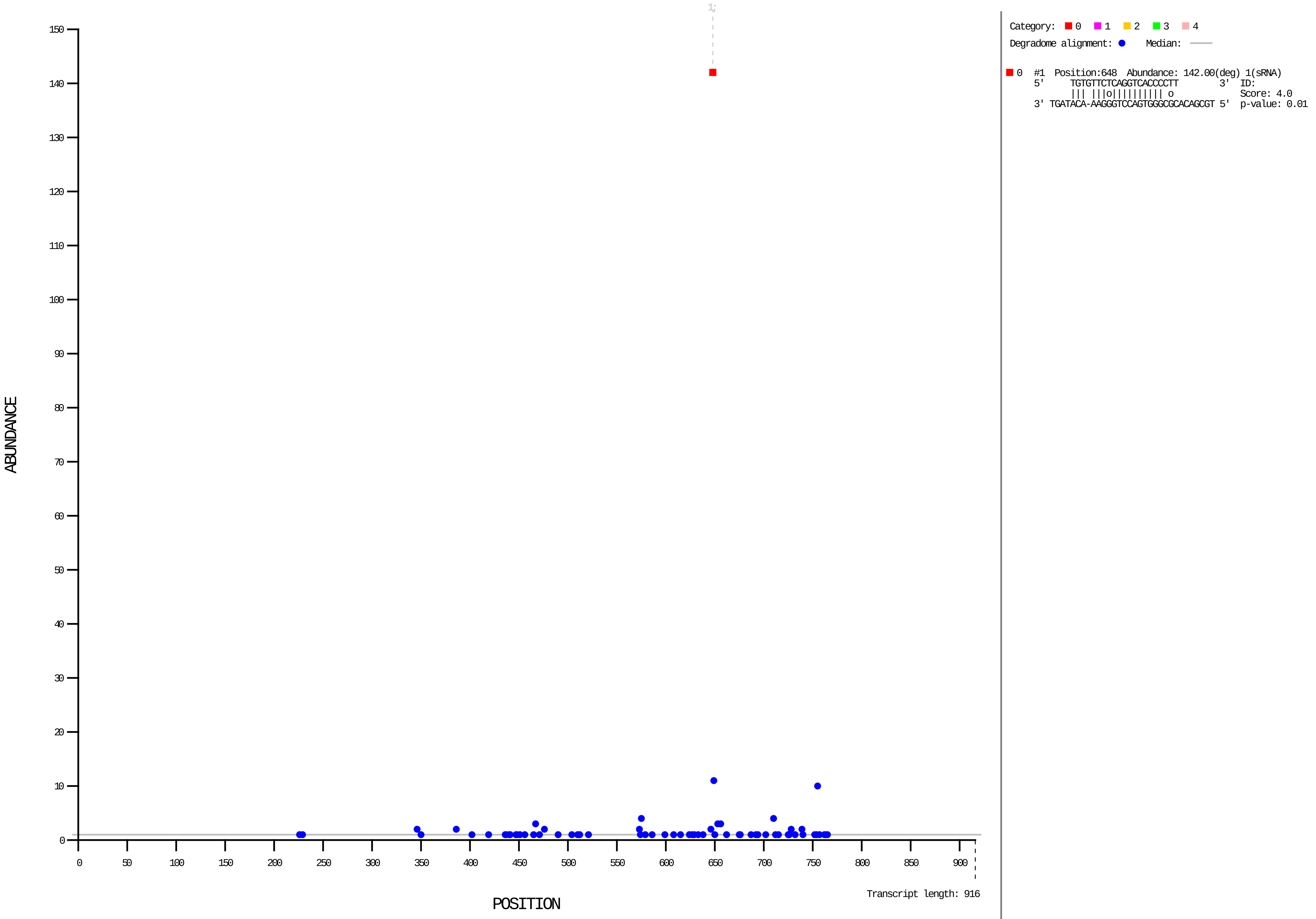

Cs7g10850.1 gene=Cs7g10850 CDS=1-1407

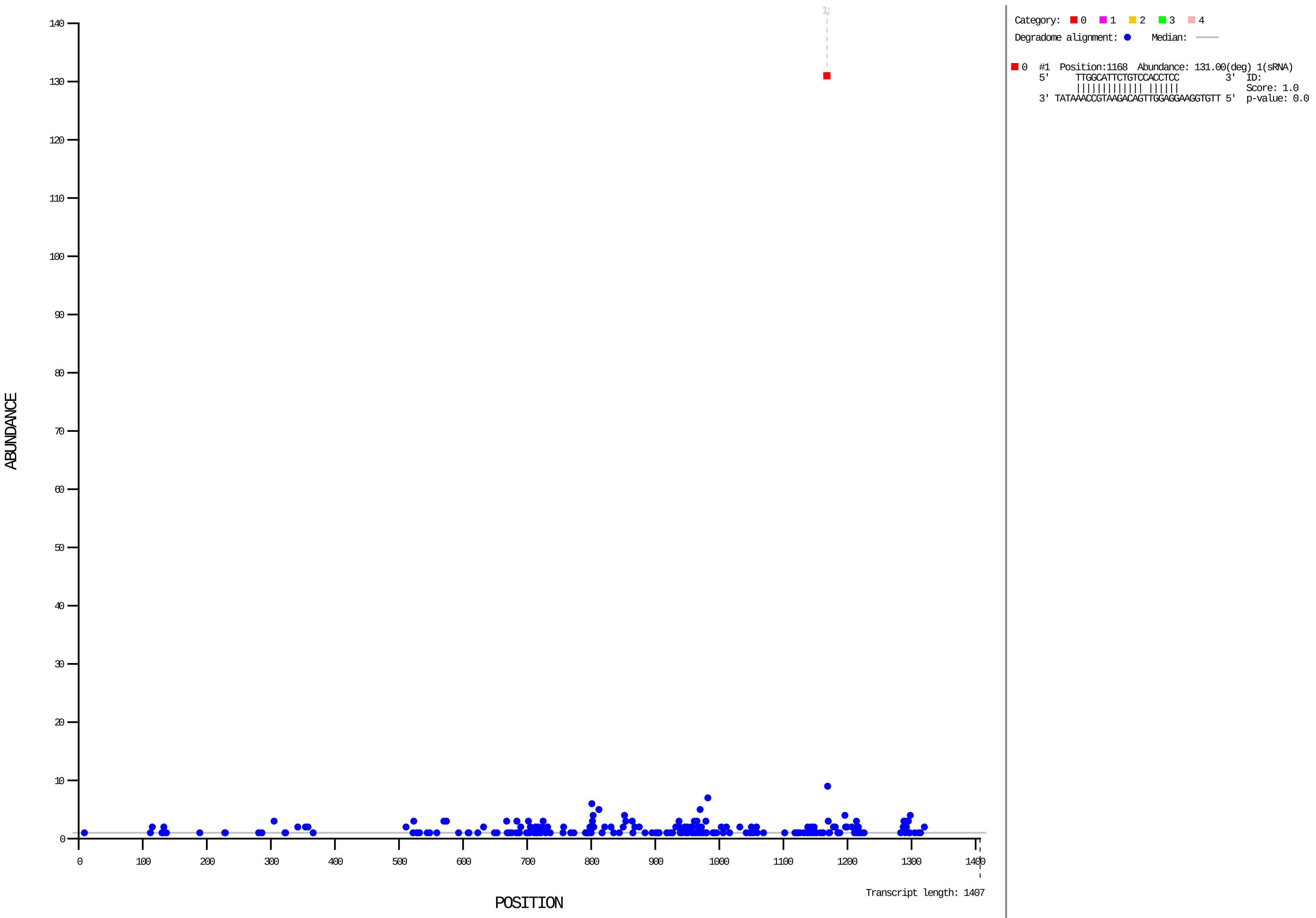

Cs6g21520.1 gene=Cs6g21520 CDS=283-1329

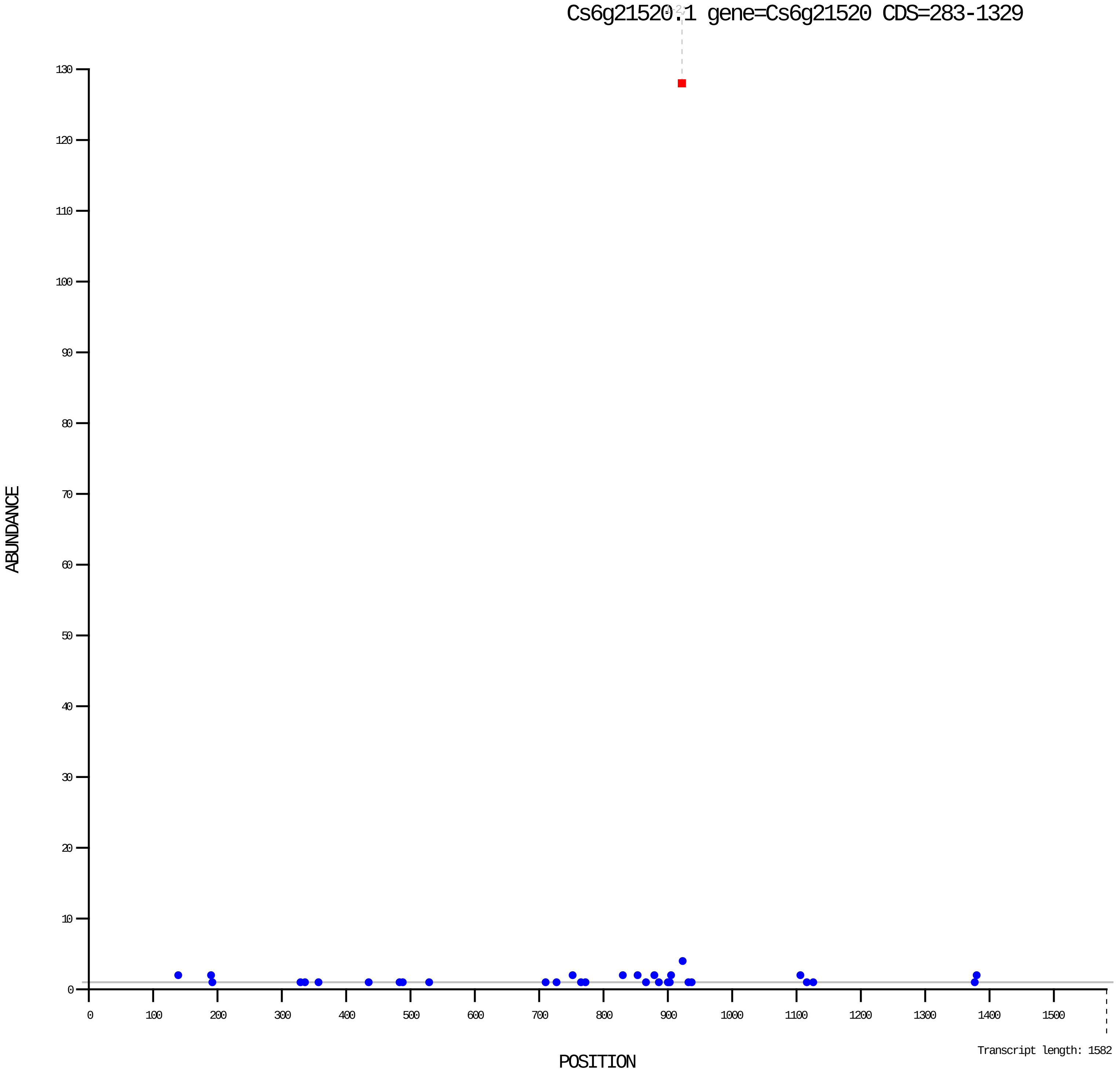

Category: 0 1 2 3 4

Degradome alignment: ● Median:

```
0  #1  Position:922  Abundance: 128.00(deg) 1(sRNA)
   5'     TGGAGAAGCAGGGCACGTGCA         3'  ID:
          o||||||||||| ||||||| |            Score: 2.5
   3' CGTAGCCTCTTCGTCCAGTGCAC-TCTCCAAA 5'  p-value: 0.0

0  #2  Position:922  Abundance: 128.00(deg) 1(sRNA)
   5'     TGGAGAAGCAGGGCACATGCT         3'  ID:
          o||||||||||| ||| ||               Score: 4.5
   3' CGTAGCCTCTTCGTCCAGTGCACTCTCCAAAG 5'  p-value: 0.02
```

orange1.1t04055.1 gene=orange1.1t04055 CDS=576-2135

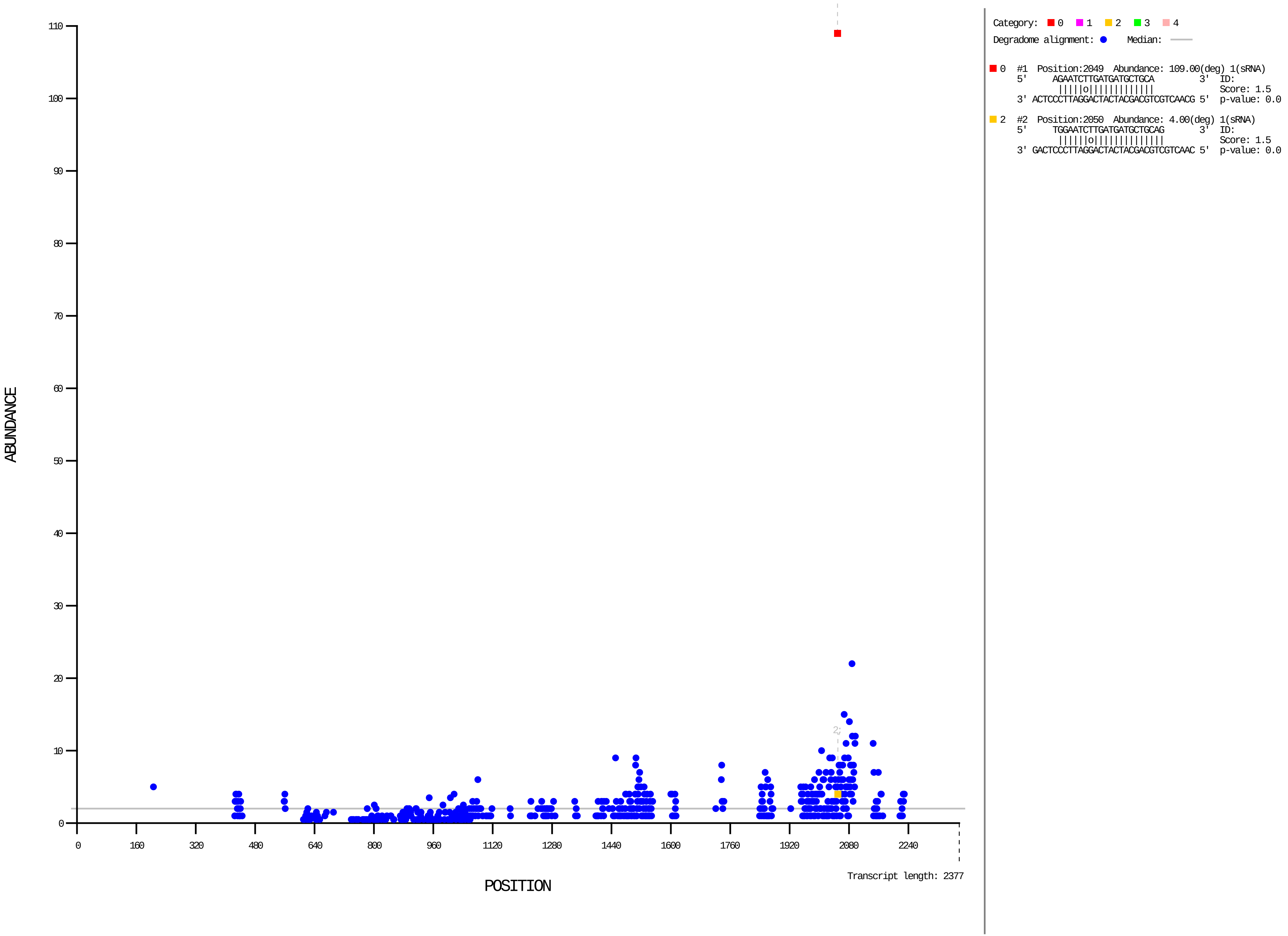

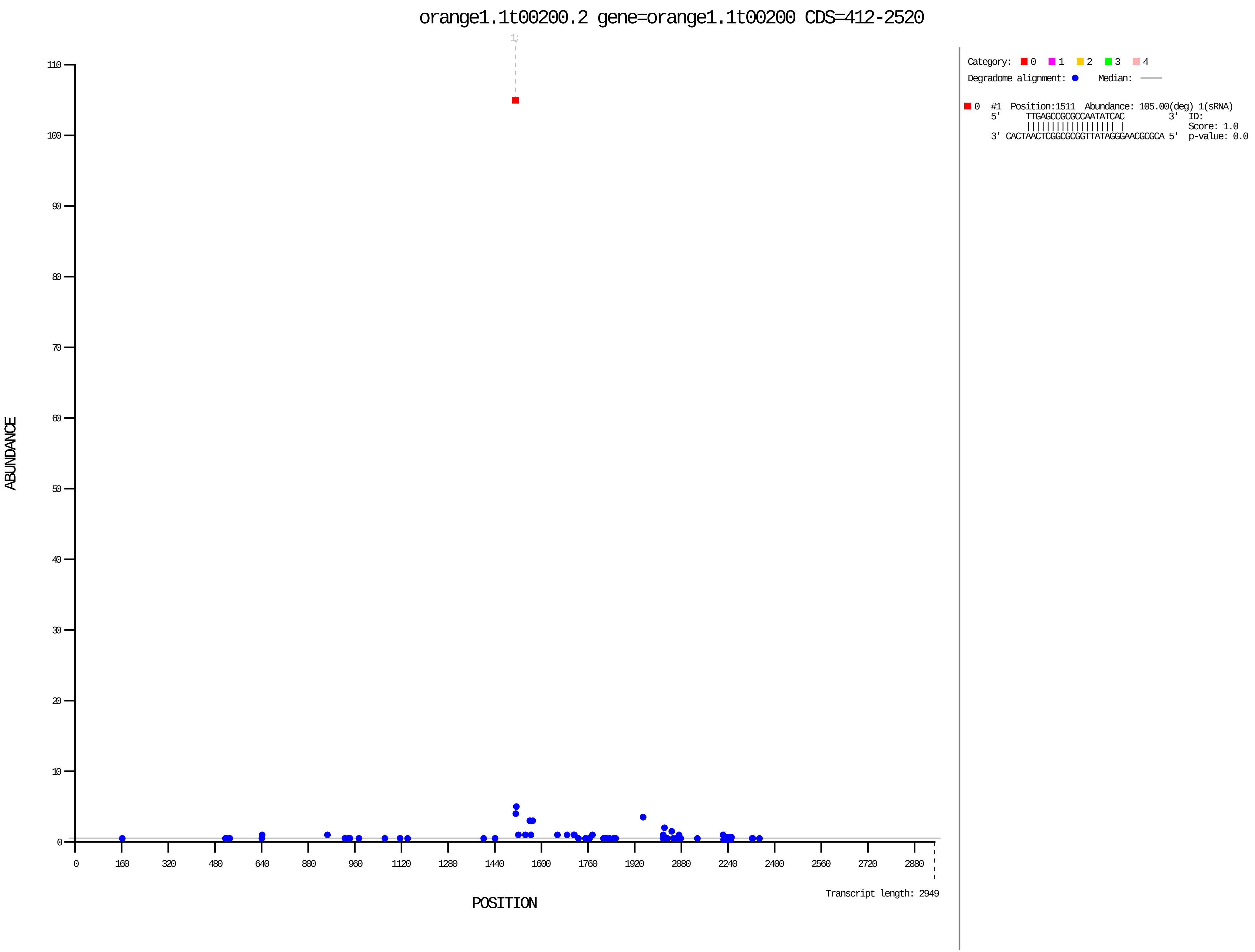

orange1.1t00200.2 gene=orange1.1t00200 CDS=412-2520
ABUNDANCE
POSITION
Transcript length: 2949
Category: 0 1 2 3 4
Degradome alignment:
Median:
0 #1 Position:1511 Abundance: 105.00(deg) 1(sRNA)
5' TTGAGCCGCGCCAATATCAC 3' ID:
Score: 1.0
3' CACTAACTCGGCGCGGTTATAGGGAACGCGCA 5' p-value: 0.0

Cs6g06920.1 gene=Cs6g06920 CDS=1-1737

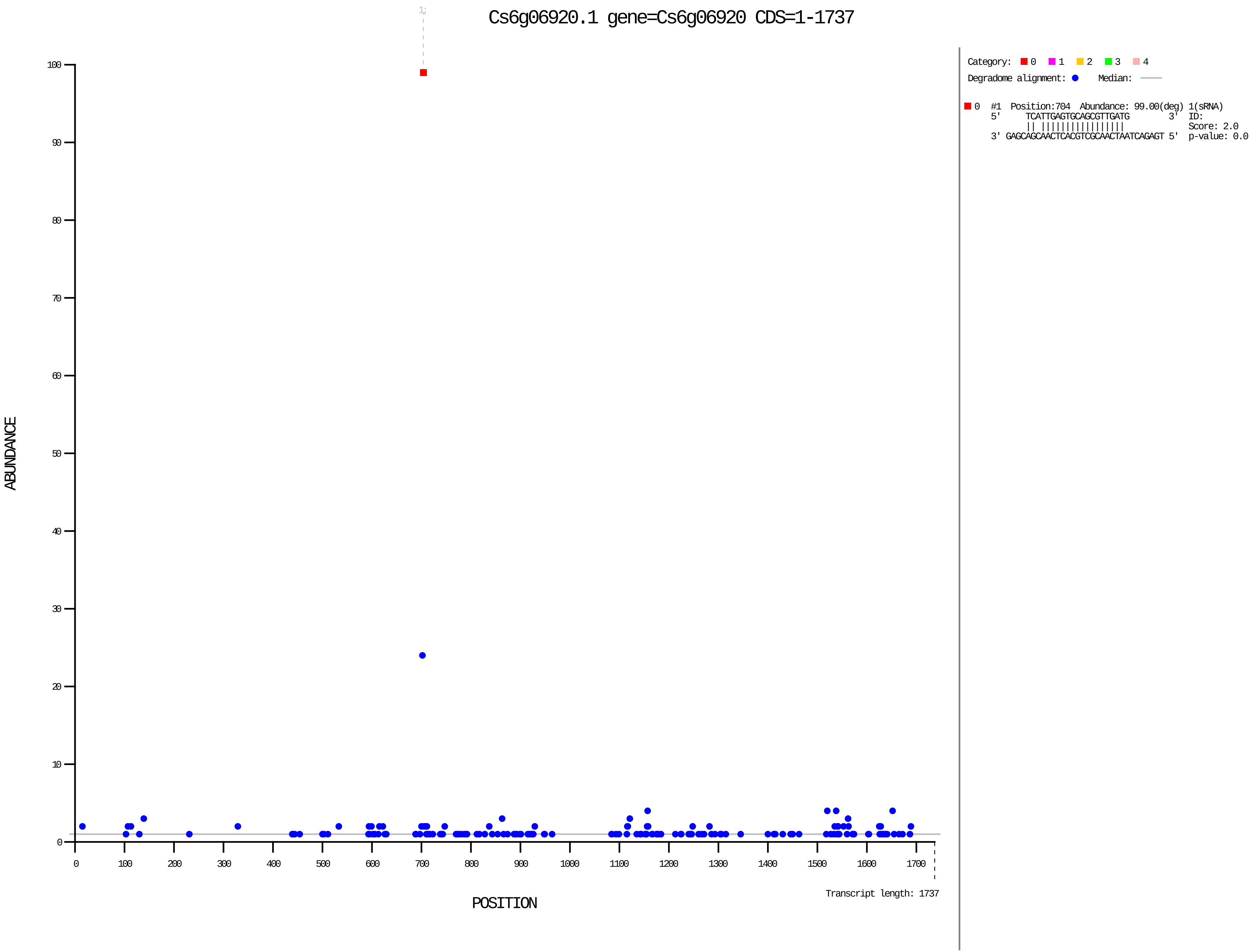

Cs7g31620.1 gene=Cs7g31620 CDS=26-1687

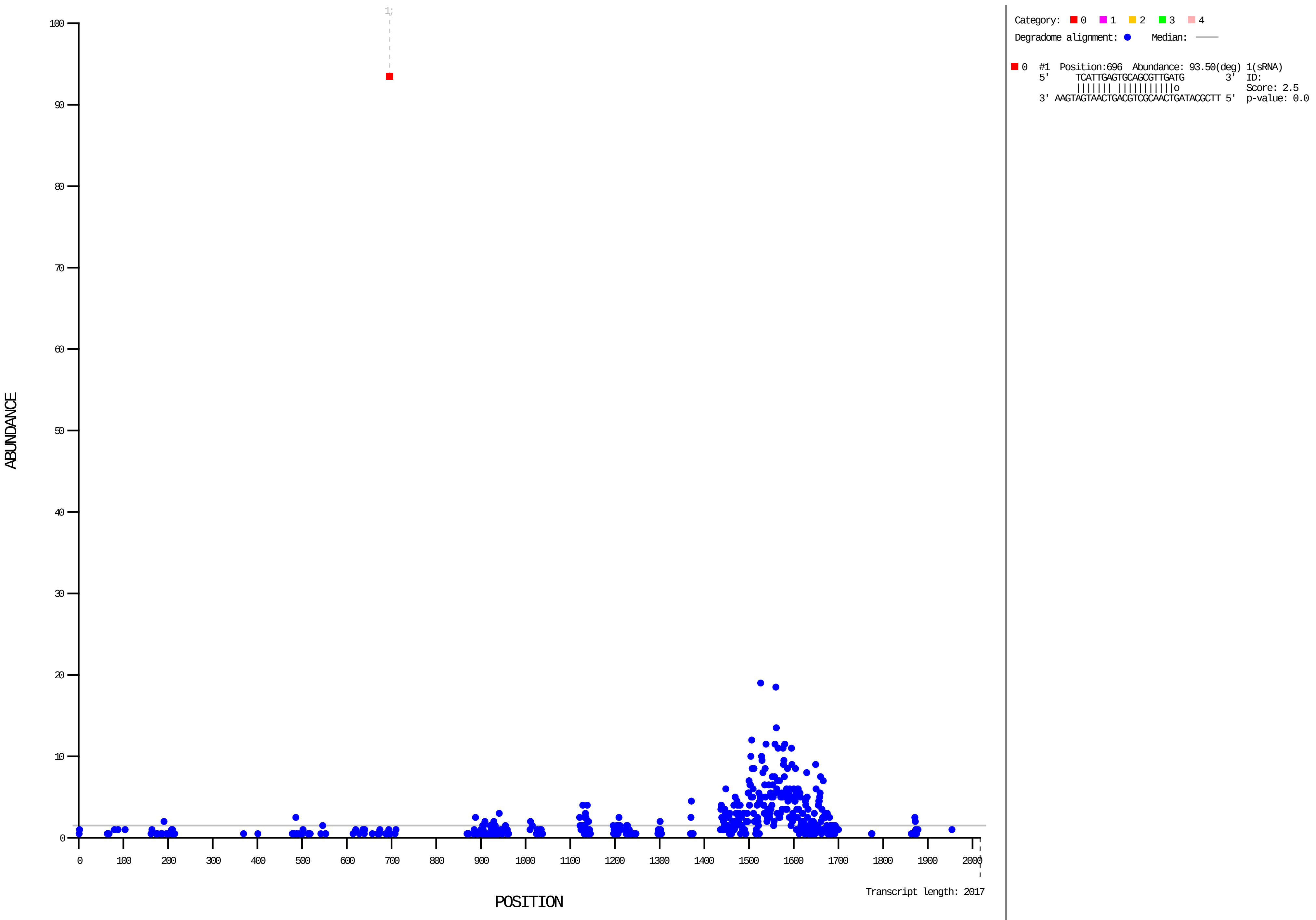

Cs7g31620.2 gene=Cs7g31620 CDS=197-1777

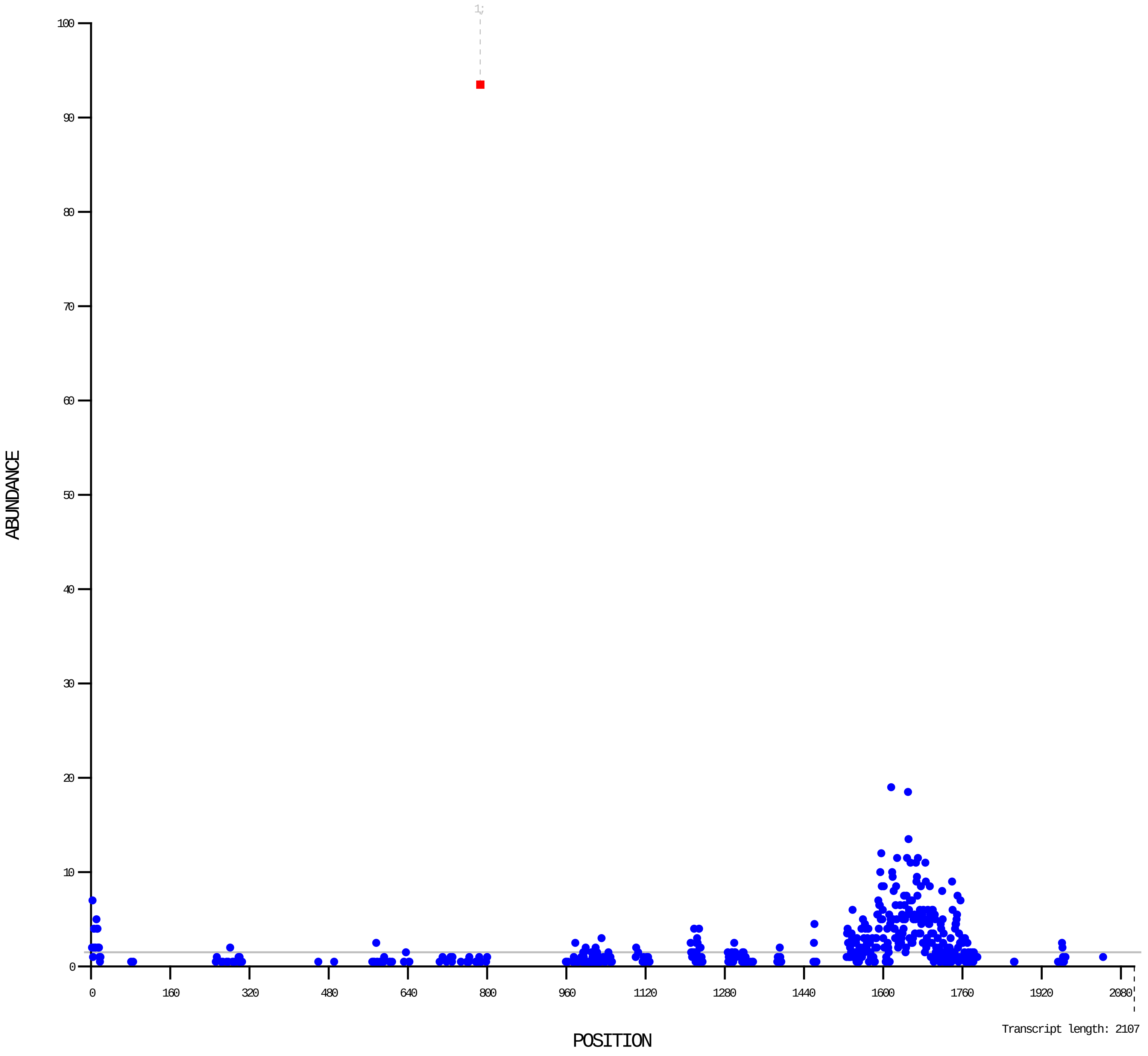

Category: 0 1 2 3 4
Degradome alignment: Median:

```
0   #1  Position:786  Abundance: 93.50(deg) 1(sRNA)
    5'     TCATTGAGTGCAGCGTTGATG        3'  ID:
           ||||||| |||||||||||o             Score: 2.5
    3' AAGTAGTAACTGACGTCGCAACTGATACGCTT 5'  p-value: 0.0
```

Cs2g10760.1 gene=Cs2g10760 CDS=227-3172

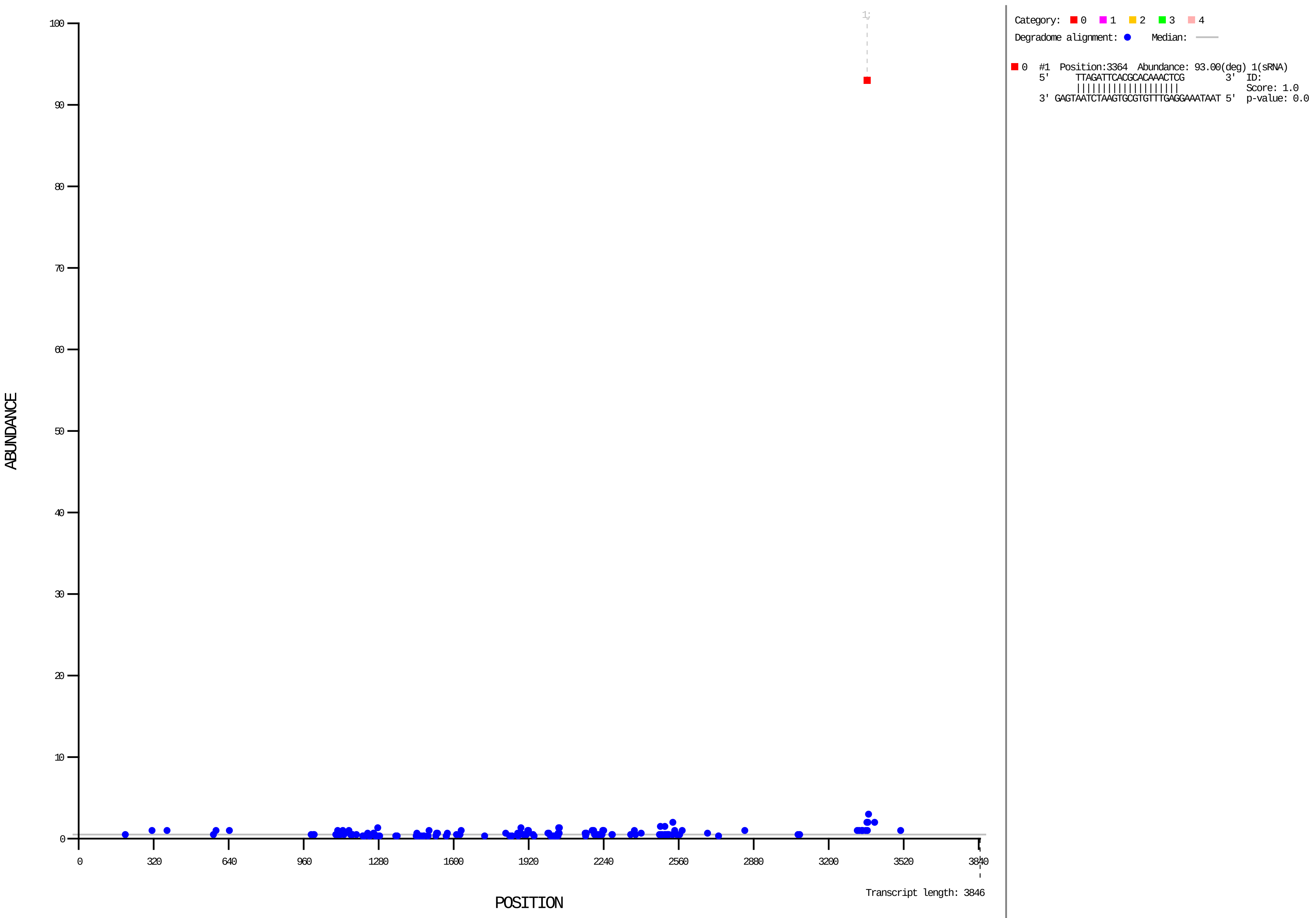

Cs9g06846.1 gene=Cs9g06846 CDS=857-1000

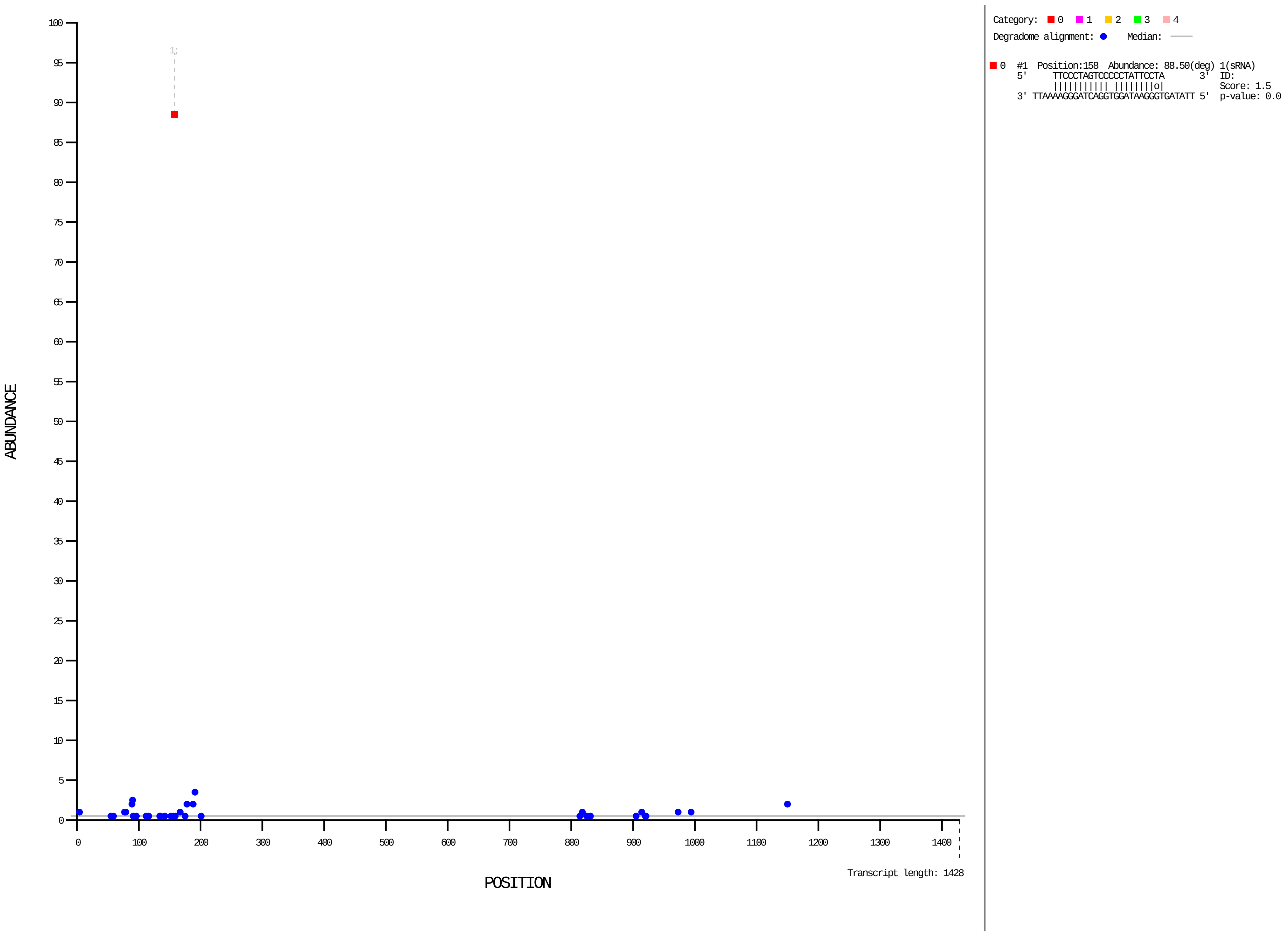

Cs9g06846.2 gene=Cs9g06846 CDS=857-1000

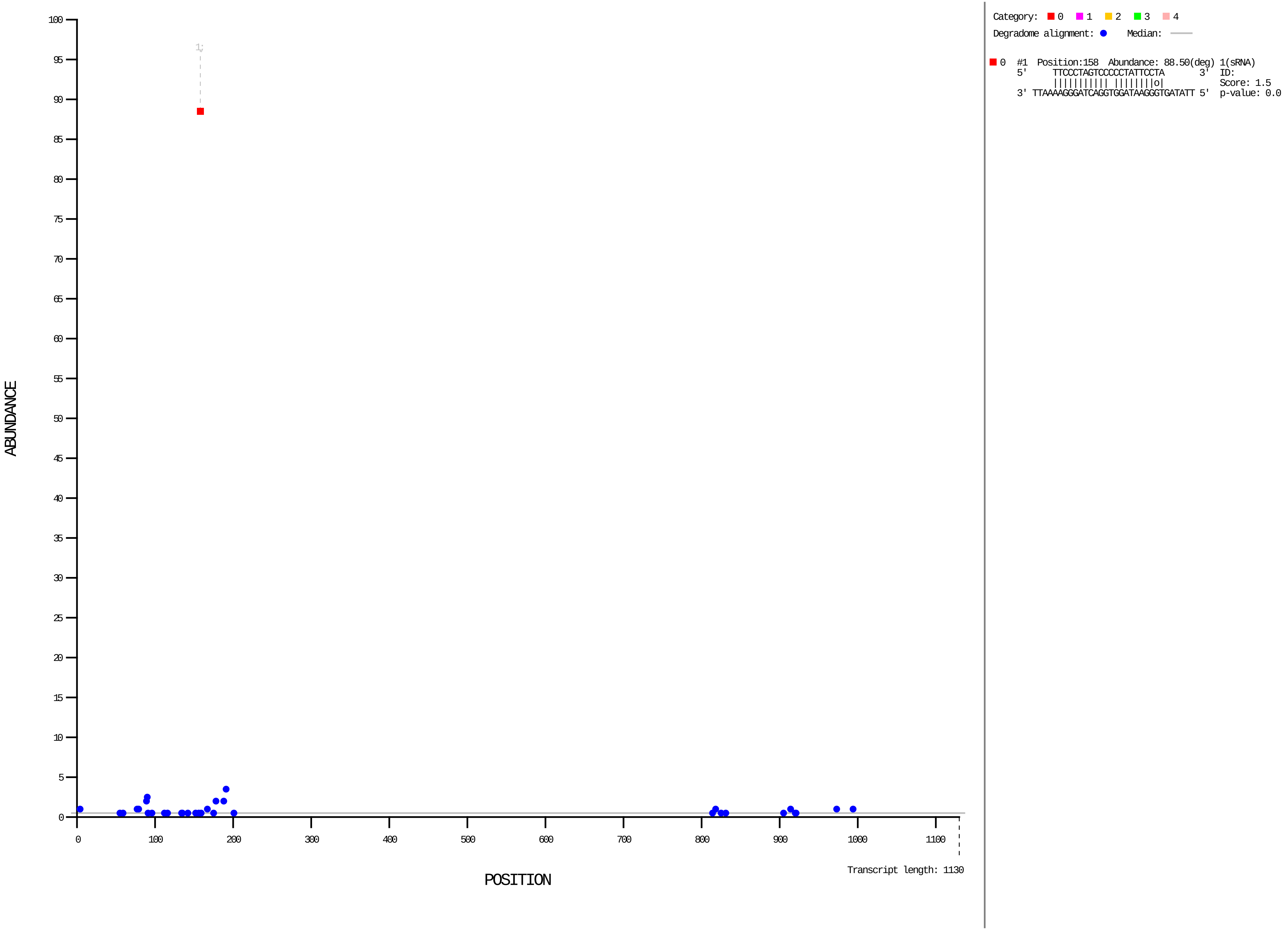

Cs8g16510.3 gene=Cs8g16510 CDS=1500-3476

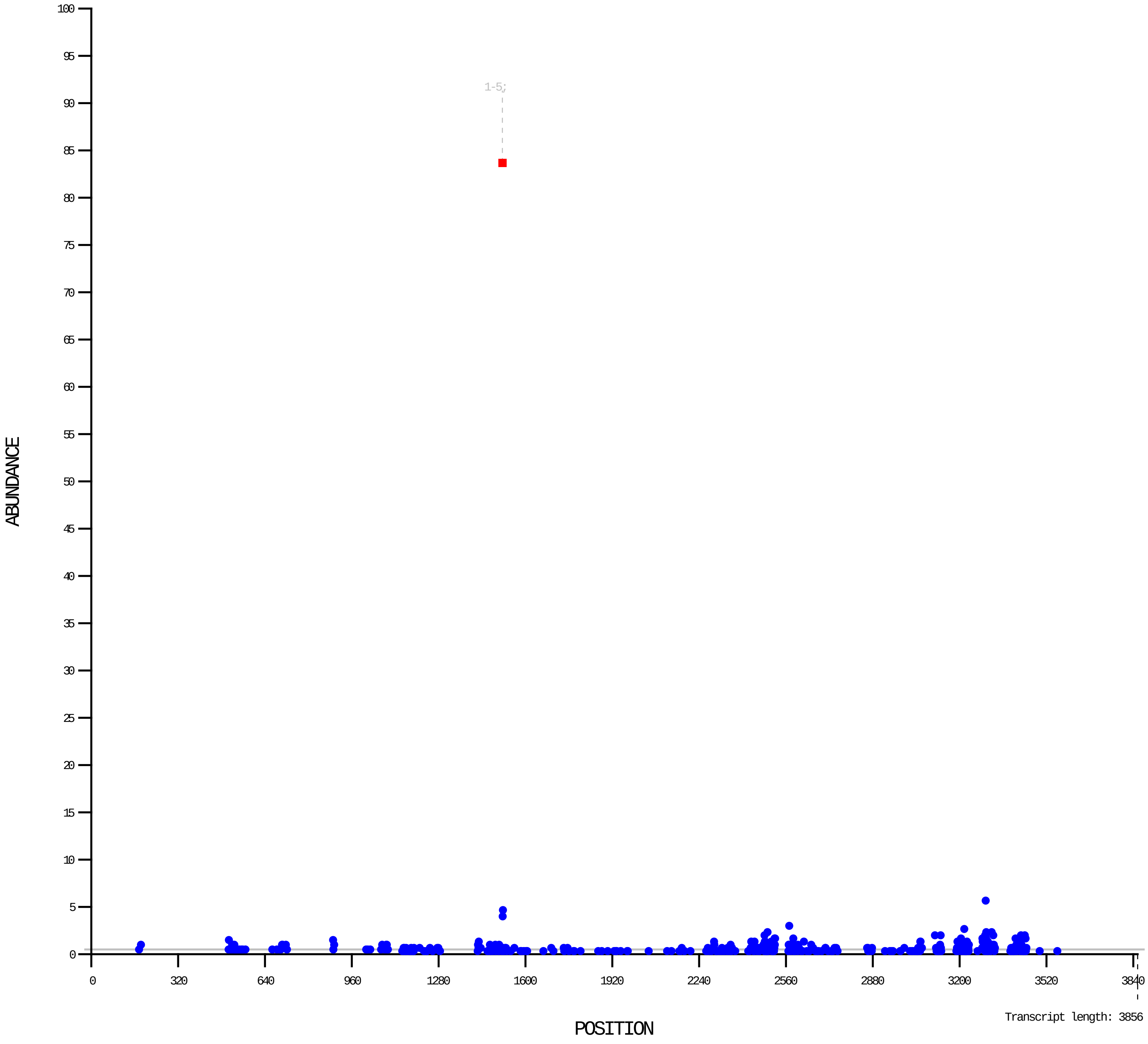


```
Category: 0  1  2  3  4
Degradome alignment:     Median:

0  #1  Position:1515  Abundance: 83.67(deg) 1(sRNA)
   5'     TCGGACCAGGCTTCATTCCC         3'  ID:
          o|||||||||||||||| |||            Score: 1.5
   3' CTTAGGCCTGGTCCGAAGTA-GGGTCCGTAGA 5'  p-value: 0.0

0  #2  Position:1515  Abundance: 83.67(deg) 1(sRNA)
   5'     TCGGACCAGGCTTCATTCCCT        3'  ID:
          o|||||||||||||||| |||            Score: 2.5
   3' CTTAGGCCTGGTCCGAAGTA-GGGTCCGTAGA 5'  p-value: 0.0

0  #3  Position:1515  Abundance: 83.67(deg) 1(sRNA)
   5'     TCGGACCAGGCTTCATTCCCC        3'  ID:
          o|||||||||||||||| |||            Score: 2.5
   3' CTTAGGCCTGGTCCGAAGTA-GGGTCCGTAGA 5'  p-value: 0.0

0  #4  Position:1515  Abundance: 83.67(deg) 1(sRNA)
   5'     TCGGACCAGGCTTCATTCCCGT       3'  ID:
          o||||||||||||||||o|| |           Score: 3.0
   3' CTTAGGCCTGGTCCGAAGTAGGGTCCGTAGAC 5'  p-value: 0.0

0  #5  Position:1515  Abundance: 83.67(deg) 1(sRNA)
   5'     TCGGACCAGGCTTCATTCCCCC       3'  ID:
          o|||||||||||||||| |||            Score: 3.5
   3' CTTAGGCCTGGTCCGAAGTA-GGGTCCGTAGA 5'  p-value: 0.0
```

Cs8g16510.2 gene=Cs8g16510 CDS=184-2415

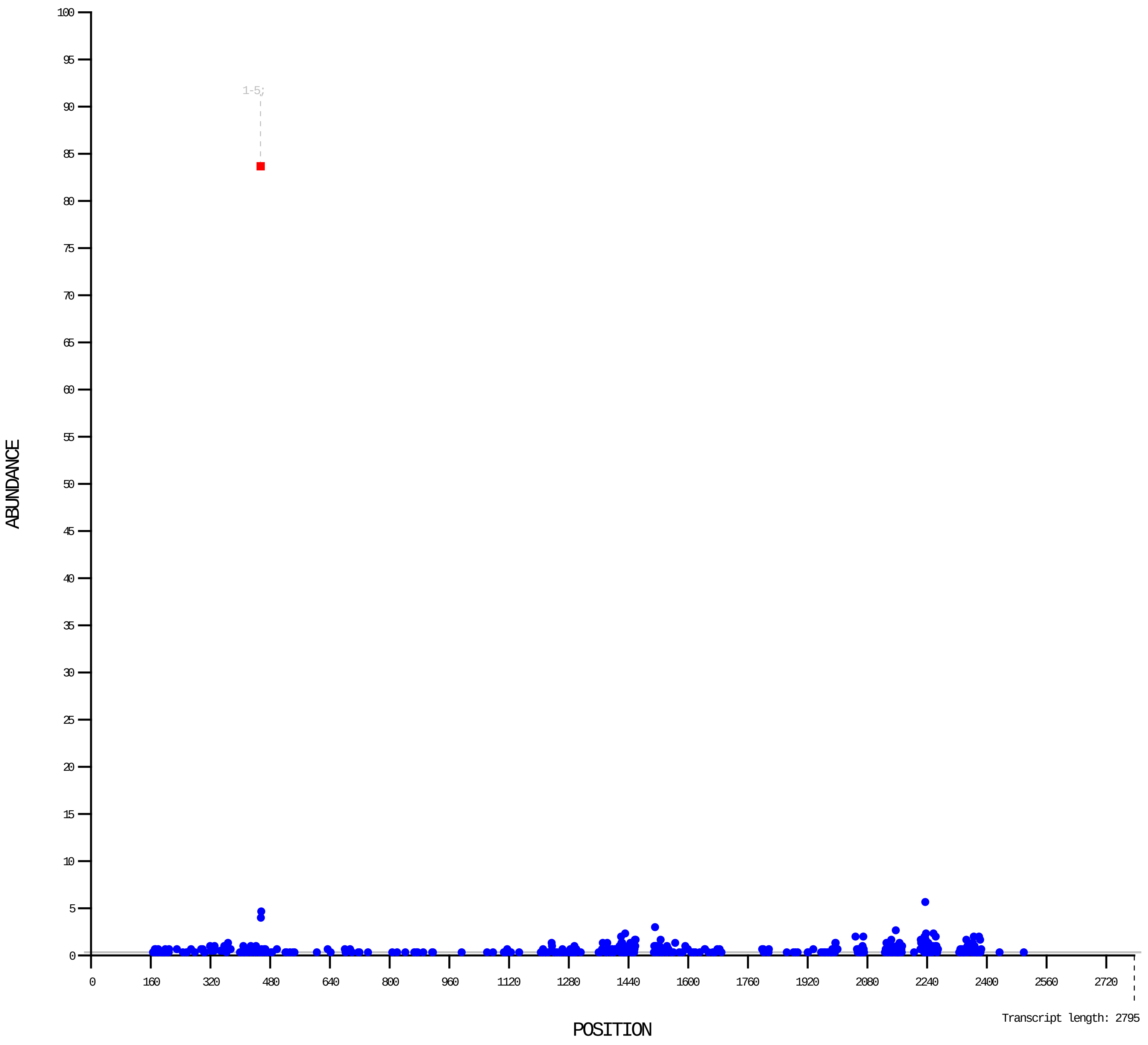

0 #1 Position:454 Abundance: 83.67(deg) 1(sRNA)
```
5'      TCGGACCAGGCTTCATTCCC        3'  ID:
        o||||||||||||||| |||            Score: 1.5
3' CTTAGGCCTGGTCCGAAGTA-GGGTCCGTAGA 5'  p-value: 0.0
```

0 #2 Position:454 Abundance: 83.67(deg) 1(sRNA)
```
5'      TCGGACCAGGCTTCATTCCCT       3'  ID:
        o||||||||||||||| |||            Score: 2.5
3' CTTAGGCCTGGTCCGAAGTA-GGGTCCGTAGA 5'  p-value: 0.0
```

0 #3 Position:454 Abundance: 83.67(deg) 1(sRNA)
```
5'      TCGGACCAGGCTTCATTCCCC       3'  ID:
        o||||||||||||||| |||            Score: 2.5
3' CTTAGGCCTGGTCCGAAGTA-GGGTCCGTAGA 5'  p-value: 0.0
```

0 #4 Position:454 Abundance: 83.67(deg) 1(sRNA)
```
5'      TCGGACCAGGCTTCATTCCCGT      3'  ID:
        o|||||||||||||||o|| |           Score: 3.0
3' CTTAGGCCTGGTCCGAAGTAGGGTCCGTAGAC 5'  p-value: 0.0
```

0 #5 Position:454 Abundance: 83.67(deg) 1(sRNA)
```
5'      TCGGACCAGGCTTCATTCCCCC      3'  ID:
        o||||||||||||||| |||            Score: 3.5
3' CTTAGGCCTGGTCCGAAGTA-GGGTCCGTAGA 5'  p-value: 0.0
```

Cs8g16510.1 gene=Cs8g16510 CDS=855-3395

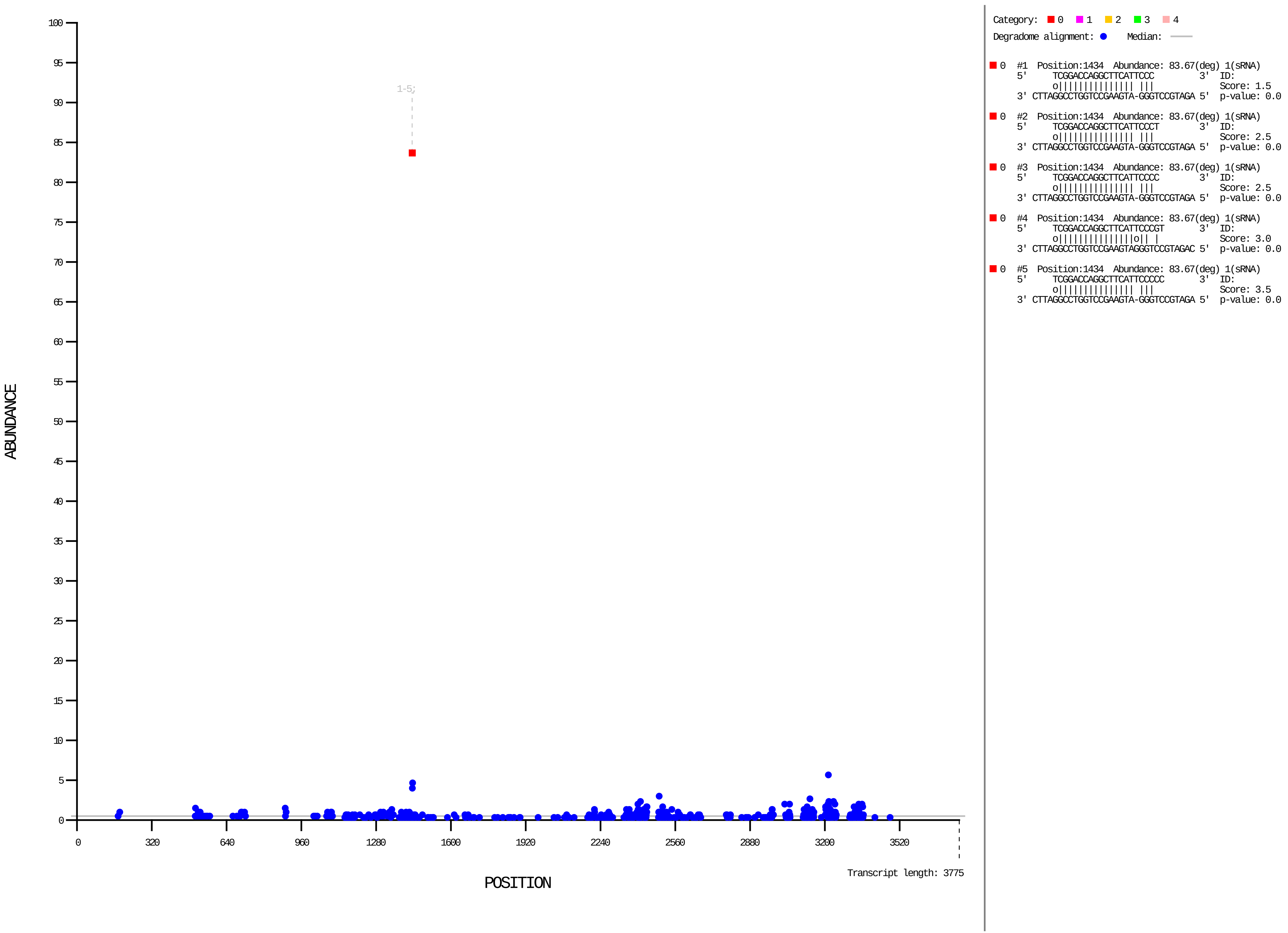

Cs1g15640.3 gene=Cs1g15640 CDS=1130-3646

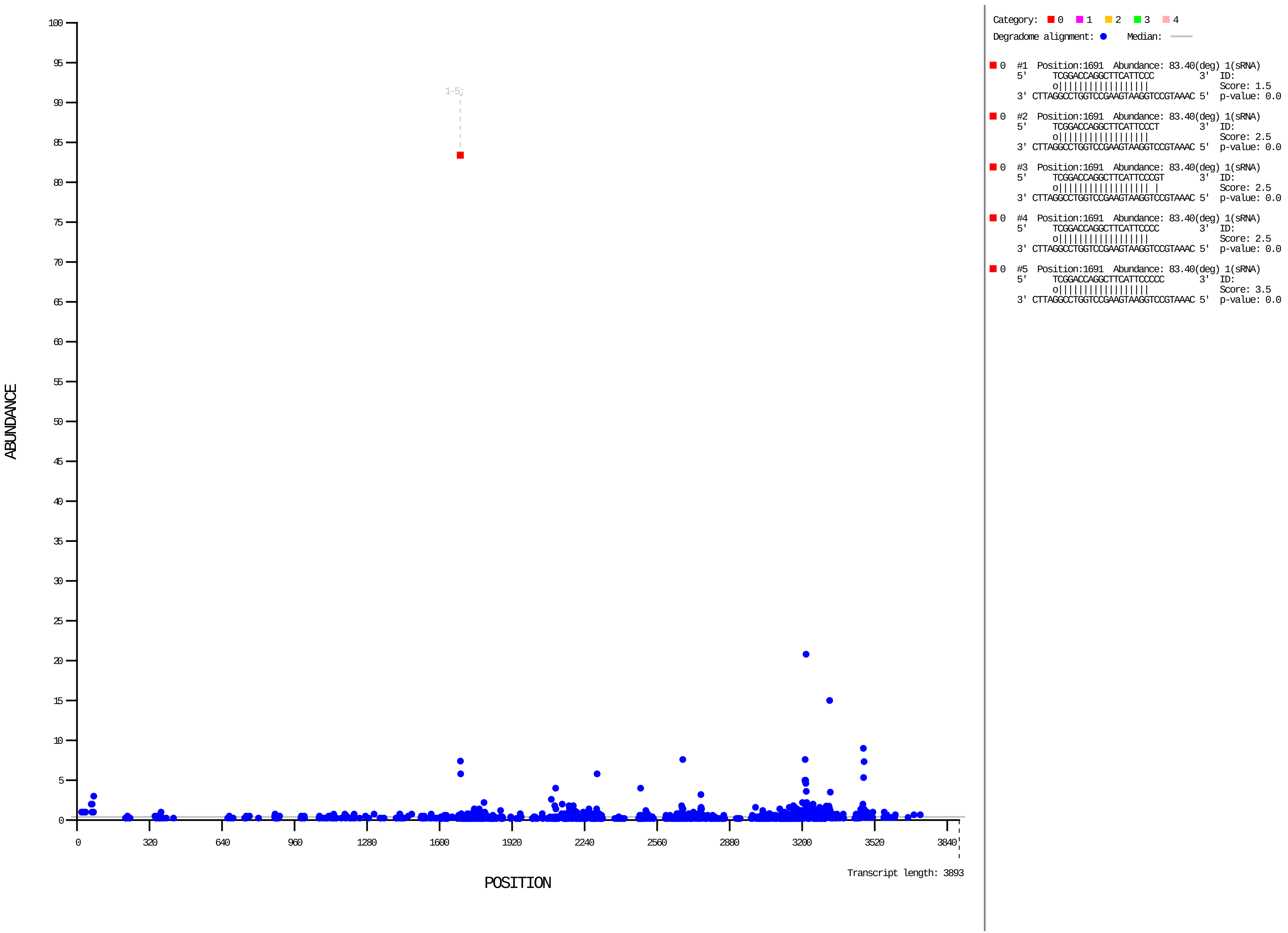

Cs1g15640.1 gene=Cs1g15640 CDS=1220-3736

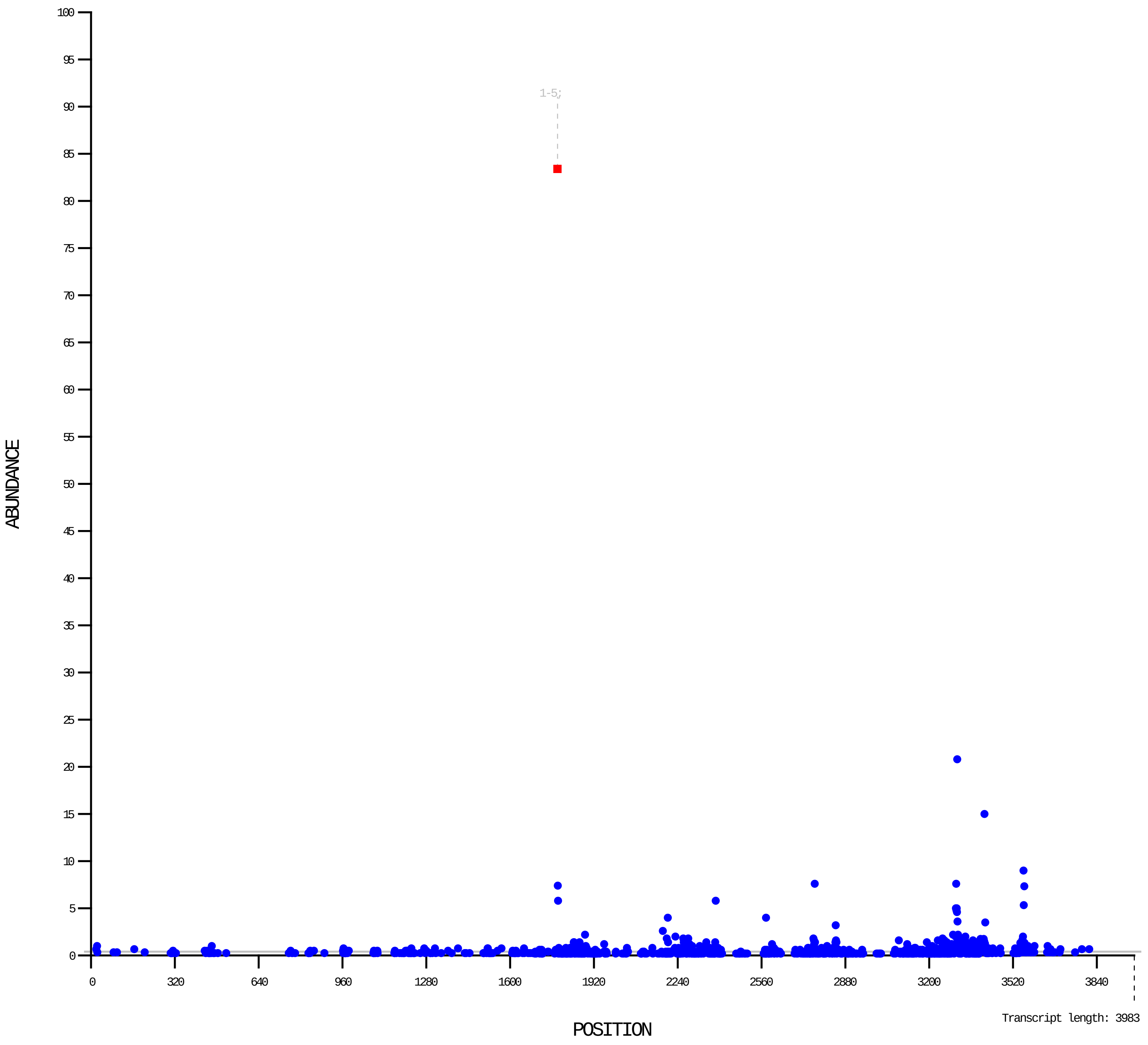

Category: 0 1 2 3 4
Degradome alignment: ● Median:

■ 0 #1 Position:1781 Abundance: 83.40(deg) 1(sRNA)
```
5'     TCGGACCAGGCTTCATTCCC        3'  ID:
       o|||||||||||||||||||            Score: 1.5
3' CTTAGGCCTGGTCCGAAGTAAGGTCCGTAAAC 5'  p-value: 0.0
```

■ 0 #2 Position:1781 Abundance: 83.40(deg) 1(sRNA)
```
5'     TCGGACCAGGCTTCATTCCCT       3'  ID:
       o|||||||||||||||||||            Score: 2.5
3' CTTAGGCCTGGTCCGAAGTAAGGTCCGTAAAC 5'  p-value: 0.0
```

■ 0 #3 Position:1781 Abundance: 83.40(deg) 1(sRNA)
```
5'     TCGGACCAGGCTTCATTCCCGT      3'  ID:
       o||||||||||||||||||| |          Score: 2.5
3' CTTAGGCCTGGTCCGAAGTAAGGTCCGTAAAC 5'  p-value: 0.0
```

■ 0 #4 Position:1781 Abundance: 83.40(deg) 1(sRNA)
```
5'     TCGGACCAGGCTTCATTCCCC       3'  ID:
       o|||||||||||||||||||            Score: 2.5
3' CTTAGGCCTGGTCCGAAGTAAGGTCCGTAAAC 5'  p-value: 0.0
```

■ 0 #5 Position:1781 Abundance: 83.40(deg) 1(sRNA)
```
5'     TCGGACCAGGCTTCATTCCCCC      3'  ID:
       o|||||||||||||||||||            Score: 3.5
3' CTTAGGCCTGGTCCGAAGTAAGGTCCGTAAAC 5'  p-value: 0.0
```

Cs1g15640.4 gene=Cs1g15640 CDS=1220-3505

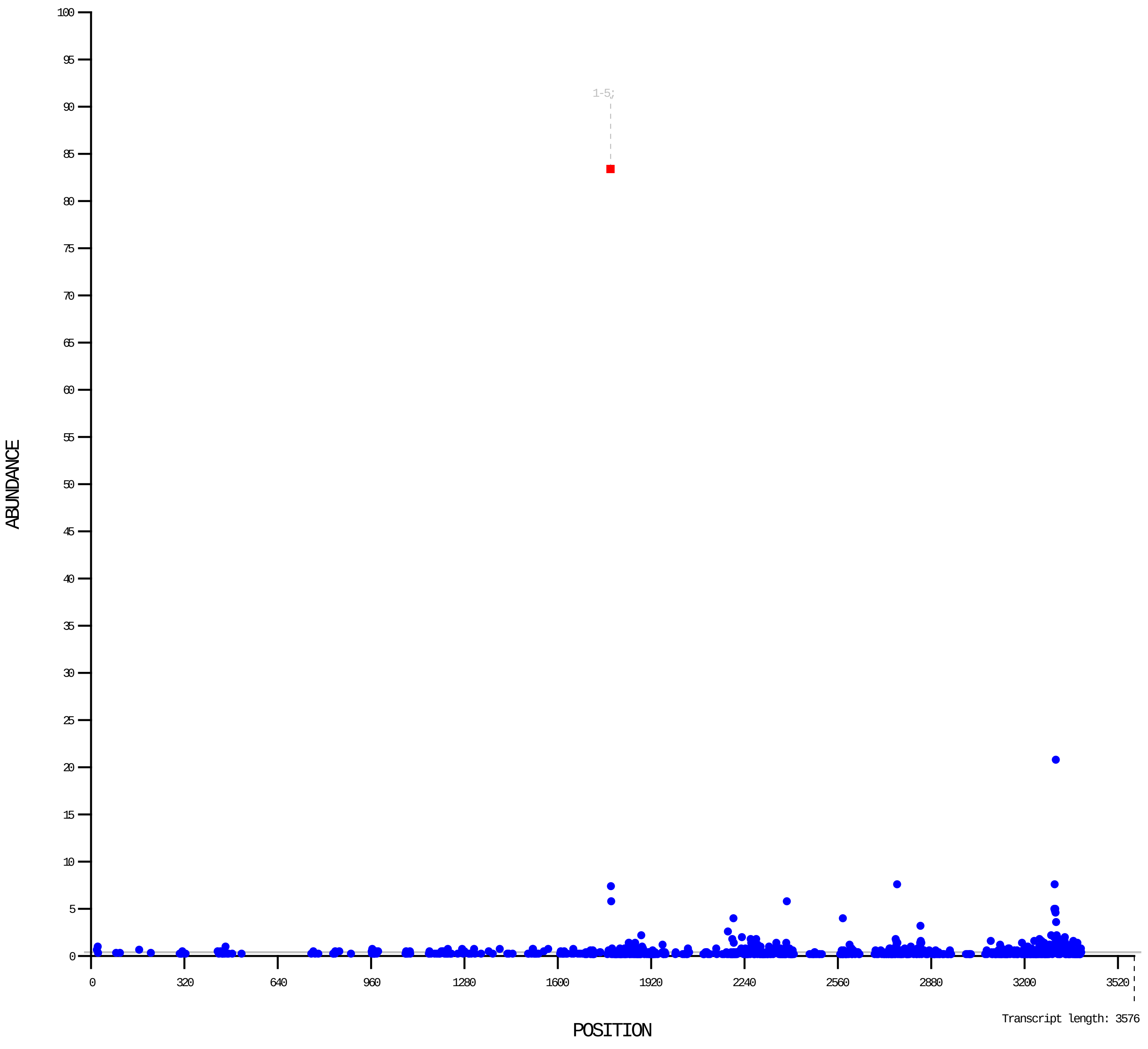

Category: 0 1 2 3 4

Degradome alignment: ● Median: —

```
0  #1  Position:1781  Abundance: 83.40(deg) 1(sRNA)
   5'     TCGGACCAGGCTTCATTCCC          3'  ID:
          o||||||||||||||||||               Score: 1.5
   3' CTTAGGCCTGGTCCGAAGTAAGGTCCGTAAAC 5'  p-value: 0.0

0  #2  Position:1781  Abundance: 83.40(deg) 1(sRNA)
   5'     TCGGACCAGGCTTCATTCCCT         3'  ID:
          o||||||||||||||||||               Score: 2.5
   3' CTTAGGCCTGGTCCGAAGTAAGGTCCGTAAAC 5'  p-value: 0.0

0  #3  Position:1781  Abundance: 83.40(deg) 1(sRNA)
   5'     TCGGACCAGGCTTCATTCCCGT        3'  ID:
          o|||||||||||||||||| |             Score: 2.5
   3' CTTAGGCCTGGTCCGAAGTAAGGTCCGTAAAC 5'  p-value: 0.0

0  #4  Position:1781  Abundance: 83.40(deg) 1(sRNA)
   5'     TCGGACCAGGCTTCATTCCCC         3'  ID:
          o||||||||||||||||||               Score: 2.5
   3' CTTAGGCCTGGTCCGAAGTAAGGTCCGTAAAC 5'  p-value: 0.0

0  #5  Position:1781  Abundance: 83.40(deg) 1(sRNA)
   5'     TCGGACCAGGCTTCATTCCCCC        3'  ID:
          o||||||||||||||||||               Score: 3.5
   3' CTTAGGCCTGGTCCGAAGTAAGGTCCGTAAAC 5'  p-value: 0.0
```

Cs1g15640.5 gene=Cs1g15640 CDS=121-2091

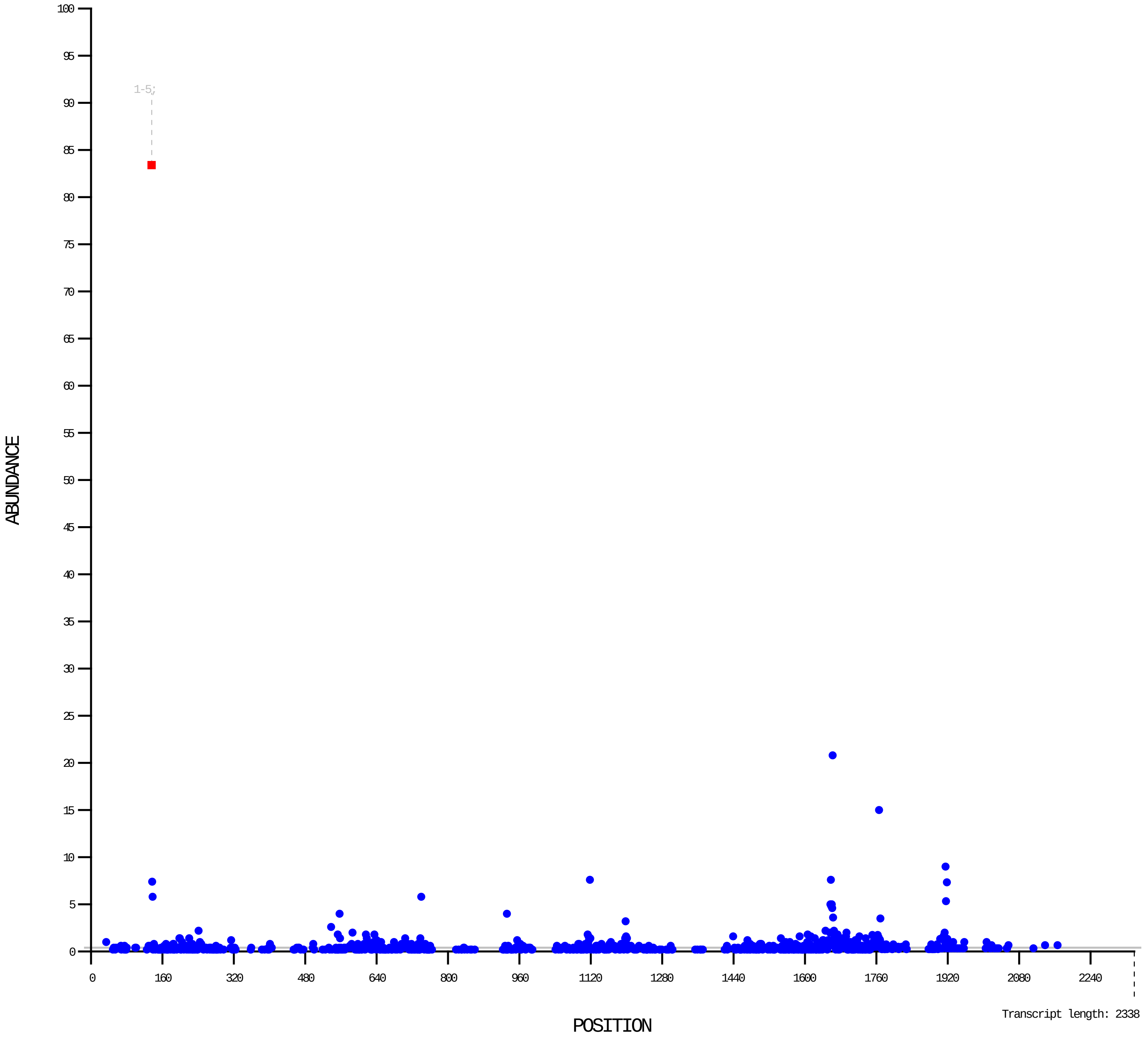


```
Category: ■ 0 ■ 1 ■ 2 ■ 3 ■ 4
Degradome alignment: ●    Median: ——

■ 0  #1  Position:136  Abundance: 83.40(deg) 1(sRNA)
     5'     TCGGACCAGGCTTCATTCCC        3'  ID:
            o|||||||||||||||||||            Score: 1.5
     3' CTTAGGCCTGGTCCGAAGTAAGGTCCGTAAAC 5'  p-value: 0.0

■ 0  #2  Position:136  Abundance: 83.40(deg) 1(sRNA)
     5'     TCGGACCAGGCTTCATTCCCT       3'  ID:
            o|||||||||||||||||||            Score: 2.5
     3' CTTAGGCCTGGTCCGAAGTAAGGTCCGTAAAC 5'  p-value: 0.0

■ 0  #3  Position:136  Abundance: 83.40(deg) 1(sRNA)
     5'     TCGGACCAGGCTTCATTCCCGT      3'  ID:
            o||||||||||||||||||| |          Score: 2.5
     3' CTTAGGCCTGGTCCGAAGTAAGGTCCGTAAAC 5'  p-value: 0.0

■ 0  #4  Position:136  Abundance: 83.40(deg) 1(sRNA)
     5'     TCGGACCAGGCTTCATTCCCC       3'  ID:
            o|||||||||||||||||||            Score: 2.5
     3' CTTAGGCCTGGTCCGAAGTAAGGTCCGTAAAC 5'  p-value: 0.0

■ 0  #5  Position:136  Abundance: 83.40(deg) 1(sRNA)
     5'     TCGGACCAGGCTTCATTCCCCC      3'  ID:
            o|||||||||||||||||||            Score: 3.5
     3' CTTAGGCCTGGTCCGAAGTAAGGTCCGTAAAC 5'  p-value: 0.0
```

Cs1g15640.2 gene=Cs1g15640 CDS=1220-3649

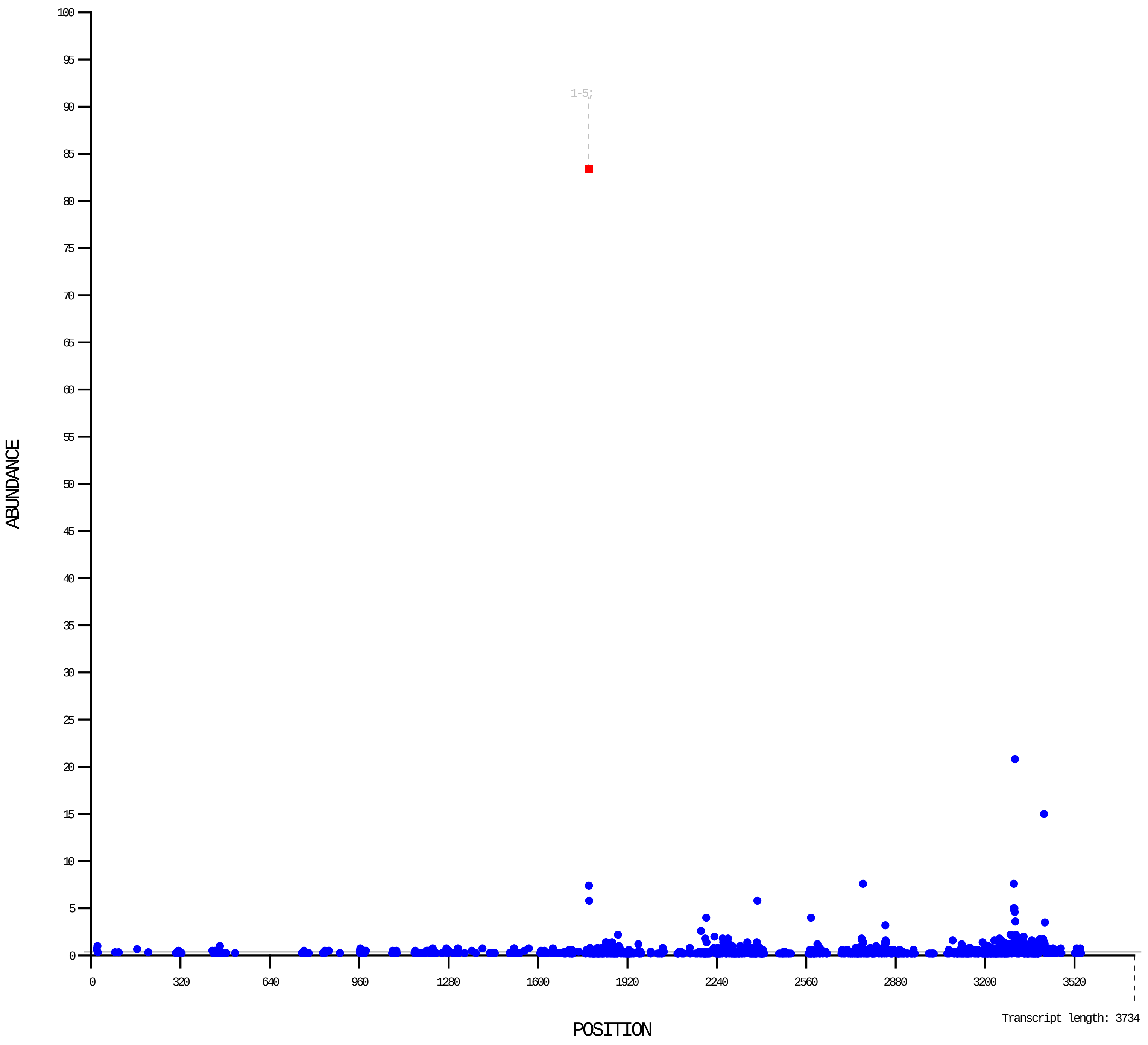

Category: 0 1 2 3 4

Degradome alignment: ● Median: —

```
0  #1  Position:1781  Abundance: 83.40(deg) 1(sRNA)
   5'      TCGGACCAGGCTTCATTCCC        3'  ID:
           o||||||||||||||||||             Score: 1.5
   3' CTTAGGCCTGGTCCGAAGTAAGGTCCGTAAAC 5'  p-value: 0.0

0  #2  Position:1781  Abundance: 83.40(deg) 1(sRNA)
   5'      TCGGACCAGGCTTCATTCCCT       3'  ID:
           o||||||||||||||||||             Score: 2.5
   3' CTTAGGCCTGGTCCGAAGTAAGGTCCGTAAAC 5'  p-value: 0.0

0  #3  Position:1781  Abundance: 83.40(deg) 1(sRNA)
   5'      TCGGACCAGGCTTCATTCCCGT      3'  ID:
           o|||||||||||||||||| |           Score: 2.5
   3' CTTAGGCCTGGTCCGAAGTAAGGTCCGTAAAC 5'  p-value: 0.0

0  #4  Position:1781  Abundance: 83.40(deg) 1(sRNA)
   5'      TCGGACCAGGCTTCATTCCCC       3'  ID:
           o||||||||||||||||||             Score: 2.5
   3' CTTAGGCCTGGTCCGAAGTAAGGTCCGTAAAC 5'  p-value: 0.0

0  #5  Position:1781  Abundance: 83.40(deg) 1(sRNA)
   5'      TCGGACCAGGCTTCATTCCCCC      3'  ID:
           o||||||||||||||||||             Score: 3.5
   3' CTTAGGCCTGGTCCGAAGTAAGGTCCGTAAAC 5'  p-value: 0.0
```

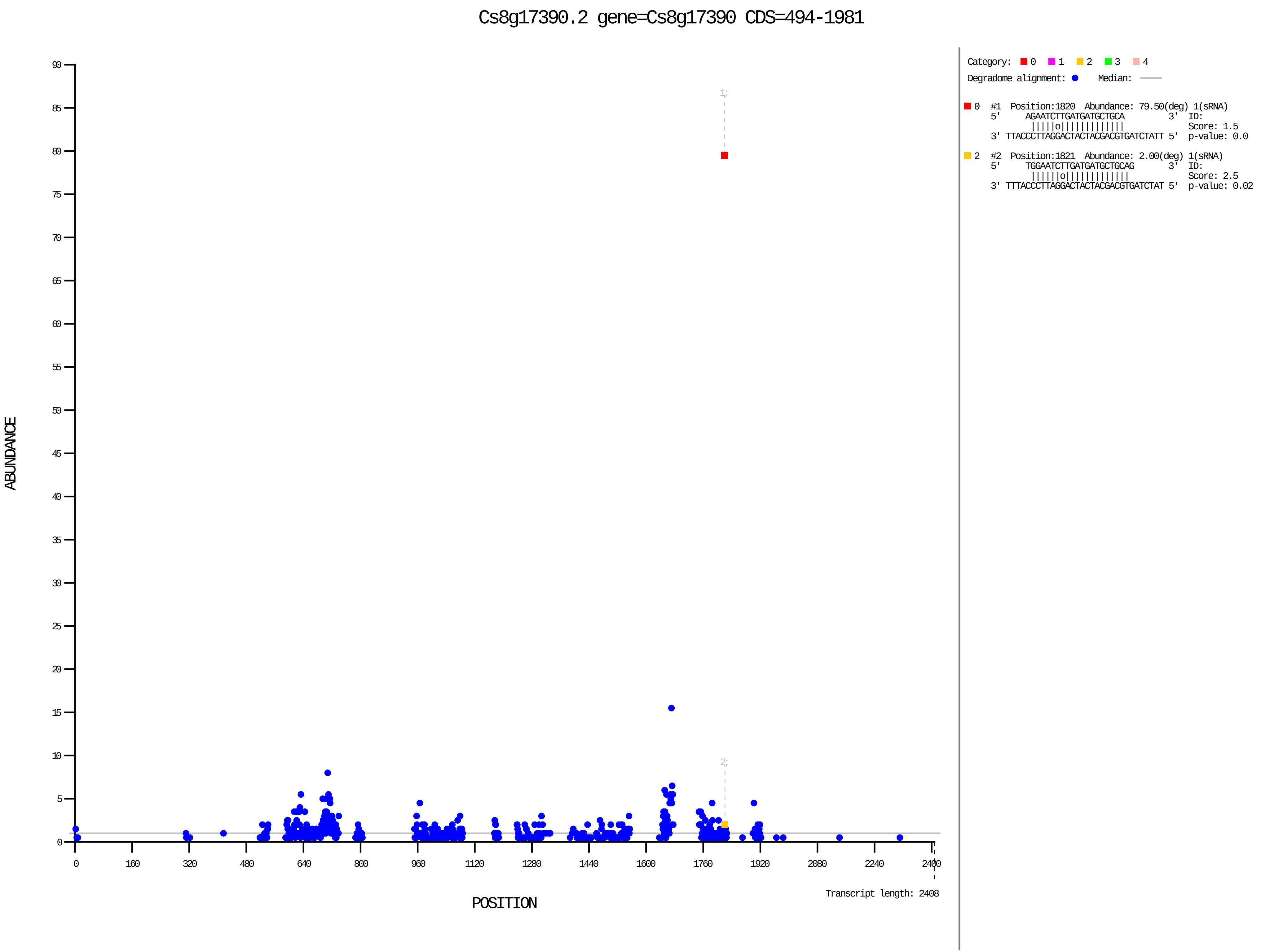
Cs8g17390.2 gene=Cs8g17390 CDS=494-1981
ABUNDANCE
POSITION
Transcript length: 2408
Category: 0 1 2 3 4
Degradome alignment:
Median:
0 #1 Position:1820 Abundance: 79.50(deg) 1(sRNA)
5' AGAATCTTGATGATGCTGCA 3' ID:
|||||o||||||||||||| Score: 1.5
3' TTACCCTTAGGACTACTACGACGTGATCTATT 5' p-value: 0.0
2 #2 Position:1821 Abundance: 2.00(deg) 1(sRNA)
5' TGGAATCTTGATGATGCTGCAG 3' ID:
||||||o||||||||||||| Score: 2.5
3' TTTACCCTTAGGACTACTACGACGTGATCTAT 5' p-value: 0.02

Cs8g17390.1 gene=Cs8g17390 CDS=494-2026

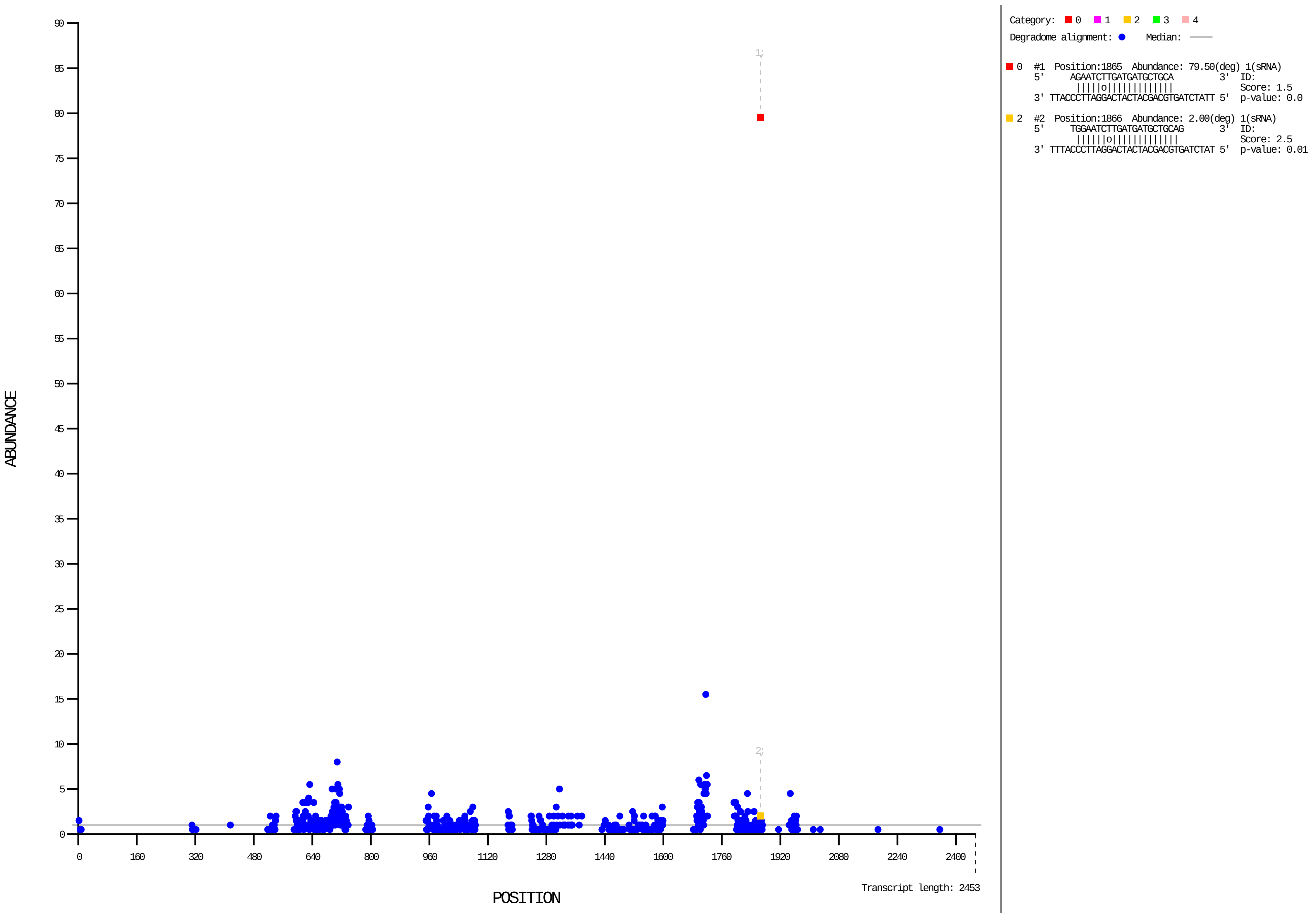

Cs7g15220.1 gene=Cs7g15220 CDS=1-999

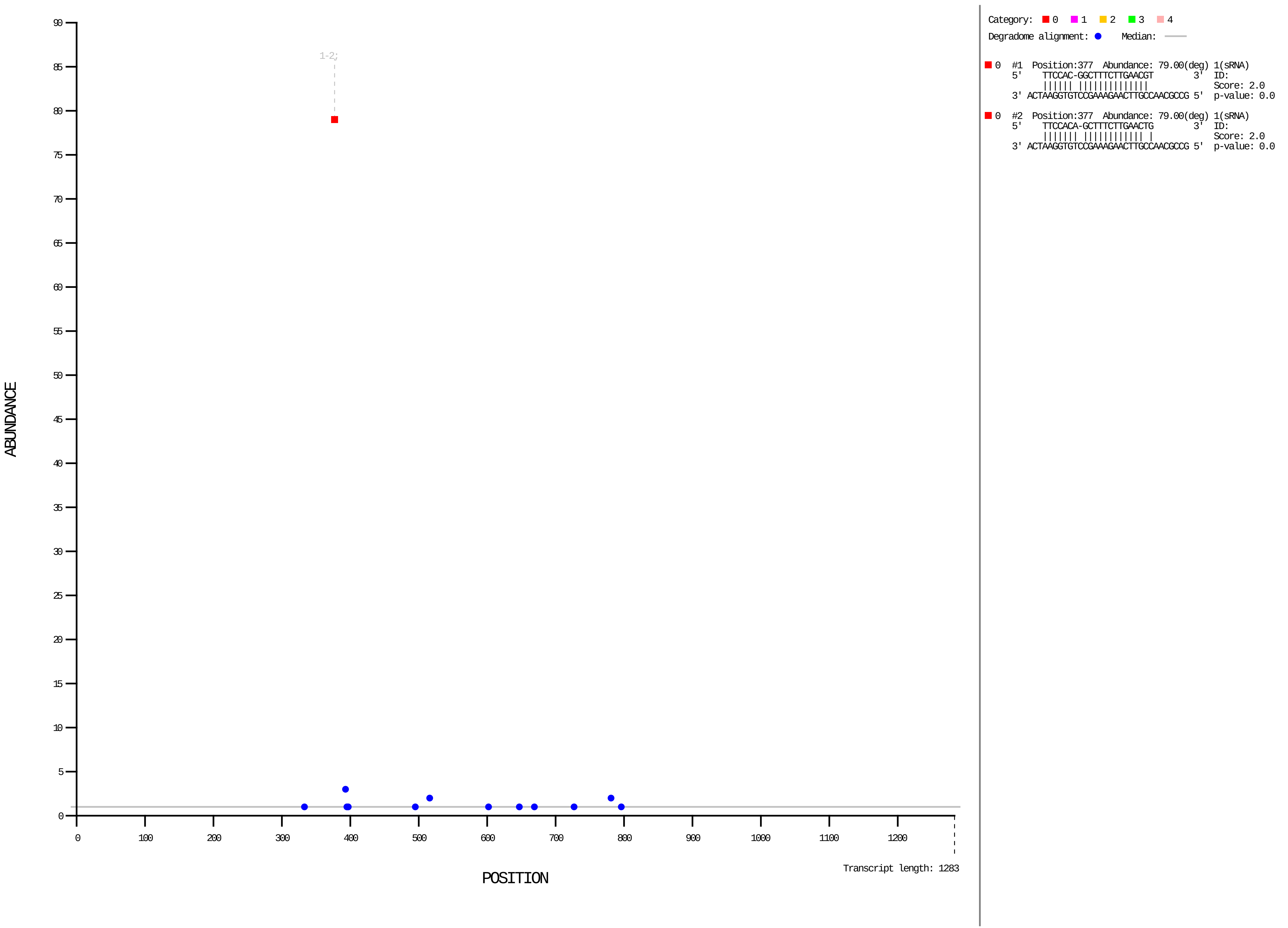

Cs6g06880.1 gene=Cs6g06880 CDS=33-1769

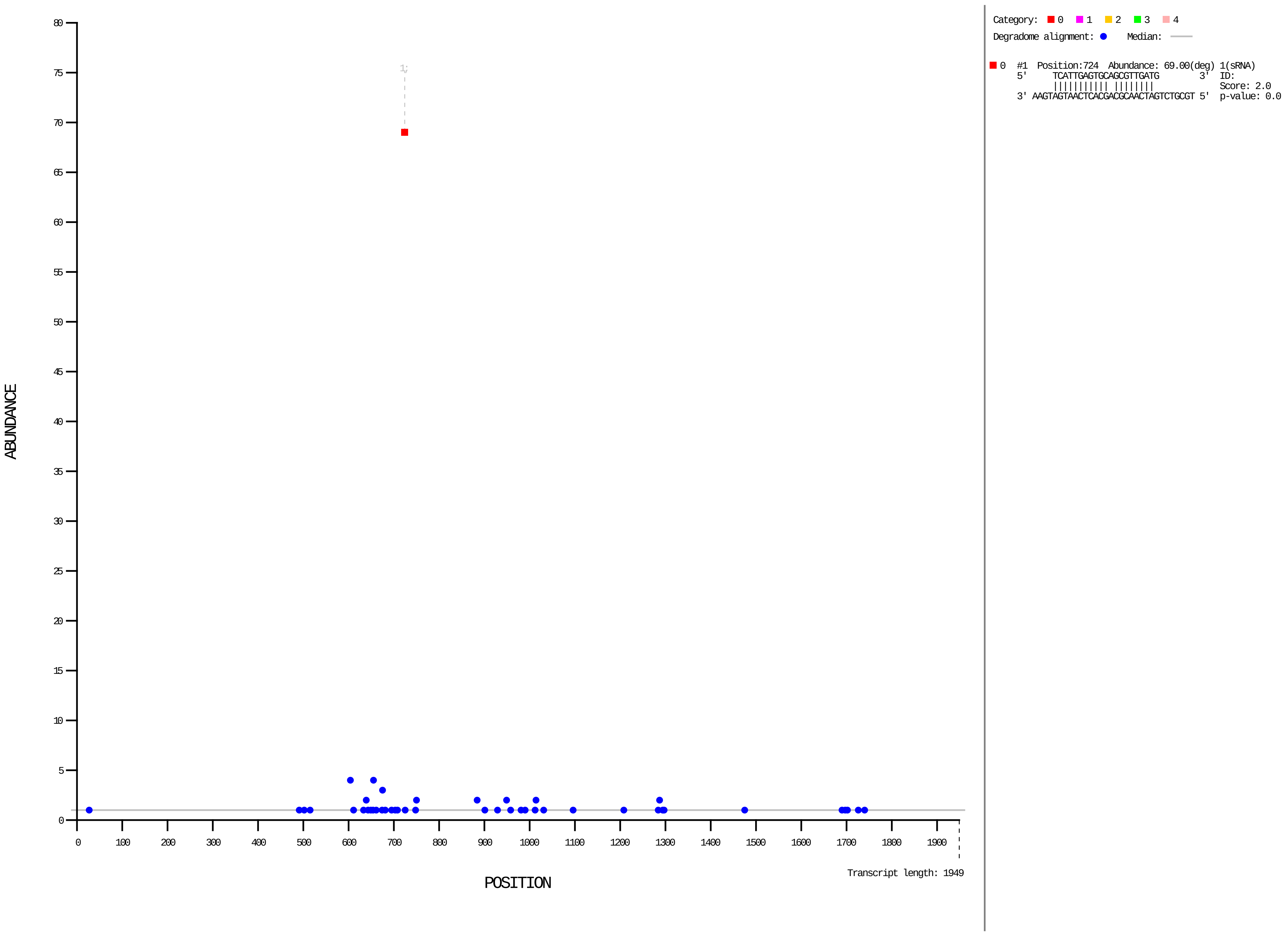

Cs6g07410.1 gene=Cs6g07410 CDS=96-1796

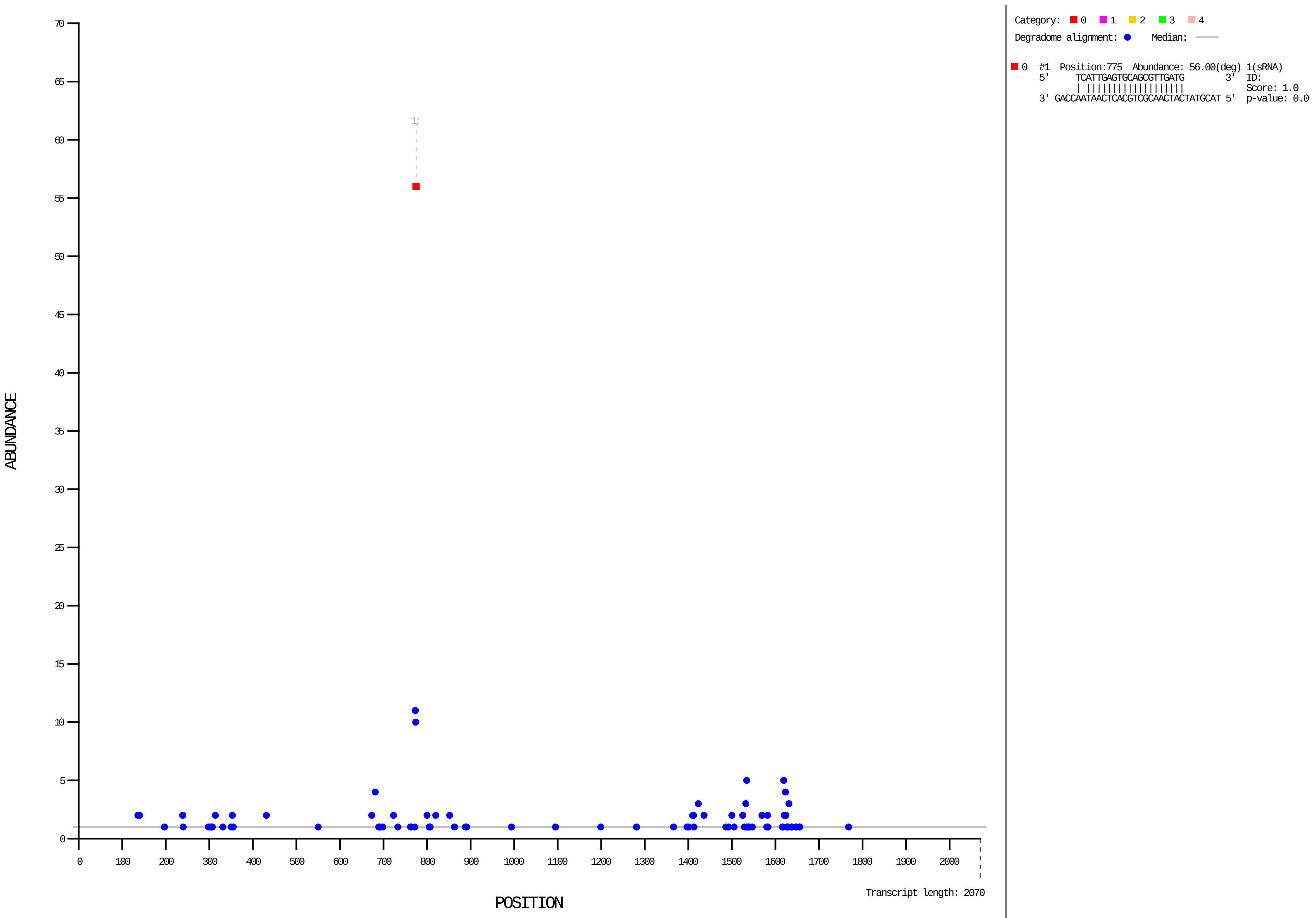

Cs6g11800.1 gene=Cs6g11800 CDS=650-2761

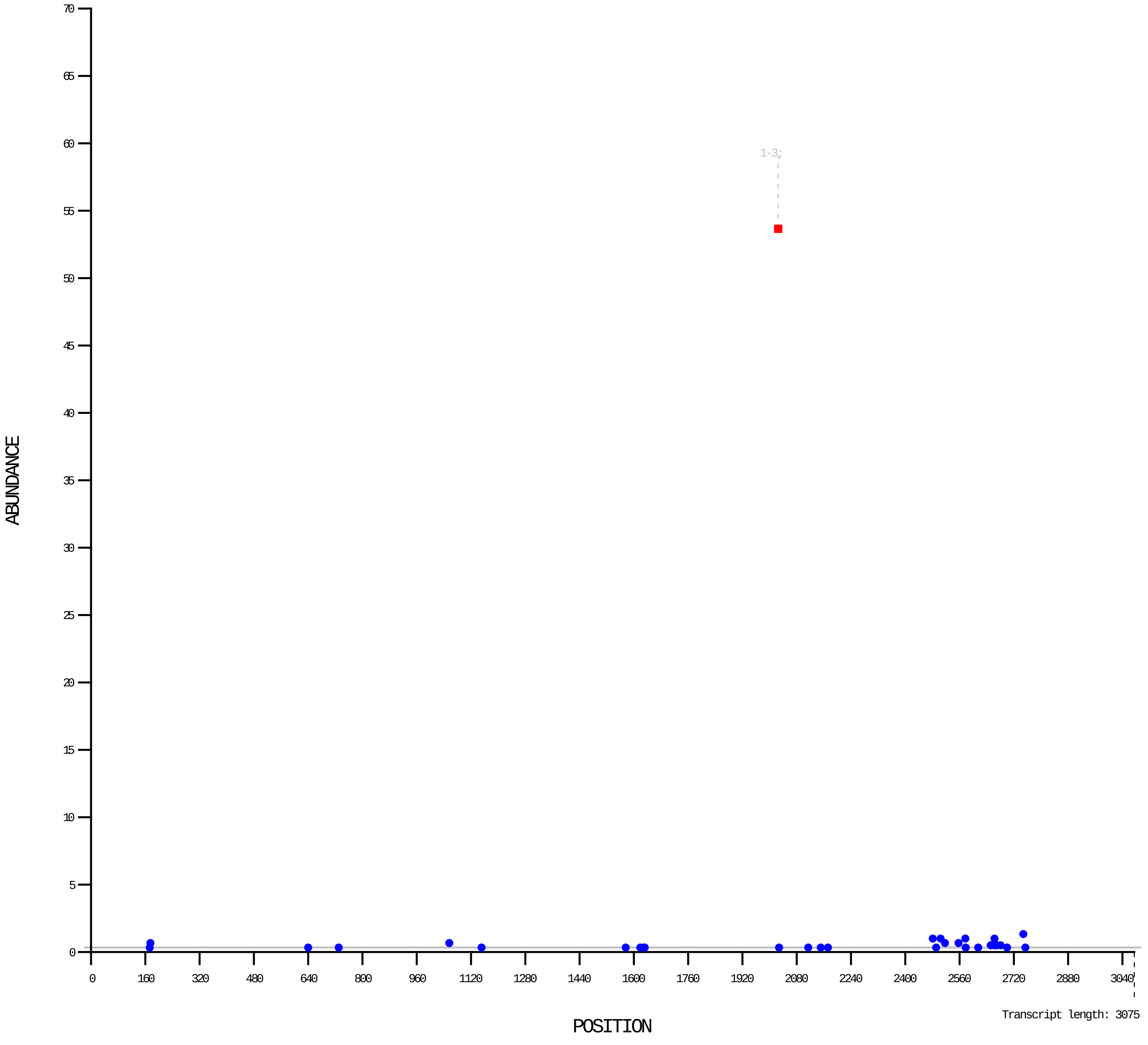


```
Category: 0  1  2  3  4
Degradome alignment:     Median:

0  #1  Position:2025  Abundance: 53.67(deg) 1(sRNA)
   5'     TGCCTGGCTCCCTGTATGCCA        3'  ID:
          ||||||||||||||o||||||            Score: 0.5
   3' TCGTACGGACCGAGGGACGTACGGTCGTCCTC 5'  p-value: 0.0

0  #2  Position:2025  Abundance: 53.67(deg) 1(sRNA)
   5'     TGCCTGGCTCCCTGTATGCCG        3'  ID:
          ||||||||||||||o|||||o            Score: 1.0
   3' TCGTACGGACCGAGGGACGTACGGTCGTCCTC 5'  p-value: 0.0

0  #3  Position:2025  Abundance: 53.67(deg) 1(sRNA)
   5'     TGCCTGGCTCCCTGTATGCTT        3'  ID:
          ||||||||||||||o||||o             Score: 2.0
   3' TCGTACGGACCGAGGGACGTACGGTCGTCCTC 5'  p-value: 0.0
```

Cs6g11800.3 gene=Cs6g11800 CDS=650-2797

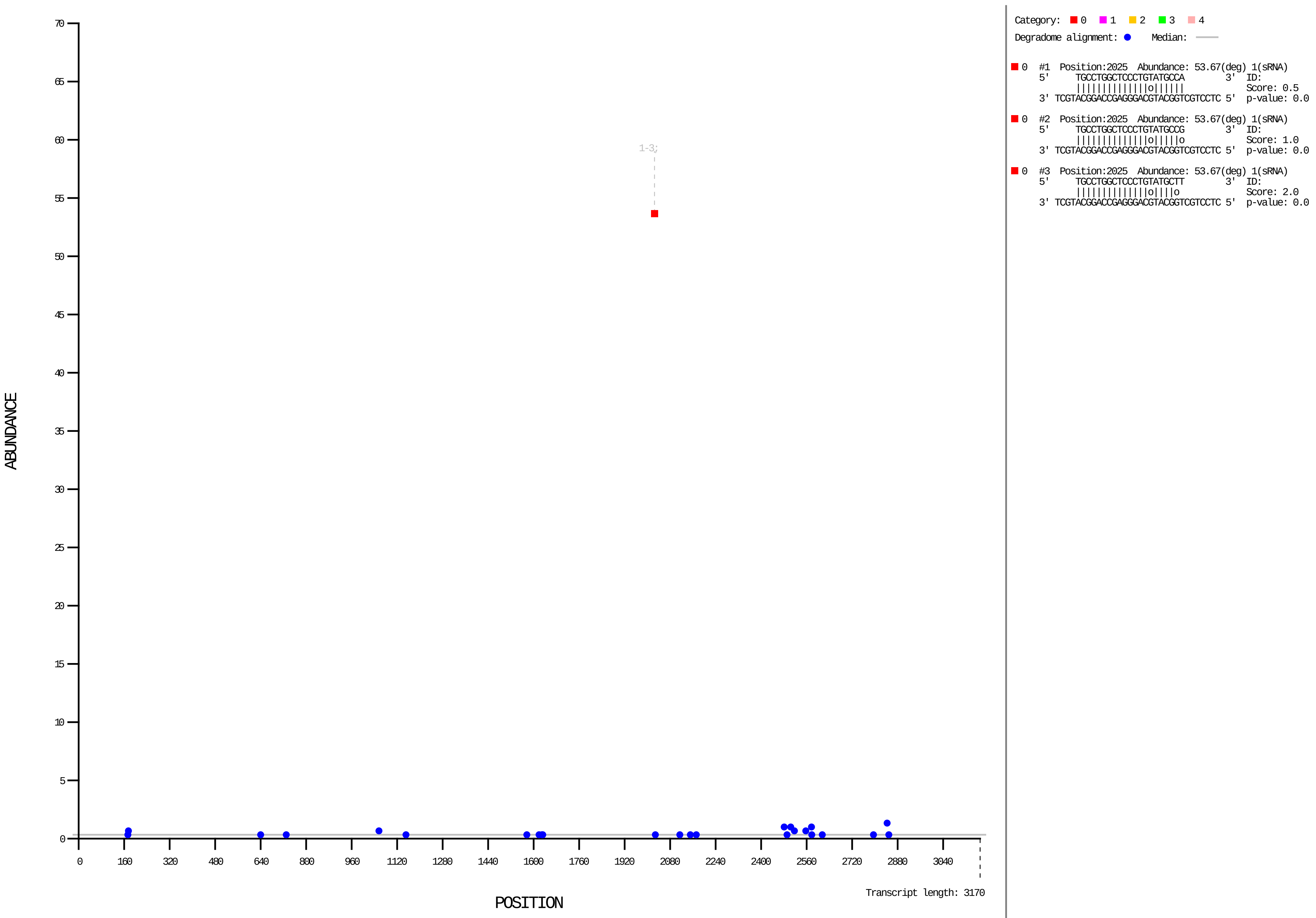

Cs6g11800.2 gene=Cs6g11800 CDS=778-2889

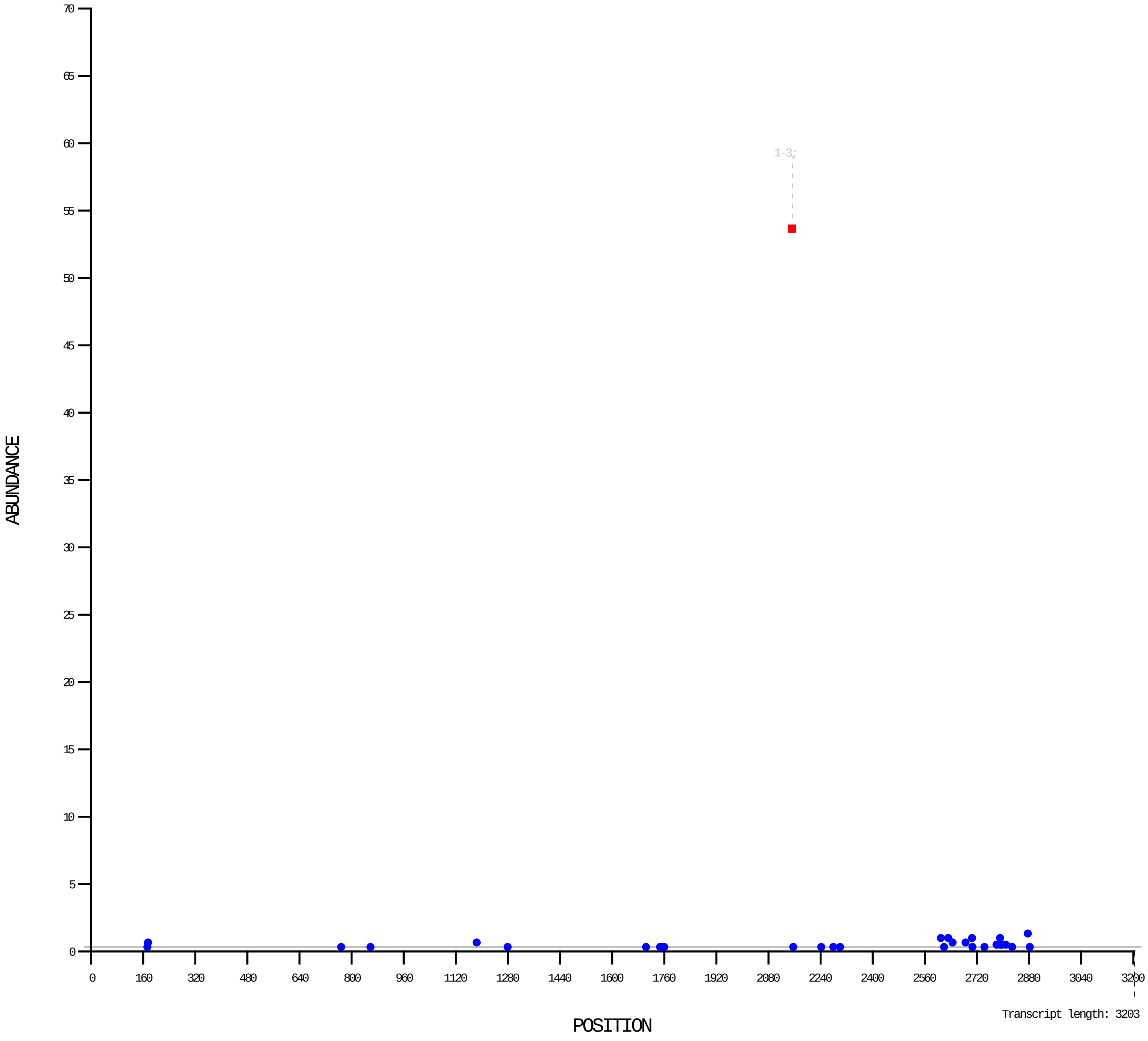


```
Category: ■ 0 ■ 1 ■ 2 ■ 3 ■ 4
Degradome alignment: ●    Median: ——

■ 0  #1  Position:2153  Abundance: 53.67(deg) 1(sRNA)
     5'      TGCCTGGCTCCCTGTATGCCA         3'  ID:
             ||||||||||||||o||||||             Score: 0.5
     3' TCGTACGGACCGAGGGACGTACGGTCGTCCTC 5'  p-value: 0.0

■ 0  #2  Position:2153  Abundance: 53.67(deg) 1(sRNA)
     5'      TGCCTGGCTCCCTGTATGCCG         3'  ID:
             ||||||||||||||o|||||o             Score: 1.0
     3' TCGTACGGACCGAGGGACGTACGGTCGTCCTC 5'  p-value: 0.0

■ 0  #3  Position:2153  Abundance: 53.67(deg) 1(sRNA)
     5'      TGCCTGGCTCCCTGTATGCTT         3'  ID:
             ||||||||||||||o||||o              Score: 2.0
     3' TCGTACGGACCGAGGGACGTACGGTCGTCCTC 5'  p-value: 0.0
```

Cs3g12340.1 gene=Cs3g12340 CDS=1-4731

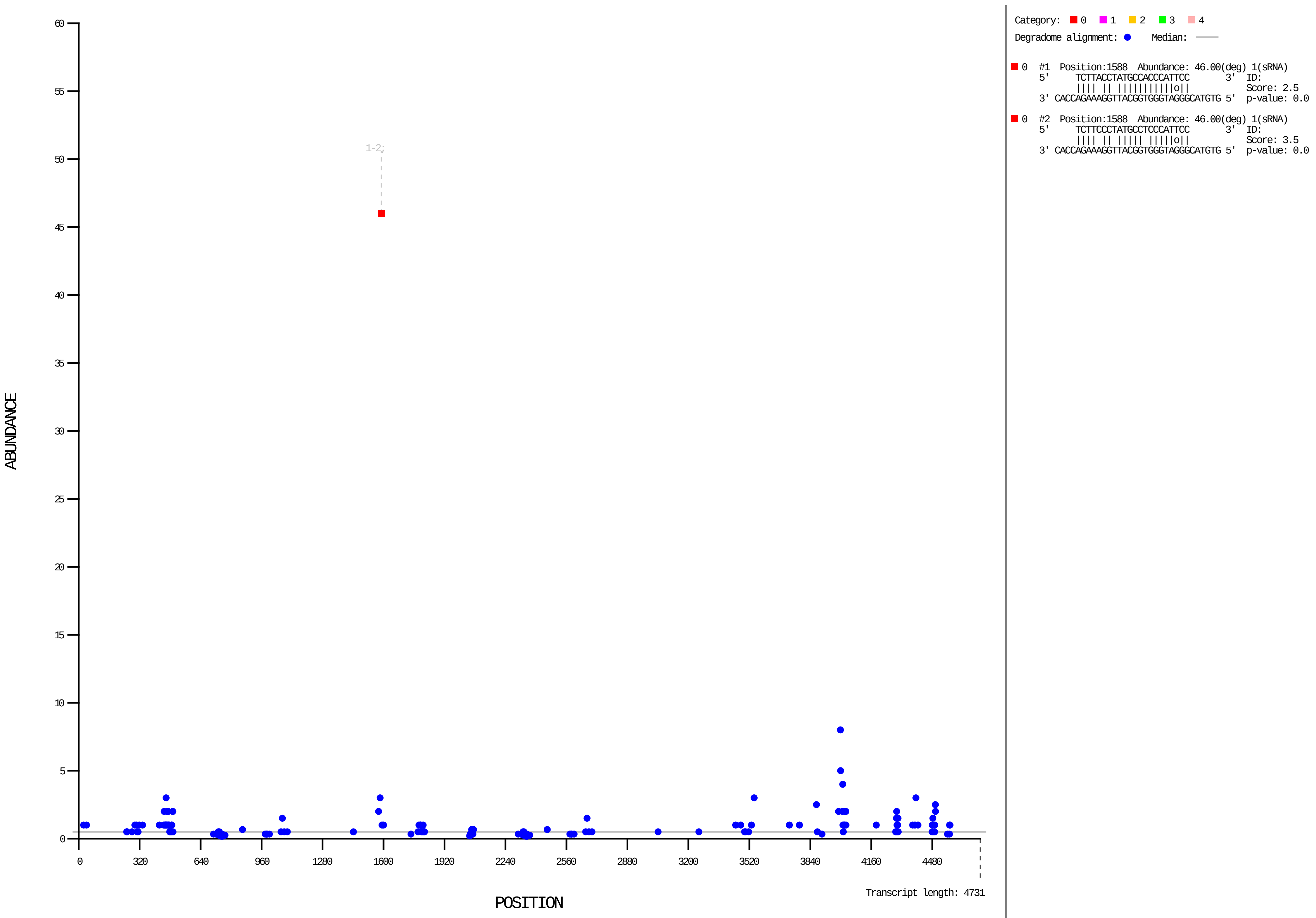

Cs5g21975.1 gene=Cs5g21975 CDS=82-3237

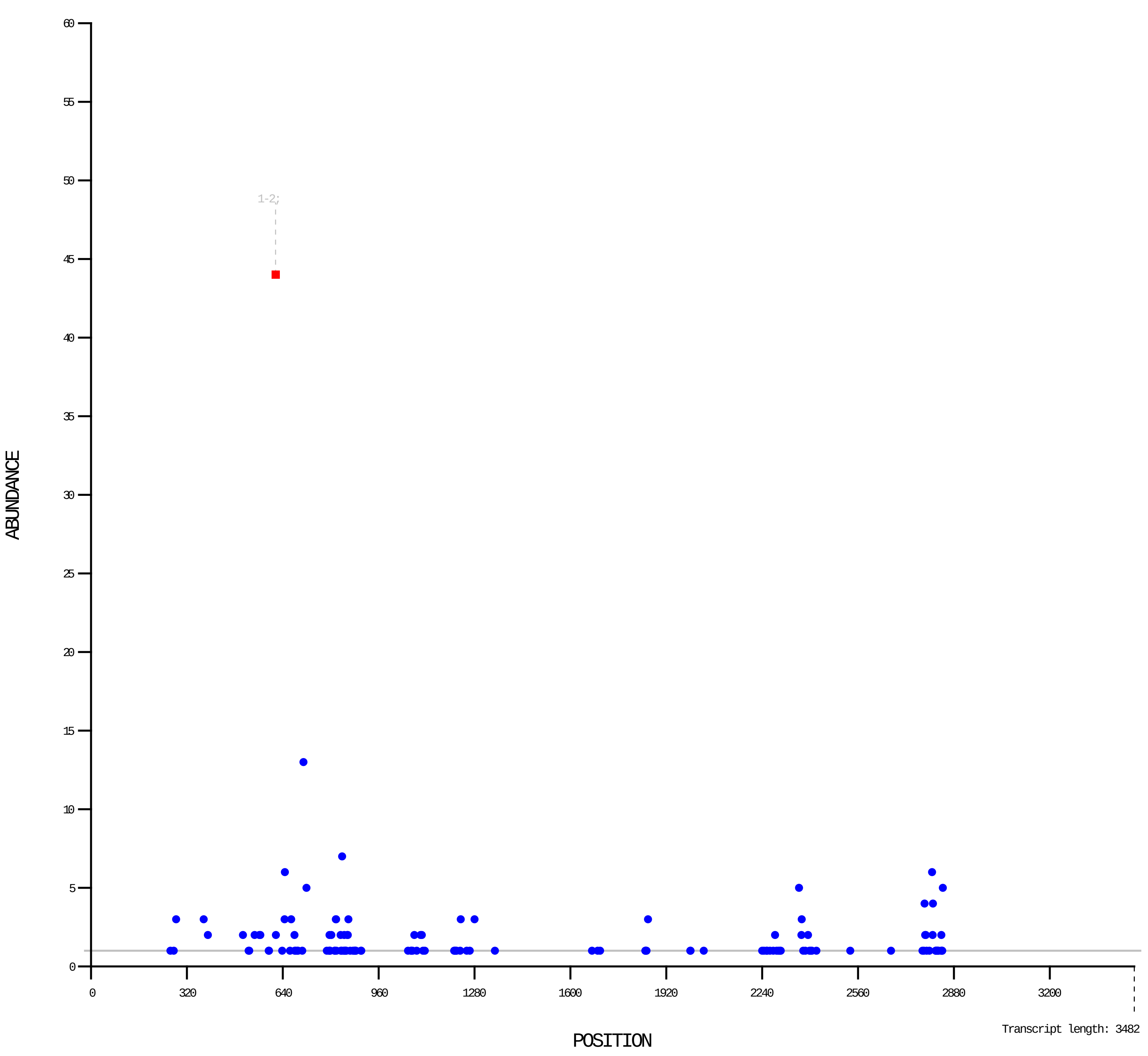


```
0  #1  Position:616  Abundance: 44.00(deg) 1(sRNA)
   5'     TCTTACCTATGCCACCCATTCC       3'  ID:
          |||| ||||| ||||||||o||           Score: 2.5
   3' TATCAGAAAGGATAAGGTGGGTAGGGGTGTTA 5'  p-value: 0.0

0  #2  Position:616  Abundance: 44.00(deg) 1(sRNA)
   5'     TCTTCCCTATGCCTCCCATTCC       3'  ID:
          |||| ||||| || |||||o||           Score: 3.5
   3' TATCAGAAAGGATAAGGTGGGTAGGGGTGTTA 5'  p-value: 0.0
```

orange1.1t03122.1 gene=orange1.1t03122 CDS=243-1316

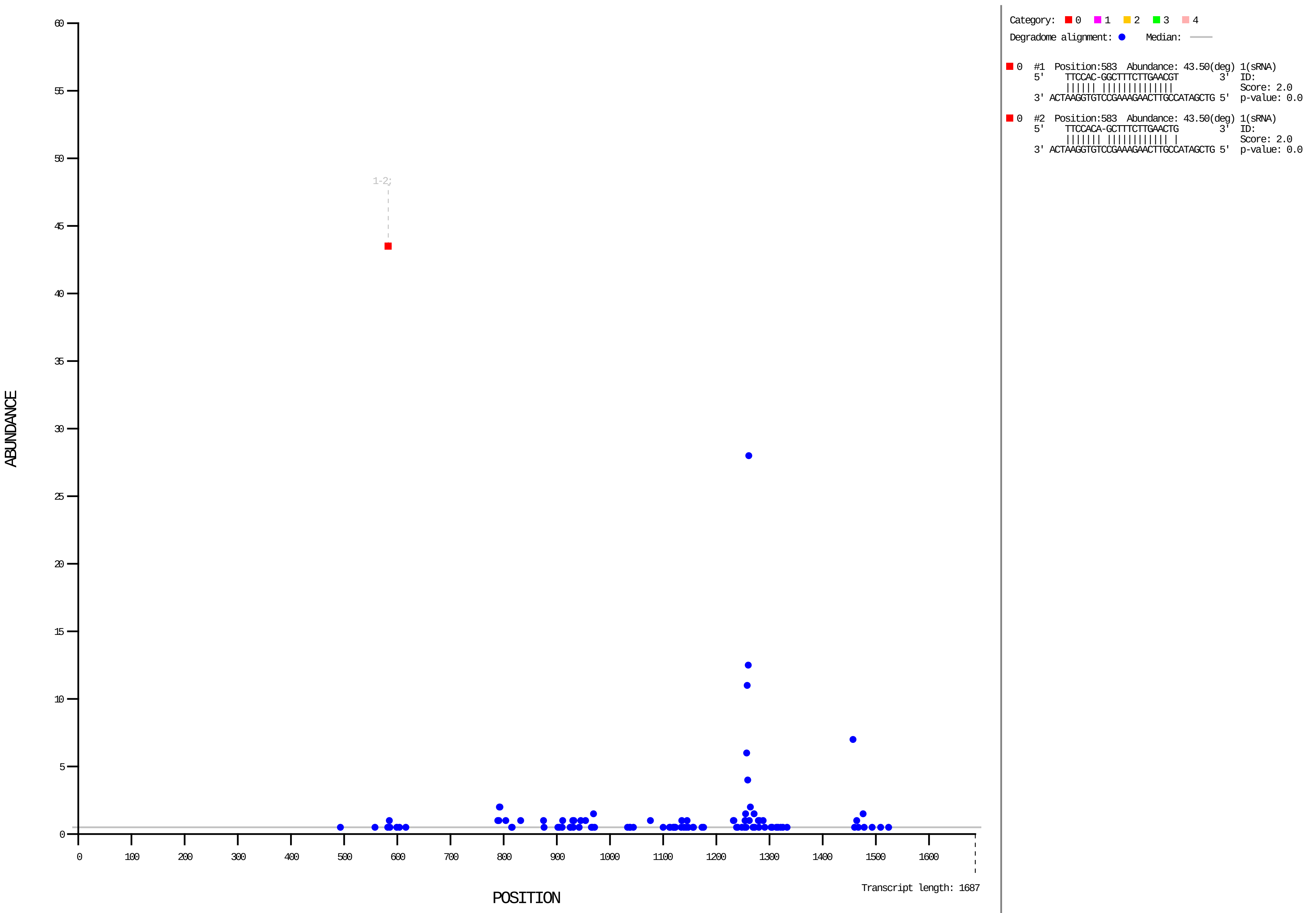

orange1.1t03122.2 gene=orange1.1t03122 CDS=243-1328

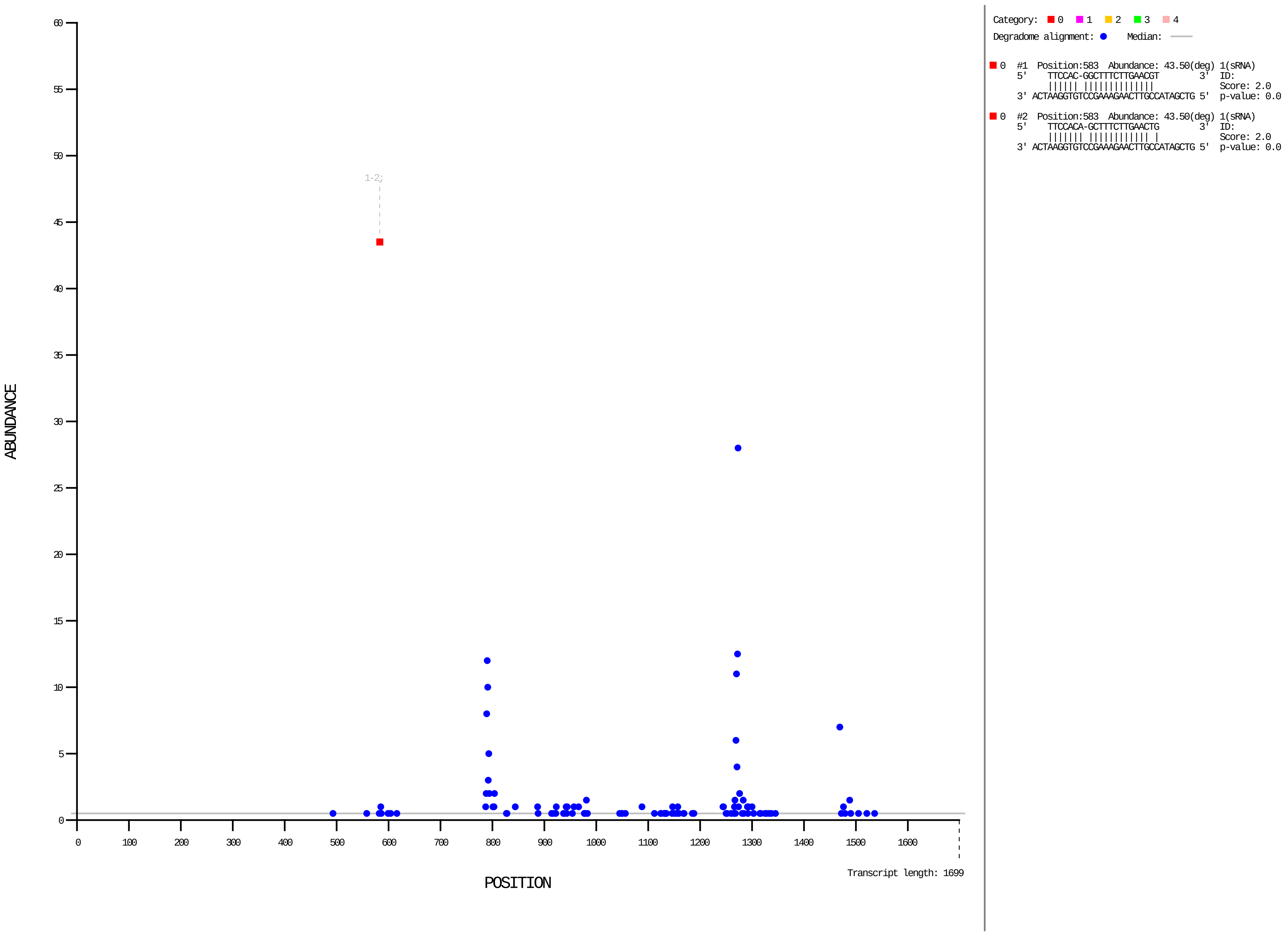

Cs1g24520.1 gene=Cs1g24520 CDS=191-2740

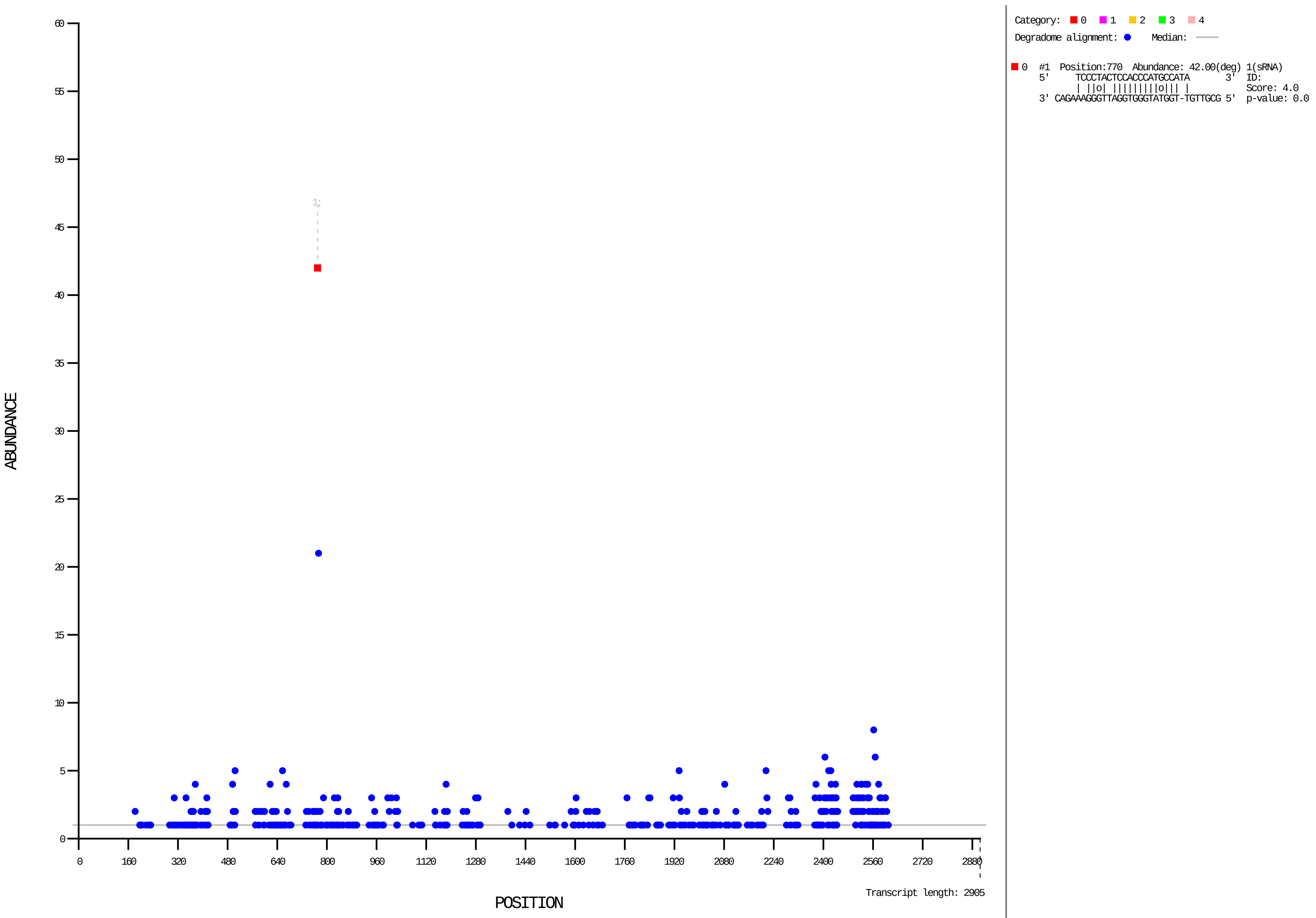

Cs2g06030.2 gene=Cs2g06030 CDS=1325-4183

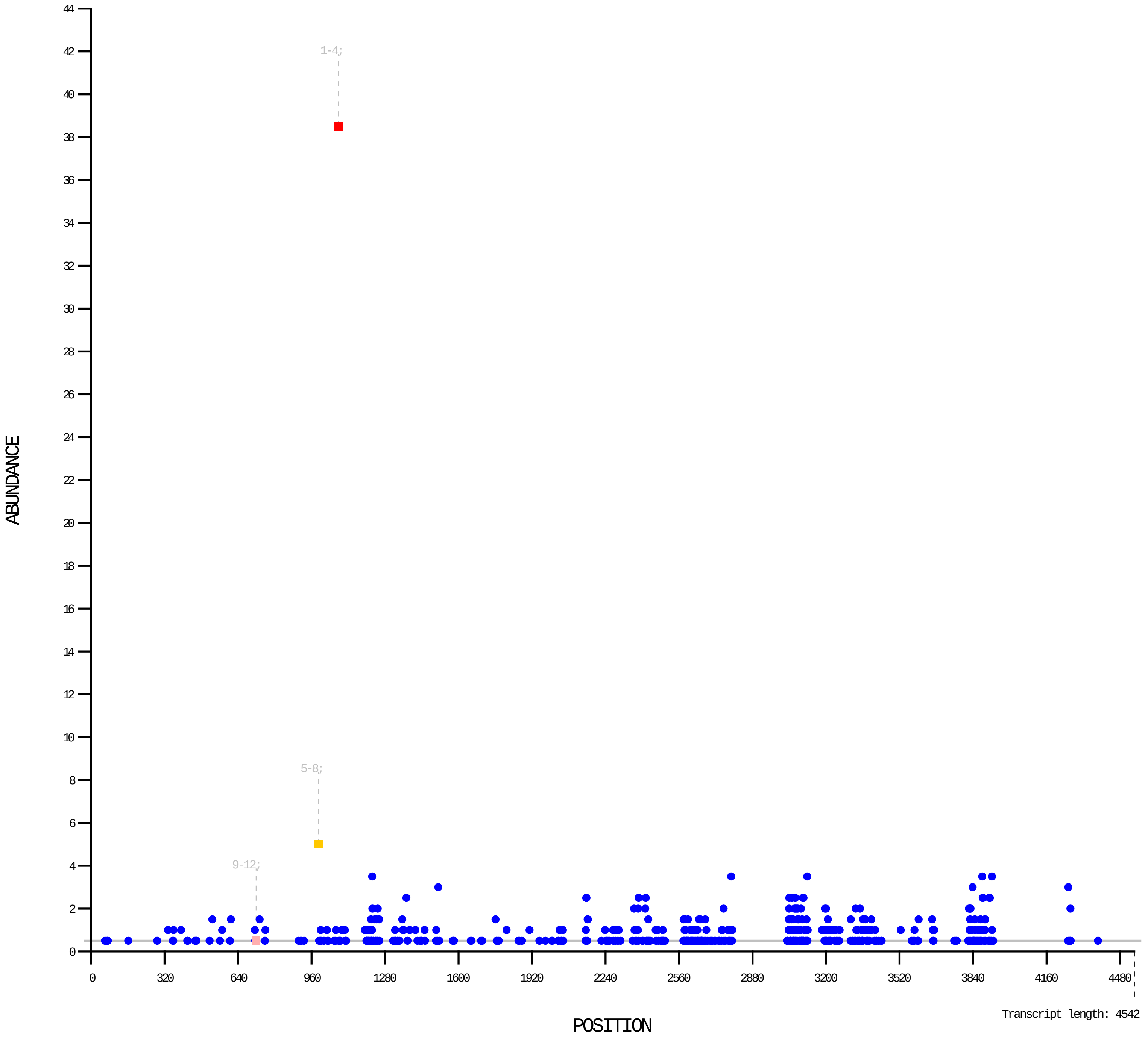


```
Category: 0  1  2  3  4
Degradome alignment:    Median:

0  #1  Position:1077  Abundance: 38.50(deg) 1(sRNA)
   5'     TGCCAAAGGAGATTTGCCCGG        3'  ID:
          ||||||||||||||||||| o            Score: 1.5
   3' GATAACGGTTTCCTCTAAACGGGGTTGTCCCT 5'  p-value: 0.0

0  #2  Position:1077  Abundance: 38.50(deg) 1(sRNA)
   5'     TGCCAAAGGAGAGTTGCCCTA        3'  ID:
          |||||||||||| ||||||o|            Score: 1.5
   3' GATAACGGTTTCCTCTAAACGGGGTTGTCCCT 5'  p-value: 0.0

0  #3  Position:1077  Abundance: 38.50(deg) 1(sRNA)
   5'     TGCCAAAGGAGAGTTGCCCTG        3'  ID:
          |||||||||||| ||||||oo            Score: 2.0
   3' GATAACGGTTTCCTCTAAACGGGGTTGTCCCT 5'  p-value: 0.0

0  #4  Position:1077  Abundance: 38.50(deg) 1(sRNA)
   5'     TGCCAAAGGAGAATTGCCCTG        3'  ID:
          |||||||||||| ||||||oo            Score: 2.0
   3' GATAACGGTTTCCTCTAAACGGGGTTGTCCCT 5'  p-value: 0.0

2  #5  Position:991  Abundance: 5.00(deg) 1(sRNA)
   5'     TGCCAAAGGAGATTTGCCCGG        3'  ID:
          |||||||||||||||||||oo            Score: 1.0
   3' GTTTACGGTTTCCTCTAAACGGGTTACTTGTC 5'  p-value: 0.0

2  #6  Position:991  Abundance: 5.00(deg) 1(sRNA)
   5'     TGCCAAAGGAGAGTTGCCCTA        3'  ID:
          |||||||||||| |||||| |            Score: 2.0
   3' GTTTACGGTTTCCTCTAAACGGGTTACTTGTC 5'  p-value: 0.01

2  #7  Position:991  Abundance: 5.00(deg) 1(sRNA)
   5'     TGCCAAAGGAGAGTTGCCCTG        3'  ID:
          |||||||||||| |||||| o            Score: 2.5
   3' GTTTACGGTTTCCTCTAAACGGGTTACTTGTC 5'  p-value: 0.03

2  #8  Position:991  Abundance: 5.00(deg) 1(sRNA)
   5'     TGCCAAAGGAGAATTGCCCTG        3'  ID:
          |||||||||||| |||||| o            Score: 2.5
   3' GTTTACGGTTTCCTCTAAACGGGTTACTTGTC 5'  p-value: 0.03

4  #9  Position:719  Abundance: 0.50(deg) 1(sRNA)
   5'     TGCCAAAGGAGATTTGCCCGG        3'  ID:
          |||||||||||||||||||oo            Score: 1.0
   3' AAAAACGGTTTCCTCTAAACGGGTTGTCACCT 5'  p-value: 0.0

4  #10  Position:719  Abundance: 0.50(deg) 1(sRNA)
   5'     TGCCAAAGGAGAGTTGCCCTA        3'  ID:
          |||||||||||| |||||| |            Score: 2.0
   3' AAAAACGGTTTCCTCTAAACGGGTTGTCACCT 5'  p-value: 0.0

4  #11  Position:719  Abundance: 0.50(deg) 1(sRNA)
   5'     TGCCAAAGGAGAGTTGCCCTG        3'  ID:
          |||||||||||| |||||| o            Score: 2.5
   3' AAAAACGGTTTCCTCTAAACGGGTTGTCACCT 5'  p-value: 0.0

4  #12  Position:719  Abundance: 0.50(deg) 1(sRNA)
   5'     TGCCAAAGGAGAATTGCCCTG        3'  ID:
          |||||||||||| |||||| o            Score: 2.5
   3' AAAAACGGTTTCCTCTAAACGGGTTGTCACCT 5'  p-value: 0.02
```

Cs2g06030.1 gene=Cs2g06030 CDS=1325-4045

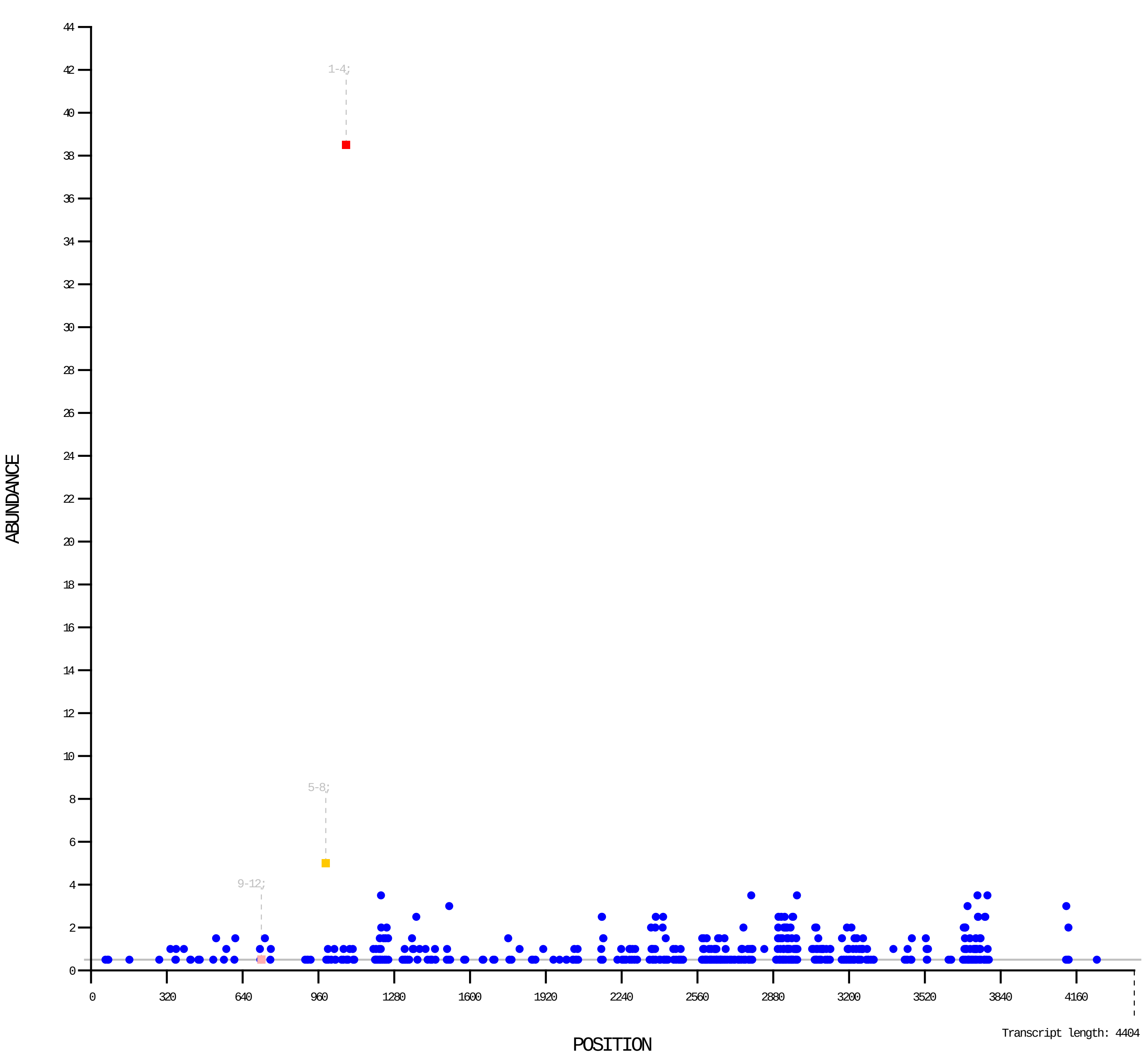


```
Category: 0  1  2  3  4
Degradome alignment:      Median:

0  #1  Position:1077  Abundance: 38.50(deg) 1(sRNA)
   5'     TGCCAAAGGAGATTTGCCCGG        3'  ID:
          |||||||||||||||||| o             Score: 1.5
   3' GATAACGGTTTCCTCTAAACGGGGTTGTCCCT 5'  p-value: 0.0

0  #2  Position:1077  Abundance: 38.50(deg) 1(sRNA)
   5'     TGCCAAAGGAGAGTTGCCCTA        3'  ID:
          |||||||||||| ||||||o|            Score: 1.5
   3' GATAACGGTTTCCTCTAAACGGGGTTGTCCCT 5'  p-value: 0.0

0  #3  Position:1077  Abundance: 38.50(deg) 1(sRNA)
   5'     TGCCAAAGGAGAGTTGCCCTG        3'  ID:
          |||||||||||| ||||||oo            Score: 2.0
   3' GATAACGGTTTCCTCTAAACGGGGTTGTCCCT 5'  p-value: 0.0

0  #4  Position:1077  Abundance: 38.50(deg) 1(sRNA)
   5'     TGCCAAAGGAGAATTGCCCTG        3'  ID:
          |||||||||||| ||||||oo            Score: 2.0
   3' GATAACGGTTTCCTCTAAACGGGGTTGTCCCT 5'  p-value: 0.0

2  #5  Position:991  Abundance: 5.00(deg) 1(sRNA)
   5'     TGCCAAAGGAGATTTGCCCGG        3'  ID:
          |||||||||||||||||||oo            Score: 1.0
   3' GTTTACGGTTTCCTCTAAACGGGTTACTTGTC 5'  p-value: 0.0

2  #6  Position:991  Abundance: 5.00(deg) 1(sRNA)
   5'     TGCCAAAGGAGAGTTGCCCTA        3'  ID:
          |||||||||||| |||||| |            Score: 2.0
   3' GTTTACGGTTTCCTCTAAACGGGTTACTTGTC 5'  p-value: 0.0

2  #7  Position:991  Abundance: 5.00(deg) 1(sRNA)
   5'     TGCCAAAGGAGAGTTGCCCTG        3'  ID:
          |||||||||||| |||||| o            Score: 2.5
   3' GTTTACGGTTTCCTCTAAACGGGTTACTTGTC 5'  p-value: 0.05

2  #8  Position:991  Abundance: 5.00(deg) 1(sRNA)
   5'     TGCCAAAGGAGAATTGCCCTG        3'  ID:
          |||||||||||| |||||| o            Score: 2.5
   3' GTTTACGGTTTCCTCTAAACGGGTTACTTGTC 5'  p-value: 0.01

4  #9  Position:719  Abundance: 0.50(deg) 1(sRNA)
   5'     TGCCAAAGGAGATTTGCCCGG        3'  ID:
          |||||||||||||||||||oo            Score: 1.0
   3' AAAAACGGTTTCCTCTAAACGGGTTGTCACCT 5'  p-value: 0.0

4  #10  Position:719  Abundance: 0.50(deg) 1(sRNA)
   5'     TGCCAAAGGAGAGTTGCCCTA        3'  ID:
          |||||||||||| |||||| |            Score: 2.0
   3' AAAAACGGTTTCCTCTAAACGGGTTGTCACCT 5'  p-value: 0.01

4  #11  Position:719  Abundance: 0.50(deg) 1(sRNA)
   5'     TGCCAAAGGAGAGTTGCCCTG        3'  ID:
          |||||||||||| |||||| o            Score: 2.5
   3' AAAAACGGTTTCCTCTAAACGGGTTGTCACCT 5'  p-value: 0.03

4  #12  Position:719  Abundance: 0.50(deg) 1(sRNA)
   5'     TGCCAAAGGAGAATTGCCCTG        3'  ID:
          |||||||||||| |||||| o            Score: 2.5
   3' AAAAACGGTTTCCTCTAAACGGGTTGTCACCT 5'  p-value: 0.03
```

Cs1g13430.1 gene=Cs1g13430 CDS=175-2796

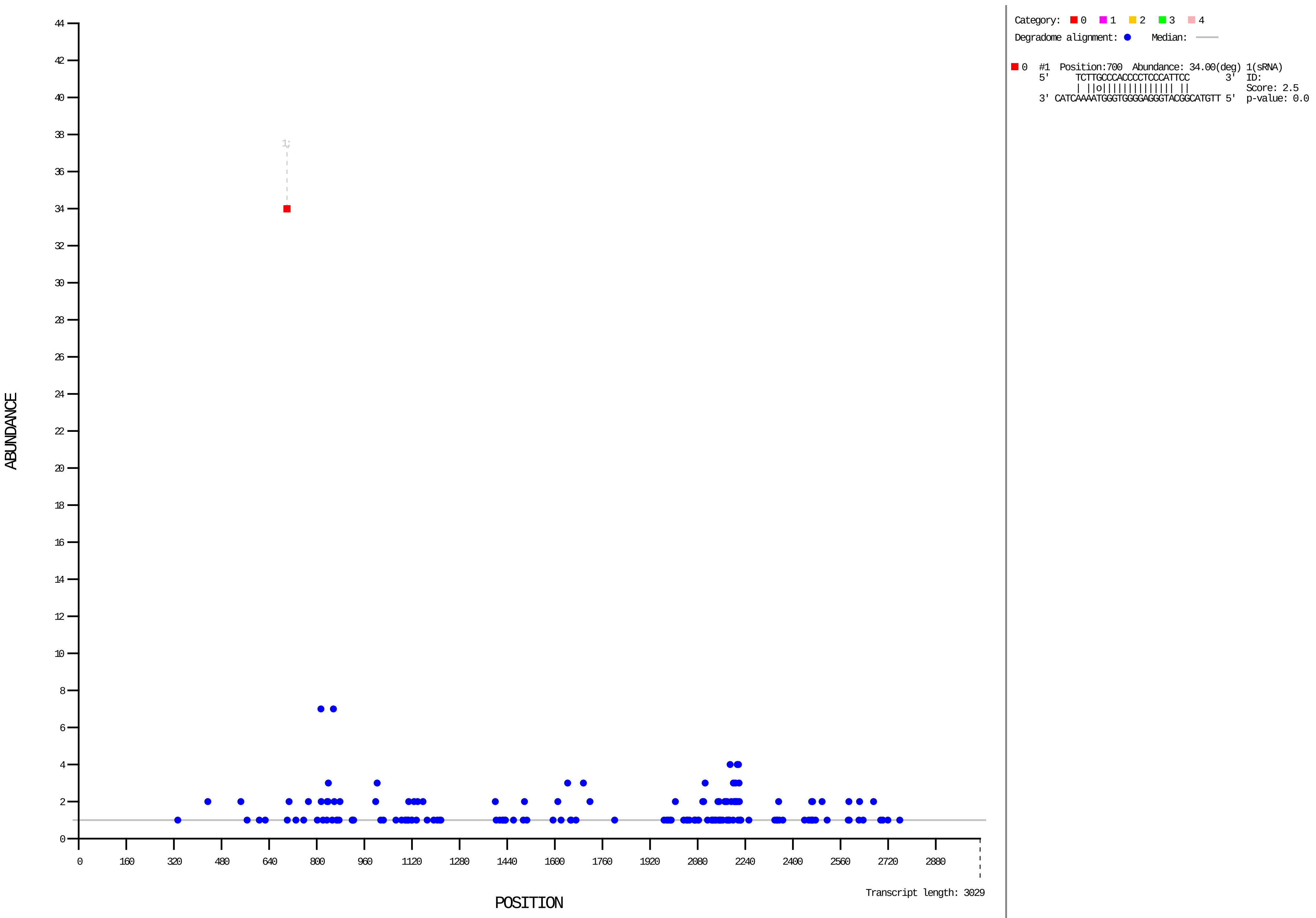

Cs1g21350.1 gene=Cs1g21350 CDS=350-2152

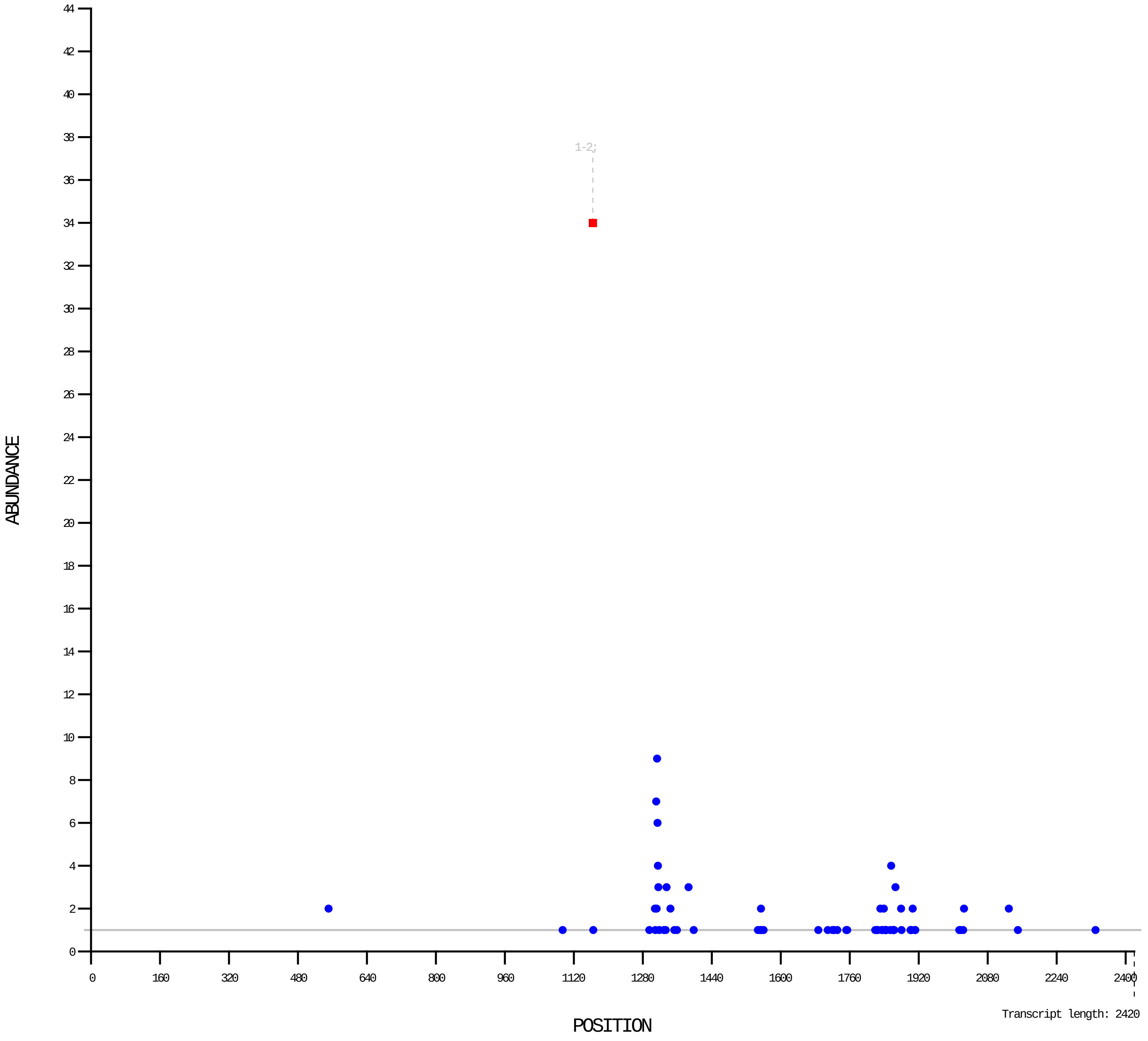

0 #1 Position:1164 Abundance: 34.00(deg) 1(sRNA)

```
5'    TTCCAC-GGCTTTCTTGAACGT        3'  ID:
      |||||| ||||||||||||||             Score: 2.0
3' CGGAAGGTGTCCGAAAGAACTTGCCACCGCCG 5'  p-value: 0.0
```

0 #2 Position:1164 Abundance: 34.00(deg) 1(sRNA)

```
5'    TTCCACA-GCTTTCTTGAACTG        3'  ID:
      ||||||| |||||||||||| |            Score: 2.0
3' CGGAAGGTGTCCGAAAGAACTTGCCACCGCCG 5'  p-value: 0.0
```

orange1.1t04537.1 gene=orange1.1t04537 CDS=344-3817

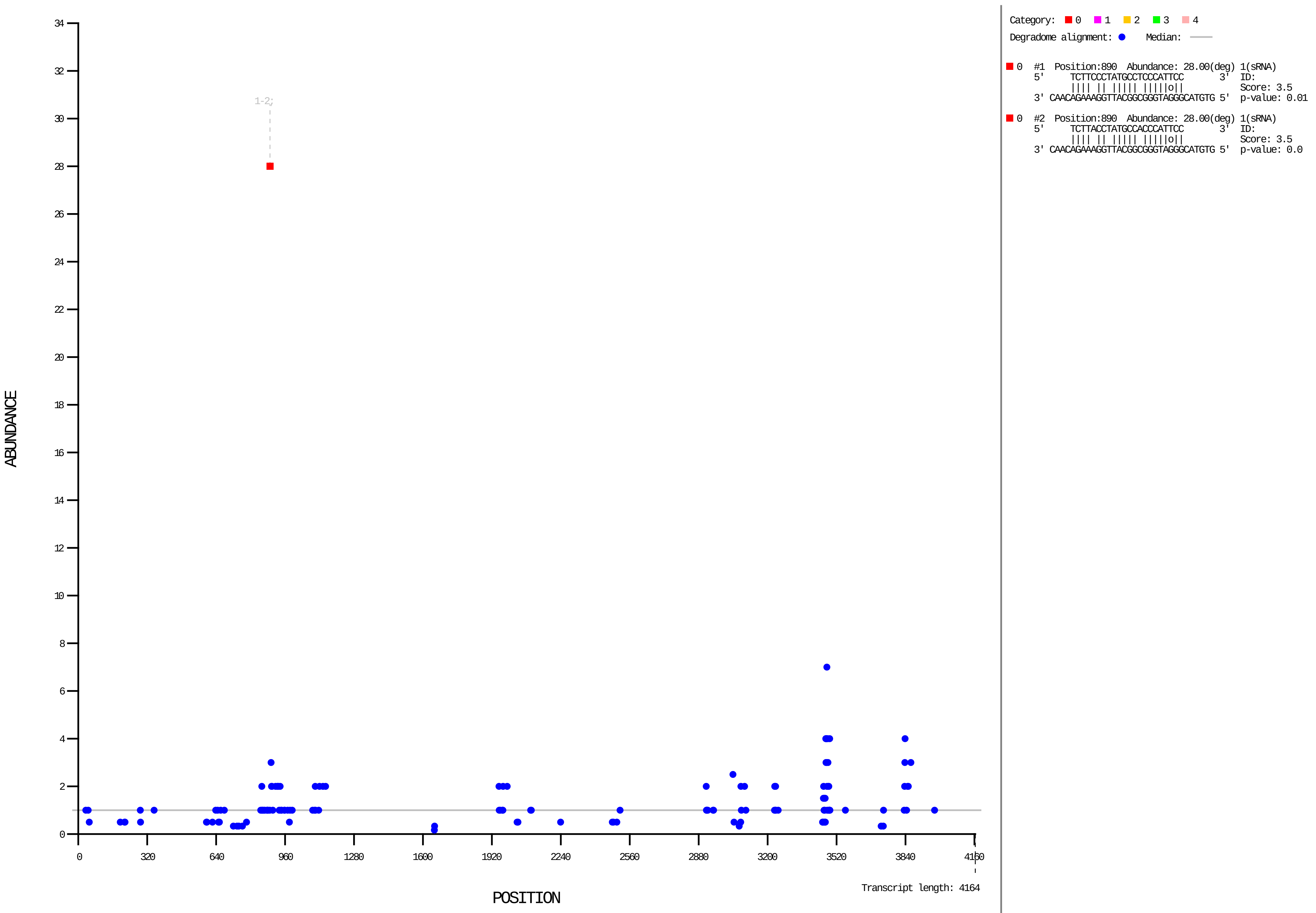

Cs3g24010.3 gene=Cs3g24010 CDS=152-964

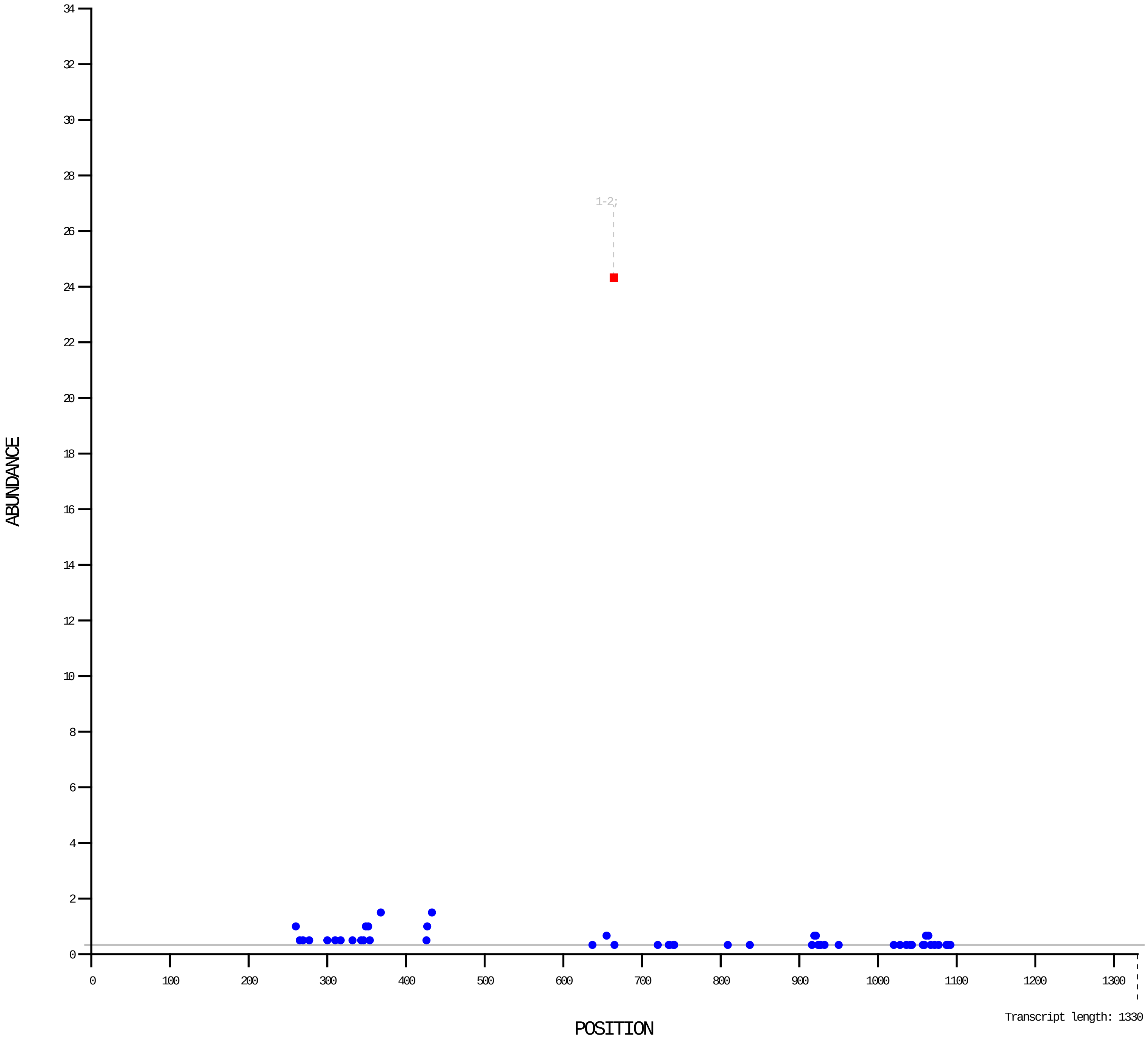


```
Category:  0  1  2  3  4
Degradome alignment:     Median:

0  #1  Position:664  Abundance: 24.33(deg) 1(sRNA)
   5'     TTCCACGGCTTTCTTGAACGT        3'  ID:
          ||||||o| ||||||||| |o            Score: 3.0
   3' AACTAAGGTGTCTAAAGAACTTCCGCTCAGGA 5'  p-value: 0.0

0  #2  Position:664  Abundance: 24.33(deg) 1(sRNA)
   5'     TTCCACAGCTTTCTTGAACTG        3'  ID:
          |||||||| |||||||||               Score: 4.0
   3' AACTAAGGTGTCTAAAGAACTTCCGCTCAGGA 5'  p-value: 0.02
```

Cs3g24010.1 gene=Cs3g24010 CDS=152-964

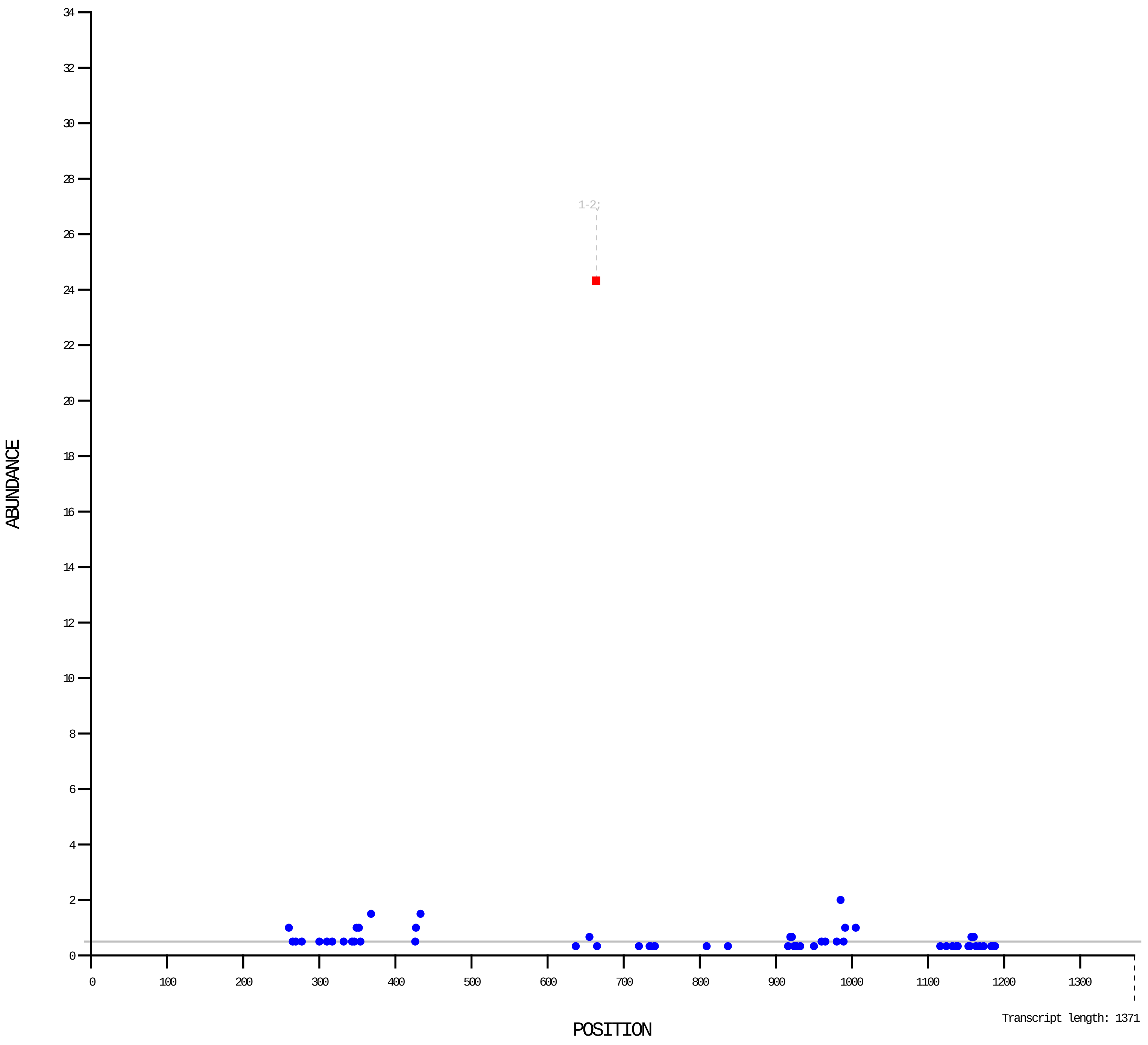

Category: 0 1 2 3 4
Degradome alignment: Median:

0 #1 Position:664 Abundance: 24.33(deg) 1(sRNA)
```
5'     TTCCACGGCTTTCTTGAACGT        3'  ID:
       ||||||o| ||||||||| |o            Score: 3.0
3' AACTAAGGTGTCTAAAGAACTTCCGCTCAGGA 5'  p-value: 0.0
```

0 #2 Position:664 Abundance: 24.33(deg) 1(sRNA)
```
5'     TTCCACAGCTTTCTTGAACTG        3'  ID:
       |||||||| |||||||||               Score: 4.0
3' AACTAAGGTGTCTAAAGAACTTCCGCTCAGGA 5'  p-value: 0.01
```

Cs3g24010.2 gene=Cs3g24010 CDS=43-678

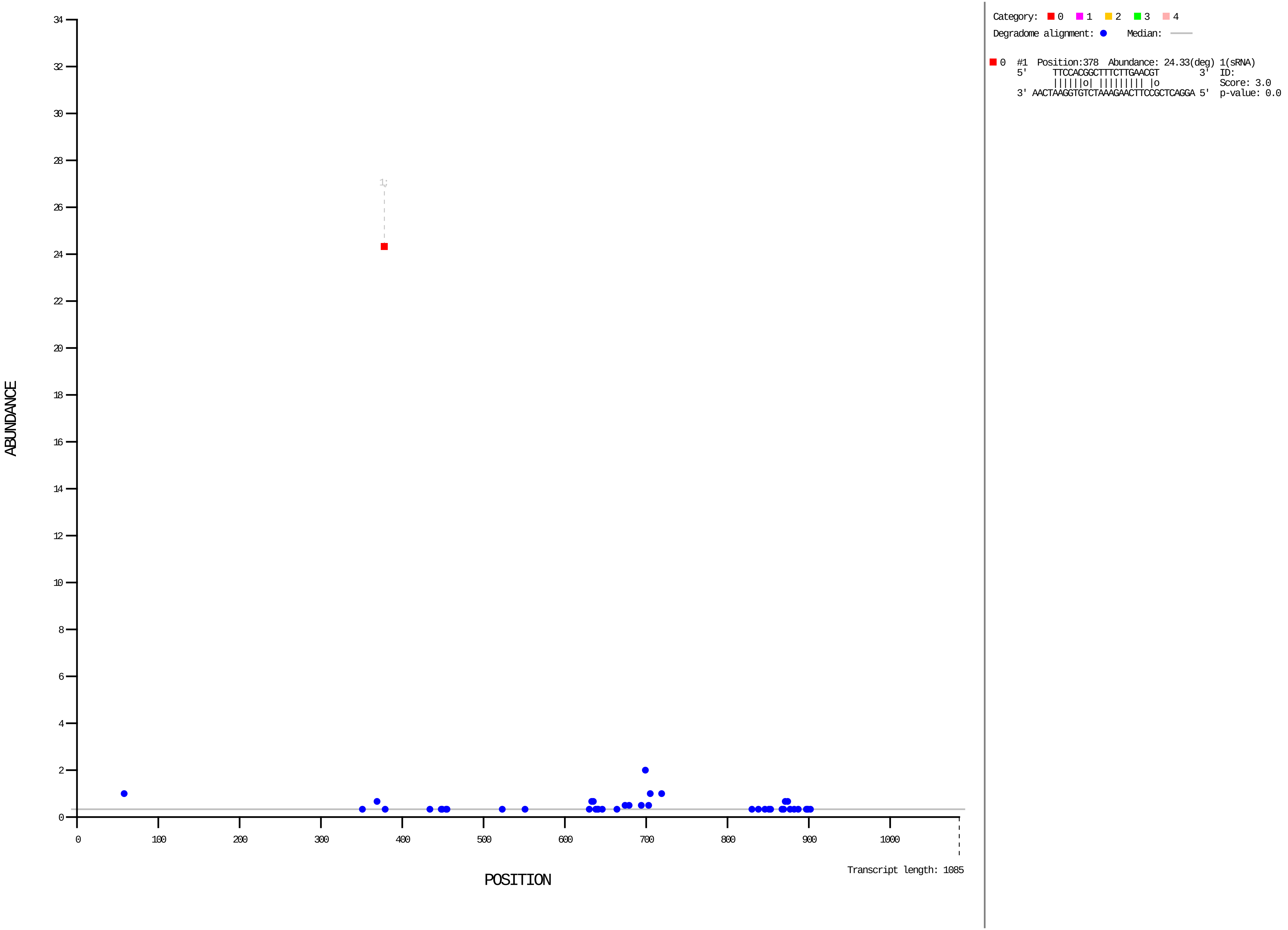

Cs2g09440.2 gene=Cs2g09440 CDS=826-3525

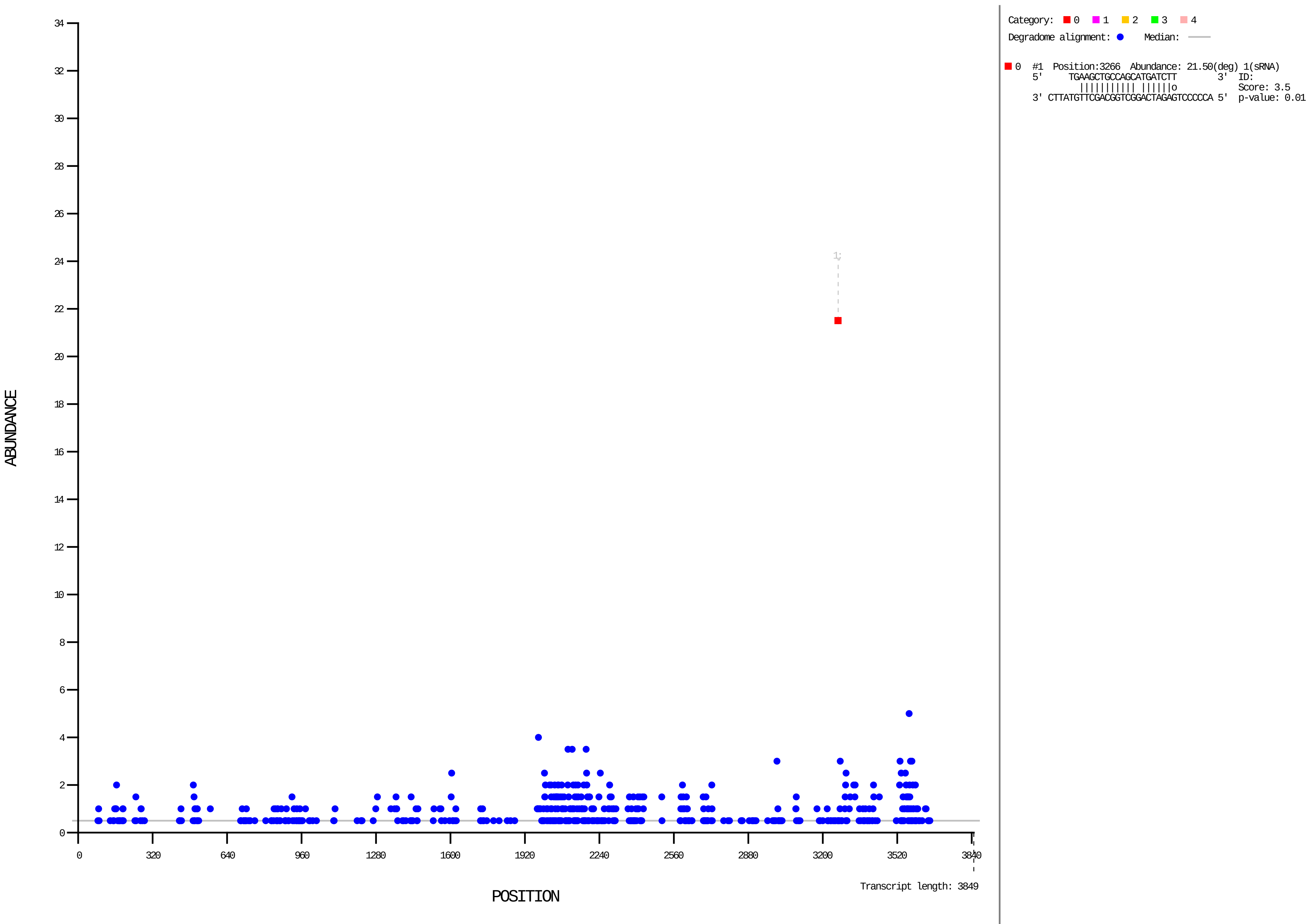

Cs2g09440.1 gene=Cs2g09440 CDS=826-3516

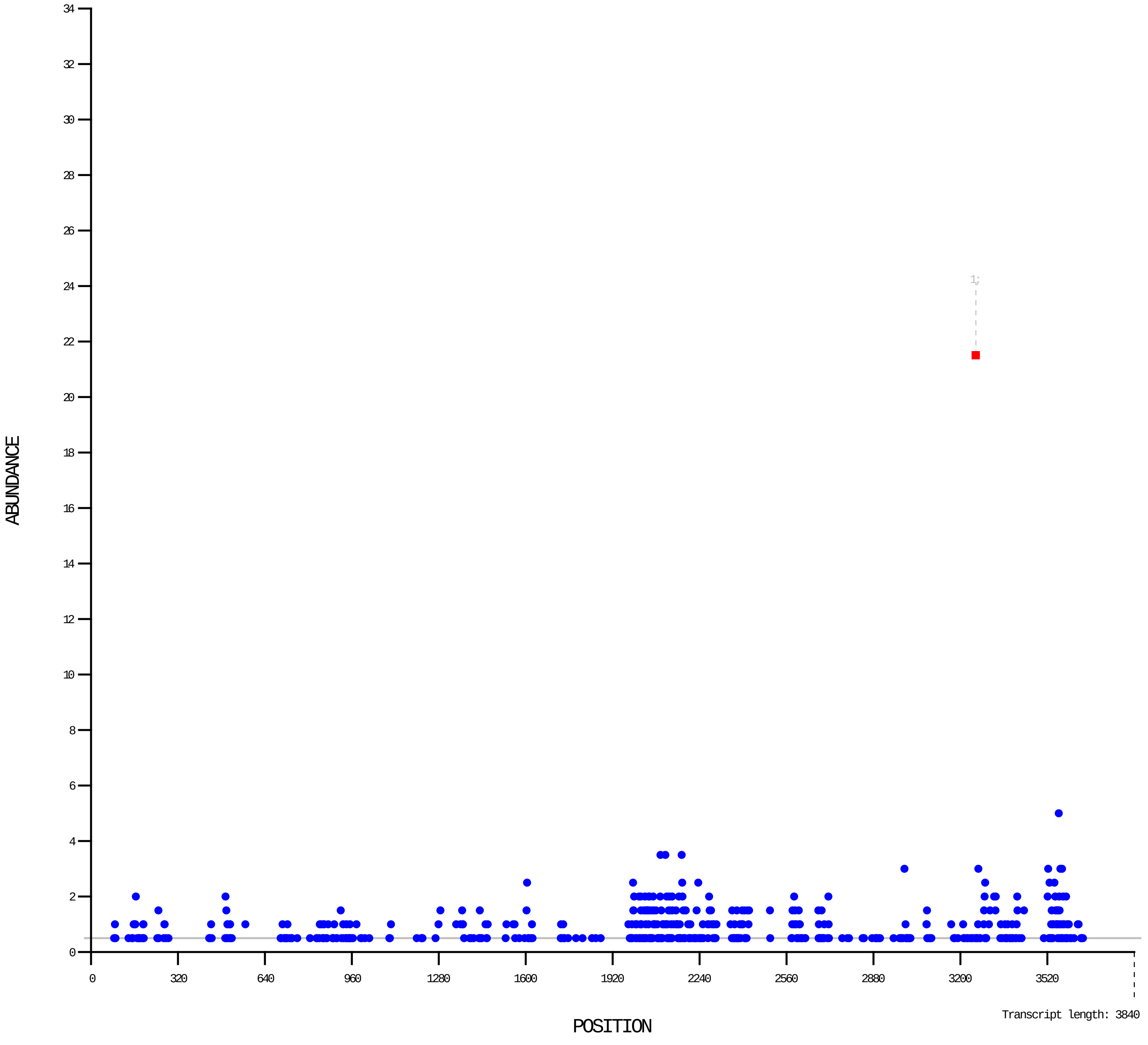

Category: 0 1 2 3 4
Degradome alignment: Median:

0 #1 Position:3257 Abundance: 21.50(deg) 1(sRNA)

```
5'     TGAAGCTGCCAGCATGATCTT        3'  ID:
          |||||||||||| ||||||o          Score: 3.5
3' CTTATGTTCGACGGTCGGACTAGAGTCCCCCA 5'  p-value: 0.01
```

Cs3g12850.1 gene=Cs3g12850 CDS=1-3774

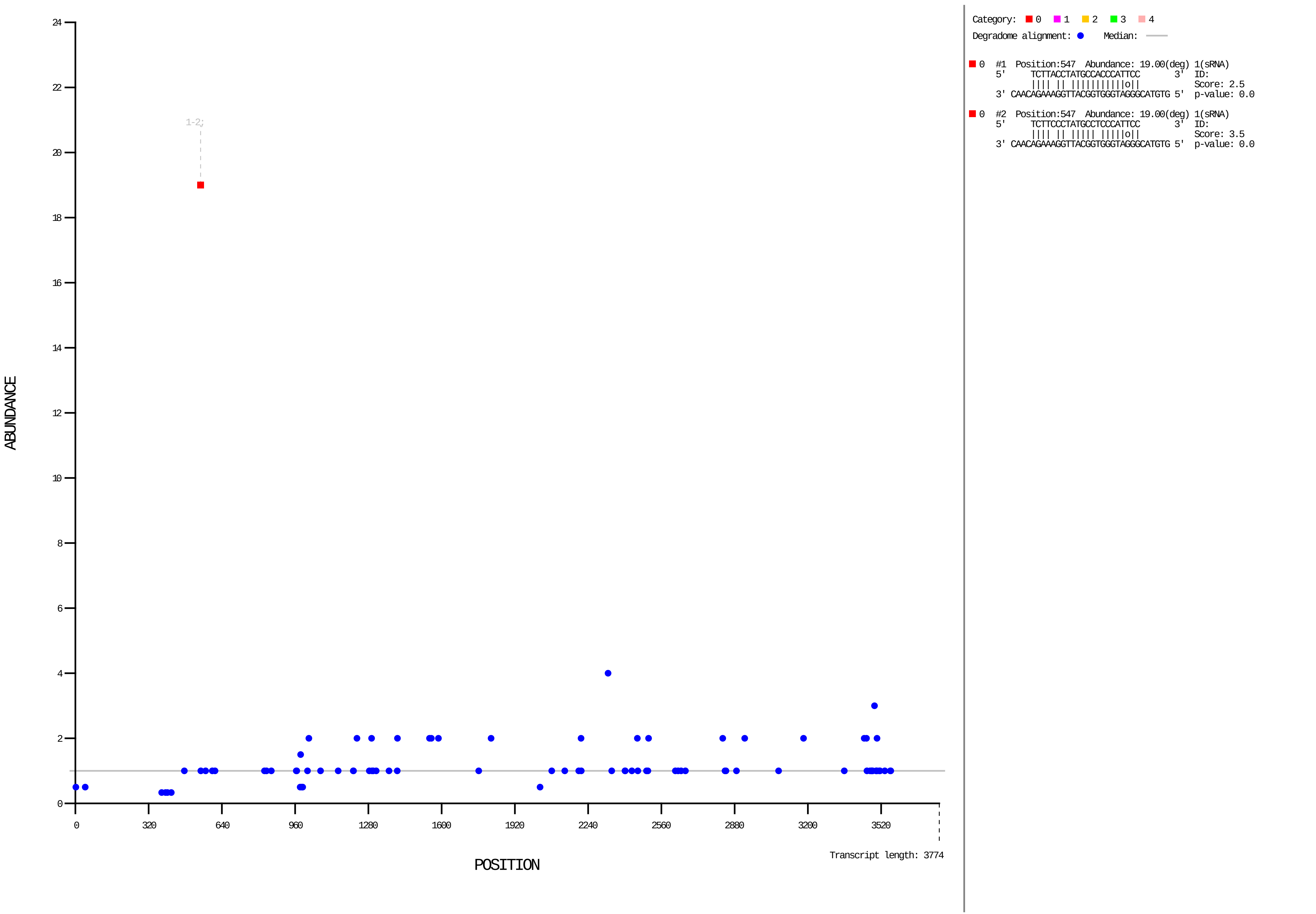

Cs3g16860.1 gene=Cs3g16860 CDS=44-1603

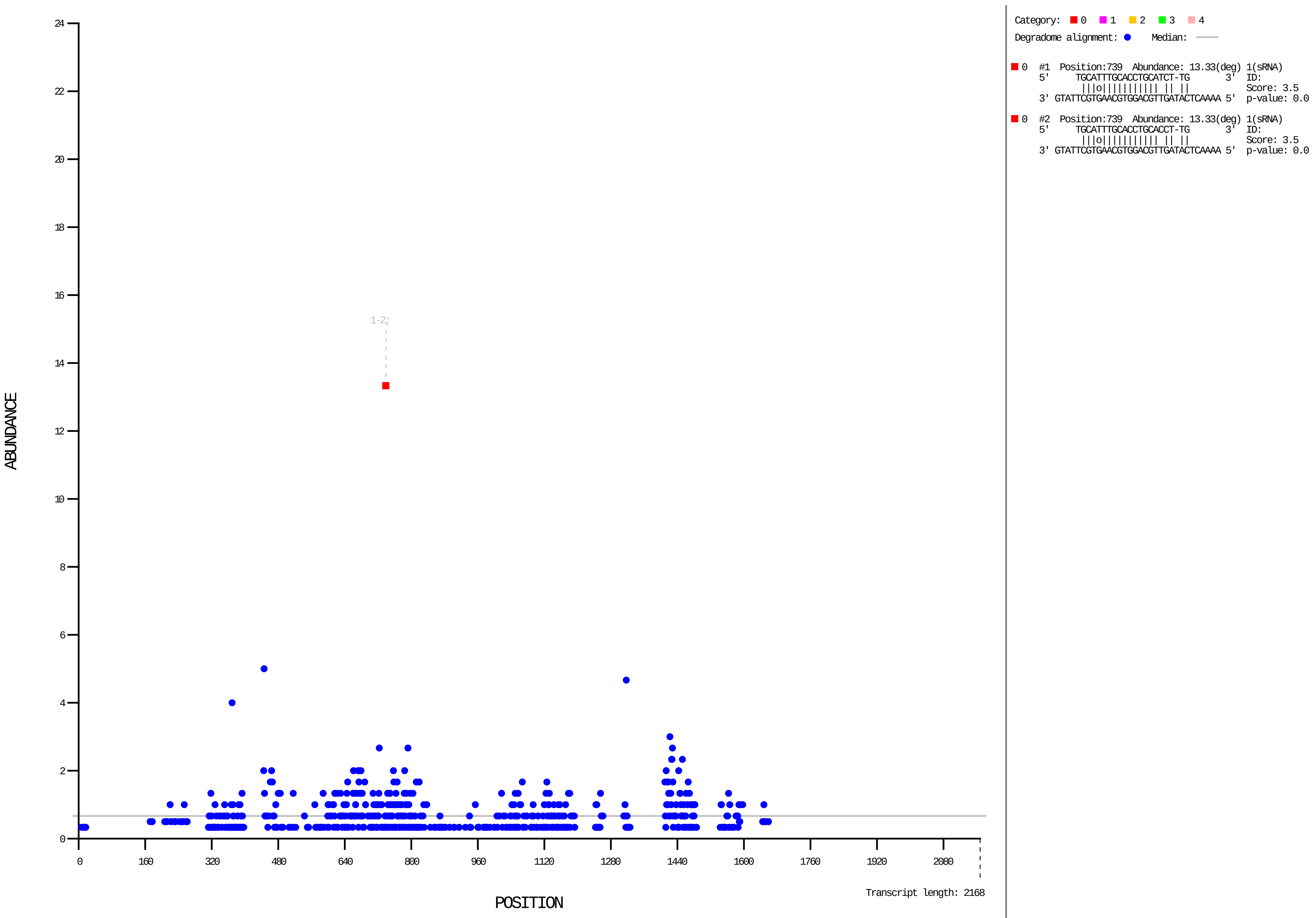

Cs3g16860.2 gene=Cs3g16860 CDS=44-1603

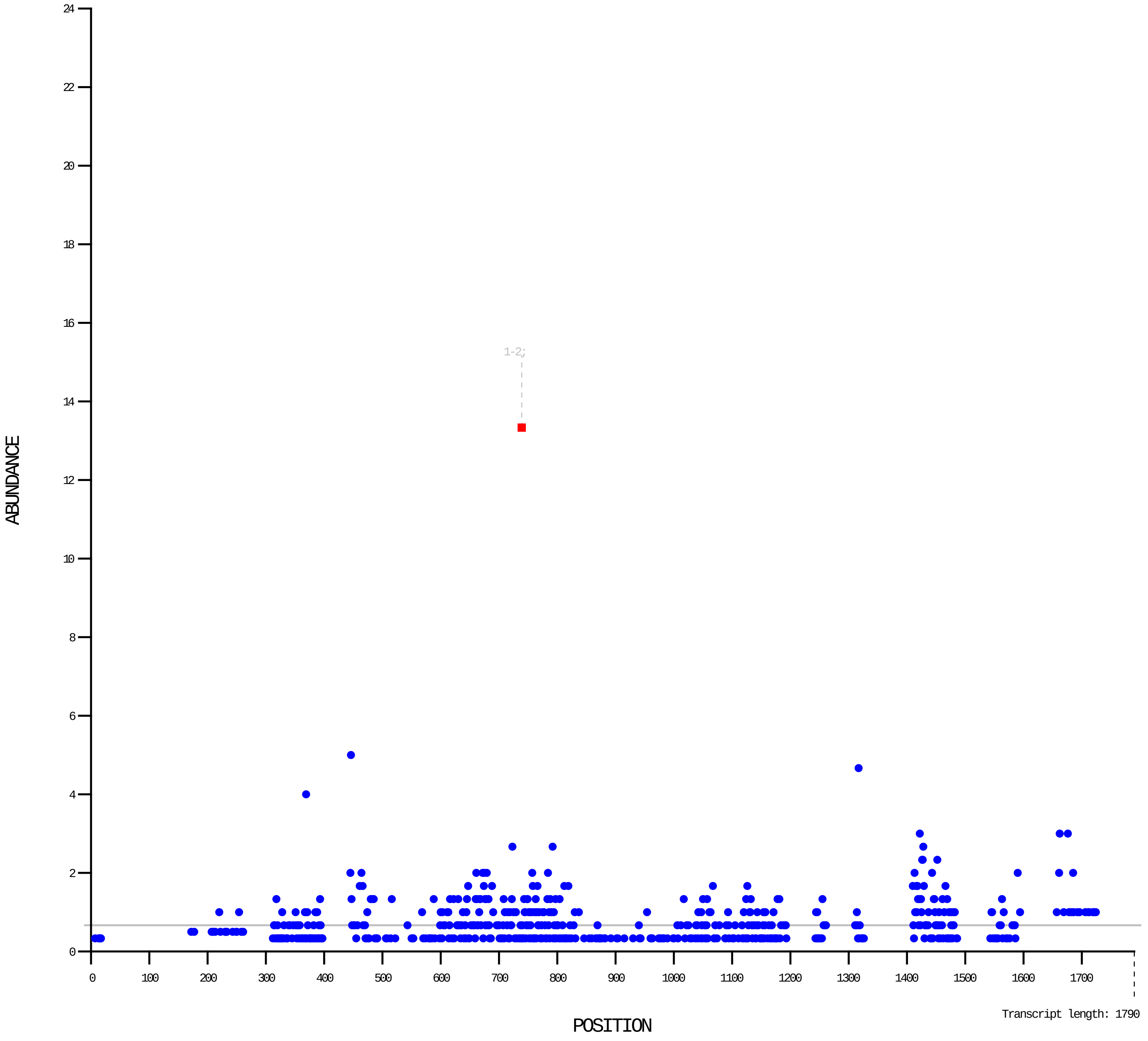


```
Category: 0 1 2 3 4
Degradome alignment:     Median:

0  #1  Position:739  Abundance: 13.33(deg) 1(sRNA)
   5'     TGCATTTGCACCTGCATCT-TG       3'  ID:
           |||o||||||||||| || ||           Score: 3.5
   3' GTATTCGTGAACGTGGACGTTGATACTCAAAA 5'  p-value: 0.01

0  #2  Position:739  Abundance: 13.33(deg) 1(sRNA)
   5'     TGCATTTGCACCTGCACCT-TG       3'  ID:
           |||o||||||||||| || ||           Score: 3.5
   3' GTATTCGTGAACGTGGACGTTGATACTCAAAA 5'  p-value: 0.02
```

Cs3g16860.3 gene=Cs3g16860 CDS=44-1468

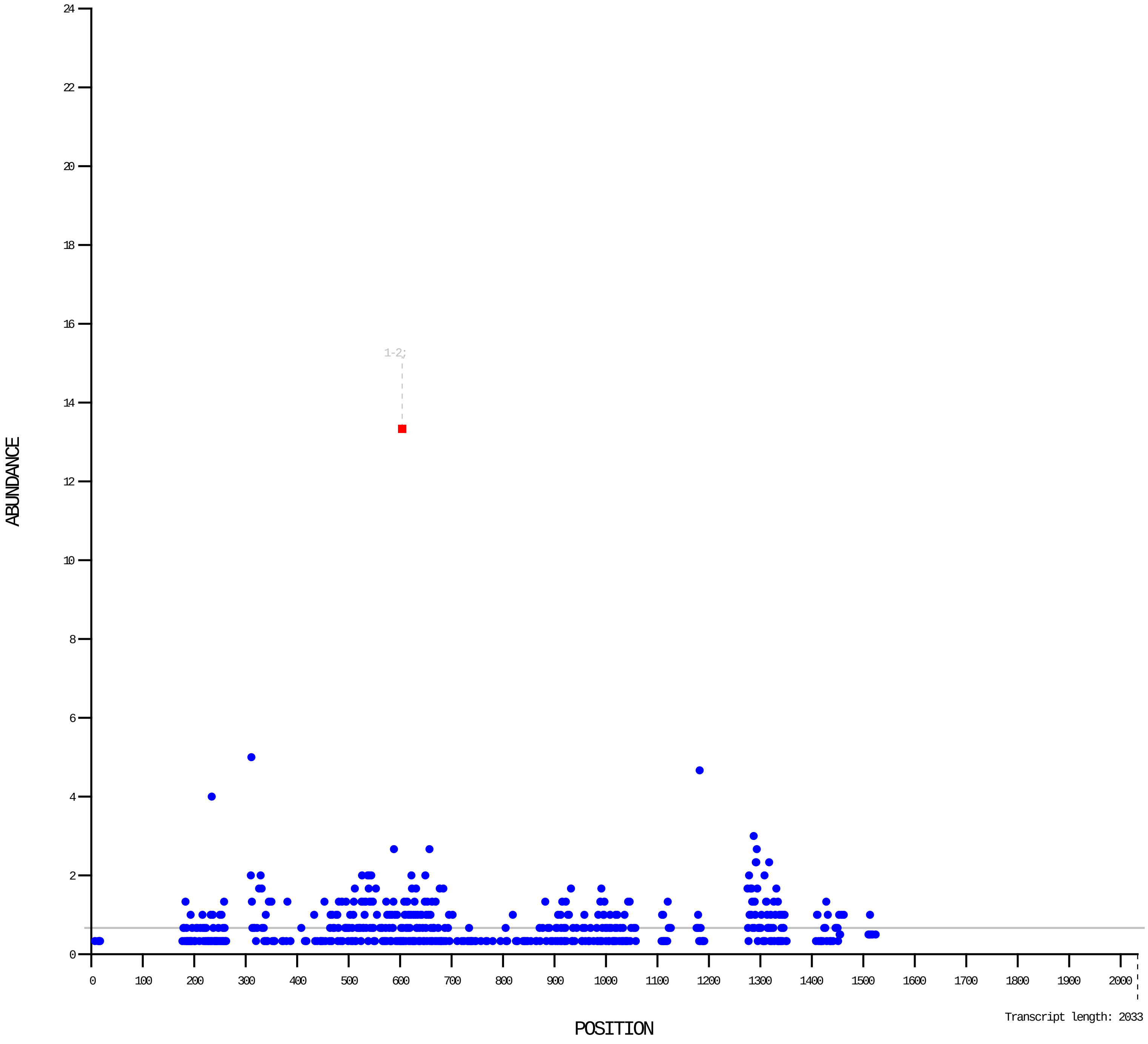

Category: 0 1 2 3 4
Degradome alignment: Median:

0 #1 Position:604 Abundance: 13.33(deg) 1(sRNA)
5' TGCATTTGCACCTGCATCT-TG 3' ID:
|||o||||||||||| || ||
3' GTATTCGTGAACGTGGACGTTGATACTCAAAA 5'
Score: 3.5
p-value: 0.0

0 #2 Position:604 Abundance: 13.33(deg) 1(sRNA)
5' TGCATTTGCACCTGCACCT-TG 3' ID:
|||o||||||||||| || ||
3' GTATTCGTGAACGTGGACGTTGATACTCAAAA 5'
Score: 3.5
p-value: 0.0

Cs1g13410.1 gene=Cs1g13410 CDS=96-3065

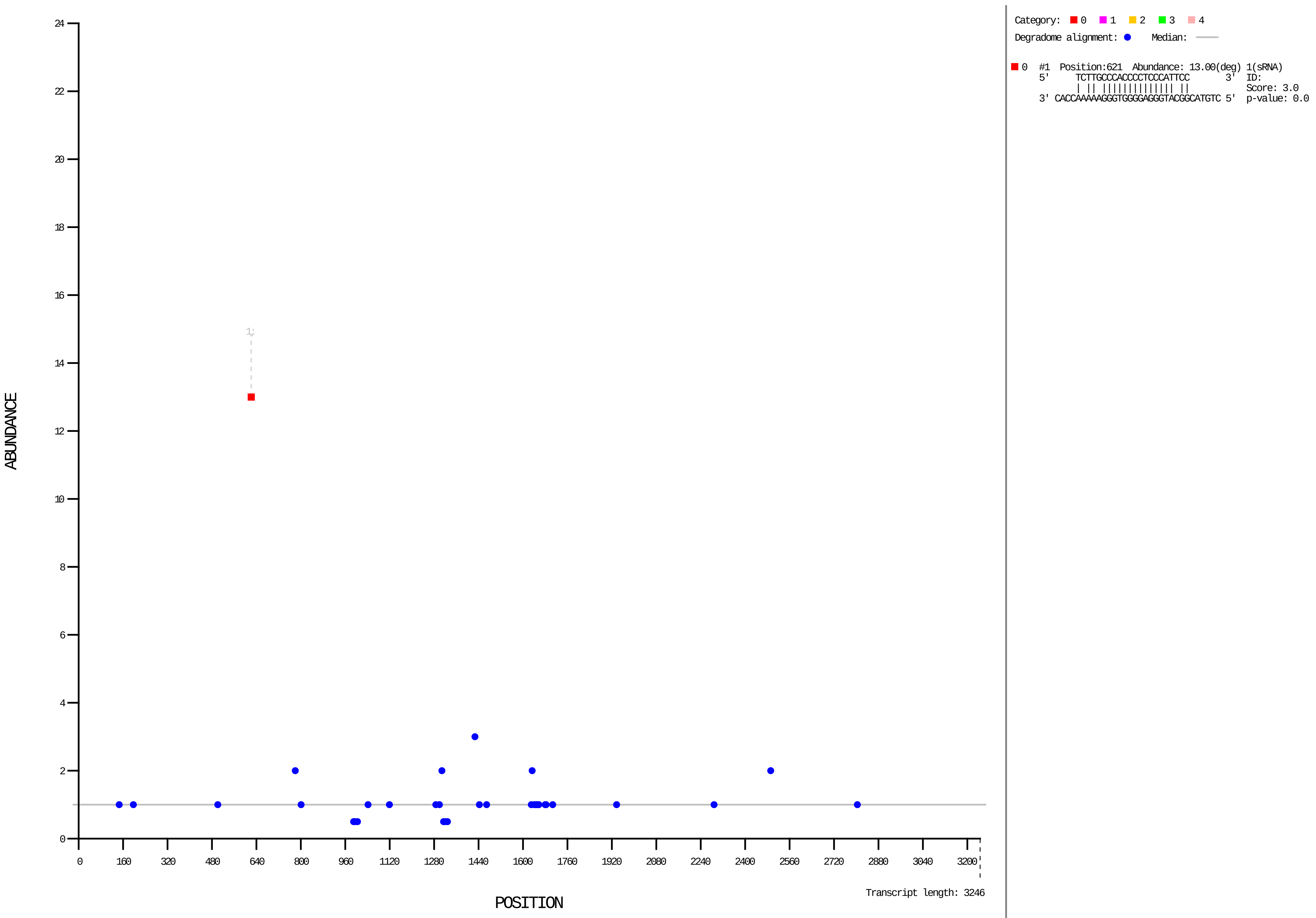

Cs6g18380.1 gene=Cs6g18380 CDS=1-2145

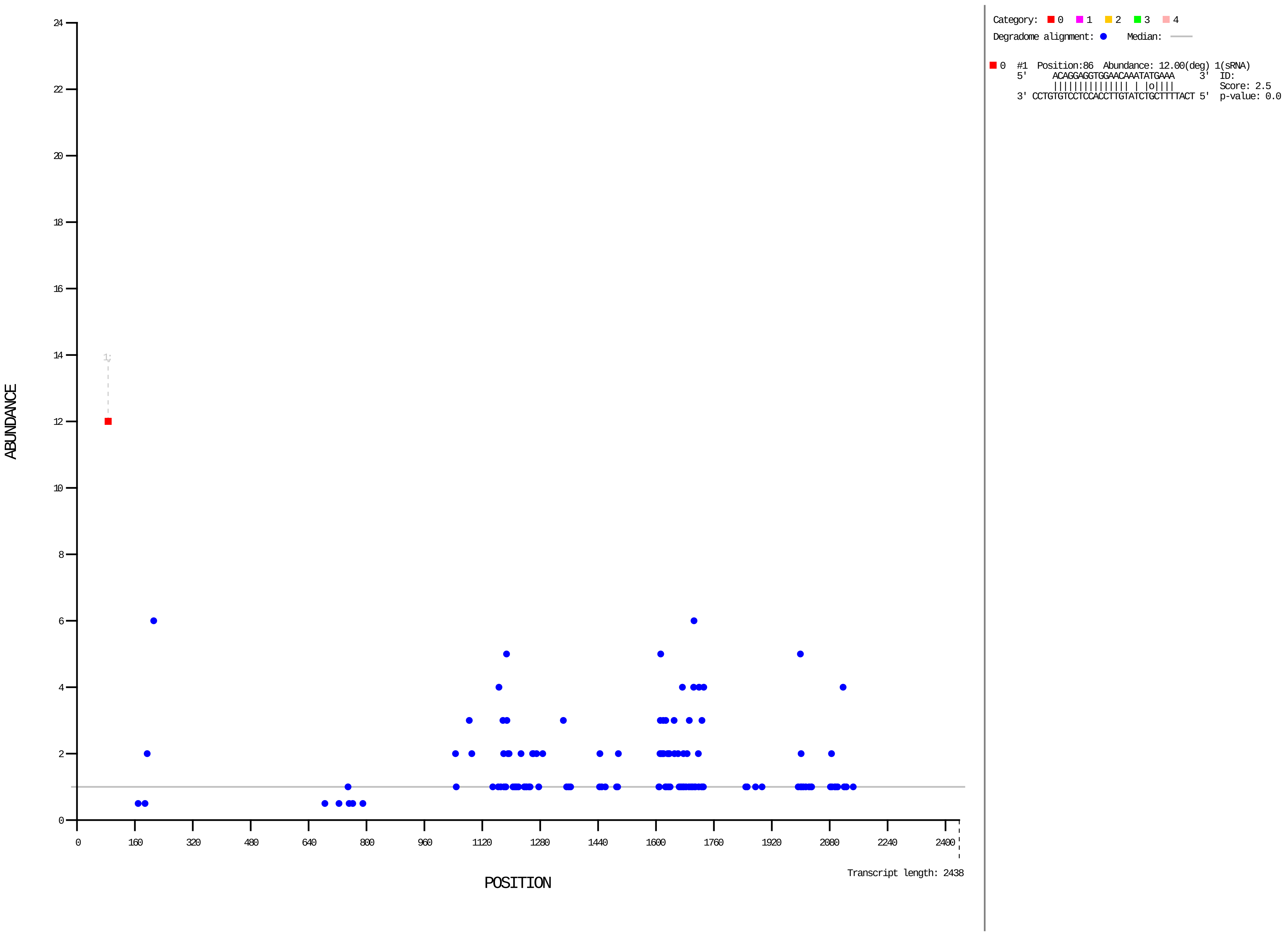

Cs3g05280.1 gene=Cs3g05280 CDS=121-3564

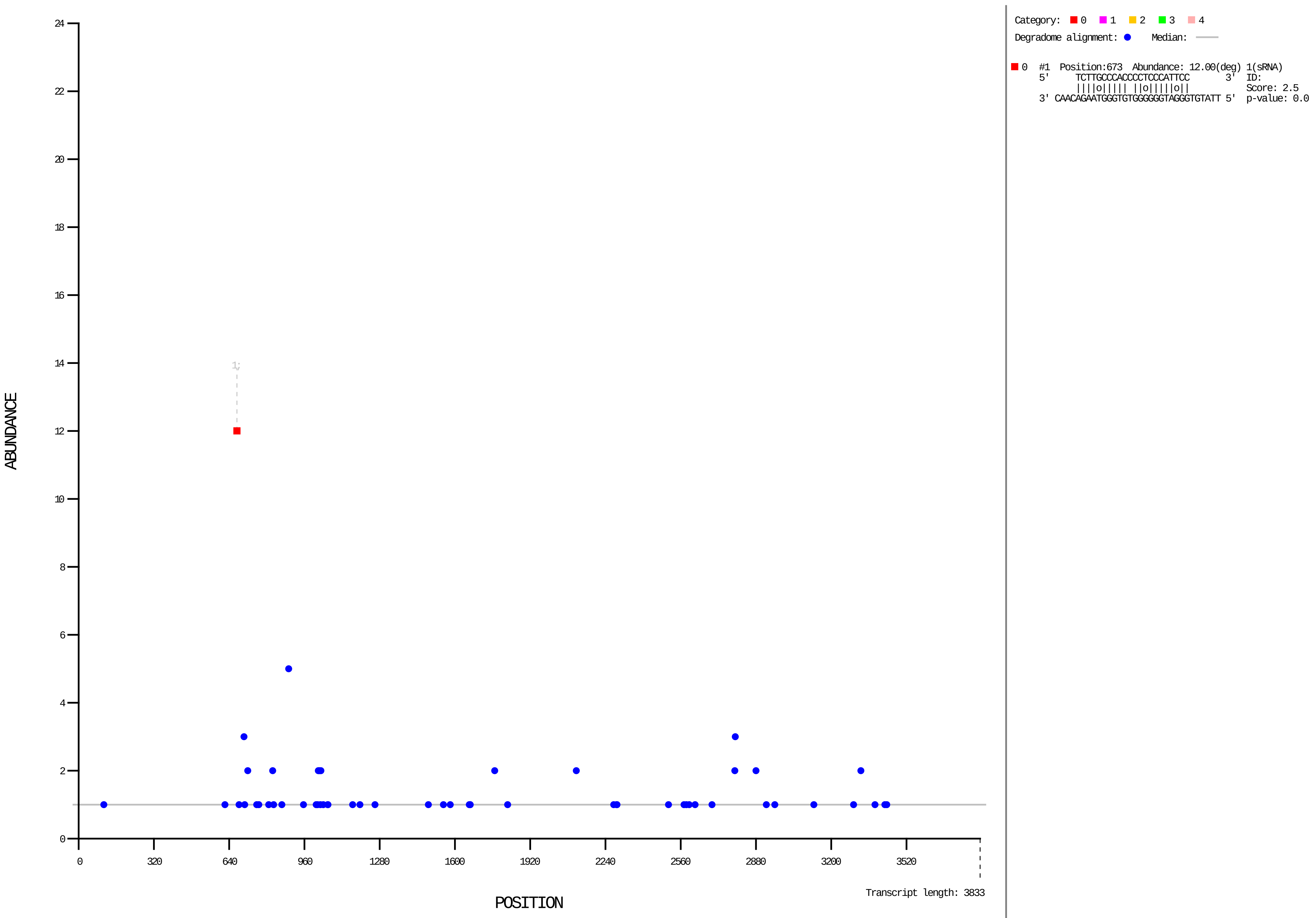

Cs9g07790.1 gene=Cs9g07790 CDS=195-3284

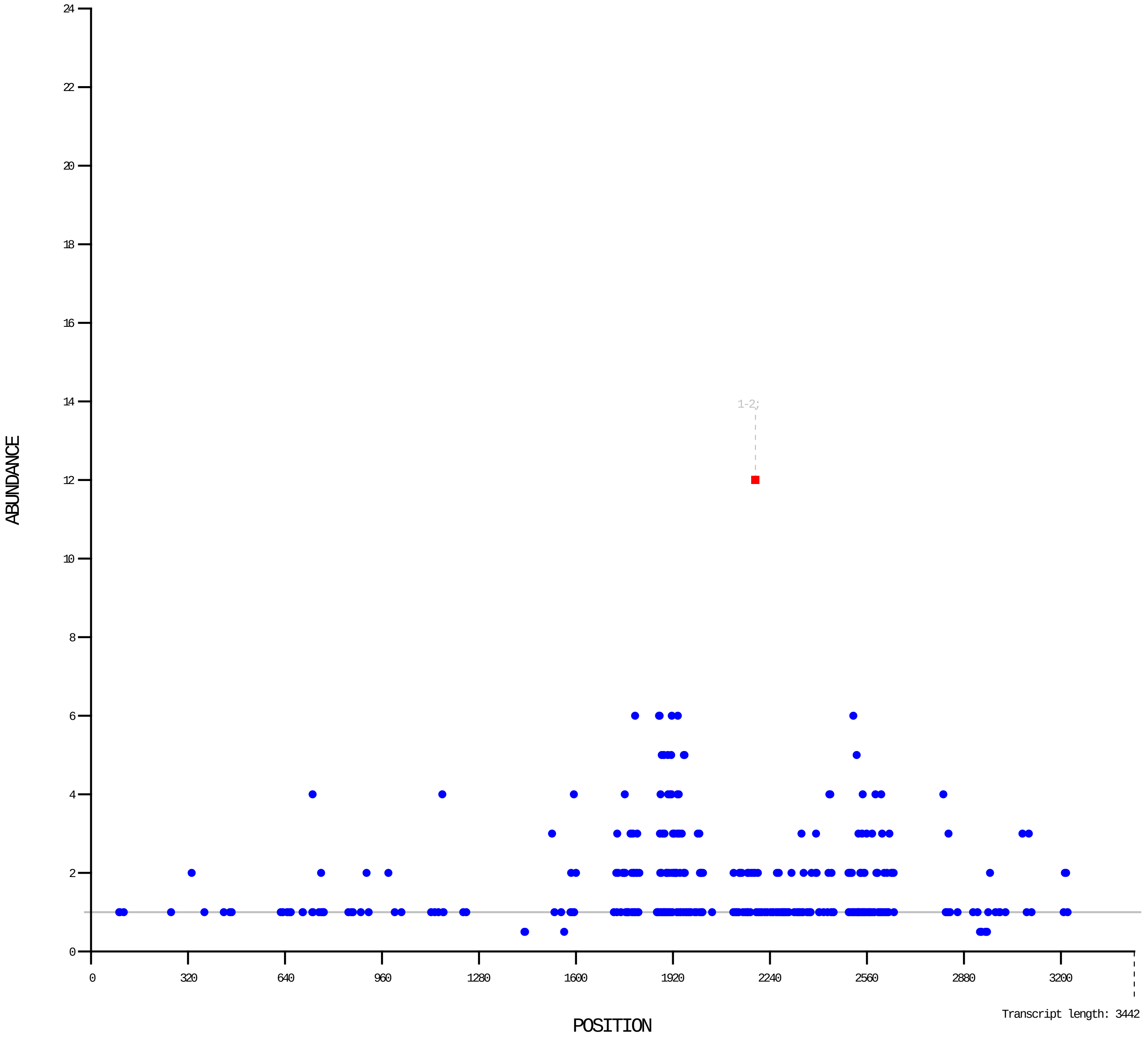


```
Category: 0  1  2  3  4
Degradome alignment:     Median:

0   #1  Position:2192  Abundance: 12.00(deg) 1(sRNA)
    5'     TTCCACAGCTTTCTTGAACTG          3'  ID:
           ||||||||||||||| | |o               Score: 3.5
    3' TTAGAAGGTGTCGAAAGAAATGGGGGCCTGCT 5'  p-value: 0.01

0   #2  Position:2192  Abundance: 12.00(deg) 1(sRNA)
    5'     TTCCACGGCTTTCTTGAACGT          3'  ID:
           ||||||o|||||||| | | o              Score: 4.0
    3' TTAGAAGGTGTCGAAAGAAATGGGGGCCTGCT 5'  p-value: 0.03
```

orange1.1t03734.1 gene=orange1.1t03734 CDS=197-2974

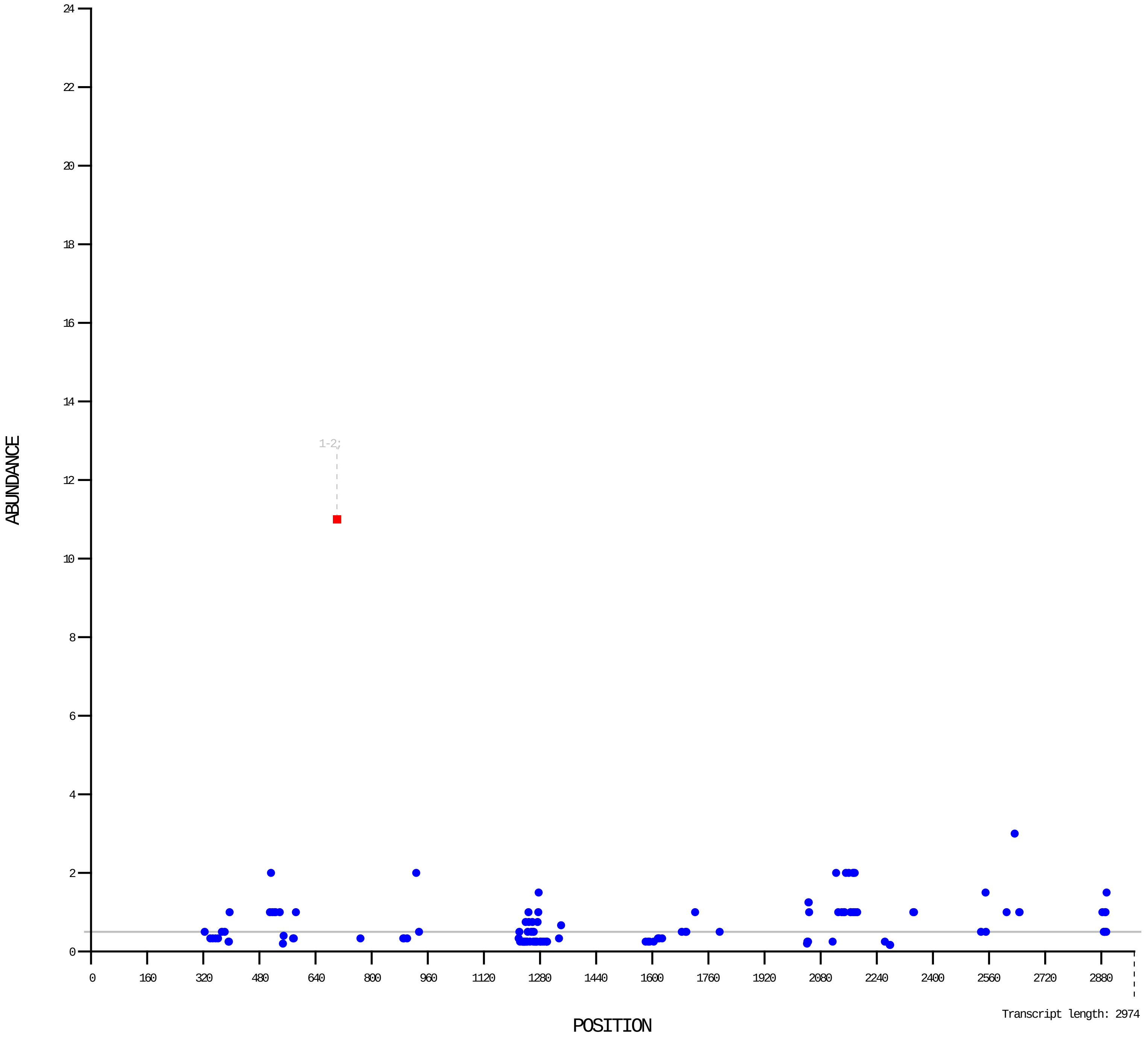


```
Category: 0  1  2  3  4
Degradome alignment:    Median:

0  #1  Position:701  Abundance: 11.00(deg) 1(sRNA)
   5'     TCTTCCCTATGCCTCCCATTCC       3'  ID:
          | ||||||||||||||||| ||           Score: 2.0
   3' CATCAAAAGGGATACGGAGGGTATGGTTGTTA 5'  p-value: 0.0

0  #2  Position:701  Abundance: 11.00(deg) 1(sRNA)
   5'     TCTTACCTATGCCACCCATTCC       3'  ID:
          | || |||||||| ||||| ||           Score: 4.0
   3' CATCAAAAGGGATACGGAGGGTATGGTTGTTA 5'  p-value: 0.0
```

Cs2g18470.1 gene=Cs2g18470 CDS=284-4603

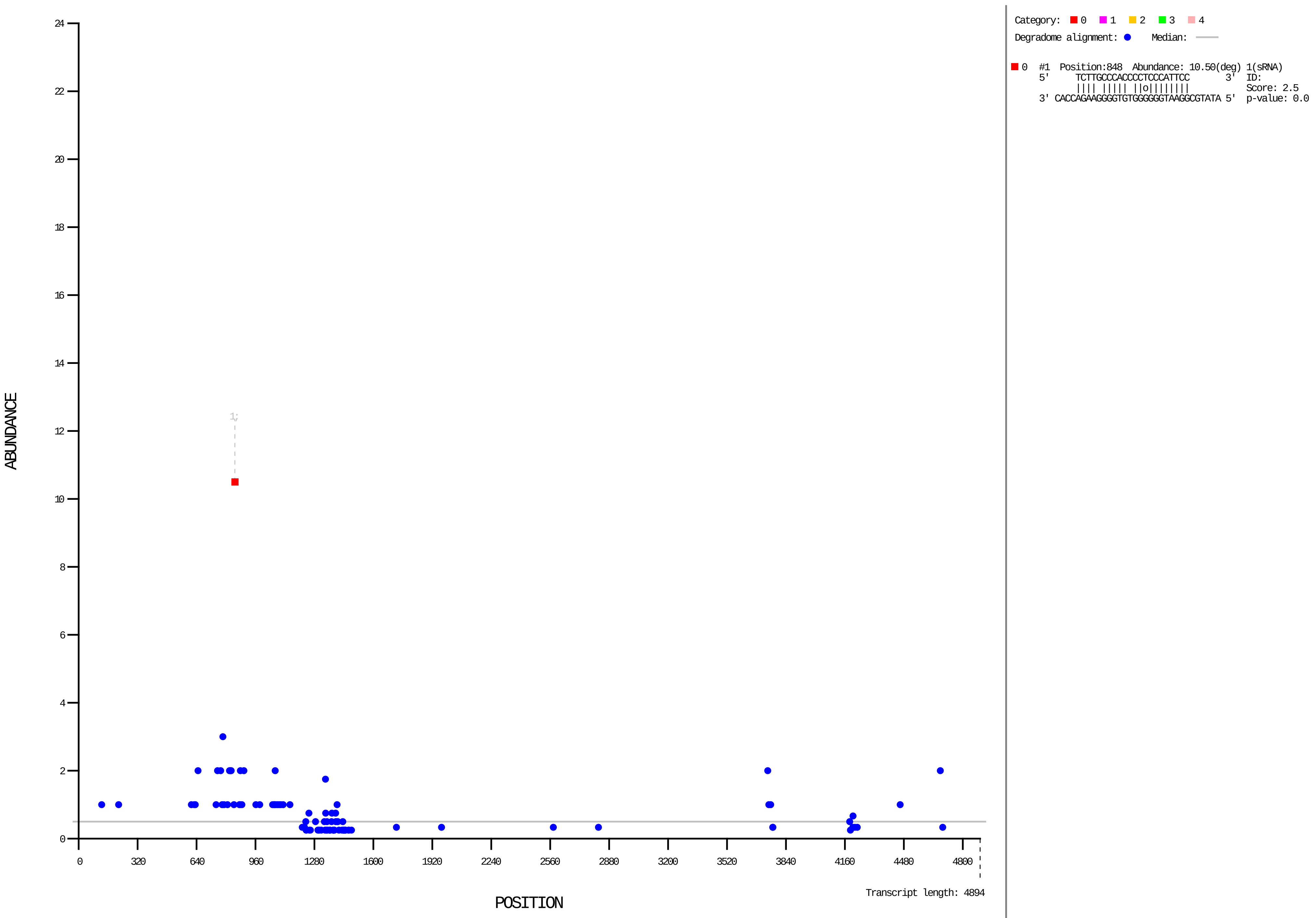

Cs2g18480.1 gene=Cs2g18480 CDS=67-4203

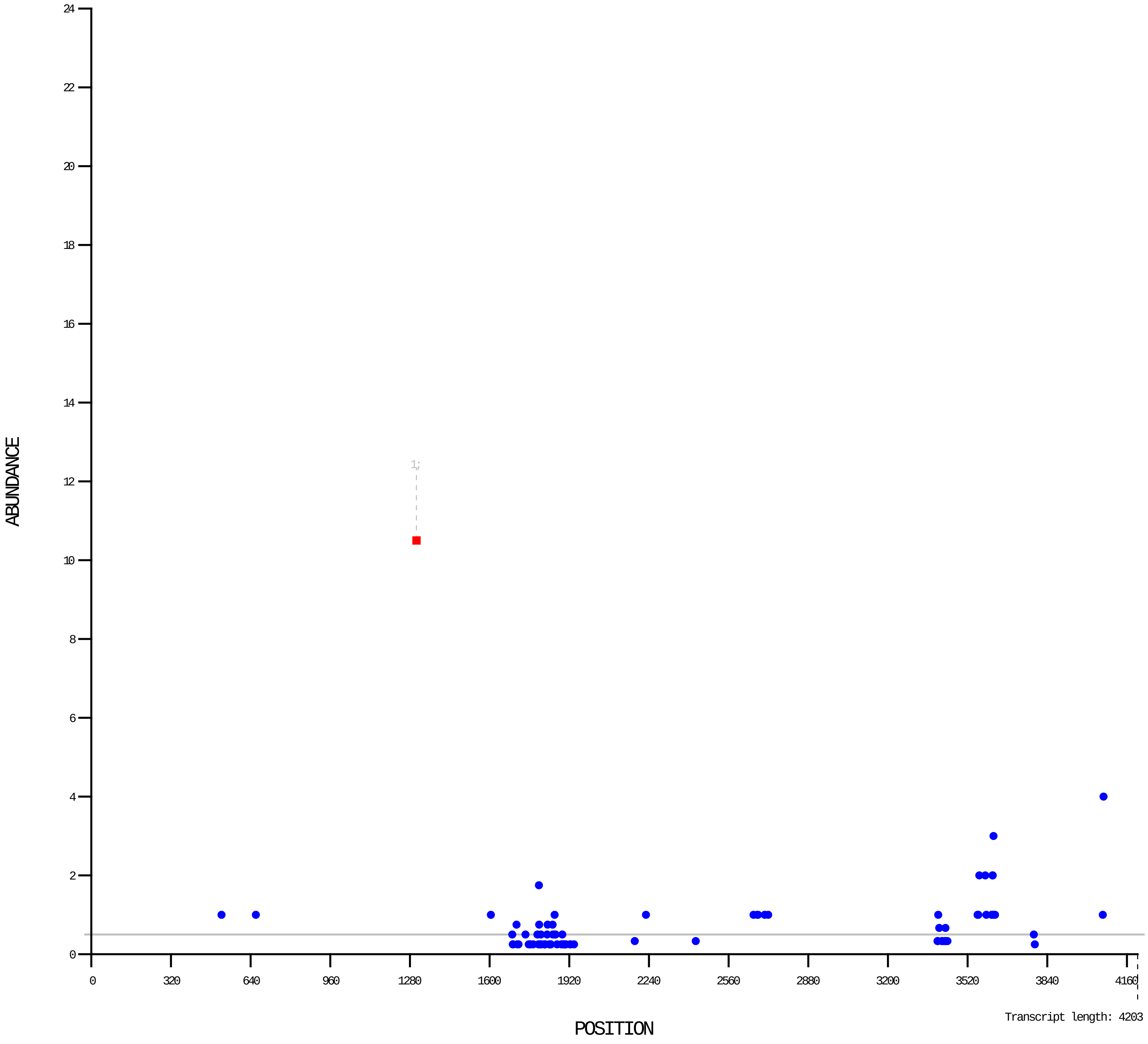

Category: 0 1 2 3 4
Degradome alignment: Median:

```
0  #1  Position:1306  Abundance: 10.50(deg) 1(sRNA)
   5'     TCTTGCCCACCCCTCCCATTCC       3'  ID:
          |||| ||||| || ||||||||           Score: 3.0
   3' CACCAGAAGGGGTGTGGCGGGTAAGGTGTATA 5'  p-value: 0.0
```

Cs5g12260.1 gene=Cs5g12260 CDS=458-2131

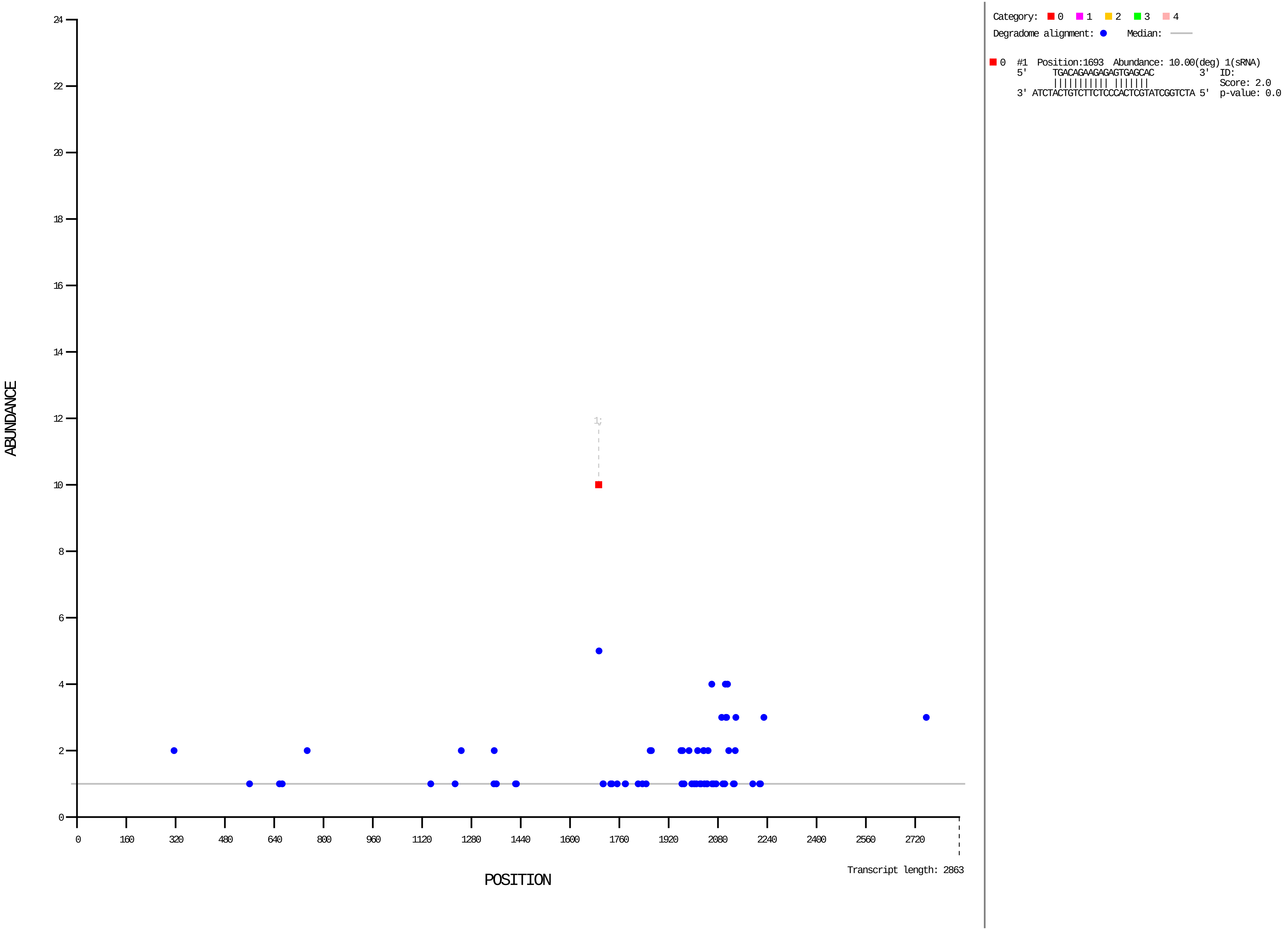

Cs7g30410.1 gene=Cs7g30410 CDS=40-1779

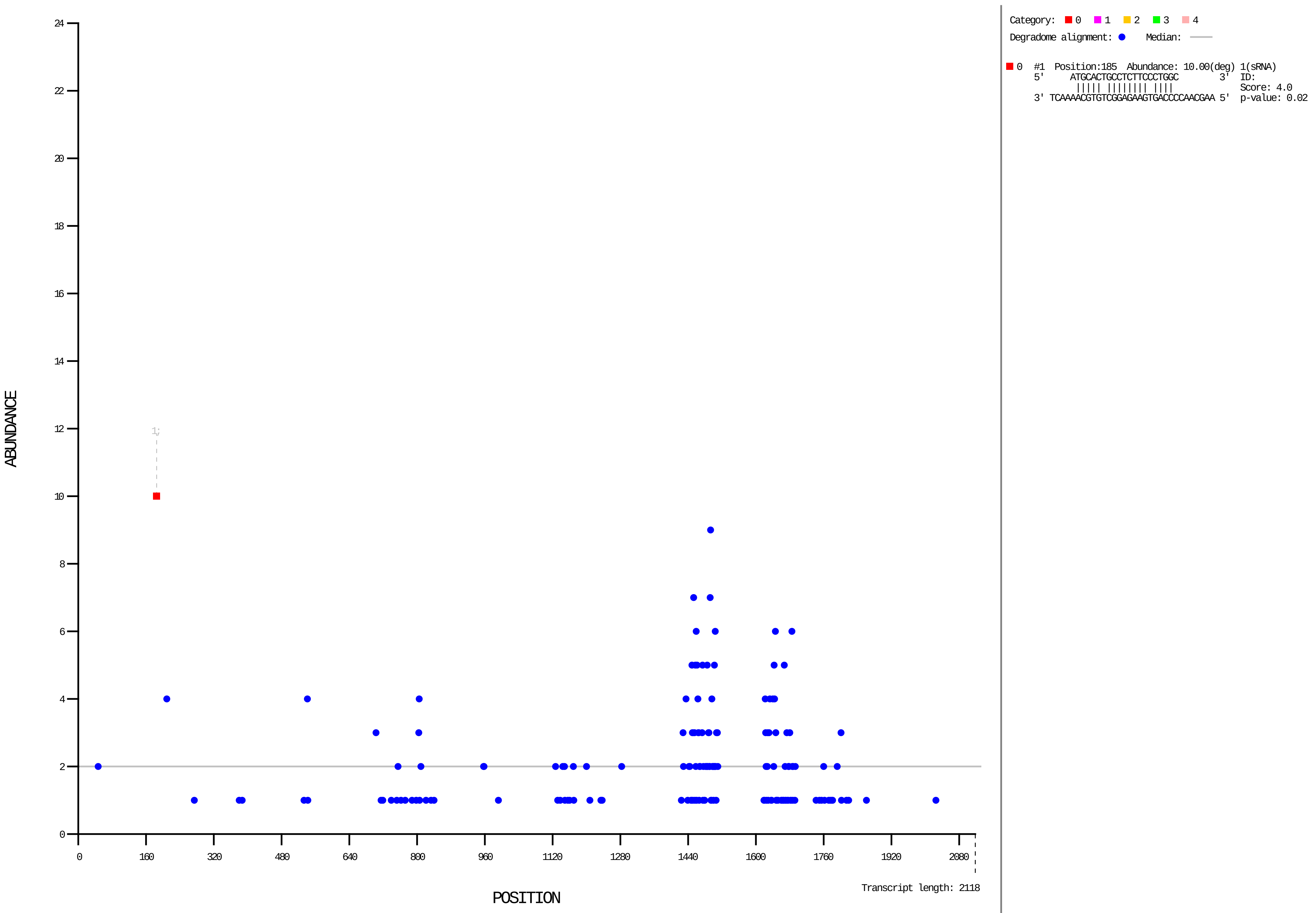

Cs1g19720.1 gene=Cs1g19720 CDS=236-1567

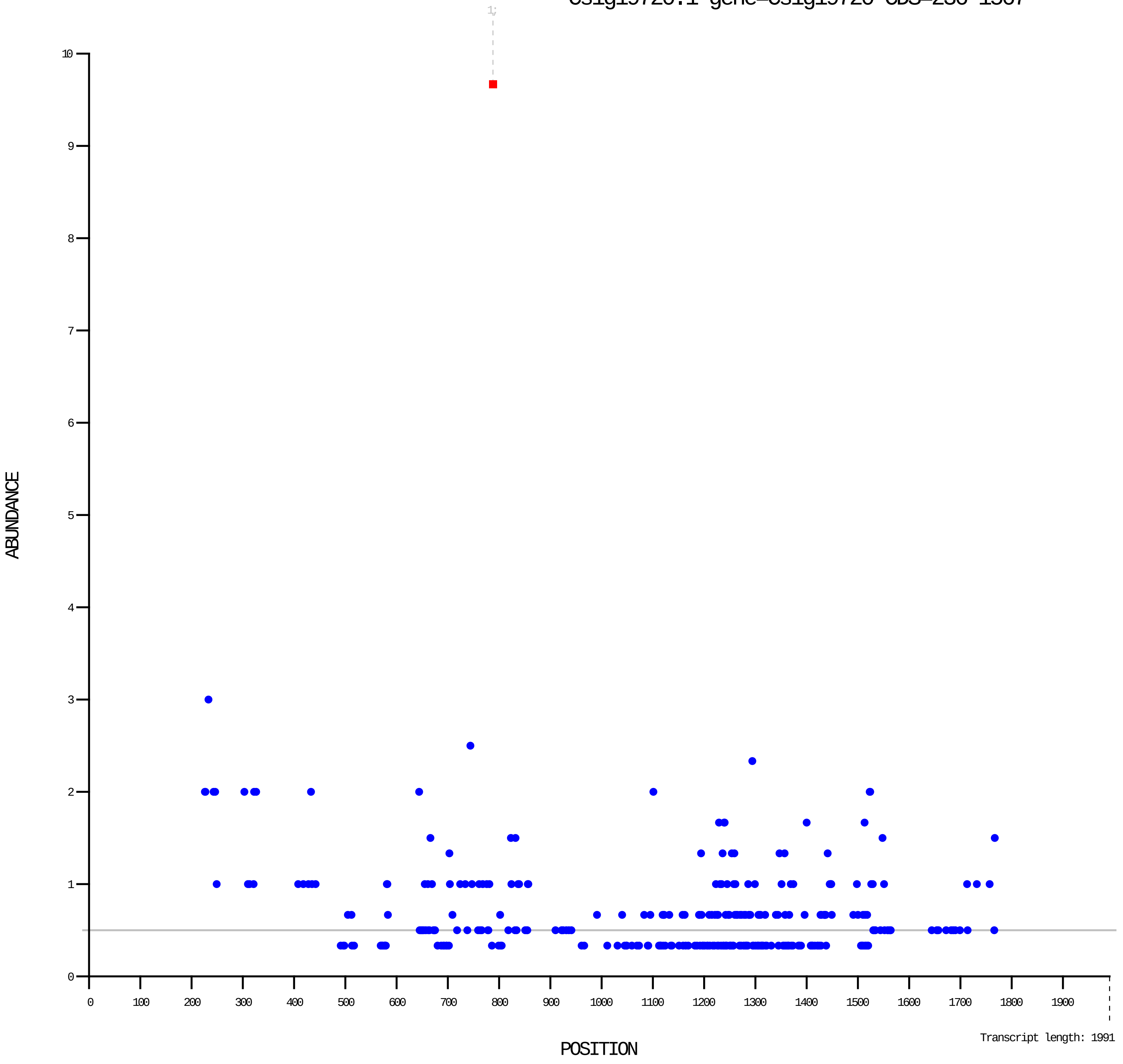

Cs1g19720.2 gene=Cs1g19720 CDS=12-1136

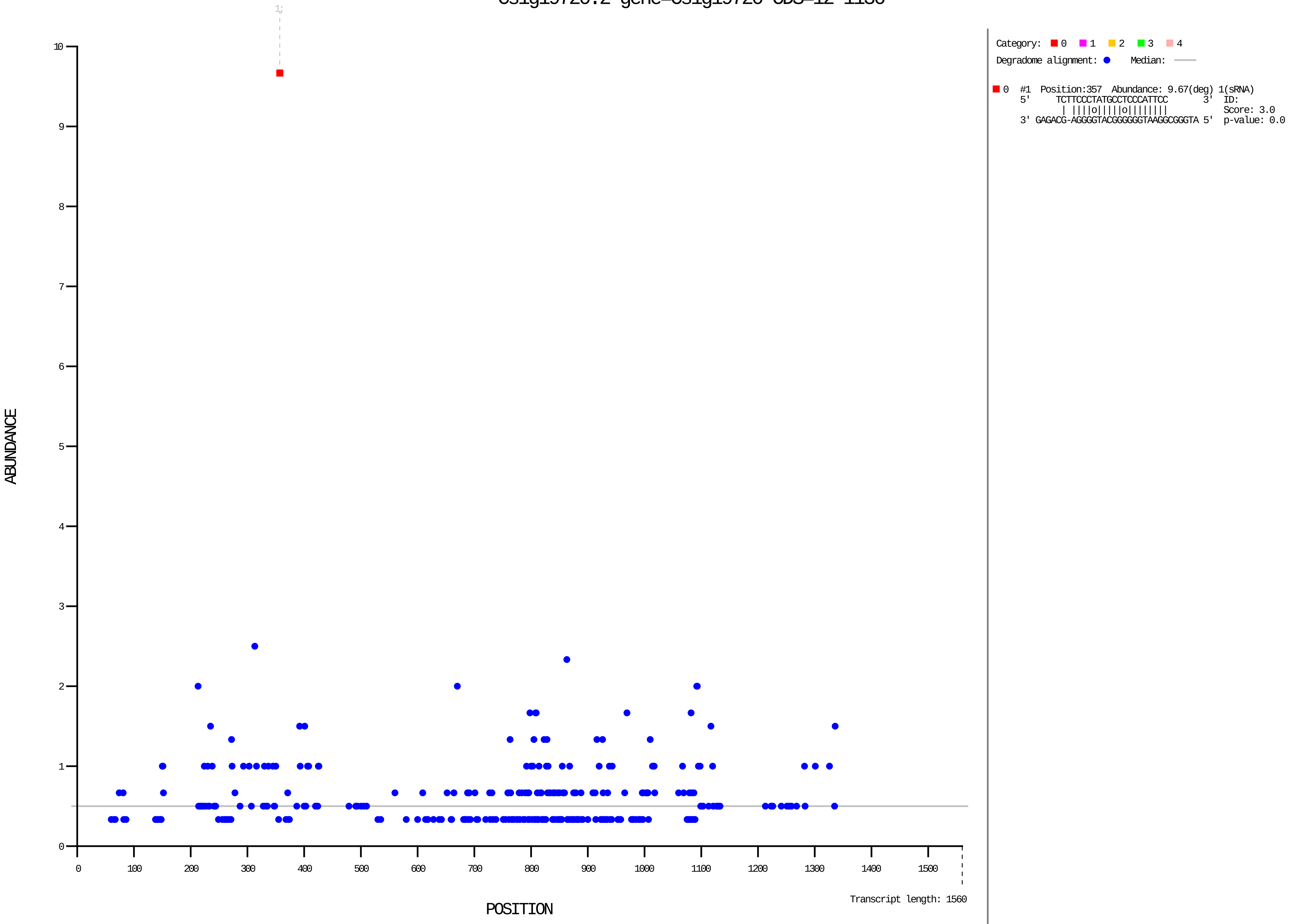

Cs1g19760.1 gene=Cs1g19760 CDS=1-1332

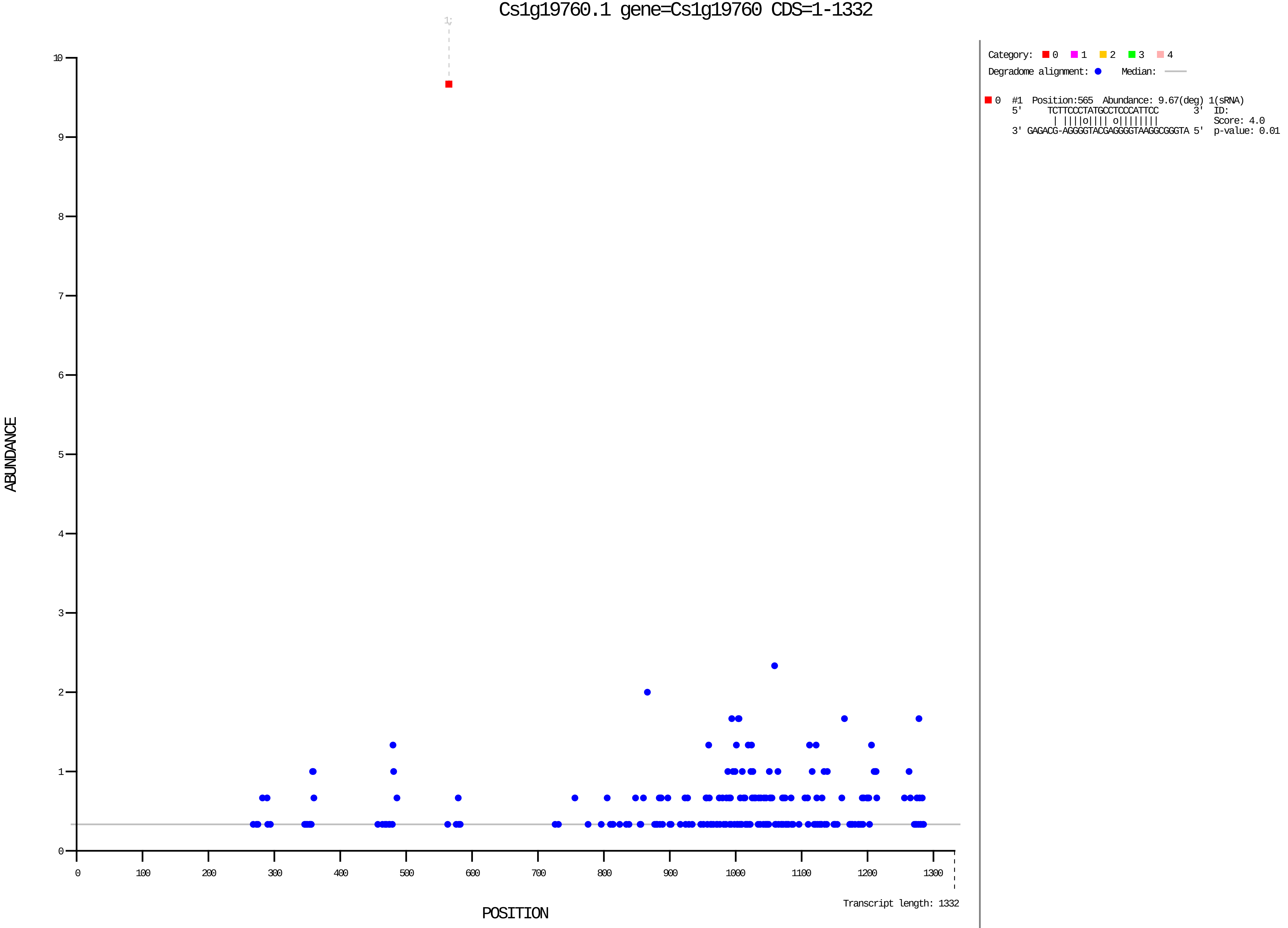

Cs9g17340.1 gene=Cs9g17340 CDS=252-1823

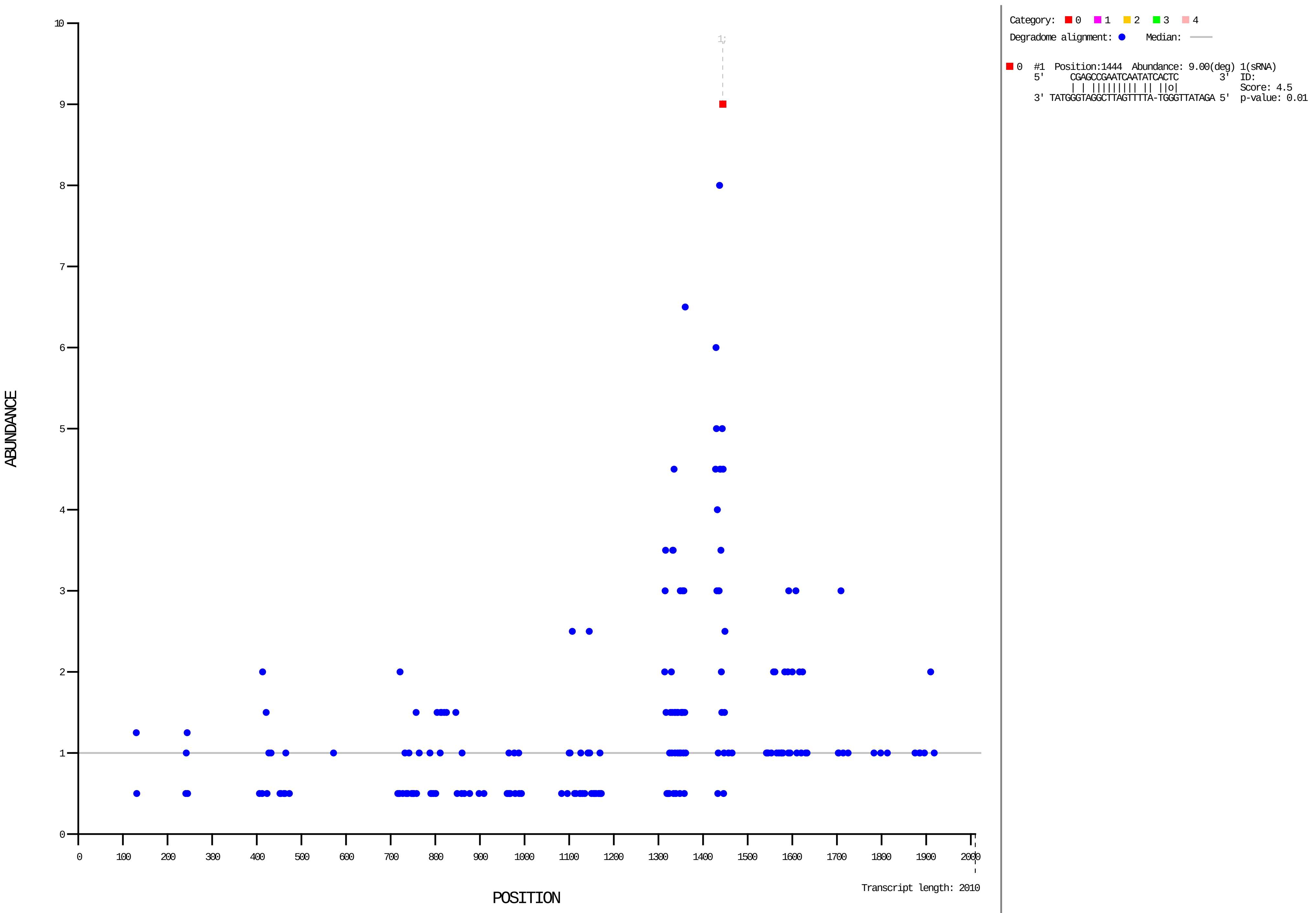

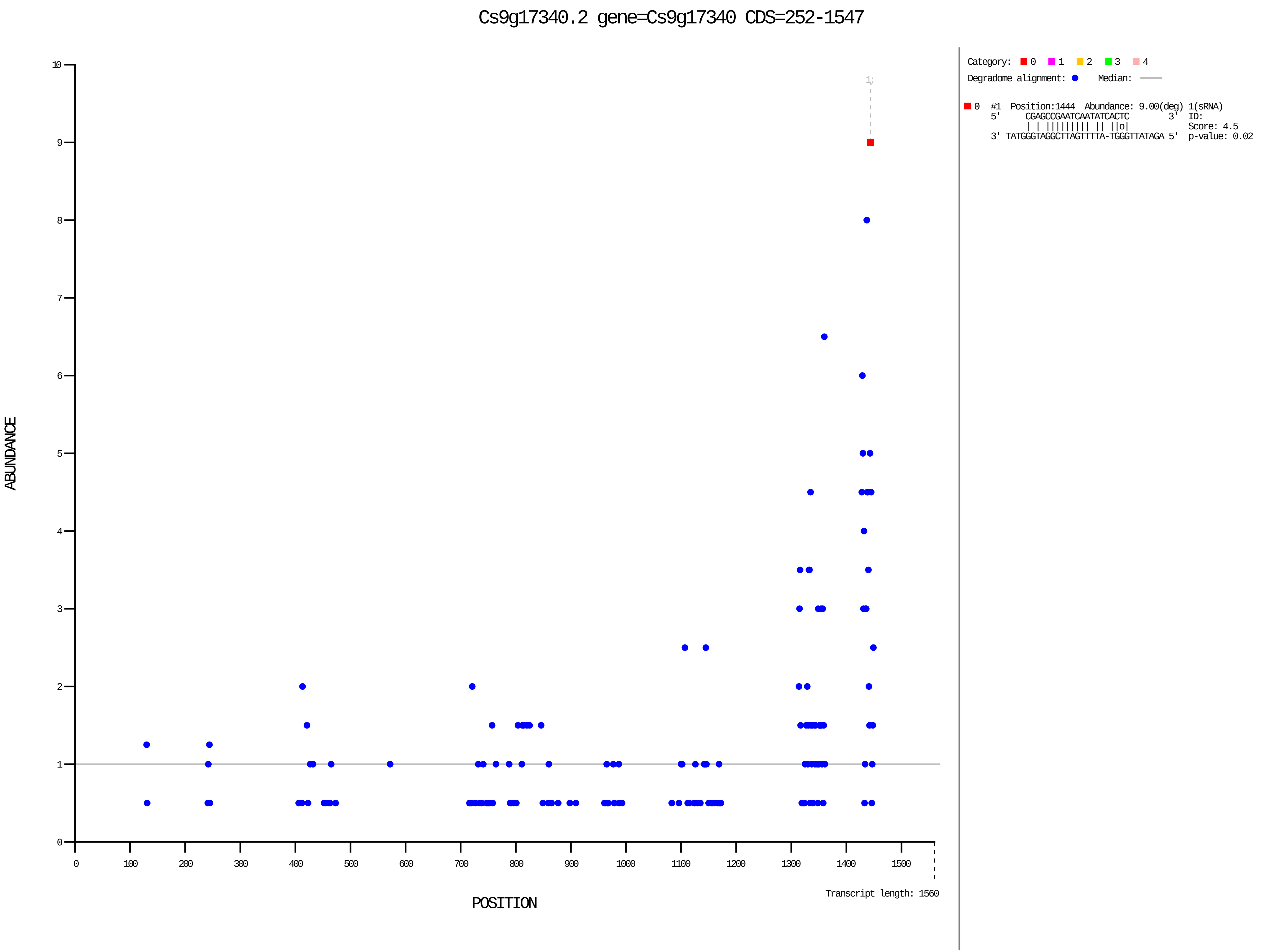

Cs9g17340.2 gene=Cs9g17340 CDS=252-1547
ABUNDANCE
POSITION
Transcript length: 1560
Category: 0 1 2 3 4
Degradome alignment:
Median:
0 #1 Position:1444 Abundance: 9.00(deg) 1(sRNA)
5' CGAGCCGAATCAATATCACTC 3' ID:
| | ||||||||| || ||o|
Score: 4.5
3' TATGGGTAGGCTTAGTTTTA-TGGGTTATAGA 5' p-value: 0.02

orange1.1t01829.2 gene=orange1.1t01829 CDS=135-3863

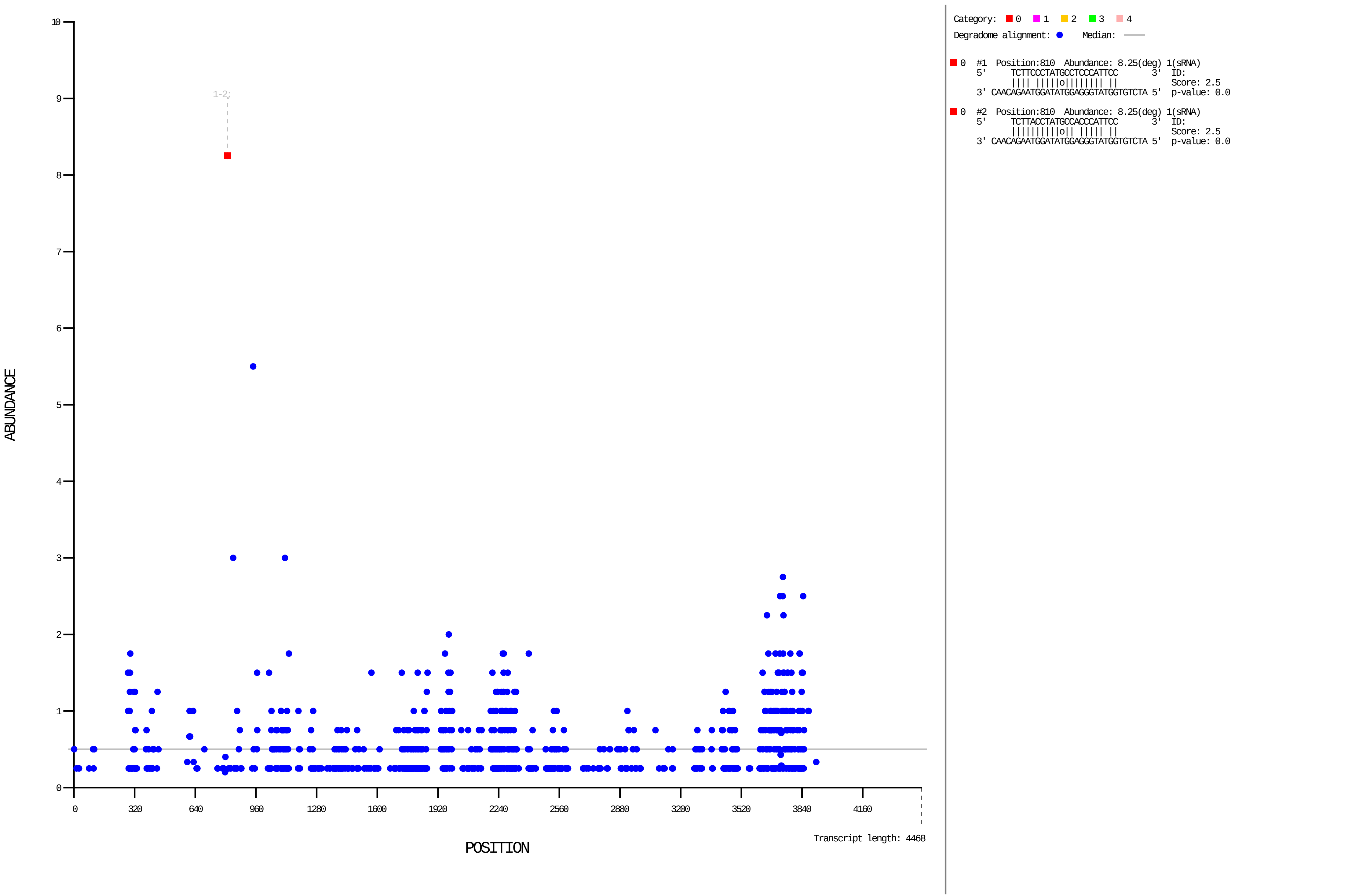

orange1.1t01829.3 gene=orange1.1t01829 CDS=135-3863

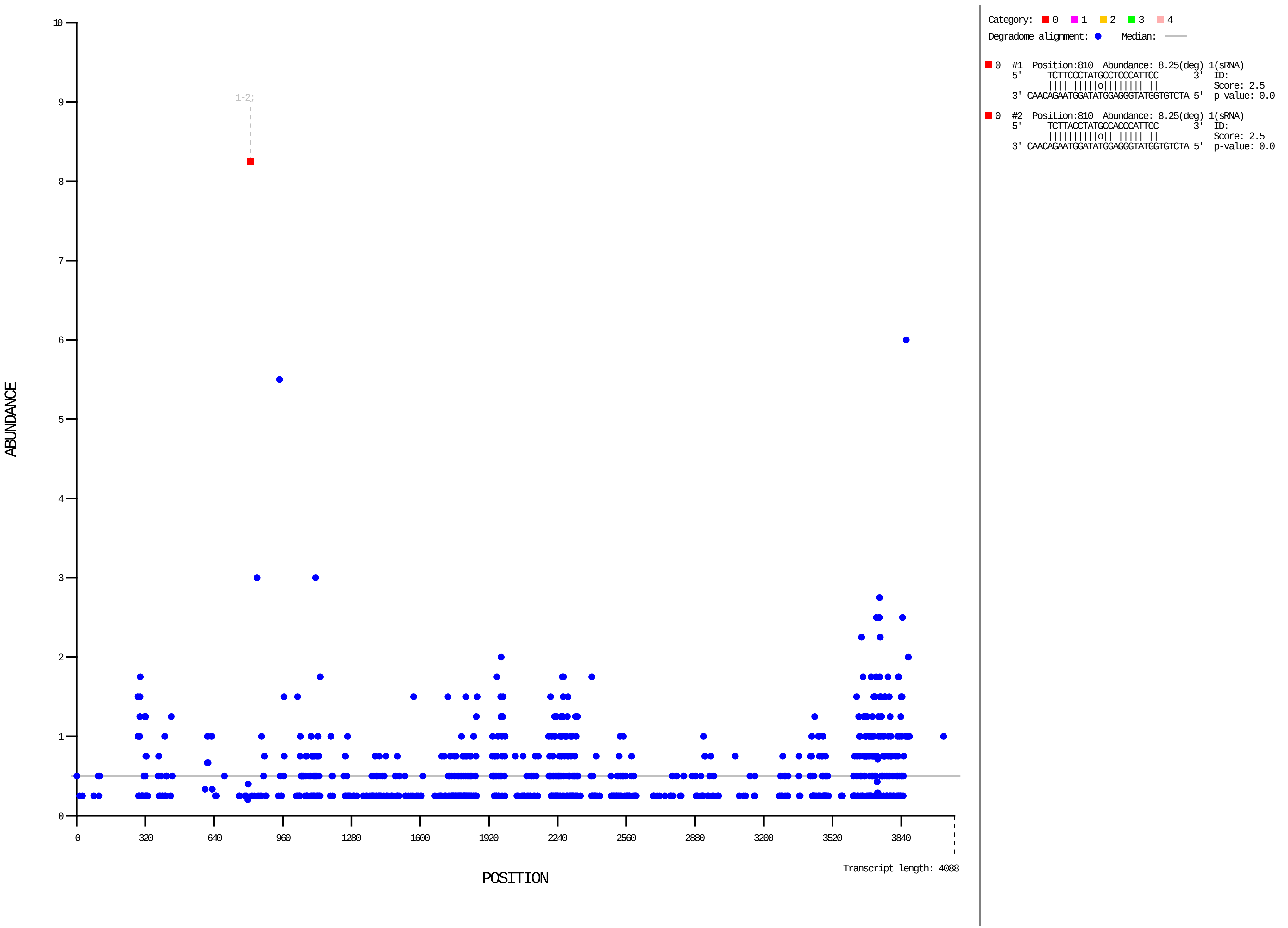

orange1.1t01829.4 gene=orange1.1t01829 CDS=135-3905

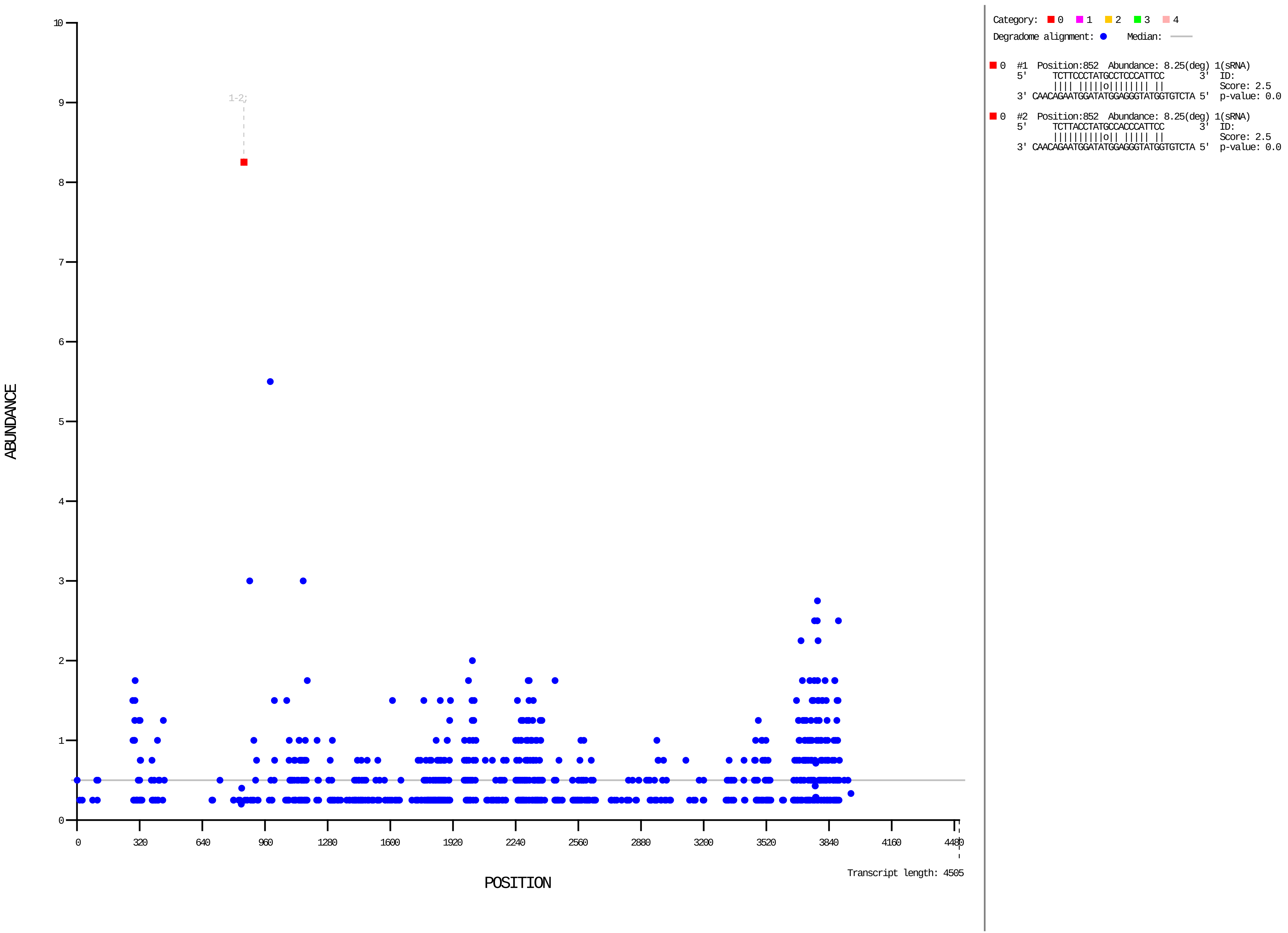

orange1.1t01829.1 gene=orange1.1t01829 CDS=135-3863

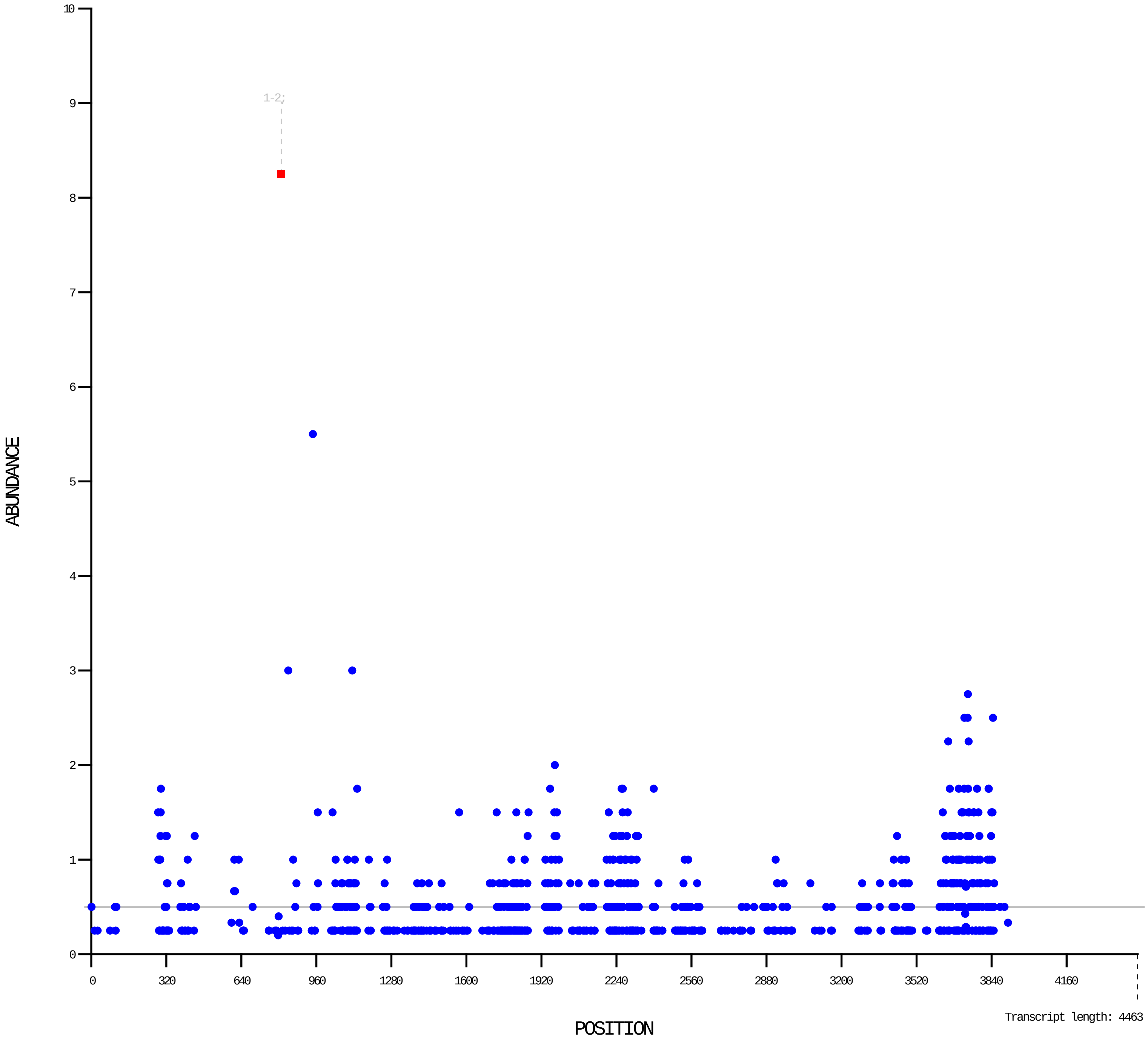

Category: 0 1 2 3 4
Degradome alignment: Median:

0 #1 Position:810 Abundance: 8.25(deg) 1(sRNA)
5' TCTTCCCTATGCCTCCCATTCC 3' ID:
|||| |||||o|||||||| || Score: 2.5
3' CAACAGAATGGATATGGAGGGTATGGTGTCTA 5' p-value: 0.0

0 #2 Position:810 Abundance: 8.25(deg) 1(sRNA)
5' TCTTACCTATGCCACCCATTCC 3' ID:
||||||||||o|| ||||| || Score: 2.5
3' CAACAGAATGGATATGGAGGGTATGGTGTCTA 5' p-value: 0.0

Cs6g03520.3 gene=Cs6g03520 CDS=243-3659

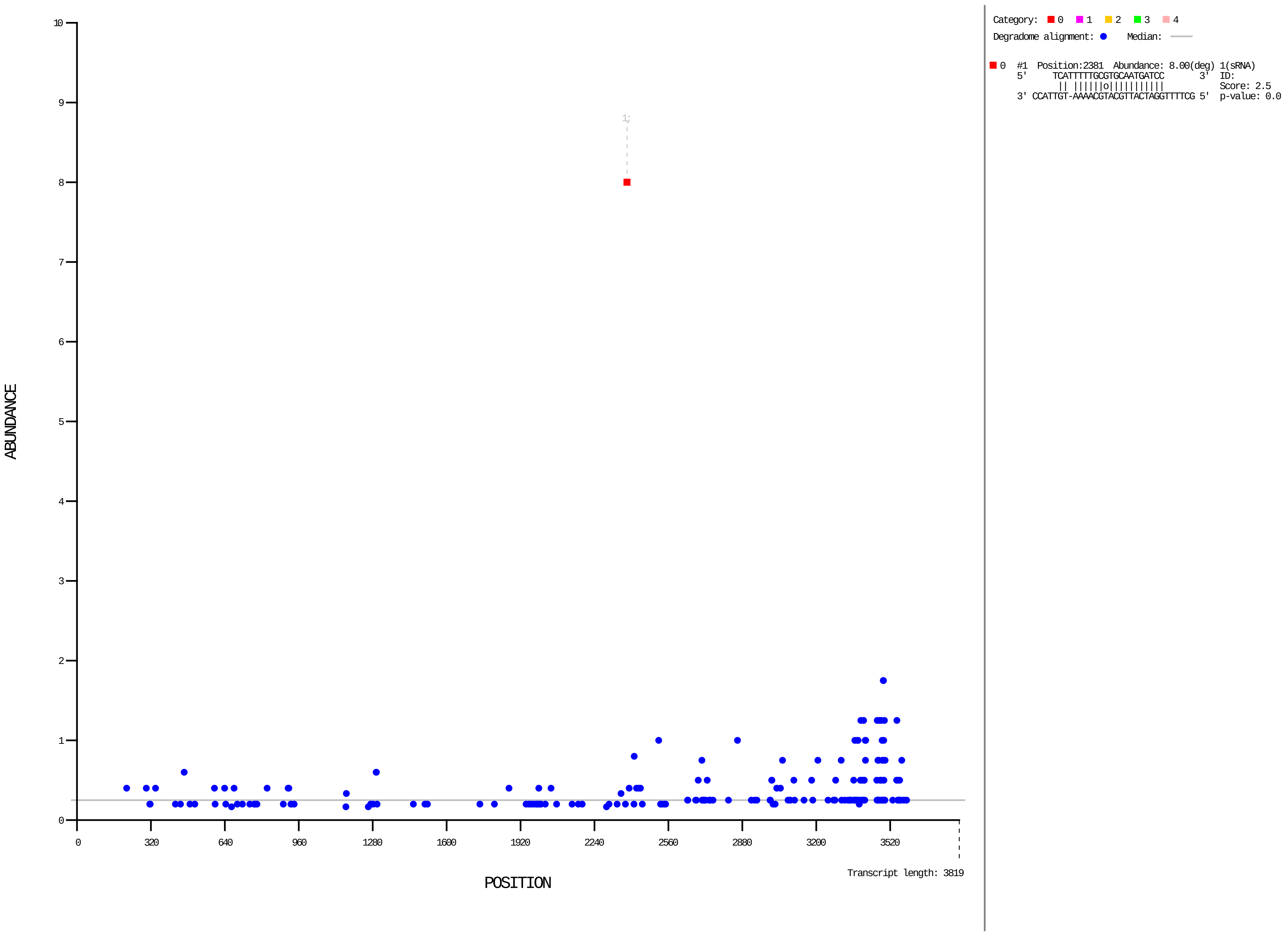

Cs6g03520.4 gene=Cs6g03520 CDS=457-4275

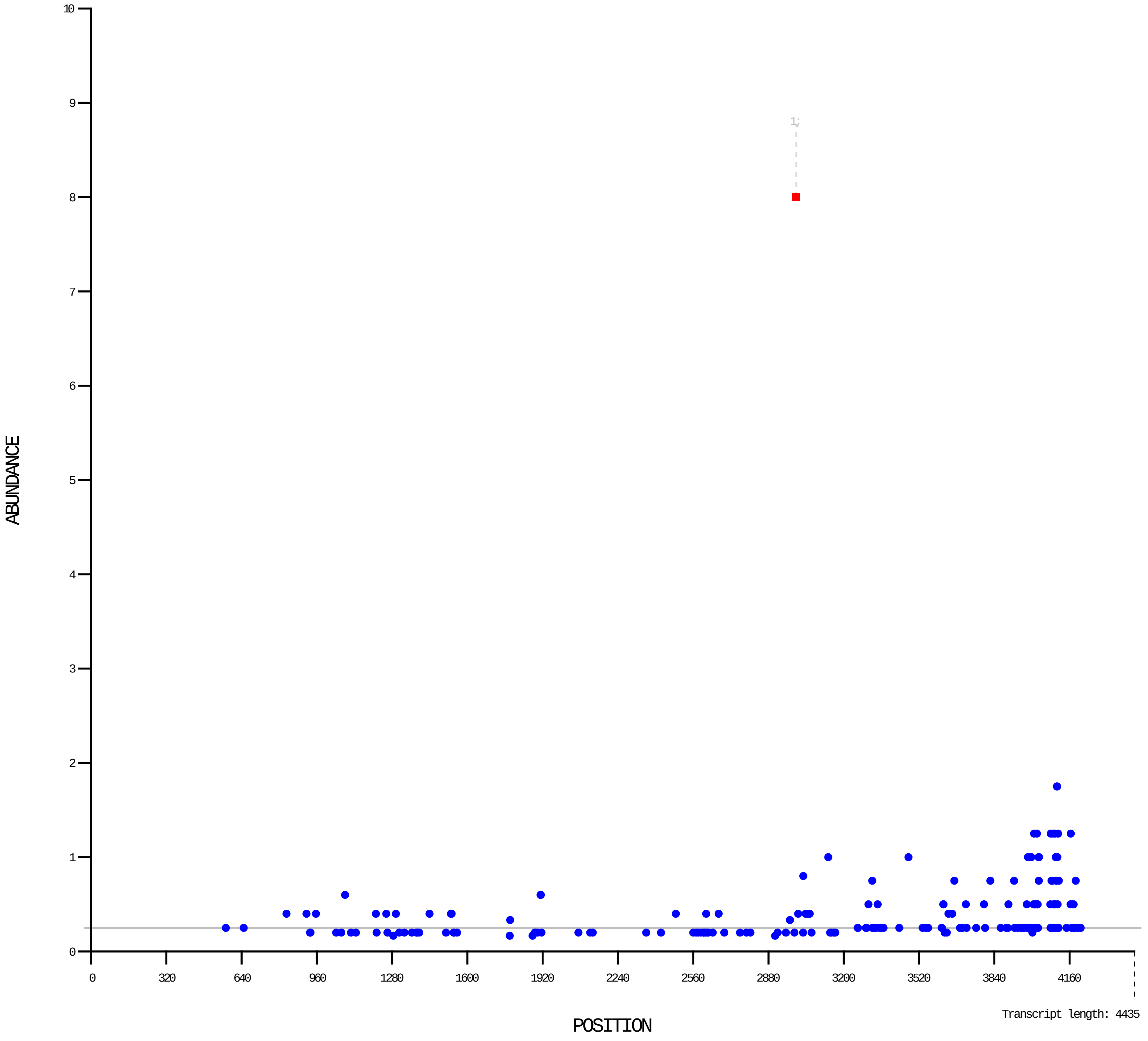


```
Category: 0 1 2 3 4
Degradome alignment:    Median:

0  #1  Position:2997  Abundance: 8.00(deg) 1(sRNA)
   5'     TCATTTTTGCGTGCAATGATCC       3'  ID:
           || ||||||o|||||||||||           Score: 2.5
   3' CCATTGT-AAAACGTACGTTACTAGGTTTTCG 5'  p-value: 0.0
```

Cs6g03520.1 gene=Cs6g03520 CDS=223-4413

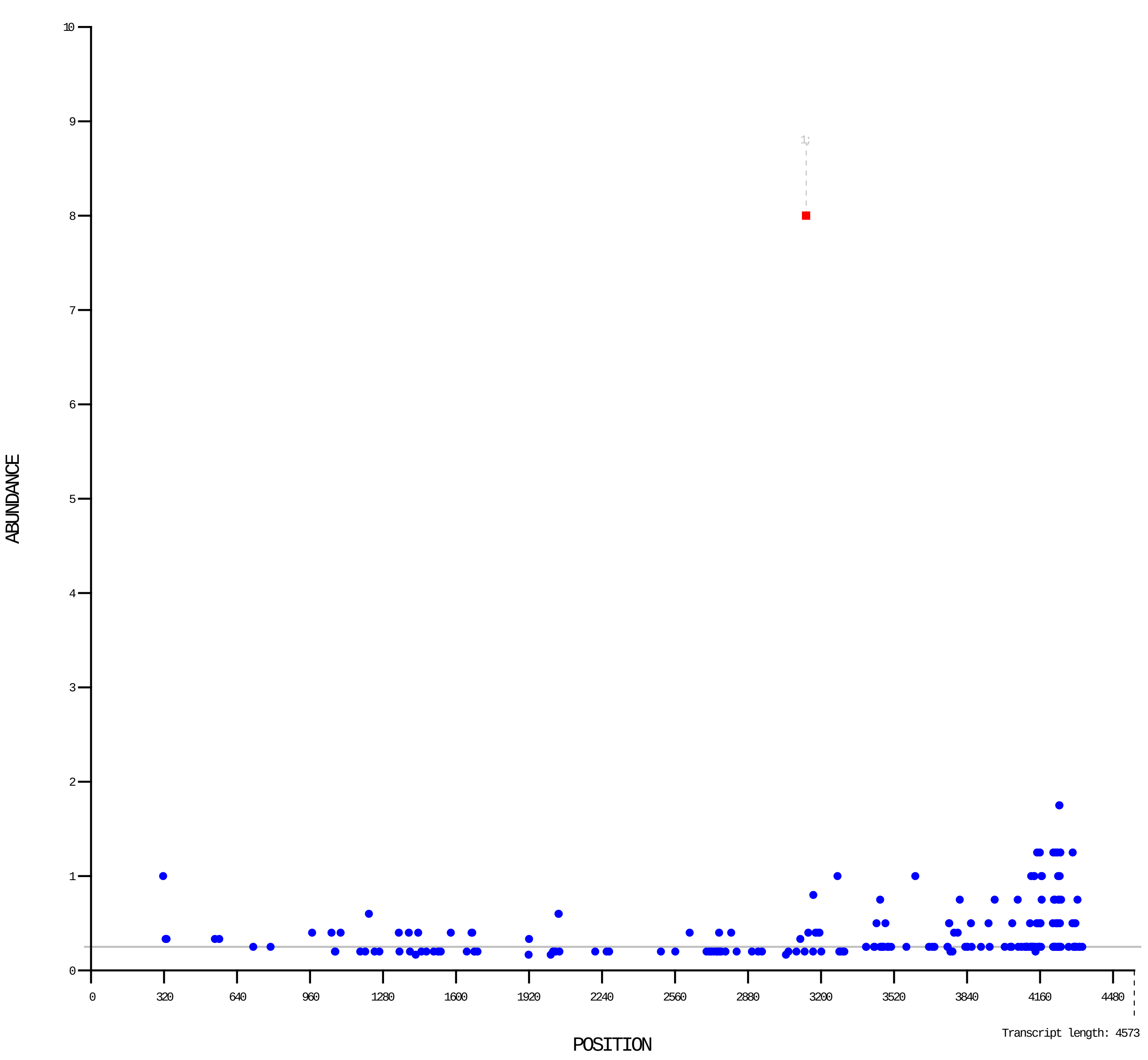

orange1.1t01918.1 gene=orange1.1t01918 CDS=1-2835

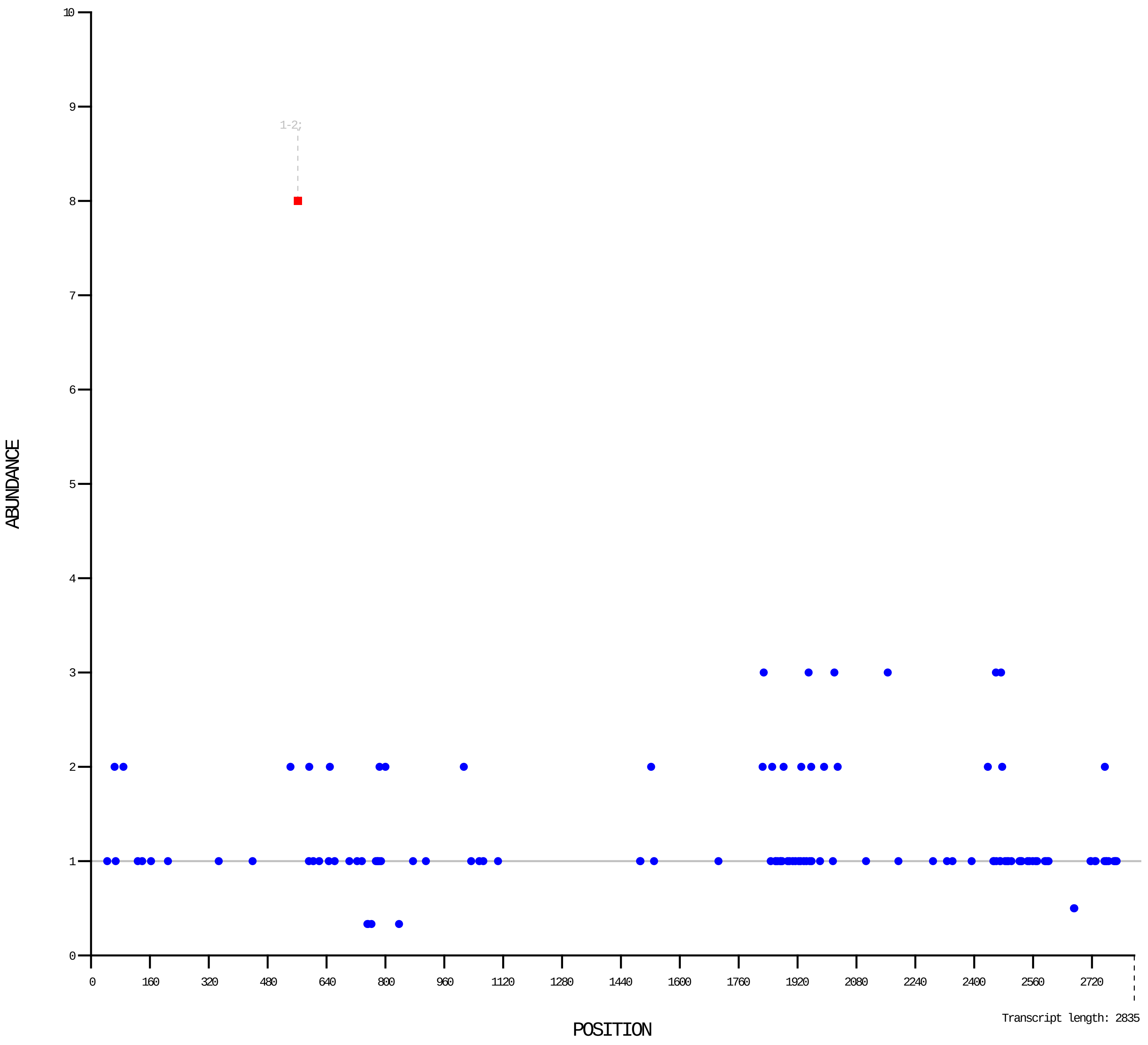


```
Category: ■ 0  ■ 1  ■ 2  ■ 3  ■ 4
Degradome alignment: ●    Median: —

■ 0  #1  Position:562  Abundance: 8.00(deg) 1(sRNA)
     5'     TCTTCCCTATGCCTCCCATTCC       3'  ID:
            | ||||||||o|||||||| ||           Score: 2.5
     3' CACCAAAAGGGATATGGAGGGTATGGTTGTTA 5'  p-value: 0.0

■ 0  #2  Position:562  Abundance: 8.00(deg) 1(sRNA)
     5'     TCTTACCTATGCCACCCATTCC       3'  ID:
            | || |||||o|| ||||| ||           Score: 4.5
     3' CACCAAAAGGGATATGGAGGGTATGGTTGTTA 5'  p-value: 0.04
```

Cs6g03520.2 gene=Cs6g03520 CDS=223-3402

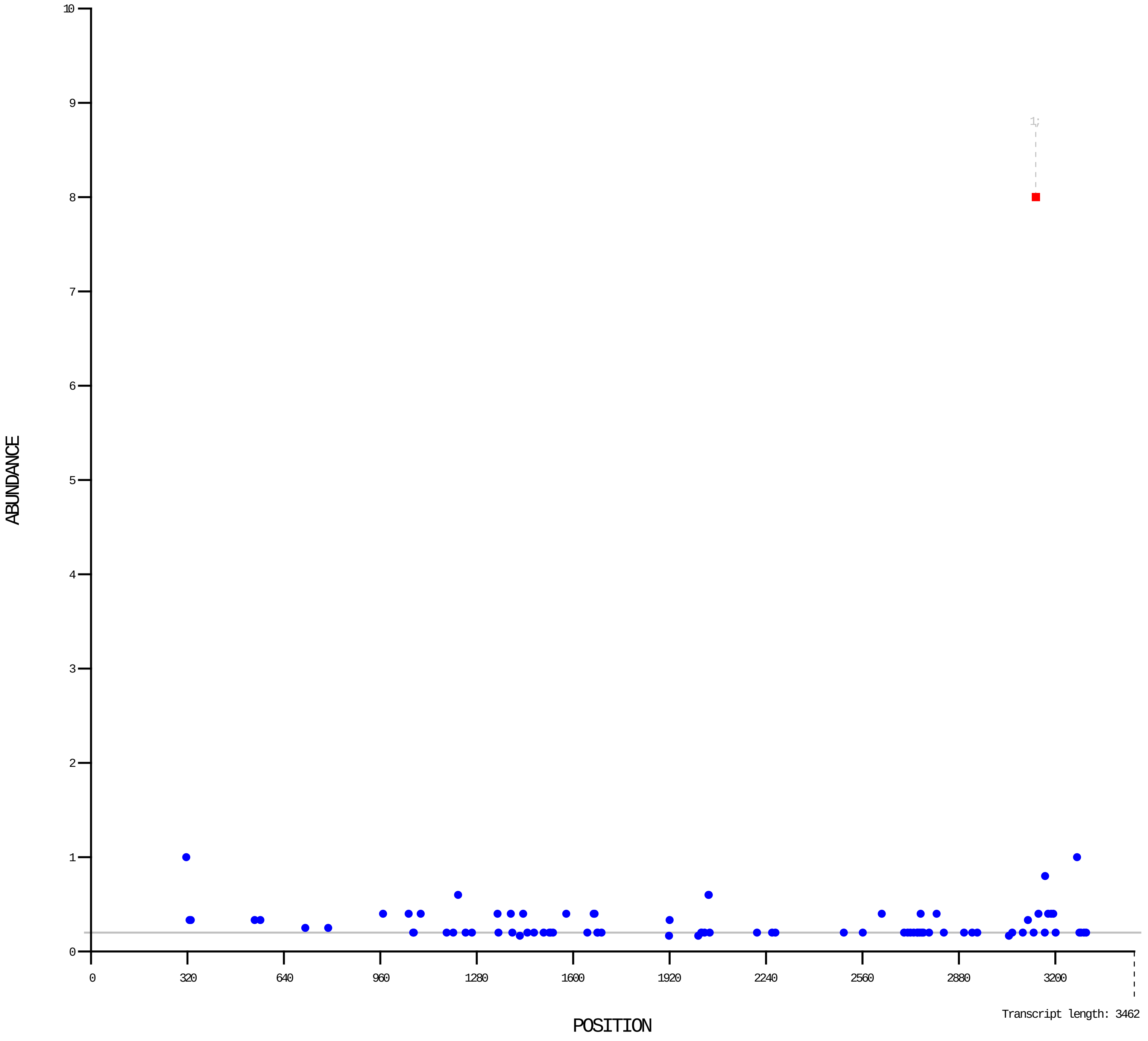

Category: 0 1 2 3 4
Degradome alignment: Median:

0 #1 Position:3135 Abundance: 8.00(deg) 1(sRNA)
```
5'     TCATTTTTGCGTGCAATGATCC      3'  ID:
        || ||||||o|||||||||||          Score: 2.5
3' CCATTGT-AAAACGTACGTTACTAGGTTTTCG 5'  p-value: 0.0
```

Cs6g03520.5 gene=Cs6g03520 CDS=166-4197

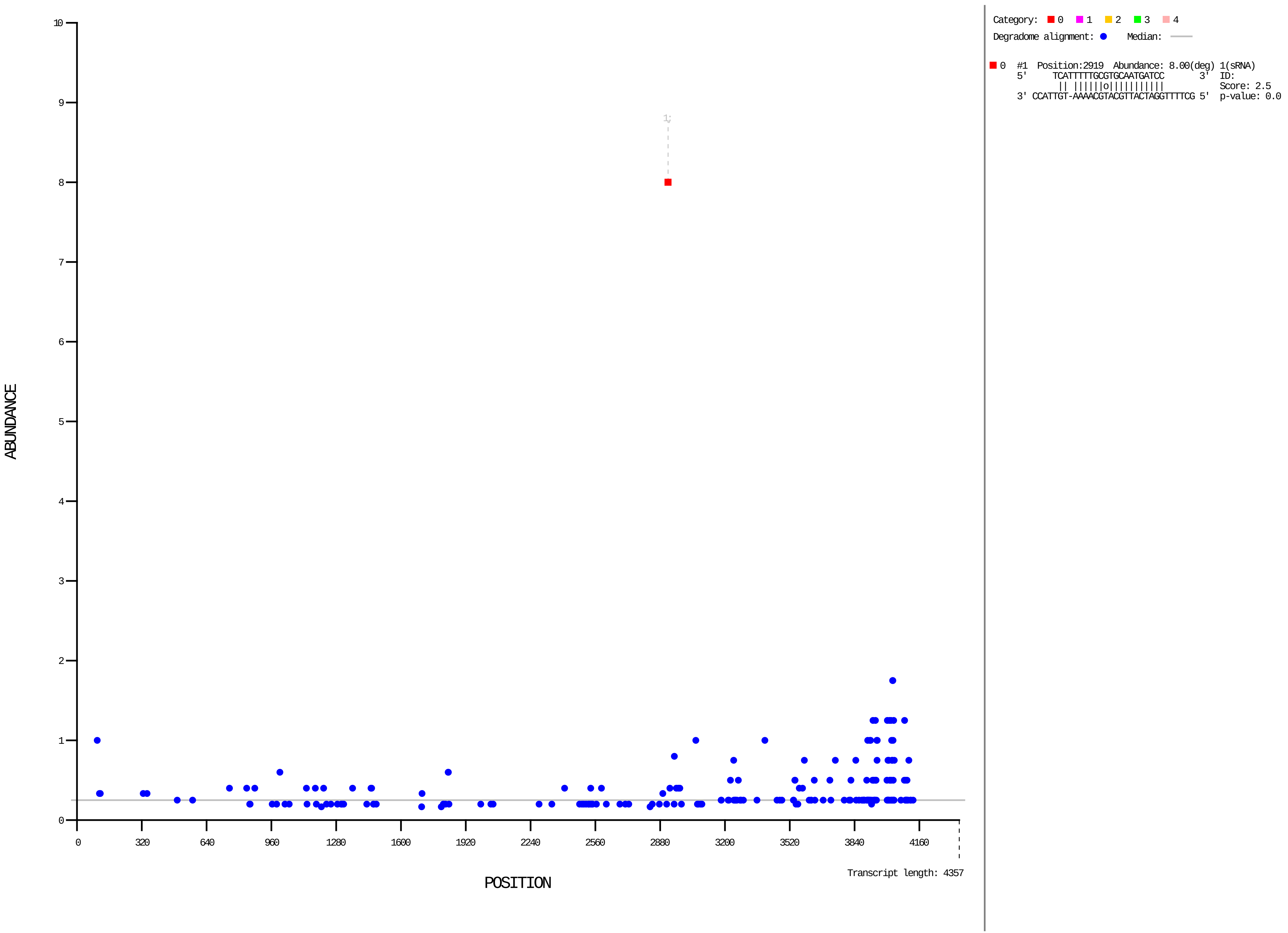

Cs5g01380.1 gene=Cs5g01380 CDS=451-1941

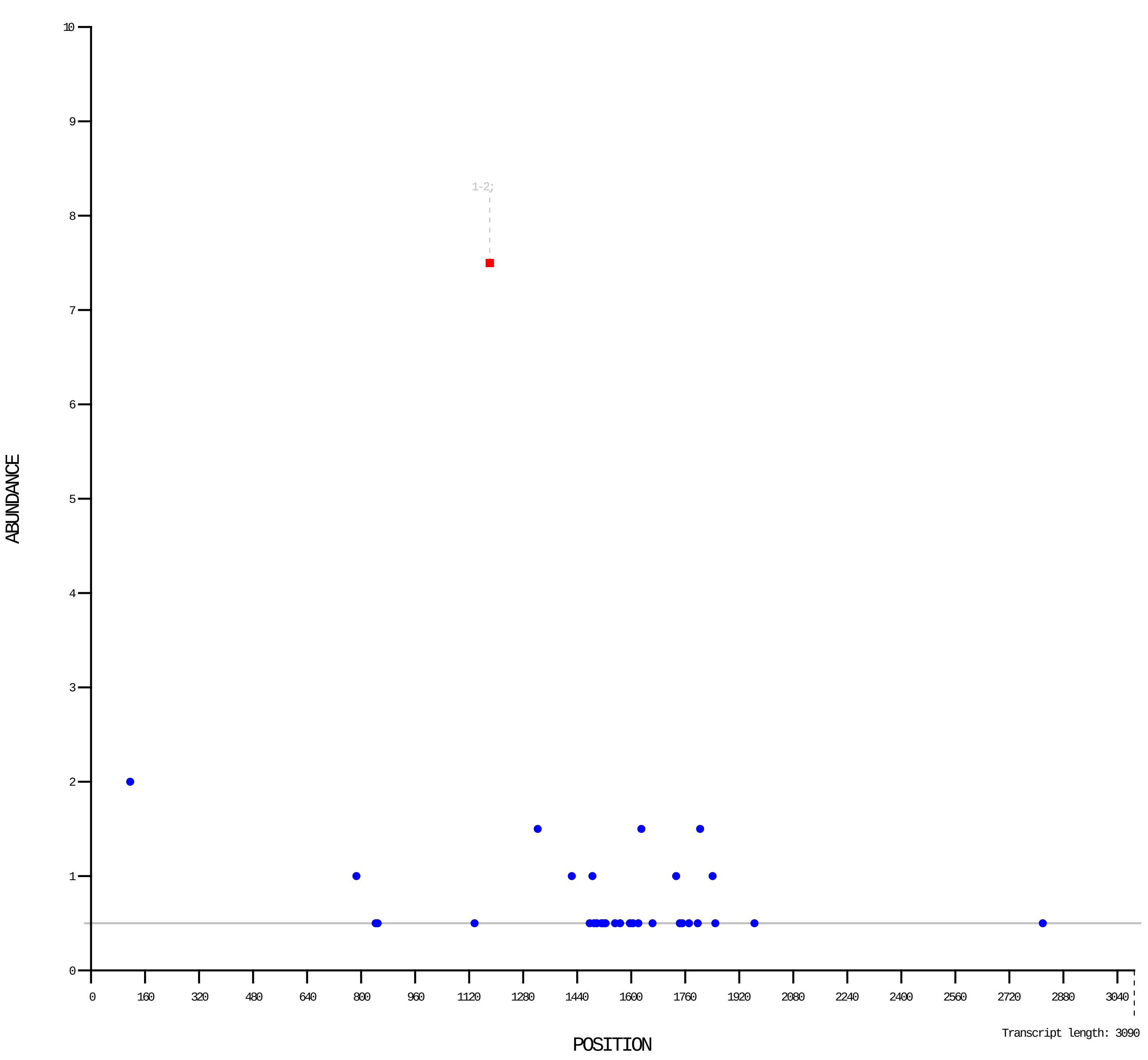

Category: 0 1 2 3 4
Degradome alignment: Median:

0 #1 Position:1181 Abundance: 7.50(deg) 1(sRNA)
```
5'    TTCCAC-GGCTTTCTTGAACGT       3'  ID:
      |||||| |||||||||||||||           Score: 2.0
3' ATTAAGGTGTCCGAAAGAACTTGCCCCTGCCG 5'  p-value: 0.0
```

0 #2 Position:1181 Abundance: 7.50(deg) 1(sRNA)
```
5'    TTCCACA-GCTTTCTTGAACTG       3'  ID:
      ||||||| |||||||||||| |           Score: 2.0
3' ATTAAGGTGTCCGAAAGAACTTGCCCCTGCCG 5'  p-value: 0.0
```

Cs5g01380.2 gene=Cs5g01380 CDS=95-1213

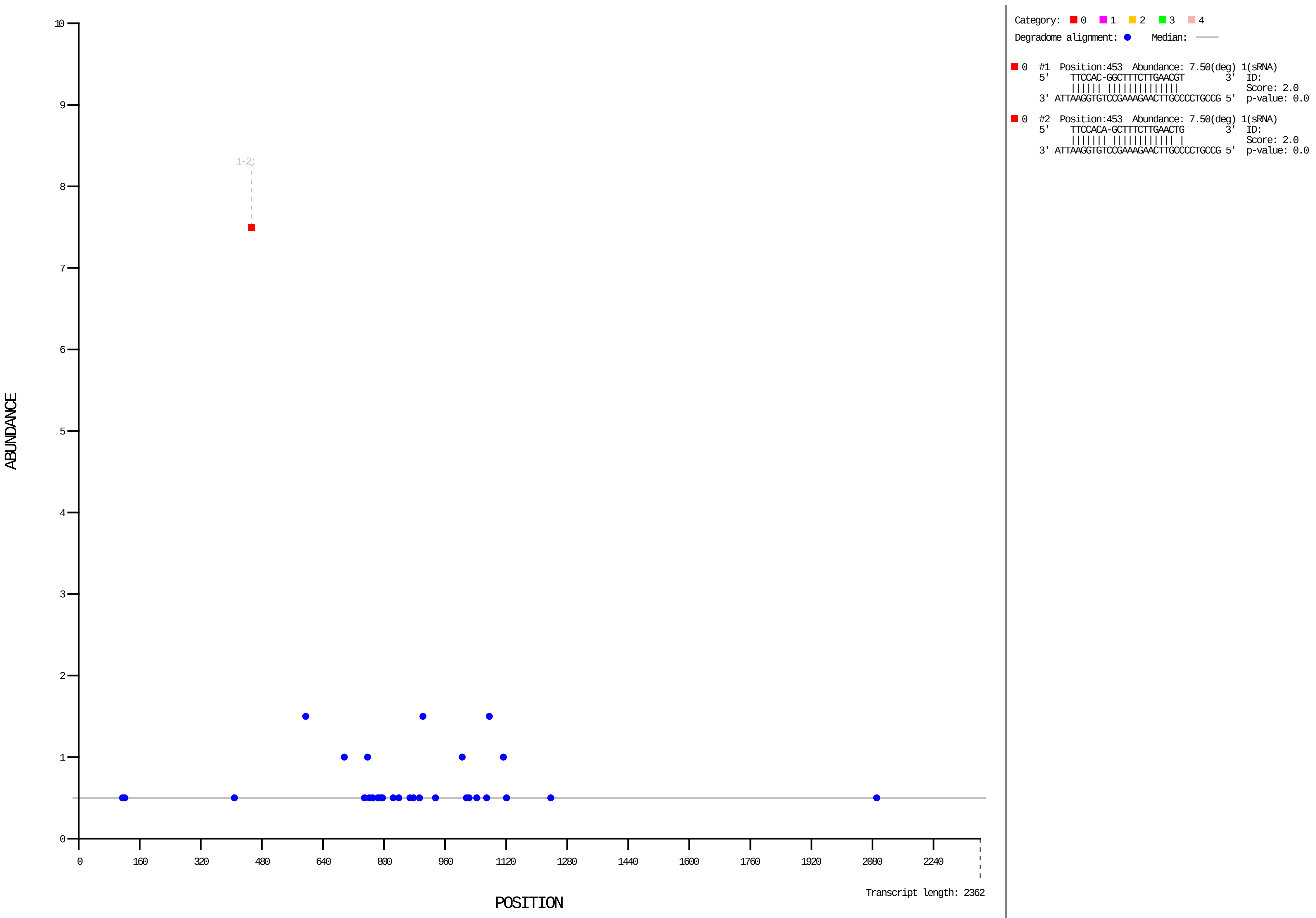

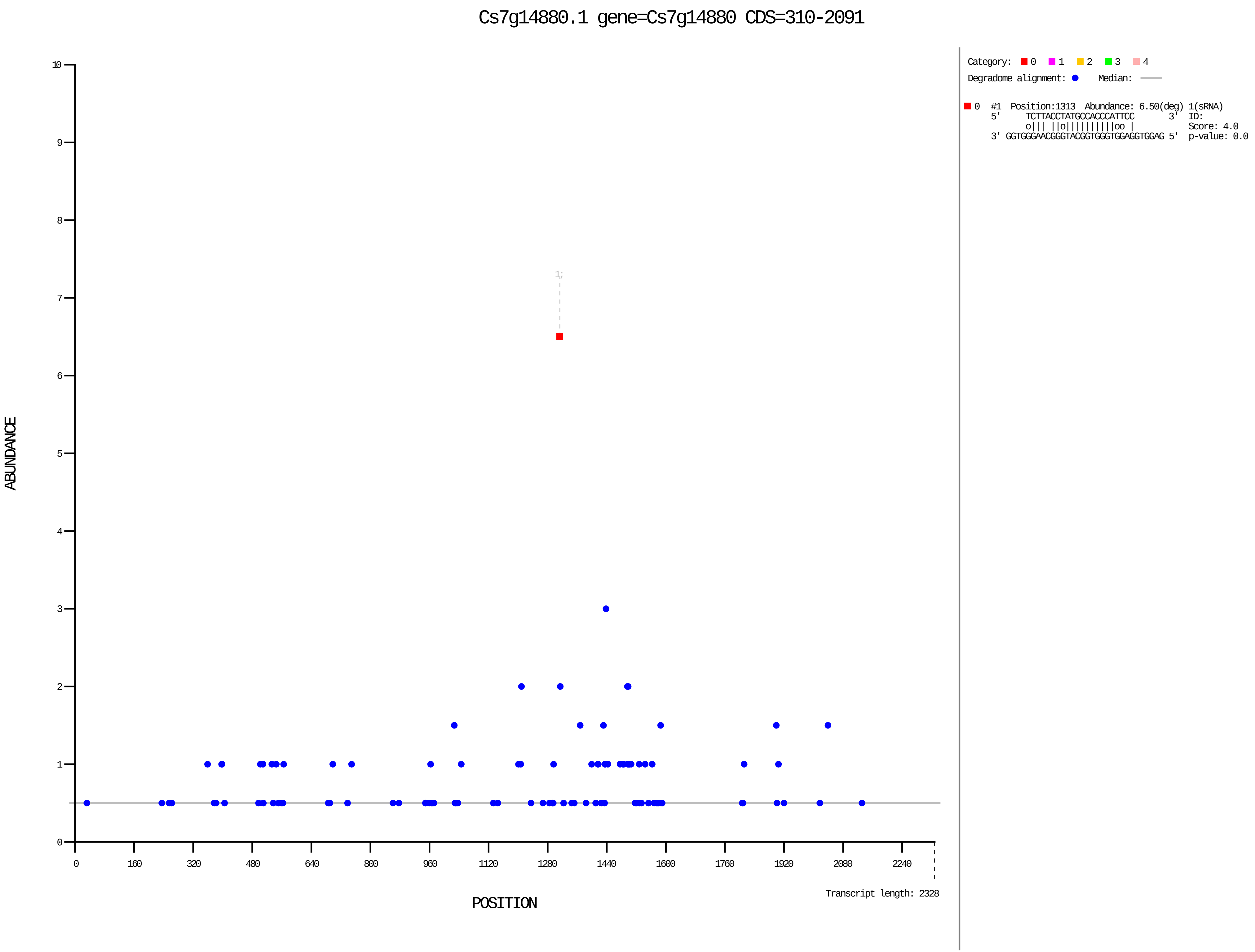

Cs7g14880.1 gene=Cs7g14880 CDS=310-2091
Category: 0 1 2 3 4
Degradome alignment:
Median:
0 #1 Position:1313 Abundance: 6.50(deg) 1(sRNA)
5' TCTTACCTATGCCACCCATTCC 3' ID:
o||| ||o||||||||||oo |
Score: 4.0
3' GGTGGGAACGGGTACGGTGGGTGGAGGTGGAG 5' p-value: 0.0
ABUNDANCE
POSITION
Transcript length: 2328

Cs7g14880.2 gene=Cs7g14880 CDS=310-2064

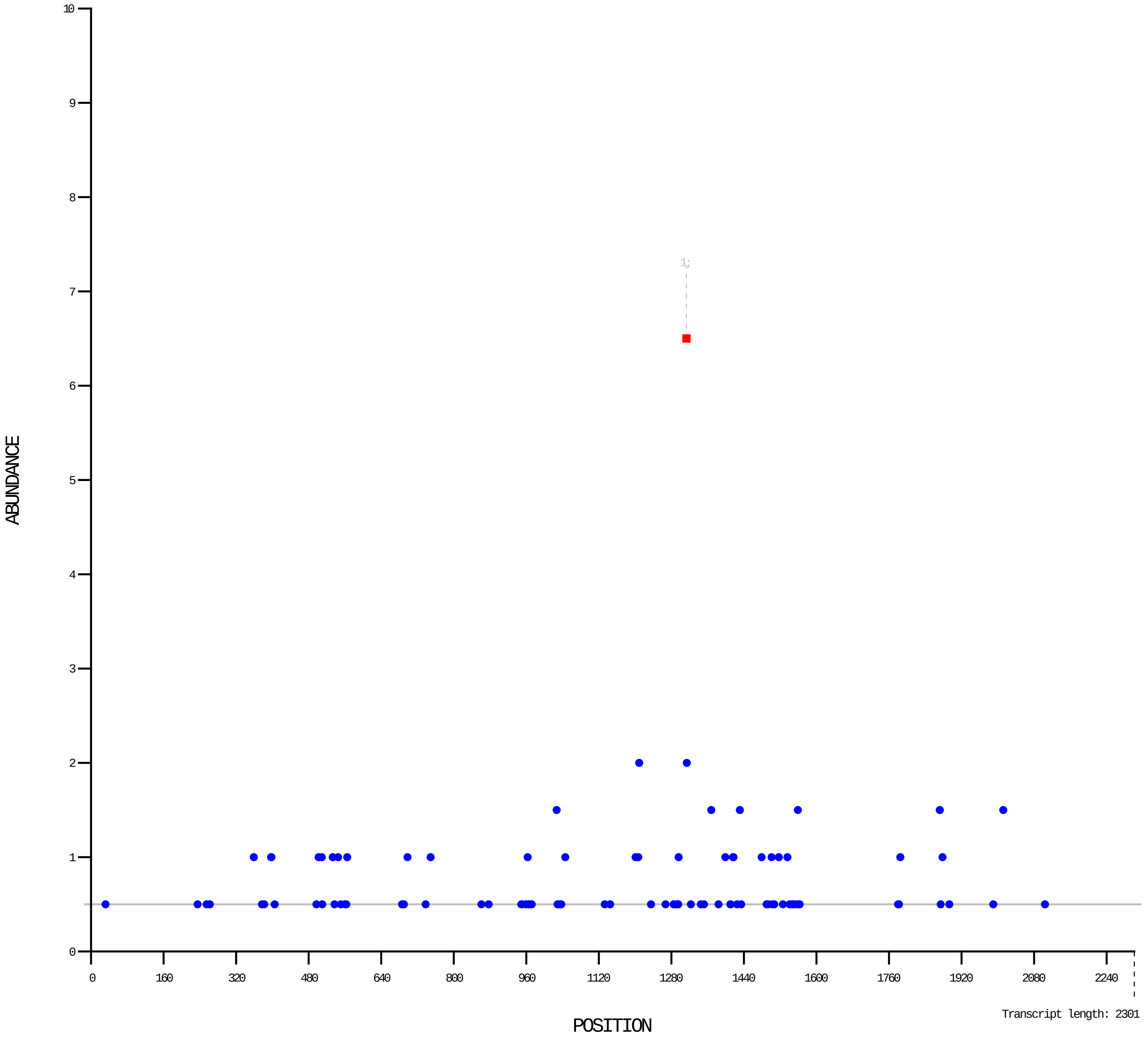

Category: 0 1 2 3 4

Degradome alignment: Median:

0 #1 Position:1313 Abundance: 6.50(deg) 1(sRNA)

```
5'     TCTTACCTATGCCACCCATTCC       3'  ID:
       o||| ||o||||||||||oo |           Score: 4.0
3' GGTGGGAACGGGTACGGTGGGTGGAGGTGGAG 5'  p-value: 0.0
```

Cs6g03500.1 gene=Cs6g03500 CDS=289-4494

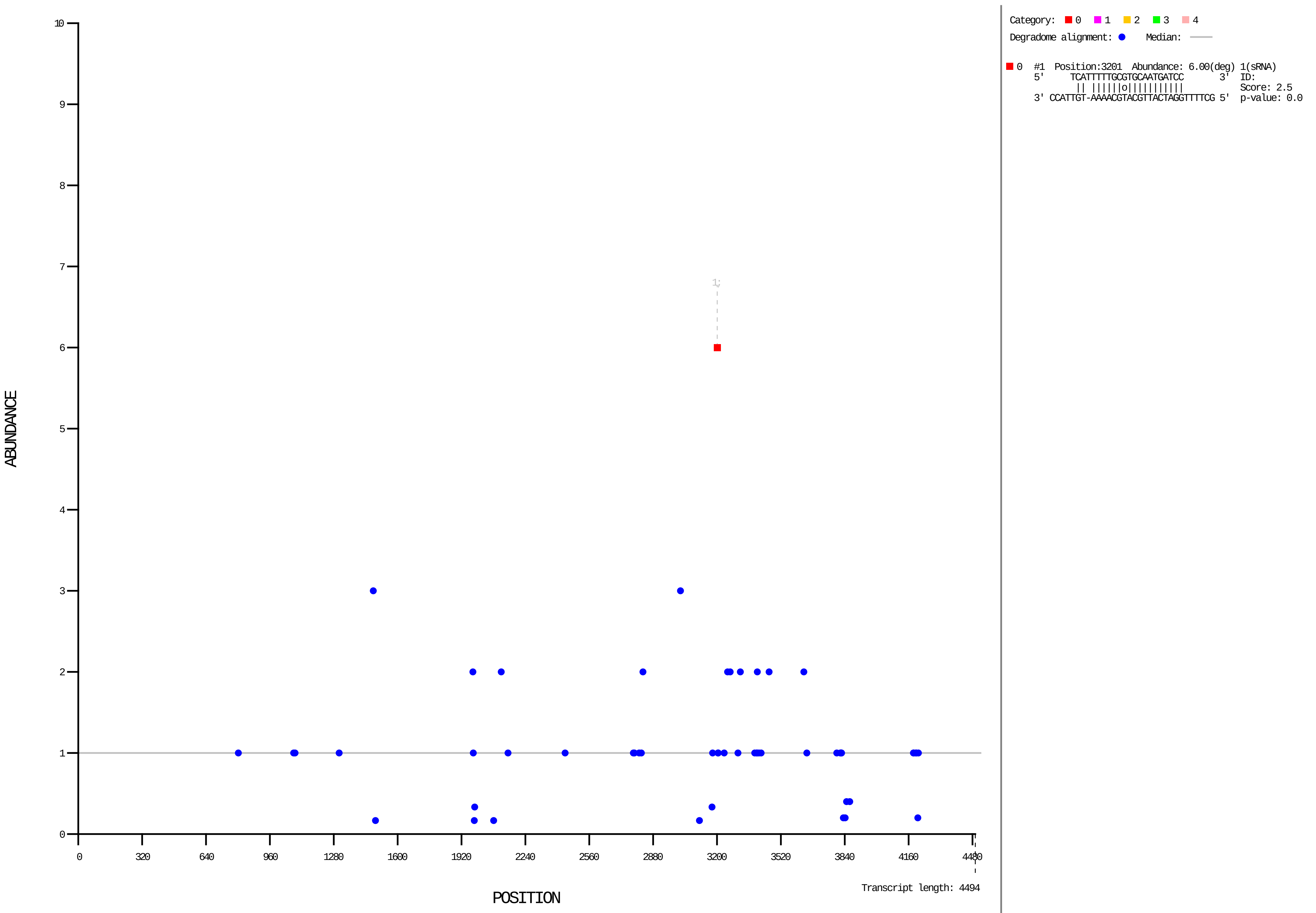

Cs6g06890.1 gene=Cs6g06890 CDS=85-1830

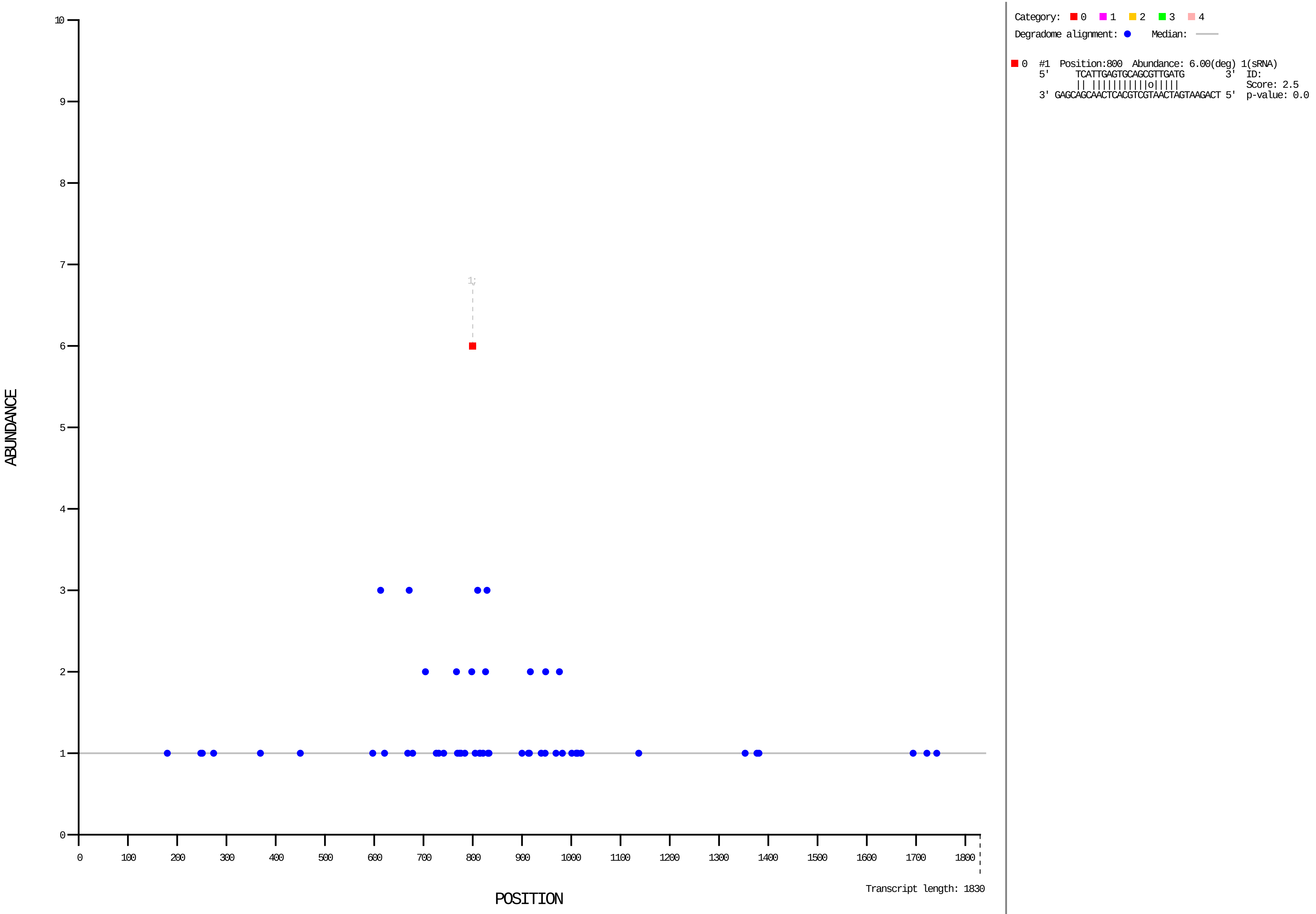

orange1.1t05262.1 gene=orange1.1t05262 CDS=292-1914

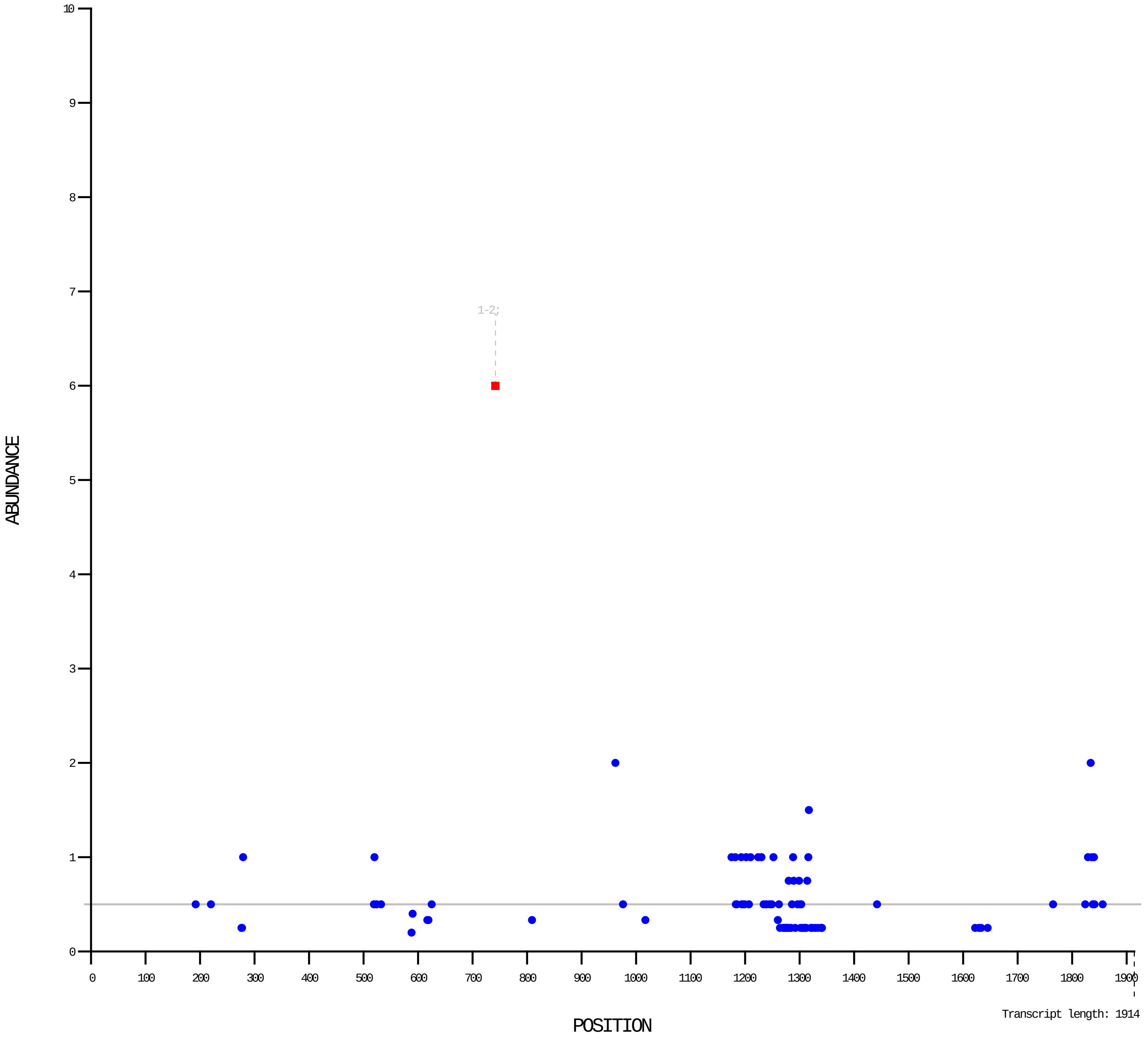

Category: 0 1 2 3 4
Degradome alignment: Median:

```
0  #1  Position:742  Abundance: 6.00(deg) 1(sRNA)
   5'     TCTTCCCTATGCCTCCCATTCC       3'  ID:
          | ||||||||||||||||| ||           Score: 2.0
   3' CAACAAAAGGGATACGGAGGGTATGGTTGCTA 5'  p-value: 0.0

0  #2  Position:742  Abundance: 6.00(deg) 1(sRNA)
   5'     TCTTACCTATGCCACCCATTCC       3'  ID:
          | || |||||||| ||||| ||           Score: 4.0
   3' CAACAAAAGGGATACGGAGGGTATGGTTGCTA 5'  p-value: 0.0
```

Cs4g19310.5 gene=Cs4g19310 CDS=275-2770

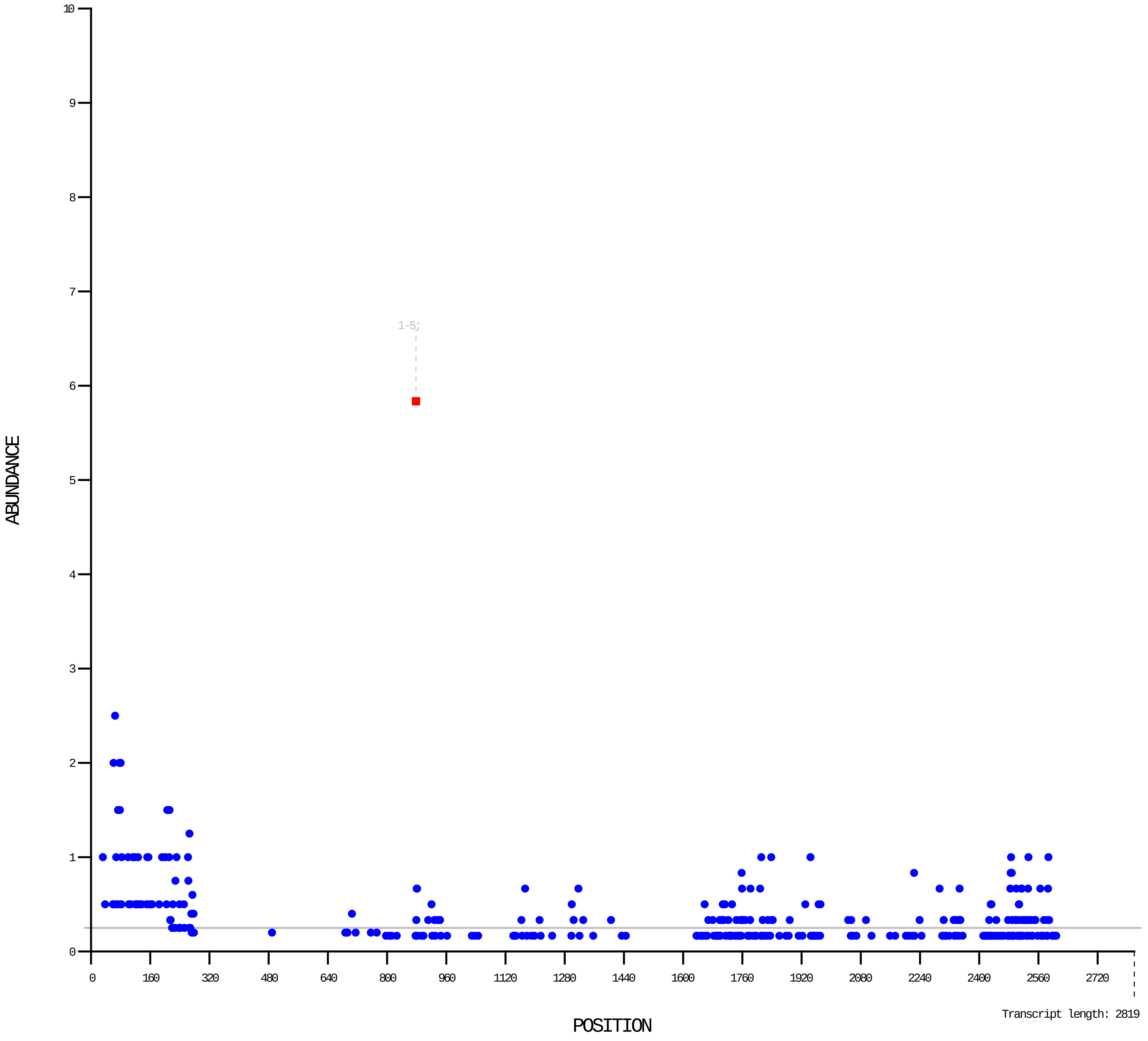

Category: 0 1 2 3 4
Degradome alignment: Median:

```
0  #1  Position:878  Abundance: 5.83(deg) 1(sRNA)
   5'     TCGGACCAGGCTTCATTCCC          3'  ID:
          o|||||||||||||||| |||              Score: 1.5
   3' CTTAGGCCTGGTCCGAAGTA-GGGCCCGTAAA 5'  p-value: 0.0

0  #2  Position:878  Abundance: 5.83(deg) 1(sRNA)
   5'     TCGGACCAGGCTTCATTCCCT         3'  ID:
          o|||||||||||||||| |||              Score: 2.5
   3' CTTAGGCCTGGTCCGAAGTA-GGGCCCGTAAA 5'  p-value: 0.0

0  #3  Position:878  Abundance: 5.83(deg) 1(sRNA)
   5'     TCGGACCAGGCTTCATTCCCGT        3'  ID:
          o|||||||||||||||| ||||             Score: 2.5
   3' CTTAGGCCTGGTCCGAAGTA-GGGCCCGTAAA 5'  p-value: 0.0

0  #4  Position:878  Abundance: 5.83(deg) 1(sRNA)
   5'     TCGGACCAGGCTTCATTCCCC         3'  ID:
          o|||||||||||||||| |||              Score: 2.5
   3' CTTAGGCCTGGTCCGAAGTA-GGGCCCGTAAA 5'  p-value: 0.0

0  #5  Position:878  Abundance: 5.83(deg) 1(sRNA)
   5'     TCGGACCAGGCTTCATTCCCCC        3'  ID:
          o|||||||||||||||| |||              Score: 3.5
   3' CTTAGGCCTGGTCCGAAGTA-GGGCCCGTAAA 5'  p-value: 0.0
```

Cs4g19310.1 gene=Cs4g19310 CDS=74-2626

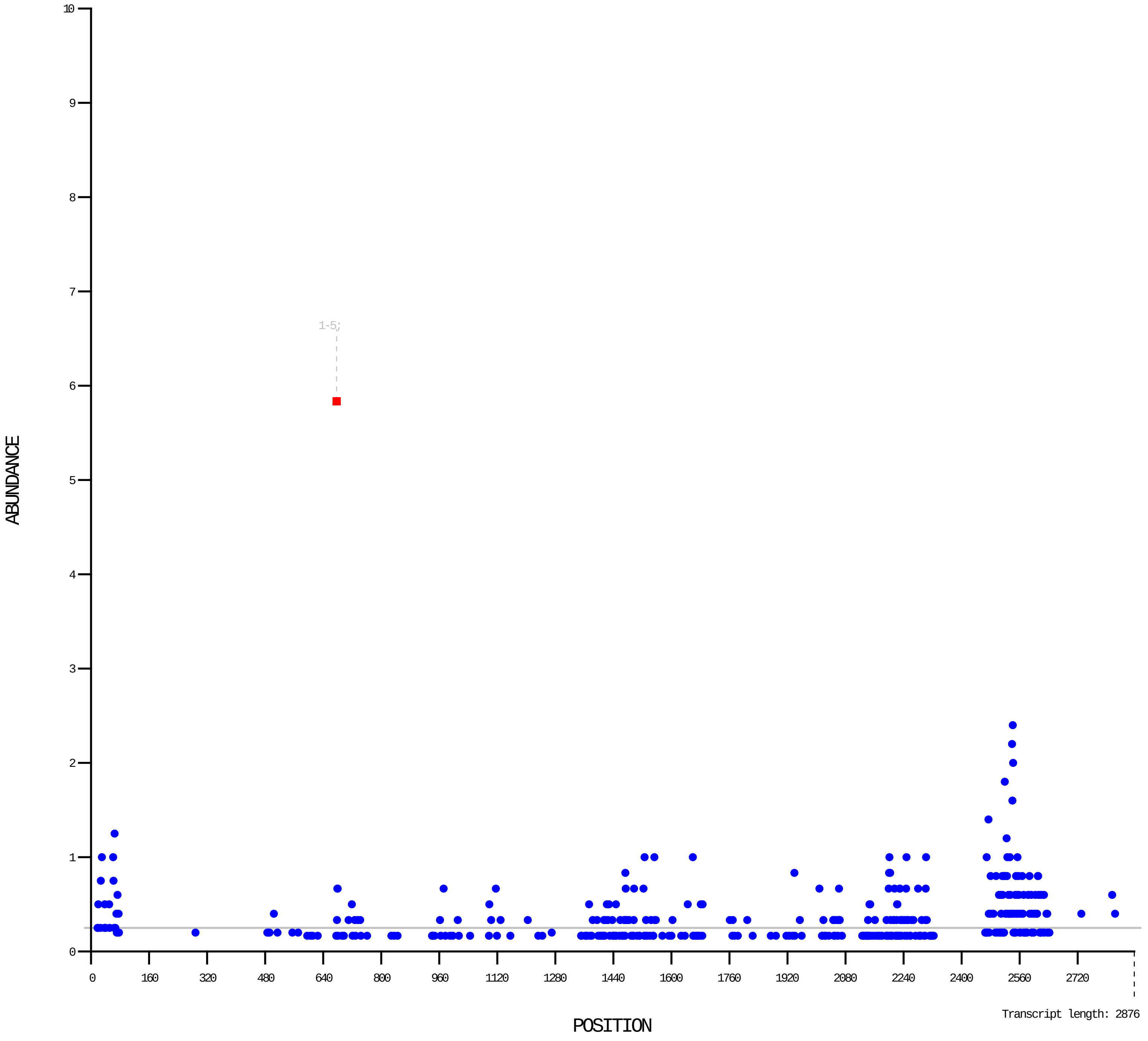

Category: 0 1 2 3 4
Degradome alignment: Median:

0 #1 Position:677 Abundance: 5.83(deg) 1(sRNA)
5' TCGGACCAGGCTTCATTCCC 3' ID:
o||||||||||||||| ||| Score: 1.5
3' CTTAGGCCTGGTCCGAAGTA-GGGCCCGTAAA 5' p-value: 0.0

0 #2 Position:677 Abundance: 5.83(deg) 1(sRNA)
5' TCGGACCAGGCTTCATTCCCT 3' ID:
o||||||||||||||| ||| Score: 2.5
3' CTTAGGCCTGGTCCGAAGTA-GGGCCCGTAAA 5' p-value: 0.0

0 #3 Position:677 Abundance: 5.83(deg) 1(sRNA)
5' TCGGACCAGGCTTCATTCCCGT 3' ID:
o||||||||||||||| |||| Score: 2.5
3' CTTAGGCCTGGTCCGAAGTA-GGGCCCGTAAA 5' p-value: 0.0

0 #4 Position:677 Abundance: 5.83(deg) 1(sRNA)
5' TCGGACCAGGCTTCATTCCCC 3' ID:
o||||||||||||||| ||| Score: 2.5
3' CTTAGGCCTGGTCCGAAGTA-GGGCCCGTAAA 5' p-value: 0.0

0 #5 Position:677 Abundance: 5.83(deg) 1(sRNA)
5' TCGGACCAGGCTTCATTCCCCC 3' ID:
o||||||||||||||| ||| Score: 3.5
3' CTTAGGCCTGGTCCGAAGTA-GGGCCCGTAAA 5' p-value: 0.0

Cs4g19310.6 gene=Cs4g19310 CDS=142-2106

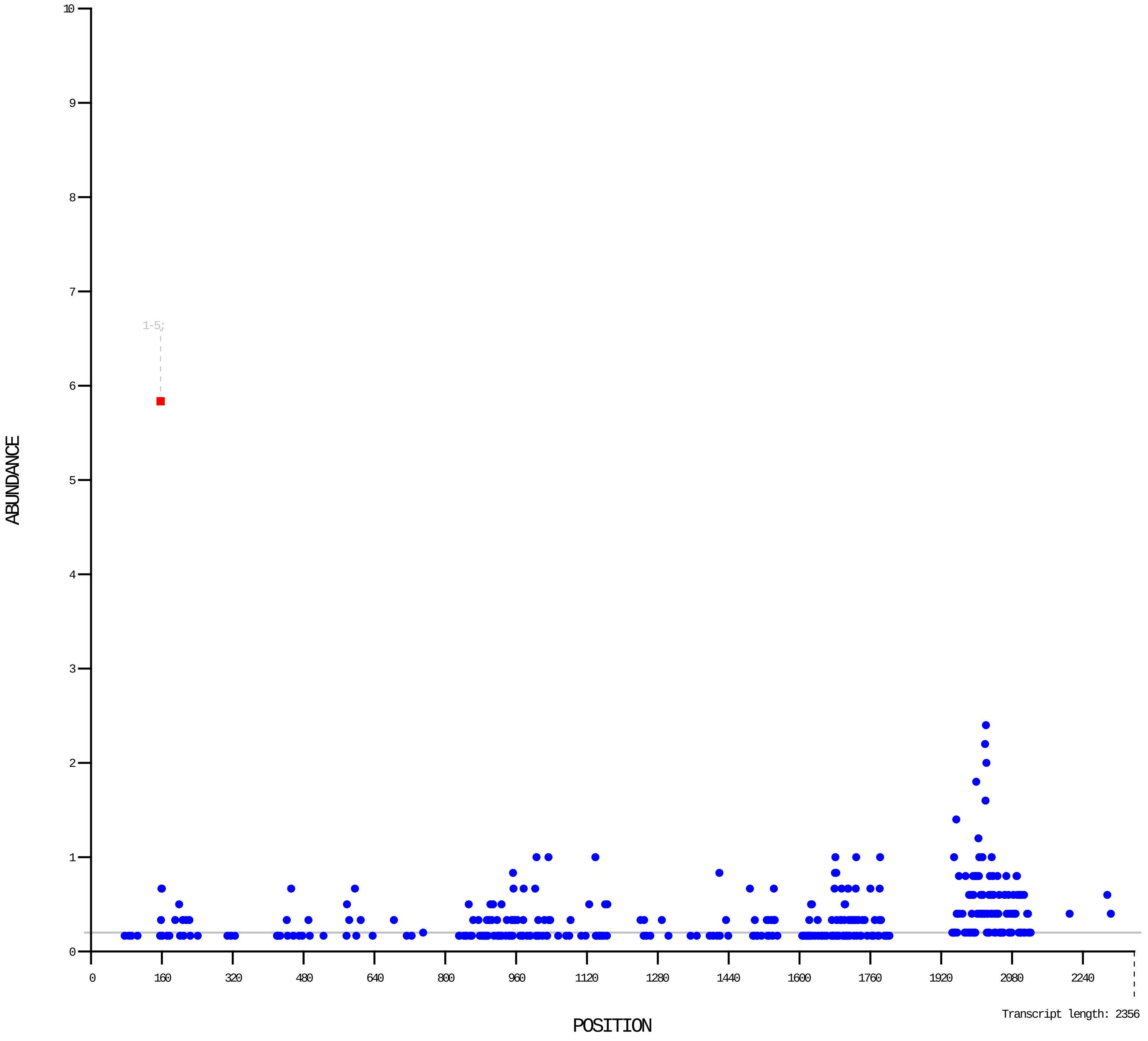


```
Category: ■ 0 ■ 1 ■ 2 ■ 3 ■ 4
Degradome alignment: ●    Median: —

■ 0  #1  Position:157  Abundance: 5.83(deg) 1(sRNA)
     5'     TCGGACCAGGCTTCATTCCC         3'  ID:
            o|||||||||||||||  |||            Score: 1.5
     3' CTTAGGCCTGGTCCGAAGTA-GGGCCCGTAAA 5'  p-value: 0.0

■ 0  #2  Position:157  Abundance: 5.83(deg) 1(sRNA)
     5'     TCGGACCAGGCTTCATTCCCT        3'  ID:
            o|||||||||||||||  |||            Score: 2.5
     3' CTTAGGCCTGGTCCGAAGTA-GGGCCCGTAAA 5'  p-value: 0.0

■ 0  #3  Position:157  Abundance: 5.83(deg) 1(sRNA)
     5'     TCGGACCAGGCTTCATTCCCGT       3'  ID:
            o|||||||||||||||  ||||           Score: 2.5
     3' CTTAGGCCTGGTCCGAAGTA-GGGCCCGTAAA 5'  p-value: 0.0

■ 0  #4  Position:157  Abundance: 5.83(deg) 1(sRNA)
     5'     TCGGACCAGGCTTCATTCCCC        3'  ID:
            o|||||||||||||||  |||            Score: 2.5
     3' CTTAGGCCTGGTCCGAAGTA-GGGCCCGTAAA 5'  p-value: 0.0

■ 0  #5  Position:157  Abundance: 5.83(deg) 1(sRNA)
     5'     TCGGACCAGGCTTCATTCCCCC       3'  ID:
            o|||||||||||||||  |||            Score: 3.5
     3' CTTAGGCCTGGTCCGAAGTA-GGGCCCGTAAA 5'  p-value: 0.01
```

Cs4g19310.3 gene=Cs4g19310 CDS=275-2827

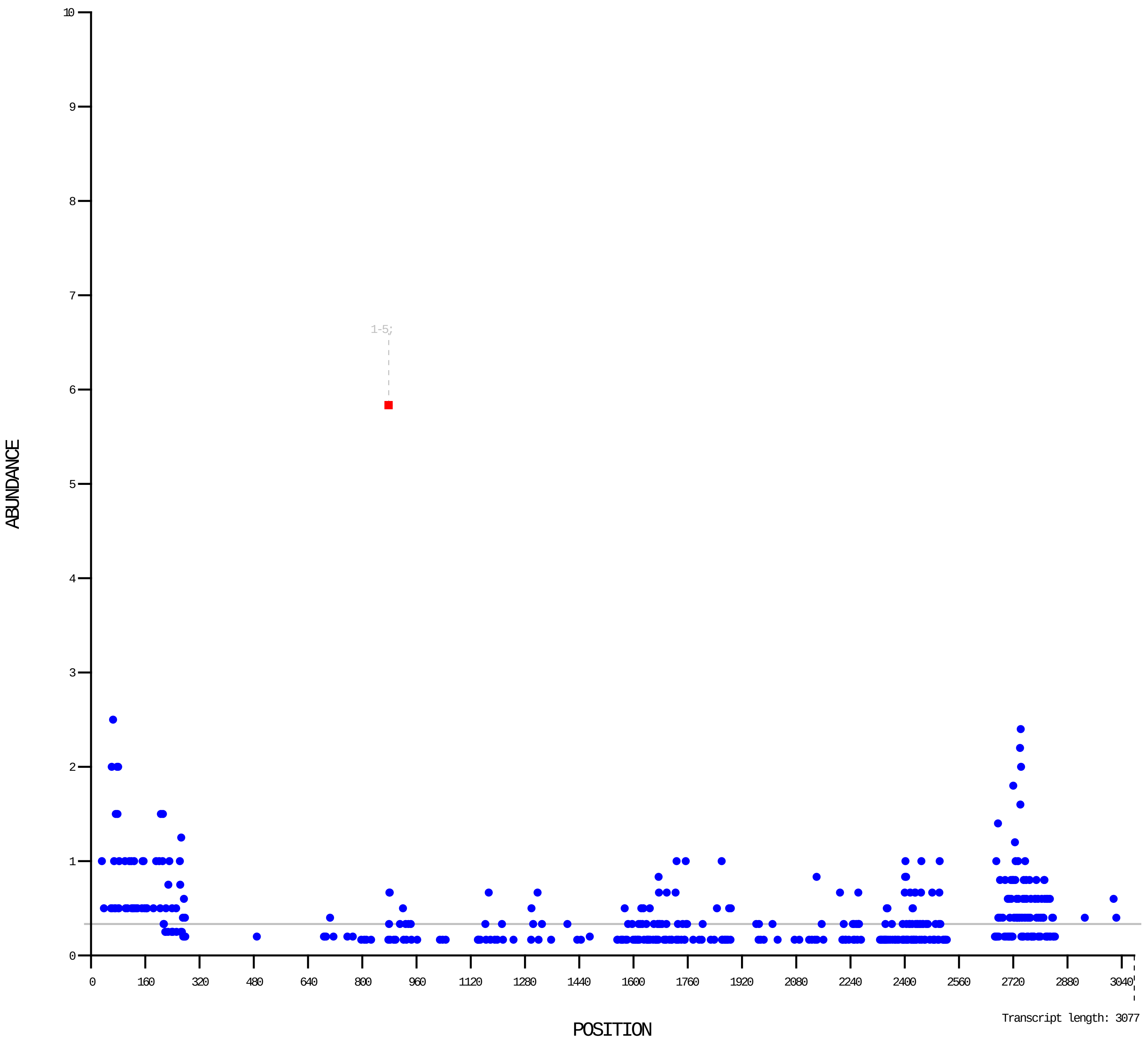

Category: 0 1 2 3 4

Degradome alignment: ● Median:

```
0  #1  Position:878  Abundance: 5.83(deg) 1(sRNA)
   5'     TCGGACCAGGCTTCATTCCC         3'  ID:
          o||||||||||||||| |||             Score: 1.5
   3' CTTAGGCCTGGTCCGAAGTA-GGGCCCGTAAA 5'  p-value: 0.0

0  #2  Position:878  Abundance: 5.83(deg) 1(sRNA)
   5'     TCGGACCAGGCTTCATTCCCT        3'  ID:
          o||||||||||||||| |||             Score: 2.5
   3' CTTAGGCCTGGTCCGAAGTA-GGGCCCGTAAA 5'  p-value: 0.0

0  #3  Position:878  Abundance: 5.83(deg) 1(sRNA)
   5'     TCGGACCAGGCTTCATTCCCGT       3'  ID:
          o||||||||||||||| ||||            Score: 2.5
   3' CTTAGGCCTGGTCCGAAGTA-GGGCCCGTAAA 5'  p-value: 0.0

0  #4  Position:878  Abundance: 5.83(deg) 1(sRNA)
   5'     TCGGACCAGGCTTCATTCCC         3'  ID:
          o||||||||||||||| |||             Score: 2.5
   3' CTTAGGCCTGGTCCGAAGTA-GGGCCCGTAAA 5'  p-value: 0.0

0  #5  Position:878  Abundance: 5.83(deg) 1(sRNA)
   5'     TCGGACCAGGCTTCATTCCCCC       3'  ID:
          o||||||||||||||| |||             Score: 3.5
   3' CTTAGGCCTGGTCCGAAGTA-GGGCCCGTAAA 5'  p-value: 0.0
```

Cs4g19310.2 gene=Cs4g19310 CDS=78-2630

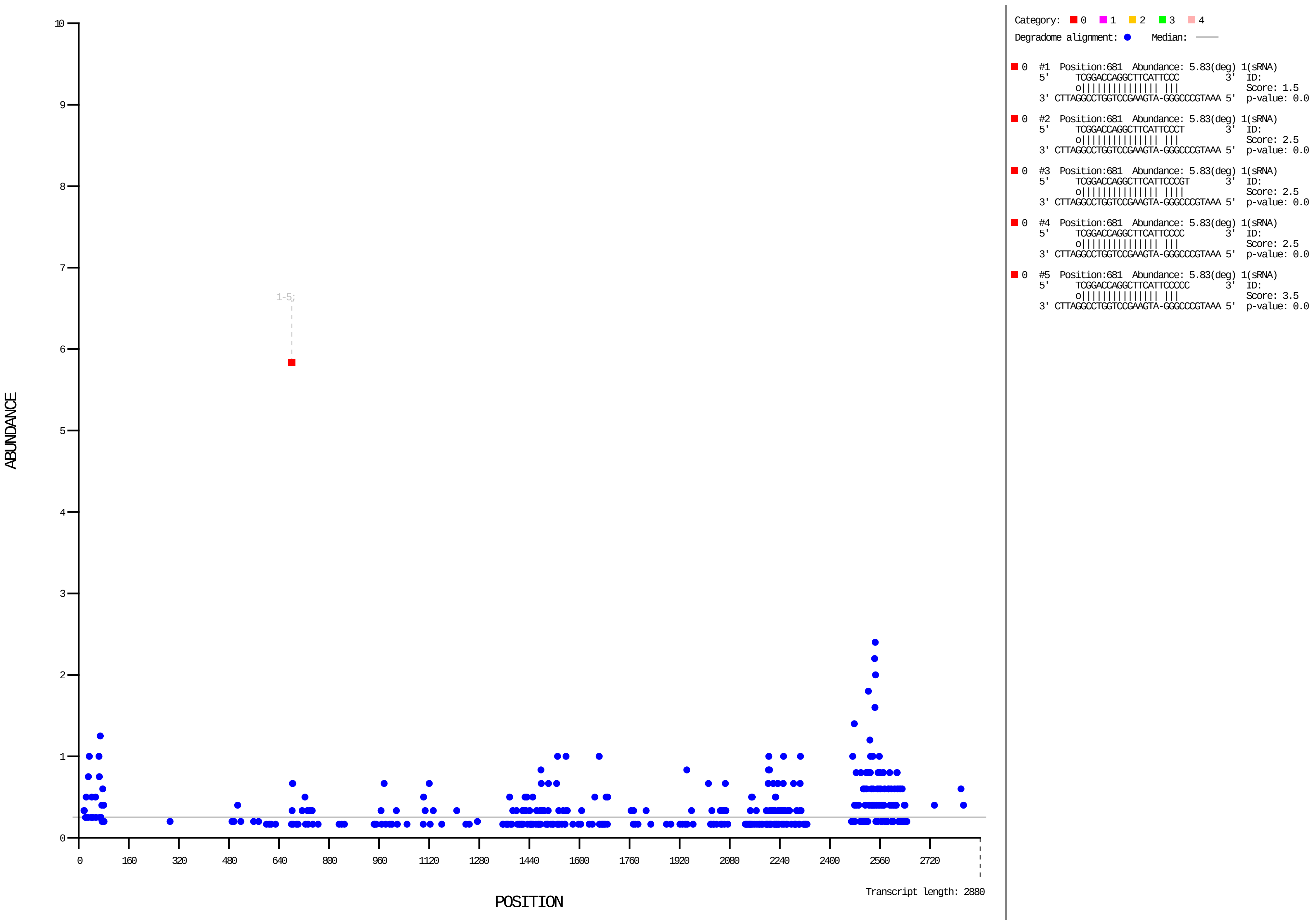

Cs4g19310.4 gene=Cs4g19310 CDS=39-2591

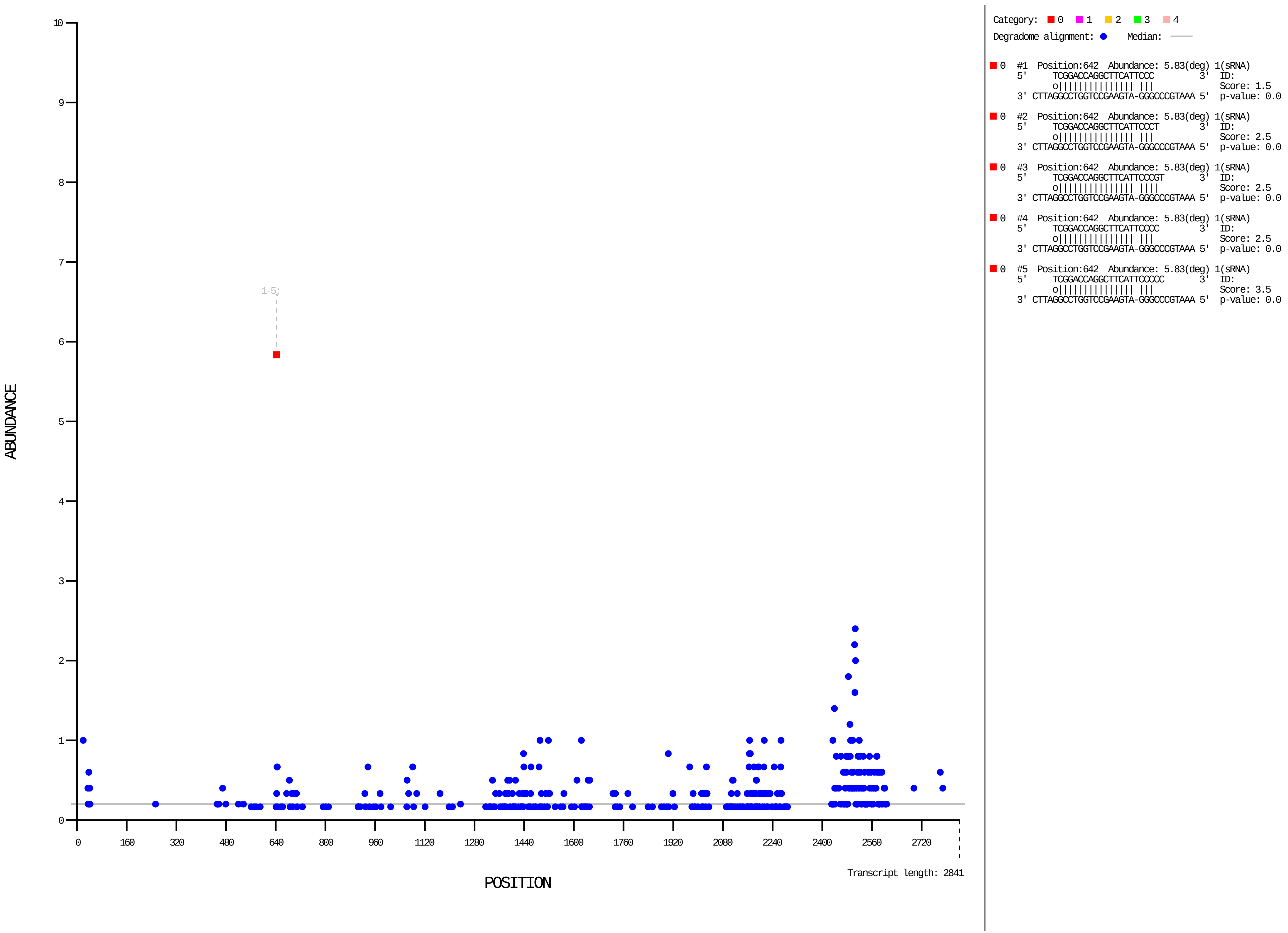

Cs5g28880.4 gene=Cs5g28880 CDS=132-1247

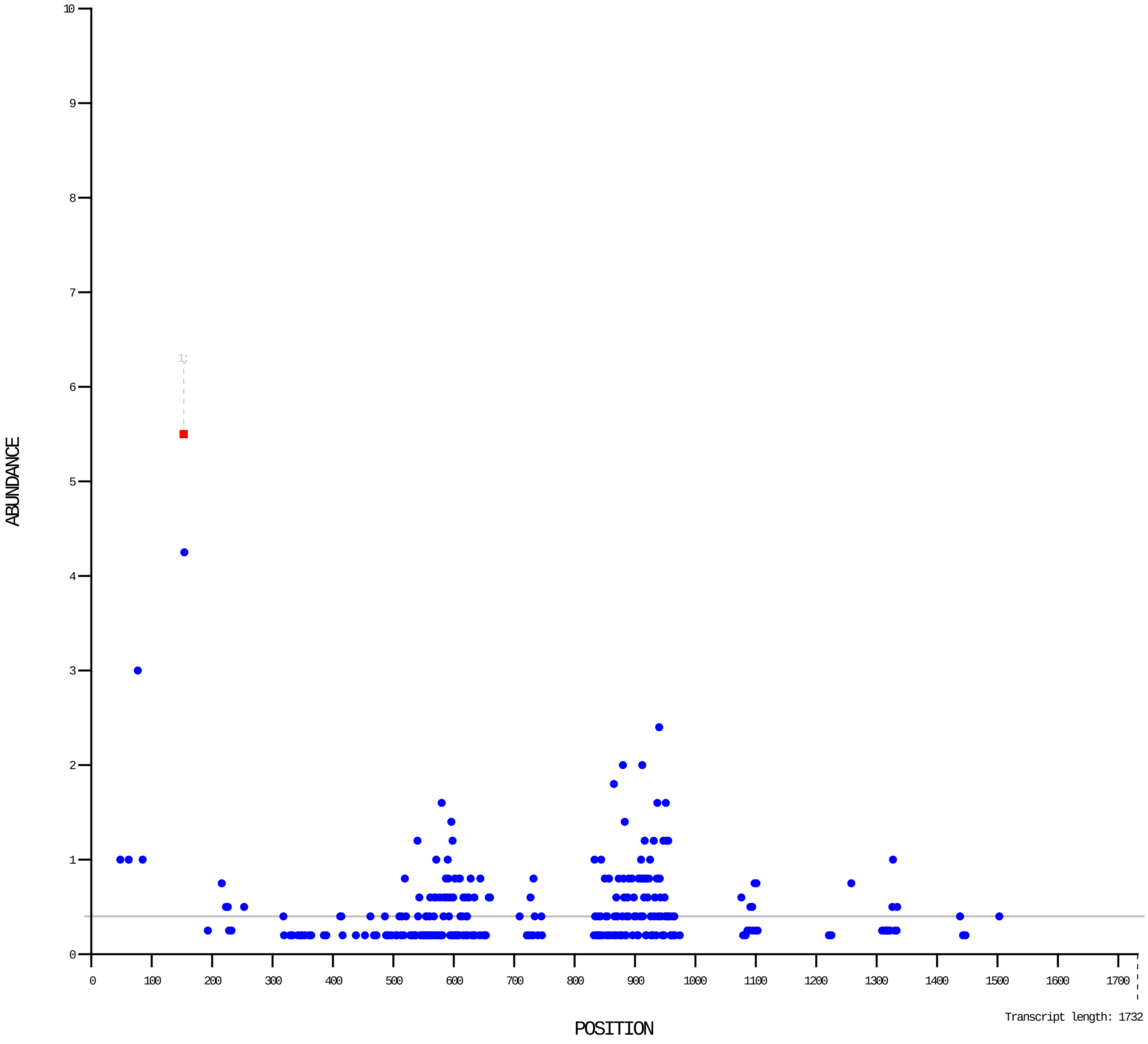

Category: 0 1 2 3 4
Degradome alignment: Median:

0 #1 Position:153 Abundance: 5.50(deg) 1(sRNA)
5' TGTGTTCTCAGGTCACCCCTT 3' ID:
|| ||||o||||o|||| |o Score: 4.5
3' AACAACCCAAGGGTCCGGTGGCGGCGAAATGT 5' p-value: 0.05

Cs5g28880.1 gene=Cs5g28880 CDS=179-1318

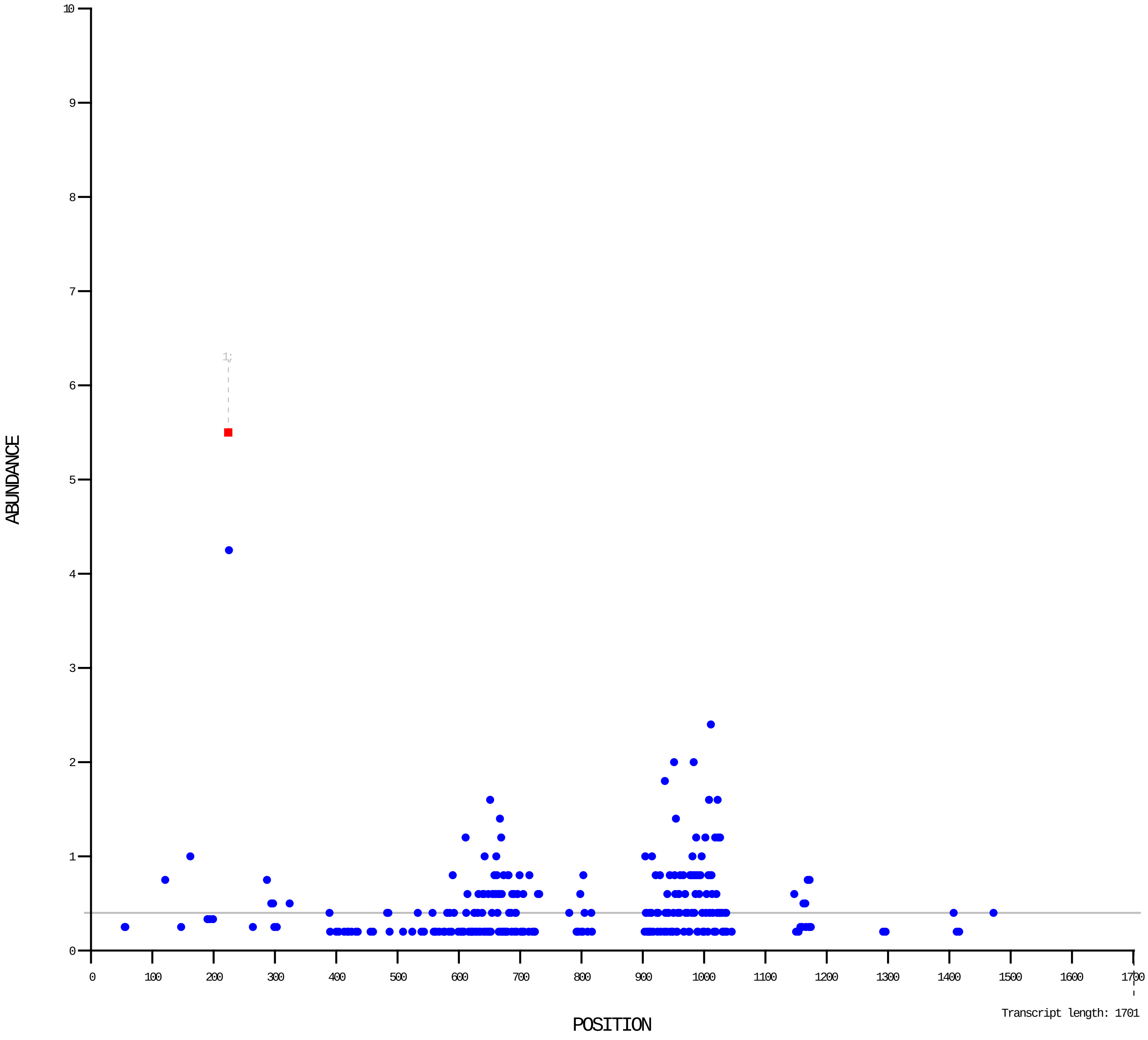

Category: 0 1 2 3 4

Degradome alignment: ● Median:

```
0  #1  Position:224  Abundance: 5.50(deg) 1(sRNA)
   5'     TGTGTTCTCAGGTCACCCCTT          3'  ID:
          || ||||o||||o|||| |o               Score: 4.5
   3' AACAACCCAAGGGTCCGGTGGCGGCGAAACGT 5'  p-value: 0.05
```

Cs5g07330.1 gene=Cs5g07330 CDS=1-630

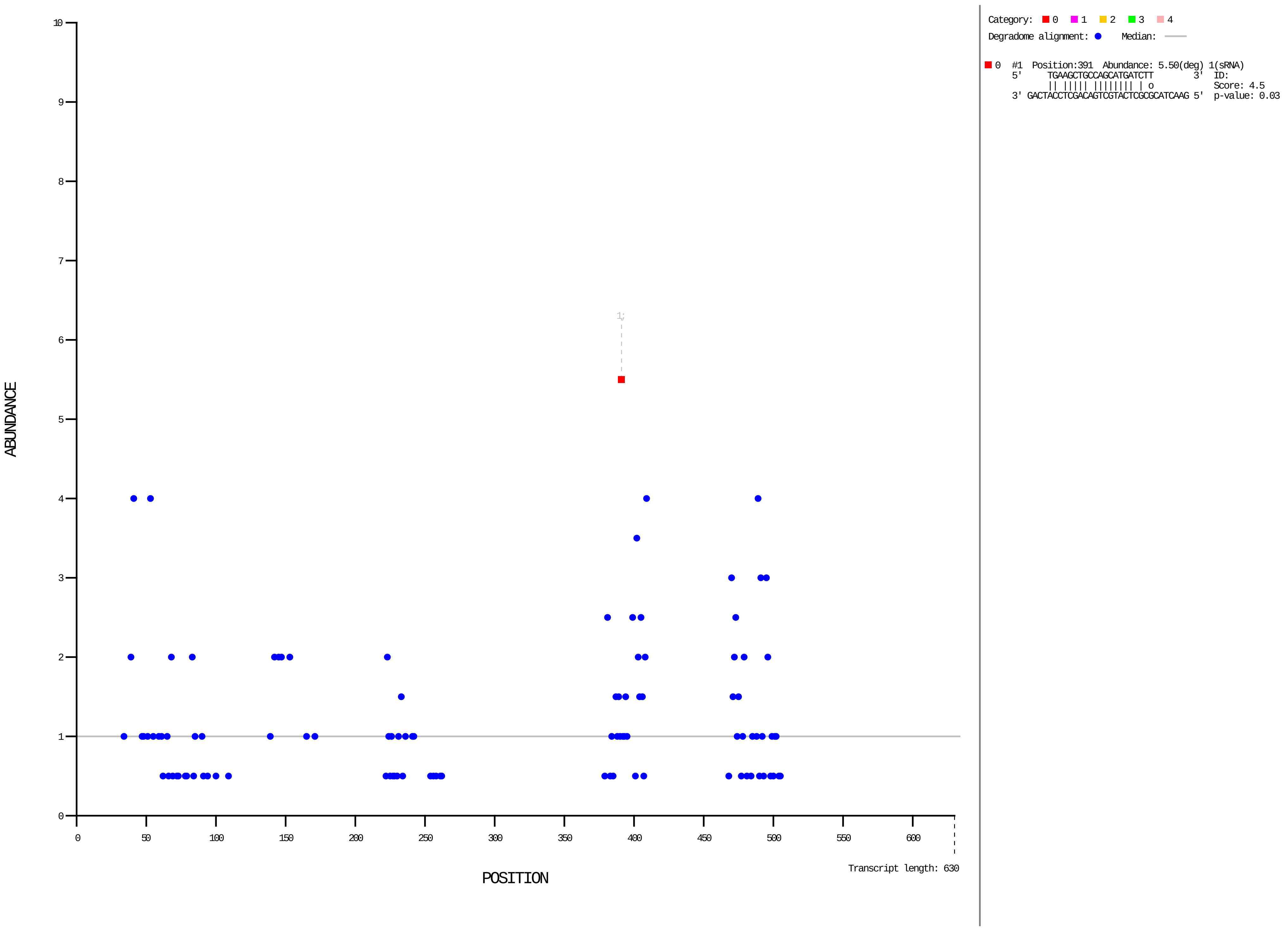

Cs5g28880.5 gene=Cs5g28880 CDS=190-1227

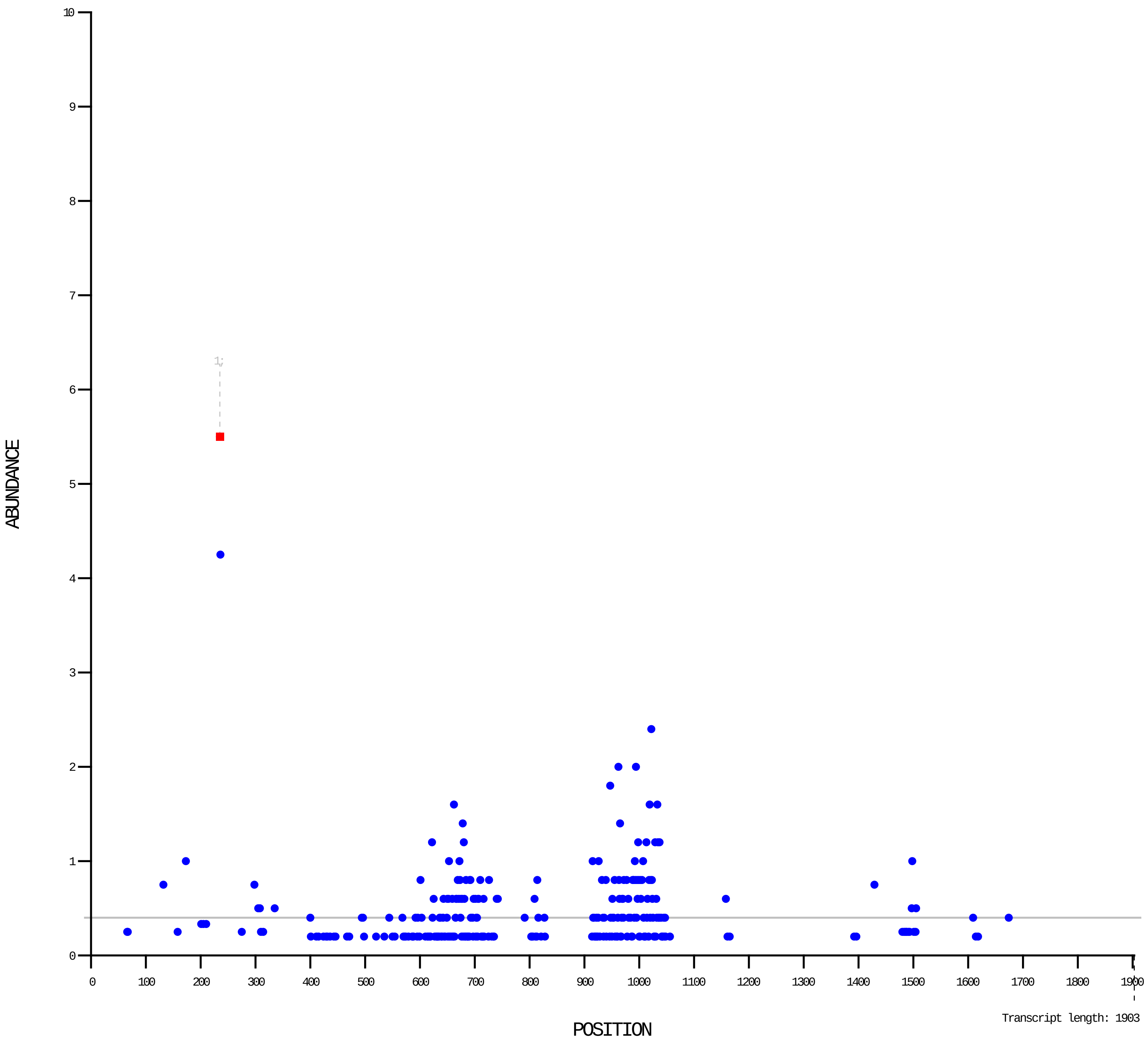

Category: 0 1 2 3 4

Degradome alignment: ● Median:

```
0  #1  Position:235  Abundance: 5.50(deg) 1(sRNA)
   5'     TGTGTTCTCAGGTCACCCCTT         3'  ID:
          || ||||o||||o|||| |o              Score: 4.5
   3' AACAACCCAAGGGTCCGGTGGCGGCGAAACGT 5'  p-value: 0.03
```

Cs5g28880.2 gene=Cs5g28880 CDS=271-1410

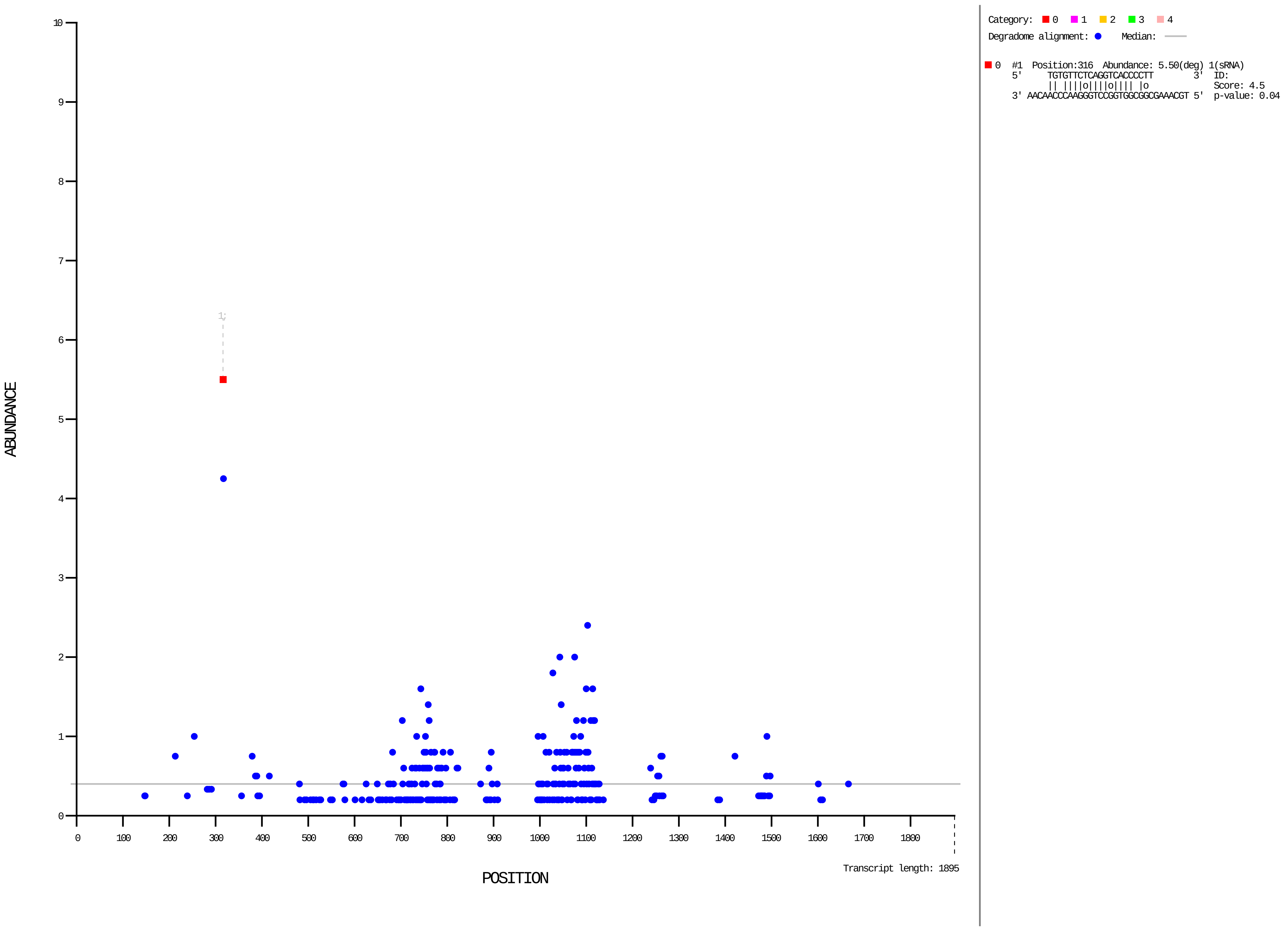

Cs1g07400.2 gene=Cs1g07400 CDS=1477-2718

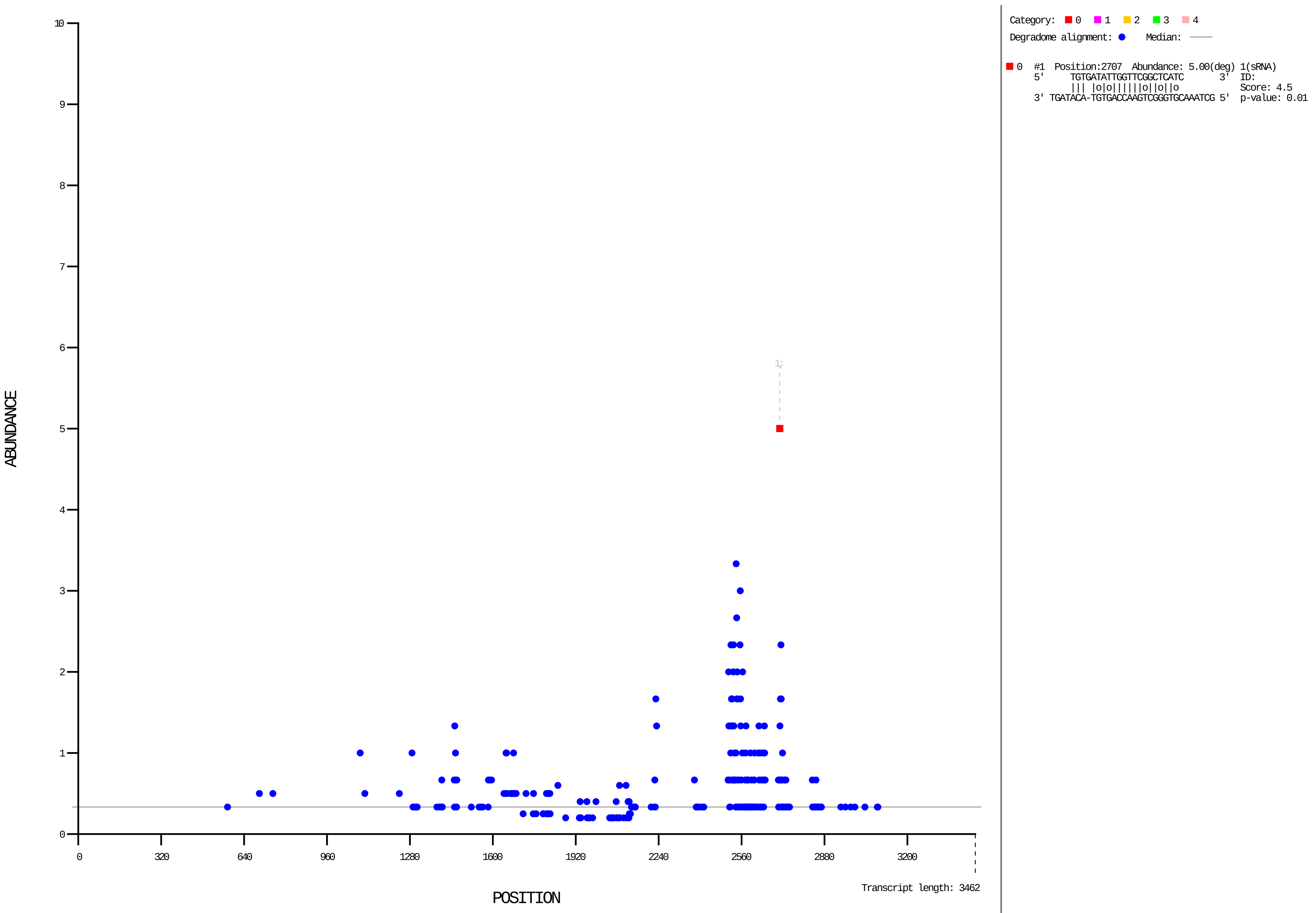

Cs1g07400.3 gene=Cs1g07400 CDS=869-2110

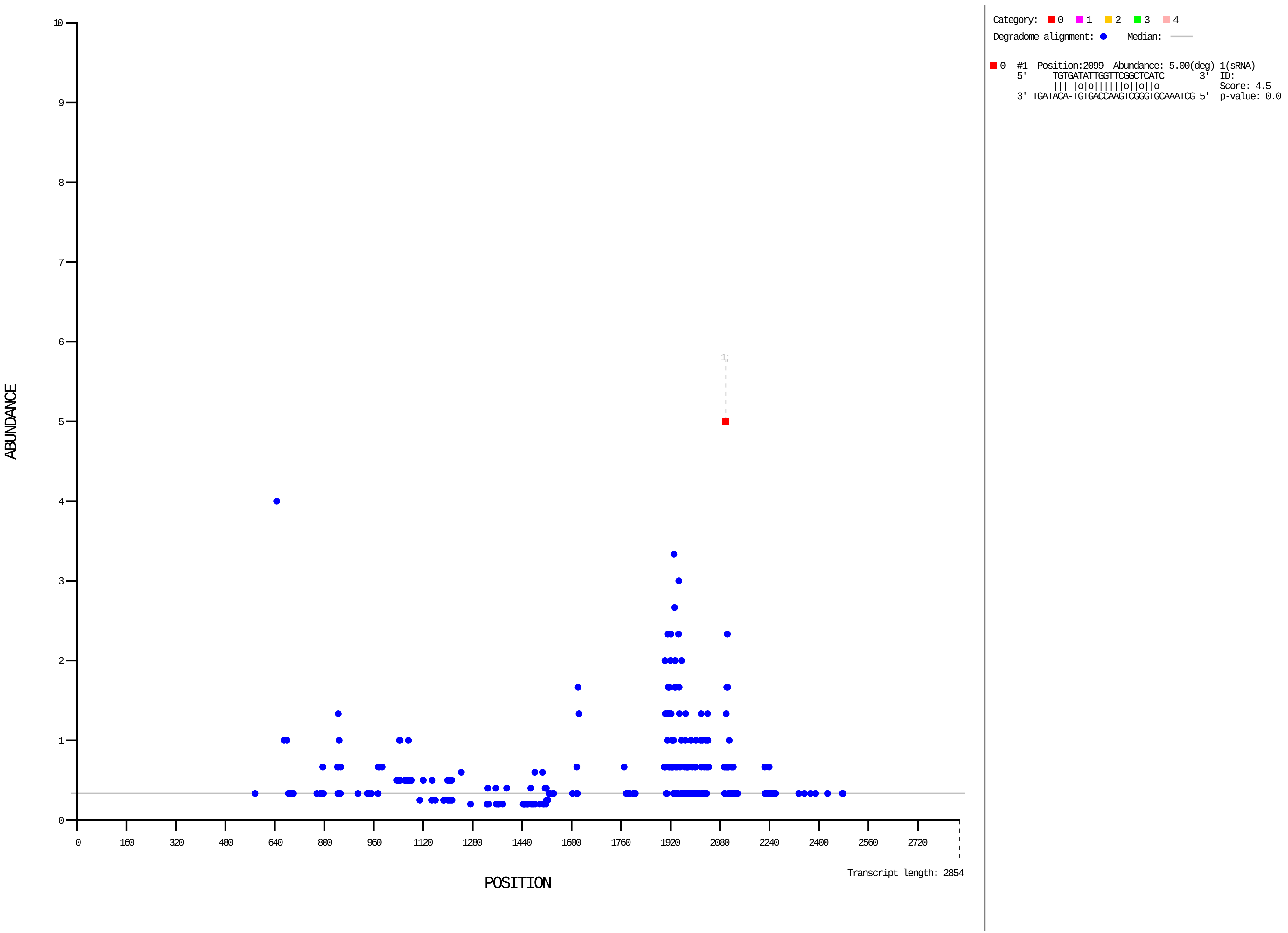

Cs1g07400.1 gene=Cs1g07400 CDS=1521-2645

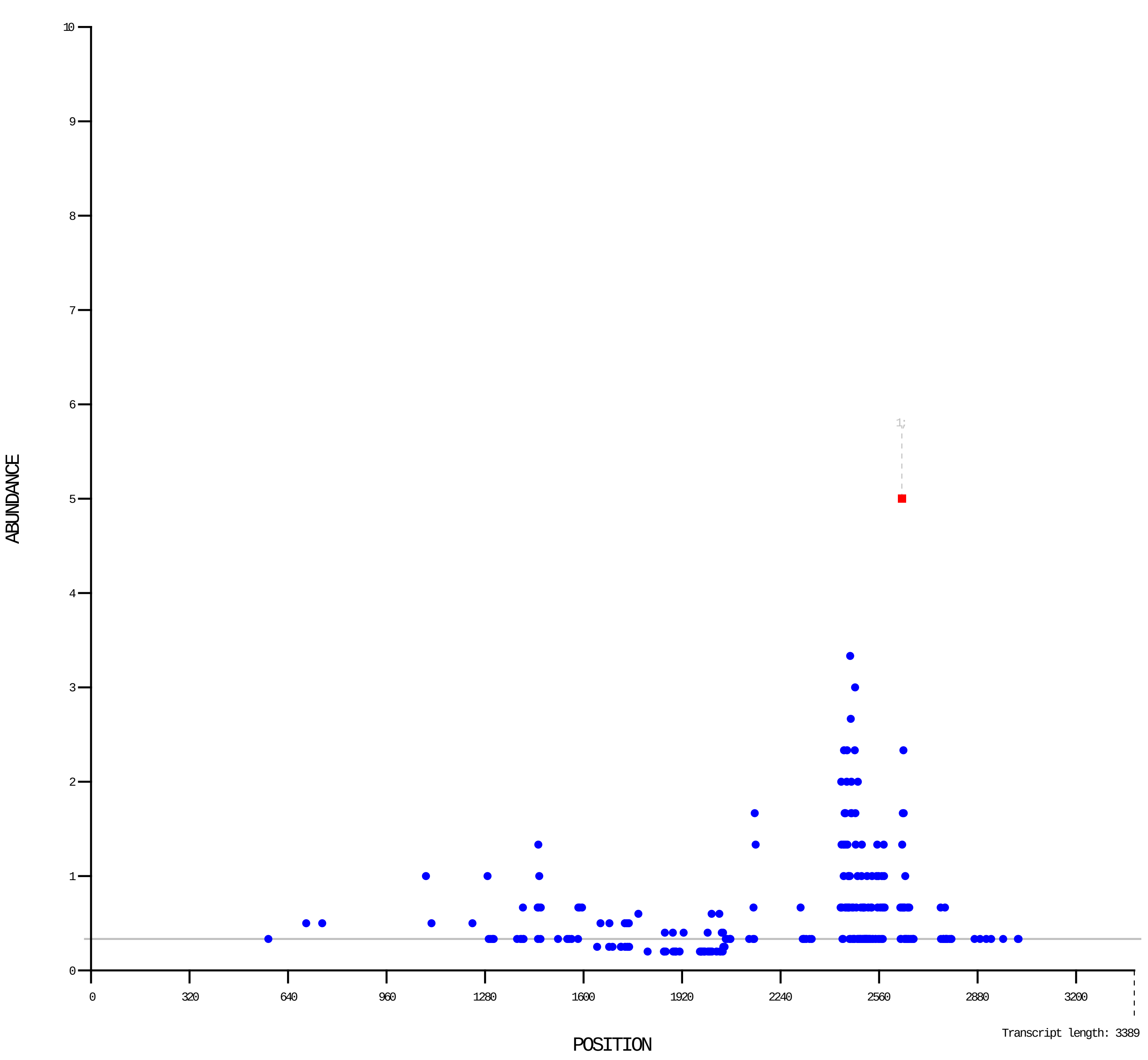

Category: 0 1 2 3 4

Degradome alignment: Median:

```
0  #1  Position:2634  Abundance: 5.00(deg) 1(sRNA)
   5'     TGTGATATTGGTTCGGCTCATC       3'  ID:
          ||| |o|o||||||o||o||o            Score: 4.5
   3' TGATACA-TGTGACCAAGTCGGGTGCAAATCG 5'  p-value: 0.01
```

Cs3g17060.2 gene=Cs3g17060 CDS=271-1182

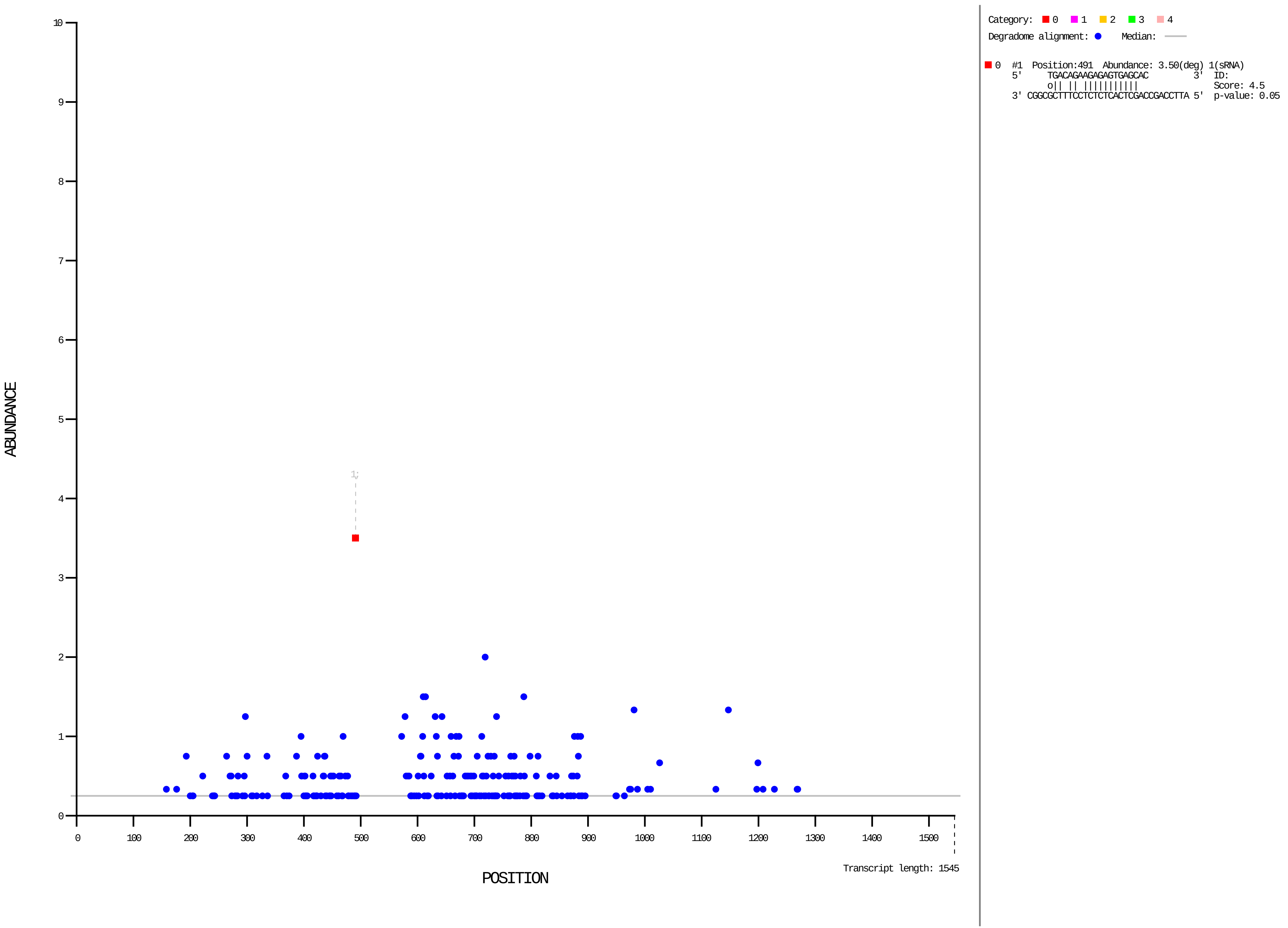

Cs3g24450.2 gene=Cs3g24450 CDS=81-1241

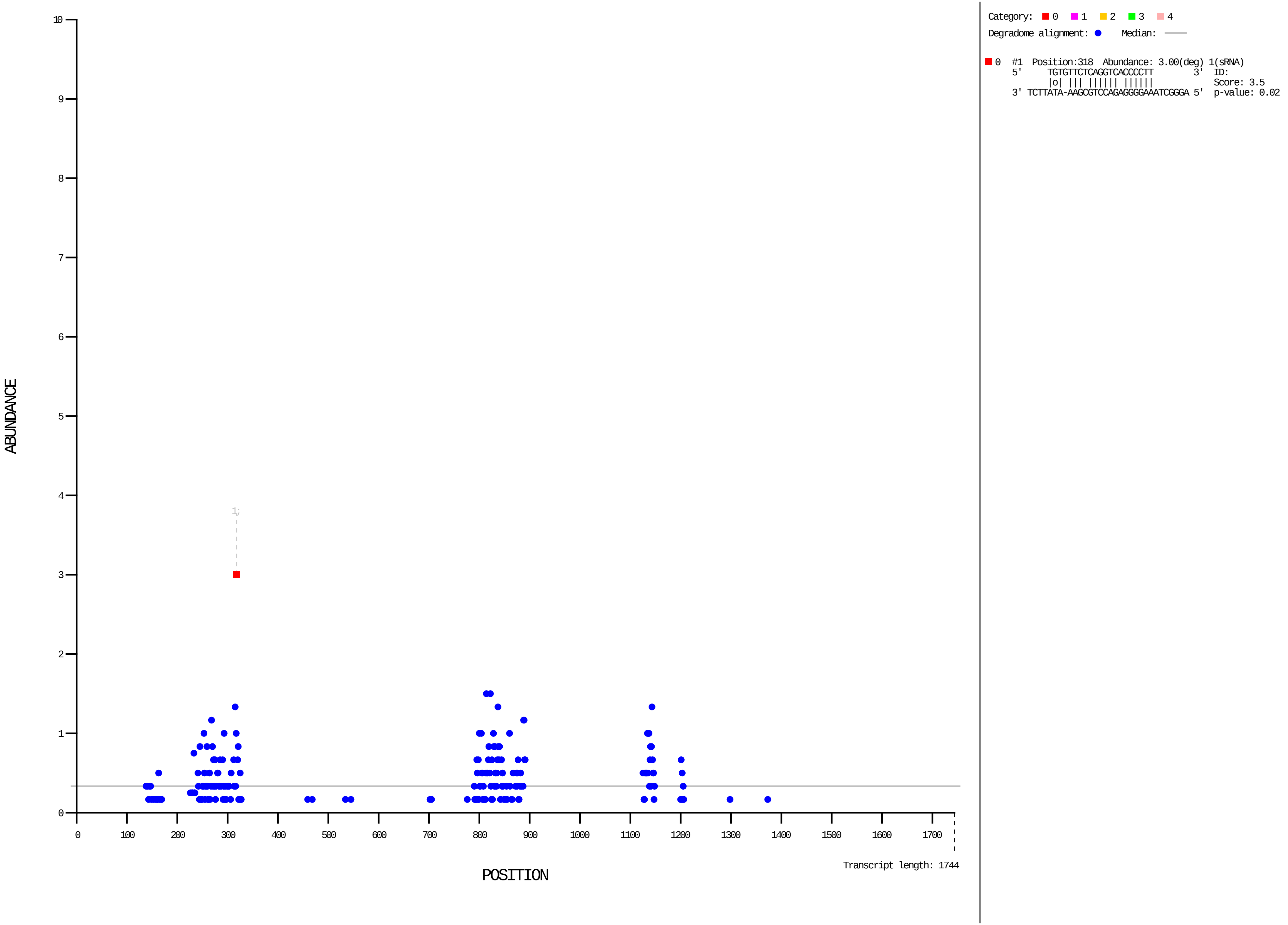

Cs3g24450.5 gene=Cs3g24450 CDS=506-1423

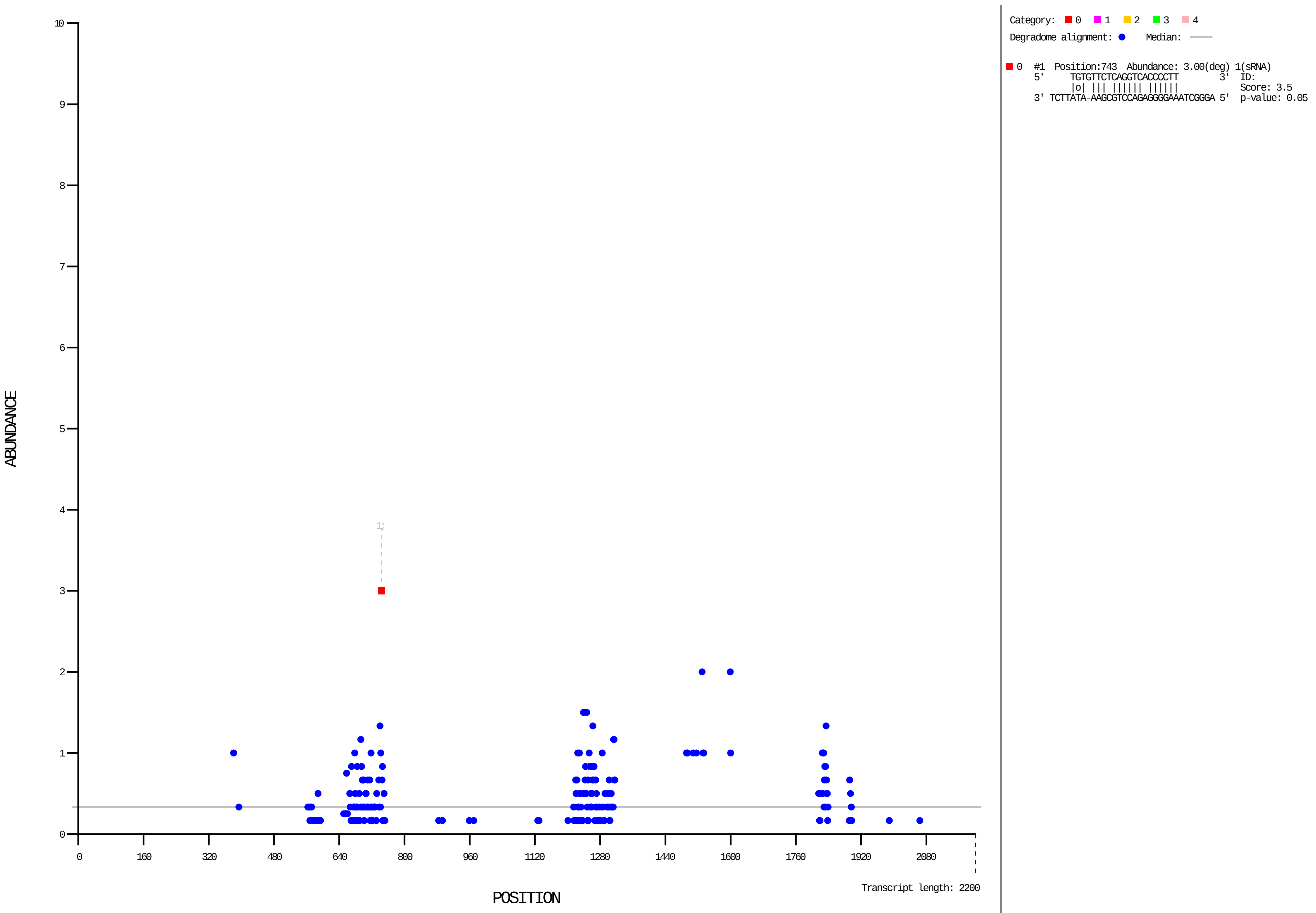

orange1.1t04538.1 gene=orange1.1t04538 CDS=1-2040

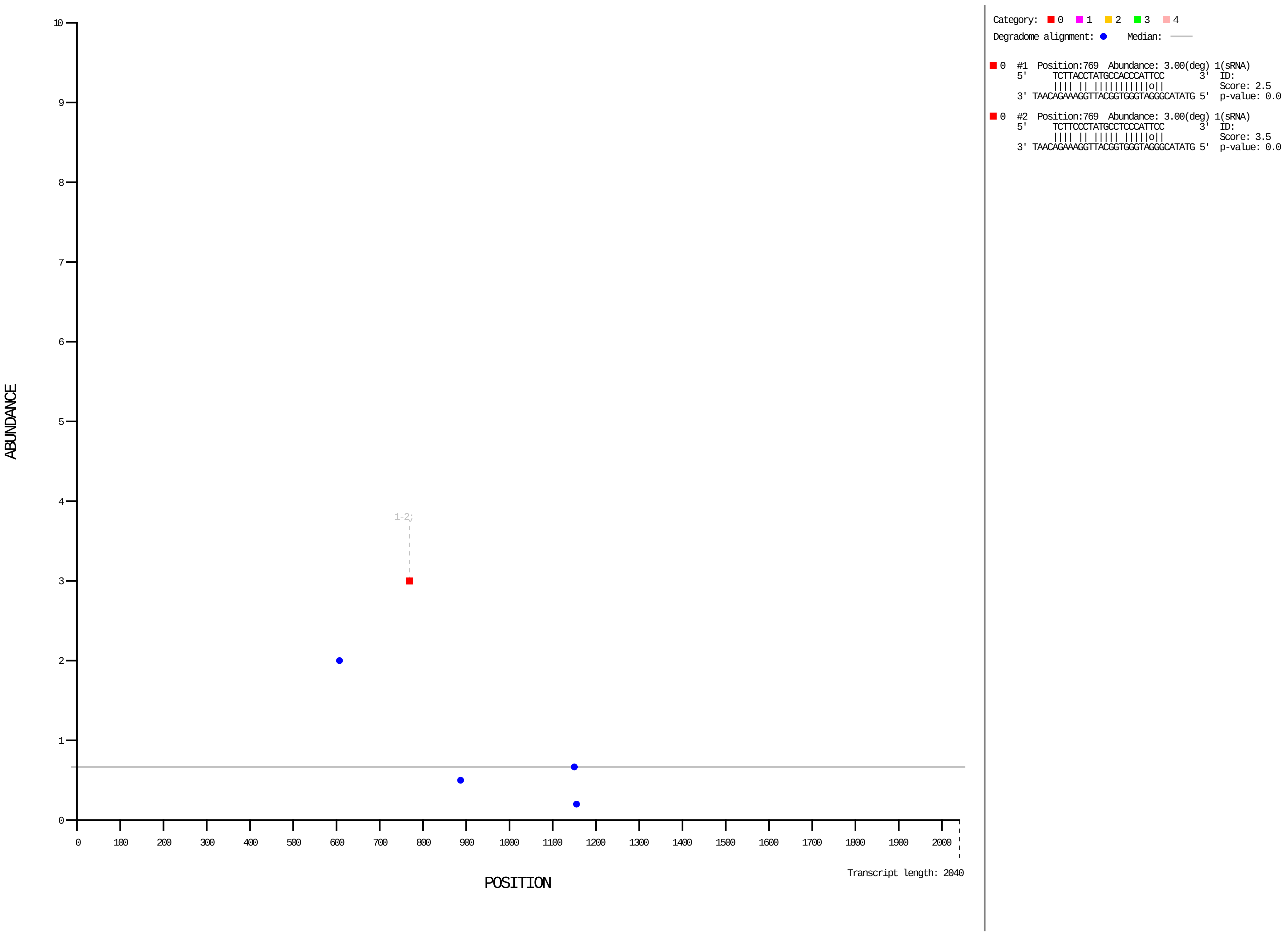

Cs3g24450.1 gene=Cs3g24450 CDS=310-1470

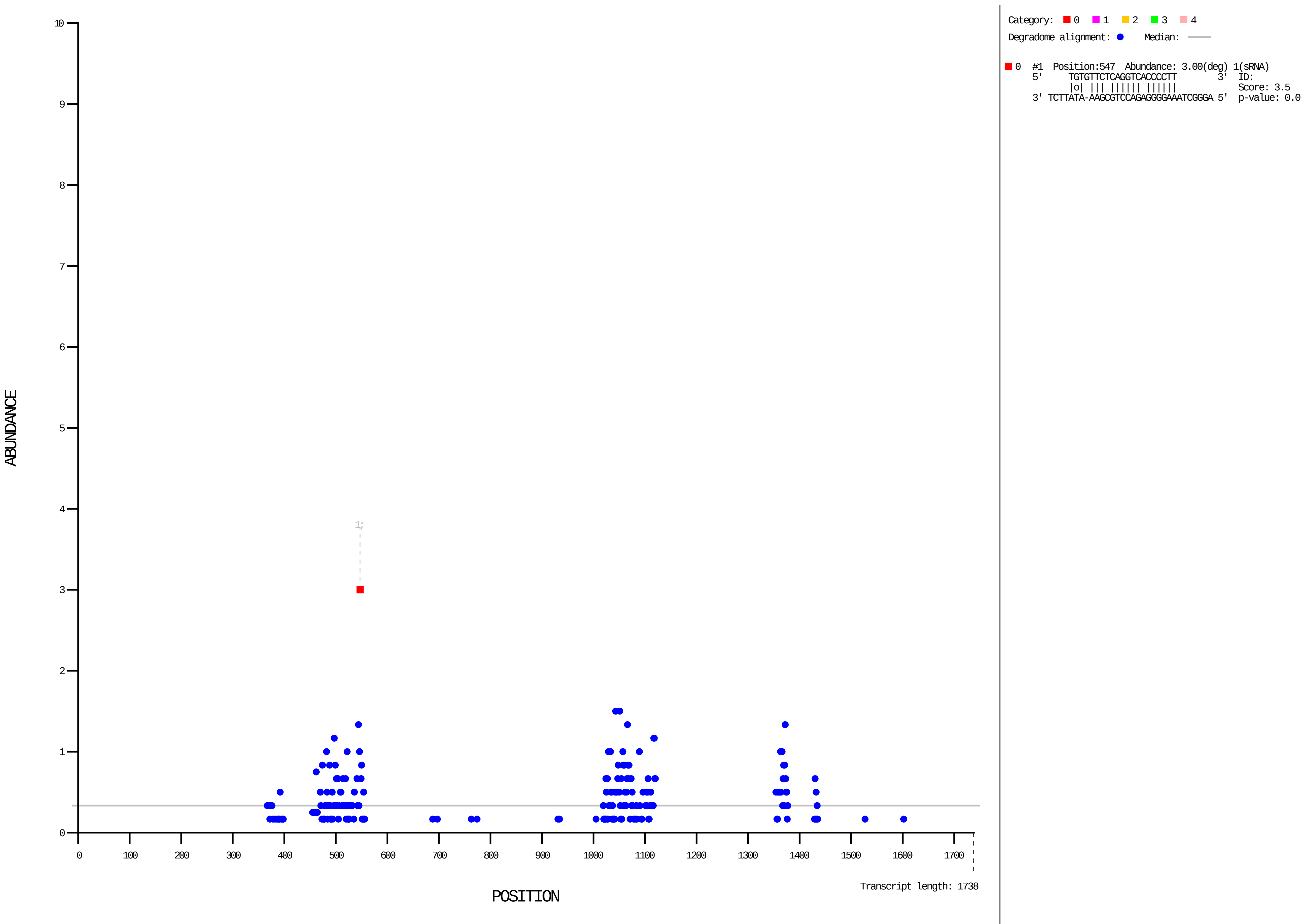

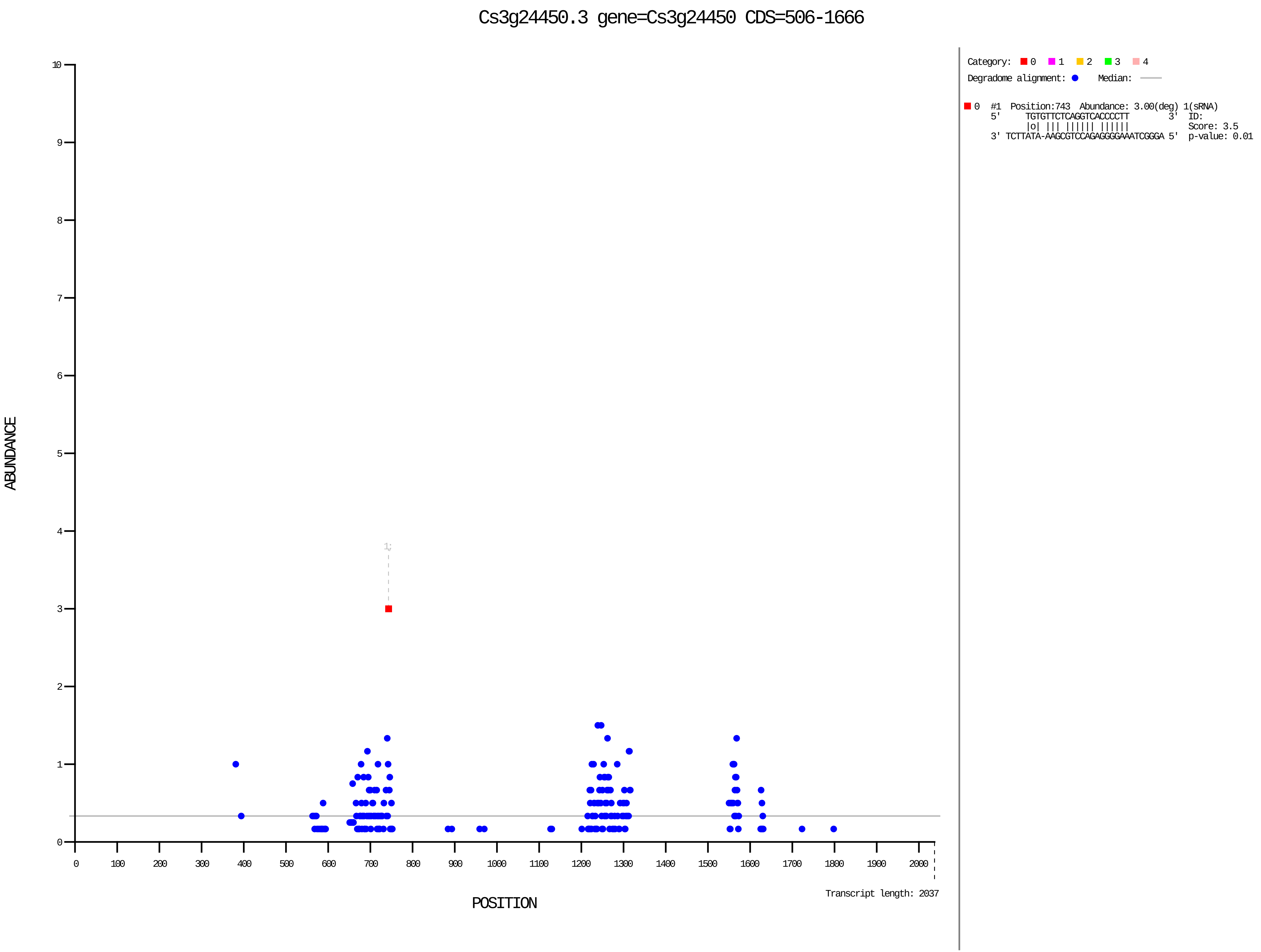

Cs3g24450.3 gene=Cs3g24450 CDS=506-1666
ABUNDANCE
POSITION
Transcript length: 2037
Category: 0 1 2 3 4
Degradome alignment: Median:
0 #1 Position:743 Abundance: 3.00(deg) 1(sRNA)
5' TGTGTTCTCAGGTCACCCCTT 3' ID:
|o| ||| |||||| ||||||
Score: 3.5
3' TCTTATA-AAGCGTCCAGAGGGGAAATCGGGA 5' p-value: 0.01

Cs3g24450.6 gene=Cs3g24450 CDS=1502-2509

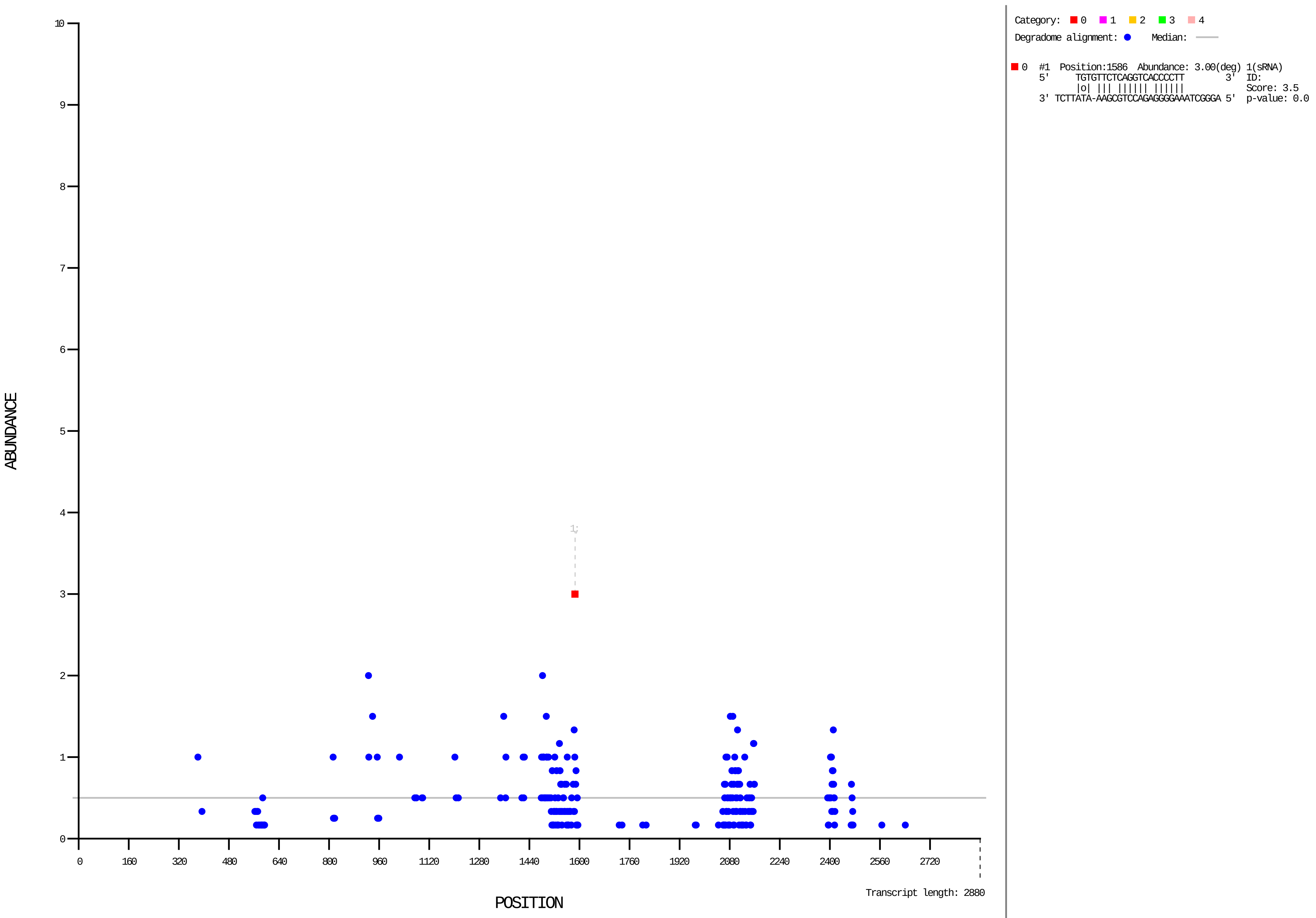

Cs3g24450.4 gene=Cs3g24450 CDS=1077-2084

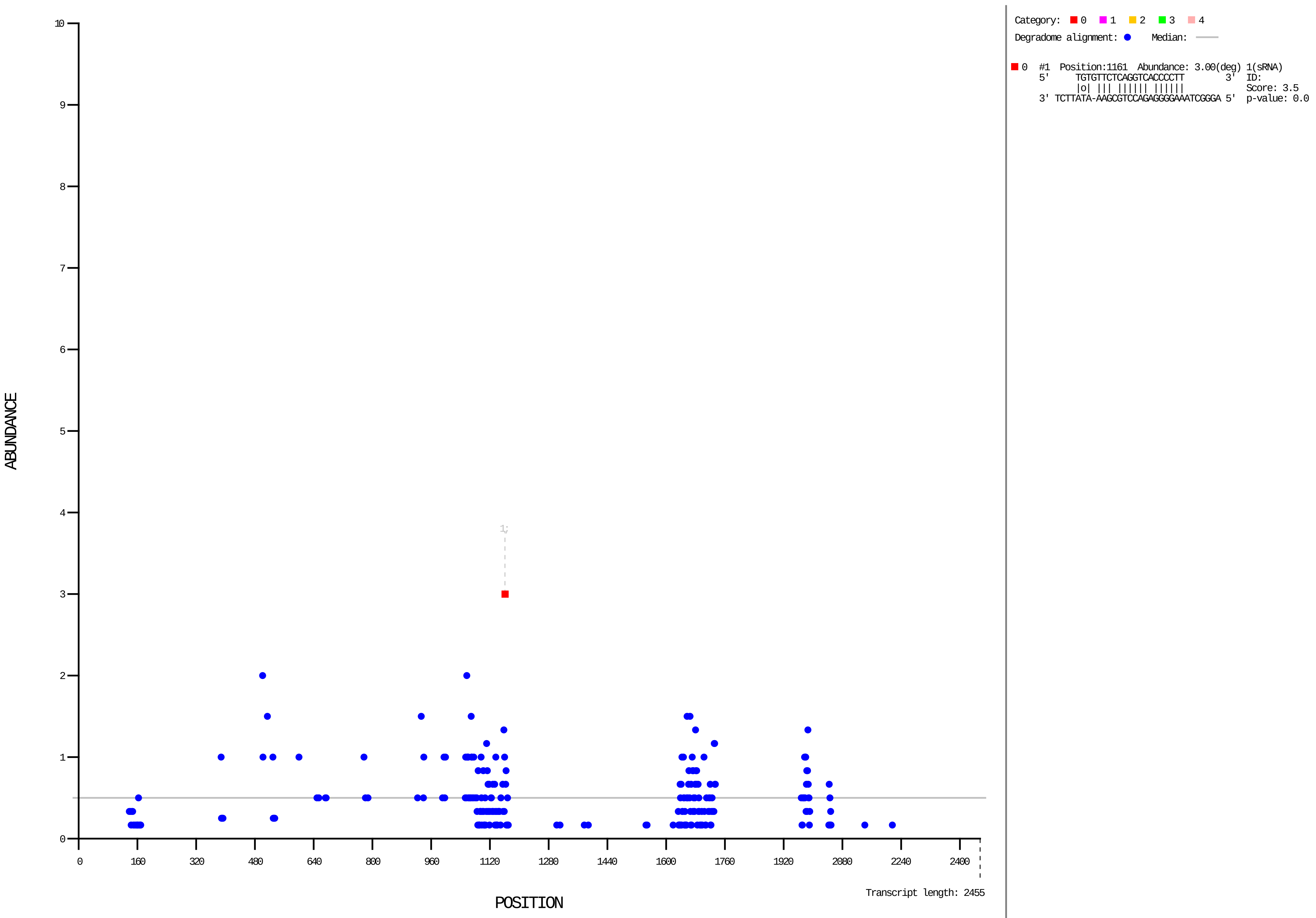

orange1.1t04830.1 gene=orange1.1t04830 CDS=15-2501

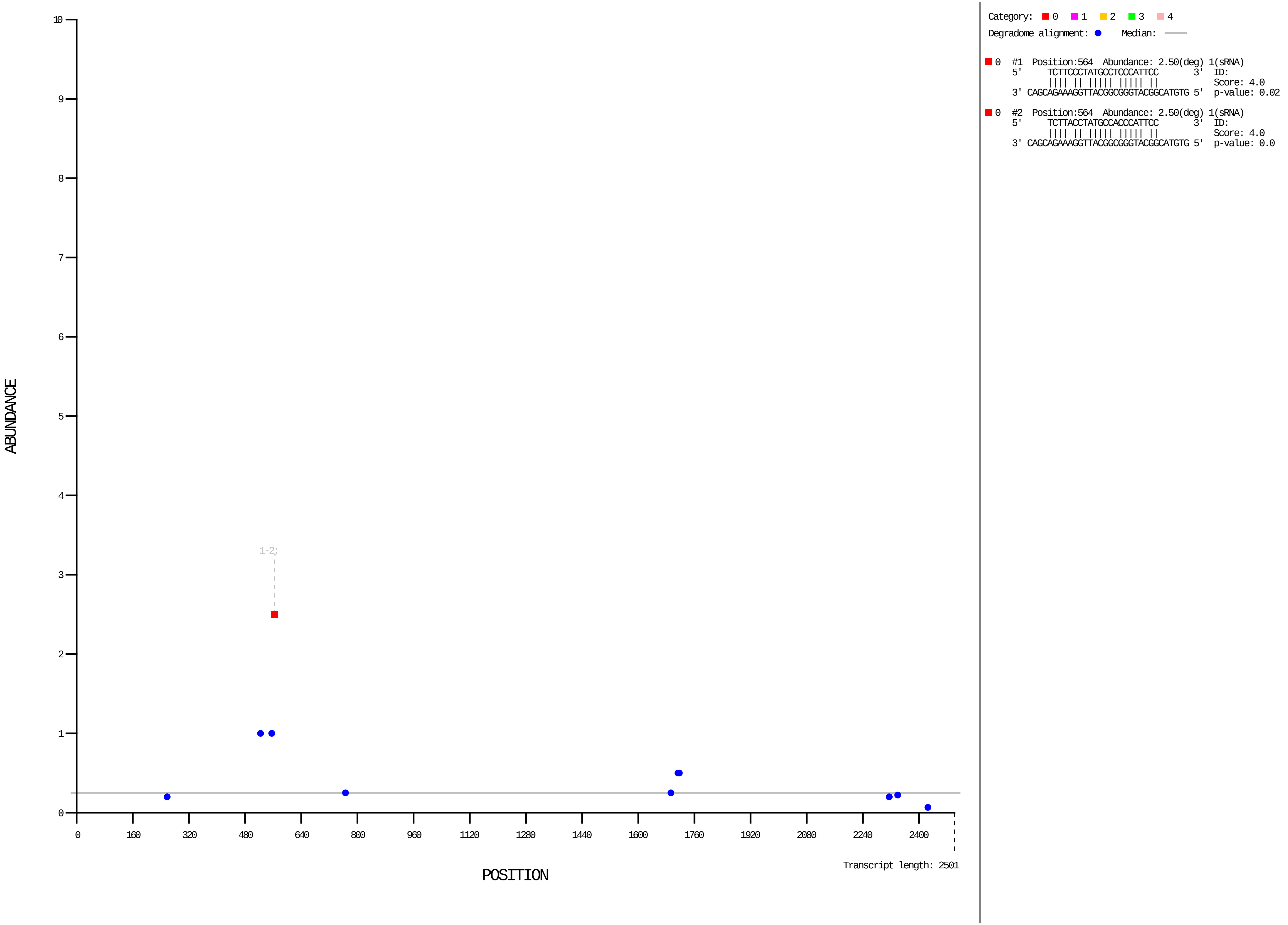

orange1.1t05193.1 gene=orange1.1t05193 CDS=1-1208

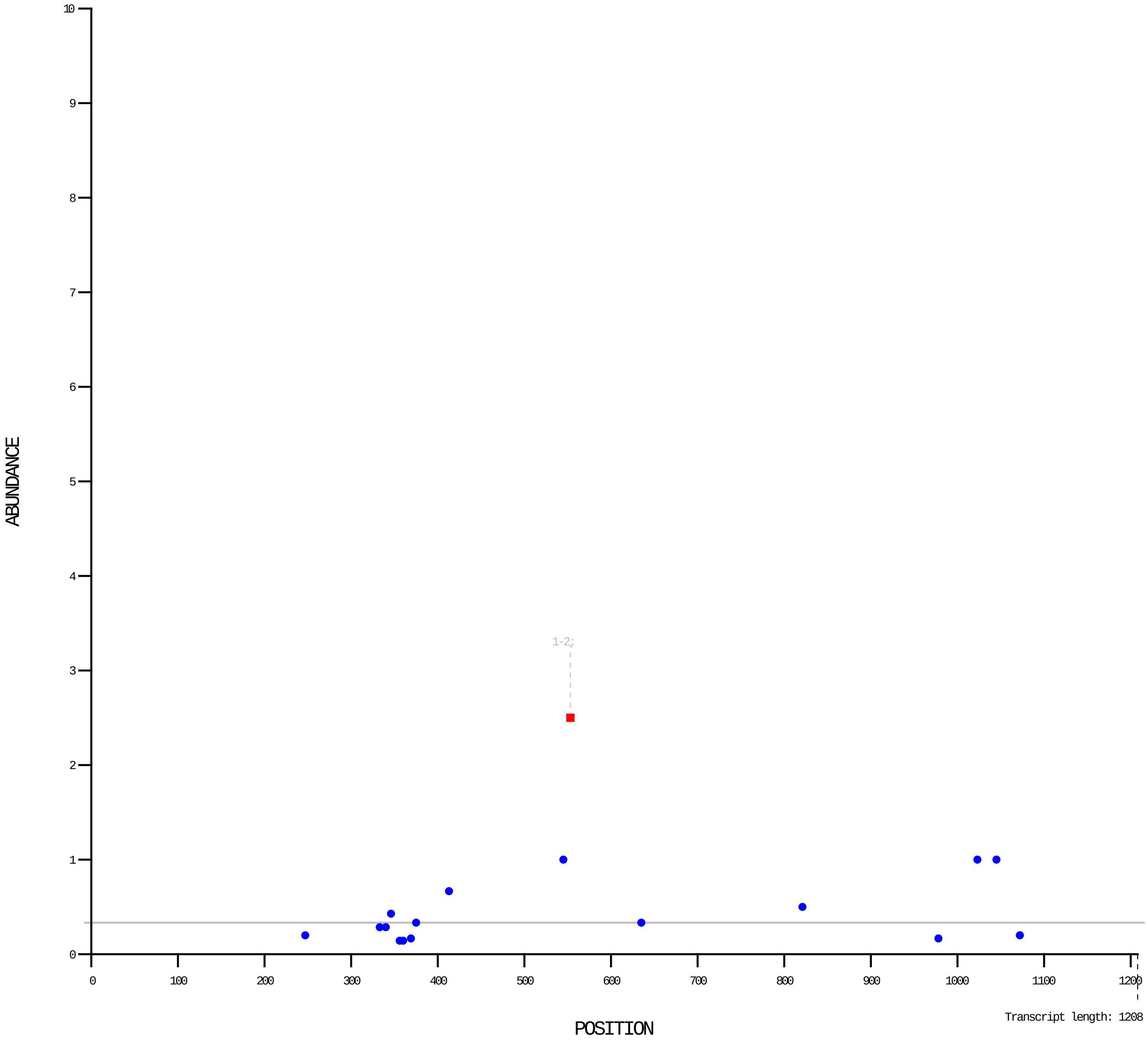


```
Category: 0  1  2  3  4
Degradome alignment:    Median:

0  #1  Position:553  Abundance: 2.50(deg) 1(sRNA)
   5'     TCTTCCCTATGCCTCCCATTCC       3'  ID:
          |||| || ||||| ||||| ||           Score: 4.0
   3' CAGCAGAAAGGTTACGGCGGGTACGGCATGTG 5'  p-value: 0.01

0  #2  Position:553  Abundance: 2.50(deg) 1(sRNA)
   5'     TCTTACCTATGCCACCCATTCC       3'  ID:
          |||| || ||||| ||||| ||           Score: 4.0
   3' CAGCAGAAAGGTTACGGCGGGTACGGCATGTG 5'  p-value: 0.01
```

Cs6g06810.1 gene=Cs6g06810 CDS=27-944

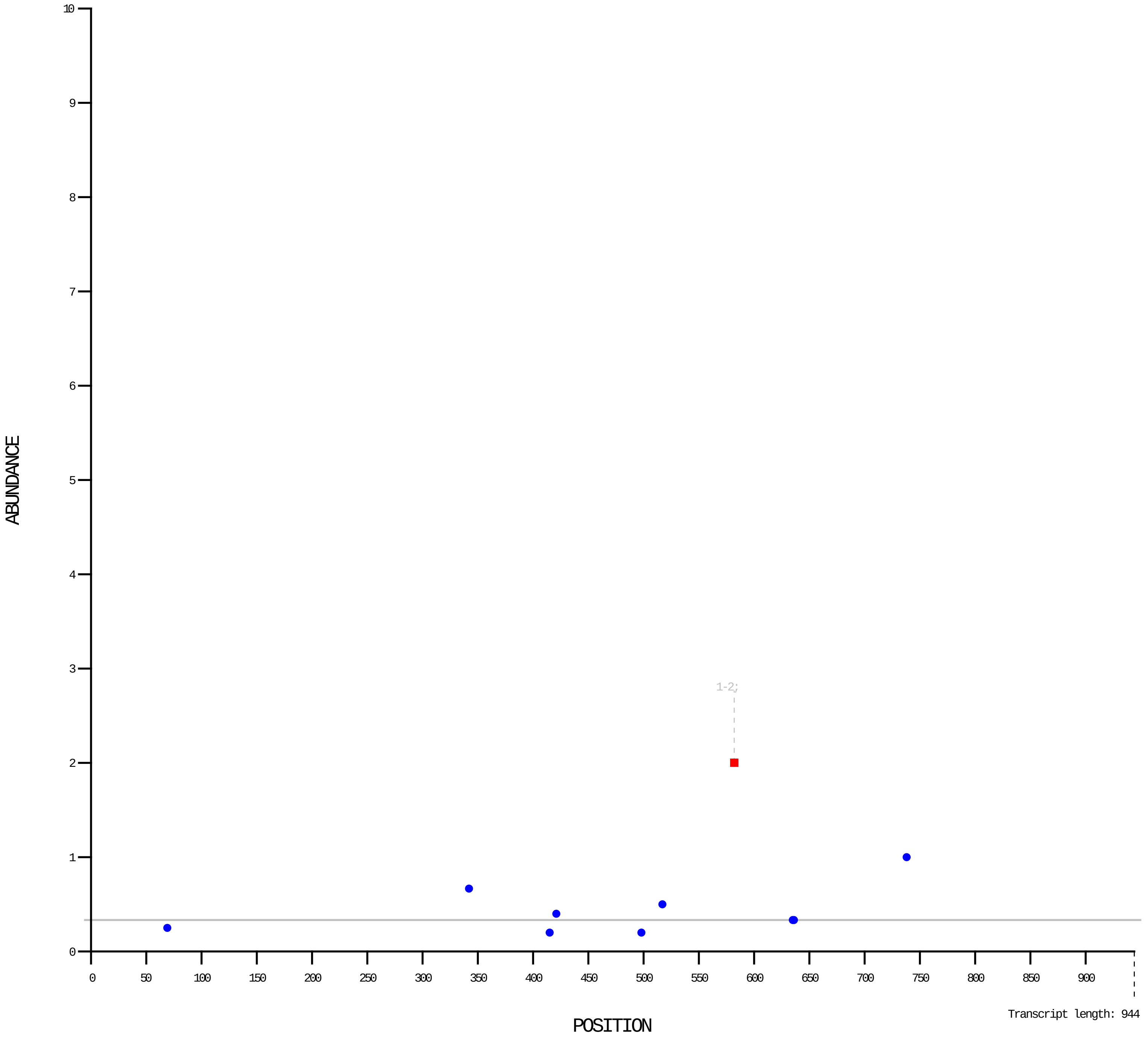

Category: 0 1 2 3 4
Degradome alignment: Median:

```
0  #1  Position:582  Abundance: 2.00(deg) 1(sRNA)
   5'     TCTTACCTATGCCACCCATTCC       3'  ID:
          |||| || |||||||||||o||           Score: 2.5
   3' TAGCAGAAAGGCTACGGTGGGTAGGGTATTTA 5'  p-value: 0.0

0  #2  Position:582  Abundance: 2.00(deg) 1(sRNA)
   5'     TCTTCCCTATGCCTCCCATTCC       3'  ID:
          |||| || ||||| |||||o||           Score: 3.5
   3' TAGCAGAAAGGCTACGGTGGGTAGGGTATTTA 5'  p-value: 0.0
```

Cs5g09850.1 gene=Cs5g09850 CDS=271-969

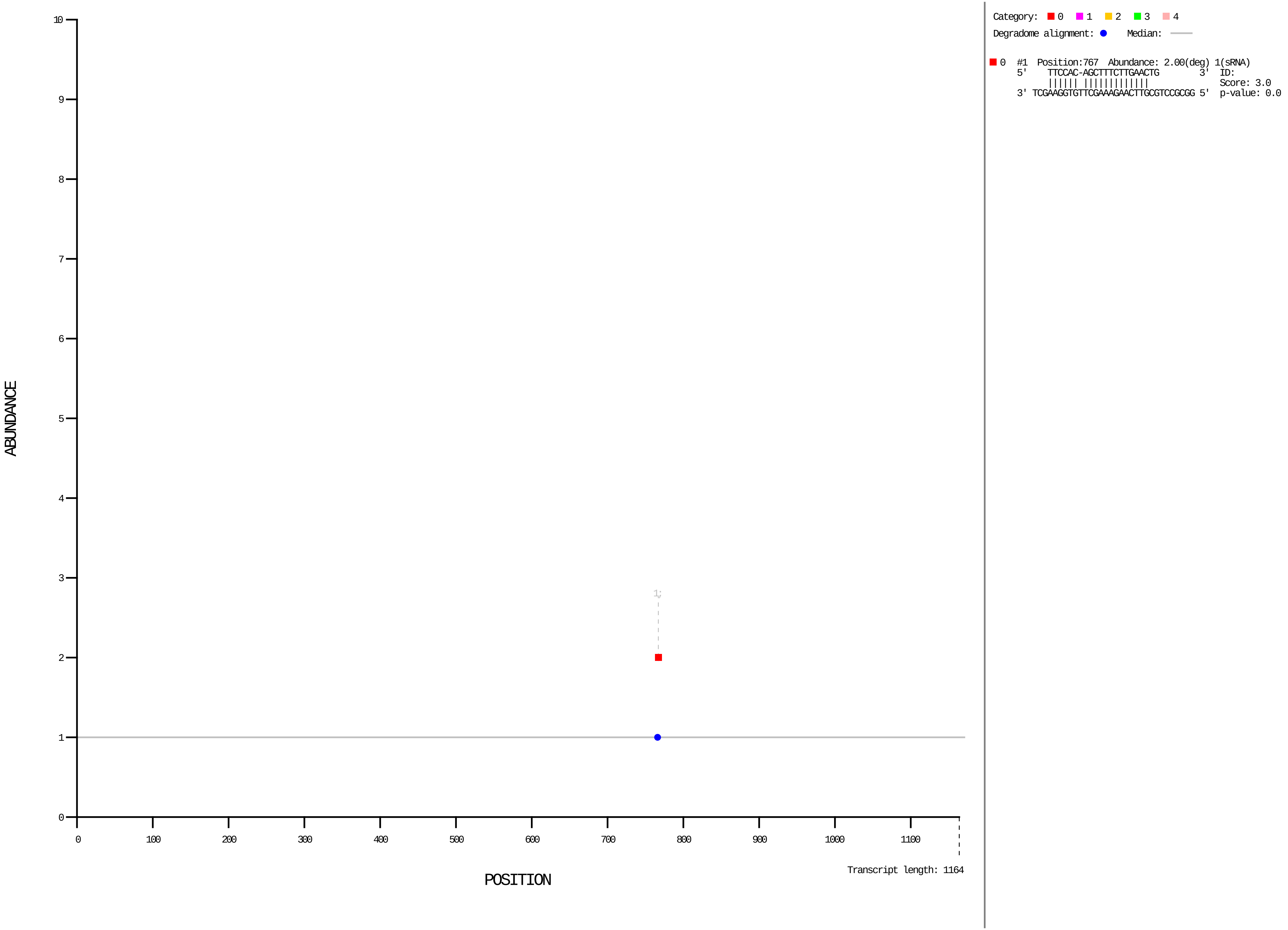

Cs3g10845.1 gene=Cs3g10845 CDS=78-274

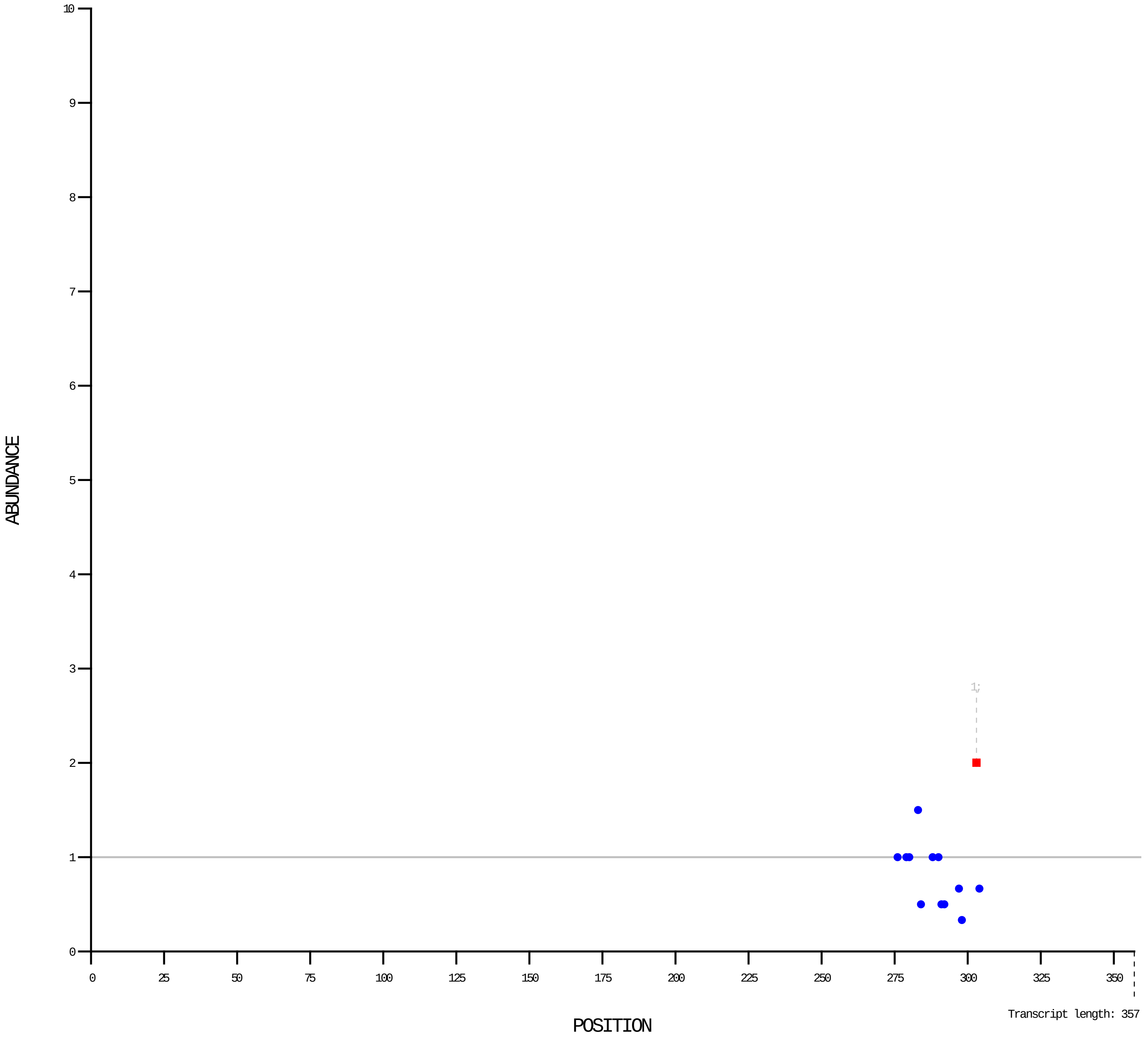

Category: 0 1 2 3 4
Degradome alignment: Median:

```
0  #1  Position:303  Abundance: 2.00(deg) 1(sRNA)
   5'     TTGAGTTCTGCAAGCCGTCGA        3'  ID:
          |||||o||||| ||||o|  |            Score: 4.0
   3' CCTAAACTCGAGACGGTCGGTAAATTTAAATT 5'  p-value: 0.0
```

Cs3g10845.2 gene=Cs3g10845 CDS=28-224

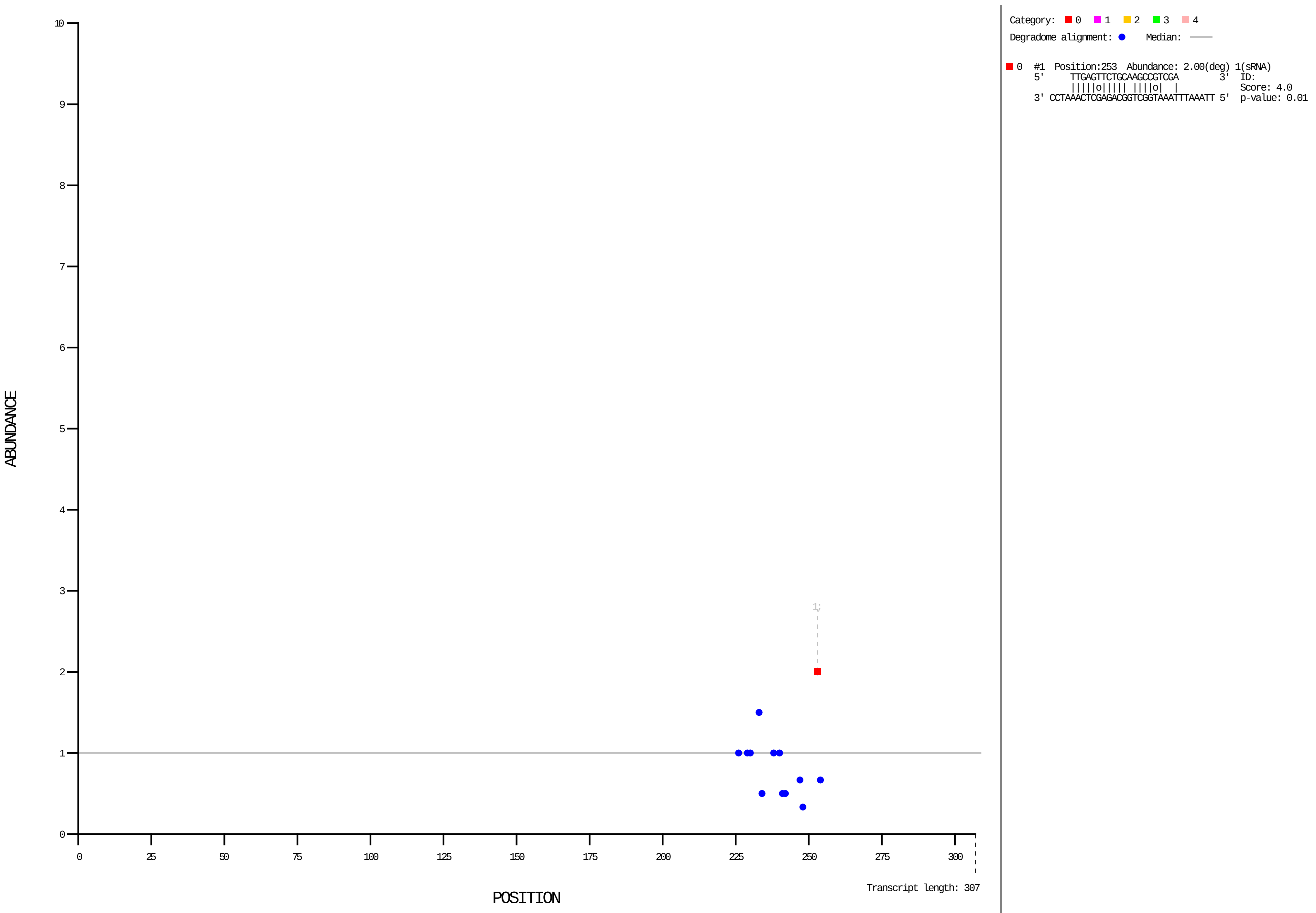

Cs1g01140.1 gene=Cs1g01140 CDS=1-3429

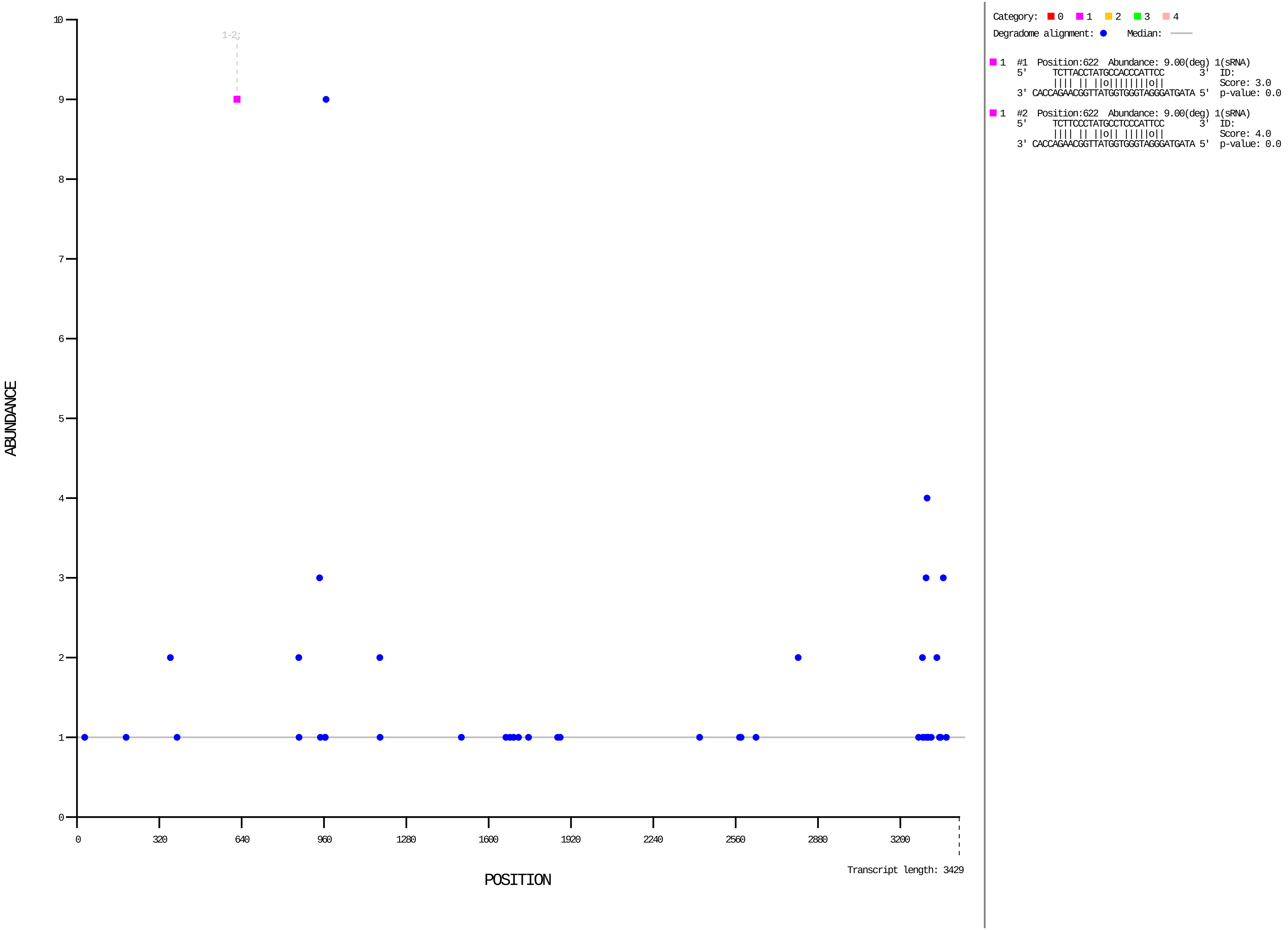

Cs9g06390.1 gene=Cs9g06390 CDS=210-2216

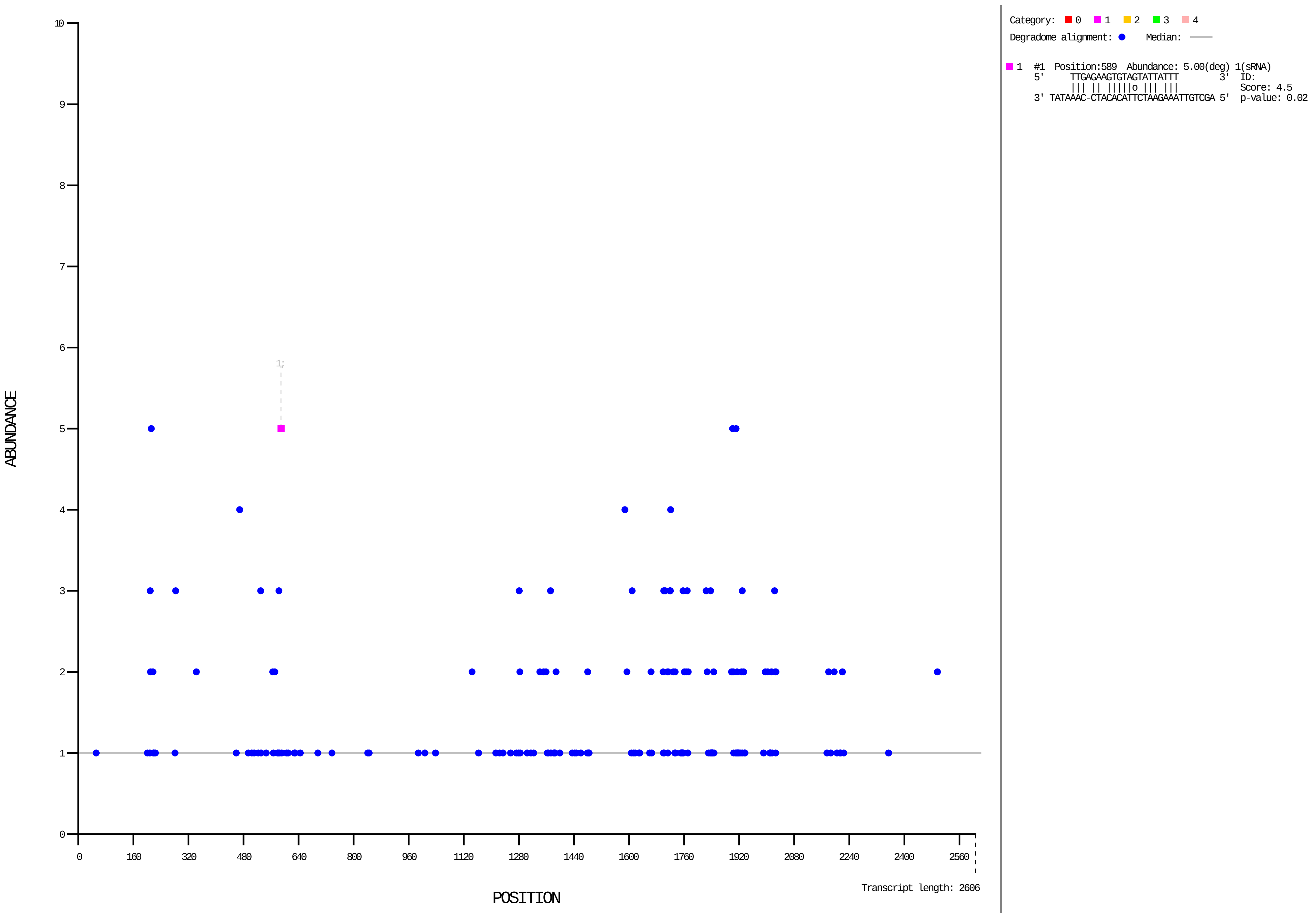

Cs6g04120.1 gene=Cs6g04120 CDS=213-1679

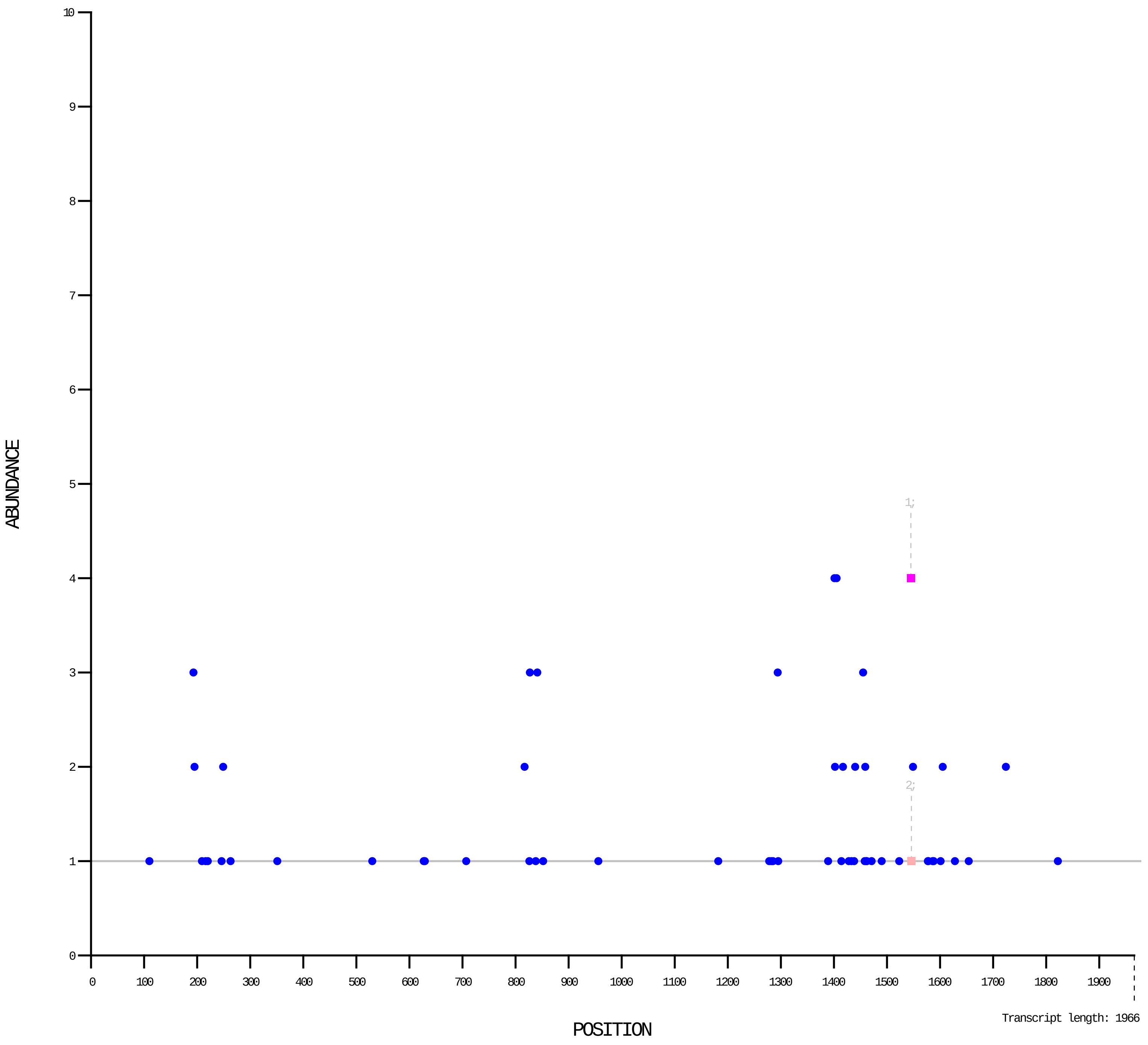

Category: 0 1 2 3 4

Degradome alignment: ● Median:

```
1  #1  Position:1545  Abundance: 4.00(deg) 1(sRNA)
   5'     AGAATCTTGATGATGCTGCA          3'  ID:
          |||||o||||||||||||||              Score: 1.5
   3' CTTCCCTTAGGACTACTACGACGTCATCTCTT 5'  p-value: 0.0

4  #2  Position:1546  Abundance: 1.00(deg) 1(sRNA)
   5'     TGGAATCTTGATGATGCTGCAG        3'  ID:
          ||||||o||||||||||||||             Score: 1.5
   3' ACTTCCCTTAGGACTACTACGACGTCATCTCT 5'  p-value: 0.0
```

Cs3g12760.1 gene=Cs3g12760 CDS=491-2215

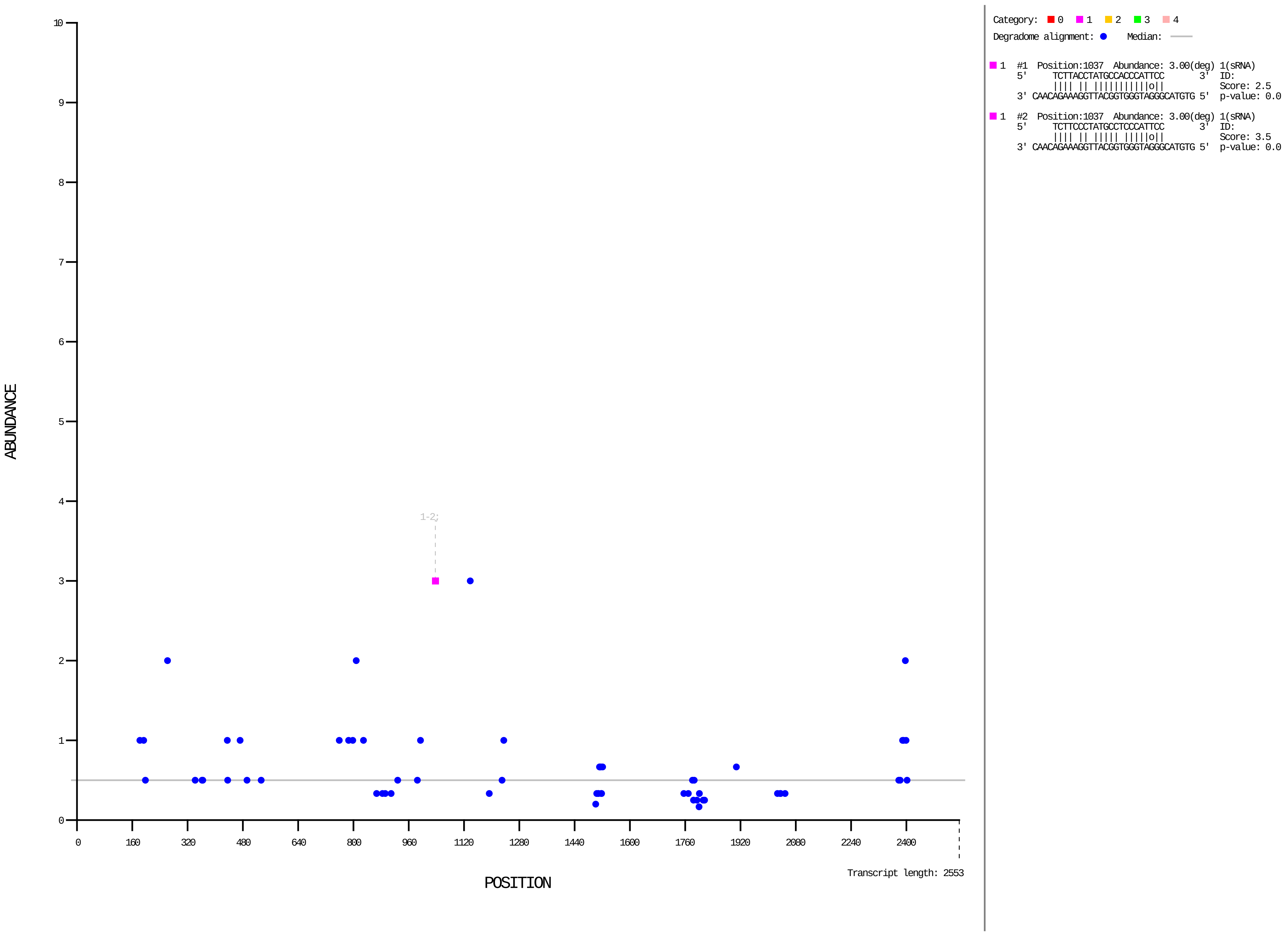

Cs1g03790.1 gene=Cs1g03790 CDS=911-1666

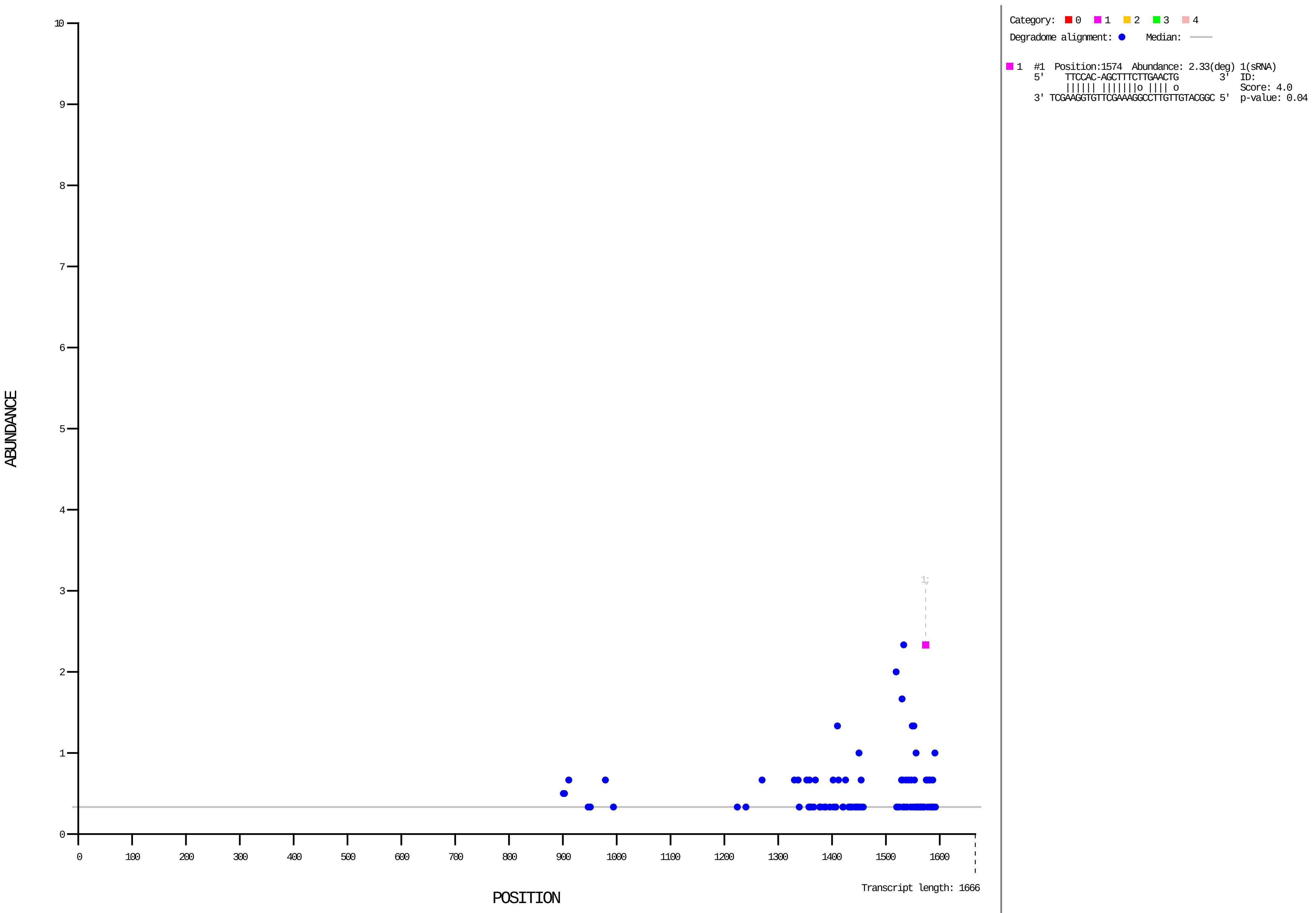

Cs5g08750.1 gene=Cs5g08750 CDS=1-528

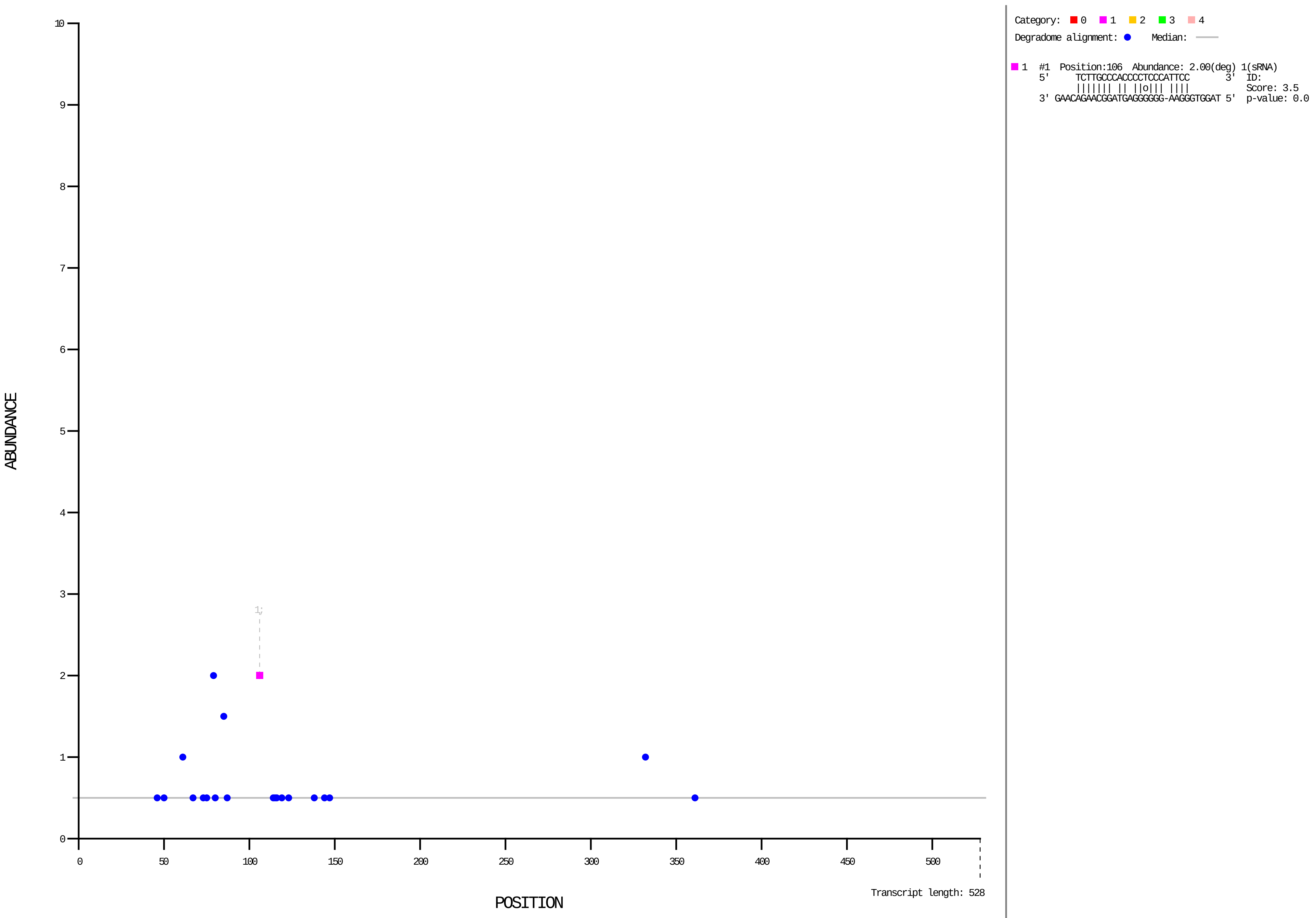

Cs1g02450.1 gene=Cs1g02450 CDS=197-610

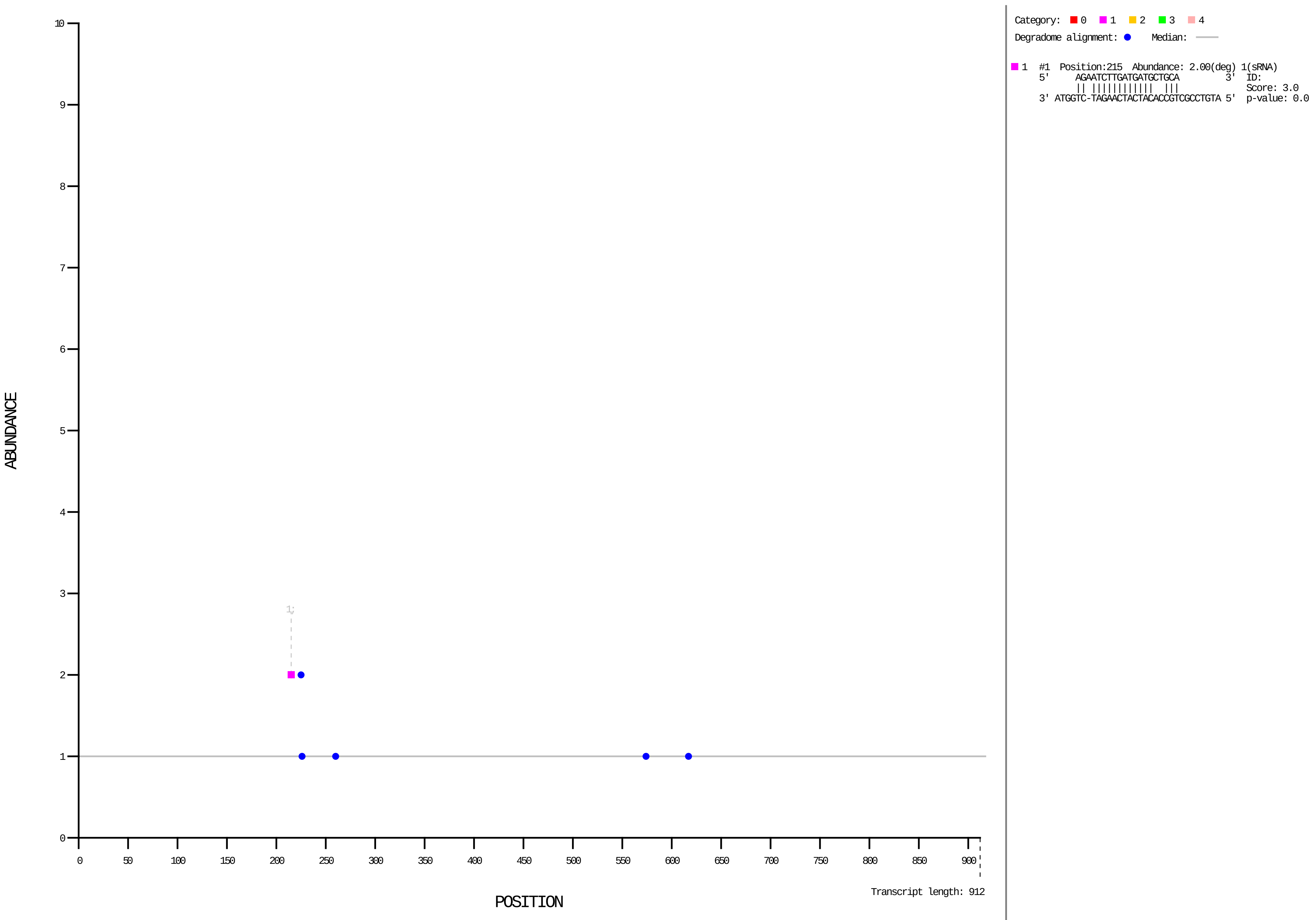

Cs1g14910.3 gene=Cs1g14910 CDS=120-1439

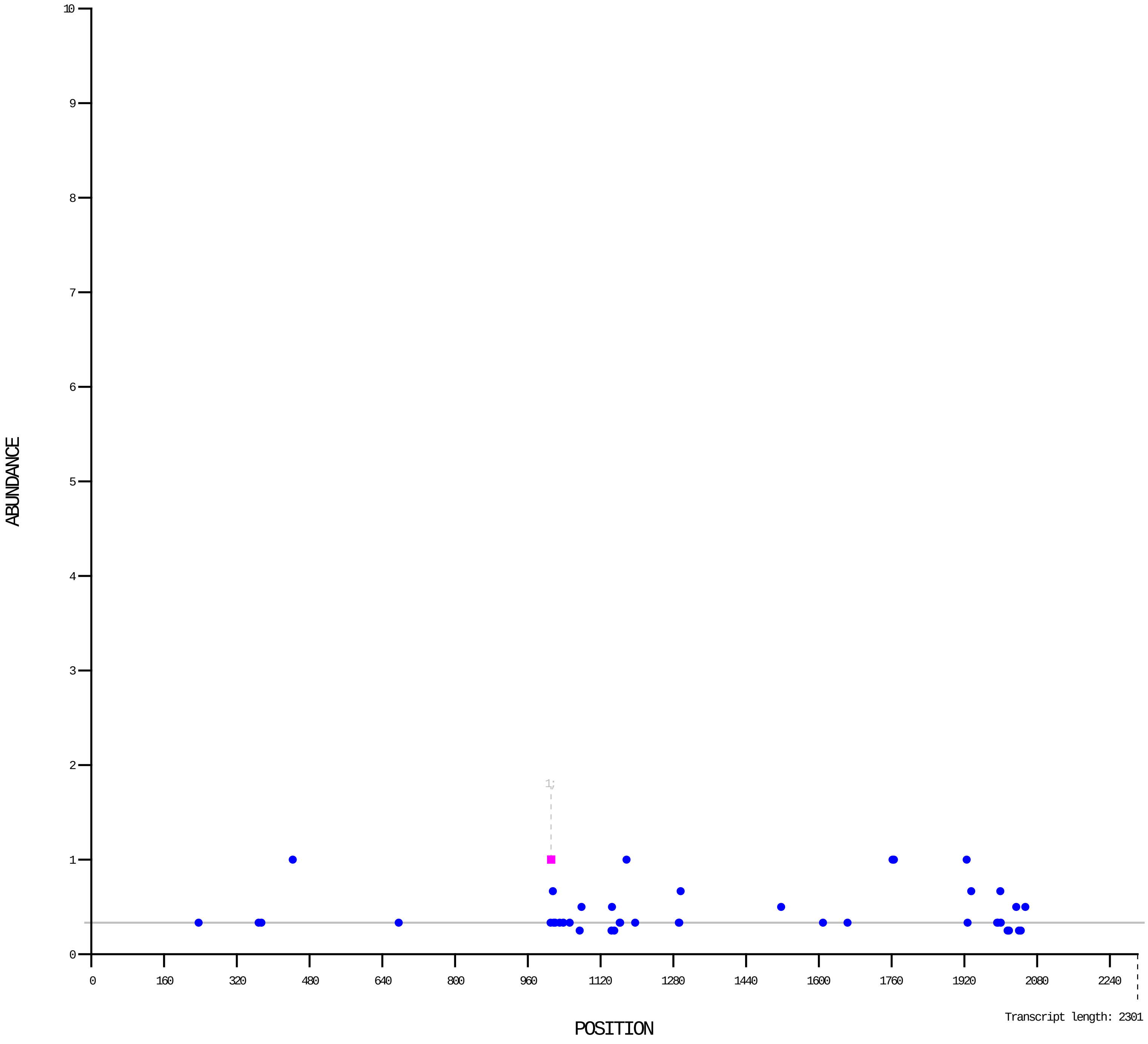

Category: 0 1 2 3 4

Degradome alignment: ● Median: —

1 #1 Position:1011 Abundance: 1.00(deg) 1(sRNA)

```
5'     TTAGATTCACGCACAAACTCG        3'  ID:
       |o| ||||||o| || ||||o            Score: 4.5
3' GCGAAGTGTAAGTGTGAGTGTGAGTGTGTGAC 5'  p-value: 0.03
```

Cs1g14910.1 gene=Cs1g14910 CDS=1-1428

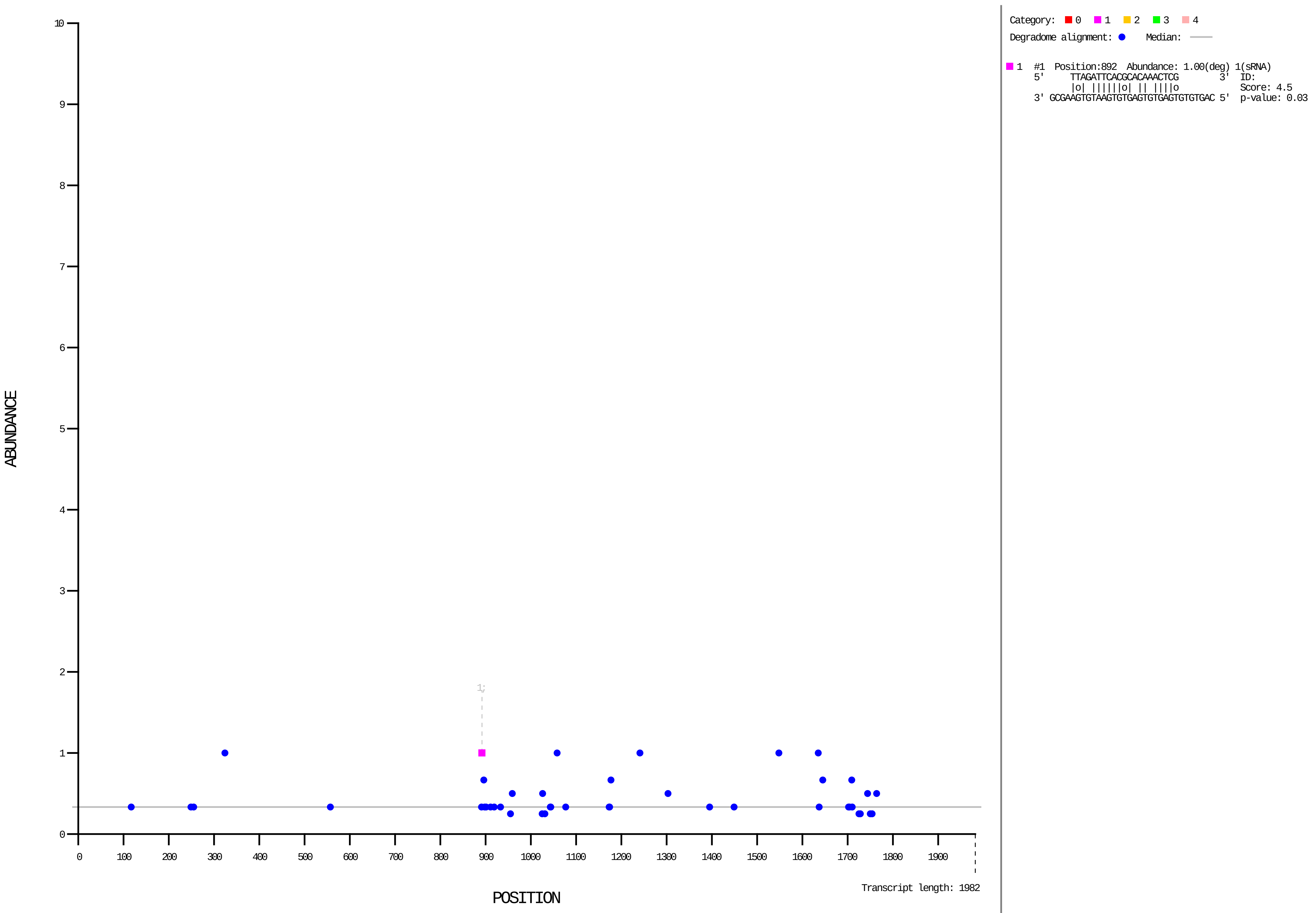

Cs3g13430.1 gene=Cs3g13430 CDS=613-4896

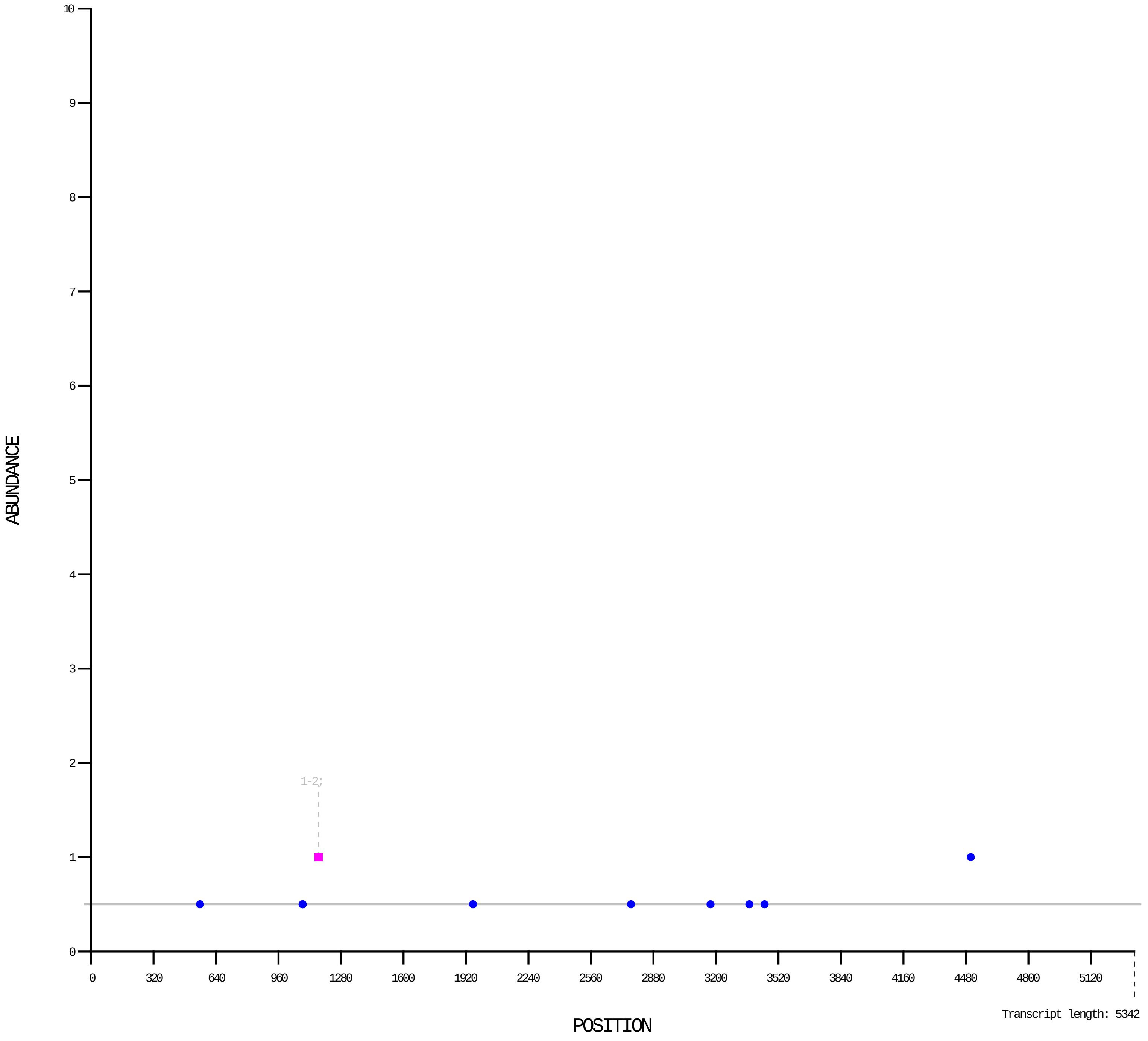

Category: 0 1 2 3 4

Degradome alignment: Median:

```
1  #1  Position:1165  Abundance: 1.00(deg) 1(sRNA)
   5'     TCTTCCCTATGCCTCCCATTCC       3'  ID:
          ||||||| ||||| ||||| ||           Score: 3.0
   3' CACCAGAAGGGTTACGGCGGGTACGGCATGTG 5'  p-value: 0.0

1  #2  Position:1165  Abundance: 1.00(deg) 1(sRNA)
   5'     TCTTACCTATGCCACCCATTCC       3'  ID:
          |||| || ||||| ||||| ||           Score: 4.0
   3' CACCAGAAGGGTTACGGCGGGTACGGCATGTG 5'  p-value: 0.01
```

Cs3g13430.2 gene=Cs3g13430 CDS=613-4896

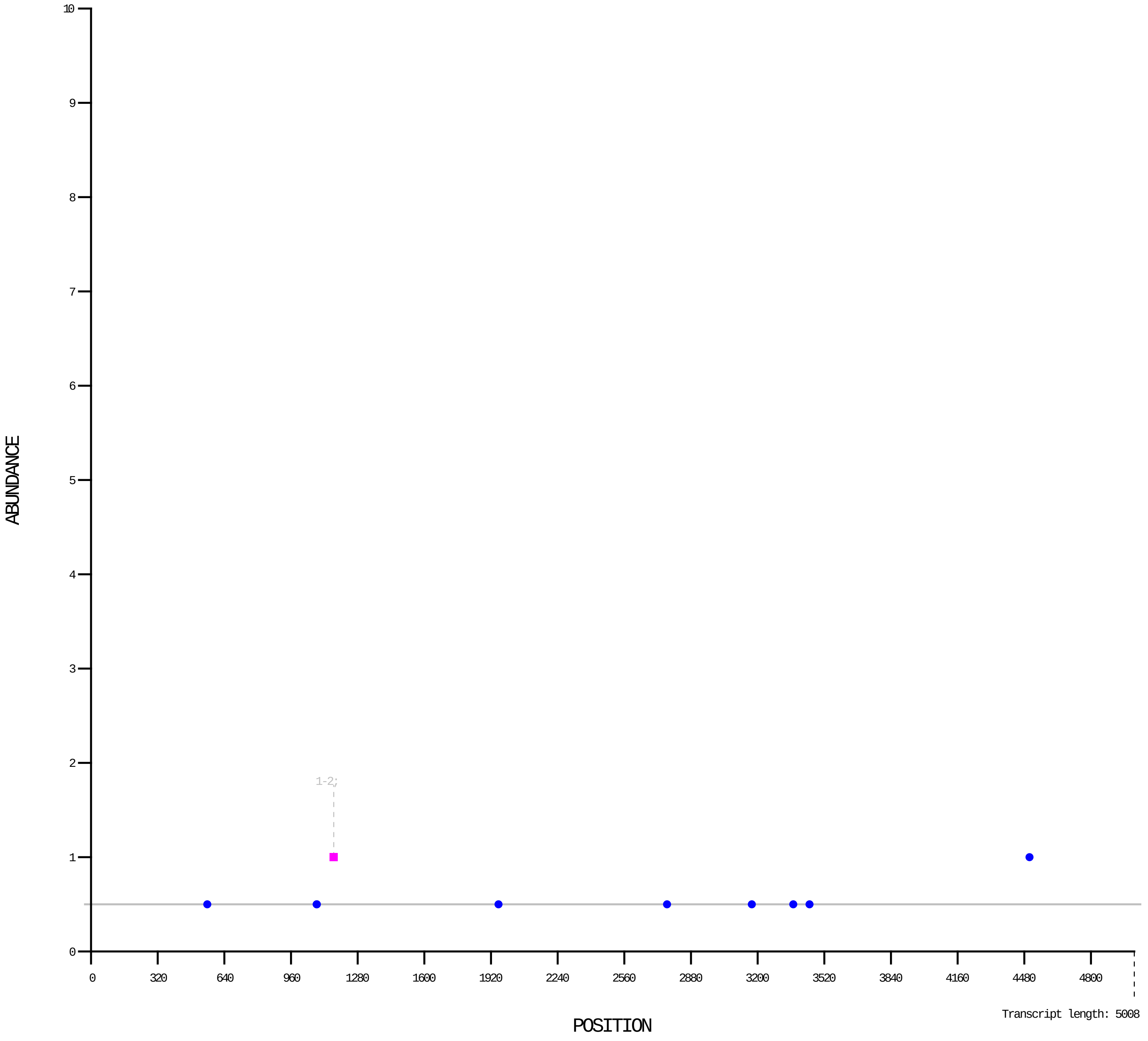

Category: 0 1 2 3 4

Degradome alignment: ● Median: —

```
1  #1  Position:1165  Abundance: 1.00(deg) 1(sRNA)
   5'     TCTTCCCTATGCCTCCCATTCC       3'  ID:
          ||||||| ||||| ||||| ||           Score: 3.0
   3' CACCAGAAGGGTTACGGCGGGTACGGCATGTG 5'  p-value: 0.0

1  #2  Position:1165  Abundance: 1.00(deg) 1(sRNA)
   5'     TCTTACCTATGCCACCCATTCC       3'  ID:
          |||| || ||||| ||||| ||           Score: 4.0
   3' CACCAGAAGGGTTACGGCGGGTACGGCATGTG 5'  p-value: 0.0
```

Cs1g14910.2 gene=Cs1g14910 CDS=1-1545

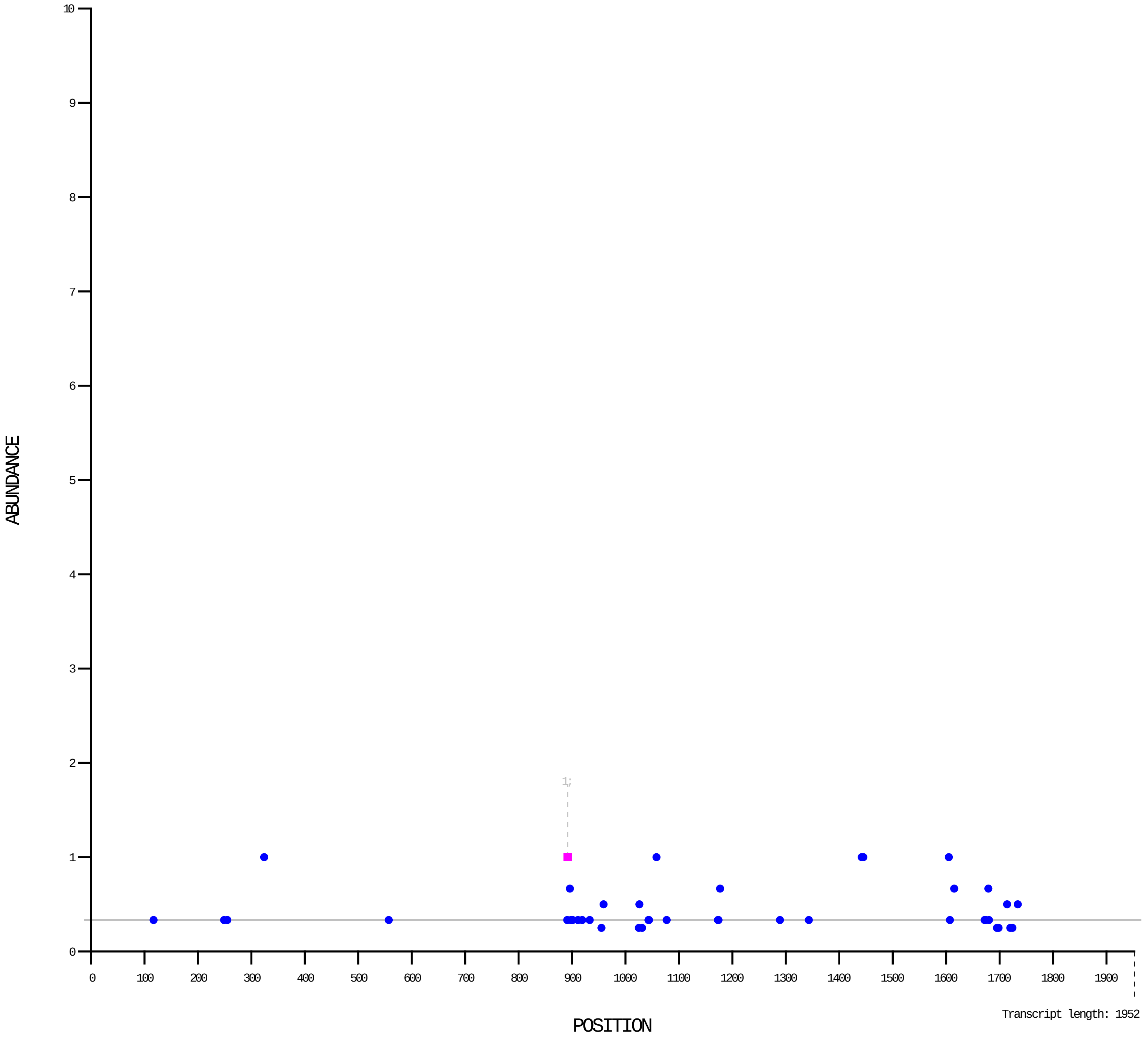

Category: 0 1 2 3 4

Degradome alignment: Median:

```
1  #1  Position:892  Abundance: 1.00(deg) 1(sRNA)
   5'     TTAGATTCACGCACAAACTCG        3'  ID:
          |o| ||||||o| || ||||o            Score: 4.5
   3' GCGAAGTGTAAGTGTGAGTGTGAGTGTGTGAC 5'  p-value: 0.02
```

Cs9g07750.1 gene=Cs9g07750 CDS=208-1602

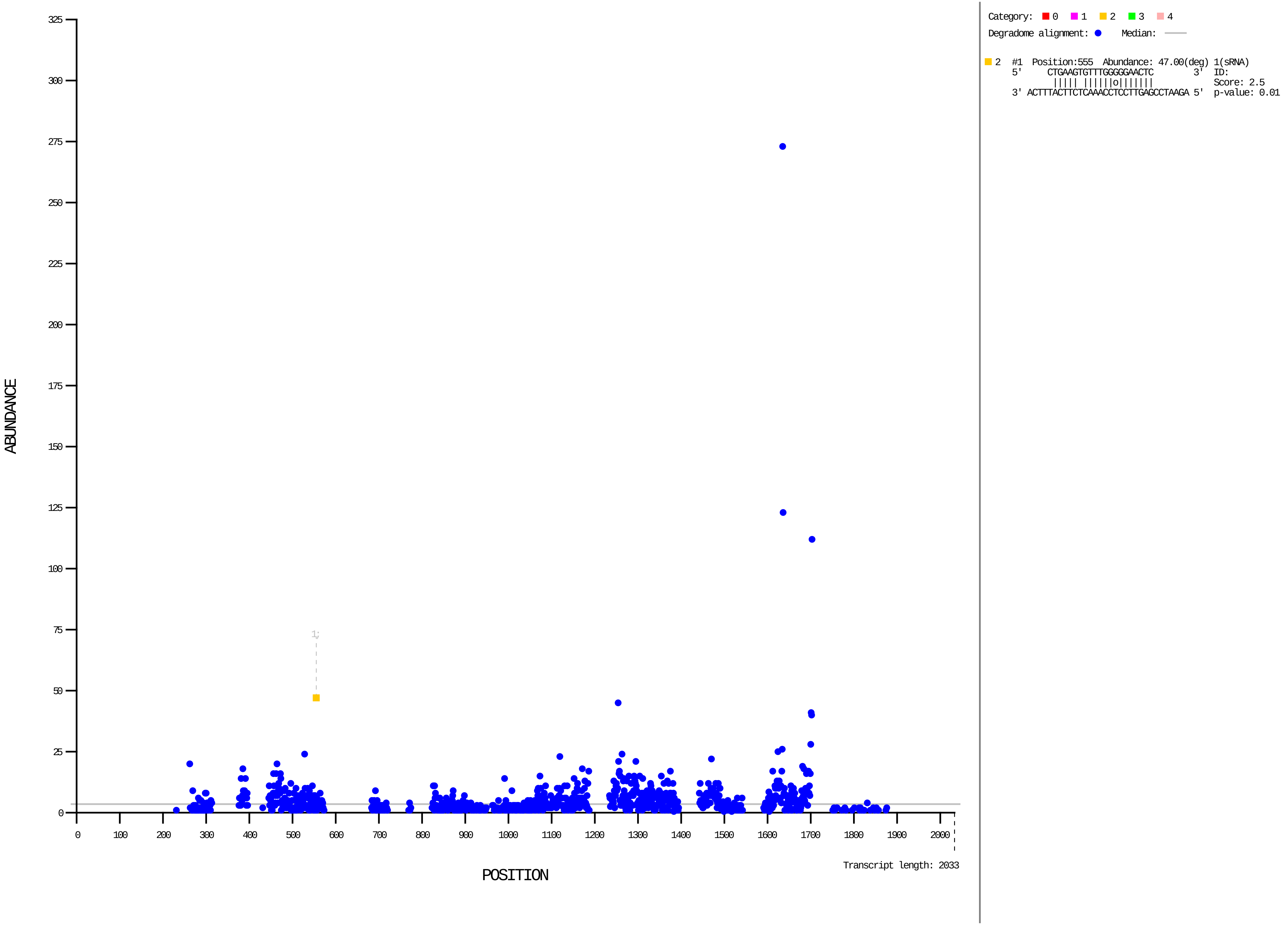

Cs2g19590.1 gene=Cs2g19590 CDS=238-1779

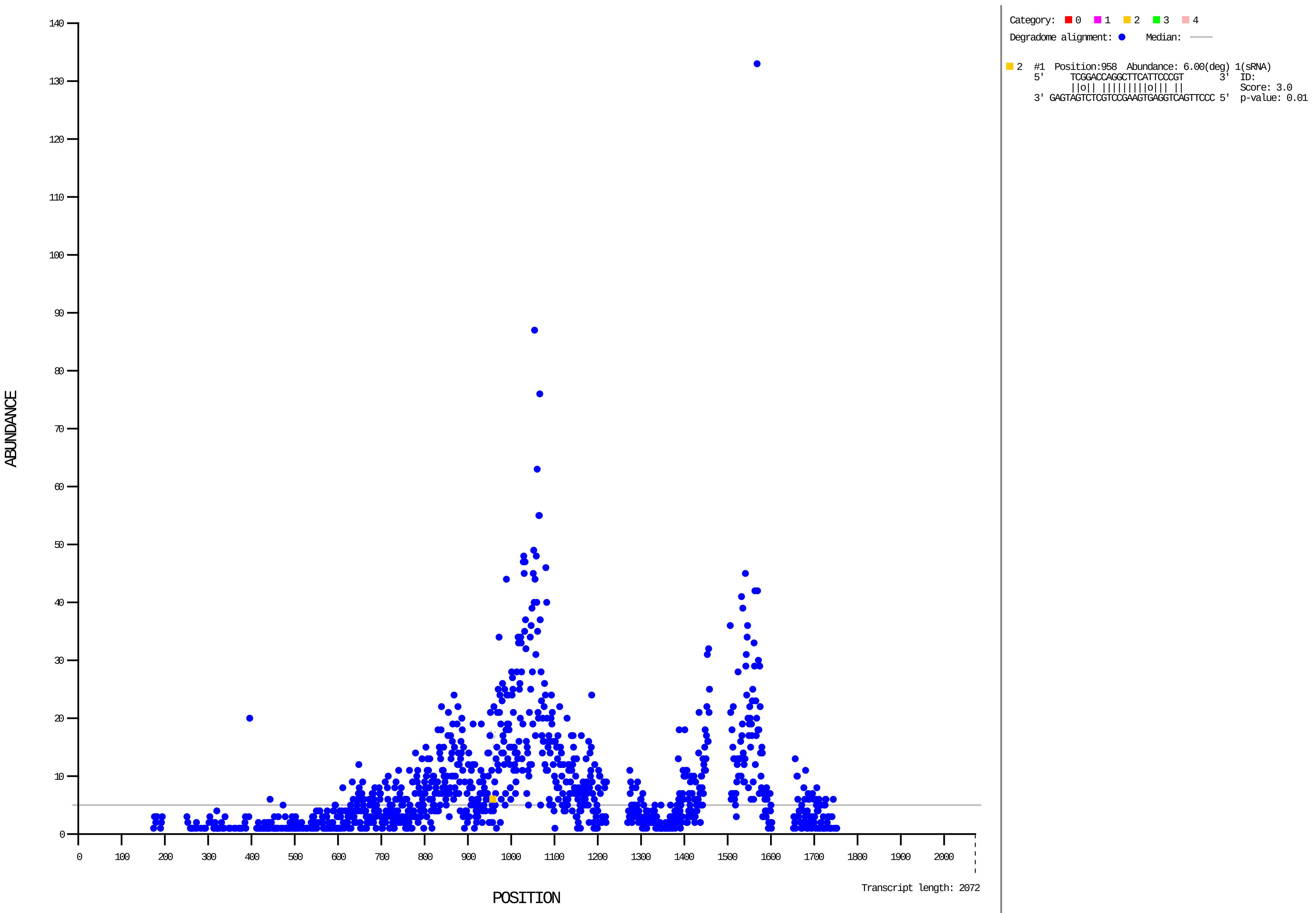

Cs5g32500.1 gene=Cs5g32500 CDS=368-2125

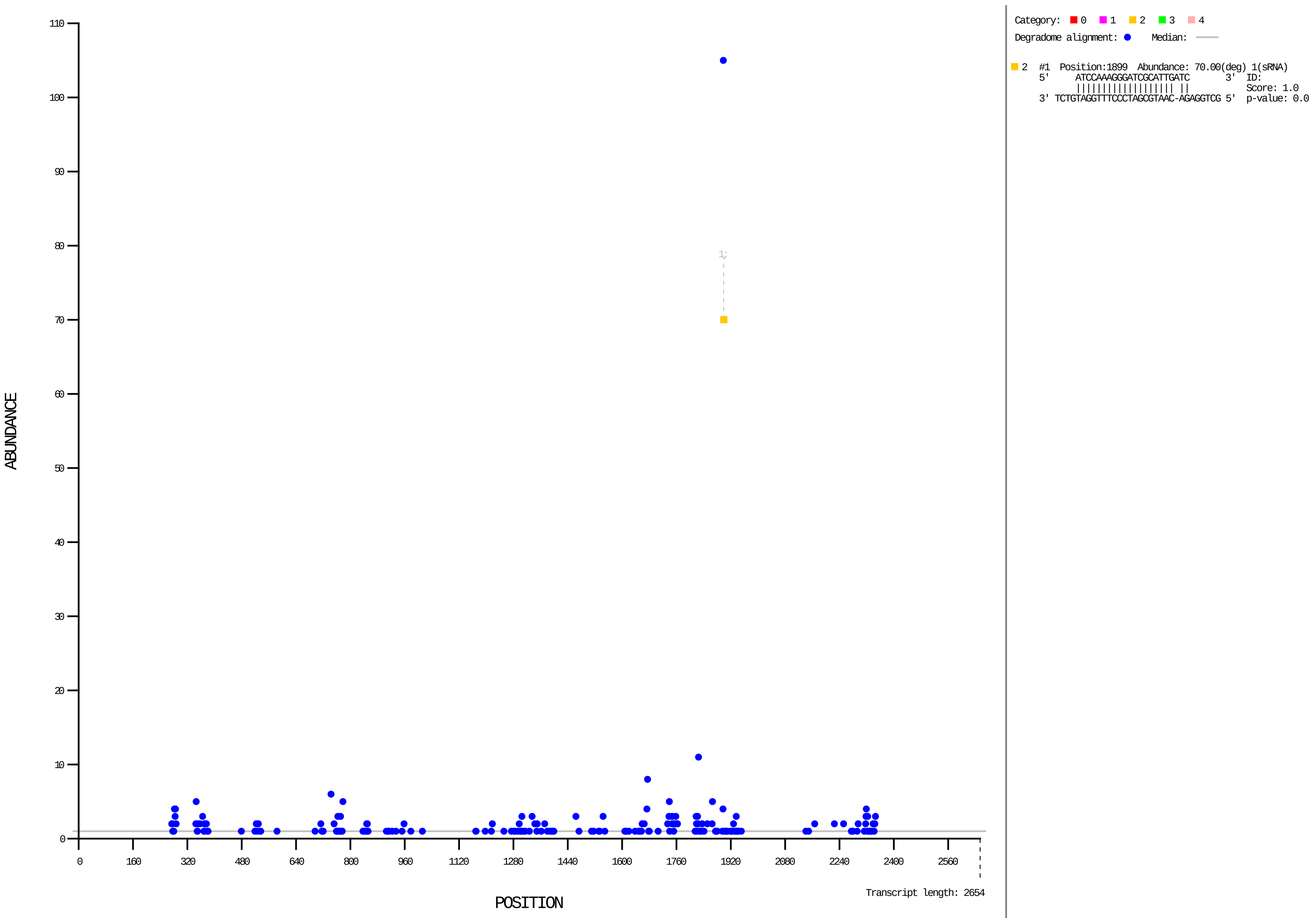

Cs7g02980.1 gene=Cs7g02980 CDS=528-1901

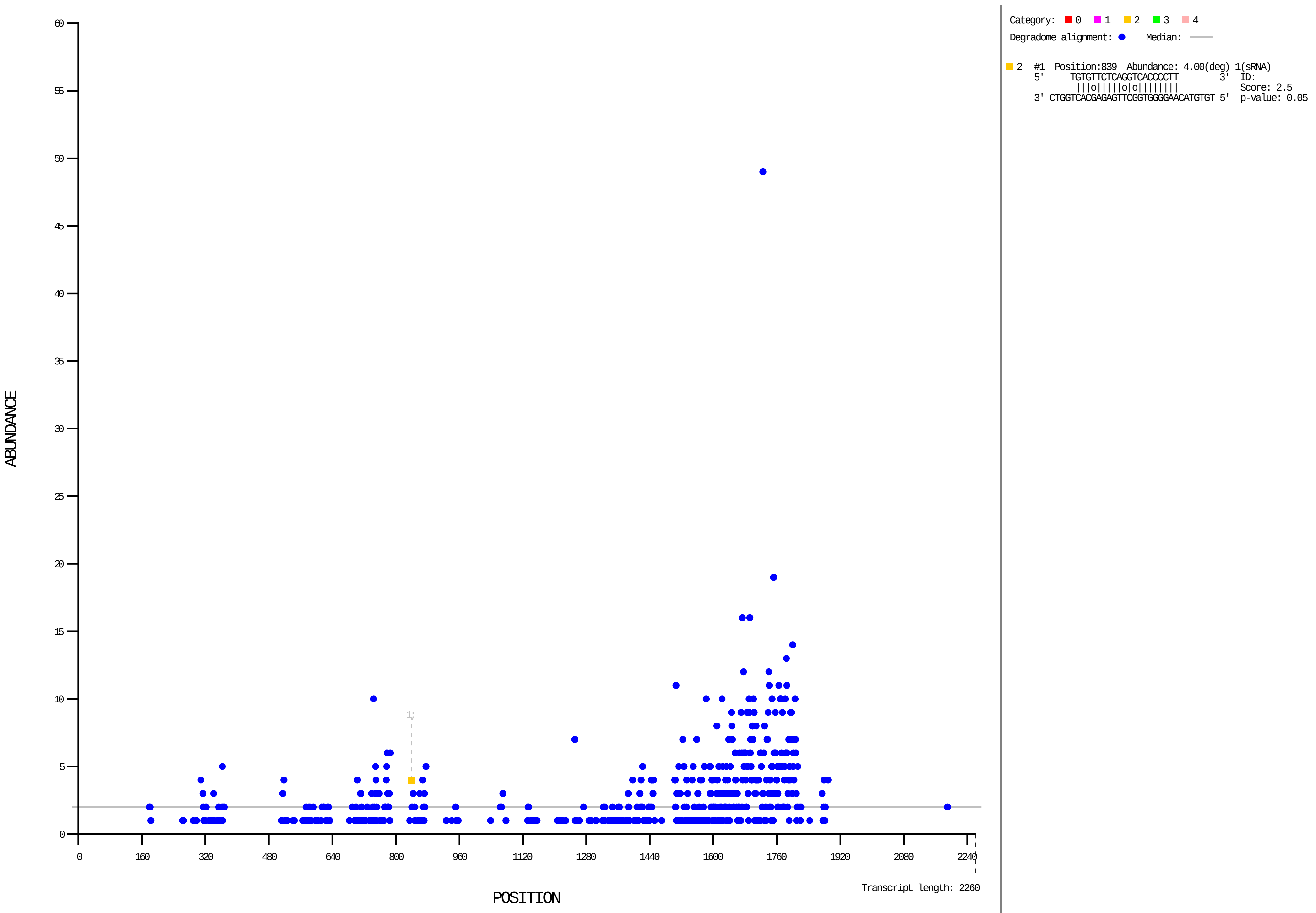

Cs4g18660.3 gene=Cs4g18660 CDS=320-1201

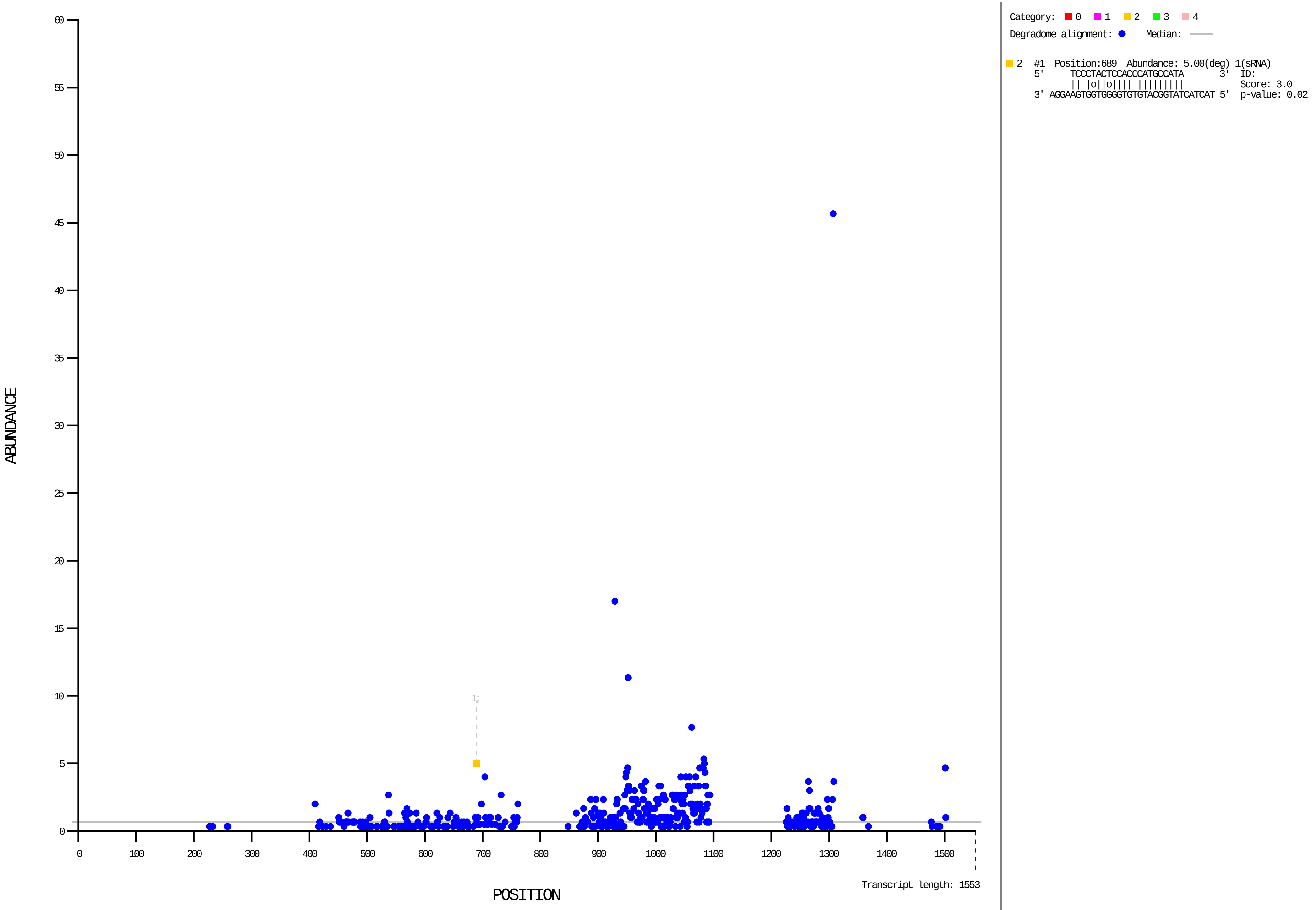

Cs4g18660.2 gene=Cs4g18660 CDS=335-1381

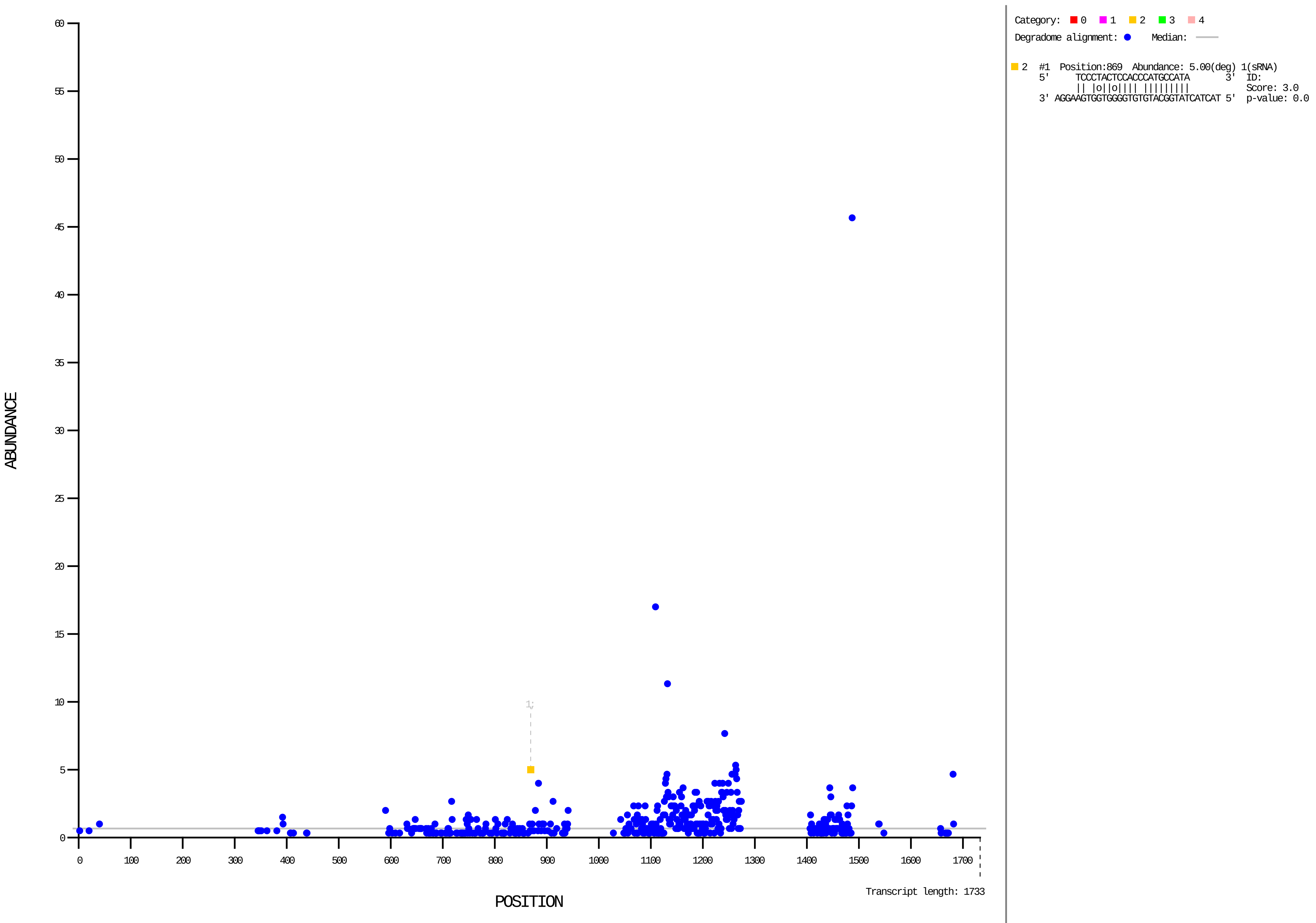

Cs8g16450.1 gene=Cs8g16450 CDS=828-3173

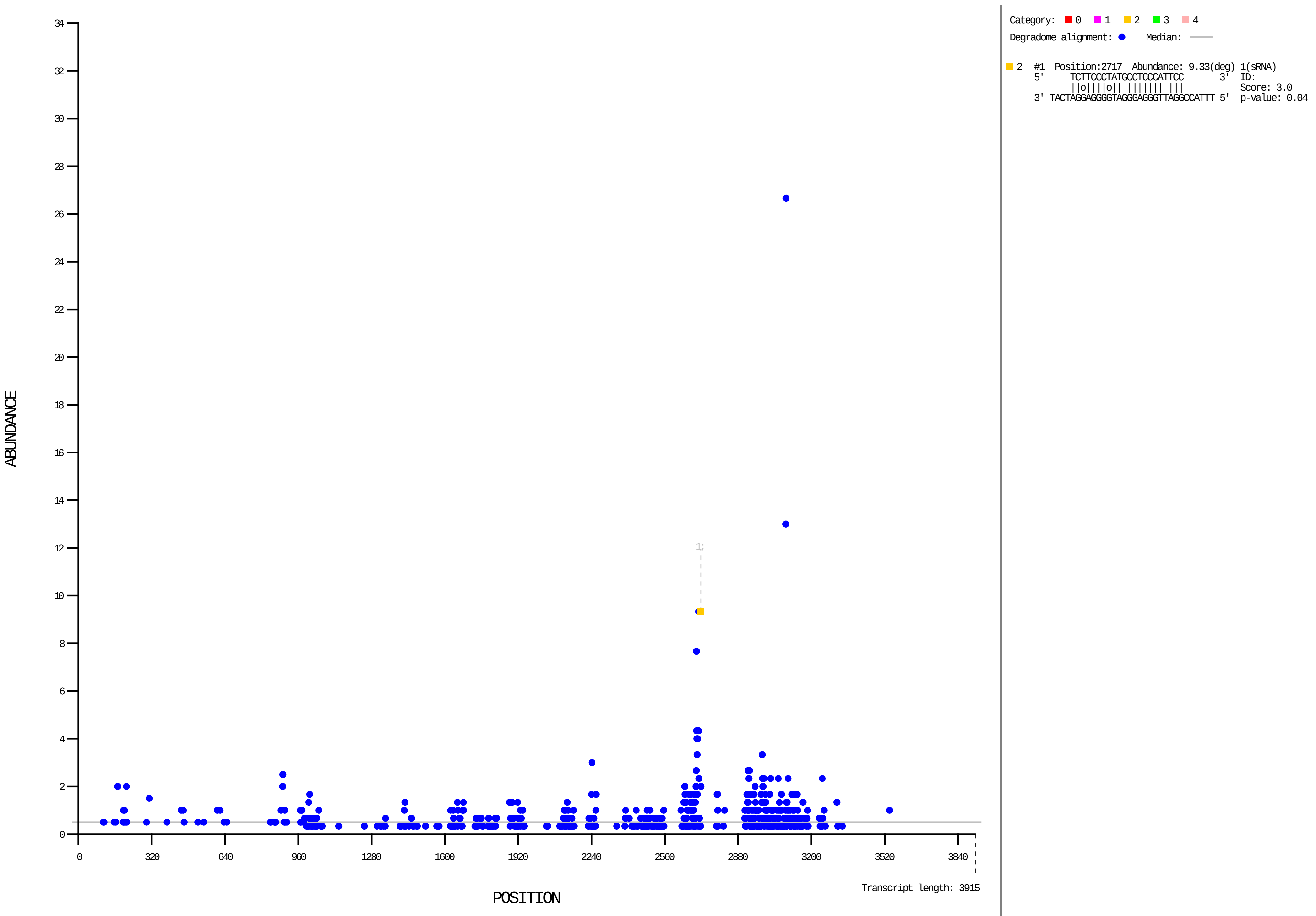

Cs8g16450.3 gene=Cs8g16450 CDS=93-2264

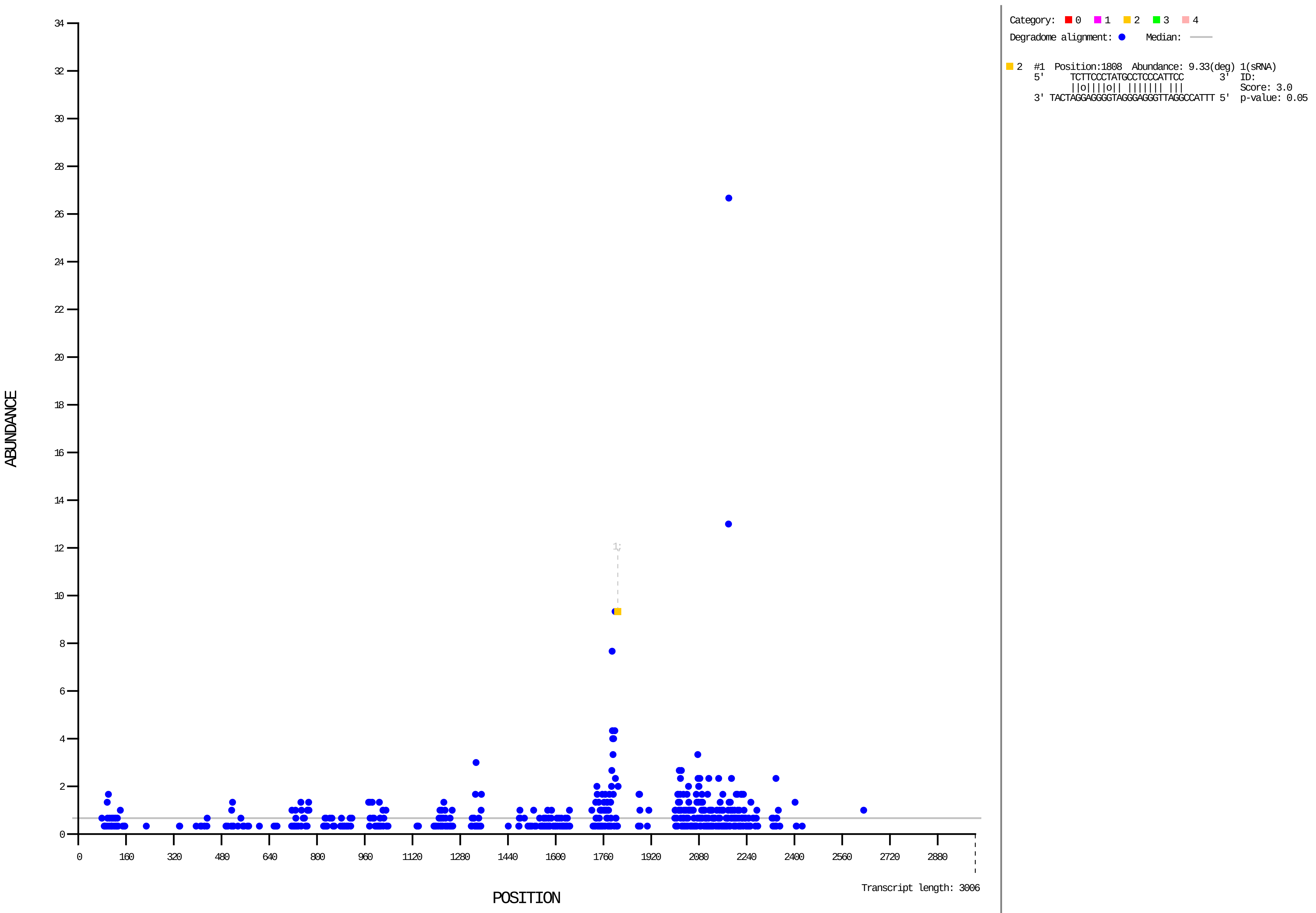

orange1.1t04084.1 gene=orange1.1t04084 CDS=1-6423

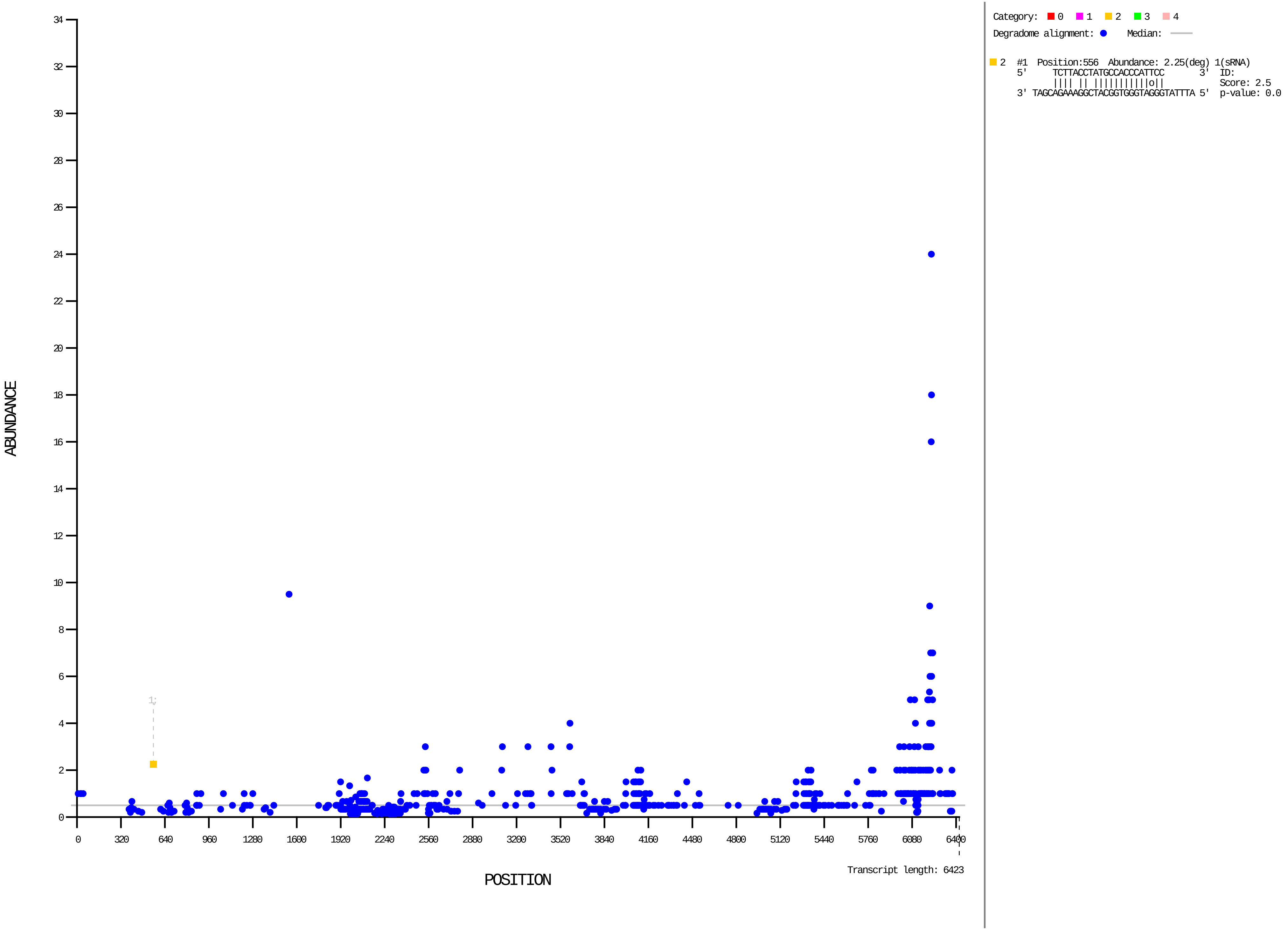

Cs7g11770.2 gene=Cs7g11770 CDS=381-1778

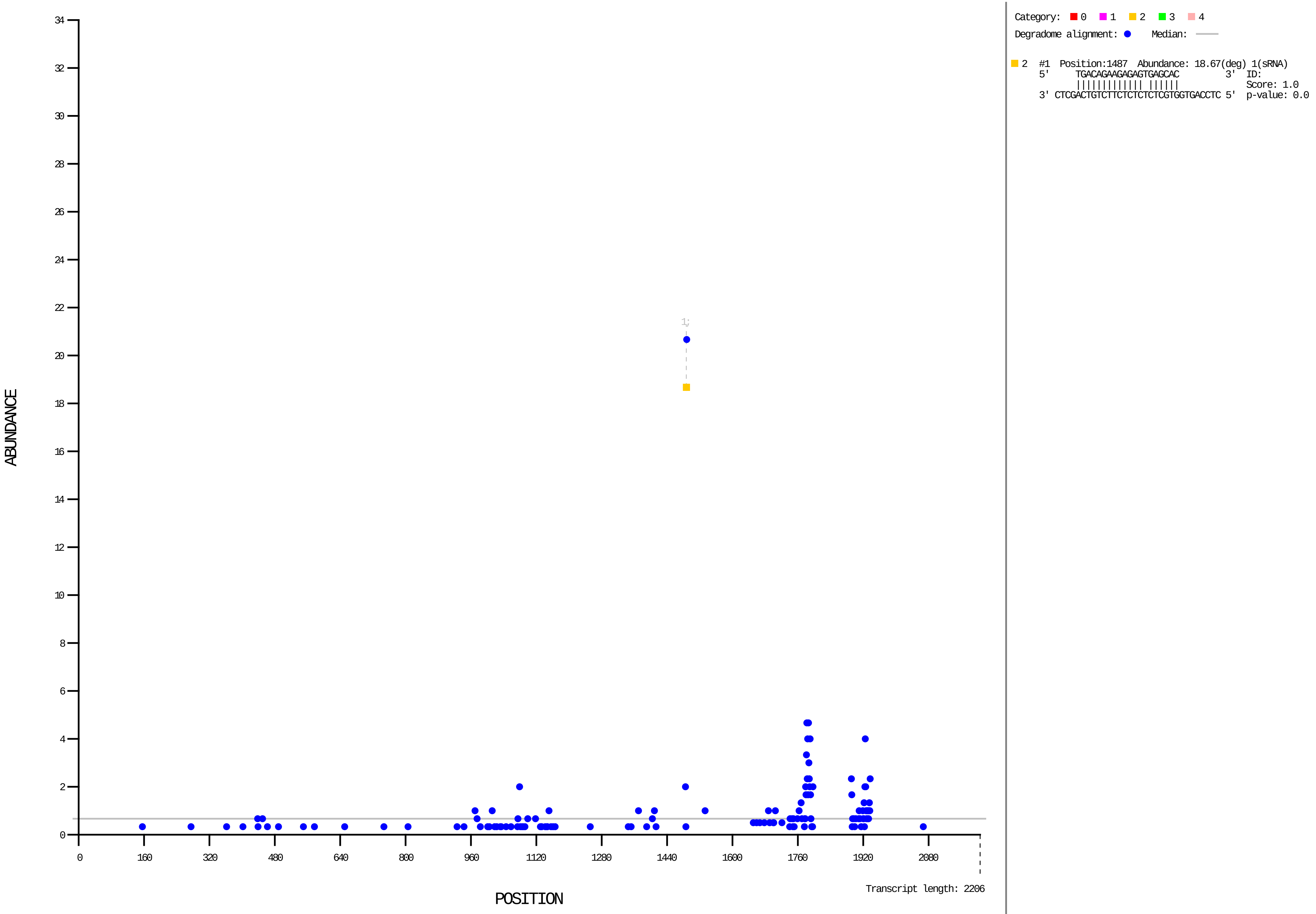

Cs7g11770.1 gene=Cs7g11770 CDS=381-1607

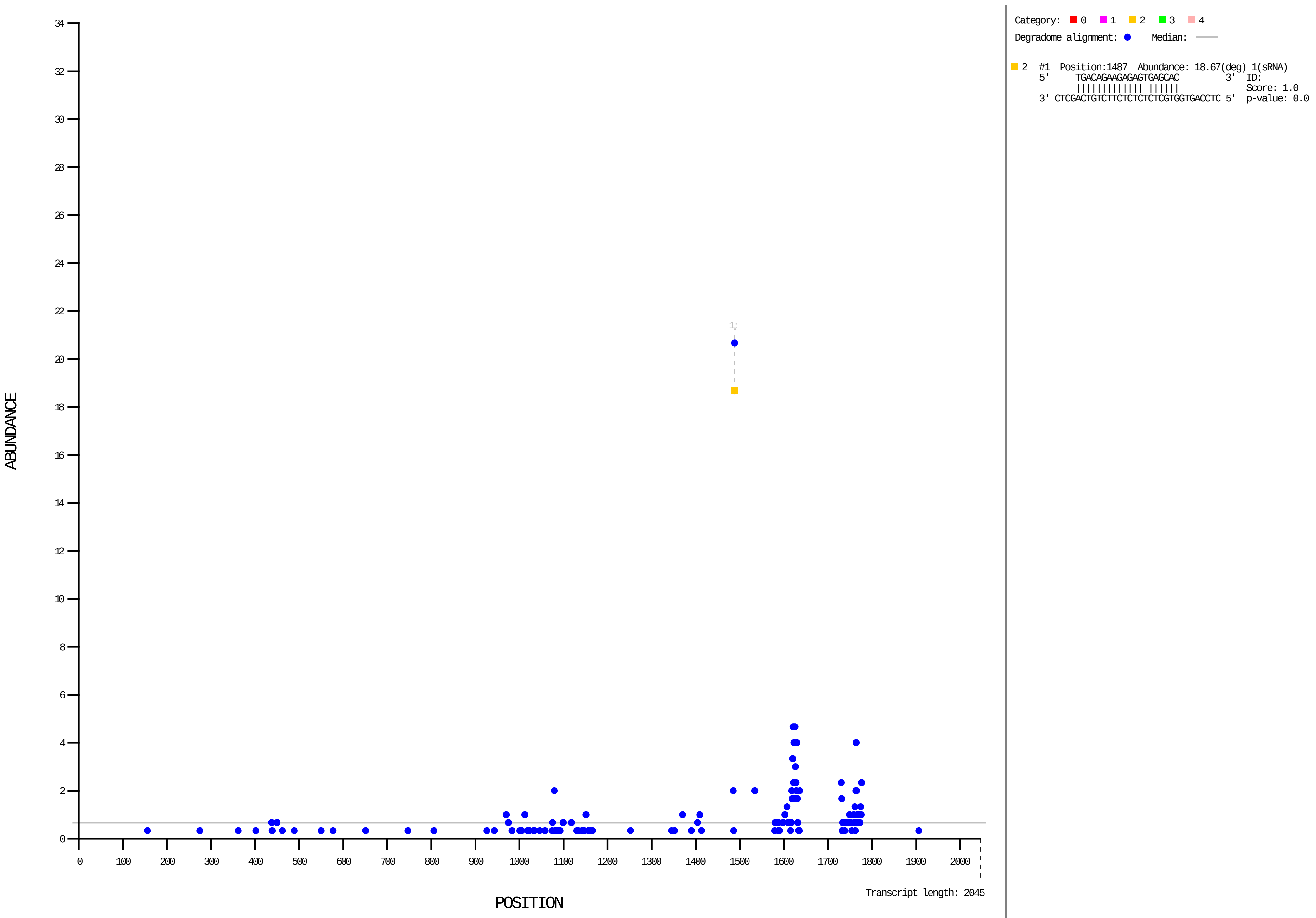

Cs7g11770.3 gene=Cs7g11770 CDS=381-1901

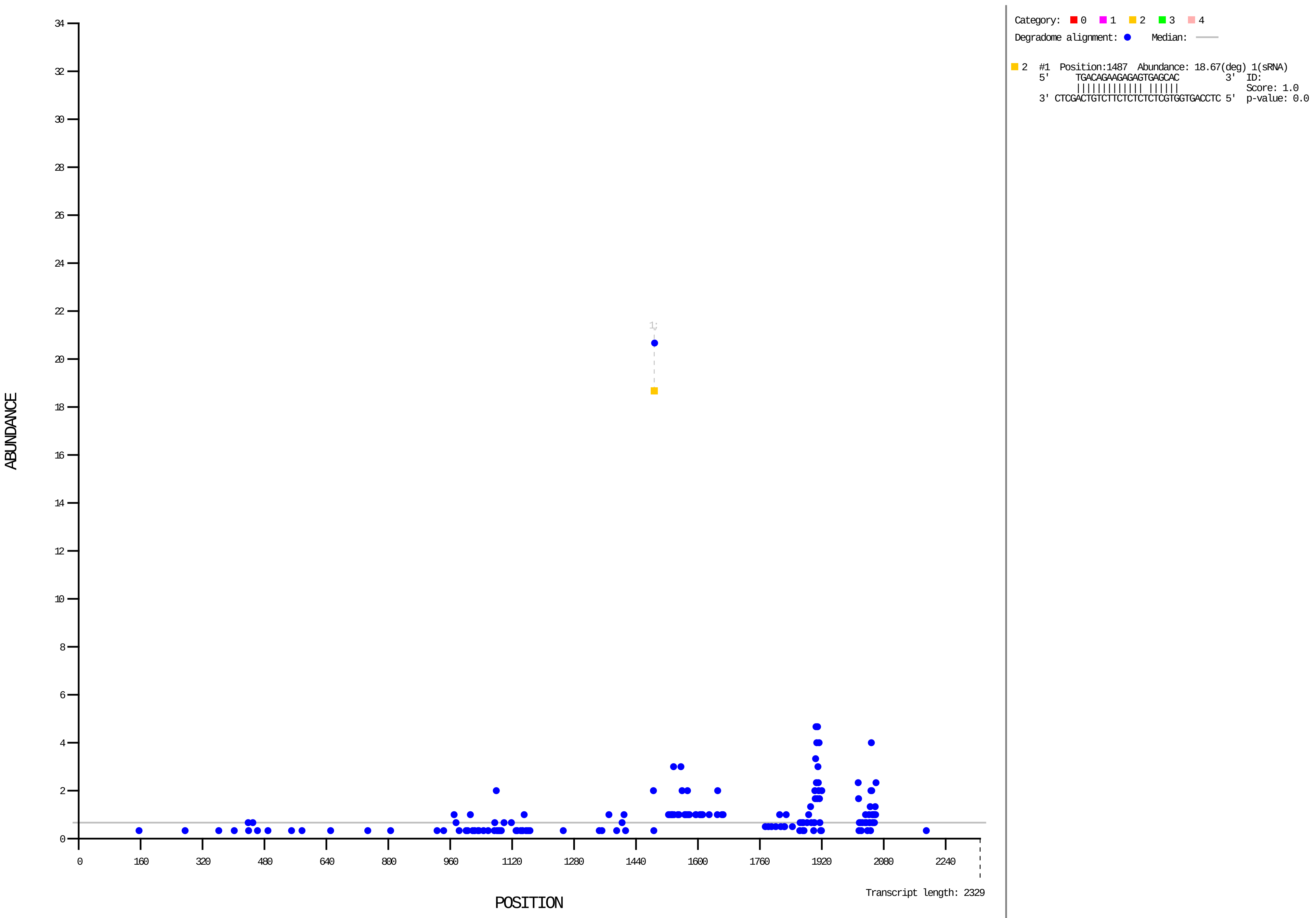

Cs7g10830.2 gene=Cs7g10830 CDS=464-1906

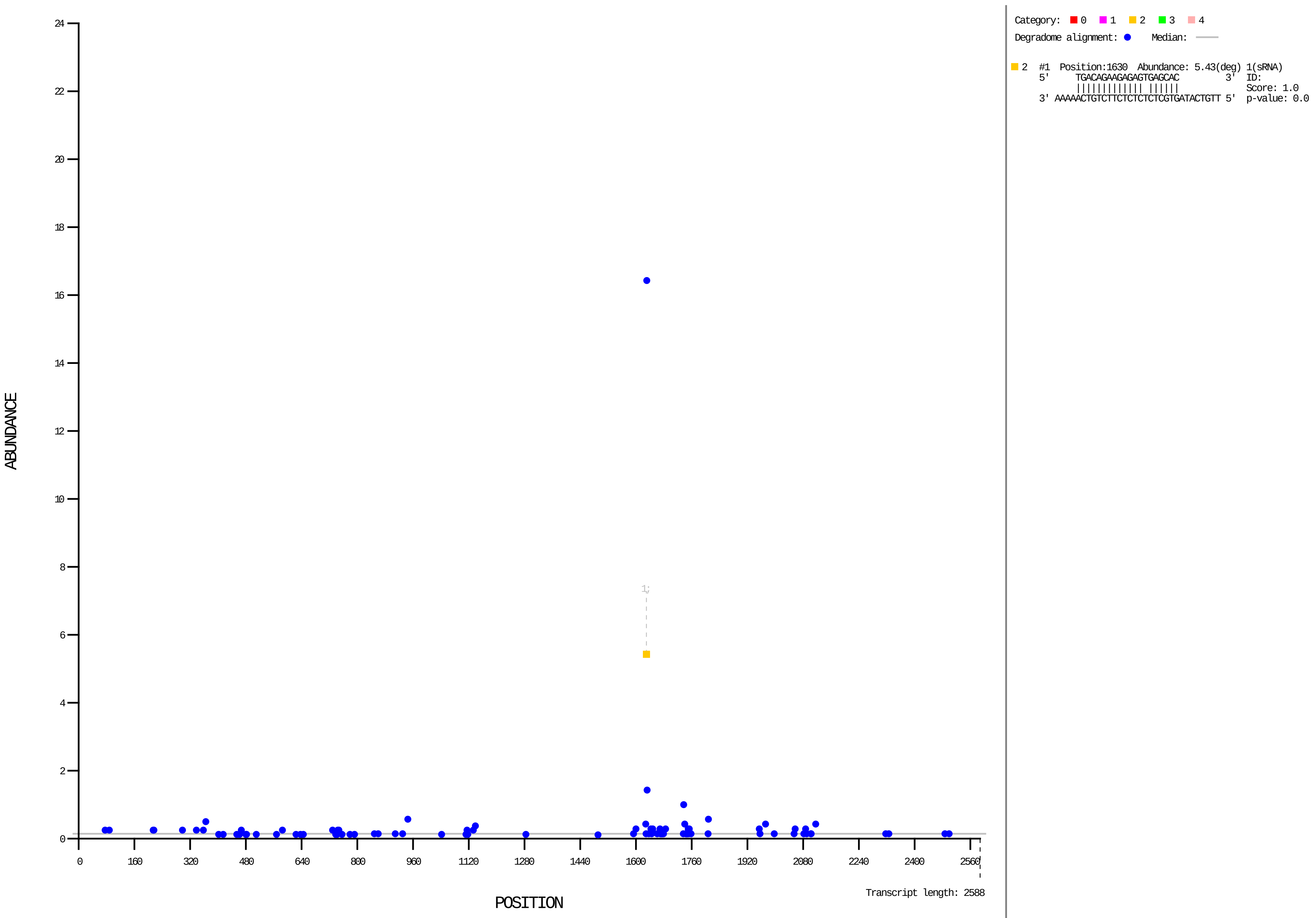

Cs7g10830.6 gene=Cs7g10830 CDS=195-1637

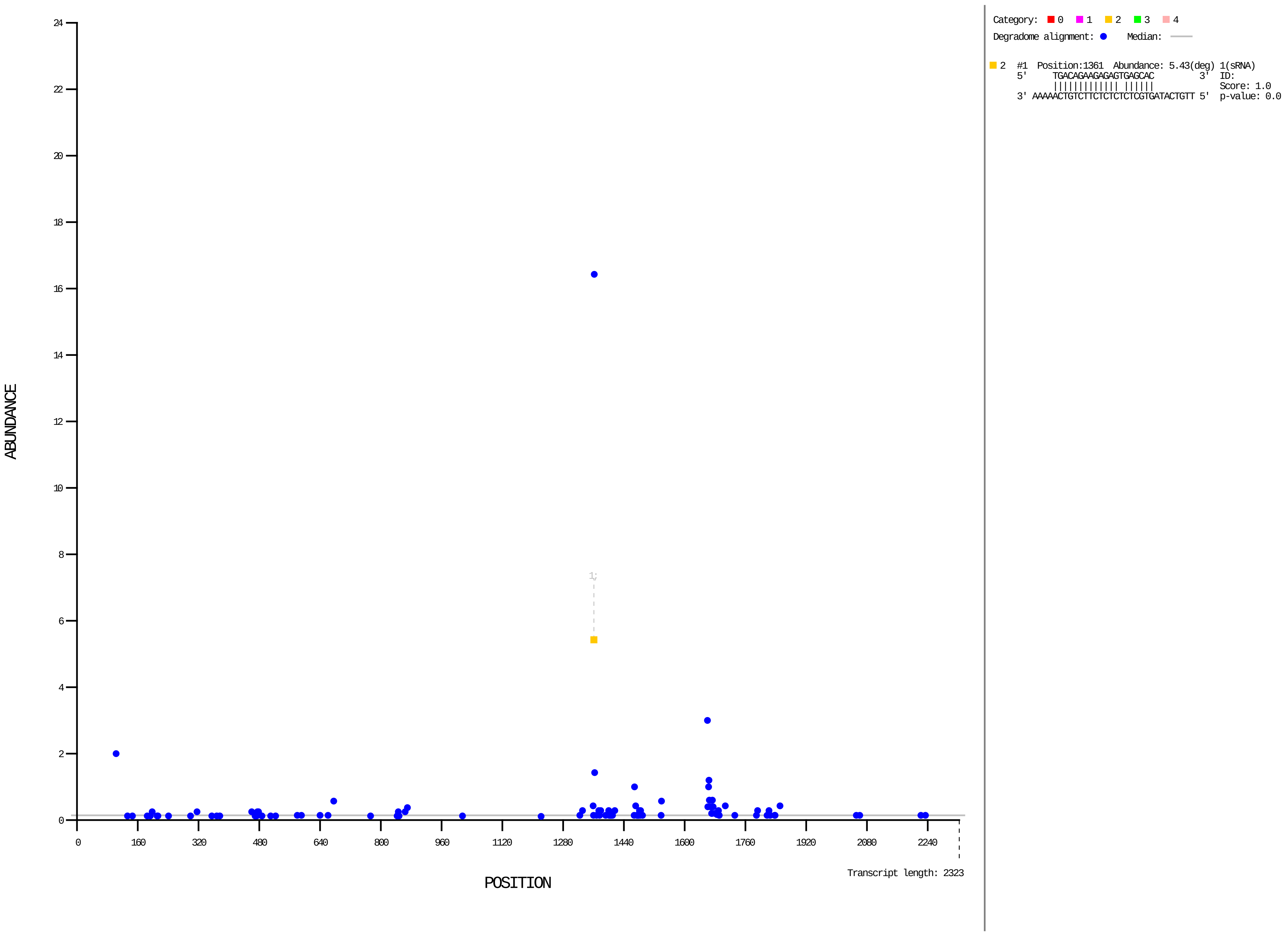

Cs7g10830.1 gene=Cs7g10830 CDS=109-1458

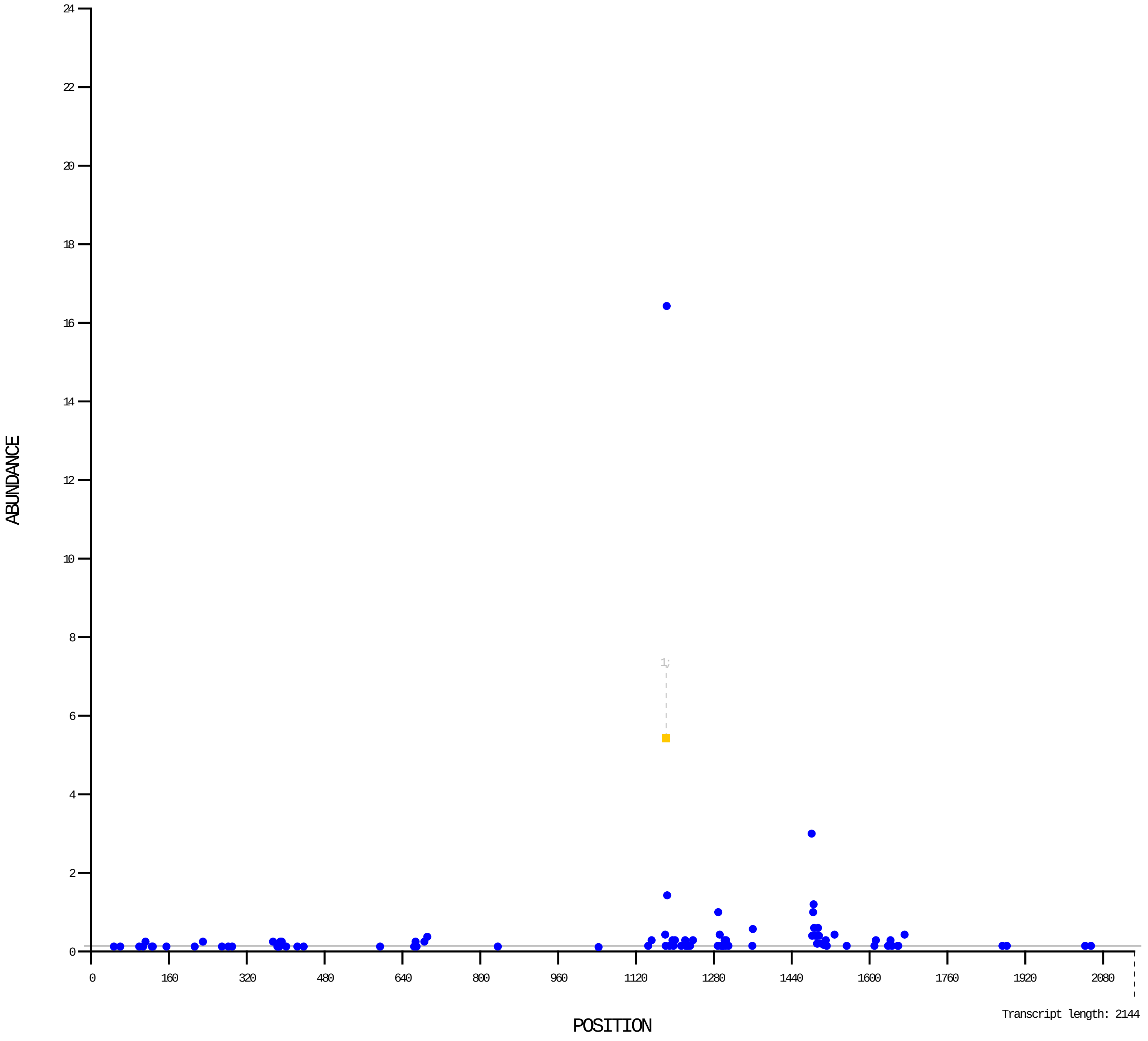

Category: 0 1 2 3 4
Degradome alignment: Median:

2 #1 Position:1182 Abundance: 5.43(deg) 1(sRNA)
5' TGACAGAAGAGAGTGAGCAC 3' ID:
||||||||||||| |||||| Score: 1.0
3' AAAAACTGTCTTCTCTCTCTCGTGATACTGTT 5' p-value: 0.0

Cs7g10830.4 gene=Cs7g10830 CDS=187-1629

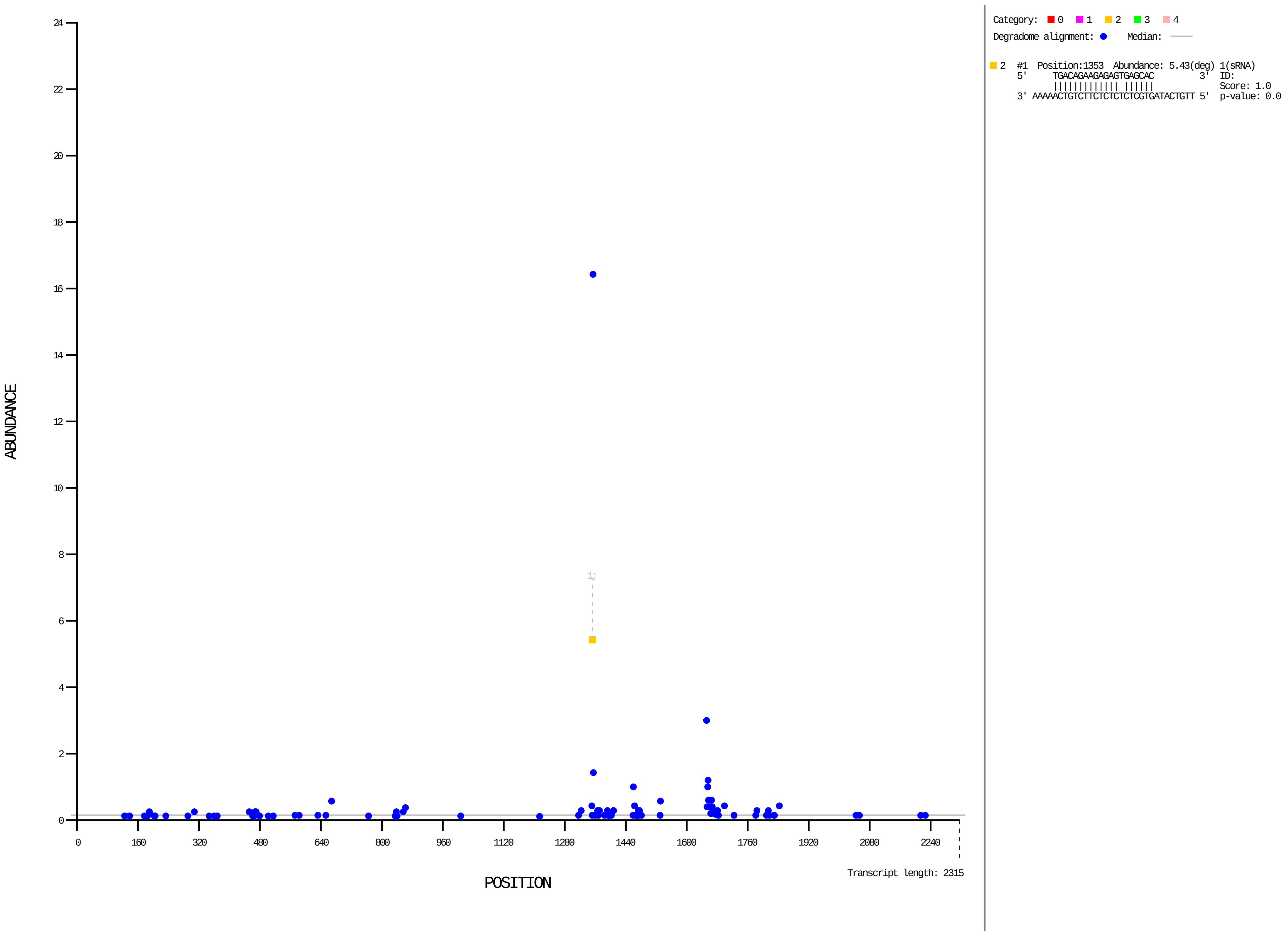

Cs7g10830.5 gene=Cs7g10830 CDS=464-1906

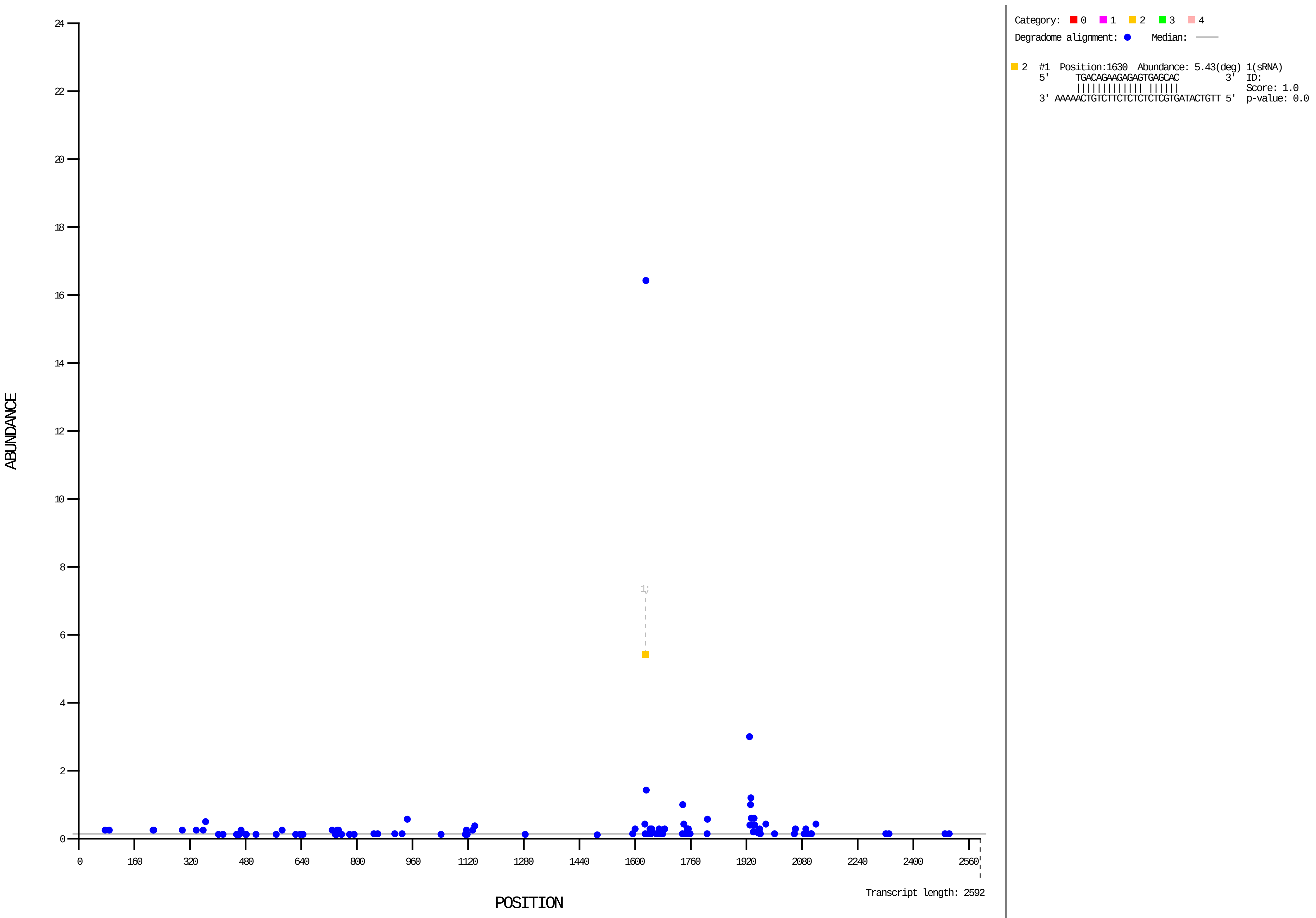

Cs7g10830.3 gene=Cs7g10830 CDS=100-1542

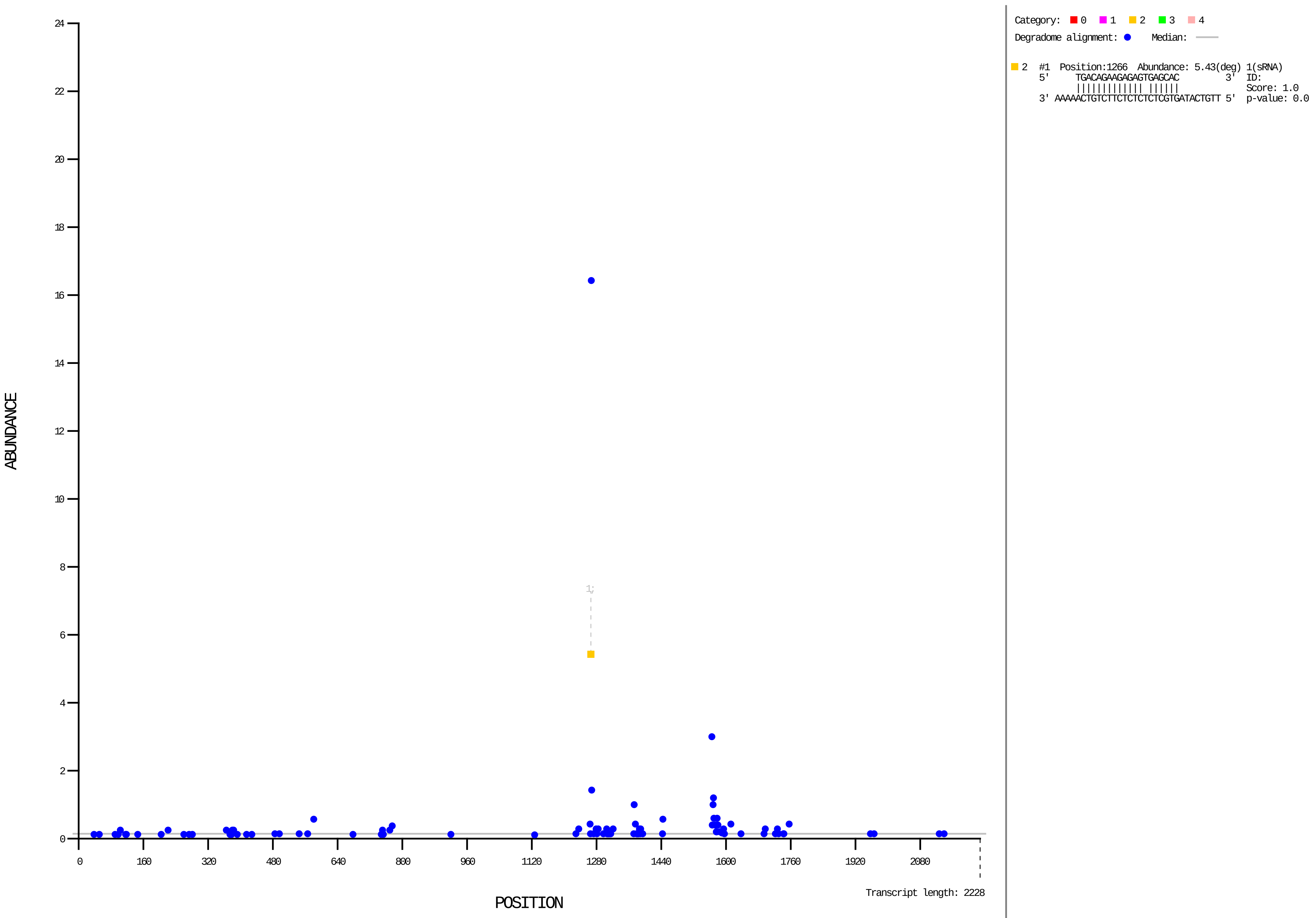

Cs7g10830.7 gene=Cs7g10830 CDS=464-1906

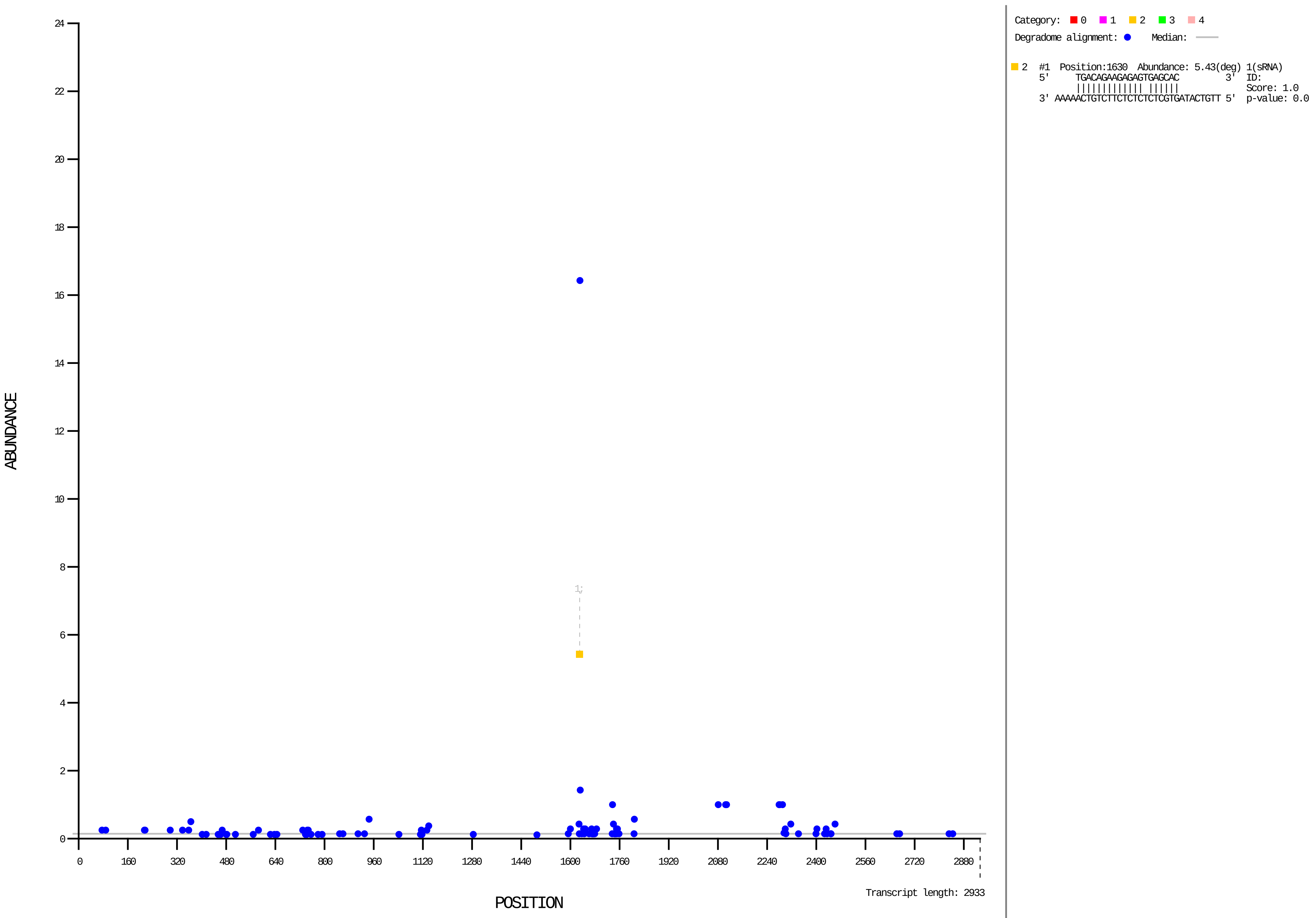

Cs3g12810.1 gene=Cs3g12810 CDS=1-3225

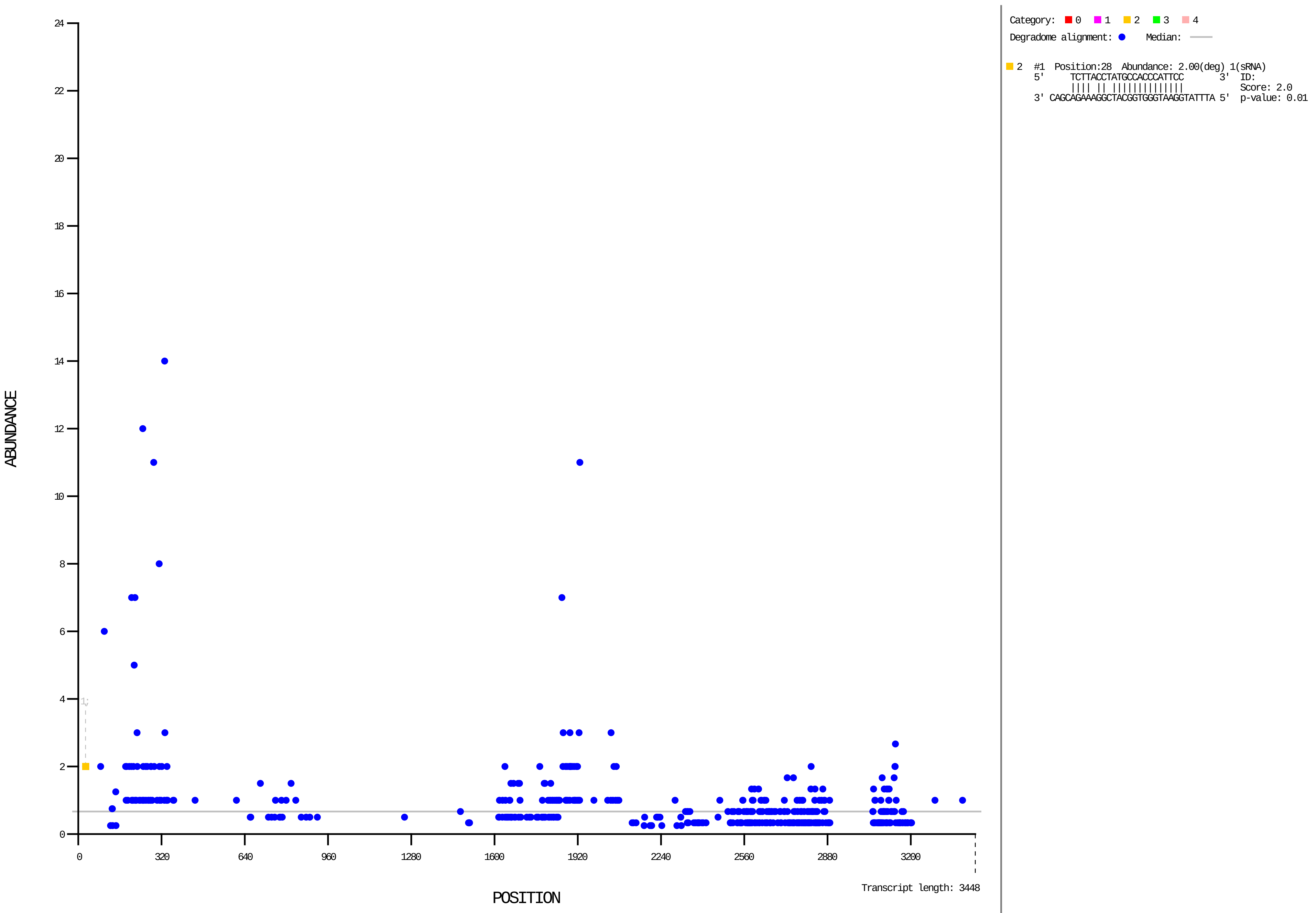

Cs5g19430.1 gene=Cs5g19430 CDS=1-3363

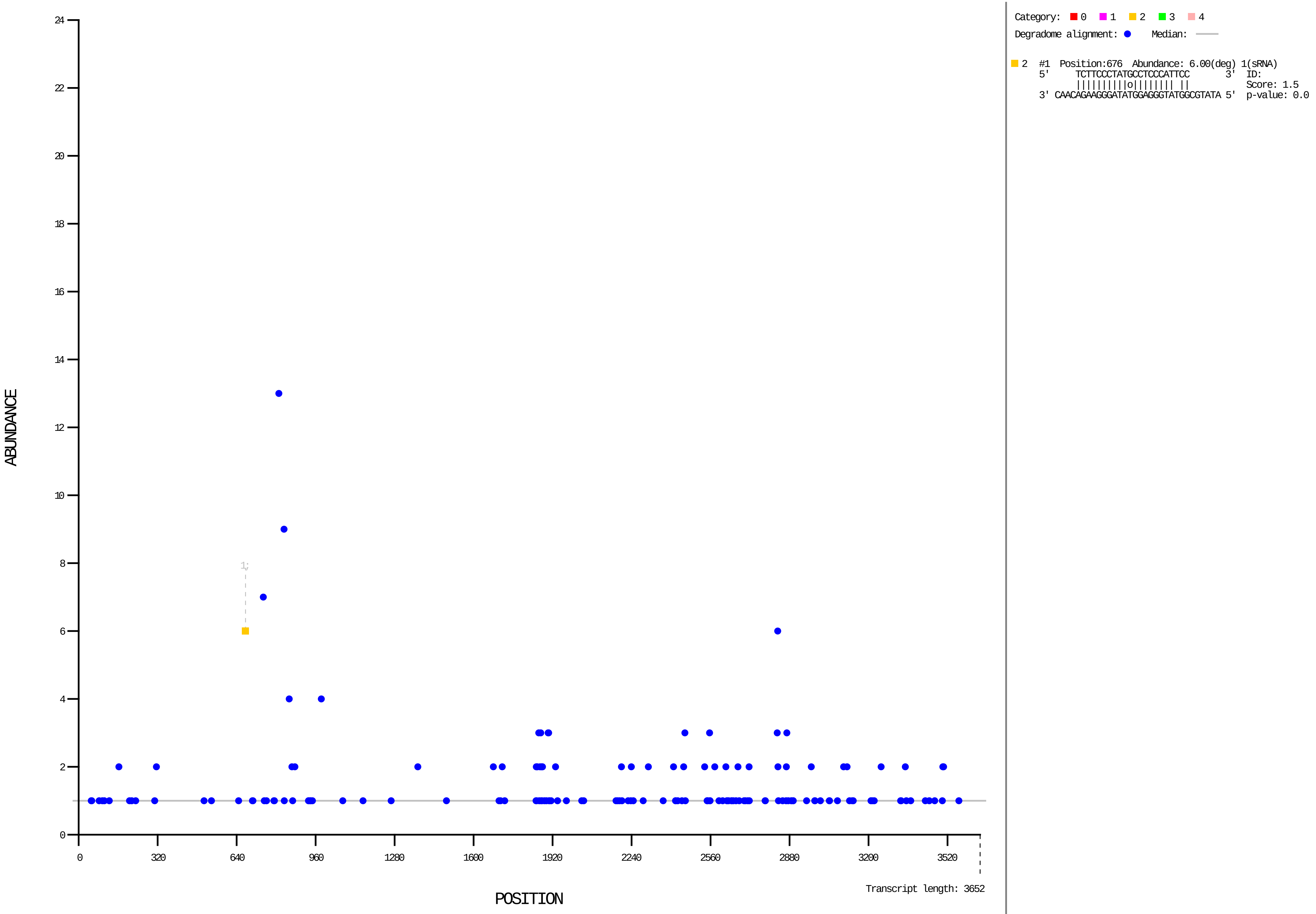

Cs4g04300.3 gene=Cs4g04300 CDS=201-2867

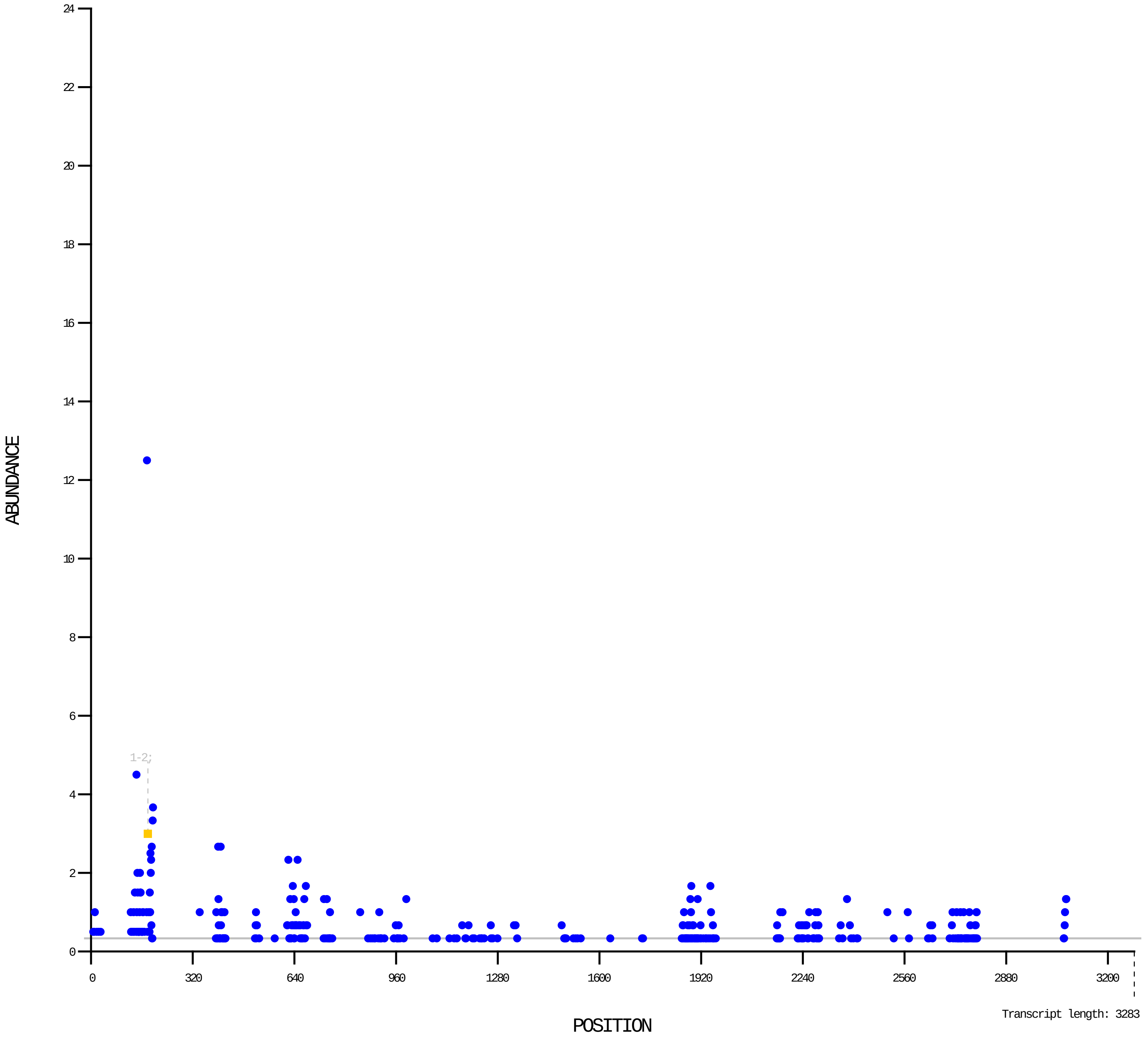


```
Category: ■ 0  ■ 1  ■ 2  ■ 3  ■ 4
Degradome alignment: ●    Median: ——

■ 2  #1  Position:179  Abundance: 3.00(deg) 1(sRNA)
     5'    TTG-TCGCCGGAGAGATAGCACC        3'  ID:
           o|| |||| |||||||| |||||            Score: 3.5
     3' TTTGACAAGCGTCCTCTCTACCGTGGCAGTTA 5'  p-value: 0.04

■ 2  #2  Position:179  Abundance: 3.00(deg) 1(sRNA)
     5'    TTG-TCGCAGGAGCGTTGGCACC        3'  ID:
           o|| ||||||||| | |||||||            Score: 3.5
     3' TTTGACAAGCGTCCTCTCTACCGTGGCAGTTA 5'  p-value: 0.01
```

Cs4g04300.2 gene=Cs4g04300 CDS=220-3438

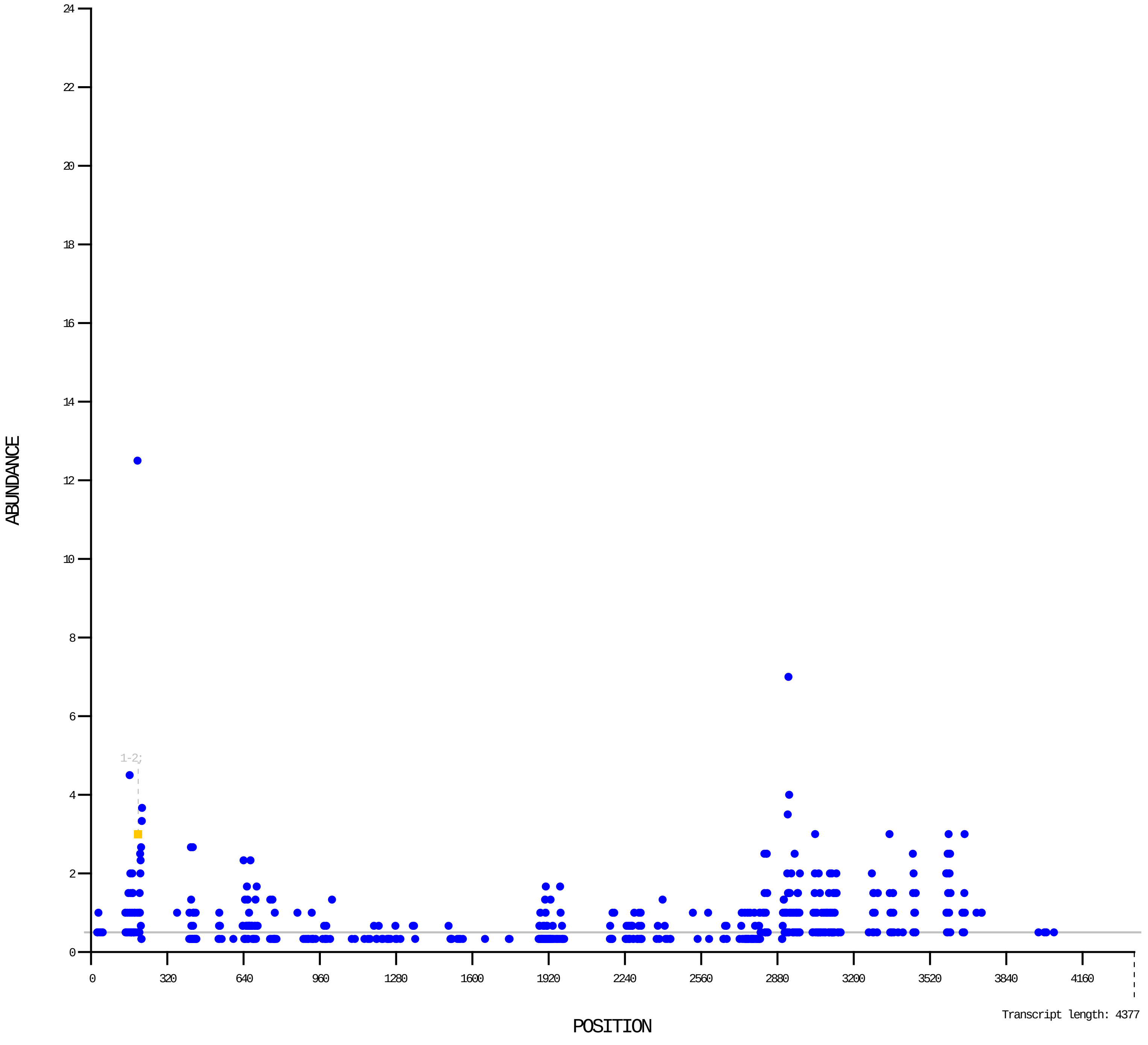

Category: 0 1 2 3 4
Degradome alignment: Median:

```
2  #1  Position:198  Abundance: 3.00(deg) 1(sRNA)
   5'    TTG-TCGCCGGAGAGATAGCACC       3'  ID:
         o|| |||| |||||||| |||||           Score: 3.5
   3' TTTGACAAGCGTCCTCTCTACCGTGGCAGTTA 5'  p-value: 0.02

2  #2  Position:198  Abundance: 3.00(deg) 1(sRNA)
   5'    TTG-TCGCAGGAGCGTTGGCACC       3'  ID:
         o|| ||||||||| | |||||||           Score: 3.5
   3' TTTGACAAGCGTCCTCTCTACCGTGGCAGTTA 5'  p-value: 0.03
```

orange1.1t03059.6 gene=orange1.1t03059 CDS=285-2258

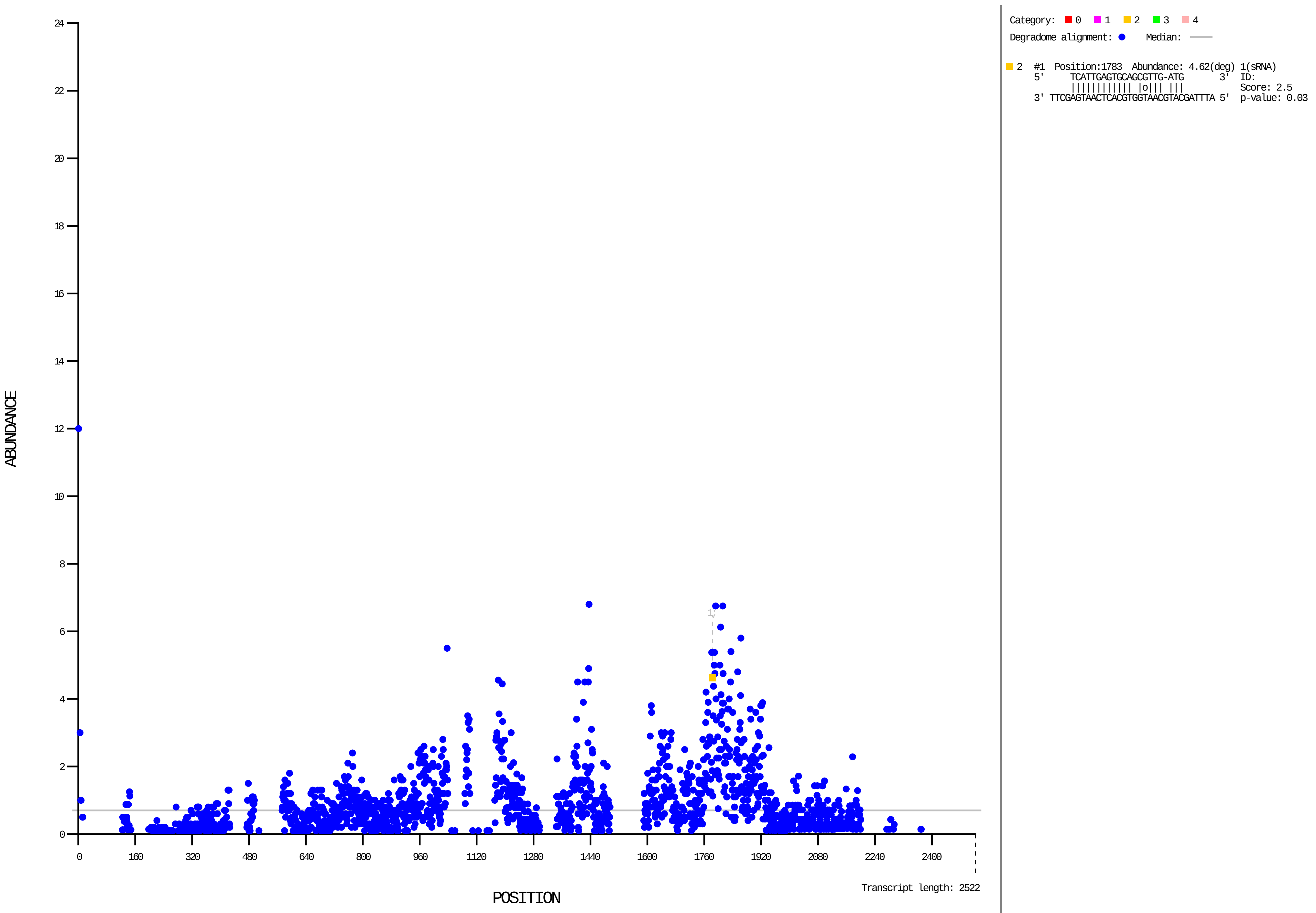

Cs1g08080.2 gene=Cs1g08080 CDS=116-3190

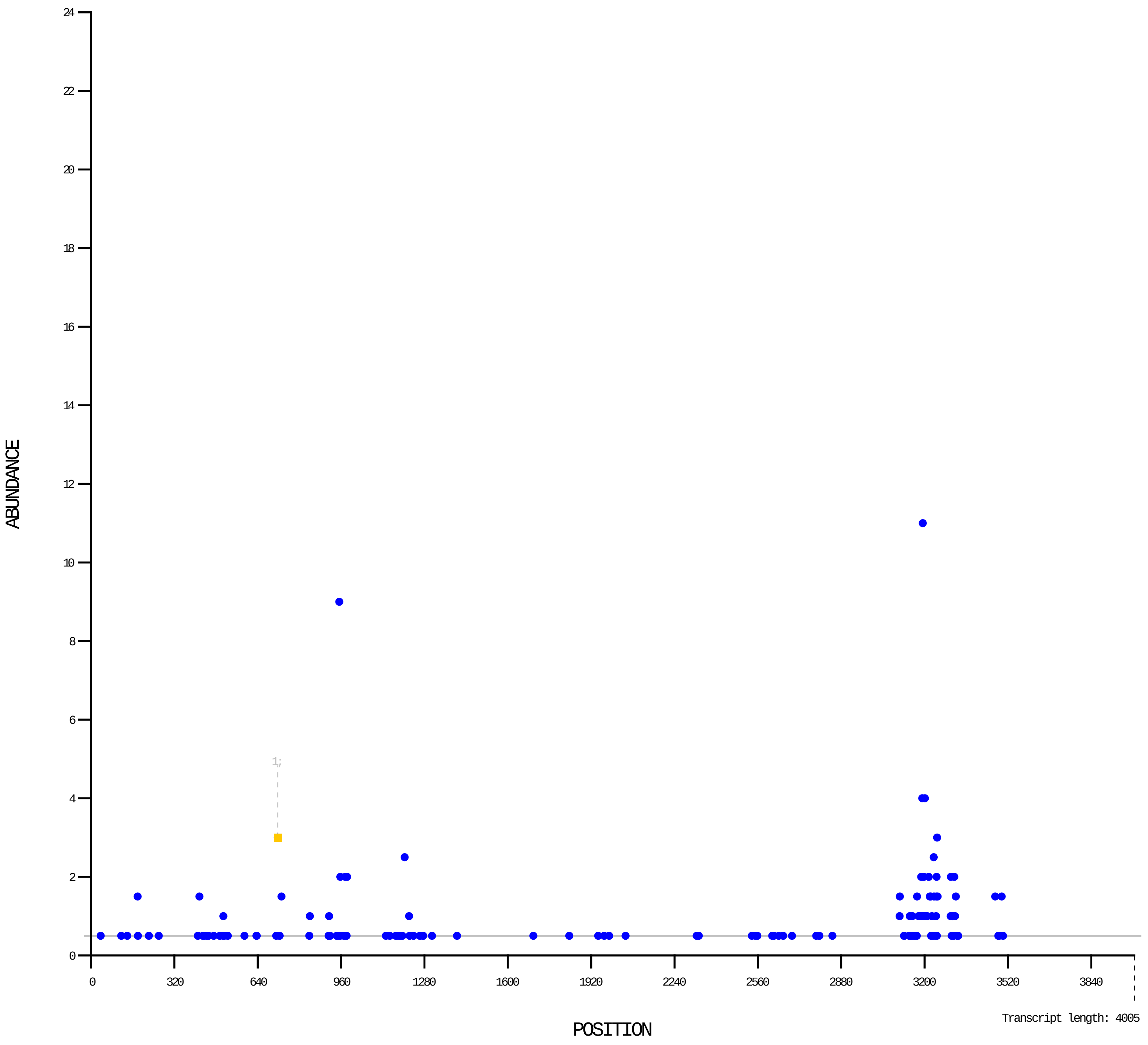

Category: 0 1 2 3 4
Degradome alignment: Median:

2 #1 Position:717 Abundance: 3.00(deg) 1(sRNA)

```
5'     TTCCCTAGTCCCCCTATTCCTA       3'  ID:
       |||||o|o|||||||||| ||o|          Score: 2.5
3' GCAAAAGGGGTTAGGGGGATATGGGTGATATC 5'  p-value: 0.01
```

orange1.1t04079.1 gene=orange1.1t04079 CDS=1-7716

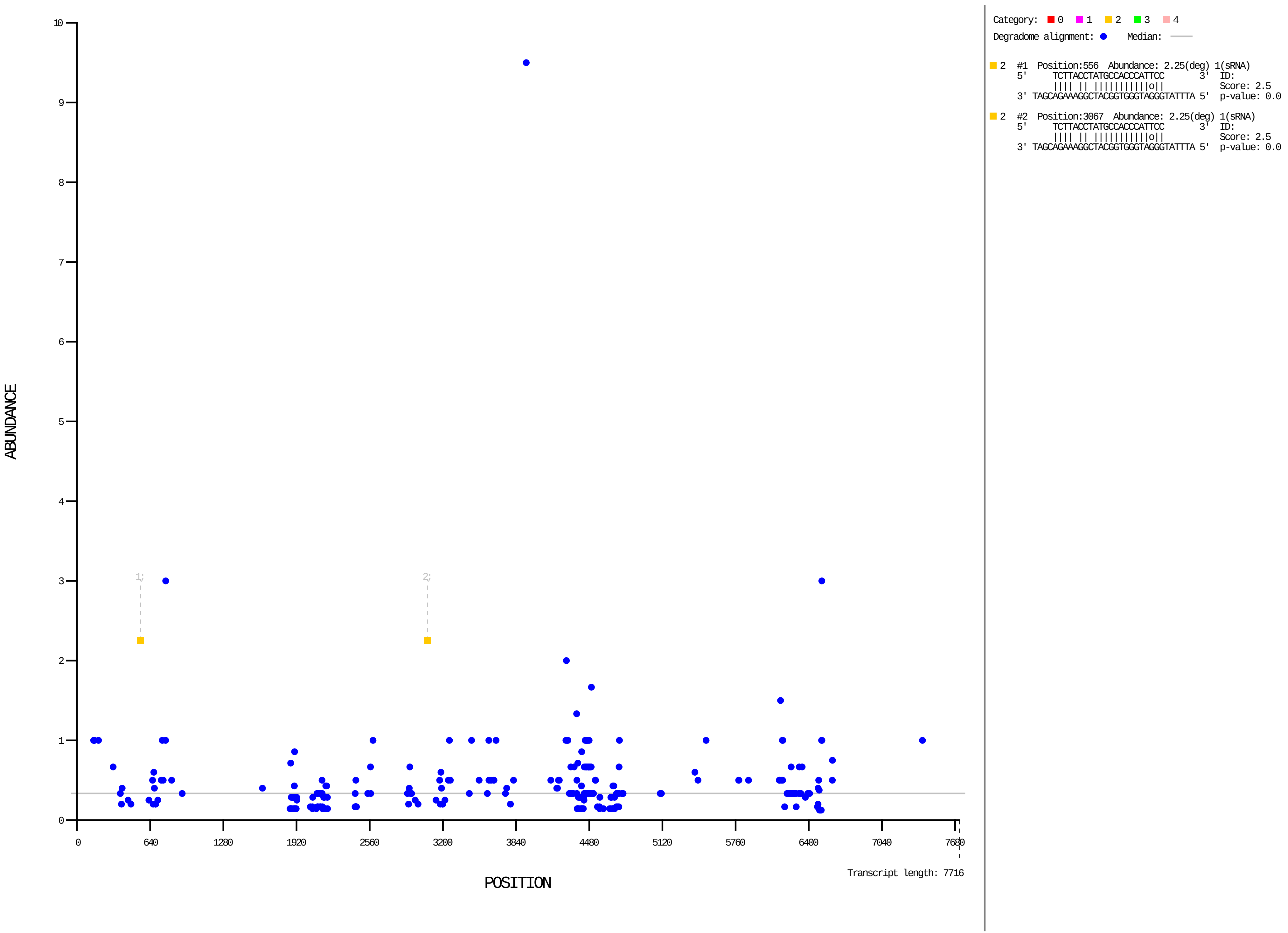

Cs1g08080.1 gene=Cs1g08080 CDS=116-3190

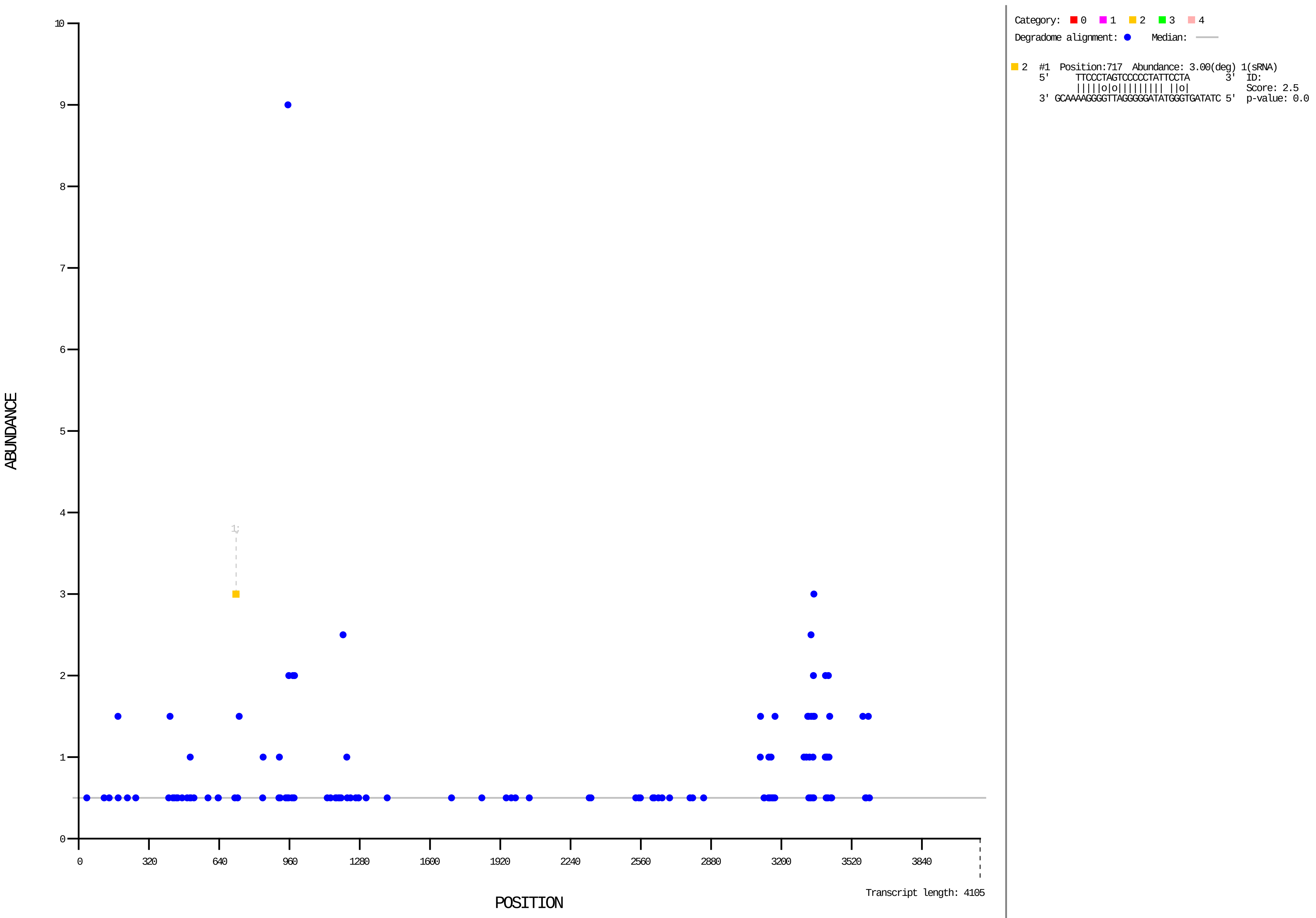

Cs1g19500.7 gene=Cs1g19500 CDS=1299-3272

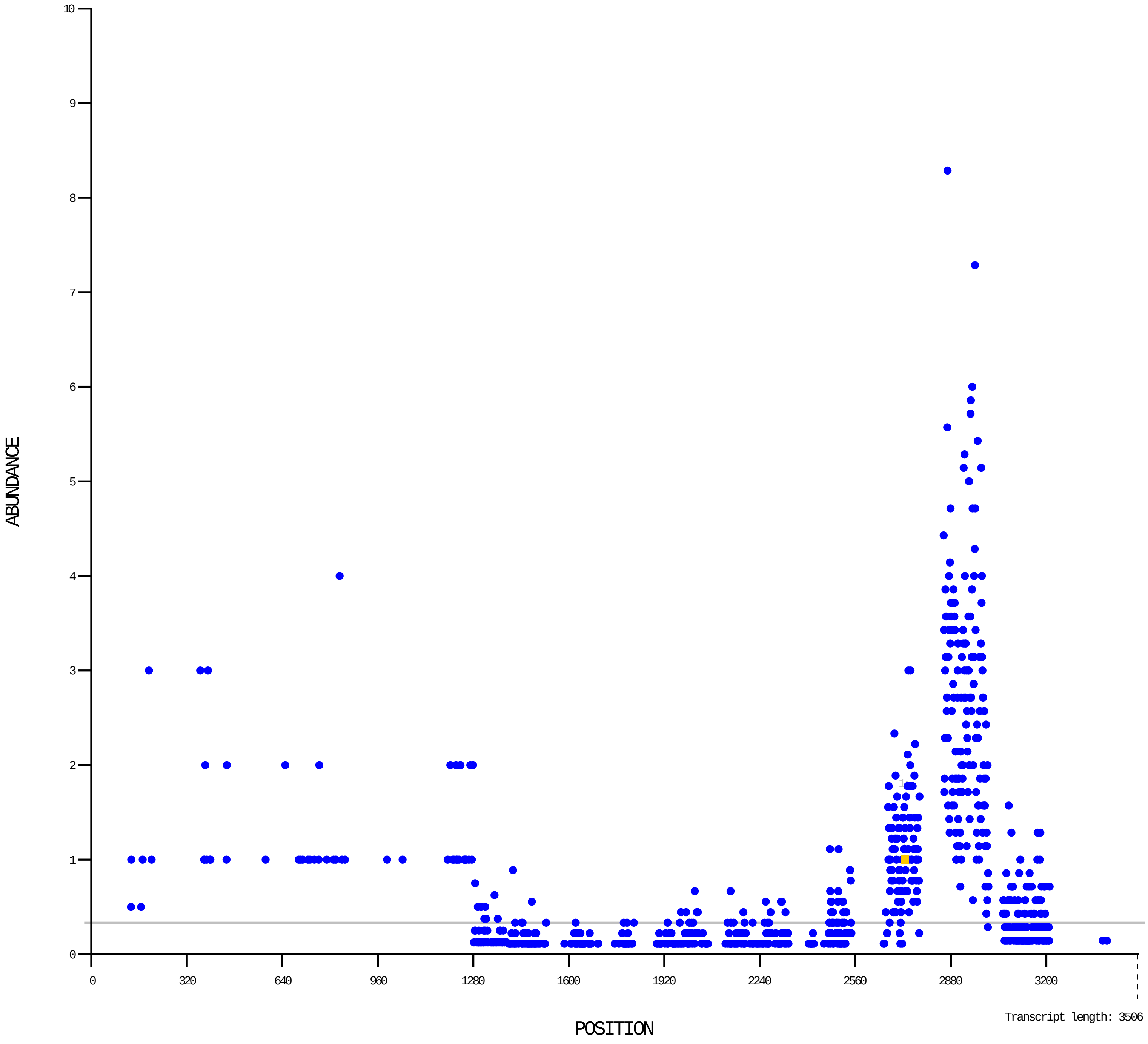

Category: 0 1 2 3 4

Degradome alignment: ● Median: —

```
2  #1  Position:2725  Abundance: 1.00(deg) 1(sRNA)
   5'    TGAGCTGTTTGGCTATTCTCGC       3'  ID:
         ||||||| |||o |||o|||||           Score: 3.0
   3' ATATACTCGAC-AACTAATAGGAGCGAGTAAA 5'  p-value: 0.04
```

Cs1g19500.6 gene=Cs1g19500 CDS=86-2059

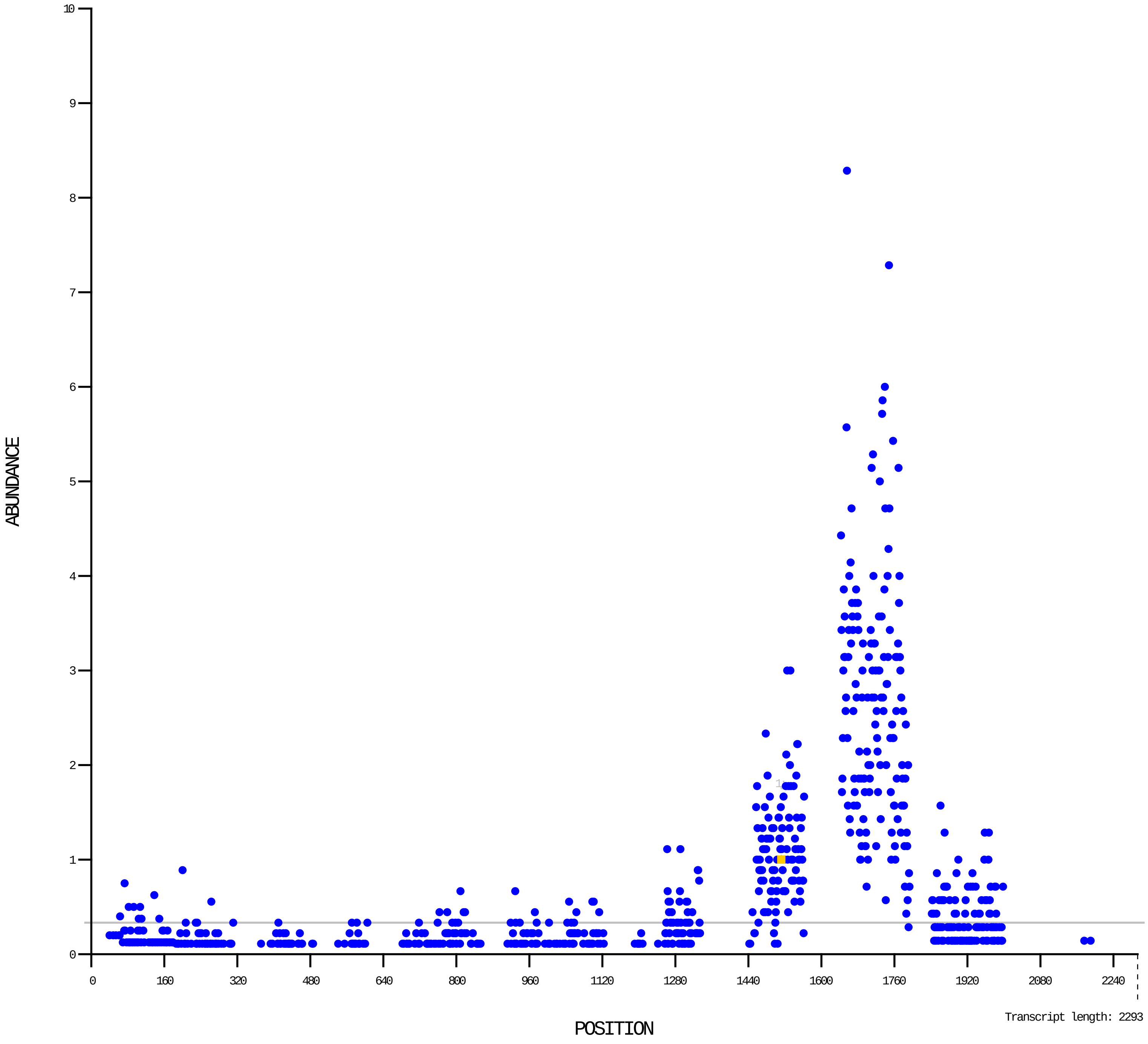

Category: 0 1 2 3 4
Degradome alignment: Median:

```
2  #1  Position:1512  Abundance: 1.00(deg) 1(sRNA)
   5'    TGAGCTGTTTGGCTATTCTCGC      3'  ID:
         ||||||| |||o |||o|||||           Score: 3.0
   3' ATATACTCGAC-AACTAATAGGAGCGAGTAAA 5'  p-value: 0.03
```

Cs1g19500.3 gene=Cs1g19500 CDS=405-2378

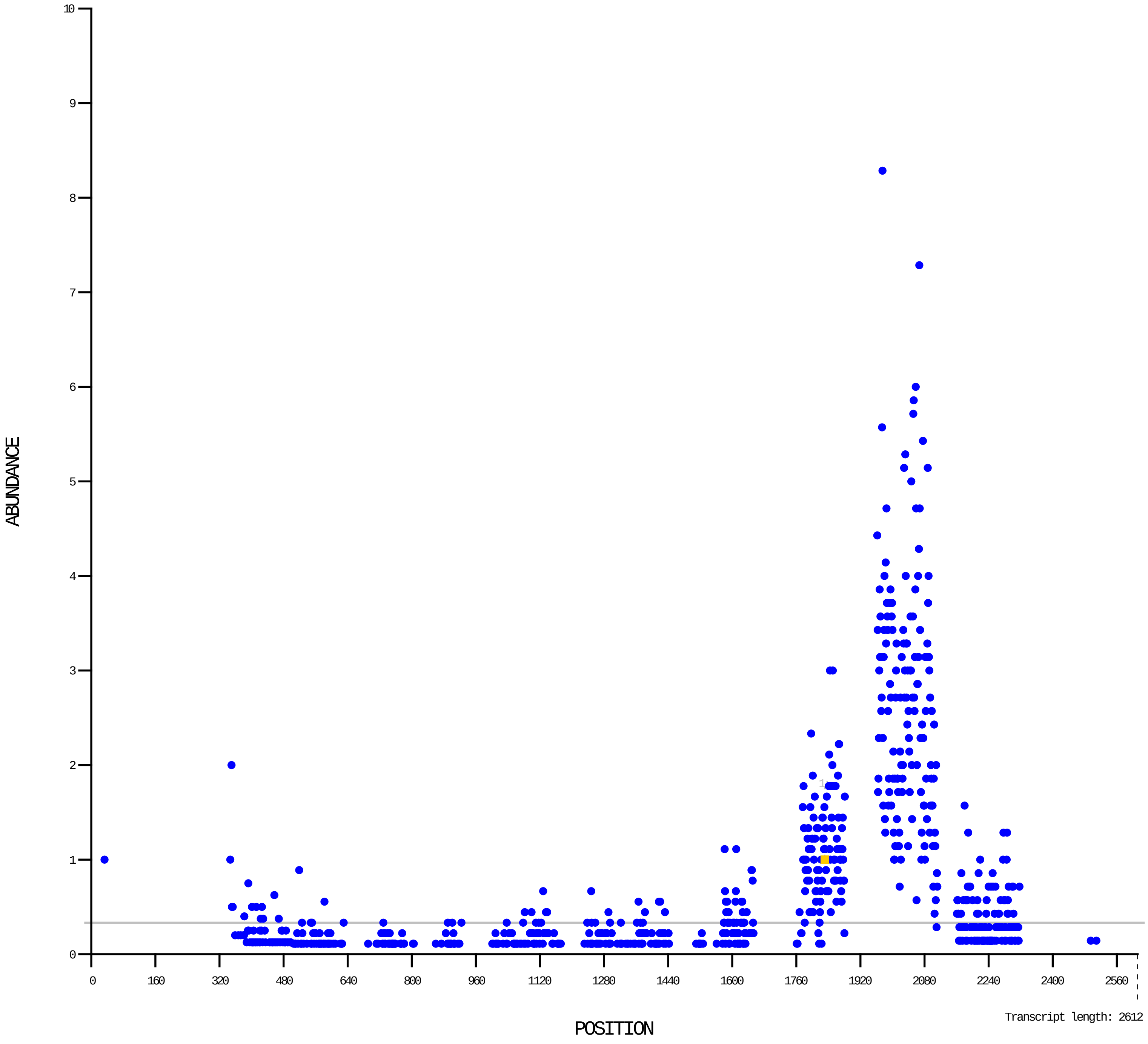

Category: 0 1 2 3 4
Degradome alignment: Median:

```
2  #1  Position:1831  Abundance: 1.00(deg) 1(sRNA)
   5'     TGAGCTGTTTGGCTATTCTCGC       3'  ID:
          ||||||| |||o |||o|||||           Score: 3.0
   3' ATATACTCGAC-AACTAATAGGAGCGAGTAAA 5'  p-value: 0.03
```

Cs1g19500.2 gene=Cs1g19500 CDS=78-2051

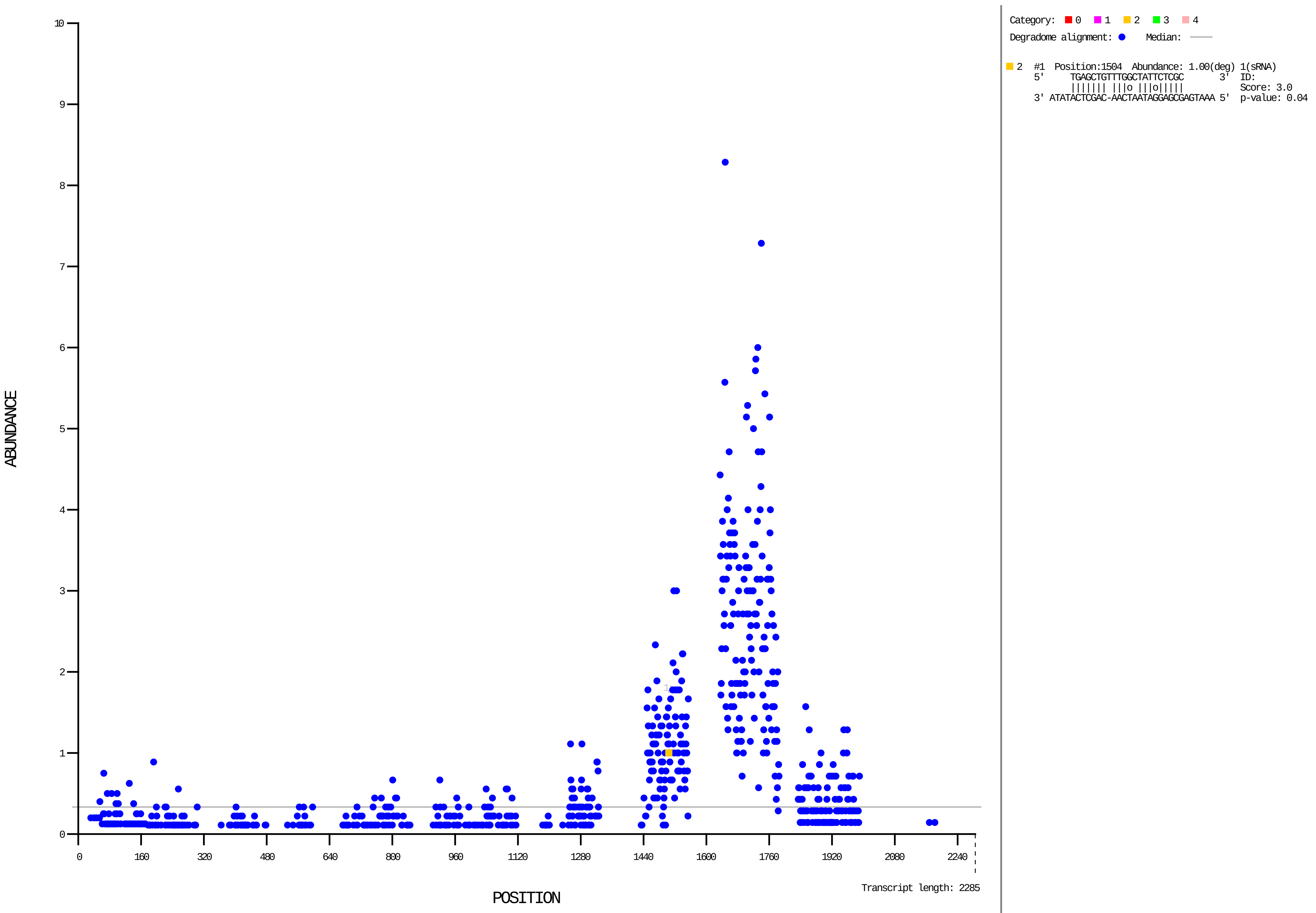

Cs1g19500.5 gene=Cs1g19500 CDS=54-2027

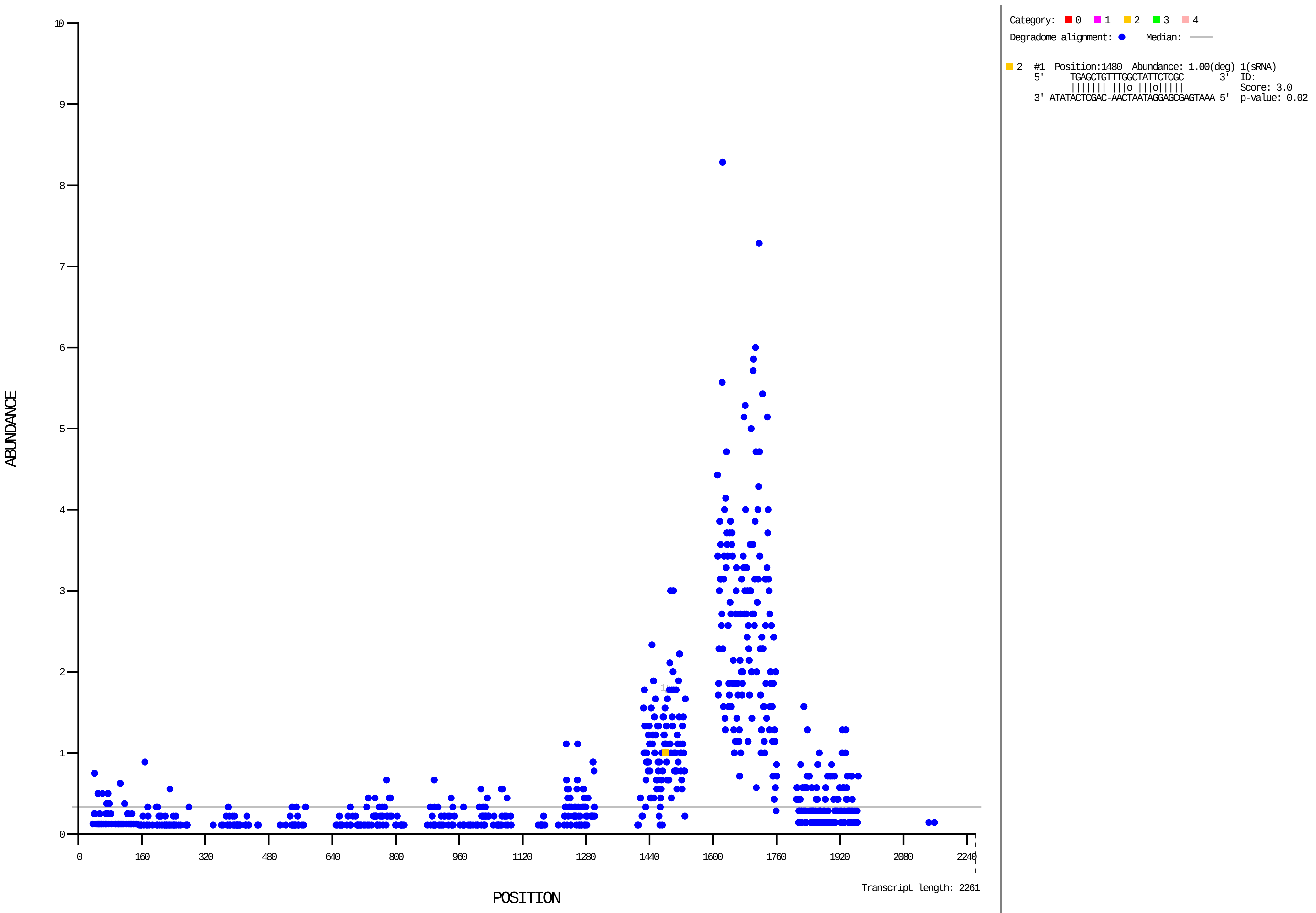

Cs1g19500.1 gene=Cs1g19500 CDS=414-2108

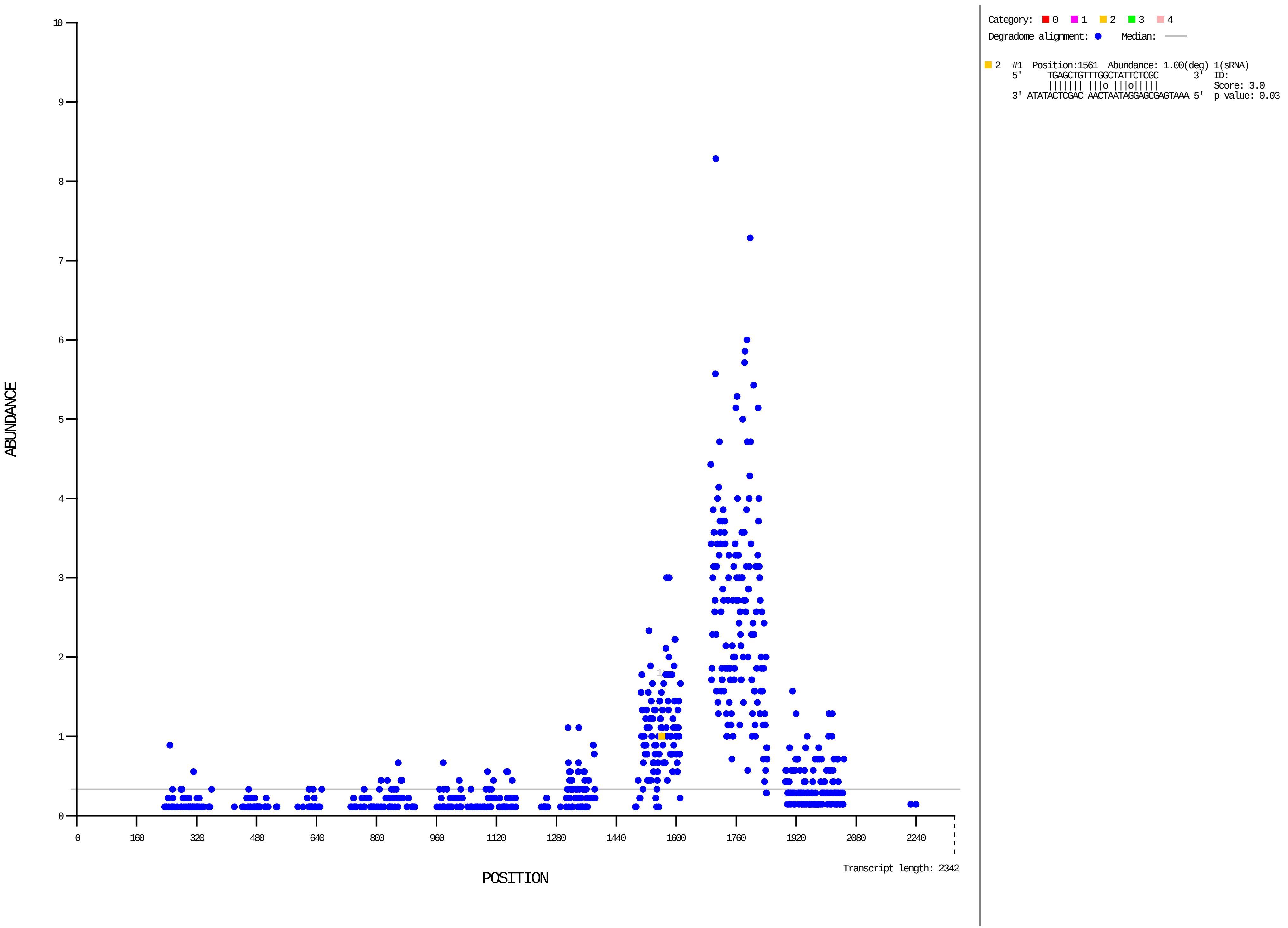

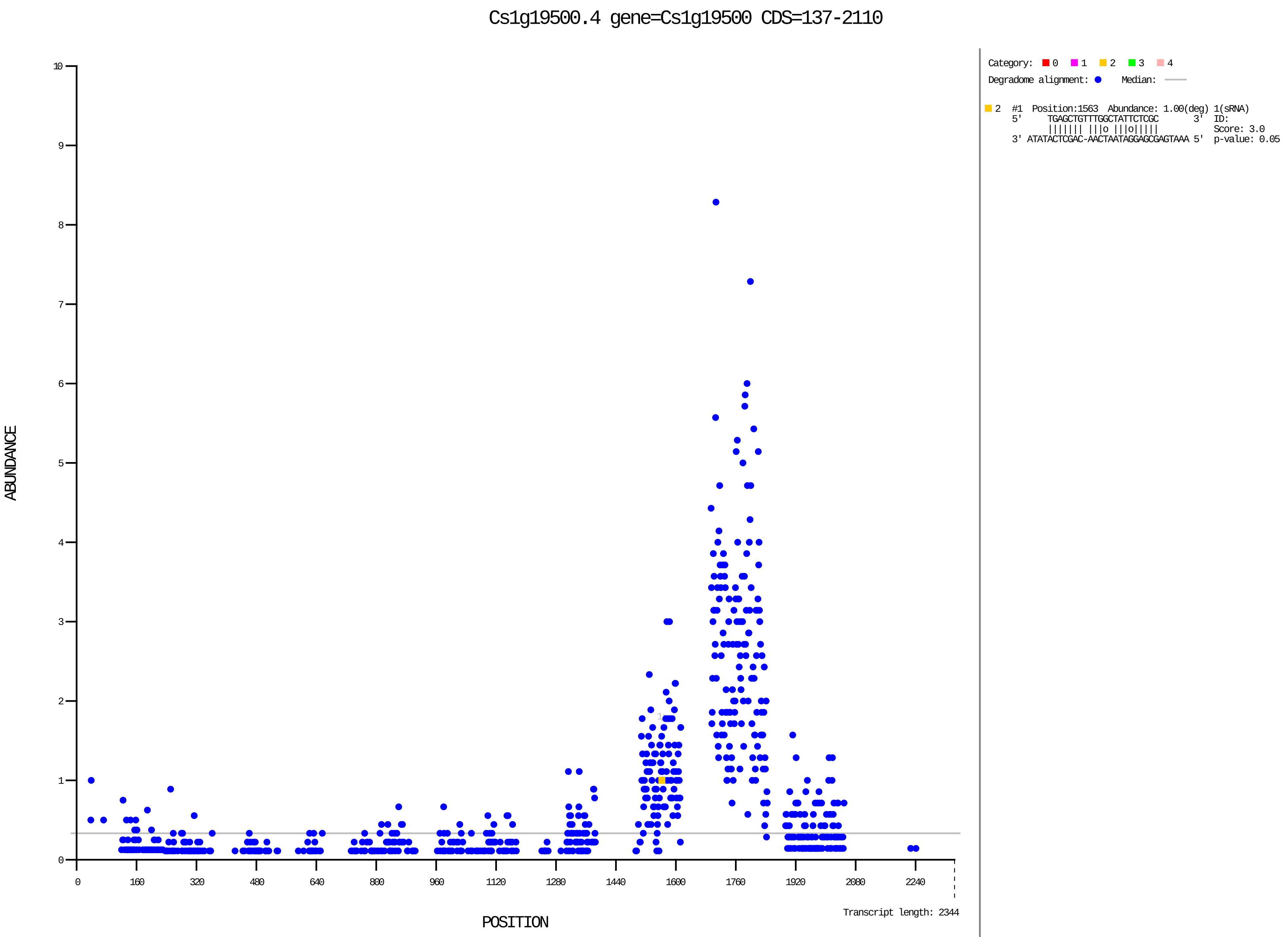
Cs1g19500.4 gene=Cs1g19500 CDS=137-2110
ABUNDANCE
POSITION
Transcript length: 2344
Category: 0 1 2 3 4
Degradome alignment:
Median:
2 #1 Position:1563 Abundance: 1.00(deg) 1(sRNA)
5' TGAGCTGTTTGGCTATTCTCGC 3' ID:
||||||| |||o |||o|||||
3' ATATACTCGAC-AACTAATAGGAGCGAGTAAA 5'
Score: 3.0
p-value: 0.05

orange1.1t03059.3 gene=orange1.1t03059 CDS=574-2721

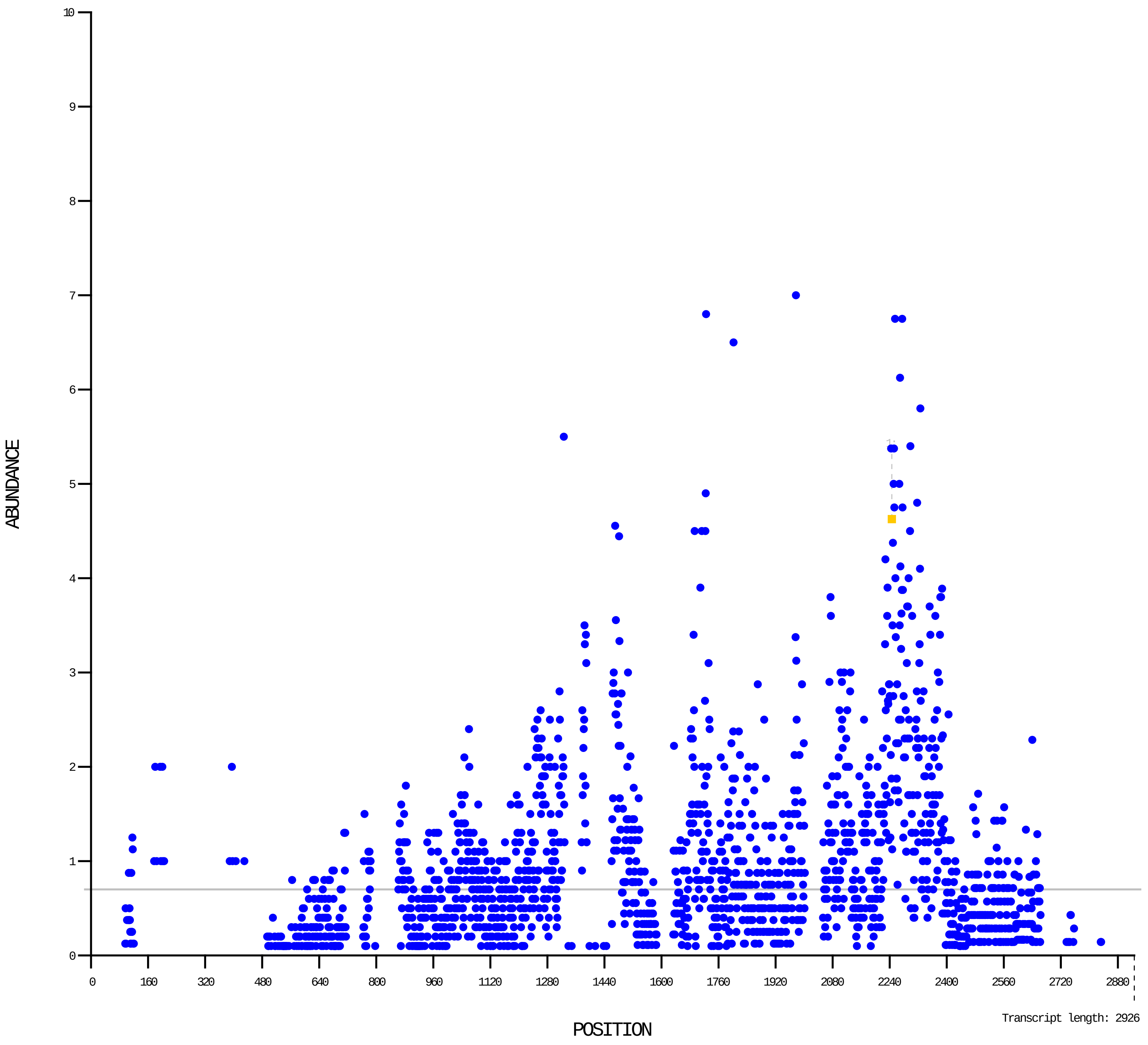

orange1.1t03059.4 gene=orange1.1t03059 CDS=276-2423

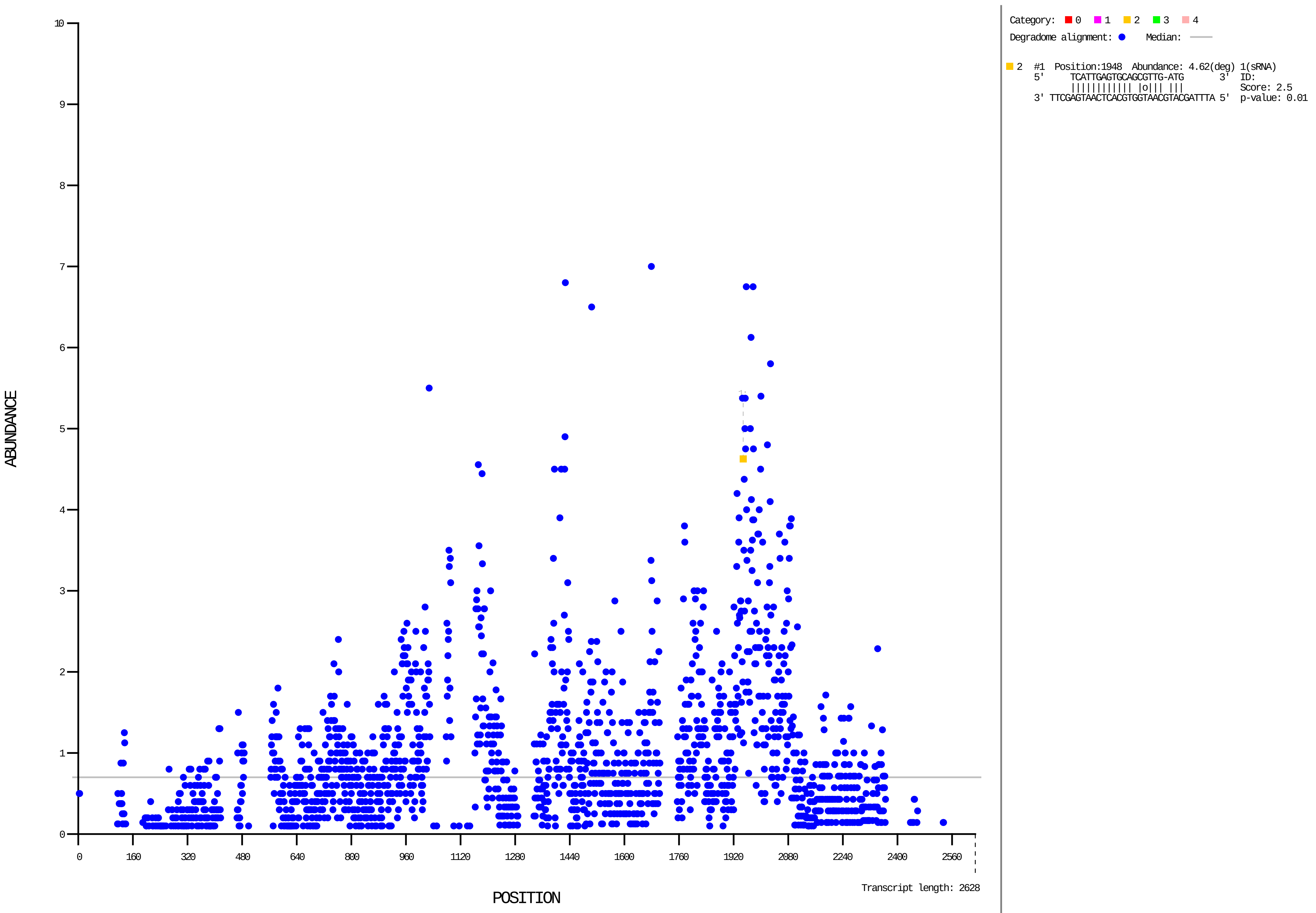

orange1.1t03059.7 gene=orange1.1t03059 CDS=257-2188

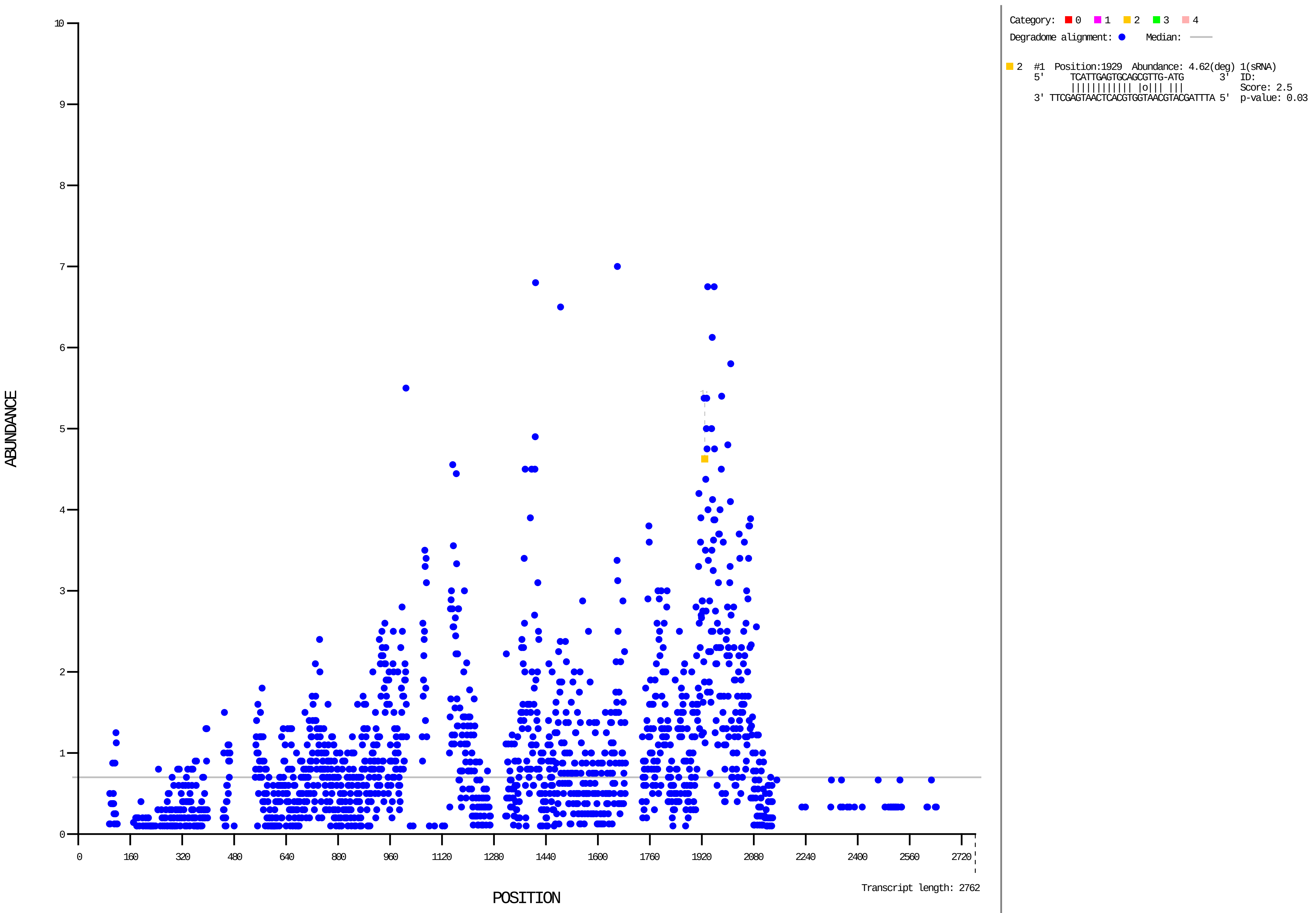

orange1.1t03059.1 gene=orange1.1t03059 CDS=257-2413

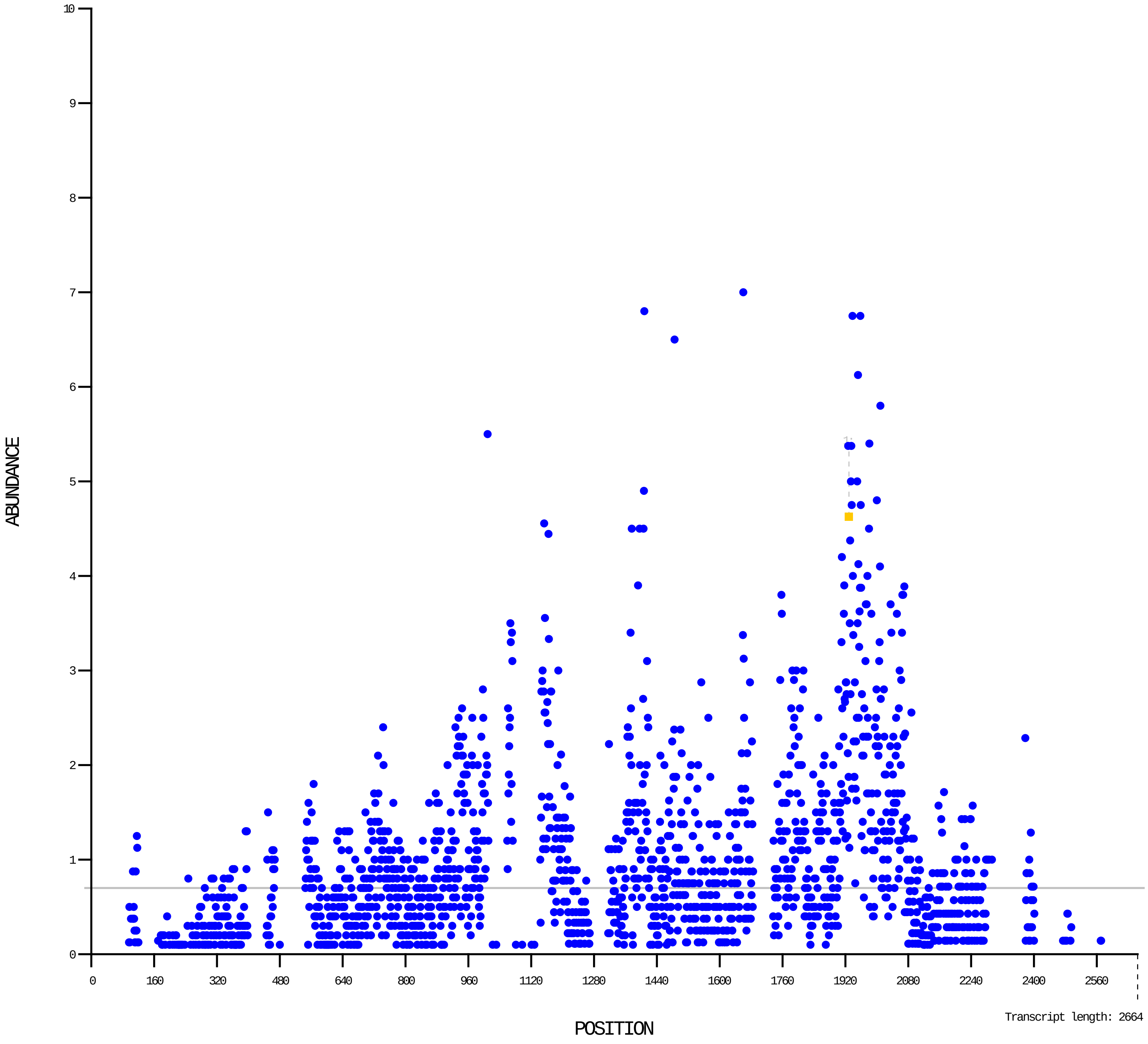

Category: 0 1 2 3 4
Degradome alignment: Median:

```
2  #1  Position:1929  Abundance: 4.62(deg) 1(sRNA)
   5'     TCATTGAGTGCAGCGTTG-ATG       3'  ID:
          ||||||||||||| |o||| |||          Score: 2.5
   3' TTCGAGTAACTCACGTGGTAACGTACGATTTA 5'  p-value: 0.02
```

orange1.1t03059.5 gene=orange1.1t03059 CDS=515-2662

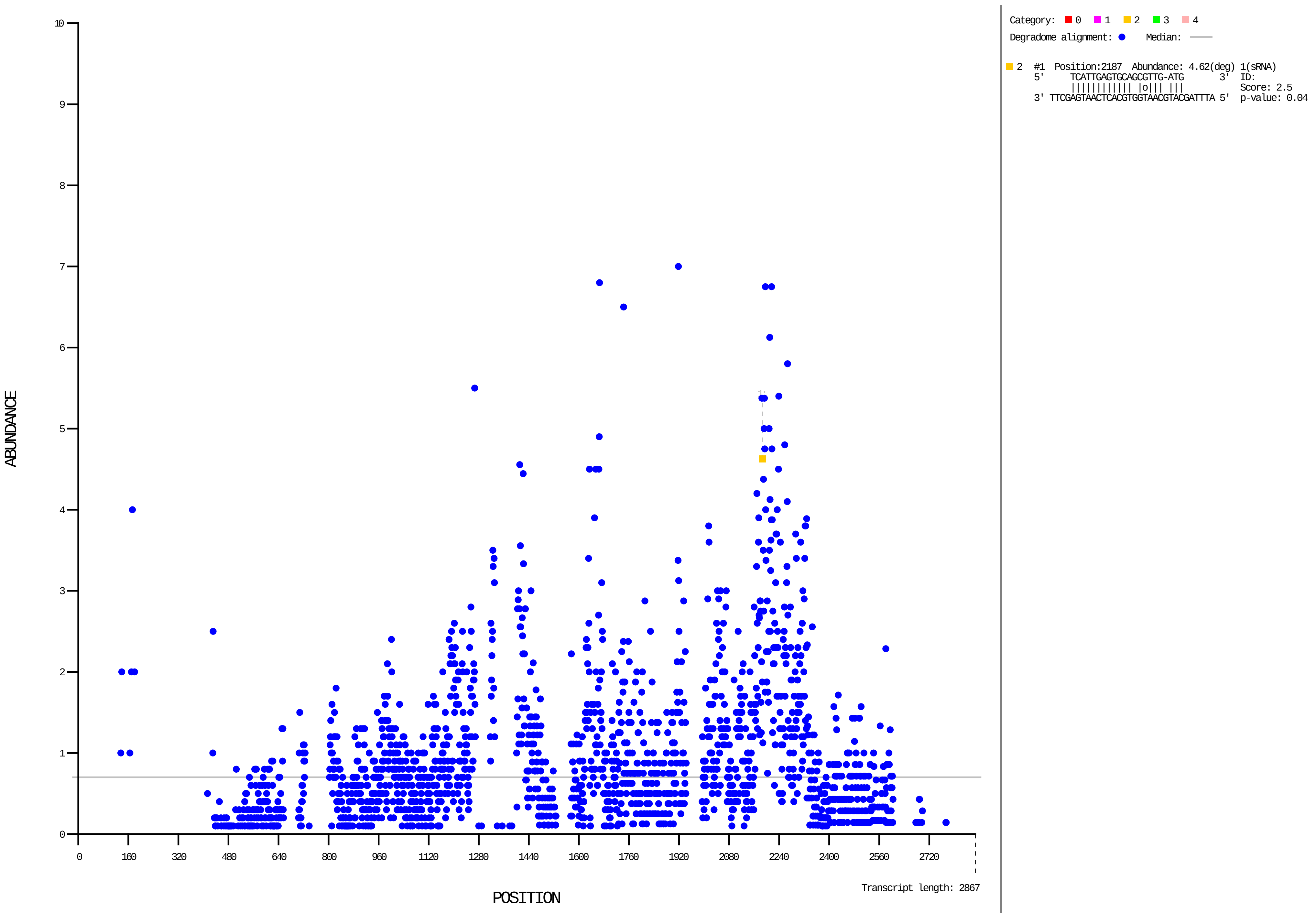

orange1.1t03059.9 gene=orange1.1t03059 CDS=257-2023

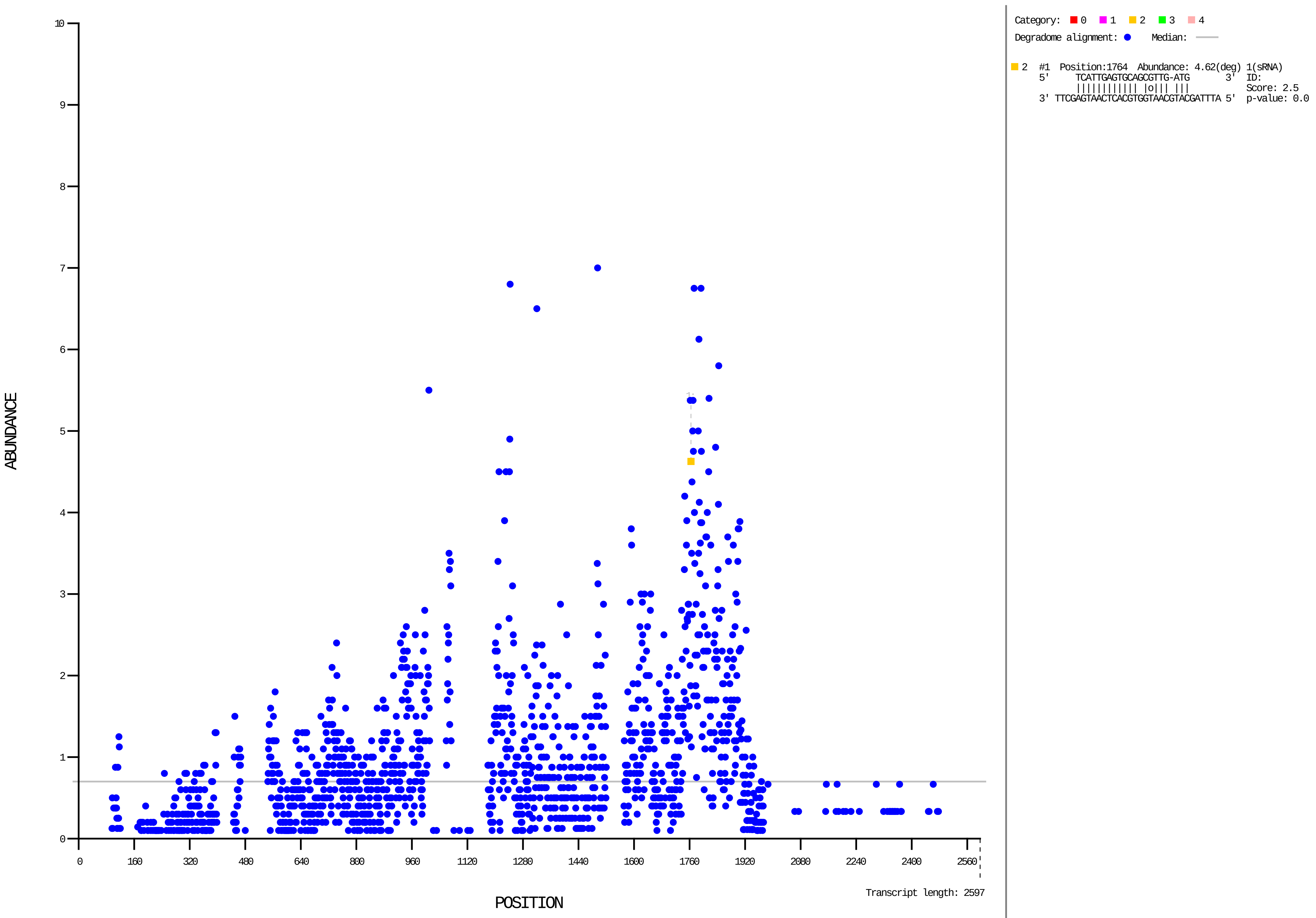

orange1.1t03059.8 gene=orange1.1t03059 CDS=131-2104

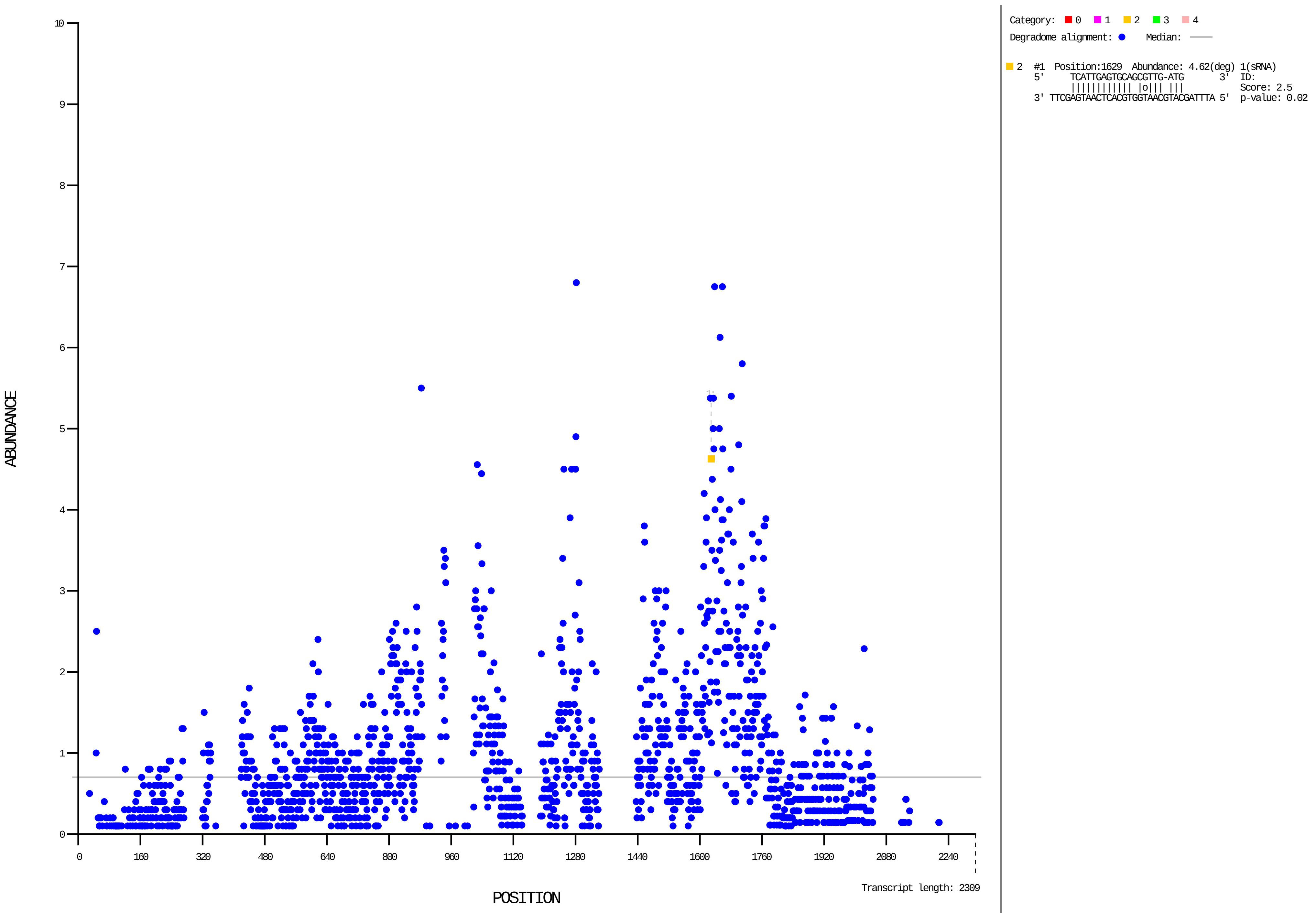

Cs4g03760.1 gene=Cs4g03760 CDS=503-3376

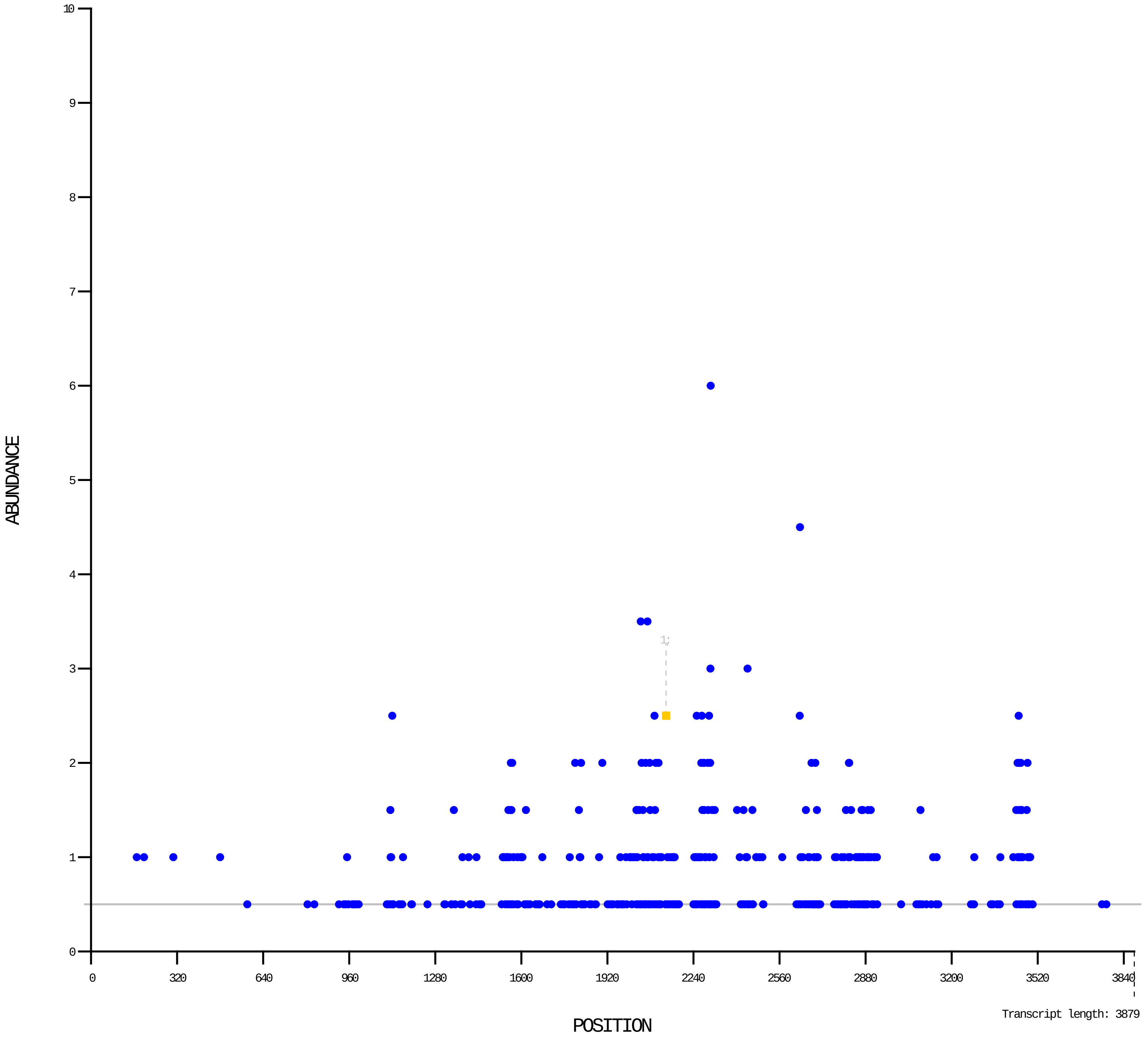

Category: 0 1 2 3 4
Degradome alignment: Median:

2 #1 Position:2138 Abundance: 2.50(deg) 1(sRNA)

```
5'     AGAATCTTGATGATGCTGCA          3'  ID:
       ||||| ||o|||||||o|||              Score: 2.0
3' TACTTCTTA-AATTACTACGGCGTTGTCCAAG 5'  p-value: 0.01
```

Cs4g03760.2 gene=Cs4g03760 CDS=1-2874

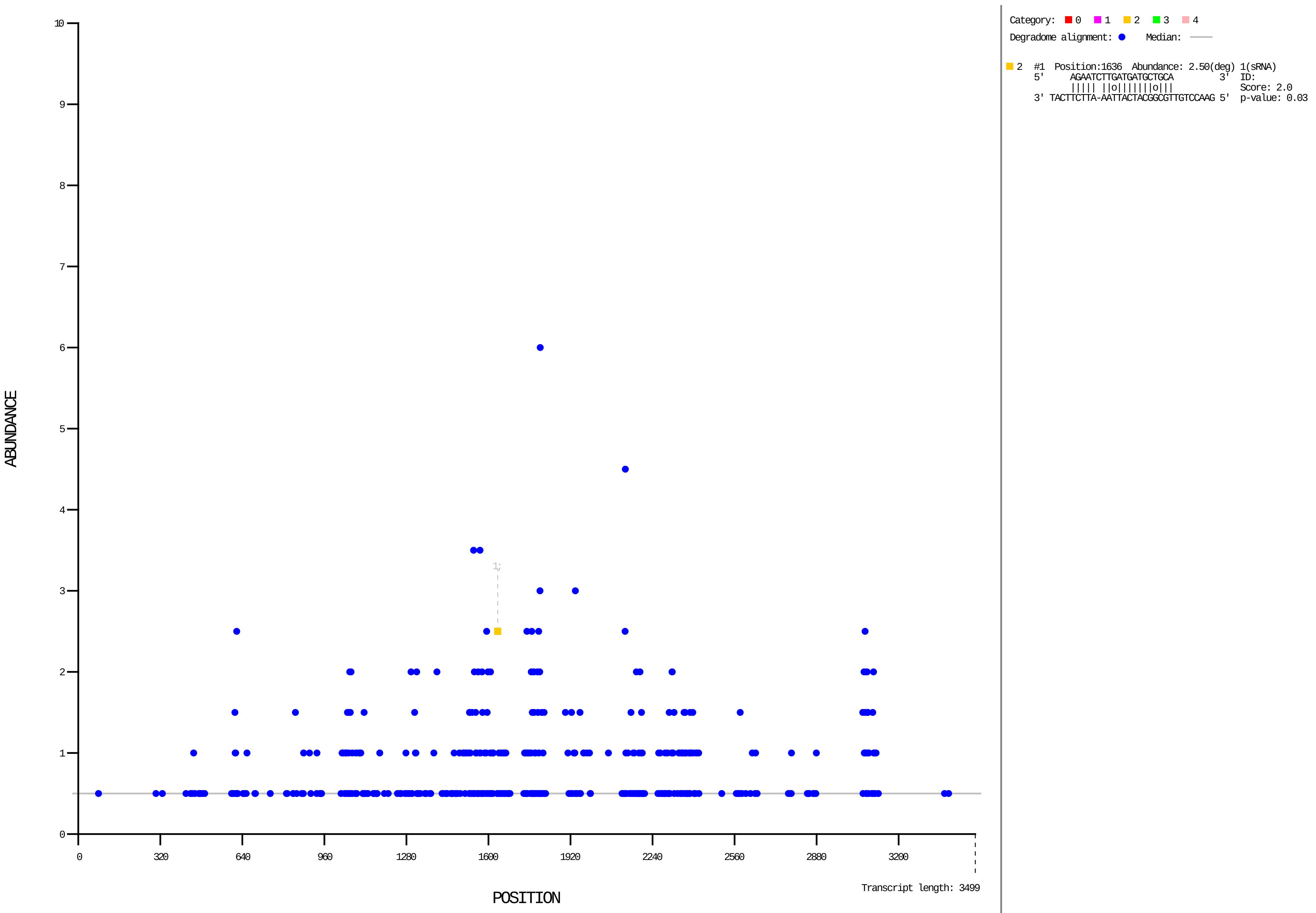

orange1.1t01185.2 gene=orange1.1t01185 CDS=44-1240

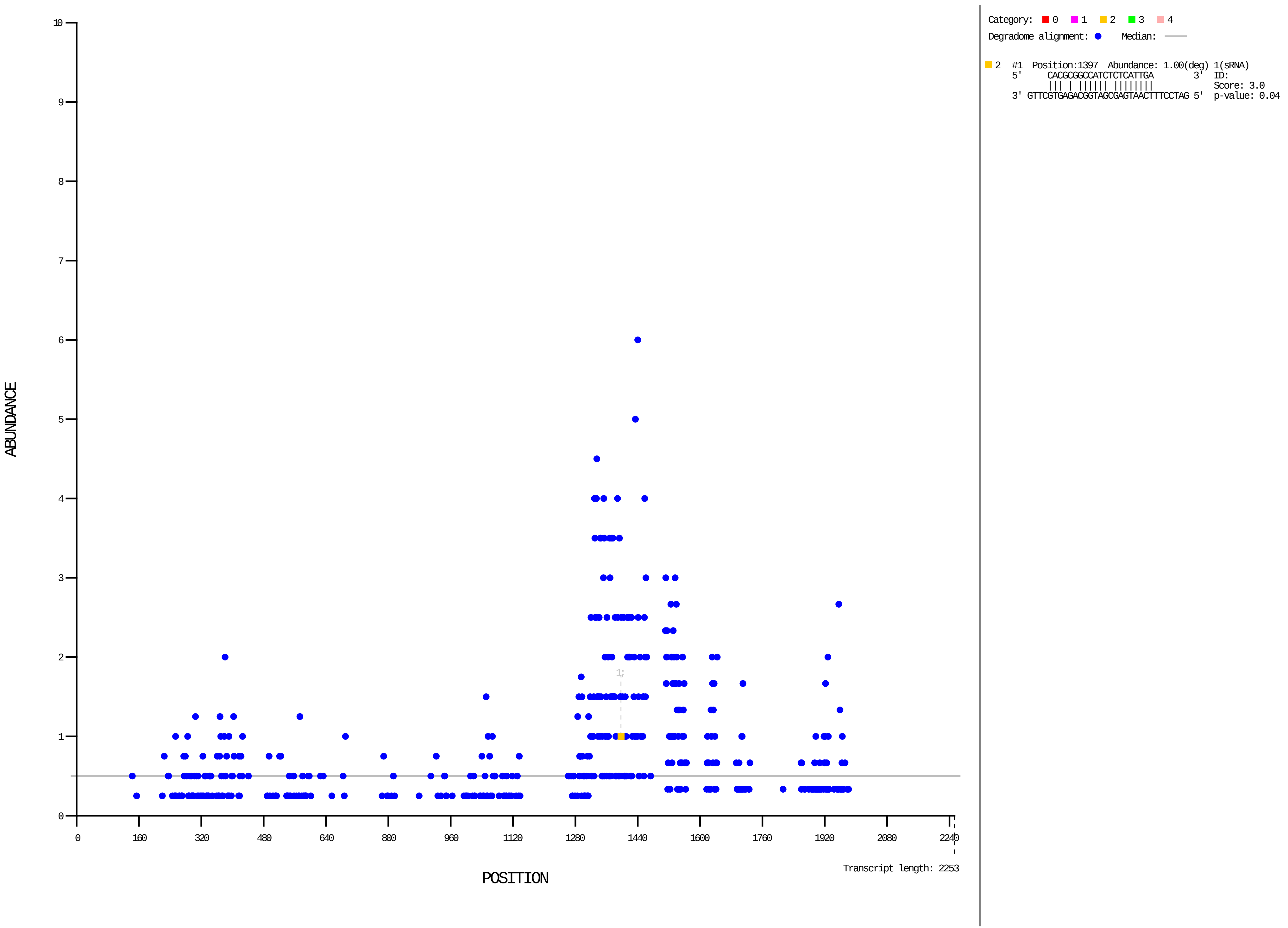

orange1.1t01185.1 gene=orange1.1t01185 CDS=44-1591

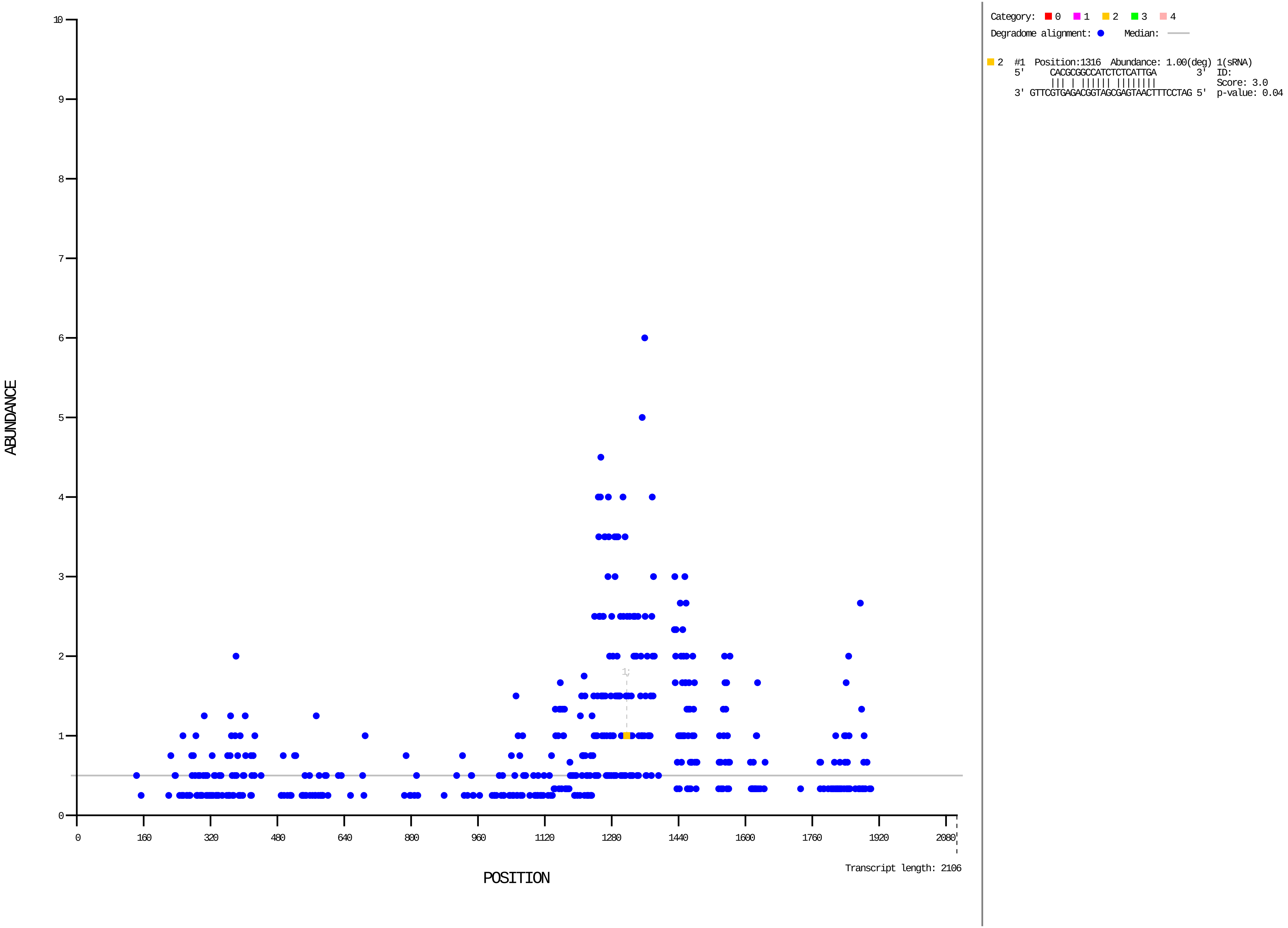

Cs9g05070.2 gene=Cs9g05070 CDS=367-1641

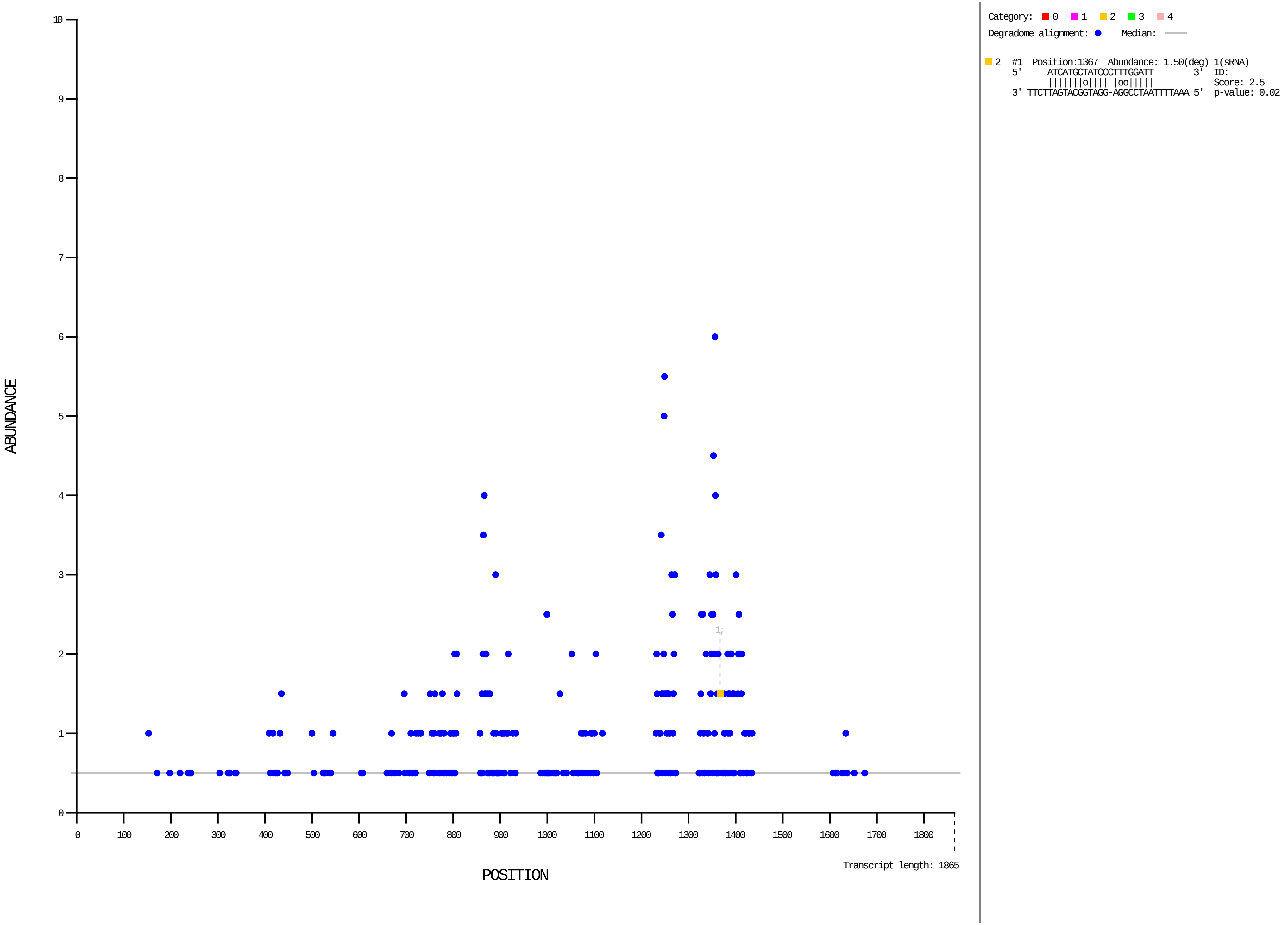

Cs9g05070.1 gene=Cs9g05070 CDS=279-1874

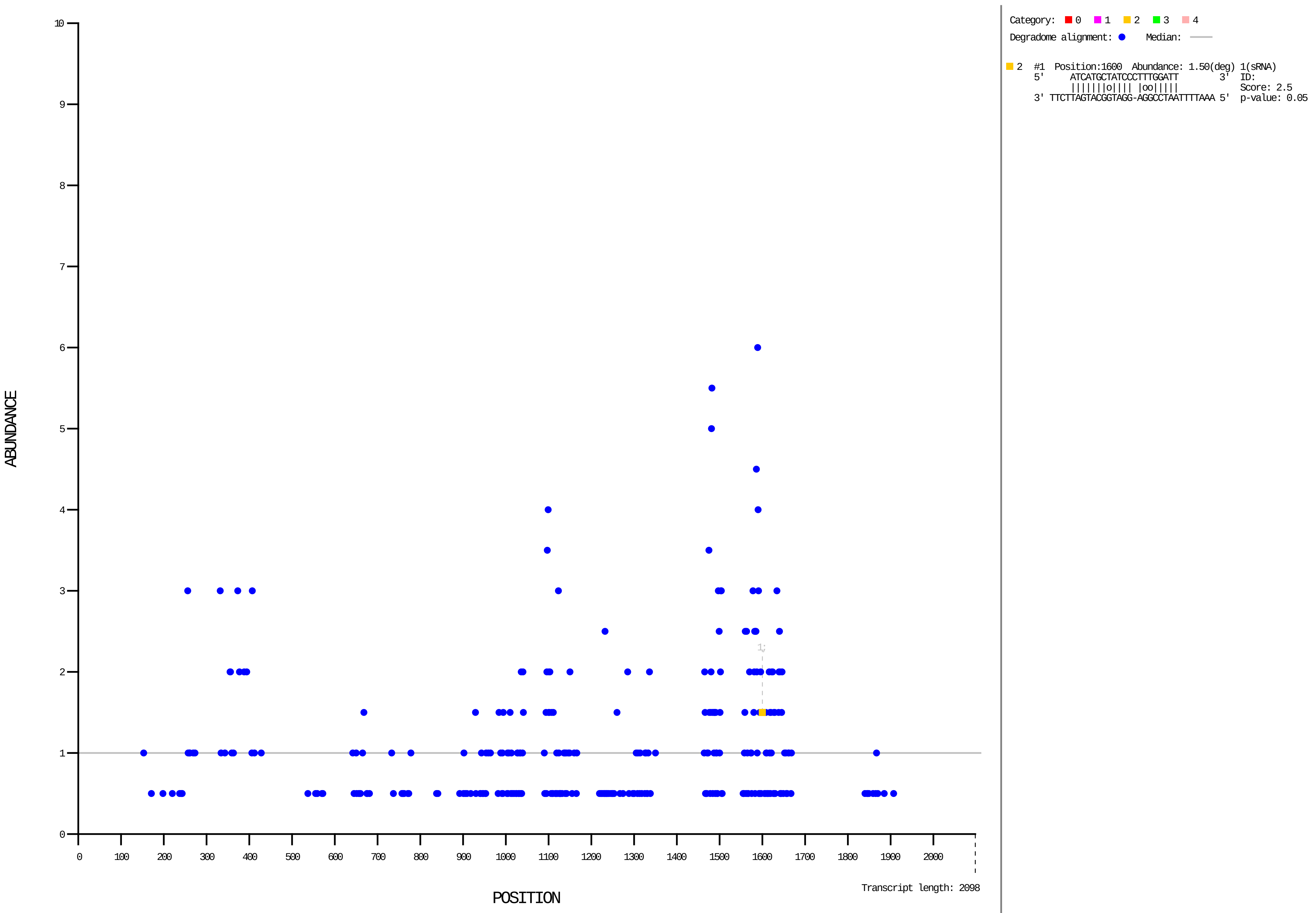

Cs9g09860.1 gene=Cs9g09860 CDS=191-2482

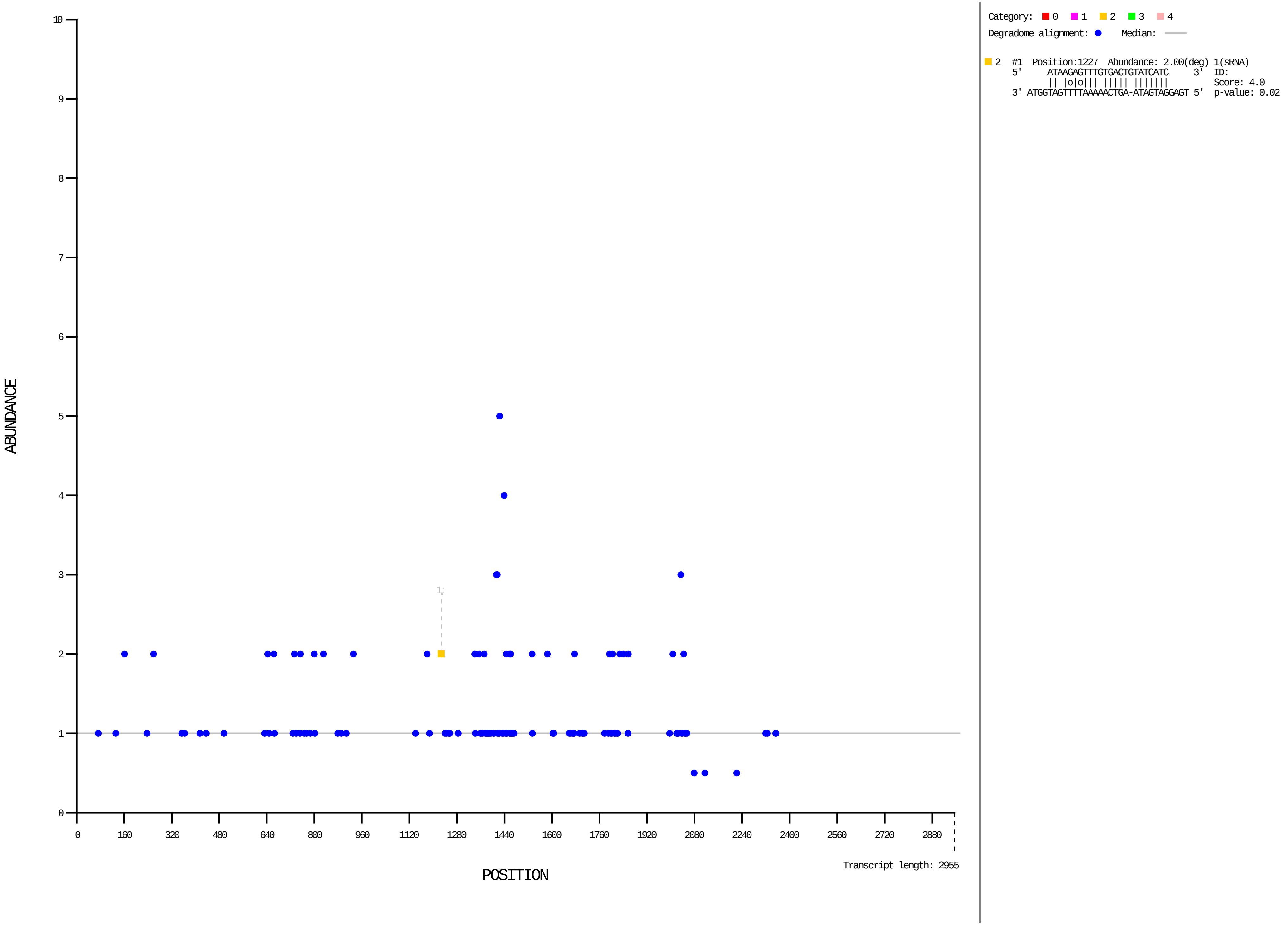

Cs5g19570.1 gene=Cs5g19570 CDS=1483-3201

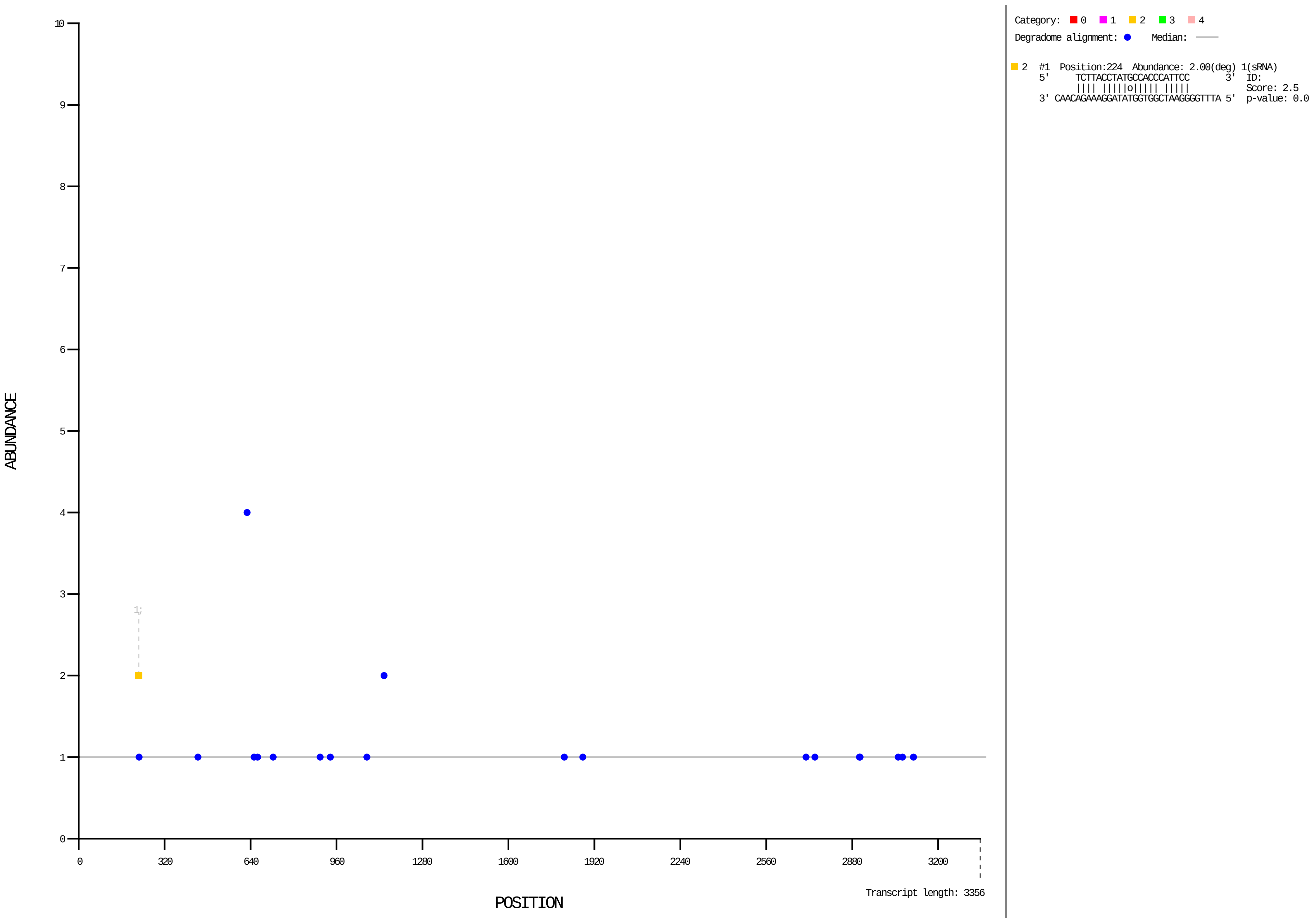

Cs8g19850.2 gene=Cs8g19850 CDS=1-1587

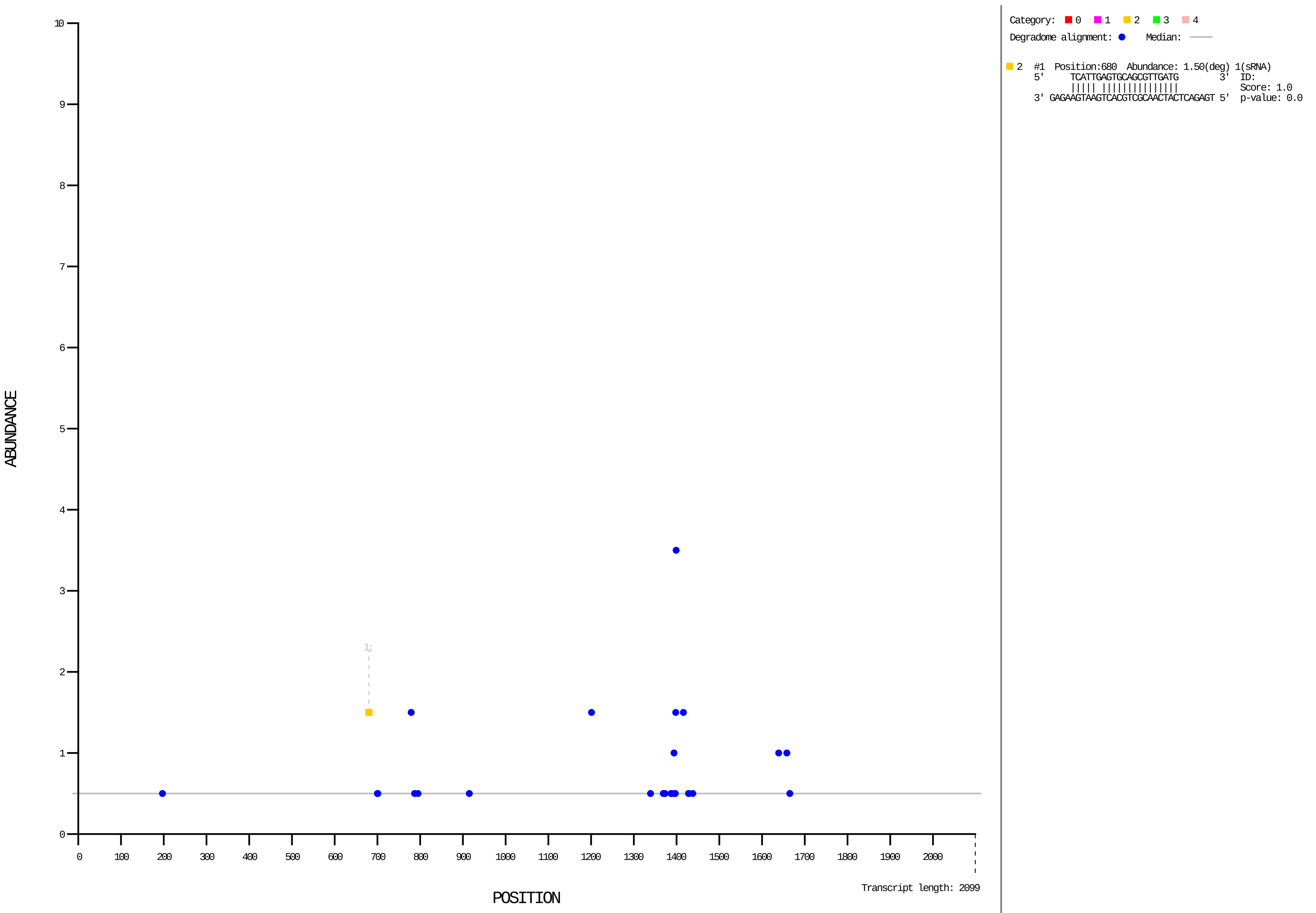

Cs8g19850.1 gene=Cs8g19850 CDS=1-1674

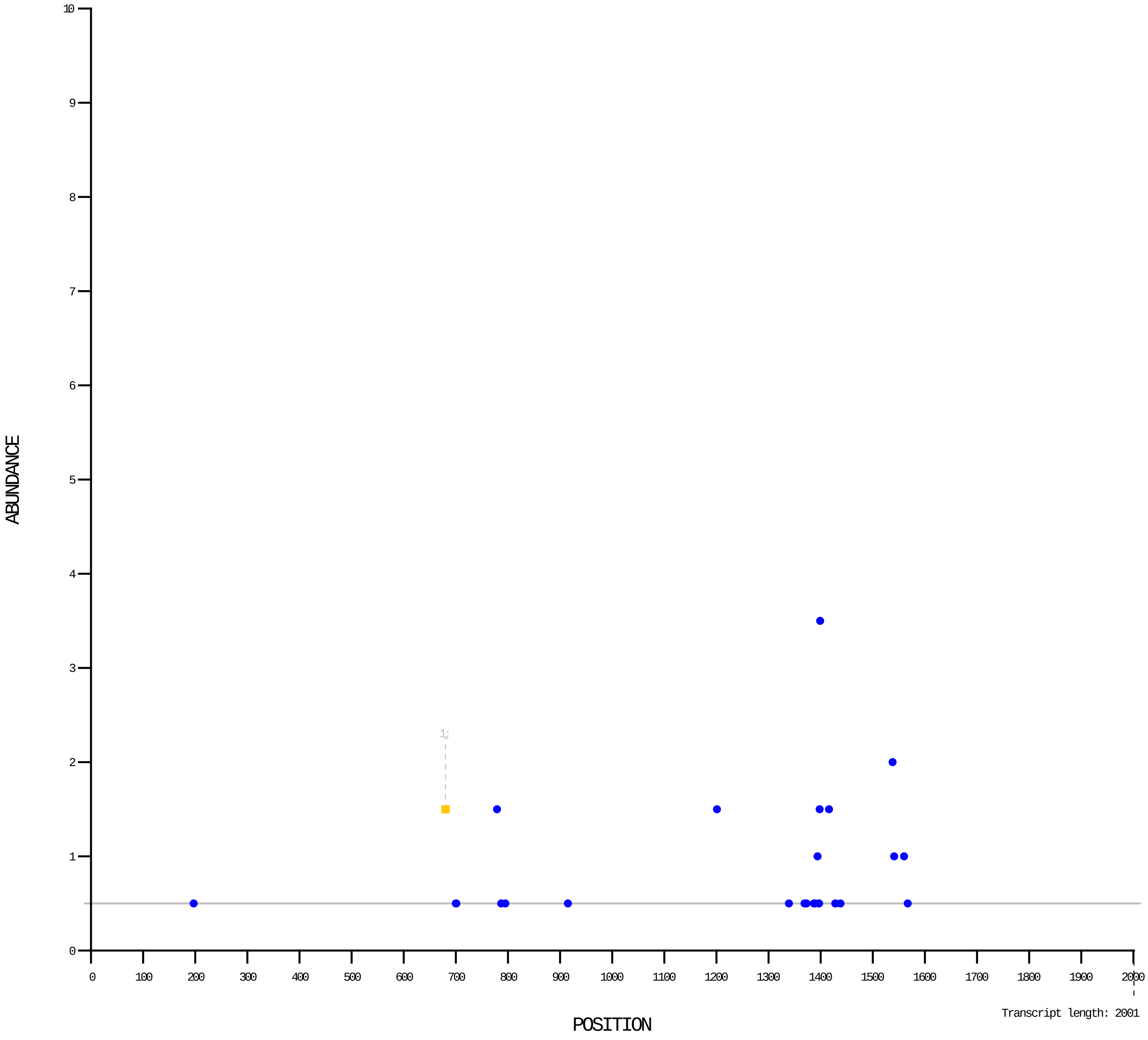

Category: 0 1 2 3 4
Degradome alignment: Median:

2 #1 Position:680 Abundance: 1.50(deg) 1(sRNA)

```
5'     TCATTGAGTGCAGCGTTGATG        3'  ID:
       ||||| |||||||||||||||            Score: 1.0
3' GAGAAGTAAGTCACGTCGCAACTACTCAGAGT 5'  p-value: 0.0
```

Cs1g19500.8 gene=Cs1g19500 CDS=173-1768

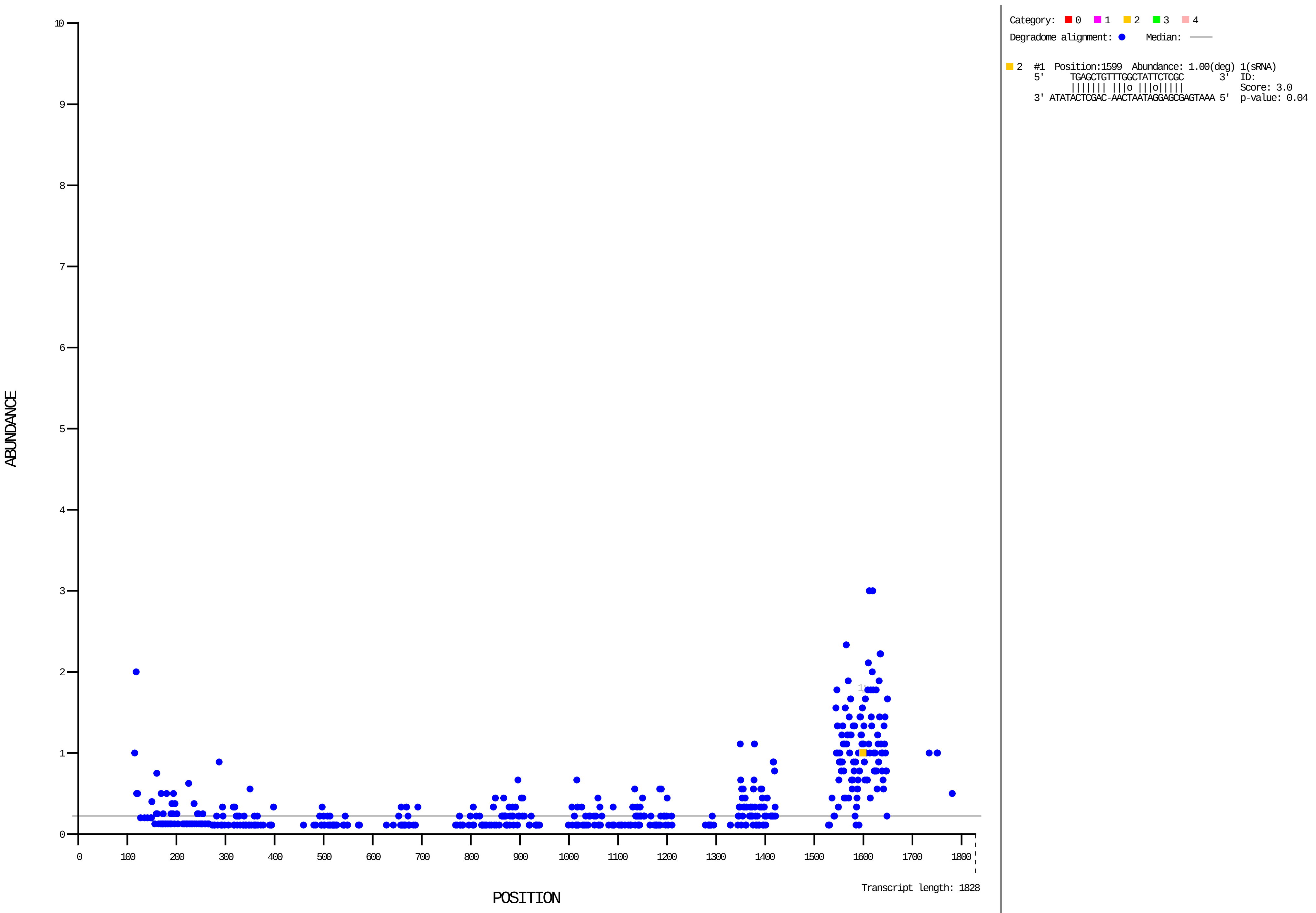

Cs1g19500.9 gene=Cs1g19500 CDS=86-1681

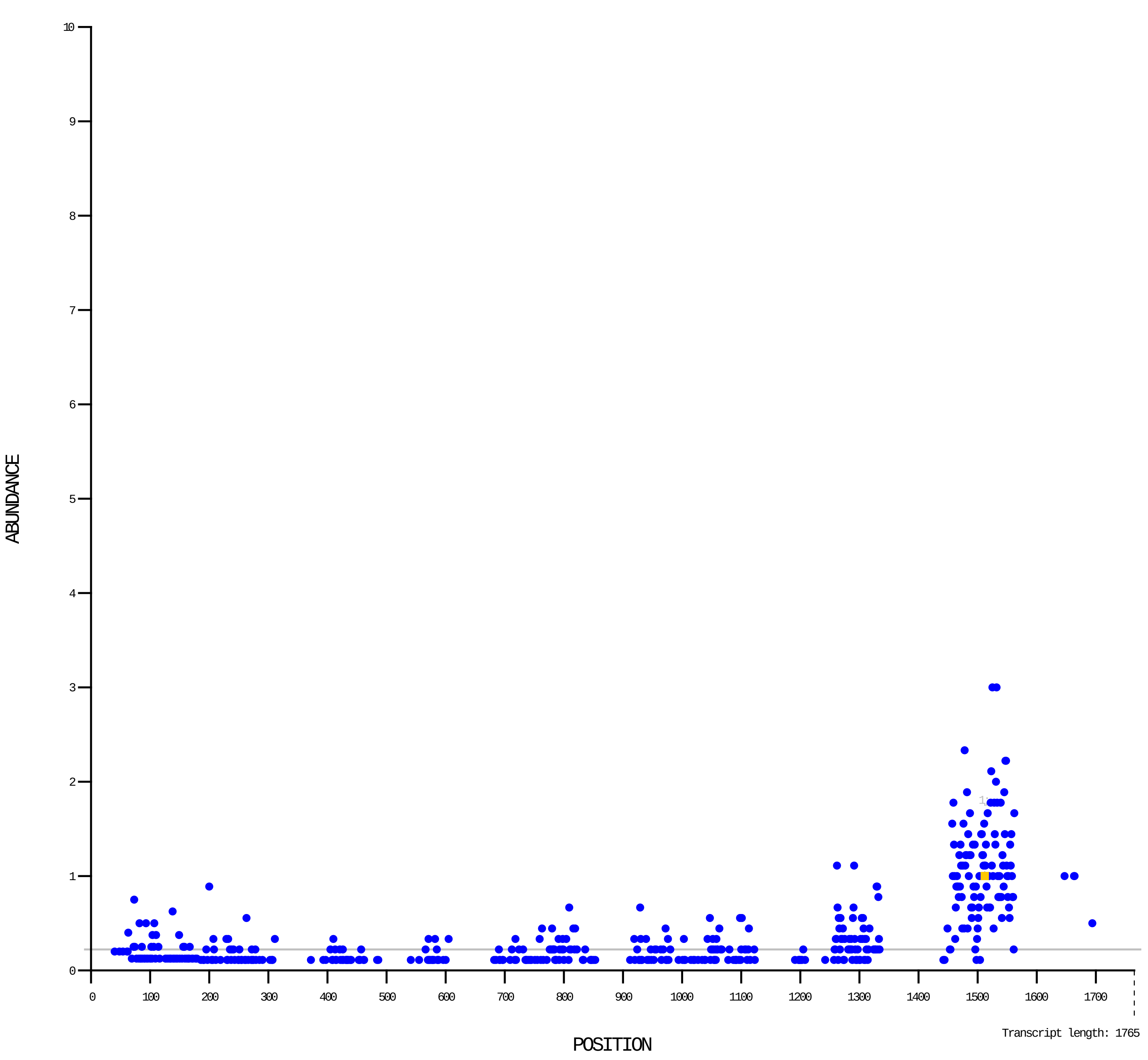

Cs5g19440.1 gene=Cs5g19440 CDS=1-2436

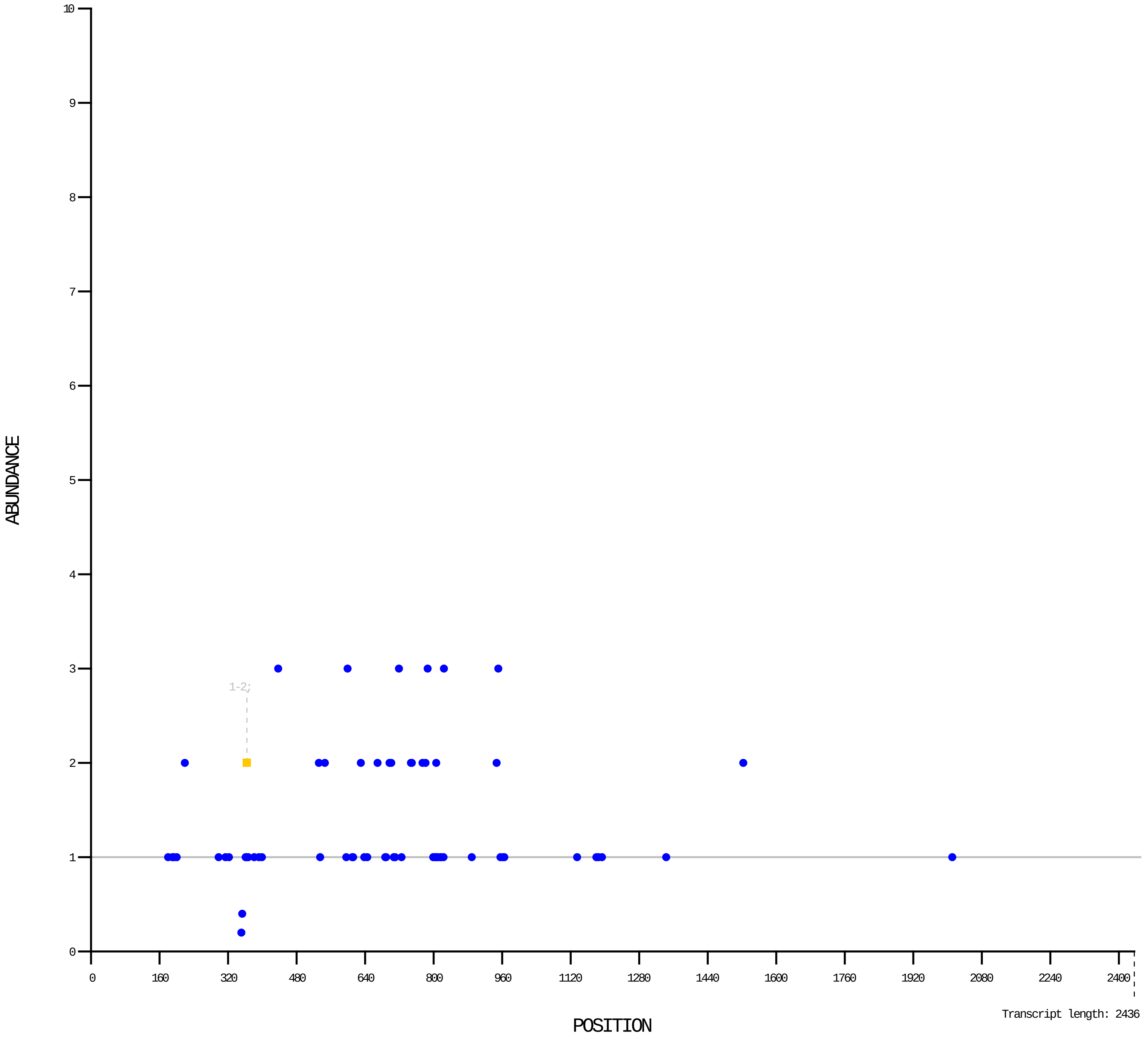

Category: 0 1 2 3 4
Degradome alignment: Median:

2 #1 Position:364 Abundance: 2.00(deg) 1(sRNA)
```
5'      TCTTCCCTATGCCTCCCATTCC        3'  ID:
        |||| |||||o|||||||| ||            Score: 2.5
3' CAACAGAATGGATATGGAGGGTATGGTGTCTA 5'  p-value: 0.02
```

2 #2 Position:364 Abundance: 2.00(deg) 1(sRNA)
```
5'      TCTTACCTATGCCACCCATTCC        3'  ID:
        ||||||||||o|| ||||| ||            Score: 2.5
3' CAACAGAATGGATATGGAGGGTATGGTGTCTA 5'  p-value: 0.0
```

Cs1g08230.1 gene=Cs1g08230 CDS=214-891

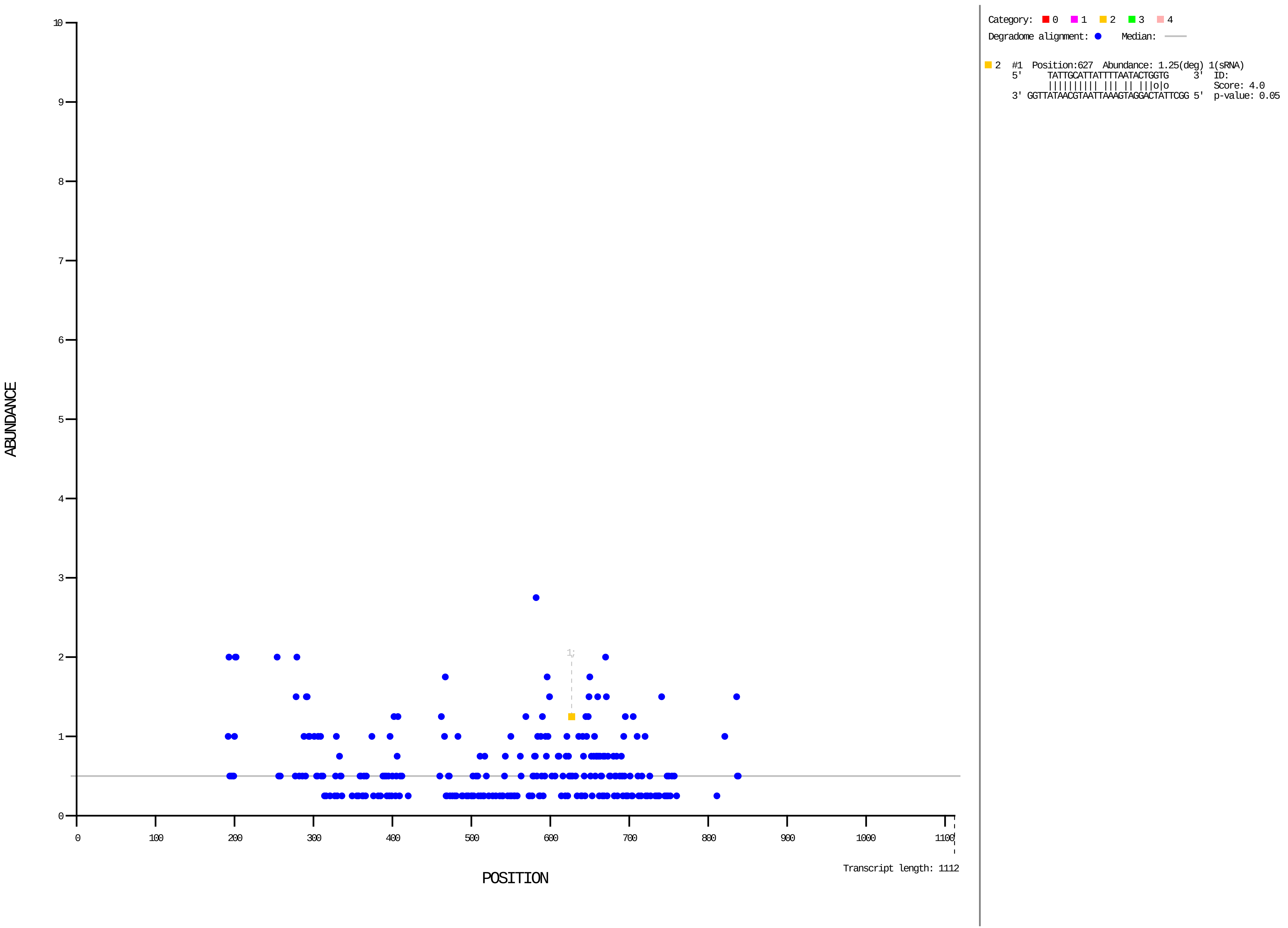

Cs3g10410.3 gene=Cs3g10410 CDS=196-1272

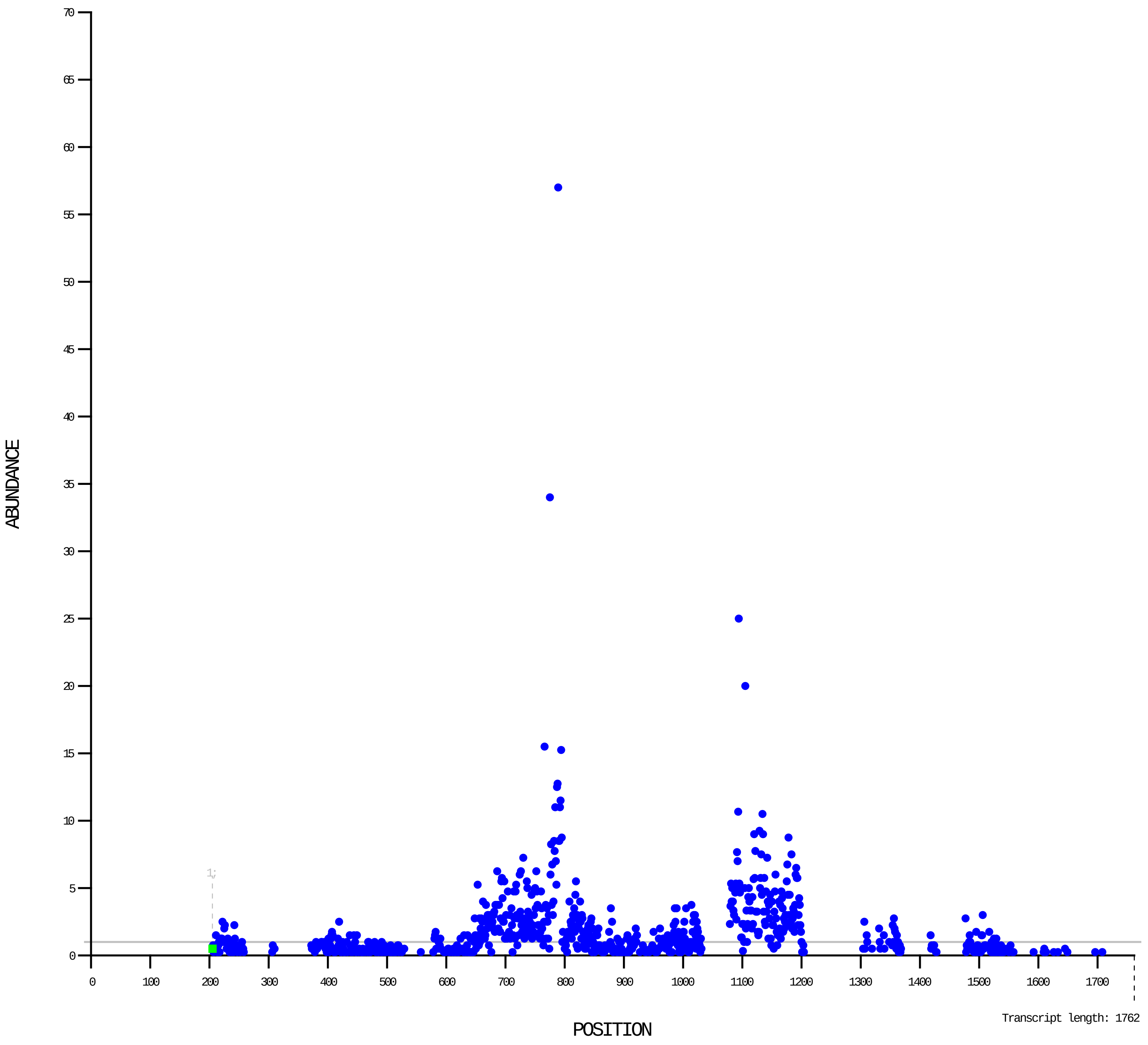

Category: 0 1 2 3 4

Degradome alignment: Median:

```
3  #1  Position:205  Abundance: 0.50(deg) 1(sRNA)
   5'     TCATTTTTGCGTGCAATGATCC       3'  ID:
          o||||o||||| ||||o || |           Score: 4.5
   3' ACCGGGTAAGAACGC-CGTTGGTAAGACTTTA 5'  p-value: 0.05
```

Cs6g07800.1 gene=Cs6g07800 CDS=72-1739

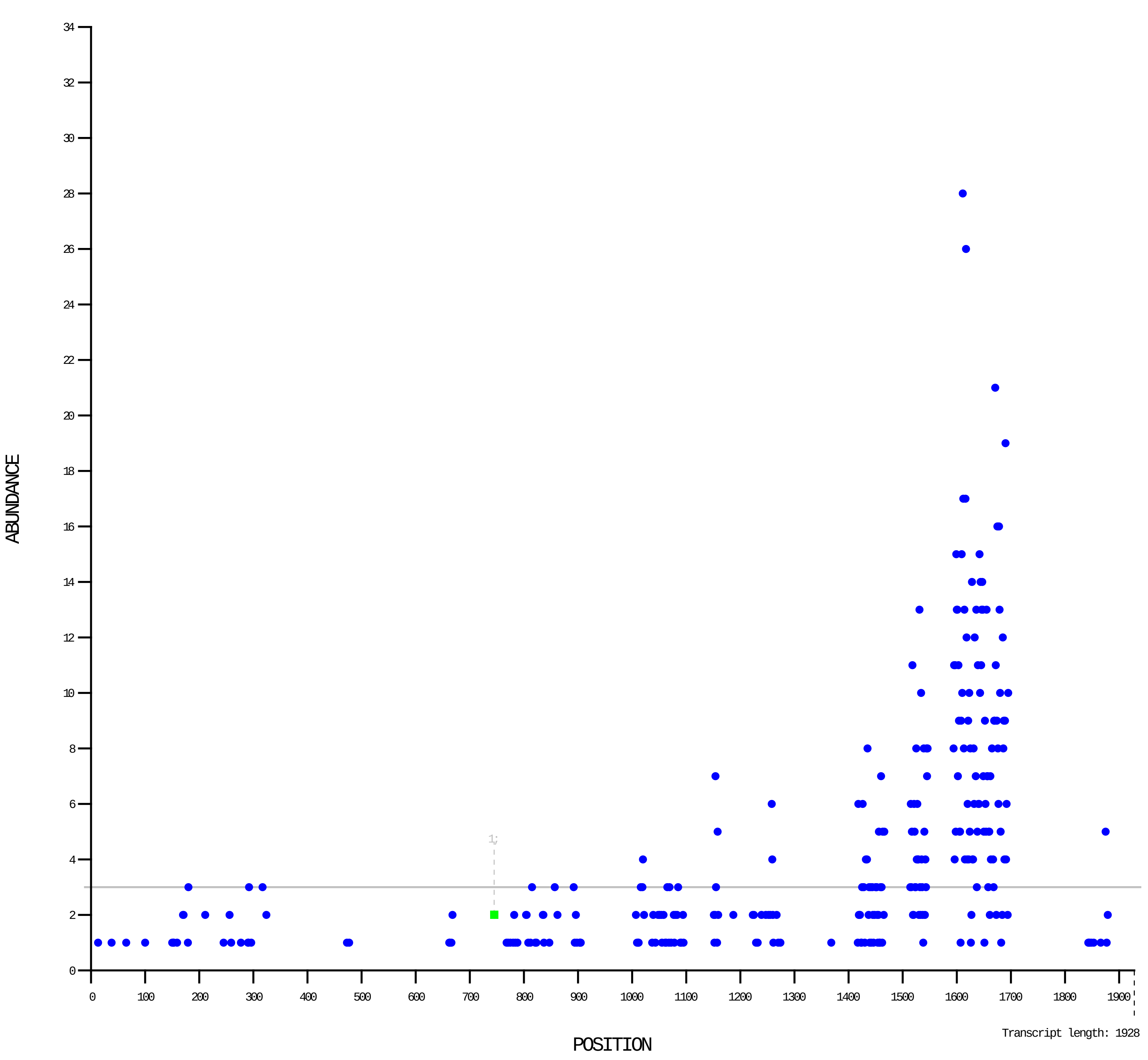

Category: 0 1 2 3 4
Degradome alignment: Median:

3 #1 Position:745 Abundance: 2.00(deg) 1(sRNA)
5' TCATTGAGTGCAGCGTTGATG 3' ID:
|||||||||||||o||||| Score: 1.5
3' GAGAAGTAACTCACGTCGTAACTAATAAGCAT 5' p-value: 0.0

Cs5g22660.1 gene=Cs5g22660 CDS=401-1264

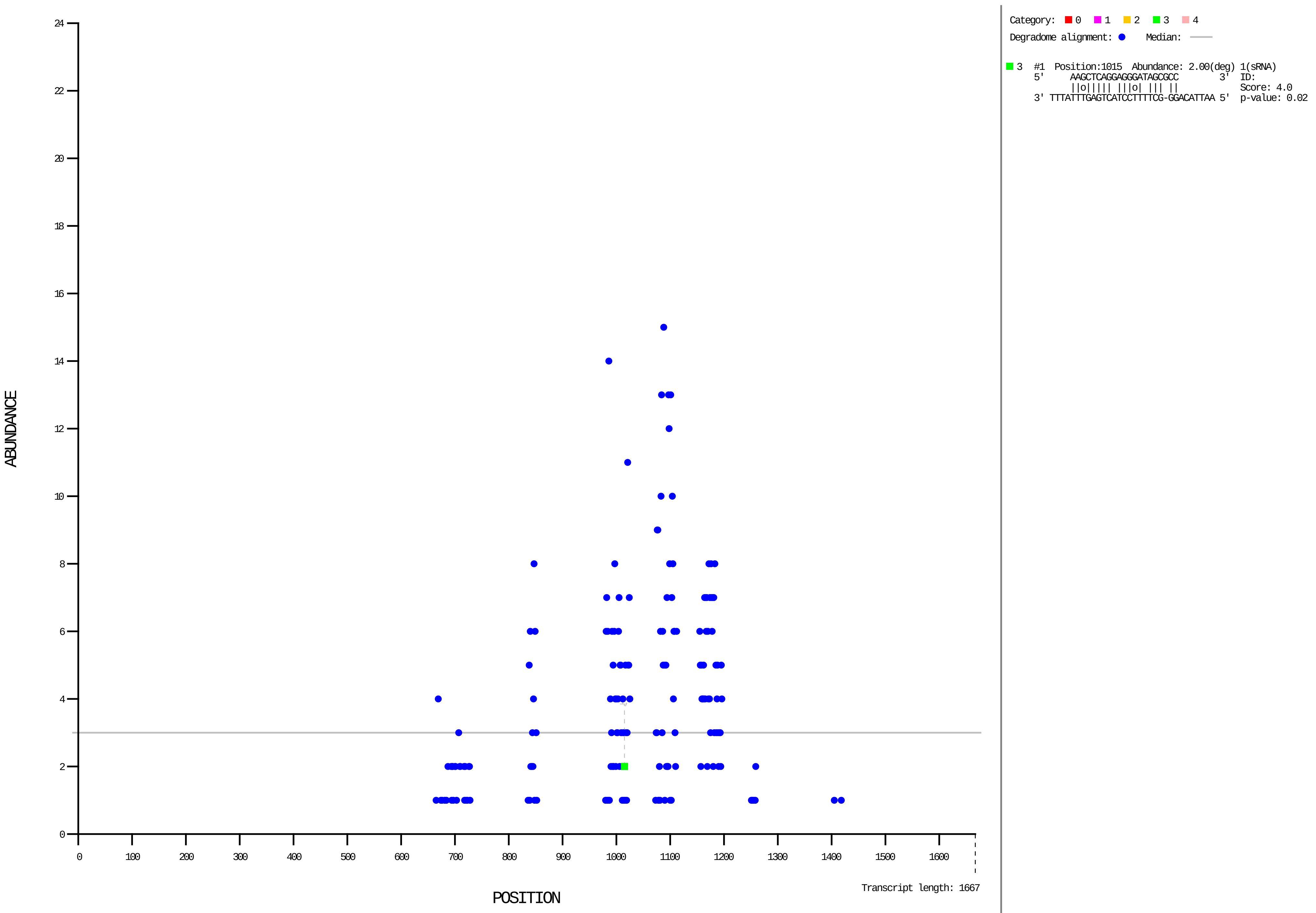

Cs8g20700.2 gene=Cs8g20700 CDS=1-1320

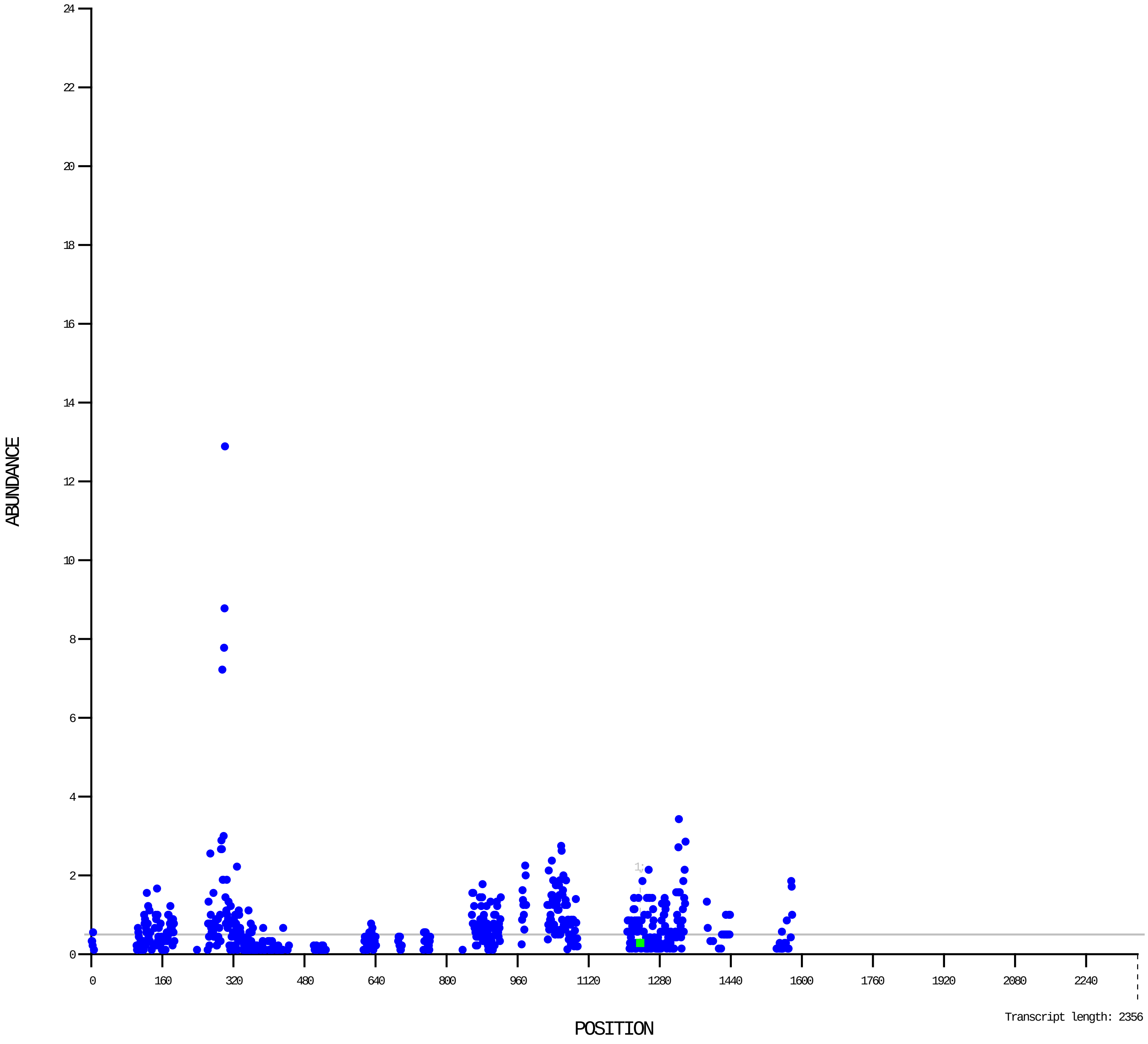

Category: 0 1 2 3 4
Degradome alignment: ● Median:

```
3   #1  Position:1236  Abundance: 0.29(deg) 2(sRNA)
    5'     GTGCTCTCTACCATTGTCATA        3'  ID:
             || ||||o|||||||o||||           Score: 3.0
    3' CGCGGAC-AGAGGTGGTAACGGTATCGGGGGA 5'  p-value: 0.03
```

Cs8g20700.6 gene=Cs8g20700 CDS=1-1320

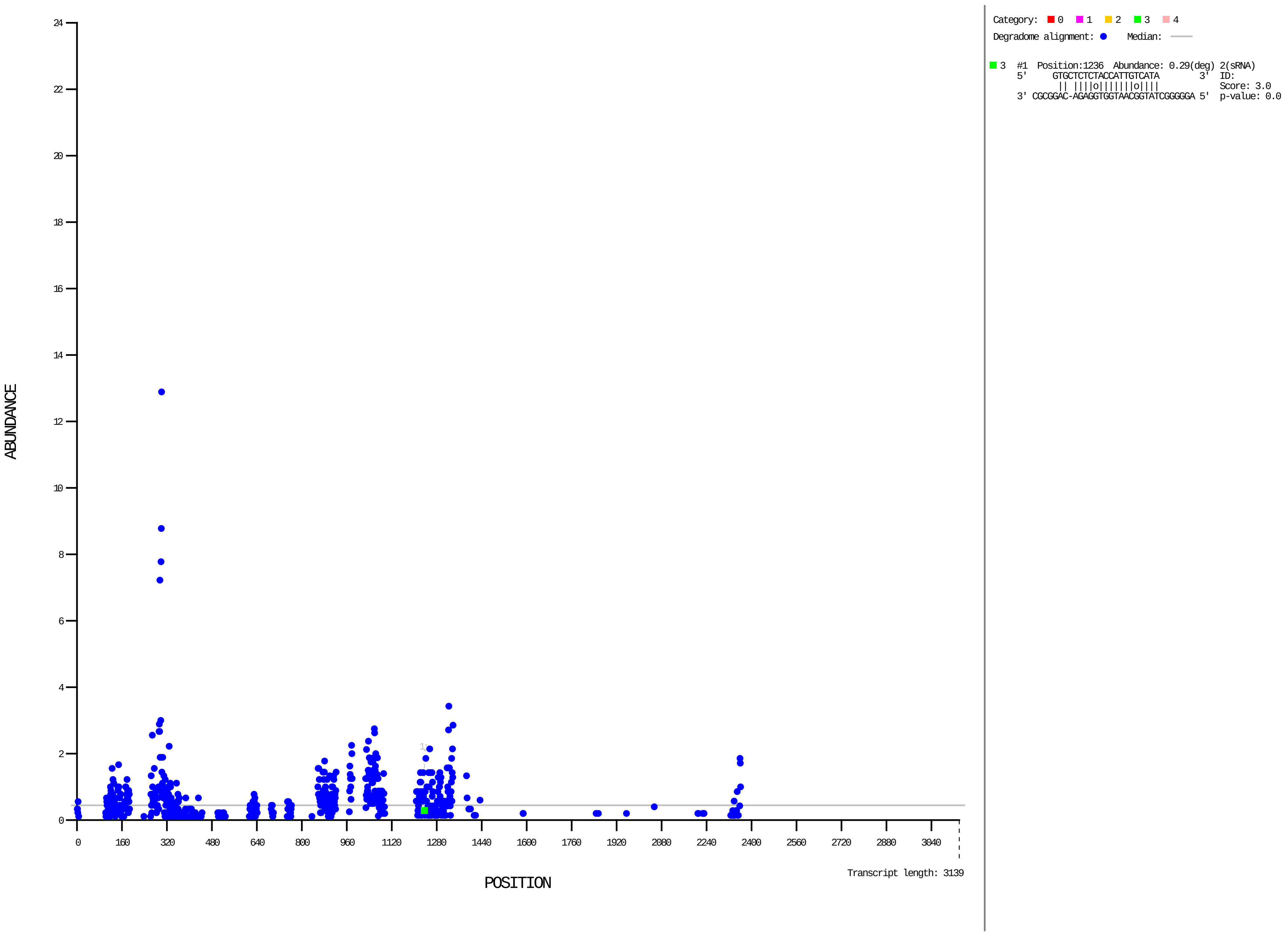

Cs8g20700.7 gene=Cs8g20700 CDS=89-1252

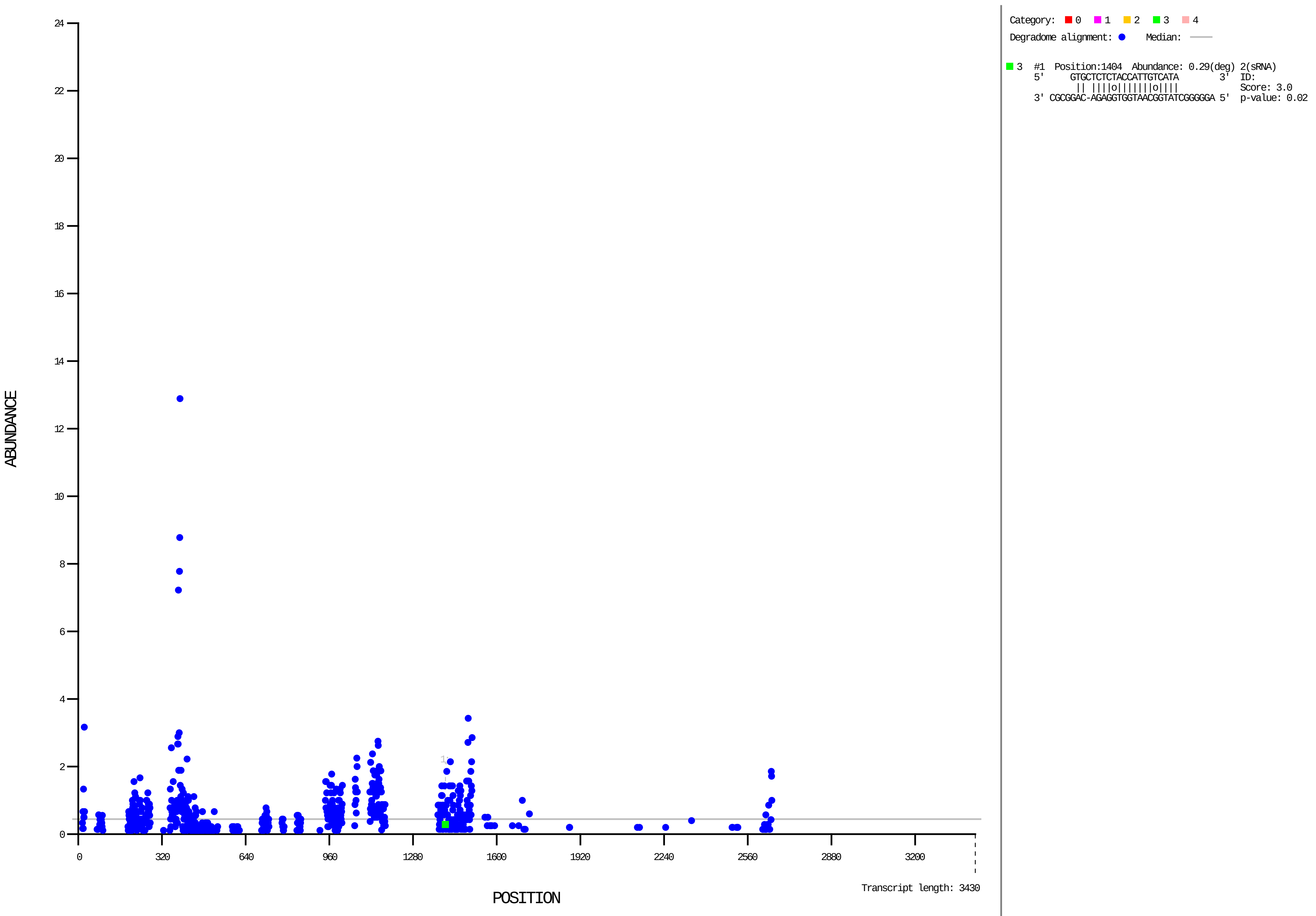

Cs8g20700.4 gene=Cs8g20700 CDS=51-1370

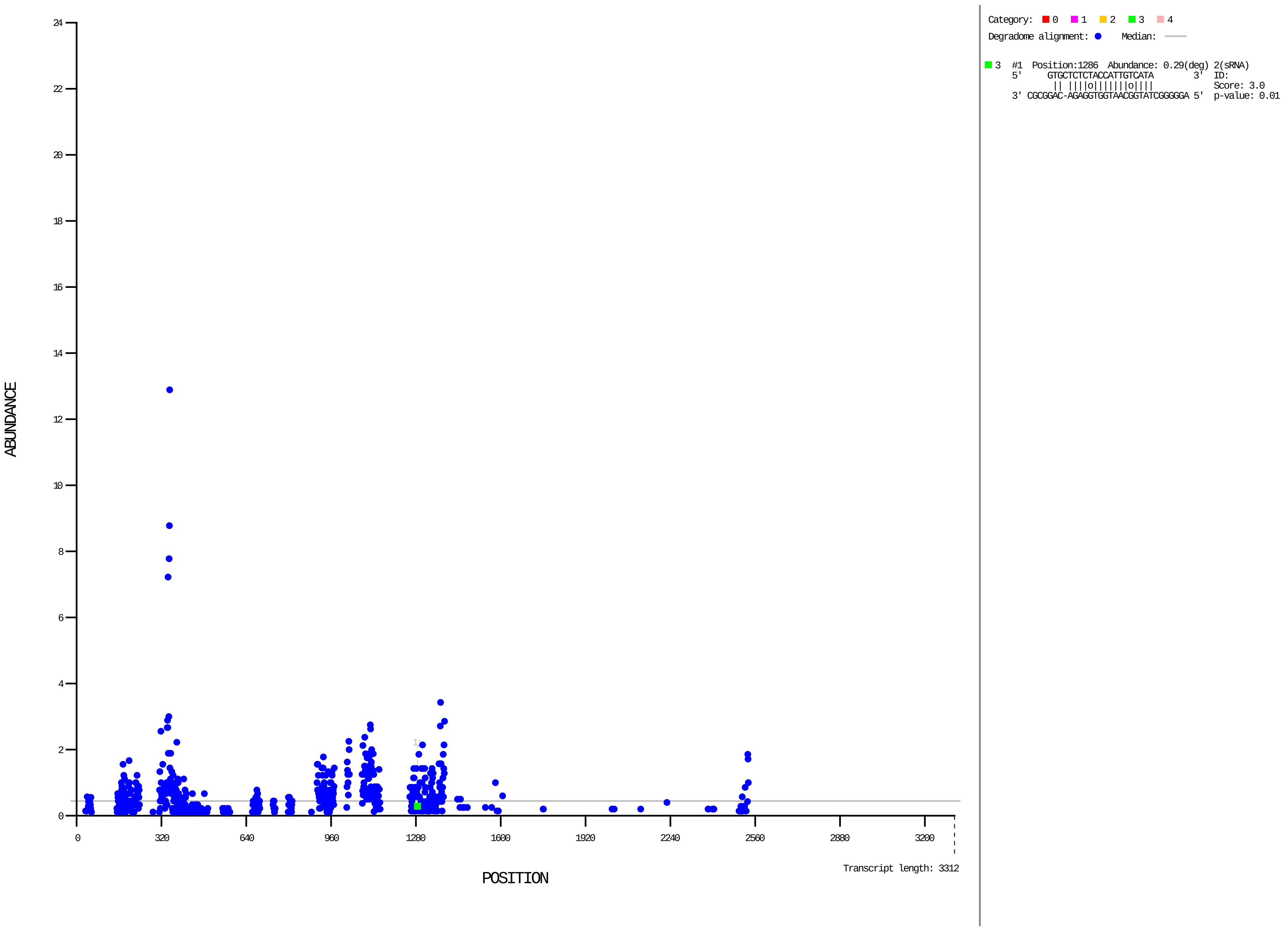

Cs8g20700.5 gene=Cs8g20700 CDS=89-1408

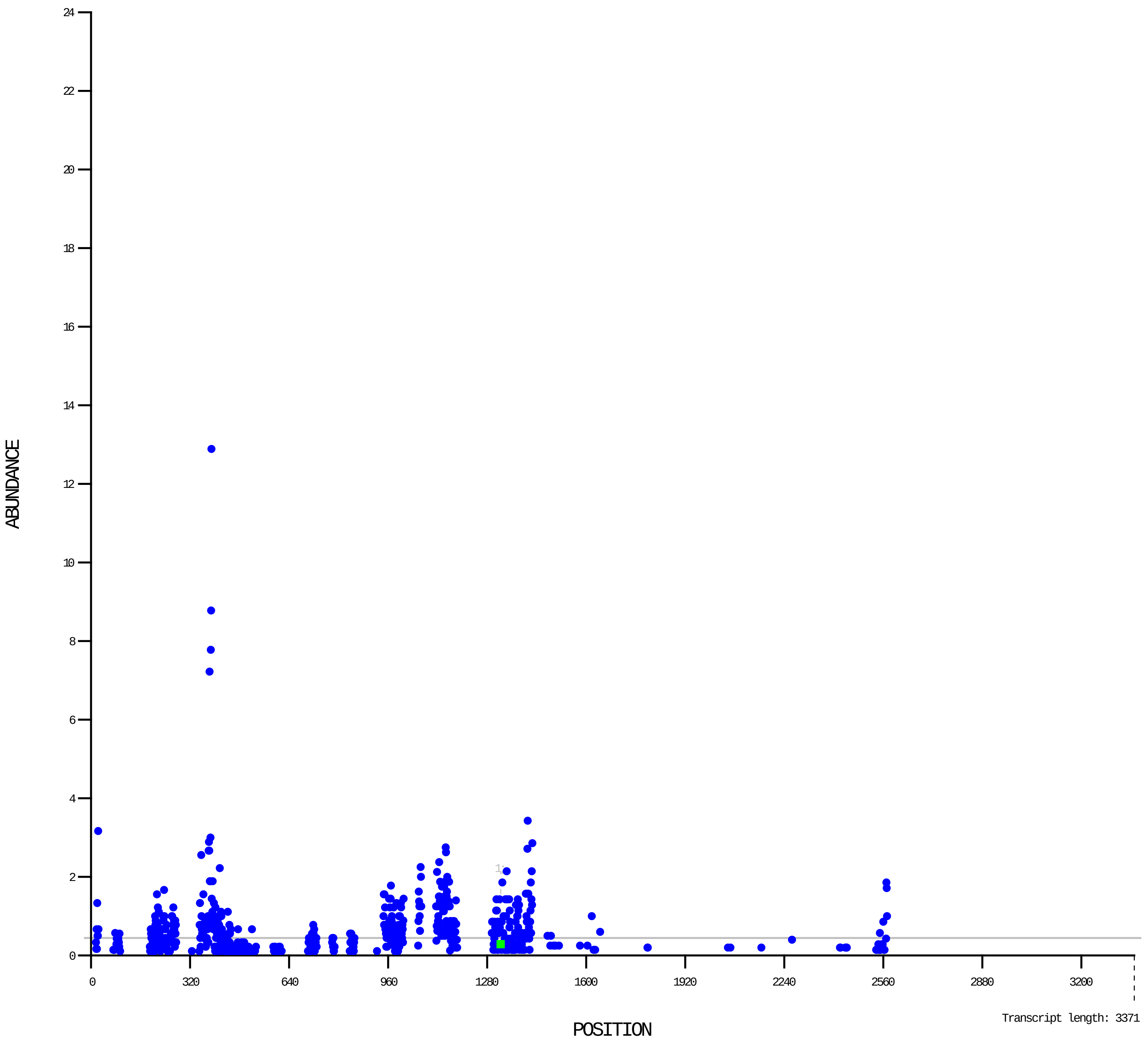

Category: 0 1 2 3 4

Degradome alignment: ● Median:

3 #1 Position:1324 Abundance: 0.29(deg) 2(sRNA)

```
5'     GTGCTCTCTACCATTGTCATA        3'  ID:
         || ||||o|||||||o||||           Score: 3.0
3' CGCGGAC-AGAGGTGGTAACGGTATCGGGGGA 5'  p-value: 0.01
```

Cs8g20700.1 gene=Cs8g20700 CDS=89-1408

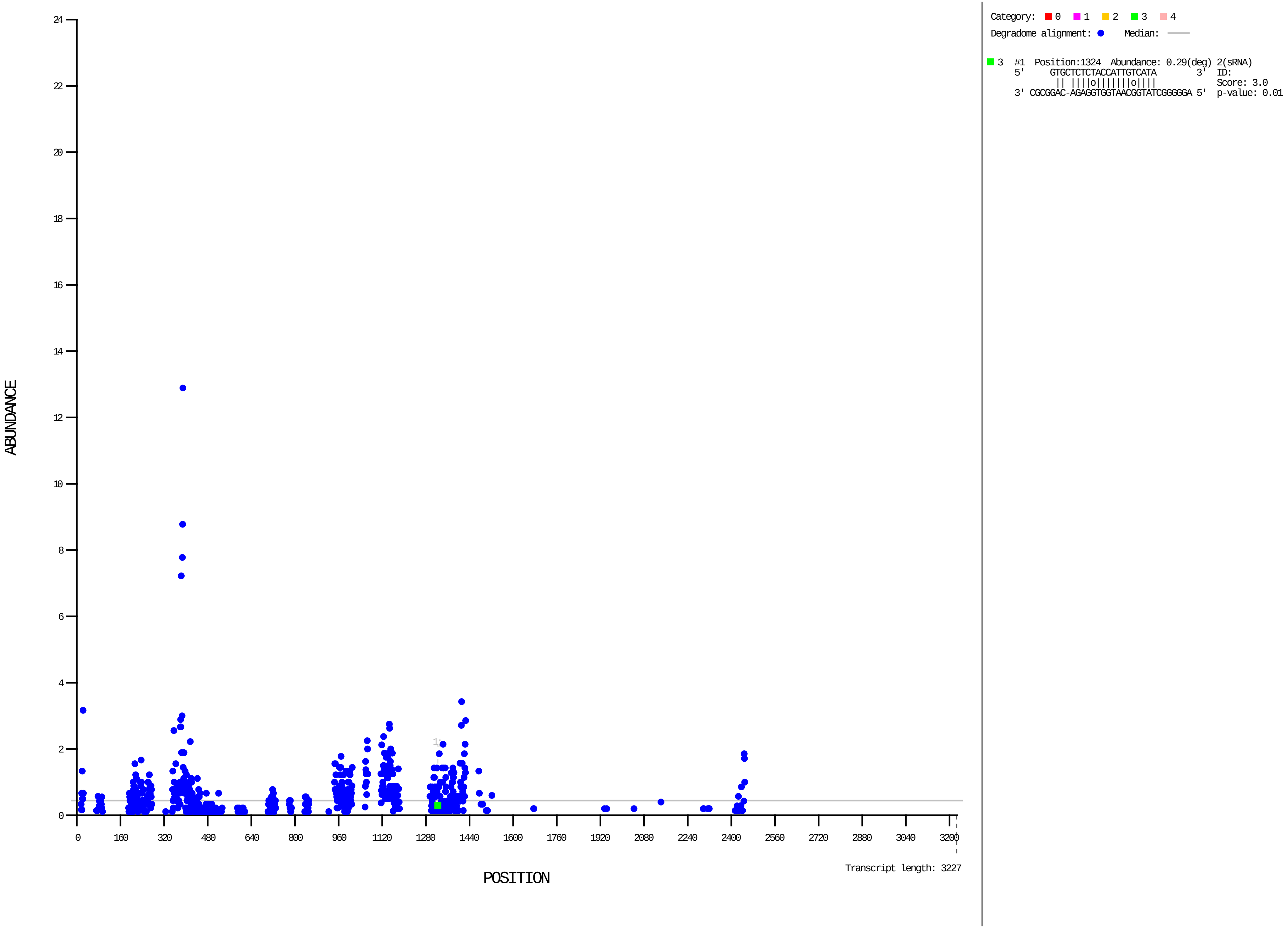

Cs8g20700.3 gene=Cs8g20700 CDS=89-1252

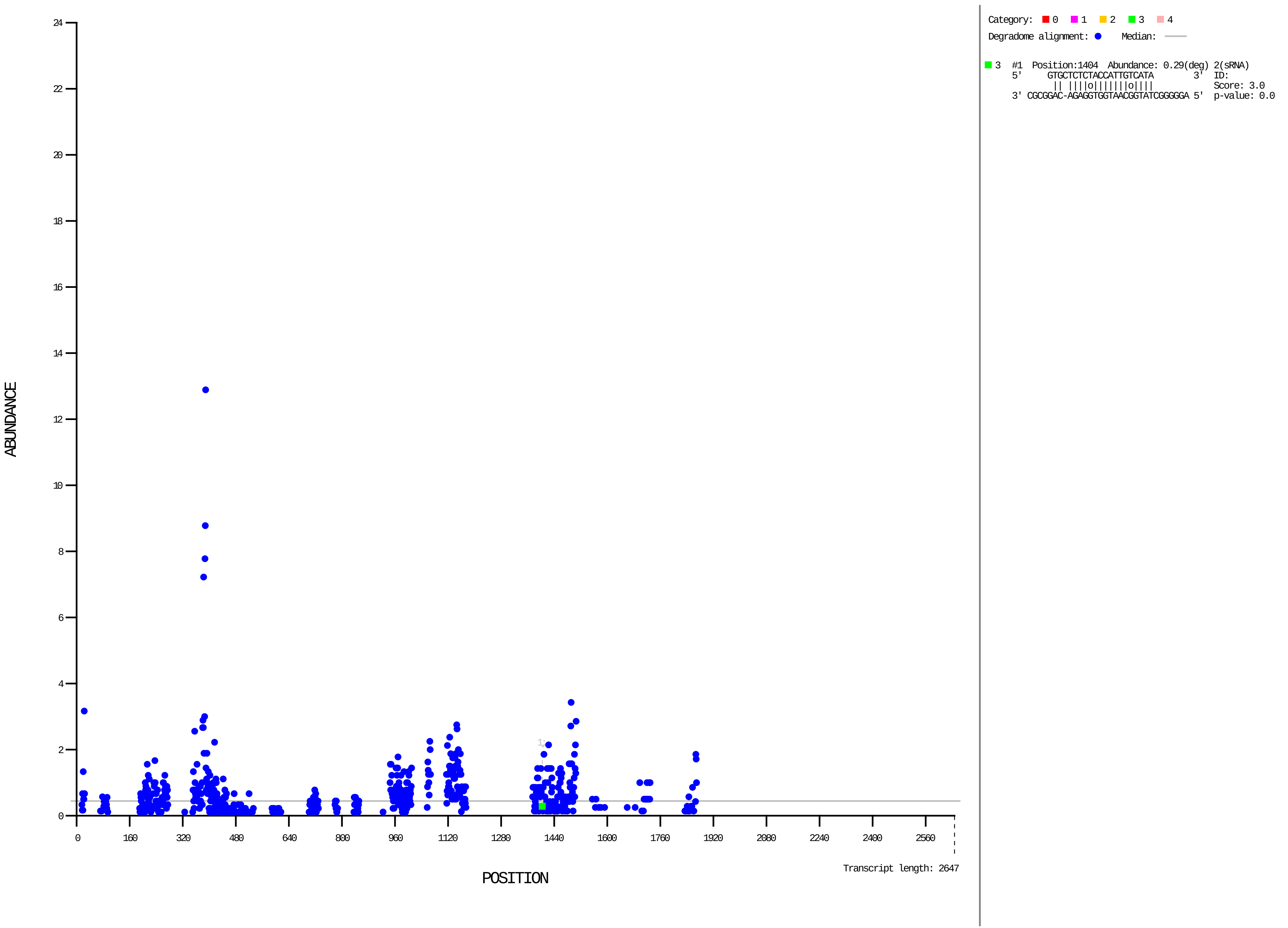

Cs2g14270.1 gene=Cs2g14270 CDS=985-2697

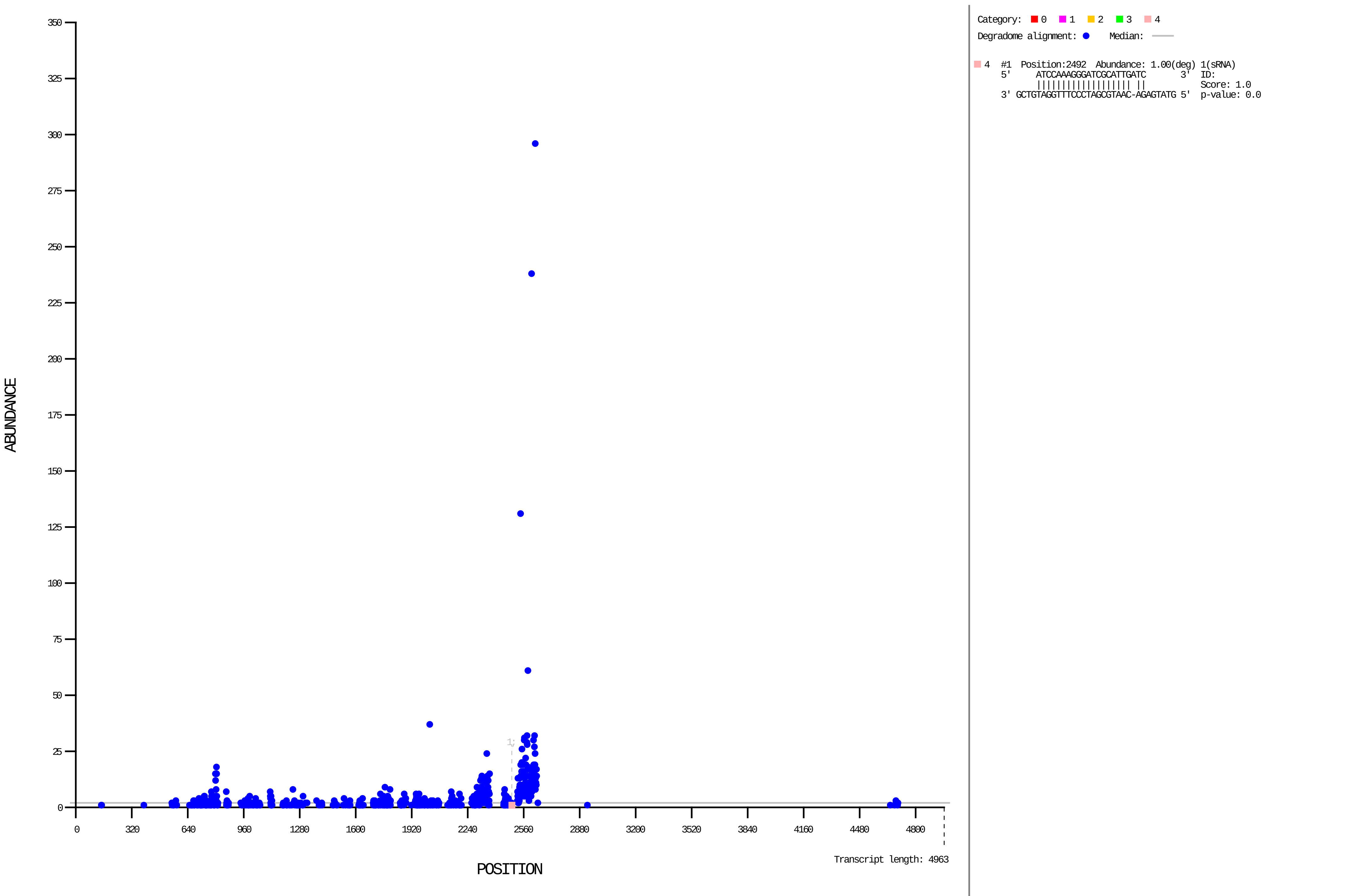

Cs3g01450.1 gene=Cs3g01450 CDS=234-2015

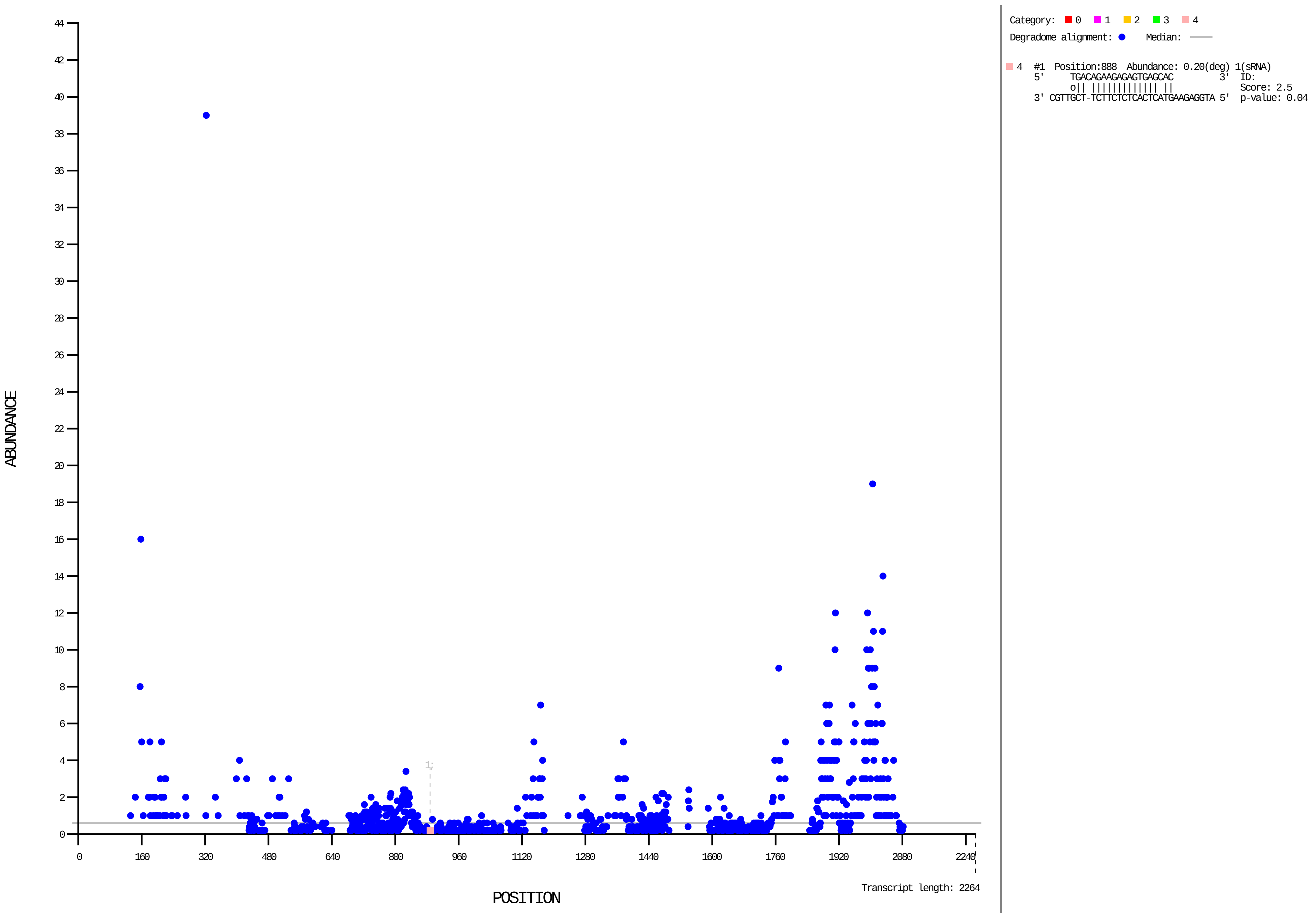

orange1.1t00584.1 gene=orange1.1t00584 CDS=1-5892

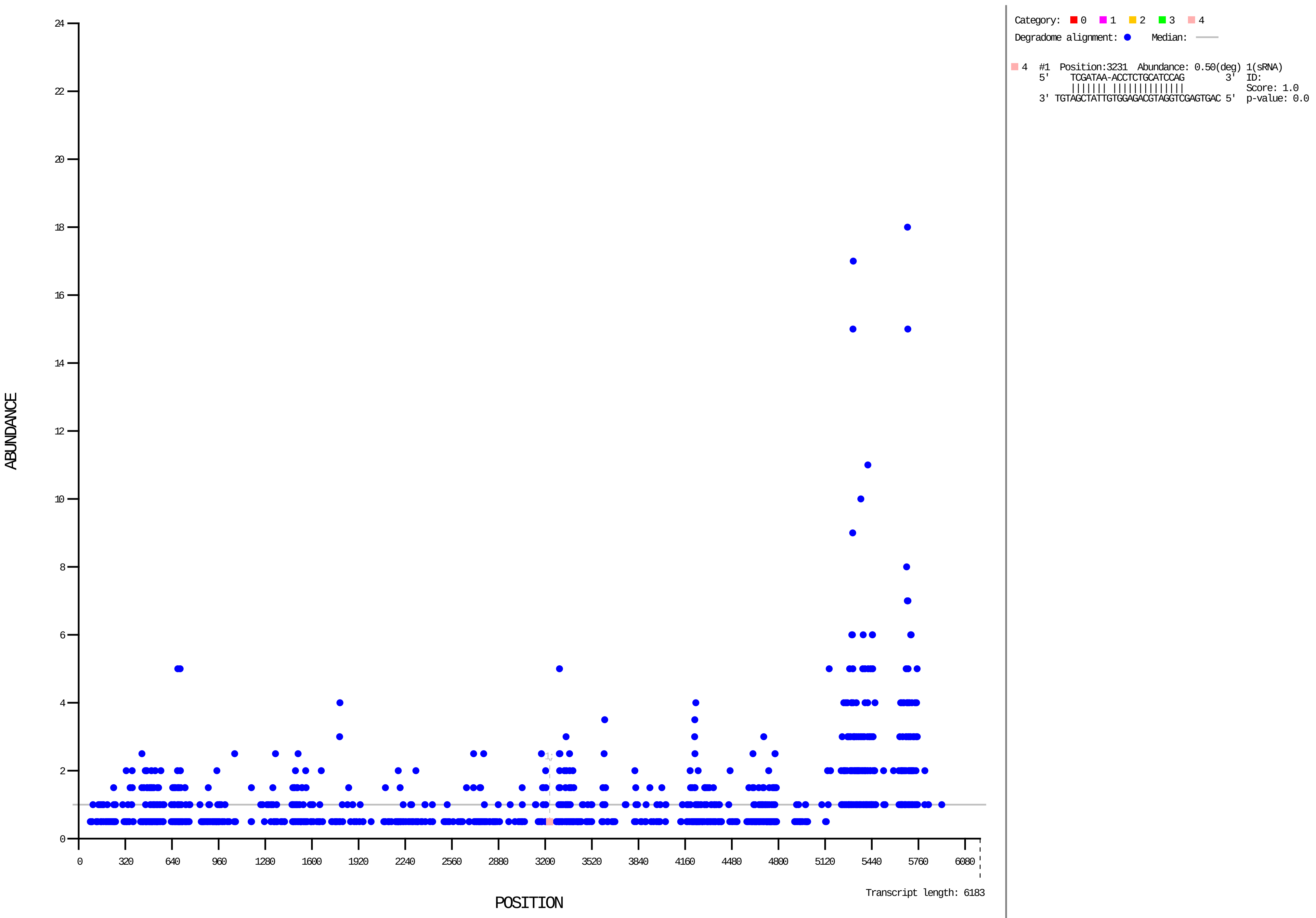

Cs5g10180.1 gene=Cs5g10180 CDS=275-2371

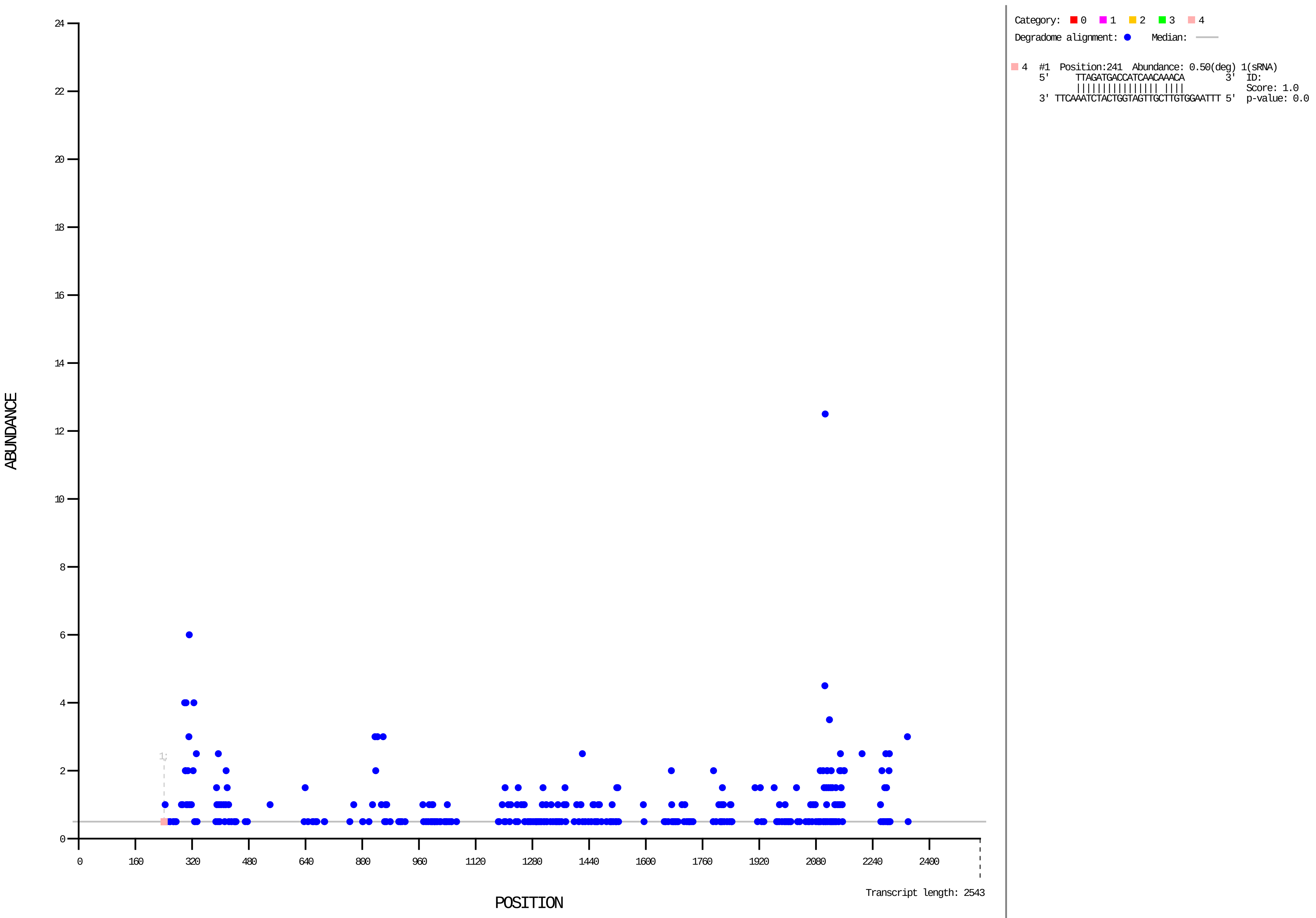

Cs5g10180.2 gene=Cs5g10180 CDS=1719-3230

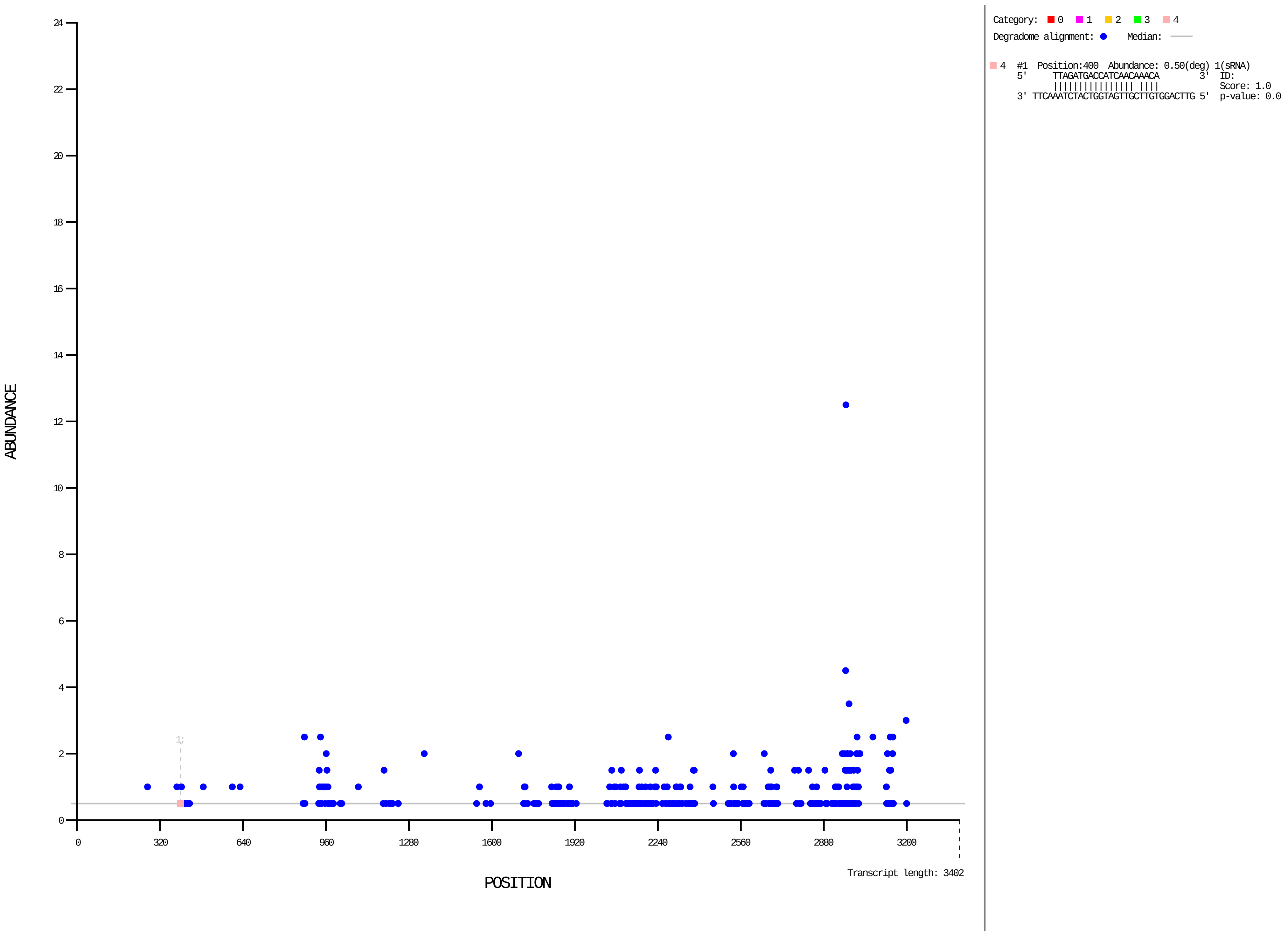

Cs5g17280.3 gene=Cs5g17280 CDS=156-2138

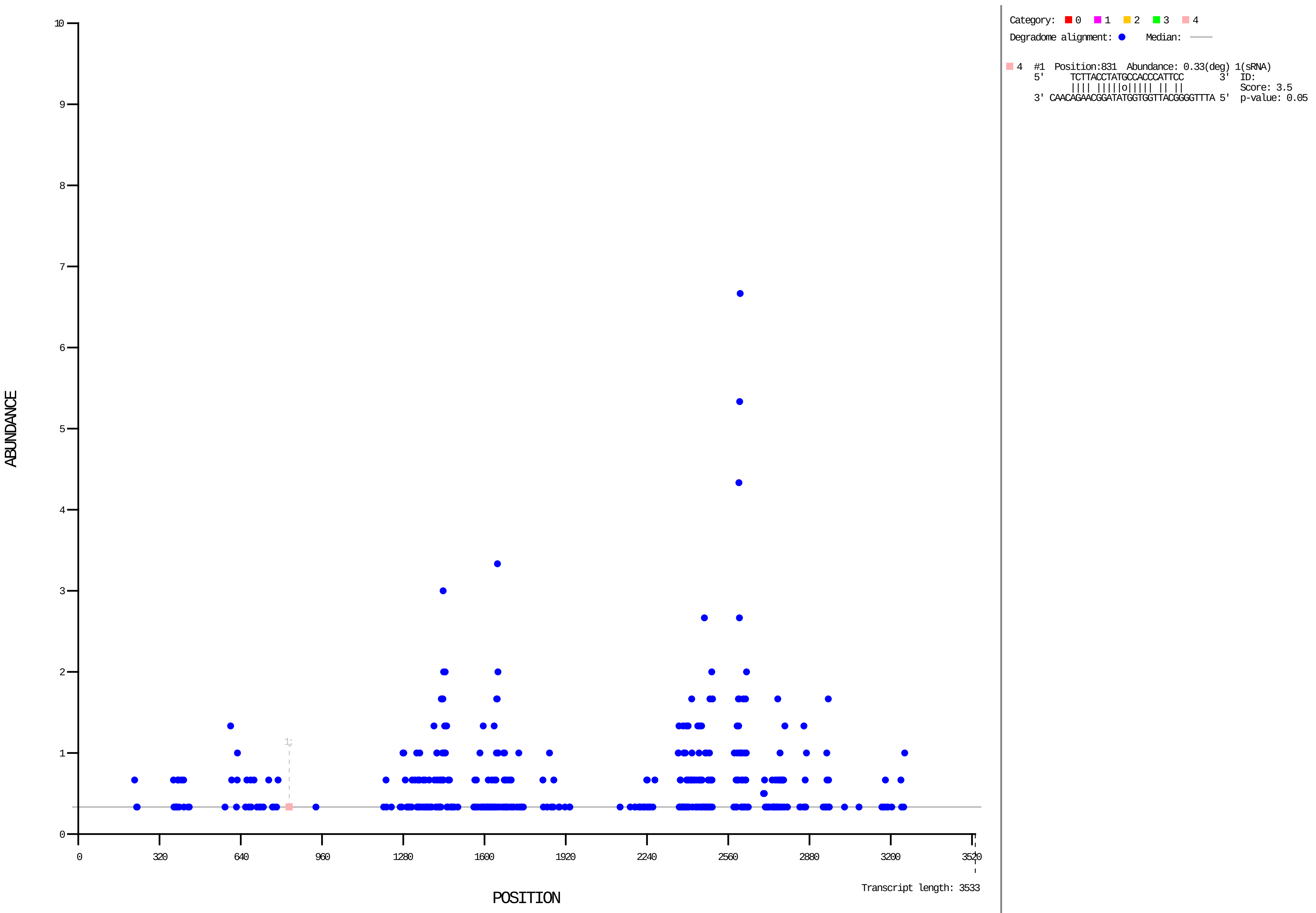

orange1.1t00584.2 gene=orange1.1t00584 CDS=1-5202

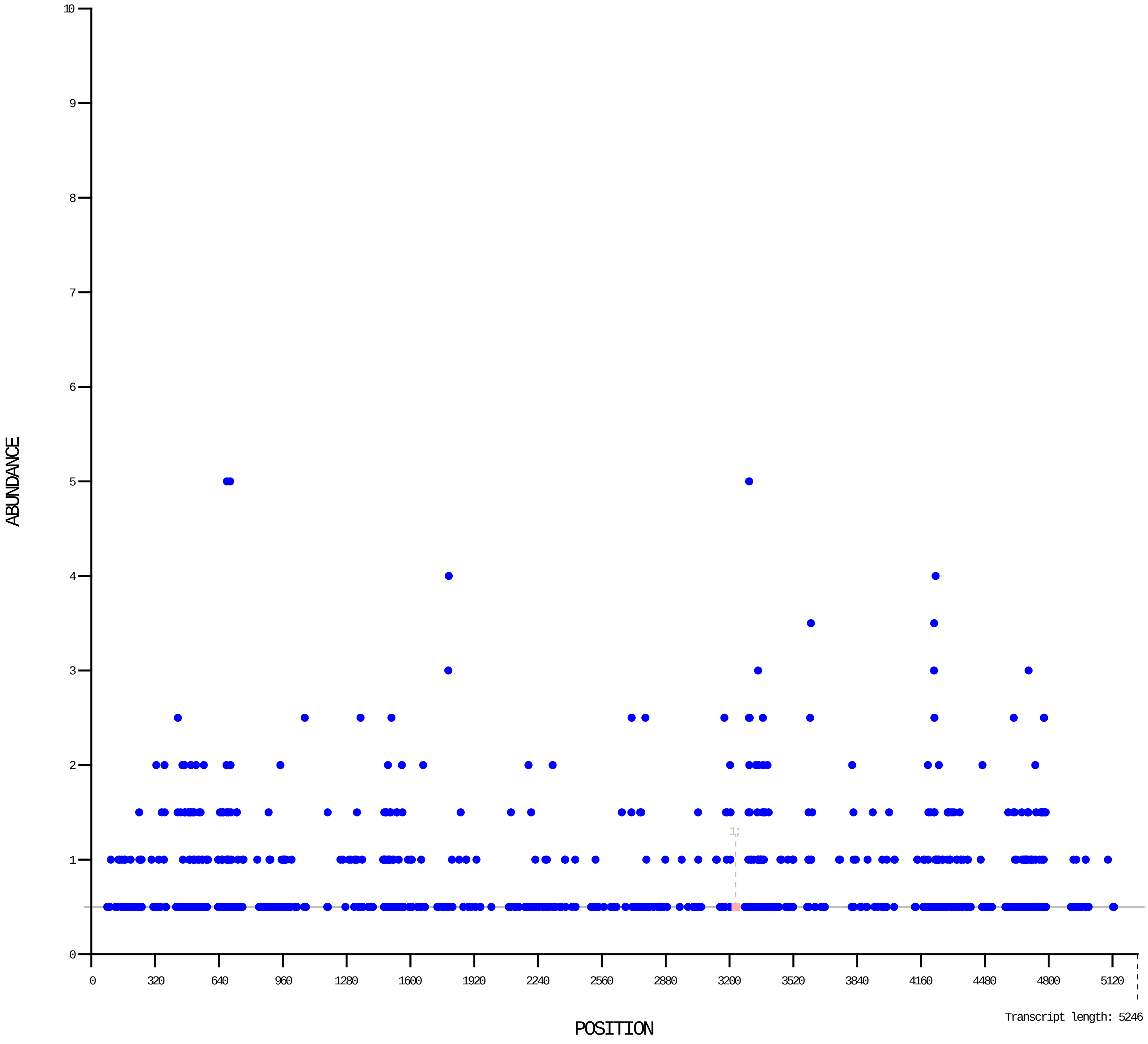

Category: ■ 0 ■ 1 ■ 2 ■ 3 ■ 4

Degradome alignment: ● Median: —

■ 4 #1 Position:3231 Abundance: 0.50(deg) 1(sRNA)

```
5'    TCGATAA-ACCTCTGCATCCAG        3'  ID:
      ||||||| ||||||||||||||            Score: 1.0
3' TGTAGCTATTGTGGAGACGTAGGTCGAGTGAC 5'  p-value: 0.0
```

Cs5g27990.1 gene=Cs5g27990 CDS=169-2022

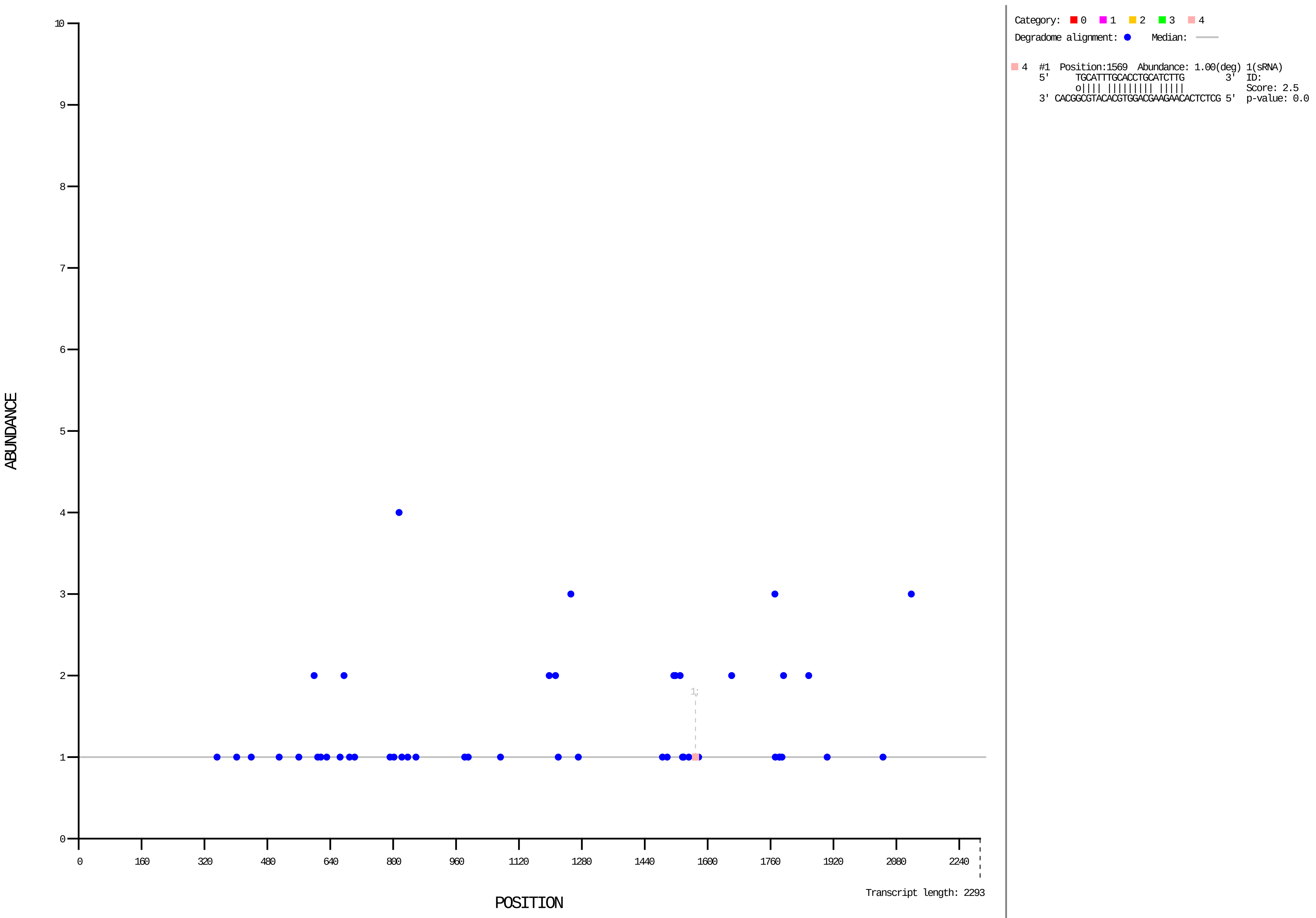

Cs8g09090.1 gene=Cs8g09090 CDS=441-1025

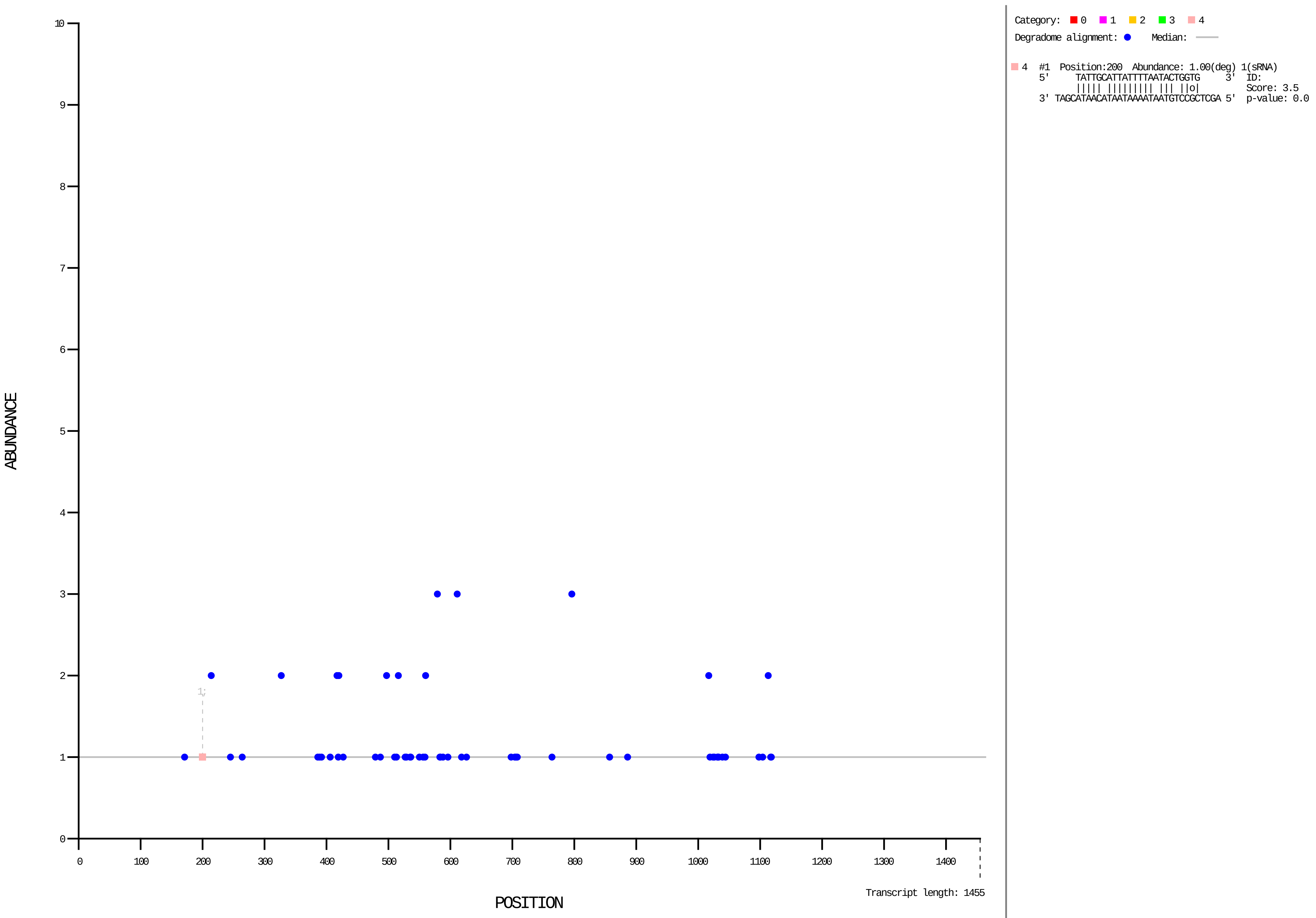

Cs2g05730.1 gene=Cs2g05730 CDS=102-689

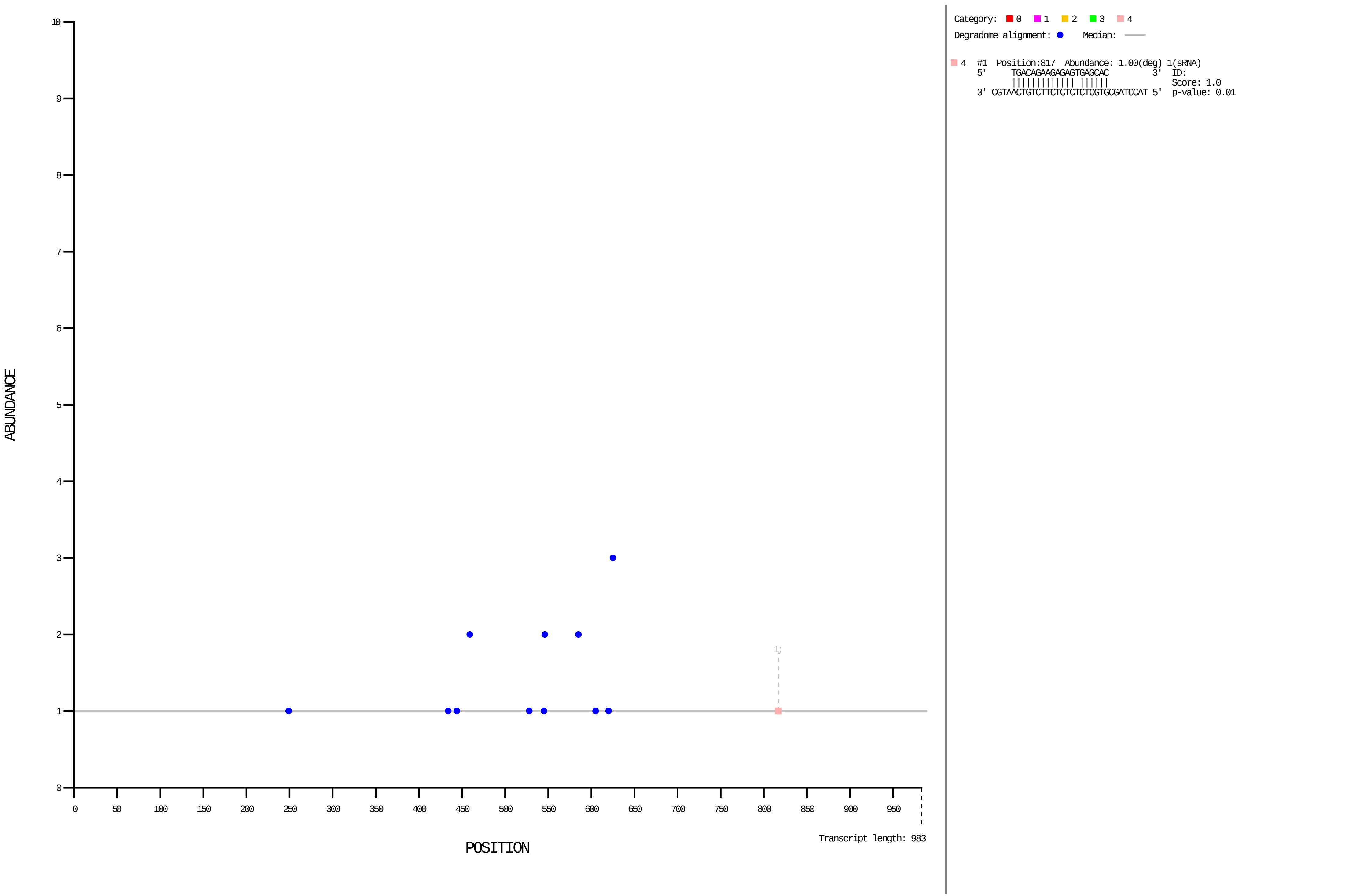

orange1.1t01926.1 gene=orange1.1t01926 CDS=90-2978

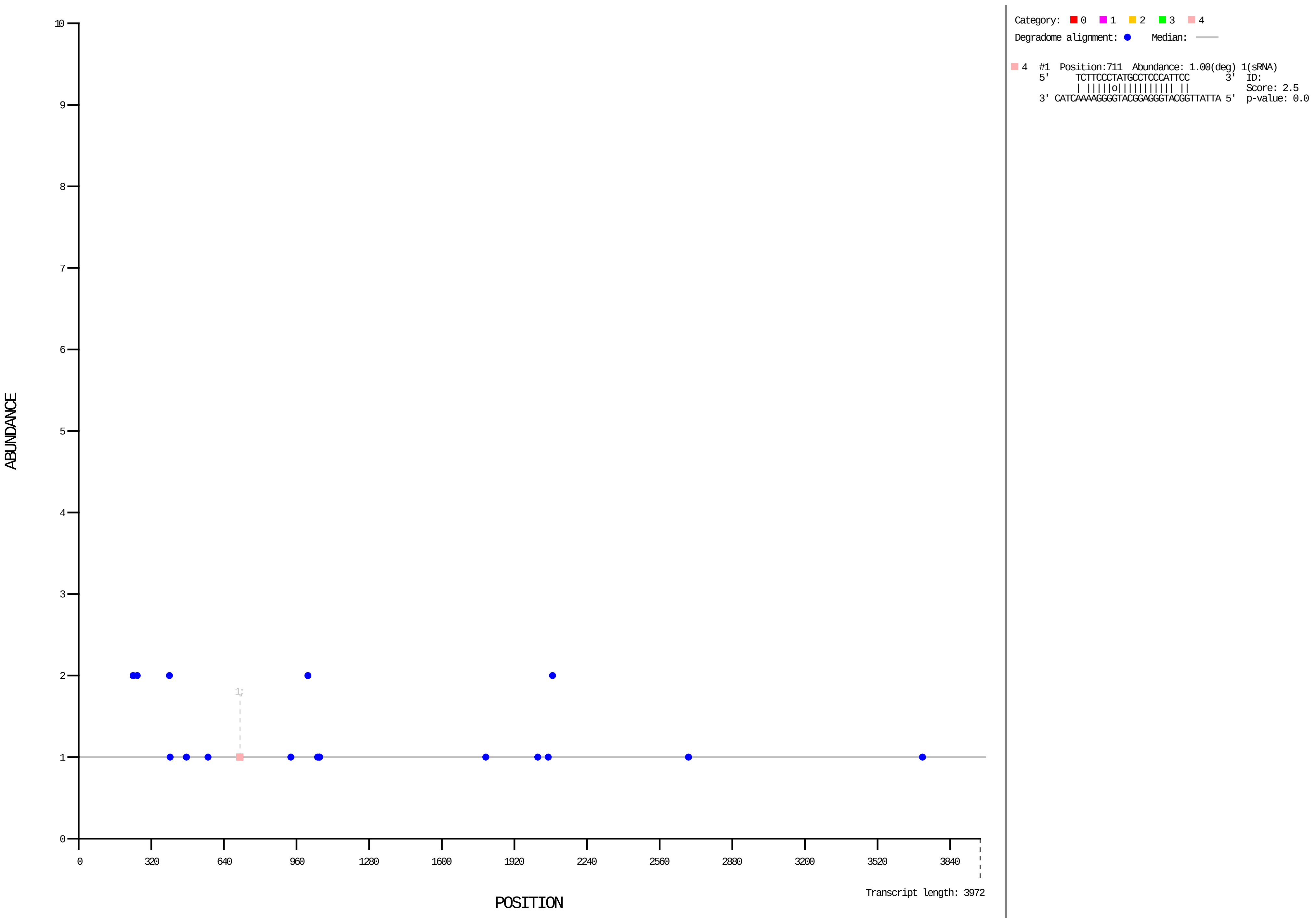

orange1.1t01536.4 gene=orange1.1t01536 CDS=309-2261

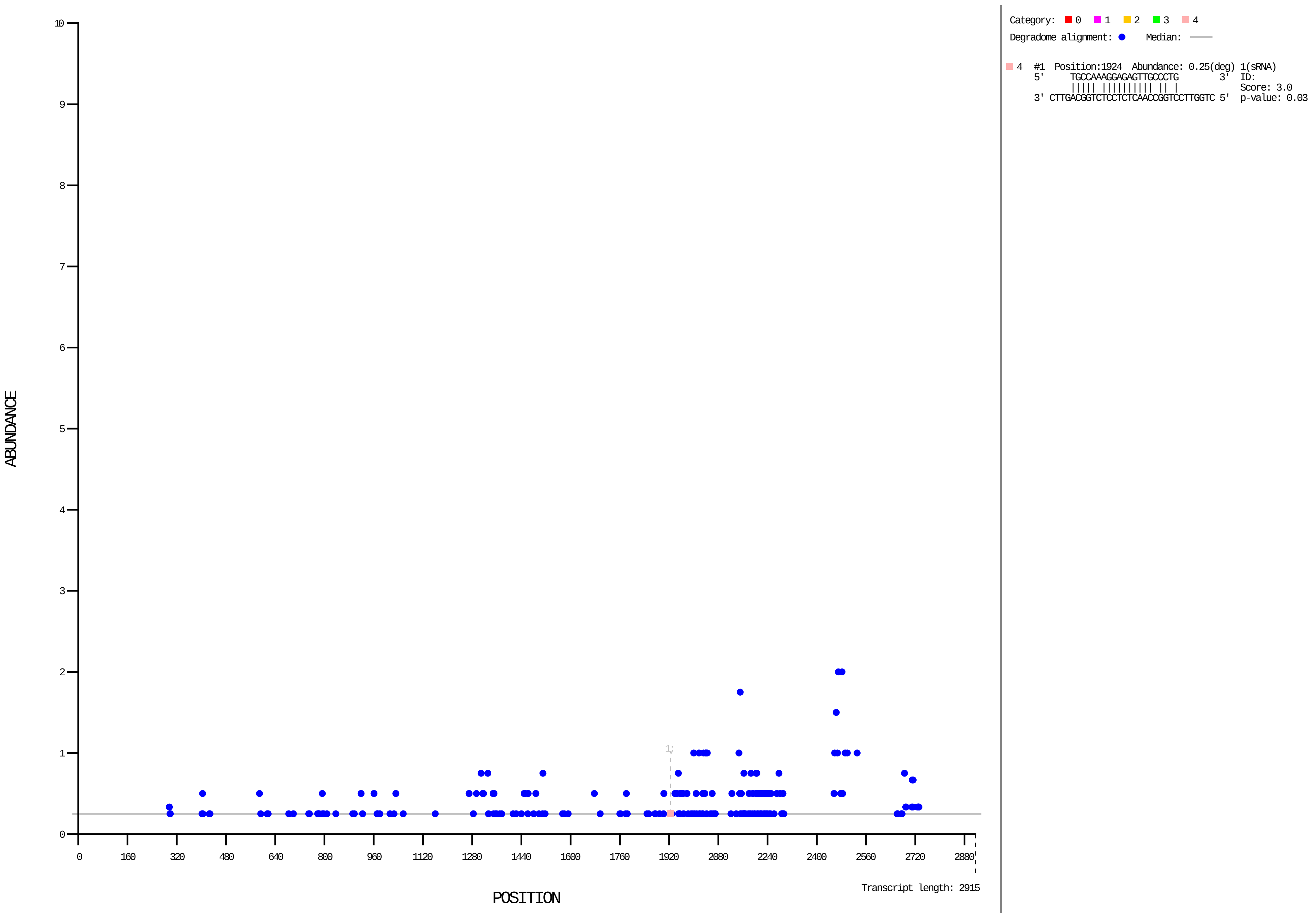

Cs3g26830.1 gene=Cs3g26830 CDS=3227-3550

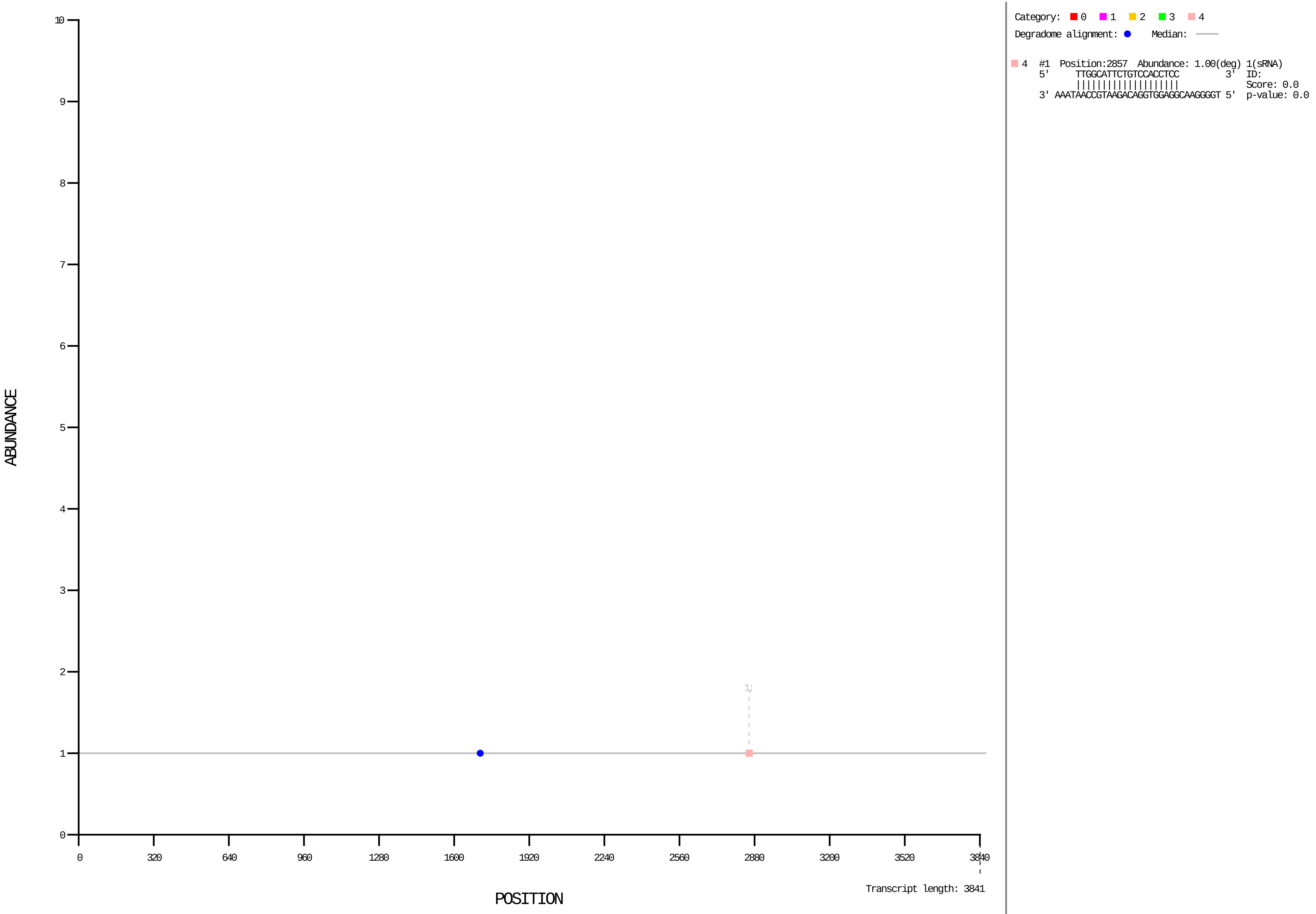

orange1.1t00172.1 gene=orange1.1t00172 CDS=280-1155

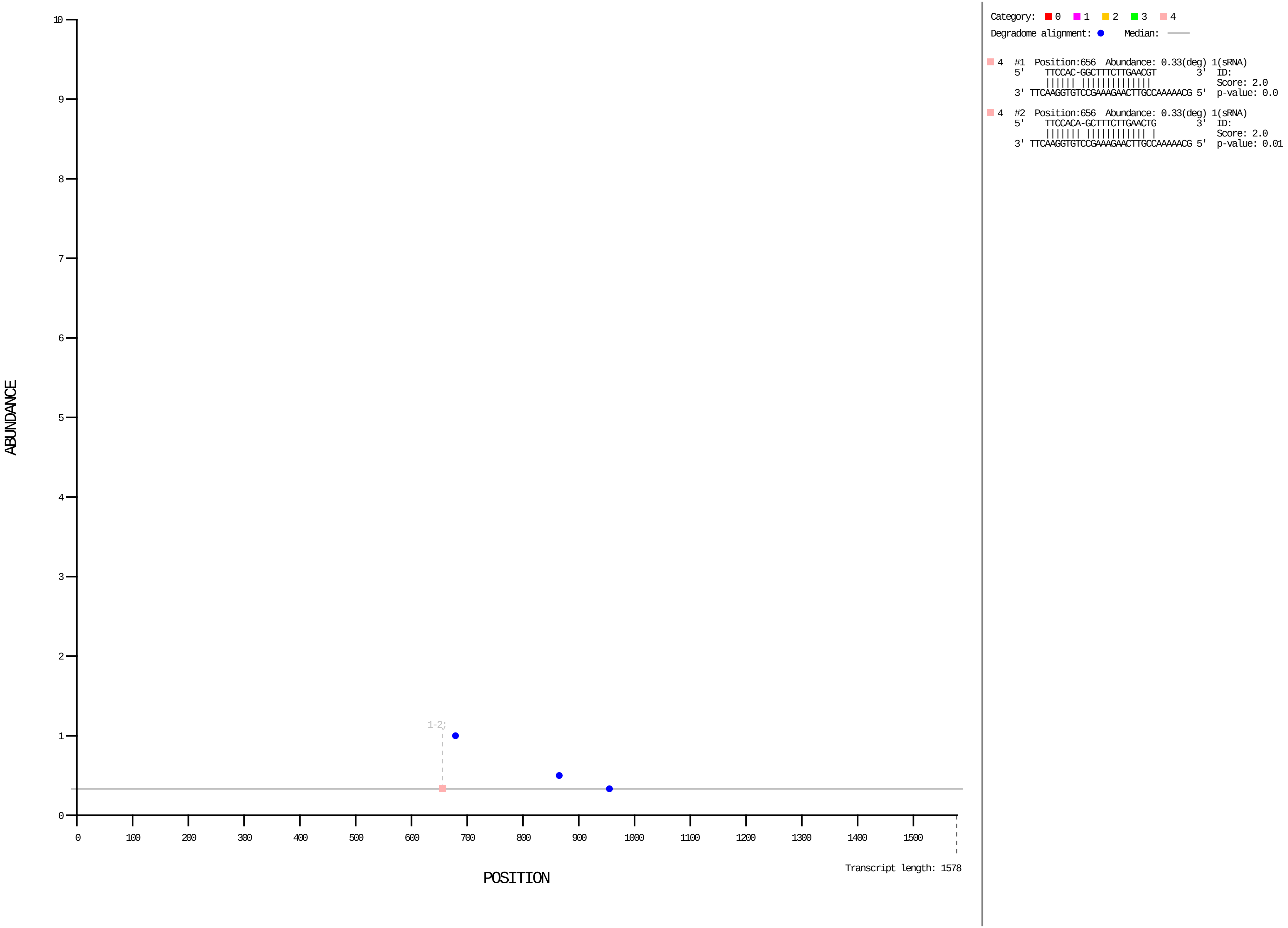

Cs6g15330.2 gene=Cs6g15330 CDS=203-1426

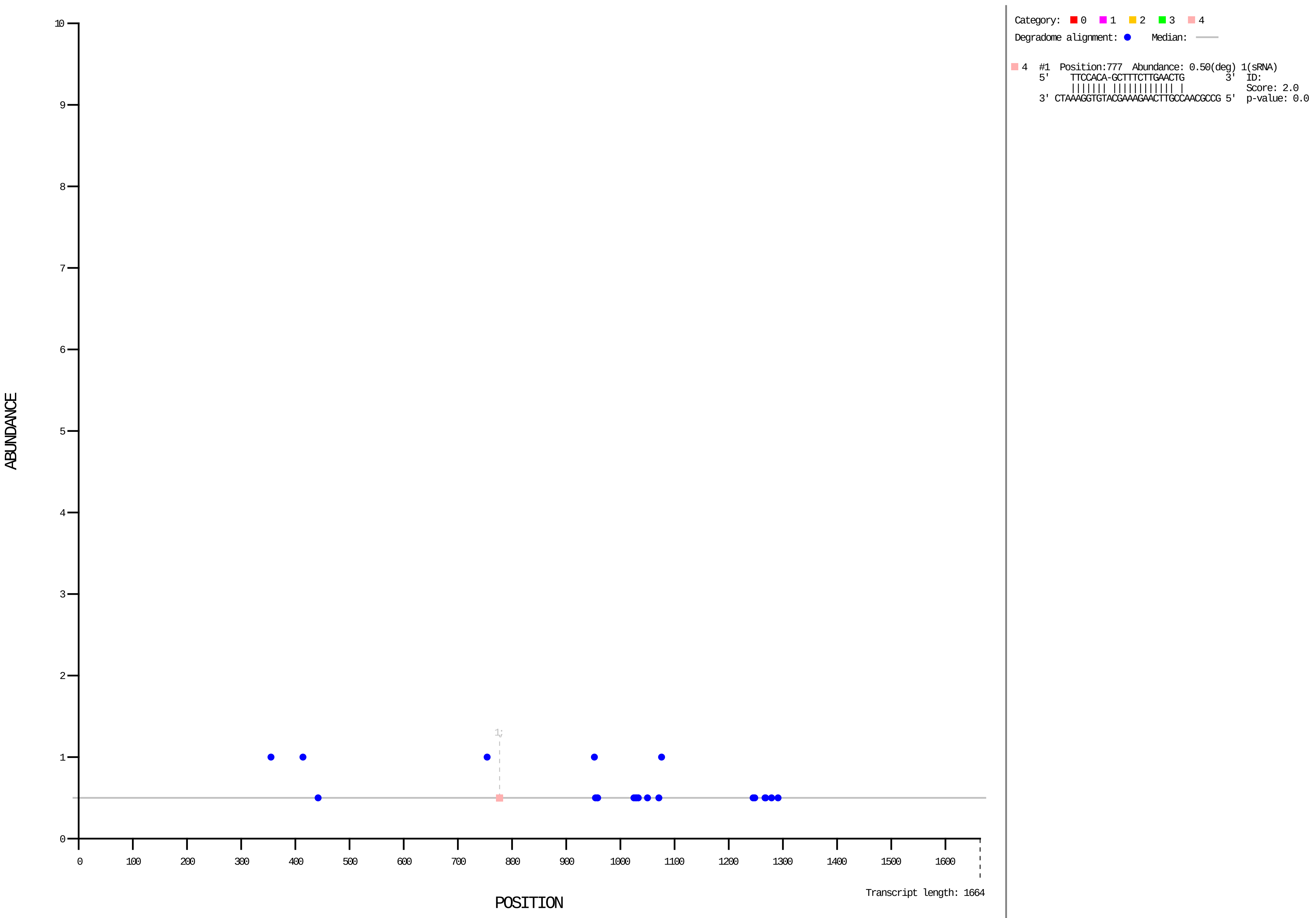

orange1.1t00172.2 gene=orange1.1t00172 CDS=280-1155

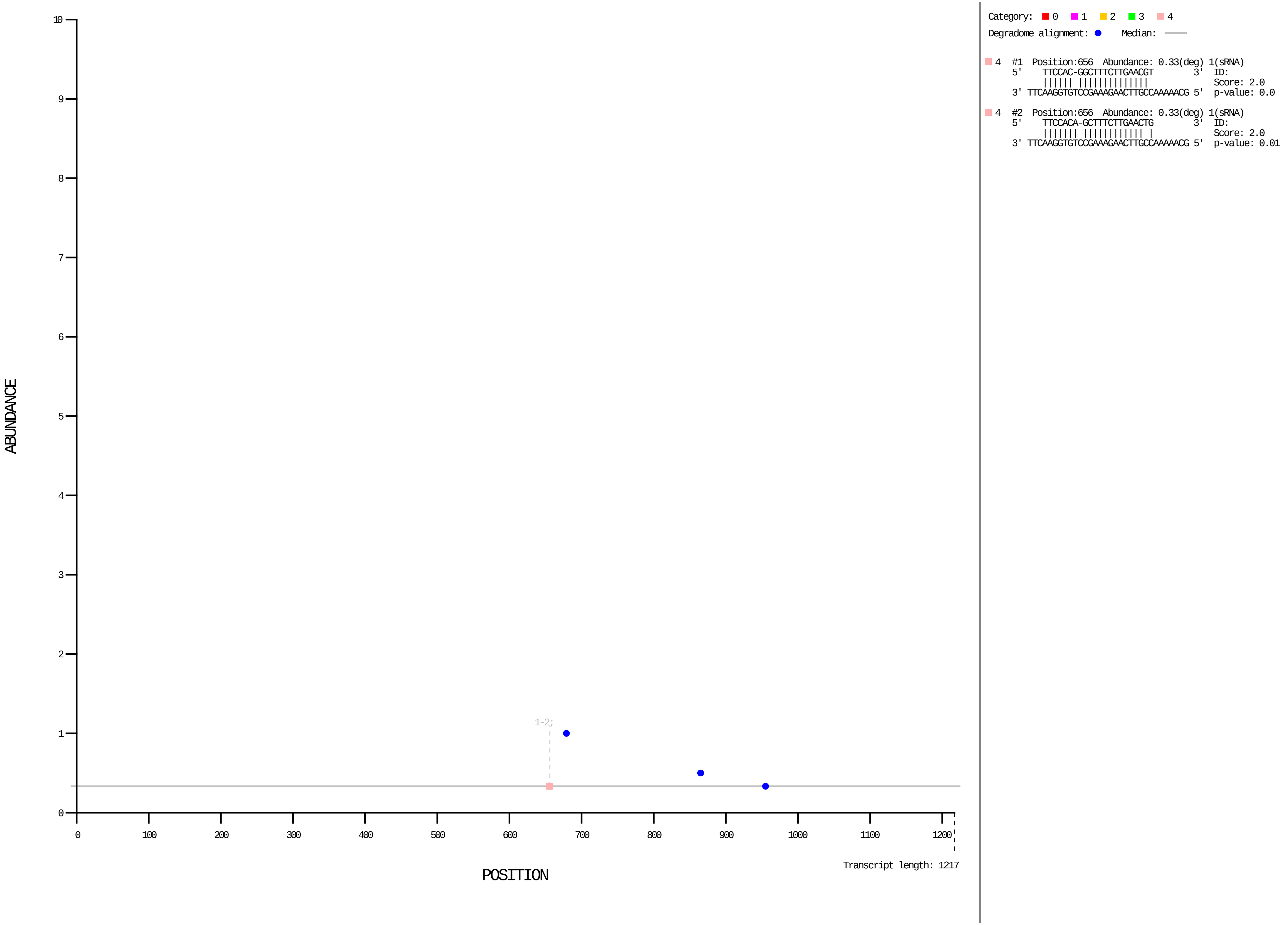

Cs6g15330.1 gene=Cs6g15330 CDS=37-1098

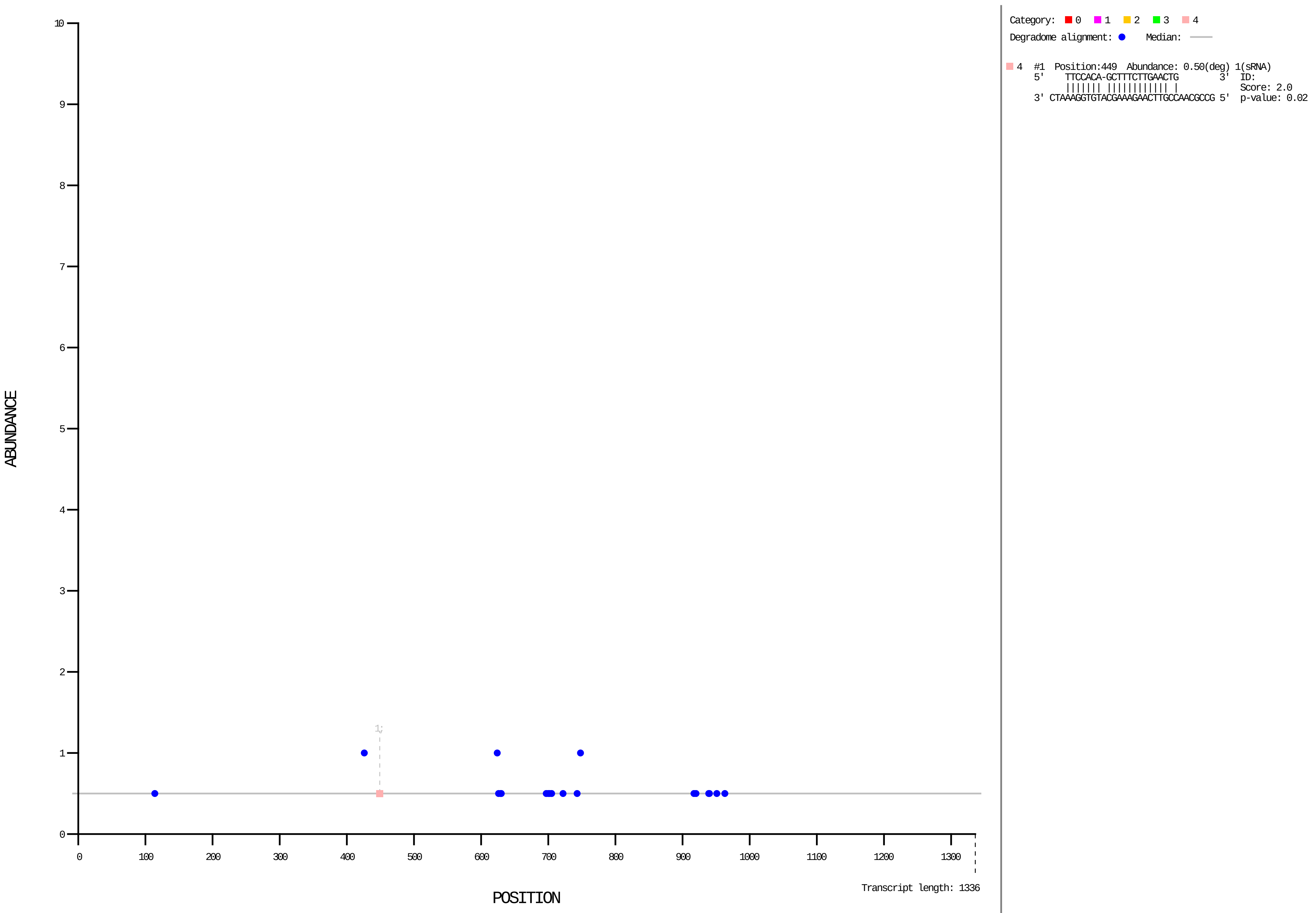

orange1.1t00172.3 gene=orange1.1t00172 CDS=280-1248

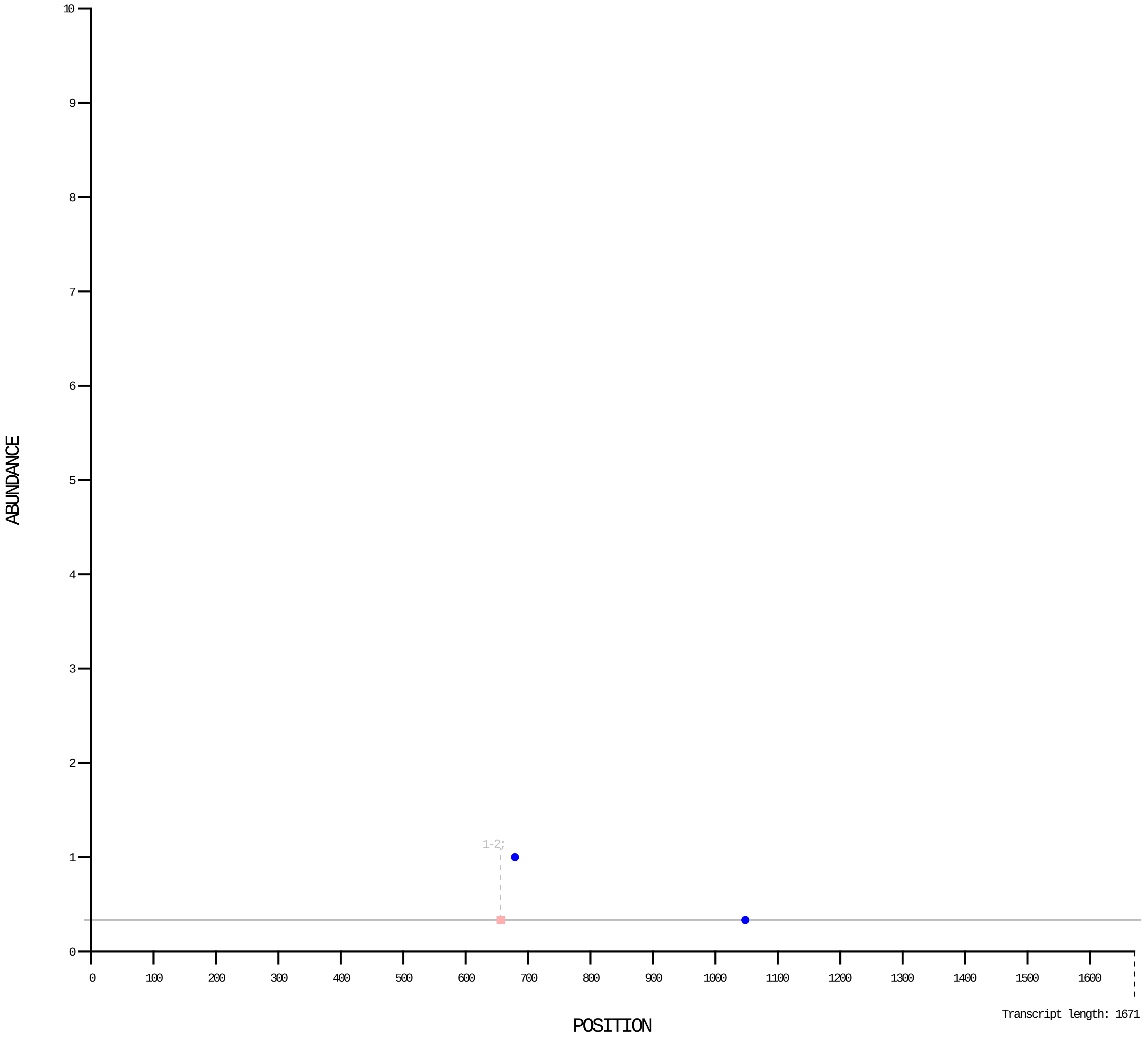

Category: 0 1 2 3 4
Degradome alignment: Median:

4 #1 Position:656 Abundance: 0.33(deg) 1(sRNA)
```
5'    TTCCAC-GGCTTTCTTGAACGT        3'  ID:
      |||||| ||||||||||||||             Score: 2.0
3' TTCAAGGTGTCCGAAAGAACTTGCCAAAAACG 5'  p-value: 0.02
```

4 #2 Position:656 Abundance: 0.33(deg) 1(sRNA)
```
5'    TTCCACA-GCTTTCTTGAACTG        3'  ID:
      ||||||| |||||||||||| |            Score: 2.0
3' TTCAAGGTGTCCGAAAGAACTTGCCAAAAACG 5'  p-value: 0.02
```

orange1.1t04094.1 gene=orange1.1t04094 CDS=1-4731

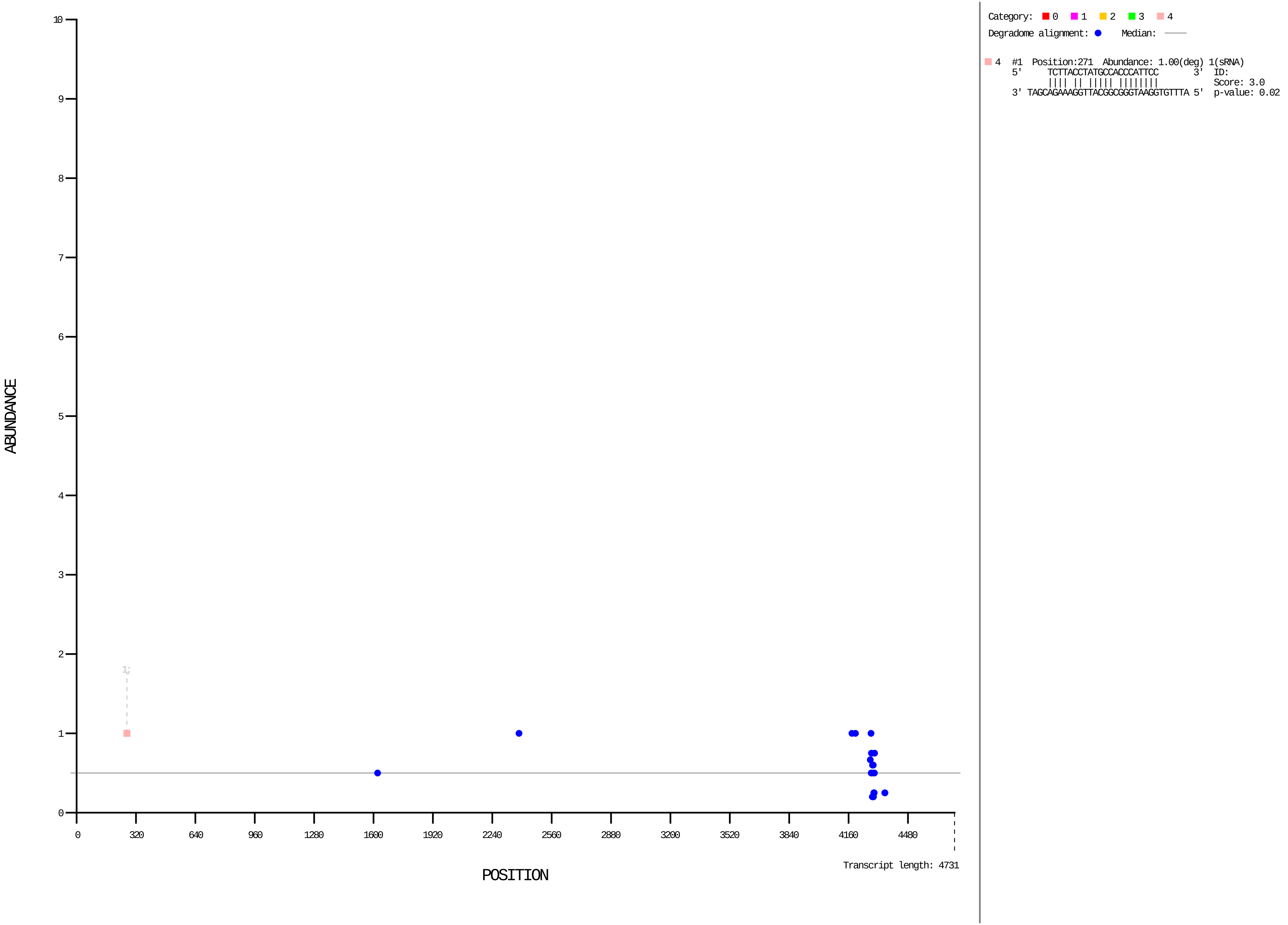

Supplement: Supplementary file 9 — Figure S7 [file 41438_2018_116_MOESM9_ESM.pdf]
